# Supplementary material for: The Gain and Loss of Cryptochrome/Photolyase Family Members during Evolution
Source: Genes (Basel). 2022 Sep 8;13(9):1613. doi: 10.3390/genes13091613 (PMC9498864; doi:10.3390/genes13091613)
Supplement: Supplementary file 1 [file genes-13-01613-s001.zip › genes-1883542-supplementary/Supplement 1 2249 sequences.pdf]

>Acanthaster_pla_XP_022085333.1_cry-2-like
MNSPMAKHRSLHWFRKGLRLHDNPSLLAAVKDSQEFRAIFVLDPVFVKSGTVGINRWRFLLQSLRDLDEGLKR
LGSRLYVLRGKPTEVFPDIFKSWQISHLTFEHDTEPYAKIRDEAVTTIAEEHGVRVTQKTSHTLYDIQSILKA
NGGKPPSTYQSLLSVLSKMPPPRKPEPALSKEHLKDCKLPVGTDHDGEFGVPSLEELGLDPNDAGPVLFPGGE
TEALQRMEEHLERQDWVCKFEKPKTEPNTLSPSTTVLSPYLKFGCLSPRLFYHRVQDVYARKKSHSKPPVSLH
GQLLWREFFYTVAAATPNFHKMEGNAVCLQVPWDTNEEYFQAWKQARTGYPFIDAIMTQLKQEGWIHHLARHA
VACFLTRGDLWISWEEGQKVFEEWLLDADYSLNAGNWMWLSASAFFHQYFRVYSPTVFGKKTDKNGDYIRKYL
PVLKKMPAQYIYEPWTAPRSVQEKAGCIMGTDYPRPIVDHSVVMKRNMERMKRARAAKYGDSNANSKSQKEAS
SSKKKVNAGGLKRKSKAEDSGHSTKKTKEISDFF
>Acanthaster_pla_XP_022093922.1_cry-1-like
MGKRKRSRGSSRRGVSLVHWFRNKGLRLHDNPALREALVGAKTFRCVYILDPWFARSPNQGVNKWRFLLQCLE
DLDSSLRKLNSRLFLVRGQPSDVFPKLFKEWGVTRLSFEEDPEVFGIQRDAKIKTLAATFKVQVITRMSHTLY
DPKEIVKMNNSNPPLTYKHFQYLISLLDQPTFPEETITLSDIEGINTAIAANHDSKYGLATLEELGFDSEDIP
PPVWIGGETVAKQQLDRHLERKAWVANFERPRMSSASLMACPTGLGPYLRFGCLSPRLFYWRLTELYQKMKKT
SNIPLFLHGQLYWREFFFTLGYNNQKLDQMVGNPICIQIPWEKNEEALQRWRDGQTGFPWIDAIMIQLRQEGW
VHPVARHAVACFLTRGDLWISWEEGAKIFEDLLLDADWSVNAGNWIWLSCSSFYQQFFHCYTFCPVKFGQQTD
PNGDFIRKYIPALKNFSSKYIYNPWTAPLEIQEQAKCTIGKDYPFPMVNHNEAIRRNTERMNNILQNVSSKTP
KYIHDECSTGVPSFCRTSSSTWVSNTPQEPLRKMPHLSQDSMPVLSHDRMLSQGSMALLPQDEVPGTSGYMST
NQSTMLTLTDPYTCNMTQESLVAAQPSQDAPGTSWNDSNNLANQYFNQ
>Acanthaster_pla_XP_022095977.1_photolyase
MAKGEKGSNQTTKKGKRKHEDSSSSEAAALPSKKLKIETKTSDTTKQEVKSTSDAQGIMEKLMALRLRTASSI
SAFKFNKKRVRVLSGVQDVPEGCQGILYWMSRDQRVQDNWGLLYAQRLAMKLTVPLHVCFCLVPKFLDATIRH
FGFMLKGLAEVEQELKELDIPFHLLTGEATDVLPSFVKDHGIGGMVTDFSPLRVPSKWVKEATDSLPHIPVCQ
VDAHNIVPCWEASPKLEYGARTIRPKITKLLSTFLTEFPPVVKHPHKAKTMPEPVDWEAADCSLDVDRTVEEV
SWAKPGTKAGLLMLEEFCLQRLKYFEADRNNPNKTALSNLSPWIHFGQVSAQRAALVVRGHRSKYSKSVESYI
EESVVRRELADNFCFYNEHYDSIEGTNDWAKETLKVHAKDKREYVYSKEQLEKARTHDDLWNAAQVQLVKEAK
MHGFLRMYWAKKILEWTPSPEDALEIAIYLNDKYSLDGRDPNGYVGCMWSICGIHDQGWAERPVFGKIRYMNY
NGCKRKFDVAQFVRKYKQKL
>Acanthaster_pla_XP_022106989.1_cry_DASH-l
MANKLRTVICLLRNDLRYHDNEVLHWANQNADYVLPLYCLDPRHYHGTWHFSFPKTGPHRLKFLLQSLQDLKE
TLQQVGSDLVISRGKPESVIQDLVRQLGKDNVKAVALQEEATREELDVEAAIAKSCQVPVQKFWGSTLYHRDD
IPFRIRELPNVFTEFRKKTENQSRVRPTFPMPSRLNPLPPGIDPGLTPSMEDMGMAEPVKDDRSAFPYSGGER
QALTRIQHYFWDYDYVATYKETRNGLIGADYSTKFSSWLALGCLSPRRIYEEIKKYEKERRANQSTYWVLFEL
LWRDYFKFVALKFGDRLFYLSGLLGNRREWRENKALFDAWREGQTGVPFVDANMRELAATGFMSNRGRQNVAS
FLTKDLKLDWRLGAEWFEHLLIDHDVCSNYGNWLYCAGVGNDPRQDRKFNMIKQGLDYDAQGDYVRLWVPELQ
GIKGGAVHTPWALSSSLLIQANVRLGHTYPMPIVKSPEWGKYTGSQERGKSGKGPGPRQQTRGLDFYFSSAKQ
RGHKK
>Acanthaster_pla_XP_022112081.1_cry-1-like
MPARPASGTDEDRKKGTIAVHWFRHGLRLHDNPSFIDAINLASELYPVFIFDGEVAGTKVCGFNRWRFLYEAL
EDIDRRLKEVGGRLYTFKGDPCTIFDQLIEEWGVNLITFEQDPEPIWQDRDNAVKRLCSERGIQYIERVSHTL
WDPNEVTARNGGTPPVTYAMFQEVVSMIGLPQRPEKEPDFSLVNLPICDNFGQFALPRFQDIGPKMEHPDQER
QLWIGGETRALELFEVRVRKEAAAFKAGYCLPNQYMPELLGPPMSLSPYLRFGCLSVRKFYWRIHDTYTELNN
AISPSHLTAQLIWREYFYTMSVGNKNFDRMNGNNICLKIDWNQDKEKLEKWIEGETGYPWIDACMKQLKIEGW
IHQVARHAVACFLTRGDLWISWEEGLKVFYQYLLDADWSICAGNWMWISSSAFEKVLQCPNCFCPVRYGRRMD
PNGEYVRKFLPVLANMPLRYLFEPWKAPLAVQEKVGCIIGKDYPMPIVEHRSAAASNREKMNKTVQSLEEKFA
HCTPSNETEVREFSWLPETIRGVDKCTADDLCEILGI
>Acanthisitta_chlo__KFP78511.1
RFLLQSLEDLDNSLRKLNSRLFVVRGQPTDVFPRLFKEWGVTRLTFEYDSEPFGKERDAAIIKLAKEAGVEVV
IENSHTLYDLDRIIELNGHKPPLTYKRFQAIISRMELPKKPVSTVISQQMETCKVDIQENHDDVYGVPSLEEL
GFPTDGLAPAVWQGGETEALARLDKHLERKAWVANYERPRMNANSLLASPTGLSPYLRFGCLSCRLFYYRLWE
LYKKVKRNSTPPLSLYGQLLWREFFYTAATNNPKFDRMEGNPICIQIPWDRNPEALAKWAEGKTGFPWIDAIM
TQLRQEGWIHHLARHAVACFLTRGDLWISWESGVRVFDELLLDADFSINAGSWMWLSCSAFFQQFFHCYCPVG
FGRRTDPSGDYVKRYLPKLKGFPSRYIYEPWNAPESVQKAAKCIIGVDYPKPMVNHAETSRLNIERMKQIYQQ
LSRYRGLCLLASVPSCAEDLSGPVTDSASGQGCSTSTVARLSQADQVSPKRKHEGVEEPCAEELYKRAKVTGF
PASEIPGK
>Acanthisitta_chlo__XP_009068753.1
KIKCSFTIPCSFLLQCLEDLDANLRKLNSRLFVIRGQPADVFPRLFKEWNIAKLSIEYDSEPFGKERDAAIKK
LASEAGVEVIVRISHTLYDLDKIIELNGGQPPLTYKRFQTLISRMEPLEMPVETITPEVMKKCTTPVSDDHDE
KYGVPSLEELGFDTDGLPSAVWPGGETEALTRLERHLERKAWVANFERPRMNANSLLASPTGLSPYLRFGCLS
CRLFYFKLTDLYKKVKKNSSPPLSLYGQLLWREFFYTAATNNPRFDKMEGNPICVQIPWDKNPEALAKWAEGR

TGFPWIDAIMTQLRQEGWIHHLARHAVACFLTRGDLWISWEEGMKVFEELLLDADWSVNAGSWMWLSCSSFFQ
QFFHCYCPVGFGRRTDPNGDYIRRYLPVLRGFPAKYIYDPWNAPEGIQKAAKCIIGVNYPKPMVNHAEASRLN
IERMKQIYQQLSRYRGLGLLATVPSNPNGNGNGGLMGYSPGESMSGCGSTGGAQLGTGDGHTVVQSCALGDSH
TGASGIQQQGYCPASSILHYAHGDNQQSHLLHAGRTALGTGISAGKRPNPEEETQSVGPKVQRQSTN
>Acanthisitta_chlo__XP_009072123.1
MQESGRAELEAAVPAPPGRSVGGHTAVCLLRCDLRAHDNQVLHWAQDNADFIIPLYCFDPRHYLGTHCYGFPK
TGPHRLRFLLESVKDLRETLKKKGSTLVVRKGKPEDVVHDLIAQLGSVSAVAFHEEATQEELDVEKGLCQVCR
QHGVKIQTFWASTLYHRDDLPFKPITRLPDVYTHFRKAVESEAKVRPTLRMAEQLKPLTPGVEEGCIPTMEDL
GQKDPVADPRTAFPCSGGETQALMRLQYYFWDMNLVASYKETRNGLVGMDYSTKLAPWLALGCISPRYIYEQI
QKYEKERTANQSTYWVLFELLWRDYFRFVALKYGKRIFSLRGLQSKEISWKKDLQLFDCWKEGKTGVPFVDAN
MRELVATGFMSNRGRQNVASFLTKDLALDWRMGAEWFEYLLVDYDVCSNYGNWLYSAGIGNDPRDNRKFNMIK
QGLDYDGNGDYVRLWVPELQGIKGADIHSPWALSSAALSQAGVTLGETYPQPVVTAPEWSRHINQRPGGSLHP
RGRRGPAQVPVQHKDRGIDFYFSRKKDVC
>Acanthisitta_chlo__XP_009083471.1
FKYNKKRVRLISQGSDLKDDARCILYGSGAPMAGKSPDNWAFLYAQRLALKQELPLHVCFCLVPKFLEATLRH
YGFMLRGLREVAEECAELNISFHLLLGYAKDVLPTFVVEHGVGGLVTDFSPLRLPRQWVEDVGQRLPEDVPFA
QVDAHNIVPCWVASPKQEYSARTIRGKIHAQLPEFLTEFPPIVHHPYPPSCPAEPIAWEACYSSLQVDHTVKE
VEWATPGTTAGLAVLKSFIAERLKSFSSHRNDPNKAALSNLSPWFHFGEFSTQRAILEVQKHRHKYKESVDAF
VEEAVVRRELAENFCYYNENYDSVQGAYDWAQTTLRLHAKDKRPYLYDLQELEQGTTHDPLWNAAQIQMVQEG
KMHGFLRMYWAKKILEWTRSPEEALRFAIYLNDRYELDGRDPNGYVGCLWSICGIHDQGWAERAIFGKIRYMN
YAGCKRKFDVDQFERRYAPAASQ
>Acanthopagrus_latu__XP_036936079.1
MSTARTVICLLRNDLRLHDNELFHWAQRNAEYIVPLYCFDPRHYVGTHNYNLPKTGPFRLRFLLDSVRDLRNT
LLNKGSNLVVRRGKPEDVVADLIKQLGSVSTVAFHEEITSEELNVEKRVKDVCAQMKVKVHTCWGSTLYHRDD
LPFHHISRLPDVYTQFRKAVETQSKVRPVFPTPEQLKPLPQGLEEGAVPTAEDLQQTEPLADPRSAFPCSGGE
SQALARIKHYFWDTDAVATYKETRNGLIGVDYSTKFAPWLAMGCISPRYIYHQIQQYEKERTANQSTYWVIFE
LLWRDYFKFVAVKYGDRLFQIEGLQDKSIPWKKDMKLFDAWKEGRTGVPFVDANMREVAMTGFMSNRGRQNVA
SFLTKDLGLDWRMGAEWFEYLLIDHDVCSNYGNWLYSAGIGNDPRENRKFNMIKQGLDYDNNGDYVRQWVPEL
QGIKGADVHAPWTLSSAALSHAHVTLGETYPTPIVMAPEWSRHVNKKPSGTGPSPRGKKGPSHTPKQHRDRGI
DFYFSKGKKQ
>Acanthopagrus_latu__XP_036938635.1
MSGKKRNSNSAAAGPRAKQQKVAAMKEEKMEEKRERAPGWLQDFLQQQRAEGKEIKLNKKRIRVISDTEKIKQ
GSEGVLYWMSRDHRVQDNWALIHAQRLAVKENLPLHVCACLNVPKSELSTLRHYSFMLKGLEEVAEECKRLNI
QFHLLHGTAGEVLPGFVSDRSLGAVVTDFSPLRGPLQWLEDIKKKLPKDIPLIQVDAHNVVPCWVASPKLEYA
ARTIRGKITKVLSDFLTDFPPVEKHPYTATRTAKPVDWEQTLASLEVDRIVGETEWAKPGTKAGTAMLESFID
LRLKIFDTKRNDPNTPALSQLSPWIRFGHLSAQRVALQVQHSGKKAGVTVSSYIEELVVRRELTDNFCFYNKK
YDSVEGAYEWAQKTLRDHAKDKRPYIYTREQLEKAKTHDKLWNAAQYQMVTEGKMHGFLRMYWAKKILEWTSS
PEEALSIALYLNDRYELDGQDPNGFVGCMWSICGVHDQGWAERPIFGKIRYMNYKGCLRKFDVARFERTYSPK
NL
>Acanthopagrus_latu__XP_036952501.1
MAHTCVHWFRKGLRLHDNPALMAALRDCKELYPVFVLDPNLHNRTCVGINRWRFLIGALKDLDRSLRKLNSRL
FVVRGKPEDVFPTLFNEWKVTKLTYEYDTEPYSVSRDKTVTALAKEHGVEVIYKVSHTLYDIDRIIEENNGKP
PLTYNRMQAIVKTLGPPKKPIPAPTMENMKDVQTPCSENHKKKYGIPTLEELDHDAAALGEDKFPGGEQEALR
RLDEHMKRTGWVCGFEKPQTSPNSLSPSTTVLSPYVTFGCLSARTFWWRLTDVYQGKKHSDPPVSLHGQLLWR
EFFYTASVGIPNFNKMEGNSVCTQVDWDTNPEYLAAWREARTGFPFIDAIMTQLRQDGWIHHLARHAVACFLT
RGDLWINWEEGQRVFEELLLDGDWALNAGNWQWLSASTFFHQFFRVYSPVAFGKKTDKNGDYIKKYLPLLKKF
PAQYIYEPWKAPHSVQQAAGCIVGKDYPHPIVQHEVISKTNIQRMKLAYSKRSADPAASPSKKQGVKRKASPV
VDMLKKKNKTN
>Acanthopagrus_latu__XP_036962465.1
MKDHQLSTPSQDSPLQQPGDRPNQRRSVQLASVSQPQGSSHAQLADVVGRRKNTQSSQGAPWVKSSQPQTTQE
TELQESAHVYKCNKETPGTCKAECVSRLSGSVSKQDTVEKGNQMMDEGELTESQKPKRSKNRRQRRKGAGQHV
VGLPCSPSAQPPVLLWFRRDLRLCDNPALIGSLEVGAPVIPVFIWSPEEEEGPGITVAMGGACKYWLHQALSC
FSLSLENIGSHLVFLKANRERSGVGSSVQTVKELVKETGARTVVANALYEPWLKERDDAVVSALQKDGVECKM
FDSYCLRDPYSVSTEGVGLRGIGSVSHFMNCCSQNPGSALGAPLDPPVSLPAPSNWPQGVSLDILGLARMPRR
KDGTTIDWAANIRKAWDFSEEGAHARLEAFLQDGVYRYEKESGRADAPNTSSLSPYLHFGQLSPRWLLWDAKE
ARCRPPKFQRKLAWRDLAYWQLTLFPDLPWESLRPPYKALRWSSDRGHLKAWQRGRTGYPLVDAAMRQLWLTG
WMNNYMRHVVASFLIAYLHLPWQEGYRWFQDTLVDADVAIDAMMWQNGGMCGLDHWNFVMHPVDAAMTCDPCG
TYVRKWCPELADLPDELIHKPWKCPASMLRRSGVVFGQTYPERVVSDLEERRTQSLQDVAVVRKKFGQYVDKR
SGCDLVPLPQRLVSEALGLSHSDGGVVTTGKQFLLPVITRMEFKHQLEDPDADAASNPYNAVLKGYVSRKRDE
TIAFLNERDFTASVMYEGTQRRERLESDYRRMEGLPRPAAPRGRARRTPTAKDRFSVVPGGAVTSLR

>Acanthopagrus_latu__XP_036963662.1
MVVNTVHWFRKGLRLHDNPSLRESIRGADSLRCIYILDPWFAGSSNVGINRWRFLLQCLEDLDASLRKLNSRL
FVIRGQPTDVFPRLFKEWQINRLSYEYDSEPFGKERDAAIQKLACEAGVEVMVRTSHTLYDLSRIIELNDGHP
PLTYKRFQGLIDRMDAVELPAETVTLEVIRKCDTPISDDHDDKFGVPSLEELGFDTDGLTTAVWPGGETEALM
RLERHLERKAWVANFERPRMNANSLLASPTGLSPYLRFGCLSCRLFYFKLTDLYRKVKKNNNPPLSLYGQLLW
REFFYTTATNNPCFDKMEGNPMCVQIPWDRNPEALAKWVEGQTGFPWIDAIMTQLRQEGWIHHLARHAVACFL
TRGDLWLSWEEGMRAFEELLIDADWSVNAGSWMWLSCSSFFQQFFHCYCPVGFGRRTDPNGDFIRRYLPVLKG
FPAQYIYEPWNAPEELQKSVKCIIGVHYPKPMVNHAEASRINIERMKQIYQQLSCYRGLGLLATIPVNPHNST
NRSNVGRVNAGTSRVPDRPSENLPMLGETSRTERLHAAQKRRCEDPPLESSCKSWRQSK
>Acanthopagrus_latu__XP_036963795.1
MVVNSVHWFRKGLRLHDNPALQEALNGADTVRCVYILDPWFAGAANVGINRWRFLLEALEDLDSSLKKLNSRL
FVVRGQPTDVFPRLLKEWKVTRLTFEYDPEPYGKERDGAIMKMAQEFGVETIVRNSHTLYNLDRIIEMNNNSP
PLTFKRFQTIVSRLELPRRPLPPITQQQMDKCCTKIADNHDQLYSIPSLEELGFRTEGLPPAIWRGGESEALD
RLNKHLDKKVWVANLEHPRVNTCSLYASPTGLSPYLRFGCLSCRVLYYNLRELYMKLRKRCSPPLSLFGQLLW
REFFYTAATNNPNFDRMDGNPICVQIPWDQNPEALAKWAEGRTGFPWIDAIMTQLRQEGWIHHLARHAVACFL
TRGDLWVSWESGMRVFEELLLDADWSVNAGSWMWLSCSAFFQQFFHCYCPVGFGRRTDPSGDYIRRYIPILKD
YPNRYIYEPWNAPESVQKAANCVVGVDYPKPMINHAEGSRLNIERMKQVYQQLSHYRGLSLLASVPTIQEEAE
PPMTDESQTSSGPDSPPRGPADSEAAGCSTAPDSSTVCASSTYALHPDLKDKGDNYPSKKPYTSSGSLTQCSA
AITSTSASHSPCTTAPLSPAQSPLSRSKPSSPSSSCPTFSESLSPTTTPTQTSSLGQRRKGLAHKVRRSQRQR
GRQSWTPAPREGGRKLEEEEREETGWEERMEEDMEQDEERMEEEPSEETAGRQQ
>Acipenser_ruth__RXM29285.1
MAEKPGKPRKTKDENGNAGKRDQKVVEGGRQAGSSSGREAKAARKQEPGKRETTLWGSSGDELSNTGARKKRR
LGGGDAVTSPPGGVGGLASSIKESRLQAGPSVKEFEFNKKRVRLISEAVEMKDGSEGIVYWMSRDQRVQDNWA
LLYAQHLALSHELPLHVCFCLVPRFLDATIRHFGFMLRGLEEVAKECAELNISFHLLTGYAKDVLPEFVQMRG
AGGVVTDFSPLRVPLQWVQDVKERLPRDVPLVQVDAHNIVPCWVASEKQEYGARTIRKKIHDKLSEYLTEFPP
VLRHPHGAGCEVEAVDWEQARSSLEVDRSVKEVDWARPGTEEGLAMLESFIKERLKLFGSERNNPNRPALSNL
SPWFHFGQVSVQRAVLEVRKCGSKHWQSVHSFVEEAVVRRELADNFCYYNKNYDRVEGASDWARKTLQDHAQD
ERSHLYSRAQLEEGKTHDPLWNAAQMHGFLRMYWAKKILEWTSCPEEALETALYLNDRYELDGRDPNGYVGCM
WSICGIHDQGWGERPVFGKIRFMNYAGCKRKFDVGQFERKYHPEKSTV
>Acipenser_ruth__RXM33382.1
MVVNTVHWFRKGLRLHDNPSLRESIQGADSIRCVYILDPWFAGSSNVGINRWRFLLQCLEDLDASLRKLNSRL
FVIRGQPADVFPRLFKEWNINRLSFEYDSEPFGKERDAAIKKLACEAGVEVMVQISHTLYDLDRIIELNGGQP
PLTYKRFQTLISRMKTLETPVESITVEVMGKCTTPVSDDHDDKFGVPSLEELGFDTEGLPSSAVWPGGETEAL
TRLERHLERKAWVANFERPRMNANSLLASPTGLSPYLRFGCLSCHLFYFKLTDLYKKVKKNSSPPLSLYGQLL
WREFFYTAATSNPRFDKMEGNPICVQIPWDKNPEALAKWAEGRTGFPWIDAIMTQLRQEGWIHHLARHAVACF
LTRGDLWLSWEEGMKVFEELLLDADWSVNAGSWMWLSCSSFFQQFFHCYCPVGFGRRTDPNGDYIRRYLPILR
GFPAKYIYDPWNAPESVQKIAKCVIGVHYPKPMVNHAEASRLNIERMKQIYQQLSCYRGLGLLASVPSNPSGN
GNGGMMGLSPGEDMHGGTPVEVGKGNAGMQQQVTRQYTPV
>Acipenser_ruth__XP_033851735.2
MKHSSIHWFRKGLRLHDNPALLAALRDCAEIRPVFILDPWFPRNTRVGINRWRFLLDALKDLDGSLRKLNSRL
FVVRGKPTETFPRLFRQWKVTQLTFEMDTEPYSRQRDAEVVRLAEEHGVEVIQKVSNTLYDSERVIVENDGTP
PLTYKRLQTVLSFLGLPKRPVPAPKLEDMKDVHTPCSDSHEEEFGVPTLEELGQDPLKAGPHLYPGGEGEALR
RLDQHMQRVAWVCNFKKPETEPNTLTPSTTVLSPYMKFGCLSARTFWWRLTDVYRGKKHSEPPVSLHGQLLWR
EFFYTAGAGIPNFHRMEGNSVCVQVDWDTNEEHLAAWREGRTGYPFIDAIMTQLRSEGWIHHLARHAVACFLT
RGDLWISWEEGMKVFEDLLLDADWSLNAGNWQWLSASAFFHQFFRVYSPVAFGKKTDKKGDYIRKYIPLLRKF
PVEYIYEPWKAPLSIQERAGCIVGRDYPRPIVEHDIVSKRNVQRMKAAYARRAAPDSETRAVKTGAKRKADSV
ADRQRN
>Acipenser_ruth__XP_033909724.2
MVVNSVHWFRKGLRLHDNPALQRALEGANTVRCVYFLDPWFAAGTTDVGINRWRFLLESLQDLDTSLRKLNSR
LFVVRGQPANVFPSLLKEWNVTRLTFEYDSEPFGKERDAAIIKMAQELGVEVAVKNSHTLYNLDRIIELNKNS
PPLTYKRFQAIISRLELPKKPVGTVTGEQMERCRTGITENHDEHYGVPSLEELGFKTASLGPAKWKGGETEAL
RRMDKHLERKAWVASFERPKIDVNSLIGSQTGLSPYLRFGCLSCREFYYRLLELYKKVKKSNSPPLSLYGQLL
WREFFYTAATNNPKFDRMQGNPICVQIPWDRNPEALAKWAEGCTGYPWIDAIMTQLRQEGWIHHLARHAVACF
LTRGDLWISWESGMRVFEELLLDADFSVNAGSWMWLSCSAFFQQFFHCYCPVGFGKRTDPSGDYIRRYIPKLK
KYPNKYIHEPWNAPESVQKAARCIVGVDYPKPMIKHAEASRLNIERMKQIYQQLSHYKGLSLLASVPSFSEEL
GSTITDRLHGDSSNSGSSMVWHSRTSPLGKRDRPSEPDCEEKCTRPKILCGCPTNGTNIQELQVENSKRMQKN
>Acropora_digiti_XP_015758632.1
MKDNPVCLQVNWDKNEEFIHAWTEARTGYPFIDAIMTQLRREGWIHHLARHAVACFLTRGDLWISWEEGLKVF
ENLLLDHDWSLNAGNWLWLSASAFFHAYFRVYSPVAFGKKTDPNGQYIKKYLPALKKYPAKYIYEPWKAPLTV

QQAAGCLIGKDYPKPIVDHTKVVKVNLAKMKDAREKQYGGSSEQGAKKDEKSSSLKRKMREKPPSPEKKMKKI
TDFLKG
>Acropora_dig_XP_015760686.1
MSLNLKSVEDNNSVVGAEKRQGKLKAKHAIHWVRKDLRLHDNPSLLEAVKGSDTVRIIYVLDTKVDHATGIGL
NLWRFLLQSLEDVDDSLRKLNSRLFVVRGQPADVFPRLFREWKTSFLTFEEDSEPFGREKDAAIRLLAQESGV
EVAVGRSHTLYDPQLIIKHNSGTAPLTYKKFLAIVRSLGNPEHPCATLDVHLLGGCSTPVSEDHEEKFGVPSL
KELGLDVAKLSTEIWHGGETEALIRLDRHLERKAWIASFEKPKVTPNSLFPSPTGLSPYLRFGCLSPRLFYHR
LSELYRKVKCKDPPISLYGQLLWREFFFTVAANNPNFDQMDENPVCLQIPWTANPEWLKKWEQVFMHFL
>Acropora_dig_XP_015763606.1
MASQPPAKRRKEESSENGDDILASCMAKRAQVCASVADFKFNKKRVRLLSKSTDISDDCKAIAYWMWRDQRVQ
DNWALLYAQRMALKQEVPLVVCFCLPSKYLHSTFRQYSFMIKGLQQVEKELASLNINFHMLLGEPNIVLPDFV
KSENIGGIIADFTPLRKPMKWLNDVMDKLPKNVPVCQVDSHNIIPCWQASPKLEYGARTIRPKIHNQMREFLT
EFPPVIKHPHAGKASIKTDWKAADEFIEVDRNVQEVNWAEPGTKAGLEMLESFCKDRLKFFATDRNNPTKEAL
SDLSPWFHAGQISVQRAILRVREFRGSSKDSVETFIEEAVVRRELADNFCFYNQEHYDSIKGAKDWAQKTLND
HAADKREYLYTREQLEKGQSHDDLWNAAQLQLVNEGKMHGFLRMYWAKKILEWTESAEEGLEISLYLNDKYSL
DGTDPNGYVGCMWSVCGIHDQGWAERPVFGKIRYMNYNGCKRKFDVGEFVRKYGATTSKEADGPAKKRKKAK
>Acropora_dig_XP_015773600.1
MEDKKPRTSLHWFRKNLRLHDNPALCEAIKESDDFLALYILPPSVPDGNIAANRWNFLIECLEDLNRSLASVG
SRLLVVQGYPAQVIPKLLKRLHVTKLTFESESEPFSKQRDAVITHLAESMGVEVVSCTSHTLYDVGKVLEANN
NVTPMLFDSFLDVVATLGPPEPPTQTVSQDMMSSLLHKANMFGDCDIPAVEDLGIDRAKVTCQEIWCGGESEA
LQKLHAFLKELVDVDFQVESVSELFLHGFTDKLSPYLRFGCLSPRTYYHALLDTYYTAKKQYPPQSLFFSLLW
RDFYFVLGSSNPTLNQMENNPLCLQINWDQNVVALEKWKMGQTGFPFVDAIMRQLCKEGWISSLARQVVGSFL
TQGCMWMSWEEGFKIFGELQLDAEWSLSAGSWLSISGSSFDQNRLALFCPVETAKKLDPMGEYIRKYVPEVKD
LPLEYVFEPWTAPSAVQEAAGCVLGRNYPLPLADHKEQQKVCMQRLKELAQNLGTDMRGRERGHTSVHLFRKD
LRLHDNPSLCSCLEGSTTFYPVYVLDTEAARKSKISPNRWNFLLECLRDLDDQLAALGSRLFVVRGREVEVIS
KLIQEWGVTRISFESDSEPFGVQRDAVIQKLAEEAGVEVLSKTSHTLFKPEDIIHANHGVAPMIFEDFLEVLK
DNLLKAPSPVKQVDRQLFGCCVTPVADHQTNFGLPELSELGVRDTRLVTSSCLWKGGEQEALRRVLLFEKQVP
NTVISMRFFMQVWEDSVEYLKRWKKGTTGFPWIDAIMRQLRTEGWIHNIARRAVASFLTRGCLWVNWEEGFKV
FDEFQLDAERSLNVGNWLWVSTSTFVKGPVPWFCPVGVGKKIDPTGEYVRKYVPEIRNLPTEYIFEPWLTPRD
LQRSYGCVIGRDYPAPIVNHVKQRAICVQRMQELAFKLASK
>Acropora_dig_XP_015775701.1_cry_DASH-l
MFHVPRCSNCFLVCSSLLRRRGSFRSVAASRRIKVTLAMDAQTGNSIAIYLIRNDLRVHDNECLSWAQQNADF
VIPLFCFDKEIFGHGAETWHFKFPKTAIHRARFILESVVDLKDSLKKGGSDLLLRSGQTMKEAVLGVIQLCHQ
QSIQNLSLVYQREITKEEKDVEAELLELCKKENVAVKSFWGLTLYHVEDLPFASVRHLPDTYTEFRKSVEARG
RVRPVIPAPNRLKPIPDFITSDNMGGIPTLTELVKDQGTTPDARSAFPFCGGETAAIERMDNYLWTTDNVSKY
KETRNGLIGAEYSTKLSPWLAVGALSPRKIYECVKQYEKERTANQSTYWVLFELLWRDYFKFVCFKFGDSVFY
LSGIMKKGGLSWKQDMSSFDKWRFGQTGVPFVDANMRELLYTGWMSNRGRQNVASFLVKDLGLDWRLGAEWFE
SLLVDHDVCSNYGNWNYSAGIGNDPRENRKFNMIKQAFDYDADGDFVRLWVPELVDLKGARVHIPWTLTSSEL
KVAGITLGESYPRPMVNPPEWKRHTSKVKGSGNSTRRHNRGIDFYFKSPKDTNPSKKRH
>Acropora_mil_ABP97099.1_cry_CRY2,_
ICVQRLKDLCFSLDILGHTSLHWFRKDLRLHDNPSLRECLRNSKVFYGVYFLPPSEAKQGSVSLNRWGFLLES
LRDLDTSLVECGSRLFVIRGNPVEMLPNLFKKWNINQMSFEVDSEPYSNSRDLVISHLAKENGIEVISRVSHT
LYDPRILRGLSSGIVPLLFDEFKESVLQKRQPEKPVPKVGRKLFGACVTPVGSDHQQCYGVPRLDEIGGKFSK
SGSVCSELYKGGESEALKRMETALQWMVKNDFTEPEITIHSLLXSATHLSPYLRCGCLSPRLLHQRITEEYIK
SKGVSPPSELFVKLLWREFFFVVGGQIPDAHEMINNPISLEIPWEDSVEYLERWKKGTTGFPWIDAIMRQLRT
EGWIHNIARRAVASFLTRGCLWVNWEEGFKVFDEFQLDAERSLNVGNWLWVSTSTFVKGPVPWFCPVGVGKKI
DPTGEYVRKYVPEIRNLPTEYIFEPWLTPRDLQRSYGCVIGRDYPAPIVNHVKQRAICVQRMQELAFKLASKI
RWRQVLNVI
>Acropora_millep_XP_029185027.1
MATKTSRVHNTIHWFRKGLRLHDNQSLMEACETSVTLRPVFFIDPDYVKHGNMGFNRWRFLIESLNDINDSLE
KIGSRLFVVHGKPEIEFPKLFRKWEVTKLTFEFDHEPDGRARDSKITKLAAQNGIEVQTRICHSLYDLDEIIE
KNDGRAPLTYKRLCSVVSSLGPPSKPVPTVDQETFKGCSTPASESDEKDYHVPTLEELGIERPHQTSGILFPG
GETEALRRLNEHMEKEDWVNKFEKPNTSPNSLEPSTTVLSPYVMFGCLSARLFYHKLSEVYARARKHSQPPVS
LHGQLLWREFFYTVAYGTPNFNKMKDNPVCLQVNWDENEEFIHAWTEAHTGYPFIDAIMTQLRREGWIHHLAR
HAVACFLTRGDLWISWEEGLKVFENLLLDHDWSLNAGNWLWLSASAFFHAYFRVYSPVAFGKKTDPNGQYIKK
YLPALKKYPAKYIYEPWKAPLTVQQAAGCLIGKDYPKPIVDHTKVVKVNLAKMKDAREKQYGGSSEQGAKKDD
KSSSRKRKIREKPPSPEKKMKKITDFLKG
>Acropora_millep_XP_029212918.1
MFHVQRCNNRFSVFSSLLRRGGSFRSVAASRRIKVTLAMDAQTSNSIAIYLIRNDLRVHDNECLSWAQQNADF
VIPLFCFDKEIFGHGAETWHFKFPKTAIHRARFILESVVDLKDSLKKGGSDLLLRSGQTMKEAVLGVIQLCHQ

QSIQNLSLVYQREITKEEKDVEAELLELCKKENVAVKSFWGLTLYHVEDLTFASVRHLPDTYTEFRKSVEARC
RIRPVIPAPNRLKPIPDFITSDNMGGIPTLTELVKDQATTPDARSAFPFCGGETAAIERMDNYLWTTDNVSKY
KETRNGLIGAEYSTKLSPWLAVGALSPRKIYECVKQYEKERTANQSTYWVLFELLWRDYFKFVCFKFGDSVFY
LSGIMKKVGLSWKQDMSSFDKWRFGQTGVPFVDANMRELLYTGWMSNRGRQNVASFLVKDLGLDWRLGAEWFE
SLLVDHDVCSNYGNWNYSAGIGNDPRENRKFNMIKQAFDYDADGDFVRLWVPELAGLKGAKVHIPWTLTSSEL
KVAGITLGESYPRPMVNPPEWKRHTSKVKGPGNSTRRHNRGIDFYFKSPKDTNPSKKRH
>Acropora_mil_XP_029187851.1_photolyase
MASQPPAKRRKKESSENGDDILASCMAKRAQVCASVADFKFNKKRVRLLSKSTDISDDCKAIAYWMWRDQRVQ
DNWALLYAQRMALKQEIPLVVCFCLPSKYLHSTFRQYSFMIKGLQQVEKELASLNINFHMLLGEPNVVLPDFV
KSENIGGIIADFTPLRKPIKWLNDVMDKLPKNVPVCQVDSHNIIPCWQASPKLEYGARTIRPKIHNQMREFLT
EFPPVIKHPHAGKASIKTDWKAVDEFIEVDRNVQEVNWAEPGTKAGLEMLESFCKDRLKFFATDRNNPTKKAL
SDLSPWFHAGQISVQRAILRVREFRGSSKDSVETFIEEAVVRRELADNFCFYNQEHYDSIKGAKDWAQKTLND
HAADKREYLYTREQLEKGQSHDDLWNAAQLQLVNEGKMHGFLRMYWAKKILEWTESAEEGLEISLYLNDKYSL
DGTDPNGYVGCMWSVCGIHDQGWAERPVFGKIRYMNYNGCKRKFDVGEFVRKYGATTSKEAGGPAKKRKKAK
>Acropora_mil_XP_029194452.1_cry-1-like
MSLNLKSVEDNNSAVSAEKRQGKLKAKHAIHWVRKDLRLHDNPSLLEAVKGSDTVRIIYVLDTKVDHATGIGL
NLWRFLLQSLEDVDDSLRKLNSRLFVVRGQPADVFPRLFREWKTSFLTFEEDSEPFGREKDAAIRLLAQESGV
EVAVGRSHTLYDPQLIIKHNSGTAPLTYKKFLAIVRSLGNPQHPCATLDVHLLGGCSTPVSEDHEEKFGVPSL
KELGLDVAKLSTEIWHGGETEALIRLDRHLERKAWIASFEKPKVTPNSLFPSPTGLSPYLRFGCLSPRLFYHR
LSELYRKVKCKDPPISLYGQLLWREFFFTVAANNPNFDQMDENPVCLQIPWTANPEWLKKWEQGQTGFPWIDA
IMIQLKQEGWIHHLARHAVGCFLTRGDLWISWEEGMKVFERWLLDAEWSLNAGNWMWLSCSAFFQQFFNCICP
VGFGRKLDPNGDYVRKYLPVLKGFPAKYIHAPWTAPENVQRAARCIIGKDYPRPIVDHHKVSTANLEKMRNVF
KALLRYKESTVVATSEKSDGSKDKQAKLKQQMLNLEDKENR
>Actinia_ten_AOQ25781.1_photolyase
MCSVQVNRPVILALYTEFKRVLHLFNLPNKALYTRKPRSCWKSSVLLRFLSKSYESNPSNAIKTMTEPPNKKA
KKEHKLDDSADDDDILRRYHKKRKAVCESVLDFKFNKKRVRLLSKVTEVNDDCKGIVYWMWRDQRVQDNWALL
YAQRLALKQQVPLHVCFCLPKTFLDAAIRKYGFMLKGLKHVEKELSELDISFHLCLGEPDIVLPELIKKFNFG
GVVCDFCPLRKPMQWVDKLIKALPKSIPVCQVDAHNIVPCWETSPKLEYGARTIRPKIHKVLQSFLTEFPPVI
KHPHVPKVKVKETDWKAAYDFIDVDDTVKEVDWAEPGTEAGLKMLEEFCAQRLKHFATSRNDPTKKAISNLSP
WFNTGQISNQRTILKIREFRGKYKESVESFIEESVVRRELSENFCYCNDNYDSIDGTNDWAKKTLEDHAGDKR
EPLYDRETLETAKTYDDLWNAAQRQLVCEGKLHGFLRMYWAKKILEWTKSPEVALADAIYFNDKYALDGIDPN
GYVGCMWSICGIHDQGWKERPIFGKIRYMNYKGCQRKFDVAAFVKRYKP
>Actinia_ten_XP_031549143.1_cry-1-like
MAGKKAHNTIHWFRKGLRLHDNPSLKEAFESSVNIRPIYVLDSDDVQHGNIGVVRWRFVLQSLEDLDRSLRKL
NSRLFVVRGKPTVLFPKLFKEWNITKLTFEVDTDGPARQRDAEIIKLANDAGVDVVQKVSHTLYNIDDVLGKN
LGRAPSTYKSFQAVISSLGPPEVPIPTIEETDVGVCKTPIEDDHDGLYGLPTLEEIGLTVXDESKEVLYPGGE
TEALRRMHEYMAKEDWVCKFEKPNTSPNSLEPSTTVLSPYLTFGCLSARLFYVQLSDIYSRAKKHSQPPVSLX
GQLLWREFFTTCAYNIPNFNRMEGNPVCLQIPWDKNDEYLKAWEEARTGYPFIDAIMTKLRREGWIHHLARHA
VACFLTRGDLWISWEEGLKVFENLLIDHDWSLNAGNWMWLSASAFFHAYFRVYSPVAFGKKTDPNGDFIRKYI
PILKRFPAKYIYEPWNSPLNVQEKAGCMIGKDYPRPIIEHSKAVAKNLARMKKAREAKYGSKESNPEKATKTS
PSKRKSTSKDDKTAKKVRKITDFTT
>Actinia_ten_XP_031550954.1_cry-2-like
MVNSLHWFRKGLRIHDNQPLRDAIQXSTTFRAVFFLDRTTVRNAKVSVNRWRFLLECLKDLDNKLKEFGTRLY
IVQGHPIDVFPRLIKEWGITRLTFEYDSEPYPSQRDLAVRRLAENEGVEVIVKSSHSLFDISRFVKENNGQAP
ITFKQFHSLLRKVGLPEACVETVDKELFGSCVMPIEDDHDEKFGIPSFEELGLAVEEEKGPGVWVGGESQALK
VFQTAIKELFESNEKNSKYSDDSPFPKSTKLSPYLRFGCISPRLVFHKMTKLASKVSNPAACFGIQKSLLWRE
FFFVIASKNPALDKMCNNCMCLQFPWDKNNNSLERWKNGKTGFPWIDAIMRQLRKEGWIHHLARQAVGCFLTR
GCLWVSWEEGFKAFEEMQLDAEWSLNASCWLWLSCSAFFNSTPTPWFCPVNFGKKLDPAGNYVRKYVPEVRNL
PTEYIFEPWLAPVEVQRQAGCVIGTDYPKNILDHDIARKICSNRMRDVYNGLVVKVSSAPGLGHNVVHWFRKD
LRLHDNPALFEALPNCRTFYAVYIFDPVNIKAAKVSANRWNFLLECLRDLDNNLKKCGTRLHVLRGQPTTIFP
KLFREWNITKLTLESECEPFGQQRDEAIASLAEEYGVEMSSRSSHTLYSIDKILEMNGGKCPTLFKEFESIIR
KLGPPDSPVDPVTRKCFSACICPINGAHEARYAVPTLSELNIDESQVTCGDLWVGGESEALTRLTELEEKIFI
GTKRESPESAAALLPSRTQLSPYLRFGCLSPKMYYKRLTMAYIKMKSKPPPMSLYCQLVWREFFFTLASKNPN
MDTIENNPLCLEIGWEHNEEALTCWKEGKTGFPWIDAIMRQLRQEGWIHHIARQAVGCFLTRGCLWVSWEEGF
KVFEELQLDAEWSVNSGSWLWLSCSAFFHSQIPWFCPVSVGKKLDPSGNYIRKYIPELRTMPNKYIYHPWTAP
EGIQKAANCVIGKDYPKPIXDYVERRKVCLQRLKEVCCQIKTTTESAICTT
>Actinia_ten_XP_031553703.1_cry-1-like
MEENDSSQRRFQKTNSLYWARKDLRLHDNPALREALLCCDTFRVVYILDTKSVEGSRIGLNLWRFLLQSLEEL
DNSLRKLKSRLFVVRGQPADVLPKLFLEWNITKLTFGEDTDPFGKERDSAIQMLAREAGIDVIVRQSNTLYDW
PELIYQNGGKLTCSYKSFLNVIQTMAPPDLPEETITCNSPGLGYTPLDEDCFDKYEVPSFEELGLDSSKLCTE

AWHGGEIEALNRLNRHLERKAWIASFEHPKITPNSLHAMAHSVSPYLRFGCLSPRLFYSRLTDLYEKVKRRKP
SISLYGQLLWREFLYMVSTNNPNFDKMHDNPYCLQIQWRDDNCVEEDLQKWKLGQTGFPWIDAIMAQLRQEGW
VHPMACYATACFLTRGALWISWEEGMKVFEEYLLDADWSVNAGNWMWYSCSAFSQHFYLPQCPVEFGKKLDEN
GDYIRKYLPILKGFSNEYIHEPWKAPCRVQKIARCMIGKDYPSPMFDQEEKRKQNMERIQKIIDSQQPSIEIQ
QC
>Actinia_ten_XP_031561645.1_cry_DASH-l
MASKSRVIICLLRNDLRYHDNEVLCWAHKNADLVLPLYCFDPRHFRGTWHFGFPKTGPFRTKFLLESVRDLRQ
KLQDVASNLVVRCGKPEEIIPSLITSINNESVKVEGISLQKEPTKEEIDVENAIEARSSVPVQKFWGLTLYHP
DDLPFSIKKVPNVYTEFRKNVERHSGVRSTIKMPDRMKPLPDGIEPGNIPTFDELGVKEPSQDSKSAFPFQGG
ETSGLQRLQYYLFDTDYVAKYKETRNGLIGQEYSTKFSTWLAHGCLSPRKIYEEIKKYEHQRTSNQSTYWVIF
ELLWRDYFKFVAIKFGDRLFYLSGLLAKKVEWRQDENLFRAWKEGKTGVPFVDANMRELAATGFMSNRGRQNV
ASFLTKDLKMDWRLGAEWFEYLLIDHDVCSNYGNWLYSAGVGNDPRQDRKFNMVKQGLDYDPKGDYVRQWVPE
LSGVPGGDIHTPWRLSRASLAHASVSLGETYPMPIVMAPEWDRHSTKPKRDLSSQKRQSNQRKQKRGIDFYFS
SNSNNH
>Acyrthosiphon_pis_NP_001164532.2_chryptochr
MTVAVHWFRNGLRLHDNPALIEAHNNAEKLITLFIFDETTFNPKWYGYNPMRFLLESLIDLNNNLALVGGRLY
ILQGNPVNIFKMIKEKIGLHFITYEQDCAHLGRTRDEKVKSFCDENDIKCIETVSHTLWNPKSIIEKNGGVPP
FTFKQFQNTAKQIGHPPSPVGNVDWLSVIFEELPASIQDEFKNLHNPTPETFGIYPEVPENLTSTYRWYGGET
RALEQLKERLEYEKEAFVNGFYLPNQVNPDLLSPPSSLSAALRYGCLSIRKFYWELTKLFINKFEGDLLPMYS
VTSQLIWRDYFYTMSIDNKNFGQIEDNPACISIPWNDIKIPENKKMLECWKTGKTGYPFIDAGMRQLMQEGWI
HHVVRNSLASFLTRGDLWISWVEGLNHFMKYLLDADFSVCSGNWIWVSSSTFEQLLDCPLCVCPVSYGLRLDP
SGEYIKRYVPELKNMPAQFLYEPWKCPESVQKQVGCIIGKDYPNCIVDHTIASKGNRKKMLALRVSMTNENRV
PHCCPSDREEVQKFMYLPDECMQQLLPLENQDSKAYDIYKSH
>Acyrthosiphon_pis_NP_001164573.1_chryptochr
MQNKHTVHWFRKGLRIHDNPSLREGLINAKTFRCIFILDPWFAGASNVGINKWRFLLQCLSDLDNSLKKLNSR
LFVIKGQPAEALPKLFRQWGTTNFTFEEDPEPFGRVRDQNIKVMCSEMGISVITRCSHTLYQLDKIINVNGGK
APLTYHLFQKLLECIDPPERAVPSIDKEFLGNAFTPIKYDHDEIFGVPTLEELGFKEINNITRHVWVGGETEA
LIRLQCHLERKAFIASFGKPKMTPQSLVASPTGLAPYLKFGCLSTRLFFSELNELYKKIRKSQPPLSLHGQLL
WRDFFYCASTNNPNFDRMVGNPICVQIPWDKNPRALSKWANGQTGYPWIDAIMIQLRQEGWIHCIARHAVACF
LTRGDLWLSWEEGMKVFDELLLDADWSVNAGYWMWYSCSSFYQEFIHCYCPVRFGRKVDPNGDYIRRYIPALN
NMPNQYIHEPWLAPESIQFSANCIIGIDYPLPIVNHINASKINLERMKLAYQQLSNCQPQLENGKLILISSLR
R
>Acyrthosiphon_pis_XP_001946012.1_cry-2
MDKNDADVGRETTVHWFRKGMRLHDNPAFKLSCEAKNSNGERYKLRPIYILDPYFRKYIRAGANRWRFLQQSL
VDLDTTLRKLGTRLYVIRGLPHEVFPDLFAKWNVKLLTFELDTEPYARERDNQVEQLARKHGVKVEQKVSHTI
YNTELVLRANGGSVPMTYQKFVSVVGSMPTPRRPIPAPDMLPSECLLDDDLNNPEFDVPTLDELLTLKGFNPA
ELKPCLYPGGEKEALRRLEEYMKNKTWVCKFEKPNTSPNSLKPSTTVLSPYMKFGCLSASHFYYRLKEVIGNS
PHSKPPVSLIGQLYWREFYYTVGASTPNFDKMVGNSICCQVPWDNNPDALEAWTNGKTGYPFIDAIMRQLRDE
GWIHHLARHAVACFLTRGDLWISWEKGLAVFEELLLDADWSMNAGNWMWLSASAFFHQFFRVYSPVAFGKKTD
KSGDYIRKYIPELAKYPDQYIYEPWSAPKSIQERAGCVVGVHYPKRVVVHEDVYKNNIAKMSLAYKSTKAGKS
SNTKKSRDMSTSPDKKNIKKPKLK
>Acyrthosiphon_pis_XP_001949151.1_photolyase
MSGNEPLTKKFKKTISEVGTSKTSNFLNDVAAERNKTASSIMEFKFNKKRVRVLSEQKEVPEWAEGVIYWTFR
DERIHDNWALLYAQKLAIKNKVSLHITFCRLKQFLDCSLRHYKHIFQGLEELETECKSLDIQFHFLIGCAADI
LPDFVKKHKLGAIIVDFMPVREHISWTKQLADRIGSEVPVIQVDAHNIVPCWVASDKQEYSARTIRNKINNKL
PEFLTEFPPVIKHPFRSTFKAKPTNWDEADKTLEVDRSVVSVPGLKAGFKAGMSELEQFLKKRLPKYSTDRNN
PVKDGLSKLSPWLHFGQISAQRCILEVSKLSKQYPDSVAAYREEAIVRRELSDNFCFYNPKYDKIEGAPNWAQ
TSLNEHRKDKRMFVYTREELESSRTHDDLWNSAQIQLVKEGKMHGFLRMYWAKKILEWTDTPDRALADAIYLN
DKYSMDGRDPSGFVGCMWSICGIHDQGWKEREIFGKIRYMNYAGCKRKFDINAFIARYGGMVHKYTKK
>Adineta_ricc__CAF0888048.1
MKRLAIVLFRYDLRLHDNELLVWAHANHDYVIHTYCFDPRQITDKTYKCDLVKCDKYRLKFLIETIEDLNISL
VNRGSDLQTYRDTPENVLGQLVQQFKGEFEISIGYHQEATKEETDVEKAIQQLAKDNKLHVKEFWTSTLYHPD
DLPYNNPKAFPDVFTQLRVALEKQHVQVRRLVNPPNAFKSPPDGLLKTTIPSLLDFGYTNVTLHSNSVFILKG
GESSGLAHLRSYIWEKNLVHSYKQTRNSLIGPENTTKFSAWLANGSLSPRQIFFELKSYESHQGQRDAGYWII
FELLWRDFFKFIAMKYGARLFYSRGLRQEPYAWKHDIKEYEAWRTGNTGIPFVDANMREIMATGWMSNRGRQN
VASFLTKDLGIDWRYGAVWFESILIDHDVCSNYGNWIYVAGIGNDPRENRHFNMVKQGFDYDPAGVFIRTWCP
ELAQLSNEYIQTPWLAPTHVLAEAGVELGVNYPRPMLVVPQWNQQFKNRRTTTQNQHHTQQRGINFYFRNHDK
RQ
>Adineta_ricc__CAF0918551.1

MPKRKSTMPTVDKREKKVSESDHNQTSEKDDNAEEKQKDELANKVAKTKTNTTVIDYFSIKPLIKAIDHVRTG
ICTSVEDFHFNKTRVRPMNNVNHIPKDSQTILYWMSRDQRVQDNWAMLYAQRLALKQHLPLHVAFCLVPTFLA
APLRAYRFMLKGLREVEQECQQLNIIFHLLTGQAEKVLPKFVEKYQVGTIITDFSPLRVPRQWVENVAKKITT
IPIVQVDAHNVVPCWYASTKLEYGARTIRNKLHKNMDEFFTEYPPVIKHPHTSPAHHQVKPTDWQAADDSLQV
DRTVDEVAWAVPGYTGGIEQLESFVNERVCNFALGRNDPNKGAISNLSPWIHYGQISPQRALLVIAKLRPKFK
EACDSFIEEAFVRRELGDNYCYYQENYDNINGAWDWAKKTLNDHRNDKRTHVYSRDDLEYARTYDKLWNASQI
QMIKEGKMHGFVRMYWAKKILEWTNSPEEAIEIAIYLNDKYNLDGRDPNGYVGVMWSICGIHDQGWRERPVFG
KIRYMNYDGCKRKFDVAKYENKYSSL
>Adineta_stei__CAF0714394.1
MKRLGIVWFRDDLRLHDNETFAWAHMNNDYVIHIYCFDPRQITVKTYKCDFVKTDKYRLKFLIETIEDLNTSL
INKGSGLLTYRDTPENVLNQLIEQFKNIFEITIAFQQQVTQEETDVEKIIRQLTIDNNIHVKEFWTLTLYHPD
DLPYNNPKAFPDVYTQLRVALEKQGVRVRRLIDIPNTFKPLPDGIISTTVPNLSDYGYSNETIHTNSVFTLQG
GESSGLTHLHNYIWNKNLIKSYKETRNSLTGPENSTKFSPWLSNGSLSPRKIFFEIKKYENEQNIHDAGYWII
FELLWRDFFKFIAMKYGTRLFYGRGLKLEPYVWKHNSNEFEAWRTGNTGVPFVDANMREIMATGWMSNRGRQN
VASFLTKDLGIDWRYGAVWFESILIDHDVCSNYGNWIYVAGVGTDPRENRHFNVVKQGFDYDPNGIFVRIWCP
ELARLPNEYIQTPWLAPAHILKEAGVELGVNYPRPIIIVPQWNQQFQNRRKSIQNQHHTQQRGINFYFKNHEK
R
>Adineta_stei__CAF0758554.1
MSKRKGTMPTINKREKKTSESDNNTSEKEDHFENEEEEQKNELAEKVTNTKTNTTIIDYFTIKPLQSAIDNIR
KNICSSVNDFHFNKTRVRPMNNMNIIPQDSQTILYWMSRDQRVQDNWAMLYAQRLALKQQLPLHVAFCLVPTF
LAAPLRAYRFMLKGLREVERECQQLNIAFHLLTGQAENVLPKFIEKYKVGTIITDFSPLRVPRQWVDNVAKKI
TTIPIIQVDAHNVVPCWHASPKLEYAARTIRNKLHVNMTEFFTEYPPVIKHPHVSPTHLQAKPTDWQAADDSL
QVDRTIDEVSWATPGYTGGIEQLETFINQRVANFAIGRNDPNKCVISNLSPWLHYGQISPQRALLIMAKLRSK
CKEACDSFIEEAFVRRELSDNFCYYQPNYDNINGAWEWAQKTLNDHRNDKRTHIYSRDDLEYARTYDKLWNAS
EIQMIKEGKMHGFLRMYWAKKILEWTNTPEEAIEIAIYLNDKYNLDGRDPNGYVGIMWSMCGVHDQGWKERPV
FGKIRYMNYDGCKRKFDVAQYEKRYSSL
>Aegithalos_caud__NWH83821.1
RFLLQSLEDLDNSLRKLNSRLFVVRGQPTDVFPRLFKEWGVTRLTFEYDSEPFGKERDAAIVKLAKEAGVEVV
IENSHTLYDLDRIIELNGHKPPLTYKRFQAIISRMELPKKPVSTVISQQMETCKVDIQENHDDVYGVPSLEEL
GFPTDGLAPAVWQGGETEALARLDKHLERKAWVANYERPRMNANSLLASPTGLSPYLRFGCLSCRLFYYRLWE
LYKKVKRNSTPPLSLYGQLLWREFFYTAATNNPKFDRMEGNPICIQIPWDRNPEALAKWAEGKTGFPWIDAIM
TQLRQEGWIHHLARHAVACFLTRGDLWISWESGVRVFDELLLDADFSVNAGSWMWLSCSAFFQQFFHCYCPVG
FGRRTDPSGDYVKRYLPKLKGFPSRYIYEPWNAPESVQKAAKCIIGVDYPKPMVNHAETSRLNIERMKQIYQQ
LSRYRGLCLLASVPSCVEDLSGPVTESASGQGCSTSTAARLSQADQASPKRKHEGAEEPCPEELYKRAKVTGL
PASEIPGKS
>Aegithalos_caud__NWH86447.1
KVDYDVCSNYGNWLYSAGIGNDPRDNRKFNMIKQGLDYDGNGDYVRLWVPELQGIKGADIHTPWALSSAALSQ
AGVTLGETYPWPVVTAPEWSRHIPQRPGGSPHPRGRRGPEQHKDRGIDFYFSRKKDA
>Aegithalos_caud__NWH87675.1
MLHRTIHLFRKELRLHDNPVLLAALESSEALYPVYILDKAFLTSSMHIGALRWHFLLQSLEDLHKNLGQLGSC
LLVIQGEYETVLRDHIQKWNITQVTLDAEMEPFYKEMEANIQRLGAELGFEVLSLVGHSLYNTERILDLNGGS
PPLTYKRFLHILSLLGDPEVPVRNLTAEDFQRCRAPDPGLAECYRVPLPVDLEISPESLSPWRGGETEGLQRL
EQHLTDQGWVTSFTKPRTIPNSLLPSTTGLSPYFSMGCLSVRTFFYRLSNIYAQAKHHSLPPVSLQGQLLWRE
FFYTVASATPNFTQMAGNPICLQICWYKDAERLHKWKTAQTGFPWIDAIMTQLRQEGWIHHLARHAVACFLTR
GHLWISWEEGMKVFEELLLDADYSINAGNWMWLSASAFFHQYTRIFCPVHFGKRTDPQGNYIRKYLPVLKNFP
SKYIYEPWTASEEEQKQAGCIIGQDYPFPMVNHKEASDHNLQLMKQVREEQHRTAQLTRDDADDPMEIKVKYD
HSEETISKRKVRKVARMTE
>Aegithalos_caud__NWH88797.1
DNWAFLYAQRLALKQELPLHVCFCLVPKFLEATIRHYRFMLRGLQEVAEECAELNISFHLLLGYAKDVLPTFV
VEQGVGGLVTDFSPLRLPRQWVEDVRERLPEDVPFAQVDAHNIVPCWVASPKQEYSARTIRGKIHAQLPEFLT
EFPPVVRHPHPPSCPAEPIAWEACYSSLQVDHTVKEVEWATPGTSAGMAVLKSFIAERLKSFSTHRNDPNKAA
LSNLSPWLHFGQVSTQRAILEVQKHRRNYKDSVDAFVEEAVVRRELAENFCYYNENYDSVQGAYDWAQTTLKL
HAKDKRPYLYSLQELEQGTTHDPLWNAAQLQMVQEGKMHGFLRMYWAKKILEWTQSPEEALQFAIYLNDRYEL
DGRDPNGYVGKLQGCLWSICGIHDQGWAERPIFGKIRYMNYAGCKRKFDVDQFERRYAPAH
>Aegithalos_caud__NWH92449.1
KAWVANFERPRMNANSLLASPTGLSPYLRFGCLSCRLFYFKLTDVKKNSSPPLSLYGQLLWREFFYTAATNNP
RFDKMEGNPICVQIPWDKNPEALAKWAEGRTGFPWIDAIMTQLRQEGWIHHLARHAVACFLTRGDLWISWEEG
MKVFEELLLDADWSVNAGSWMWLSCIHCYCPVGFGRRTDPNGDYIRRYLPVLRGFPAKYIYDPWNAPESIQKA
AKCIIGVNYPKPMVNHAEASRLNIERMKQIYQQLXLLATVPSNPNGNGNGGLMGYSPGESMSGCGSTGGAQLG

TGDGHSVVQSCALGDSHTGTSGIQQQGYCQASSILHYAHGDNQQSHLLQAGRTALGTGISAGKRPNPEEETQS
VGPKVQRQSTN
>Agelaius_phoe__NWZ04222.1
RFLLQSLEDLDNSLRKLNSRLFVVRGQPTDVFPRLFKEWGVTRLTFEYDSEPFGKERDAAIVKLAKEAGVEVV
IENSHTLYDLDRIIELNGHKPPLTYKRFQAIISRMELPKKPVSTVISQQMETCKVDIQENHDDVYGVPSLEEL
GFPTDGLAPAVWQGGETEALARLDKHLERKAWVANYERPRMNANSLLASPTGLSPYLRFGCLSCRLFYYRLWE
LYKKVKRNSTPPLSLYGQLLWREFFYTAATNNPKFDRMEGNPICIQIPWDRNPEALAKWAEGKTGFPWIDAIM
TQLRQEGWIHHLARHAVACFLTRGDLWISWESGVRVFDELLLDADFSVNAGSWMWLSCSAFFQQFFHCYCPVG
FGRRTDPSGDYVKRYLPKLKGFPSRYIYEPWNAPESVQKAAKCIIGVDYPKPMVNHAETSRLNIERMKQIYQQ
LSRYRGLCLLASVPSCVEDLSGPVTDSASGQGCSTSTAARLSQADQASPKRKHEGAEEPCPEELYKRAKVTDL
PTSEISGKSL
>Agelaius_phoe__NWZ06291.1
MLHRTIHLFRKELRLHDNPVLLAALESSEALYPVYILDRAFLTSSMHIGALRWNFLLQSLEDLHKNLGKLGSC
LLVIQGEYELVLRDHIQKWNITQVTLDAEMEPFYKEMEANIQRLGAELGFEVLSLVSHSLYNTQRILDLNGGS
PPLTYKRFLHILSLLGDPEVPVRNLTAEDFQRCRAPDPGLAECYRVPLPVDLKISPESLSPWRGGETEGLQRL
EQHLTDQGWVTSFTKPRTIPNSLLPSTTGLSPYFSMGCLSVRTFFYRLSNIYAQAKHHSLPPVSLQGQLLWRE
FFYTVASATPNFTQMAGNPICLQICWYKDAERLHKWKTAQTGFPWIDAIMTQLRQEGWIHHLARHAVACFLTR
GDLWISWEEGMKVFEELLLDADYSINAGNWMWLSASAFFHQYTRIFCPVRFGKRTDPQGDYIRKYLPILKNFP
SKYIYEPWTASEEEQKQAGCIIGQDYPFPMVNHKEASDHNLQLMKQVREEQHKTVQLTRDDADDPMEIKVKRD
HSEENISKGKVARTAE
>Agelaius_phoe__NWZ09730.1
MGVNAVHWFRKGLRLHDNPALRECIQGADTVRCVYILDPWFAGSSNVGINRWRFLLQCLEDLDANLRKLNSRL
FVIRGQPADVFPRLFKEWNIAKLSIEYDSEPFGKERDAAIKKLASEAGVEVIVRISHTLYDLDKIIELNGGQP
PLTYKRFQTLISRMEPLEMPVETITPEVMKKCTTPVSDDHDEKYGVPSLEELGFDTDGLPSAVWPGGETEALT
RLERHLERKAWVANFERPRMNANSLLASPTGLSPYLRFGCLSCRLFYFKLTDLYKKVKKNSSPPLSLYGQLLW
REFFYTAATNNPRFDKMEGNPICVQIPWDKNPEALAKWAEGRTGFPWIDAIMTQLRQEGWIHHLARHAVACFL
TRGDLWISWEEGMKVFEELLLDADWSVNAGSWMWLSCSSFFQQFFHCYCPVGFGRRTDPNGDYIRRYLPVLRG
FPAKYIYDPWNAPESIQKAAKCIIGVNYPKPMVNHAEASRLNIERMKQIYQQLSRYRGLGLLATVPSNPNGNG
NGGLMGYSPGESISGCGSTGGHLLPASPGAQLGPGDGHSVVQSCALGDSHTGTSGIQQQGYCQASSILHYAHG
DNQQSHLLQAGRTALGTGISAGKRPNPEEETQSVGPKVQRQSTN
>Agelaius_phoe__NWZ14047.1
DNWAFLYAQRLALKQELPLHVCFCLVPKFLEATIRHYRFMLRGLQEVAEECAELNISFHLLLGYAKDVLPTFV
TEHGVGGLVTDFSPLRLPRQWVEDLRERLPEDVPFAQVDAHNIVPCWLASPKQEYSARTIRGKIHAQLPEFLT
EFPPVVRHPHPPSCPAEPIAWEACYSSLQVDHTVKEVEWATPGTAAGMAVLKSFIAERLKSFSSHRNDPNKAA
LSNLSPWLHFGQVSTQRAILEVQKHRRSYKDSVDAFVEEAVVRRELAENFCYYNENYDSVQGAYDWAQTTLKV
HAKDKRPYLYSLQELEQGTTHDPLWNAAQLQMVREGKMHGFLRMYWAKKILEWTHSPEEALQFAIYLNDRYEL
DGRDPNGYLTLVSLCPAGCLWSICGIHDQGWAERPIFGKIRYMNYAGCKRKFDVEQFERRYAPTH
>Agelaius_phoe__NWZ15919.1
KVDYDVCSNYGNWLYSAGIGNDPRENRKFNMIKQGLDYDGNGDYVRLWVPELQGIKGADIHTPWALSSAALSQ
AGVTLGETYPQPVVTAPEWSRHIRQRPGGSPHPRGKRGPAQHKDRGIDFYFSRKKDA
>Agrilus_pla_XP_018319593.2_photolyase
MASITPRVGSKVDWTKFTKENFISAIKESRSKEGERVEDFKFNKNRIRILSDCKEVKESSDGILYWMCRDCRV
QDNWAMLFAQRLARKNKIPLFVCYFTKNGSELVPTRRHNNFLINGLKEVAEECSRLNIKFHLINGDPCKSLVD
IVIRNNIGSVICELNPLRKPKKWQQTVKGMLPNDVSLVQVDAHNIVPVWITSSKQELAARTIRPKINRNLEEY
LTGFPMVSKHEYAGELNLNNMEYSFEDAIKSFESEKDVDLPEIKWAKPSGTTAGIEMLHLFLTTRLKNYATTS
GDPSKDNLSRMSSWVNFGQLSAQRIALEVENFKNSYKESVERYLEELIVRRELSDNYCFYNENYDNIEGADKW
ARTTLDIHRKDKREYIYTKEQFAQAKTHDEIWNAAQVQVINEGRMHGYIRMYWCKKILEWTKSPEEAIEIGLH
LNDTFALDGNNPNGFVGVMWSICGVHDHGWTERPIFGKIRFMVFWSMRKKFDADAFCEQYGRRIDNATAPKAK
KQSVSNIKQTYLNKKQSTKSHSESDNEEKVQEKKSRPRRNKNETSRLQPKKKTKLK
>Agrilus_pla_XP_018321938.1_cry-2_X1
MEPRQATVIHWFRKGLRLHDNPALLEAIQVAVSQQAFLRPLFILDPNIPKWMKIGPNRWRFLIQSLKNLNNSL
KAVNSKLYVVRGKPEKVFRRILLEWSVTLITFESDIEPFAKERDTKIEKLAKELNIKVIQRISHTIYNPDLIL
TKNFGNPPLTYQKFISLIEQFKKPINLAKHPPNVPDYCKPFADLLEKNNSNCYEIPTLQELGVNEEGLGENLY
SGGETEALARMEKYLENAQWICKFEKPNTSPNSLEPSTTVLSPYLKFGCLSSRLFYNRLNDVIRGAYCTKPPV
SLIGQLLWREFYYTVAAATPNFDKMVGNSICYQIDWDTNERHLQAWKTGKTGYPFIDAIMRQLRTEGWIHHLA
RHAVACFLTRGDLWISWEEGLKVFEEFLLDADWALNAGNWLWMSASAFFHQFYRVYSPTAFGKKTDKKGDYIR
KYVPELRKFPEAYIYEPWLASKNVQKEAGCVVGVDYPERIVIHETVYKINIGRMSAAYKRNKKVNDNGKSAKS
SNCSSNLKRKRK
>Agrilus_pla_XP_018331212.1_cry-1-like

MTGTIKHPSNQSPKKPVKHMVHWFRKGLRLHDNPALREGLRDATTFRCVFVLDPWFAGSSNVGINKWRFLLQC
LEDLDSSLKKLNSRLFVIRGQPADALPKLFKEWGINALTFEEDPEPFGRVRDHNISALCKEMGITVIQKTSHT
LYNLDAIISRNGGRAPFTYHQFQTVIAGMDPPPTAEAAVTDKTLNGAVTPVNEDHDDRFGVPTLDELGFDTEG
LLPPVWQGGETEALARLERHLERKAWVASFGRPKMTPQSLLASQTGLSPYLRFGCLSTRLFYYQLTDLYKKIK
KAFPPLSLHGQLLWREFFYCAATKNPNFDKMVGNPICVQIPWDKNPEALAKWANGQTGFPWIDAIMTQLREEG
WIHHLARHAAACFLTRGDLWISWEEGMKVFEELLLDADWSVNAGMWMWLSCSSFFQQFFHCYCPVKFGRKADP
NGDYIRKYLPVLKNMPTQYIHEPWNASDAVQKAAKCIIGKDYPLPIVNHTLASKINMERMKQVYQQLSKYRSF
DTQKLNCISSSKLKDYQPSVVTVGNLKNLQI
>Agrilus_pla_XP_025837056.1_cry-1
MHFIQYCTDIYVLAPLTDILGKLFISQSNTKSTIKLEVFAMQVSILWFRHGLRLHDNPALHAALKKKQPFLPI
FIFDGETAGTQLIGYNRMRFLCEALYDLDRQLKQLGGRLYYAKGAVVQIFTKIWQELGIGRLCFEEDCEPIWR
ARDDSVKIMCKDLGIECVECVSHTLWDPKKIIETNGGQPPLTYQMFLYTVDILGDPPRPKDEPRWSRVKFSNP
FPLLEKVLYSFQTLPNPEDFGVFPEITETPLVRWIGGETQALKKLEERLKLEERAFRQGYYLPSQNTPDLLGP
PASQSAALRHGCLSVRKFYWSIQDLFKVIHGDRLPTVHSITGQLIWREYFYTMSVKNPNYAVMKENPICLDIN
WIQSTSTDYNIRLTSWKEGLTGYPFIDAIMRQLKAEGWIHHMARNMVACFLTRGDLWISWEEGLQHFLKYLID
ADWSVCAGNWMWVSSSAFEQLLDCSTCICPINFGKHFDPSGEFIKRYVPELKKFPSTLIYEPWKASLEQQIAA
ECIVGKDYPNRIINHKEAAERNRLAMKTLREDLTKPPTHCCPSNENEVRQFMWLQDTCLKHQYVSI
>Agrobacterium_fab_WP_174020122.1_DNA_photol
MSLKTAPVIVWFRKDLRLSDNLALLAAVEHGGPVIPVYIREKSAGPLGGAQEWWLHHSLAALSSSLEKAGGRL
VLTSGDAERILRDLISETGADTVLWNRRYDPTGMATDKALKQKLRDDGLTVRSFSGQLLHEPSRLQTKSGGPY
RVYTPFWRALEGGEEPHAPVDAPKSLTGPKTSPQSEKLADWKLLPTKPDWAKDFSDIWSPGENGALEKLDDFI
DGALKGYEEGRDFPAKPATSLLSPHLAAGEISPAAVWHATKGLSRHIASNDISRFRKEIVWREFCYHLLFHFP
ELDEKNWNDSFDAFPWRDDDTSFNAWTRGMTGYPIVDAGMRQLWKHGTMHNRVRMIVASFLIKHLLIDWRKGE
KWFRNTLVDADPASNAANWQWVAGSGADASPFFRIFNPILQGEKFDGDGDYVRRFVPELEKLERKYIHKPFEA
PKDALKKAGIELGKTYPLPIVDHGKARERALAAYAAVKKTT
>Albugo_can_CCI11597.1_unnamed_pr
MTTIRCRLESCPKTERIQLNRIRWLYDGAISAKKTDTSAQDKKYILYWMQTSVRTTYNYGLEYALEAATALAL
PLQVIYFLSDRSAVPKKQLARDPNAFAFSTERHAKFALEGLASTKSRLKERGIDLKIVLSGNTKQESENEENE
DEFGATRYHLLDRCARNAVLVVTDRAYIRFSANDTLRCAEYAKESKNAWGLVQVEGDVVVPSAIASDKEEYSA
RTIRPKVTRHLAKFLVQLKEQDISDKIKSLDLNLLHFLPFKLLNKNTSTKDCILEIDPARKAEMLEALDLDRS
VVPVESMLGGEDEAERHAREFARGKLCKYAEARNEPGADGSSNLSCYLHYGHISPVWIALLVKEIDDANNKNS
QDAFLEEMIVRRELSVNFVVYNPDRYDSIEGIPDFAKKTLNDHRGDERPHIYTLKDLESGLTEDKYWNAAQLE
VVHSGRMHGYMRMYWGKKILEWSKTPEEAFERTVYLNNKYSLDAPHPNSYTGVAWTFGKHDQGWREREIFGKV
RYMNETGLKRKFDMDGYVQKIKSLRESCGDE
>Albugo_can_CCI43115.1_unnamed_pr
MESHQDLLDFGAFPADFYENDESASGIDAFEIQNCFDVFEDSSLESKITDKHATMPSIRAIMWFRRDLRLHDN
LALHRALKFVEAHKDTESVQLIPLYIIHRPKRLLCGVNRFQFLLESVGSLAEAIASRGSRLIIARGEGIEVFR
RILPAWKITHMFFDQTIEPFATARDERVRELAASLDIHVETTRGYTLYDLDQVMEKNGGKAPATYTSFLRTVA
TFPEPAKPLPIPSSIPGWEDSREGLYKRLVEEEYLNVSHEQAISTQSQSWIDVIAGPQKLFRVPELSEFGFER
PERHSFIYGGENVALKILQEYCKNEGQVVKFEKPKTSPAETCPSKSTTSLSPYLYFGCISTRTFFHRVRDIQN
RHAKASSAPPVSLDGQLLWREFFHCNGHANPHFDKMEGNPLCLHIDWRWHTLPKTIEEEEALSEDEKLAKAQF
EAWKEGQTGYPWIDAIMIQLKEEGWMHHLARHSVACFLTRGDLYISWVRGLEVFQERLIDHDWSINCGNWLWL
SASAFFTAYFRVYSPSTFGKKWDVEGKYIRKYIPALAKMPAKYIYEPWKAPVTVQRLANCLVGKDYPFPIVDH
KIAMRRCMAGMKASYAARKYGEQSHETNASPHPKKRSREYDEVKDEK
>Albula_glos__KAG9335192.1
MLYKLSPLSVLLSPRFLLQCLEDLDASLRKCNSRLFVIRGQPTDVFPRLFKEWNITRLSYEYDSEPFGKERDA
AIRKLASEADVEVMVRISHTLYDLDKIIELNGGQSPLTYKRFQTVISRMPPVEMPAEVITAEVIGQCTTPIAD
DHDEKFGVPSLEELGFDTEGLSTAVWPGGETEALTRLERHLERKAWVANFERPRMNANSLLASPTGLSPYLRF
GCLSCRLFYFKLTDLYKKVKRHSSPPLSLYGQLLWREFFYTAATNNPRFDKMEGNPICVQIPWDRNPEALAKW
AEGCTGFPWIDAIMTQLRQEGWIHHLARHAVACFLTRGDLWISWEEGMKVFEELLLDADWSVNAGSWMWLSCS
SFFQQFFHCYCPVGFGRRTDPNGDYIRGFPAKYIYDPWNAPESVQKVAKCVIGVHYPKPMVHHAEASRLNIER
MRQIYQQLSCYRGLGLLATVPSNPNGSGEGMPAAMGPCSGEGSQDAGYQMCVTPQRDWSTGMITYPQGDSQSG
SGTQQPGFPGGSNVVCYRQDSQQTHGAFLQPGHHTSASNTVKRHSEDSPLSVSSKVQRQSSN
>Albula_glos__KAG9336928.1
MLGSSVNRTDNITRQSFYLTMSSGDEAKLVSQIRREMRELVLGRQDPEEFFCRSVSMLGHQGAQEQLLDLIEP
LVFEHAEVHARLVTMFLDYFATQAHSPVKKTEEEELELALALSLQEMRGSEFVAPQPPSHAQPHYGSYAQQAA
SGVRVSQRKVLSHGNKASVICDTDLAGDGRASRESTKQRADTQSGSLCEAGPGSCSEEGVKVDLSSNISDNPK
SSRRRRQRKKNQLAMTQSPSSPRPALLWFRRDLRLIDNPALIGSLEVGGPVIPVFLWSPDEEEGPGVTVAMGG
ASKYWLHHSLLCLNQSLEKLGSHLVTVKVERSSLESLQSLAAETGAGTVLATALYEPWLKQRDELVFSSLERQ
GIKCQLYHSYSLRHPSSISTEGVGLRGIGSVSHFMSCCQRSPDSGLVPTAWPQGCALSQLGLARMPRRKDGTV

VDWAANIRSSWDFSEDGAQARLDAFFQDGIYRYEKESCRADAPNTSSLSPYLHYGQISPRWLLWQPPKFQRKL
AWRDLAYWQLSLFPDLPWESLRPPSEHTHLKAWQRGRTGYPLVDASMRQLWLTGWMNNYMRHVVASFLIAYLH
LPWQEGYRWFQDTLVDADVAIDAMMWQNGGMCGLDHWNFVMHPVSAALTCDPCGAFVRRWCPELADLPDELIH
KPWQCPTSLLRRAGVVLGGNYPERIVTDLEERRAQSLQDVAAVRKRFGEYVDRRSGCDLVPLPDRLVKEALGI
GMGDLVHSGGRFLLPVITRLEFKHQTHDPDSDAGANPYSAVLRGYVSRKRDETVAFLNERDFTASVMNESAQR
RDRQERDQRALEGLPRPPAPRGRGRRTPSKDTFSTVPGGVLVPHG
>Albula_glos__KAG9347773.1
MSARRTIICLLRNDLRLHDNEVFHWAQRNAELIVPLYCFDPRHYLGTTCYNFPRTGPFRLSFLLDSVKDLRAT
LQRNGSNLVVRRGKPEEVVGDLIKQLGSVSAVAFHKEVTQDDLDVEKGVEDVCSQLKVNVQTFWGSTLYHIED
LPFNHISRLPDVYTQFRKAVETQGRVRPVLPTPDVLKPLPTGLEEGPIPTHEDLEQMEMMADPRSAFPCSGGE
SQALARLQHYFWDTDAVASYKETRNSLIGVNYSTKFAPWLSMGCISPRYIYEQIKKYESERTANQSTYWVIFE
LLWRDYFRFVAVKYGNRIFALEGLQDKSVPWKTDMQLFNAWKEGRTGVPFVDANMRELAQTGFMSNRGRQNVA
SFLTKDLGLDWRMGAEWFQYLLVDHDVCSNYGNWLYSAGIGNDPRENRKFNMIKQGLDYDNNGDYVRQWVPEL
QGIKGGDVHTPWTLSSGALAHAKVSLGETYPSPIVMAPEWSRYVKKKPSGAGPTSRGRKGPSHTPRQHKDRGI
DFYFSRSKDFYIEIKNYEKRDAPFEKILDDWESQCPDATVGKLLSMLEEAGRHDVVTDHIDLIEEDVRKYLEK
MKEPVQVPDIDSWTPEKNGITIKDDPSGNVPETFDAFICYCQSDIEFVHEMIRQLEQTDHRLKLCVFDRDVLP
GTCVWTITSELIEKRCKRMVVVISDDYLESEACDFQTKFALSLSPGAQKKRLIPVIYKTMKKPFPSILRFLTV
CDYTRPCTQSWFWVRLAKALSLP
>Albula_glos__KAG9351737.1
MSHISIHWFRKGLRLHDNPALMAALQGCVEIYPLFVLDPSEKLAGINSCRFLLGSLKDLDCSLQKLNSRLFQK
WKVTRLTFESDTEPDSVDRDRRVEKLAKEHDVEVIQKVSHTLYDTERVIEKNNGKPPLTFNRLQTVLQAMGPP
EKPVPSPSLEDMKSVQTTGWKKHEEEYGVPTFEELGKDPQSVGSELYPGGEQEALRRLDEHMKRTGWVCNFQK
PETSPNALNPSTTVLSPYIRYGCLSARTFWWRLTDVYRGKKHSQPPVSLHGQLLWREFFYTASVGIPNFNRMK
GNSVCVQVDWDSKPELLAAWREARTGFPFIDAIMTQLRQEGWIHHLARHAVACFLTRGDLWISWEEGQQVFEE
LLLDGDWALNAGNWQWLSASAFFHQYFRVYSPIAFGKKTDKHGEYIKKYLPILKKFPTEYIYEPWKAPRSIQE
RAGCIVGKDYPCPIVNHEVISKENIKRMKLAYSKRSPDPSESKGKVKGEKRKAVSVIDMLTKKKKAGPY
>Albula_glos__KAG9352121.1
MVVNSVHWFRKGLRLHDNPSLQEALNGADTVRCVYILDPWFAGSANVGVNKWRFLLESLEDLDASLRKLDSRL
FVVRGQPAEVFPRLFKEWNVTRLTFEYDSEPYGKERDAAIIKMALEFGVETRVKNSHTLYNLDRIIELNNSSP
PLTFKRFQAIVNRLGLPKRPLATVTKQQLDSCRTEISDNHNDRYGVPSLEELGFKTHGLSPSVWKGGETEALD
RLDKHLDRKAWVANFERLRINICSLMASPTGLSPYLRFGCLSCRLFYYNLRELYMKVRKHPSPPLSLFGQLLW
REFFYTAATNNPNFDRMEGNPICVQIPWDHNPEALAKWAEGRTGFPWIDAIMTQLRQEGWIHHLARHAVACFL
TRGDLWLSWESGMRVGPPPSEFLHLTFLGHRLILEPSLGCNPH
>Albula_glos__KAG9353080.1
MTHRTIHLFRKGLRLHDNPSLVGALESSEVVYPVYVLDRAFLEGSMATGALRWRFLLQSLQDLQSSLAARGSL
LYVLQGSHEAVLRQLIQQWSITQLTFDTEVEPFYTQLDDHLCALCKELGVVVHSCVAHTLYDVKRIIKANGGE
PPLTYKKFLHVLSILGEPDTPARDITLQDFQKCKTPVEDGREEQYQVPSLEDLGIEVDVEVLWPGGESHGLKR
LEKHIESQGWVVNFSKPRTIPNSLLPSTTGLSPYFSLGCLSVRSFYYRLSNIYAQSKNHSLPPVSLQGQVLWR
EFFYTVASATPNFTKMAGNPICLQIHWYENQAALDKWRTAQTGFPWIDAIMTQLRQEGWIHHLARHAVACFLT
RGDLWISWEEGMKVFEKYLLDADYSVNAGNWMWLSASAFFHQYTRIFCPVRFGRRTDPDGQYLRKYLPVLKNF
PSKYIYEPWTAPLEVQLEAGCVIGKDYPLPMVNHLEVSQRNTALMRQVRSEQENTAQLTRDVADDPMEIGMKR
ECRDQRPSDDFSEKTERANKRFSPEGEHKSLSCNWASDTIRLPELSGEVM
>Alectura_lath__NXL85029.1
AQLSVLFSSFVLKVDYDVCSNYGNWLYSAGIGNDPRDNRKFNVIKQGLDYDGNGDYVRLWVPELQGVKGADIH
TPWALNSATLSQAGVTLGETYPQPAVTAPEWSRHISQRPQGRSPHPRGRRRPAYTAMQHKDRGIDFYFSRKKD
A
>Alectura_lath__NXL85365.1
MPHRTIHLFRKGLRLHDNPALLAALRSSHVLYPVYILDRTFMTSSMHIGALRWRFLLQSLEDLQENLRRLGSC
LLVIQGEYESVVREHVQKWSITQVSLDAEMEPFYKEMEANIRALGEELGFEVLSLAGHSLYNTERILDLNGGT
PPLTYKRFLHILSLLGDPEVPVRNLTAEDFQRCRSPEPGLAERYRVPLPAELKIPAESSSPWRGGETEGLQRL
ERHLADQGWVASFTKPRTIPNSLLPSTTGLSPYFSTGCLSVRSFFYRLSNIYAQAKHHSLPPVSLQGQLLWRE
FFYTVASATPNFTQMAGNPICLQISWYEDAERLHRWKTAQTGFPWIDAIMTQLRQEGWIHHLARHAAACFLTR
GDLWISWEEGMKVFEELLLDADYSINAGNWMWLSASAFFHQYTRIFCPVRFGRRTDPEGHYVRKYLPILKNFP
SKYIYEPWTASEEEQKQAGCIIGQDYPFPMVDHKQASDRNLQLMKQVREEQHGTAQLTRDDADDPMEMKLKRD
RAGEDGTKAKAARMAE
>Alectura_lath__NXL86003.1
RFLLQCLEDLDANLRKLNSRLFVIRGQPADVFPRLFKEWNIAKLSIEYDSEPFGKERDAAIKKLASEAGVEVI
VRISHTLYDLDKIIELNGGQPPLTYKRFQTLISRMEPLEMPVETITPEVMEKCTTPVSDDHDEKYGVPSLEEL
GFDTDGLPSAVWPGGETEALTRLERHLERKAWVANFERPRMNANSLLASPTGLSPYLRFGCLSCRLFYFKLTD
LYKKVKKNSSPPLSLYGQLLWREFFYTAATNNPRFDKMEGNPICVQIPWDKNPEALAKWAEGRTGFPWIDAIM

TQLRQEGWIHHLARHAVACFLTRGDLWISWEEGMKVFEELLLDADWSVNAGSWMWLSCSSFFQQFFHCYCPVG
FGRRTDPNGDYIRRYLPVLRGFPAKYIYDPWNAPESVQKAAKCVIGVNYPKPMVNHAEASRLNIERMKQIYQQ
LSRYRGLGLLATVPSNPNGNGNGGLMGFSPGESISGCASTGGVPLGTGDGQTAGVQTCALGDSHTGASGIQQQ
GYCQASSILRYAHGDNQQSHLLQAGRTSLGTGISAGKRPNPEEETQSVGPKVQRQSTN
>Alectura_lath__NXL89338.1
CIYILDPWFAASSAVGINRWRFLLQSLEDLDNSLRKLNARLFVVRGQPTDVFPRLFKEWGVTRLTFEYDSEPF
GKERDAAIIKLAKEAGVEVVIENSHTLYDLDRIIELNGNKPPLTYKRFQAIISRMELPKKPVSSIVSQQMETC
KVDIQENHDDVYGVPSLEELGFPTDGLSPAVWQGGETEALARLDKHLERKAWVANYERPRMNANSLLASPTGL
SPYLRFGCLSCRLFYYRLWELYKKVKRNSTPPLSLYGQLLWREFFYTAATNNPKFDRMEGNPICIQIPWDKNP
EALAKWAEGKTGFPWIDAIMTQLRQEGWIHHLARHAVACFLTRGDLWISWESGVRVFDELLLDADFSVNAGSW
MWLSCSAFFQQFFHCYCPVGFGRRTDPSGDYVKRYLPKLKGFPSRYIYEPWNAPESVQKAAKCIIGVDYPKPM
VNHAETSRLNIERMKQIYQQLSRYRGLCLLASVPSCVEDLSGPVADSASGQGSSTSTAIRLPQSDQASPKRKH
EGAEELCAEELYKRAKVTGLPAPEIPGKS
>Alectura_lath__NXL96297.1
QECTELDIPFHMLLGYPKDVLPTFVVQNSVGGLVTDFCPLRISRQWVEDVRDRLPEDVPFAQVDAHNIVPCWV
TSPKQEYSASTIRGKIHRQLPEFLTEFPPVIQHPHPPSCPAEPVMWDACYSSLQVDRTVPEVAWVTPGTAAGL
AMLQSFIAKRLKSFSSQRNNPNKAALSNLSPWFHFGQVSTQRAILEVQKHRRAYKESVDAFVEEAVVRRELAE
NFCYYNENYDSVQGAYGWAQTTLKLHAKDKRPFLYELPQLEQAKTHDPLWNAAQLQMVQEGKMHGFLRMYWAK
KILEWTCCPEEALRLAIYLNDRYELDGRDPNGYVGKRCLWSICGIHDHGWKERNVFGKIRYMNYAGCKRKFDV
EQFERRY
>Alkalihalobacillus_sha_WP_059102964.1_DNA_photol
MSIHIVWFRRDLRWNDHTALAAALDACEGDDRVMLVFHINPKMTKSFSQRHDYFYESLKAFVDAGKDKGVSVH
FLHGEPEEAFRELLEQVKDIRKVHFNKDESGAGRQRDQAVQKLLEAKGVSVHLYTDHHLHGAEEVTKEDGSMY
QVFTPYYRKWRSLPKPELRIIDEQKLQSTSIDQRDTFAKGREAYNQLMQQKTGKWSDRAGEVNGRERLKTFLN
DGLATYHEERDVPAIDGTSRLSPYLRTGALSPRMVHKALQIVEDKYGGSKGADTFIQELAWRDFYNMIYYAHP
NGKDEEWNQTYRNLDWSYDEEELERWKRGETGFPIVDAAMRQLQQEGWMHNRLRMVVASFLTKDLLLDWRLGE
RHFEEMLIDYDPASNIGGWQWAASTGTGAVPYFRVFNPTRQSERFDPDGTFIKMYLPELKELPLDYIHEPHRM
SSEQQEKAECIIGKQYPEPMVNHKERRRQAIEMFEGEKT
>Allacma_fusc__CAG7721498.1
MASSPKKTKTSDNNILEFSDKIHEQRLQLAKSPAEFKFEKSRCRMLTKEENLPANHRGILYWMSRDQRVQDNW
AMIYAQRLAIAEKVPLIVCFCLVPKFLEATLRQYGFMLRGLSLVREELCKLKIPFHLFFGDAKKTIPEFVTKH
NIGGVVTDFAPLRVPRLWVKQVGDKLPEDVPFCQVDAHNIVPLWVTSSKQEHAARTIRPKINNNLPKYLTEFP
PVTQHPFKDASMKCQNPFENWDKLINFLEINKSVKEVEWAKPGYEESLKVLKSFIDDRLKNFEHKRNDPNVNA
LSNLSPWFHFGQISVARAVLEVKKSSHKSAAAVFIEEAVVRRELSDNFCYYNDNYDSLDGALDWARTSLNLHK
KDKRPRLYKLKEFEGANTYDDLWNAAQIQLNLEGKMHGFLRMYWAKKILEWSESPEKALEIAIYLNDKYNIDG
RDPNGYVGCMWSICGIHDQGWREREVFGKIRYMNYEGCKRKFDVDGFVFKYKRLAKTTADAKKEDKGSPPTSK
KETEEGSTSKSKSVAGSSSNSRKGTR
>Allacma_fusc__CAG7824192.1
MRKINPQGRAQMSQKQATVIHWFRKGLRLHDNPALIAAVEKLDTLPSAHLRPIFILDTKLVETYRVGANRWRF
LVDSLADLDKSLRSLGTRLYVLRGDPVSQFKFALKNWNVKLITYETDTEPYARKRDVEVFKLAEEAGVEVKYF
FSHTLYEPIVLLRQNKSEAPKTYQKFLDVVSKAGEPPASKVAVTEVYKQSPPKDPDKDKYQLPTLRELHVDES
KLGPHLYPGGETEALRRLTQVMTRKTYISNFEKPNTSPNSIEPSTTVLSPDPNLLICKQIDWDVNPEYLSAWA
EGRTGYPFIDAIMTQLRKEGWIHHLARHAVACFLTRGDLWISWEEGQLVFEELLLDADVYLNAGNWMWLSASA
FFHQYYRVYSPIVFGKKTDPHGEYIRKYLPKLAKYPPNYIYEPWTAPMSVQKAAGCIIGTDYPKPIVDHAKIH
KVNLQRMNAAYKKVPSPPKKNTPKFTRASREDDDDSSVPFAKKFRSEFC
>Allacma_fusc__CAG7829298.1
MVSAPLVVVPPTASHDSKRADAPGRTSRDAVQEWPIFHLTDSETYNNVKMNGKHTVHWFRRGLRLHDNPSLIA
GLQGAVTFRCVFIIDPWFAGSSNAGVNKWRFLLQCLDDLDCSLRKLNNSRLFVIRGQPADVLPKYFKEWGITC
LTFEEDPEPFGRVRDQNIIAQCEELGIEVIVHPSHTLYKLDLIIEKNGGKPPLTYRQFQNVIASMGPPELPCP
PVEEVAFSVTPIEPDHDDKFGVLTLEELGFDTDCLNPAVWRGGESEAIARLERHLERKAWVASFGRPKMTPRS
LLASQTGVSPYLRFGCLSPKLFYHQLTALYRKIKKGEPPLSLHGQVLWREFFYTAATKNPNFDRMVGNTICVQ
IPWDNNPIGLAKWASGETGFPWIDAIMTQLREEGWIHHLARHAVACFLTRGDLWISWEEGMKVFDELQLDADW
SVNAGMWMWLSCSSFFQQFFHCYCPVRFGRKADPNGEFIRKYLPVLRNFPNNFIHEPWMAPESVQMSAKCIIG
EDYPKPMINHAVASRINMERMRQVYMQLSKYQSEDVRTMVALAALMSLRTLNFKVLLARI
>Allacma_fusc__CAG7836899.1
MAVTGRRFETGLEFIPSSSMCVVLESLRRISNQAIGKTVFEGISRNTYSCNLNKMISSGSSQTVQQPKYRNCV
CWLRNDLRYHDNEVILWAHENSERVMPLYCFDPRHFGKTYKYGFPKTGPFRAKFLLESVQDMHETLKTHGSKL
SVFVGKPEEVIASLLENLEVDVIVYQKEVTKEETDVEAAVQNVAKRNKIKFNTIWGSTLYHEDDLRFSVKFIP
DTYTEFRKSVEAQSKIRILKPMPDVLKPLPTGEIKCSGVPSLVELGVKDFPLHESSAFPFKGGETAALQRLNS
YFWESQAIEKYKETRNGLIGSEYSTKFSCWLAFGCISPRYIYHQIKSYERERTKNDSTYWVIFELIWRDYFRF

VCAKYGNLVFYPGGIMQKKITWKYDLNTFQKWADGQTGVPFVDANMRELLKTGWMSNRGRQNVASFLTKDLQL
DWRLGAEWFEYLLIDYDVCSNYGNWNYSAGIGNDPRENRKFNMIKQGTDYDSEGNYIRTWVPELQPILGSKIH
IPWTLHPSDLHGVKLGVHYPFPILVASEWTRHFDKTKNPRWPDHRQKNQKGIDFYFKNNSKR
>Alligator_miss__XP_006264229.2
MFPKKQRQAAGADTEPSGSRKRGRQDAEVVPGAESLAEAVKQARRRAALSVREFKFNKKRVRLLSQESHLQDG
ALGILYWMSRDQRVQDNWAFLYAQRLALKQQLPLHVCFCLVPKFLDATIRHFGFLLKGLQEVAEECGELGIPF
HLLQGYAKDVLPAFVTRHGIGGVVTDFSPLRVPLQWVEDVKERLPQGVPLVQVDAHNIVPCWVASNKQEYGAR
TIRPKIHAQLPEFLTEFPPIIQHPFPAAAPAQPIDWDACYAGLEVDRTVPEVKWATPGTAAGLAVLQAFVTRR
LPGYSEYRNNPNKAALSNLSPWFHFGQVSVQRAVLEVQKSRDRHRESVNSFVEEAVIRRELADNFCFYNKSYD
QLEGAHDWARDTLILHARDKREYLYDLHQLEQGKTHDQLWNAAQIQMVREGKMHGFLRMYWAKKILEWTRSPE
DALRFAIYLNDRFELDGRDPNGYVGCMWSICGTHDQGWAERNVFGKIRYMNYAGCKRKFNVGQFEHKYRP
>Alligator_miss__XP_006274133.1
MAASRSFPSSVPARAGPCRAVHWFRRGLRMHDNPALQAALRDAASVRCIYILDPWFAASSAVGINRWRFLLQS
LEDLDNSLRKLNSRLFVVRGQPTDVFPRLFKEWGVTRLTFEYDSEPFGKERDAAIIKLAKEAGVEVVVENSHT
LYDLDRIIELNGNKPPLTYKRFQAIISRMELPKKPVSSITSQQMEKCKAEIQENHDDVYGVPSLEELGFPMDG
LAAAVWQGGEMEALARLDKHLERKAWVANYERPRMNANSLLASPTGLSPYLRFGCLSCRLFYYRLWELYKKVK
RNSTPPLSLYGQLLWREFFYTAATNNPKFDRMEGNPICIQIPWDRNPEALAKWAEGKTGFPWIDAIMTQLRQE
GWIHHLARHAVACFLTRGDLWISWECGVRVFDELLLDADFSVNAGSWMWLSCSAFFQQFFHCYCPVGFGRRTD
PSGDYVRRYLPKLKGFPSRYIYEPWNAPESVQKAAKCIIGVDYPKPMVNHAETSRLNIERMKQIYQQLSRYRG
LCLLASVPSCAEDLSGPVTDPASVQGCSTSTALKPSQSGQASPKRKHEGIEEMCTEDLYKRAKVTGLHGPEIP
SKSL
>Alligator_miss__XP_006275665.1
MKHSCIHWFRKGLRLHDNPALLAAMKDCSELYPIFILDPWFPKNMQVSVNRWRFLVESLKDLDESLKKLNSRL
FVVRGHPAEVFPGLFKAWKVTRLTFEVDTEPYSKQRDAEVVRLAAAHGVQVIQKVSHTLYDTDRVIAENNGKV
PLTYRQLQAVLAGLGPPKQPVLAPTLETLKDCCTPGRDNRDPKYEIPTLEELGQDPKEAGPCLYPGGESEALS
RLAFHMKRMTWVCNFKKPDTEPNTLSPSTTVLSPYVKFGCLSVRTFWWKLAEIYRGKKHSSPPVSLHGQLLWR
EFFYTAGAGIPNFNRMEGNPICVQVDWDDNPEYVKAWKEGRTGYPFIDAIMTQLRTEGWIHHLARHAVACFLT
RGDLWVSWEEGQKVFEELLLDADWSLNAGNWQWLSASAFFHQFFRVYSPVAFGKKTDKSGAYIKKYLPILRKF
PAEYIYEPWKAPRSMQEQAGCIIGRDYPKPIVEHEALSKRNIMRMKAAYAQRSHSKAAQVEKESTKKGGKRKL
PAGPSVVELLTKKPKAKSS
>Alligator_miss__XP_019345130.1
MGVNAVHWFRKGLRLHDNPALRECIQGADTVRCVYILDPWFAGSSNVGINRWRFLLQCLEDLDANLRKLNSRL
FVIRGQPADVFPRLFKEWNITKLSIEYDSEPFGKERDAAIKKLASEAGVEVIVRISHTLYDLDKIIELNGGQP
PLTYKRFQTLISRMEPLEMPVETITAEVMEKCTTPVSDDHDEKYGVPSLEELGFDTDGLPSAVWPGGETEALT
RLERHLERKAWVANFERPRMNANSLLASPTGLSPYLRFGCLSCRLFYFKLTDLYKKVKKNSSPPLSLYGQLLW
REFFYTAATSNPRFDKMEGNPICVQIPWDKNPEALAKWAEGRTGFPWIDAIMTQLRQEGWIHHLARHAVACFL
TRGDLWISWEEGMKVFEELLLDADWSVNAGSWMWLSCSSFFQQFFHCYCPVGFGRRTDPNGDYIRRYLPVLRG
FPAKYIYDPWNAPEGVQKAAKCMIGINYPKPMVNHAEASRLNIERMKQIYQQLSRYRGLGLLATVPSNPNGNG
NGGLMGYSPGENVSGCSRAGAQMGSSDGHTVSVQPCALGESHGGSNGIQQQGYFQASSILHFPHGDDQQSHLL
QQGRTSLSSGISAGKRPNPEEETQSIGPKVQRQSTN
>Alligator_sinensis_XP_025063697.1_cryptochro
MTLKETKLLATCCLLISQEIAFRNRTGFLLQSLEDLDNSLRKLNSRLFVVRGQPTDVFPRLFKEWGVTRLTFE
YDSEPFGKERDAAIIKLAKEAGVEVVVENSHTLYDLDRIIELNGNKPPLTYKRFQAIISRMELPKKPVSSITS
QQMEKCKAEIQENHDDVYGVPSLEELGFPTDGLAAAVWQGGEMEALARLDKHLERKAWVANYERPRMNANSLL
ASPTGLSPYLRFGCLSCRLFYYRLWELYKKVKRNSTPPLSLYGQLLWREFFYTAATNNPKFDRMEGNPICIQI
PWDRNPEALAKWAEGKTGFPWIDAIMTQLRQEGWIHHLARHAVACFLTRGDLWISWECGVRVFDELLLDADFS
VNAGSWMWLSCSAFFQQFFHCYCPVGFGRRTDPSGDYVRRYLPKLKGFPSRYIYEPWNAPESVQKAAKCIIGV
DYPKPMVNHAETSRLNIERMKQIYQQLSRYRGLCLLASVPSCADDLSGPVTDPASVQGCSTSTALKPSQSGQA
SPKRKHEGIEEMCTEDLYKRAKVTGLHGPEIPSKSL
>Alligator_sin_XP_006021416.1_cry-1-like
MKHSCIHWFRKGLRLHDNPALLAAMKDCSELYPIFILDPWFPKNMQVSVNRWRFLVESLKDLDESLKKLKSRL
FVVRGHPAEVFPGLFKAWKVTRLTFEVDTEPYSKQRDAEVVRLAAAHGVQVIQKVSHTLYDTDRVIAENNGKV
PLTYRQLQAVLAGLGPPKQPVLAPTLETMKDCCTPGRDNRDPKYEIPTLEELGQDPKEAGPCLYPGGESEALS
RLDFHMKRMTWVCNFKKPDTEPNTLSPSTTVLSPYVKFGCLSVRTFWWKLTEIYRGKKHSSPPVSLHGQLLWR
EFFYTAGAGIPNFNRMEGNPICVQVDWDDNPEYVKAWKEGRTGYPFIDAIMTQLRTEGWIHHLARHAVACFLT
RGDLWVSWEEGQKVFEELLLDADWSLNAGNWQWLSASAFFHQFFRVYSPVAFGKKTDKSGAYIKKYLPILRKF
PAEYIYEPWKAPRSMQEQAGCIIGRDYPRPIVEHEALSKRNIMRMKAAYAQRSHSKAAQVEKESTKKGGKRKL
PAGPSVVELLTKKPKAKSS
>Alligator_sin_XP_006024339.1_cry-1

MGVNAVHWFRKGLRLHDNPALRECIQGADTVRCVYILDPWFAGSSNVGINRWRFLLQCLEDLDANLRKLNSRL
FVIRGQPADVFPRLFKEWNITKLSIEYDSEPFGKERDAAIKKLASEAGVEVIVRISHTLYDLDKIIELNGGQP
PLTYKRFQTLISRMEPLEMPVETITAEVMEKCTTPVSDDHDEKYGVPSLEELGFDTDGLPSAVWPGGETEALT
RLERHLERKAWVANFERPRMNANSLLASPTGLSPYLRFGCLSCRLFYFKLTDLYKKVKKNSSPPLSLYGQLLW
REFFYTAATSNPRFDKMEGNPICVQIPWDKNPEALAKWAEGRTGFPWIDAIMTQLRQEGWIHHLARHAVACFL
TRGDLWISWEEGMKVFEELLLDADWSVNAGSWMWLSCSSFFQQFFHCYCPVGFGRRTDPNGDYIRRYLPVLRG
FPAKYIYDPWNAPEGVQKAAKCMIGINYPKPMVNHAEASRLNIERMKQIYQQLSRYRGLGLLATVPSNPNGNG
NGGLMGYSPGENVSGCSSAGAQMGSSDGHTVSVQPCALGESHGGSNGIQQQGYFQASSILHFPHGDDQQSHLL
QQGRTSLSSGISAGKRPNPEEETQSIGPKVQRQSTN
>Alligator_sin_XP_025058533.1_photolyase
MFPKKQRQAAGADTEPSGSRKRGRQDAEVAPGVESLSEAVKQARRRAALSVREFKFNKKRVRLLSQESHLQDG
ALGILYWMSRDQRVQDNWAFLYAQRLALKQQLPLHVCFCLVPKFLDATIRHFGFLLKGLQEVAEECGELGIPF
HLLQGYAKDVLPAFVTRHGIGGVVTDFSPLRVPLQWVEDVKERLPQGVPLVQVDAHNIVPCWVASNKQEYGAR
TIRPKIHAQLPEFLTEFPPIIQHPFPAAAPAQPIDWDACYAGLEVDRTVPEVKWATPGTAAGLAVLQAFVARR
LPGYSEYRNNPNKAALSNLSPWFHFGQVSVQRAVLEVQKSRDRHRESVNSFVEEAVIRRELADNFCFYNKSYD
QLEGAHDWARDTLILHARDKREYLYDLQQLEEGKTHDQLWNAAQIQMVREGKMHGFLRMYWAKKILEWTRSPE
DALRFAIYLNDRFELDGRDPNGYGFPIPEIKLAPLYFGPGPYWPARQAQAAHSRTDLPSPTQLPHSTIPLAPA
AREGAGTRGGAGQPDGDPSLCWAERNVFGKIRYMNYAGCKRKFNVGQFEHKYRP
>Allomyces_mac_KNE59778.1_AMAG_05242
MPPATTNLARNVLMWFRRDLRLHDNPALLSALGQPDRIPAAQHLYPVYCLEIPTKKKCGHRRWIFLLECLHDL
DANLRKLGSRLFVVRGLAPDVLPLLVQHWNVSELAVGECHEPFAVARDNDVAQQIAPHGCDFKSFPGRTLFRP
QDVLARTGNGKAPLTMAAFSKIAERLPVPTPVDAPASLPSLGDDAITALRNALDPATFDALAGPQGDFSVPTI
EPDGTVPELHLTTPIPAHEQSPHRGGETEALRRLDAYLSDHARVTQFEKPKTSPAAFYPNADTTILSPYTTYG
ALSVRTFRAGIDRVYRAAKGKHSKPPVSLLAQLYWREFFMTAGATTLNFEQMRRNPICLQTEWKCIPGETTRE
SHPDDYAMLDAWAEGQTGYPWIDAIMVQLRREGWVHHLARHSVACFLTRGDLYISWERGAEVFEDLLLDHEWA
LNRGNWMWLSASAFFHQYFRVYSPVKWGQSWDSEGVFIRKYLPVLRKMPKQYIYEPWKAPIAVQRAAGCVVGV
DYPKRIVDHDVVVKVNMTKMKTSHAAQREQAAMENGDSNGGRNGTRERSGNMRDRPL
>Allomyces_macro_KNE55283.1
MAPAASRRPRSSSATNSDAYSSNSSDNSTMAPPPAKKPRATATAAASTSSSSTRPASPRQQGPVVFWFRSKDL
RVRDNTALYRAWVQAAQVERAARCSRRKGVACVGAHDECIVAPGVVKSKDNKVYTVYTPFRKAWQDLVKRKHP
QDWLAMSPDPGANPASVRTVVPDLFGTAIPEATHEALSGYTLPIDESVLAHTRVAWPAGETAAHDRLKRFAQA
KLSAYSVDRDFPALDGCSSMSGYLTAGVISARACFNVARAANGGQIDTGDKGAVHWMQELVWREFYRHILVAF
PRVSMNQPFKLDTKLVKWTDHDPATKDEAERHFTAWCEGKTGYPIVDAAMRMLVREGYLSNRLRMVAAMFLTK
DLLIDWRRGERFFMRHLIDGDLAQNNGGWQWSASTGTDSQPYFRVFNPYLQSEKFDPDAVTIRKYVPELRAVK
DSKALHEPHARLPAAVFVRTGYPRPIVDHKAARVRAINAFKIAAGKVV
>Alopecoenas_beccarii_NXW85743.1_CRY2_prote
RFLLQSLEDLDNSLRKLNSRLFVVRGQPTDVFPRLFKEWGVTRLTFEYDSEPFGKERDAAIIKLAKEAGVEVV
IENSHTLYDLDRIIELNGHKPPLTYKRFQAIISRMELPKKPVSTIMSQQMEACKVDIQENHDDVYGVPSLEEL
GFPTDGLAPAVWQGGETEALARLDKHLERKAWVANYERPRMNANSLLASPTGLSPYLRFGCLSCRLFYYRLWE
LYKKVKRNSTPPLSLYGQLLWREFFYTAATNNPKFDRMEGNPICIQIPWDRNPEALAKWAEGKTGFPWIDAIM
TQLRQEGWIHHLARHAVACFLTRGDLWISWESGVRVFDELLLDADFSVNAGSWMWLSCSAFFQQFFHCYCPVG
FGRRTDPSGDYVKRYLPKLKGFPSRYIYEPWNAPESVQKAAKCIIGVDYPKPMVNHAETSRLNIERMKQIYQQ
LSRYRGLCLLASVPSCVEDLSGPVTDSASGQGCSTSTAMRLSQADQASPKRKHEGAEELCTEDLYKRAKVTGL
PAPEIPGKSV
>Alopecoenas_bec_NXW86659.1_CRY1
MPHRTIHLFRKGLRLHDNPTLLAALESSETIYPVYVLDRGFLTAAMHIGALRWHFLLQSLEDLHKNLSHLGSR
LLVIQGEYECVLRDHVQKWNITQVTLDAEMEPFYKEMEANIRRLGAELGFEVLSRVGHSLYDTKRILDLNSGN
PPLTYKKFLHILSLLGDPEVPVRNLTAEDFQRCRSPDPGLAERYRVPVPADLEIPPQSLSPWRGGETEGLRRL
EQHLTDQGWVANFTKPRTIPNSLLPSTTGLSPYFSMGCLSVRTFFHRLSNIYAQAKHHSLPPVSLQGQLLWRE
FFYTVASATQNFTQMAGNPICLQIRWYEDAERLHKWKTAQTGFPWIDAIMTQLRQEGWIHHLARHAVACFLTR
GDLWISWEEGMKVFEELLLDADYSINAGNWMWLSASAFFHHYTRIFCPVRFGKRTDPEGQYIRKYLPVLKNFP
TKYIYEPWTASEEEQRQAGCIIGRDYPFPMVNHKEASDRNLQLMRRVREEQCGTAQLTRDDADDPMETKRDCS
EENTAKAARMME
>Alopecoenas_bec_NXW94530.1_CRY1
VLQEWNITKLSIEYDSEPFGKERDAAIKKLASEAGVEVIVRISHTLYDLDKIIELNGGQPPLTYKRYQTLISR
MEPLEMPVETITPEVMEKCTTPVSDDHDEKYGVPSLEELGFDTDGLPSAVWPGGETEALTRLERHLERKAWVA
NFERPRMNANSLLASPTGLSPYLRFGCLSCRLFYFKLTDLYKKVKKNSSPPLSLYGQLLWREFFYTAATNNPR
FDKMEGNPICVQIPWDKNPEALAKWAEGRTGFPWIDAIMTQLRQEGWIHHLARHAVACFLTRGDLWISWEEGV
KVFEELLLDADWSVNAGSWMWLSCSSFFQQFFHCYCPVGFGRRTDPNGDYIRRYLPVLRGFPAKYIYDPWNAP
ESIQKAAKCIIGVNYPKPMVNHAEASRLNIERMKQIYQQLSRYRGLGLLATVPSNPNGNGNGGLMGYSPGESI

SGCGSTGGAQLGTGDGHTVVQPCALGDSHTGASGIQQQGYCQASSILHYAHGDNQQSHLLQAGRTALGTGVSA
GKRPNPEEETQSVGPKVQRQSTN
>Alosa_alos__KAG5267328.1
MVVNTIHWFRKGLRLHDNPSLRESIQGADSVRCVYILDPWFAGSSNVGISRWRFLLQCLEDLDASLRKLNSRL
FVIRGQPTDVFPRLFKEWNISRLSYEYDSEPFGKERDAAIKKLASEAGVEVIVRISHTLYDLDKIIELNGGQS
PLTYKRFQTLISRMEPVEMPAEVITADVMGSCTTPIADDHDDKFGVPSLEELGFDTEGLSSAVWPGGETEALT
RLERHLERKAWVANFERPRMNANSLLASPTGLSPYLRFGCLSCRLFYFKLTDLFRKVKKNSSPPLSLYGQLLW
REFFYTAATNNPRFDKMEGNPICVQIPWDRNAEALAKWAEGRTGFPWIDAIMTQLRQEGWIHHLARHAVACFL
TRGDLWISWEEGMKVFEELLLDADWSVNAGSWMWLSCSSFFQQFFHCYCPVGFGRRTDPNGDYIRRYLPILRG
FPAKYIYDPWNAPESVQKAAKCIIGVHYPKPMVNHAEASRLNIERMKQIYQQLSCYRGLGLLATVPSNPNGNG
ESSSGMMGFPTAENTQESLAATSSGYQMPMASQGEWHSGVMAYPQTDPKAQRSGYVGNSSSMACYRQEAQQMP
GPSVQQAWAPRCSGRAALSQEFG
>Alosa_alos__KAG5273095.1
MPVDSRVSQVRHLLRELLAGREDPESFFCFCVSILGHQETRVRFRELISPITYVHHQLHSQLLSIYQEYFLRD
VDDELELALALSLVNTNQTSTQQPVPRSYAQLASATPAAPVRGHTQGMDTNISMGTHKTPGWTGKSPNVPKTP
DRPKESVTNKTATSENKGKAEATSSLANDNDSQEKLDADNQSEMSRKSRRRRQRKRQQQLLKSPSSPRPILMW
FRRDLRLLDNPALIGCLELGGPVIPLFLWNDQEEEGPGVTVATGGACKYWLHQALVCLNQALERIGSHLLTVK
AEPSSAEVLQRLVKETGAGTVVATALYEPWLKERDEGVASTLQQQGISWQLHHSYCLRDPYTVTTQGVGLRGI
GSVSHFMSCCQQNPTGAQGTPLDAPPTLPVPSTWPTGCPLEQLGLARMPRRKDGTTVDWAVNIRRDWDFTEEG
AHARLEAFLQDGVYRYEKESGRADAPNTSCVSPYLHWGQLSPRWLLWDARAARCRSPKFQRKLAWRDLAYWQL
TLFPDLPWESLRPPYKALRWSSERAHLRVWQKGTTGYPLVDAAMRQLWQTGWMNNYMRHVVASFLVAYLHLPW
QEGYRWFQDTLVDADVAIDAMMWQNGGMSGLDHWNFVMHPVDAAMTCDPIGTYVRRWCPELAPLPDELIHKPW
KCPTSILRRAGVVLGQTYPERVVTDLEERRTQSLRDVAEVRQRFKEYVDQHSGCDLVPLPDRLVEEALGSTGE
VLRCGSRFLLPVITRMEFKHQTADPDKADGRGNPYNAVLKGYVSRKRDETIAFLNERDFTASVMHESTQQRER
AERHHCLLEGLPASPSSATSRGRPRRTPSKDPYSIVPGGVAMPQR
>Alosa_alos__KAG5274603.1
MSRFLKSVSSSLLQLSEGPVARSKPPPTVPATTAMPNRTIHLFNKGLRLHDNPTLVAALESSAAVYPVFVLDR
HFVESSTRMGALRWRFILQSLEDLHRSLQRLGSRLYVLQGTHEAVLRRLVAQWGITQLSFDVEVEPHYKRLDE
ELRAMASELGLGVLSCVAHTLYDVKRIIKANGGQPPLTYKKFLQVLSILGKPDMPAREITEMDIQKCPMPSNE
GGEEHYQVPSLSELGLEVDSEVLWPGGESHALERLERHFQSQGWVANFSKPRTIPNSLLPSTTGLSPYLSLGC
LSVRTFYHRLSKIYAQSKNHSLPPVSLQGQVLWREFFYTAALATPNFTRMVGNPICLQIGWYEDPEALKKWKM
AQTGFPWIDAIMTQLRQEGWIHHLSRHAVACFLTRGDLWISWEEGMKVFEEFLLDADYSINAGNWMWLSASAF
FHQYTRIFCPVRFGRRTDPDGHYLRKYLPVLKNFPSKYIYEPWNAPEDVQLQSGCIIGKDYPLPIVNHLDASQ
RNMALMKTVRCEQANTTELTRDVADDPMEVGVKRELSEEDAVAQGDGTECNGGISVGKKHCSSENEGRTCSWT
PDTHRLQEISSKVM
>Alosa_alos__KAG5286003.1
MTSKKTISPDIPIIQVDAHNVVPCWEASGKLEYSARTIRGKITKLLPTFLTEFPVVDKHPHSSSRTAKAVNWS
EILSSLVVDRGVGEVDWAKPGTSGGMAMLESFIDQRLGDFAALRNNPNVAALSQLSPWIHFGHLSAQRVVRQV
QKNGKKFSDSVASFTEELVVRRELADNFCFYNKNYDNIEGAYEWARKTLQDHAKDKRTHVYTREQLEQAKTHD
KLWNAAQTQVLTEGKMHGFMRMYWAKKILEWTSSPEEALAIAIYLNDRYSLDGCDPNGYVGCMWSICGIHDQG
WAERPVFGKIRYMNYDGCKRKFDVAQYERRYSKKEV
>Alosa_sapi__XP_041922980.1
MSSSRTIICLLRNDLRLHDNEVFHWAQKNAEHIVPLYCFDPLHYLGTSCFNFPKTGPFRLRFLLDSVNDLRAS
LKKRGSNLVVRRGKPDQVVGDLIKQLGSVTAVAFHEEVTQEEKEVEKQIKDVCLKNQVKVQSFWGSTLYHRHD
LPFNHISRLPDVYTQFRKAVESQSRVRPVLPTPAQLRSLPLGLEEGPVPTYQDLEQLEPVTDPRSAFPCHGGE
SEALARLKHYFWDTDAVATYKETRNGLIGVNYSTKFAPWLAMGCISPRYIYEQIKKYEQERTANQSTYWVIFE
LLWRDYFRFVALKYGNRLFHLNGLQDKHVPWKTDMKLLDAWKEGRTGVPFVDANMRELALTGFMSNRGRQNVA
SFLTKDLGLDWRMGAEWFEYLLVDYDVCSNYGNWLYSAGIGNDPRENRKFNMIKQGLDYDNNGDYVRQWVPEL
QGIKGGDVHTPWALSSGALSHAKVSLNETYPSPVVMAPEWSRHVNKKPSGGSGPAARGRKGLSHTPKQHKDRG
IDFYFSKSRGLKTSNEGL
>Alosa_sapi__XP_041944778.1
MPNRTIHLFNKGLRLHDNPTLVAALESSAAVYPVFVLDRHFVESSTRMGALRWRFILQSLEDLHRSLQRLGSR
LYVLQGTHEAVLRRLVAQWGITQLSFDVEVEPHYKRLDEELRAMASELGLGVLSCVAHTLYDVKRIIKANGGQ
PPLTYKKFLQVLSILGEPDMPAREITEMDIQKCPMPSNEGGEEHYQVPSLSELGLEVDSEVLWPGGESHALER
LQRHFQSQGWVANFSKPRTIPNSLLPSTTGLSPYLSLGCLSVRTFYHRLSKIYAQSKNHSLPPVSLQGQVLWR
EFFYTAALATPNFTRMVGNPICLQIGWYEDPEALQKWKMAQTGFPWIDAIMTQLRQEGWIHHLSRHAVACFLT
RGDLWISWEEGMKVFEEFLLDADYSINAGNWMWLSASAFFHQYTRIFCPVRFGRRTDPDGHYLRKYLPVLKNF
PSKYIYEPWNAPEDVQLQSGCIIGKDYPLPIVNHLDASQRNMALMKTVRCEQANTTELTRDVADDPMEVGVKR
ELSEEDAVAQGDGTECNGGISVGKKHCSSENEGRTCSWTPDTHRLQEISSKVM
>Alosa_sapi__XP_041961620.1

MTHNCIHWFRKGLRLHDNPALIAAMRDCKQLYPVFILDPWYPNNTKIGINRWRFLIGALKDLDCSLRELNSRL
FVVRGKPDEVLPELFNKWKITRLTFEVDTEPHSLQRDEEVLKLARDHGVEVIQKISHTLYNLERIIDENHGKA
PLTYVRFQTVLKGVGPPKRPVPPPTLGNMKGVSTPCTDKHEEKYGIPTLDELGQDPQLACPEVYPGGEQEALK
RLDEYMDKQAWVCSFEKPQTSPNALSPSTTVLSPYIRFGCLSARTFWWRLSAVYQGKKHSQPPVSLHGQLLWR
EFFYTAALGVPNFNKMEGNPVCVQVDWDNNPDHLAAWREARTGFPFIDAIMTQLRQEGWIHHLARHAVACFLT
RGDLWVSWEEGQKVFEELLLDADWALNAGNWQWLSASAFFHQYYRVYSPIAFGKKTDKHGDYIKKYLPLLKKF
PEQYIYEPWKAPRSVQEQAGCIVGKDYPRPIVEHEVISKKNIQRMKAAYAKRSPQTDESPAKEKGKKHKGESI
KDMLTKKKKKSA
>Alosa_sapi__XP_041964697.1
MPVDSRVSQVRHLLRELLAGREDPESFFCFCVSILGHQETRVRFRELISPITYVHHQLHSQLLSIYQEYFLRD
VDDELELALALSLVNTNQTSTQQPVPRSYAQLASATPAAPVRGHTQGMDTDISMGTHKTPGWTGKSPNVPKTP
DRPKESVTNKTATTENKGKAEATSSLANDNDSQEKLDADNQSEMSRKSRRRRQRKRQQQLLKSPSSPRPILMW
FRRDLRLLDNPALIGCLELGGPVIPLFLWNDQEEEGPGVTVAAGGACKYWLHQALVCLNQALERIGSHLLTVK
AEPSSAEVLQRLVKETGAGTVVATALYEPWLKERDEGVASTLQQQGISWQLHHSYCLRDPYTVTTQGVGLRGI
GSVSHFMSCCQQNPTGAQGTPLDAPPTVPVPSTWPTGCPLEQLGLARMPRRKDGTTVDWAVNIRRDWDFTEEG
AHARLEAFLQDGVYRYEKESGWADAPNTSCVSPYLHWGQLSPRWLLWDARAARCRSPKFQRKLAWRDLAYWQL
TLFPDLPWESLRPPYKALRWSSERAHLRVWQKGTTGYPLVDAAMRQLWQTGWMNNYMRHVVASFLVAYLHLPW
QEGYRWFQDTLVDADVAIDAMMWQNGGMSGLDHWNFVMHPVDAAMTCDPIGTYVRRWCPELAPLPDELIHKPW
KCPTSILRRAGVVLGQTYPERVVTDLEERRTQSLRDVAEVRQRFKEYVDQHSGCDLVPLPDRLVEEALGSTGE
VLRCGSRFLLPVITRMEFKHQTADPDKADGRGNPYNAVLKGYVSRKRDETIAFLNERDFTASVMHESTQQRER
AERHHCLLEGLPASPSSATSRGRPRRTPAKDPYSIVPGGVAMPQR
>Alosa_sapi__XP_041966611.1
MVVNTVHWFRKGLRLHDNPSLRDSIRGADTLRCVYILDPWFAGSSNVGISRWRFLLQCLEDLDASLRKLNSRL
FVIRGQPTDVFPRLFKEWDISRLSYEYDSEPFGKERDAAIQKLASEAGVEVTVRISHTLYELDKISELNGGQS
PLTYKRFQTLISRMDVVEEPVDTITPELMGHCVTPISDDHDDKLGVPSLEELGFETDGLSTAVWPGGESEALT
RLERHLERKAWVANFERPRMNANSLLASPTGLSPYLRFGCLSCRLFYSKLTDLYRKVKKNSAPPLSLYGQLLW
REFFYTTATNNPRFDKMEGNPICVQIPWDRNAEALAKWAEGRTGFPWIDAIMTQLRQEGWIHHLARHAVACFL
TRGDLWISWEEGMKVFEELLLDADWSVNAGSWMWLSCSSFFQQFFHCYCPVGFGRRTDPNGDYIRRYLPILRG
FPAKYIYEPWNAPEGVQKAAKCIIGVHYPKPMVNHAEASRVNIERMKQIYQQLSCYRGLGLLATVPSNSSNAA
EGMAAGPSSGDGTQEGASTSGGSLMVHQREGQVGTPVGAHGEPCHAETSHHHQQQQQQGFTMGEATTSHRQQA
QPATLQRGRGTTGTIKRRNEDFGSSRSKVQRQNSH
>Alosa_sapi__XP_041967619.1
MVVCGLLYLSALRHRSIVKFRPAKAEPLSQAFPGPCLRIYSTSDTIMAPKKADLKRKAQSLKTSRAKQQKVDR
VEGWLESQVAELRREARDMKFNNKRLRFLSDTETVQQGSEGVLYWMGRDQRVQDNWALIYAQQLAITEKLPLH
ICFCLMPTFLEATFRHYSFMLKGLQEVAKESSVLDIQFHLLSGFPGDTLPGFIKEWNMGVVVTDFWPLRDPTQ
WVDDVKKTISPDIPIIQVDAHNVVPCWEASGKLEYSARTIRGKITKLLPTFLTEFPVVDKHPHSSSRTAKAVN
WSEILSSLVVDRGVGEVDWAKPGTSGGMAMLESFIDQRLGDFAALRNNPNVAALSQLSPWIHFGHLSAQRVVR
QVQKNGKKFSDSVASFTEELVVRRELADNFCFYNKNYDNIEGAYEWARKTLQDHAKDKRTHVYTREQLEQAKT
HDKLWNAAQTQVLTEGKMHGFMRMYWAKKILEWTSSPEEALAIAIYLNDRYSLDGCDPNGYVGCMWSICGIHD
QGWAERPVFGKIRYMNYDGCKRKFDVAQYERRYSKKEV
>Amazona_aest__KQK77804.1
MSGSVRTAICLLRCDLRAHDNQVLHWAQSNADFVVPLYCFDPRQYLRTHCYGFPKTGPHRLRFLLESVKDLRE
TLKKKGSTLVVRKGKPEDVIHDLITQLSSVSAVVFHEEAXQEELDVEKELCRVCSQHGVKVHTFWASTLYHRD
DLPFRPIARLPDVYTHFRKAVESEAKVRPTLRMADQLQPLAPGVEEGCIPTMEDXGQKALIELVLENLLVXYA
NIXFPSEPEQPFSNWNLXSEGDQGEFRFPYIPCINLVASYKEIRNGLVGMDYSTKFAPWLALGCISPRYIYEQ
IKKYEKERTANQSTYWVLFELLWRDYFRFVALKYGRRIFSLREGKTGVPFVDANMRELAATGFMSNRGRQNVA
SFLTKDLGLDWRMGAEWFEYLLVDYDVCSNYGNWLYSAGVGXDPRDNRKFNMVKQGLDYDGNGDYVRMWVPEL
QGIKGADIHTPWALSSAALSQAGVTLGETYPQPIVTAAEWSRHISQRPVAFLRFFFMDIHSADIPTGE
>Amazona_aest__KQK79631.1
MRRRQRKRKASEEEEVPSVSLRRTEKEEKVQEAWRRAAPSVREFKFNKKRVRLISQGSDLKDDGRCILYWMSR
DQRLQDNWAFLYAQRLAXKQELPLHVCFCLVPKFLEATIRHYGFMLRGLQEVAEECAELNIAFHLLRGYAKDV
LPAFVVEHGVGGLVTDFSPLRLPWQWVQDVRERLPEDVPFAQVDAHNIVPCWVASPKQEYSAYTLRGKIHAQL
PEFLTEFPPVVHHPYSPSCPAEPIAWEACYSSLEVDRTVKEVEWATPGTAAGLAVLQSFIRKRLKYFGSHRND
PNKAALSNLSPWFHFGQVSTQRAILEVQKHQHKYKESVDVFMEEAVVRRELAENFCYYNENYDRVQGAYPWAQ
TTLKLHAKDERPFLYKLEELEQGTTHDPLWNAAQKILEWTHSPEEALQFAIYLNDRYELDGRDPNGYMPPSRG
NADAVCPHPAGCLWSICGIHDHGWTERAVFGKIRYMNYAGCKRKFDVGRFERRYAPCTLSQ
>Amazona_aest__KQK81400.1
MGVNAVHWFRKGLRLHDNPALRECIQGADTVRCVYILDPWFAGSSNVGINRWRFLLQCLEDLDANLRKLNSRL
FVIRGQPADVFPRLFKEWNIAKLSIEYDSEPFGKERDAAIKKLASEAGVEVIVRISHTLYDLDKIIELNGGQP
PLTYKRFQTLISRMEPLEMPVETITPEVMEKCTTPVSDDHDEKYGVPSLEELGFDTDGLPSAVWPGGETEALT

RLERHLERKAWVANFERPRMNANSLLASPTGLSPYLRFGCLSCRLFYFKLTDLYKKVKKNSSPPLSLYGQLLW
REFFYAAATNNPRFDKMEGNPICVQIPWDKNPEALAKWAEGRTGFPWIDAIMTQLRQEALTCLLRAKAVTFCS
RVWHLGTEHSVNIARRYLPVLRGFPAKYIYDPWNAPESIQKAAKCIIGVNYPKPMVNHAEASRLNIERMKQIY
QQLSRYRGLGLLATVPSNPNGNGGLMGYSPGESISGCSSAGGKSCIHKTVLRELSWELVMVIQLFSHVLGTLI
QQEQVEFSSKVPVKQVVSYIMLMETISNHTYCKQEERPLVLALVQGNAQIQKKKLRALDQKSSDRAQIKLHYW
KRPYHSKNTQDKIPATLYRN
>Amazona_aest__KQK85176.1
MAAVALGPAPARCRSVHWFRRGLRLHDNPALQEALRDAASLRCIYILDPWFAASSAVGINRWRFLLQSLEDLD
NSLRKLNSRLFVVRGQPTDVFPRLFKEWGVTRLTFEYDSEPFGKERDAAIIKLAKEAGVEVVIENSHTLYDLD
RIIELNGHKPPLTYKRFQAIISRMELPKKPAWVANYERPRMNANSLLASPTGLSPYLRFGCLSCRLFYYRLWE
LYKKVKRNSTPPLSLYGQLLWXEFFYTAATNNPKFDRMEGNPICIQIPWDRNPEALAKWAEGKTGFPWIDAIM
TQLRQEGWIHHLARHAVACFLTRGDLWISWESGVRVFDELLLDADFSVNAGSWMWLSCSAFFQQFFHCYCPVG
FGRRTDPSGDYVKRYLPKLKGFPSRYIYEPWNAPESVQKAAKCIIGVDYPKPMVNHAETSRLNIERMKQIYQQ
LSRYRGLCLLASVPSXVEDLSGPVIDSASGQGCSTSTAVRLSQADQASPKRKHEGAEELCTEELFKRAKVTGL
PAPEVPGKSL
>Amblyraja_radiata_XP_032894640.1_cryptochro
MVRNAVHWFRKGLRLHDNPALQRALSGADTVRCVYILDPCFAGSSNIRINWWRFLIQSLEDLDISLRKLKSRL
FVVRGQPTDVFPRLFKEWNVTQLTFEYDSEPFGKERDAAIMKLAKIAGVEVIVQISHTLYDVDRIIKANGNHA
PLTYKRFCAIIRKLKLPKAPVNTVTLDQMEKCTQNMQESQEEQYGIPSLEELGFDTSELCCAVWQGGETKALA
WLHKHFEGKACTASYESSNLNPDSLMSSSTGLCPYLCLGCLSCRKFYYTLLEFYRMDHNKSPLPLYGELLRRE
FFYTAATNNPRFDRMEGNPVCVQIPWDKNQQALAKWADGQTGFPWIDAIMKQLRQEGWIHHLARRAVGCFLTR
SDLWISWEEGMKVFNELLLDADFSVSAGWWMWLSCSAFFQRFLPCYCPVAFGRRTDPNGDYVRQYLPILRGYP
TKYIHDPWNAPVEVQAAAKCIIGVHYPRPMVNHIEAIRLNTERMKQIYLQLSHDKGLCLPVMALSLTDNQESS
IAKSDGHGKETGSCSANHDKTPNVFGKRGKPENAENRDEFKQIKIPRLQAPEDVSRIKAEPMVIED
>Amblyraja_rad_XP_032893747.1_cry-1
MGCNSIHWFCKGLRLHDNPALLESVQGVDTIRCVYIVDPWFAGTSSLAINRWGFLLQCLEDLDTNLRKLNSRL
FVIRGQPADVFPRLFKEWNISRLSFEYDSEPFSKKRDAVIKKLATEAGVEVIMRISHTLYDMEVIIERNGGQP
PLTYKHFQKLISQMGPVEMPVKMITSETMGRCTMPVLDDHGDRFGVPSLEELGFETEGLTSAVWPGGETEALA
RLERHLERKAWVANFERPGMNSKSLLASPTGLSPYLRFGCLSSRLFYFKLTELYKKVKKNSSPPLSLYGQLFW
REFFYAAGTNNPKFDKMEGNPICVQIPWDRNPEALAKWAEGKTGFPWIDAIMTQLRQEGWIHHLARHAVACFL
TRGDLWISWEEGMKVFEELLLDADWSVNAGNWMWLSCSSFFQQFFHCYCPVGFGRRTDPNGDYIRRYLPVLRG
YPAKHIYDPWNAPEDVQRAANCVIGVDYPRPMVHHAEASRLNIERMKQIYQQLSCYRGLGLLASVPSNPSGSG
GIMQYSAEETIPGCSNVADRNTLEGSLTAGKLFNPEEEMSGIGLKYQRQSMD
>Amborella_tri_blue-light_XP_006854471.1
MESNPHSDSNPSAPVEEQEEQQAQNTLSLSSSNLTTPYASISLSLSSLFSSSSILSSPKPPFKPSQTACFTHI
PLKPSVSLQFRNLNFSQAPKSNSKSSISAASLSTPLFQTPHLSSEPSNPVGGRKCSIVWFRNDLRVHDNEALN
SANEESISLLPVYIFDPRDYGKSSSGFDKTGPYRATFLLDCVSNLRENLKKRGSDLVVRIGKPESVLTELVKA
VGADGVYAHMEVSHDEVKGEERVEKVIRENGVECKLFWGSTLYHIEDLPFGLEDMPSNYGGFRERVKGLAVRK
TIEALDRLKGMPKRGGVEPGDLPTLMDLGLSPASTVSKDGKPAANASLVGGETEALERLKRFASECLAQPYKG
DKANGRDSIYGANFSCKISPWLAMGCLSPRFMFNELKKGAKRMGFASVSQKTNEGATDGGMNWLMFELLWRDF
FRFITKKYSSNKKNIAATPATACTGALA
>Amborella_tri_ERN14624.1_AMTR_s0003
MTRFGVELATLHGDTSRCRSDTLTFEDESEKGSNALLARAWSPGWSNADKALTNFINGPLIEYSINRKKADSA
TTSLLSPHLHFGEVSVRKVFHLVRIKQVFWANEGNTAGEESINLFLKSIGLREYSRYLTFNHRSSHERPLLGH
LKFFPWVVDESYFKAWRQGRTGYPLVDAGMRELWATGWLHDRIRVVVASFFVKVLQLPWTWGMKYFWDTLLDA
DLESDALGWQYISGTLPDSREFDHIDNPQIEGYKFDPQGEYVRRWLPELARLPTEWIHHPWDAPHSVLQAAGV
ELGTNYPLPIIEISAAKERLQEALSEMWQLEANSRAAMENGTEEGLGDSSESPPLDFPQEEMEVDNQPARPTG
QIGATRRLADQMVPSMTVSSFRVEEEEEPSSNTGSSRDNRPEVPSNASFGSEADRRDEVLNLGNMGELERERN
ASEFNVSVSLQNAVHFTAESSSSLRERDGGVVPVWSPSTSSRSEQLIGEESGLGNGGSYLQRHAQSHQLMTWR
QISRTRSRNWEVDNAVQPNAIG
>Amborella_tri_XP_006841901.1_(6-4)DNA_p
MEVNSLMWFRKGLRIHDNPALDYATKNAKHLYPVFVLDPLYLNPDTSAFSPGSKKSALNRIQFLLESLSDLDS
SLKALGSRLLLFNGDPVHTIKRLLNRWNIGRLCFEFDTEPYSLTRDGQVQDFASHTGIEVFSPVSHTLFDPRE
VIKKNGGKPPLTYQSFLRIVGIPSPPHANTISSLPPLGNIEDSEVSNVPTIEELGYADVKQEEYSPFRGGESE
ALKRLKASLMDKDWVANFEKPKGKPTSFENPATTVLSPYLKFGCLSSRLFYQGLQDVFKEVKKHTLPPVSLVG
QLLWREFFYVVAIGTPNFDQMKGNKICKQIPWSGDDKLFIAWREAQTGYPWIDAVMVQLRKWGWMHHLARHAV
ACFLTRGDLFVHWEKGRDVFERLLIDSDWAINNGNWLWLSCSSFFYQYNRIYSPTSFAKKYDPSGIYIRHFIP
VLKDMPEDYIYEPWTAPLEVQIKANCVIGRDYPKPVVDHDIVSKDCRRKMGAAYAMNSTASDVVTGEELGSLG
RKLEKQPIQISDTKKQKQLKFTDYLG
>Amborella_tri_XP_006851849.2_photolyase

MYLCRISPLNLCYSRVLGLFIQASSLKVPLISSFCTSRLSSSSSMEPAKKSMKKRSEEGAPSSNGRLLKKLKN
KSGIGMESEPEDEETGVKVQSGRVRVLKAGETGGNGPVVYWMFRDQRCNDNWALVYALEQGARLNVPVVVAFN
LFDQFLGAKARQLGFMLRGLRDLEQSLKVLGVPFFLFRGEADENIPKFTSQCNASLLVTDFSPLRIIQNIKEN
ICHRVTEEIPVHEVDAHNIVPVWVASDKLEYGARTIRTKINKLLPEYLTEFPDLKAPQIRWKDESPVEIDWEN
LIAEVLRKGGAVPEVHWIEPGEQAALDMLMGSKNGFLSTRLKNYSTDRNNPSRPKGLSCLSPYLHFGQISSQR
CALEARKFRKLYSQSVDSFLEELIVRRELADNFCFYQPNYDSLQGAWEWARKTLRDHAGDKREHIYTMEQLEK
AKTADPLWNASQLEMVHYGKMHGFMRMYWAKKILEWTSGPEEALSIAIHLNDKYELDGRDPNGYVGCMWSICG
IHDQGWRERSVFGKIRYMNYAGCKRKFDVDGYIAYVKKLAAETKKRVPIKEIMEHNN
>Amborella_tri_XP_020524346.1_cry-1_isof
MNMGTHCKTIVWFRRDLRIEDNPALAQAARVGCVLPVYIWCPKEEGQFYPGRVSRWWLKQSLVHLDQSLRSLG
APLLLIKTEDTLSALLRCISAIGATQVVYNHLYDPVSLVRDHCVKQKLVEQGIIVQSLNGDLLYEPWEVYDDE
GLAFTTFDEYWNKCISMSVEPVCLLPPRRLVPASEKMVGCTIEELGLENEYEKSSNALLSRAWSPGWCHADKA
LSEFISQRLEDYSMNRLKAEGLGTALLSPYLHFGELSIRKVLHSVRMKQLSWAREGNSDAEESVNLFLRSIGF
REYSRYLCFNFPFTHEKSLLGNLKFFPWRTDHSNFKAWRQGRTGYPLVDAGMRELWATGWLHNRIRVVVSSFC
VKFLQLPWTWGMKYFWDTLLDADLECDILGWQYISGSLPDGHELDRLDHPEDQGYKFDPEGEYVRQWLPELAR
MPTEWIHHPWDAPVSVLKAAGVELGLNYPNPIVEIDSAKECLAQSLSKMWELEAASRTAKANGSDENVGDNLN
SINEIPNVPRVVIKKKEAPESSSWDQRVPTVQSMHKRNGVNNLVGEDVNRVNLRNQINGGEVLSRNDEMSSTA
DSSSIRKRSLNESCFNVPSSSLPQSGSWLKGIDIDEVSTSKVWLKASS
>Amborella_tri_XP_020526959.1_cry_DASH,_
MTLSLSSPISAFPLKRLFFPTSLKPSIPTPVFLSLSLAKHCLNMSSVPVSLPKYDFQTNWDVPQFSDDQMDAV
ADETLKRYSLSRDRKRNESGVAIVWFRNDLRVLDNEALFQAWKSSQAILPVYCLDPRLFGTTHYFGFPKTGAV
LRAQFLIDCLANLKKNLMKRGLNLLIQHGKPEDILPSIAKAFGAHTVYAHQETCSEEILVEKLLTNSLRRVVL
PPVDIATSIPKTPKLQLIWGSTMYHLDDLPFGSSNVPDVYTQFRKSVESKCKIRGCIKIPPSLGPLPSGSLDE
IGGWGCVPSLDQLGLNMEKHNGGMHFIGGENAALARVNEYFWNKDLLRVYKETRNAMLGPDYSTKFSPWLAFG
CLSPRYAYEEVKRYEKERVANDSTYWVLFELIWRDYFRFLSIKYANSIFHLGGPRRVKGLNWNQDMALFESWR
DGRTGYPLIDANMKELNSTGFMSNRGRQIVCSFLVRDMGLDWRMGAEWFETCLVDYDPCSNYGNWTYGAGVGN
DPREDRYFSIPKQAQTYDPEGEYVSYWLPELRVLPKEKRHFPGTSYIKQVVPLKFGNANPRNSNQFKGQSHRG
GFERRKGQRS
>Ameiurus_mela__KAF4089512.1
MRGISGRIIRCGLEVRNFSVIMSENKRNLKRKGEPMKGLEKPGAARGAERGAGWLQEKVAEMRRNSSECKFNT
KRLRFLSDAKKARQGCGAVLYWMARDQRVQDNWALVYAQQLALEENLPLHVCFCLVPRYLEATYRQYAFMLRG
LQEVAKECTSLSIQFHLLRGEPELTLPGFVKHWDVGAVVTDFNPLRLPLQWTERVKKELPPDIPFVQVDAHNV
VPCWGASSKLEYAARTIRGKITKLLPEFLTEFPPVVMHPHPSTKTAKKVDWEKVMSSLEVDRSVGEVLWAQPG
IAGGVTMLESFIDQRLRIFASERNNPNANALSQLSPWIHSGQLSAQRVLLEVKRWGKHSTESVSSFTEELLVR
RELSDNFCFYNDNYDNINGAYDWAKKTLQDHAKDARPYLYTREQLENADTHDQLWNAAQRQLMLEGKMHGFMR
MYWAKKILEWTASPEEAVNISIYLNDRYALDGCDPNGYVGCMWSICGIHDQGWAERPVFGKVRYMNYAGCKRK
FDVGQFERRYAGKKA
>Ameiurus_mela__KAF4091667.1
MVVNAVHWFRKGLRLHDNPSLLESVVGADSLRCIYILDPWFAGSSNVGINRWRFLLQCLEDLDASLRKLNSRL
YVIRGQPTDVFPRLFKEWKITRLSYEYDSEPFGKERDAAIKKLANEAGVEVIVRISHTLYDPEKIIELNGGQS
PLTYKRFQTILSRMDAVDIPAETITAEILSKCIMTVCDDHDERFGVPSLEELGFETKALPSAVWPGGETEALT
RLERHLERKAWVANFEHPRMNANSLLASPTGLSPYLRFGCLSCRLFYFKLTDLYKKVMKNSVPPLSLYGQLLW
REFFYTAATNRPSFDKMEGNPICVQIPWDRNSEALAKWAEGRTGFPWIDAIMTQLRQEGWIHHLARHAVACFL
TRGDLWISWEEGVKVFEELLLDADWSVNAGSWMWLSCSSFFQQFFHCYCPVGFGRRTDPNGDYIRRYLPILRG
FPAKYIYDPWNAPESVQKLAKCIIGTHYPKPMVNHAEASRINIERMKQIYQQLSCYRGLGLLASVPSNASGNR
EDAGASLNSTDHTQESSAAGRGTHTLKRHSEESVSNLGCKIQKKNSDEPHSVTTAHTSQAVSRM
>Ameiurus_mela__KAF4094369.1
MVVNSVHWFRKGLRLHDNPALQEALNGADTARCVYVLDPWFAGAANVGVNRWRFLLESLEDLDTSLRKLNSRL
FVVRGQPADVFPRLFKEWNVTRLTFEYDSEPYGKERDGAIIKLAQEFGVETIVRHSHTLYNPDRIIEMNSNNP
PLTFKRFQAIVSRLELPRKPLSTITPEQMASCSAQLSENHDEHYRVPSLEELGFKTPGEPVWRGGETEALDRL
NKHLDKKAWVANFERPRIKAHSLFASPTGLSPYLCFGCLSSRVLYYHLRELYMKLRRHSSPPLSLFRQLLWRE
FFYTAASNNPSFDRMEGNPICVQIPWDQNPEALAKWAEGRTGFPWIDAIMRQLRQEGWIHHLARHAVACFLTR
GDLWISWESGMRVFEEMLLDADWSVNAGSWMWLSCSAFFQQFFHCYCPVGFGRRIDPSGDYIRRYIPELKDYP
SRYIYEPWNTPESLQKAANCIVGVDYPKPMINHAESSRLNIERMKQVYQQLSHYRGLSLLASVPTIQEEAEPS
MSDDSQASSSGALSASRQPQSECRPSQTDPDPNQDPDPEPSSSAPAAMRKRACSDRADEDEGQ
>Amia_calv__MBN3298502.1
MSRDQRVQDNWALLYAQQLALAERLPLPMIFDSVMIFFLSISFYLLLFSLSTVYSDFLCVYQECSALGLEFHL
LQGCAGERLPRFVQELGVGAVVTDFSPLRVPLQWVEEVKKALPRDISLIQVDAHNVVPCWVASNKQEYSAKTI
RNKINSKLPEFLKEFPLMAKHPHCASKPAKPVDWERARSSLEVDQSVKAVSWARPGSVAGLAVLESFIDQRLQ
YFDSLRNNPNSDALSNLSPWLHFGHVSAQRVVLEVERSGQRWPSSVKAFVEETVVRRELADNFCFYNKNYDCV

EGAYEWAQKTLRDHAEDPRPYLYTRDELEAAKTHDKLWNAAQFQMVSEGKMHGFLRMYWAKKILEWTSSPHEA
LAIAIYLNDRYELDGRDPSGYVEVGFDRYKLVVQVVIGEQRGEGVKMAARCFWDADTDSYAQDIYMNDSLFCV
AAVFGCFHY
>Amia_calv__MBN3300458.1
MPHSCIHWFRKGLRLHDNPALLASLKDCAELRPIFILDPWFPKTMQVGINRWRFLIGALKDLDSSLRKLNSRL
FVVRGTPAEVFPRLFKQWKVTRLTFEIDTEPYAQQRDAQVLSLAEEHGVEVIQKISHTLYDTERIIVENNGKA
PLTYNRLQALLKTLGGPKRPVPRPTLEDMKGTCTPFSEQHDEEFGVPTLEELGQDPQKAGPELYPGGEGEALR
RLEQHMERVSWVCNFQKPETEPNTLNPSTTVLSPYVKFGCLSARTFWWRITDVYHGKKHSQPPVSLHGQLLWR
EFFYTAGMGIPNFDRMEGNPVCVQVDWDSNQEHLAAWREGRTGFPFIDAIMTQLRLEGWIHHLARHAVACFLT
RGDLWISWEEGQKVFEELLLDADWALNAGNWQWLSASAFFHQYFRVYSPIAFGKKTDKNGDYIRKYLPVLKKF
PVQYIYEPWKAPRSIQERAGCIVGKDYPRPIVEHDTASKKNLQRMKLAYARRSGTSEEPESKGKGEPGTAQSS
NWDQSFIILVQSIPSSSYVTYFQLLFHPLSLLLKYNPWATH
>Amia_calv__MBN3301867.1
MVVNTVHWFRKGLRLHDNPSLRESIQGADTVRCVYILDPWFAGSSNVGINRWRFLLQCLEDLDASLRKLNSRL
FVIRGQPADVFPRLFKEWNINRLSFEYDSEPFGKERDAAIKKLASEAEVEVIVRISHTLYDLEKIIELNGGQP
PLTYKRFQTLISRMDPLETPVEPITPEVMGKCTTPLSDDHDEKFGVPTLEELGFDTEGLPSAVWPGGETEALT
RLERHLERKAWVANFERPRMNANSLLASPTGLSPYLRFGCLSCHLFYFKLTDLYKKVKKNSSPPLSLYGQLLW
REFFYTTATNNPRFDKMEGNQICVQIPWDKNPEALAKWAEGRTGFPWIDAIMTQLRQEGWIHHLARHAVACFL
TRGDLWISWEEGMKVFEELLLDADWSVNAGSWMWLSCSSFFQQFFHCYCPVGFGRRTDPNGDYIRRYLPVLRG
FPAKYIYDPWNAPESVQKVAKCVIGVHYPKPMVNHAEASRLNIERMKQIYQQLSCYRGLGLLASVPSNPTGSG
NGGMVGFSSGEGMHASHTPTGEYSLPFFFFM
>Amia_calv__MBN3303224.1
VLHWAQRNAEHIVPLYCFDPRHYLGTHCYNFPKTGPFRLRFLLESVKDLRETLQKKGRNLLVRKGKPEDVVGD
LIKQLGSVSAVAFHEEATKEEQDVEKALKQVCSQFKVKVHTCWGSTLYHREDLPFNPIARLPDVYTQFRKAVE
TRSRVRPTITMPECLKPLPLGLEAGSIPSPEDLGQTDCLSDPRTAFPCSGGETQALARLKYYFWDTNLVASYK
ESRNGLIGMDYSTKFAPWLALGCISPRYIYEQIKKYENERTANQSTYWVIFELLWRDYFRFVAVKYGNRIFYL
KGLQQKSVPWKKDSKLFDAWKEGRTGVPFVDANMRELALTGFMSNRGRQNVASFLTKDLGLDWRMGAEWFEYL
LVDFDVCSNYGNWLYSAGIGNDPRENRKFNMIKQGLDYDNSGDYVRLWVLELRGIKEGDVHTPWTLSSAALAH
AEVSLDGTYPSPIVTAPEWSRHVRNKPVSSPLDPLSLRSKGAALEFTAWVSLLILLQDWCFSSVQGHVGKLPL
GL
>Amia_calv__MBN3303333.1
MTHRTIHLFRKGLRLHDNPALLGALESSAVVYPVFALDRDFLQAGALTGGLRWRFLLQSLQDLERSLATLGSR
LYVLQGRYEQVLRDRVRRWGVTQVTFDTEVEPYYTQQEDCIRALGREMGFGVRSCVAHTLYDLRRIVQANGGE
PPLTYKKFLHVLALLGDPEKPARQITPEDFQKCHTPTEEGTEQDRVPSLEDLGIAVDTEPLWIGGESQGLQRL
EKHMENQGWVENFAKPRTIPNSLLPSTTGLSPYFSTGCLSVRTFYHQLSNIYAQSKNHSLPPVSLQGQVLWRE
FFYTVASATPNFTKMASNPICLQIDWATKAEALEKWKMAKTGFPWIDAIMTQLRQEGWIHHLARHAVACFLTR
GDLWISWEEGMKVFEEYLLDADYSVNAGNWMWLSASAFYHQYTRIFCPVRFGRRTDPEGQYLRKYLPVLKNFP
SKYIYEPWTAPQHVQEEAGCVIGRDYPLPMVDHREASERNLVLMKRVHSEQENTAQLTRDVADNPMEIRMKRA
YPKHVEETGRADGFLETTGCAKHFHHEGELKSLPCKWPSKAIQVPELSGEVM
>Amia_calv__MBN3308662.1
MAVNSVHWFRKGLRLHDNAALQEALSGADSVRCVYVLDPWFAGSASVGVNRWRFLLESLEDLDLSLRKLNSRL
FVVRGQPADVFPRLFKEWNVTRLTFEFDSEPFGKERDAAIVKMAREFGVETIVKNSHTLYSLDRIIELNNNSP
PLTFKRFQAIVSRLGLPKKPLGTVTAEQMDRCRTDISENHDEHYRVPSLEELGFKTHGISPSTWKGGETAALE
RLHKHLERKAWVANFERPRINAYSLMASPTGLSPYLRFGCISCRLFYYNLLELYRQVRKNSSPPLSLFGQLLW
REFFYTAATNNPKFDQMEGNPICVQIPWDRNPEALAKWAEGQTGYPWIDAIMTQLRQEGWIHHLARHAVACFL
TRGDLWISWESGMRVFEELLLDADFSVNAGSWMWLSCSAFFQQFFHCYCPRVHTFKCAPFPPRRYIPKLKEYP
NRYIYEPWNAPEAVQKAANCIIGVDYPKPMVNHAEASRLNIERIKQIYQQLSHYKGLSKFLPLVNLLNLNQHH
QHI
>Amphibalanus_amp_KAF0290832.1_cry_DASH
MSVTDTDLSFFHPKVLHKAHHSAELVVPLYCFDPRHFASTHHFGFPKTGAHRARFLLESVRDLRGRLAERGSG
LVVRSGTPETAVPELAAALAGHKLSLVLQKEATDEEVLVERALAERCRALGVPVHTVWGATLYHRDDLPFAVN
KTPDVYTQFRKAVEGGGRVRPPLQMPDRLRPLPDGLDCGPLPAPADLGLPEGESTGEPRTAFPWPGGETSGLA
RVHHYLWETDAVATYKETRNGLSGEDYSTKFSPWLSLGCLSPRYIHSEIGRYEKQRTANKSTYWVLFELLWRD
YFRFVALKYGNRLFHLTGLQNKKVPWKNDMEAFNRWKGGSLGIDRG
>Amphibalanus_amp_KAF0300247.1_cry-1
MSPPVVIHWFRKGLRLHDNPALLKAVDYATQSGYHLRPVFILDPQFTRSGRVGANRWRFLVGALSDLDSSLQL
LGSRLLVCRGSPETVLPKLIREWDVRRITFETDTEPYAVARDARVAQLARELSCAVTSVTSHTLYEPAQVLAA
NGGKPPLTYQRMVTLVEQLGAPVAAEPAPRSLPPPGQPAGHFSVPSLAELGVDAEQLGEELYPGGETEGLARL
ERSLARPDWVCRFEKPKTAPNSLQPSTTVLSPYVKFGCVSARLFYHRLADVYSRRGNYTRPPVSLHGQLLWRE
FFYVVGANTPNFDRMEGNAICRQIPWIENEEFLKAWSEGRTGYPWIDAVMTQLRQEGWIHHLARHAVACFLTR

GDLFISWEHGMKVFDELLLDADWSLNAANWMWLSASAFFHQFFRVYSPVAFGKKTDPNGDYIKKYLPKLRKYP
SQYIYEPWKAPIATQKAAGCIVGRDYPKPIVDHDKVRPRNIEWMAAAYKKGKAAHPAPAAGLGADSQDGKGHS
G
>Amphibalanus_amp_KAF0301665.1_cry-1
MTRPNTVCTGFRKGLRLHDNASLLEGVAGSTTFRCIYILDPWFAGSSNVGINKWRFLLQCLEDLDKSLQRLNS
RLFVIRGQPADVLPSLFKKWNITCLSFGEDPEPFGKVRDQNVRKMALEHNVEVISRTCHTLYDVDTVIERAGG
RSPLTYKQFQTVLLSMPPPPAPLHALNHDRFDTESAHKGVWHGGETEALVRLERHMERKAWIASFGRPKMTPQ
SLKASVTGVSPYLRFGALSPRLFYRDLAYLYRKVSSLSPRLFYRDLAYLYRKVSSLSPRLFYRDLAYLYRKSV
SWLFYRDLAYLYRKVSSLSPRLFYRDLAYLYRKIKHADPPLSLHGQLLWREFFYVAATNNPNFDRMVGNPICV
QIPWDKNPKALAKWASGQTGFPWIDAIMTQLRQEGWIHHLARHAVACFLTRGYLWLSWEEGMQVFEELLLDAD
WSVNAGTWMWLSCSSFFQQFFHCYSPVRFGRKVDPEGDYIRTYLPVLKGMPSRYIHEPWLAPKSVQKAAKCIV
GEDYPLPMVCHTDASRINVERMRQIYSRLLGYKGHKGELMGERRAARRVLLLPCKGKITMLAC
>Amphibalanus_amp_KAF0313106.1_cry-1
MKEAVGVHWFRHGLRLHDNPALCQLLETCNKVYLIFIFDGESGGMRLSGYNRLAFLLESLTDLDRQLRLRGGR
LLTIRGKPTDALQLMRTEYGMTHLSFEQASGEGGDCEPVWAPRDELVRAWCDEHAVEIIERISHTLWDPAEVI
EANGGEPPLTFSMFHQVAMSIGLPPTPEGDPVFRAGQLAVADPTRPHGHPVQASAPDCSVYRLTEFSNLTPLP
FQLFDEVPPVSELSDLKPSPGEHKKRYQGGETRGLQLLAERVAWEELALSRGIVMPNQYCPDLLGPPRSMSAY
LRHGCISVRRMYHQIQKAFQACTDAQGHSLSICVQLVWREYFYTMSVKNPYYDQMEPNPICLNINWRTDDQQL
QQWKEGQTGYPFIDAAMRQLIQEGWIHHVARNATSCFLTRGDLWLNWVDGLRVFQEYLLDADWSVNAGNWMWV
SSSAFENFLDTSDTISPVNYGRMFEPTGEYIRRYVPELRQMPTEYIFSPWEAPLSVQREAGCIIGKDYPCRMV
NHEVVSRHNRKLMSKIAEQLGTCPPHCMPAGDAEVKHFFGLPGASLEDMLGGH
>Amphibalanus_amp_XP_043225499.1
MAEPASKKQKKDDSLSQSQSIHQQVEERRKEFGKPGWKFNKKRVKVLTAEEIPDKAKSVVYWMSRDQRVQDNW
AFLFAQKLALKFKLPLHVCFCLVPKFLDATIRHYGFMMKGLQEVEKECSQLGINFHLLVGPASNTLVQFAKDH
QTGAVVTDFSPLRVPRQWLDEVIKALPKDVSVCQVDAHNVVPCWVASEKQEYGARTIRRKIMDRLPEYLTEFP
AVVRHPHPAQQAVKPVDWAAAEASLQVDRSVTEVQWARPGTAAALSQLSEFCSGRLRLFADKRNDPTVAALSN
LSPWLHFGQLSAQRAVLEVRRLRSRHSKAVEAFVEEAVVRRELADNFCFYQPHYDSVQGAYGWAAETLKVHSK
DKREYLYSREQLAEAQTHDDLWNAAQLQLVKEGKMHGFLRMYWAKKVLEWTRSPEEALATAIYLNDRFSLDGR
DPNGYVGCMWSICGIHDQGWAERPVFGKIRYMNYQGCKRKFNVDAFVARYGGKKHKYVPPKE
>Amphimedon_que_XP_003386569.1_cry-2-like
MDSYSNGFYEEEERTLTVFNNVDGASEEEQDSFFDSYSASASSSPSSLCSSNWHSERLPVSLHWFRSFSLRLH
DNSALMASLLRPQTQFRAVFVLDPWFTETDKKFGANRLKFLLECLHDLSNQLEALGLKLYVAQGQTTAVLAGL
CQEWGVTHLSYQKSQEPRSKVEERTISEMASMMNIEVEEFHNHTLYSPTELIKLNEGKPILTFKDFRTLLSKL
KPPAVPISSPSTKQKYNEDNIPMFKVQTFQIPSLASLGCYDVELGTGPWVGGETEALKRLDNYCTVRSKPFDN
FLDCLFDKTSLSPYVRFGCLSVRHFWHYVRQLASTDKSKMTLVQEVSSKLLQREFYFLVSSQVPNFDTDTQNP
ICIQLPWEFDADGLRRWRSGMTGYPWIDAAIRQMLKEGWIHNLLRESIATFLTRGDLWISWLKGAESFEMYML
DYEPSVSCGCWMKSSSTAFITGTIEHYNPISYGQQLDPNGDYIRHFVPEVKNLPKEYIFCPWMAPIEVQREAG
CIVGTDYPHPVVDHMTAGVICMERLKSFMQSLSSHQTNP
>Amphimedon_que_XP_003388746.2_photolyase
MAAGSQAAGAARGAALQESLLIAGSSFNMKRCRLITKPTAGKSSIVKGPVLYWMSRDQRVQDNWGLVYSQELA
NKHGVPLLVAFTLVPKFLDATWRQYSFMMSGLQEVEKELLKLKIPFHLLLGKAQSCLPSFIAKESVSVVVCDF
SPLRVPLGWVKETGAELDKIKVPLVQVDAHNIVPVWLASDKQEYAARTIRNKIHKFLPEFLTEFPPVTVHTHN
SKLTMKSTNWIKARESLEVDMTVSEVSWATPGTNAGLKVLDDFCTKRLKFFAAQRNDPNKDSLSNLSPWYHFG
QVGVQRAILKVKSYSSKHSESVSAYIEEAVVRRELADNFCYYNPHYDSISGAAQWAQDTLKAHKKDKREYIYT
QEQFESSSTHDPLWNAAQLQMVQEGKMHGFLRMYWAKKILEWTSSPEEGLRIAIYLNDKYEIDGRDPNGYVGC
MWSICGIHDQGWAERSVFGKIRYMNYQGCKRKFDVGQFERKYNKKRKLDKDELPAAFKKFKKQK
>Amphiprion_ocel__XP_023118164.1
MVVNTIHWFRKGLRLHDNPSLRDSIRGADTLRCIYILDPWFAGSSNVGINRWRFLLHCLEDLDASLRKINSRL
FVIRGQPTDVFPRLFKEWQITHLSYEYDSEPFGKERDAAIQKLASEAGVEVMVRISHTLYNLDNIIELNGGQP
PLTYKRFQGLINRMDAVELPAETITSDVVKNCLTPISEDHDDKFGVPSLEELGFETEGLTTAVWPGGETEALM
RLERHLERKAWVANFERPRMNANSLLASPTGLSPYLRFGCLSCRLFYFKLTDLYRKVKKNSSPPLSLYGQLLW
REFFYTTATNNPCFDKMEGNPMCVQIPWDRNPEALAKWAEGRTGFPWIDAIMTQLRQEGWIHHLARHAVACFL
TRGDLWIRWEEGMKVFEELLLDADWSVNAGSWMWLSCSSFFQQFFHCYCPVGFGRRTDPNGDYIRRYLPILRG
FPAKYIYDPWNAPEEVQKAAKCMVGIHYPKPMVNHAEASRINIERMKQIYQQLSCYRGLGLLATVPANPNSGA
NGSNTGTNQGAGRSSEDPSLQEGTSRTERGQSMQKRRREETPPESSSKSWKQSK
>Amphiprion_ocel__XP_023122072.1
MSTSRTVICLLRNDLRLHDNELFHWAQRNAEYIVPLYCFDPRHYVGTYNYNLPKTGPFRLRFLLESIRDLRNT
LLNKGSNLVVRRGKPEEVVADLIKQLGSVSSVAFHEEVTSEELNVEKKVKEVCAKMKVRVHTCWGSTLYHRDD
LPFNHMSRLPDVYTQFRKAVEAQSRVRPVFPSPDKLNPLPSGLEEGAIPSAEDLQQTEAVNDPRSAFPCSGGE
SHALARLKHYFWDTDAVATYKETRNGLIGVDYSTKFAPWLAMGCISPRYIYDQIKQYESKRTANQSTYWVIFE

LLWRDYFKFVAVKYGNRMFQLKGLQDKSVPWKKDMKLFNAWKEGRTGVPFVDANMRELAMTGFMSNRGRQNVA
SFLTKDLGLDWRMGAEWFEYLLVDHDVCSNYGNWLYSAGIGNDPRENRKFNMIKQGLDYDGNGDYVRQWVPEL
QAVRGGDVHTPWTLSTAALSHAQVSLGDTYPTPIVIAPEWSRHANKKPTGMGPSPRGKKGPSHTPKQHRDRGI
DFYFSKSKNL
>Amphiprion_ocel__XP_023146997.1
MPPPAADGQTEVRKMLREVLVGREDPEGFFAMCVSILGHRETRTVFPSLIQPLSTANRALHSILISIYQEYFT
KTEDDELELVLALSLLEMKDQQPSVPSQECQLLQQPGDRHNQSSSVQLSSGSKSQGSSYTQLADVPGTRKNTD
SAQTGPWRKESPPQTTKETELLKSIQLDKRKKQTPGTGQPVSRQDTVRADDEMMGEEDLNQSEKPKRSKNRRQ
RRKGAGQQLLGLPGSPSAPPPVLLWFRRDLRLCDNPALMGSLEVGAPVIPVFIWSPEEEEGPGITVAMGGACK
YWLHQALSCFCSSLERIGSHLVFLKANREGNEVGSSLQTLKELVKETGARTVLANALYEPWLKERDDAVVSAL
QKEGVECRVFHSYCLRDPYSVSTEGVGLRGIGSVSHFMSCCKQNPSSTLGVPLDPPVSLPTPSHWPQGAPLDT
LGLARMPRRKDGTTIDWAANIRTSWDFSEEGAHARLDAFLHDGVYRYEKESGRADAPNTSSLSPYLHFGQLSP
RWLLWDAKGARCRPPKFQRKLAWRDLAYWQLTLFPDLPWESLRPPYKALRWSSDRSHLKAWQRGKTGYPLVDA
AMRQLWLTGWMNNYMRHVVASFLIAYLHLPWQEGYRWFQDTLVDADVAIDAMMWQNGGMCGLDHWNFVMHPTD
AAMTCDPYGSYVRKWCPELADIPDELIHKPWKCPASMLRRAGVVFGQTYPERIITDLEGRRNQSLQDVALVRR
EFGQYVDKRSGCDLVPLPPRLVSEALGLSHMGGAVVKEGKEFLLPVITRMEFKHQLEDPDADAASNPYNAVLK
GYVSRKRDETIAFLNERDFTASVMYEGAQRRERLESDYRRMEGLPLLPSPRGRVRRTPTAKDRFSIVPGGAVT
SLK
>Amphiprion_ocel__XP_023150648.1
MLHCVCRSSSRISLLLIHSNRRPITVVFSTDSFTSETTMSDKKRKATSAAAAKEPSAKQPKLAPVKAEKKAAG
WLQSLVQQQRSEKNDMKFNKKRLRFISDAVKIKQGSEGVLYWMLRDNRVQDNWALIHAQRLAMKENLPLHVCF
CLVVPKSQLSTLRHFSFMLKGLEEVAKECKHLDIQFHLLHGAPGEVLPGFVSDRSFGAVVTDFSPLREPLQWL
KDVKKRLPKEIPLMQVDAHNIVPCWEASPKLEYAARTIRGKITKVLSDFLTDFPLVEKHPHTATRTAKPVDWD
KTLASLSVDRAVGETEWAKPGTKAGMAMLESFIDVRLKLFDTQRNDPNAAALSQLSPWIRFGHISAQRVALQV
QRSGRTAGVSVASFIEELVVRRELTDNFCFYNEKYDSVEGAYEWAQKTLKDHAKDKRPYLYTKEQLEKAKTHD
KLWNAAQYQMVSEGKMHGFLRMYWAKKILEWTSSPEEALSITLYLNDRYELDGQDPNGFVGCMWSICGIHDQG
WGERPIFGKIRYMNYKGCLRKFNVAQFEKKYCPKNL
>Amphiprion_ocel__XP_023151758.1
MVVNSVHWFRKCLRLHDNPALQEALNGADTVRCVYILDPWFAGAANVGINRWRFLLEALEDLDNSLKKLNSRL
FVVRGQPTEVFPRLFKEWNVTRLTFEYDPEPYGKERDGAIIKMAQEFGVEAVIRNSHTLYNLDRIIEMNNNSP
PLTFKRFQTIVSRLELPRRPLPPITRQQMDKCHTQIGENHDLLYSIPSLEELGFRTEDLSPAVWRGGESEALD
RLNKHLDKKVWVANLEHPRVNTCSLYASPTGLSPYLRFGCLSCRVLYYNLRELYMKLRKRCSPPLSLFGQLLW
REFFYTAATNNPNFDRMEGNPICVQIPWDQNPEALAKWAEGRTGFPWIDAIMTQLRQEGWIHHLARHAVACFL
TRGDLWISWESGMKVFEELLLDADWSVNAGSWMWLSCSAFFQQFFHCYCPVGFGRRTDPSGDYIRRYIPILKD
YPNRYIYEPWNAPESLQKAANCVVGVDYPKPMINHAEGSRLNIERMKQVYQQLSHYRGLSLLASVPTIQEEAE
PPMTDESQTSSGPDSPPRDAADGEAAGCSVAPDSSTVCSFSVPPPDLEDRPPCTSSLPRLPSPRSKPSSPSST
SSPSPSPASVAQRRKGIARKARRSQRQRGRPSCPPAARGEEMGEERMEEEEEQLEEKMEEEEKMEEEEEATGQ
QQ
>Amphiprion_ocel__XP_023153353.1
MAHTCIHWFRKGLRLHDNPALMAALRDCKELYPVFILDPHLHNNSCVGINRWRFLIGALKDLDCSLRKLNSRL
FVVRGRPQDVFPKLFQKWNVTKLTYEYDTEPYSLSRDKKVTELAKEHGIEVIYKISHTLYDIERIIEENNGKT
PLTYNRLQAIVKGLGSPKRPIPAPTMENMKDVKTPCSEKQEKKYGIPTLEELGHDTTSLGEDLFPGGEREALR
RLEEHMKRTGWVCGFEKPQTSPNSLSPSTTVLSPYVTFGCLSARTFWWRLADVYQGKKHSAPPVSLHGQLLWR
EFFYTASVGIPNFNKMEGNHVCTQVDWDTNPEYLAAWREARTGFPFIDAIMTQLRQEGWIHHLARHAVACFLT
RGDLWINWEEGQKVFEELLLDGDWALNAGNWQWLSASTFFHQFFRVYSPVAFGKKTDKNGDYIKKYLPHLKKF
PAQYIYEPWKAPHSIQQAAGCIVGKDYPRPIVEHEVISKKNIQRMKMAYAKRSPDTTESPSKKEGAKRKAPSV
VDMLKKKSRTN
>Anabarilius_grah__ROJ13861.1
MSSLFSTCLYIRYSRRALLLRAISDPNTILLHYNSMSANKRSLKRQKESPSSAGGKQPKLTAERGRESGWLLK
EVTELRKAEQGCEFNKKRLRYLSSSQKIKQGSDGVLYWMSRDQRVQDNWALIYAQQLALAEKLPLHICFCLVP
RYLDAAYRQYAFMLKGLQEVATECKSLGIQFHLLSGEPEQNLPSFVKNWNFGAVVTDFNPLRIPLQWIETVKK
HLPSDIPFVQVDAHNVVPCWEASEKLEYGARTIRGKITKLLPEFLSEIPLVDTHPHSASKTAKPVNWEKLLSS
LEVDRSVGEVEWAQPGTSAGMAMLESFIDQRLRFFATLRNNPNSDALSHLSPWLHTGQLSAQRVVRQVKREKN
ASESVASFTEELVVRRELADNFCFYNHNYDDISGAYDWAKKTLQDHAKDTRQYLYTQEQLENAKTHDQLWNAA
QRQLLSEGKMHGFLRMYWAKKILEWTASPEEALSIAIYLNDRLSLDGCDPNGYVGCMWSVCGIHDQGWAERPI
FGKIRYMNYAGCKRKFDVAQFERKYAAIKEISKKDTKKSL
>Anabarilius_grah__ROK23383.1
MSASRTVICLLRNDLRLHDNEVFHWAQRNAEHIIPLYCFDPRHYQGTHHFNFPKTGPFRLHFLLDSVKDLRAT
LKKRGSTLVVRQGKPEEIVGDLIKQLDSVTAVAFHEEVASEEKTVEKKLKDICCQNKVKVQTFWGSTLYHRDD
LPFGHIGRLPDVYTQFRKVVEGQDRVRPVLSTPEQVKSLPLGLEEGPIPTFEGLGQTESVMDCRSAFPCRGGE

TEALARLKHYFWDTNAVATYKETRNGMIGVDYSTKFSPWLAFGCISPRYIYEQIKKYEAERTANQSTYWVIFE
LLWRDYFKFVGLKYGNRIFHINEGRTGVPFVDANMRELALTGFMSNRGRQNVASFLTKDLGLDWRLGAEWFEY
LLVDHDVCSNYGNWLYSAGIGNDPRENRKFNMIKQGLDYDNNGDYVRQWVPELGGIKGGDVHTPWTLSNSALS
HAKVSLNETYPSPIVIAPEWSRHVNNKSAGPSSAKGRKGSSYTPRQRKDRGIDFYFSKKKSF
>Anabarilius_grah__ROL43371.1
MVVNTIHWFRKGLRLHDNPSLRDSIIGADTLRCVYILDPWFAGSSNVGISRWRFLLQCLEDLDASLRKLNSRL
FVIRGQPTDVFPRLFKEWKITRLSYEYDSEPFGKDRDAAIKKLATEAGVEVVVRISHTLYDLDKIIELNGGQS
PLTYKRFQTLISRMDPVEIPAETITAEVMGKCTTPVSEDHDDKFGVPSLEELGFETEGLSSAVWPGGETEALT
RLERHLERKAWVANFERPRMNANSLLASPTGLSPYLRFGCLSCRLFYFKLTDLYRKVKKNSVPPLSLYGQLLW
REFFYTAATNNPHFDKMEFNPICVQIPWDRNPEALAKWAEGQTGFPWIDAIMTQLRQEGWIHHLARHAVACFL
TRGDLWISWEEGMKVFEELLLDADWSVNAGSWMWLSCSSFFQQFFHCYCPVSFGRRTDPNGDYIRRYLPILRG
FPAKFIYDPWNAPDSVQKVAKCIIGVHYPKPMVNHAEASRINIERMKQIYQQLSCYRGLGLLATVPSNTAKNG
DGNGAAPSSADNTQESTHITVQPSPASFQPEFPPFTMSCDQQPVNSVQPHPGYSGGGMVGQVYKQSQRGEFCQ
NGLRGLITDFHIKLF
>Anabarilius_grah__ROL43523.1
MSHRTIHLFRKGLRLHDNPGLLGALASSAALFPVYVLDHAFLEGAMRLGALRWRFILQSLEDLDGRLRTLGSR
LYVLQGSTRAVLSDLVTRWGITQISYDTEVEPYYARMEREIQALAEERRLQTHTCVSHTLYDVERIIQANGGS
PPLTYKKFLHVLSVLGEPEKPARDVSAEDFLRCPTPPDHEQAYGVPSLADLGLEVQSEVLWPGGETHALQRLD
KHFQSQGWVASFSKPRTVPNSLLPSTTALSPYLSLGCLSVRTFYHRLSSIYAQSKNHSLPPVSLQGQVLWREF
FYTVASATPNFTKMEGNPICLQIDWYHDPESLEKWRTAQTGFPWIDAIMTQLHQEGWIHHLARHAVACFLTRG
DLWISWEEGMKVFEEFLLDADYSLNAGNWMWLSASAFFHKYTRIFCPVRFGRRTDPQGQYLRKYLPVLKNFPS
QYIYEPWKAPEDVQLSAGCIIGKDYPCPMVCHMEASQRNLGLMRQVRTEQQKTAELTRDVADDPMEAGLKREL
QEEEELLEGAEPCSKSKRFSGSTDQKSRPCSWTPETLRLSEESVNSEVQSRSETYEGGEEHTEPNPSADGGKE
HPTAPPGMIWRVTGGLVSATKGVVGATVGGVAWLGGKSYEITKSAVTAVPSMGVGLVKGGVSAVAGGVSTVGS
TVANKVPFTGKKKDKSE
>Anabarilius_grah__ROL54170.1
MSHNSIHWFRKGLRLHDNPALIAALKDCRHIYPLFLLDPWYTSNTHIGINRWRFLIEALKDLDSSLKKLNSRL
FVVSGPPTEVLPKLFEKWKITRLTFEVDTEPYSQSRDQEVMKLAKEHGVEVIPKISHTLYNINRIIDENNGKT
PLTYVRFQSVVKAIGPPKKPVPAPTEEDMKGVSTPFSEDHEDEFEIPSLEDLGLDTSSLGPDLFPGGEQEALR
RLDEHMQRTGWVCKFEKPQTSPNALIPSTTVLSPYVRFGCMSARTFWWRLTDVYRGKKHSEPPVSLHGQLMWR
EFFYTTAVGIPNFNKMEGNTNCVQVDWENNPERLAAWREARTGFPFIDAIMTQLRQEGWIHHLARHAVACFLT
RGDLWISWEEGQKVFEELLLDADWSLNAGNWQWLSASAFFRQYFRVYSPIAFGKKTDKHGDYIKKYLPVLKKF
PAEYIYEPWKAPRSIQERAGCIVGKDYPRPIVEHEVVHKQNIQRMKAAYAKRSPEDKPTTKGVKRKATSIVDM
LEKKSKR
>Anabas_test__XP_026217746.1
MVINTIHWFRKGLRLHDNPSLKESLLGADSVRCVYILDPWFAGSSNVGISRWRFLLQSLEDLDSSLRKLNSRL
FVIRGQPTDVFPRLFKEWKITRLSYEYDSEPFGKERDAAIKKLASEAGVEVTVRISHTLYDLDKIIELNGGQS
PLTYKRFQTLISHMDAVEMPAESITAEIMGKCSTPLSEDHDDKFGVPSLEELGFDTEGLSSAVWPGGETEALT
RLERHLERKAWVANFERPRMNANSLLASPTGLSPYLRFGCLSCRLFYFKLTDLYRKVKKNSSPPLSLYGQLLW
REFFYTAATNNPCFDKMESNPICVQIPWDRNPEALAKWAEGRTGFPWIDAIMTQLRQEGWIHHLARHAVACFL
TRGDLWISWEEGMKVFEELLLDADWSVNAGSWMWLSCSSFFQQFFHCYCPVGFGRRTDPNGDYIRRYLPILRG
FPAKYIYDPWNAPESVQKAAKCIIGVHYPKPMVHHAEASCLNIERMKQIYQQLSCYRGLGLLATVPSNSSGNG
NVNSETPSDVMGFPSEATHSAAAASSYVAPSSSMMCYTQGTQQIPAPVVQKGSEHHSTPQTSGKRHSEDSGNG
KGSKVQRQSSH
>Anabas_test__XP_026218962.1
MFCKIVSENVTDAFCSSDPRSRRTSDTVSKRNGLVLIQIQVLQTSRRGRSMTTSRTLICLLRNDLRVHDNELF
HWAQKNAEHIVPLYCFDPRHYMGTHNYNLPKTGPFRLRFLLESIRDLRNTLLSKGSNLVVRRGKPEHVVAELI
KQLGSVSTVAFHKEVTSEELNVEKKLKDVCDQMKVKVHTCWGSTLYHIDDLPFHHISGLPDVYTQFRKEVETQ
SRVRPVFPTPEQLKPLPPGLEEGTIPTADDLQQTEPVTDPRSAFPCSGGESQALARLKHYFWDTDAVANYKET
RNGLIGVDYSTKFASWLALGCISPRYIYQQIKQYEKQRTANQSTYWVIFELLWRDYFRFVSVKYGNRIFQVKG
LQDKSVPWKNDMKLFNAWKEGRTGVPFVDANMRELAMTGFMSNRGRQNVASFLTKDLGLDWRMGAEWFEYLLT
DYDVCSNYGNWLYSAGVGNDPRENRKFNMIKQGLDYDYNGDYIRLWVPELQKIRGADVHTPWTLSTAALSHAE
VSLGENYPTPIVIAPEWSKHINNKPRGTGPSPRGKKGPSHTPKQHRDRGIDFYFSRSKNL
>Anabas_test__XP_026220618.1
MPLSAAGGGDSEVLVRKILREVLLGREDPEGFFAMCLSVLGHQETRSQFLSLIHPLSTANSSLHSTLTSIYQE
FFTQTEDDELELALALSLLEMKDDQLSTSSHKTGFLQFGDKQNESRSSQLTSESQPQGSSPTRRAEVVGTQET
ELQKRPRVKKYEECDQMTSNLAEKSKKNRRQRRKAARQQVVGLQCSLSAPPPVLVWFRRDLRLCDNPALSASL
ELGAPVIPVFIWSPDEEEGPGITLAIRGAGKYWLHQALSHLCASLERIGSHLVFLKASGEENGVGSSLHSLKK
LIKDTGARTVLANALYEPWLKERDDAVVAALQKDGTEVRMFQSYCLRDPYSVSTEGVGLRGIGSVSHFMSCCR
QNPASPLGVTLDPPVSLPTPAHWPQGVSLDTLGLANMPRRKDGTVINWAANIQKSWDFSEEGAQAHLEAFLQD

GVYRYDKESGRADAPNTSCLSPYLHFGQLSPRWVLQAARGARCQPLKFQRKLAWRDLAYWQLTLFPDLPWESV
RPPYKALQWSSNRGHLKAWQRGLTGYPLVDAAMRQLWLTGWMNNYMRHVVASFLIAYLHLPWQEGYRWFQDTL
VDADVAIDAMMWQNGGMCGLDHWNFVMHPIDAAMTCDPYGNYVRKWCPELADLPDELIHKPWKCPSSMLRRAG
VVFGQTYPERIITDLEERRSQSLQDVARVRREFQQYVDKRTGCDLVPLPPSLVTKALGLSHQDSSVVIEGKQF
LLPVITRMEFKYQQENPNADASSNPYNALLKGYVSRKRDETIAFLNERDFTASVMFEAVQRKERLESDHRRIE
GLPHAPAPRGSARRTPTAKDKFSIVPGGALTSLR
>Anabas_test__XP_026221361.1
MAVNSVHWFRKGLRLHDNPALQEALNGAHTVRCVYVLDPWFAGAANVGINRWRFLLEALEDLDKSLKKLNSRL
FVVRGQPTDVFPRLFKEWKVTRLTFECDPEPYGKERDGAIIKLAQEFGVETIVRNSHTLYNLDRIIEMNNNNP
PLTFKRFQTIVSRLELPRRPLLPVTQQQMDKCCTKIADNHDQLYSIPSLEELGFRTEGLPPAVWRGGESEALE
RLNKHLDKKVWVANFEHSRVNTCSLYASPTGLSPYLRFGCLSCRVLYYNLRELYMKLRKRCSPPLSLFGQLLW
REFFYTAATNNPNFDRMEGNPICVQIPWDQNPEALAKWAEGRTGFPWIDAIMTQLRQEGWIHHLARHAVACFL
TRGDLWISWESGMRVFEELLLDADWSVNAGSWMWLSCSAFFQQFFHCYCPVGFGRRTDPSGDYIRRYIPILKD
YPNRYIYEPWNAPDSVQKAANCVVGVDYPKPMINHAEGSRLNIERMKQVYQQLSHYRGLSLLASVPTIQEEAE
PPMTDESQASSGPDSPHRGPDDSEAAGSSFAPDSSTVCAPSSSAPNPDSQEANIHILQTACTSSGSNSQPTAA
TTPTSASHLASTAAPLTLAQSPVSRSKPSSPSSSCPTLSPSPSPAATPTQTSCPGQRRKGLIRKVRRSQRQRG
RQSCGPPARDGGGRAEEEGEVEEAEGEERMEEDVEQDEDRMEEETCGETAGLQQ
>Anabas_test__XP_026231066.1
MLFCINRSSSFVSAPGRFLKLVVHSDQWSSPLLSFIFPSGSIMPAKKRRATSAAAAAAADEEPSAKQQKLAST
KEDRAEDRAEDRAEDRAEDRAEDRAEDRAEGLFNRKRLRFISDTEKIKQGSEGVLYWMSRDQRVQDNWALIHA
QQLALEEKLPLHVCFCLFVPKSELSTLRHYSFMLKGLEEVAQECKKLNIQFHLLRGSAGEVLPGFVSDHSLGA
VVTDFSPLREPLQWLEDVKKKLSKDIPFIQVDAHNIVPCWVASPKLEYGARTIRGKINNLLPEFLSDFPLIEK
HPYTAIRTAKHVDWDKTLASLQVDRSVGETEWAKPGTKAGMAMLQSFIDARLKLFGTHRNDPNAAALSQLSPW
IRFGHLSAQRAALQVKRHGKSTSQSIASFLEELVVRRELTDNFCFYNKKYDSVEGAYEWAQKTLKDHAKDKRS
YLYTREQLENAKTHDKLWNAAQYQMIIEGKMHGFLRMYWAKKILEWTSSPEEALSIALYLNDRHELDGQDPNG
FVGCMWSICGIHDQGWAERPVFGKIRYMNYKGCTRKFDVAKFEKKYCPKNL
>Anabas_test__XP_026234836.1
MAHTCLHWFRKGLRLHDNPALMAALRDCEELYPVFILDPHLHNKTCVGLNRWRFLIGALTDLDCSLRKLNSRL
FVVRGKPDEVFPKLFNKWKITKLTYEYDTEPYSLSRDTEVTALAKEHGVEVIYKISHTLYDINRIIEENNGAA
PLTYKRLQAIANSLGPPKRPIPPPTLEDMEGVKTPRLEKHEKKYGIPSLEELGHDTTALTEELFPGGEQEALR
RLDEHMKRTGWVCSFEKPQTSPNSLSPSTTVLSPYVTFGCLSARTFWWRLTDVYQGKKHSHPPVSLHGQLLWR
EFFYTASVGIPNFNKMEGNPACTQVDWDTNAEYLAAWREARTGFPFIDAIMTQLRREGWIHHLARHAVACFLT
RGDLWISWEEGQKVFEDLLLDGDWALNAGNWQWLSASTFFHQFFRVYSPVAFGKKTDKNGAYIKKYLPLLKKF
PAEYIYEPWKAPRSIQQAAGCIVGKDYPHPIVQHEVVSKKNIQRMKLAYARRSSDPTESPSKKQGVKRKAPSV
VDMLMKKKRKI
>Ananas_com_PHR2_OAY69588.1
MEQTKPLLPPLTSPQTLETLETLESQSPSQEEEHVKPLPIASLSLSLSSLLSLASKPSPPTLSSFKTRIKIPS
QITSLAHLSSSSPKPPRRSSSAAAAGAASVLRAPSQLLRRPPADPRAGAAAGRRCGLVWFRADLRVHDNEALA
AASDASLSLLPVYLFDPRDYGRSSSGFDRTGPYRAAFLRDSVADLRRRLRARGSDLVVRIGRPEVVLPELARA
SGADAVYAHREVSHDEVRAEERVAKAMEKEGVEVKYFWGSTLYHVDDLPFDLDRMPSTYGSFREKVQGVAVRR
TIEALDQIKGLPKRGGVEPGEIPTLAELGLNPAPAMSQDGKPAAVTSSLVGGETEALERLKKFAAECSMQPKK
GDKDSTRDSIYGANFSCKISPWLATGCLSPRFMYEELKKTATKAISAATNQKNGAAGSDNGMNWLMFELLWRD
FFRFITKKYSTGKNAEVEPATACTGALA
>Ananas_com_XP_020089413.1_(6-4)DNA_p
MLFQIPAVRYPSTPSPPSSSSSCSSPSPRNPPRRSPRSRMESNAMVWFRKGLRIHDNPALDHARRGGCARLFP
VFVLDPRYLEPDPTAFSPGSARSGLNRIRFLLDSLADLDSALRSLGSRLLLLRGEPAPLICRVLKDWNIGKLC
FEFDTEPYSLARDKQVSDFASASGIQVFSPVSHTLFNPADIIEKNGGRAPLTYQSFVALAGKPPEPLSVTYHE
LPPVGDTGEYQLLSVPKIEELGYGDISQEEFSPFRGGESEALRRLKESLKDKEWVAKFEKPKGDPSAFLKPAT
TLLSPYLKFGCLSSRYFYQCLHDIYKSVGKHTSPPVSLAGQLLWRDFFYTVAFGTPNFDCMRGNKICKQVPWR
DDEELLVAWREGQTGYPWIDAIMIQLKKWGWVHHLARHSVACFLTRGDLFIHWEKGRDVFERLLIDSDWAINN
GNWLWLSCSSFFYQYYRIYSPISFGKKYDPNGNYIRHFLPVLKDMPNEYIYEPWTAPLSVQKKARCIIGKDYP
KPVVDHDIASKECRKKMAEAYASNHSGENVPSGRSSNVLRRKLQNENREGSPPTNSKQRKKHSLL
>Ananas_com_XP_020094540.1_photolyase
MCPAIPASSPVHPNRIRILSPGPPGPPSAGPVVYWMFRDQRLRDNWALLHAAHLAAAASAPLAVAFALFPGPP
FLLGARARHLLFMLRSLRRLLLLSPFPFFLLLHSPPSHSLPALLRRLSASALVADFSPLRPFRSALDHIVRSV
GADVAVHQVDAHNVVPVWAASDHLEYSARTLRSKIHRLLPEYLVEFPELPAPAIPWTGEKPLEIDWDELFTDI
NREVGDAPEITWCEPGEDAAMETLLGTNDGFLTKRIRNYDTDRNDPTKPRGLSGLSPYLHFGQISAQRCALEA
RARRSSNPKAVDAFLEELIVRRELADNFCYYQPKYDSLDGAWEWARKTLMEHAVDKREHIYTREQLEKAQTAD
PLWNASQLEMVYHGKMHGFMRMYWAKKILEWTNKPEEALSIAIYLNDKYEIDGRDPNGYVGCMWSICGVHDQG
WKERPVFGKIRYMNYAGCKRKFDVDAYISYVNRLVGETKKRKPENPLSLLKKKHSKSER

>Ananas_com_XP_020095834.1_cry-1-like
MSTSAKTIVWFRRDLRIEDNPALAAASKDACVLPVFVWCPSEEGQFYPGRVSRWWLKQSLGHLDQSLKLLGCP
LALIRAESTLAALLQCIGAIGATRVVYNHLYDPVSLVRDHKIKNQLAALGVSVQSFNADLLYEPWEVSDENGH
AFTTFDAFWEKCMNLPIDLASPLPPWKLVPAAGIENIQSCSIEDLGLENEPEKSSNALLSRGWSPGWNNADKV
LAEFVEGSLLEYSRNRMKVEGTTTSLLSPYLHFGEISVRKIYQTVRMKQINWAKEGNCEAEESVSLFLRSIGL
REYSRYLCFNFPFTHERSLLGNLRHYPWRADENQFKSWRQGRTGYPLVDAGMRELWATGWVHNRIRVVVASFF
VKFLLLPWTWGMKYFWDTLLDADLESDILGWQYISGSLPDGHELNRIDSPEVQGQKFDPDGKYVRNWIPELAR
MPTEWIHHPWDAPKPVLKAAGVELGLNYPNPIIEIDTARERLDDAVSMMWELDRAAKIAALNDSNEVVADNLV
NINSLDIPKVVVKKEVSCSMSSLDQRVPSVHNLKVSACNYEELKSLSGRGADKQVLDDQAPTSNVDEDLRSTA
ESSSARKRSISSSTCAVPTASFSTSEANPLKESSGRASMGSSVKEEDDEDAQSSIKGLRPSKRCG
>Ananas_com_XP_020109087.1_cry_DASH,_
MLHLLSPPHSTPLKSLIFSPVPTPIRFLFRSNLTMSLSSSSIPLSNDRLRPVRCYMVPGLSSEEAEMASDEAF
RRYSSPSLKRTGKGVAIVWFRNDLRVLDNEALVRAWATSESVLPVYCVDPRNFGTTHYFGFPKTGALRAQFLI
ECLADLKKNLIKKGLNLLVKHGKPEDILPSVVKAFGVHTVYAHKETCSEELLVEHRVLKRLQQVVLPQSPDQE
PINPKLQLIWGTTMYHIDDLPFSIRDLPDVYTQFRKAVESKCLTRSCCKLPLSFGPPPSASLDEIGGWGDIPS
LEQLGLEEKKCDKGMSFVGGENAAIGRVTEYFWKKDKLRVYKETRNGMLGPDYSTKFSSWLASGSLSPRYIYE
EVKRYEKQRISNDSTYWVLFELIWRDYFRFLSIKYGNSIFHLGGPRKIVSNWSQDRALFEAWRDGRTGYPIID
ANMKELSTTGFMSNRGRQIVCSFLVRDMGIDWRMGAEWFETCLLDYDPASNYGNWTYGAGVGNDPREDRYFSI
PKQAKTYDPEGEYVAYWLPELRSLPKERRNFPGNSYTKQIVPLKFDTTYQKKDYSKRERADKMYVR
>Anas_plat__EOB07201.1
RFLLQCLEDLDANLRKLNSRLFVIRGQPADVFPRLFKEWNIAKLSIEYDSEPFGKERDAAIKKLASEAGVEVI
VRISHTLYDLDKIIELNGGQPPLTYKRFQTLISRMEPLEMPVETITPEVMEKCTTPVSDDHDEKYGVPSLEEL
GFDTDGLPSAVWPGGETEALTRLERHLERKAWVANFERPRMNANSLLASPTGLSPYLRFGCLSCRLFYFKLTD
LYKKVKKNSSPPLSLYGQLLWREFFYTAATNNPRFDKMEGNPICVQIPWDKNPEALAKWAEGRTGFPWIDAIM
TQLRQEGWIHHLARHAVACFLTRGDLWISWEEGMKVFEELLLDADWSVNAGSWMWLSCSSFFQQFFHCYCPVG
FGRRTDPNGDYIRRYLPVLRGFPAKYIYDPWNAPESIQKAAKCIIGVNYPKPMVNHAEASRLNIERMKQIYQQ
LSRYRGLGLLATVPSNPNGNGSGGLMGFSPGESVSGCGSTGGAQLGTGDSQTVGVQTCALGDSHAGTSGIQQQ
GYCQASSILHYAHGDNQQSHLLQAGRTSLGTGISAGKRPNPEEETQSVGPKVQRQSTN
>Anas_plat__XP_027300846.1
MPHRTIHLFRKGLRLHDHPALLAALESSEVVYPVYILDRKFMTSVMHIGALRWHFLLQSLEDLQKNLCRLGSH
LLVIQGEYEPVLRDHVQKWNITQVTLDAEMEPFYKEMEANIRCLGEELGFEVLSVVGHSLYDTKRILDLNDGT
PPLTYKRFLHILSLLGDPEVPVRNLTAEDFQRCRPPDLDLAECYRVPLPADLKIPPESISPWRGGETEGLQRL
EQHLADQGWVASFTKPRTIPNSLLPSTTGLSPYFSMGCVSVRTFFYRLSNIYAQAKHHSLPPVSLQGQLLWRE
FFYTVASATPNFTKMAGNPICLQISWYEDAERLHKWKTAQTGFPWIDAIMTQLRQEGWIHHLARHAAACFLTR
GDLWISWEEGMKVFEELLLDADYSINAGNWMWLSASAFFHQYTRIFCPVRFGKRTDPEGQYIRKYLPVLKNFP
SKYIYEPWTASEEEQKQAGCIIGKDYPFPMVDHKEASNHNLQLMKHVREEQYRTAQLTRDDTDDPMEMKLKRD
HSEENVTKAKTARMTEQT
>Anas_plat__XP_027306164.2
MAGLQTESLQAIFERYITGAGLKGGPWRGLAVCLLRCDLRAHDNQVLHWAASNADYLIPLYCFDPRHYAGTHC
YGFPKTGPHRLRFLLESVKDLRETLKKKGSTLVVRKGKPEDVVRDLITQLGCVGVVAFHEEATQEELDVEKEL
CQVCEQHGVEVKPFWAATLYHRDDLPFGPIARLPDVYTQFRKAVEAEAKVRPALQLPDQLKPLAPGVEEGCIP
TMEDLGQKDPGTDPRTAFPCRGGETQALARLQYYFWDTNLVASYKETRNGLIGMDYSTKFAPWLALGCISPRY
IYEQLRKYEKERGANDSTYWVLFELLWRDYFRFVALKHGRRIFSVRGLQSKEVPWKKDLQLFDCWKEGKTGVP
FVDANMRELAATGFMSNRGRQNVASFLTKDLGLDWRMGAEWFEHLLVDYDVCSNYGNWLYSAGVGNDPRDKRK
FNVIKQGLDYDGNGDFVRLWLPELRGIAGADAHAPWALSSAALRQAGVILGQTYPQPLVRAPEWSRHINQRLQ
GRSPHPRGRRGPADAPAQRKDRGIDFYFSRKKDE
>Anas_plat__XP_027313887.2
MAAAVAAPSRSVHWFRRGLRLHDNPALQEALRGAASLRCIYILDPWFAASSAVGINRWRFLLQSLEDLDNSLR
KLNSRLFVVRGQPTDVFPRLFKEWGVTRLTFEYDSEPFGKERDAAIVKLAKEAGVEVVIENSHTLYDLDRIIE
LNGNKPPLTYKRFQAIISRMELPKKPVSSVTSQQMETCKVDIQENHDDVYGVPSLEELGFPTEGLAPAVWQGG
ETEALARLDKHLERKAWVANYERPRMNANSLLASPTGLSPYLRFGCLSCRLFYYRLWELYKKVKRNSTPPLSL
YGQLLWREFFYTAATNNPKFDRMEGNPICIQIPWDKNPEALAKWAEGKTGFPWIDAIMTQLRQEGWIHHLARH
AVACFLTRGDLWISWESGVRVFDELLLDADFSVNAGSWMWLSCSAFFQQFFHCYCPVGFGRRTDPSGDYVKRY
LPKLKGFPARYIYEPWNAPESVQKAAKCIIGVDYPKPMVNHAETSRLNIERMKQIYQQLSRYRGLCLLASVPS
CVEDLSGPVADSASGQGSSTSTAVRLPQSDQASPKRKHEGAEELCTEELYKRAKVTGLPASEIPGKSL
>Anas_plat__XP_027319951.2
MSRDQRVQDNWAFLYAQRLALKQELPLRVCFCLVPKFLGATIRHYGFMLRGLQEVSKECTELNIPFHLLLGYA
KDVLPPFVVQQGVGGLVTDFCPLRVPRQWVEDVRERLPEDVPFVQVDAHNIVPCWVTSPKQEYSARTIRGKIH
SQLPEFLTEFPPVVRHPHPPSCPAEPIAWDACYSSLQVDRSVAEVEWATPGTSAGLAVLQSFIAERLKSFSTQ
RNDPNKAALSNLSPWFHFGQVSTQRAILEVQKHRRAHKESVEAFVEEAVVRRELADNFCYYNENYDSLQGAYD

WAQTTLRLHAKDKRPFLYELQQLEQATTHDPLWNAAQLQMVREGKMHGFLRMYWAKKILEWTRSPEEALQFAI
YLNDRYELDGRDPNGYVGCLWSICGIHDQGWKERDVFGKIRYMNYSGCKRKFDVDQFERRYGPRELAQ
>Anguilla_anguilla_XP_035274077.1_cryptochro
MVVNSVHWFRKGLRLHDNPSLQEALNGADTVRCVYILDPWFAGSANVGVNKWRFLLESLEDLDTSLRKLDSRL
FVVRGQPAEVFPRLFKEWNVTRLTFEYDSEPYGKERDAAIIKMAQEFGVETRVQNSHTLYNLDRIIELNNNSP
PLTFKRFQAIVNRLGLPKRPLTTVTKQQLDSCRTSISDNHNERYGVPSLEELGFKTHGLSPAVWKGGETEALE
RLSKHLDRKAWVANFERLRINICSLIASPTGLSPYLRFGCLSCRLFYYDLRELYMKVRKHSTPPLSLFGQLLW
REFFYTAATNNPNFDRMEGNPICVQIPWDHNPEALAKWAEGRTGYPWIDAIMTQLRQEGWIHHLARHAVACFL
TRGDLWLSWESGMRVFDELLLDADYSVNAGSWMWLSCSAFFQQFFHCYCPVGFGRRTDPSGDYIRRYIPQLKD
YPNRYIYEPWKAPETVQKAANCVVGKDYPKPMINHAEGSRLNIERMKQIYQQLSHYKGLSLLASVPSIQEEAE
PPMTDDSKAHSSSTSDDCHV
>Anguilla_ang_XP_035242993.1_cry_circad
MTHRTIHLFRKGLRLHDNPSLVAALESSAAVYPVFVLDRAFLEGATRMGPLRWRFLLQSLRDLRDGLAALGSR
LHVLQGGHEAVLRRLVRRWGVTQLTFDAEVEPFYARLDDGLRALCQELGVLVRSCVAHTLYDVRRIIKANGGE
PPLTYKKFLQVLSVLGEPDKPAREITLQDFQKCKTPVDDGNDEQYRIPSLEDLGIEVDLEVLWPGGESEGLKR
LEKHFESQGWVVNFSKPRTIPNSLLPSTTGLSPYFSLGCLSVRSFYYKLSNVYAQSKNHSLPPVSLQGQLLWR
EFFYTVASATPNFTKMAGNPICLQIGWYEDQAALEKWRTAQTGFPWIDAIMTQLRQEGWIHHLARHAVACFLT
RGDLWISWEEGMKVFEEFLLDADYCVNAGNWMWLSASAFFHQYTRIFCPVRFGRRTDTDGQYLRKYLPVLKNF
PSKYIYEPWTAPLEVQQEAGCIIGKDYPQPMVNHQEVSQRNMALMRQLRSEQEGTAQLTRDVTDDPMESGVKR
DRQDNGPSEDASEKLERANKRFSPDGDCKAQSVSWTSDPIRLPELSEGVM
>Anguilla_ang_XP_035243646.1_cry-1-like
MAPNSIHWFRKGLRLHDNPALREAVRGAGTVRCVYFLDPWFAGSSNLGVNRWRFLLQCLEDLDASLRKLNSRL
FVIRGQPANVFPRLFKEWKISRLTFEYDSEPFGKERDAAIRKLAMEAGVEVIVKTSHTLYDLDKIIELNGAQP
PLTYKRFQTLVSRMDPPEMPVEPLSEAFMGRCTTPVGDDHGDKYGVPSLEELGFDTEGLASAVWPGGETEALT
RIERHLERKAWVANFERPRMNANSLLASPTGLSPYLRFGCLSCRLFYFKLTDLYRKVKKNSSPPLSLYGQLLW
REFFYTAATNNPRFDKMEGNPICVRIPWDWNAEALAKWAEAKTGFPWIDAIMTQLRQEGWIHHLARHTVACFL
TRGDLWISWEEGMKVFEELLLDADWSVNAGSWMWLSCSSFFQQFFHCYCPVGFGRRTDPNGDFIRRYLPVLRG
FPAKYIYDPWNAPHSVQTAAKCIIGIHYPKPMVNHAEASRLNIERMKQIYQQLSRYRGLGLLASVPSNHNGNG
NGGGSGGGGNGAMMAYSPGEQPQGNSAAHGYHTVSDPGQAIASHRLYQASSSHEFAVPQHPGRLSHVTGKRER
EAEREAGGDSDTPSSALKVQRPNSEKRELQDVNLAPY
>Anguilla_ang_XP_035278411.1_photolyase
MRNYECEIMDQFCAGLNISCRQYQRQTSNPDRRSLLYPVCGGLPQRVSPARPNRAPRSLCSLIQLRVSFSCSS
MRAGKKAETGAAVAEPENSEGRKVTGKRRGEPVEGDGGKRAKAMPQKEGWLAEKIAEERRSARCVKFNERRVR
FLSQAQKIKEGVEGVLYWMYRDQRVQDNWALIYAQNLALEAQLPLHVCFFLELEGPGATLREYSFLLKGLEEV
AEECGALGVQFHLLRGSAGVMLPGFVKEWGLGAVVTDFSPLRTPLQWAEDVKEGLPPDVPFVQVDAHNVVPCW
VASDKQEYAARTIRGKITKLLPEFLTQFPLVDKHPHCAARLAKPVDWAQTLSSLELDHSVGEVEWARPGTAAG
TAMLESFIDERLRHYAPLRNNPNSEALSQLSPWVHFGHLSAQRVVLQVQRSGASRAQSVPAFVEEMVVRRELA
DNFCYYNKKYDQVEGAYDWARKTLQDHAGDPRPYVYTREELEKAQTYDNLWNAAQYQMVTEGKMHGFMRMYWA
KKILEWTSSPEEALSIAIYLNDRYELDGRDPNGYVGCMWSICGIHDQGWKERPIFGKVRYMNYAGCTRKFDVA
QFEKQYCPKRL
>Anguilla_japo__BCT36447.1
MAPNSIHWFRKGLRLHDNPALREAVRGAGTVRCVYFLDPWFAGSSNLGVNRWRFLLQCLEDLDASLRKLNSRL
FVVRGQPANVFPRLFKEWKISRLTFEYDSEPFGKERDAAIKKLAMEAGVEVCVKTSHTLYDLDRVIELNGGQP
PLTYKRFQTLISRMDPPEPPVDPLCASFMGRCVTPVADDHGDKYGVPSLEELGFDTEGLPSAVWPGGETEALT
RIERHLERKAWVANFERPRMNANSLLASPTGLSPYLRFGCLSCRLFYLKLTDLYKKVKRNSSPPLSLYGQLLW
REFFYTAATNNPRFDKMEGNPICVQIPWDRNPEALAKWAEGCSGFPWIDAIMTQLRQEGWIHHLARHAVACFL
TRGDLWISWEEGMKVFEELLLDADWSVNAGSWMWLSCSSFFQQFFHCYCPVGFGRRTDPNGDYIRRYLPVLRG
FPAKYIYDPWNAPESVQKAAKCVIGVQYPRPMVQHAEASRLNIERMRQIYQQLSCYRGLGLLATVPSNPGGNE
GAAAMGACAAEGPHEGATAAGYQLSVTPQIDWSTGVHPYPPKESQSSSSTQQQGFTGGGSSLVCYRQESQQTL
TAPQGLSSILSTVKRHSEDAPPSVSSKVQRQNSN
>Anguilla_japo__BCT36448.1
MVVNSIHWFRKGLRLHDNPALQEALNGADTVRCVYILDPWFAGSANVGVNRWRFLLESLEDLDTSLRKLNSRL
FVIRGQPADVFPRLFKEWNITRLTFEYDSEPYGKERDGAIIKMAQEFGVETRVKNSHTLYNLDRIIELNNNSP
PLTFKRFQALVSRLELPKKPLPPVTRQQMDGCPAHIADNHDERYGVPSLEELGFKTHGLSPAVWKGGETEAMD
RLNKHLDRKAWVANFERPRIGASSLMASPTGLSPYLRFGCLSCRVFYYKLRELYQQVRRHGSPPLSLFGQLLW
REFFYAAATDNPNFDRMAGNPICVQ
>Anhinga_anhi__NXC73818.1
KVDYDVCSNYGNWLYSAGIGNDPRDNRKFNMIKQGLDYDGDGDYVRLWVPELQGIKGADIHTPWALNSAALSQ
AGVTLGETYPQPVVTAPEWSRHVTQRPQGRSPHPRRGPARTPVQRKGRGIDFYFSRKKDV
>Anhinga_anhi__NXC74850.1

RFLLQCLEDLDANLRKLNSRLFVIRGQPADVFPRLFKEWNIAKLSIEYDSEPFGKERDAAIKKLASEAGVEVI
VRISHTLYDLDKIIELNGGQPPLTYKRFQTLISRMEPLEMPVETITPEVMEKCNTPVSDDHDEKYGVPSLEEL
GFDTDGLPSAVWPGGETEALTRLERHLERKAWVANFERPRMNANSLLASPTGLSPYLRFGCLSCRLFYFKLTD
LYKKVKKNSSPPLSLYGQLLWREFFYTAATNNPRFDKMEGNPICVQIPWDKNPEALAKWAEGRTGFPWIDAIM
TQLRQEGWIHHLARHAVACFLTRGDLWISWEEGMKVFEELLLDADWSVNAGSWMWLSCSSFFQQFFHCYCPVG
FGRRTDPNGDYIRRYLPVLRGFPAKYIYDPWNAPESVQKAAKCIIGVNYPKPMVNHAEASRLNIERMKQIYQQ
LSRYRGLGLLATVPSNPNGNGNGGLMGYSPGESISGCGSTGGAQLGTGDGHTVVQPCALGDSHTGASGIQQQG
YCQASSILHYAHGDNQQSQLLQAGRTALDTGISAGKRPNPEEETQSVGPKVQRQSTN
>Anhinga_anhi__NXC75889.1
RFLLQSLEDLDNSLRKLNSRLFVVRGQPTDVFPRLFKEWGVTRLTFEYDSEPFGKERDAAIIKLAKEACVDVV
IENSHTLYDLDRIIELNGHKPPLTYKRFQAIISRMELPKKPVSTVMSQQMETCKVDIQENHDDVYGVPSLEEL
GFPTDGLAPAVWQGGETEALARLDKHLERKAWVANYERPRMNANSLLASPTGLSPYLRFGCLSCRLFYYRLWE
LYKKVKRNSTPPLSLYGQLLWREFFYTAATNNPKFDRMEGNPICIQIPWDRNPEALAKWAEGKTGFPWIDAIM
TQLRQEGWIHHLARHAVACFLTRGDLWISWESGVRVFDELLLDADFSVNAGSWMWLSCSAFFQQFFHCYCPVG
FGRRTDPSGDYVKRYLPKLKGFPSRYIYEPWNAPESVQKAAKCIIGVDYPKPMVNHAETSRLNIERMKQIYQQ
LSRYRGLCLLASVPSCVEDLSGPVADSASGQGCSTSTAMRLSQADQASPKRKHEAAEELCAEELYKRAKVTCL
PAPEIPGKSL
>Anhinga_anhi__NXC77043.1
DNWAFLYAQRLALKQELPLHVCFCLVPKFLDATIRHYGFMLRGLQEVAEECAELNIPFHLLLGYAKDVLPTFV
VEHGVGGLVTDFCPLRLPRQWAEDVRQRLPEDVPFAEVDAHNIVPCWIASPKQEYSARTIRGKIHAQLPEFLT
EFPPVVRHPYPPSCPAEPIAWEACYSSLQVDRTVKEVEWATPGTAAGLAVLQSFIAERLKSFGSHRNDPNKAA
LSNLSPWFHFGQVSTQRAILEVQKHRGKYKESVDAFVEEAVVRRELAENFCYYNENYDSMQGGAYNWAQTTLK
LHAKDKRPFLYKLQELEQGTTHDPLWNAAQLQMVREGKMHGFLRMYWAKKILEWTRSPEEALQFAIYLNDRYE
LDGTDPNGYGRADALSPCPAGCLWSICGIHDQGWAERAIFGKIRYMNYAGCKRKFDVGQFERRYAPPT
>Anhinga_anhi__NXC78071.1
MPHRTIHLFRKGLRLHDNPTLLAALESSEVVYPIYILDRKFVTSVMQIGALRWHFLLQSLEDLQKNLCQLGSC
LLVIQGEYESVLRDHIQKWNITQVTLDAEMEPFYKEMEANIRRLGEELGFEVLSLVGHSLYDTKRILDLNGGT
PPLTYKRFLHILSLLGDPEVPVRNLTAEDFQRCRPSDPGLAECYRVPLPMDLKIPPESPSPWRGGETEGLQRL
EQHLTDQGWVASFTKPRTIPNSLLPSTTGLSPYFSMGCLSVRTFFYRLSNIYAQAKHHSLPPVSLQGQLLWRE
FFYTVASATPNFTKMAGNPICLQIGWYEDAERLHKWKTAQTGFPWIDAIMTQLRQEGWIHHLARHAVACFLTR
GDLWISWEEGMKVFEELLLDADYSINAGNWMWLSASAFFHQYTRIFCPVRFGKRTDPEGQYIRKYLPILKNFA
SKYIYEPWTASEEEQKQAGCIIGQDYPFPMVNHKEASDHNLQLMKQVREEQYRTAQLTRDDTDDPMEMKVKRD
RSEENVSKPKVARMTE
>Anhinga_anhi__NXC78535.1
MQHSSIHWFRKGLRLHDNPALLAAAADCRCLHPLFILDPSSSRAGAIARRFLLDALRDLDGNLRKMGSRLFVV
RGCPEEVFPRLFHAWGTTRLTFEVDTEPPARQRDAAVAELAARHGVEVIQEVSHTLYDTERVLALNDGKAPLT
YKRLQTLLASLGPPEKPAPALTREHLRDGGTRPARRAHGEDGQCSAPITLISGLLCIPDGGGHPKTRPWCASC
PIQAWVCGFKKPETEPNSLSPSTTVLSPYLKFGCLSVRTFWWRLDEIYQGREHSRPPVSLHGQLLWREFFYTA
GASVPNFDRMVGNPVCLQVDWDDNPQHLRAWKEGRTGYPFIDAIMTQLRTEGWIHHLARHAVACFLTRGDLWV
SWEEGLKVFEELLLDADWSLNAGNWLWLSGSAFFHRYFHVYSPVAFGKKTDRDGAYIRKYLPILKDFPAKYIY
EPWKAPRAMQERAGCLVGTHYPQPIVKHEAASKRNLGRMKAARAQK
>Anisakis_sim_VDK18609.1_unnamed_pr
MNELKVKLLCVKEKNYTETLKEVHKLTDNACEVVIDAAYLRNDRDFEDSLNDQLIKSCRRLTRVEANVSVPVN
VSSNQQEFGARTLRPKVWQYVKAMMKEKWEDVPTEPCKDWKKLVKADDLKEMDLEAELKKAESECKSDSGLTG
GEKAAKKMLDFFITNRLVNYHPGRNVPGSKYQSVLSPYLHFGMLSTIETIKRAKEGKAAKHPIDSFIEELLVR
RDLAHNFVYYARKNYDSLDSLPDWAKKTLKEHKNDKREYVYSYEELENAKTHDVYWNAAQLEMIHTNKMHGYM
RMYWGKKVIEWTPDYETAYKFLIEQNDKYELDGRDPNGYTGVMWNFGLHDRAHAVIHVFLALICTF
>Anneissia_jap_XP_033101802.1_cry-1-like
MPNRKRKRSDSPTFSRRCSVHWFRKGLRLHDNPSFLEGLKGAAEFRCVYILDPWFAGSYDNTGINRWRFLLQC
LEDLDSNLKKLNSRLFVIRGQPSDVFPKIFKEWKVNRLSFEVDSEPFARKRDQAIRTMATEANVEVISCISHT
LYDPLEILTLNDNEPPLTYKRFQTLISGMDPPHPCPAPTTEQLEGIERGIELDHYEKYGVPTLEELGFDTDN
IPPVCWQGGETEAKKRLDRHLERKAWIANFESPKMTPVSLMASQTGLTPYLRFGCLSSRLFYWKLSELFMMVR
KTKTVPLSLQGQLLWREFFYTVACNNPNFDCMMGNQICVKIPWDKNKQALDKWARAKTGFPWIDAIMTQLRQE
GWIHSIARHAVGCFLTRGDLWVSWEEGMKVFEELLLDADWSINAGNWIWLSCSSFFQQFFHVYCPVTFGKRTD
PSGDYVRKYLPVLKHFPTKYIYAPWEAPTEVQIQAKCIVGKDYPFPMVDHEEASRLNIERMKQVYSQLSHYAG
PSLLAAIPSSKDSNDGKPPRMGLKSIIQHTSMHSQMTTVGTQRSSNHLHSSGMISSVPSRTSEHRRQHGALQS
ISSTNVQPQEHKIAYANPSTSCHQLQTYDNPSTSGHQLQTMQNVTDQSGLTKYTRTFPLVLEFS
>Anneissia_jap_XP_033101937.1_cry-1-like
MLSAEILKVGSGDVGKKYPGGETAALELLHLRVQLEEKTFAKNEFMPNQTVPDLMGKPMSMSPYLRFGCISVH
RMFWMIRDTYCKVNSAEVAPTYLTSQLIWREFFYTMSATNPMYNRMKGNPICLQIDWYDDDESYQKWVQGKTG

FPWIDAIMIQLHKEGWIHQVCRHAVSSFLTRGDLWISWEKGFDTFEKHLLDADWSVCAGNWMWLSSSAFEKFL
QCPNCFCPVRYGRRMDPSGEYIKSFLPVLKNMPLKYLFEPWKAPLKVQEEAKCIVGKDYPKPMVKHSDVSKRN
KKHMNDVMQVFKDIVVNHCAPSSEQEVYDLTWLPEIDLERPTKCTADEFCTKVH
>Anneissia_jap_XP_033103659.1_photolyase
MTPKTMPKRPAKTQQDVSPKKSKLKVEAANSSEPQDFLSNVAHLRLQTAESVADFKFNKKRVRVISDAKDVPE
QNHGIVYWMSRDQRVQDNWAMLFAQRLALKQEVPLHVCFCVVPKFLDATIRQYGFMMKGLREVEQELKELDIS
FHLLIGYAKDVLPKFIEEHQIGGLVTDFSPLRILSEWVQEVASELPSNVPFCQVDAHNIVPCWEASPKLEYGA
RTIRPKIHKVLSTYLTQYPPVIKHKHKPKTEPEPNVWDAAMDSLEVDKTVSEIDWAQPGTTAGLFALEDFFEN
RLRSYEVERNDPNKTALSNLSPWFHFGQISVQRAILTVQPYRRKHSKAAEAFIEESVIRRELADNFCFYNDKY
DSVEGTNDWAKTSLQLHAKDKREYVYSKEQLEQSRTHDDLWNAAQIQMVSEGKMHGFLRMYWAKKILEWTESP
EKALEIAIFFNDKYSIDGTDPNGYVGCMWSVCGIHDQGWKERPVFGKIRYMNYAGCKRKFDVDQFVRKYKAKK
Y
>Anolis_carolinensis_XP_003214689.1_PREDICTED:
MAALPGPLGRCSVHWFRRGLRLHDNPALQAAIRDGGPVRCIYILDPWFAASSSVGINRWRFLLQSLEDLDNSL
RKLGSRLFVVRGQPTDVFPRLFKEWRVTRLTFEYDSEPFGKERDAAIVKLAKEAGVEVITENSHTLYDLDRII
ELNGHKPPLTYKRFQTIISRMDLPKKPVASITHQQMEMCKTEIQDNHDETYGVPSLEELGFPTESLAPAVWLG
GETEALTRLDKHLERKAWVANYERPRMNANSLLASPTGLSPYLRFGCLSCRLFYYRLWELYKKVKRNSTPPLS
LYGQLLWREFFYTAATNNPKFDRMEGNPICIQIPWDRNPEALAKWAEGKTGFPWIDAIMTQLRQEGWIHHLAR
HAVACFLTRGDLWISWESGVRVFDELLLDADFSVNSGSWMWLSCSAFFQQFFHCYCPVGFGRRTDPSGDYVRR
YLPKLKGFPSRYIYEPWNAPESVQKAAKCIIGVDYPKPMVNHAETSRLNIERMKQIYQQLSRYRGLCLLASVP
SCMEDLSNPVADTSSSHGNCIGTASRQTHCGQTSPKRKHDVVQEYKEELYKRAKVVASQFAENPRQELVHHEM
GN
>Anolis_caroline_XP_003227011.1
MPQKRKKRKEPASGDCENQVEGDGGTSFGSESGDKKTEGKPEEKKPKKTAAVAKGPPLTEAVKASRLKMAPSI
AEFHFNKKRVRMISENSDLKEGAQGILYWMSRDQRVQDNWAFLYAQRLALKQNLPLHVAFCLVPKFLEATIRQ
FGFMLKGLKEVAEECQELSIPFHLLIGFAKDTLPPFVAKHNIAGVVTDFAPLRVPMQWVQEVQERLPPDVPFV
QVDAHNVVPCWVASEKQEYGARTIRRKIHDRLCEFLTGFPPVIQHPHQAASQAEPIDWDACSASLEVDRSVPE
VEWAKPGSAAGLLVLGEFVQERLKFFSSDRNNPNRAALSNLSPWFHFGQVSVQRAILEVRKHRTRCRESVEAF
IEEALIRRELADNFCFYNPNYDKVEGAYEWAKTTLKLHATDKRPHLYTLKQLEEGKTHDQLWNAAQLQMVHEG
KMHGFLRMYWAKKILEWTNSPEEALKFSIYLNDRYELDGRDPNGYVGCMWSVCGIHDQGWAERAVFGKIRYMN
YAGCTKKFDVGQFERKYNPRKIAY
>Anolis_caroline_XP_008108122.1
MSHRTIHLFRKGLRLHDNPTLLAALKSSEIVFPVYILDRKFMTSAMQLGTIRWHFLLQSLADLQASLKKLNSC
LWIIQGEYEAILREHVKKWNITQVTFDDEIEPFYKEMERTIQNIGKEMGFEVFSMVGHTLYDVKRIVELNRGT
PPLTYKRFLQILTTLGDPEMPVEDLTAENSQKLCPLPEEDLDECYRVPLPEDLGISKEHLSPWRGGETTGLQR
LEEHMKNQGWIAKFTKPRTIPNSLLPSTTGLSPYFSLGCLSVRMFFYRLSKIYAQSKNHSLPPVSLQGQLLWR
EFFYTVASATPNFTKMVGNPICLQIEWYEDAEKLHKWKTAQTGFPWIDAIMTQLRQEGWIHHLARHAVACFLT
RGDLWISWEEGMKVFEELLLDSDYSINAGNWMWLSASAFFHQYSRIFCPVRFGKRTDPEGHYIRKYLPVLKNF
PSKYIYEPWTAPAEAQREAGCIIGKEYPFPMVNHKEVSDNNLQLMKQVREEQYKTAQLTRDDVDDPMEMGLKH
RHSEEVTPKGKHARKAEEL
>Anolis_caroline_XP_008110409.2
MASSLPRTALCLLRNDLRCHDNEVLHWAQSHADRIVPLYCFDPRHYAQTYHYNFPKTGPFRLRFLLESVKDLR
ETLKKKGSNLVVRKGKPEDVVRDLIIQLGSVASVSFHEEATKEELDVEKALIRVCTEHGVEVQTFWGSTLYHR
QDLPFKHISQLPDVYTQFRKAVESQARVRPVLKMEGQMKSLPPEIEEGNIPSLEDFGMTEPLRDPRSAFLCSG
GETQALMRLQHYFWETNLVASYKETRNGLIGLDYSTKFAPWLALGCISPRYIYDQIQKYEKERTANQSTYWVL
FELAWRDYFRFVALKYGRKIFFPRGLQDKDISWKKDPKLFDAWKEGRTGVPFVDANMRELATTGFMSNRGRQN
VASFLTKDLGLDWRMGAEWFESLLVDYDVCSNYGNWLYSAGIGNDPRENRKFNMIKQALDYDGNGDYARLWVP
ELQCIRGGDVHTPWALSNGALSQAGVSLGSSYPVPIIKAPEWGRHINQKPSNQGPKGRKGPSHTPKQHKNRGV
DFYFSHKKDLS
>Anolis_car_XP_003220970.1_cry-1_X1
MGVNAVHWFRKGLRLHDNPALREVIQGADTVRCVYILDPWFAGSSNVGINRWRFLLQCLEDLDANLRKLNSRL
FVIRGQPADVFPRLFKEWNITKLSIEYDSEPFGKERDAAIKKLASEAGVEVIVRISHTLYELDKIIELNGGQP
PLTYKRFQTLISRMEPLEIPVETITAEVMSKCTTPVSDDHDEKYGVPSLEELGFDTDGLPSAVWPGGETEALT
RLERHLERKAWVANFERPRMNANSLLASPTGLSPYLRFGCLSCRLFYFKLTDLYKKVKKNSSPPLSLYGQLLW
REFFYTAATNNPRFDKMEGNPICVQIPWDRNPEALAKWAEGRTGFPWIDAIMTQLRQEGWIHHLARHAVACFL
TRGDLWISWEEGMKVFEELLLDADWSVNAGSWMWLSCSSFFQQFFHCYCPVGFGRRTDPNGDYIRRYLPVLRG
FPAKYIYDPWNAPDGVQKAAKCIIGVNYPKPMVNHAEASRLNIERMKQMYQQLSRYRGLGLLASVPSNGNGNG
NGGLMGYSTGENIPGCTNTNGSQMGMNEGHIGNVQACTMGESHTGTSGIQQQGYSQGSGILLYSHGDNQKTHS
AQKGRISLGTGVCTGKRPSPEVETQSVGPKVQRQSSN
>Anolis_car_XP_003225762.1_cry-1

MAHVSIHWFRKGLRLHDNPALLAAMKNSAEIYPIFILDPWFPKNMQVSINRWRFLIESLKDLDESLKKLNSRL
FVVRGRPAEVFPELFTKWKVTRLAFEVDTEPYARRDAEVVRLAAEHGVQVIQKVSHTLYDTERIIVENSGKAP
LTYTRLQTLVASLGPPKQPVPAPKLEDMKDCCTPVKEDHDLEYGTPSYEELGQDPKTAGPHLYPGGETEALAR
LDLHMKRTSWVCNFKKPETHPNSLTPSTTVLSPYVKFGCLSVRMFWWKLAEVYQGRKHSDPPVSLHGQLLWRE
FFYTAGAGIPNFDRMENNPVCVQVDWDNNQEYLRAWREGQTGYPFIDAIMTQLRTEGWIHHLARHAVACFLTR
GDLWISWEEGQKVFEELLLDADWSLNAANWQWLSASAFFHQFFRVYSPVTFGKKTDKNGEYIKKYLPFLRKFS
NDYIYEPWKAPRSLQERAGCIIGQDYPKPIVEHEKVYKRNLERMKAAYARRSPNLVIQAKDKVSQKKGVNRKR
PEAPTKAKVQAKKVKTKSS
>Anseranas_semi__NXI69464.1
CIYILDPWFAASSAVGINRWRFLLQSLEDLDNSLRKLNSRLFVVRGQPTDVFPRLFKEWGVTRLTFEYDSEPF
GKERDAAIVKLAKEAGVEVVIENSHTLYDLDRIIELNGNKPPLTYKRFQAIISRMELPKKPVSSVTSQQMETC
KVDIQENHDDVYGVPSLEELGFPTDGLAPAVWQGGETEALARLDKHLERKAWVANYERPRMNANSLLASPTGL
SPYLRFGCLSCRLFYYRLWELYKKVKRNSTPPLSLYGQLLWREFFYTAATNNPKFDRMEGNPICIQIPWDKNP
EALAKWAEGKTGFPWIDAIMTQLRQEGWIHHLARHAVACFLTRGDLWISWESGVRVFDELLLDADFSVNAGSW
MWLSCSAFFQQFFHCYCPVGFGRRTDPSGDYVKRYLPKLKGFPSRYIYEPWNAPESVQKAAKCIIGVDYPKPM
VNHAETSRLNIERMKQIYQQLSRYRGLCLLASVPSCVEDLSGPVADSASGQGSSTSTAMRLPQSDQASPKRKH
EGAEELCTEELYKRAKVTGLPAPEIPGKS
>Anseranas_semi__NXI71217.1
DNWAFLYAQRLALKQELPLRVCFCLVPKFLDATIRHYGFMLRGLQEGGKECTELNIPFHLLLGYAKDVLPAFV
VEHGVGGLVTDFCPLRVPRQWVEDVREQLPEDVPFVQVDAHNIVPCWVTSPKQEYSARTIRGKIHSLLPEFLT
EFPPIIRHPHPPSCPAEPIAWDACYSSLQVDRTVTEVEWATPGTAAGLAVLQSFIDKRLKSFGSQRNDPNKAA
LSNLSPWFHFGQVSTQRAILEVQKHRRTYKESVDAFVEEAVVRRELADNFCYYNENYDSVQGAHDWAQTTLKL
HAKDKRPFLYELQQLEQATTHDPLWNAAQLQMVREGKMHGFLRMYWAKKILEWTRSPEEALRFAIYLNDRYEL
DGRDPNGYVGKLREGSRGCLWSICGIHDQGWKERDVFGKIRYMNYAGCKRKFDVDQFERRY
>Anseranas_semi__NXI71662.1
KVDYDVCSNYGNWLYSAGIGNDPRDNRKFNMIKQGLDYDGTGDYIRLWVPELQGIKGADIHTPWALNSATLSQ
AGVTLGETYPQPVVTAPEWSRHISQRPHGRSPHPRGRRGPAHTPMQHMGRGIDFYFSRKKDE
>Anseranas_semi__NXI73833.1
RFLLQCLEDLDANLRKLNSRLFVIRGQPADVFPRLFKEWNIAKLSIEYDSEPFGKERDAAIKKLASEAGVEVI
VRISHTLYDLDKIIELNGGQPPLTYKRFQTLISRMEPLEMPVETITPEVMEKCTTPVSDDHDEKYGVPSLEEL
GFDTDGLPSAVWPGGETEALTRLERHLERKAWVANFERPRMNANSLLASPTGLSPYLRFGCLSCRLFYFKLTD
LYKKVKKNSSPPLSLYGQLLWREFFYTAATNNPRFDKMEGNPICVQIPWDKNPEALAKWAEGRTGFPWIDAIM
TQLRQEGWIHHLARHAVACFLTRGDLWISWEEGMKVFEELLLDADWSVNAGSWMWLSCSSFFQQFFHCYCPVG
FGRRTDPNGDYIRRYLPVLRGFPAKYIYDPWNAPESVQKAAKCIIGVNYPKPMVNHAEASRLNIERMKQIYQQ
LSRYRGLGLLATVPSNPNGNGNGGLMGFSPGESISGCGSTGGAQLGTGDGQTVGVQTCALGDSHTGASGIQQQ
GYCQASSILHYAHGDNQQSHLLQAGRTSLGTGISAGKRPNPEEETQSVGPKVQRQSTN
>Anseranas_semi__NXI73871.1
MPHRTIHLFRKGLRLHDNPTLLAALESSEVVYPVYILDRKFMTSVMHIGALRWHFLLQSLEDLQKNLCQLGSC
LLVIQGEYESIVRDHVQKWNITQVTLDAEMEPFYREMEANIRCLGEELGFEVLSLVGHSLYDTKRILDLNDGT
PPLTYKRFLHILSLLGDPEVPVRNLTGEDFQRCRPPDLGLAECYRVPLPADLKIPPESISPWRGGETEGLQRL
EQHLTDQGWVASFTKPRTIPNSLLPSTTGLSPYFSMGCLSVRTFFYRLSNIYAQAKHHSLPPVSLQGQLLWRE
FFYTVASATPNFTKMAGNPICLQISWYEDAERLHKWKMAQTGFPWIDAIMTQLRQEGWIHHLARHAAACFLTR
GDLWISWEEGMKVFEELLLDADYSINAGNWMWLSASAFFHQYTRIFCPVRFGKRTDPEGQYIRKYLPVLKNFP
SKYIYEPWTASEEEQKQAGCIIGQDYPFPMVDHKEASDHNLQLMKQVREEQYRTAQLTRDDTDDPMEMKLKRD
HSEENVTKAKAAKMTE
>Antrostomus_caro__KFZ50447.1
DNWAFLYAQRLALKQELPLHVCFCLVPKFLDATIRHYGFMLKGLQEVAKECAELNIPFHLLLGYAKDVLPTFV
VEHGVGGLVTDFCPLRLPRQWVEDVRERLPEDVPFAQVDAHNIVPCWVASPKQEYSARTIRGKIHAQLPEFLT
EFPPVICHPYPPSCKAEPIAWEACYSSLQVDRTVKEVEWATPGTAAGLAVLQSFIAERLKSFGAGPSTAARTS
SPTCSLPMFPGQVSTQRAILEVQKHQSKYKESVDAFVEEAVVRRELAENFCYYNENYDSVQGAYDWAQTTLKL
HAKDKRPFLYELQKLEQGTTHDPLWNAAQLQMVREGKMHGFLRMYWAKKILEWTHSPEEALRFAIYLNDRYEL
DGRDPNGYVGKLQDGGMGLRPHPWPAASGPSAICGIHDQGWAERAIFGKIRYMNYAGCKRKFDVGQFERRYAP
>Antrostomus_caro__XP_010162591.1
MSHRTIHLFRKGLRLHDNPTLLAALESSEAVYPVYILDRKFMTSVMHIGALRWHFLLQSLEDLQKNLCQLGSC
LLVIQGEYESVLRDHVQKWNITQVTLDAEVEPFYKEMDANIRCLGEELGFEVLSLVAHSLYDTKRILDLNGGT
PPLTYKRFLHVLSLLGDPEVPVRNLTAEDFERCRPPDLGLDESYKVPLPADLKLPSESLSPWRGGETEGLRRL
EQHLIDQGWVTSFTKPRTIPKSLLPSTTGLSPYFSVGCLSVRTFFYRLSNIYAQAKHHSLPPVSLQGQLLWRE
FFYTVASATPNFTKMAGNPICLQIRWYEDAERLHKWKTAQTGFPWIDAIMTQLRQEGWIHHLARHAVACFLTR
GDLWISWEEGMKVFEELLLDADYSINAGNWMWLSASAFFHQYTRIFCPVRFGKRTDPEGQYIRKYLPVLKNFP

SKYIYEPWTASEEEQKQAGCIIGQDYPFPMVNHKEASDHNLQLMKQVREEQYRTAQLTRDDTDDPMELKVKRD
HSEESVSKGKVAKMTEQTEIPPGITCWEPKNGKRGKAEGS
>Antrostomus_caro__XP_010167338.1
NLVASYKETRNGLVGMDYSTKFAPWLALGCISPRYIYEQIQKYEKERTANQSTYWVLFELLWRDYFRFVALKY
GKRIFSLRGLQSKEIPWKKDLQLFDCWKEGKTGVPFVDANMRELAVTGFMSNRGRQNVASFLTKDLGLDWRMG
AEWFEYLLVDYDVCSNYGNWLYSAGVGNDPRDNRKFNMIKQGLDYDGN
>Antrostomus_caro__XP_010173579.1
FLLQCLEDLDANLRKLNSRLFVIRGQPADVFPRLFKEWNIAKLSIEYDSEPFGKERDAAIKKLASEAGVEVIV
RISHTLYDLDKIIELNGGQPPLTYKRFQTLISRMEPLEMPVETITPEVMEKCTTPVSDDHDEKYGVPSLEELG
FDTDGLPSAVWPGGETEALTRLERHLERKAWVANFERPRMNANSLLASPTGLSPYLRFGCLSCRLFYFKLTDL
YKKVKKNSSPPLSLYGQLLWREFFYTAATNNPRFDKMEGNPICVQIPWDKNPEALAKWAEGRTGFPWIDAIMT
QLRQEGWIHHLARHAVACFLTRGDLWISWEEGMKVFEELLLDADWSVNAGSWMWLSCSSFFQQFFHCYCPVGF
GRRTDPNGDYIRRYLPVLRGFPAKYIYDPWNAPESIQKAAKCIIGVNYPKPMVNHAEASRLNIERMKQIYQQL
SRYRGLGLLATVPSNPNGNGNGGLMGYSPGESISGCGNTGGAQLGTGDSHTVVQPCALGDSHSGASGVQQQGY
CQASSILHYAHGDNQQSHLLQAGRTSLGTGISAGKRPNPEEETQSVGPKVQRQSTN
>Antrostomus_caro__XP_010173836.1
MQHNSIHWFRKGLRLHDNPALLAAVRDSRCLHPLFILDPTDAPPGANARRFLLDSLWDLDGNLRKMGSRLLVA
WGCPQEVFPRLFHAWRTTRLTFEVDTEPSARRRDAAVAELAAQHGVEVIQEVSHTLYDTQRVLALNDGKAPLT
YKHLQKLLVSLGPPEKPVPALTQEHLQGCHTLCQLSHDTNYDVPTLEELGQDPNEVGPHLYPGGETAALARLD
TLMEKTTWVCSFKKPETEPTSLIPSTTVLSPHLKFGCLSVRTFWWRLDEIYQGHSQPPVSLHGQLLWREFFYM
AGAGIPNFDRMVGNPICLQVNWDDNPQHLHAWREGRTGYPFIDSIMTQLRTEGWIHHLARHAVACFLTRGDLW
ISWEEGLKVFEELLLDADWSLNAGNWLWLSGSAFFHRYFHVYSPVAFGKKTDQDGAYIRKYLPILKDFPAEYI
YEPWKAPRAVQERAGCLVGTHYPQPIVEHGVVRERNMGRMKAARAQKGTKREPPADPSVDKSRRKKPKAERC
>Antrostomus_caro__XP_010174076.2
FLLQSLEDLDNSLRKLNSRLFVVRGQPTDVFPRLFKEWGVTRLTFEYDSEPFGKERDAAIIKLAKEAGVEVVM
ENSHTLYDLDRIIELNGHKPPLTYKRFQAIISRMELPKKPVSTVTSQQMEACKVDIQENHDDVYGVPSLEELG
FPTDGLAPAVWQGGETEALARLDKHLERKAWVANYERPRMNANSLLASPTGLSPYLRFGCLSCRLFYYRLWEL
YKKVKRNSTPPLSLYGQLLWRXGVPPATNNPKFDRMEGNPICIQIPWDRNPEALAKWAEGKTGFPWIDAIMTQ
LRQEGWIHHLARHAVACFLTRGDLWISWESGVKVFDELLLDADFSINAGSWMWLSCSAFFQQFFHCYCPVGFG
RRTDPSGDYVKRYLPKLKGFPSRYIYEPWNAPESVQKAAKCIIGVDYPKPMVNHAETSRLNIERMKQIYQQLS
RYRGLCLLASVPSCVEDLSGPVADSASGQGCSTSTAVRLSQADQASPKRKHEGAEELCTEELYKRAKVTGLPA
PEIPGKSL
>Apaloderma_vitt__XP_009863296.1
RFLLQSLEDLDNSLRKLSSRLFVVRGQPTDVFPRLFKEWGVTRLTFEYDSEPFGKERDAAIVKLAKEAGVEVV
IENSHTLYDLDRIIELNGHKPPLTYKRFQAIISRMELPKKPVSTVASQQMETCKVDIQENHDDVYGVPSLEEL
GFPTDGLAPAVWQGGETEALARLDKHLERKAWVANYERPRMNANSLLASPTGLSPYLRFGCLSCRLFYYRLWE
LYKKVKRNSTPPLSLYGQLLWRGXVTPATNNPKFDRMEGNPICIQIPWDRNPEALAKWAEGKTGFPWIDAIMT
QLRQEGWIHHLARHAVACFLTRGDLWISWESGVRVFDELLLDADFSVNAGSWMWLSCSAFFQQFFHCYCPVGF
GRRTDPSGDYVKRYLPKLKGFPSRYIYEPWNAPESVQKAAKCIIGVDYPKPMVNHAETSRLNIERMKQIYQQL
SRYRGLCLLASVPSCVEDLSGPVTDSASGQGCSTSTATRLSQADQASPKRKHEGAEELCAEELYKRAKVTGLP
APEIPGKSL
>Apaloderma_vitt__XP_009864284.1
MPHRTIHLFRKGLRLHDNPTLLAALESSEVVYPVYILDRTFVTSAMRVGALRWHFLLQSLEDLQKNLCQLGSC
LLVLQGEYEAVLRDHVRRWEITQVTLDAEMEPFYKEMEANIQRLGEELGFKVLSLVGHSLYDTKRILELNGGT
PPLTYKRFLHILSLLGDPEVPVRNLTAQDFQXSLSHVRCKSPDRSLADCYRVPLPADLKIPPESLSPWRGGET
EGLQRLEQHLTDQGWVASFTKPRTIPNSLLPSTTGLSPYFSLGCLSVRTFFYRLSNIYAQAKHHSLPPVSLQG
QLLWREFFYTVASVTPNFTRMAGNPICLQISWYEDAERLHKWRTAQTGFPWIDAIMTQLRQEGWIHHLARHAV
ACFLTRGDLWISWEEGMKVFEELLLDADYSINAGNWMWLSASAFFHQYTRIFCPVRFGKRTDPEGHYIRKYLP
ILKNFASKYIYEPWMASEEEQKQAGCIIGQDYPFPMVNHKEASDHNLQLMKQVREEQHRTAQLTRDDTDDPMD
VKVKRDHSEENVAKGKAAKMTEQTEIPSGITCCEPENGKKCEPEAS
>Apaloderma_vitt__XP_009867267.1
SVREFKYNKKRVRLISQGSDLKDDARCILYWMSRDQRVQDNWAFLYAQRLALKQELPLHVCFCLVPKFLDATI
RHYGFMLRGLQEVAKECAELNIPFHLLLGYAKDVLPAFVVEHGVGGLVTDFSPLRLPRQWVEDVRERLPEDVP
FAQVDAHNIVPCWVASSKQEYSAWTFRSKINAQLPWFLTEFPPIIRHPYPPSCSAEPIAWEACYSSLQVDRTV
KEVEWATPGTAAGLAVLQSFIAERLKSFGSHRNNPNKAALSNLSPWFHFGQVSTQRAILEVQKHQHKYKESVD
VFVEEAVVRRELSENFCYYNENYDSVQGAYDWAQTTLKLHTKDKRPYLYKLQELEQGSTHDPLWNAAQLQMVR
EGKMHGFLRMYWAKKILEWTRSPEEALQFAIYLNDRYELDGRDPNGYVGCLWSICGIHDHGAVFGKIRYMNYA
GCKRKFNVGEFERRYAPGDGHSVP
>Apaloderma_vitt__XP_009868834.1

FDTDGLPSAVWPGGETEALTRLERHLERKAWVANFERPRMNANSLLASPTGLSPYLRFGCLSCRLFYFKLTDL
YKKVKKNSSPPLSLYGQLLWREFFYTAATNNPRFDKMEGNPICVQIPWDKNPEALAKWAEGRTGFPWIDAIMT
QLRQEGWIHHLARHAVACFLTRGDLWISWEEGMKVFEELLLDADWSVNAGSWMWLSCSSFFQQFFHCYCPVGF
GRRTDPNGDYIRRYLPVLRGFPAKYIYDPWNAPESIQKAAKCIIGVNYPKPMVNHAEASRLNIERMKQIYQQL
SRYRGLGLLATVPSNPNGNGNGGLMGYSPGESISGCGSTGGAQLGTGDGHTVVQPCALGDSHAGASGIQQQGY
CQASSILHYAHGDNQQSHLLQAGRTALGTGISAGKRPNPEEETQSVGPKVQRQSTN
>Apaloderma_vitt__XP_009873484.1
KDLRETLKKKGSTLVVRKGKPEDVVRDLIIQLGSVCAVAFHEEATQEELDVEKGLCQVCSQHGVKVHTFWAST
LYHRDDLPFRPIARLPDIYTHFRKAVESEAKVRPTLQVADQLKPLAPGVEEGSIPTMEDLGQEDPVTDPRTAF
PCSGGETQALMRLQYYFWDTNLVASYKETRNGLLGMDYSTKFAPWLALGCISPRYIYEQIRKYEKERTANQST
YWVLFELLWRDYFRFVALKYGRRIFSVRGLQSKDVPWKKDLQLFDRWKEGKTGVPFVDANMRELAATGFMSNR
GRQNVASFLSKDLGLDWRMGAEWFEYLLVDYDVCSNYGNWLYSAGVGNDPRDNRKFNVIKQGLDYDGNGDYVR
LWVPELQGIKGADVHGPWALSRAALSQAGVTLGETYPWPVVTAPEWSRHINQRPVSTGKVWMGRRGPAHKDRG
IDFYFSRKKDV
>Aphanomyces_eut_KAF0733358.1_Ae201684_0
MDERPAPKRKEPSSPQERAASTPSPRKKQAVDKDMGPTSLIWFRKGLRLHDNPALVEAIRGARHLIPVMVIDP
ALAHPGKCGARPLQFFLECVQDLDRSLQARQSRLVILHGDPSELIPRYVKRWGVSKLCFEEDTGRYSQARDAA
VRDSIASSVAVRSFSTHTLYKSADVRKVYAPNGYPQKYEAFLTRVRSLEIDDPLPDASLPETFPPVPDDAISE
SQSIPTIQSLGFDCQLDPIPLVGGETSALERMEKALEDEKWVRDFQKPQMSPLAIAPASTTCLSPYLARGCLS
VRLLYSKIETILAKGTHSKPPVSLKGQLLWREFYYAASYLTPNYYEMEGNPVCRQIPWSRDAEKLEAWTNALT
GYPWIDAAMTQLRQEGWIHHLARHAVACFLTRGDLWVDWRLGRDVFDKMLLDTDPALNNGNWQWLSASAYFHQ
YFRVYGPVSFAKKYDSSGAYIRHYLPVLRKMPDKFIYEPWKAPKAVQSAAGCVLGKDYPKRIVVHEVVSKELT
ERMAAVYKASK
>Aphanomyces_eut_KAF0744011.1_Ae201684_0
MRLQAADLARKGLRKERLQWLRGTATEDGTHVLYWMQTSLRTKFNFALEVAVDAASRLKQPLHILHTIETPAT
ERHIAFLLESLVDVHTTLAKKNLHLSVAHGAKPSVEVALLASKGASVVIVDHPYLRDANALNQNFAQSATTSV
LQVEGDAVVPVEHASDKEEHAARTIRPKITKLLDKYLVPLEPADASNIPESKPLSETLQDCGWLDMSQSVDDL
LASFPSLDRSIFRVSTFKGGETSAQANLASFLKNKLVKYDTSRNEPSGDGSSNMSPYLHYGNISPVDIALQTK
AFPGTGPALSASKAAFLEELIVRRELSMNFVWYNPRYDTFAALPAFAVQTLHDHADDARPHTYTFEQLDKAKT
ADVYWNAAQLDMILTGKMHNYMRMYWGKKIIEWTASPEDAFRYAIELNDKYNLDGYDPNSYTGIAWVFGKHDQ
GWKERPIFGKVRYMNAEGLERKFNMAGYIGKIRKLAQARGLSVGSTLTLPPAATKKRKR
>Aphrocallistes_vas_CAD24679.1_photolyase
MESLSITEFSPSAPSPISSSSSPDEETDRDIVAKDIEQDVEPQGKVLLHIFNNRHLRLKDNTALYQAMAQNPD
KFYAVYIFDGFDSKPVAPVRWQFLIDCLEDLKEQLNGFGLELYCFRGETIDVLATLVQAWKVKLLSINMDPDV
NFTFFNEKIVKMCTINAVQLYNDMDSHRLLYLPPKYKSAIPMSKFRVLLAEAITAKQNNLESEAKIQDITPPL
NPEQLSDLGNKPRLDSPLPSEIPKLNALFTEEEIAKLNFIFQGGERRTEDYLNEYREARLRDVSGDEDASPIA
AKAMGISPHLRFGCITPRHLFNFLVKTIKDANYSRIKINKVLAGIMARDFALQVSQLQTIPERIISLNKICLP
IPWDKNNNEIVEKLTDAQTGFPFFDAAITQLKTEGYVINEVSEALATFVTNSLLWVSWEEGQNFFSQHLICFD
LAMSTHSWLEASGSTMVTGRQKSYQDPLLFVSKKLDPNGEYIKRYLPKFINFPIEFIHKPGNASLEAQQAANC
VIDIDYPKPLFEYECRNGICCKRLRVFMEVVDSAAKATKLPHVIENCSGKFP
>Apis_mel_NP_001077099.1_cry_2
MTGSRSSEINPKEGLYDEGGKHTVHWFRKGLRLHDNPSLREGLAGASTFRCVFVLDPWFAGSTNIGINKWRFL
LQCLEDLDCSLRKLNSRLFVIRGQPADALPKLFKEWGTTNLTFEEDPEPFGRVRDHNISALCKELGISVVQKV
SHTLYKLDEIIERNGDKPPLTYHQFQTVVASMDPPEPPVPTVTSACVGSAYTPLKEDHDDHYGVPTLEELGFD
TEGLLPPVWVGGESEALARLERHLERKAWVASFGRPKMTPQSLLPSQTGLSPYLRFGCLSTRLFYYQLTDLYK
KIKKAVPPLSLHGQLLWREFFYCAATKNPNFDRMQGNPICVQIPWDKNVEALAKWANGQTGFPWIDAIMTQLR
EEGWIHHLARHAVACFLTRGDLWISWEEGMKVFDELLLDADWSVNAGMWMWLSCSSFFQQFFHCYCPVRFGRK
ADPNGDYIRRYLPVLKNFPTRYIHEPWNAPLNVQRAAKCIIGKDYSLPMVNHSKSSRINIERMKQVYQQLNKY
RGNGVSLKGETVGLLNALPPSSMKETEEEKKKTKQSLSPSENQSKMEILPKTTQRQHHH
>Apis_mel_XP_006564509.1_deoxyribod
MEELNPFKRRKVFDLLKKFEDNRKNTSESIMTFKFNKKRIKRLNNLNDIKENCNGILYWMFRDIRIQDNWALL
FAQKAALKNNVPLHICFCLISNFLNASIRYYKFLLKGLEEIEKECKKLNINFHLLHGEPNINILKFIKIYNMG
AIITDFYPLKLPMLWIDNVKKNLPKDIPICQVDAHNIVPCWYASSKQEFAAKTIRNKINTKLEEFLTEFPPVI
KHPYTTKEKFEKNNWKIALQNVEVDKSVKEITWAKPGYENGIKELENFLQNRLKKYGDERNNPLSNAISNLSP
WFHFGMISVQRCILEIKEYKKLYKKSVESFMEEAIIRKELSDNFCFYNEKYDLIEGAYPWAIETLNKHRKDKR
KYIYFLNHLENSETHDDLWNACQNQMVTIGKMHGFLRMYWAKKILEWTETPEIALKWANYLNNKYSIDGCDPN
GYVGCMWSICGVHDHGWPERDIFGKIRYMNYEGCKRKFNIAEFVMKWGKKKTNELI
>Aplysia_cal_XP_005089742.1_cry-1_X1
MEENDTKSHVSVHWFRHGLRLHDNPALLDSLENCREFYPVFILDGNVAGISSAAFPRMQFLFETLQDLDDNLR
SHGSRLFVLRGKPVDVFAKMFEEWGVTRLTFEQDPEPVWQERDNSVKALCDKQNVEWIERVSHMLWEPRLILE

ENGGEPPLTYAMFNQVAQVVGPPPKPVGDPDFSGISLPCRKDDEESYKLPSLEEIDIRPESEEQASPFGRYLG
GETKALKLLSSRLEKEHEAFSIGQCLPTQLNPNLIGMPMSLSPHLRFGSLSVRKFYWSLRDTFSQLFPDRPVP
GSITSQLVWREYFYCMSVNNPMYNRMKENPICLDIDWYDDEQKFEKWKKGQTGFPWIDACMRQLLQEGWIHQV
CRHAVACFLTRGDLWIDWQKGLKVFDRYLLDADWSVCAGNWMWVSSSAFEKVLQCPRCICPVRYGKRIDPTGA
YVRRYVPELKRMPLQYLFEPWKAPLKLQEEVDCIIGKDYPEPMVDHRTASKECKTKMEAIKKVSKDVPHIAPA
NEEEVLTLMWSGKQTRSELMDACTHNLDKICL
>Aplysia_cal_XP_005098341.1_cry_DASH_X
MKVNICMLSVLRFTGIDHSFTFCRCLIKTPIGVSASISSTPVVERKSCRDYSTRLKSKHHRPEKVAKLQSGEP
KMSSFSSSPVNTVIYLFRNDLRVHDNEALLLANQKGTRLLPLYCFDPRHYSGTYHFGFPKTGNHRLSFLLDSV
KDLQKNLKSRGSDLVIRKGKPEEVIPDLLRTLNVTRDAAVVFQKEVMEEEVKVEKAIEAHVNVPVNTTWGHTL
YHVEDLPFQPIRLPDVYTQFRKKVEDNTTIRKCVHVPDALKPLPEGIEVGSLPSYEDLGVSEPEKDSRAAFPF
LGGETTALERLHSYLWGTDNVSTYKETRNGMIGSDYSTKFSPWLAHGCLSPRKIYWEIKKYEKERTSNQSTYW
VIFELIWRDYFRFVGLKYGNKLFHSGGIKGDRVEWKVNKEQFKAWQEGRTGVPYVDANMRELMATGFMSNRGR
QNVASFLTKDLHLDWRLGAEWFESMLIDHDVCSNYGNWLYSAGIGNDPRENRKFNVVKQGLDYDAEGDYVRLW
VPELAGIKTGSVHCVWTLSPAVLESGDVSLGQTYPLPLVKAPEWSRHVNRPGSSGGGRDKGPGSARGRGQNKG
SHRGQGHQSQQAGQKRGLDFYFSSSKPH
>Aplysia_cal_XP_012935158.1_photolyase
MSSSDEEVVNGGATSKTEQEEKTKDSKGKRAVKRKQDSKTNTSAKKAKTAETAASEDSNGESDSPSKSEAGGD
TGDFIERINKQRTSVCASVDKFKFNKNRVRVLSKEEDFPDDSDGVVYWMSRDQRVQDNWAFLYAQRLALKLEA
PLHVCFCLVPKFLEATIRQFGFLLKGLAQVEKECRELNIAFHLLLGEASKVLPQFVKDNSIGGVVTDFSPLRT
PAKWVDELKEALPEDVPLCQVDAHNIVPCWLASPKLEYGARTIRNKIHNQLGSFLTEFPPVSRHPHPPSKMPP
VTDWKAADGYLQVDRSVAEVTWAKPGTDPGLRMLESFCEKRLKSFATSRNDPNKQALSNLSPWIHFGQISVQR
CILMVKKYRSKSSEGVNAFIEEAVIRRELADNFCFYNEKYDSVKGAYDWAQKTLKIHSGDKRPYLYDREKLEQ
GKTHDDLWNAAQIQMVTEGKMHGFLRMYWAKKILEWTPSPAVALTEAIYLNDKYNLDGRDPNGYVGCMWSICG
IHDQGWAERDVFGKIRYMNYKGCQRKFDVANFVRKYGKKKA
>Aplysia_cal_XP_012941094.1_cry-1
MKENLPDNPTNMSQDPKKHSLFWFRKGLRIHDNPALIAAIEGASTYRCVYILDPWFAGASQVGINKWRFLLES
LEDLDSSLRKLNSRLFVVRGQPADVLPRLFQEWAITCLAFEEDPEPYGKERDSAISAMAREFNVQVIAKSSHT
LYDPKLVIAANGNSPPLTYKRFQSVLSTLEPPNQPCETMTARLLHEMTTPVADDHDDRFSVPSLDELGFDTDT
LDVAHFRGGESEAVARLHRHLERKAWVASFERPKMSPQSLYPSGTGLSPYLRFGCLSARTFYWKLTELYKKVK
KGAEPPLALHGQLLWREFFYTVATNNPNFDRMVGNSICVQIPWEHNPEALVKWAEGMTGFPWIDAIMVQLKKE
GWIHHLARHAVACFLTRGDLWISWEEGMKVFDEMLLDADWSVNAGMWMWLSCSAFFQQFFHCYCPVGFGKRAD
PSGDFVRHYLPVLKAMPTKYIYEPWTAPESVQKVAKCIVGKDYPMPMVVHSDVSRINLERMRQVYKRLVLQSS
TSKRMFLDLPKIPKVSMFLSKAKMFLSSSSDDEVSQKQRRRKQVDE
>Aplysia_cal_XP_035828705.1_cry-1
MGKHRAIHWFRKGLRLHDNPALLKACEQASELYPIFVLDPWFASSPQCHVGCNRWRFLLQALQDLDQSLQSLN
LRLFVIRGQPEVEIEKLCQQWSITKLTYEADTEPYAVQRDAKVEARMEALGVEVIKCVSHTLFDVNLTVKNNG
GCAPLTYQKFQSVLSKMGTPAKALLSPTLTDLEGCKTPVSSSHDQYYGVPTLEELGKSSSDCGPLLFPGGETE
ALDRMERHLKKINWICNFEKPKTSPNSLSPSTTVLSPYLKFGCLSPRLFYHRLQQVYSGKKHSQPPVSLHGQL
LWREFFYTVGAVTPNFDRMKGNPVCRQIPWGSKPEHLEAWTEARTGYPFIDAVMTQLRLEGWIHHLARHAVAC
FLTRGDLWVSWEQGMKVFEELLLDADWSLNAGNWMWLSASAFFHQYFRVYSPVAFGKKTDPNGDYLRKYLPVL
KKFPAKYIFEPWLAPSSVQQAAGCIVGKDYPRPIVDHDRARQENIKKMAAAYAAAKSSNEGSPVKTSGGGKKK
LSTELDKQPNKKLKK
>Apolygus_luc_KAF6198971.1_GE061_0069
MQPNLLGAPTSQSPALRFGCLSIRRFYWTLHDLFREMYGDMPSLSQSVTGQLIWREYFYTMSVDNPYYAEMER
NPICLKINWLSRTDPTYQKKLDSWKNGMTGYPFIDAVMRQLQAEGWINHVARNAVACFLTRGNLWISWEDGLQ
HFLKYLLDADWSVCAGNWMYVSSSAFEQLLDCSYCICPVNFGRRLDPYGEYVK
>Apolygus_luc_KAF6206655.1_GE061_0178
MAPCCSRQEIRDIVTPPRDWIFPRAVTLRSHVFHPTTLPFVSVANFVSAVFPEKSLQLTAFLGWHCFMCYSDS
PKRVTSSLPTIEINDILDCLGNNNLIQLRGLRLHDNPSLKQGLSNATTFRCIFILDPWFAGSSNVGINKWRFL
LQCLDDLDRSLRKLNSRLFVIRGQPADILPKLLKEWGTTCLSFEEDPEPFGRVRDQNIIALCRSMNITVHTSV
AHTLYKLEAIIEKNHGKAPLTYHQFQGIISSMDPPPQPEPPVTLATIGRAKSIIREDHEDKFGVPTLEELGFD
IEGLRPGVWLGGESEALSRLERHLERKAWVASFGRPKMTPQSLHASQTGLSPYLRFGCLSTRLFYYQLNDLYR
KIKRAIPPLSLHGQILWREFFYCAATRNPNFDRMVGNPICVQVPWDKNPEALAKWANGQTGFPWIDAIMTQLR
EEGWIHHLARHAVACFLTRGDLWLSWEEGMKVFDELLLDADWSVNAGMWMWLSCSSFFQQFFHCYCPVRFGRK
ADPNGDYIRKYLPILKNMPTKYIHEPWNAPENVQKAVKCVIGVDYPVPMLNHSVVAKNNIERMKQVYHQLIKY
KGPGIMSSSPSGNYQEKKKEETIYDHNNIFATPVAPTLVKPASNIR
>Apolygus_luc_KAF6213161.1_GE061_0108
MATAVLRTSRKAISVHLHLLEVRADKIFRRIYIIRMASAAKKLKISGESSSSTNKELKDFLHSVEKNRKEAAT
SIADFKFNKNRVRVLSKEEAVADDNWALLYAQKLALKFKLSLRVCFCTLPKYMDSTIRQSMFMLRGLEEVNAE

LKQLDIPFHLLYSNNKEEVAQNVLNLVDDHDVGCVVVDFSPLKIARAWVDDLKEILPKDIPLCEVDAHNIVPC
WVASDKLEYGARTIRNKINSKLGEFLTKFPPLIQHPYPVDKHSWSINWEDEAKKFEVDRTVEEITWAQPGYQA
GMKTLHEFCEKRLRNFATKRNNPLVNALSNLSPWYHFGQISIQRCILHVNTFKSKYNESVNAFCEESIVRREL
ADNFCFYNAKYDKIDGAYDWAKKTLNDHKKDKRTYVYSCDELASSKTHDDLWNSAQIQLVKEGKMHGFLRMYW
AKKILEWTPSPEEALRIALYLNDRFSIDGRDPNGFVGCMWSICGIHDQGWREREIFGKIRFMNYDGCKRKFDV
PAFVARYGGKVYKPSKK
>Apostichopus_jap_PIK34639.1_putative_c
MIIVTFAFMIAYVSVNAFVTPSLFLLDCLEDLDKSLRKLNSRLFLVRGQPADVFPRLLKEWSITHLSFEEDCE
PYGREKDQVIKALAQEAGVEVLRLNNNEPPLTYSRFQEIVSIIGPPEHPVAPVTAQDVEGVDMAIASDHYEKY
GIPSLEELGFDTENIPVKVWVGGETEALQRLNRHLERKAWVANFERPRMSPIALNPSPTSLSPYLRFGCLSSR
MFYWELTDLFIKRTNKIPLSLHGQLLWREFFFSVGCNNPNLDQMENNPICIQIPWDRNPDYLKRWAEGKTGFP
WIDAIMTQLRLEGWIHPLARHAIACFLTRGDLWISWEEGVKVFEELLLDADWSVNAGNWIWLSCSSFYQQFFH
CYCPVKFGRRTDPEGEFIRKYLPVLKNMPTKYIFDPWTAPVEVQEEAKCIIGKHYPAPIVNHAEASHTNIEKM
KKVFKNISKYCGPGALKIDLDNFIFNKIFEFNNILVGQLRTMLIKSNVWKNKSYVSPVNTQKEAPKETTDSLC
MQQQNVIQQVQKVYSSPVDMMKMPGMSHSQGGDQQRQVNFMGNQIGLAGSRVTQMSSNLQGMGHSFFS
>Apostichopus_jap_PIK41746.1_6-4_photol
MKHHSVHWFRKGLRLHDNPSLIAALKSSRKIWPIFILDPHFVKSGTVGINRWRFLLESLMDLDRSFQELGSRL
FVIRGSPAEVFPKIFKDWEISQLTFEIDTEPYARQRDSEVIKLAEEHEVKVVQTVSHTLFDSEKIIKANGGKP
PLTYQRMQTVLSKMGPPSEPMAAPTKENFTECKTPEESDHSSKYGVPTLEDLGLSTEGISPVIFPGGETEALN
RMERYLARDKWVCNFEKPKTSPNSLKASTTVLSPYLKFGCLSVRSFYYGIQAVYSGKTHSKPPVSLHGQLLWR
EFFYTAAAGTPNFHQMKGNPVSLQIPWDENRAFLDAWTWFTLQSVYELVAFPVALIDDLLLSTDTSIAGRQNW
LPIHRCYYDAIRKEGWIHHLARHAVACFLTRGDLWISWEEGERVFEELLLDADYSLNAGNWMWLSASAFFHQY
FRVYSPIVFGKKTDPNGDYIKKYLPILKKFPPKYIYEPWVAPRSMQEQAGCIIGEDYPKPIVDHATISKKNIS
RMKEARANQLGTEGKKSASTAKDGKRKFDDASDEKPPPSTKSKRDSKKQKKLTQFMKK
>Apostichopus_jap_PIK43288.1_putative_c
MQSINSVARQYIDQSKYICNPLVDWKNYQSECQLPNLVEEIVICVLRNDLRYHDNEVLLWAHNNADHVLPIYC
FDPRHYEGTWHFNFPKTGPFRLKFLLESLGDLRSILKKAGSGLIVRRGEVEKVVPDLIQTIGKSNVHAVAFQE
EVTKEELDVQTALVKNLAGTGIKKIWGSTLFHKDDIPFKSDRVPDVFTEFRKRCEGGATIRSTLQMPDQFRPL
PPGIEEGNLPTMEDLGQAAPVNDSRTAFPFKGGETTGLDRIQQYFWGSNNISTYKETRNGLIGSEYSTKFSTW
LAHGCLSPRKIYEEVKKYERQRTSNQSTYWVIFELIWRDYFKFVALKFGDRLFYLHGLMGVRKPWKRDQRLFD
AWKDGRTGVPFVDSNMRELKSTGFMSNRGRQNVASFLTKDLGLDWRLGAEWFEYLLVDHDVCSNYGNWLYSAG
VGNDPRQDRKFNMVKQGLDYDPQGDYIRQWIPELNTIRDGSVHTPWTLSLGALRQSEVSLGETYPNPIVKAPE
WNRHSHRNNKGGNRGASGGGRGKGGHSRGKQRGMDFYFSSSQKK
>Apostichopus_jap_PIK55338.1_putative_c
TQLCGFNRWQFLHDCLKDIDDQFRAEGGMLYCFEGDPTEVLRKQFLEWNVDVLCFEQDPEPIWQERDNKVKEL
CKEMGVQCIEQVSHTLWNPLTVISENGGSPPLTYSLFIEVVQRIGSPPKPLKTPNPAKWKCIPKTEEADRSYK
LKPLQYFACQEMAGQEKKRWTGGETRALDLYEMRLKYEHEGFKSDIVMINQFNPKLLDPPLSLSPYLRFGALS
IRKFYWGIQEIYEEVKCTSAQNHVTAQLIWREYFYTMSVNNTYFDKMEKNPICLRINWYQDEEKLKAWTDGET
GYPWIDACMKQLRMEGWIHQVARHAVACFLTRGDLWISWEEGLQVFYKYLLDADYSVCSGNWMWVSSSAFEKF
LQCPSCFCPVNYGRRMDPQGHFVKKYIPALAKMPLKYLFNPWTAPPEFLLTADHPRRRGAEFAWLDPVPDVKC
EASDNCKKAEMYYHK
>Apostichopus_jap_PIK56101.1_photolyase
MADTDKGQKRQAEADNGTTEEKKLKADTEQASQSLLSKVAHFREECAASIADFNFNKKRVKVVSDVKDMPEEN
EGILYWMMRDQRVQDNWAMLYAQRLALKQEVPLHVCFCLDPSNLLPLRHHSFMLNGLKEVEQELSKLDISFHL
LIGETAETIAKFVGEKKIGGLVADFSPLRKPQEWVQDVAKAIPKNIPFCQVDAHNIIPCWVTSEKLEYAARTI
RPKVHKQLGSFLTEYPPVTVHPHKATTKHEVVNWDEPMNDQKIDRSITEVDWATPGSTAAMDMLESFVKERLK
VFATERNDPTKEALSNISPWLHFGQIAAQRCVLVVKQFKNKYKESVDSYTEELVVRRELAENFCFYNEKYDSI
EGTNEWAKKTLEVHAGDERKYVYTKEQFEEGKTHEDLWNAAQTQLVQEGKMHGFLRMYWAKKILEWSKSPQEA
LNIAIHLNDKYSLDGRDPNGYTGCMWSICGIHDQGWAERDVFGKVRYMNYEGCKRKFDVDLFVRRYKAKMN
>Apteryx_mantelli_mantelli_XP_013814986.1_PREDICTED:
RFLLQSLEDLDNSLRKLSSRLFVVRGQPTDVFPRLFKEWGVTRLTFEYDSEPFGKERDAAIVKLAKEAGVEVV
IENSHTLYDLDRIIELNGHKPPLTYKRFQAIISRMELPKKPVSSIISQQMETCKVDIQENHDDVYGVPSLEEL
GFPTDGLAPAVWQGGETEALARLDKHLERKAWVANYERPRMNANSLLASPTGLSPYLRFGCLSCRLFYYRLWE
LYKKVKRNSTPPLSLYGQLLWREFFYTAATNNPKFDRMEGNPICIQIPWDRNPEALAKWAEGKTGFPWIDAIM
TQLRQEGWIHHLARHAVACFLTRGDLWISWESGVRVFDELLLDADFSVNAGSWMWLSCSAFFQQFFHCYCPVG
FGRRTDPSGDYVKRYLPKLKGFPSRYIYEPWNAPESVQKAAKCIIGVDYPKPMVNHAETSRLNIERMKQIYQQ
LSRYRGLCLLASVPCVEDLSGQVADPALGQSSSTSTVMRLPQSDQASPKRKHEGAEELCTEELYKRAKVGGLP
TPEIPGKSL
>Apteryx_man_XP_013799507.1_LOW_QUALIT

MSHCTIHLFRKGLWLHDNPTLLAALESSEVVYPIYILDRQFMTQXXTXGHFLLQSLENLQKNMLQLDSCLLVI
QVECESVLXDHVKKWNITQVTLDAEMEPFYKEMEANIQSLGEELGFAVLSLIGHSLYDAKRILDLNGGTPPLT
YKRFPHILSRLGDPEVPVRNLAAEDFQRCRPPDLCLAECYRVPLPVDLEIPTKSLFRWKGGETEGLLRLEQHL
LNQGWVASFTKLRTIPNSLLPSTTGLSPYFSLGYLSVRTFFYRLSNIYAQSKHRSLPPVSLQGQLLWREFFYM
VASATPNFTKMAGNPICLQINWYEDAEKLHKWKTAQTGFPWIDAMMTQLRQEGWIHHLARHAAACFLTRGDLW
ISWEEGMKVFEEMLLDADYSINAGNWMWLSASAFFHQYTRIFCPVRFGKRTDPEGQYIRKYLPILKNFPSKYI
YEPWTASEEEQKEAGCIAGQDYSFPMANHKEVSDHNLQLMEQVREEQYRTAQLTRDDADDPMEMKIKRDHSEE
NVKKGKVARTTEQT
>Apteryx_man_XP_013803208.1_cry-1_X1
MNLQGLRFLLQCLEDLDANLRKLNSRLFVIRGQPADVFPRLFKEWNIAKLSIEYDSEPFGKERDAAIKKLASE
AGVEVIVRISHTLYDLDKIIELNGGQPPLTYKRFQTLISRMEPLEMPVETITPEVMEKCTTPVSDDHDEKYGV
PSLEELGFDTDGLPSAVWPGGETEALTRLERHLERKAWVANFERPRMNANSLLASPTGLSPYLRFGCLSCRLF
YFKLTDLYKKVKKNSSPPLSLYGQLLWXXXXXXYTAATNNPRFDKMEGNPICVQIPWDKNPEALAKWAEGRTG
FPWIDAIMTQLRQEGWIHHLARHAVACFLTRGDLWISWEEGMKVFEELLLDADWSVNAGSWMWLSCSSFFQQF
FHCYCPVGFGRRTDPNGDYIRRYLPVLRGFPAKYIYDPWNAPESVQKAAKCIIGVNYPKPMVNHAEASRLNIE
RMKQIYQQLSRYRGLGLLATVPSNPNGNGNGGLMGYSPGENISACGSTGGAQLGTGDGHTVGVQTCALGDSHT
GASGIQQQGYCQASSILHYAHGDNHQSHLLQTGRTSLGTGISAGKRPNPEEETQSIGPKVQRQSTN
>Apteryx_man_XP_013814739.1_photolyase
MPQKKGKRKAGQGGEPEAEPDATELPRLSPKRSKKEAAMKKLEEAVQEARRKAAPSVQEFKYNKKRVRLISQG
SDLKDNAKCILYWMSRDQRVQDNWAFLYAQRLALKQELPLHACFCLVPKFLDATIRHYGFMLRGLQEVAEECT
ELNIPFHLLLGYAKDVLPTFVVRHGVGGVVTDFCPLRVPQQWVEDVRERLPEDVPFAQVDAHNIVPCWVTSPK
QEYSARTIRSKIHSQLPEFLTEFPPVIQHPYPPSTPAEPIAWEACYSSLQVDCTVKEVEWATPGTAAGLAVLQ
SFIAERLKSFGSHRNDPNKAALSNLSPWFHFGQVSTQRAILQVQKHHRKYKESVDIFVEEAVVRRELADNFCY
YNKNYDSVQGAYDWAQTTLKLHAKDKRPFLYELQELEQGNTHDLLWNAAQLQMVREGKMHGFLRMYWAKKILE
WTRSPEEALQFAIYLNDRYELDGRDPNGYVGCLWSICGVHDQGWAERAVFGKIRYMNYAGCKRKFDVVQFERR
YAPCKLNK
>Apteryx_rowi__XP_025912603.1
MNLQGLRFLLQCLEDLDANLRKLNSRLFVIRGQPADVFPRLFKEWNIAKLSIEYDSEPFGKERDAAIKKLASE
AGVEVIVRISHTLYDLDKIIELNGGQPPLTYKRFQTLISRMEPLEMPVETITPEVMEKCTTPVSDDHDEKYGV
PSLEELGFDTDGLPSAVWPGGETEALTRLERHLERKAWVANFERPRMNANSLLASPTGLSPYLRFGCLSCRLF
YFKLTDLYKKVKKNSSPPLSLYGQLLWREFFYTAATNNPRFDKMEGNPICVQIPWDKNPEALAKWAEGRTGFP
WIDAIMTQLRQEGWIHHLARHAVACFLTRGDLWISWEEGMKVFEELLLDADWSVNAGSWMWLSCSSFFQQFFH
CYCPVGFGRRTDPNGDYIRRYLPVLRGFPAKYIYDPWNAPESVQKAAKCIIGVNYPKPMVNHAEASRLNIERM
KQIYQQLSRYRGLGLLATVPSNPNGNGNGGLMGYSPGENISACGSTGGAQLGTGDGHTVGVQTCALGDSHTGA
SGIQQQGYCQASSILHYAHGDNHQSHLLQTGRTSLGTGISAGKRPNPEEETQSIGPKVQRQSTN
>Apteryx_rowi__XP_025919553.1
MSHHTIHLFRKGLRLHDNPTLLAALESSEVVYPIYILDRQFMTQXXTPGHFLLQSLENLXKNMLQLDSCLLVI
QVECESVLXDHVKKWNITQVTLDAEVEPFYKEMEANIQSLGEELGFAVLSLIGHSLYDAKRILDLNGGTPPLT
YKRFPHILSRLGDPEVPVRNLAAEDFQRCRPPDLCLAECYRVPLPVDLEIPTKSLFRXKGGETEGLLRLEQHL
LNQGWVASFTKLRTIPNSLLPSTTGLSPYFSLGYLSVRTFFYRLSNIYAQSKHRSLPPVSLQGQLLWREFFYT
VASATPNFTKMAGNPICLQINWYEDAEKLHKWKTAQTGFPWIDAMMTQLCQEGWIHHLARYAAACFLTRGDLW
ISWEEGMKVFEEMLLDADYSINAGNWMWLSASAFFHQYTRIFCPVRFGKRTDPEGQYIRKYLPILKNFPSKYI
YEPWTASEEEQKEAGCIAGQDYPFPMANHKEVSDHNLQLMEQVREEQYRTAQLTRDDADDPMEMKIKRDHSEE
NVKKGKVARTTEQT
>Apteryx_rowi__XP_025921631.1
MNRAQLKRPGILRMALGGSAADPLLPADGGEGRPAPFLLRALRIAVVVCLYWFTSITMVFLNKYLLDSPSLRL
DAPLFVTFFQCALTATLCLGLSLGAACGPPGCPDLPALRLDPKVSRSVLPLSAVFIGMITSNNLCLKYVGVAF
YNVGRSLTTVFNVLLSYLLLKQTTSLYALLACGIIIGGFWLGVDQEGAEGTLSWAGIFFGILASLCVSLNAIY
TKKVLPVVDGSIWRLTFYNNVNACVLFLPLMMLLGEFHTLYHFDKLGSPSFWGMMTLGGVFGFAIGFLLQSLE
DLDNSLRKLSSRLFVVRGQPTDVFPRLFKEWGVTRLTFEYDSEPFGKERDAAIVKLAKEAGVEVVIENSHTLY
DLDRIIELNGHKPPLTYKRFQAIISRMELPKKPVSSIISQQMETCKVDIQENHDDVYGVPSLEELGFPTDGLA
PAVWQGGETEALARLDKHLERKAWVANYERPRMNANSLLASPTGLSPYLRFGCLSCRLFYYRLWELYKKVKRN
STPPLSLYGQLLWREFFYTAATNNPKFDRMEGNPICIQIPWDRNPEALAKWAEGKTGFPWIDAIMTQLRQEGW
IHHLARHAVACFLTRGDLWISWESGVRVFDELLLDADFSVNAGSWMWLSCSAFFQQFFHCYCPVGFGRRTDPS
GDYVKRYLPKLKGFPSRYIYEPWNAPESVQKAAKCIIGVDYPKPMVNHAETSRLNIERMKQIYQQLSRYRGLC
LLASVPCVEDLSGQVADPALGQSSSTSTVMRLPQSDQASPKRKHEGAEELCTEELYKRAKVGGLPTPEIPGKS
L
>Apteryx_rowi__XP_025926657.1
MAEEVMFLFTGVAYHHTTPARLVIPFATAWSRPWRLQYLSQHCPPTRTVLGMPQKKGKRKAGQGGEPEAEPDA
TELPRLSPKRSKKEAAMKKLEEAVQEARRKAAPSVQEFKYNKKRVRLISQGSDLKDNAKCILYWMSRDQRVQD

NWAFLYAQRLALKQELPLHACFCLVPKFLDATIRHYGFMLRGLQEVAEECTELNIPFHLLLGYAKDVLPTFVV
RHGVGGVVTDFCPLRVPQQWVEDVRERLPEDVPFAQVDAHNIVPCWVTSPKQEYSARTIRSKIHSQLPEFLTE
FPPVIQHPYPPSTPAEPIAWEACYSSLQVDCTVKEVEWATPGTXAGLAVLQSFIAERLKSFGSHRNDPNKAAL
SNLSPWFHFGQVSTQRAILQVQKHHRKYKESVDIFVEEAVVRRELADNFCYYNKNYDSVQGAYDWAQTTLKLH
AKDKRPFLYELQELEQGNTHDLLWNAAQLQMVREGKMHGFLRMYWAKKILEWTRSPEEALQFAIYLNDRYELD
GRDPNGYVGCLWSICGVHDQGWAERAVFGKIRYMNYAGCKRKFDVVQFERRYAPCKLNK
>Apteryx_rowi__XP_025947336.1
KYGRRISSLRGLQGKEVPWKKDLQLFDCWKEGKTGVPFVDANMQELAATGFVSNRGRQNVTSFLTKELGLDWR
MGAEWFECLLVDYGICSNYGNWLYSTGIGNDPRDNRKFNTIRHGLDYDGGGDXVRLRVPELQGIKGGDIHTPW
ALSSASLSQAGTTLGETYPWPVVTAPEWSRRISKGPQGRGPHPKGRKGPAHTPMQHKDRGMDFYFSHKKDI
>Aquila_chry_chrysaetos_XP_029852375.1
MAAAALGPAPASAPAPALCRSVHWFRRGLRLHDNPALQEALRDAESLRCIYILDPWFAASSAVGINRWRFLLQ
SLEDLDNSLRKLNSRLFVVRGQPTDVFPRLFKEWGVTRLTFEYDSEPFGKERDAAIVKLAKEAGVEVVIENSH
TLYDLDRIIELNGHKPPLTYKRFQAIISRMELPKKPVSTVISQQMETCKVDIQENHDDVYGVPSLEELGFPTD
GLAPAVWQGGETEALARLDKHLERKAWVANYERPRMNANSLLASPTGLSPYLRFGCLSCRLFYYRLWELYKKV
RQKPPPPLSLYGQLLWREFFYTAATNNPKFDRMEGNPICIQIPWDRNPEALAKWAEGKTGFPWIDAIMTQLRQ
EGWIHHLARHAVACFLTRGDLWISWESGVRVFDELLLDADFSVNAGSWMWLSCSAFFQQFFHCYCPVGFGRRT
DPSGDYVKRYLPKLKGFPSRYIYEPWNAPESVQKAAKCIIGVDYPKPMVNHAETSRLNIERMKQIYQQLSRYR
GLCLLASVPSCVEDLSGPVTDSASGQGCSTNTAMRLSQADQASPKRKHEGAEELCTEELYKRAKVTGLPTPEI
PGKSL
>Aquila_chry_chrysaetos_XP_029856377.1
MPHRTIHLFRKGLRLHDNPTLLAALECSEVLYPVYILDRKFMTSVMHIGTLRWHFLLQSLEDLQKNLYKLGSC
LLVIQGEYESVLRDHIQKWNITQVTLDAEMEPFYKEMEANIRRLGEELGFEVLSLVGHSLYNTKRILDLNSGT
PPLTYKRFLHILSLLGDPEVPVRNLTAEDFQKCRPPDLGLAECYRVPLPVDLKITAASLSPWRGGETEGLQRL
EQHLTDQGWVASFTKPRTIPNSLLPSTTGLSPYFSMGCLSVRTFFYRLSNIYAQAKHHSLPPVSLQGQLLWRE
FFYTVASATPNFTKMAGNPICLQISWYEDAERLHKWKTAQTGFPWIDAIMTQLRQEGWIHHLARHAVACFLTR
GDLWISWEEGMKVFEELLLDADYSINAGNWMWLSASAFFHQYTRIFCPIRFGKRTDPEGQYIRKYLPILKNFP
SKYIYEPWTATEEEQKQAGCIIGQDYPFPMVNHKEASDHNLQLMKQVREEQYRTAQLTRDDSDDPMEMKVKRD
HSEENVSKGKVARMTEQTKIPSGITHWEPKNGKRGEPEAS
>Aquila_chry_chrysaetos_XP_029865135.1
MSGAARTAICLLRCDLRAHDNQVLHWAQSNADFVIPLYCFDPRHYVGTPCYGFPKTGPHRLRFLLESVKDLRE
TLKKKGSTLVVRKGKPEDVVCDLITQLGSVSAVVFHEEATQEELDVETELCQVCSQHGVKIHTFWASTLYHRD
DLPFRPIARLPDVYTHFRKAVESEAKVRPTLRMADQLKPLAPGVEEGCIPTMEDLGQKDPVTDPRTAFPCSGG
ERQALMRLQYYFWDTNLVASYKETRNGLVGMDYSTKFAPWLALGCISPRYIYEQIQKYEKERTANQSTYWVLF
ELLWRDYFRFVALKYGRRIFSLRGLQNKEVPWKKDLQLFDCWKEGKTGVPFVDANMRELAATGFMSNRGRQNV
ASFLTKDLGLDWRMGAEWFKYLLVDYDVCSNYGNWLYSAGIGNDPRENRKFNMIKQGLDYDSNGDYVRLWVPE
LQGIKGADIHTPWALNSAALSQAGVSLGETYPQPVVTAPEWSRHINQKPQGRSPHPRGRRGPAHTPMQHKDRG
IDFYFSHKKDV
>Aquila_chry_chrysaetos_XP_029878270.1
MSYIYQTRHFFPPKIHPHLSFPRLHPPWTPPAMQHSSIHWFRKGLRLHDNPALLAAATDCRHLHPLFILDPSS
SRAGTNAWRFLLDALRDLDGSLQEMGSRLFVVQGCPEEVFPCLFHAWGTTRLTFEVDTEPSARRRDATVAKLA
ERHGVEVIQEVSHTLYDTERVLALNDGKAPLTYKGLQSLLASLGPPEKPAPTLTLEHLQGCHTPCQVSHDTEY
GVPTLEELGQDPTEVGPHLYPGGERAALARLNALMDRTAWLCSFKKPETEPTSLSPSTTVLSPYLKFGCLSVR
TFWWWLHEVYQGREHSQPPVSLHGQLLWREFFYTAGAGIPNFDRMVSNPICLQVDWDNNPQHLRAWREGQTGY
PFIDAIMTQLRTEGWIHHLARHAVACFLTRGDLWISWEEGLKVFEELLLDADWSLNAGNWLWLSGSAFFHRYF
HIYSPVAFGKKTDRDGAYIRKYLPVLKDFPAKYIYEPWKAPRAVQEQAGCLVGTHYPPAHRGARGGEQEESGA
HEGGPRPERHQAGTTSWPLSS
>Aquila_chry_chrysaetos_XP_029885129.1
MRRGQGKRKAEVRELPRVSRRRKEEEEEAVQEARRRAAPSVREFKYNKKRVRLVSQGSDLKADARCILYWMSR
DQRVQDNWAFLYAQRLALKQELPLHVCFCLVPKFLDATIRHYGFMLRGLQEVAEECGELNIPFHLLLGYAKDV
LPMFVVEHGVGGLVTDFCPLRLPRQWVEDVRERLPEDVPFAQVDAHNIVPCWVASPKQEYSARTIRGKIHAQL
PEFLTEFPPVIRHPYPPSCPAEPVAWEACYSSLQVDHTVKEVEWATPGTAAGLAMLQSFIAERLKSFGSHRND
PNKAALSNLSPWFHFGQVSTQRAILEVQKHRGKYKESVDAFVEEAVVRRELAENFCYYNENYDSVQGAYDWAQ
TTLKLHAKDKRPFLYKLQELEQGTTHDPLWNAAQLQMVREGKMHGFLRMYWAKKILEWTRSPEEALQFAIYLN
DRYELDGRDPNGYVGCLWSICGIHDQGWAERAIFGKIRYMNYAGCKRKFDVGQFERRYTPRTLSQ
>Aquila_chry_chrysaetos_XP_029895934.1
MGVNAVHWFRKGLRLHDNPALRECIEGADTVRCVYILDPWFAGSSNVGINRWRFLLQCLEDLDANLRKLNSRL
FVIRGQPADVFPRLFKEWNIAKLSIEYDSEPFGKERDAAIKKLASEAGVEVIVRISHTLYDLDKIIELNGGQP
PLTYKRFQTLISRMEPLEMPVETITPEVMEKCTTPVSDDHDEKYGVPSLEELGFDTDGLPSAVWPGGETEALT
RLERHLERKAWVANFERPRMNANSLLASPTGLSPYLRFGCLSCRLFYFKLTDLYKKVKKNSSPPLSLYGQLLW

REFFYTAATNNPRFDKMEGNPICVQIPWDKNPEALAKWAEGRTGFPWIDAIMTQLRQEGWIHHLARHAVACFL
TRGDLWISWEEGMKVFEELLLDADWSVNAGSWMWLSCSSFFQQFFHCYCPVGFGRRTDPNGDYIRRYLPVLRG
FPAKYIYDPWNAPESVQKAAKCMIGVNYPKPMVNHAEASRLNIERMKQIYQQLSRYRGLGLLATVPSNPNGNG
NGGLMGYSPGESISGCGSTGGAQLGTGDGHTVVQPCALGDSHTGASGIQQQGRTALGTGISAGKRPNPEEETQ
SVGPKVQRQSTN
>Arabidopsis_tha_PHR2_NP_182281.1
MDSSNVEENLNPETKSAEEQNPLAIFHSSLPIASLSLTLFPSSTQFLKLFAHHPNKVKIPTQASSLTHLSLSS
VSPFPSSRISFKSTIAANPLQSPLSIVPRRPVDPSSAAALRRAAVVWFRNDLRLHDNECLNSANDECVSVLPV
YCFDPRDYGKSSSGFDKTGPFRAQFLIESVSELRKNLQARGSNLVVRVGKPEAVLVELAKEIGADAVYAHREV
SHDEVKAEGKIETAMKEEGVEVKYFWGSTLYHLDDLPFKIEDLPSNYGGFKDKVQKLEIRKTIAALDQLKSLP
SRGDVELGDIPSLLDLGISPTPRTSQEGKPTMVGGETEALTRLKSFAADCQARLSKGNQKGGNNSVFGANFSC
KISPWLAMGSISPRSMFDELKKTISASTTTTTPRNGPGDTGLNWLMYELLWRDFFRFITKKYSSAKTQVEAGP
ATACTGAFA
>Arabidopsis_tha_AAL16379.1_cry_2
MKMDKKTIVWFRRDLRIEDNPALAAAAHEGSVFPVFIWCPEEEGQFYPGRASRWWMKQSLAHLSQSLKALGSD
LTLIKTHNTISAILDCIRVTGATKVVFNHLYDPVSLVRDHTVKEKLVERGISVSSYNGDLLYEPWEIYCEKGK
PFTSFNSYWKKCLDMSIESVMLPPPWRLMPITAAAEAIWACLIEELGLENEAEKPSNALLTRAWSPGWSNADK
LLNEFIEKQLIDYAKNSKKVVGNSTSLLSPYLHFGEISVRHVFQCARMKQIIWARDKNSEGEESADLFLRGIG
LREYSRYICFNFPFTHEQSLLSHLRFFPWDADVDKFKAWRQGRTGYPLVDAGMRELWATGWMHNRIRVIVSSF
AVKFLLLPWKWGMKYFWDTLLDADLECDILGWQYISGSIPDGHELDRLDNPALQGAKYDPEGEYIRQWLPELA
RLPTEWIHHPWDAPLTVLKASGVELGTNYAKPIVDIDTARELLAKAISRTREAQIMIGAAPDEIVADSFEALG
ANTIKEPGLCPSVSSNDQQVPSAVRYNGSKRVKPEEEEERDMKKSRGFDERELFSTAESSSSSSVFFVSQSCS
LASEGKNLEGIQDSSDQITTSLGKNGCK
>Arabidopsis_tha_NP_566520.1_DNA_photol
MQRFCVCSPSSYRLNPITSMATGSGSLIWFRKGLRVHDNPALEYASKGSEFMYPVFVIDPHYMESDPSAFSPG
SSRAGVNRIRFLLESLKDLDSSLKKLGSRLLVFKGEPGEVLVRCLQEWKVKRLCFEYDTDPYYQALDVKVKDY
ASSTGVEVFSPVSHTLFNPAHIIEKNGGKPPLSYQSFLKVAGEPSCAKSELVMSYSSLPPIGDIGNLGISEVP
SLEELGYKDDEQADWTPFRGGESEALKRLTKSISDKAWVANFEKPKGDPSAFLKPATTVMSPYLKFGCLSSRY
FYQCLQNIYKDVKKHTSPPVSLLGQLLWREFFYTTAFGTPNFDKMKGNRICKQIPWNEDHAMLAAWRDGKTGY
PWIDAIMVQLLKWGWMHHLARHCVACFLTRGDLFIHWEQGRDVFERLLIDSDWAINNGNWMWLSCSSFFYQFN
RIYSPISFGKKYDPDGKYIRHFLPVLKDMPKQYIYEPWTAPLSVQTKANCIVGKDYPKPMVLHDSASKECKRK
MGEAYALNKKMDGKVDEENLRDLRRKLQKDEHEESKIRNQRPKLK
>Arabidopsis_tha_NP_567341.1_cry_1_
MSGSVSGCGSGGCSIVWFRRDLRVEDNPALAAAVRAGPVIALFVWAPEEEGHYHPGRVSRWWLKNSLAQLDSS
LRSLGTCLITKRSTDSVASLLDVVKSTGASQIFFNHLYDPLSLVRDHRAKDVLTAQGIAVRSFNADLLYEPWE
VTDELGRPFSMFAAFWERCLSMPYDPESPLLPPKKIISGDVSKCVADPLVFEDDSEKGSNALLARAWSPGWSN
GDKALTTFINGPLLEYSKNRRKADSATTSFLSPHLHFGEVSVRKVFHLVRIKQVAWANEGNEAGEESVNLFLK
SIGLREYSRYISFNHPYSHERPLLGHLKFFPWAVDENYFKAWRQGRTGYPLVDAGMRELWATGWLHDRIRVVV
SSFFVKVLQLPWRWGMKYFWDTLLDADLESDALGWQYITGTLPDSREFDRIDNPQFEGYKFDPNGEYVRRWLP
ELSRLPTDWIHHPWNAPESVLQAAGIELGSNYPLPIVGLDEAKARLHEALSQMWQLEAASRAAIENGSEEGLG
DSAEVEEAPIEFPRDITMEETEPTRLNPNRRYEDQMVPSITSSLIRPEEDEESSLNLRNSVGDSRAEVPRNMV
NTNQAQQRRAEPASNQVTAMIPEFNIRIVAESTEDSTAESSSSGRRERSGGIVPEWSPGYSEQFPSEENGIGG
GSTTSSYLQNHHEILNWRRLSQTG
>Arabidopsis_tha_OAO96306.1_CRY3
MNDHIHRVPALTEEEIDSVAIKTFERYALPSSSSVKRKGKGVTILWFRNDLRVLDNDALYKAWSSSDTILPVY
CLDPRLFHTTHFFNFPKTGALRGGFLMECLVDLRKNLMKRGLNLLIRSGKPEEILPSLAKDFGARTVFAHKET
CSEEVDVERLVNQGLKRVGNSTKLELIWGSTMYHKDDLPFDVFDLPDVYTQFRKSVEAKCSIRSSTRIPLSLG
PTPSVDDWGDVPTLEKLGVEPQEVTRGMRFVGGESAGVGRVFEYFWKKDLLKVYKETRNGMLGPDYSTKFSPW
LAFGCISPRFMYEEVQRYEKERVANNSTYWVLFELIWRDYFRFLSIKCGNSLFHLGGPRNVQGKWSQDQKLFE
SWRDAKTGYPLIDANMKELSTTGFMSNRGRQIVCSFLVRDMGLDWRMGAEWFETCLLDYDPCSNYGNWTYGAG
VGNDPREDRYFSIPKQAQNYDPEGEYVAFWLQQLRRLPKEKRHFPGRLMYMDTVVPLKHGNGPMAGGSKSGGG
FRGSHSGRRSRHNGP
>Arabidopsis_tha_OAP18495.1_UVR2_
MASTVSVQPGRIRILKKGSWQPSDQTVGPVVYWMFRDQRLKDNWALIHAVDLANRTNAPVAVVFNLFDQFLDA
KARQLGFMLKGLRQLHHQIDSLQIPFFLLQGDAKETIPNFLTECGASHLVTDFSPLREIRRCKDEVVKRTSDS
LAIHEVDAHNVVPMWAASSKLEYSARTIRGKINKLLPDYLIEFPKLEPPKKKWTGMMDKKLVDWDSLIDKVVR
EGAEVPEIEWCVPGEDAGIEVLMGNKDGFLTKRLKNYSTDRNNPIKPKALSGLSPYLHFGQVSAQRCALEARK
VRSTSPQAVDTFLEELIVRRELSDNFCYYQPHYDSLKGAWEWARKSLMDHASDKREHIYSLEQLEKGLTADPL
WNASQLEMLYQGKMHGFMRMYWAKKILEWTKGPEEALSISIYLNNKYEIDGRDPSGYVGCMWSICGVHDQGWK
ERPVFGKIRYMNYAGCKRKFNVDSYISYVKSLVSVTKKKRKAEEQLTRDSVDPKITIV

>Aramus_guar__NXO48908.1
MSHRTIHLFRKGLRLHDNPTLLAALESSEAIYPVYILDRRFMTSVMHIGALRWHFLLQSLEDLQQNLCQLGSC
LLVIQGEYESVLRDHIQKWNITQVTLDAEMEPFYKEMEANIRCLGEELGFEVLSLVGHSLYDTERILDLSGGT
PPLTYKRFLHILSLLGDPEVPVRNLAAEDFQRCRSPDLALAECYRVPLPVDLKIPLESLSPWKGGETEGLRRL
EQHLTDQGWVASFTKPKTIPNSLLPSTTGLSPYFSMGCLSVRTFFYRLSNIYAQAKHHSLPPVSLQGQLLWRE
FFYTVASATPNFTKMAGNPICLQISWYEDAERLHKWKTAQTGFPWIDAIMTQLRQEGWIHHLARHAVACFLTR
GDLWISWEEGMKVFEELLLDADYSINAGNWMWLSASAFFHQYTRIFCPVRFGKRTDPEGQYIRKYLPILKNYP
SKYIYEPWTASEEEQKQAGCIIGQDYPFPMVNHKEASDHNLQLMKQVREEQYRTAQLTRDDTDDPMETKVKRD
HSEENVSKGKVARLTE
>Aramus_guar__NXO50838.1
RFLLQCLEDLDANLRKLNSRLFVIRGQPADVFPRLFKEWNIAKLSIEYDSEPFGKERDAAIKKLASEAGVEVI
VRISHTLYDLDKIIELNGGQPPLTYKRFQTLISRMEPLEMPVETITPEVMEKCTTPVSDDHDEKYGVPSLEEL
GFDTDGLPSAVWPGGETEALTRLERHLERKAWVANFERPRMNANSLLASPTGLSPYLRFGCLSCRLFYFKLTD
LYKKVKKNSSPPLSLYGQLLWREFFYTAATNNPRFDKMEGNPICVQIPWDKNPEALAKWAEGRTGFPWIDAIM
TQLRQEGWIHHLARHAVACFLTRGDLWISWEEGMKVFEELLLDADWSVNAGSWMWLSCSSFFQQFFHCYCPVG
FGRRTDPNGDYIRRYLPVLRGFPAKYIYDPWNAPESVQKAAKCIIGVNYPKPMVNHAEASRLNIERMKQIYQQ
LSRYRGLGLLATVPSNPNGNGNGGLMGYSPGESISGCGSTGGAQLGPGDGHTVVQPCALGDSHTGASGIQQQG
YCQASSILHYAHGDNQQSHFLQAGRTALGTGVSAGKRPNPEEETQSVGPKVQRQSTN
>Aramus_guar__NXO52292.1
VSSLRFLLQSLEDLDNSLRKLNSRLFVVRGQPTDVFPRLFKEWGVTRLTFEYDSEPFGKERDAAIVKLAKEAG
VEVVIENSHTLYDLDRIIELNGHKPPLTYKRFQAIISRMELPKKPVSTVISQQMETCKVDIQENHDDVYGVPS
LEELGFPTDGLAPAVWQGGETEALARLDKHLERKAWVANYERPRMNANSLLASPTGLSPYLRFGCLSCRLFYY
RLWELYKKVKRNSTPPLSLYGQLLWREFFYTAATNNPKFDRMEGNPICIQIPWDRNPEALAKWAEGKTGFPWI
DAIMTQLRQEGWIHHLARHAVACFLTRGDLWISWESGVRVFDELLLDADFSVNAGSWMWLSCSAFFQQFFHCY
CPVGFGRRTDPSGDYVKRYLPKLKGFPSRYIYEPWNAPESVQKAAKCIIGVDYPKPMVNHAETSRLNIERMKQ
IYQQLSRYRGLCLLASVPSCVEDLSGPVTDSASGQGCSTSTVMRLSQADQASPKRKHEGAEELCTEELYKRAK
VTGLPAPEIPGKS
>Aramus_guar__NXO58702.1
KVDYDVCSNYGNWLYSAGIGNDPRDNRKFNMIKQGLDYDGNGDYVRLWVPELRGIKGADIHTPWALTSAALSQ
AGVTLSETYPQPVVTAPEWSRHINQRPQGRSPHPRGSRGPAHTPVQRTGIDFYFSRKKDA
>Araneus_ven_GBM49213.1_cry-1
MTSHKQKHVVHWFRKGLRLHDQPALREGLQGATTLRCVFILDPWFAGSSNVGINKWRFLLQCLENLDSNLRKL
NSRLFVIRGQPADVFPRLFKLWKTTHLTFEKDPEPYGRIRDHNTTTMAQENGVTVISRTSHTLYRLEKIVQKN
GSKSPLTYKQFQNILANMEPPPPPQPRITLEDVGSCYTPISDDHDEKYGVPTLEELGFDTENLKPPVWIGGET
EALARLERHLERKAWVASFGRPKMTPQSLLPSQTGLSPYLRFGCLSARLFYQELTELYRKIKKVNPPLSLHGQ
ILWREFFYCAATNNPKFDHMIGNSMCVQIPWDTNSEALAKWTNGQTGFPWIDAIMTQLREEGWIHHVARHAVA
CFLTRGDLWISWEEGMKVFDELLLDADWSVNAGSWMWLSCSSFFQQFFHLYCPVRFGRKADPNGDYIRRYLPV
LKNFPTKYIHEPWVAPEKVQIAAKCVIGKDYPMPMVNHQEVSHINLERMKQVYRQLSHYRGASMYSSSHNQQT
NQREGDDGYPKNSKRRAVEYPEDSGQVQKRDTTLK
>Araneus_ven_GBM67889.1_cry-1
MKVGPNRWRFLVQSLCDLDESLKQFNSRLFVIRGKPTEVLPDAIKRWKIKYLTFESDTEPYAKARDEEIENLM
RTLDVEVIKCCTNTLYDPEKIIAFNGKVPLTYQAFVNTADKIGLPSKPVPSVDQLKDLVKEQGRSTPVEDDHD
VKYSVPTLKELNVNESELNPCLYPGGETEALARLERVTANQEYICKFEKPNTSPNSINPSTTVLSPYVKFGCL
SARTFYYEIRDIYLKSKKGYSKPPVSLHGQLFWREFFYTAGSATPNFDQMIGNPVCKQIPWIDNDSFLQSWAH
AQTGYPFIDAIMTQLRKEGWVHHLARHSVACFLTRGDLWVSWERGMKVFEELLLDADWSLNAGNWMWLSASAF
FHQYYRVYSPIAFGKKTDKNGDYIRKYIPVLKKFPPEYIYEPWKAPKKLQEQLGCVIGKDYPSPIVDHDKVRV
ENLRRMDAVYKSKQENKDKDKKGSPSKSKESSVSKKAPSKGKRSLDENSQVKISKFLKNK
>Araneus_ven_GBN19159.1_cry-1
MSNAKFIHWFRADLRLHDVPTITTALKSCSTFYPVYIFDENDINEQKIKYNKVRFILDALKDIDSRLQLNGGR
LYVFKGNPAKIFSDLFKKWDISYLSYAEDVQPMWKETEKSVQEVCEQNGVKIIQEVSHTLWNLDEILNANKGQ
PPVTYEVFCHVVEVLGTPPRPEPELKLEGISMPRDDSLMCDLPTDPEELGVHAECIQQKNKIWIGGETRAFEH
LAERLKVEEAALADGFFMPNQVKPDLLGPSMSLSAALSLGCLSVRKFYWSLQDLHSSVNPDLCVPESLIGQLL
WREYFYAMCKNNPNYGTMQENPICLNIPWLTDDEKLKSWTNGQTGFPFIDAAMRQMIQEGWLHHVGRNAVACF
LTRGDLWISWEDGFKVFMKYLIDADFPVCAGNWMWVSSSAFENILQCPTCISPISYGRCFEPSGDYIRKYVPE
LKSMPTKYIFEPWLAPLSVQEQAGCIIGTDYPYPIVNHQEAAEINGKRMDSIKAGFYQKPFPLHCAPSNALET
RVFMWLPEKCIENLVQENN
>Araneus_ven_GBN22197.1_photolyase
MSSGKQKTLDDFGKPAKKQKTEESEQNNDHEFIKEIKHSRLSTAASIQDFKFNKKRVRVLSNTKDISENAESI
VYWMSRDQRVQDNWAFLYAQRLALKLDLPLCVCFCLVPKFLDATIRHYRFMLKGLKEVAEECESLNIHFHLLL
GEAKTVLPEFVKENKVGGIVTDFCPLRVPRKWVSDILTKLPDDVSLCQVDAHNIVPCWIASDKQEYGARTIRR

KIHDKLKEFLTLFPPVMKQKEIANCKLQDIDWEKVENSLEVNMDVKEVAWATPGTTAGLLKLSEFCSKRLKGF
SDLRNDPTKDNLSNLSPWFHFGQISIQRAILTVQKFKGKYGPSVDAFVEETVVRRELADNFCYYNKNYDKVEG
AYDWAKKTLKDHSKDKREYIYTKEQLENAKTHDLLWNAAQRQMTKEGKMHGFLRMYWAKKILEWTNSPEEALE
IAIYLNDKYELDGRDPNGYVGCMWSICGIHDQGWAERAVFGKIRFMNYKGCCRKFDVNEFIRRYREK
>Archocentrus_cent__XP_030575581.1
MVINTIHWFRKGLRLHDNPSLKESLLGADTVRCVYILDPWFAGSSNVGINRWRFLLQSLEDLDSSLRKLNSRL
FVIRGQPTDVFPRLFKEWKITRLSYEYDSEPFGKERDAAIKKLASEAGVEVTVRISHTLYDLDKIIELNGGQS
PLTYKRFQTLISRMDPVEVPAESITAEIMGKCTTPLSEDHDEKFGVPSLEELGFDTEGLSSAVWPGGETEALT
RLERHLERKAWVANFERPRMNANSLLASPTGLSPYLRFGCLSCRLFYFKLTDLYRKVKKNSSPPLSLYGQLLW
REFFYTAATNNPCFDKMESNPICVQIPWDRNPEALAKWAEGRTGFPWIDAIMTQLRQEGWIHHLARHAVACFL
TRGDLWISWEEGMKVFEELLLDADWSVNAGSWMWLSCSSFFQQFFHCYCPVGFGRRTDPNGDYIRRYLPVLRG
FPAKYIYDPWNAPEVVQKAAKCIIGVHYPKPMVHHAEASRLNIERMKQIYQQLSCYRGLGLLATVPSSSNGNG
NGNAETSSDAMRFSETSRDAAAPSTYQMPVHSQGEWQSGVMMYLQGDPQTSAGMHQHGPEHHPPTQTSGKRHS
EDSGNGKSSKVQRQSIH
>Archocentrus_cent__XP_030577901.1
MFLCIVFVIHMFAGMAHTCIHWFRKGLRLHDNPALMAALKDCKKLYPVFILDPYFQNNTCVGINRWRFLIGAL
KDLDCSLRKHNSRLFVVRGRPEDVFPKLFTKWKVTRLTYEYDTEPYSLQRDSKVTSLAKEHGVEVLYKVSHTL
YNIDRIIEENSGKPPLTYKHLQAIVKTIGPPKRPVSAPTMDDMKENHEKEYGIPALKELGLDTTTLGEELFPG
GEQEALRRLDEHMKRTKWVCTFEKPQTSPNSLSPSTTVLSPYVTFGCLSVRTFWWRLTEVYKGNKHSDPPVSL
HGQLLWREFFYTASLGIPNFNKMEGNSVCTQVDWDTNPEYLAAWSEARTGYPFIDAIMTQLRQEGWIHHLARH
AVACFLTRGDLWISWEEGQKVFEELLLDGDWALNAGNWQWLSASTFFHQYFRVYSPVAFGKKTDKNGDYIRKY
LPLLKKFPAEYIYEPWKAPRRIQEVAGCIVGKDYPHPIVQHEVISKKNIQRMKLAYAKRSPDTTESPNKKQGV
KRKAPSVIDMFKKKAKV
>Archocentrus_cent__XP_030586440.1
MPPLGADAQATVRRWLREVLVGREDPEGFFAVCVSILGHQETRSQFLSLIQPLSTANRLLHSSLTSIYQEYFI
KTEEDELELALALSLLDMKDQPLQSPNQESQPLQSGDAQDQSGSVQLNPASKSEGSNCTNQTGTWKRERQLQT
TGNTEVQINTYPNKEIPGTGQTVRVSEEAQDLDQSQKPKRSKNRRQRRKCTGQTLVGLPCSPSALPPVLLWFR
RDLRLCDNPALMASLEVGAPVIPVFIWSPEEEEGRGLTVAMGGACKYWLHQALLCFCSSLEHIGSHLVFLKAN
TEGDGVRSSLEALKELIKESGARTVLANALYEPWLKERDDAVVSTLQKQGVECRMFHSYCLRDPYSVTTEGVG
LRGIGSVSHFMNCCRQNPGSTVGVPLEPPVSLPTPAHWPKGVSLDELDLARMPRRKDGTMIDWAVNIRKSWDF
SEEGAHARLKAFLHDGVYRYERESGRADAPNTSCLSPYLHFGQLSPRWLLWDAKGACCRPPKFQRKLAWRDLA
YWQLTLFPDLPWESIRPPYKALRWSSDRGHLKAWQRGRTGYPLVDAAMRQLWQTGWMNNYMRHVVASFLIAYL
HQPWQEGYRWFQDTLVDADVAIDAMMWQNGGMCGLDHWNFVMHPVDAALTCDPYGTYVRKWCPELADLPDELI
HKPWKCPASMLRRAGVVFGQTYPERVITDLEERRNRSLQDVALVRREFEQYVDKHSGCDLVPLPPRLVSEALS
LSHRDGALVTQGKQFLLPVITRMEFKHQQDDPDADAASNPYNAVLKGYVSRKRDETIAFLNERDFTASVMHEV
VQRKERLNSDYRKMEGLPSPSPRGRARRTPTAKDRFSIVPGGVATSLK
>Archocentrus_cent__XP_030586668.1
MVVNSVHWFRKGLRLHDNPALQEALNGADTVRCVYILDPWFAGSANVGINRWRFLLEALEDLDSSLKKLNSRL
FVVRGQPTDVFPRLFKEWNVTRLTFEYDPEPYGKERDGAIIKMAQEFGVETIVRNSHTLYNLDSRIIEMNNNS
PPLTFKRFQTIVSRLELPRRPLPLVTQEQMDKCCTKIADNHDQLYSIPSLEELGFRTDGLRPAVWRGGESEAL
DRLSKHLDKKVWVTSLENPRVNTCSLYANPTGLSPYLRFGCLSCRVLYYNLRELYMKVRKRCSPPLSLFGQLL
WREFFYTAATNNPNFDHMDGNPICVQIPWDQNPEALAKWAEGRTGFPWIDAIMTQLRQEGWIHHLARHAVACF
LTRGDLWISWESGMKVFEELLLDADWSVNAGSWMWLSCSAFFQQFFHCYCPVGFGRRTDPSGDYIRHYVPILK
DYPNRYIYEPWNAPESLQKAANCVVGVDYPKPMINHAESSRLNIERMKQVYQQLSHYRGLSLLASVPTIQEEA
EPPMTDESQTSSGPDSPLRVPADREAAGCSEAPDSFTVCTSSTSVQHPEQADQAPCISSGSHAEPIHPPSLSS
TLALSPLSKSKLSSPSSSCPGPSTALAQSSSLGPKRKGLNRKVRRGHRQRGRQYCTLAAREREATGGEEKMEE
DVEQNKDRMEDDDQTSGETARQLQ
>Archocentrus_cent__XP_030608567.1
MCCLFGYVHVLFSIRAEAALFMALHPVCCAAFGGRFVKLAIHHHQRPSSPRLFFLTSLFTCQPTMSDKKRKAS
CPSAGKEPRAKQQKLAPVKEEKKERVAGWLQSLVQQQRTEKDIKFNKKRLRFISDTQKIRQGSEGVLYWMLRD
QRVQDNWALIHAQLLAAEENLPLHICFCLEVPKSELSTQRHYSFMLKGLKEVAKECKHLNIQFHLLHGAPGNV
LPGFASDHSLGAVVTDFSPLREPLQWLEDVKKKLPQDIPFIQVDAHNIVPCWVASPKLEYSARTIRGKITNLL
SEFLTDFPLVEKHPYTAAGTAKEVDWDKTLASLEVDRTVEETEWAKPGTKAGMDMLESFIDVRLKLFGSQRND
PNAPALSQLSPWLRFGQLSAQRVALQVQRSGKNAGQSIPAFIEELVVRRELTDNFCFYNKKYDSVEGAYEWAQ
KTLKDHAKDKRPYLYTREQLEKAKTHDKLWNAAQYQMVTEGKMHGFLRMYWAKKILEWTSSPQEALSIALYLN
DRYELDGQDPNGFVGCMWSICGIHDQGWAERPVFGKIRYMNYKGCLRKFNVAQFEKTYCPKNL
>Archocentrus_cent__XP_030612430.1
MSTSRTVICLLRNDLRVHDNELFHWAQRNAEYIVPLYCFDPRHYVGTYNYNLPKTGPFRLRFLLESIRDLRNT
LINKGSNLVVRLGKPEDVVADLIKQLGSVSSVAFHEEVTSEELNVEKKVKEVCAQMKVRVHTCWGSTLYHRDD
LPFPHMSRLPDVYTQFRKAVESEGRVRPVFPTPEKLNPLPPGLEEGPIPIAEDLQQTEPVTDPRSAFPCSGGE

SHALTRLKHYFWDTDAVAAYKETRNGLIGVDYSTKFSPWLAMGSISPRYIYHQIKQYEKERTANQSTYWVIFE
LLWRDYFRFVGVKYGNRIFQVKGLQDKSVPWKKEMNLFDAWKEGRTGVPFVDANMRELATTGFMSNRGRQNVA
SFLTKDLGLDWRMGAEWFESLLIDHDVCSNYGNWLYSAGIGNDPRENRKFNMIKQGLDYDSNGDYVRRWVPEL
QSIRGGDVHTPWALSAAALSHAHVTLGGTYPTPIVMAPEWSRHISKKVGATGPSPRGKKGPSHTPKQHRDRGI
DFYFSRSKNL
>Armadillidium_nasa__KAB7496070.1
MTGTSLKGKPKHVVHWFRKGLRIHDNPSLRLGLKNSTTFRCIFILDPWFAGSSNVGINRWRFLLQSLEDLDRS
LRKFNSRLFVIRGQPAKALPDIFREWGTNILTFEEDPEPFGKARDANIIAMAREMGIKVIVRTSHTLYKLDSE
INPLPFFITLFFLFLISYIKIGVIILIFLFLGFDTDHLHAPVWKGGEIEALERLEYHLERKAWVASFGKPKMT
PQSLYASPTGLSPYLRFGCLSARQFFLKLNDLFQKIKKLPPPLSIHGQLLWREFYYCAATNNPKFDHMEGNPI
CVQIPWDKNPEALAKWANGQTGYPWIDAIMTQLRQEGWVHNVARHAVACFLTRGDLWVSWEEGMKVFDELLLD
ADWAVNAGSWMWLSCSSFFQQFFHCYCPVRYGRKADPNGDYIRTYLPILKNFPTKYIHEPWTAPESIQRAAKC
IIGKDYPMPMVDHIKQSQHNIERMKQVYQQLTHYRGSINSGSKSGKENNNLLPPSSDFEKIHQRRKMKDFKRE
TNQTVSIHA
>Armadillidium_nasa__KAB7504233.1
MKMLSVDFLLGFISVRQISAQRCILEVNRYRQTYPESVDAYFEECIVRRELADNFCFYQTNYDNINGAADWAR
NTLEIHKKDVRPYLYSKKTLEKAKTHDDLWNSAQIQLIKEGKMHGALRMYWAKKILEWTKTPEEALSVAMYLN
DRYSLDGRDPSGYVGCMISICGIHDQMSWLERDIFGKIRFMNYEGCKRKYNVKTFVARHGGKAYKNTSNSNKH
FSMTSKKRKGGLEGSSKKIKLIDETFVEKLESERNEVARSVTDFSFNKNRVRMLSKQLHIPEKCDGVVYWMSR
DQRVQGLQEVESDCQTLNISFHLLLGQAKEVLPKFVKEENMGGVVTDFAPLRLPLQWVKDIENSLPGNVPFGQ
VDAHNVVPCWVTSSKQEYAARTIRSKINKNLKEFLTKFPAVIKHPHSSKVKYKIGNQLTSCLKLDKTVGPVEW
AKPGSKEGLKMLHEFCIKRLKMFANKRNDPNENALSGLSPWIHFGQISAQRCILEVKEYRERFPESVDAYIEE
CIVRRELADNFCFYQTNYDNINGASDWAKNSLEIHKKDVRTYIYNKKTLDEAKTHDDLWNSAQIQLVKEGKMH
GFLRMYWAKKILEWTKTPEEALAIAIYLNDRYNLDGRDPNGYVGCMWSICGIHDQGWAERSIFGKIRYMNYEG
CKRKFDVKAFVSRYGGKVYTNTSISQKTREVVLSPFKLF
>Armadillidium_vul_RXG59456.1
MGGVVTDFSPLRLPLQWVEDVEKSLPEDVPFGQVDGHNIIPCWVTSTKQEFTARTIRFKINNNLKEFLTKFPA
VIKHPYSTKMKYKNINWESAYQMQKMDKTVGPVEWAKPGSKAGLKMLHEFCIKRLKLFAKKKNDPNENALSGL
SPWIHFGQISAQRCVLEVNRYRQTYPESVDAYFEECIVRRELADNFCFYQTNYDNINGAAEWARKTLEIHKKD
VRPYLYSKKTLEKAKTHDDLWNSAQIQLIKEGKMHGALRMYWAKKILEWNKSPEEALSVSMYLNDRYSLDGRD
PSGYVGCMISICGIHDQMSWLERNIFGKIRFMNYEGCKRKYNVKTFVARYGGKAYKYTSN
>Armadillidium_vul_RXG60369.1_cry-2
MTGTSLKGKPKHVVHWFRKGLRIHDNPSLRLGLKNSTTFRCIFILDPWFAGSSNVGINRWRFLLQSLEDLDRS
LRKFNSRLFVIRGQPAKALPDIFREWGTNILTFEEDPEPFGKARDANIIAMAREMGIKVIVRTSHTLYKLDRI
IEKNGGKPPLTYKSFQNILSQIGPPPLPDPAITPEEINGGVSPISEDHDDKYGVPTLEDLGFDTDHLQAPVWK
GGEIEALERLEHHLERKAWVASFGKPKMTPQSLYASPTGLSPYLRFGCLSARQFFLKLNDLFQKGQTGYPWID
AIMTQLRQEGWVHNVARHAVALFSYKRRSLGVVFDELLLDADWAVNAGSWMWLSCSSFFQQFFHCYCPVRYGR
KADPNGDYIRTYLPILKNFPTKYIHEPWTAPESIQRAAKCIIGKDYPMPMVDHIKQSQHNIERMKQVYQQLTH
YRGSINSGSKSGKENSNLFPPSSDFEKIHQRRKMKDFKRETNQTVSIHA
>Armillaria_ost_SJL01787.1_uncharacte
MQKRVHSLSTSPPASAKKSRTTGSSSTFKPNKIATQEAAAAVDANPPFPVLLKAVKDGLKDPVKGKSVVYWMR
MGDLRKCTKVYANMEYEVDEMRRDVKVCSLAKQEGINATFVHDKCIVDPGAIVSAENKPYVVYSAYQRRWLVN
LNDNLPYYLEDCAPPKPNSDSIQSSRYSNLFDSSIPTSVEGFELDDPIKEKMAFIWPAGEEKALSMLDRFLKT
KTRSSQMGSTDPLASGEEESKPSSRIVKYDTRRDAADSDTTSRLSPYLSAGVISARTCVRATMELLGVNKVDG
GKTTGIGRWVQELAWRDFYINLVVAFPRVSMGRPWLEKYAAVVWEAHQSSGKGERSKDSDSELLQAWKNGMTG
YPIVDAAMRCIQEMGWLHNRLRMITAMFLTKNLMFDWRVGERYFMENLIDGDLASNNGGWQWCASTGVDPCPY
FRIFNPYNQSVKADPKGDFIRQFVPELASIKTSEIHNPSAKTARALGYPLPIVDYKETRQRALRRFKTPGEM
>Arthrospira_pla_WP_014276518.1_photolyase
MSDLILFWHRRDLRISDNWGLAAARRVTSQVVGVFCLDPEILSGDDIAPVRVAYMLGCLQCLQEDYQRGGSQL
LIIQDNPAQGLRKLAVALGAIAVYWNQDVEPLPRQRDRSVEESLTEVGIKVETFWDQVLHSPEAIFTGSKEPY
KVYTPYWRKWIQQPKPEPCQRLKSTGLTEAQEKAALEAGAIALPTAKDLGFIWSEPFILEPGETAASDRLEIF
CDRAIYEYDEGRNFPAIDGTSVLSPALRFGAIGIRTLWQKTQEIIALTRSDEARHHIQTWQQELAWREFYQQA
LYHFPALAIGAFRQPFDRFPWSDNQEHFQAWCQGRTGYPIVDAAMRQLNQTGWMHNRCRMIVASFLTKDLIIN
WQWGEKYFMQTLIDGDLASNNGGWQWSSSSGMDPKPLRIFNPASQTQKYDPEAEYIRQWLPELRNLDTAHLVT
GKISSGDRQACGYPQPIVDHNRQQKLFKSIYSQLKA
>Artibeus_jama__XP_037016316.1
MGVNAVHWFRKGLRLHDNPALKECIQGADTIRCVYILDPWFAGSSNVGINRWRFLLQCLEDLDANLRKLNSRL
FVIRGQPADVFPRLFKEWNITKLSIEYDSEPFGKERDAAIKKLAMEAGVEVIVRISHTLYDLDKIIELNGGQP
PLTYKRFQTLISKMEPLEIPVETITSEAIEKCTTPLSDDHDEKYGVPSLEELGFDTDGLPSAVWPGGETEALT
RLERHLERKAWVANFERPRMNANSLLASPTGLSPYLRFGCLSCRLFYFKLTDLYKKVKKNSSPPLSLYGQLLW

REFFYTAATNNPRFDKMEGNPICVQIPWDKNPEALAKWAEGRTGFPWIDAIMTQLRQEGWIHHLARHAVACFL
TRGDLWISWEEGMKVFEELLLDADWSINAGSWMWLSCSSFFQQFFHCYCPVGFGRRTDPNGDYIRRYLPILRG
FPAKYIYDPWNAPEGIQKVAKCLIGVNYPKPMVNHAEASRLNIERMKQIYQQLSRYRGLGLLASVPSNPNGNG
GLMGYSPGENIPGCSSSGRRSSMGTGLGSGKRPSQEEDTQGIGPKVQRQSTH
>Artibeus_jama__XP_037022087.1
MAATAGTAAAAAPAPASGAGTDGASSVHWFRKGLRLHDNPALLAAVRGARCVRCVYILDPWFAASSSVGINRW
RFLLQSLEDLDTSLRKLNSRLFVVRGQPADVFPRLFKEWGVTRLTFEYDSEPFGKERDAAIMKMAKEAGVEVV
TENSHTLYDLDKIIELNGQKPPLTYKRFQAIISRMELPRKPVGSVTSQQMERCPAEIQENHDETYGVPSLEEL
GFPTEGLGPAVWQGGETEALARLDKHLERKAWVANYERPRMNANSLLASPTGLSPYLRFGCLSCRLFYYRLWD
LYKKVKRNSTPPLSLFGQLLWREFFYTAATNNPRFDRMEGNPICIQIPWDRNPEALAKWAEGKTGFPWIDAIM
TQLRQEGWIHHLARHAVACFLTRGDLWVSWESGVRVFDELLLDADFSVNAGSWMWLSCSAFFQQFFHCYCPVG
FGRRTDPSGDYIRRYLPKLKGFPSRYIYEPWNAPESIQKAAKCIIGVDYPRPIVNHAETSRLNIERMKQIYQQ
LSRYRGLCLLASVPSCMEDLSNPVAEPSSTQTGSTSSAGPRPLPSGPASPKRKLEAAEEPPGEELSKRARVAE
LPAPELPSRDV
>Asplenium_yun_AGI61039.1_blue-light
RTTKLCTIVWFRRDLRLEDNPALSVAARAGAVVPVFIWSPEEEGQFHPGRVSRWWLKESLIQLDISLRSLGAF
LVLRKTHDTLSTLLEIVKTTGATQVVYNHLYDPISLVRDHRVKQGLTENGVQVQSFNGDLLYEPWEVLDDHGK
PFTFFDSYWTKCLSMPFEPDSPLLPPRRIMALSGSIPSLTPEELGLEDETEKSSNALLARAWAPGWSNADRAL
EAFLAGPLLEYSRNRHKIDGPTTSLLSPHMHFGEVSVRKVYHSVRRLQILWAKDGSKLGEESINIFMRSIGFR
EYSRYLSFNFPFTHERSLLSNLKSFPWRVDETFFKVWRQGRTGYPLVDAGMRELWATGWLHNRVRVIVSSFCV
KFLQLPWRWGMKYFWDTLLDADLESDVLGWQYISGSLPDGHELDRMDNPQIEGYKHDPLGEYVRRWLPELVRL
PTEWIHHPWDAPPSVLRAAGVELGSNYPRPIVEVMAARERLQQAVSQMWEREAALQAAGVGGDGEALDADLQA
KVVTQPLVSAQLANSVVVGQCDPGSQGVQLPSRSSYRDQMVPSHAKVLEARTDLPREGGLNSEILMSSQQDNL
PPSTPPPETRNISQPSPDSCGKPNVNHDQAAARPACSEPPPPVSDANSTAESSSSPRRPIVEMDKRHDAPTVW
VPAITHQRPSMDFGCVPYAGDAGQLRKHLLSLQNSRPVNQ
>Asterias_rub_XP_033627319.1_cry_DASH-l
MSTKLKTIICLLRNDLRYHDNEVLHWAHLNADFVVPLYCLDPRHYSGTWHYSFPKTGPHRLKFLLQTLHDLKD
SLKSVGSELVVSRGEPEKVITNLISQLGKENVKAVALQQEATREETDVERAVEKSCQVPVHKVWGSTLYHKED
IPFRIPQLPNVFTEFRKRTENQSRVRPTLSMPSRLKPLPTGVDPGCIPTMEEYGMKEPAQDPRTAFPYGGGER
QALARIQHYFWDHDYIASYKETRNGLIGPDYSTKFSSWLALGCLSPRKIYEEIKRYEKERKTNQSTYWVIFEL
LWRDYFKFVALKFGDRLFYLSGLLGKHREWQENQKLFDAWREGKTGVPFVDANMRELATTGFMSNRGRQNVAS
FLTKDLKLDWRLGAEWFEHLLIDHDVCSNYGNWLYCAGVGTDPRPARKFNMVKQGLDYDAQGDYIRSWIPELQ
AIKGGAIHTPWALSSSMLMKCEVTLGHTYPMPVVKAPEWAKHIRTQERGKSGRGPEQRQQTRGVDFYFSSSSQ
KYKK
>Asterias_rub_XP_033628662.1_cry-1-like
MNPSVRLSPRKNNERKMGKRKRTKGSSRRGVSLVHWFRNKGLRLDDNPALKEALVGAKTFRCVYILDPWFARS
PNQGVNKWRFLLQCLVDLDSSLRKLNSRLFLIRGQPSDVFPKLFKEWGVNRLSFEEDPEVFGKQRDAKIKTLA
ATFQVQVITRLSHTLYDPKEILSVNSNSPPLTYKHFQYLISLLDPPPFPEDTITLADIEGVNTAIMSDHDSKY
GLSSLEELGFDTEDLQPPVWIGGETVAKQRLDRHLERKAWVANFERPRMSSASLMTSPTGLGPYLRFGCLSPR
LFYWRLTELYCKMKKTTDIPLYLHGQLYWREFFFTLGYNNAKLDQMIDNPICIPIPWEENDKKLQQWRNGETG
FPWIDAIMIQLRQEGWIHPIARHAVACFLTRGDLWISWEEGAKVFEDLLLDADWSVNAGNWIWLSCSSFHQQF
FHCYKFCPVMFSQQTDPNGDYVRKYIPALKNFSAKYIYNPWTAPLDVQEKAKCVIGKDYPLPMVNHDKVSRIN
SDRMNKILQGVARKPSYDESFTASTLHTKVQNRHRIPNMQKEPPRKLARLTQDHLSTHPNNYMPAQDDLTAGT
SSYMQTQDDLTTGNNSYMPTQDELSVGTNSYLQTQDELTAGNNSYMQTQDELTAGTMSYMPTRDELTAGNNSY
MATQNEQSAGNNSYMPTQDELTVGTNSYLQSQDDLTAGSNSYMSADDELTAGTNSYLPATYQSSIHPVQDTYS
IQDAFTMPQDNLLEQDPRQNSPWDDMTRGILHAHVQTTLPGNNWQ
>Asterias_rub_XP_033629349.1_cry-1-like
MPSKPKSGLDLRKTTIAVHWFRHGLRLHDNPSLLDAINLGDELYPIFIFDGEVAGTKVCGFNRWRFLYESLED
IDRRLKEFGGRLYTFKGDPCTIFSQLIEEWGMNLLTFEQDPEPIWQDRDNAVKRLCEERGIKCIERVSHTLWD
PNEVISRNGGSPPVTYAMFQEVVSMIGLPHRANDEPDFRGVKLPICEETFSTYMLPPFEDFGVKMEHVEQERQ
LWIGGETRALELFEVRIRKEAAAFKAGYCLPNQYMPELLGPPMSLSPYLRFGCLSVRRFYWRIHDTYTELNNV
ISPTQLTAQLIWREYFYTMSIGNKNFDQMNGNNICLKIDWSEDQEKLQKWTNGQTGYPWIDACMKQLKQEGWI
HQVARHAVACFLTRGDLWISWEEGLKVFFQYLLDADWSICAGNWMWISSSAFEKVLQCPNCFCPVRYGRRMDP
TGEFVRNFLPVLRNMPLRYLFEPWKAPLAIQEKSGCIIGKDYPEPIVEHRIAASSNREKMNRTVARLEDKFSY
CTPSSKTEVLEFSWLPETARGVDKCTADDLCEILGI
>Asterias_rub_XP_033643654.1_cry-1-like
MAKHQRSLHWFRKGLRLHDNPSLLAAVKGSKEFRAVFILDPFFVKSSNIGINRWRFLMESLRDLDDGLKQLGS
RLYVLRGQPSEVFPKLFTQWKVTRLTFESDTEPYAVKRDDLVKTIAEEHGVEIIQKMSHTLYHPQSVIKANGG
TPPLTYQRLLTVLSKMTLPKQPEPALSKECIGGCALPVGSDHESEFGLPSLEELGLDPSNAGPHLFPGGETEG
LRRMKEQLARQDWVCKFEKPKTEPNTLKPSTTVLSPYLKFGCVSARAFYHGVQDVYTRKKTHSKPPVSLHGQL

LWREFYYIAAAGTPNFDKMEGNSVCLQVPWDTNEELLQAWKQGRTGYPFIDAIMIQLKQEGWIHHLARHAVAC
FLTRGDLWISWEEGQKVFDKWLLDADWSLNAGNWMWLSASAFFHQYFRVYSPIAFGKKTDKNGDYIRKYLPVL
KKMPTQYIYEPWIAPRSVQEKAGCLVGTDYPRPIVVHSDVMKRNMARMKQARATKYGDKSNADTTKEAMTDKK
KEGSKKRKTKDTDKTSTSKKSKTLTDFY
>Astyanax_mexi__KAG9263125.1
MKHSCIHWFRKGLRLHDNPALKYALHDCKHMYPVFILDPWFPKNAQVGINRWRFLIEALKDLDSSLRKLNSRL
FVIRGVPREVFPRLFEEWKVTRLTFEVDSEPYSQKRDKEVMDLAKQHGVEIITKISHTLYNLERVIEENNGKA
PLTYVRFQTVLNKIGPPKRPVPAPTREDMKGVSTPSSETDKENYGVPTLDELGQDSTTLGPVLYPGGEQEALR
RLDEYMQRTNWVCSFEKPQTSPNALKPSTTVLSPYVKFGCLSARTFWWRLAEVYHGKKHSAPPVSLHGQLLWR
EFFYTAAYGIPNFNKMEGNTICVQVDWDSNPEHLAAWREARTGFPFIDAIMTQLRQEGWIHHLARHAVACFLT
RGDLWISWEEGQKVFEELLLDADWALNAGNWQWLSASAFFHQYFRVYSPIAFGKKTDKNGDYIKKYLPILKKF
PAQYIYEPWKAPKSIQEQAGCIVGKDYPRPIVDHDTISKKNIQRMKAAYAKRSPDSSDGSAERKGQKRKSASI
MEMLTKKAKK
>Astyanax_mexi__KAG9272970.1
MSVSAMSEVRLKLRDLLLGLEDAEGFFCLCLSALGERDTLALFLDLIKPLASGHVQLYSQLSAIHTNYFSRDE
EEALEVAIALSLHDTNQRSFDQLAERGSENREPGDPEKLIQKGCTLKHIRPQRDSTDTYTSGPSLVVDDVPLK
NEDRGHLTKPKKSRRHRKKQQMLKNPSAPRPVLLWLRRDLRMWDNPALIGCLELGAPIIPVFLWNATEEEGSG
LTKAAGGASKYWMHQALVCLNRSLEERGSHLVTQRAEPSSLTALQALVAETGAGTVVATALYEPWLQERDEAV
WATLEKQGVKCHIYHSYCLRDPNTVSTRGVGLRGIGSVSHFISCCNQNPGPGIGAPLDAPGALPSPSSWPQGC
PLTQLELALMPVRKDGTVIDWAANIRQAWDFSEEGAQARLETFLKDGVYRYEKESCRADVPNTSCLSPYLHFG
QLSVRSLLWDARDARCRPQKFQRKFAWRDLAYWQLCLFPDLPWESLRPPYKALPWNSDRAHLKAWQRGRTGYP
LVDAAMRQLWLTGWMNNYMRHVAASFLIAYLHIAWQEGYRWFQDTLVDADVAIDAMMWQNGGMCGLDHWNFVM
HPVDAALTCDPNGNYVRQWCPELKGLPDDFIHKPWMCPGSVLRRAGVILGQNYPERVVVELEERRAQSLQDVR
MVRKRFSEYVCQQTGCDLLPLPTKLVQEALGSTDDLVQRGGNEFLLPVITRMEFRLEPEDTASHSAVLKGYVS
RKRDEKIAFLNKCDFTASVMYENAQRRERMEKNHCMLEDLQCPPTNRGRGRHRNSRPVSGAAQQR
>Astyanax_mexi__KAG9273094.1
MVVNTIHWFRKGLRLHDNPSLLDSIAGADSLRCVYILDPWFAPHVSRPGTGHMDLVLMFLSLSFYRFLLQCLE
DLDASLRKLNSRLFVIRGQPTDVFPRLFKDWKITRLSYEYDSEPFGKERDAAIKKLASEAGVEVTVRISHTLY
DLDKIIELNGGQSPLTYKRFQALISTMDAVETPAETITAEIMGRCATPISDDHDEKFGVLSLEELGFETEGLS
SAVWPGGESEALTRLERHLERKAWVANFERPRMNANSLLASPTGLSPYLRFGCLSCRHFYFKLTDLYRKVKKN
SVPPLSLYGKLLWREFFYTAATNNPCFDKMEGNPICVQIPWDRNPEALAKWAEGRTGFPWIDAIMTQLRQEGW
IHHLARHAIACFLTRGDLWISWEEGMKVFEELLLDADWSVNAGSWMWLSCSSFFQQFFHCYCPVGFGRRTDPN
GDYIRRYLPILRGFPAKYIYDPWNAPEGVQKLAKCIIGVHYPKPMVNHAEASRINIERMKQIYQQLTCYRGLG
LLASVPSNSSGNGDNGGAGSGDSTQEGSAAVHLTPANPREDFSANTSDQQPAQQQIGFSGAGRAGSHRQDSKQ
ARGRGTGTMKRHSEDPASSNGCKIQRQSCN
>Astyanax_mexi__KAG9273647.1
MHCFVVAAKRTLNIFQTAAKLPRAAGLHALLDGRAASMSAKKAELKRRGGSGQAEGAGGKKQRSGESAAAAGG
REDGWLGLEVAELRAKNTGCKFNDKRVRFLSEEQKVKQSCSGVLYWMSRDQRVQDNWALIYAQQLALAEELPL
HICFCLVPRYLDAAYRQYAFMIRGLQEVAKECKSLDIQFHFLRGDPEQLLLDFVKSWNIGALVTDFNPLRLHL
QWIENVRKGLPSNIPFLQVDAHNVVPCWEASPKLEYGARTIRGKITKQLPDFLTEFPPVDTHPHASKKTAKSV
NWEEVLDSVEVDRTVGEVEWARPGTSGGMAMLESFIQQRLRLFATERNNPNSEAVSHLSPWLRAGQLSAQRVV
KEVQRWGKNARESVASFTEELVVRRELADNFCYYNKEYDSIAGAYDWAKTTLKIHAKDKRAYLYTQEQLETGK
THDQLWNAAQRQLLLEGKMHGFMRMYWAKKILEWTSSPEEALTIALYLNDHYSLDGCDPNGYVGCMWSICGIH
DQGWAERPVFGKVRYMNYAGCKRKFDVSRFERKYAVKTD
>Astyanax_mexi__KAG9283299.1
MASPRTVVCLLRNDLRLHDNEVFHWALRNADHIVPLYCFDPRHYLGTQHFNFPFRLRFLLDSVKDLRATLKKT
GSSLIVRRGKPEEVVNDLIKQLGSVSTVVFHEEVTSEEKQVEKKLKHVCFQNKVKIQTFWGSTLYHRDDLPFR
HIGGLPDVYTAFRKAVEERSGVRPVLPSADHIRPLPPGLEEGPIPTFEDLEQTEPLEDPRSAFPCRGGESEAL
TRLQHYFWDTNAVATYKETRNGLIGVEYSTKFAPWLAVGCISPRYIYEQIKRYEAERTANQSTYWVIFELLWR
DYFRFVALKYGNRIFHLNGLQDKHVPWKTDMDLFNAWKEGRTGVPFVDANMRELFLTGFMSNRGRQNVASFLT
KDLGLDWRLGAEWFEYLLVDYDVCSNYGNWLYSAGVGNDPRENRKFNMIKQALDYDSNGDYVRQWVPELKGIT
GGDIHAPWTLSSASLSHSGVELDNTYPTPIIMAPEWSRHINQKPSGAMSSRGKKRGPSHTPRQHRDRGIDFYF
SKSKNL
>Athene_cuni__XP_026705138.1
MPSAATGPVVAPAPALCRSVHWFRRGLRLHDNPALQEALRDASSLRCIYILDPWFAASSAVGINRWRFLLQSL
EDLDNSLRKLNSRLFVVRGQPTDVFPRLFKEWGVTRLTFEYDSEPFGKERDAAIVKLAKEAGVEVVIENSHTL
YDLDRIIELNGHKPPLTYKRFQAIISRMELPKKPVSTVISQQMETCKVDIQENHDDVYGVPSLEELGFPTDGL
APAVWQGGETEALARLDKHLERKAWVANYERPRMNANSLLASPTGLSPYLRFGCLSCRLFYYRLWELYKKVKR
NSTPPLSLYGQLLWREFFYTAATNNPKFDRMEGNPICIQIPWDRNPEALAKWAEGKTGFPWIDAIMTQLRQEG
WIHHLARHAVACFLTRGDLWISWESGVRVFDELLLDADFSVNAGSWMWLSCSAFFQQFFHCYCPVGFGRRTDP

SGDYVKRYLPKLKGFPSRYIYEPWNAPESVQKAAKCIIGVDYPKPMVNHAETSRLNIERMKQIYQQLSRYRGL
CLLASVPSCVEDLSGPVTDSASGQGSSTSTAMRLSQADQASPKRKHEGAEELCTEELYKRAKVTGLPAPEIPG
KSL
>Athene_cuni__XP_026710518.1
MRRGQGKRKADAPELPRVSQRRKEEEEEMREARRRAAPSVREFKYNKKRVRLVSQGSDLKDDARCILYWMSRD
QRVQDNWAFLYAQRLALKQELPLHICFCLVPKFLDATIRHYDFMLRGLQEVAEECSELNIPFHLLLGYAKDVL
PTFVVEHGVGGLVTDFCPLRLPRQWVEDVRERLPDDVPFAQVDAHNIVPCWVASPKQEYSARTIRGKIHAQLP
EFLTEFPPVIRHPYPPSCPAEPIAWEACYSSLQVDRTVKEVEWATPGTATGLSVLQSFIAERLKSFGSHRNDP
NKAALSNLSPWFHFGQVSTQRAILEVQKHRGKYKESVDAFIEEAVVRRELAENFCYYNENYDSVQGAYDWAQT
TLKLHAKDKRPFLYKLQELEQGATHDPLWNAAQLQMVREGKMHGFLRMYWAKKILEWTRSPEEALQFAIYLND
RYELDGRDPNGYVGCLWSICGIHDQGWAERAIFGKIRYMNYAGCKRKFDVGQFERRYAPHTPSQ
>Athene_cuni__XP_026720170.1
MPRHTVHLFWKGLWLLDKPTLLAVMESSVIYPVCILDRKFMMSMMHTGALRWHFLLRSLEVLQKNLWELGSCL
LVIQGESVPRDHIHKWNIMQVALDAEMEPFCKEMAANVRCLGDELGFEVLSLVGHSLCDTKRYVIVLKTSEVS
PGRFISDLTSIGEKRSLCRRPLFHIRRCRAPDPGLAECYRVPLPVDLKIPPKSLSPWRGDETQGLQRLEEHLT
DQGWVASSTKPRTVLNSLLPNTTGLSPYFSMGCLSVRNFFYRLSNIYAQAKHHSLPPVWLQGQLLWREPCGVA
SAIPNFTRMVGNPICLQVSWYEDAERLHKQRKTGIPWIDVVMTPLCQEGWIHHLARHTVXFLLRRDLWISWEE
GMKVFEELLLDADYSISTRNWMWMSASAFFHQVXIFCPVHFGKHMDPKGQDLWKYLPILKNFPSKYISEDXTA
SEEEQKQAVCITGQDYPFPLVSHGEXSDHAARLRKRVGAEQRXLVNFDSFLLAVDDTDDPMEMKVKHELCEEN
ISKGKMARMTEQTEILSGITCWEPKHGPRGKPEAS
>Athene_cuni__XP_026721435.1
MEPLEMPVETITPEVMEKCTTPVSDDHDEKYGVPSLEELGFDTDGLPSAVWPGGETEALTRLERHLERKAWVA
NFERPRMNANSLLASPTGLSPYLRFGCLSCRLFYFKLTDLYKKVKKNSSPPLSLYGQLLWREFFYTAATNNPR
FDKMEGNPICVQIPWDKNPEALAKWAEGRTGFPWIDAIMTQLRQEGWIHHLARHAVACFLTRGDLWISWEEGM
KVFEELLLDADWSVNAGSWMWLSCSSFFQQFFHCYCPVGFGRRTDPNGDYIRRYLPVLRGFPAKYIYDPWNAP
ESIQKAAKCIIGVNYPKPMVNHAEASRLNIERMKQIYQQLSRYRGLGLLATVPSNPNGNGNGGLMGYSPGESI
SGCGSTGGAQLGAGDGHTVVQPCALGETHTGASGIQQQGYCQASSILHYAHGDNQQSHLLQAGRTALGTGISA
GKRPNPEEETQSVSGPKVQRQSTN
>Atractosteus_spat__MBN3317673.1
MEALSNLSPWLHFGQVSAQRVVLEVRRYGRRWPQSVDVFVEEVVVRRELADNFCFYNKKYDRVDGAYEWAQKT
LREHAKDRRPYLYSRGELEAFRTHDKLWNAAQFQMVSEGKMHGFLRMYWAKKILEWTASPEEALATAIYLNDR
YELDGRDPNGYVGCMWSICGIHDQGWKEREIFGKVRYMNYAGCTRKFNVPKFEIKYNPKRCDDEDGNRKRGGD
>Atractosteus_spat__MBN3318031.1
MMHRSIHWFRKGLRLHDNPALLASLRDCAELWPVFLLDPWFPKNARVSVNRWRFLLRALQDLDGNLRKLGSRL
FVVRGSPAEVFPRLFEQWKVTRLTFEVDTEPYARQRDAQVGKIAEEHGVEVIQKVSHTLYDTERILVENNGKA
PLTYNRLQALLRTIGAPKRPVPPPTMEDMKGVCTPCLERHDEEFGVPTLEELGQDPRTAGPELYPGGETEALS
RLDRHMQRTAWVCGFQKPNTEPNALSPSTTVLSPYLKFGCLSARTFWWRLTDVYRGRKHSEPPVSLHGQLLWR
EFFYTAGLGIPNFDRMEGNPVCVQIDWDENAEHLAAWREQCQRCSRRAKKVLSLHGQYSRAVGSLWLVTSSAA
ALFCPTSVRVCVSLRVCVVFEELLLDADWALNAGNWQWLSASAFFHQYYRVYSPIAFGKKTDKNGDYIRKYLP
VLKKFPSAYIYEPWKAPRSVQEQAGCIVGKDYPRPIVDHDVVSKKNIQRMKLAYGRRAQLSGEQEGTGKGMKR
KGQSVADLLTKKQKRDSVEEKRTLSG
>Atractosteus_spat__MBN3321972.1
MVVNTVHWFRKGLRLHDSPALRESLKGAASLRCVYILDPWFAGSSNVGINRWRFLLQCLEDLDASLRKLNSRL
FVIRGQPADVFPRLFKEWNINRLSFEYDSEPFGKERDAAIKKLASEADVEVIVRVSHTLYDLDKIIELNGGQP
PLTYKRFQTLISRMDPLETPAEPITAEVMGKCTTPVSDDHDEKFGVPSLEELGFDTEGLSSAVWPGGETEALT
RLERHLERKAWVANFERPRMNANSLLASPTGLSPYLRFGCLSCRLFYFKLTDLYKKVKKNSSPPLSLYGQLLW
REFFYTAATNNPRFDKMEGNPICVQIPWDKNPEALAKWAEGRTGFPWIDAIMTQLRHEGWIHHLARHAVACFL
TRGDLWVSWEEGMKVFEELLLDADWSVNAGSWMWLSCSSFFQQFFHCYCPVGFGRRTDPNGDYIRRYLPVLRG
FPAKYIYDPWNAPDSVQKAAKCVIGVHYPKPMVHHAEASRLNIERMKQIYQQLSCYRGLGLLASVPSNPNSSG
VAGMTGFSSGEGMHSSNAAGGVEGQAVTALSYSHGESHRGLQQQGYQMPSGRSSLPSYSSGDSQAGSNLLPQG
YTRGSTIVHYTQGDSQQVSSVILQQGRGHLPSVLTTVKRPSPEESTQGIVPKVQRQSSN
>Atractosteus_spat__MBN3324451.1
MTHRTIHLFRKGLRLHDNPTLLGALESSAVLYPVFILDRAFMEEAMACGVLRWRFILQSLQDLQRSLAALGSR
LYVVQGRYDQVLREKVHQWGITQVTFDTEVEPFFTRLDDDIRALGQEMGFSVQSCVAHTLYDVRRIIRANGGE
APLTYKKFLHVLALLGAPETPARRITREDFRKCRTPTEEESAEKYRVPSLEDLGIAVESEALWVGGETEGLQR
LEQHMQNQGWIENFTKPRTIPNSLLPSTTGLSPYFSLGCLSVRMFYHRLSHIYAQSKNHSLPPVSLQGQVLWR
EFFYTVAAATPNFTRMAGNPICLQIDWYKDQEALDRWKTARTGFPWIDAIMTQLRQEGWIHHLARHAVACFLT
RGDLWISWEEGMKVFEEHLLDADYSVNAGNWMWLSASAFFHQYTRIFCPVRFGRRTDPEGHYLRKYLPVLKNF
PSQYIYEPWTAPQQVQLEAGCIIGKDYPLPMVNHREVSESNLALMREVRREQEKTAQLTRDTADDPMEVGKKR
ACQREAETDRSDAFLEKPERAKRFGHAEGEAKALACNWPSETLRLPALGREVM

>Aureococcus_ano_XP_009033552.1
KLRAWQLGLTGFPLVDAAMRQLWVWGWMPNYVRHVVASFLVEHLHLDWRHGEAWFHDTLVDADAAINAFMWQN
GGRSGMDQWNFVMHPVHAAKNCDPEGDYVRRWCPELAALPRDHIHQPWRAPASL
>Aureococcus_ano_XP_009033826.1_AURANDRAFT
MAPKTTAVWFRKCLRLHDNAPLVEAAKRGGRLLPIFVLDPHFARPEFVGAPRYRFLLESLADLDASLRKRGSR
LCVVRGETEATLDGLFAGACDALPGVKVDAILWEEELVEPWGVARDARVAAAAKARGVAASAVAGGHLLFDPR
EVLGACGGKAPTTMPAMVKAAAALGAVPAPLDPPATLPPPPDGLPDSSVPTLEAMGYEAPPAAAFGVAGGESA
GLARLEAALARDGGKWARAFEKPKTKSTKFDVDPSTTVLSPYLKFGCVSARLFRARLADVYAGGSHAQPPTSL
LGQLYFREMAYANALKHGAAFAASPSPVCADIPWDDPATDAAAAARLRAWEAGETGYPFVDAAMRQLRETGWI
HHLARHAVACFLTRGDLWLPWTEGARVFDRDLLDADWAVNNFNWLGLAGVASWSPPFFRVYSPVPNHKQSSLN
VDDAAGAYVDRFVPELRKLPAKYKYAPWTAPAAVLAAAGVVLGDTYPRPLVDHKVQSKDNIARFKEAQQRNRA
NKPKPPPKGAPKTKKQKV
>Aureococcus_ano_XP_009034388.1
MSALAATAKPTTIVWLRDELRVHDNALLAEAAARGGPVLPVYVLDDRVFDAAATSESGGSRKCGAKRARFTLE
ALDDLRATLGARGSGLVVERGRPADVLAGLCAAVGGDATVLCSDAACSEERKDEAAVAKVAPLAKVWEGTLYH
PEDLRGVAFHDLFTAWRTKVEKAGTRVRGDVFPKAATLPAPPAGVDAKLAAPLPTLDALGYDAADAACDGRGD
FFAPAGGETAALARLRKYVFEEDRLKDYFNTRDGMIGQGYSTKLAPWLSRGCVSPRAVARACADYERTRGIKN
KSTYWVVFELTWRDYFVFYAQKYGSKLFRPYGVKDIRERTWRADPEALRRWKAGETGAPLVDANMRELAATGF
MSNRGRQNVASYLVLDLGLDWRLGAEHFEEHLVDYTPEANWGNWHAAAGLSGGRVNRFNILKQSKDYDPKGDY
VKLWIPELAKVPAPQCFTPSSLSKADREAAGAGDYPAPLPTQGFKFPDGARPGKKSHQQDRKGKGGERAQIQR
KRKQKSRVQASAWAE
>Auxenochlorella_pyr_BAE48230.1_CPD_photol
WMSRDQRVRDNWALIYAAQAAAKRGVPVAVAFSLVTEFLGAGARQFGFMVRGLQLIQPKLEALNIPFFLLKGD
PSETIPKLVQDTGASLLVTDYAPLRLGRQWRQAVAEQLEVPFHEVDAHNVVPVWVASDKREYAARTIRPKIHS
KLPEFLKEYPELPQQATWAHEVKPEAVDWEALIAEVLERGKAVPEVDWCKPGEDAAMEALAGGKGFLSKARLS
KYDAQRNDPTVADALSNLSPYLHFGHLAPQRAAIEAAKHKSTHKASVEGFLEELVVRRELSDNYCHYVENYDS
LDAAYDWARQTLNDHRNDKREHVYTKEQFEKGATHDKLWNAAQLEMVHCGKMHGFMRMYWAKKILEWTASPEE
ALEIAIYLNDKYELDGRDPNGYVGCMWSIAGIHDQGWAERAVFGKIRYMNYAGCKRKFDVDKYVARINAMVSS
IKAKAKAAKA
>Balaeniceps_rex__NXS41518.1
KVDYDVCSNYGNWLYSAGIGNDPRDNRKFNMIKQGLDYDGNGDYVRLWVPELQGIKGADIHTPWALNSAALSQ
AGVTLGETYPQPVVIAPEWSRHINQSPQGRSPHPRGRRGPARTPVQHKDRGIDFYFSRKKDV
>Balaeniceps_rex__NXS42748.1
CIYILDPWFAASSAVGINRWRFLLQSLEDLDNSLRKLNSRLFVVRGQPTDVFPRLFKEWGVTRLTFEYDSEPF
GKERDAAIVKLAKEAGVEVVIENSHTLYDLDRIIELNGHKPPLTYKRFQAIISRMELPKKPVSTVTSQQMETC
KVDIQENHDDVYGVPSLEELGFPTDGLAPAVWQGGETEALARLDKHLERKAWVANYERPRMNANSLLASPTGL
SPYLRFGCLSCRLFYYRLWELYKKVKRNSTPPLSLYGQLLWREFFYTAATNNPKFDRMEGNPICIQIPWDRNP
EALAKWAEGKTGFPWIDAIMTQLRQEGWIHHLARHAVACFLTRGDLWISWESGVRVFDELLLDADFSVNAGSW
MWLSCSAFFQQFFHCYCPVGFGRRTDPSGDYVKRYLPKLKGFPSRYIYEPWNAPESVQKAAKCIIGVDYPKPM
VNHAETSRLNIERMKQIYQQLSRYRGLCLLASVPSCAEDLSGPVADSVSGQGCSTSTAMRLSQADQASPKRKH
EGAEELCTEELYKRAKVTCLPAPEIPGKSL
>Balaeniceps_rex__NXS44423.1
MPHRTIHLFRKGLRLHDNPTLLAALESSELVYPVYILDRKFMTSVMHIGALRWHFLLQSLEDLQKNLRQLGSC
LLVIQGEYESVLRDHIQKWNITQVTLDAEMEPFYKEMEANIRRLGEELGFEVLSLVGHSLYDTKRILDLNGGT
PPLTYKRFLHILSLLGDPEVPVRNLTAEDFQRCRPPDPGLAECYRVPLPVDLKIPPESLSPWRGGETEGLQRL
EQHLTDQGWVASFTKPRTIPNSLLPSTTGLSPYFSMGCLSVRTFFHRLSNIYAQAKHHSLPPVSLQGQLLWRE
FFYTVASATPNFTKMAGNPICLQISWYEDAERLHKWKTAQTGFPWIDAIMTQLRQEGWIHHLARHAVACFLTR
GDLWISWEEGMKVFEELLLDADYSINAGNWMWLSASAFFHQYTRIFCPVLFGKRTDPEGQYVRKYLPVLKNFP
SKYIYEPWTASEEEQKQAGCIIGQDYPFPMVNHKEASDHNLQLMKRVREEQYRTAQLTRDDTDDPMEMKVKHD
HSEENISKGKVARMTE
>Balaeniceps_rex__NXS47370.1
DNWAFLYAQRLALKQELPLHVCFCLVPKFLDATIRHYGFMLSGLQEVAKECAELNIPFHLLLGYAKDVLPAFV
VEHGVGGLVTDFCPLRLPQQWVQDVRERLPEDVPFAQVDAHNIVPCWVASPKQEYSARTIRGKIHAQLPEFLT
EFPPVIHHPYPPSCPAEPIAWEACYSSLQVDRTVKEVEWATPGTSAGLAVLQSFIAERLKSFSSHRNDPNKAA
LSNLSPWFHFGQVSTQRAILEVQKQRGKYKESVDAFVEEAVVRRELAENFCYYNENYDSVQGAYDWAQTTLKL
HAKDKRPFLYELQELEQGTTHDPLWNAAQLQMVREGKMHGFLRMYWAKKILEWTRSPEEALQFAIYLNDRYEL
DGRDPNGYLTLCCLWSICGIHDQGWAERAIFGKIRYMNYAGCKRKFDVGQFERRYAPRT
>Balaeniceps_rex__NXS50437.1
MQHSSIHWFRKGLRLHDNPALLAAAANCCRLHPLFIFDPSRDRAGANARRFLLDALRDLDRSLREMGSRLFVV
RGCPEEVFPRLFHAWGTTRLTFEVDTEPPARRRDAAVAELAARHGVEVIQEVSHTLYDTERVLALNDGKAPLT

YKRLQSLLASLGPPEKPVPALTEEHLQGEHGVDLGRARARSWVSVSLTVDPGAERWPSHPHCPRHPRLPHPAW
VCGFKKPETEPTSLSPSTTVLSPYLKFGCLSVRTFWWRLDEVYQGREHSRPPVSLHGQLLWREFFYTAGASIP
NFDRMVGNPVCLQVDWDDNPQHLRAWREGQTGYPFIDAIMTQLRTKGWIHHLARHAVACFLTRGDLWVSWEEG
LKVFEELLLDADWSLNAGNWLWLSGSAFFHRYFRVYSPIAFGKKTDRDGAYIRKYLPVLKDFPTEYIYEPWKA
PRAVQEQVGCLVGTHYPQPIVEHRAASERNLGRMKAAR
>Baryphthengus_mart__NXG68295.1
MPHRTVHLFRKGLRLHDNPTLLAALESSEVLYPVYILDRRFMTSAMRVGALRWRFLLQSLEDLRKNLGQLGSS
LLVLQGEHEAVLRELVQEWNITQVTLDAEVEPFYKEMEANIRRLGEELGFEVLSMVGHSLYDTNRILDLNGGT
PPLTYKRFLHVLSLLGDPELPVRDLTAGDFQRCGAPEPGLAERYKVPLPVDLEVPPESLSPWRGGETEGLRRL
EQHLTDQGWVTSFTKPRTIPNSLLPSTTGLSPYFSVGCLSVRTFFYRLSNIYAQAKHHSLPPVSLQGQLLWRE
FFYTVASATPNFTQMAGNPICLQIRWYEDEEMLHKWKTAQTGFPWIDAIMTQLRQEGWIHHLARHAVACFLTR
GDLWISWEEGMKVFEELLLDADYSVNAGNWMWLSASAFFHQYRRIFCPVRFGKRTDPEGQYIRKYLPVLKNFA
SKYIYEPWTASEEEQKQAGCIIGQDYPFPMVNHKEASDHNLQLMQQVREEQSRTAQLTRDESDDPMEMKTKRD
RAEENLSKGKVAKTTE
>Baryphthengus_mart__NXG70584.1
KVDYDVCSNYGNWLYSAGIGNDPRDNRRFNVIKQGLDYDGDGDYVRLWIPELGGIEGADIHAPWALSSTALSQ
AGVTLGDTYPQPVVTAPEWGRHISQRPQGRSPHPKGRRGPTHPPKQHKDRGIDFYFSRKKDA
>Baryphthengus_mart__NXG76848.1
DNWAFLYAQRLALKQELPLHVCFCLVPKFLDATIRHYGFILRGLQEVAKECAELNIPFHLLLGCAKDVLPTFV
VERGVGGLVTDFCPLHLPRQWVEDVKEQLPEDVPFVQVDAHNIVPCWVTSPKQEYSARTIRGKIHAQLPEFLT
EFPPVIRHPYSSSCPAETITWEACYSSLQVDRTVKEVAWATPGTAAGLAVLRSFIAERLKSFGSHRNDPNKAA
LSNLSPWFHFGQLSTQRAILEVQKHRGKYKESVDAFVEEAVVRRELAENFCYYNENYDSVQGAYDWAQTTLKL
HTKDKRPFLYELQELEQGTTHDPLWNAAQLQMVREGKMHGFLRMYWAKKILEWTRSPEEALRFAIYLNDRYEL
DGTDPNGYLMLCPHPTGCLWSICGIHDQGWAERAIFGKIRYMNYAGCKRKFDVGQFERRYAPRT
>Baryphthengus_mart__NXG77614.1
SLEDLDNSLRKLNSRLFVVRGQPTDVFPRLFKEWGVTRLTFEYDSEPFGKERDAAIVKLAKEAGVEVVIENSH
TLYDLDRIIELNGHKPPLTYKRFQAIISRMELPKKPVSTVIRQQMETCKVDIQENHDDVYGVPSLEELGFPTD
GLAPAVWQGGETEALARLDKHLERKAWVANYERPRMNANSLLASPTGLSPYLRFGCLSCRLFYYRLWELYKKV
KRNSTPPLSLYGQLLWREFFYTAATNNPKFDRMEGNPICIQIPWDRNPEALAKWAEGKTGFPWIDAIMTQLRQ
EGWIHHLARHAVACFLTRGDLWISWESGVRVFDELLLDADFSVNAGSWMWLSCSAFFQQFFHCYCPVGFGRRT
DPSGDYVKRYLPKLKGFPSRYIYEPWNAPESVQKAAKCIIGVDYPKPMVNHAETSRLNIERMKQIYQQLSRYR
GLCLLASVPSCVEDLSGPVTDSSSGQGCSTSAAMRLSQADQASPKRKHEGAEELCAEELYKRAKVTGLPAAEI
PGKNL
>Baryphthengus_mart__NXG79270.1
FLLQCLEDLDANLRKLNSRLFVIRGQPADVFPRLFKEWNIAKLSIEYDSEPFGKERDAAIKKLASEAGVEVIV
RISHTLYDLDKIIELNGGQPPLTYKRFQTLISRMEPLEMPVETITPEVMEKCTTPVSDDHDEKYGVPSLEELG
FDTDGLPSAVWPGGETEALTRLERHLERKAWVANFERPRMNANSLLASPTGLSPYLRFGCLSCRLFYFKLTDL
YKKVKKNSSPPLSLYGQLLWREFFYTAATNNPRFDKMEGNPICVQIPWDKNPEALAKWAEGRTGFPWIDAIMT
QLRQEGWIHHLARHAVACFLTRGDLWISWEEGMKVFEELLLDADWSVNAGSWMWLSCSSFFQQFFHCYCPVGF
GRRTDPNGDYIRRYLPVLRGFPAKYIYDPWNAPESTQKAAKCIIGVNYPKPMVNHAEASRLNIERMKQIYQQL
SRYRGLGLLATVPSNPNGNGNGGLMGYSPGESISGCGSTGGAQLGTGDGHTVVQSCALGDSHTGSSGIQQQGY
CQASSILHYAHGDNQQSHLLQAGRTTLGTGISTGKRPNPEEETQSVGPKVQRQSTN
>Basidiobolus_mer_ORX77140.1_cry
MVNSIMLHSTPTRRSVIICLFRHDLRIRDNPILHYANSHPTATHMLPVYCFDPRQVNLTRARGKDYAEPKSRL
FNLPRSSKLRTRFLVESVLNLKTNLKKLGSELLIALEPPEEFLPRVADELLPEYDVEAVYLQEEVTDEEIRVQ
QKLIHRMNAPVESFHGATLVHPEDLPFPMEDLPDVYTKFRKAVESMDTPFRPVLPTPKKLKPLPGNDLLDQML
TVDADHLLPSMGVVDTAPDLRTVFPFGGGEDQAIERLTDWVWNTKSISTYKETRNGLLGTDYSSKLSPWLALG
CLSPREILKQVEAYEDSNEANISTYWIRFELLWRDYFWFVGGKYGNGIFHRDGVLANRSDDQPSWKVDPNLMK
AWIHGETGIPWVDANMRELRNTGYMSNRGRQNVASFLAKDLRVDWRFGGEWFETHLLDYDPCSNYGNWQYGWF
SVAGVGNDPRENRHFNMIKQAKDYDPEGKYVAMWCPELRDVPVKHIHTPWKMPHDVQQKANCVIGRDYPEPIV
LQEVWEKHVARPPKDTKKSSGNPNERVSKKDSRRRQRRKAKASKPHSHR
>Basidiobolus_mer_ORX97314.1_DNA_photol
MNTFIWFRTDLRTADNTALHAAAECARQGAGALLGVFVISPEEWKAHDLSPFKVDFMLRNLTQLRKKLKELNV
PLVIEHAENQSDVPQIVLSCCQKYNVDKVFCNQEYEVDEEKRDAKTEALLGQHNIEFYRRHDQCIVEPGVVVT
QAEKPYTVFTPFKKNWLRKVGSDKKYHNPLPAPSPFPEQNLSRCRTSAQTDPPGELPEFPLSPEMKKVASSMY
PEGEEKAIERLDRFVQKKISNYDTGRDFPIDDGTSVLSPYLALGVISPRQCLAKALGANENRIASGNEGVQTW
ISELAWRDFYRNVLVSFPRVCKNRAFKPETEDIEWNDNPKHFTAWCEGRTGYPIVDAGMRQLNSIGWMHNRLR
MVVAMFLTKDLLINWQKGERYFMNHLVDGDFASNNGGWQWAASTGTDSQPYFRIFNPALQSERFDKNGDFIRK
WVPELKPVKGKAIHEPYKHLSPSEFKKLGYPKPIIDHSKARQTTLAAFKAIAKK
>Bathycoccus_pra_XP_007509298.1_predicted_

MSPALSNVDDGKTKKKKRVILWHRNDLRVHDNLTLKEALTFCSESSELCELVPTYIFDPRWFLSDDAFDRSDQ
KRREKSLTNAPKCSQRRAEFLLESVLDLKERYKRLGSDLLIKIGKSENVLDALDADVVVCSREVCEDERALER
KVKRKAKGELKLVWDNTLYHYEDVFESGNCYQNGLNDLPTQFTQFKNKVESKVSVRKPILNDAELSDGLKKFS
SPSNVSEEEMQFVPTIEDIPLSDDARQMHAAIPKDNSITPVYSFKGGESEALKRVQRYLYETDAVATYFDTRN
GMLEDLESTKLAPYLALGCISPRFIENEIRKYEKERVENKSTYWVIFELTWRDFYRFFALRHGAKIFKLDGIG
SNGKMPPWTAEHEGRDEIFEKWISGTTGYPLIDANMRELKLTGWMSNRGRQNVASWLALDARIDWRMGAEWFE
FLLLDYDPASNWGNWVAAAGLTRGRVNKFNIPKQTKDYDPQMKYCRKFIPELDSKDYPRPVQLKKFAFEESSS
SRNNGGRNGPGRGTTNGRGGRRGKQNRQPRNNAFYDDDRQNGGGGFS
>Bathycoccus_pra_XP_007511873.1_CPD_photol
MLLRTATRCCSPLLNNNRFALFKSSKRTPARRSATTCAFFPSSSLFTNLTTTTTTASATARGFQDHFARRSNR
ASSNRTLLTLLQSPNGGRFTTRRKKHTAAISSTFVVYAGTTNTTIDINENNNNNKNEDKNMIVHPDRVMLIND
QPILREGNGPIVYWMSRDQRVNDNWAMLYAIELANKEKKPLVVVFNVVTKFLGAGARQFGFMLRGLREVESAL
EERDIPFKLLHGGDEPNAEIEKFCNEVNASAVVTDFSPLRLGLKWRDDFAKETKRSVRVVDAHNIVPCWVASP
KLEVGARTLRGKLAKLYGDFMVPFPDNFPNVENKDAALHAKIKSVKTDWDDVLGQALERGKDVPEVTWAVPGE
KAAMAVLDNFLTKRMSLYGLRNDPAKPQALSGLSPYLHFGQISGQRCAMKALEAKKGSNGKAVDVFFEELVVR
RELADNFCYYSPQYDTIEGQKYDWAKDTLRMHAGDKREYTYTYEEFEQAKTHDNLWNAAQRELVYGGKMHGFM
RMYWAKKILEWSDTPENALKYAIALNDRWSLDGRDPSGYVGCMWSIVGVHDQGWKEREIFGKIRYMAYSGCEK
KFKIPEYIKRVDALVEAVQKCEVSYKSNPGAWEIGRDDVLPKVAKSDNDDDDERKSKKQKK
>Bathycoccus_pra_XP_007514096.1_protein
MGNEATTSIVWFRKGLRIHDNPALSEALIDAKNFGGGDESKKVLPLFILDPWFCNEKSVGANRMNFLLQSLTN
LNENLGSLLSIENVNDANNCLTVVQGKPKDVLPNVIKKFNVSSVYFEKEQVEPFGKQRDEEIIEMCKKLNVDV
NTYASHTLYDQEFLLSKASAKGIPPSTMGAFQKVLANVGEPPSPVPEPPKNSLRVIPNAGDALSKEFKYANGI
PTLEDLGYKAKSLSFKGGKGGETEGLRRMRGALCRGGDGYKYVQEFDKPQSSPCQLFAKEKGSGKALNPFEMA
KKASSSSSPKEESLLWPSTTALSPHLKFGTVSVRQFYFELQDILKNELKGKHTNPPTSLMGQILWREFYYVNA
CGTPNYDKMVGNRICKQIKWKHDPENLAKWENAQTGFPWIDAAMTQLKEEGWMHHLARHAVACFLTRGDLFIS
WEEGARVFDRDLVDADWALNNGNWMWLSASSFFYQYFRVYGPHSFAKKYDKEGAYVKHFLPVLKDMPSKYVYE
PWTAPLEVQKKAGCIVGVDYPKPMVDHAIASKQCMAWMNEAYAAGKEKKSGGEEKSNKKAKTK
>Batillaria_attr__KAG5688704.1
MPGDSNQKKEDNWALLYAQRLALKLELPLYVVFCLVPKFLDATIRQFGFMLKGLAEVEKECRELGISFHLLMG
EARTVLPNFVKKNNIGGVVTDFSPLRTPTKWVQDMAKELPQEIPFCQVDAHNIVPCWEASPKLEYSARTIRTK
IHNVLSEYLTEFPPVVKHPHKVKAMPEPVDWDAAHKFLEVDRSVGEVEWATAGTAAAYSTLESFCTQRLKYFS
SERNDPNKPALSNLSPWIHFGQISVQRCILMVKMFKSRFKDGADAFIEEAVVRRELADNFCFYNEHYDSIKGA
YKWAQDSLDAHRGDKRPYIYTRQQLEDAKTHDDLWNAAQVQMVREGKMHGFLRMYWAKKILEWTESPEQALAE
AIYLNDKYNLDGRDPNGYVGCMWSICGIHDQGWAERAVFGKIRYMNYNGCKRKFDVPGFVRKYRKK
>Batillaria_attr__KAG5693470.1
MLLSVLWQLNPGILEANNNQPPLTYRRFQSILSGLPPPPLPLDPITTSHVQHTQTPISDDHDEKYGVPTLEEL
GFETDSLGPARFQGGESEALARTYRHLERKAWVASFERPKMSPQSLFPSQTGLSPYLRFGCLSPRLFYWKLTE
LYKKVKKGQDPPLALHGQLLWREFFYSAATNNPNFDKMVGNSVCVQIPWDHNPEALAKWAEGHTGFPWIDAIM
VQLKKEGWIHHLARHAVACFLTRGDLWISWEEGMKQFFHCYCPVNFGKRADPTGDFVRHYLPVLKWFPTQYIY
EPWTAPEAVQKAAKCIIGKDYPLPMVDHSHVSRINMERMRQVYKHLLVCSTVSGYRRKLDLPKIPKMDMFCLK
ANMFSSTSSDDDHEQTSVIRRENKI
>Batillaria_attr__KAG5702948.1
MNACRKKPAAPLHPIPVPKKKWSLVGMDIIGPLQVTDRGNQYIVALTDHFTKFPVAKAIPCKSSEEVARFMYE
TICIFGAFDSLITDQGREFINFALDILTDRFNINHRISSAYHPETNGQRERDNRTLKDTLVKITDNDDWDELL
PAALFAYRTSVHKSTQFSPFLALYGEQAKLPFDVTGDANEQDLNEEIMDTMKGIHEEVKNKIGENISRAQKKQ
KEYYDSRRAYDHGFRIGDKVLIINAARVHRMGGKMAPRFVGPYELFKLLDSNWICKFEKPQTEPNSLQPSTTV
LSPYLKFGCLSARTFYYRLLEVYRLNKFHSQPPVSLHGQLLWREMFYTVGAGTPNFDRMEGNPVCRQIPWDTN
PEYLAAWKEGRTGYPFIDAIMTQLRQEGWIHHLARHAVACFLTRGDLWISWEEGQKVFEELLLDADWSLNAGN
WMWLSASAFFHQYFRVYSPIAFGKKTDKNGDYIKKYLPILRKFPAQYIYEPWKAPRSVQEAAGCVIGRDYPKP
IVDHDTISKKNIKRMAEAYASHKEGKDSSEMDEEKYQ
>Batillaria_attr__KAG5704110.1
TNGAGYPRMRFLLECLDDLDKTLRQKGGRLFVLHGQPEDVFPKLFQEWGVTRLTFEQDPEPVWQDRDNKVKAL
CKELEVECIEKVSHTLWDPLNIIQANGGSPPLTYAMFCQVTEIVGLPPRACPDPDFTGVKLPSSVHGDAKFHV
PTCEELGVYPESDRQSHPLSGYSGGECRALELLELRIEKERQAFERGQCLPNQINPDLTGLPMSLSPHLRFGS
LSVRHFYWLIHRTFMEVTQSETVPTSITGQLIWREYFYCMSVNNPKYNCMEGNPICLDIDWYRNMEQFEKWSK
GQTGYPWIDACMRQLREEGWIHHVCRQSVACFLTRGDLWIDWQMGLEVFDRYLVDADWSVCAGNWMWVSSSAF
EKVLQCPRCVCPVRYGRRMDPKGDYVRRYVPELQNMPLLYLFEPWKAPAAVQETAGCVVGRDYPPPMVDHKQA
SKMCMAKMEKIKGKCRDVPHIAPSNEAEVISFIWIGNKSEDDKNIRCDQHDMRLRDKVR
>Batillaria_attr__KAG5707978.1

MNNLYEPCLKARDDKICKQLESQGVEVKRFHSYLLHEPGSVNTESMGMRGIGSVTHFMECCRQSSTQSIGLPA
DKPPCLPASGSMPQSQALDELGLSRLPRRRDGTVIDWAKGIRESWDFSEDGAWNALELFVDEGIRHYEKESGR
ADMLNTSRSSPYLHFGQVSPRAVLRHAQGMRSPKFLRKLAWRDLSYWLLTLFPEMPSKPMRPHYQAQRWSQDK
SHLRAWQKGKTGYPLVDAAMRQLWLTGWMNNYTRHVVASFLISYLHLHWEHGYAWFQDTLLDADVAINAMMWQ
NGGMSGLDQWNFVMHPVDAAMTCDPNGQYVRQWCPELAALPDDFIHKPWKCPPSVLRRAGVELGKTYPHRIIT
DLEEAREQSLRDVVTLRLANHQYVDPYTGNDLVPLPSGLLLPVITRKEFKYKTANPHAKDNPHTAVLRGYRSR
KRDEAIAFANQVDFTASTMNECALRQKRYEQANRFVQF
>Batillaria_attr__KAG5712394.1
ALQWAHRNSDHILPLYCFDPRHFKGTYHFGFPKTGPHRLKFLLESIEDLRQNLQKHGSNLIVRQGKPEAVVPE
LVKQLGTENVEALVFQKEVTQEEVDVEKKLKEKCGVKVHTVWGHTLNHVDDLPFKPHNLPDVYTQFRKRVESQ
DRVRQEIAMPETLRPLPPDVDLGNVPSFDDFGMPKPQAASSSVFPFPGGETTALCRLESYLWKTDNVATYKET
RNGMIGSEYSTKFSPWLAHGCISPRKIYWEIKKYERERTANQSTYWVIFELLWRDYFRFVALKYGNRIFFLEG
IQGKNIPWKKDMKQFEAWKEGRTGVPYVDANMRELMATGFMSNRGRQNVASFLTKDLHLDWRLGAEWFESALI
DHDVCSNYGNWLYSAGLGNDPREDRKFNVVKQGLDYDSNGDYVRLWVPELSKVQGGSVHFVWTLNSAVLSHSD
VSLGETYPTPLVVAPEWSRHFGRP
>Bemisia_tab_XP_018904762.1_cry-2
MAARGTAIHWIRKGMRLHDNPALIAAIESQKNVGEICILRPVYILDPYFRKYMRVSPNRWRFLQQALEDLDKS
FRAIGTRLYVLHGKPEEIFPELLKEWNVKLLTFESDIEPYACKRDSSVMSIAKKFGVKVLQSVSHTLYNPEII
IKKNLGKAPLTYQKFTTVVDSIGLPPKPVDAPNALPKEAQPEDLAAAKYNVPTLAEFGLDVKSLHPTKFPGGE
TEGLARLAEKLSDKAWVCKFEKPNTSPNSLEPSTTVLSPYLKFGCVSARLFYYKVKDVISKAPHSKPPTSLIG
QLFWREFYYTVASVTPNFHKMEGNPVCCQVPWDSNSEHLDAWAFGRTGYPFIDAIMRQLRQEGWIHHLARHAV
ACFLTRGDLWISWEEGQGVFEELLLDADWALNAGNWMWLSASAFFHQFFRVYSPVAFGKKTDKFGDYIKKYVP
ELGKYPSEYIYEPWNAPKSLQERAGCVIGVDYPKRIVIHENVYKNNIQRMSLAYKNNKQNTETTNSNPAGSSK
SKTGKGGSTKRKPEAEVAASGIKKFLKKN
>Bemisia_tab_XP_018905816.1_photolyase
MSSAPPAKKAKAETSESSNGSSSSEGGFEEFLKQIEEDRKKAAKSVLEFKFNKKRVRILSKSKEVPEWGKGVI
YWTFRDQRIHDNWAFLFAQKLALKNDVPLHFAFCRLPKFLDATLRHFKFIFEGLKETEKECKKLNIQFHFLIG
CGKDVLPDFVKKHKLGAVVIDFMPLRGPMAWADELSKSLPDGVPLVQVDAHNIVPVWETSEKLEYAARTIRNK
VNGKLPEFLTQYPPVIKHPHSGDLKASPIDWEEAEKTLEVDKSVGPVKWAKPGYRGGMQQLYTFVTKRIKNYG
TARNDPNKNALSMLSPWFHFGHISVQRAILEVKKVKGYSESVAAFCEEAIVRRELSDNFCFYNKNYDSIKGAY
DWAKKTLKDHSGDKRPYLYTKAELEQGHTHDDLWNAAQNTLINDGKIHGFMRMYWAKKILEWTESPEQALQYA
IYLNDKYSLDGRDPNGFVGCMWSICGIHDQGWSERAVFGKIRYMNYEGCKRKFDINGYIIKHGGKVYTKGKEN
TMDKFVKKKK
>Bemisia_tab_XP_018906320.1_putative_c
MASPKILIYLLRRDLRVHDNPIFHKLTSMSSQANAPFTHLLPLYVFPAHQIEVSGFLSSSDEKSPYPEARSKV
GKFWRCGQLRAKFLAESVWDLKQNLETIGSGLEIRVGMLHDVVKQLVEGFKSKGVQVKGLWMTSEEGYEEKAE
ERQVRKIIVNAGGDFQLWKDEKYFIDDDDIPFDDPQKYPDVFTKYRNTVEPLREAPRKVLPTPKKLPPLPQNI
PPQAHPFKIPGNLKDLIAALQKPLDAGLGLKNPPQMPSAGASSAVPFAGGATSGQKRLKHLIESGAMTRYKDT
RNGMVGTDYSSKLSLWLALGSLTAREVHSALIDFEEGKTDVGKGAEGYGKGENKGTTHMRFELLWRDYMRLCT
RKYGSRLFLVGGFRNARNIQWKHDNSIMQRWLEGTTGIGLVDAAQRELFLTGFTSNRARQNVASFLTKHLEQD
WRLGAEWYECNLVDYDVSSNWGNWQYTAGVGNDPREDRKFNPVKQASDYDPKAEFVKAWIPEVRELQPEEAWQ
CWKASYAAKQKPGLRGNIMAERPLAKISFTPRSGGNDGHRGGGRSRGRAQWF
>Bemisia_tab_XP_018915448.1_cry-1
MSEDRSPRVSVLWFRHGLRIHDNPALLRALEDTDIFYPIFIFDGESAGTKLISYNRMKFLLESLEDLDNQFRA
KGGKLYVFFGSPIEIFAKFKAHLNFYKLTFEQDCEPIWQARDNSVKKLCDDLDIIWDESISHTLWDPREIIDT
NGGSPPLTYSMFLHTVSVIGPPPRPVEDPCWDGIKFGVLPDSLKRELDVRDKIPVPADFNLKPELKQSEYTIE
WFGGENQALSNLRKRIAVEEKAFRQGFCQPNQTQPDLLGPPTSQSAALRYGCLSVRKFFWALHDTFSDVHNMP
SNSNITSQLIWREYFYTMSVDNKYYAEMARNPVCLNIPWSSVKDETAKANLKAWEDGKTGYPFIDALMRQLKQ
EGWIHHIGRNAVSSFLTRGDLWISWEEGLRHFLRYLLDADWSVCAGNWMWVSSSAFEKLLDCNFCICPVNYGR
RLDPYGEFVKRYVPELQKMPVEYVYEPWRAPLEVQEHANCVIGRDYPKRIVDHNVVSHENKEKMKEICKSLKV
RTPHCAPSDELEIRQYMWLPDSCTSHLSTLK
>Bemisia_tab_XP_018916708.1_cry-1-like
MLDDNMGQTSKHTMHWFRKGLRLHDNPSLMEGLIGASTFRCVFIIDPWFASTSNVGINKWRFLLQCLEDLDCS
LRKLNSRLFVIRGQPADALPKLFKEWNITCLSFEEDPEPFGKVRDQNIMALCKELSIEVISRVSHTLYKLDKI
IEKNGGRAPLTYHQFQTVIAQLDPPQQAESPVSVRLIANAFTPISEDHDETYGVPTLEELGFSTEGLLPPVWL
GGEAEAMTRLKRHLERKAWVASFDRPKMTPESLQASQTGLSPYLRFGCLSTRLFYYQLNDLYKKVIDIKKAVP
PLSLHGQLLWREFFYCAATNNPNFDKMVGNPICVQIPWDKNAEALAKWANAQTGFPWIDAIMTQLREEGWIHH
LARHAVACFLTRGDLWMSWEEGMKVFEELLLDADWSVNAGMWMWLSCSSFFQQFFHCYDPVRFGRKADPNGDY
IRKYLPVLKNMPTKYIHEPWIAPGPVQKAAKCLIGEDYPLPMVNHAVASRINIERMKQVYLQLSKYKNLGQHC
MPISTKESTKSIMNSNIPIPLMDYS

>Betta_splendens_XP_029009113.1_cryptochro
MVVNSVHWFRKGLRLHDNPALQEALNGADTVRCVYILDPWFAGAANVGINRWRFLLEALEDLDNSLKKLNSRL
FVVRGQPTDVFPRLFKEWKVTRLTFEYDPEPYGKERDGAIIKMAQEFGVETVIRNSHTLYNLDRIIEMNNNNP
PLTFKRFQTMVSRLELPRRPLMPVTQQQMEKCCTQIADNHDQLYSIPSLEELGFKTEGLPPAVWHGGESEALD
RLNKHLDKKSVQVWVANLEHSRVNTCSLYASPTGLSPYLRFGCLSCRVLYYNLRELYMKLRKRCSPPMSLFGQ
LLWREFFYTAATNNPNFDRMEGNPICVQIPWDQNPEALAKWAEGRTGFPWIDAIMTQLRQEGWIHHLARHAVA
CFLTRGDLWISWESGMKVFEELLLDADWSVNAGSWMWLSCSAFFQQFFHCYCPVGFGRRTDPTGDYIRRYIPI
LKDYPNRYIYEPWNAPESVQKAANCVVGVDYPKPMINHAESSRLNIERMKQVYQQLSHYRGLSLLASVPTIQE
EAEPPMTDESQTSSGPDSPHRGPNDCEETGCSMAPDSSTTCPSCCAPPPDPEETLCTASGSKLNLTAASASLT
QATATPLLPASSVRGPLSRAKPSSPSSLCSALSLSSSPSPTQASCSSQRRKGLVRKMRRSQRHRGRQSCATSY
REGGGKPEDEEQEEAGGDERMEGDGEQDEERMEEDSTGLNQ
>Betta_splendens_XP_029010221.1_cryptochro
MVVNTIHWFRKGLRLHDNPSLRDSIRGADTLRCIYILDPWFAGSSNVGINRWRFLLQCLEDLDASLRKLNSRL
FVIRGQPTDVFPRIFKEWQITRMSYEYDSEPFGKERDAAIQKLASEAGVEVMVQISHTLYSLDKIMELNGGQP
PLTYKHFQALINHMDPVELPAETITSDVMKTCATPISEDHDEKFGVPSLEELGFETEGLTTAVWPGGETEGLM
RLERHLERKAWVANFERPRMNANSLLASPTGLSPYLRFGCLSCRLFYFKLTDLYRKVKKNSTPPLSLYGQLLW
REFFYTTAANNPCFDKMEGNPVCVQIPWDRNPEALAKWAEGQTGFPWIDAIMTQLRQEGWIHHLARHAVACFL
TRGDLWISWEEGMKVFEELLLDADWSVNAGSWMWLSCSSFFQQFFHCYCPVGFGRRTDPSGDYIRRYLPVLRG
FPARYIYDPWNAPEEVQKAAKCVIGLHYPKPMVHHAEASRVNIERMKQIYQQLSCYRGLGLLAMVPSNNGSNG
SNVPGINTESRQGHEDRSIKEGVPHFERQSTQKRRHEEAPSEISSKCWRQSK
>Betta_spl_XP_028986920.1_CPD_photol
MSDRKRKATSAAPAPEEPSAKQQKLPSVKKGADAGCCLQDLVDQQRSEKRGMAFNRKRVRFVSGTEKVKQGSK
GVLYWMSRDQRVQDNWALIHAQQLAVEEKLPLHVCFCLVVPKSELSTLRYYSFMLKGLEEVAQECKSLNIQFH
LLHGSAGEVLPGFVSDHSFGALVTDFSPLREPLQWLEDVKKKLPKDVPFIQVDAHNIVPCWVASPKLEYAART
IRGKITNLLPEFLTGFPLVEKHPHTAIRTAKPVDWDKTLASLNVDRSVGETAWASAGTRAGMAVLQSFIDERL
KLFKTHRNDPNAAALSQLSPWIRFGHLSAQRVALQVKHCKKGSSESVASFLEELVVRRELTDNFCFYNKNYDS
VEGAYDWAQQTLKAHAKDKRSYLYTREHLETAKTHDKLWNAAQYQMIIEGKMHGFLRMYWAKKILEWTSSPEE
ALSIALYLNDRYNLDGQDPNGFVGCMWSICGIHDQGWAERPVFGKIRYMNYSGCKRKFDVAKFERSYCPKTL
>Betta_spl_XP_029000458.1_cry_circad
MAHSTLHWFRKGLRLQDNPALMAALRDCKELYPVFILDPYLHNKTCMGPNRWRFLIGSLKDLDLSLRKLNSRL
FVVRGKPGEMLRELFSRWKITKLTYEYDTEPYSLSRDKEVAAVAQEHGVEVVYKVSHTLYDVDRIIEQNNGKT
PLTYKRLQELTRSLGPPKKPIPAPSVENMQDVKTPCSENHEGKYGIPTLEELGHNTASLPEELFPGGEQEALR
RLDEHMRRASWVCSFEKPQTSPNSLSPSTTVLSPYVTFGCLSARTFWWRLADVYQGKKHSQPPVSLHGQLLWR
EFFYTASVGVPNFNRMEGNPVCTQVDWDTNSEYVAAWREARTGFPFIDAIMTQLRQEGWIHHLARHAVACFLT
RGDLWISWEEGQKVFEYLLLDGDWALNAGNWQWLSASTFFHQYFRVYSPIAFGKKTDKNGDYIKKYLPLLKKF
PAEFIYEPWKAPRSVQLAAGCIVGKDYPRPIVQHEVVSKANIGRMKAAYARRPHDAAESPSKSPNNKPGAKRK
VLSVADMLMKKQRKE
>Betta_spl_XP_029004449.1_cry_DASH
MSTYRIIICLLRNDLRVHDNELFHWAQTNADYIVPLYCFDPRHYLGTHNYNLPKTGPFRLRFTLESIRDLRNT
LLSKGSNLVVRRGEPERVVAALIKHLGSVSTVAFQKEVTSEELNVEKNVNDVCDKMKVKVHTCWGSTLYHIDD
LPFHHISGLPDVYTQFRKAVETQSRVRPVFPMPEQLKPLPPGLEEGEIPTAEALQQTEPVSDARSAFPCSGGE
SQALARLKHYFWDTDAVATYKETRNGLIGVDYSTKFASWLALGCISPRYIYHQIKQYERERTANQSTYWVIFE
LLWRDYFRFVAIKYGNKIFQLKGLQDKSVPWKKDMKLFTAWKEGQTGVPFVDANMRELAMTGFMSNRGRQNVA
SFLTKDLGLDWRMGAEWFEYLLIDYDVCSNYGNWLYSAGIGNDPRENRKFNMIKQGLDYDNNGDYVRLWVPEL
QAIRGADVHTPWTLSTSALSHAGVSLGETYPTPIVIAPEWSRHLNNKARGTGPSPRGKKGPSHTPKQHRDRGI
DFYFSRSKNL
>Betta_spl_XP_029010334.1_photolyase
MPRSAAAIDDSKAWVRKMIREVLVGREDPEGLFAMCLSVLGPQETRSQFLCLIRPLAAANAELHFRLSSIYQE
YFSKFQTEEDELELALALSLMEIKDQDVLSTQSQESRFLQPGDEGLKQTPPENAVGTCAAEAIARGVPVMEEG
GVHQSEKGNRVKSRRRRHRAARQQAVGLPSSLSAPPPVLLWFRRDLRLCDNPALSASLECGAPVIPVFIWSPE
EEEGPGTTLATGGASKYWLHQALSCLCASLEHIGSHLVYIKAHGGGAGVGSSLHSLQELIKDTGARTVLANAL
YEPWLKARDDAVASALQKDGTELRMFHSYCLRDPYSVSTEGVGLRGIGSVSHFMSCCKQNPASVLGMALDPPA
SLPAAAQWPQGVSLDTLGLAQMPRRKDGTVINWAADIQKCWDFSEEGAQARLEAFLQDGVYRYHKESGRADAP
NTSCLSPYLHFGQLSPRWVLQAAKRTCCQTSKFQRKLAWRDLAYWQLTLFPDLPWESVRPPYKALCWSSRRDH
LKAWQRGRTGYPLVDAAMRQLWLTGWMSNYMRHVVASFLIAYLHLPWQQGYRWFQDTLVDADVAIDAMMWQNG
GMCGLDHWNFVMHPVDAAMTCDPCGTYVRKWCPELADLSDEFIHKPWKCPISMLRRSGVVFGQTYPERIITDL
EERRSQSLQDVAHVRKEFNQYVDKHTGCDLVPLPPRLLSETMGLSQKDDCVVLEGKQFLLPVITRMEFKYQQE
NPDTDAAANPYNALLKGYVSRKRDETIAFLNERDFTASVMYEAVQRKERLESDHRRMEGLPEPPALRGKVRRT
PTAKDKFSIVPGGGAVTSFE
>Bifiguratus_ade_OZJ01509.1_BZG36_0555

MATLFWFRNGLRVHDNRALLKALDYDLDEKTSCIRNADKKHSALVYPVYILEPDRLKSGLIGPNRVKYLQVKT
CHCREAAVDKSAGDPLSKIPKLCADYKVNKLVFERDTDEYCRGRDENIKELMKKCHVQVETYSGHTLWDLDDI
MDRSGGQLTKSYGPFVKLLEKMPQPQNRFPSIPTMQGTLDPHMATDPKYDLPNVEDYLQGKEPTTIHKGGESE
GLRRLDNYLQDTTRTRVFRKPQTSPIDTHPASTTILSPFMKFGSLSCRTFWHRVNEVYKKGQHSAPPESLIGQ
LYWREFFYSQGEVALLIPWKGYNENKEDEEKFQAWAEGRTRYPWIDAVMHQLIKHGWMHQLARHCVACFLTRG
QLFISWEKGAEVFHKYLLDADYNLNNGNWLWLSASAYYTTYFRVYSPTKFPQKYGKSDGAKFIRQFVPEVAKL
PDLYIFEPWKAPPSVQKSSNCVLGKDYPHPIMDAENAKAENMEIMRICYEKKVHGNCSPKEYEDHMALFKIVL
AKDYNQYGNGASNKAPRKGQINLETAMQQLAKRTQRQRENN
>Bifiguratus_ade_OZJ06231.1_BZG36_0080
MPAQNRRTSLDEYFRTGGPPLKKEKIEDNVKTESSQSNGGAATETGSATTSIHQALASRWTTTSSNGVANGAF
YSVKHESLPKPNGKMLNCLHWFRRDLRLQDNPALSYATFIANQCQTHACAIYIISPEDMKLHDDAPIKIDFWM
RNLKCLQQELDKLNIPLVIKTVPKRRDIPQVLLDFCDQHGISELTINHEYEVDEDARDKKVQRLFESKGKHVH
GFHEQCVIQPGKLMTQSNKPYTVFTPFKRAWLSLVEVHKISPGQFADLQLAPKLEANDVKVRDSNSSFHTLFT
DKSLTTIPDSVPGFEMPADLQKTAHQMYPAGEADAHSRLDNFVRDKVKLYDKKRSLPGEPYTSTLSPHFASGI
ISSRQCLQAAVKANNGKLSSGSDGIQSWIQEIIWRDFYRHVLVHYPKVCKNQPFRDEGNNIQWATYNEDEENE
AFFAWCEGRTGYPIIDAGMRQMNATGWMHNRLRMATAMFLSKDLLINWQRGERYFMQCLIDGDLASNNGGWQW
AASTGTDAQRYFRIFNPMLQSEKFDKSGDYIRRWVPELKTIRGPAIHDPYHVLDRKAFEALNYPKPMVDHKFG
RDRALQAYKAAMHAGSAKQ
>Biomphalaria_gla_XP_013065471.1_cry_DASH-l
MQTASENPSVAICLFRNDLRVHDNEALFTANQKGKFLLPLYCFDPRHHAGTYHFGFPKTGCHRLRFLLESVTD
LRLKLQSMGSNLIVRLGKPEDVIAELVKNLNLQNNVAVVIQKEVTEEEVKVEQDIRSKCRVPLLTVWGHTLYH
IEDLPFEPKHLPDVYTEFRKKVEDNLPVRKLINLPVQLNPLPMGIDQGLIPTFPDFGFSVPDRDPRSAFPFIG
GESSALQRLNDYFWVTDNVSTYKETRNGMIGTNFSTKFSPWLAHGCLSPRKIYWELKRYERERASNQSTYWVI
FELLWRDYFRYVSLKYGNKIFFSGGIKNEPTQWKTNHQHFKAWQEGRTGVPYIDANMRELAATGFMSNRGRQN
VASFLTKDLQLDWRLGAEWFESLLIDHDVCSNYGNWLYSAGIGNDPRENRKFNVVKQGLDYDGDGNYVRLWVP
ELAAIKSGKVHCIWTLSNTQLQAGGVLLGQTYPNPIIVAQEWSRHVNRPSSSTSTGASSGKGKKRSSGSQKSL
EHQGQYPGQKRGLDFYFSHTNPR
>Biomphalaria_gla_XP_013068586.1_photolyase
MPLCPPNYFGLVDGNIWSSFQVDAHNIVPCWVASPKLEYGARTIRSKIHKQLDEFLTEFPPLVKHPHSPEKMP
EETDWEKADSYLEVNRKVAEVSWAKPGAESGLRMLESFCEKRLKYFSSDRNDPNKQALSNLSPWFHFGQISVQ
RCILTVKLYRSKSNDSVNAFIEEAVVRRELADNFCFYNPHYDAVQGTNDWAKKSLDLHRKDKRPYLYSRDQLE
QSKTHDDLWNAAQTQLVNEGKMHGFLRMYWAKKILEWTKSPENALADAIYLNDKYNLDGRDPNGYVGCMWSIC
GIHDQGWAERAVFGKIRYMNYQGCKRKFDVPAFVRKYKRT
>Biomphalaria_gla_XP_013075939.1_cry-1-like
MSDIEKKNHVTIHWFRHGLRLHDNPALLDGLEDCVEFYPIFIFDGNVAGISTAAFPRMQFLLETLQDLDENLK
SHGSRLYSFQGDPVEIFKKLIEEWGVTRITFEQDPEPVWQDRDTRVKNMCLELEIECIERVSHTLWDPQQIIK
ENGGTPPLTYAMFCQVADIVGPPPKPREDPNFTGISLPVSEHHDEKYRLPTLAELGVEAECEKQASPYCHYLG
GESKAMKLLSIRLEKERKAFSLNQSLPNQLFPELSGMPMSLSPHLRFGSMSIRKFYWLLREAYAEVHPNAHIP
SSITGQLIWREYFYCMSVNNPNYNRMVGNPICLKIDWYDNDNHLKAWKMGMTGYPWIDACMRQLLQEGWIHQV
CRHATACFLTRGDLWIDWVKGLQVFDRYLLDADWSVCAGNWMWVSSSAFEKVLQCPKCICPVRYGRRMDPKGE
YVRRYVPELKDMPLQYLFEPWKAPLKVQEEAKCIIGEDYPLPIVNHKTASRDCQSRMEIIKSKFKDVPHIRPA
NEDEVYTSMWVCKELEKMDLCRHDNEKLLFNM
>Biomphalaria_gla_XP_013080710.1_cry-1-like
METVSYMLTAWHDGRLMWNTSLYSGIDTVKVPASELWTPDLVPYTSVCYVLLEATLRLLTARNNLNYLFCFFY
YFRFLLESLEDLDSSLRKLNSRLFVVRGQPADVLPRLFQEWGITHLAFEEDPEPYGKERDSAISALARELSIQ
VITRSSHTLYDPKVVIAANGNNPPLTYKRFQSILSKLDPPLQPCEALTSAMVAEVTTPVSDDHDDRFSVPSLD
ELGFDVDSLGPAQFRGGESEALARLYRHLERKAWVASFERPKMSPQSLYPSGTGLSPYLRFGCLSPRLFYWKL
IELYKKVNIVLPLLFLIQLLWREFFYTVATNNPNFDRMVGNSICVQIPWDHNPEALSKWAEGMTGYPWIDAIM
VQLRKEGWIHHLARHAVACFLTRGDLWISWEEGMKVFDEMLLDADWSVNAGMWMWLSCSAFFQQFFHCYCPVG
FGKRADPSGDFVRHYLPILKGFPTQYIYEPWTAPESVQKAAKCIIGKDYPLPMVQHTEACRVNLERMRQVYKR
LVLKSTASKRMFLDLPKIPKVSLFLSKAKMFLSSSSDEDMSQKQRRRKVDE
>Biomphalaria_gla_XP_013089589.1
MVVASFLVKNLNIHWHIGRDWFWNCLFDADLGNNSTSWQWVSGCGVDPVPYFRIFNPITQGEKFDKNGEYTRK
YVPELMYMPDSYLFKPWMAKESILKSANVVIGKSYPAPIVDLISSRNSAL
>Biomphalaria_gl_XP_013065770.1_cryptochro
MSKYKVIHWFRKGLRLHDNPAFLKAIEESTEIYPVFVLDPWFVTSSAIGINRWRFLLQSLVNLDSNLKMLNLR
LFVVKGKPEIEIKRLCQEWKINRLTFEVDTEPYAVQRDKIVEEIVQAMGVEVIKCVSHTLYDVHRTVQTNGGK
APISYQGFQAVLSKLGPPAPALPAPTVADLSGCKTPISPTHEKDYGVPTLSDLKKSETDCGPLLYPGGETEAL
DRMERHIKKINWICTFEKPNTSPNSIIPSTTVLSPYLKFGCLSARLFYHKLADVYSGKKHSQPPVSLHGQMLW
REFFYTVGAVTKNFDRMQGNSVCKQIPWGSNKEHLLAWTHAKTGYPFIDAIMTQLRLEGWIHHLARHAVACFL

TRGDLWISWEEGQKVFEELLLDADWSLNAGNWMWLSASSFFYQYFRVYSPIAFGKKTDPNGDYIKKYLPVLKK
YPAKYIYEPWKAPLSMQQTAGCVIGKDYPKPIVDHDTVRKENIKKMTEAYALDKQTKEQKVLPRNGHQIQDPN
QALQ
>Blattella_ger_PSN30513.1_cry-1
MSEETTNRSKVSVLWFRHGLRLHDNPALLEAIRDTDEFYPIFIFDGESAGTKAVGYNRMCYLLEALNDLNEQF
KKYGGKLYVFKGSPTQIFKRMWEELELGKICFEQDCEPLWYNRDSSVKTMCKELNINCVEKVSHTLWEPKAVI
ETNGGSPPLTYQMFLHTVSVIGPPPRPVSDVNWNTVTFGKLPDYLAEEFKMFPEIPHPEDFNIFREAKTEKLI
RWDGGETKALKQLLDRLDVEESAFRQGYYLPNQAQPDLLGPPTSLSPALRFGCLSVRRKFPSISVPNKSTIHH
LVCKVRKTGTFADKKRNRKRTVLTEEKLDDIGACFEQSPHKSLSKLTQQITHKDYNPHTLDELKENIRQQIAL
ISMDELQRVFRNFRKRCEACIRHGGRHVHDLFNEVHKGEPPACQNITGQLIWREYFYTMSVNNEFYAEMERNP
ICLNIPWHDGGDIETGHLAKWKQGKTGFPFIDAVMRQLLQIGQFVLAIGCGFQVLLLNNFWIVHIVSVLSIMD
VVLTHGEIIRYVPELNNYPVEYLYEPWKAPIEVQEKAGCVVGRDYPERIVDHLKASEQNRADMNKIILGNIYS
YRENRVILL
>Blattella_ger_PSN38921.1_C0J52_1186
MASKTKKMKLDMPASSSSQLSKTDFFLKLKEDRTTASESVMEFKFNKKRVRVLSEAAEVPERAEDNWAMLYAQ
KLALKNEVPLHVSFCLVPKFMDATIRHYQFMLKGLQEVAEECKDLNIQFHLLLGQAVNILPKFVMQHKMGAVV
TDFAPLRVPTKWVKDVQAALPKDVPFCQQAESILEVDRSVKAVTWATPGTKAGLEVLHSFCTSRLKLFASKRN
DPNANALSNLSPWFHFGGYNYLRKGLNTVVQVLEKGMYPLLSRIAALVAEAVRNSFQSASAIRTNSQFPGSTR
TALRRLKDEKLFARIAANKELLKDEHKLFRLAFSEENTNRNWNRLIFSDDVTFSSTNGGKVIVYRPRGAGYDE
RYVAKQIRRESVDAFCEEAIVRRELADNFCFYNEKYDSLDGAYDWAKITLEAHRKDKRQYLYTREELEKAVTH
DDLWNSAQLQLVQEGKMHGFLRMYWAKKILEWTESPKKALADAIYLNDRFSIDGRDPNGFVGCMWSICGIHDQ
GWAERPIFGKIRYMNYEGCKRKFDIAAYIIKWGGKKHKK
>Blattella_ger_PSN44595.1_cry-1
MENGIKTSVLPQARLEGLWLLDGFPFEICARFLVGILPIVPTTMFRRFGSEFAQTELASVCHKRTTCRCTPKT
IQAPLTYHQFQTVVASMGPPPPAEPSITQKSINGTYTPIGEDHDDKFGVPTLEELERKAWVASFGRPKMTPQS
LLASQTGLSPYLRFGCLSTRLFYYQLTDLYKKIKKACPPLSLHGQLLWREFFYCAATKNPKFDRMIGNPICVQ
IPWDKNAEALAKWANGQTGFPWIDAIMTQLREEGWIHHLARHAVACFLTRGDLWISWEEGMKVFEELLLDADW
SVNAGMWIRYLPVLKNFPTRYIHEPWNAPESVQKAAKCIVGKEYSLPMVNHAVASRINIERMKQVYQQLSKYR
GPGTEEESSAKGCTTVGLLATVPTSQSQSTNILNPMLMHREFKKSPPLEPKWERSPPDNAMNGHSPTYGEDPT
KGIQLNCYQLQQQQQMQIQNPHHQKQENEECE
>Bodo_sal_CUG26768.1_DNA_photol
MALRRTQLLWWASSSSRRLTRHLHNNPNNVFSLQDTVAIDDGIIHHFDSPPMSHVVTPPAFPQPMGSSVSTDD
EVMMQSASSVVAHATTEVAVTRRHAAQQRSTKALAAFHQRQLGILELWKSLPPLAAGYPAESSVGVCYWLNDL
RVHDHYPLALACRRAKETGGVPVVAMMCLDPRCFAQPSQILGCYRMGPERAKFTLECIASLRGELGALGVPLV
IRIGLPEVILPTLFAQLRCHHVYTTTQYAPHEKVIHDSLLQLTAAQVHSVWHTTLVHVDDLPTPLASMRTGFR
WFHDDAVMATIRPTKPYDCSDALKELPSVLPFFQAFDEDNGRTTRSTTHNSKLKTHNPSHQRAVGETSISEEG
MGALPTMADLGYGSYVPELAVTHVIATSSSHHGGERSATQRLEEWLEQGGMKRMVKLGVVKRPTVDMYATNLL
RISPYLSTGCMSPRRLYERLREYAYDNVMDGSSQSQFQEAMLRLFRRDYFHFQGLLHGNSMFYSYGPTPENTD
EAPDWRYDDKLLRRWCNGLTGFPFADAAMRELLATGWVGAPGRQALMWLLSIGLEQDWRAGAEWFERCSLDFD
PFVCYGNAAYYSKLTPDDYDDAVHTHHYLAGMHDQTGIYIKKWLPQLSKVPSVYIHRPHVMSERMQEMHGVFL
GKTYPYPIKLWDGAQSTMGPSQLTAYFETAEQRRSRGPMEALEFSRDKMAPLSSLHLTTGPQRYRNVASAMLH
ISENMEVDDDENVLVPASRQLKDDAMHLATMAHSINN
>Bodo_sal_CUG86883.1_photolyase
MSSKRSRSPSAEMSPTKAHRPDKMRINVFLFRRDLRVADNTALIHLAKQHPQTPILPAFFFNPMQIDPKQNPY
YGAPCVRFMFESLKDLNLQLDDRLVLLHGSDEECFKTLQKKFDIENISFNRDYTPFAQLRDAALDEWCKVNHV
QVCSSVQASLWDYSLLPLDCVKSGAEKPYAMFTPYYNKVMAAHLHQVPSPASESDTTAVKKLLISAKIKSTLF
DRVAESLIDSLPTNPDSPVQGGRTNALWRLEHVLPDMKQYETQRNFLSPQRTSLLSPFMKFGCVSVREVYAAA
AAAYGAKSCFVKELFWREFYAMLLFNNPSLCAGQLSPLSKKVKAEGNMPFQEKFAPFRWKWPETTQHWVAFQQ
GKTGVPLVDAAVRCLNATGWCHNRNRMVIGSFATKVLFVDWRICEQWFATKAVDYDVASNSGGWQWCSGQGAD
AMPYFRTFNPFQQAKKFDESCDFIKKWVPELKEVDKKVIIAWNEKGTTTLSKTTGYPLPIVDVRESTKRVIAE
YGIITSVTTXXXXXXXXXXXXXXXXXXXXXXXXXXXXXXXXXXXXXXXXXXXXXXXXXXXXXXXXXXXXXXXX
XXXXXXXXXXXXXXXXXXXXXXXXXXXXXXXXXXXXXXXFLYFIGARTTCTCSCSSVVNGFFTTLATLLKTHALE
HLRTLFPAAVDMQILYRVPVATHGDATVVHVNSESDFQVGLQVWNDSIRPLQLARQEPLLAAFEALASSGAPQ
TVISPLPPATGSGAPSSFVTPKKKSTRAPAPDNSTADVSSILPSTPQQVQQQPTAIGTDWYHSREKASNLLSS
FAPESPTDPPTIRITLRAGQASSRIRLHPYNRPDVNDLRQMVQSELLRRLTSAEQEQILSGMSNSTVKFLMYV
NTPAAPVDIDVEDNTDVTFVCDEIKKYGNDYSVQLMCEWVDATEVPCAREDVRLVETDLVEVVLPPRSALTFN
LAHTPKREHHEVKGIDMGEE
>Boleophthalmus_pect__XP_020781257.1
MSTARTIICLLRNDLRLHDNELLHWAQRHAEHIVPLYCFDPRHYEGTYYYNFPKTGPFRLRFLLDSVKDLRNT
LVTKGSNLIVRQGKPEDVVADLIKQLGTVSTVAFHEEITSEELNVEKNVKLVCAQMKIKVQSCWGSTLYHRDD

LPFNHISRLPDVYTQFRKAVESQSSVRPIFPTPEKLKPIPPGLEEGAIPTAEDLKQTEPIDDLRSAFPCRGGE
SEALARIKHYFWDTNAVATYKETRNGLIGVDYSTKLAPWLALGCISPRYIYHQIKTYEAQRTANQSTYWVIFE
LLWRDYFKFVALKYGNRMFHINGLQNKSVPWKKDMKLFNTWKEGRTGVPFVDANMRELAMTGFMSNRGRQNVA
SFLTKDLGLDWRMGAEWFEYLLIDYDVCSNYGNWLYSAGVGNDPRENRKFNMIKQGLDYDNNGDFVRQWVPEL
QAVRGSDVHMPWTLSSPALSNAQVSLGETYPVPIVMAPEWSRHVNKKQPGGASGSSHRGKKGPSHTPKQHKDR
GIDFYFSKGKGFP
>Boleophthalmus_pect__XP_020786820.1
MVVNTIHWFRKGLRLHDNAALRDSIRGSDSLRCIYILDPWFAGSSNVGINRWRFLLQCLEDLDASLRKLNSRL
FVIRGQPTDVFPRLFKEWQINRLSYEYDSEPFGKERDAAIQKLACEAGVELMVRISHTLYNLDKIIELNGGQP
PLTYKRFQGLINHMGPVELPAETITAEVIRTCVTPIGHDHDDKYRVPSLEELGFETEGLVTAVWPGGETEALM
RLERHLERKAWVANFERPRMNANSLLASPTGLSPYLRFGCLSCRLFYFKLTDLYKKVKKSSHPPLSLYGQLLW
REFFYTTATSNPCFDKMDGNPVCVQIPWDRNPEALAKWAEGRTGFPWIDAIMTQLRQEGWIHHLARHAVACFL
TRGDLWISWEEGMKVFEETLLDADWSVNAGSWMWLSCSSFFQQFFHCYCPVGFGRRTDPNGDFIRRYLPILRA
YPAKYIYDPWNAPEDVQKAAKCIIGLHYPKPMVNHAEASCINIERMKQIYQQLSCYRGLGLLATVPANLSNGT
SQGPVGPVEEPSVLERPPRTERPQSAQKRRHEEPLTEGNAKSWRPSK
>Boleophthalmus_pect__XP_020790376.1
MSEDVSTVQNKSVLNIQNSDQSSRQSSPSSKQNSSEPKRSKRRRQRSKGTGQQVVGLPTAPSSQPPVLLWFRR
DLRLCDNPAMIGALEIGAPVIPVFIWAPDEEEGPGTTVAMGGACKFWLHQALSCLRSSLERIGSSLVFLKAES
GSSLQTLRGLLQETGACTVVANALYEPWLKQRDDLVLSGLHKDGVGLKMYHSYCLRDPYSVSTVGVGLRGIGS
VSHFMSCCKQNPGPPLGATLDPPVSLPTPSHWPLGVTLDTLELAKMPRRKDGTIIDWAANIRKSWDFSEEGAH
AQLFTFLQDGVYRYEKESGRADYPNTSGLSPYLHFGQLSPRWLLWDAKEAKCRPPKFQRKLAWRDLAYWQLSL
FPDLPWESLRPPYKALRWSSNQSHLKAWQRGRTGYPLVDAAMRQLWLSGWMNNYMRHVVASFLIAYLHLPWQE
GYRWFQDTLVDADVAIDAMMWQNGGMCGLDHWNFVMHPVDAAMTCDPCGAFVRQWCPELADLPDELIHKPWKC
PASMLRRAGVVLGQSYPERIITDLEERRTQSLKDVAQVRLQFKQYVDQGSGCDLVPLPPRLFLLPLITRMEFK
HQLEEPDADAVSNPYNAVLKGYVSRKRDETIAFLNERDFTASVMNEGSQRRERMDVNYRKMEGLPQSREPRGR
ARRTPTAKDRFSIVPGAVVNTLR
>Boleophthalmus_pect__XP_020790995.1
MVVNSVHWFRKGLRLHDNPALQEALNGADTVRCVYILDPWFAGAANVGINRWRFLLEALEDLDNSLKKLNSRL
FVIRGQPTDVFPRLFKEWNVTRLTFEYDPEPYGKERDGAIIKMAQEFGVETLVRNSHTLYNLDRIIEMNNNNP
PLTFKRFQTIVSRLELPRRPLPPITPQQMDKCHTKISDNHDQLYNIPSLEELGFRTEGLPTAVWRGGESEALE
RLNKHLDKKVWVASIEHPRVSTCSLYASPTGLSPYLRFGCLSCRVLYYNLRELYMKLRKRCSPPLSLFGQLLW
REFFYTAATNNPNFDRMEGNPICVQIPWDQNPEALAKWAEGRTGFPWIDAIMTQLRQEGWIHHLARHAVACFL
TRGDLWISWESGMKVFEELLLDADWSVNAGSWMWLSCSAFFQQFFHCYCPVGFGRRTDPTGDYIRRYIPILKD
YPNRYIYEPWNAPESVQKAANCVVGVDYPKPMINHAEGSRLNIERMKQVYQQLSHYRGLSLLASVPTIQEEAE
PPMTDESQTSSGPDSPPKLSDVEAAGCSTAPDSSTMDPFSFSVSVPYTALDETTCPKNEPPTAISSTSPDPMT
AQPKPSSPASPSPNVAPSPGRSTSLGPRRKGMARKTRRSQRQRVRQNCATATKEGDRRTDDESRDSTTTEDKM
EEEIEQDDEKMEEEMLGGESTGHLQ
>Boleophthalmus_pect__XP_020795196.1
MVTCLIQSEFSLCMRYTFFRTFCVTAPKGLLKCPLNTPLCPHFDQFHSFRCKPIMSQKRKATTDAGAEKPNKQ
PKITSEKKAAGWLQEQVLKQRAEKKEMKFNKKRLRFLTDTQKIKQGAEGILYWMNRDQRVQDNWALIHAQRLA
LKEELPLHVCFCLLVPKSKLSTYRHFSFLLKGLEEVSQELKTLGIEFHLLHGPPGEVLPGFVSDRSLGAVVTD
FSPLREPLQWLEDLKKKLPKDIPFIQVDAHNIVPCWEASPKLEYAARTIRGKINKVLPEFLTDFPLVEKHPYI
AIRTAKQVDWAKVLASLQVDKTVGETDWAKPGTKAGMAMLESFIDVRLKLFATQRNDPNAAALSQLSPWIRFG
HISAQRVALQVKKKGGESSAPFIEELVVRRELTDNFCYYNKKYDSIKGAYDWAQKTLKDHAKDKRPYVYTQDQ
LEKAKTHDDLWNGAQFQMISEGKMHGFLRMYWAKKILEWTSSPEEALSIALYLNDRYSLDGQDPNGFVGCMWS
ICGIHDQGWAERPIFGKIRYMNYKGCQRKFDVARFEKKYRPKDL
>Boleophthalmus_pect__XP_020797397.1
MSHTCIHWFRKGLRLHDNPALIAALKECKELYPVFVLDPYLYNNAFGLNRWRFLIGSLRDLDCSLKKLNSRLF
VVRGKPEDVFPGLFKKWKVSKLTYEYDTEPYSLSRDQTVTKLAKEHGVEVIYKVSHTLFDINRIIEANNGKPP
LTYNRMQAIVSDLGSPKXXXXXXTMDDVKNMSTPYSKDHEEEFEVPKLDELGHDISSIPEELFPGGEVEALKR
LDEHMKKTQWVCNFEKPQTSPNSLIPSTTVLSPYVTFGCLSARTFWWRLADVYQGKKHSSPPVSLHGQLLWRE
FFYTASLGIPNFNKMKDNPVCTQVDWDNNPEFLAAWREARTGFPFIDAIMMQLRTEGWIHHLARHAVACFLTR
GDLWISWEEGQKVFEELLLDGDWALNAGNWQWLSASTFFHQFFRVYSPVAFGKKTDKNGDYIKKYLPLLKKFP
PQYIYEPWKAPRSVQQAAGCIIGKDYPKPIVEHEVVSKKNIQRMKLAYAKRSADKPESPSKKQGVKRKAPSVV
DMLNKKAKQ
>Bombycilla_garr__NXN81953.1
MGVNAVHWFRKGLRLHDNPALRECIQGADTVRCVYILDPWFAGSSNVGINRWRFLLQCLEDLDANLRKLNSRL
FVIRGQPADVFPRLFKEWNIAKLSIEYDSEPFGKERDAAIKKLASEAGVEVIVRISHTLYDLDKIIELNGGQP
PLTYKRFQTLISRMEPLEMPVETITPEIMKKCTTPVSEDHDEKYGVPSLEELGFDTDGLPSAVWPGGETEALT
RLERHLERKAWVANFERPRMNANSLLASPTGLSPYLRFGCLSCRLFYFKLTDLYKKVKKNSSPPLSLYGQLLW

REFFYTAATNNPRFDKMEGNPICVQIPWDKNPEALAKWAEGRTGFPWIDAIMTQLRQEGWIHHLARHAVACFL
TRGDLWISWEEGMKVFEELLLDADWSVNAGSWMWLSCSSFFQQFFHCYCPVGFGRRTDPNGDYIRRYLPVLRG
FPAKYIYDPWNAPESVQKAAKCIIGVNYPKPMVNHAEASRLNIERMKQIYQQLSRYRGLGLLATVPSNPNGNG
GLMGYSPGESISGCGSTGGAQLGTGDGHSVVQSCALGDSHTGTSGIQQQGYCQASSILHYAHGDSQQSHLLQA
GRTALGTGISAGKRPNPEEDTQSVGPKVQRQSTN
>Bombycilla_garr__NXN82933.1
CIYILDPWFAASSAVGINRWRFLLQSLEDLDNSLRKLNSRLFVVRGQPTDVFPRLFKEWGVTRLTFEYDSEPF
GKERDAAIVKLAKEAGVEVVIENSHTLYDLDRIIELNGHKPPLTYKRFQAIISRMELPKKPVSTVISQQMETC
KVDIQENHDDVYGVPSLEELGFPTDGLAPAVWQGGETEALARLDKHLERKAWVANYERPRMNANSLLASPTGL
SPYLRFGCLSCRLFYYRLWELYKKVKRNSTPPLSLYGQLLWREFFYTAATNNPKFDRMEGNPICIQIPWDRNP
EALAKWAEGKTGFPWIDAIMTQLRQEGWIHHLARHAVACFLTRGDLWISWESGVRVFDELLLDADFSVNAGSW
MWLSCSAFFQQFFHCYCPVGFGRRTDPSGDYVKRYLPKLKGFPSRYIYEPWNAPESVQKAAKCIIGVDYPKPM
VNHAETSRLNIERMKQIYQQLSRYRGLCLLASVPSCVEDLSGPVTDSASGQSCSTSTAVRLSQADQASPKRKH
EGAEEPCPEELYKRAKVTGLPASEIPGKS
>Bombycilla_garr__NXN85679.1
DNWAFLYAQRLALKQELPLHVCFCLVPKFLEATIRHYRFMLRGLQEVAEECAELNISFHLLLGYAKDVLPTFV
AEHGVGGLVTDFSPLRLPRQWVEDVKERLPEDVPFAQVDAHNIVPCWIASPKQEYSARTIRGKIHAQLPEFLT
EFPPVVRHPHPPSCAAEPIAWEACYSSLQVDHTVKEVEWATPGTAAGMAVLKSFIAERLKSFSTHRNDPNKAA
LSNLSPWLHFGQVSTQRAILEVQKHRRHYKDSVDAFVEEAVVRRELAENFCYYNENYDSVQGAYDWAQTTLKL
HAKDKRPYLYSLQELEQGTTHDPLWNAAQLQMVGEGKMHGFLRMYWAKKILEWTRSPEEALQFAIYLNDRYEL
DGRDPNGYVGKCRMGTRGCLWSICGIHDQGWAERPVFGKIRYMNYAGCKRKFDVEQFERRYAPTH
>Bombycilla_garr__NXN88810.1
LCSPSVLKVDYDVCSNYGNWLYSAGIGNDPRDNRKFNMIKQGLDYDGNGDYVRLWVPELQGIKGADIHTPWAL
SSAALSQAGVTLGETYPWPVVTAPEWSRHIPQRPGGSPHPRGRRGPAQHKDRGIDFYFSRKKDV
>Bombycilla_garr__NXN90577.1
QAKHHSLPPVSLQGQLLWREFFYTVASATPNFSQMAGNPICLQICWYKDTEKLHKWKMAQTGFPWIDAIMTQL
RQEGWIHHLARHAVACFLTRGDLWISWEEGMKVHCWLLFSSVLPSP
>Bombyx_mor_NP_001182627.1_cry_2
MSAAPETLPPPSAQAHTPARPTHMSAPRRTPGKHTVHWFRKGLRIHDNPALREGIIDAVTFRCVFIIDPWFAS
SSNVGINKWRFLLQCLEDLDKSLKKLNSRLFVVRGQPADALPKLFREWGTTALTFEEDPEPYGRVRDHNIISK
CREVGITVTSRVSHTLYKLDKIIERNGGKAPLTYHQFQALIASMPPPPPAEVTITPQMLNGATTPITDNHDDR
FGVPTLEELGFETEGLKPPIWIGGESEALARLERHLERKAWVASFGRPKMTPQSLLASQTGLSPYLRFGCLST
RLFYYQLTELYKRVKRVRPPLSLHGQILWREFFYCAATRNPNFDRMEGNPICVQIPWEKNQDALAKWANGQTG
YPWIDAIMIQLREEGWIHHLSRHAVACFLTRGDLWISWEEGMKVFDELLLDADWSVNAGMWMWFSCSSFFQQF
FHCYCPVRFGRKTDPNGDFIRKYIPALKNMPTRYIHEPWMAPESVQAAAQCSIGRDYPMPMVDHTKASQINIE
RIKQVYAQLARYKPQATLNANAVQRPNVLQSSPSPTSIIASINQSNFLCSQTPDPQNATSHSFKEPSDTFPIN
KKNKDTESNQTRYKRVIIVQKTRNSNVEINVSAPQDRRASDNYITINEGDPAHSTDAKKQENNYDFKNLAIDN
HIRGFNNIQNINSQYYLTNYSTKKKETEIKNETDEGPFVRPESYGTDRAYTINRCPSMSNEKEFETPKENLKQ
>Bombyx_mor_NP_001182628.1_cry_1
MLGGSVLWFRHGLRLHDNPSLHSALEETSGPFFPIFIFDGETAGTKVVGYNRMRYLLEALDDLDKQFKKYGGR
LLLVKGKPSAVFRRLWEEFGIRKLCFEQDCEPVWRPRDESVKTACREIGVTCREHVSHTLWEPDTVIKANGGI
PPLTYQMFLHTVATIGDPPRPVDNAKLRGIKFGTLPLCFYEEFTVYDKVPNPEDLGVFLENEDIRMIRWVGGE
TAALKQMQHRLAVEYETFCRGSYLPTHGSPDLLGPPISLSPALRFGCLSVRKFYWSLQDLFQQVHQGSLCSTQ
YITGQLIWREYFYTMSVNNPHYGQMTDNPICLDIPWKSPEGDELERWASGRTGFPFVDAAMRQLRLEGWLHHA
VRNTVASFLTRGTLWLSWEHGLAHFLKYLLDADWSVCAGNWMWVSSSAFEALLDSGECACPVRLGQRLDPSGE
YVRRYVPELARVPTEYIYEPWKAPLDVQERANCIIGKDYPAPVVNHIVAAQRNRNAMEDTKMEKNHAKQRGRP
LASSNSGRRWNSPLDYQQTGREKIY
>Bombyx_mor_XP_004930027.2_photolyase
MLHIPLVNQFNTVLKRYSVFIMASASKKRKTSIPMALGKSNESTSTIEEFLKKLQKKREETAESILNFDFNKK
RIRIISQEQIIPDDCDGVVYWMSRDSRVQDNWAFLFAQKLALKNEVPLHVCFCLISKYLDASLRQFHFLVKGL
EKVAAECKKLNIQFHLLEGSGAEVLPQWVVDHNIGAVVCDFNPLRVPMGWLDGVKGKLRKDVPLIQVDTHNVV
PCWVASDKQEYSARTIRNKINSKLDEFLTEFPPVIKHPYSSKFEAEPIDWHEAIETREADKSVGPVDWAKPGY
DEAVKMMKSFLDKRLNIYASKRNDPTQDALSNLSPWFHFGQISVQRVALCVQEYKSKFTESVNAFLEEAIVRR
ELADNFCFYCEHYDSVKGASAWAQKTLDDHRKDKRTHIYTLEKLSKGETHDDLWNSAQLQLVKEGKMHGFLRM
YWCKKILEWTPTPEDALKYGIYLNDHYSIDGRDPSGFVGCMWSICGIHDQGWAERAVFGKIRYMNYDGCKRKF
DIKAFIARYGGKVHKYVPKK
>Bombyx_mor_XP_037874830.1_cry-1
MSKTPTVIHWFRLDLRIHDNLALRNAINEAENRKHLLRPIYFLDPNIKDKVGINRLRFLLQSLEDLDNNLKKL
NTCLYVLRGKAVDLLPKLFDDWQVKYLTCQVDIDPEFVQQDEYIEDIAEKKGVFINKRVQHTVYDVHKVLREN
NGAVPLTYQKFLSLVKSINVKEPIEISNVLSSHCKPIDIQSENYSIPNLKELQIDEETLAPVKYHGGETEALK

RLNLYMSKKEWVCKFEKPNSSPNSIEPSTTVLSPYISHGCLSAKLFYYKLKEVENGRQHTLPPVSLMGQLMWR
EFYYTAGTGVANFDKMVGNAICIQIPWTKNDAFLKAWAEGKTGYPFVDAIMRQLKQEGWIHHLARHMVACFLT
RGDLWISWEEGAKIFEDYLLDYDWSLNAGNWMWLSASAFFYKYFRVYSPVAFGQKTDKEGVYIKKYVPELKKY
PRE
>Bos_taur__DAA29534.1
MGVNAVHWFRKGLRLHDNPALKECIQGADTIRCVYILDPWFAGSSNVGINRWRFLLQCLEDLDANLRKLNSRL
FVIRGQPADVFPRLFKEWNITKLSIEYDSEPFGKERDAAIKKLATEAGVEVIVRISHTLYDLDKIIELNGGQP
PLTYKRFQTLISKMEPLEIPVETITSEVIEKCTTPLSDDHDEKYGVPSLEELGFDTDGLPSAVWPGGETEALT
RLERHLERKAWVANFERPRMNANSLLASPTGLSPYLRFGCLSCRLFYFKLTDLYKKVKKNSSPPLSLYGQLLW
REFFYTAATNNPRFDKMEGNPICVQIPWDKNPEALAKWAEGRTGFPWIDAIMTQLRQEGWIHHLARHAVACFL
TRGDLWISWEEGMKVFEELLLDADWSINAGSWMWLSCSSFFQQFFHCYCPVGFGRRTDPNGDYIRRYLPVLRG
FPAKYIYDPWNAPEGIQKVAKCLIGVNYPKPMVNHAEASRLNIERMKQIYQQLSRYRGLGLLASVPSNPNGNG
GLMGYSPGENIPGCSSNASCTQGSGILHYAHG
>Bos_taur__NP_001276715.1
MAAAAAAATQAPAARGDGASSVHWFRKGLRLHDNPALLAAVRGAHCVRCVYILDPWFAASSSVGINRWRMMKN
EATQSLLRVLTLQWRKLASGGQLPCHMEWGVTRLTFEYDSEPFGKERDTAIMKMAKEAGVEVVTENSHTLYDL
DKIIELNGQKPPLTYKRFQAIISRMELPRKPVGSVTSQQMEGCRAEIQESHDETYGVPSLEELGFPTEGLGPA
VWQGGETEALARLDKHLERKAWVANYERPRMNANSLLASPTGLSPYLRFGCLSCRLFYYRLWDLYKKVKRNST
PPLSLFGQLLWREFFYTAATNNPRFDRMEGNPICIQIPWDRNPEALAKWAEGKTGFPWIDAIMTQLRQEGWIH
HLARHAVACFLTRGDLWVSWESGVRVFDELLLDADFSVNAGSWMWLSCSAFFQQFFHCYCPVGFGRRTDPSGD
YIRRYLPKLKGFPSRYIYEPWNAPESIQKAAKCVIGVDYPRPIVNHAEASRLNIERMKQVYQQLSRYRGLCLL
ASVPSCVEDLSNPVAEPSSIQAGSSSSVGPRPLPGGPASPKRKLEAAEEPPGGELSKRARVAESLPSELPSRG
V
>Brachionus_caly__CAF0775459.1
MPKTGIHWFRHGLRIHDNPALVACQDEVERFIPIFIFDGKTAGTDVCGYLRFRFLLQSLQDLENQFASNGINF
MCFHGEPHEILEKLIKEWEVRVISFEKDSESTWHKRDILVKEMCSKNNVQVIELVSHTLYDPEEIFSINSNSP
PNTCEELRKSCYKIGNPEQPIPRPDLKFLSAHLLSTNDLYIPEIHKVPNVEYFGLKPECKEQEFCLFPGGETK
ALELCHSRIQYEKESFKSGKINPNLSKPVLFTKEISLSPYLRFGCLSIRKLYWDLKKAYVKNFTGPRNLYCPA
EQLFWREYFYQLSYKNENFAQVDGNHMCFKIPWDYYGKQDLFLKWEQGQTGFPWIDACMRQLVQEGWIHHVCR
NSVALFLTRGVLFLSWERGMKTFFKYQIDADWSVCSGNWNWIATGDPEEILNSANTFCPVKYGKKIDPQGNYI
KKYVTELKDFPLEYLNEPWKAPLDIQEKSNCIIGKDYPEPIVDYEEAFKENIAKLQQFFHSEKKEIFETFLND
KNALKPANSQEYRAYTFAKFLESEKSSKQSSKLSARDNSSVPDLEQFL
>Brachionus_caly__CAF0961683.1
MPPKKSIKTEKESVNDTKPLIKTEIKKEPEPTQSLNSDNLKMEKVTNCIYWFRKALRLHDNPSLLNAIEKSQY
IYPIFILDPWFVQHSKVGPNRWRFLLESLNDLDEQLKRKNSRLILIRGQPKKVFTEKIKEWNINLICFESDTE
PYAKKRDEEITSLANELGVRVETKYGHTLYNPEYVYKKNNGKVPLQYQSFLGILAKIGDPPKPLDELSGMFNK
IEIDYDLYKVPDLESIGVDSSKCGPRLYPGGETEALRRFEEKFKNDSWVCAFEKPNTSPNSLQPSTTVLSPYL
KFGCLSSKKFYWRLKQVYAKKKHSLPPVSLEGQLLFREFFYYVGSYTPNFDKIEGNTVCRQIKWDDKPEYVQA
WKEARTGYPFIDAIMTQLRTEGWIHHLARHAVACFLTRGDLYCSWEKGQEVFEEYLLDQDWSLNAANWQWLSA
STFFHQYFRVYSPIAFGKKTDDNGDYIRKYLPILKSFPTKYIYEPWTAPLEIQKRHNCIIGKDYPRPIVDHNI
ISKINISRMKKAYEETKATAAADDGEEEDGKKTKPTTSKSVKTVAKSEKTSPDSRPPSKRAKK
>Brachionus_caly__CAF1108553.1
MSKVPSGVKKASSKQLKLDKLVIQSPGKTSRPKFDEDSPEQEPPAKKPNSRLNSEDETDSKKTNFPIFKSAFQ
APNLEAISECTLAEEIRKKRHELFESVNAFRFNKKRVQVLTEVKEFPENSKGILYWMSRDQRVQDNWSLLYAQ
KLALKQNLPLYVCFCLVPTFLNATIRHYYFMLEGLKEVEKELNELNIQFHLLIGQAKDQVPKFIQENKIAGLI
CDFSPLRIHKNWVEELKKNLPDNIPFAQVDAHNVVPCWIASDKLEYAARTIRNKINSKLDDYLTEFPPVIKHP
VVSEIEKKTIDWEACYSSLQVDQSVKVVKWARPGYLGAIKNLEDFIKNKIKFYDSERNDPNKNALSNMSPWYH
FGQVSVQRCMLEVKKYASKNSEAVKALMEESIVRRELADNFCYYQPNYDNLEGAYDWAKLTLEAHRKDKRPAL
YSLSELENFKTSDRLWNASQIQLRVEGKMHGFLRMYWAKKILEWTESPEQGLEYAIYLNDKYSLDGRDPNGYV
GCMWSICGIHDQGWAERAVFGKIRYMNYEGCKRKFDVERFITRYYARKSEI
>Brachionus_plic__RNA12022.1
MSKLPTNVKKAATKQLKLDKLVIQSPGKTARARSKSPEQGPPRKKPNSQLNSEDEVEAKKQETKQTFPVFQSG
MAASSTGNTKSPCQLAEEIKCKRNELFESVNAFRFNKKRVQVLTEAREFPEDAQGILYWMSRDQRVQDNWALL
YAQKLALKQNLPLYVCFCLVPTFLNATIRQYHFMLEGLKEVQKELTDLNIQFHLLIGLAKDQVPKFVAEHKIG
GLICDFSPLRTHKNWLQEMRKNLPSNVPLAQVDAHNVVPCWIASDKLEYAARTIRNKINSKLDEYLSEFPPVI
SHPVPAQIDCKPIDWDACFKSLECDQSVKIVKWASPGYLAGIKNLEDFIQNRLKNYDSDRNDPNKNSLSNMSP
WYHFGQVSVQRCMVEVKKYASKFSNSVKGLMEETIVRRELADNFCYYQPNYDNINGANEWAKISLDAHRKDKR
PALYSLSELENFKTADKLWNASQIQMRVEGKMHGFMRMYWAKKILEWTESPEQALEYSIYLNDKYNLDGRDPN
GYVGCMWSICGIHDQGWAERAVFGKVRYMNFEGCKRKFDIERYCLKYMAKKSEI
>Brachionus_plic__RNA16403.1

MPKTGIHWFRHGLRLHDNPALLACQDEVERFIPIFIFDGKSAVFLPCTLLTGTETCGYVRLRFLLQSLQDLES
QFKEHGINFMCFHGEAHNVLENLIKEWEVRVISFEKDSESIWHKRDNLVKEMCARHNVQVIECVSNTLYDPEE
VFSLNSNQPPNTCEELRKSCYKIGEPEQPVPRPDLKFIASHLVSTNDLYDPVRHKIPDLDFFNIKPENEKQKK
CIFIGGETKALELCKIRIQYEKESFLAGKLNPNLSKPVLFTKEVSLSPYLRFGCLSVRKLYADLKKVYVKNFN
GLKNVYCPVEQLFWREYFYQLSYKNEKFAQVDGNPMCFKIPWDYHGKQDLFLKWESGQTGFPWIDACMRQLVQ
EGWIHHVCRNSVALFLTRGVLFMSWERGMRTFLKYLIDADWSVCSGNWNWIATGDPEEVLNPTVTFCPVKYGK
KIDPKGEYIKNYVPELREFPLEYLYEPWKAPLDMQEKAGCVIGKDYPEPIVDNEQAFRENIAKLQQFFHSERK
EIFETFLNDKNALKPANSKEYRAYTFAKFLESEFEDF
>Brachionus_plic__RNA17082.1
MSDLQKQRLGILWFRNDLRLNDNLSLDKTIHLVKEKKLDLVLPIYCFDKLLFEGRSREAQLPKLGPFRRNFLI
ESVENLKEKLQKKLNSNLCILYGEQDVEIKKLIEKILTENPGIHIEYLIASREVPSDEVDLENKVKKYLDSKR
IKNFFLWDNLMIHPDDLPFGHFSKTPDMFTQFRKSVELKESNYNVRKSVQIESNFSLPTFSQNDIVGQSIPEK
AKITESPKSAILGMKGGEDQAELRMQQYFFKTDGLFKYKTTRNGLVGTEYSSKLSMWLALGCISPRYLYWKVK
EFETMKCSNESTKCLAFELLWRDFFKYHSLKFGKRIFYQNGCYTGAAGYQMNWKKDQRLFEKWINGETGYPFV
DANMKELKETGWMSNRGRQNVASFLTKDLEIDWRFGAEYFETMLIDYDCTSNWGNWQYVAGVGTDPRADRYFN
VIKQAYDYDQNGEFVKLWIPALQNVSDEFVHCPFRMSLVEQKSFNCILNKDYPLPIVQMKHKWNPYSNKANKF
AARQKRAK
>Brachionus_plic__RNA42711.1
MAPKREKIKNESDPSLGFGLKNEPSEPINATDMEKTAKCIYWFRKALRLHDNPSLMHAIQKSQYIYPIFILDP
WFVQKAKVGPNRWRFLLQSLNDLNEQLIRKNSRLILIRGNPSAVFAEKIKEWDINLICFETDTEPYAKLRDQE
ITKIATELNVKVETKYGHTLYNPDYVYKKNNEKIPLQYQSFVGALAKIGDPPKPIDEPSNVFNKLEIDYDLYK
IPDLVSIGIDPSACGPLLFPGGETEALRRLEDKFKNESWICSFEKPNTSPNSLAPSTTVLSPYLKFGCLSARK
FYWRLKQVYAKRKHSMPPVSLEGQLLFREFFYYVGAFTPNFNKIEGNPVCRQIKWDDKPEYVKAWKEARTGFP
FIDAIMTQLRTEGWIHHLARHAVACFLTRGDLYCSWEKGQEVFEEYLLDQDWSLNAANWMWLSASAFFHQYFR
VYSPIGFGKKTDENGDYIRKYIPVLKTFPAKYIFEPWTAPLEVQKRHNCVIGKDYPRPIVDHNVISKINISRM
KKAYEESKLVSTDDGDDKKSRPTSSKSTKNPPKSEKTSPDTRPPSKKSKN
>Bradysia_cop_XP_037034936.1_cry_DASH-l
MGKFKNCLVWLRNDLRFHDNEALLWAHENAETITPLFCFDPRMFATTHRYQFPKTGNFRSKFLIECVDDLNRT
IKAKGSGLIILSKRPEEALAFIRGKCKFEAIAFQKEITKEEINVERECENFCLQNNITFQAIWGSTLYHKDDL
PYDVKSTPDTYTQFRKDVETRSKVRTEKRMPEKLKPLPAELVGISEKIPLLQDLKLEEPNLHANSAFPFNGGE
TAALHRLKSYLWDTQAVEKYKITRNGLLGSEYSTKLSPWLAFGCISPRRIFYEIRKYETERVKNDSTYWVIFE
LLWRDYFRFVAVKYGNSLFYSGGIKKMKYKWTEDMEQFKKWADGKTGVPFVDANMRELLQTGWMSNRGRQNVA
SFLTKDLKLDWRLGAEWFEYLLIDYDVCSNYGNWNYSAGIGNDPRADRKFNMIKQALDYDAEGEFIKLWIPEL
VNIHKTGIHAPWTLRQGELGGVKLGIDYPNPMVTAQEWSRHLHKSKSSDWAAASKSKQKGHDFYFKPNTSRK
>Bradysia_cop_XP_037040970.1_cry1-like
MTRSKFNSQLSGEKHTVHWFRKGLRLHDNPAIIEGLTGSKTLRCIFIIDPWFAGISNAGINKWRFLLQCLEDL
DQNLRKLNSRLFVIRGQPADALPKLFKQWGTTCLTFEEDPEPFGKVRDDNIAALCKESNISVIKTVSHTLYDL
ETIIELNGGEAPLTYNQFQAIVASMEQPAAAVPTVTLEHLSTVFTPISEDHDEKYGVPTLYELGFETDNLQPP
VWIGGETEALIRLERHLERKAWVASFGRPKMTPQSLLASQTGLSPYLRFGCLSTRLFYHQLSDLYKKIKKACP
PLSLHGQLLWREFFYCVATKNSNFDRMQNNPICVQIPWQKNPDALVKWAMGFTGFPWIDAIMTQLRNEGWIHH
LARHAVACFLTRGDLWISWEEGMKVFDELLLDADWSVNAGQWMWLSGSSFFQQIFHCYCPVRFGRKADPNGDY
IRKYLPCLKTFPLRYIHEPWSAPEDVQRVAKCIIGQDYPLPIVNHIRASRINMDRMRQAYQQLSKFRNANNTN
NKRGKQLSVNLNTSNDNVPAVQQLDKGFLHPQRISIPSDSTYRQSYAHQLHQFKPELFAENVNYNSKRENSHT
TVLAYSYNRQTHNDDKFENFDFSASSLNANASNYVDDDQKDGGHSKHRKCELNENFDSFMEAENENDPTRMT
>Bradysia_cop_XP_037041975.1_cry1-like
MDKQQKTIIHWFRKGLRIHDNPALAEAIDLIRKSPNKYVLRPIFVLDPEIPNWLKVGPNRWRFLQESLLQLDS
NLRKIKSRLYVVRGDPKVIFPKLFNEWQVELLTFESDIEPYARQRDMLVEKQAKKFNVNVITTVSHTIYNPEL
IIQKSQGIAPKQYEGFLKIVSTMSIPEPLEAPAALPAKSKPSKDSNEHTDSKCYDCPALHELGVNEADLGDVL
YPGGETEGLKRFQAILRKPDWICGFEKPKTSPNSLKPSTTVLSPYLKFGCISSRLMYSDLQKVLKNKKKHTTP
PVSLIGQLLWREFFYTAAASDPNFNRMVGNSFCRQIPWKKNVQHLEAWTYGRTGYPFIDAIMRQLRQEGWIHH
LARHAVACFLTRGDLWISWEDGQRVFEELLLDADWALNAGNWMWLSASAFYHLYYRVYSPVSFGKKTDKDGEY
IRKYVPELANYPKSMIYEPWTATAAQQREFGCIIGKDYPQRIVIHDVVLKKNMAKMADAYQKHRDGGGGGKFD
VDNTQAKPEPQSPKTEPVSPSTSSGCSSSKILKMEQNSTIVKYFKKDPKSP
>Bradysia_cop_XP_037043249.1_deoxyribod
MRHLNEIRLIVVYAKHLKINLSMSSSSISPPPKKRNKSNNPSTSSEVSTEVDSKGRPKSVNFKEQFKNARIQT
AESVLDFKFIKSRVRILSANGNVPESARGIVYWMSRDARVQDNWAFLFAQKLALKNEVPLHICFCLVPKFLDA
TIRHYKFLLNGLKEVEAECSALNISFHLLIGTAATTIPKFVKDNGIGAVVCDFSPLRVPVQWVDDVLHKLPKT
VPLVQVDAHNIVPVWETSDKQEYAARTIRGKINKKLDNFLTDFPPVIRHPYESDAKKIKIDWQTAFDSLEVDM
TVDEVDWAKPGYSHGVEEMEQFCNHRLKIFNDKRNDPLANALSNLSPWFHFGQISVQRTILTVKKYKKLYPSS
VDAYVEEAVVRRELSDNFCFFNKNYDSINGLSGWAKQTLIDHKKDKREWIYTRKQLDSSQTHDDLWNSAQIQL

RLEGKMHGFLRMYWAKKILEWTNSPEEALEIAIYLNDRYSLDGRDPSGYVGCMWSIGGVHDMGWKERAIFGKI
RYMNYKGCEKKFDVKAFVSRYGGKVHSKGKKK
>Branchiostoma_belc__XP_019616322.1
MSLRLFISSVPINSLFLARTKFVHRASILNFLCAKKSPFVLGKQYIAVSSRNMSSGDKRKSSGSGESPSKKAK
VENGASSSSQDGAGSEDFLQQIADRRTGVCESVAGFKFNKKRCRQMSDGGDLKQNCGGILYWMSRDQRVQDNW
ALLFAQRLALKHKVPLYVCFCLVPKFLDATIRHYGFMLKGLEEVEKELTELDVSFHLLTGHAVDVLPEFVSEK
NIGGVVTDFSPLRTPMKWVHDLAGKLPSDVPFVQVDAHNIVPCWEASPKQEYGARTIRNKIHNQLSSYLTEFP
PVCKHPHPTKDKPEPTDWAAARASLKVDMTVAEVTWATPGTTGGLRQLESFCKQRLKNFGTDRNNPNKRALSN
LSPWIHFGQISVQRCILTVKKYRSKYKESVDAYIEEAIIRRELSDNFCFYNHDKYDSIKGAAAWAQKTLKDHA
GDKREYLYSREQLEQAKTHDDLWNAAQIQLNKEGKMHGFLRMYWAKKILEWTESPQVALKEAIYLNDKYSLDG
RDPNGYVGCMWSICGIHDQGWAERPVFGKIRFMNYNGCKRKFDVAQFVTKYKAKGFLKK
>Branchiostoma_belc__XP_019620932.1
MEALSVFTSKSSTHFCLISTEMTNKEVGMEVQEQHNTIHWFRKGLRFHDNPSLLHALRTSRHVYPVYILDLDF
MKDFKIRCGANQWRFMIQCLEDLDERLRKYGLRLFVVRGSAETFFREQFRRWNITQLTHDVETEHYHRFRDAA
VRKIATDAGVEVVNYVAHTLYNIDKIIEYNGGTAPLTYRKFQKVLRDFGAPPQASEMATAEHFANCSLPPEDL
HDEKYDMVTLEELGVKCENPFKFVGGETEGLRRVEKHLQNQGWITQFEKPKTAPTSLLPSTTGLSPYFSVGCL
SVRYFYHRLNNIYAKAKDHSLPPVSLQGQLLWREFFYVAAAQTPNFVVMAGNPICLQIPWYENKEHLRRWRHA
ETGFPWIDAIMSQLQQEGWIHHLARHAVACFLTRGDLWISWEEGQKVFEELLLDADYSVNAGNWMWLSGSAFY
HQYTRIFCPVRFGKRTDPNGTFIRAYLPVLKDFPAQYIYEPWKAPLKVQEAAGCIVGRDYPFPMVDHEIVSQQ
NLELMREIREQQRQITLLTLEGREDESSTSTDGGDSRKRKRPVHPANDDEIIKVVVTTAL
>Branchiostoma_bel_XP_019620887.1_MCRY
MKSTIHWFRKGLRLHDNPSLRDALQMLQPGDVWRCVYVLDPWFAGSSNVGVNRWRFLLQCLEDLDASLRKLNS
RLYVIRGQPTDVFPRLFKEWKVSCLSFEEDSEPFGRERDMAVMKLAKEAGVKVILRTSHTLYKLQDIIDVNGG
QPPLTYKRFQAILSKMDPPDQPVDTITASTVDKLTTPIQDDHDEKYGVPSLEELGFDTDNLNPVVWPGGETEA
LTRLERHLERKAWVASFERPKMTASSLLASPTGLSPYLRFGCLSPRLFYKKLTELYKKVKRSNHPPLSLYGQL
LWREFFYTAATNNPNFDKMVNNTICVQIPWDKNPEALAKWAEGQTGFPWIDAIMSQLQQEGWIHHLARHAVAC
FLTRGDLWISWEEGMKVFEELLLDADWSVNAGSWMWLSCSSFFQQFFHVYCPVNFGRRTDPNGDFIRKYLPKL
RGFPAKYIYDPWNAPESVQKAAKCIIGKDYPLPMVNHAETSRINVERMKQVYQQLSRYRGAGTQCLLATIPYP
DGGNGNPTGHKKHALVQGMERPHGERREREHGQPAHMPFLKRRRWDIRFQAPEAIDQSEGDMSNVD
>Branchiostoma_floridae_XP_035688923.1_cryptochro
MANKVQHSTIHWFRKGLRFHDNPSLLHALRTSRHVYPVYVVDQNWMKEHDIRYGANWWRFVIQCLEELDTRLR
KYGLRLFVVRGSAEDFFKEHFRKWKVTQLTHDVDTEPFYRIRDVAVRKIASDMGVEVVTHVAHTLYDIDRTVE
RNGGTPPLTYRKFLKVYNEMGKPPKEKETATTEHFANCTLPPEALQDKKFDMVTLEELGMRCDFPAKFVGGET
EALRRLEKSLEDKDWVLKFEKPKTSPNSLLPSTTVLSPYITVGCLSARLFYHQLDKIYSKAKNHSQPPVSLHG
QLIWREFFYTAAAHTPNFNQMEGNRVCLQIPWNKNDEHLTRWRNAQTGFPWIDAAMTQLRKEGWIHHLARHAV
ACFLTRGDLWISWEEGMKVFEELLLDADWSLNAGNWMWLSASAFFHQYFRVYSPIVFGKKTDPKGTFIRHYLP
VLKNFPKEYIYEPWKAPRNVQEKAGCIVGKDYPRPIVDHKEASQRNLDIMRDVRKDQKETASVTLGYGK
>Branchiostoma_flo_XP_035662699.1_cry-1-like
MKSTIHWFRKGLRLHDNPSLRDALQMLQPGDVWRCVYVLDPWFAGSSNVGVNRWRFLLQCLEDLDASLRKLNS
RLYVIRGQPTDVFPRLFKEWKVSCLSFEEDSEPFGRERDMAVMKLAKEAGVKVILRTSHTLYKLQDIIDVNGG
QPPLTYKRFQAILSKMDPPDQPVDSITASTVDNLTTPIRDDHDEKFGVPSLEELGFDTDNLNPVVWPGGETEA
LTRLERHLERKAWVASFERPKMTASSLLASPTGLSPYLRFGCLSPRLFYKKLTELYKKVKRSNHPPLSLYGQL
LWREFFYTAATNNPNFDKMVNNTICVQIPWDKNPEALAKWAEGQTGFPWIDAIMAQLQQEGWIHHLARHAVAC
FLTRGDLWISWEEGMKVFEELLLDADWSVNAGSWMWLSCSSFFQQFFHVYCPVNFGRRTDPNGDFIRKYLPKL
RGFPAKYIYDPWNSPESVQKAAKCIIGKDYPLPMVNHAETSRINVERMKQVYQQLSRYRGAGLLATIPYPDGG
NGNPTGHKKHAIVQGMERQHAERREHGQPAHAAEARDQSEGDMSNVD
>Branchiostoma_flo_XP_035692392.1_photolyase
MEAGSEDFLDQIAERRQNVCESVAGFKFNKKRCRQMSDVGDLKQNCGGIMYWMSRDQRVQDNWALLFAQRLAM
KHKVPLYVCFCLVPKFLDANIRHYGFMLKGLEEVEKELTDLDVSFHLLTGYAVDVLPGFVDEKNIGGVVTDFS
PLRTPLKWVHDLAGKLPSDVPFVQVDAHNIVPCWEASPKQEYGARTIRGKIHNQLSSYLTEFPPVSQHPHPPK
DKPEPTDWAAARASLKVDMTVAEVTWATPGTTGGLRMLESFCKQRLKNFGTDRNNPNKRALSNLSPWIHFGQI
SVQRCILTVKKYRSKYKESVDAYIEEAIIRRELSDNFCFYNHDKYDSIKGASAWAQKTLKDHAGDKREYLYSR
EQLEQAKTHDDLWNAAQIQLNKEGKMHGFLRMYWAKKILEWTESPQVALKEALYLNDKYSLDGRDPNGYVGCM
WSICGIHDQGWAERPVFGKIRFMNYNGCKRKFDVAQFVTKYKAKGYLKK
>Bremia_lac_TDH69075.1_CCR75_0051
MASVRRALEKLKLPAGLHCERIQWLCEGAALQTKDRYLLYWMQTSVRTKHNYALEYAVAAANELKMPLYVVYL
FPDRSVVPALNNQVKDDEWKAEAIQDANAHAFATERHAFFALEGLTDAHERLASRGVSLDVFHHQHHASACPT
RSELLEICARKATLVVTDRPYLRPWRSALDTLVSSVETTQDDGKTGSSYGIVQIEGDVVVPVEEVTDKEEYAA
RTIRSKITKHLDRYIVPLEVMTLDKTCQTNGQSIVSLLENPHEIQALDVTSTKAIEATLKLLDVDRSVKSVKW
HFKGGEKAASACVKKFLTPANLEKYATERNEPSLDAGSDLSHYLRYGHISVVRIVLAAHKLKGVARAQEGVKS

FLEELIVRRELSVNLVVFNQENYDSMRCLPSYAQKTLQEHAEDKRNVIYSRDTLESFQTDDRYWNAAQRELVI
SGKMHGYMRMYWGKKLLEWTRTPEEGFNAALTLNNKYAIDAPDANSYAGIAWTFGKHDQGWRERAIFGKVRYM
NEAGLKRKFKMDAYVLKVGRLAAKAKRGAASDNTDNVDGHVESGGKRKADSEQTGAVAKSKKL
>Bremia_lac_TDH74364.1_CCR75_0094
MKVLVWFRRDVRMHDNLALHAALQQIKNTSGKGDVELIPLYILHRPQLLRCGVHRFQFVLESVADLAKSFQDR
GSPLLIAKGDGLKVLECLLPAWGITHLFFDGACEPFGIERDNQVLDVAKTLNIQTHVTNGYNLYDLKKVITSH
SNQAPKTYRSFLKAIGNQSKPNEPISAPEKLPFSLRNPTELYQNVLNYWNDSNQSNQSQKNKNKKLIEDIGGP
KHDFTLPDLQHFQFTPPEKHSFIYGGEEIALEMLKKFCADETRVAKFEKPKTSPAATPPSPSTTVLSPYLHLG
CLSPRTFYHTVQAILQRHSNVTKPPTSLLGQLLWRDFYHCHGQANPYFDKMEESPTCLQINWRYHLIPEKKED
WSADDKLARSQFEAWRDGKTGFPWIDAIMIQLKEEGWMHHLARHAVACFLTRGDLYISWIRGLEVFQERLIDH
DWSVNAGNWLWLSSSYFFTAYFRVYSPISFGKKYDPEGLLIKKYIPALKKFPAKYIYEPWKAPESIQRSAGCR
IGTDYPAPIVEHPKAKKKCLEGLKMSYANHEYGTFPTAKDTRRSTKRQREDSENGE
>Bucco_cape__NXH11539.1
CIYILDPWFAASSAVGINRWRFLLQSLEDLDNSLRKLNSRLFVVRGQPTDVFPRLFKEWGVTRLTFEYDSEPF
GKERDAAIVKLAKEAGVEVVIENSHTLYDLDRIIELNGHKPPLTYKRFQAIISRMELPKKPVSTVTSQQMEVC
KVDIQENHDDVYGVPSLEELGFPTDGLAPAVWQGGETEALARLDKHLERKAWVANYERPRMNANSLLASPTGL
SPYLRFGCLSCRLFYYRLWELYKKVKRNSTPPLSLYGQLLWREFFYTAATNNPKFDRMEGNPICIQIPWDRNP
EALAKWAEGKTGFPWIDAIMTQLRQEGWIHHLARHAVACFLTRGDLWISWESGVRVFDELLLDADFSVNAGSW
MWLSCSAFFQQFFHCYCPVGFGRRTDPSGDFVKRYLPKLKGFPSRYIYEPWNAPESVQKAAKCIIGVDYPKPM
VNHAETSRLNIERMKQIYQQLSRYRGLCLLASVPSCVEDLSGPVTDSASGQGCSTSTAIRLAPAEQASPKRKH
EGVEELCTEELYKRAKVTGLPAPEIPGKS
>Bucco_cape__NXH15799.1
GEKSHQGLTQLFLLFSSCAPQVDYDVCSNYGNWLYSAGIGSDPRDNRKFSMIKQGLDYDGNGDYVRLWIPELQ
GIKGADIHTPWALSSATLSQAGVTLGETYPQPVVTAPEWSKHINQRPQGRSSHPRGRRGPAHTPVQHKDRGID
FYFSHKKDV
>Bucco_cape__NXH16479.1
LXWFRKGLRLHDNPALRECIQGADTVRCVYILDPWFAGSSNVGINRWRFLLQCLEDLDANLRKLNSRLFVIRG
QPADVFPRLFKEWNIAKLSIEYDSEPFGKERDAAIKKLASEAGVEVIVRISHTLYDLDKIIELNGGQPPLTYK
RFQTLISRMEPLEMPVETITPEVMEKCTTPVSDDHDEKYGVPSLEELGFDTDGLPSAVWPGGETEALTRLERH
LERKAWVANFERPRMNANSLLASPTGLSPYLRFGCLSCRLFYFKLTDLYKKVKKNSSPPLSLYGQLLWREFFY
TAATNNPRFDKMEGNPICVQIPWDKNPEALAKWAEGRTGFPWIDAIMTQLRQEGWIHHLARHAVACFLTRGDL
WISWEEGMKVFEELLLDADWSVNAGSWMWLSCSSFFQQFFHCYCPVGFGRRTDPNGDYIRRYLPVLRGFPAKY
IYDPWNAPESVQKAAKCMIGVNYPKPMVNHAEASRLNIERMKQIYQQLSRYRGLGLLATVPSNPNGNGNGGLM
SYSPGESISGCGNTGGGAQLGTGDGHTVVQPCALGDSHTGTSGIQQQGYCQASSILHYAHGDNQQSHLLQAGR
TSLGPGISAGKRPNPEEETQTVGPKVQRQSTN
>Bucco_cape__NXH19275.1
MPHHTLHLFRKGLRLHDNPTLLAALQSSEVLYPVYILDRKFMTSVIHIGALRWHFLLQSLEDLQKNLCQLGSC
LLVLQGEYESVLRKHIQEWNITQVTLDEEMEPFYKEMEANIRHLGEELGFEVLSRGSHTLYDTKRILELSGGT
PPLTYKRFLHILSLLGDPEQPVRSLTAEDFQRCQPPQQGLAEQYRVPLPVDLRIAPESLSPWRGGETEGLQRL
EQHLTDQGWVASFTKPKTTPNSLLPSTTGLSPYFSMGCLSARSFFYRLSSIYAQAKHHSLPPVSLQGQLLWRE
FFYTVASATPNFTKMAGNPICLQISWYEDAEKLHRWKMAQTGFPWIDAIMTQLRQEGWIHHLARHAVACFLTR
GDLWISWEEGMKVFEELLLDADYSINAGNWMWLSASAFFHQYRRIFCPVRFGKRTDPEGHYIRKYLPVLKNFA
SKYIYEPWTASVEEQKQAGCIIGQDYPFPMVNHKEASEHNLQLMKQVREEQYRTAQLTRDDTDDPMEGKVKRE
HSEENTSKGKVARTTE
>Bucco_cape__NXH23233.1
DNWAFLYAQRLALKQELPLHVCFCLVPKFLDATIRHYDFMLKGLQEVAKECAKLNIAFHLLLGYAKDVLPTFV
VEHGVGGLVTDFCPLRLPRQWVEDVREQLPEDVPFAQVDAHNIVPCWVTSHKQEYSAWTIRGKIHALLPEFLT
EFPPVICHPYPPSCPAETIAWEACYSSLQVDCTVKEVEWATPGTAAGLKVLQSFITERLKSFCSHRNNPNKAA
LSNLSPWFHFGQVSTQRAILEVQKHRGKYKDSVDAFVEEAVVRRELAENFCYYNDNYDSVQGAYDWAQTTLTL
HAEDKRPFLYELPELEQGITHDPLWNAAQRQMVWEGKMHGFLRMYWAKKILEWTCSPTEALRIAIYLNDRYEL
DGRDPNGYVGCLWSICGVHDHGWAERAVFGKIRYMNYAGCKRKFDVGQFERRY
>Buceros_rhin_silvestris_KFO83926.1
GSEVVLFYLAYFFSRIIELNGHKPPLTYKRFQAIISRMELPKKPVSTVISQQMETCKVDIQENHDDVYGVPSL
EELGFPTDGLAPAVWQGGETEALARLDKHLERKAWVANYERPRMNANSLLASPTGLSPYLRFGCLSCRLFYYR
LWELYKKVKRNSTPPLSLYGQLLWREFFYTAATNNPKFDRMEGNPICIQIPWDRNPEALAKWAEGKTGFPWID
AIMTQLRQEGWIHHLARHAVACFLTRGDLWISWESGVRVFDELLLDADFSINAGSWMWLSCSAFFQQFFHCYC
PVGFGRRTDPSGDYVKRYLPKLKGFPSRYIYEPWNAPESVQKAAKCIIGVDYPKPMVNHAETSRLNIERMKQI
YQQLSRYRGLCLLASVPSCVEDLSGPVADSASGQGCSTSTAVRLSQADQASPKRKHEGAEELCAEELYKRAKV
TGLPAPEISGKS
>Buceros_rhin_silvestris_KFO90010.1

RFLLQCLEDLDANLRKLNSRLFVIRGQPADVFPRLFKEWNIAKLSIEYDSEPFGKERDAAIKKLASEAGVEVI
VRISHTLYDLDKIIELNGGQPPLTYKRFQTLISRMEPLEMPVETITPEVMEKCTTPVSDDHDEKYGVPSLEEL
GFDTDGLPSAVWPGGETEALTRLERHLERKAWVANFERPRMNANSLLASPTGLSPYLRFGCLSCRLFYFKLTD
LYKKVKKNSSPPLSLYGQLLWREFFYTAATNNPRFDKMEGNPICVQIPWDKNPEALAKWAEGRTGFPWIDAIM
TQLRQEGWIHHLARHAVACFLTRGDLWISWEEGMKVFEELLLDADWSVNAGSWMWLSCSSFFQQFFHCYCPVG
FGRRTDPNGDYIRRYLPVLRGFPAKYIYDPWNAPESVQKAAKCIIGVNYPKPMVNHAEASRLNIERMKQIYQQ
LSRYRGLGLLASVPSNPNGNGNGGLMGYSPGESISGCGSTGGAQLGTSDGHTVVQPCALGDSHTGASGIQQQG
YCQASSILHYAHGDNQQSHLLQAGRTALGTGISAGKRPNPEEETQSVGPKVQRQSTN
>Buceros_rhin_silvestris_KFO93683.1
DNWAFLYAQRLALKQELPLHVCFCLVPKFLDATIRHYSFMLRGLQEVAKECAELNIPFHLLLGYAKDVLPTFV
VEHGVGGLVTDFCPLRLPRQWVEDVRDQLPEDVPFAQVDAHNIVPCWVASPKQEYSAWTFRCKINPQLPEFLT
EFPPVVRHPYPPSCPAEPIAWEACYSSLQVDRTVKEVEWATPGTAAGLAVLQSFIADRLKSFGSHRNNPNKAA
LSNLSPWFHFGQVSTQRAILEVQKHRSKYKESVEVFVEEAVVRRELAENFCYYNENYDSVQGAYSWAQTTLKL
HAKDKRPFLYNLQELEQGTTHDPLWNAAQLQMVREGKMHGFLRMYWAKKILEWTHSPEEALQFAIYLNDRYEL
DGRDPNGYVGKRCLWSICGIHDHGWTERAVFGKIRYMNYAGCKRKFDVGQFERRYAP
>Bucorvus_abys__NWR58043.1
MGVNAVHWFRKGLRLHDNPALRECIQGADTVRCVYILDPWFAGSSNVGINRWRFLLQCLEDLDANLRKLNSRL
FVIRGQPADVFPRLFKEWNIAKLSIEYDSEPFGKERDAAIKKLASEAGVEVIVRISHTLYDLDKIIELNGGQP
PLTYKRFQTLISRMEPLEMPVETITPEVMEKCTTPVSDDHDEKYGVPSLEELGFDTDGLPSAVWPGGETEALT
RLERHLERKAWVANFERPRMNANSLLASPTGLSPYLRFGCLSCRLFYFKLTDLYKKVKKNSSPPLSLYGQLLW
REFFYTAATNNPRFDKMEGNPICVQIPWDKNPEALAKWAEGRTGFPWIDAIMTQLRQEGWIHHLARHAVACFL
TRGDLWISWEEGMKVFEELLLDADWSVNAGSWMWLSCSSFFQQFFHCYCPVGFGRRTDPNGDYIRRYLPVLRG
FPAKYIYDPWNAPESVQKAAKCIIGVNYPKPMVNHAEASRLNIERMKQIYQQLSRYRGLGLLATVPSNPNGNG
NGGLMGYSPGESISGCGSTGGHLFPASPGAQLGTGDGHTVVQPCALGDSHTGASGIQQQGYCQASSILHYAHG
DNQQSHLLQAGRTALGTGISAGKRPNPEEETQSVGPKVQRQSTN
>Bucorvus_abys__NWR62381.1
DNWAFLYAQRLALKQELPLHVCFCLVPKFLDATIRHYGFMLRGLREVAEECAELNIRFHLLLGYAKDVLPTFV
VEHGVGGLVTDFCPLRLPRQWVEDIRERLPEDVPFAQVDAHNIVPCWVASPKQEYSAWTFRGKIHAQLPEFLT
EFPPVVRHPYTPSCPAEPIAWEACYSSLQVDHTVKEVEWATPGTAAGLAVLQSFIAERLKSFSSHRNNPNKAA
LSNLSPWFHFGQVSTQRAILEVQKHRGKYKESVEVFVEEAVVRRELAENFCYYNENYDSVQGAYHWAQTTLKL
HAKDKRPFLYKLQELEQGTTHDPLWNAAQLQMVREGKMHGFLRMYWAKKILEWTHSPEEALQFAIYLNDRYEL
DGRDPNGYLMLCCLWSICGVHDHGWTERAIFGKIRYMNYAGCKRKFDVGQFEHRYAPRM
>Bufo_bufo__XP_040265064.1
MEAASTAAVSSLHWFRKGLRIHDNPALVAALRGARCVRCVYILDPWFAASSSGGVNRWRFLLQSLEDLDSSLR
KLGSRLYVVRGQPADVFPRLFKEWGVNRLTFEYDSEPFSKERDAAIMKLAKEAGVEVIVENSHTLYDLDKIIE
LNGNSPPLTYKRFQAIISRMELPRRPVLTITRQQMAPCRGEIKNTHDETYGVPSLEELGFPRENQGAAVWPGG
ETEALARLDRHLERKAWVANYERPRMNANSLLASPTGLSPYLRFGCLSCRLFYYRLQELYQKVKKNSPPPLSL
YGQLLWREFFYTAATNNPKFDQMEGNPICVQIPWDRNPEALAKWTEGKTGFPWIDAIMTQLRLEGWIHHLARH
AVACFLTRGDLWNSWECGVKVFDELLLDADFSVNAGSWMWLSCSAFFQQFFHCYCPVGFGRRTDPSGDYVRRY
LPVLKGFPSRYIYEPWNAPESVQKEAKCVIGVDYPKPIVNHAEASRLNIERMKQTYQQLSRYRGLCILASVPS
CAEDLSGPISDPVAHQISIEAAPKLSLCAPDSPKRRYKKSVEGACKKARMQSVAEVEMTAKDF
>Bufo_bufo__XP_040284355.1
MSQKARAPKRKSEGSGNEKSDTSQKDDSKTKSITATKSQGKRNPQEHKNKSKVISDASSSETSEVDVKKKKLQ
EKKVDVDGGLLEAVKKSRLSVATSVSDFKFSKKRVRLVSAEENLKDDALGIVYWMSRDQRVEDNWAFLYAQRL
ALKQKLPLHVVFCLVPKFLDATIRHYGFMLKGLQEVAEDCKGLNIPFHLLIGYAKDVLPDFVKEHGIGGVVTD
FSPLRVPLQWVSDICERLPEDVPLVQVDAHNIVPCWVASNKQEYGARTIRRKIHDQLSQFLTDFPPVITHPYK
SNLKAEPVDWDKCYASLEVDRTVKEVEWAKPGSKAGMNTLHSFISERLKFFNSDRNNPNRRALSSLSPWFHFG
QLSVQRAILEVQKYRSKYKESVDSFVEEAVVRRELADNYCYYNKNYDKVEGAYDWAKNTLKDHAKDKRTHLYT
LEQLESGKTHDPLWNAAQMVHEGKMHGFLRMYWAKKILEWTSSPEEGLRFAIYLNDRFQLDGRDPNGYVGCMW
SICGIHDQGWAERAVFGKIRYMNYQGCKRKFDVAEFERRYHPKKFTA
>Bufo_bufo__XP_040287471.1
MPQRSIHWFRKGLRLHDNPALLAAIKGCEELHPIFILDPWFPRNMKVSGNRWRFLIEALRDLDENLKKINSRL
FVVRGKPTEVFPQLFKKWKVTRLTFEVDTEPYSRQRDAEVEKMASEHNVQVIQKVSNTLYDVDRVIAENNGKP
PLTYVRLQTVLASIGPPKRPCPAPTKEDMKDCCTPWKPSYEEIYGVPTLEELGQDPSKLGPHLYPGGESEALR
RLDLNMKRTAWVCNFKKPETEPNSLTPSTTVLSPYMKFGCLSTRIFYWRLTEIYQGKKHSSPPVSLHGQLLWR
EFFYTAGAGIPNFNKMEGNSVCVQVDWDENKEYLKAWSEARTGYPFIDAIMTQLRTEGWIHHLARHAVACFLT
RGDLWISWEEGQQVFEDLLLDADWSLNAGNWQWLSASAFFHQFFRVYSPVAFGKKTDKNGDYIKKYLPILKKF
PPEYVYEPWKSPRSVQERAGCIIGKDYPKPIVEHEIVSKRNIQRMKAAYARRSGTGGEKVPKSQGVKRKVPSV
SQFFKKK
>Bufo_bufo__XP_040288557.1

MKMAALSRTIICLLRNDLRYHDNEVLLWAHRNADHIVPLYCFDPRHYLGTHYYNFPKTGPHRLKFLLETVQDL
RNTLQKKGSNLLLRRGQPEDVIEDLIKQLGGVSAVVLQEEATKEELDVEKSVKQVCTQHGVKFHTIWGSTLYH
RDDLPFRHISSLPDVYTQFRKVVEAQCKVRPTLQMPEKLKSLPAGLEEGSVPTAEDFEQQDPLTDPRTAFPCN
GGESQSLQRLQYYFWDTNLVASYKDTRNGLVGMDYSTKFAPWLALGCISPRYIFEQIRKYERDRTANQSTYWV
IFELLWRDYFRFVALKYGRKIFFLKGLQDKKVPWKKDLKQFDAWKEGRTGVPFVDANMRELAMTGFMSNRGRQ
NVASFLTKDLGLDWRMGAEWFEYLLVDYDVCSNYGNWLYSAGIGNDPRENRKFNMIKQGLDYDGKGDYIRLWV
PELQEIKGGDVHTPWALSNAALAHANVTLGETYPFPIVMAPEWSRHVNQKPDSGATASGRRPGPSHMPKQHKN
RGIDFYFSRNKNV
>Bufo_bufo__XP_040295401.1
MGVNAVHWFRKGLRLHDNPALKECIRGADTIRCVYILDPWFAGSSNVGINRWRFLLQCLEDLDANLRKLNSRL
FVIRGQPADVFPRLFKEWKISKLSIEYDSEPFGKERDAAIKKLASEAGVEVIVRISHTLYDLDKIIELNGGQP
PLTYKRFQTLISKMEPLEIPVETITAEVMDKCSTPVSDDHDEKYGVPSLEELGFDTEGLPSAVWPGGETEALT
RLERHLERKAWVANFERPRMNANSLLASTTGLSPYLRFGCLSCRLFYFKLTDLYKKVKKNSSPPLSLYGQLLW
REFFYTAATNNPRFDKMDGNPICVQIPWDRNPEALAKWAEGRTGFPWIDAIMTQLRQEGWIHHLARHAVACFL
TRGDLWISWEEGMKVFEELLLDADWSVNAGSWMWLSCSSFFQQFFHCYCPVGFGRRTDPNGDYIRRYLPILKG
FPPKYIYDPWNAPEAVQKVAKCIIGVNYPKPMVNHAEASRLNIERMKQIYQQLSRYRGLGLLASVPSNPNGNG
NGGLMSYSPGDTMPCCSNNGGPQMGASEGNSSSNSNNNQGESHSGTGGMQGYWQGSSILHYNYGDSQQSYLIQ
DVGNRRVT
>Bulinus_trun__KAH9490127.1
MCYKSVCFVFSKIWTLLLKKIFEVIKALKRNINVNTLNLRNLLIIKRSIKMSKHKVIHWFRKGLRLHDNPALR
EALKQSSEVYPVFVLDPWFVNSSKIGINRWRFLLQSLENLDNNLKSLNLRLYVVRGKPETELPRLCKEWEITR
LTFEVDTEPYAVQRDKKVEEIVQPMGVEIVKCVSHTLYDVHEIIKANGDKAPLTYQGFQSMLSKLGPPAQALP
APSKQDVSDIKTLISSTHDVKYGVPTLSDLGKSESSCGPLLYPGGETEALDRMERHIKKINWICTFEKPNTSP
NSIIPSTTVLSPYLKFGCLSARLLYHKLAEVYKGKKHSQPPVSLHGQMLWREFFYTVAAVTPNFDHMKDNPVC
KQIPWTSNQEHLSAWTHAKTGYPFIDAIMTQLRQEGWIHHLARHAVACFLTRGDLWISWEEGQKVFEELLLDA
DWSLNAGNWMWLSASAFFHQYFRVYSPIAFGKKTDPNGDYIKKYIPVLKKFPAKYIYEPWTAPSSVQQAAGCI
IGKDYPKPIVEHDKVRNENIKKMADAYAQDKLNKEGSQSSPKKRPHSSDSKNTSAKKSRTK
>Bulinus_trun__KAH9490293.1
MKRSFSGKESKKTTKKRKQDEGQTSEKSTASITCAEEKDTVPEVVDFLEKITKRRERLHENVAKFNFNKKRVR
VLSKTEEFPEKSHGVLYWMSRDQRTQDNWAFLYAQRLAMKLEVPLYVCFCLIPKFLEATIRQFGFMMKGLALV
EKECKDLGIPFHLLLGEASQCIPPFLQEKNIGGVVTDFSPLRVPMKWVDDLKKALPTDIPFCQVDAHNIVPCW
VASPKLEYGARTIRSKIHNQLGQFLTEFPPLVNHPHSPEKMPAATDWKKAESYLEVDKKVTEVEWAKPGTEAG
LRMLESFCEKRLKYFSTDRNDPNKEALSNLSPWFHFGQISVQRCILTVRLYRSKSSDSVNAFIEEAVVRRELA
DNFCFYNPDYDSVKGTNDWAKKSLDLHRKDKRPYMYTREQLERSQTHDDLWNAAQTQLVIEGKMHGFLRMYWA
KKILEWTESPEKALADAIYLNDKYNLDGRDPNGYVGCMWSICGIHDQGWAERAVFGKVRYMNYEGCKRKFDVP
AFVRNGLQLSTVVYNDLQLSKVVYNDLQLSTVVYNDLQLSTVVYNDLQ
>Bulinus_trun__KAH9507287.1
MQIASENPSIAICLFRNDLRVHDNEALYTANQKGKFLLPLYCFDPRHYAGTYHFGFPKTGCYRLQFLLESVTD
LRLKLQSLGSNLIVRLGKPEDVIAELVKSLNIPNNVAVVIQKEVTEEEVKVEQDIRSKCRVPLLTVWGHTLYH
IEDLPFEPKHLPDVYTQFRKKVEDNIPVRKLINLPVQLNPLPMGIDPGLIPTFANFGFTAPDRDPRTAFPFFG
GETSALERLHHYMWVTDNICTYKETRNGMIGPDFSTKFSPWLAHGCISPRKIYWEIKKYERERTSNQSTYWVI
FELLWRDYFRYVALKYGNKIFFSGGIKGESVPWKTNHSHFKAWQEGRTGVPYVDANMRELAATGFMSNRGRQN
VASFLTKDLQLDWRLGAEWFESLLIDHDVCSNYGNWLYSAGIGNDPRENRKFNVVKQGLDYDGDGNYVRLWVR
ELAGIKTGKIHCIWTLSNSQLQSGGVILGQTYPSPIVIAPEWSRHVNRPSSSSGASSGKGKGKSSSSHKSQVQ
QGPYPGQKRGLDFYFSNTNPR
>Bulinus_trun__KAH9508662.1
MKENHREYSMAQSQTSLPTKKHSLFWFRKGLRLHDNPALLAAIDGASTYRCIYILDPWFAGASQVVINKWRFL
LESLEDLDSSLRKLNSRLFVVRGQPADVLPRLFQEWGITHLAFEEDPEPYGKERDAAISALAREQSIQVITKS
SHTVYDPKIVIAANGNSPPLTYKRFQSILSKLDPPLQPCESLSSNMVAEVSTPVSDDHDDRFNVPSLEELGFD
VDSLGPAQFRGGESEALARLYRHLERKAWVASFERPKMSPQSLYPSGTGLSPYLRFGCLSPRLFYWKLTELYK
KVKKGAEPPLALHGQLLWREFFYTVATNNPNFDRMAGNSICVQIPWDHNPEALAKWAEGMTGYPWIDAIMAQL
RKEGWIHHLARHAVACFLTRGDLWISWEEGMKVFDEMLLDADWSVNAGMWMWLSCSAFFQQFFHCYCPVGFGK
RADPSGDFVRHYLPVLKGFPTQYIYEPWTAPESVQKAAKCIIGKDYPLPMVQHAEACRVNLERMRQVYKRLVL
KSTASKRMFLDLPKIPKVSLFLSKAKMFLSSSSDEDVSQKQRRRKIDE
>Bulinus_trun__KAH9520043.1
MSHNEKKGHVTVHWFRHGLRLHDNPALLDGLEDCREFYPIFIFDGDVAGTSTAAFPRMQFLFESLQDLDDNLK
SHGTRLYVFKGDPVDVFKRLIEEWGVTRITFEQDPEPVWQARDSRVKKLCADQDIECIERVSHTLWDPQLIIK
ENGGTPPLTYAMFCQVADIVGLPPKPKGNPDFSGISLPVSEHHDEKYQLPTLAEMGVEAESEQQASPYCRYMG
GETKAFKLLTIRLEKERKAFTMNQSLPNQLYPELSGMPLSLSPHLRFGSMSIRKFYWSLRQVYSEVHPNTQIP
AAITGQLIWREYFYCMSVNNPNYNRMIGNPICLKIDWYDNDSHLKAWKMGKTGYPWIDACMRQLLQEGWIHQV

CRHATACFLTRGDLWIDWVKGLEVFDRYLLDADWSVCAGNWMWVSSSAFEKVLQCPKCICPIRYGRRIDPKGE
YVRRYVPELKNMPLRYLFEPWKAPQKVQEEAQCIIGEDYPMPIVNHGIASKECQTRMEIIKSKSKDIPHIRPA
NEDEVYSSMWVSKELEKMDLCRHNNDRILFDM
>Cafeteria_roe_KAA0161729.1_FNF31_0367
MSAAAAAAAVPGRRAKAAIHWFRKGLRLHDNPALLRACEAAAVYPVFVIDPWFVSEERVGVNRMRFLLETLED
LDAALRARSSKLSVLRGRPEEVLPRAAAHWGADLLTFEADFEPYALQRDEAVRSLLAERGVRVESFVSHSLYD
PRGVAAAGPGGKPPLSYGPFVKLLRGIGPPPRPARDPPAAMPSEGWKPAPAALTFTDNATTASGTGDGASSLP
ASGEPASAAHGDRLAEARIPSLADLGYAPPEHAPLFPGGEQEGLRRMAAKLSRGDWVRAFAKPKTSPTAVGEP
DTTGLSPYLKFGCVSSRRFWHELAAVNAGGPHGSPPTSLEGQLLWREFYICHGFSFPNYDRMAGNPVCRQIAW
QDDPELLRRWEEGTTGFPWIDACMRQLRAQGWLHHLGRHAVACFLTRGDLYQSWERGARVFDRLLVDSDWALN
NGNWMWLSASAYFFQFFRVYSPVAFPKHTDPDGDYVRRWVPELANFPKAYIYEPWKASKQNQRKWGCEIGSDY
PEPIVDHVAVSKLNMARHKAAYEAHKAPGPPRDVPVGDSPGAFQVGSAPRAPKRARGDGDEGDAAAPGGSGEE
RDGASAAKRAKPASP
>Cafeteria_roe_KAA0175826.1_FNF27_0254
MMARRAGRVAAPVSSGLAGAAAASEGRRAVATGATSASGGTAVVWFKATDLRLHDHGPLAKAHAAHSHVVHVM
CIDPRWFGASPTGFAKTGFHRARFLSESVADLARSLAARGSSLVVRASRPEVALPAVAAACGASAVYAHSEVC
SEELAADRAVRAALHGLPSPVRLSQCWGNTLHDVADLPFDASSDTPQVFSQFRRLVEAKCKPRPPLPPPPALR
PAPPGFAAGTSPHGLAMLPLDGGGPGSQPPHSHAAVRPLPASAVDAVLAGRVPMAWELGCALPPGMDTSALRP
VAPPAGVGLPPGVALASGPGAASGAAGGAVPPAAPAAPAAPAPALSARQVECGGLRDPRCAIQLFGGETAALA
RLRLYLWDEDRLREYKQTRNGMAGQGFSSKLSPWLAVGALSPRQVAAEVARYERERVANESTYWLLFELLWRD
YFRFACIGWGSDVFRLAGPKRALDPGRGWSRDPAVWGPWTRAQTGYPLVDACMRELTQSGFMSNRGRQNVASF
LARDLEADWRVGAEWFESLLADHDPCSNYGNWTYVAGVGADPREDRYFSIPKQARNYDGHGTFMRLWLPEGLD
VVRRRAKLAFQENAHLTDEVAIKRAVAKGRYWVREIHATHALHKYRSIKKRYY
>Cafeteria_roe_KAA0176536.1_FNF27_0181
MATAGRAPKRDRSEGEAAAAAPPPKKGRAPAAAVSPRRQRALNSVPVRPGPILYWMSRDQRAEDNWALLAAQD
LAQARGEPLVVAFSLVRGFLGATQRQFDWMLRGLEETEADLRAKNIPFVLVRGPCERTIQSLAERGKFGAIVC
DFAPVRIGRRWRDTVASNAGVAVIEVDARNIVPAWLASDKLEYAARTIRPRIHRQLGEFLTEFPSLAKQEDFF
PGGWDALQRLASEASADAASAEGTTVQGGVGASAAAASAGAGAEALPCGAGAGIPWPEVRRSLDVDATVPPVA
WLAPGPAAAMAQVGRFLARIRAYGKDRNDPSKGATSLLSPYLHFGHLSSQRAALLARAQRSKAPESVDGFIEE
MVVRRELAENYVFYNQDGYDRIDGMYPQFGNDSWAQRSLREHASDKRSVLYTREELESGKTHDPLWNAAQAEM
VHLGHMHGFNRMYWAKKILEWTPSPEEALATCIALNDKYQLDGRDPNGWVGCAWAVAGVHDNGWKERPVFGKI
RYMNLTSTIRKFSIDSYVARVAQRVEAETGLRSDVTKGMAAKQAAPGKKGQLWGLAGLRRVKAE
>Calidris_pugn__XP_014800382.1
MSGAARTAICLLRCDLRAHDNQVLHWAQGNADFVVPLYCFDPRHYLGTHCYGFPKTGPHRLRFLLESVKDLRE
TLKKKGSTLVVRKGKPEDVVRDLITQLGSVSAVAFHEEATQEELDVEKGLCQVCSQHGVKIQTFWASTLYHRD
DLPFRSISRLPDVYTHFRKAVESEAKVRPTLRMADQLKPLPPGVEEGCIPTMEDLGQKDPVTDPRTAFPCSGG
ETQALMRLQYYFWDTNLVASYKETRNGLVGMDYSTKFAPWLALGCISPRYIYEQIQKYEKERTANQSTYWVLF
ELLWRDYFRFVALKYGKRIFSLRGLQSKEVPWKKDLQLFDCWKEGKTGVPFVDANMRELAATGFVSNRGRQNV
ASFLTKDLGLDWRMGAEWFEYLLVDYDVCSNYGNWLYSAGLGNDPRDNRKFNMIKQGLDYDGNGDYVRLWVPE
LQGIKGAEIHTPWALSAAALSQAGVTLGDTYPQPVVTAPEWSRHINQRPQGQRPHPRGKRGPAHTPRQHKDRG
IDFYFSRKKDVQ
>Calidris_pugn__XP_014801325.1
MRRGQGKRKAEATEVPGVSRRRKEEEEAMQEARRRAAPSVREFKYNKKRVRLVSQGSDLKDDARCILYWMSRD
QRVQDNWAFLYAQRLALKQELPLHVCFCLVPKFLDATIRHYGFMLKGLREVAEECAELNIPFHLLLGYAKDVL
PAFVVERGVGGLVTDFCPLRLPRQWVEDVKERLPEDVPFAQVDAHNIVPCWVASPKQEYSARTIRGKIHAQLP
EFLTEFPPVIRHPYSPSSPTEPIAWEACYSSLQVDRTVKEVEWATPGTAAGLAVLQSFIAERLKSFGSHRNDP
NKAALSNLSPWFHFGQVSTQRAILEVQKHRAKYKESVDAFVEEAVVRRELAENFCYYNENYDSVQGAYDWAQT
TLKLHAKDKRPFLYKLQELEQGTTHDPLWNAAQLQMVREGKMHGFLRMYWAKKILEWTRSPEEALQFAIYLND
RYELDGRDPNGYVGCLWSICGIHDQGWAERAIFGKIRYMNYAGCKRKFDVAQFERRYAPRTLSQ
>Calidris_pugn__XP_014801760.1
MSHRTIHLFRKGLRLHDNPTLVAALESSEVLYPLYILDRKFMTSVMHIGALRWHFLLQSLEDLQKNLSQLGSC
LLVAQGEYEAVLRDHVQKWNITQVTLDAEMEPFYKEMEAKIRRLGEELGFEVLSLVGHSLYDTKRILDLNGGT
PPLTYKRFLHILSLLGDPEVPVRNLTAEDFQRCQPPDPGLAEGYGVPLPVDLKLPPESPSPWRGGETEGLRRL
EQHLTDQGWVASFTKPRTVPNSLLPSTTGLSPYFSMGCLSVRTFFYRLSNIYAQAKHHSLPPVSLQGQLLWRE
FFYTVASATPNFTKMAGNPICLQIPWYEDAERLHKWKTAQTGFPWIDAIMTQLRQEGWIHHLARHAVACFLTR
GDLWISWEEGMKVFEELLLDADYSINAGNWMWLSASAFFHQYTRIFCPVRFGKRTDPEGHYIRKYLPVLKNFP
SKYIYEPWTASEEEQQRAGCLIGRDYPFPMVNHKEASAHNLQLMKQVREEQPRTAQLTRDETDDPMEMKVKRD
HSEENIPKGKVARMADDTEVPLEPKKGKRGEAGGN
>Calidris_pugn__XP_014805817.1

MAAVAPLAPLAPGPAPSLCRSVHWFRRGLRLHDNPALQEALRDAASLRCIYILDPWFAASSAVGINRWRFLLQ
SLEDLDNSLRKLNSRLFVVRGQPTDVFPRLFKEWGVTRLTFEYDSEPFGKERDAAIVKLAKEAGVEVVIENSH
TLYDLDRIIELNGHKPPLTYKRFQAIISRMELPKKPVSTIMSQQMETCKVDIQENHDDVYGVPSLEELGFPTD
GLAPAVWQGGETEALARLDKHLERKAWVANYERPRMNANSLLASPTGLSPYLRFGCLSCRLFYYRLWELYKKV
KRNSTPPLSLYGQLLWREFFYTAATNNPKFDRMEGNPICIQIPWDRNPEALAKWAEGKTGFPWIDAIMTQLRQ
EGWIHHLARHAVACFLTRGDLWISWESGVRVFDELLLDADFSVNAGSWMWLSCSAFFQQFFHCYCPVGFGRRT
DPSGDYVKRYLPKLKGFPSRYIYEPWNAPESVQKAAKCIIGVDYPKPMVNHAETSRLNIERMKQIYQQLSRYR
GLCLLASVPSCVEDLSGPVPDSASGQGCSTSTAMRLSQADQASPKRKHEGAEEPCAEELYKRAKVTGLPAPEI
PGKSL
>Calidris_pugn__XP_014816511.1
MQHSSIHWFRKGLRLHDNPALLAAVTDCRRLHPLFILDPSGSRAGANAWRFLLDALQDLDRSLQEMGSRLFVV
RGCPEEVFPRLFHAWGTTRLTFEVDTEPLAHRRDAAVAELAAQHGVEVIQKVSHTLYDTERILALNDGKAPMT
YKRLQSLLASLGPPEKPAPALTREHLQGCRVPCQVSHNADYGVPTLEELGQDPTKVGPHLYPGGETAALTRLD
THMERTAWVCSFKKPETEPNSLSPSTTVLSPYLRFGCLSVRTFWWRLDEIYQGREHSQPPVSLHGQLLWREFF
YTAGATIPNFDQMLGNPVCLQVDWDDNPQHLQAWREGRTGYPFIDAIMTQLRSEGWIHHLARHAVACFLTRGD
LWVSWEEGLKVFEELLLDADWSLNAGNWLWLSGSAFFHRYFHVYSPVTFGKKTDQDGAYIRKYLPILKDFPTE
YIYEPWKAPRAVQERAGCLVGTNYPQPIVEHQAVRERNMGRMKAARARKGNGSPKKHQAPPTVLLSPRLPSSP
PANKREPPASPSVAESQRKKPKAEQR
>Calidris_pugn__XP_014819465.1
MEPLEMPVETITPEVMEKCTTPVSDDHDEKYGVPSLEELGFDTDGLPSAVWPGGETEALTRLERHLERKAWVA
NFERPRMNANSLLASPTGLSPYLRFGCLSCRLFYFKLTDLYKKVKKNSSPPLSLYGQLLWREFFYTAATNNPR
FDKMEGNPICVQIPWDKNPEALAKWAEGRTGFPWIDAIMTQLRQEGWIHHLARHAVACFLTRGDLWISWEEGM
KVFEELLLDADWSVNAGSWMWLSCSSFFQQFFHCYCPVGFGRRTDPNGDYIRRYLPVLRGFPAKYIYDPWNAP
ESVQKAAKCIIGVNYPKPMVNHAEASRLNIERMKQIYQQLSRYRGLGLLATVPSNPNGNGNGGLMGYSPGESI
SGCGSTGGAPLGTGDGHAVVQPCALGDSHTGASGIQQQGYCQASSILHYAHGDNQQSHLLQAGRTALGTGISA
GKRPNPEEETQSVGPKVQRQSTN
>Caligus_rog_QQP54369.1_cry1
MKNAEPPLALFNQLLWREFFYAAAFQNPNFDRMKGNPICLQIPWDKDSVALRKWSLGKTGFPWIDAIMTQLRE
EGWVHFICRHAVVCFLTRGDLWISWEEGMKVRNSYSRKDWPSGYPLELKILFIFNIQAF
>Callithrix_jacc__XP_002752992.2
MGVNAVHWFRKGLRLHDNPALKECIQGADTIRCVYILDPWFAGSSNVGINRWRFLLQCLEDLDANLRKLNSRL
FVIRGQPADVFPRLFKEWNITKLSIEYDSEPFGKERDAAIKKLATEAGVEVIVRISHTLYDLDKIIELNGGQP
PLTYKRFQTLISKMEPLEIPVETITSEVIEKCTTPLSDDHDEKYGVPSLEELGFDTDGLSSAVWPGGETEALT
RLERHLERKAWVANFERPRMNANSLLASPTGLSPYLRFGCLSCRLFYFKLTDLYKKVKKNSSPPLSLYGQLLW
REFFYTAATNNPRFDKMEGNPICVQIPWDKNPEALAKWAEGRTGFPWIDAIMTQLRQEGWIHHLARHAVACFL
TRGDLWISWEEGMKVFEELLLDADWSINAGSWMWLSCSSFFQQFFHCYCPVGFGRRTDPNGDYIRRYLPVLRG
FPAKYIYDPWNAPEGIQKVAKCLIGVNYPKPMVNHAEASRLNIERMKQIYQQLSRYRGLGLLASVPSNPNGNG
GFMGYSAESIPGCTSSGSCSQGSGILHCAHGDSQQTHLLKQGRSSMSTGISGGKRPSQEEDTQSIGPKVQRQS
TN
>Callithrix_jacc__XP_002755255.1
MAATVATAAAAVPAPAPGTDGASSVHWFRKGLRLHDNPALLAAVRGARCVRCVYILDPWFAASSSVGINRWRF
LLQSLEDLDTSLRKLNSRLFVVRGQPADVFPRLFKEWGVTRLTFEYDSEPFGKERDAAIMKMAKEAGVEVVTE
NSHTLYDLDRIIELNGQKPPLTYKRFQAIISRMELPKKPVGSVTSQQMESCRAEIQENHDETYGVPSLEELGF
PTEGLGPAVWQGGETEALARLDKHLERKAWVANYERPRMNANSLLASPTGLSPYLRFGCLSCRLFYYRLWDLY
KKVKRNSTPPLSLFGQLLWREFFYTAATNNPRFDRMEGNPICIQIPWDRNPEALAKWAEGKTGFPWIDAIMTQ
LRQEGWIHHLARHAVACFLTRGDLWVSWESGVRVFDELLLDADFSVNAGSWMWLSCSAFFQQFFHCYCPVGFG
RRTDPSGDYIRRYLPKLKGFPSRYIYEPWNAPESIQKAAKCIIGVDYPRPIVNHAETSRLNIERMKQIYQQLS
RYRGLCLLASVPSCVEDLSHPVAEPSSSQAGSMSSAGPRPLPSGPASPKRKLEAAEEPPGEELSKRARVAELP
TPELPSKDV
>Callorhinchus_milii_XP_007885950.1_cryptochro
MVVNSVHWFRKGLRLHDNPALQAAVSGADTVRSVYILDPWFAGSSNVRINKWRFLLQCLEDLDISLRKLKSRL
FVVRGQPTDVFPRLFKEWNVTRLTFEYDSEPFGKERDVAIVNLARKAGVETIVHTSHTLYDVNRIIEANGNKP
PLTYKRFQAIISRLELPKSPVGSVTLEQMENCTTDVEANHSEKYGIPSLDELGFNTSELGPATWNGGEIEALA
RLNKHLERKAWVANYERPKLNADSLVSSPTGLSPYLRFGCLSCRLFYYRLQELYKTVKKGAIPPLSLYGQLLW
REFFYTTATNNPKFDRMEGNPICVQIPWDKNPHALTKWADGLTGFPWIDAIMKQLRKEGWIHHLARHAVSCFL
TRGDLWISWEEGMKVFEELLLDADFSINAGSWMWLSCSAFFQQFFHCYCPVGFGKRTDPSGDYIRRYLPLLKE
YPTKYIYEPWNAPLAVQKAAKCIIGIDYPKPIVNHAEASRLNIERMKQIYQQLSHYKGLCLLPSVPSCTDDSA
SLAVRTHVDCTNTAALNSSYHVQSPVVMGKREKPECFTEDGCIIAKIPRLQSPEGNGRIV
>Callorhinchus_mil_XP_007893621.1_cry-1

MGGNSIHWFRKGLRLHDNPALMKTVLTSDRVRCVYILDPWFAGSSNVGINRWRFLLQCLEDLDANLRKLNSRL
FVIRGQPADVFPRLFKEWDISLLSFEYDSEPFGKERDAAIKKLASEAEVKVIVDVSHTLYNLDKIIELNGGQP
PLTYKRFQTLISRMEPLDLPVETITREVMGRCTTPILDDHDEKYGVPSLEELGFDTEGLSSAVWPGGESEALT
RLERHLERKAWVANFERPRMNSNSLLASPTGLSPYLRFGCLSCRLFYFKLTDLYKKVKKNGSPPLSLYGQLLW
REFFYAAATNNPRFDKMEGNPICVQIPWDRNAEALAKWAEGKTGFPWIDAIMTQLRQEGWIHHLARHAVACFL
TRGDLWISWEEGMKVFEELLLDADWSVNAGSWMWLSCSSFFQQFFHCYCPVGFGRRTDPNGDYIRRYLPVLRG
FPAKYIYDPWNAPESVQKAAKCILGVHYPKPMVNHAEASCLNIERMKQIYQQLSCYRGLGLLDSVPSNPNGNG
GIMGYSAGENTHGCNNLTGDLPSTNGNHSLPYTQSEFQLRNNQQQSYITGVNGQGSNLLCFSQRDSQLSHTTL
LQQGRNTPMSGKRHSPEEEMQGFGPKVQRQNSN
>Calonectris_bore__NXV89335.1
IRRCRSPDPALAECYRVPLPVDLKIPPESLSPWRGGETEGLQRLEQHLTDQGWVASFTKPRTIPNSLLPSTTG
LSPYFSLGCLSVRTFFYRLSNIYAQAKHHSLPPVSLQGQLLWREFFYTVASATPNFTKMAGNPICLQIRWYED
AERLHKWKTAQTGFPWIDAIMTQLHQEGWIHHLARHAVACFLTRGDLWISWEEGMKVFEELLLDADYSINAGN
WMWLSASAFFHRYTRIFCPVRFGKRTDPEGQYIRKYLPILKNFPSKYIYEPWTASEEEQKQAGCIIGRDYPFP
MVNHKEASDHNLQLMKQVREEQHGTAQLTRDDTDDPMEMKVKRDH
>Calonectris_bore__NXV89640.1
RFLQEWGVTRLTFEYDSEPFGKERDAAIVKLAKEAGVEVVIENSHTLYDLDRIIELNGHKPPLTYKRFQAIIS
RMELPKKPVSTVMSQQMETCKVDIQENHDDVYGVPSLEELGFPTDGLAPAVWQGGETEALARLDKHLERKAWV
ANYERPRMNANSLLASPTGLSPYLRFGCLSCRLFYYRLWELYKKVKRNSTPPLSLYGQLLWREFFYTAATNNP
KFDRMEGNPICIQIPWDRNPEALAKWAEGKTGFPWIDAIMTQLRQEGWIHHLARHAVACFLTRGDLWISWESG
VRVFDELLLDADFSVNAGSWMWLSCSAFFQQFFHCYCPVGFGRRTDPSGDYVKRYLPKLKGFPSRYIYEPWNA
PESVQKAAKCIIGVDYPKPMVNHAETSRLNIERMKQIYQQLSRYRGLCLLASVPSCVEDLSGPVADSASGQGC
STSTAMRLSQADQASPKRKHEGAEELCTEELYKRAKVTCLPAPEIPGKSL
>Calonectris_bore__NXV90725.1
KVDYDVCSNYGNWLYSAGIGNDPRDNRKFNMIKQGLDYDGNGDYVRLWVPELQAIKGADIHTPWALNSAALSQ
AGVTLGETYPQPVVTAPEWSRHINQR
>Calonectris_bore__NXV93746.1
MQHSSIHWFRKGLRLHDNPALLAAAADCHRLHPLFILDPSSSRTGANARRFLLDALRDLDGSLREMGSRLFVV
RGCPEEVFPRLFHAWGTTRLTFEVDTEPPARRRDTAVAELAARHGVEVIREVSHTLYDTERVLALNDGKVPLT
YKRLQSLLASLGPPEKPAPALTREHLQGCRTPCQVSHDADYGVPTLEELGEDPAELGPHLYPGGETAALARLD
TLMERTTWVCGFKKPETEPTSLSPSTTVLSPYLKFGCLSVRTFWWRLDEVYQGREHSQPPVSLHGQLLWREFF
YTAGASIPNFDRMVGNPVCLQVDWDDNPQHLRAWREGRTGYPFIDAIMTQLRAEGWIHHLARHAVACFLTRGD
LWVSWEEGLKVFEDLLLDADWSLNAGNWLWLSGSAFFHRYFHVYSPITFGKKTDRDGAYIRKYLPVLKDFPAA
YIYEPWKAPRAVQERAGCLVGTHYPRPIVEHGAASKRNLERMKAAR
>Calonectris_bore__NXV94998.1
MGVNAVHWFRKGLRLHDNPALRECIQGADTVRCVYILDPWFAGSSNVGINRWRFLLQCLEDLDANLRKLNSRL
FVIRGQPADVFPRLFKEWNIAKLSIEYDSEPFGKERDAAIKKLASEAGVEVIVRISHTLYDLDKIIELNGGQP
PLTYKRFQTLISRMEPLEMPVETITPEVMEKCTTPVSDDHDEKYGVPSLEELGFDTDGLPSAVWPGGETEALT
RLERHLERKAWVANFERPRMNANSLLASPTGLSPYLRFGCLSCRLFYFKLTDLYKKVKKNSSPPLSLYGQLLW
REFFYTAATNNPRFDKMEGNPICVQIPWDRNPEALAKWAEGRTGFPWIDAIMTQLRQEGWIHHLARHAVACFL
TRGDLWISWEEGMKVFEELLLDADWSVNAGSWMWLSCSSFFQQFFHCYCPVGFGRRTDPNGDYIRRYLPVLRG
FPAKYIYDPWNAPESVQKAAKCIIGVNYPKPMVNHAEASRLNIERMKQIYQQLSRYRGLGLLATVPSNPNGNG
NGGLMGYSPGESISGCGSTGHLFPSSPGAQLGTGDGHTVVQPCALGDSHTGASGIQQQGYCQASSILHYAHGD
NQQSHLLQAGRTAVGTGISAGKRPNPEEETQSVGPKVQRQSTN
>Calonectris_bore__NXV98579.1
DNWAFLYAQRLALKQELPLHVCFCLVPKFLDATIRHYGFMLRGLQEVAKECTELNIPFHLLLGYAKDVLPAFV
VEHGVGGLVTDFCPLRLPRQWVEDVREQLPDDVPFAQVDAHNIVPCWVASPKQEYGARTIRGKIHAQLPEFLT
EFPPVVRHPYLPSCPAEPIAWEACYSSLQVDRTVKEVDWATPGTAAGLAVLQSFIVERLKSFSSHRNDPNKAA
LSNLSPWFHFGQVSTQRAILEVQKHRGKYKESVDAFVEEAVVRRELAENFCYYNENYDSVQGAYDWAQTTLKL
HAKDKRPFLYDLQELEQGTTHDPLWNAAQLQMVQEGKMHGFLRMYWAKKILEWTRSPEEALRFAIYLNDRYEL
DGRDPNGYVGKLQDRGCLWSICGIHDQGWAERAIFGKIRYMNYAGCKRKFDVGQFERRYAPRT
>Calypte_anna__KFO95415.1
RFLLQCLEDLDANLRKLNSRLFVIRGQPADVFPRLFKEWNIAKLSIEYDSEPFGKERDAAIKKLATEAGVEVI
VRISHTLYDLDKIIELNGGQPPLTYKRFQTLISRMEPLEMPVETITPEVMEKCTTPVSDDHDEKYGVPSLEEL
GFDTDGLPSAVWPGGETEALTRLERHLERKAWVANFERPRMNANSLLASPTGLSPYLRFGCLSCRLFYFKLTD
LYKKVKKNSSPPLSLYGQLLWREFFYTAATNNPRFDKMEGNPICVQIPWDKNPEALAKWAEGRTGFPWIDAIM
TQLRQEGWIHHLARHAVACFLTRGDLWISWEEGMKVFEELLLDADWSVNAGSWMWLSCSSFFQQFFHCYCPVG
FGRRTDPNGDYIRRYLPVLRGFPAKYIYDPWNAPESVQKAAKCIIGVNYPKPMVNHAEASRLNIERMKQIYQQ
LSRYRGLGLLATVPSNPNGNGNGGLMGYSPGESVSGCGSTGGAQLGTGDGHTVVQPCALGDSHTGASGIQQQG
YCQASSILHYAHGDNQQSHLLQAGRTTLGTGISAGKRPNPEEETRGVGPKVQRQSTS

>Calypte_anna__KFP00512.1
DNWAFLYAQRLALKQELPLHICFCLVPKFLDATIRHYGFMLRGLQEVAKECAELNIPFHLLLGYAKDVLPAFV
VDHGVGGLVTDFCPLRLPRQWVEDVKERLPEDVPFAQVDAHNIVPCWVASPKQEYSARTIRGKIHAQLPEFLT
EFPPIIPHPYSPSCPAEPIAWEACYSSLQVDRTVKEVEWATPGTAAGMAVLKSFITERLKSFSSHRNDPNKAA
LSNLSPWFHFGQVSTQRAILEVQKHRRKYKESVDAFVEEAVVRRELAENFCYYNENYDSVKGAYDWAQTTLNL
HAKDKRPFLYKLQELEQGTTHDPLWNAAQLQMVQEGKMHGFLRMYWAKKILEWTRSPEEALQFAIYLNDRYEL
DGRDPNGYVGCLWSICGIHDQGWAERAIFGKIRYMNYAGCKRKFDVGEFERRYTP
>Calypte_anna__XP_008500450.1
MTAICLLRCDLRAHDNQVLHWAQSNAEFVVPLYCFDPRHYLGTHCYGFPKTGPHRLRFLLESVKDLRETLKRK
GSTLVVRKGKPEDVVHDLITQLGSVSAVAFHEEATQEELDVEKGVSQVCKQHGVRTRTFWAATLYHRDDLPFR
PIVRLPDVFTQFRKVVESQVKVRPTLQMADHLKPLPPGLEEGCIPTMEDLGQKDPVTDPRTAFPCSGGETQAL
MRLQYYFWDTNLVASYKETRNGLVGMDYSTKFAPWLALGCISPRYIYEQIQKYEKERTANESTYWVLFELLWR
DYFRFVALKYGNRIFSLRGLQNKYIPWKKDLQLFDCWKEGNTGVPFVDANMRELSASGFMSNRGRQNVASFLT
KDLGLDWRMGAEWFEYLLVDYDVCSNYGNWLYSAGVGNDPREHRKFNMVKQGLDYDGKGDYVRLWVPELQSIK
GADIHTPWALNTAALSQAGVTLGETYPQPVVTAPEWSRHINQKPQGRSPHPRGKRGPAHTPRQHRDRGIDFYF
SHKKDL
>Calypte_anna__XP_030307868.1
MAAMAAPAAVPCSPPVPCSAPVPAFCRSVHWFRRGLRLHDNPALQEALRDAASLRCIYILDPWFAASSAVGIN
RWRFLLQSLEDLDNSLRKMNSRLFVVRGQPTDVFPRLFKEWGVTRLTFEYDSEPFGKERDAAIIKLAKEAGVE
VVIENSHTLYDLDRIIELNGHKPPLTYKRFQAIISRMELPKKPVSTIMSQQMETCKVDIQENHDDVYGVPSLE
ELGFPTEGLGPAVWHGGETEALARLDKHLERKAWVANYERPRMNANSLLASPTGLSPYLRFGCLSCRLFYYRL
WELYKKVKRNSTPPLSLYGQLLWREFFYTAATNNPKFDRMEGNPICIQIPWDRNPEALAKWAEGKTGFPWIDA
IMTQLRQEGWIHHLARHAVACFLTRGDLWISWESGVRVFDELLLDADFSVNAGSWMWLSCSAFFQQFFHCYCP
VGFGRRTDPSGDYVKRYLPKLKGFPSRYIYEPWNAPEAVQKAAKCIIGVDYPKPMVNHAETSRLNIERMKQIY
QQLSRYRGLCLLASVPSCVEDLGGPVTDSTSGQGCSTSAAMRLLQADPASPKRKHEGAEELYSEELYKRAKMT
GLPASEIPDKSS
>Camelus_drom__XP_010994769.1
MGVNAVHWFRKGLRLHDNPALKECIQGADTIRCVYILDPWFAGSSNVGINRWRFLLQCLEDLDANLRKLNSRL
FVIRGQPADVFPRLFKEWNITKLSIEYDSEPFGKERDAAIKKLATEAGVEVIVRTSHTLYDLDKIIELNGGQP
PLTYKRFQTLISKMEPLEIPVETITSEVIEKCTTPLSDDHDEKYGVPSLEELGFDTDGLPSAVWPGGETEALT
RLERHLERKAWVANFERPRMNANSLLASPTGLSPYLRFGCLSCRLFYFKLTDLYKKVKKNSSPPLSLYGQLLW
REFFYTAATNNPRFDKMEGNPICVQIPWDKNPEALAKWAEGRTGFPWIDAIMTQLRQEGWIHHLARHAVACFL
TRGDLWISWEEGMKVFEELLLDADWSINAGSWMWLSCSSFFQQFFHCYCPVGFGRRTDPNGDYIRRYLPVLRG
FPAKYIYDPWNAPEGIQKVAKCLIGVNYPKPMVNHAEASRLNIERMKQIYQQLSRYRGLGLLASVPSNPNGNG
SLMGYSPGENIPGCSSSGSCTQGSGILHYAHGDSQQAHLLKQGKSSTGTGLSSGKRPSQEEDTQSIGSKVQRQ
STN
>Camelus_drom__XP_010998928.2
MAAAVATTAAAAPAPAAGAEGATSVHWFRKGLRLHDNPALLAAVRGAHCVRCVYILDPWFAASSSVGINRWRF
LLQSLEDLDTSLRKLNSRLFVVRGQPADVFPRLFKEWGVTRLTFEYDSEPFGKERDAAIMKMAKEAGVEVVTE
NSHTLYDLDRIIELNGQKPPLTYKRFQTIISHMELPRKPVGSVTSQQMESCRAEIQENHDETFGVPSLEELGF
PTEGLGPAVWQGGETEALARLDKHLERKAWVANYERPRMNANSLLASPTGLSPYLRFGCLSCRLFYYRLWDLY
KKVKRNSTPPLSLFGQLLWREFFYTAATDNPRFDRMEGNPICIQIPWDRNPEALAKWAEGKTGFPWIDAIMTQ
LRQEGWIHHLARHAVACFLTRGDLWVSWESGVRVFDELLLDADFSVNAGSWMWLSCSAFFQQFFHCYCPVGFG
RRTDPSGDYIRRYLPKLKGFPSRYIYEPWNAPESIQKAAKCIIGVDYPRPMVNHAETSRLNIERMKQIYQQLS
RYRGLCLLASVPSCVEDLSNPVAEPSSSQAGSMSSAGLRPLPGGPASPKRKLEAAEEPPGEELSKRARVAELP
STELPGRDV
>Camponotus_flo_EFN74495.1_cry-1
MTGSSNNEMVQGVASGVRGDGRKHTVHWFRKGLRLHDNPSLKEGLAGASTFRCVFVLDPWFAGSTNVGINKWR
FLLQCLEDLDCSLRKLNSRLFVIRGQPADALPKLFKEWGTTNLTFEEDSEPFGRVRDHNISALCKELGISVVQ
KVSHTLYKLDEIIERNNGKPPLTYHQFQNIVASMNPPEPPVSTVTAACIGNAYTPLKDDHDDHYGVPTLEELG
FDTEGLLPPVWVGGESEALARLERHLERKAWVASFGRPKMTPQSLLPSQTGLSPYLRFGCLSTRLFYYQLTDL
YKKVQDDCNPIVIKKAVPPLSLHGQLLWREFFYCAATKNSNFDRMQGNPICVQIPWDKNVEALAKWANGQTGF
PWIDAIMTQLREEGWIHHLARHAVACFLTRGDLWISWEEGMKVFDELLLDADWSVNAGMWMWLSCSSFFQQFF
HCYCPVRFGRKADPNGDYIRRYLPVLKNFPTRYIHEPWNAPLSIQHAAKCVIGKEYSLPMVNHNKTSRINIER
MKQVYQQLNKYRGE
>Candidatus_Lok_TFG08969.1_photolyase
MKKTRVRNLSKPKKSKGAIVYWMSRDQRVNDNWALIFAQDMAIREKLPMMVIFCLIPSFLGATLRQYDFMLSG
LEQIHEKLTELNIPFKIMSGFPQQEIPKFIDENDVGCLITDFSPLRIKRNWIKSIKEKLTIHFYEVDTHNIIP
CWTASPKREYAAYTFRPKVRKLLPQYLDEFPPMKKMPDKSLENDLGINWEGLKKHLKIDPSVLPINWIQSGEK
AAAEKLKNFMKDKLADYDALRNDPNKNGQSELSPYLHFGQISAQRIVLELNKIKKNGKSKNAFLEELVVRREL

SDNFCFYTPEYDKFDSFPPWAKQTLKKHEKDKREYLYSLDELEGAKTHDDAWNAAQIEMVTKGKMHGYMRMYW
GKKILEWTKTPEKALEIALYLNDKYEIDGRDPNGYVGVAWSIGGVHDRAWKEREIFGKVRYMSYDGLKRKFNL
KQYIERYINKS
>Candidatus_Pos_DAC31551.1_TPA:_photo
MQVAIVWFRNSLRIHDNPVLSWAYESENIEAILPIYIFDEEINQRGTQTMGEHRLRFQFDCVEDLHENLKSKL
DLDLHIFSGNCVAVLESINEQLQPYEVILLTEYSTSPQDREQNEVIENNIITTHQTWSAKAIPSSQTILDVEA
ITSSPKYKPPKSMKDMVRLFKLEFGDFENIVFQTNELNPSLSSRKKGLSVNSQYQTSLDKIRPLLTTPGYFRG
GENEAIARLENKVISQHDYVNSFNKPKSISTNVKGNPFEPTTTGLSPYISTGCLSIRRLWNSCSEIQQSSEHT
VPPVSLHGQILFREMFYLLSRSTANWDQDIGNSQCKEIEWGELNQVQLDAWESGQTGFPLIDAMMRQLNATGW
MHHLGRHAVSCFLTRGQLWQNWTHGRDVFERKLVDSDWAVNNGNWLWLAGVAPFSMPYYRIYNPCPDAKSSLN
VETKDAEFIRHWVPELAEFPSKYIFEPNLAPLHIQESAGCLIGKDYPSPIIDRKESRKKNLQSFKLSLENLR
>Candidula_unif__CAG5117961.1
MAKRASSSAGSKTRNSTKAAPAENDPKKEVKKAVKRKQEPDEVVPVKSKKPSKEIPESEDSAKSVKNGEDKKL
STTGDFIEKIIQKRAAVCESVAKFKFNKKRVRVITDMEEFSLSSNGVLYWMSRDQRVQDNWAFLYAQRLALKL
EAPLHVCFCLVPTFLEATIRQFGFMMKGLAEVEKECRDLNIPFHLLLRVASTSVPNFVKENNIGGIVTDFSPL
RTPLKWLDDLKKALPKDVPLCQVDAHNLVPCWVASPKLEYAARTIRTKIHNQLEGFLTDFPPLVQHPHPPDKM
PSVTDWKAADEFLKVDRTVQEVSWAKPGSIHGLRMLEDFCENRLKNFATHRNDPNKKALSNLSPWLHFGQISA
QRCILTVKQYQGRHKDSVNAFIEETVIRRELADNFCFYNENYDSIKGTYDWAQKTLEAHRGDKRQYLYSRKDL
ELAKTHDNLWNAAQIQMVTEGKMHGFLRMYWAKKILEWTKSPEEALVEAIYLNDRYNLDGRDPNGFVGCMWSV
CGVHDQGWMERPVFGKIRYMNYEGCKRKFDVIAFCTRYRKLKA
>Candidula_unif__CAG5120301.1
MEELEFHDSVDSKRHVTVHWFRHGLRLHDNPALLAGLRGCREFYAVFIFDGAVAGISTAAFPRMQFLMETLQD
LDENLKTCGSRLHVFRGNPVEVFGKLIEEWGVTRVSLEQDPEPIWQKRDSDVKALCSKREVEWIERVSHTLWD
PQLIIQENGGSPPLTYAMFCQVMEIVGQPPVPVRNPDFSGVSLPVSDNHDQKYGLPPLDALGVKAESDHQASP
YCRYLGGETKALKLLSSRLEKERAAFSRGLSLPNQLYPDLTGMPMSLSPHLRFGSLSIRKLYWSLRLVYTQVH
PNAPVPASITSQLIWREYFYCMSVNNPAYNRMKENPICLNIDWYDDENKFQQWKTGQTGYPWIDACMRQLMQE
GWIHQVCRHATACFLTRGDLWIDWQKGLEVFDRYLLDADWSVCAGNWMWVSSSAFENVLQCARCICPVRYGRR
MDPSGEYVRRYVPELKGMPLQYLFEPWKAPITVQQEAGCIIGQDYPSPMVDHKIASQECKLRMDMVRKRSEDV
AHIGPASEAEALSFLWVTKHSNAEIMEVCNHNSL
>Candidula_unif__CAG5124456.1
MKDNTNEHFMSQTQLHSHGTKRHTLFWFRKGLRLHDNPALIAAIENATTYRCVYILDPWFAGASQVGINKWRF
LLECLEDLDSSLRKLNSRLFVVRGQPADVLPRLFQEWGINTLAFEEDPEPYGKERDAAISAMVREFNIQVIAK
SSHTLYDPKIVIAANGNSPPLTYKRFQSILATLEPPRQPCETLTGQMLVNVSTPVADDHDDRFSVPSLDELGF
DTDTLGPAQFRGGESEALARLYRHLERKAWVASFERPKMSPQSLYPSGTGLSPYLRFGCLSPRTFYWKLAELY
KKVKKGVDPPLALHGQLLWREFFYTVATNNPNFDRMVGNSICVQIPWEHNPEALAKWAEGLTGFPWIDAIMVQ
LRKEGWIHHLARHAVACFLTRGDLWISWEEGMKVFDEMLLDADWSVNAGMWMWLSCSAFFQQFFHCYCPVGFG
KRADPSGDFVRHYLPVLKGIPTQYIYEPWTAPESVQKAAKCIIGKDYPLPMVQHAEACRVNLERMRQVYKRLV
LKSTANKRIFLDLPKIPKVSMFLSKAKMFLSSSSDEEVHQKQRRRKPVDEEFTHQS
>Capitella_tel_ELT97477.1_CAPTEDRAFT
MPEPPLKKQKSSSSSSSSSKHVSSDVFLERIKGLREDAASSISDFSFNKNRVRTLTPCKKPSPDSKGVLYWMSR
DQRVQDNWALLYSQKLALDLEVPLYINFCLVPKFLEATIRQFGFMLKGLQEVEKECKSLRISFHLHLGHATDH
LPQFVEDHNIGAIVTDFCPLRVPTKWVTDLVKSLPDEIPLFQVDAHNIVPCWEASPKQEYGARTIRNKIHNQL
SKYFTEFPPVIQHAFEAKAPCPAVDWIEAEKSLEVDRSVKEVDWATPGTTAGLKNLESFIQKRLGNFDADRNN
PNKNALSNMSPWFHFGHVAPQRAMLMVNEMKTKYSKSVAAYIEEGVIRRELCDNYCFYQPNYDNIDGAYEWAR
LTLKAHRKDKREYVYSKEQLEKGKTHDDLWNAAQTQLLIEGKMHGFLRMYWAKKILEWTETPERGLEEAIYLN
DKYSLDGRDPNGYVGCMWSICGIHDQGWAERAVFGKIRFMNYNGCKRKFDVAAFVSRYRTQAAKLK
>Capitella_tel_ELU04573.1_CAPTEDRAFT
MCEENAQIPGEPSKKKKKNVLYWFRRCLRLHDNAALVEVLKEADTFRCIFILDPWFAGASQVGINKWRFLLQS
LEDLDSRLRKLNSRLFVIRGQPTDIFPKLFQKWDISALAFEEDPEPFGKERDSAVCTKSQDAGIEVIIKTSHT
LFNLQKILDKNSGVPPLTYKRFQRILARMDPPPRPVEAVTSVTIGSVVTPINSDHDDQYGVPTLEDLGFDTDN
LEAAVWKGGETEALSRLDRHLERKAWVASFEKPKMTPQSLMASPTGLSPYLRFGCLSTRLFYWRLTDLYRKVK
KRTDMPLSIHGQLLWREFFYTAATNNPKFDRMVGNPICVQVPWDKNPEALAKWAECKTGFPWIDAIMTQLRQE
GWIHHLARHSVACFLTRGDLWISWEEGMKVFEEQLLDADWSINAGMWLWLSCSSFFQQFFHTYCPASFGRKAD
PTGDYIRKYLPVLKAFPTKYIYEPWTAPMEIQVAVRCVIGVDYPLPIVNHAEISQINMERMKQIYHQISLKSG
PLSKYVRRRQRHMMEMTEGVSRHVFMYGPGNPQNIPNLSSARGIPLQAAKWQMSLQQSKRNHSSSESDQDRKV
VRGDSRHMMMHNQ
>Capitella_tel_ELU12703.1_CAPTEDRAFT
MPSEGTRKSLRHRETLTGEKGDGDRDQVVVHWFRHGLRLHDNPSLMEGLRNCKELYPIFILDGEVAGTGTAGY
NRMRFLHQCLEDLDKSFQKFGGRLYIFKGNPVDILAALFDEWQVTKLTFEQDPEPIWEDRDNKVKDLCMKRDV
TYVERISHTLFHPDDVIEANGGNPPHTFSLMKQVLNMLGDPERPSSSPDLSLVSLPLSNDFDDRCALDTLKDF

AGEYEMEEQEEPINKWIGGESRALDLLQGRLKLEENAFKGGFLQPNQYRPDLIGPPLTLSPHLRFGCLSVRYF
YWAIHDIYSEVREELAPQSITSQLIWREYFYIMSVKNRNYAQMENNPICLQIPWYRDDNQLERWEMGKTGYPW
IDACMNQLRREGWIHHVGRHAVSCFLTRGDLWIHWEDGLKVFLKYLLDADWSVCAGNWMWVSSSAFENCLQCP
QCFSPIMYGMRMDPTGEYVRRYVPELRQVPLKYLFHPWKAPKEVQEKAGCIVGTDYPAPMVDHNTASKDCKDR
MQAVKALYKDPLSILHCAPSNMQEVRQFCWLPDTMATGKMCTADLLCEGIEGM
>Capsaspora_owc_XP_004347271.1_photolyase
MPRWSIPVRAAATAVGEAAAVKAASAAATPASSEASATVGTRAKRRASVDASPSHQPKHARQESDADGDTDQN
PSPPSSNPLLQLGSLLHAPPYVTKPKMVDERRIRRILPTGADASAPPMPIPSGHDGVLYWMSRDQRVQDNWAL
IYAQQLAIQHRVPLFVGFCLVPHFLDATIRQYDFMMEGLREVETALRGLNISFHLFTGTAAEHLPGFVESKRL
GAVVTDFSPVRVIQRWTTELANSLAQTTIATTGATQSHTGIPLIQLDAHNVVPCWHASPKLEYAARTIRPKLH
KLLSEFLTEFPEVAAHPCSARQSPSSPQSARASSAASSNAHFQSGLSAPVDWDAARASLQVNRTITAVPDIVP
GTRAGMTTLRTFVEQRMRYFGAVRNDPNERVLSNLSPYFHFGQIAPQRAILVAYAAHARCRESVDVFVEEALV
RRELSDNFCFYNPNYDSIAGASAWAQETLQAHAGDAREFVYTEEELESATTHDLLWNAAQLEMVREGKMHGFM
RMYWAKKILEWTASPSEALRIAIHLNDKYNLDGRDPNGYVGCMWSICGIHDQGWGERPIFGKIRFMNLKGCQR
KFDVAKYTARFPSTPSSQQAAKRRGAAAGGIDRGSNYARATTTTSNRSTS
>Capsaspora_owc_XP_004349794.2_photolyase
MSLPPPQPPQSRSLSQLLLQHPPSQAAAAAAVPQESRNACHWFRINALRLDDNPALLAAIEYSSSSSSSSSAP
SASAPTTTSASASHSGTAEPHNTLHNVFVLDPSDATIPSSGLPLVFLLESLTNLQSRLTWLGSHLTILRGQVE
QVLPDYLSKHDISLVTYEESTPEPASSQVAQRLEQALHPLRIRQRLETSAFVTHTLYDMNMLLVACKNTPPLT
YGKFLSLIESIGPPVAPLDAPHRLAERGPAGQTDAATGGIPSLAELGISEPHTPLMFHGGETAGQARMHHYLG
DLARVANFSKPELDPTLFEEPSSSVMSPYLRFGCVSPRRFFFEINKVYIDSGVTPTQPPVSLLGQLYWREFFY
LVAAGTPNFDRAAGNPICKQIQWDVNPEYLAAWESGRTGYPWIDAAMNQLREVGWMHHLARHAVACFLTRGDL
FLSWELGRDYLQRTLLDADWSISNANWMWMSASAFFHAYFRVYSPITFAQHYDPEGKFVRRFVPALRNFPSEY
IYMPWSAPLEVQREAGCIVGVDYPKPIVDHEQVCRTNMERMRDAYAQQPDAAALMAVSAVSMLTRQPHDAVTL
AQMHGQSSGTLSIKSSDSISAPFFKPLDTSANGVADTSRLGDTTSVHSGSTDSIPRAHQKTASEEAARVTNDA
RRWSTTPVPVLSRDLGQILQQWHADMLQRAQAASQLVHHNHSDEAHRHASEAHHHALEPDSSTSLSSGSVTPP
SQVGSAGTISNREVHFDPALHSMMATEVVGQLHPAVGLVADHLNAAISTRVTRRSSEAFGDSHAAAAAAAAAS
QPGSAKRSRASTPPSVETSTRCTSPRKR
>Caranx_mela__KAG7216105.1
MTKEGDSNQSEKPKRSKNRRQRRKAGGQQQVVGLPCSPSASRPVLLWFRRDLRLCDNPALIGSLEVGAPVIPV
FIWSPEEEEGPGVTVAVGGACKYWLHQALSCFCSSLETIGSQLVFLKANGESNGIGSVSHFMSCCKQNPGSAL
GVPLDSPESLPTPDHWPQGVPLDTLGLARMPRRKDGTTIDWAANIRKSWDFSEEGAHARLEAFLHDGVYRYEK
ESGRADAPNTSCLSPYLHFGQLSPRWLLWDAKGARCRPPKFQRKLAWRDLAYWQLSLFPDLPWESLRPPYKTL
RWSTDRGHLKAWQRGRTGYPLVDAAMRQLWLTGWMNNYMRHVVASFLIAYLHLPWQEGYCWFQDTLVDADVAI
DAMMWQNGGMCGLDHWNFVMHPVDAAMTCDPYGTYVRKWCPELADLPDELIHKPWKCPASMLRRAGVVFGQTY
PDRIITDLEERRSQSLQDVALVRKKFGQYVDKRSGCDLVPLPRASSLRPWAYRTAMVMW
>Caranx_mela__KAG7222414.1
MAQTCIHWFRKGLRLHDNPALLAALRDCKELYPVFILDPYLHNNTRMGINCWRFLIGALKDLDCSLRKLNSRL
FVVSGKPEDVFPKLFNKWKVTKLTYEYDTEPYSLSRDKKVTELAKDHQVEVIYRISHTLYDTDRIIEENNGKA
PLTYNRMQAIVKTLGPPKKPMPAPTMEDIKDVKTPCSENHEKTYGIPGLDKLGHNTAAIGEECFPGGEQEALR
RLVEHMERTAWVCSFEKPQTSPNSLSPSTTVLSPYVTFGCLSARTFWWRLTEVYQGKKHSDPPVSLHGQLLWR
EFFYTASVGIPHFNKMEGNPVCTQVDWDTNPEYLAAWREARTGFPFIDAIMTQLRQEGWIHHLARHAVACFLT
RGDLWISWEEGQKVFAELLLDGDWALNAGNWQWLSASTFFHQFFRVYSPVAFGKKTDKNGDYIKKYLPFLKKF
PAQYIYEPWKAPRSIQQAAGCIVGKDYPHPIVQHEVISKKNIQRMKLAYAKRPSNPTESPSKKQGITRKKLEV
HPF
>Caranx_mela__KAG7224924.1
MIILSVQLFHWAQRNAEYIVPLYCFDPRHYVGTYNYNLPKTGPFRLRFLLESIRDLRNTLLNKGSNLVVRRGK
PEDVVAELIKQLGSVSTVAFHEEVTSEELNVETKVKDVCAQMKVKVHTCWGCTLYHRDDLPFHHMSRLPDVYT
QFRKAVETQSMVRPAFPTPEQLKPLPPGLEEGAIPTAQDLQQTGECPHDKVATYKETRNGLIGVDYSTKFAPW
LAVGCISPRYIYHQIKQYERERTANQSTYWVIFELLWRDYFKFVAVKYGNRLFQLKGLQDKSVPWKKDMKLFN
AWKEGRTGVPFVDANMRELAMTGFMSNRGRQNVASFLTKDLGLDWRMGAEWFEYLLIDHDVCSNYGNWLYSAG
IGNDPRENRKFNMVKQGLDYDNNGDYVRQWVPELQGIRGADVHTPWTLSTAALSHAQVSLGETYPTPIVMAPE
WSRHVNKKPIDHDVCSNENLVRSTDTVVDYDNNLDRLRNPEETRLVCCYYVFFIRVLTKRTAVSLRCFTKNST
NMAYADPDVDMDSIPLSALNVTVRKKLGLYLNPRNAVAADWMSVAEEMDFTYLEIQNYVETKNPTAMVLDDWQ
ARSTDATVGKLLSILKKVDRSDAVEDLQPFIDEDVRRYCENQKKKADPPLQVPEVDSCVPRTPGITLEDQEGA
PELFDAFICYCQSDFHFVHEMIRELEQTEYRLKLCVFDRDVLPGSCVWTITSELIERRCKRMVVVISDEYLDS
DACDFQTKFALSLCPGARNKRLIPVVYKPMTKPFPSILRFLTVCDYTRPCTQAWFWVRLAKALSLP
>Caranx_mela__KAG7227261.1
LLEPSNCYKTKHERAHVTGCVKWEQHSYIRVRPRQLVFVLFLFGLLCGRSFSEHLLCCMLLCIWTRSSFSSAP
RRLLKLITHSDQRHTLIPPLLIKSFTSETIMSDKKRKATSAPAGKEPTAKQQKLVPGKEGKKERAEGWLQEQL

REQRSEKKEMKFHKKRLQFISDTEKIKQGSEGVLYWMSRDHRVQDNWALIHAQRLALKEKLPLHICFCLVVPK
SELSTLRHYSFLLKGLEEVAKECKALDIQFHLLRGSAGEVLPGFVSDRSLGAVVTDFYPLREPLQQLEDVKKK
LPKDIPLIQVDAHNIVPCWVASPKLEYAARTIRSKITKLLPEFLTDFPLVEKHPYTATRTAKPFDWAKTLASL
QVDRTVGEPKWATPGTKGGMAMLESFIDVRLKLFDTLRNDPNAAALSSLSPWIRFGHVSAQRVALQVQRSGKS
SGQSVASFIEELVVRRELTDNFCFYNKKYDRKTLKDHAKDKRPYLYTRQQLEEAKTHDKLWNAAQYQMVIEGK
MHGFLRMYWAKKILEWTSSPEEALSIALYLNDRYELDGQDPNGFVGCMWSICGIHDQGWAERPVFGKIRYMNY
KGCTRKFDVARFERTVTSGGGGDMIHKVLRWVICNVERICQLLE
>Caranx_mela__KAG7231144.1
MVVNSVHWFRKGLRLHDNPALQEALNGADTVRCVYILDPWFAGAANVGINRWRFLLEALEDLDSSLKKLNSRL
FVIRGQPTDVFPRLFKEWKVTRLTFEYDPEPYGKERDGAIIKMAQEFGVEAIVRNSHTLYNLDRIIEMNNNSP
PLTFKRFQTIVSRLELPRRPLAPITQQQMDKCRTNIADNHDQLYSIPSLEELGFRTEGLPPAVWRGGESEALD
RLNKHLDKKVWVANLEKPRVSTCSLYASPTGLSPYLRFGCLSCRVLYYNLRELYMKLRKRCSPPLSLFGQLLW
REFFYTAATNNPNFDRMEGNPICVQIPWDQNPEALAKWAEGRTGFPWIDAIMTQLRQEGWIHHLARHAVACFL
TRGDLWISWESGMKVFEELLLDADWSVNAGSWMWLSCSAFFQQFFHCYCPVGFGRRTDPSGDYIRRYIPILKD
YPNRYIYEPWNAPELVQKAANCVVGVDYPKPMINHAEGSRLNIERMKQVYQQLSHYRGLSLLASVPTIQEEAE
PPMTDESQTSSGPDSPPRDPVDSEAAGCSTAPDSSTVCASSAFAPHPILEDTLNTPQTPATHTQPSAAVSSTS
ASNPPPLAAFPSPSSTPIQSPLSRSKPSSPSSSCPTLSPSPSPASNLAQTFPKRKGPTRKVRRSQRQRGRQSC
PPSGREGERRMEEEEKKETAGDESMEENVEPEEERMEEETSGEPAGLQQ
>Caranx_mela__KAG7244469.1
MVVNTIHWFRKGLRLHDNPSLRDSIRGADTLRCIYILDPWFAGSSNVGINRWRFLLHCLEDLDASLRKLNSRL
FVIRGQPTDVFPRLFKEWQITRLSYEYDSEPFGKERDAAIQKLANEAAVEVMVRISHTLYDLDKIIELNGGQP
PLTYKRFQALINRMDPVELPAETITAEVIKKCASPITSDHDDKYGVPSLEELGFEIEGLTTAVWPGGETEALM
RLERHLERKAWVANFERPRMNANSLLASPTGLSPYLRFGCLSCRLFYFKLTDLYRKVKKNSTPPLSLYGQLLW
REFFYATATNNPCFDKMEGNPVCVQIPWDRNPEALAKWAEGRTGFPWIDAIMTQLRQEGWIHHLARHAVACYL
TRGDLWISWEEGMKVFEELLLDADWSVNAGSWMWLSCSSFFQQFFHCYCPVGFGRRTDPNGDYIRRYLPILRG
FPARYIYDPWNAPEEVQKAAKCIIGVHYPKPMVNHAEASRINIERMKQIYQQLSCYRGLGLLATVPANPNNGA
NGSNVTGVSTGTSQGQGGSSEHPSTQEGTSRTERGQLTQKRRREEAPSETSSKSWKQSK
>Carcharodon_carc__XP_041029294.1
MNLSRLFSITCQSFCHRHSVICFFRLSFRVWVQSAKQRSNMLKNRKQANREPGCDAEGSDYSKKIEESSKKMA
TTFSGSSNSVNTSKKESRKELNQNDSNQKEALNLRAKEKRKVKEITPGLNEGELSNPSNGKKRKVEQQTKAAP
SRGGDLSESIKKSRLSTATSVEEFNYNKKRVRLLSKTKGIRDNCQGIIYWMSRDQRVQDNWAFLYAQWLALKH
KLPLHVCFCLVPKFHDATIRHFDFMLKGLQEVAEECKQLDISFHLLIGFAKDVLPEFVKENNIGSVVTDFSPL
RSPLQWVENVRHNLPEDLQFVQLIDWDAAYAKLQVDLKVKEVDWAKPGTAAGLTRMESFIEEQLKYFSTDRNN
PNKTALSNMSPWFHFGQISVQRAILEIRKYRNKFKDSVDSYIEEAVVRRELADNFCFYNKQYDKIEGASDWAQ
KTLKIHSKDQRSHLYTLKQLEDGKTHDPLWNAAQLQMVLEGKMHGFLRMYWAKKILEWTTSPEEALQFSIYLN
DRYQLDGKDPNGYVGCMWSICGIHDQGWAERPVFGKIRYMNYNGCKKKFDVGQFERKYNHKKLTKPL
>Carcharodon_carc__XP_041052556.1
MTLEWQSRLERFLLQCLEDLDSSLRKLKSRLFVVQGQPTDVFPRLFKEWNMTRLTFEYDSEPFGKARDATIVK
LAKNAGIEVVVRTSHTLYDVDRIIEANGNNPPLTFKHFQTIIRRLEVPKAPVNMVTLDQMEKCTTAVQENHDE
IYGIPSLEELGFDTSELGPAVWQGGETEALARLYKHLQKKASVTNYEKPKLSADSLLSSPIGLSPYLRFGCLS
CRTFYYTLLQLYRMIKKHSNPPLSLFGKLLWREFFYTAATNNPSFDKLQGNPICMQIPWDKNQQALTKWAHGQ
TGFPWIDAIMKQLRQEGWIHQLARHAVACFLTRGDLWISWEEVMKVFDEMLLDADFSVNAGSWMWLLCSALFQ
QFFQFYCPVGFGRRTDPSGDYVRRYLPVLQGYPTEYIYEPWNAPVEVQKAANCIVGVHYPKPMINHLETSQLN
IERMKQIYQKLSHHKGFCLVALVPS
>Carcharodon_carc__XP_041071962.1
MGRHSIHWFRKGLRLHDNPALLEALLGSDTIRCVYIVDPWFAGSSGVGINRWRFLLQCLEDLDASLWKLNSRL
FVVRGQPADVFPRLFKEWNISRLTFEYDSEPFSKERDAAIKKLAIEAGVEVIVQISHTLYDLDIIIERNGGQP
PLTYKHFQTLISRMESIEMPVGTITAEAMGKCTTPVFDDHDEKYGVPSLEELGFDTEGLPSAVWPGGETEALK
RLERHLERKAWVANFERPRMNSTSLLASPAGLSPYLRFGCLSCRLFYFKLTDLYKKVKKNSSPPLSLYGQLLW
REFFYAAATNNPRFDKMEGNPICVQVPWDRNPEALAKWAEGKTGFPWIDAIMTQLRQEGWIHHLARHAVVCFL
TRGDLWISWEEGMKVFEELLLDADWSVNAGIWMWLSCSSFFQQFFHCYCPVGFGRRTDPNGDYIRRYLPALRG
FPAKHIYDPWNAPESVQKAAKCIIGVHYPKPMVNHAEASRLNIERMKQIYQQFSCYRGLGLLASVPSNPNALG
GAMDYSPGESTHRCSSITGRSCLSSSLSSGKQPSPGEQMPAEGPKVHRQSSN
>Cariama_cris__KFP56213.1
HLAYSISRIIELNGHKPPLTYKRFQAIISRMELPKKPVSTVISQQMETCKVDIQENHDDVYGVPSLEELGFPT
DGLAPAVWQGGETEALARLDKHLERKAWVANYERPRMNANSLLASPTGLSPYLRFGCLSCRLFYYRLWELYKK
VKRNSTPPLSLYGQLLWREFFYTAATNNPKFDRMEGNPICIQIPWDRNPEALAKWAEGKTGFPWIDAIMTQLR
QEGWIHHLARHAVACFLTRGDLWISWESGVRVFDELLLDADFSVNAGSWMWLSCSAFFQQFFHCYCPVGFGRR
TDPSGDYVKRYLPKLKGFPSRYIYEPWNAPESVQKAAKCIIGVDYPKPMVNHAETSRLNIERMKQIYQQLSRY

RGLCLLASVPSCVEDLSGPVTDSASGQGCSTSTAMRLSQADQASPKRKHEGAEELCTEELYKRAKVTGLPAPE
IPGK
>Cariama_cris__KFP63074.1
DNWAFLYAQRLALKQELPLHVCFCLVPKFLDAPIRHYGFMLRGLQEVAEECAELNIPFHLLLGCAKDVLPAFV
VEHGVGGLVTDFCPLRLPRQWVEDVRERLPEDVPFAQVDAHNIVPCWVASPKQEYSARTIRGKIHDQLPEFLT
EFPPVVRHPYPPSCPAEPIAWEACYSSLQVDRTVKEVEWATPGTAAGLAVLQSFIAERLKSFGSHRNDPNKAA
LSNLSPWFHFGQVSTQRAILEVQKHRGKYKESVDVFVEEAVVRRELAENFCYYNDNYDSVQGAYDWAQTTLKL
HAKDKRPFLYELQELEQGTTHDPLWNAAQLQMVREGKMHGFLRMYWAKKILEWTRSPEEALQFAIYLNDRYEL
DGRDPNGYVGCLWSICGIHDQGWAERAIFGKIRYMNYAGCKRKFDVSQFERRYAP
>Cariama_cris__XP_009694612.1
LPDVYTHFRKAVESEAKVRPTLRMADQLKPLAPGVEEGCIPTMEDLGQKDPVTDPRTAFPCSGGETQALMRLQ
YYFWDTNLVASYKETRNGLVGMDYSTKFAPWLALGCISPRYIYEQIQKYEKERTANQSTYWVLFELLWRDYFR
FVALKYGRRIFSLRGLQSKEVPWKKDLQLFDCWKEGKTGVPFVDANMRELAATGFMSNRGRQNVASFLTKDLG
LDWRMGAEWFEYLLVDYDVCSNYGNWLYSAGIGNDPRDNRKFNMIKQGLDYDGNGDYIRLWVPELRGIKGADI
HTPWALNSAALSQAGVTLGETYPQPVVTAPEWSRHINQRPQGRSPHLRGRRGPAHTPMQHKDRGIDFYFSRKK
DV
>Cariama_cris__XP_009696013.1
ILDLNGGTPPLTYKRFLHVLSLLGDPEVPVRNVTAEDFQRCRPPDPSLAERYRVPLPVDLKIPLESLSPWRGG
ETEGLQRLEQHLTDQGWVANFTKPRTIPNSLLPSTTGLSPYFSMGCLSVRTFFYRLSNIYAQAKHHSLPPVSL
QGQLLWREFFYTVASATPNFTKMAGNPICLQISWYEDAEKLHKWKTAQTGFPWIDAIMTQLRQEGWIHHLARH
AVACFLTRGDLWISWEEGMKVFEELLLDADYSINAGNWMWLSASAFFHRYTRIFCPVLFGKRTDPEGQYIRKY
LPILKNFPSKYIYEPWTASEEAQKQAGCIIGQDYPFPMVNHKEASDHNLQLMKQVREEQYRTAQLTRDDTDDP
MEMKVKRDHSEENISKGKVVRMTEKNEIPPEITSWEPKDGKRGEPEAS
>Cariama_cris__XP_009703473.1
FLLQCLEDLDANLRKLNSRLFVIRGQPADVFPRLFKEWNIAKLSIEYDSEPFGKERDAAIKKLASEAGVEVIV
RISHTLYDLDKIIELNGGQPPLTYKRFQTLISRMEPLEMPAETITPEVMEKCTTPVSDDHDEKYGVPSLEELG
FDTDGLPSAVWPGGETEALTRLERHLERKAWVANFERPRMNANSLLASPTGLSPYLRFGCLSCRLFYFKLTDL
YKKVKKNSSPPLSLYGQLLWREFFYTAATNNPRFDKMEGNPICVQIPWDKNPEALAKWAEGRTGFPWIDAIMT
QLRQEGWIHHLARHAVACFLTRGDLWISWEEGMKVFEELLLDADWSVNAGSWMWLSCSSFFQQFFHCYCPVGF
GRRTDPNGDYIRRYLPVLRGFPAKYIYDPWNAPESVQKAAKCIIGVNYPKPMVNHAEASRLNIERMKQIYQQL
SRYRGLGLLATVPSNPNGNGNGNGGLMGYSPGESISGCGSTGGAQLGTGDSHTVVQPCALADSHTGASGIQQQ
QGYCQASSILHYAHGDNQQSHLLQAGRTALGTGISAGKRPNPEEETQSVGPKVQRQSTN
>Castor_cana__XP_020027965.1
MAAAVGTVAATVPAPATGADGASSVHWFRKGLRLHDNPALLAAVRGARCVRCVYILDPWFAASSSVGINRWRF
LLQSLEDLDTSLRKLNSRLFVVRGQPTDVFPRLFKEWGVTRLTFEYDSEPFGKERDAAIMKMAKEAGVEVVTE
NSHTLYDLDRIIELNGQKPPLTYKRFQAIISRMELPKKPVGSVTSQQMESCRADIQENHDDTYGVPSLEELGF
PTEGLGPAVWQGGETEALARLDKHLERKAWVANYERPRMNANSLLASPTGLSPYLRFGCLSCRLFYYRLWDLY
KKVKRNSTPPLSLFGQLLWREFFYTAATNNPRFDRMEGNPICIQIPWDRNPEALAKWAEGKTGFPWIDAIMTQ
LRQEGWIHHLARHAVACFLTRGDLWVSWESGVRVFDELLLDADFSVNAGSWMWLSCSAFFQQFFHCYCPVGFG
RRTDPSGDYIRRYLPKLKGFPSRYIYEPWNAPESIQKAAKCIIGVDYPRPIVNHAETSRLNIERMKQIYQQLS
RYRGLCLLASVPSCVEDLSHPVAEPGSNQAGSISSMGPRPLPSGPASPKRKLEAAEEPPGEELNKRARVAELP
APELPSKDS
>Castor_cana__XP_020036490.1
MGVNAVHWFRKGLRLHDNPALKECIQGADTIRCVYILDPWFAGSSNVGINRWRFLLQCLEDLDANLRKLNSRL
FVIRGQPADVFPRLFKEWNITKLSIEYDSEPFGKERDAAIKKLATEAGVEVIVRISHTLYDLDKFQTLISKME
PLEIPVETITSEVIEKCTTPLSDDHDEKYGVPSLEELGFDTDGLSSAVWPGGETEALTRLERHLERKAWVANF
ERPRMNANSLLASPTGLSPYLRFGCLSCRLFYFKLTDLYKKVKKNSSPPLSLYGQLLWREFFYTAATNNPRFD
KMEGNPICVQIPWDKNPEALAKWAEGRTGFPWIDAIMTQLRQEGWIHHLARHAVACFLTRGDLWISWEEGMKV
FEELLLDADWSINAGSWMWLSCSSFFQQFFHCYCPVGFGRRTDPNGDYIRRYLPVLRGFPAKYIYDPWNAPEG
IQKVAKCLIGVNYPKPMVNHAEASRLNIERMKQIYQQLSRYRGLGLLASVPSNPNGNGGLMGYAPGENIPGCS
NSGSCSQGSGILHYAHGDSQQTHLLKQGRSSMVTGLSGKRPSQEEDTQSIGPKVQRQSTN
>Casuarius_casu__NXE46009.1
RFLLQCLEDLDANLRKLNSRLFVIRGQPADVFPRLFKEWNIAKLSIEYDSEPFGKERDAAIKKLASEAGVEVI
VRISHTLYDLDKIIELNGGQPPLTYKRFQTLISRMEPLEMPVETITPEVMEKCTTPVSDDHDEKYGVPSLEEL
GFDTDGLPSAVWPGGETEALTRLERHLERKAWVANFERPRMNANSLLASPTGLSPYLRFGCLSCRLFYFKLTD
LYKKVKKNSSPPLSLYGQLLWREFFYTAATNNPRFDKMEGNPICVQIPWDKNPEALAKWAEGRTGFPWIDAIM
TQLRQEGWIHHLARHAVACFLTRGDLWISWEEGMKVFEELLLDADWSVNAGSWMWLSCSSFFQQFFHCYCPVG
FGRRTDPNGDYIRRYLPVLRGFPAKYIYDPWNAPESVQKAAKCIIGVNYPKPMVNHAEASRLNIERMKQIYQQ
LSRYRGLGLLATVPSNPNGNGNGGLMGYSPGENISACGSTGGAQLGTGDGHTVGVQTCALGDSHTGASGIQQQ
GYCQASSILHYAHGDNQQSHLLQTGRTSLGTGISAGKRPNPEEETQSIGPKVQRQSTN

>Casuarius_casu__NXE46688.1
KVDYDVCSNYGNWLYGAGIGNDPRDNRKFNVIRQGLDYDGSGDYVRLWVPELRDLKGGDIHAPWALSSASLSQ
AGIALGETYPRPVVTAPEWSRHISQRPQGRGPHPKGRKGPAHTPMQHKDRGIDFYFSRKKDL
>Casuarius_casu__NXE51766.1
DNWAFLYAQRLALKQELPLHVCFCLVPKFLDATIRHYGFMLRGLQEVAKECTELNIPFHLLLGYAKDVLPTFV
VRLGVGGLVTDFCPLRVPQQWVEDVRERLPEDVPFVQVDAHNIVPCWVTSPKQEYNARTIRSKIHSQLPEFLT
EFPPVIQHPYSPSTPAEPIAWEACYSSLQVDRTVKEVEWATPGTASGLAVLQSFIAKRLKSFGSHRNNPNKAA
LSNLSPWFHFGQVSTQRAILEVWKHHRKYKESVDIFVEEAVVRRELADNFCYYNKNYDSVQGAYNWAQNTLKL
HAKDKRPFLYELQELEQGNTHDPLWNAAQLQMVREGKMHGFLRMYWAKKILEWTCSPEEALQFAIYLNDRYEL
DGRDPNGYVGKLHKGAWGCLWSICGIHDHGWPERAVFGKIRYMNYAGCKRKFDVVQFERRYAPCK
>Casuarius_casu__NXE54110.1
MPHRTIHLFRKELRLHDNPTLLAALESSEVVYPIYILDRKFMTSVMNIGALRWHFLLQSLENLQKNLLQLDSC
LLVIQGEYGAVLRDHVKKWNITQVSLDAEVEPFYKEMEANIRSLGEELGFEVLSLIGHSLYDAKRILDLNGGT
PPLTYKRFLHILSLLGDPEAPVRNLAAEDFQRCRPPDLCLAECYRVPLPVDLKIPTECLSPWKGGETEGLQRL
EQHLMNQGWVASFTKPRTIPNSLLPSTTGLSPYFSLGCLSVRTFFYRLSNIYAQSKHHSLPPVSLQGQLLWRE
FFYTVAAATPNFTKMSGNPICLQINWYEDAERLHKWKTAQTGFPWIDAIMTQLRQEGWIHHLARHAAACFLTR
GDLWISWEEGMKVFEEMLLDADYSINAGNWMWLSASAFFHQYTRIFCPVRFGKRTDPEGQYIRKYLPILKNFP
SKYIYEPWTASEEEQKEAGCIIGQDYPFPMVNHKEVSDHNLQLMKRVREEQYRTAQLTRDDADDPMEMKMKRD
HSEENVKKGKVARTTE
>Casuarius_casu__NXE56516.1
CIYILDPWFAASSAVGINRWRFLLQSLEDLDNSLRKLSSRLFVVRGQPTDVFPRLFKEWGVTRLTFEYDSEPF
GKERDAAIVKLAKEAGVEVVIENSHTLYDLDRIIELNGHKPPLTYKRFQAIISRMELPKKPVSSIISQQMETC
KVDIQENHDDVYGVPSLEELGFPTDGLAPAVWQGGETEALARLDKHLERKAWVANYERPRMNANSLLASPTGL
SPYLRFGCLSCRLFYYCLWELYKKVKRNSTPPLSLYGQLLWREFFYTAATNNPKFDRMEGNPICIQIPWDRNP
EALAKWAEGKTGFPWIDAIMTQLRQEGWIHHLARHAVACFLTRGDLWISWESGVRVFDELLLDADFSVNAGSW
MWLSCSAFFQQFFHCYCPVGFGRRTDPSGDYVKRYLPKLKGFPSRYIYEPWNAPESVQKAAKCIIGVDYPKPM
VNHAETSRLNIERMKQIYQQLSRYRGLCLLASVPCVEDLSGQVADPALGHSSSTSTVMRLPQSDQTSPKRKHE
GAEELCTEELYKRAKVGGLPAPEIPGKSL
>Catenaria_ang_ORZ31450.1_FAD_bindin
MKKLTTLLWFRRDLRLHDHPALLHAVSTSATLFPVYCLEVPTKKRCGHLRWLFLLESLADLDSQLRALGSRLF
VVRGKAPDVLPQLVKAWAVDQVLVQSSPEPFARARDEEVAQRVAKEGGEFHGFPGRTLFEVSQLLAGAGNGAS
DKGIPLPVTMTGFAKVADRLTVPKPEPAPTSIPLPLDSESALHQLKSLLDPVSFASLTGPHHDLSLPRISPTD
HTVPELGLLTPLAARSPHTGGESHALARLDTYMSHTAKVLAFEKPKTSPAALPDPVTGLVDTTVLSPYMTYGC
LSPRLFHSRITSAYRAAKGKHSKPPVSLLAQLYWREFFFAHGATVDGFELATEANPTCPTPIDWRLLSAADSD
PEAHDDLKAWAEARTGYPWIDACMTQLRSEGWIHHLGRHSLACFLTRGDLYISWERGAEVFEDVLLDHDWALN
RGNWAWLSGTSVFFRQWFRVYSPVKWGQSWDAEGKYIKRHLPQLAKFPKQYIYEPWKAPLAVQKQAGCIVGKD
YPKPIVDHAEASKANMAKLKACFARGRDKNGAGEDEGDADEDIEKDKVGSSRAGRAAARDRVKRK
>Catenaria_ang_ORZ37966.1_DNA_photol
MPKAAASSSKKGNKRSHADITDESTDEQPNTMSPASHSRPASAAIAAAASAATKHARRSGPVVFWFRKDLRLR
DNKGLYKAWVQAAHVNRTATTGGGDPMNGALLGIFVVCGRQWHHHDLSPAKVDLMLRSVQSLQERMESPQLNI
PLTILHARNIKHVPQVVKEYLAKVKATHLYFNVEYEVDEGKRDDTVAELVQQAPLNVTCVPTHDECVVPPGLV
KTKDGTRTPVVYTPFRKSWESLVKSKQPYDYLALSPAPEPNPMAVRTCKLGAGLFGTRVPDPTADGVDATAEL
PGFEREFVPSVLEHTRANYLGTEDAAHARLVAFAHNKIKSYSVDRDFPNLDGTANISAYLALGLISARQCITI
AREANNGLLDSGDKGCVHWIQEIVWREFYRHILVAFPRVCMNQPFKLDTLNVPWTSGDEEEAYFTLGARARRL
KTTGFMSNRLRMITAMFLTKDLLVDWRKGEKYFMQHLVDGDLASNNGGWQWAASTGTDSQPYFRIFNPWLQSV
KFDPRGDFIRKYVPELRPITKAASVHEPHTKLTTSEFMRLGYVQPIVDHKKARDRVLKAFKVDPDQAH
>Centropus_beng__NXX91158.1
KVDYDVCSNYGNWLYSAGVGNDPRENRKFNMIKQGLDYDGNGDYVRLWVSELQGIKGADIHIPWALSSAALSQ
AGVILGATYPLPVVTTPEWSRHISQSLQGRSPHPKGRRAPAHTPALHRNRGIDFYFSHKKDV
>Centropus_beng__NXX91360.1
RFLLQSLEDLDNSLRKLNSRLFVVRGQPTDVFPRLFKEWGVTRLTFEYDSEPFGKERDAAIVKLAKEAGVEVV
IENSHTLYDLDRIIELNGHKPPLTYKRFQAIISRMELPKKPVSTVMSQQMETCKVDIQENHDDVYGVPSLEEL
GFPTDGLAPAVWQGGETEALARLDKHLERKAWVANYERPRMNANSLLASPTGLSPYLRFGCLSCRLFYYRLWE
LYKKVKRNSTPPLSLYGQLLWREFFYTAATNNPKFDRMEGNPICIQIPWDRNPEALAKWAEGKTGFPWIDAIM
TQLRQEGWIHHLARHAVACFLTRGDLWISWESGVRVFDELLLDADFSVNAGSWMWLSCSAFFQQFFHCYCPVG
FGRRTDPSGDYVKRYLPKLKGFPSRYIYEPWNAPESVQKAAKCIIGVDYPKPMVNHAETSRLNIERMKQIYQQ
LSRYRGLCLLASVPSCVEELSGPVTDSASGQGCSTSTAMRLSQTDQASPKRKHEGAEELGTEELYKRAKVTGL
PAPEIPGKSL
>Centropus_beng__NXX98999.1

DNWAFLYAQRLALKQELPLHVCFCLVPKFLDATIRHYGFMLKGLQEVAKECAELNISFHVLLGYAKDVLPAFV
VEHGVGGLVTDFCPLRLPRQWVEDVRERLPEDVPFAQVDAHNIVPCWVTSPKQEYSAWTIRSKIHAQLPEFLT
EFPSVIRHPHSPSCPAQPIAWEACYSSLQVDRTVGEVEWATPGTAAGLAVLHSFITERLKFFSSHRNDPNKAA
LSNLSPWLHFGECPQASHPTAHRTSSPTCSLPVPPGQVSTQRAILEVQKHRSKYKDSVDVFVEEAVVRRELSE
NFCYYNENYDSVQGAYDWAQTTLKLHAKDKRPFLYKLQELEQGTTHDPLWNAAQLQMVREGKMHGFLRMYWAK
KILEWTNSPEEALRFAIYLNDRYELDGRDPNGYVGCLWSICGIHDHGWGERAVFGKIRYMNYAGCKRKFDVGQ
FERRYGPCT
>Centropus_beng__NXX99936.1
RILDLNGGTPPLTYKRFLHILSLLGDPEVPVHTPTAEDFQSLTPRGAELCLCSQGLGRLHLESWGQFWAPHSK
KDLEGLERVQRREWSWGRGWVASFTKPRTIPNSLLPSTTGLSPYFSMGCLSVRTFFYRLSNIYAQAKHHSLPP
VSLQGQLLWREFFYTVAAATPNFTRMAGNPICLQISWYEDAEKLHRWKTAQTGFPWIDAIMTQLRQEGWIHHL
ARHAVACFLTRGDLWISWEEGMKVFEELLLDADYSINAGNWMWLSASAFFHQYTRIFCPVRFGKRTDPEGRYI
RKYLPILKNFPSKYIYEPWTASEEEQKQAGCIIGEDYPFPMVDHKEASDHNLELMRQVREEQHRTAQLTRDDA
DDPMEMKVKREHSEECTSKGKVAKVTE
>Centruroides_scu_XP_023242183.1_cry-1-like
MSNSKEKNVVHWFRKGLRLHDQPSLREGLINSTTWRCVFILDPWFAGSSNVGINKWRFLLQCLDDLDASLRKL
NSRLFVIRGQPADVFPRLFKEWKVTHLTFEKDPEPYGRIRDQNITVMAQEMGITVISRTSHTLYNLEKIIKKN
GDKTPLTYKQFQSVLATMEPPPPPQPTITQELLKNSSTPIFEDHDDKYGVLTLEELGFDTEGLKPAIWQGGET
ESLIRLERHLERKAWVASFGRPKMTPQSLLPSQTGLSPYLRFGCLSARLFYQQLTDLYKKIKKANPPLSLHGQ
LLWREFFYCSATNNPNFDRMLNNPICVQIPWDINPEALAKWANGQTGFPWIDAIMSQLREEGWIHHVARHAVA
CFLTRGDLWISWEEGMKVFEELLLDADWSVNAGSWMWLSCSSFFQQFFHLYCPVRFGRKADPNGDYIRRYLPV
LKNFPTKYLHEPWTAPEKVQVAVKCIIGKDYPLPIVNHQDVSRINLERMKQVYQQLNQYHGVFYQVFQHNPHC
LLHPQIIHQKCLKAQNINLMSASTIITISQHLSNLNIQKLN
>Ceratotherium_simum_simum_XP_014647998.1_PREDICTED:
MAAAAVVTGAGAAPAAAAAAAAAAAAAGADGASSVHWFRKGLRLHDNPALLAAVRGARCVRCVYILDPWFAASS
SVGINRWRFLLQSLEDLDTSLRKLNSRLFVVRGQPADVFPRLFKEWGVTRLTFEYDSEPFGKERDAAIMKMAK
EAGVEVVTENSHTLYDLDRIIELNGQKPPLTYKRFQALISRMELPKKPVGSVTSQQMESCRAEIQENHDETYG
VPSLEELGFPTEGLGPAVWQGGETEALARLDKHLERKAWVANYERPRMNANSLLASPTGLSPYLRFGCLSCRL
FYYRLWDLYKKVKRNSTPPLSLFGQLLWREFFYTAATNNPRFDRMEGNPICIQIPWDRNPEALAKWAEGKTGF
PWIDAIMTQLRQEGWIHHLARHAVACFLTRGDLWVSWESGVRVFDELLLDADFSVNAGSWMWLSCSAFFQQFF
HCYCPVGFGRRTDPSGDYVRRYLPKLKGFPSRYIYEPWNAPESVQKAAKCIIGVDYPRPIVNHAETSRLNIER
MKQIYQQFSRYRGLCLLASVPSCVEDLSNPVAEPSSSQAGSMSSAGPRPPPSGPASPKRKLEAAEEPPGEELG
KRARVAEPPAPELPGRDV
>Ceratotherium_sim_XP_014636064.1_cry-1_X2
MGVNAVHWFRKGLRLHDNPALKECIQGADTIRCVYILDPWFAGSSNVGINRWRFLLQCLEDLDANLRKLNSRL
FVIRGQPADVFPRLFKEWNITKLSIEYDSEPFGKERDAAIKKLATEAGVEVIVRISHTLYDLDKIIELNGGQP
PLTYKRFQTLISKMEPLEIPVETITSEVMEKCTTPLSDDHDEKYGVPSLEELGFDTDGLPSAVWPGGETEALT
RLERHLERKAWVANFERPRMNANSLLASPTGLSPYLRFGCLSCRLFYFKLTDLYKKVKKNSSPPLSLYGQLLW
REFFYTAATNNPRFDKMEGNPICVQIPWDKNPEALAKWAEGRTGFPWIDAIMTQLRQEGWIHHLARHAVACFL
TRGDLWISWEEGMKVFEELLLDADWSINAGSWMWLSCSSFFQQFFHCYCPVGFGRRTDPNGDYIRRYLPVLRG
FPAKYIYDPWNAPEGIQKVAKCLIGVNYPKPMVNHAEASRLNIERMKQIYQQLSRYRGLGLLASVPSNPNGNG
GLMGYSPGENIPGCSSSGRRSSMGTGLSSGKRPSQEEDTHSIGPKVQRQSTN
>Cervus_cana__XP_043295754.1
MGVNAVHWFRKGLRLHDNPALKECIQGADTIRCVYILDPWFAGSSNVGINRWRFLLQCLEDLDANLRKLNSRL
FVIRGQPADVFPRLFKEWNITKLSIEYDSEPFGKERDAAIKKLATEAGVEVIVRISHTLYDLDKIIELNGGQP
PLTYKRFQTLISKMEPLEIPVETITSEVIEKCTTPLSDDHDEKYGVPSLEELGFDTDGLPSAVWPGGETEALT
RLERHLERKAWVANFERPRMNANSLLASPTGLSPYLRFGCLSCRLFYFKLTDLYKKVKKNSSPPLSLYGQLLW
REFFYTAATNNPRFDKMEGNPICVQIPWDKNPEALAKWAEGRTGFPWIDAIMTQLRQEGWIHHLARHAVACFL
TRGDLWISWEEGMKVFEELLLDADWSINAGSWMWLSCSSFFQQFFHCYCPVGFGRRTDPNGDYIRRYLPVLRG
FPAKYIYDPWNAPEGIQKVAKCLIGVNYPKPMVNHAEASRLNIERMKQIYQQLSRYRGLGLLASVPSNPNGNG
GLMGYSPGENIPGCSSNASCTQGSGILHYAHGDSQQTHLLKQGRSSTGTGLGSGKRPSQEEDTQSIGPKVQRQ
STN
>Cervus_cana__XP_043336967.1
MAAAAAGTTSAAAAAQAPAARGDGASSVHWFRKGLRLHDNPALLAAVRGAHCVRCVYILDPWFAASSSVGINR
WRFLLQSLEDLDRSLRKLNSRLFVVRGQPADVFPRLFKEWGVTRLTFEYDSEPFGKERDAAIMKMAKEAGVEV
VTENSHTLYDLDKIIELNGQKPPLTYKRFQAIISRMELPRKPVGSMTSPQMEGCRAEIQENHDETYGVPSLEE
LGFPTEGLGPAVWRGGETEALARLDKHLERKAWVANYERPRMNANSLLASPTGLSPYLRFGCLSCRLFYYRLW
DLYKKVKRNSTPPLSLFGQLLWREFFYTAATNNPRFDRMEGNPICIQIPWDRNPEALAKWAEGKTGFPWIDAI
MTQLRQEGWIHHLARHAVACFLTRGDLWVSWESGVRVFDELLLDADFSVNAGSWMWLSCSAFFQQFFHCYCPV
GFGRRTDPSGDYIRRYLPKLKGFPSRYIYEPWNAPESIQKAAKCVIGVDYPRPIVNHAEASRLNIERMKQVYQ

QLSRYRGLCLLASVPSCVEDLSNPVAEPSSSQAGSSSSAGPRPLPGGPASPKRKLEAAEEPPGGELSKRARVA
ESLPSELPSRGV
>Chaetura_pela__KFU84257.1
DNWAFLYAQRLALKQELPLHVCFCLVPRFLDATIRHYGFMLRGLQEVAKECTELNIPFHLLLGYAKDVLPTFV
AEHGVGGLVTDFCPLRVPRQWVEDVKERLPLDVPFAQVDAHNIVPCWVASPKQEYSARTIRGKIHAQLPEFLT
EFPPVIRHPYPPSCPAEPIAWEACYSSLQVDRTVKEVEWATPGTAAGLAVLQSFIKERLKSFGSHRNDPNKAA
LSNLSPWFHFGQISTQRAILEVRKHRDKYKESVDAFMEEAVVRRELAENFCYYNENYDSVQGAYDWAQTTLNL
HAKDKRPFLYKLQELEQGATHDPLWNAAQLQMVREGKMHGFLRMYWAKKILEWTRSPEEALQFAIYLNDRYEL
DGRDPNGYVGKLQERSTGRRGCLWSICGIHDQGWAERAIFGKIRYMNYAGCKRKFDVGQFERRYAP
>Chaetura_pela__KFU85512.1
GANARRFLLEALQDLDSSLREIGSRLFVARGCPEEVLPHLFRAWGTTRLTFEVDTEPLTRQSDATVAELAAQH
GVEVIREVSHTLYDTERVLALNNGKAPLTYKHLQSLLASLGPPEKPAPALTQEHLQGCFTPCQASHDINYGIP
TLEELGHDPSEVGPQLYPGGETAALARLDMFMERTAWVCGFRKPETEPTSLRPSTTVLSPYLTLGCLSVRTFW
WRLDEVYQRQREHSQPPVSLHGQLLWREFFYTAGASIPNFDRMVGNPVCLQVDWDDNPQHLCAWREGRTGYPF
IDAIMTQLRTEGWIHHLARHAAACFLTRGDLWISWEKGLKVFEELLLDADWSLNAGNWLWLSGSAFFHRYFHI
YSPVAFGKKTDRDGAYIRKYLPILKDFPAKYIYEPWKAPQAVQERAGCLVGTHYPLPIVEHGVARERNLGRMK
>Chaetura_pela__KFU91102.1
RFLLQSLEDLDNSLRKLNSRLFVVRGQPTDVFPRLFKEWGVTRLTFEYDSEPFGKERDAAIVKLAKEAGVEVV
IENSHTLYDLDRIIELNGQKPPLTYKRFQAIISRMDLPKKPVSTVMSQQMETCKVDIQENHDDVYGVPSLEEL
GFPTDGLAPAVWQGGETEALARLDKHLERKAWVANYEKPRMNANSLLASPTGLSPYLRFGCLSCRLFYYRLWE
LYKKVKRNSTPPLSLYGQLRGREFFFPAATNNPKFDRMEGNPICIQIPWDRNPEALAKWAEGKTGFPWIDAIM
TQLRQEGWIHHLARHAVACFLTRGDLWISWESGVKVFDELLLDADFSVNAGSWMWLSCSAFFQQFFHCYCPVG
FGRRTDPSGDYVKRYLPKLKGFPSRYIYEPWNAPESVQKAAKCIIGVDYPKPMVNHAETSRLNIERMKQIYQQ
LSRYRGLCLLASVPSCVEDLSGPVTDSASGQGCSTSAAMRLSQADQASPKRKHEEAEELCTEELYKRAKVTGL
PAAEIPGKS
>Chaetura_pela__XP_009992545.1
MSHRTIHLFRKGLRLHDNPTLLAALESSQVIYPVYILDEKFMTSVMHMGTLRWHFLLQSLEDLQKNLCQLGSC
LLVLQGEYELVLRDHVQKWKITQVTLDAEMEPFYKEMEANIRHLGDELGFEVLSLVGHTLYNTQRILELNGGN
PPLTYKRFLHILSLLGDPEVPVRSVTAEDFQRCRPPDPGLAGCHRVPLPVDLKIPLENLSPWRGGETEGLRRL
ERHLTDQGWVANFTKPRTIPNSLLPSTTGLSPYFSLGCLSVRTFFYRLSNIYAQAKDHSLPPVSLQGQLLWRE
FFYTVASATPNFTRMAGNPICLQISWYEDAEQLHKWKTAQTGFPWIDAIMTQLRQEGWIHHLARHAAACFLTR
GDLWISWEEGMKVFEELLLDADYSINAGNWMWLSASAFFHQYTRIFCPVRFGKRTDPQGHYIRKYLPVLKNFS
SKHIYEPWTASEEEQKEAGCIIGRDYPFPMVNHPAASAHNLQRMRQVREEQHRTAQLTRDDTDDPMEMKVKRD
HSEENVSRGKVTRMGEQTEVPSGIAPGEPKSGQGGDPEES
>Chaetura_pela__XP_010004154.1
MAVFLLQCLEDLDANLRKLNSRLFVIRGQPADVFPRLFKEWNIAKLSIEYDSEPFGKERDAAIKKLASEAGVE
VIVRISHTLYDLDKIIELNGGQPPLTYKRFQTLISRMEPLEMPVETITPEVMEKCTTPVSDDHDEKYGVPSLE
ELGFDTDGLPSAVWPGGETEALTRLERHLERKAWVANFERPRMNANSLLASPTGLSPYLRFGCLSCRLFYFKL
TDLYKKVKKNSSPPLSLYGQLLWREFFYTAATNNPRFDKMEGNPICVQIPWDKNPEALAKWAEGRTGFPWIDA
IMTQLRQEGWIHHLARHAVACFLTRGDLWISWEEGMKVFEELLLDADWSVNAGSWMWLSCSSFFQQFFHCYCP
VGFGRRTDPNGDYIRRYLPVLRGFPAKYIYDPWNAPESVQKAAKCVIGVNYPKPMVNHAEASRLNIERMKQIY
QQLSRYRGLGLLATVPSNPNGNGNGGLMGYSPGESVSGCGSTGGAQLGTGDGHTVVQPCALGDLHTGASGIQQ
QGYCQASSILHYAHGDNQQLHLLQAGRTTLGTGISAGKRPNPEEETQSVGPKVQRQSAS
>Chaetura_pela__XP_010004307.1
MEERKDEETEEKSRYCLLNPASGFAEFFLENVICNSVTSTEHAKRKTIGARDMVLSWAESSSDFVIPLYCFDP
RHYLGTRSFGFPRTGPHRLRFLLESVRDLRETLKKKGSTLVVRKGKPEDVVCDLITQVGSVSAVAFHEEATQE
ELDVEKGLCQVCSQHGVKIQTFWASTLYHRDDLPFRPIARLPDVYTHFRKAVESQVKVRPTLPVADQLKPLPP
GLEEGCIPTMEDLGQKDPVTDPRTAFPCSGGETQALMRLQYYFWDTNLVASYKETRNGLVGMDYSTKFAPWLA
LGCISPRYIYEQIKKYEKERTANESTYWVLFELLWRDYFRFVALKYGNRIFSSRGLQSKEIPWKKDLQLFDCW
KEGRTGVPFVDANMRELAATGFMSNRGRQNVASFLTKDLGLDWRMGAEWFEHLLVDYDVCSNYGNWLYSAGVG
NDPRDNRKFNMVKQGLDYDGNGDYVRLWVPELQGIKGADVHTPWALNSAALSQVGITLGDIYPQPLVTAPEWS
RHISQRLQGRCPHPRGRRGPAHTPMQHKGRGIDFYFSPKKDV
>Channa_argu__KAF3686229.1
MLSNNDFLRKHEPSGNREARFRLVSMAHQCIHWFRKGLRLHDNPALMAALRDCKELYPVFILDPNLYNTTSVG
INRWRFLIGALKDLDCSLKKLDSRLFVVRGKPEDVFPELFKKWKITAMTYEYDTEPYSLTRDKKVAALAKEHG
VKVVYKISHTLYDIDRIIEENNGKPPLAYKRLQAIVKSLGPPKRPIPAPTMEDMEDVTTPCSEQHEQTYGIPT
LEELGRDTAALGEELFPGGEQEALRRLDEHMKRTGWVCSFEKPETSPNSLSPSTTVLSPYVTFGCLSARTFWW
RLADVYQGKKHSLPPVSLHGQLLWREFFYTASVGIPNFNKMESNPVCTQVDWDTNPEYLAAWREARTGFPFID
AIMTQLRQEGWIHHLARHAVACFLTRGDLWISWEEGQKVFEDLLLDGDWALNAGNWQWLSASTFFHQFFRVYS

PVAFGKKTDKRGDYIRKYLPFLKKFPAEYIYEPWKAPRSVQQAAGCIVGKDYPRPIVQHEVVSKRNVQRMKVA
YAKRSPHPTESPSKSPSKKRGDVIMDEILFKKQRKV
>Channa_argu__KAF3688887.1
MSLSVAGVEDNEVIVRKMLRDVLGGREDPEGFFAMCVSVLGHQETRSQFLYLIHPLSTAHSSLHSILTSIYKD
YFTTLQAEDDEVELAMALSLLEMKDHQLSTPSQESRLMQLEDKTNQSCSFLLNSDSHPQEGSHTLLPEVVKRR
QNTHSAPISPWGNGPAPTTHVDIYNQETVIEREQMMMERDLTQSEKPKHMKNRRQRRKPAGQHIVGLPFSLST
PPPVLLWFRRDLRLCDNPALNASLEVGAPVIPVFIWSSKEEEGPGTTVAMGGASKYWLHQALSCLCASLERIS
SHLVFLKANGEGSRVGSSLNTLKKLIKDTGARTVLANALYEPWLKERDDAVVSALQEDGIECRMYHSYCLRDP
YSVSTEGVGLRGIGSVSHFMSCCRQNPGSALGVTLDPPVSLPTPAHWPQGVSLDTLGLARMPRRKDGTTINWA
ANIHKSWDFSEEGAHVWLKAFLEDGVYRYDKESCRADAPHTSCLSPYLHFGQLSPRWLLWAAKGARGQSPKFQ
RKLYWRDLAYWQLTLFPDLPWESVRPPYKALRWSSSRDHLKAWQQGRTGYPLVDAAMRQLWMTGWMNNYMRHV
VASFLIAYLHLPWQEGYCWFQDTLVDADVAIDAMMWQNGGMCGLDHWNFVMHPVDAAMTCDPYGNYVRKWCPE
LAELPDELIHKPWKCPTSMLRRAGVAFGQTYPQRIITDLEERRSQSLQDVARVRKEFEQFVDKHTGCDLVPLP
PRLVTEALGLSQRYSGVASEGKRFLLPLITRMEFKYQQENPHADAALNPYNALLKGYVSRKRDETIAFLNERD
FTASVMYEAVQRKERLECDQRRMEGLPLSPAPQGRARRTTTAKDKFSIVPGGAGTSLR
>Channa_argu__KAF3689050.1
MVASTIHWFRKGLRLHDNPSLRESIRKADTLRCIYILDPWFAGSSNVGINRWRFLLQCLEDLDASLRKLNSRL
FVIRGQPTDVFPRLFKEWKITHLSYEYDSEPFGKERDAAIQKLASEAGVEVIVRISHTLYDLDKIIELNGGQP
PLTYKHFQAIINHMDAVELSVETITSEVIKTCSTPISEDHDDKFGVPSLEELGFETEGLTTPVWPGGETEGLM
RLERHLERKAWVANFERPRMNANSLLASPTGLSPYLRFGCLSCRLFYFKLTELYRKVKKNSTPPLSLYGQLLW
REFFYTTATNNPCFDKMEGNPVCVRIPWDRNPEALAKWAEGRTGFPWIDAIMTQLKIEGWIHHLARHAVACFL
TRGDLWISWEEGMRVFEELLLDADWSVNAGSWMWLSCSSFFQQFFHCYCPVGFGRRTDPSGDYIRRYLPVLRG
FPSRYIYDPWNAPEEVQKAAKCIIGVHYPKPMVSHAEASRINLERLKQIYQQLSCYRGLGLLATVPSNPNNGA
KGSGVADINTGTNEAPGGSSKDPSIKGGTSQTERKSTQKRCHEETPPESSPKCWRQSK
>Channa_argu__KAF3689281.1
MAVNSVHWFRKGLRLHDNPALQEALSGADTVRCVYILDPWFAGAANVGINRWRFLLEALEDLDSSLKKLNSRL
FVVRGQPTDVFPRLFKEWKVTRLTFEYDPEPYGKERDGAIIKMAQEFGVETVVRNSHTLYNLDRIIEMNNNSP
PLTFKRFQTIVSRLELPRRPLLPVTQQQMDKCLTKIADNHDQLYSIPSLEELGFRTEGLPSTVWRGGESEALD
RLNKHLDKKVWVANFEHSRVNTCSLYASPTGLSPYLRFGCLSCRVLYYNLRELYMKLRKRCSPPLSLFGQLLW
REFFYTAATNNPNFDRMEGNPICVQIPWDQNPEALAKWAEGRTGFPWIDAIMTQLRQEGWIHHLARHAVACFL
TRGDLWISWESGMKVFEELLLDADWSVNAGSWMWLSCSAFFQQFFHCYCPVGFGRRTDPSGDYIRRYIPILKD
YPNRYIYEPWNAPESVQKAANCVVGVDYPKPMVNHAESSRLNIERMKQVYQQLSHYRGLSLLATVPTIQEEAE
PPMTDDSQTSSGPDSPHRFPGDSEAAGFSIAPDSSTAFPSSSSAPCPDLEDSTNSHAPQTLYTSSSSHIQPST
AITSTSAIHFPPTGAPPSVGSTLAQSPLSTSKPSSPSPSCSTLFPFPSLAMTPTQTSCLGQRRKVLARKVRRS
QKQCGRQCGTPSTKETGGKMNEEEREEVGGEERMEEEPEQDERMEEDTCGETTGYQ
>Channa_argu__KAF3699093.1
MSERKRKSTSDGAAGEGPSAKQQKVSSMKKERAGGWLVDLVNQQRSEIKGSMFNQKRLRFINDTKKIKQGSKG
VLYWMSREQRVQDNWALIHAQQLAVQEEVPLHVCVCLVVSKSELSTLRHYSFMLKGLQEVAQECKTLNIQFHL
LHGTAGEILPGFVSDHSLGAVVTDFSPLKAPLQWLEDVKKNLPKEIPFLQVDAHNIVPCWVASPKLEYSARTI
RGKITNLLPEFLTDFPLVERHPYTATRIAKPIDWNKTLGSLQVDRSVGETEWAKPGTKAGMAMLESFIDVRLK
LFGTQRNDPNAAALSQLSPWIRFGHLSAQRVALEVKRNGKGKDNSVGSFIEELVVRRELTDNFCYYNKKYDSV
EGAYMWAQKTLKDHANDKRPYLYTCEQLEKAQTHDKLWNAAQYQMVVDGKMHGFLRMYWAKKILEWTSSPEEA
LSIALYLNDRYELDGQDPNGFVGCMWSICGIHDQGWGERPIFGKIRYMNYKGCTRKFDVAKFEKNYCPKKL
>Channa_argu__KAF3703631.1
MSTCRTIIYLLRNDLRVHDNELFHWAQRNAEYIVPLYCFDPRHYEGTTNYNLPKTGPFRLHFLLESIRDLRNT
LINKGSNLVVRRGKPEHVVAELIQQLESVSTVAFHKEVTSEELSVEKKLKDVCDQMKVKVHTCWGSTLYHIDD
LPFHHISGLPDVYTQFRKAVETQSRVRPVLPTPEQLKPLPPGLEEGAIPTAGDLQQTEPVTDPRSAFPCSGGE
SQALARLKHYFWDTNAVASYKETRNGLIGVDYSTKFAPWLALGCISPRYIYHQIRQYERERTANQSTYWVIFE
LLWRDYFRFVGVKYGNRIFQVKGLQEKSIPWKKDMKLFSAWQEGRTGVPFVDANMRELLMTGFMSNRGRQNVA
SFLTKDLGLDWRMGAEWFEYLLIDYDVCSNYGNWLYSAGVGNDPRENRKFNMIKQGLDYDNNGDYVRQWVPEL
QNIRSADVHTPWTLSTAALSHAQVSLGENYPVPIVIAPEWSRHVNKTSGTGPSPRGKKGPSHTPKQHRDRGID
FYFSRSKNL
>Chanos_chanos_XP_030625976.1_cryptochro
MVVNSVHWFRKGLRLHDNPALEEALNGADTVRCVYILDPWFAGSANVGVNRWRFLLESLEDLDTSLRKLNSRL
FVVRGQPTDVFPKLFKEWNVTRLTFEYDSEPYGKERDGAIIKMAQEFGVETIVRNSHTLYNPDRIIEMNNNSP
PLTFKRFQAIINKLELPRRPLPTITKEQMDRCRTKISDNHDEHYSVPSLEELGFKNQGQSAAVWKGGETEALE
RLSKHLDRKAWVANFERPRINSSSLFASPTGLSPYLRFGCLSCRVFYYNLRELYMKLRKRSSPPLSLFGQLLW
REFFYTAATNNPNFDRMEGNPICVQIPWDHNPEALAKWAEGRTGFPWIDAIMTQLREEGWIHHLARHAVACFL
TRGDLWISWERGMKVFEELLLDADWSVNAGSWMWLSCSAFFQQFFHCYCPVGFGRRIDPSGEYIRRYIPKLKD
YPNRYIYEPWNAPESVQKAANCVVGVDYPKPMINHAESSRLNIERMKQVYQQLSHYRGLSLLASVPTIQEEAE

PPTSDDSSQASSSTSGPVPTPASTPTPDPASTQQQQQQQQQQAAIAAIPAQRKRGRPAELQSTQKSKVQHTSQ
AKADDQSEDKQ
>Chanos_chanos_XP_030646865.1_cryptochro
MVVNTIHWFRKGLRLHDNPSLRDSIQGADSLRCVYILDPWFAGSSNVGINRWRFLLQCLEDLDASLRKLNSRL
FVIRGQPTDVFPRLFKEWKIKRLSYEYDSEPFGKERDAAIKKLANEAGVEVIVRISHTLYDLDKIIELNGGQS
PLTYKRFQTLISRMDPVEMPAETITAEVMGKCATPVNDDHDDKFGVPSLEELGFETEGLSSAVWPGGETEALT
RLERHLERKAWVANFERPRMNANSLLASPTGLSPYLRFGCLSCRLFYFKLTDLYRKVKKNSIPPLSLYGQLLW
REFFYTAATNNPRFDKMEGNPICVQIPWDRNPEALAKWAEGRTGYPWIDAIMTQLRQEGWIHHLARHAIACFL
TRGDLWISWEEGMKVFEELLLDADWSVNAGSWMWLSCSSFFQQFFHCYCPVGFGRRTDPNGDYIRRYLPILRG
FPAKYIYDPWNAPESVQKAAKCIIGVHYPTPMVNHAEASRINIERMKQIYQQLSCYRGLGLLATVPSNSGGDG
TGAGLGTGTGHVSGDSSQENTTTAHQIPTNTRGDLLGGMAACSHGDQQPSTSAQQQQGYSGGSRATSHQETKQ
MQRGRGVSSLKRHSEEPASDSGSKIQRQNNN
>Chanos_chano_XP_030647938.1
MTHSSIHWFRKGLRLHDNPALIAALRDCKQLYPVYILDPWYSNNTNIGVNRWRFLLGALKDLDRSLKKLNSRL
FVVSGKPTEVFLKLFEKWKITRLTFEIDTEPYSQCRDEEVQKLAKDHGVEIIKEVSHTLYNLERIIDENKGKA
PLTYKRFQTIVSAIGPPRKPVPPPTQQDMQGVNTPYSEKDDKKYGIPTLDELCQDADFAGPENFPGGEQEALR
RLDKHLERTGWVCSFEKPQTSPNSLTPTTTVLSPYLSFGCLSARTFWWGLSDVYQGKKHSQPPVSLHGQLLWR
EFFYTAGLGIPNFDKMEGNGVCVQVNWDNNPEYLAAWREARTGFPFIDAIMTQLRQEGWIHHLARHAVACFLT
RGDLWVSWEEGKKVFEELLLDADWALNAANWQWLSASAFFQQYFRVYSPVAFGKKTDKNGDYIKKYLPLLRKF
PAEYIYEPWKAPRSIQERAGCIVGKDYPRPIVQHETISKKNIQRMKEAYAKRSPDPAVSPEKEKGKKRKAGSV
LDMLTKKTKK
>Chanos_cha_XP_030625371.1_cry_DASH
MSSSRTIICLLRNDLRLHDNEVFYWAQRNADHIVPLYCFDPRHYVGTHCFNFPRTGPFRLRFLLESVKDLRAT
LKKRGSTLVVRHGKPEEIVSDLIKQLGTVSAVTFHEEVTSEEERIEKKVKDVCTQSKIRVQTFWGSTLYHRDD
LPFNHIGGLPDVYTQFRKAVEAQGRVRPVIPTPEQVKPVPPGLEEGAIPTYENLGQIEPLTDPRSAFPCQGGE
SEALARLKHYFWDTDAVATYKETRNGLVGVDYSTKFSPWLALGCVSPRYIYDQIKKYEAQRTANQSTYWVIFE
LLWRDYFKFVAAKYGDRLFHLDGLQEKHVPWKTDMKLFNAWKEGRTGVPFVDANMRELALTGFMSNRGRQNVA
SFLTKDLGLDWRMGAEWFEYLLVDHDVCSNYGNWLYSAGIGNDPRENRKFNMVKQGLDYDNNGDYVRQWVPEL
QGIKGGDVHTPWALSSSALSHAQVSLNETYPSPVVMAPEWSRHVNKKPSGAVASGRGRKGPSHTPRQHKDRGI
DFYFSKNKNF
>Chanos_cha_XP_030631243.1_photolyase
MAAEKVDRKRKRETTEMTGGKKQKLVSIHEGKREREAGWLEGEVAALRREAQGCRFDKKRLRFLSEAQKIKQG
SDGVLYWMARDQRVQDNWALIHAQQLAMAENLPLHICFCLVPRYLDSTFRQYAFMLKGLQEVAKECSVLDIQF
HLLRGEPGDILPGFVKDQNFGAVVTDFNPLRVPLQWVENVKKGLQPDIPFMQVDAHNVVPCWEASGKLEYGAR
TIRGKITKLLPKFLTEIPLVDKHPHTSKKTAEPVNWDEIFSSLEVDRSVGEVEWAKPGTAGGMATLESFIDVR
LRHFATQRNNPNSAALSQLSPWVHFGQISAQRVVKEVQRSGKNASQSVASFTEELVVRRELADNFCFYNANYD
NVKGAYEWAQKTLKDHAKDHRPYLYTREQLENAKTHDQLWNAAQRQMVLEGKMHGFLRMYWAKKILEWTSSPE
EGLSIAIYLNDRYSLDGCDPNGYVGCMWSICGIHDQGWKERSIFGKVRYMNYAGCTRKFDVAQFERRYATKKA
>Chanos_cha_XP_030634406.1_cry-1-like
MTHRTIHLFRKGLRLHDNPSLVAALESSVAVYPVFVLDRAFVEGAMATGSLRWRFMLQSLEDLHRSLKARGSR
LHVLQGSYQDVLRKLVKRWAITQLSFDTEVEPHYVQLDGELHILAQELGLTIHSCVAHTLYDIKRVVKANGGE
PPLTYKKFLHILSLLGEPDKPAREITAQDFEKCRTPLDEEHEAEYRVPSLSELGIEVEYEVLWPGGESHALKR
LEKHFESQGWVANFSKPRTIPNSLLPSTTGLSPYLSLGCLSVRTFYHRLSKIYAQSKNHSLPPVSLQGQVLWR
EFFYTVASATPNFTKMEGNPICLQIGWYEDPESLQKWRMAQTGFPWIDAIMTQLRKEGWIHHLARHAVACFLT
RGDLWISWEEGMKVFEEYLLDADYSVNAGNWMWLSASAFFHQYTRIFCPVRFGRRTDPDGQYIRKYLPVLKNL
PSKYIYEPWTAPEEVQLKAGCIIGKDYPLPMVDHVEVSQRNLTLMKQVRCEQESTAELTRDVADDPMEIGLKR
DFQDDGLADGTGEHRVNGTTKRFSSEKDHQAQPCSWTTETPRLTEISGEVM
>Chanos_cha_XP_030646307.1_uncharacte
MSVAKDAKAVSQVRQMLRDLLLGREDPEGFFGLCLSVLGDNDTRTLFMDLIKPISSGHVQLHSQLSAIYHEYF
SKGEDVEVELALALSLQEVSQEVKEQRSAKPPQVKSYAELAGGRWAWSVNGAGVQDRGSRGLEKQPTVTKWSL
TDAEVQGPTTALTKGRNAELHLAEEGDDFIKEHDAGQSQKLKRSRRRRQRKNQELSLLQSPSGSKPILLWLRR
DLRMWDNPALIGCLEMGAPIIPVFLWNAAEEEGPGVTVAAGGASKYWLHQVLVCFNQSLEQRGSHLITQRAEP
SSLEALLRLVAETGAGTVVATALYEPWLKERDEVVFRALERKGIHCQLFHSYCLRDPYSITTQGVGLRGIGSV
SHFMSCCQQNPGTGLGTPLDAPGALPVPSVWPQGCPLTALGLARMPRRKDGTVVDWAANIREAWDFSEDGAQA
RLDAFLQDGVYRYEKESCRADAPNTSSLSPYLHFGQLSARWLLWDARGARCRPPKFQRKLAWRDLAYWQLCLF
PDLPWESLRPPYKALRWSSERAHLRAWQRGGTGYPLVDAAMRQLWLSGWMNNYMRHVVASFLIAYLHLPWQEG
YRWFQDTLVDADVAIDAMMWQNGGMCGLDHWNFVMHPVDAALTCDPRGDYVRKWCPELAALPDDLIHKPWHCP
ASMLRRAGVVLGGNYPERIVVDLEELRAQSLRDAAEVRRRFGEFVDCHSGCDLVPLPARLVQEALGSRAELVR
SGTQFLLPVITRMEFKHQTSDPDGTDNPYSAVLKGYVSRKRDETIAFLNERDFTASVMCESAQRRERQDRHQR
LLEGLPKAPPTTPRGRARRTPARDPYSTVPGGVAVPRR

>Chara_bra_CBR_g37632_GBG83833.1
MEAAGGLGSSLKILVLGTTGVGKSLVIDTLLGRKASKSSRRGRFQLQEFQQAVSGRQLHVLEASGMSKPGDLE
EVRRQLIKRQLAVDVIIYVDRAGVPDSSSLNLLQLAGDKFGGGMWGVTLFALTYSGALEPEVVVPDVDQLIAD
WTAGMERALRGEERKHVLSLPEVVSLRVGGGNDLFEVDRASIFGAVERVVRNGSVFVPDSQDRRASRRSSGLI
ELGNLMDNNVVRAERILLSGRRQESARPQPKPALREDGGAALHGGKKKERDVCVEESGGCVEQQNAGRPLEEH
RMLAARPQIAAAASLSLALQAATMAVTPLVSKSLAELSAGGRGRQSSSVSPATANKQPSIALYRQSRQEEEGG
ALAQSAGEEVSKTRGASALMASLAPVQAPAMVLGVPSGAQSKVLPAPAKKASSGAALGAAAAGLNLSVTAGMT
SGSSTDRSRAGRLSAFPDFQRLPSTGRGSTDPKGAPGARKASLVWFRNDLRVHDNEALLKANNESLSILCVYC
FDPRDYGKSSSGFDKTGPYRAKFLLECVADLRKNLRAKGNDLVVRIGKPEEVIGKLAREVGAEAVYAHQEVTH
EELEMEERVAATLKEEGIETKYSWGSTLYHLEDLPFKLEDMPSNYGGFREKVQNLRVRATAQAPDSLRPLPAR
GGVKVGEIPTLQELGLNVGSTGNSRQDRKGAGAQFELIGGETEAMSRLKTFVAEANGRRKASANGEKLYGANF
SCKISPWLAMGCLSPRKMYEDLKNTKGAAARGISSDGAWSTDESNGLNWLVFELLWRDFFRFITKKYGSHRKD
QELSPAMASSGAALACA
>Chara_bra_GBG67780.1_CBR_g905
MANQGNDVTPTSVEDLNRVRESVERCYDEGVCPDDAYLGEVVEDREGKRFEVNKMVDEVKEQWLRERSVVVTF
QGEARNLQRSVKEDLIRAYEDGWTAKKLFQPGLRRGRVKFEGANVASYVAKAKEIADWLVQQKEMSIKLANKV
YSVSFKPRLSRQELQDARIQEAESKFWIMALRVPLDAYYFLRSAVPGVSEWVSDHMAKWEAAKPHFGSTAEWL
DGGIAITSGILDAVSRILARERNIKEADFKRRVKEAEERMEGHPISVMVWAAEREKRLEEWDNFQVEKQRWWT
ELLQDKGIETHDKMTKETFQKLLPSRSQHQMVELKHPFNESAPTACSSTGMLQYARLYYEDVLSTRRPQDDVD
SDLSTSSDMWDNTGVRLSTTARLDLDRPLTLEEVTQMLKTMARGKSPGVDGLTVEFYITNWGAFGESLVKLYN
KVTTGGKLGKGMTHGVISVLFKKGDKARGRVIARRRQAEVITADRRRGCAEISSSQAVTMAAGALATRHVTSR
ASVVAASAKGAALDTNRRRGEGTSIVWFRNDLRVHDNEALLRAWQDSDAILPVFCFDPRLFKKTFSFGLPKTG
EIRAQFLLESVEDLQRTLESQGSVLVVRHGKPEDIIPGIVRASGAHTVYGQKETCSEELYVERKMSEALKSLQ
TATSGKRNERGVKLQLFWGSTLYHLDDLPFRSSHVPDVYTQFRKSVESSCRVRLSLKMPTSLGPKIAADVIDA
IGGKGMLPSFQELGYSQKRQLDPRAVLQFRGGETVALARLEDYFWKKDKLRVYKETRNGMLGADYSTKFSPWL
AFGCLSPRFIHDELIWRDYFRFLTIKYGNNIFKLGGPRNVAGKQLWNRSTSLFDAWKEGRTGYPLVDANMREL
SSTGFMSNRGRQIVCSFFVRDMGMDWRMGAEWFETCLLDYDPASNYGNWTYGAGVGNDPREDRYFSIPKQSAT
YDPDGEYVAHWIPELAQVPMPKRHAPYRLSTEEQLQYGVRIGEGGDYPEPIVPLKFGDGGGSRPVNGPRPRSA
PGRGNFRARPAGRSMSSYLQRGND
>Chara_bra_GBG70219.1_CBR_g6351
MDSSGGGPSCTVVWLRRDLRLEDNPALIWAARAGAVVPVFIWSPEEEGQFYPGRVSRWWIKQSLKHLNNALQV
LGSRLIMRRSRDSLSALLEIVEALGAKQVFYNHLYDPVSLVRDHRVKQGLVQHGIVVQSFNADLLYEPWEVYD
EEGQAFTTFQKFWDKCILMAFEPDPPMLPPKQLYGPGEDLGGPTEEQLGLEDQAEMASNLLLSRAWTPGWGHA
DKALDSFIRGPLIEYADSRAKADSATTSLLSPHMHFGEISVRKIFHEVQKMHFKWKREGVMQGVSSVELFLRS
LGFREYSRYLSFNFPFTHERSLLANLKWFPWRVDERMFKAWRQGRTGYPLVDAGMRELWATGWLHNRIRVVVS
SFFVKVLQLPWRWGMKYFWDTLLDADLECDVLGWQYISGSLPDGHELDRMENPEVEGMKYDPDGEYVRRWLPE
LVRLPTEWIHHPWDAPPEVLRAAGIELGVNYQLPIVDMGEARQKLQEALSEMWARENDNNCMRAAGNTEEGYG
ETMIVVEGSGRRATENMVVERVIVARQKAQQKSSSMFKDQLVPDMVTESTKKAAASILAPAAYPASEVETRSE
QEGQEAQPMLQEDAASSGTGLRLEDTSGHAAGVEVEVQVREAAGGAAAQHGSQGREDVMSTAESYTSPSSDRG
GMATVPVWSQSGSSSRSPSLAVDGRVPTVPVVPDVRKGIGSGGRSRRRMQPRQGVGRVGPLKPQGDEDKPLAK
RKRKKQTDPANAD
>Chara_bra_GBG74222.1_CBR_g17934
MIPACSPRSSEWRRLCGTDQTVGIAISVGENYRQLLPLRLGYQDKWRLHVFPQRFSRWQKVSSCCQIGHVAAS
LSHRGRRSGGGGGGGGGGGDRSWRNQRSTRLLAPHPVFCCCRSFNYTCPINEVFHELISYNGTSEWNSTKPRL
NRRCRLPDRCMPRHRCMPRDRCMQRTRSVSLWQSYCVVQQLPLIGGLCIGRPLSDTWQRLRGVPLITSSSASS
LFFSLTSSPSPRLIEAREAMKRSATEKGGGGEGSGRGGEENEAPSLKKTKLSVAEETSKANTATPSGKNATSS
GKNATSSAKNATSSAKNATSASRKSAGSSPAKKPSSAAATSAAKTAAAADVASSDVAAGATNQTPTSGRDEGR
TKTKEGGGGGGRREVDDVRVNPKRVRVLKPKQKKPDVDKVVGKAVDVNTGPVIYWMSRDQRSCDNWALLHAWE
EAREREVPLAVVFNLVPKFLGAGARHFGFLLRGLKVVAGKLEERKIPFFLFRGEAMDTIPAFVEECHASLLVM
DFSPLRIGRKWRDTICEKVSIPVHEVDAHNVVPVWVASEKLEYGARTIRTKIHKNLPEFLVPFPALPDAPRPW
SSPDKGGKLKLPQVRPIDWDKLIEQALRIGADVPEVDWCLPGEDNAIEWLMGEKSGFVTKRLAKYDSMRNDPS
KHGALSGISPWLHFGQISAQRCALEAKKARKQYPQAVDAFLEELIVRRELADNYCYYQPKYDSLAGAWDWARY
SLQQHAADKREYIYTKEQFEKGKTHDPLWNAAQLEMVHYGKMHGFMRMYWAKKILEWTSSPEEALSVAIYLND
KYELDGRDPNGYVGCMWSIAGIHDQGWAERPVFGKIRYMNFNGCKRKFDVEAYVQFVNKLTSNVLKKKREAAA
GGAPASAKGSDSAVKTAPSKGSGLAAKVAAKG
>Chara_braunii_GBG80215.1
MLKVGLEGGAIDEDVIEVDDNTDFEEVAEDVIHGGLKCGRGIGGGDEGGPLEEGVRSEIGVMAVGHFGVPPAS
GTVEESAAGDGEPLDDKDVVEGEVGFVGCVTMVGVVVDVGGGVDDVLAERTVDVDGKGSGADVVWGEVVVTTV
MAGLFPSIATTRLEMVEMEVFMSVMEVFTREMDDWRDCMIWSRAAKSGAAGVAAGGFSAGPSVQVLVQSVYSQ
PPLQVAVTGQPTVSQPAQPQGTQPQQQPVQQPAPQGPQPGVAQVPGQSQWVPKTVIAAPKPFTGDKRGEHLDM

WLRAVPVYVKCTLTLPHEEVLVAASYLEGSAARWLSGLVQLQGYVHDFRAWAAHQKLEDFLKMVEDRWHDPQE
AQKATDVILTLHTRQFKSGREATDAVERLICVPGVRYDPQVLLTSYPRCFPIPLRNQLAGEANINMHHFPSFS
KKALDLEAIGHGQTPTTDGRKKSLPPNWKAKGRIMFVDNDGSTIELDNEFQAGVGSEAGSLEASGGGVVVVVA
QKGVVEREVVHTIEIILGSSIPNGRIYQTSPVELDELRRQLKELVEKGWIRLSVSPYGSPVLFVPKKEGTLRM
CIDYRGLNAITVKNREPLPRIGDLLDRVQGCRYLSKIDLKSGYHQIAIRPEDQHKTTFQTRYGLYEFVVMPFG
LCNAPGTFQHTMNQIFHDYLDKFVIVYLDDILIHDYLDKFVIVYLDDILIFSRTVEEHVVHLDKVLSLLRQHK
FKINGEXCEFGRTRVLYLGHEISVEGLKPNDAKVASIRDWPRPRSVIEMRSFLGMTGYYCNFVQNYSIVAAPL
PDLTRLDTPWEWTDRCEAAFKQLKHALTHHEVLKLSDLDKPCVVTTDASQYGIGAVLAQQEGKKLRLVEYMSK
TMPSQKLAKSTYGKELYAIYKALTHWRHYLHGSAVADCSPTLAAQAKQLEERINHVVASLGDISKFAGASIVS
NQLQMLSDRVQQRPTAVAKEWKMSNFKIEKFDDYHKTGPLQWWMTFNVEADVHHTPPLRRLDALYLQLIGGAQ
AFMTHMVATLECTIATLHTKITWEEFEKKWKTRFMVNNDKRHALNKIFRIFQGQQTSREWLTEWQRLVATPEL
NLPFDSIRAEFFAQSCHTLTAALGSELQYQTFDEMISKARELIQVPLPSTDGGVAVVDLRSYLVKIDREHVTQ
RYVDIDTPFLYIHIQIGKAICSALIDCGASRNDFSQDFMACAGLGPRVRRRSQPTHVLLANGHTQKSIDRWEF
FYTNGYGNVNFDRMVGNPICKQIPWHQNDELFVAWRDAKTGYPWIDAVMVQLQKWGWMHHLARHCVACFLTRG
DLFLHWEKGRDVFDRLLIDSDWSINNGNWLWLSASAFFSQYHRIYSPITFPKKYDPSGKFVRHFLPVLKDMPS
QYIYEPWTSPIDVQKKAKCIIGKDYPRPVVDHAEASKLCRQRMAAAYQANRDVTDPGVSGNRLPKPSSNARKA
SSSTATKRKRE
>Charadrius_voci__KGL88511.1
RFLLQSLEDLDNSLRKLNSRLFVVRGQPTDVFPRLFKEWGVTRLTFEYDSEPFGKERDAAIVKLAKEAGVEVV
IENSHTLYDLDRIIELNGHKPPLTYKRFQAIISRMELPKKPVSTIMSQQMETCKVDIQENHDDVYGVPSLEEL
GFPTDGLAPAVWQGGETEALARLDKHLERKAWVANYERPRMNANSLLASPTGLSPYLRFGCLSCRLFYYRLWE
LYKKVKRNSTPPLSLYGQLLWREFFYTAATNNPKFDRMEGNPICIQIPWDRNPEALAKWAEGKTGFPWIDAIM
TQLRQEGWIHHLARHAVACFLTRGDLWISWESGVRVFDELLLDADFSVNAGSWMWLSCSAFFQQFFHCYCPVG
FGRRTDPSGDYVKRYLPKLKGFPSRYIYEPWNAPESVQKAAKCIIGVDYPKPMVNHAETSRLNIERMKQIYQQ
LSRYRGLCLLASVPSCVEDLSGPVADSASGQGCSTSTAMRLSQADQASPKRKHEGAEELCAEELYKRAKVTGL
PAPEIPGKS
>Charadrius_voci__XP_009879608.1
MQHRTIHLFRKGLRLHDNPTLLAALESSEAVYPIYILDRKFLTSVMHLGALRWHFLLQSLEDLQKNLCQLGSC
LLVIQGEYERVLRDHIQKWNITQVTLDAEMEPFYKEMEANIRRLGEELGFEVLSLVGHSLYDTKRILDLNGGT
PPLTYKRFLHILSLLGDPEVPVRNLTAEDFQRCRPPDPGLAECYRVPLPVDLEIPLESLSPWRGGETEGLQRL
EQHLTDQGWVTSFTKPRTIPNSLLPSTTGLSPYFSTGCLSVRTFFYRLSNIYAQAKHHSLPPVSLQGQLLWRE
FFYTVASATPNFTKMAGNPICLQINWYEDAERLHKWKTAQTGFPWIDAIMTQLRQEGWIHHLARHAVACFLTR
GDLWISWEEGMKVFEELLLDADYSINAGNWMWLSASAFFHQYTRIFCPVRFGKRTDPEGQYIRKYLPVLKNFP
SKYIYEPWTASRXXXXXEEEQKQAGCIIGRDYPFPMVNHKEASDRNLQLMKRVREEQYGTAQLTRDDTDDPME
MKVKRRHSEENVSKGKVARMTEKKEVPSGISPQEPKNGERGEPEAS
>Charadrius_voci__XP_009880290.1
MLLEDRFLLQCLEDLDANLRKLNSRLFVIRGQPADVFPRLFKEWNIAKLSIEYDSEPFGKERDAAIKKLASEA
GVEVIVRISHTLYDLDKIIELNGGQPPLTYKRFQTLISRMEPLEMPVETITPEVMEKCTTPVSDDHDEKYGVP
SLEELGFDTDGLPSAVWPGGETEALTRLERHLERKAWVANFERPRMNANSLLASPTGLSPYLRFGCLSCRLFY
FKLTDLYKKVKKNSSPPLSLYGQLLWREFFYTAATNNPRFDKMEGNPICVQIPWDKNPEALAKWAEGRTGFPW
IDAIMTQLRQEGWIHHLARHAVACFLTRGDLWISWEEGMKVFEELLLDADWSVNAGSWMWLSCSSFFQQFFHC
YCPVGFGRRTDPNGDYIRRYLPVLRGFPAKYIYDPWNAPESVQKAAKCIIGVNYPKPMVNHAEASRLNIERMK
QIYQQLSRYRGLGLLATVPSNPNGNGNGGLMGYSPGESISGCGSTGGAQLGTGDGHTVVQPCALGDSHTGASG
IQQQGYCQASSILHYAHGDNQQSHLLQAGRTALGTGISAGKRPNPEEETQSIGPKVQRQSTN
>Charadrius_voci__XP_009881711.1
MRRGQGKRKAEAAELPRVSRRREEEEEALQEARRRAAPSVREFKYNKKRVRLVAQGSDLKDDARCILYWMSRD
QRVQDNWAFLYAQRLALKQELPLHICFCLVPKFLDATIRHYGFMLRGLQEVAKECGELNIPFHLLLGYAKDVL
PEFVVEHGVGGLVTDFCPLRLPRQWVEDVRERLPEDVPFAQVDAHNIVPCWVASPKQEYSARTIRGKIHAQLP
EFLTEFPPVIRHPYPPSCPAEPIAWEACYSSLQVDRTVKEVEWATPGTAAGLAVLQSFIAERLKSFGSHRNDP
NKAALSNLSPWFHFGQVSTQRAILEVQKHRGKYKESVDAFVEEAVMRRELAENFCYYNENYDSVQGAYDWAQT
TLKLHAKDKRPFLYKLQELEQGTTHDPLWNAAQLQMVREGKMHGFLRMYWAKKILEWTRSPEEALQFAIYLND
RYELDGRDPNGYVGCLWSICGIHDQGWAERAIFGKIRYMNYAGCKRKFDVGQFERRYAPRALSQ
>Charadrius_voci__XP_009888783.1
MIIALLRCGLRAHDNQVLHWAQSNADFVIPLYCFDPRHYLGTHLYGFPKTGPHRLRFLLESVKDLRETLKKKG
STLVVRKGKPEDVVCDLITQLGSVSAVAFHEEATQEELDVEKGLCQVCSQHGVKIQTFWASTLYHRDDLPFRP
IARLPDVYTHFRKVVESGVKVRPTLRMAEQLKPLAPGVEEGCIPTMEDLGQNDPVTDPRTAFPCSGGETQALM
RLQYYFWDTNLVASYKETRNGLVGMDYSTKFAPWLALGCISPRYIYEQIQKYEKERAANQSTYWVLFELLWRD
YFRFVALKYGKRIFSLRGLQSKEVPWKKDLQLFDCWKDGKTGVPFVDANMRELAATGFMSNRGRQNVASFLTK
DLGLDWRMGAEWFEYLLVDYDVCSNYGNWLYSAGIGNDPRDNRKFNMIKQGLDYDGSGDYVRLWVPELQGIKG

ADIHTPWALSSAALSQAGVTLGETYPQPLVTAPEWSRHINQRPVSTGKVRVGRGPAHTPMQHKDRGIDFYFSR
KKDVR
>Charadrius_voci__XP_009889134.1
MQHSSIHWFRKGLRLHDNPALLAAATDCCRLHPLFILDPSSNRAGANARRFLLDALLDLDGSLREMGSRLFVV
RGCPQEVFPCLFRAWGTTRLTFEVDTEPPARQRDTAVAELAAQHGVEVIQKVSHTLYDTERVLALNGGKAPLT
YKHLQSLLTSLGPPEKPAPALTQEHLQGCHTPCQTSHDTDYGVPTLEELGQNPAEMGPHLYPGGETAALARLD
AHMERTAWVRGFKKPETEPTSLSPSTTVLSPYIKFGCLSVRTFWWRLDEVYQGREHSQPPVSLHGQLLWREFF
YTAGASIPNFDRMVGNPICLQVDWDDNPQYLRAWREGQTGYPFIDAIMTQLRSEGWIHHLARHAVACFLTRGD
LWVSWEEGLKVFEELLLDADWSLNAGNWLWLGSSAFFHRYFHVYSPVAFGKKTDRDGAYIRKYLPILKDFPAE
YIYEPWKAPRAVQERAGCLVGTHYPRPIVEHRAASERNLGRMKAARAQKGTKREPPAGPSVAESRRKKSKAEQ
H
>Chauna_torq__NXK44152.1
RFLLQSLEDLDNSLRKLNSRLFVVRGQPTDVFPRLFKEWGVTRLTFEYDSEPFGKERDAAIVKLAKEAGVEVV
IENSHTLYDLDRIIELNGNKPPLTYKRFQAIISRMELPKKPVSSVTSQQMETCKVDIQENHDDVFGVPSLEEL
GFPTDGLAPAVWQGGETEALARLDKHLERKAWVANYERPRMNANSLLASPTGLSPYLRFGCLSCRLFYYRLWE
LYKKVKRNSTPPLSLYGQLLWREFFYTAATNNPKFDRMEGNPICIQIPWDKNPEALAKWAEGKTGFPWIDAIM
TQLRQEGWIHHLARHAVACFLTRGDLWISWESGVRVFDELLLDADFSVNAGSWMWLSCSAFFQQFFHCYCPVG
FGRRTDPSGDYVKRYLPKLKGFPSRYIYEPWNAPESVQKAAKCIIGVDYPKPMVNHAETSRLNIERMKQIYQQ
LSRYRGLCLLASVPSCVEDLSGPAADSASGQGSSTSTAMRLPQSDQASPKRKHEGAEELCTEELYKRAKVTGL
PAPEIPGKSL
>Chauna_torq__NXK48124.1
RFLLQCLEDLDANLRKLNSRLFVIRGQPADVFPRLFKEWNIAKLSIEYDSEPFGKERDAAIKKLASEAGVEVI
VRISHTLYDLDKIIELNGGQPPLTYKRFQTLISRMEPLEMPVETITPEVMEKCTTPVSDDHDEKYGVPSLEEL
GFDTDGLPSAVWPGGETEALTRLERHLERKAWVANFERPRMNANSLLASPTGLSPYLRFGCLSCRLFYFKLTD
LYKKVKKNSSPPLSLYGQLLWREFFYTAATNNPRFDKMEGNPICVQIPWDKNPEALAKWAEGRTGFPWIDAIM
TQLRQEGWIHHLARHAVACFLTRGDLWISWEEGMKVFEELLLDADWSVNAGSWMWLSCSSFFQQFFHCYCPVG
FGRRTDPNGDYIRRYLPVLRGFPAKYIYDPWNAPESIQKAAKCIIGVNYPKPMVNHAEASRLNIERMKQIYQQ
LSRYRGLGLLATVPSNPNGNGNGGLMGFSPGESISGCGSTGGAQLGTGDGQTVGVQTCALGDSHTGGSGVQQQ
GSYCQASSILHYAHGDNQQSHLLQAGRTSLGTGISAGKRPNPEEETQSVGPKVQRQSTN
>Chauna_torq__NXK48987.1
KVDYDVCSNYGNWLYSAGIGNDPRDNRKFNMIKQGLDYDGNGDYIRLWVPELQGIAGADIHTPWALNSATLSQ
AGVTLGETYPPPVVTAPEWSRHINQRPGRSPHPSGRRGPARAPMQREHRGIDFYFSRNKDM
>Chauna_torq__NXK52963.1
DNWAFLYAQRLALKQELPLHVCFCLVPKFLDATIRHYSFMLKGLQEVGKECTELNIPFHLLLGYAKDVLPTFV
VEHGVGGLVTDFCPLRVPRQWVEDVRERLPEDVPFVQVDAHNIVPCWVTSAKQEYSARTIRGKIHSQLPEFLT
EFPPIIRHPHPPSCPPEAIAWDACYSSLQVDRTVTEVEWATPGTAAGLAVLQSFIDERLKSFSSQRNDPNKAA
LSNLSPWFHFGQVSTQRAILEVQKHRHMYKESVDAFVEEAVVRRELADNFCYYNENYDSVRGAYDWAQTTLEL
HAKDKRPFLYELQQLEQATTHDPLWNAAQLQMVREGKMHGFLRMYWAKKILEWTRSPEEALRFAIYLNDRYEL
DGRDPNGYAGKLRERSWGCLWSICGIHDQGWKERDVFGKIRYMNYAGCKRKFDVDQFERRYARRK
>Chauna_torq__NXK56767.1
MPHRTIHLFRKGLRLHDNPTLLAALESSEVVYPVYILDRKFMTSVMHIGTLRWHFLLQSLEDLQKKLCQLGSC
LLVIQGEYESIVRDHVQKWNITQVTLDAEMEPFYKEMEANIRCLGEELGFEVLSLVGHSLYDTKRILDLNDGT
PPLTYKRFLHILSLLGDPEVPVRNLTAEDFQRCRPPDLGLAGCYRVPLPADLKIPPESISPWRGGETEGLQRL
EQHLTDQGWVASFAKPRTVPNSLLPSTTGLSPYFSMGCLSVRTFFYRLSNIYAQAKHHSLPPVSLQGQLLWRE
FFYTVASATPNFTKMAGNPICLQIRWYEDAERLHKWKTAQTGFPWIDAIMTQLRQEGWIHHLARHAAACFLTR
GDLWISWEEGMKVFEELLLDADYSINAGNWMWLSASAFFHQYTRIFCPVRFGKRTDPEGQYIRKYLPILKNFP
SKYIYEPWTASEEEQKQAGCIIGQDYPFPMVDHKEASDHNLQLMKQVREEQYRTAQLTRDDTDDPMEMKLKRD
YSEENVTKAKAARMME
>Cheilinus_undu__XP_041648513.1
MAHTCIHWFRKGLRLHDNPALVAALRDCKELFPVFILDPHLHNKTCYGINRWRFLIGALKDLDCSLRKLNSRL
FVLRGNPEDVFPKLFNQWKVTKLTYEYDTEPYSLSRDKTVTALAKDHGVEVIYKISHTLYDIERIIEENNGKT
PLTYVRMQAIMKGLGAPKRPIPVPTMEDMKDVKTPHFEKHERDYGLPTLEELGHNTTGLFEEKFPGGEQEALR
RLDEHMQKTGWVCSFEKPQTSPNSLTPSTTVLSPYVTFGCLSARTFWWRLTEVYQGKKHSDPPVSLHGQLLWR
EFFYTASVGIPNFNKMEGNSVCTQVDWDTNPEYLAAWREARTGFPFIDAIMTQLRQEGWIHHLARHAVACFLT
RGDLWINWEEGQKVFEELLLDGDWALNAGNWQWLSASTFFHQFFRVYSPVAFGKKTDKNGDYIKKYLPLLKKF
PAAYIYEPWKAPRSVQEAAGCIVGKDYPRPIVEHEVISKKNIQRMKSAYAKRSANPESPSKKQGVKRKQPSIA
DVMKKKEKKK
>Cheilinus_undu__XP_041651051.1
MVVNSVHWFRKGLRLHDNPALQEALNGADTVRCVYILDPWFAGAANVGINRWRFLLEALEDLDNSLRKLNSRL
FVVRGQPTDVFPRLLKEWKVTRLTFEYDPEPYGKERDGAIIKMAQEFGVETIVRNSHTLYNLDRIIEMNNNSP

PLTFKRFQTIVSRLELPRRPLPPINQQQMDKCPTKISDNHDQLYSIPSLEELGFRTEGLSPAIWRGGESEALD
RLNKHLDKKVWVANFEHPRPNMCSLYASPTGLSPYLRFGCLSCRVLYYNLRELYMKLRKRCSPPLSLFGQLLW
REFFYTAATNNPNFDRMDGNPICVQIPWDQNPEALAKWAEGRTGFPWIDAIMTQLRQEGWIHHLARHAVACFL
TRGDLWLSWESGMKVFEELLLDADWSVNAGSWMWLSCSAFFQQFFHCYCPVGFGRRTDPSGDYIRHYIPILKD
YPNRYIYEPWNAPESVQKAANCVVGVDYPKPIINHAEVSRLNIERMKQVYQQLSHYRGLSLLASVPTIQEEAE
PPMTDESQTSSGPDSPPRGPADSEAAGCSTAPDSSTVCASSSYTPMHEDTADFKNQTPCTSSNTNPSAAITST
STFMSTSLPPSTATPPSPAQSPLSRSKPSTPSSSCPSLPPSPSPATTPNQTPSSLGPKRKGPGRKVRRSQRQR
GRQSCTAAAVREGERRSEEEREEAGEERMEEDVEQGEEKMEEETCGETARHQQ
>Cheilinus_undu__XP_041651086.1
MVVNTIHWFRKGLRLHDNPSLRDSIRGADTLRCIYILDPWFAGSSNVGINRWRFLLQCLEDLDASLRKLNSRL
FVIRGQPTDVFPRLFKEWKITRLSYEYDSEPFGKERDAAIQKLASEAKVEVIVRISHTLYDLARIIELNNGQP
PLTYKRFQALITRMDAVELPAETITQQTIKNCATPIRDDHDDKFGVPTLEELGFETEGLSTAVWPGGETEALI
RLERHLERTAWVANFERPRMNANSLLASPTGVTPYLRFGCLSCRLFYHKLTDLYRKVKRNDAPPLLLYSQLLW
REFFYTTATNNPCFDKMEGNPVCVQIPWDRNPEALAKWAEGQTGFPWIDAIMTQLRQEGWIHPLARRSVVCFL
TRGDLWVSWEEGMKVFEELLIDADWSVNAGSWMWLSCSSFFQQFFNCCCPVGFGRQMDPNGDYIRRYLPILRG
FPARYIYDPWNAPEEVQKVEKCIIGVHYPKPMVNHAEASRINIERMKQIYQQLSCYRGLGLLASVPANSSSNM
RGVGAGTGPGSGRTSEKNPTQEGTRQTERGQTTLKRRREEAAPESSCKTMKQSK
>Cheilinus_undu__XP_041651995.1
MPPSAGSDEDSKALVRKALREVLVGREDPEGFFAMCVSALGHQETRLHFLSIIQPLSTANISLHSILTSIYRE
YFSKTEDDELEVALALSLLEMRDHQLSAPSQESQLQQHLERHIQKSSSQQISRSQSWGNSHTQHTNVAESREN
TNLPKTGDLERVNQPKINLKGNPGKSLTMCAPVTSNSLREHGTAEGGGGMMHEGDLDQPEKRKHSKNRRQRRK
GACQRIVGLPTSPSARPPVLLWFRRDLRLCDNPALNGSLEVGAPVIPVFIWSPKEEEGPGITVAMGGACKYWL
HQALSCFCSSLERIGSHLVFLQAKGESKEVGSSLCCLKELLKETGARTVLANALYEPWLKERDDEVVSALQKD
GVEFKMFHSYCLRDPYSVSTEGVGLRGIGSVSHFMSCCKQNPGSALGVPLDPPVALPSPSHWPQGVSLDELGL
ATMPRRKDGTTIDWAANIRKSWDFSEEGAHARLEGFLHDGVYRYEKESGRADAPNTSCLSPYIHFGQLSPRWL
MWDAKGARCRPPKFQRKLAWRDLAYWQLTLFPDLPWESLRPPYKVLRWSNNHSHLKAWQRGRTGYPLVDAAMR
QLWLTGWMNNYMRHVVASFLIAYLYLPWQEGYRWFQDTLVDADVAIDAMMWQNGGMCGLDHWNFVMHPVDAAM
TCDPCGSYVRKWCPELSDLPDEVIHKPWKCPVSMLRRAGVVFGQTYPERIVTDLEERRNHSLQDVALVRREFG
QYVDKRSGCDLVPLPPRLVSEALGLSHRDGGVVKEGKQFLLPVITRMEFKHQLEDPDADAASNPYNAVLKGYV
SRKRDETIAFLNERDFTASVMYEGAQRKERLESDYRRMEGLPRLPAPRGKARRTTTAKDRFSTVPGGAVTSLK
>Cheilinus_undu__XP_041659813.1
MLKCILIRASSPFARRYFLRLLHHSTERSIPIFFHQTKSFTSQTSMSDKKRKAPAAAKEPSAKQQKLTPMKVE
KAGGWLQDHLRQQRTDNKDMKFNKKRLRFISETEKIKQGSEGVLYWMLRDQRVQDNWALIHAQRLAVKENLPL
HICFCLVVPKSELSTLRHFSFMLNGLREVAKKCKNLDIQFHLLHGSAGEVLPGFVSDHGLGAVVTDFSPLREQ
LQWLEDVKKKLPKDIPLIQVDTHNIVPCWVASPKLEYAARTIRGKITKLLPEFLTDFPLVEKHPHTAARTAKP
IDWDKTLASLKVDRTVGETEWAKPGTKAGVAMLESFIDVRLKMFDTQRNDPNAPALSQLSPWIRFGHLSAQRV
ALQVQHSGRKAGVTVTSFIEELVVRRELTDNFCFYNKKYDCVEGAYDWAQKTLKDHAKDKRPYLYTREQLEKA
KTHDKLWNAAQYQMVSEGKMHGFLRMYWAKKILEWTSSPEEALSIALYLNDRYELDGQDPNGFVGCMWSICGI
HDQGWAERPIFGKIRYMNYKGCLRKFDVARFERKYCPKDL
>Cheilinus_undu__XP_041669776.1
MSASRTVICLLRNDLRLHDNELFHWAQRNAEYIVPLYCFDPRHYVGTYTYNLPKTGPFRLRFLLEGIQDLRNT
LVNKGSNLVVRRGKPEEVVADLIKQLGSVSTVAFQEEVTSEELNVEKRVKDVCAQMKVKVHTCWGSTLYHRDD
LPFPHMSRLPDVYTQFRKAVETQSRVRPLFPTPEQLKPLPQELEEGTIPTAEELQQTEPVNDPRSAFPCSGGE
SQALARLKHYFWDTNAVAAYKETRNGLIGVDYSTKFAPWLAMGCISPRYIYHQIQQYERERTANQSTYWVIFE
LLWRDYFKFVAAKFGNRLFQVEGLQDKSVPWKKDMKLFNAWKEGRTGVPFVDANMRELAMTGFMSNRGRQNVA
SFLTKDLGLDWRMGAEWFEYLLIDYDVCSNYGNWLYSAGIGNDPRENRKFNMIKQGLDYDNNGDYVRQWVPEL
QGIRGADIHTPWTLSSAALSHAQVSLGETYPTPIVMAPEWSRHVNKKPSGPGPSPRGKKGPSHTPKQHRDRGI
DFYFSRSKNL
>Chelmon_rost__XP_041794067.1
MVVNSVHWFRKGLRLHDNPALQEALNGADTVRCVYILDPWFAGAANVGINRWRFLLEALEDLDSSLKKLNSRL
FVVRGQPTDVFPRLLKEWKVTRLTFEYDPEPYGKERDGAIIKMAQEFGVETIVRNSHTLYNLDRIIEMNNNSP
PLTFKRFQTIVSRLELPRRPLPPVTQQQMDKCHTKIADNHDQLYSIPSLEELGFRTEGLPPAIWQGGESEALD
RLNKHLDKKVWVANFEHPRVNMCSLYASPTGLSPYLRFGCLSCRVLYYNLRELYMKLRKRCSPPLSLFGQLLW
REFFYTAATNNPNFDRMEGNPICVQIPWDQNPEALAKWAEGRTGFPWIDAIMTQLRQEGWIHHLARHAVACFL
TRGDLWISWESGMKVFEELLLDADWSVNAGSWMWLSCSAFFQQFFHCYCPVGFGRRTDPSGDYIRRYIPILKD
YPNRYIYEPWNAPESVQRAANCVVGVDYPKPMINHAEGSRLNIERMKQVYQQLSHYRGLSLLASVPTIQEEAE
PPMTDESQTSSGPDSPPRGPADIEAAGCSTAPDSSTVCASSTYPPHPDLEEAADNYPSQTPCTSLHSCTHPSA
AITSTSTSHPPSTATPPSPAQSPLSRSKPPSPSSSCPTLSPSPGPATPMTQTSSLGQRRKGLARKVRRIQRQR
GRQTCTPASREGDRRSEEEEEREEAVGEERMEEDVEQEEERMEEETSEETAGHQQ
>Chelmon_rost__XP_041794469.1

MPPSAAAGAEDSKALVRKLLREVLVGREDPEGFFVKCVSALGHQETRSQFLSLIEPLSTANSSLHSTLTSIYM
EYFSKTEDDELELALALSLLEMKDHQMSAPSQESSLQHPGGDRPNQRSSIQLAPVSQPQESNHSQLADVADRR
KNINSPQGGPWVKVSPPQTTRETELQKSAHVYKYNKETPGTSQTVCVSRLSGSISKQDTVEEGDQMMDEGDVN
PSEKPKHSKNRRQRRKGAGQQVVGLPCSPSAPPPVLLWFRRDLRLCDNPALTGSLEFNAPVIPVFIWSPEEEE
GPGITVAMGGACKYWLHQALSCFCSSLERIGSHLVFLKANGEGNEVGSSQRALKELVKETGARTVLANALYEP
WLKERDDAVVSALQKDGVECRMFHSYCLRDPYSVSTEGVGLRGIGSVSHFMSCCRQNPGSALGVPLDPPVSLP
TPAHWPQGISLDTLGLARMPRRKDGTTIDWAANIRKSWDFSEEGAHARLEAFLRDGVYRYEKESGRADAPNTS
CVSPYLHFGQLSPRWLLWDAKGARCRPPKFQRKLAWRDLAYWQLTLFPELPWESLRPPYKALRWSSHRGHLKA
WQRGRTGYPLVDAAMRQLWLTGWMNNYMRHVVASFLIAYLYLPWQEGYRWFQDTLVDADVAIDAMMWQNGGMS
GLDHWNFVMHPVDAAMTCDPHGTYVRKWCPELADLPDELIHKPWKCPASMLRRAGVVFGQTYPERIVTDLEER
RSHSLQDVALVRKEFGQYVDKRSGCDLVPLPPRLVSEALGLSHRDGGVVTAGKQFLLPVITRMEFKHQLEDPD
ADATSNPYNAVLKGYVSRKRDETIAFLNERDFTASVMYEGTQRRERLESDYRRMEGLPRPPAPRGRPRRTPTA
KDRFSIVPGGAITSLR
>Chelmon_rost__XP_041799864.1
MAHTCVHWFRKGLRLHDNPALMAALRDCKELYPVFILDPHLHNNSCVGINRWRFLIGALKDLDCSLRKLNSRL
FVVRGKPEDVFPKLFNEWKVTKLTYEYDTEPYSVSRDKTVTALAKEHGVEVIYKISHTLYDIDRIIEDNNGKA
PLTYNRMQAIVKTLGPPKRPIPAPTMEDMKDVKTPISENHEKKYGIPTLEELGRDTAALVEEQFPGGEQEALR
RLDEHMKRTAWVCSFEKPQTSPNSLSPSTTVLSPYVTFGCLSARTFWWRLTDVYRGKKHSDPPVSLHGQLLWR
EFFYTASVGIPHFNKMEGNSVCTQVDWDTNPEYLAAWREARTGFPFIDAIMTQLRQEGWIHHLARHAVACFLT
RGDLWISWEEGQKVFEELLLDGDWALNAGNWQWLSASAFFHQFFRVYSPVAFGKKTDKHGDYIKKYLPLLKKF
PAEYIYEPWKAPHSVQQAAGCIVGKDYPHPIVQHEVISKKNIQRMKLAYAKRSADPAASPSKKQGVKRKTPSV
VDMLNKKERKK
>Chelmon_rost__XP_041804411.1
MLHCIWRSSSCSSLARPFLRLVARSTRRPTSVFCCRIKPFTGEAIMSEKKRKATSAAATKEPSAKQLKVAAAK
EQGWLEDMVQQQRSEKKEMKFNKKRLRFISDTEKMKQGSEGVLYWMSRDHRVQDNWALVHAQRLAVKENLPLH
VCVCLLVPKSELSTLRHYSFMLKGLEEVAKKCKSLNIQFHLLRGSAGDVIPGFVSDRSLGAVVTDFSPLREPL
QWLEDVKKKLPKDIPLIQVDAHNIVPCWVASPKLEYAARTIRGKITKLLPEFLTDFPPVEKHPYTATRTAKPV
DWDETLASLQVDRTVGEPEWAKPGTKAGMAMLESFIDVRLKLFDSQRNDPNAAALSQLSPWIRFGHLSAQRVA
LQVQRSGKSTGQPVASFIEELVVRRELTDNFCFYNKKYDSVEGAYEWAQKTLKDHAKDKRPYLYTREQLEKAK
THDKLWNGAQYQMVSEGKMHGFLRMYWAKKILEWTSSPEEALSIALYLNDRYELDGQDPNGFVGCMWSICGIH
DQGWAERPIFGKIRYMNYKGCLRKFDVARFERKYCPKNL
>Chelmon_rost__XP_041817076.1
MSTSRIIICLLRNDLRLHDNELFHWAQRNAEYIVPLYCFDPRHYVGTYNYDLPKTGPFRLRFLLESIRDLRNT
LLNKGSNLVVRRGKPEEVVADLIKQLGSVSTVAFHEEVTSEELNVEKRVKDVCAQMKVKVHTCWGSTLYHRDD
LPFNHISRLPDVYTQFRKAVETQSRVRPVFPTPEQLKPLPQGLEEGAIPTAEDLQQTEPVTDPRSAFPCSGGE
SQALARLKHYFWDTDAVATYKETRNGLIGVDYSTKFAPWLAMGCISPKYIYHQIKQYERERTANQSTYWVIFE
LLWRDYFKFVAVKYGNRLFQVKGLQDKSIPWKKDTKLFNSWKEGRTGVPFVDANMRELAMTGFMSNRGRQNVA
SFLTKDLGLDWRMGAEWFECLLIDHDVCSNYGNWLYSAGVGNDPRENRKFNMIKQGLDYDSNGDYVRRWVPEL
QGIKGADVHTPWALSTAALSHAQVSLGETYPTPIVTAPEWSRHVNKKASGTGPSPRGKKGPSHTPKQHRDRGI
DFYFSRSKNL
>Chelmon_rost__XP_041819920.1
MVINTIHWFRKGLRLHDNPSLKESLLGADTVRCVYILDPWFAGSSNVGINRWRFLLQSLEDLDSSLRKLNSRL
FVIRGQPTDVFPRLFKEWNISRLSYEYDSEPFGKERDAAIKKLACEAGVEVTVRISHTLYDLDKIIELNGGQS
PLTYKRFQTLISRMDAVEGPAESVTADVMGKCTTPLSEDHDDKFGVPSLEELGFDTEGLSSAVWPGGETEALT
RLERHLERKAWVANFERPRMNASSLLASPTGLSPYLRFGCLSCRLFYFKLTDLYRKVKKNSSPPLSLYGQLLW
REFFYTAATNNPCFDKMESNPICVQIPWDRNPEALAKWAEGRTGFPWIDAIMTQLRQEGWIHHLARHAVACFL
TRGDLWIGWEEGMKVFEELLLDADWSVNAGSWMWLSCSSFFQQFFHCYCPVGFGRRTDPNGDYIRRYLPILRG
FPAKYIYDPWNAPENVQKAAKCIIGVHYPKPMVHHAEASRLNIERMKQIYQQLSCYRGLGLLATVPSTSNGNR
NGNSETGFPVEASQDAAAPSGYQMPVHSQEDWQSGVMMYMQGDPQTSISTHQQGYAGTSNSVMCYTQGTQQIP
GPALQKGPEHHSTTQTSSKRHSEDSGNGKCSKVQRVQSSH
>Chelonia_mydas_XP_037738454.1
MKHISIHWFRKGLRLHDNPALLAAMTDCHKLYPIFILDPWFPKNMRVSVNRWRFLVESLRDLDESLQKLNSRL
FVVRGHPTEIFPVLFKEWKVTRLTFEIDTEPYSRQRDSEVVRLAADHGVQVIQKVSNTLYDTDRVIAENNGKV
PLTYVRLQTLLAALGPPKRPVPAPTLENLKDCCTPCKDNHDSEYGIPTLEELGQDPEQAGPRLYPGGESEALC
RLDLHMNRTAWVCNFQKPQTEPNSLSPSTTVLSPYIKFGCLSVRTFWWRLSEIYQGRKHSSPPVSLHGQLLWR
EFFYTAGAGIPNFNKMEGNPVCVQVDWDDNPEHLRAWSEGRTGYPFIDAIMTQLRTEGWIHHLARHAVACFLT
RGDLWISWEQGQKVFEELLLDADWSLNAGNWQWLSASAFFHQFFRVYSPVAFGKKTDKGGEYIKKYLPCLRKF
PAEYIYEPWKAPRSMQEQAGCVIGRDYPKPIVVHEVVSKRNVERMKAAYARRSASTTAQLEGGGGKKGARRKT
PAGPSVAELLTKKPKTKSS
>Chelonia_mydas_XP_037747093.1

MSGAGPRTVICLLRHDLRFHDNEVLHWAQNNADYIIPLYCFDPRQYLRTYYYGFPKTGPHRLKFLLESVKDLR
QTLKKKGSNLVVRKGNPEDVVHDLITQLGTVAAVAFHEEATKEELDVEKALQRVCAQHGVKIQTFWASTLYHR
DDLPFSHISRLPDVYTQFRKAVEAQAKVRPTVQMADQLKPLAPGVEDGCIPAPEEFGQKDPLTDPRTAFPCSG
GETQALMRLQHYFWDTNLVASYKETRNGLIGIDYSTKFAPWLALGCISPRYIYEQIRKYEKERTANQSTYWVI
FELLWRDYFRFVALKYGTRIFLLKGLQDKEVPWKKDPKLLDAWKEGRTGVPFVDANMRELAATGFMSNRGRQN
VASFLTKDLGLDWRMGAEWFEYLLVDYDVCSNYGNWLYSAGIGNDPRENRKFNMIKQGLDYDSNGDYVRLWVP
EIQCVKGGEIHTLWALSNAALSEAGIALGETYPLPVVLAREWSRHIKQRPNKRDHPPRGRKDPSDTPRQHKDR
GVDFYFSRNKDLS
>Chelonia_mydas_XP_037756053.2_LOW_QUALIT
MTGYVGGAPEAARARGGAGVGVWRSRAAEMAAAGSSLPGLARSVHWFRRGLRLHDNPALQAALRDASSVRCIY
ILDPWFAASSAVGINRWRFLLQSLEDLDNSLRKLGSCLFVVRGQPTDVFPRLFKEWGVTRLTFEYDSEPFGKE
RDAAIIKLAKEAGVEVVIENSHTLYDLDRIIELNGHKPPLTYKRFQTIISRMELPKKPVNSITSQQMEKCKSE
IQENHDDTYGVPSLEELGFPTDGLAPAVWKGGETEALARLDKHLERKAWVANYERPRMNANSLLASPTGLSPY
LRFGCLSCRLFYYRLWELYKKVKRNNTPPLSLYGQLLWREFFYTAATNNPKFDRMEGNPICIQIPWDRNPEAL
AKWAEGKTGFPWIDAIMTQLKQEGWIHHLARHAVACFLTRGDLWISWESGVRVFDELLLDADFSVNAGSWMWL
SCSAFFQQFFHCYCPVGFGRRTDPSGDYVRRYLPKLKGFPSRYIYEPWNAPESVQKAAKCIIGVDYPKPMVNH
AETSRLNIERMKQIYQQLSRYRGLCLLASVPSCVEDLSSPVTDTTSGQGSSTSTVQRLLHCGQTSPKRKHEGV
EELCTEELYKRAKVMGLHISEIPSKNL
>Chelonia_myd_EMP41017.1_cry-1
MPHRTIHLFRKGLRLHDNPTLLAALESSEVVYPVYILDRKFLMSAMNMGTLRWHFLLQSLEDLQRNLTKLGSC
LLVIQGEYESVLRDRVKKWNITQVTLDAEIEPFYKEMERTIQALGGEMGFDVLSLMGHTLYDIRRILDMNGGT
PPLTYKRFLHILSVLGDPDMPVRNLTAEDFQRCKPPPELCLAEDDRVPLPEDLDIPRESQSPWRGGETEGLQR
LEQHLKNQGWIANFTKPRTIPNSLLPSTTGLSPYFSLGCLSVRRFFYRLSNIYAQSKNHSLPPVSLQGQLLWR
EFFYTVASATPNFTRMAGNPICLQINWYQDAERLYKWRMAQTGFPWIDAIMTQLHQEGWIHHLARHAVACFLT
RGDLWISWEEGMKVFEEMLLDADYSINAGNWMWLSASAFFHQYTRIFCPVRFGKRTDPEGQYIRKYLPVLTNF
PSKYIYEPWTAPEEAQKQAGCIIGQDYPLPMVNHKEASDRNLQLMKQVREEQYGTAQLTRDDMDDPMEMGLKH
ELSEENPKKGKRARNTEQT
>Chelonia_myd_XP_037736147.1_cry-1
MGVNAVHWFRKGLRLHDNPALRECIQGADSVRCVYILDPWFAGSSNVGINRWRFLLQCLEDLDANLRKLNSRL
FVIRGQPADVFPRLFKEWNIAKLSIEYDSEPFGKERDAAIKKLASEAGVEVIVRISHTLYDLDKIIELNGGQP
PLTYKRFQTLISRMEPLEMPVETITAEVMEKCTSPVSDDHDEKYGVPSLEELGFDTDGLPSAVWPGGETEALT
RLERHLERKAWVANFERPRMNANSLLASPTGLSPYLRFGCLSCRLFYFKLTDLYKKVKKNSSPPLSLYGQLLW
REFFYTAATNNPRFDKMEGNPICVQIPWDRNPEALAKWAEGRTGFPWIDAIMTQLRQEGWIHHLARHAVACFL
TRGDLWISWEEGMKVFEELLLDADWSVNAGSWMWLSCSSFFQQFFHCYCPVGFGRRTDPNGDYIRRYLPILRG
FPAKYIYDPWNAPESIQKAAKCVIGVHYPKPMVNHAEASRLNIERMKQIYQQLSRYRGLGLLATVPSNPNGNG
NGGLMGYSPGENISGCSSAGGAQLGSTDGHTVGVQTCALEDSHAGSSGIQQHGYSQGSSILHYAHGDHQQSHL
LQQGRTGSTSISTGKRPNPEKETQSIGPKVQRQSTN
>Chelonia_myd_XP_037765512.1_photolyase
MSQKSRNRRAHGGEDTTASPAGSGDTARTRAAAVPRGQLKGKRKVLAEPSGAEALTVAKRRRKEGEAAEGGGA
GSLGEAVRQARLRVAPSVQEFRYNKKRVRLVSESSDLKDAAKGVVYWMSRDQRVQDNWAFLYAQRLALKQQLP
LHVCFCLVPKFLEATIRHFSFMLKGLQEVAKECQELDIPFHLLIGFAKDVLPAFVTDHGLGGVVTDFSPLRVP
MQWVEDVKERLPGDVPFVQVDAHNIVPCWVTSDKQEYGARTIRRKIHDRLPEFLTEFPPVIKHPFPPTAPAEP
IDWSACRASLQVDCSVKEVRWATPGTAAGLAMLESFIGERLKFFGTDRNNPTKAALSNLSPWFHFGQVSVQRA
ILEVQKHRSQYKESVDGFIEEAVVRRELADNFCFYNRNYDKVEGAYDWAKTTLKLHAQDKRSHLYELKQLEEG
KTHDPLWNAAQLQMVHEGKMHGFLRMYWAKKILEWTRSPEEALKFAIYLNDRYELDGRDPNGYVGLLAGSWGP
VKRCRRVQET
>Chiloscyllium_plag__XP_043558343.1
MSKNGKQTDRNAKDSEHEKKIEEKSNKKETAFSSSSNSLKTSTKKSTKKRNHNNSSREDSPKLQAKGKRTVEE
RPPGLIERKLSNSRINKKRKTEHQAETSLSGEGNLLKSINKSRLATAKSVEEFNFNEKRIRLLSKTESVRENS
WGIIYWMSRDQRVQDNWAFLYAQRIALKHKLPLHVCFCLVPKFLDATIRHFGFMLKGLQEVAEECKQLNISFH
LLIGFAKDVLPEFVKGHNIGGIVTDFSPLRLPLEWIEDVRHNLPENLPIIQVDAHNIVPCWVTSDKQEYSART
IRKKIHDKLPQFLTDFPPVIQHPFSSSLKAQLIDWDAAAANLEVDRTVKEVDWAKSGTAAGLIRMESFIKEQL
QHFSTDRNNPNKTALSNMSPWFHFGQVSVQRTILEIKKYRNKFKDSVDSYIEEAVVRRELADNFCFYNKQYDK
IEGASDWAHKTLKMHSKDQRQYLYTLKELEDGRTHDSLWNAAQLQMIHEGKMHGFLRMYWAKKILEWTTSPEE
ALRFSIYLNDRYELDGRDPNGYVGCMWSICGIHDQGWKERPVFGKIRYMNYNGCKRKFDIGQFERKYSYN
>Chiloscyllium_plag__XP_043565791.1
MGGHSIHWFRKGLRLHDNPALLEALVGSDTIRCVYIVDPWFAGSSSVGINRWRFLLQCLEDLDANLRKLNSRL
FVVRGQPADVFPRLFKEWNISRLTFEYDSEPFSKERDAAIKKLAIEAGVEVIVRISHTLYDLDMIIQHNGGQP
PLTYKHFQTLISRMESIEIPVETITAEDMGKCTTPVFDDHDDKYGVPSLEELGFDTEGLSSAVWPGGETEALA
RLERHLERKAWVANFERPRMNSTSLLASPTGLSPYLRFGCLSCRLFYFKLTDLYKKVKKNSTPPLSLYGQLLW

REFFYAAATNNPRFDKMEGNSICVQVPWDRNPEALAKWAEGRTGFPWIDAIMTQLRQEGWIHHLARHAVACFL
TRGDLWISWEEGMKVFEELLLDADWSVNAGSWMWLSCSSFFQQFFHCYCPVGFGRRTDPNGDYIRRYLPVLRG
FPAKHIYDPWNAPECVQKAAKCIIGVHYPKPMVNHAEASRLNIERMKQIYQQFSRYRGLGLLASVPSNPNTSA
GPMEYSQGGSTHGCSSITTGKSFLVSSRSSGKRPSLGEGIPRVEPQSQRQNND
>Chiloscyllium_pun_GCC23239.1_chiPu_0001
MIDARAKLKGECKSTAIEGQQRLRLAAQSRLLSITCRSFCHRLCVIYCPQPSGCVSLQCAKQRNNMSKNGKQT
DRNAEDSEHKKKIEEKSNKKETAFSSSSNSLKTSTKKSTKKPNHNNSSREDSPKLQAKGKRTVEERPPGLTEG
KLSNSRINKKRKTEHQAETSPSGEGNLLKSINKSRLATAKSVEEFNFNEKRIRLLSKTESVRENSWGIIYWMS
RDQRIQDNWAFLYAQRLALKHKLPLHVCFCLVPKFLDATIRHFGFMLKGLQEIAEECKQLNVSFHLLIGFAKD
VLPEFVKGHNIGGIVTDFSPLRLPLEWIEDVRHNLPENLPFIQVDAHNIVPCWVTSDKQEYSARTIRKKIHDK
LPQFLTDFPPVIQHPFSSSLKAQLIDWDAAAANLEVDRTVKEVDWAKPGTAAGLIRMEAFIKEQLQHFSTDRN
NPNKTASSNMSPWFHFGQVSVQRTVLEIKKYRNKFKDSVDSYIEEAVVRRELADNFCFYNKQYDKIEGASDWA
QKTLKMHSKDQRQYLYTLKELEDGRTHDSLWNAAQLQMIHEGKMHGFLRMYWAKKILEWTTSPEEALQFSIYL
NDRYELDGRDPNGYVGCMWSICGIHDQGWKERPVFGKIRYMNYNGCKRKFDIGQFERKYSYN
>Chiloscyllium_pun_GCC37125.1_chiPu_0015
METHSIHWFRKGLRLHDNPALKEALRGASTVRCVYILDPWFAGSSNVGVNRWRFLLQCLEDLDTSLAELNSRL
FVIRGQPADVFPRLFKEWNINRLTFEYDSEPFGKERDAAIKKLASEAGVEVIVKISHTLYDLDKIIELNGGQP
PLTYKRFQTLISQMDPPEPPVETISERSLGKSTILVLDDHNEKHGVPSLEELGFDTEGLPSAVWPGGETEALT
RLERHLERKAWVANFERPRMNALSLMASPTGLSPYLRFGCLSCRLFYFKLTELYKNIKKTDSPPLSLYGQLLW
REFFYTAATNNPCFDKMEGNPICVRIPWDKNPVSLAKWAEGKTGYPWIDAIMTQLRQEGWIHHLARHAVACFL
TRGDLWISWEEGMKVFEELLLDADWSVNAGSWMWLSCSSFFQQFFHCYCPVGFGRRTDLNGDYVRRYLPILKG
FPAKYIYDPWNAPNSVQVAAKCIIGIHYPKPMVIHAEASRLNIERMKQIYQQLSRYRGLGLLASVPSNQNGSG
GTVYCLGDQITENNGIVQAHTSGGMVGKRELERDTAVSDDMESLAQKVRRQCDDNNEDEQNLNLVSY
>Chilo_sup_AHL69753.1_cry_2
MSAAAQTLPSSTAARTHGPAALAPTPSKCNPTGKHTVHWFRKGLRLHDNPALREGLNNAVTFRCVFIIDPWFA
SSSNVGINKWRFLLQCLEDLDCSLRRLNSRLFVVRGQPADALPKLFREWGTTELTFEEDPEPYGRVRDHNIMS
KCREIGINVVSRVSHTLYKLDKIIERNGGKAPLTYHQFQALIASMPPPPPAEAAISAQGFNGATTPIADDHDD
RFGVPTLEELGFDTEGLKPPVWIGGESEALTRLERHLERKAWVASFGRPKMTPQSLFASQTGLSPYLRFGCLS
TRLFYYQLTELYKRIKRVQPPLSLHGQILWREFFYCAATRNPNFDRMEGNPICVQIPWQKNQEALAKWANGQT
GYPWIDAIMIQLREEGWIHHLARHAVACFLTRGDLWISWEEGMKVFDELLLDADWSVNAGMWMWLSCSSFFQQ
FFHCYCPVRFGRKTDPNGDFIRRYIPALKNMPTRYIHEPWLAPEAVQQSARCIVGRHYPLPMVDHTKASQINI
ERIKQVYAQLAQYKPLAILNPHTLQRPNVMQPSPSPTSILTNINQSNFLCSQNSDAQIISTVQNSTTKSSNVF
QHPGANNNSRNDTKQSQSKPVVIIQRENSANVQKIDYGRANPCPSPRPQQNYIINGQDVPHKSKEDTERAQKS
KQEENYNLKIFDINKFIQDYSNNQKTYPNQERKTEDVYQQDEKSISYGFTKPKFYMSYPNNGIVRNEHSHIET
QEDEPTTRNHEYAGESTKETKKEKQIHNNCIQ
>Chilo_sup_CDK02014.2_cry_1
MLGGSVLWFRHGLRLHDNPALLSALEDRRMPLFPIFIFDGETAGTKLVGYNRMRYLLEALDDLDNQFKKFGGR
LIMLKGKPNIVFKRLWEEFGIRKLCFEQDCEPVWRARDDSVKDACREIGVQCREHVSHTLWEPDTVIRANGGI
PPLTYQMFLHTVDTIGDPPRPVVDADLCGVKFGALPECFYQEFTVFDKTPKPEELGVFLENEDIRMIRWVGGE
TAALKQMQQRLNVEYETFCRGSYLPTHGNPDLLGPPISLSPALRFGCLSVRRFYWSVQDLFQKVHQGRLSSTH
YITGQLIWREYFYTMSVNNPNYGQMAGNPICLDIPWKSPEGDELQRWKEGRTGFPFVDAAMRQLLTEGWLHHV
VRNTVASFLTRGSLWLSWEHGLQHFLKYLLDADWSVCAGNWMWVSSSAFEALLDSGDCACPVRLGQRLDPSGE
YVRRYLPALARVPDRYIYEPWKAPIEVQEQANCIIGKDYPAPMVNHLVAAERNRFAMKDLREMLQKAPPHCCP
SSEEEIRQFMWLNDEPAIALSTT
>Chilo_sup_QCH40574.1_CRY1_X1
MANKASVVHWFRLDLRIHDNLALRNAINEAENRQYYLRPVFIIDPDIKERVGVNRLRFLFQSLQDLDSNLRKL
NSRLFILKGTADELFSKLFDQWQVKYLTSQIDIDPEIVKQDEIVDRLAEEKGIFIVRRVQHTVYDFHSVLKKN
NGSVPLTYQKFLSLVKDIKVKETIEITKTLSDYCKPPDSTCEEYNVPSLNDLGIDESSLKSCKYHGGETEGLK
RLNLYMEKKNWVCKFEKPNTSPNSIEPSTTVLSPYLSHGCLSSKLFYHRLKGVENGVAHSQPPVSLLGQLMWR
EFYYTAGAGTENFDKMVGNAVCTQIPWGKNDKYLKAWSEGMTGYPFVDAIMRQLKQEGWIHHLARHMVACFLT
RGDLWISWEEGARVFEDYLLDYDWSLNAGNWMWLSASAFFYKYFRVYSPVAFGKKTDKEGLFIRKYVPELKKY
PTEYIYEPWKAPKSVQRTAGCVVGEGYPQRIVDHDKIHKENMQKMAAAYKVNKEKKSLKRKLT
>Chilo_sup_RVE51139.1_evm_004282
MVAGFVKAESTNLPRIDALMVGEFVALNPDFCSAEQRNDKASIMASAAKKPKLSISSSSAKEETTDLTSFMQK
IQKKREETAESILQYKFNKKRLRIVSQEQMVADKCEGIVYWMSRDSRVQDNWALLFAQKLALKNEVPLHVCFC
LIAKYLDASVRQFHFLIKGLEKVAADCKKLNITFHLLEGSGAEALPQWVVDHKIGAVVCDFNPLRVPLGWLED
CKKKLKKDVPLIQVDAHNIVPCWEASNKQEYSARTIRNKINSKLGEYLTEFPPVIKHPYTSKFEPEPIDWDQA
IESREADKSVGPVDWAKPGYDEAVKMLKSFIDKRLKIFSSKRNDPTQDACSNLSPWFHFGQISVQRVALCVQE
YKSKHTESVNAFLEEAIVRRELADNFCFYCEHYDSLKGASAWAQKTLDDHRKDKRTHVYSLEQLSKAETHDDL

WNSAQLQLVKEGKMHGFLRMYWCKKILEWSPTPEDALKYSIYLNDHYSVDGRDPNGYVGCMWSICGIHDQGWA
ERAVFGKIRFMNYDGCKRKFNVNAFVARYGGKIHKNTSKK
>Chionoecetes_opi_KAG0711927.1_cry_DASH
MKFGDRVFYPSGIMGKNIQWKKDLHLFQKWKGVYLASFRTLSHHLARSHTAHSYLQLSEGRTGVPFVDANMRE
LLETGWMSNRGRQNVASFLIKDMGLDWRLGAEWFESQLAFPYRMGRHPAQSSLPTNT
>Chionoecetes_opi_KAG0725286.1_photolyase
MLSKADEVPDCDGVVYWMSRDQRVQDNWAMLFAQRLALKYKVSLHVVFCLVPKFLDATIRHYQFMLKGLEEVE
SECRELSISFHLLPGEPNAVLPEFIKSHNIGGLVTDFSPLRLPRLWVKEVKKAIPQNIPMCQVDAHNIVPCKK
ASEKQEYSARTIRKKIHDKLPDFLTKFPPVIKHPHESKGENEAVDWAAAEETLEVDMKVKAVEWAVPGTQAGL
NTLDAFCLKRLRKYDKQRNDPNANALSNLSPWLHFGQISAQRCVLEVKRYKKQLKESVDNFIEETVVRRELGD
NYCYYQENYDRLEGAYQWAITTLNDHKKDPRPYLYSLETLQEAHTHDELWNSAQIQLVKEGKIHGFMRMYWAK
KILEWTASPETALETAIYLNDRYSLDGRDPNGYVGCMWSICGIHDQGWGERAVFGKIRYMNYDGCKRKFKIDG
YVARYGGKKHKYVPPSK
>Chionoecetes_opi_KAG0726473.1_cry-1
MLNLGVFSQKRLAMKHGMKVTGYNRQHYLMGALSDLDDQLREHGSQLFMCKGSPVDVFTTLHKEMGVTHLSFE
QDCEAIWNRRDDEVRKMCSELGITMMERIAHTLWDPFEIIEANGGQPPTTYEMFVQVAGALGLPHQPSPDPDW
QDVTFGELPDEVAQKIKLCPQVPSPEEQGWTRNGECPVYSGGEKAALAHLRERLIVEDKAFR
>Chionoecetes_opi_KAG0727244.1_cry-1
MTGDLIKPRPKHHVVHWFRKGLRLHDNPSLRAGLKNAATFRCIFILDPWFAGSSNVGINKWRFLLQSLEDLDT
SLRKLNSRLFVIRGQPAHVLPELFREWDTTCLTFEKDPEPFGKVRDANIVAIAREMGINVIVKTSHTLFKPEK
IIDKNGGKPPLTYKSFQNILMTMDLPQLPLSTITLGDLENKCTPLTDDHDDKYGVPTLEELGFDTESLQQTGW
KGGESEALTRLEHHLERKAWVASFGRPKMTPQSLYPSHTGLSPYLRFGCLSCRRFFKELNELYKKIRKSPPPL
SLHGQLLWREFYYTAATNNPKFDHMEGNPICVQIPWDKNPEALAKWANGQTGYPWVDAIMTQLRNEGWIHNVA
RHAVACFLTRGDLWVSWEEGMKVFDELLLDADWSVNAGSWMWLSCSSFFQQFFHCYCPVRYGRKADPNGDYIR
TYLPVLKNFPTKYLHQPWTAPESVQRAARCVIGRDYPLPMVDHIKQSQNNIERMKQVYQQLAHYRGKINPAIK
SPTMDHYPSPPPDFDKKRSSKTGKTDIHRDSGYRVHGQTCLKLTTGDHEC
>Chionoecetes_opi_KAG0728005.1_cry-1
MTTVHWFRKGLRLHDNPALLAALKEGAELRPVFVLDPWFVKNARVGVNRWRFLSQTLEDLDACLRKLKSRQVV
QLFVVRGSPEEVFPVLFKKWNVKRITWEIDTEPYARVRDEKVERLARESGVEVVCRVGHTLYRTEKIIDANMG
KTPLTYQSLQKLVGKLGHPPRPLDTLTSLPDSCHLHGDAATDISFEPPSLKELGVDESQLEPCLYPGGETEAL
VRLERYMERKSWVCKFEKPKTSPNSLQPSTTVLSPYLKFGCLSPRTMYHQLLQVYKGSTHTQPPVSLMGQLLW
REFFYTVGATTPNFDRMEGNPVCRQIPWERDQKLMDAWTNAKTGYPFIDAIMTQLRKEGWIHHLARHAVACFL
TRGDLWQPWEDGMKVFEELLLDADWSLNAGNWMWLSASAFFNSYFRVYSPVAFGKNTDKYGEYIRKHLPQLAK
YPENYIYEPWKAPLGVQRAAGCVIGQDYPRPIVDHSIIMKRNMERMGKAYKAAKEKKAASDSQSSPRKKMKK
>Chlamydomonas_rei_12g534550v5_XP_042918714.1
MDSHGPLPLAALSLHALGAPAGLGASRHAARPTKAAPPPVSARDGVRSHPINAALATSDSSSASSGAADSAPA
QRPGPHALQRLVSAVPAALARLVTGPFHQPAFNGPASLSDMRVFPAPVPKRAAPGGGGRPAGGAARKAALVWF
RNDLRLHDNPALEQACRQSSSVLPVYVFDPRDYGKTPSGFDRTGPGRARFLLEAVADLRQRLRDAGSDLVVRL
GRPEAVLKELAAAVGAGAVYCQSEVTAEEMQVEGRVRAALDRESCELRPQWGGTLFHLEDLPFRLDAMPTSYA
DFRERVANLKVRQLSESDGGIKGLPAGNSVEPGDIPTLQRLGFSPAAATAGAAAPGSGLGAPLQLGGGPALVL
RGGESEALRHMQAFIDELRRAVSGAAAAASGSGKPGAAPPSATFSCRISPWLALGCLSPRRMYHEMRQQLAPA
GAPVIRSSSSSAIGSGSAAAPLSGPAKQGGAAAGGAGSPANWLVFELLWRDFFRFVTQKHSAGARLAAAKSAA
RSGRSAAAAPVAPAMAAALA
>Chlamydomonas_rei_AAD39433.1_class_II_D
MSSKRKATEAPAAGEDGAAGPSSKKQAAASASAAAAAAGAGSAAAGGALVNPKRVRVLKPGSIGKGPVVYWMS
RDQRLADNWALLHAIEAAQGAAGSSQVAVAFNLVPAFLGAGARQFGFMLRGLRQLAPRLEARGIKFYLLKGDP
AHTLPQLVSGLGAGLLVTDYSPLRLGRTWRDQVCSALGSVPVHEVDAHNVVPVWAASEKREVGARTLRPKIHK
ALPEFLREFPEVPTLPAWTPAVAPEAVDWDGLISEVLSRGADVPEVEWCTPGEEAALEALTGPRGFLSPARLS
LYDTKRNDPATPSALSGLSPYLHFGQLAPQRAALEAAKHRAKYKAAVESYLEELVVRRELADNFCHYCPTYDS
LEAAAEWARDSLDKHRTDKREFLYTRDQLECGATHDELWNAAQLEMVHVGKMHGFMRMYWAKKILEWTQGPEQ
AIEWAIYLNDRYELDGRDPGGYTGVLWSMAGVHDMGWAERAVFGKIRYMNYNGCKRKFDIKAYVAYVSKAVAE
AKAKGRAAKLPSAAAAGASGAAAAGATAAAAAAAAPGPSGAQAAKAAKAKAEPKEAKPKAAKAAAKAKGPKDE
KAAAAGAKRKAAKPAKSASSGEEGSDDE
>Chlamydomonas_rei_XP_001698054.1_cry_photor
MAGVKNSIIWFRKGLRLHDNPALLEACKDAKHVYPVFVLDPHFLQQSSYKVSVNRYNFLLESLEDLQRSFQAR
GSRLLVLRGKPEEVFPRVFREWGVTQLCFEHDTEPYAKVRDAAVRRLAAEAGVEVVTPISHTLYDTDMLVARN
GGAAPLTMQSFTKLVDRVGDPPAPAPDPPAAMPPPAEDMPSAAPAATGVPTWQEVGFKEPPLTVFKGGETEAL
ARLEAAFQDPKWVAGFQKPDTDPSAWEKPATTVLSPYLKFGCLSARLFHARLLEVYRRHPAHSQPPVSLRGQL
LWREFFYTVGSTTPNFHRMAGNPVCKQIDWDDNPEFLAAWREARTGFPWIDAIMTQLVTWGWMHHLARHSVAC
FLTRGDLYVSWERGMEVFEEHLIDQDHYLNAANWMWLSASAFFSQYFRVYSPVVFGKKYDPEGRFIRKFLPVL

KDMPAKYIYEPWTAPLEVQRKAGCVVGRDYPAPIVDHAVASKACIARMAAAYRRSKGEAGGDGDDSGGEGGSG
GGAAAGRKKATAAAGGSSGAGRGKKAAAAVAAGSAAGAKPATGRGKAAAKAKSDVVVSGKAAGGGSKRQRTIK
ESLGAAAAADE
>Chlamydomonas_rei_XP_001701553.1_cry_photor
MPHEFKTAVVWFRRDLRVDDNPALVAALAAAPNVIPVFIWAPEEEGQFQPGRCSRWWSKHSLVDLQQALAALG
SRLVIRRSTDSTAALLQLVTELGAEAVFFNHLYDPISLMRDHDCKRGLTAAGVAHRTFNGDMLYEPWDVLDPN
KQPYSTFDDFWNSVRAMPVPPPFPVSAPASMPAVPAAVPSMTVAEVDWFFTPEQEASSDQLKFKWKPGVGGAI
SELEHFLAERLTEFEHDRAKVDRDSTSRLSPWIHIGSISVRYIFYRVRQCQAEWLAAGTDRAQSCDDFLQQMG
YREYSRYLAFHFPFIHERSLLRHLRACPWRIDQHAFKAWRQGQTGYPIVDAAMRQLWSSGWCHNRGRVVAASF
LVKDLLLPWQWGLKHYWDAQIDADLECDALGWQYVSGGMSDAHPFSYMMDLEKEARRFDPDGEYVRRWLPALS
RLPTEYIHAPWKAPASVLAAADVELGCNYPLPIITRSDAKANVDYACGVLEKSAVAPTGSESSGRYPYRAPTY
PNAGGGGGASGGVDGAGSSGGNPTAAMGASGGAGPSSGTGTGGQGGAFRGRGDGGGSAPVSQQGGMLPPGVAV
CVTAGTGGTQPDSRTVSGAHAGVSNSAGGVACDMPPPSHSHPRGGSSGVAAAGVGGVSGGPSSGGAAAPGATR
KATAPGGAVFYHPGEASGEGHALLERILQQQRRQRGAANRQDGSGTNQPPPPVSMPGMLDAAGAAGGGGGGGG
GLAGLPGVLQGLHGGAGATAAPAVAWAGRVAAGGVDDMDAVALWQQQQQLLQGGAAAFAFEQAMELLLARRRA
GDGVSGFCGGDAPGRGGGDADEADGLMLEEDSEEVVSNTVDLHLSAFTGASCHRQSGTARGARAAERRLKKEA
GSPEVTDGGGSAGAGCGEEDAMQCEDEEHGERGSPARRTADEEAQPPAEEGGAGSEDAGAAASEGDEAEGYGS
ESASEDNGGATPVEEADQMVVDGGGGGGGGGGGGGRGGGQHKRRRSQLEGEQGDAAWE
>Chlamydomonas_rei_XP_001701871.1_cry_DASH1,
MATAGGGQRLVLWFRNDLRLHDNYIVHEAAQRVKRGEASEVLPVYVYDPRFFAATPWGALKTGAHRAKFIQEC
VADLRQRLQGLGSDLVVAVGQPEQLLPALLEGGGAAPLVLTAEEVTSEEAAVDVAVARAIKPAGGKLLRYWGH
TMYHYDDLDLPDVFTPFKEKVEKRAALPLPPAPRLGATAAAALAAPLPGWEQLPPAPPVTHPKAVLDFKGGET
AALARLKYYLWDSDLLSTYFDTRNGMLGGDYSTKFAPWLAQGCISPRKIFHEIRKYESQRFSNKSTYWVIFEL
IWRDFFRFFALKHGNRIFFETGTSGLPLVWNPDPELWARWREGRTGLPLVDANMRELAATGFMSNRGRQNVAS
YLVLDLGVDWRRGADYFEEVLLDYDVTSNWGNWVAAAGLTGGRVNHFNIAKQSKDYDPTGEYVKTWCPELKNV
PVTKVHEPWLMSKEEQERSGCRIGVDYPNPI
>Chlamydotis_macq__KFP36953.1
RFLLQCLEDLDANLRKLNSRLFVIRGQPADVFPRLFKEWNIAKLSIEYDSEPFGKERDAAIKKLASEAGVEVI
VRISHTLYDLDKIIELNGGQPPLTYKRFQTLISRMEPLEMPVETITPEVMEKCTTPVSDDHDEKYGVPSLEEL
GFDTDGLPSAVWPGGETEALTRLERHLERKAWVANFERPRMNANSLLASPTGLSPYLRFGCLSCRLFYFKLTD
LYKKVKKNSSPPLSLYGQLLWREFFYTAATNNPRFDKMEGNPICVQIPWDKNPEALAKWAEGRTGFPWIDAIM
TQLRQEGWIHHLARHAVACFLTRGDLWISWEEGMKVFEELLLDADWSVNAGSWMWLSCSSFFQQFFHCYCPVG
FGRRTDPNGDYIRRYLPVLRGFPAKYIYDPWNAPESVQKAAKCIIGVNYPKPMVNHAEASRLNIERMKQIYQQ
LSRYRGLGLLATVPSNPNGNGNGGLMGYSPGESVSGCGSTGGAQLGTGDGHTVQQCALGDSHTGASGIQQQGY
CQASSILHYAHGDNQQSHLLQAGRTTLGTGISAGKRPNPEEDTQSVGPKVQRQSTN
>Chlamydotis_macq__KFP42943.1
DNWAFLYAQRLALKQELPLRVCFCLVPKFLDATIRHYNFMLRGLQEVAEECTELNIPFHLLLGYAKDVLPAFV
VEHGVGGLVTDFCPLRLPQQWVEDVRERLPEDVPFAQVDAHNIVPCWVASPKQEYSARTIRGKIHAQLPEFLT
EFPPIIRHPYPPSCPAEPVSWEACYSSLQVDRTVKEVEWATPGTAAGMAVLQSFIAERLKSFGSHRNDPNKAA
LSNLSPWFHFGQVSTQRAILEVQKHRGKYKESVDTFVEEAVVRRELAENFCYYNENYDSVQGAYDWAQTTLKL
HAKDKRPFLYELQELEQGSTHDPLWNAAQLQMVREGKMHGFLRMYWAKKILEWTRSPEEALQFAIYLNDRYEL
DGRDPNGCLWSICGIHDHGWVERAIFGKIRYMNYAGCKRKFDVGQFERRYAP
>Chlamydotis_macq__XP_010113633.1
CRSVHWFRRGLRLHDNPALQEALRDAASLRCIYILDPWFAASSAVGINRWRFLLQSLEDLDNSLRKLNSRLFV
VRGQPTDVFPRLFKEWGVTRLTFEYDSEPFGKERDAAIVKLAKEAGVEVVIENSHTLYDLDRIIELNGHKPPL
TYKRFQAIISRMELPKKPVSTVTSQQMETCKVDIQENHDDVYGVPSLEELGFPTDGLAPAVWQGGETEALARL
DKHLERKAWVANYERPRMNANSLLASPTGLSPYLRFGCLSCRLFYYRLWELYKKVKRNSTPPLSLYGQLLWRE
FFYTAATNNPKFDRMEGNPICIQIPWDRNPEALAKWAEGKTGFPWIDAIMTQLRQEGWIHHLARHAVACFLTR
GDLWISWESGVRVFDELLLDADFSVNAGSWMWLSCSAFFQQFFHCYCPVGFGRRTDPSGDYVKRYLPKLKGFP
SRYIYEPWNAPESVQKAAKCIIGVDYPKPMVNHAETSRLNIERMKQIYQQLSRYRGLCLLASVPSCVEDLSGP
VTDSASGQGCSTSTAVRLSQADQASPKRKHEGAEELCAEELYKRAKVTGLPVPEMPGKSL
>Chlamydotis_macq__XP_010116266.1
MPHRTIHLFRKGLRLHDNPTLLAALESSEAVYPVYILDREFMTSVMHIGALRWHFLLQSLEDLQKNLRQLGSC
LLVIQGEYETVLRDHVQKWNITQVTLDAEMEPFYTEMEANIRRLGEELGFEVLSLVGHSLYDTKRILDLNGGT
PPLTYKRFLHILSLLGDPAAPVRNLTAEDFQRCRASDPGLAERYRVPLPVDLKIPAESLSPWRGGETEGLQRL
ERHLTDQGWVASFTKPRTIPNSLLPSTTGLSPYFSMGCLSVRTFFYRLSNIYAQAKHHSLPPVSLQGQLLWRE
FFYTVASATPNFTRMAGNPICLQISWYQDAERLHKWKTAQTGFPWIDAIMTQLRQEGWIHHLARHAVACFLTR
GDLWISWEEGMK
>Chlamydotis_macq__XP_010117280.1

VLHWAQSHADFVIPLYCFDPQHYLGTHCYGFPKTGPPRQKESVKDLRETLKKKGSTLVVRKGKPEDVVRDLIT
QLGSVSAVAFHEEATQEELDVEKGLCQVCSQHGVKIQTFWGSTLYHRDDLPFRPIARLPDVYTHFRKAVESEA
KVRSTLRMADQLKPLAPGVEEGCIPTMEDLGQKDLVTDPRTAFPCSGGETQALMRLQYYFWDTNLVASYKETR
NGLVGMDYSTKFAPWLALGCISPRYIYEQIQKYEKERTANQSTYWVLFELLWRDYFRFVALKYGKRIFSLRGL
QSKEVPWKKDLQLFDCWKEGKTGIPFVDANMRELAATGFMSNRGRQNVASFLTKDLGLDWRMGAEWFEYLLVD
YDVCSNYGNWLYSAGIGNDPRDNRKFNMIKQGLDYDGNGDYVRLWVPELQGIKGADIHTPWALNRAALAQAGV
TLGETYPQPVVTAPEWSRHISQKPQGRSPHPRGRREPAHTPAQHKDRGVDFYFSHKKDVHTLEKP
>Chlamys_isl_AYE92098.1_cry_1,_par
LRIHDNPALCDALENCDEFYPIFIFDGEVAGTKFSGFNRMRFLHESLEDLDNTFKKNEGRLYTFQGNAKDVLK
GLFQEWNVTRLTYEAEVEPIWEERDLEVEELCREKGIQLISRISHTLWNPSDIIDNNGGTPPVTYNHFELTTS
LVGSPDRPVPVPKLSKVNMPVSDNFDKKFALPSVKELGYEPECKEQTERINNWLGGETKALQLLEERLKNERE
PITDGIMLPNQLVPE
>Chlamys_isl_AYE92099.1_cry_2,_par
YRCVYVLDPWFAGASQVGINKWRFLLQCLEDLDCSLRKLNSRLFVVRGQPTNVFPLLFKEWNITTLSFEEDPE
PFGKERDAAIKALAMEAGVETIVRTSHTLYDLQKIINQNNGMSPLTYKRFQMILSKMDPPKQPEETINAAYLG
KCRTPLSDEHDEHYGVPTLEELGFDTEGLKPAELRGGEMEALSRLERHLERKAWVASFEKPKMTAQSLFPSQT
ALSPYLRFGCLAPRLFYWKLAELFRKVKKQSEPPLSLHGQLLWREFFYTAATNNPNFDRMKDNPICVKVPWDH
NPEGLAKWAEGMTGYPWIDAIMRQLKQTGWIHHLARHAVACFLTRGDLWISWEEGMKVFDELLLDADWSVNAG
MWMWLSCSSFFQQFFHCYCPVSFGKRADPTGDFIRSYVPELKNYPSKYIFEPWNAPPSVQKAAKCVVGKDYPL
PIIDHGDASKVN
>Chloracidobacterium_the_WP_058868137.1_DNA_photol
MTKRILCWFRRDLRLDDNTALLAAYAAAEEVVPVFIFDDAILSRPDTGAVRVAFLLESLRNLDENLRARGSQL
LLRRGRPEQVLAQLVTETAASAVYFNRDVEPFALVRDARVQAHLEGRCAVEAFDDSGLTAPDAIRTKAGTPYT
VFTPYKQAVLAQPVPHPRPAPAMLRTPANVPSDPWPSLKDLGFATSVSPPPGGETPAQARLQDFIQNGLARYA
TERDVLSADGTSRLSPYLRFGCLSPRRAYWAAREAVPEGAPGAETWIAELIWRDFYRQILFHFPYVETGAFRR
AYDDLAWENNASWFDLWCQGKTGFPIVDAAMRQLLTTGWMHNRARMIVASFLTKDLLIDWRWGERHFMKHLVD
GDLAANNGGWQWAASTGTDAQPYFRIFNPTAQGKKFDPTGAYVRQYVPELRAVPDRWIHEPAQMPVEVQRAFR
CVIGVDYPAPMVDHSRQRAKALTMFGRFASPEKRPGR
>Chlorella_sor_PRW45175.1_photolyase
MAALAAAGFCTTARIAACRGSLRSSATQRVRARWVVVQVRMSSGGGGSSGGGGGNRAVLWFRGTDLRVHDNVV
VHEAARRVQAGQVSEVLPLYCFDPRFFSASAWGNPKTGAFRAQFLLESVLDLKRQLRVLGSDLVIAMGRPEEV
LPGLMTERGSGGRTTVIAQSEVTSEETRVERKVKSAVAAAGGKLELWWGSTMFHLDDLPFREDLSDLPDVFTP
FKQKCEDRSKVRACLPTPKRGALPLPGGIPADLLAFEPQRVEDLNAVVPAGHPQLATTPLDARGVLDFKGGES
AALARLNYYLFESHRIAEYFDTRNGMLGGDYSTKFAPWLAHGCLSPRTIYHEIKRYESQTGTANKSTYWVIFE
LIWRDFFRFFALKHGNKIFFEGGVTDQAQPWSHDAEAFRRWKEGRTGWPLVDANMRELAATGFMSNRGRQNVA
SFLALDLGLDWRWGADWFESLLLDYDVCSNWGNWVAAAGLTGGRVNKFNITKQSNDYDPQGDYIRTWVPELKG
VPARRIHEPWLMSREEQAAAGVQIGVDYPSPIKSSWKGYGGGGGGGRPSSSGGYRQGGRGGGSGGGWQGGRGG
GSGQGGRGRGGAGGRSGRGPKPRSAFELYG
>Chlorella_sor_PRW58644.1_blue-light
MPAPPGPTSMEVEGSLRPHAALPEVPLPEGGLRRVIVWFRRDLRLADNPALVAALRMAPEVLPVYIWVPEEEG
QFQPGRCSRWWLAASLKALDADLRALGSRLLAYRSPDSRALLCRLARELGAGAVLFNHLYDPISMVRDNEVKA
GLAAAGVHCQSFNAEILREPWEVLAPGGKPFTCFDGFWGAHCGLPYPPPPPLPLPAALPPLPRGIEATELAQL
GIMTPEEEMSNAQLEFHWQPGSAGAHRLLDDFVRGGRLKAFDHDRAKTDRQSTSRLSPHVHYGEISARHIYYV
AKHAELEWARGGGGGGTSVADFLRQLGYREYSRYLSFHFPFTHERSLLEHLRAVPWRFDQRLFKAWRQGRTGY
PLVDAAMRELWGTGWMHNRMRVVAASFLVKNLLLPWQWGLKHYWDALLDADLESDALGWQYVAGCMADAHEFS
YMIDQPTEAKRFDPDGRYVRRWLPVLARLPAKWIHQPWLAPESVLADAGVELGVNYPWPVMEVEESAAALKAA
DAVVQACLVRGGEAGAGGEVAAPPPAGPFRPATDAEPAEAERLFMENYRHSVMAGSALPPIVDRAEGLGESED
VSSNVVLGANAAAARAAFSSAPAATTITGALSAAPAAARQAAAAAAAAAQQQQQAQTQEVQQPQRVPEQSQQV
AQQPQQAQQAIVSMAGWRQLQTTPIGGSLPAAGSGPPGSGDGTGHATATGLHSTGVGVVPSLPLSGGGESPQG
QVPQGMRPPLPHHAAQQVVEAAAHKQALLRRAAANEGQQGSNSPASGRMDEGEQQAQWPPELTLEPQQGHKRQ
RQASPPPPG
>Chlorella_sor_PRW58875.1_UVR3_3-lik
MRFATLALAALLLAACVPRPAHAASGDASYIGEVFMMATSFCPTGSVEANGATVAAASNPSLYALLGTTYGGT
STNFKLPNIKPQGSSALAAEVGGGSGGAMGATSIIWFRKGLRIHDNPALLAAAQGADHLCPLFILDPWFLQPD
KIGVNRVQFLLESLADLDASFRARGSRLLVLRGKPQEVLPPVFKEWGVTRLCFESDTEPYAKQRDAAVRQLAE
AAGVEVHSSVSHTLYDPQLLLARNGGKPPLTMQAFTKLADAVGPPPAPAADPPEKLPPLAPQAASLDAAASGV
PSLADIGYPPAGTTPFKGGETAALARMADYLSDKDWVCAFEKPKGNPALFLRPATTVLSPYLKFGCLSPRLFH
AKLQQIYSERSGKHTQPPVSLRGQLLWREFFTLCGSAVPNFDRMEGNPICRQIPWADDPARIQAWEESRTGYP
WIDAIMAQLRRQGWMHHLARHSVACFLTRGDLWCSWEAGQAVFDKYLIDADWSLNAANWQWLSASAFFSQYFR

VYSPISFGKQYDKNGDYIRHFLPALKDMPAKYIYEPWTAPLSVQQAAGCIIGKDYPRPIVDHAVVHKENMAKM
KAAYDAGKAAAGAAAGAGGAAAGSAAAAAAGGDGASSSGSKAGAKPAKRARKS
>Chlorella_sor_PRW61485.1_CPD_photol
MARVYSKAYRSDEPAMTSLARVYADANVNNPKEYWDYENLQIDWGSQDDYEVVRKVGRGKYSEVFEGVNVRNN
EKCIIKILKPVKKKKIKREIKILQNLYGGPNVIKLLDVVRDPQSKTPSLVFEYVNNTDFKVLYPTLSDYDIRY
YVMEILKALDFCHSKGIMHRDVKPHNIMIDHSQRKLRLIDWGLAEFYHPGREYNVRVASRYFKGPELLVDLQD
YDYSLDMWSLGCMLAGMIFRKEPFFYGHDNYDQLVKICKVLGTDSFYSYLNKYGLELDPQLEALVGRHSRKPW
TKFVNAENQHLVSAEAIDFIDKLLRYDHQERLTAKEAMAHPYLAPALAVHRPWAAAPAPEALRRGLQQRTQRR
SLAAAATPAAAAAMGADDKRKAEEGLEGGPAKKVAAAKLAVHPKRVRELRKGAVEGSGPVIYWMSRDQRVRDN
WALLYAAEAAAKRGVPVAVAFNLVTEFLGAGARQFGFMVRGLQELQPKLEALNIPFFLLKGDPTETLPQLVQD
TGASLLVTDFTPLRLGRQWKEGVAAKVEVPFHEVDAHNIVPVWVASDKREYAARTIRPKIHSKLPEFLQEFPE
LPEQPAWTGAKPEPVDWEALLQEVLERGKDVPEVAWCKPGEDAALEALKGEKGFLTKTRLSRYDEKRNDPTVP
DALSGLSPYLHFGQLAPQRAAIEAAKCKSMHKASVEGFLEELIVRRELSDNYCFYVDNYDSLGAAYDWARQTL
EAHRSDKREHLYTKEQFERGQTHDKLWNAAQLEMVHSGKMHGFMRMYWAKKILEWTASPEEALEVAIWLNDKS
LEVAIWLNDKYQLDGRDPNGYVGCMWSIAGIHDQGWAERAVFGKIRYMNYNGCKRKFDIDKYVARIGAMVRDV
KAAAAKQ
>Chlorocebus_saba__XP_007998737.1
MAATVATAAAVAPAPAPGTDGASSVHWFRKGLRLHDNPALLAAVRGARCVRCVYILDPWFAASSSVGINRWRF
LLQSLEDLDTSLRKLNSRLFVVRGQPADVFPRLFKEWGVTRLTFEYDSEPFGKERDAAIMKMAKEAGVEVVTE
NSHTLYDLERIIELNGQKPPLTYKRFQAIISRMELPKKPVGLVTSQQMESCRAEIQENHDETYGVPSLEELGF
PTEGLGPAVWQGGETEALARLDKHLERKAWVANYERPRMNANSLLASPTGLSPYLRFGCLSCRLFYYRLWDLY
KKVKRNSTPPLSLFGQLLWREFFYTAATNNPRFDRMEGNPICIQIPWDRNPEALAKWAEGKTGFPWIDAIMTQ
LRQEGWIHHLARHAVACFLTRGDLWVSWESGVRVFDELLLDADFSVNAGSWMWLSCSAFFQQFFHCYCPVGFG
RRTDPSGDYIRRYLPKLKGFPSRYIYEPWNAPESIQKAAKCIIGVDYPRPIVNHAETSRLNIERMKQIYQQLS
RYRGLCLLASVPSCVEDLSHPVAEPSSSQAGSVNSAGPRPLPSGPASPKRKLEAAEEPPGEELSKRAKVAELP
TPELPSKDA
>Chlorocebus_saba__XP_008002745.1
MGVNAVHWFRKGLRLHDNPALKECIQGADTIRCVYILDPWFAGSSNVGINRWRFLLQCLEDLDANLRKLNSRL
FVIRGQPADVFPRLFKEWNITKLSIEYDSEPFGKERDAAIKKLATEAGVEVIVRISHTLYDLDKIIELNGGQP
PLTYKRFQTLISKMEPLEIPVETITSEVIEKCTTPLSDDHDEKYGVPSLEELGFDTDGLSSAVWPGGETEALT
RLERHLERKAWVANFERPRMNANSLLASPTGLSPYLRFGCLSCRLFYFKLTDLYKKVKKNSSPPLSLYGQLLW
REFFYTAATNNPRFDKMEGNPICVQIPWDKNPEALAKWAEGRTGFPWIDAIMTQLRQEGWIHHLARHAVACFL
TRGDLWISWEEGMKVFEELLLDADWSINAGSWMWLSCSSFFQQFFHCYCPVGFGRRTDPNGDYIRRYLPVLRG
FPAKYIYDPWNAPEGIQKVAKCLIGINYPKPMVNHAEASRLNIERMKQIYQQLSRYRGLGLLASVPSNPNGNG
GFMGYSAENIPGCSSSGSCSQGSGILHYTHGDSQQTHLLKQGRSSMGTGLSGGKRPSQEEDTQSIGPKVQRQS
TN
>Chloroceryle_aene__NXI52898.1
MSHRTIHLFRKGLRLHDNPALLAALESSEVVYPVYILDRKFLTSVMHVGPLRWHFLLQSLEDLQKNLGQLGSC
LLVLQGEYEALLRDHVQEWNITQVTLDAEMEPFYKEMEATIRRLGEELGFEVLSLVGHSLYSTDRILELNGGT
PPLTYKKFLHILSLLGDPELPVRNLTAEDFQRCGAPDPGLAEQYRVPLPVDLKLPQENLSPWRGGETEGLQRL
EKHLTDQGWVTSFTKPRTVPNSLLPSTTGLSPYFSVGCLSVRTFFYRLSNIYAQAKHHSLPPVSLQGQLLWRE
FFYTVASATPNFTQMVGNPICLQIRWYEDAERLHRWKMAQTGFPWIDAIMTQLRQEGWIHHLARHAVACFLTR
GDLWISWEEGMKVHLGFISFVLGDRGLVEQVQQSATKMLRGLEPLSWGDRLRELGLKYLPVLKNFASKYIYEP
WTASEEEQKHAGCIIGNKRRVMFWCAFCPHPHRPSRGHRAGVREGPRNFNSSSPRAADDTDDPMELKLKRHRS
QENISKGKLARTTK
>Chloroceryle_aene__NXI53963.1
RFLLQCLEDLDANLRKLNSRLFVIRGQPADVFPRLFKEWNIAKLSIEYDSEPFGKERDAAIKKLASEAGVEVI
VRISHTLYDLDKIIELNGGQPPLTYKRFQTLISRMEPLEMPVETITPEVMEKCTTPVSDDHDEKYGVPSLEEL
GFDTDGLPSAVWPGGETEALTRLERHLERKAWVANFERPRMNANSLLASPTGLSPYLRFGCLSCRLFYFKLTD
LYKKVKKNSSPPLSLYGQLLWREFFYTAATNNPRFDKMEGNPICVQIPWDKNPEALAKWAEGRTGFPWIDAIM
TQLRQEGWIHHLARHAVACFLTRGDLWISWEEGMKVFEELLLDADWSVNAGSWMWLSCSSFFQQFFHCYCPVG
FGRRTDPNGDYIRRYLPVLRGFPAKYIYDPWNAPESVQKAAKCIIGVNYPKPMVNHAEASRLNIERMKQIYQQ
LSRYRGLGLLATVPSNPNGNGNGGLMGYSPGESISGCGSTGGAQLGTGDGHTVVQPCALGDSHTGASGVQQQG
YCQASSILHYAHGDNQQSHLLQAGRTALGTGVSAGKRPNPEEETQSVGPKVQRQSTN
>Chloroceryle_aene__NXI54913.1
RFLLQSLEDLDNSLRKLNSRLFVVRGQPTDVFPRLFKEWGVTRLTFEYDSEPFGKERDAAIIKLAKEAGVEVV
IENSHTLYDLDRIIELNGHKPPLTYKRFQAIISRMELPKKPVSTVIRQQMETCKVDIQENHDDVYGVPSLEEL
GFPTDGLAPAVWQGGETEALARLDKHLERKAWVANYERPRMNANSLLASPTGLSPYLRFGCLSCRLFYYRLWE
LYKKVKRNSTPPLSLYGQLLWREFFYTAATNNPKFDRMEGNPICIQIPWDRNPEALAKWAEGKTGFPWIDAIM
TQLRQEGWIHHLARHAVACFLTRGDLWISWESGVRVFDELLLDADFSVNAGSWMWLSCSAFFQQFFHCYCPVG

FGRRTDPSGDYVKRYLPKLKGFPSRYIYEPWNAPESVQKAAKCIIGVDYPKPMVNHAETSRLNIERMKQIYQQ
LSRYRGLCLLASVPSCVEDLSGPVTDSASGQGCSTGTAMRLSQADQASPKRKHEGAEELCTEELYKRAKVTGL
PAAEIPGKN
>Chloroceryle_aene__NXI55221.1
DNWAFLYAQRLALKQELPLHVCFCLVPKFLDATIRHYGFMLKGLQEVAEECAELNIPFHLLLGYAKDVLPAFV
VEHGVGGLVTDFCPLRLPRQWVEDIRERLPEDVPFAQVDAHNIVPCWVTSPKQEYSARTIRGKIHAQLPEFLT
EFPPVIHHPYSPSCPAEPIAWEACYSSLQVDLTVKEVEWATPGTAAGLAVLQSFIAERLKSFSFHRNDPNKAA
LSNLSPWFHFGQVSTQRAILEVQKHRGKYKESVDAFVEEAVVRRELAENFCYYNENYDSVQGAYDWAQTTLKV
HAKDKRPFLYNLQELEQGTTHDPLWNAAQLQMVHEGKMHGFLRMYWAKKILEWTRSPEEALRFAVYLNDRYEL
DGSDPNGYVGKRCLWSICGVHDQGWAERAIFGKIRYMNYAGCKRKFDVDQFEHRYAP
>Chloroceryle_aene__NXI57620.1
KVDYDVCSNYGNWLYSAGVGNDPRDNRKFNMIKQGLDYDGDGDYVRLWIPELRGIKGADIHTPWALNSAALSQ
AGVTLGETYPQPVVTAPEWSRHISQRPQGRSPRPRDRRGPAHPAAQHKDRGIDFYFSHKKDA
>Chloropicon_pri_QDZ19648.1_cry/DNA_ph
MVAVKKEGGPLRKRSLVWFRKGLRVHDNPALLEASQSAAEVYPVFVLDPSIDPTEDGVKIGANRAQFLLESLE
DLDQSLKKLGSHLICIRGRPEDVLPPLMRELDINHLGFEFDTEPYWKAQGEKMAKEAENLGARVHCPATHTLY
DPEHLMAKCMMDAPKSYGQFLKLVAKCGSPPAVASAPESLPQISKDVLGRKHSGVPTLRDLGYEPLPASERTP
FPGGETEALRRLEDQFRDTRRIALFEKPKTSPTDFETPSTTVLSPYLKFGCLSSRVFYFKLKEAYRKNGKHAD
PPVSLLGQLYWREFFYLCGYAFPNFGKMEGNPICRQIPWDHSEGGKEKLRAWESAQTGYPFIDAAMTQLRQQG
WLHHLARHAVACFLTRGDLWVSWEEGRKVFDKLLIDADWSLNNANWMWLSCSSFFYQYFRCYSPVAFGRKYDP
SGKYIRNFVPALRKMPKQYIYEPWKAPLSVQEAAGCIVGVDYPMPVCDHAVASKENMSKMAAAYKKAKSESPP
PKKKAKKAK
>Chloropicon_pri_QDZ19871.1
MLRSEAKEAKGLLDGGRRRGGVLVWFRRDLRLHDNETIYAAAMRARETNTLVNFCYIHRDVASSREPWHRRQG
GASLRALDDHLIETFGEGGKVIFRECEDEHDGVVSALAEVAEACGSSEIFFGRCFEEKLVAEEKHAQMQLSRL
HGLRSKAFSTFLLHDPEAVAIDMRRWKGHFGTLTPFHFACKQQRKELKTFPVPASMRIGEASVALEKAWTLEN
DSNGFEVFLSTGVRDGDDAALSSSESSWVDKLVQNWFDGSTECFGEVHALKLLDEFIQHKMNKYESSRGYADA
RAVSKLSPYLHFGVLSPAHMANRLEKSGCEAASKTLWRRLVWRDLAYWQLHHWPRMPTQPIRRFYEEHEWRED
AEALERWKRGRTGFPLVDAGMRELWHTGWMQQNARMCCALFLTEYLNVHWARGAEWFHETLFDADLAINSMMW
QNAGKSGLDQWNFTVGPTAKSLDPSGDYVRRWVPELKRLPKPFLHAPWEATEAVLVEAGLSLGENYPHRISAV
ADVASAKNRNLQAVLKLREKAKRSGFVDENGYDLIKAPRGSCRGSNNGKVRVFTKPEYRRNGQKKTKGGQNQK
RGKKRPKVGKETKVVLRQLSLEEALRMDH
>Chloropicon_pri_QDZ22001.1_cry
MTKMANGNGLKSSSKRLVLWFRNDLRLHDNAIVDKAKRSVASGDCKEVLPLYCFDPRVFQRSEYGSPKTGVHR
AKFLVESVMDLRERLRSVGSDLLIAVNKPENVIADLVGDNGTSVFAQREVTHEERVTESLVEGILAGSERARL
ELFWGATLYHRGDLPFKSNLRDLPDVFTPFRNKVEKQCSVRSLLSEPRAGDLPLGDAANLPLVKKMTNYEPSL
KDILPGVDVPDQDERSSLSFRGGEAAALERLRYYLWDSDLLSTYFETRNGMVGGDYSTKFSPWLANGCLSPRK
IYHEISEYERERVANKSTYWVVFELIWRDFFHFYCEKHGNSVFYPGGPAGRRNDWNNDRDALRRWKEGRTGVP
LVDANMRELLHTGFMSNRGRQNVASFLALDLKGDWRVGADHFESLLLDYDVCSNWGNWVSAAGLTGGRVNRFN
IVKQSKDYDKAGEYIKLWIPELRKVPTQYIHKPWEMPRSVQESSGCFIGQDYPNPVATSKAWETYNKGGKKSE
KSKPYRGYYEKRSVRKEKKKSNFEMYG
>Chloropicon_pri_QDZ22673.1_photolyase
MTETMSAESKPTREVHKSRWRELNVAGAENHKASGPVVYLCYRDQRARDNWALLHALHVAETKGKAVVVLFVL
TESQLRLGHRHSLFMIKGLQEMQKHLAALNVPFHLVLGEPAAVVADVIAAWDAAALVTDFSPLREARQWRDSV
AGSIQIPFHEVDAHNIVPIWITSNKQEYAARTIRSKIHRNIPQYLRAIPKPRKQDFDFSNTKGSFGEPNAVAW
DDLVALAREKGQSDVPEVSWCEPGEEAALRALKGEDNSFLTKMRMNKYDELRNNPNKPAALSNLSPYLHFGQL
SAQRCAIEAIGFKKNNPSSNKSVDNYLEELVVRRELSDNFCFYNDLYDSVKGAASWAQETLQIHSSDKREHLY
DKEALEQARTHDDLWNAAQKELTSFGKMHGFMRMYWAKKILEWTASPEEALDIAIYLNDKYSLDGRDPNGYVG
CMWSICGTHDQGWREREVFGKIRYMNYNGCKRKFDVDAYVQRIRREMKQNRYFNEK
>Chloropicon_pri_QDZ25668.1_cry/DNA_ph
MATERDERAAKRLATESAEPLPQAVGPSSVTQAGKLSRVVVWFRRDLRLEDNPALMAALRAGREVIPLFIWAP
EEEGVFTPGLSSRWWLTQSLESLSRKLEALGSRLVLFKTPTTKEGIVPFVKDNDIQAVYFNHLYDPISLVRDK
GIRTLLQGLNVHVESFGADLMYEPWEIYDEKGQVFNTFDKFWEHIVNKMPYEPFMPLPCPVAMPKVPDEVKGN
RASLDEVAIMTEEEAATSEHLKEKWMPGSDSAHMVWNKFLCEKLAFFKQEKAMVHSDCTSRLSPHMHYGEISV
SSLYFVLKKMKLSLSTALDSEQQIASVDKYIKQLGYREFSRYILFHNPFTHERPLLEHLCAVPWQYDQDLFKC
WQQGCTGFPLIDAGMQELWSTGWCHNRLRILVGHFFVKYLNLPWQWGLKYFWDTLIDADLECDALGWQYLSGC
MIDAPPFSYIMDYEEECKRFDPDGKYVKKWLPLLARVPSKFIHTPWKAPAKILRASGVDMGITYPHRVVNMDQ
VRKSVKHCCECLEAQKCGPQRGPFRHPTRSDPSRPSSMRTGTADEGMDGPYLPGKTKPEKADSVGLGGVQSHI
SETDGCHNESTGVVES
>Choloepus_dida__XP_037694977.1

MAATAVMAGSASLAPASGTEGASSVHWFRKGLRLHDNPALLAAVRGARCVRCVYILDPWFAASSSVGINRWRF
LLQSLEDLDTSLRKLNSRLFVVRGQPADVFPRLFKEWGVTRLTFEYDSEPFGKERDAAIMKMAKEAGVEVVTE
NSHTLYDLDRIIELNGQKPPLTYKRFQAIISRMELPKKPVGSVTSQQMESCRAEIQENHDETYGVPSLEELGF
PTEGLGPAVWQGGEAEALARLDKHLERKAWVANYERPRMNANSLLASPTGLSPYLRFGCLSCRLFYYRLWDLY
RKVKRNSTPPLSLFGQLLWREFFYTAATNNPRFDRMEGNPICIQIPWDRNPEALAKWAEGKTGFPWIDAIMTQ
LRQEGWIHHLARHAVACFLTRGDLWVSWESGVRVFDELLLDADFSVNSGSWMWLSCSAFFQQFFHCYCPVGFG
RRTDPSGDYIRRYLPKLKGFPSRYIYEPWNAPESIQKAAKCIIGVDYPRPIVNHAETSRLNIERMKQIYQQLS
RYRGLCLLASVPSCVEDLSNPVAEPSSSQAGSMSSAGPRPVPSGPASPKRKLEAAEEPPGEELSKRARVTELP
TPELPSKGV
>Choloepus_dida__XP_037702498.1
MGVNAVHWFRKGLRLHDNPALKECIQGADTIRCVYILDPWFAGSSNVGINRWRFLLQCLEDLDASLRKLNSRL
FVIRGQPADVFPRLFKEWNITKLSIEYDSEPFGKERDAAIKKLATEAGVEVIVRISHTLYDLDKIIELNGGQP
PLTYKRFQTLISKMEPLEIPVETITPEMIEKCATPLSDDHDEKYGVPSLEELGFDTDGLPSAVWPGGETEALT
RLERHLERKAWVANFERPRMNANSLLASPTGLSPYLRFGCLSCRLFYFKLTDLYKKIPWDKNPEALAKWAEGR
TGFPWIDAIMTQLRQEGWIHHLARHAVACFLTRGDLWISWEEGMKVFEELLLDADWSINAGSWMWLSCSSFFQ
QFFHCYCPVGFGRRTDPNGDYIRRYLPVLRGFPAKYIYDPWNAPEGIQKVAKCLIGVNYPKPMVNHAEASRLN
IERMKQIYQQLSRYRGLGLLASVPSNPNGNGGLMGYSPGENIPGCSSSGSCSQGSGILHYAHGDSQQTHLLKQ
GRSSMGIGLSSGKRPSQEEETQGIGPKVQRQSTN
>Chondrus_cri_XP_005711398.1_unnamed_pr
MPAMLLAFASGLPLNQKFLSTFFSPRNGKCQLFVQMPRTFANRNAEANNSRVGQSYTRSSSGRRGNRGGRGRY
GGNRRNAAPRAQVDPFAGDPVIPSPAPATLPSDSCAMVWLRNDLRVRDHAALALADTASVLVPFYVFDTSQYG
PDHLSPHGFQRTGPFRADFQKQAVKEMQQALRVRGSDLVVKIGDPVDEIMPVARQLIEEGFKPLRMVAHKEVT
WEENKTEERMEKAFRELGEEMHADVDIHFVWGATLHHLDDLPFNAGGPGVPETFTAYRKLIEGRNGATVREEI
KMPGRFKPYPLHIGIPSDEMPLLGDELEVAGLAEPNDHPYPHPLACVDFIGGLKEAEKRLDEFVWKADALRTY
KETRNESGTKNGSSKLSPWLALGCISPRTVYWECQSYEEKREKNESTYWMVFELMTRDYFRWIAASVGTKLFA
LNGYSGRGKNEASIWGIPWHLIKPLHKDRLQKWIDGKTGAPIVDGNMRELAGTGFMSNRGRQNVASFLIHDLG
YPDWRAGAEYLESQLIDHDVASNWANWAYLAGVGSDPRGGRKFNVVKQSMQYDPEGRFLNRWVPELVDIPVPM
KHEPHTLSKAELEDLNVTPGKTYPNPIVPLSRAPQK
>Chondrus_cri_XP_005711823.1_unnamed_pr
MSQANAARMHNPSPSPSALTQAPITNASRRIRRRAASPPAKRRRTASSTEDADSVSPPASPSPELRQPPSQAT
APQGAKWFRKPSVVWYRGNDLRVEDHPALLAASQRGGPVVPLFIWDPSDSFGSDLGHVKQWWLRESLSCLQRD
LKRLGVQLYTRTGKSTEELRNFLCDTGADAVFWNRCYEPDLLTRDEELRDELLSEGLTAESFKAELLVEPWEL
KNTEVNPRYETFRSYMRAWMTFAPPPEPFPCPSRLTAITSEVKSVGISALAFDIPHTLEEVLQKTWTPGSVQA
NAQLEKFLIEVFPAFGDNRCRRHFDGTSRLSPHIRFGELSPRRMYHSTRMRVSRWDQGSMLSLAASRKWSLAM
SNGGTKQEWKRASLLESLESGREVNREREPQGDTRSSPLTSGTHAKQEESVGDKGGRGRPQPTPPEKPPKTNF
RHMMRSAAAVQRPALPPISQSARAFLRNLCLRDFSYHILFHNPDFHAKPLIPEYVNFPWAEDKGSFTAWQTAK
TGYPIIDAAMRELRATGWIHNRMRFLLACFLTKYLLLPWSRGLKEFYGLLLDGDHSSNALGWQWTAGSNTYAA
TLRNLLNPVKSASQHDPHGSYVRQWLPELANLPTNFVHEPWKAPPDVLHAAGVELGTTYPNRIVMLKEARVRA
FEAMRLMRQIFSASGVSRKLEVEDEEDLVMGWREDHSEVFPVEESAVTGTKRTLLPCLWALLQSDQEPTYLSN
SSSGSDPLIARDTASLTEGALAVPMDDQQESIENALLTAHNHGPPPEVLVIPLEAVTADDQALGDPIYPQEKN
CETEGMMVGPGSDNLPVGHLGEPLKYHDSEVCAFPESENVSDRQAPMEADGSRVRTDSARARTRPPDTVQVGA
PNTCRGSAAQALNPSVPFPHQPQYQLQQFQQLQQLHKFQLQNHMHVHQQQQRQETQQHQRAAQQQRTSQHTSP
HQHIHYPNSALPQRQQPHVHAMADAIYGQQGCSNGSLPSTSLAGVPMPGAPVMMGPATGSQDLSANAMFTGGD
RGSSVYAHPLGFGQFYGFPPVMPILNAPAANGGERNDGILSSATTDMSKLPAPGMMAMPYGMYPGNMFEQNVL
GGAHQAGNNHYASSLPQMQDGRQSPYRYSPQMYFPHTTPEISPAQGNGHVPHRPSPSIPPQSRHLDMPSVANA
PNATAAAAVAGREREAAQVASSNANTRTLPKSEQLTDVARNTPGLKPGFGVASRSVDTTKPLNSKVTNANTRL
GQGRPRGRNNARSKAALKRKGSASNGIAKNRNEVKSSSNGRGQGSEGRSSTAQEKGPKEDTQPQGTTLKARQE
MLASVLGKEDHQFHGFAKYLSSTYELTASTDRKTSKDYIRLCNLKDDYHKQCKSDKLKIYGIKSFFSKVLNLD
VTGEWDRHNHGGVRGPYVYGIRPRRSTNPS
>Chondrus_cri_XP_005712174.1_unnamed_pr
MPTAFLFPTTRVFFGKSVQNLREYRAIATSTHCLDVHPANYLAAPRRFVATMSSLSSPVERERVRTLNDPHAN
TKGSHVVYWMNNAVRVRHNPALEEAAHMAASMEQALRVIYLMDVTAQDSQPLPERHTSFLLQGLADVAASLRQ
RKVPFVVLSPEAEADVAIPAMAKDASAVVTDASYLRPGIATRKRAARALQVPLIVVEADVVVPVETATNKAEH
AARTIRPKINRLRDDFLEPLEQVALPYQEQGDGKWDVADDWLEDGRKTEDRIDHVDVKKHDDVLASLQGLDRG
APLVSSFKGGEKEAQEVLKTFLKERLREYGPGRNEPSKQLQSDLSPYLRAGNISPVDIALQTLDFAKDKKSGM
KESLDSFLEELIVRRELAANACWFNPDKYDAYEHIVPSFARASLELHKADKRPTTYTYEELEAGLTHDDYWNA
AQLEMVVRGKQHGYMRMYWVKQIIGWVDDPAVAMDYGLRLNNRWELDAVDPNSYAGVIWCFGLHDQGWKERPI
WGKVRYMNDAGLKRKFNMAAYVAMVDRLIAEQGLPAHIAALRKARGADRRQKTIDQTMKRKRKTSAAPSNKAA
KESLLRSIKRLKE
>Chondrus_cri_XP_005718107.1_cry/photol

MTLPGSVLHWFRRDLRTADNRALNRAISLAKKSNLPLIPVFFLDPEYHNPTACGPLRMRFILESISALQRSLD
DKCSSRLRVICARPTDVFPDLVRRWGVKHLVFEYEYAPSERARDEAVVADVEGFDVDVVREHGFTLYNPNVLL
QKAKGATAPTTMSSFLSLVDDVGDPENPVEFADGRQVELTSCPDATLKDDDKDGYLLVPKLGDIGYEDCTKQT
GWERFRGGEEEGIARMDEFLSREGGKVAARFAKPKTSPAAFVKRETTVLSPYLALGCVSSRVFHARLRDLEEN
HDVAEMPQTRLWGQLMWREHFWLLAGMVPEFHRMNGNPVCRKINWRTGDEAELRLKTWAEARTGYPWIDALMM
QLKTEGFVHHLGRHSLACFLTRGDLWVSWEEGYKVFEHYLVDYDYALNSANWMWLSCSAFFSMYFRVYSPVAF
AKKWDKEGAFIKHYLPVLRKLPTKYIHEPWKAPAMVQKMAGCVIGKDYPERMVDHSKASKENIAKMKENYARN
EYGAERGERQHMNGESRSSESRDSGDGTSRKTKRRKTRR
>Chrysochloris_asia__XP_006865104.1
MAAARGTVTTAALESAPSTDGASSVHWFRKGLRLHDNPALQAAVRGARCVRCVYILDPWFAASSSVGINRWRF
LLQSLEDLDTSLRTLNSRLFVVRGQPADVFPRLFKEWGVTRLTFEYDSEPFGKERDAAIMKLAREAGVEVVTE
NSHTLYDLDRIIELNGQKPPLTYKRFQAIVSRMELPRKPVGPVSSQQMESCGAEIQENHDETYGVPSLEELGF
PTEGLGPAVWQGGETEALARLDKHLERKAWVANYERPRMNANSLLASPTGLSPYLRFGCLSCRLFYYRLWDLY
KKVKRNSTPPLSLFGQLLWREFFYTAATNNPRFDRMEGNPICIQIPWDRNPEALAKWAEGKTGFPWIDAIMTQ
LRQEGWIHHLARHAVACFLTRGDLWVSWESGVRVFDELLLDADFSVNAGSWMWLSCSAFFQQFFHCYCPVGFG
RRTDPSGDYIRRYLPKLKGFPARYIYEPWNAPEPIQKAAKCIIGVDYPRPIVSHAETSRLNIERMKQIYQQLS
RYQGLCLLASVPSCVEDLSNPVAEPSPSQAGVLSSAGPRLLPSGPASPKRKLEAAEEPPGEELSKRARVAEEP
SLKQLSKDV
>Chrysochloris_asia__XP_006865309.1
MGVNAVHWFRKGLRLHDNPALQECIQGADTVRCVYILDPWFAGSSNVGINRWRFLLQCLEDLDANLRKLNSRL
FVIRGQPADVFPRLFKEWDITKLSIEYDSEPFGKERDAAIRKLATEAGVEVIVRISHTLYDLDKIIELNGGQP
PLTYKRFQTLISKMEPLETPVETITSEVMGKCVTPLSDDHDEKYGVPSLEELGFDTDGLSLAVWPGGETEALT
RLERHLERKAWVANFERPRMNANSLLASPTGLSPYLRFGCLSCRLFYFKLTDLYKKVKKNSSPPLSLYGQLLW
REFFYTAATNNPRFDKMEGNPICVQIPWDKNPEALAKWAEGRTGFPWIDAIMAQLRQEGWIHHLARHAVACFL
TRGDLWITWEEGMKVFEELLLDADWSINAGSWMWLSCSSFFQQFFHCYCPVGFGRRTDPNGDYIRRYLPVLRG
FPAKYIYDPWNAPEGIQKVAKCLIGVNYPKPMVNHAEASRLNIERMKQIYQQLSRYRGLGLLASVPSNPNGNG
GLMGYSPGENIPGCSSSGSSSQGSGILHCAHGDSQQPHLLKQGRSPMRPGLSSGKRPSQEEETQGVGSKVQRQ
STN
>Chrysochromulina_KOO31888.1
MTQLWHVGWMPNYMRHVVAGFLVEFLNFDWRHGERWFAETLVDADTAINAYMWQNGGHSGMDQWNFVMHPVFA
AKSCDPEGDYVRRWLPQLAKLPIEFIHCPWEAPAALRATAKVVLGNGRGANYAQRILVDLEAARRRSFAAVME
VRRGAGKDYILPSGHEAMALDNGQRAVLITRVDFREGKLTTRQTAESKWDERRRERTDDLSRAMQDSMREHSA
ANSLDGGLRLAEEEQL
>Chrysochromulina_tob_KOO26945.1_photolyase
MLRGLQEVERDLRDLGIPFKVLSGNPVDEVPSFLRRHEGCALVTDFSPLRTGRAWKSAVGEKLSSTPFYEVDA
HNIVPVWVASDKQEVGARTIRKKINEKLPMWLKDFPPLEPQPIEPPLHAQAVAAPLPAEDTSGIISPVPPAPS
STSDRTSVLDEGTLMALSAQLEAAVVASPLDLATAERVLGQLEGVRIDRALLKATGVGKAANELAKAKCDLST
RARPSGSARSLALPRHPSASLLGECVAPTDWTALEKSLVIDRNVKQVDWLLPGPAAAREAVHAFCASRLALFA
DKRNDPNVHAHSDLSPYLHFGQLSAQRMALMVQAAVAAKPQAALQAGLESFLEESIVRRELSDNFCFYNPNYD
NLNGAAGWARESLELHAADKREHVYTLKQLEEAQTHEDIWNAAQRQMARTGKMHGFMRMYWAQEDSPALIASD
>Chrysochromulina_tob_KOO31536.1_6-4_photol
MARERGVQVVTRWGHTLTDLDALLKRHPGGKPTTTYSSFLNHLDKQLKAVPLAVLNKPRQLPPVVDADLAGLG
ASAATSASSSAAPASDAADAFGVPSAAALCLGPATSGVILRGGETEALARMEACLSQQGGAWVAAFEKPSTSP
TEIDPFGQHTRSTTCLSPYLKFGCLSARVLHERVAAVYAAHPKHAKPPTSLHGQVYWREFYYACAHGTPNYEM
MAANPICRQIPWEDDPELLAAWREARTGYPWIDAAMTQLREEGWIHHLARHAVACFLTRGDLWQSWEKGAEVF
EELLLDADPAINRGNWMWLSCSCFFYQYFRCYSPVAFPKKYDKDGAYVRKWLPQLKDFPAKYIYEPWKAPIAD
QKKANCIIGQDYPKPIVNHELKRALTQQKLPMSKKVLPRVEP
>Chrysochromulina_tob_KOO53579.1_photolyase
MSVAAAATLTAATAALTSSRPRFVVWFRSDLRMHDNPVLHKIAALPGAKEIVPVYCFDPRHFAKDAPHSFGCA
KTGVLRAKFMCESVADLRSSLRACGSDLLVAVGKPEELLPTLVQPASAGPTTFVTQQQLTSEELRVDRAVVSA
LKKAHGSASAPALRTIFSGTMYDDENLKTMFGDDYASMPDVFTPFRTKVEAKLVIPPPLASPKPGSLPLPANA
GAVATAAAAAAVGGAGFDSMPSLSALGFSSEEVTSAATPDPRGAMPFPGGERAALARLQHYIWDADCLGTYFE
TRNGMLGADYSSKFAPWLAHGCLSPRLVAAECAKYEKQRVKNKSTYWLVFELLWRDFFRLYAAKHGDAIFHIE
GPARVRSKWVSNPELLQRWKEGRLGVPLVDANMRELAATGFMSNRGRQNVASYLALDLALDWRTGASWFEETL
LDYDVASNWGNWASAAGVTGGRVNRFNIVKQSKDYDADGAYVRHWLPELKEVPAAKIHEPWTLSRDEQQRLRG
VFLKPRTTLSQRPMRGVPMRTAV
>Chrysoperla_carn__XP_044730763.1
MNSLFMTKINIKNSAAFCSVEYYEGTWRAMSSGVGSAGDIRKTGGPPPPDKHMVHWFRKGLRLHDNPSLREGL
VGATTFRCVFILDPWFAGSSNVGINKWRFLLQCLEDLDRSLRKLNSRLFVIRGQPADALPKLFKEWGTTSLTF
EEDPEPYGRVRDENITALCNELGISVVQRVSHTLYKLHQIIENNGGQAPLTYHQFQAVIANMDAPSAAEGGIT

QKNIGNATTPLTDDHDDKYGVPTLEELGFETEGLLPPVWQGGESEALARLERHLERKAWVASFGRPKMTPQSL
LPSQTGLSPYLRFGCLSTRLFYYQLTDLYKKIKKACPPLSLHGQLLWREFFYCAATKNANFDKMIGNPICVQI
PWDKNAEALAKWANGQTGFPWIDAIMTQLREEGWIHHLARHAVACFLTRGDLWLSWEEGMKVFEELLLDADWS
VNAGMWMWLSCSSFFQQFFHCYCPVRFGRKADPNGDYIRRYLPVLKNFPTRYIHEPWVAPESVQRAAKCIIGK
NYPLPMVNHAVASRINIQRMKQVYQQLARYRNDDAEKKNLINVANQRQNMVAMSPSPNTVMSSINNSYNLAVS
QKQSSIPNNKTESAYNIASEQYNTDYNQQQNQQTSQHQQFYTIVQTNHSINTFTESLNNQQEQFKQQHHHQQE
QQQQSNQEQYENTRQTYCEPMKQSPINTQHNYDYMRENRNNFCNEPIKKSNLHNFCEPMKHSPMVQIEQESYK
QNYQVTVQDNDGNTSVVTIVQQQQQEQLIGNCSRTNTINDDDSSMTINIKREEQRQ
>Chrysoperla_carn__XP_044734495.1
MESSALWFRHGLRLHDNPSLHEAIKDRDRLFYPIFIFDGESAGTKLCGYNRMKFLTECLEDLDKQFRKYGGQL
FVFENKPEVVFKALNKHYNLKLLCFEQDCEPVWRVRDENVKRLCDKRGVKWMECVSHTLWDPFRVIEENGGTP
PLTYQAFLYVVSNIGNPPRPVDDVNWQGIKFGIISPEIAAELKMFPKVPTAEDLGYTREAPNEIRMFRWIGGE
TQALKLLQNRLAIEKSAFDGGYFLPNQANPDLLGAPTSQSAALRFGCLSVRRFYWNLQDVFKTVHGGNLPPSP
HITGQLIWREYFYTMSVNNEYYAEMERNPICLNIPWSEPKKEDLNRWKYGKTGFPFIDAVMRQLLLEGWVHHV
ARNAVACFLTRGDLWISWEYGLHHFFKYLLDADWSVCAGNWMWVSSSAFEQVLDCSECVCPVAFGRRLDPGGE
YVKRYLPELRSLPSEYLYEPWKAPIDVQERSNCIIGKDYPPPIVDHTEASKRNKYFMQTLREQIMGGGNAPPH
CRPSDENEAKSFLGLPSQCHEHCLIQI
>Chrysoperla_carn__XP_044735319.1
MIANEYIASQKRTNSEAFKTKTKTKDDSTVKDETKTNDLITSLKEKRNQTAKSILDFDFKKKRVQILSQAKEI
KESDSSGGILYWMFRDCRVQDNWALLFAQKLALKNELPLNILFCLPQKYLEYNARHYKFLIKGLEEVQEECDE
LNINFNVCVGEPAIQVVDFIESNHIQGVVCDFLPLKDCMQWQESVLNQIDSNQIPFIRVDAHNIVPCWIASDK
LEYAARTIRNKINSQLSQYLQQYPPVIQHKYNNKNIINKKKIDWNDVLNQLKYDKSDELINNNEITWAEGGTS
NGLKMLQSFQQKRIQLFGTKRNDPTISALSNLSPWLHFGHISAQRCVLVIQELKSKYTESVNSYCEEVIIRRE
LSDNFCYYNINKYNKLDGAPSWAQLTLKQHSKDKRTYLYTLDELQHGRTHDDLWNSAQIELYKTGKLHGFMRM
YWAKKVLEWTESPEQALQYALYLNDQYALDGNDPNGYVGCMWSICGVHDQGWGEREIFGKIRFMNYQGCKRKF
DIKAYIARWGGKEYNKKQETIPFKKLKK
>Chrysoperla_carn__XP_044742491.1
MEKKTVIHWFRKGLRIHDNPALLAAIEEAVKSSSALRPIFILDPLILTWMRVGPNRWRFLQQSLDQLNENLTK
IGSKLYVIRGTPENVFKRIFKDWNISYITYEYDTEPYAKRRDALIEQLAHEANIRIESEISHTIYNTQLVIKK
NGGKAPLTMQKFLSVVDSMSKPPIPVPAPTKIPEICKPTLDSFEKENPHCYDCPTLLELKVNEKNLNACKFPG
GETEALNRLNKILLKKSWICNFEKPNTSSNSLEPSTTVLSPYLKFGCLSSRLFYYEINKIISGQKHSKPPVSL
IGQLMWREFYYTVSSDTPNFDKMVGNPICCQVPWDTNEKHLEAWTYGKTGYPFIDAIMRQLRQEGWIHHLARH
AVACFLTRGDLWISWEEGLKVFDELLLDSDWALNAGNWMWLSASAFFHQFFRVYSPVAFGKKTDKFGDYIKKY
VPELNKYPVDYIYEPWKAPLSVQTKAGCLIGKDYPKRIVIHEDVYKVNISKMSLAYKNNKTNDNNKQKGESSK
ESEPAAKKMKKVSKSTNTTKSDSKKSKQTSLTKFLKK
>Chunga_burm__NWS56282.1
KVDYDVCSNYGNWLYSAGIGNDPRDNRKFNMIKQGLDYDGNGDYIRLWVPELQGIKGADIHTPWALNSAALSQ
AGVTLGETYPQPVVTAPEWSRHINQRPQGRSPHLRGRKGPAHTPMQHKDRGIDFYFSRKKDV
>Chunga_burm__NWS56972.1
MPHRTIHLFRKGLRLHDNPTLLAALETSEAIYPVYILDRKFMTSVMHIGALRWHFLLQSLEDLQKNLCQLGSC
LLVIQGEYESVLRDHIQKWNITQVTLDAEMEPFYKEMEANVRCLGEELGFEVLSLVGHSLYDTKRILDLNGGT
PPLTYKRFLHVLSLLGDPEVPVRNITAEDFQRCRPPDSSLAERYRVPLPMDLKIPLESLSPWRGGETQGLQRL
EQHLTDQGWVANFTKPRTIPNSLLPSTTGLSPYFSMGCLSVRTFFYRLSNIYAQAKHHSLPPVSLQGQLLWRE
FFYTVASATPNFTKMAGNPICLQISWYEDAEKLHKWKTAQTGFPWIDAIMTQLRQEGWIHHLARHAVACFLTR
GDLWISWEEGMKVFEELLLDADYSINAGNWMWLSASAFFHQYTRIFCPVLFGKRTDPEGQYIRKYLPILKNFP
SKYIYEPWTASEEAQKQAGCIIGQDYPFPMVNHKEASDHNLQLMKQVREEQYRTAQLTRDDTDDPMEMKVKRD
HSEENISKGKTVRMTE
>Chunga_burm__NWS57622.1
CIYILDPWFAASSAVGINRWRFLLQSLEDLDNSLRKLNSRLFVVRGQPTDVFPRLFKEWGVTRLTFEYDSEPF
GKERDAAIVKLAKEAGVEVVIENSHTLYDLDRIIELNGHKPPLTYKRFQAIISRMELPKKPVSTVISQQMETC
KVDIQENHDDVYGVPSLEELGFPTDGLAPAVWQGGETEALARLDKHLERKAWVANYERPRMNANSLLASPTGL
SPYLRFGCLSCRLFYYRLWELYKKVKRNSTPPLSLYGQLLWREFFYTAATNNPKFDRMEGNPICIQIPWDRNP
EALAKWAEGKTGFPWIDAIMTQLRQEGWIHHLARHAVACFLTRGDLWISWESGVRVFDELLLDADFSVNAGSW
MWLSCSAFFQQFFHCYCPVGFGRRTDPSGDYVKRYLPKLKGFPSRYIYEPWNAPESVQKAAKCIIGVDYPKPM
VNHAETSRLNIERMKQIYQQLSRYRGLCLLASVPSCVEDLSGPVTDSASGQGCSTSTAMRLSQADQASPKRKH
EGAEELCTEELYKRAKVTGLPAPEIPGKGL
>Chunga_burm__NWS60171.1
DNWAFLYAQRLALKQELPLHVCFCLVPKFLDATIRHYGFMLRGLQEVAEECAELNIPFHLLLGCAKDVLPAFV
VEHGVGGLVTDFCPLRLPRQWVEDVRQRLPEDVPFAQVDAHNIVPCWVASPKQEYSARTIRGKIHAQLPEFLT
EFPPVIRHPYPPSCVAEPIAWEACYSSLQVDRTVKEVEWATPGTAAGLAVLQSFIAERLKSFGSHRNDPNKAA

LSNLSPWFHFGQVSTQRAILEVQKHRGKYKESVDVFVEEAVVRRELAENFCYYNDNYDSVEGAYDWAQTTLKL
HAKDKRPFLYELQELEQGTTHDPLWNAAQLQMVQEGKMHGFLRMYWAKKILEWTRSPEEALQFAIYLNDRYEL
DGRDPNGYVGCLWSICGIHDQGWAERAIFGKIRYMNYAGCKRKFDVSQFERRYTPCT
>Chunga_burm__NWS60537.1
MGVNAVHWFRKGLRLHDNPALRECIQGADTIRCVYILDPWFAGSSNVGINRWRFLLQCLEDLDANLRKLNSRL
FVIRGQPADVFPRLFKEWNIAKLSIEYDSEPFGKERDAAIKKLASEAGVEVIVRISHTLYDLDKIIELNGGQP
PLTYKRFQTLISRMEPLEVPAETITPEVMEKCTTPVSDDHDEKYGVPSLEELGFDTDGLPSAVWPGGETEALT
RLERHLERKAWVANFERPRMNANSLLASPTGLSPYLRFGCLSCRLFYFKLTDLYKKVKKNSSPPLSLYGQLLW
REFFYTAATNNPRFDKMEGNPICVQIPWDKNPEALAKWAEGRTGFPWIDAIMTQLRQEGWIHHLARHAVACFL
TRGDLWISWEEGMKVFEELLLDADWSVNAGSWMWLSCSSFFQQFFHCYCPVGFGRRTDPNGDYIRRYLPVLRG
FPAKYIYDPWNAPESVQKAAKCIIGVNYPKPMVNHAEASRLNIERMKQIYQQLSRYRGLGLLATVPSNPNGNG
NGNGGLMGYSPGESISGCGSTGGHLFPASPGAQLGTGDSHTVVQPCALADSHTGASGIQQQQGYCQASSILHY
AHGDNQQSHLLQAGRTALGTGISAGKRPNPEEETQSVGPKVQRQSTN
>Chytriomyces_con_TPX68125.1_CcCBS67573
MASPKPRVCYWFRTDLRLHDSPALAAALNLKPEALFPLWCWDPNVNACCSLGLESGVNRAFLQYVYKHRVGVH
RFRFLLESMTVLSDQISAIQPNSKLLVVRGEPVELLPILWKKWGISHLVFEKDPSAYGRRRDAIVLKAAKLAN
VTIVSVQGHHLYDPELVTLKNKGKPTMTITSLQNIVKKMGNVPKPVASPKELPLPLLKGSTKTERKDIFLELS
NALRGTPIFSGNCLEGDTAVDLNSASLGGERHLPVTCYETVDGGDALFSVPTLESLGMSEDKEAKIKGGEVIA
LKRLATLCDDSKYIATFSKPKSSPAQWVDKPSTTLLSPYLKFGCLSIRKMWWDVEEAKQRYIGAKTEPPENLN
GQLLFRDMYACAEYAIGDAFGKVRGNEICRYMDWNLPTVYDESGVEIVPRPPGDSVSESRLKAFKDGQTGFPW
IDAQMRQLKQEGWMHHLGRHSVAAFLTRGQCWISWERGAEIFDEYLIDWDPCSNPGNWMWLSCSAFYAQYFKL
YGLVSFPQKYDKTGALVRKYCPELAKYPDAYIYEPHKAPANVQKQAGCIIGTDYPYPILDEKNSKDENMARMK
ACYEAKFYGNSKEVLDGTANALIQQLHGLKGAPKEYVFPGSQKSGSASGSGKKESKTLDGWLKRKANSDEDGG
RKK
>Chytriomyces_con_TPX70061.1_CcCBS67573
MSIKKSAVVCVMQWNSLRASDQPVLQAALKLNSDYLVAMHVVDRQFVAAGSRASERRRAFYAASVRSLQTQLV
QRGTGLVVLEESSSGSSNGLNEVRVSSVSSISSAPATASSAVNPSEDTANPSSAKSFSASAASLIAQKVALLS
ETCRVDAVCVEDQNTVHELHFVDALKKAMAKLNLKLIVTPNAQTLVKHNAIASKYLPDTFTQFRLTVEKSGNM
YRELFQIPSKLPPLPPALEQAASQWHSSLSNETGASLEMDPRSAFPFAAGEKAALQRIQNYFTQTHAIKTYKG
TRNELIGTQYSSKLSPFLAFGCVSASTVHNLLQQYEATHGGNESTYWLLFELLWRDYFQFIGKKYGSQLYHLR
GITTSTERIESTWKYNPRKFEAWITGHTGIPFVDANMRELLQTGFMSNRGRQNVASFLVRDLNLPWTLGAKWF
EKHLLDHEPCSNYGNWQYVAGVGNDPRSDRYFNVIKQGKDYDPEGAYVKLWCPELKNVPVSVIHCPWKMSFDE
QKASEVALGVTYPLPIVEADAWKKHYAYSTKGKVGKANPSETVGRRPKRK
>Chytriomyces_con_TPX75854.1_CcCBS67573
MKRAASTSDDHDSKRVKDDAQAAPPPAAQRPVVVWIRSDLRISDNSALTRASRIAVADGNPLVVMFVVSPNEW
LEHDMAAIKCRFILHCVSALRDALALLNVSFVTLTAASRRDVPQVVCDAVVRLNANRILWNIEYEIHETQRDK
KVTKLLADRQIPSTACHDQCILMPGKARTKEGKVYTVFTPFKKTWITILQKDPSLYKLQPMSAPLPVASPALL
ETITAISTGLPTTVPQPFEAPSQELIDFADKMYPAGEANARIRLESFVKARIKGYKNDRDSPGKDLTSRLSAY
LALGVISPRECLVEALNANNQKFDSGSDGAVTWISELCWRDFYKNILVEYPRVCKNQPFKLETDNVTWTTDPE
SPLFKAWCTGHTGYPIVDAGMRQLLAEGWMHNRVRMIVASFLTKDLGIDWRLGEKHFMRHLIDGDLASNNGGW
QWAASTGTDSQPYFRIFNPTLQSEKFDPAGSYIRKFVPELKGVLGKEIHDPKGKLGLPRIAKLGYVAPIVDHK
IASQAFLEAFKRGIGK
>Ciconia_magu__NXJ33658.1
MQHSSIHWFRKGLRLHDNPALLAAAADCRRLHPLFILDPSSSQAGANAQRFLLDALRDLDGSLREMGSRLFVV
RGCPEEVFPRLFHTWGTTRLTFEVDTEPPARRRDAAVAELAARHGVEVIREVSHTLYDTERVLALNDGKAPLT
YKHLQSLLASLGPPEKPAPALTRDHLRGCCTPCQVSHDANYGVPTLEELGQDPTEVGPHLYPGGKTAALARLD
AVMERTAWVCGFKKPETEPNSLSPSTTVLSPYLKFGCLSVRTFWWRLDEVYQGREHSRPPVSLHGQLLWREFF
YTAGAGIPNFDRMVGNPVCLQVDWDNNPQHLCAWREGRTGYPFIDAIMTQLRTEGWIHHLARHAVACFLTRGD
LWVSWEEGLKVFEELLLDADWSLNAGNWLWLSGSAFFHRYFHIYSPVAFGKKTDRDGAYIRKYLPVLKEFPAE
YIYEPWKAPRAVQERAGCLVGTHYPQPIVEHGAASKRNMGRMKAARTQK
>Ciconia_magu__NXJ33862.1
CIYILDPWFAASSAVGINRWRFLLQSLEDLDNSLRKLNSRLFVVRGQPTDVFPRLFKEWGVTRLTFEYDSEPF
GKERDAAIVKLAKEAGVEVVIENSHTLYDLDRIIELNGHKPPLTYKRFQAIISRMELPKKPVSTVMSQQMETC
KVDIQENHDDVYGVPSLEELGFPTDGLAPAVWQGGETEALARLDKHLERKAWVANYERPRMNANSLLASPTGL
SPYLRFGCLSCRLFYYRLWELYKKVKRNSTPPLSLYGQLLWREFFYTAATNNPKFDRMEGNPICIQIPWDRNP
EALAKWAEGKTGFPWIDAIMTQLRQEGWIHHLARHAVACFLTRGDLWISWESGVRVFDELLLDADFSVNAGSW
MWLSCSAFFQQFFHCYCPVGFGRRTDPSGDYVKRYLPKLKGFPSRYIYEPWNAPESVQKAAKCIIGVDYPKPM
VNHAETSRLNIERMKQIYQQLSRYRGLCLLASVPSCVEDISGPVADSASGQGCSTSTAMRLSQADQASPKRKH
EGAEELCTEELYKRAKVTCLPAPEIPGKS
>Ciconia_magu__NXJ33894.1

DNWAFLYAQRLALKQELPLHVCFCLVPKFLDATIRHYGFMLRGLQEVAEECAELNVPFHLLLGYAKDVLPTFV
VEHGVGGLVTDFCPLRLPRQWVEDVRERLPEDVPFAQVDAHNIVPCWVASPKQEYSARTIRGKIHAQLPEFLT
EFPPVIRHPYPPSCPAEPIAWEACYSSLQVDRTVKEVEWATPGTAAGLAVLQSFVAERLKSFGSHRNDPNKAA
LSNLSPWFHFGQVSTQRAILEVQKHRGKYKESVDAFVEEAVVRRELAENFCYYNENYDSVQGAYNWAQTTLKL
HAKDKRPFLYELQELEQGTTHDPLWNAAQLQMVREGKMHGFLRMYWAKKILEWTRSPEEALRFAIYLNDRYEL
DGRDPNGYVGKLQDGGMGWGGCLWSICGIHDQGWAERAIFGKIRYMNYAGCKRKFDVGQFERRYAP
>Ciconia_magu__NXJ34657.1
MLHRTIHLFRKGLRLHDNPTLLAALESSEVVYPVYILDRTFMTSMMRIGALRWHFLLQSLEDLQKNLCQLGSC
LLVIQGEYESVLRDHVQKWKITQVTLDAEMEPFYKEMEANVRRLGEELGFEVLSLVGHSLYDTKRILDLNGGT
PPLTYKRFLHILSLLGDPEVPVRNPTAEDFQRCRPPDPGLAECYRVPLPVDLKIPLESLSPWRGGETEGLQRL
EQHLTDQGWVASFTKPRTIPNSLLPSTTGLSPYFSMGCLSVRAFFYRLSNIYAQAKHHSLPPVSLQGQLLWRE
FFYTVASATPNFTKMAGNPICLQISWYEDAERLHKWKMAQTGFPWIDAIMTQLRQEGWIHHLARHAVACFLTR
GDLWISWEEGMKVFEELLLDADYSINAGNWMWLSASAFFHQYTRIFCPVRFGKRTDPEGQYIRKYLPVLKNFP
SKYIYEPWTASEEEQKQAGCIIGQDYPFPMVNHKEASDHNLQLMKQVREEQYRTAQLTRDDTDDPMEMKVKRD
QSEENVSKGKVARMTE
>Ciconia_magu__NXJ37697.1
KVDYDVCSNYGNWLYSAGIGNDPRDNRKFNMIKQGLDYDGNGDYVRLWVPELQGIKGADIHTPWALNSAALSQ
AGVTLGETYPQPVVTAPEWSRHINQGPQGRSPHPRGRRGPAHTPMQHKDRGIDFYFSHKKDV
>Ciconia_magu__NXJ40253.1
RFLLQCLEDLDANLRKLNSRLFVIRGQPADVFPRLFKEWNIAKLSIEYDSEPFGKERDAAIKKLASEAGVEVI
VRISHTLYDLDKIIELNGGQPPLTYKRFQTLISRMEPLEMPVETITPEVMEKCTTPVSEDHDEKYGVPSLEEL
GFDTDGLPSAVWPGGETEALTRLERHLERKAWVANFERPRMNANSLLASPTGLSPYLRFGCLSCRLFYFKLTD
LYKKVKKNSSPPLSLYGQLLWREFFYTAATNNPRFDKMEGNPICVQIPWDKNPEALAKWAEGRTGFPWIDAIM
TQLRQEGWIHHLARHAVACFLTRGDLWISWEEGMKVFEELLLDADWSVNAGSWMWLSCSSFFQQFFHCYCPVG
FGRRTDPNGDYIRRYLPVLRGFPAKYIYDPWNAPESVQKAAKCIIGVNYPKPMVNHAEASRLNIERMKQIYQQ
LSRYRGLGLLATVPSNPNGNGNGGLMGYSPGESISGCGSTGGAQLGTGDGHTVVQPCALGESHTGASGIQQQG
YCQASSILQYAHGDSQQSHLLQAGRTALGTGISAGKRPNPEEETQSVGPKVQRQSTN
>Circaetus_pect__NXW12461.1
RFLLQSLEDLDNSLRKLNSRLFVVRGQPTDVFPRLFKEWGVTRLTFEYDSEPFGKERDAAIVKLAKEAGVEVV
IENSHTLYDLDRIIELNGHKPPLTYKRFQAIISRMELPKKPVSTVISQQMETCKVDIQENHDDVYGVPSLEEL
GFPTDGLAPAVWQGGETEALARLDKHLERKAWVANYERPRMNANSLLASPTGLSPYLRFGCLSCRLFYYRLWE
LYKKVKRNSTPPLSLYGQLLWREFFYTAATNNPKFDRMEGNPICIQIPWDRNPEALAKWAEGKTGFPWIDAIM
TQLRQEGWIHHLARHAVACFLTRGDLWISWESGVRVFDELLLDADFSVNAGSWMWLSCSAFFQQFFHCYCPVG
FGRRTDPSGDYVKRYLPKLKGFPSRYIYEPWNAPESVQKAAKCIIGVDYPKPMVNHAETSRLNIERMKQIYQQ
LSRYRGLCLLASVPSCVEDLSGPVTDSASGQGCSTNTAMRLSQADQASPKRKHEGAEELCTEELYKRAKVTGL
PTPEIPGKSL
>Circaetus_pect__NXW13967.1
MQHSSIHWFRKGLRLHDNPALLAAATDCRHLHPLFILDPSSSQAGTNAWRFLLDALQDLDGSLREMGSRLFVV
QGCPEEVFPRLFHAWGTTRLTFEVDTEPSAKKSDAAVAELAERHGVEVKKKVSHTLYDTKRVLALNDGKAPLT
YKGLQSLLASLGPPEKPAPTLTLEHLQGCHTPCQVSHDTEYRVPTLEELGQDPTEVGPHLYPGGETAALTRLN
ALMDRTAWLCSFKKPETEPASLSPSTTVLSPYLKFGCLSVRTFWWWLDEVYQGQREHSQPPVSLHGQLLWREF
FYTAGAGIPNFDRMVGNPICLQVDWDNNPQHLRAWREGQTGYPFIDAIMTQLRTEGWIHHLARHAVACFLTRG
DLWISWEEGLKVFEELLLDADWSLNAGNWLWLSGSAFFHRYFHIYSPVAFGKKTDRDGAYIRKYLPILKDFPA
KYIYEPWKAPRAVQEQAGCLVGTHYPQPIVEHGAASKRNLGRMKAARAQK
>Circaetus_pect__NXW15569.1
AQLSLLFSSSVLKVDYDVCSNYGNWLYSAGIGNDPRENRKFNMIKQGLDYDGNGDYVRLWVPELQGIKGADIH
TPWALNSAALSQAGVTLGETYPQPVVTAPEWSQHINQKPQGRSPHPRGRRGPAHTPMQHKDRGIDFYFSHKKD
M
>Circaetus_pect__NXW19350.1
MPHRTIHLFRKGLRLHDNPTLLAALECSEVVYPVYILDRKFMTSVMHIGTLRWHFLLQSLEDLQKKLCQLGSC
LLVIQGEYESVLRDHIQKWNITQVTLDAEMEPFYKEMEANIRRLGEELGFEVLSLVGHSLYNTKRILDLNSGT
PPLTYKRFLHILSLLGDPEVPVRNLTAEDFQKCRPPDLGLAECYRVPLPVDLKIPAESLSPWRGGETEGLQRL
EQHLTDQGWVASFTKPRTIPNSLLPSTTGLSPYFSMGCLSVRTFFYRLSNIYAQAKHHSLPPVSLQGQLLWRE
FFYTVASATPNFTKMAGNPICLQISWYEDAERLHKWKTAQTGFPWIDAIMTQLRQEGWIHHLARHAVACFLTR
GDLWISWEEGMKVFEELLLDADYSINAGNWMWLSASAFFHQYTRIFCPIRFGKRTDPEGQYIRKYLPILKNFP
SKYIYEPWTATEEEQKQAGCIIGQDYPFPMVNHKEASDHNLQLMKQVREEQYRTAQLTRDDTDDPMEMKVKRD
HPEENVSKGKVARMTE
>Cloeon_dip_CAB3360699.1_protein
MFTNKLTQQTNLLTQQLTTIFLTRIFSLSVANSFIRQSRTMASGPKKLKLGESSKPINNDAFFTEINEARAKA
GKSVMDFKFNKKRIKVLSKASDVPEECKGIVYWMSRDQRVQDNWAFLFAQKLAIKNKVPLHICFCLLSKFLGA

PFRHFKFMLDGLQEVQQECKKLEIQFHLLEGEAASVLPAFIKAHDIGGVVIDFSPLRTHRSWVDELKKKLPST
VPLCQVDAHNIVPCWVASDKLEYGARTIRKKVMDKLPEFLTEFPLVAKHEYPAKFKAEPIDWTAVEEKLEVDR
SVGPVSWAKPGSEAGLAMLNEFCCKRIKLFGSKRNDPNINALSNLSPWFHFGHLSVQRAIKYVQKFKSSHSES
VAAFVEEAVVRRELSDNFCMHNPKYDSIEGTNNWAKETLEVHKKDKREYIYSLEQFDQAKTHDDLWNSAQLQM
VREGKMHGFLRMYWAKKILEWTESPEQALEFAIHLNDKYNLDGRGPQWICWLHVVDLRHSRPGLGREASVWQD
SLHELPGLQTQV
>Cloeon_dip_CAB3360724.1_protein
MSGTETGTRSPPHAEKHTVHWFRKGLRLHDNPSLLEGLRAATTFRCVFILDPWFAGSSNVGINKWRFLLQCLE
DLDRSLRKLNSRLFVIRGQPADTLPKLFKEWGTTCLTFEEDPEPFGRVRDQNIMTLCKELGLSVVSRISHTLY
DLEKILEKNNGKPPLTYHQFQNVVASMDSPPQPEPMVTLDTLRGCWTPLTEDHDDKYGVPSLEELGFDTEGLL
PPVWRGGESEALARLERHLERKAWVASFGRPKMTPQSLLASQTGLSPYLRFGCLSTRLFYYQLTDLYKKIKKA
APPLSLHGQLLWREFFYCAATKNPNFDKMAGNPICVQIPWDKNPEALAKWANGQTGFPWIDAIMTQLREEGWI
HHLARHAVACFLTRGDLWVSWEEGMKVFEELLLDADWSVNGGMWMWLSCSSFFQQFFHCYCPVRFGRKADPNG
DYIRRYLPVLKNFPTKYIHEPWNAPEAVQRAAKCIIGKDYAVPMVNHAIASRINMERMKQVYQQLSKYRGAGL
LATVPSSQNGAIPPSEPLHSVTTLSGLPNNMNLKQSSLSPPKDKHRSKSPGAPMLPPARPQQHHKSNQQQQQQ
IGDCHFQKQSGI
>Cloeon_dip_CAB3366165.1_protein
MSRGTVIHWFRKGLRLHDNPALLEAIRECEKQPEVIKELRPIFILDPLILDHMRVGPNRWRFLQQSLSNLDDR
LKALGSALLVIRGKPEEVFPKLFKTWNVKCITFENDIEPYAKKRDEQIEAMAKDAGIKVIQKVSHTLYSPAKI
LNANMGKVPLTYQKFVSVAEGLGPPPAAVDEPKKVPPGYSQILESRELKIPKEEDIFGNTNVPTLEELGLDES
KLEPCLYPGGEAEALQRMQICLAKKAWICDFEKPNTSPNSLIPSTTVLSPYLKFGCLSPRLFFHELNKIIQGR
KHSKPPVSLVGQMYWREFYYVVGFATPNYDRMVGNSICCQIPWGNNQEHLKAWTEGRTGYPFIDAIMTQLRKE
GWIHHLARHAVACFLTRGDLWISWEEGQKVFEELLLDADWALNAGNWMWLSASAFFHQYFRVYSPVVFGKKTD
KFGDYIKKYLPILKKYPVEYIYEPWNAPLSVQKAAGCIIGTDYPRPIVKHEVVSKENIAKMNAAYKANKANKG
EAGKSKSSAAGGGSSAKQAKKR
>Cloeon_dip_CAB3385983.1_protein
MRIQCIGRSPPHARVIVPREAPVTWTATYAPALGAPVIQQRQWPTMNRPVQMSVLWFRHGLRLHDNPALLDAI
SGPPSMFFPIFIFDGESAGTKVIGFNRMRFLLESLADLDRQLRSRGGRLWLLRGDPSKIFQRLWEELALTKVC
FEQDCEPIWNERDQSVRATCAERGVRCEEFVSHTLWDPKAVIYANGGLPPLTYQMFLHTVSTLGEPPRPVEDP
DWTGVTFGTLPPRLVKEFQVFDEVPKPEYFGLSQPGYGLVRWIGGETRALQHLRERLTQEEQAFRKGIILPNQ
SRPDLLGPPTSQSAALRYGCLSVRRFYWNLHDLFNAVSQGDPPAGLNISGQLIWREYFYTMSVYNPHYAEMSA
NPICLDVPWDKNPTFLEKWKKGQTGFPFIDAAMRQLVSEGWIHHVARNSVACFLTRGDLWISWEDGLAFFLEH
LIDADWSVCAGNWMWVSSSAFEQLLDCSKCLCPVNYGRRLDPWGEYIKRYVPELRNLPVEYLYEPWKAPLDVQ
RDAGCIIGDHYPERIVDHQRASNQNRQVFQLIEGFNNNDFNIK
>Clonorchis_sin_GAA29131.2_photolyase
MSGASNWLKQIEQQRLNAATSVTTFKFSKARVRQLKGPDEFPAEANSGGVLYWMIRDQRVQDNWAFLYAQRLA
LKFQVPLHVCFCLVPCYQADTLRHFSFMVGGLTQVEQECRALNIHFHLLNIADGLSPSTRSQGSKHRWAEEDS
QALQPPDLYGSQVAEQLKSLVQTLNIGCIVTDFSPLRAPTMWIEHVRTQLPPQLPFCQVDAHNIVPVWCGSDK
CEFSARTIRPKLYEQTGQFLTEFPPLIKHPFSCSAPTCRKTVNWNKIRENYWGDCTVQPVDWAKPGTAAAFYV
LRSFIDERLRDFDAHRNDPANPALSGLSPWFHFGQMAPQRAVLEVAALQHKYGRSVDVFIEEAFNRRELADNF
CFHTPLYDRLQGARQWAQDTLHKHAEDKRDVAFTRAQLESSGTTDDLWNAAQRQLVQTGKMHGFLRQYWAKKI
LEWCADGPEVAIQWAIYLNDRYSLDGTDPNGYVGIMWAICGVHDQGWPERRIFGKVRYMCYNGCRRWFSVPTF
VSRFSDA
>Clunio_mar_AFS34617.1_cry_2
MPDPNQLPKKEKHIVHWFRKGLRLHDNPALREGLKNATTFRAVFFVDPWFAGSSNVGINKWRFLLQCLEDLDQ
NLRRLNSRLFVIRGQPAEKLPMLFKKWNTTCLTFEEDPEPFSKVRDNNITEMCKELNIEVISAVSHTLYKLEK
IIEKNNGKAPLTYHQFQAIIASMEPPPPAEATIAEDIIGNTRTPIDDDHDDRYGVPTLEELGFDTEGLKPPIW
IGGETEALARLERHLERKAWVASFGRPKMSPQSLLASQTGLSPYLRFGCLSTRLFYYQLTDLYKKIKKTCPPL
SLHGQLLWREFFYCCATKNPNFDKMSGNPICVQIPWDKNPEALAKWANGQTGYPWIDAIMSQLREEGWIHHLA
RHAVACFLTRGNLWISWEEGMKVFEELLLDADWSVNAGMWLWLSCSSFFQQFFHCYCPVKFGRKADPNGDYIR
KYIPALKNFPTKYIHEPWVASEAIQRTAKCIIGKDYPLPMINHVIASRNNMGRMKQVYQQLAKYRQTPNNPEF
GGSAISNILAKAQASAKAFTEQQSSSPSPTTILKTVNNSGNYMCSQRSDHIEMPPQTVLYHEPSLNASSSVLQ
QQNMMKHRNNTNDMCHQQENINEFQYDEDGTSQHHNSYNYSQKQMTALNETQQQEDQHTIEFRMNNLGNEDTG
>Clunio_mar_CRK90905.1_CLUMA_CG00
MTKSVLWFRQGLRLHDNPALIEAINTDDGRRQVTFYPVFIFDGESAGTKDVGYNRMKFLLESLLDLDEQFKQL
GAPGLFIFQGKPTEIFQNLHDNIGINKICFEQDCEYNERDNEIKYLSRELGIEVVEKVSHTLWNPEDIIRING
GFAPLTYQMLLHTVNVLGLPPRPVNNEVDFSQVNFGTIPEHHKINLRLMKSIPSPEDFLIFPENTGSDVYVNW
NGGERHALEQMIDRVKHEEEAFASGTYLPNQANVDLMGSPKSMSAALRFGCLSVRRFYYTIHDKFNEVQDKMV
YKLPGGHHITGQLMWREYFYTMSINNPYYGQIKNNPICLNIPWKSSEEDEILKWKQGRTGVPIIDASMRQLLA
EGWLHHTLRNLTATFLTRTGLWISWEVGLQHFLKYLLDADLPICGGNWMWVSSSAFETLLDSSRCSIISLAHR

LDPKGEYIKRYVPELRNFPMKYIHEPWRAPIDIQEDIECIIGQDYPSPMIDIVQALQINCNRMKKIRESLIES
QPHVRPSNEDEIRTFFWIADEIAIQCN
>Clunio_mar_CRL02690.1_CLUMA_CG01
MSKRQTAIHWFRKGLRVHDNPALATAVNEAIKRDLILRPMFMLDPTIIQWLKVGPNRWRFLQQTLADLDKNLR
KLNTKLFVVRGNPKEDFIKLFKEWNVILLTFESDIEPYSVDRDATVIKAAKQFHIEVIQECSHTIFNPELVVK
RNGGSAPLTYQKFLSVASSIKVPSPVEFPPKLPASSLPVNDHYEEKDENCYDVPTLKELGLNEKDLGSCQFPG
GESEALSRMEMNLKRKKWICDFEKPKTSPNSLEPSTTVLSPYLKFGALSSRLFYKRLKEVYKGSKHSNPPVSL
EGQIMWREFYYTVAATTPNFNKMVGNRVCAQIPWVKNQKYLEAWSFGRTGYPFIDAIMRQLRQEGWIHHLARH
AVACFLTRGDLWCHWEEGQKVFEELLLDADWALNAGNWMWLSASAFFHQYFRVYSPVAFGKKTDSEGKFIKKY
VPELKNFPSGIIYEPWKASLENQNKYGCIIGKDYPNRIVDHDIVMKENLVKMKAAYAKKKKFKR
>Clunio_mar_CRL08549.1_CLUMA_CG02
MKRFLETNDQQSSSKKTKTENSSSDFVISIQNNRLKTAESIRDFKFNKKRVKILSKVEDVAEKSNGIAYWMAR
DQRVQDNWALLFSQNLAIKNKIPLHVVFCLTDGFLGATIRHFDFMLKGLEEVANDCTTLNINFHLLRGEHTKE
IPKFIKNNKIGAVVCDFSPLRIHRSWVDGIKKELPPYVPLVQVDAHNIVPIWITSDKQEYAARTIRNKINSKL
GEFLTEFPPLVKHPHKSTEMPDKIDWKKTLQSLKVDETVGVVDWIKPGYKNAVEVLETFINKRLKVFATKRND
PTINALSNLSPFFHFGQISVQRAIIEVQKHKSKAKDSVDAFCEEAIVRRELSDNFCFHNKNYDNLDGITDWAK
ITLNAHRKDKRPYLYTRDELEKSKTHDDLWNAAQNQLRVEAKLHGFLRMYWAKKILEWTASPEEALETAIYLN
DRYNLDGRDPNGYVGCMWSIGGIHDQGWGERAVFGKIRYMNYEGCKRKFDIKAFIARYGGVVHKKK
>Clupea_hare__XP_012677759.2
MVVNSVHWFRKGLRLHDNPALQEALNGADTVRCVYILDPWFAGSANVGVNRWRFLLETLEDLDTSLRKLNSRL
FVVRGQPTDVFPRLFKEWNITRLTFEYDSEPYGKERDGAIIKMAQEFGVETVVQNSHTLYNLDKIIEMNNNRP
PLTFKRFQAIVSRLELPRKPLPTITPEQMDSCRIKIADNHDEHYSVPSLEELGFRNVSPPEAVWRGGETEALE
RLNKHLDRKGWVSNFERPRINMLSLLASPTGLSPYLRFGCLSCRVLYYNLRDLYMTLRKRCSPPLSLFSQLLW
REFFYTAATNNPNFDRMEGNPICVQIPWDNNPEALAKWAEGRTGFPWIDAIMTQLRQEGWIHHLARHAVACFL
TRGDLWISWESGMKVFEELLLDADHSVNAGSWMWLSCSAFFQQFFHCYCPVGFGRRTDPSGDFIRRYIPKLKD
YPNRYIYEPWNAPESVQKAANCVVGVDYPKPMINHAEGSRLNIERMKQVYQQLSHYRGLSLLASVPTIQEEVE
PSLSDDSQASGSSTPGAQRTSGGMKEVSTAAPAVVTSSSTASPPATSALTQRKRRWPTELQSDQCCSQSKVQH
TSNSPASQE
>Clupea_hare__XP_012696746.2
MPVENDSSVAQVRHLLRELLVGREDPGGFFCLCLSILGSRETRAGFRELIAPLTDKHRQLHSQLITIYQEYFS
REEDDELRLAPQDEDDELQLALALSLVHTSCQNSPRQPVPRSYAQLASAMPVKGQPQGVDTQKSMGTHKPAGR
VGERSPNGLKEPGATRPAATANEGRTDSSLANDSDSQEKLDSNDQSEKSRKSRRRRQRKQQQQLLKSPSSPRP
ILLWFRRDLRLLDNPALIGCLELGAPVIPVFLWNAQEEEGPGVTVATGGACKYWLHQALVCLNEALERIGSHL
VTVKAEPSSSEALQRLVEETGAGAVVATALYEPWLKERDEGVFNTLKQHRLSCHLYHSYCLQDPYTVTTQGVG
LRGIGSVSHFTSCCQQNPTGPSGAPQDAPRTLPVPSAWPAGCPLEQLGLARMPRRKDGTMVDWAVNIRRDWDF
TEEGAHARLEAFLQDGVRRYEKESARADAPNTSCLSPYLHWGQLSVRWLLWDARGARCRSPKFQRKLAWRDLA
YWQLTLFPDLPWESLRPPYKALRWSSERAHLHAWQRGRTGYPLVDAAMRQLWQTGWMNNYMRHVVASFLIAYL
HLPWQEGYRWFQDTLVDADVAIDAMMWQNGGMSGLDHWNFVMHPVDAAMTCDPNGTYVKRWCPELAPLPDELI
HKPWKCPASILRRAGVVLGQNYPERVVTDLEELRTQSLRDVAEVRRRFSEYVDQHSGCDLIPLPDRLVEEALG
STGEVLRSGSRFLLPVITRMEFKHQSVEPDAAEGRVNPYNAVLKGYVSRKRDETVAFLNERDFMASVVHEGTQ
RLERAERNQRLLEGLPAPPSATRGRPRRTPTMPQR
>Clupea_hare__XP_031416103.2
MAPKKADLKRKAKSVKISNAKQLKVDRVEGWLEGEVAEVRRGAQDVKFNEKRLRFLSDTKTVIRGTEGVVYWM
GRDQRVQDNWALIYAQQLAIAEELPLHICFCLMPNFLEATFRHYSFMLQGLQEVAKESSVLDIQFHLLSGFPG
DTLPGFIKEWNMGAVVTDFWPLRDPTRWVDDVKKAISPDIPFIQVDAHNVVPCWEASGKLEYAARTIRGKITK
LLPTFLTEFPAVDKHPHSSPKTAQAVNWNEILSSLGVDRCVGEVDWAKPGTSGGMAMLESFIDERLHCFATLR
NNPNVAAASQLSPWIHFGQLSAQRVVRQVQKNGKKFSESVASFTEEMVVRRELADNFCFYNENYDKIEGAYEW
ARKTLKDHAKDKRSHIYTREQLEQAKTHDKLWNAAQTQLLIEGKIHGFMRMYWAKKILEWTSSPEEALSIAIF
LNDRYSLDGCDPNGYVGCMWSICGIHDMGWAERPVFGKIRYMNYDGCKRKFDVAQFERLYSKMG
>Clupea_hare__XP_031420090.1
MVVNTVHWFRKGLRLHDNPSLRDSIRGADTLRCVYILDPWFAGSSNVGISRWRFLLQCLEDLDASLRKLNSRL
FVIRGQPTDVFPRLFKEWNINRLSYEYDSEPFGKERDAAIQKLASEAGVEVTVRISHTLYDLDKIIELNGGQS
PLTYKRFQTLISRMDVVETPVDAITPELMERCLTPLSDDHDDKFGVPSLEELGFETDGLSTAVWPGGETEALT
RLERHLERKAWVANFERPRMNANSLLASPTGLSPYLRFGCLSCRLFYSKLTDLYRKVKKNSVPPLSLYGQLLW
REFFYTTATNNPRFDKMDGNPICVQIPWDRNPEALAKWAEGRTGFPWIDAIMTQLRQEGWIHHLARHAVACFL
TRGDLWISWEEGMKVFEELLLDADWSVNAGSWMWLSCSSFFQQFFHCYCPVGFGRRTDPNGDFIRRYLPILRG
FPAKYIYEPWNAPEAVQKAAKCIIGVHYPKPMVNHAEASRVNIERMKQIYQQLSCYRGLELLATVPSNSSGAA
DGTAAGDGNQEGTTTSGRSLMVHQVPALTQGEGQVGTPAGTRGEPYHTDTSHHQQQQQQQQQQQVFLMGETA
ASHRQQAQTTTSQRVRGPMKRPTEDYIASRSKIQRQSIH
>Clupea_hare__XP_031423160.1

MSHRTIHLFNRGLRLHDNPTLVAALESSAAMYPVFLLDRAFMEGSVRMGALRWRFILQSLEDLHHSLQRLGSR
LYVIQGSHEAVLRRLVAQWGITQLSFDDEVEPHYKQLDKELRTLASELSLSVLSCVAHSLYDVKRIIKANSGQ
PPLTYKKFLRVLSVLGEPDTPAREITETDFQKCPTPGDEEGEEPYKVPSLSELGFEVEYEVLWPGGESHALKR
LEKHFQSQGWVANFSKPRTIPNSLLPSTTGLSPYLSLGCLSVRTFYHRLSKVYAQSKNHSLPPVSLQGQVLWR
EFFYTVASATPNFTRMVGNPICLQIDWYEDPQALHKWKMGQTGFPWIDAIMTQLRQEGWVHHLARHAVACFLT
RGDLWISWEKGMKVFEEYLLDADYSLNAGNWMWLSASAFFHRYTRIFCPIRFGRRTDPDGHYLRKYLPVLKNF
PSKYIYEPWTAPEHVQHQSGCIIGKDYPLPMVNHLEASQRNLALMKELRIEQENTADLTRDMADDPMEVGVKR
KLPEDTIAQRDATEYNSSVPVGNKRCSSPDESSWTPDPVRLPEMSSKVV
>Clupea_hare__XP_031440209.1
MSSSRTIICLLRNDLRLHDNEVFHWAQKNAEHIVPLYCFDPLQYLGTHCFNFPKTGPFRLRFLLDSVHDLRAS
LKKRGSNLVVRCGKPDQVVDDLIRQLGSVTAVAFHEEVTQEELEVEKQIKDVCSKNKVKVQSFWGSTLYHKHD
LPFNHISRLPDVYTQFRKAVESQARVRPVLSSPTQLRPLPLGLEEGSIPTYKDLEQTEPVADPRSAFPCSGGE
SEALARLQHYFWDTDAVATYKETRNGLIGIDYSTKFSPWLAMGCISPRYIYEQIKKYERERTANQSTYWVIFE
LLWRDYFKFVAMKYGNRLFNLNGLQDKHVPWKTDSKLLDAWKEGRTGVPFVDANMRELALTGFMSNRGRQNVA
SFLTKDLGLDWRMGAEWFEYLLVDHDVCSNYGNWLYSAGIGNDPRENRKFNMIKQGLDYDNNGDYVRQWVPEL
RGIKGGDVHTPWALSSAALSHAKVSLNDTYPSPVVMAPEWNRHVNKKPVGASGSSSRGRKGPSHTHKQHKDRG
IDFYFSKSKGLNASND
>Clupea_hare__XP_042562756.1
MTHNCIHWFRKGLRLHDNPALTAALRDCKQLYPVFILDPWFPNNTKIGINRWRFLIGALKDLDCSLRKLNSRL
FVVRGKPDEVLPELFRKWKITRLTFEVDTEPYSLQRDMEVLKLAGDHGVEVIQKISHTLYNLERIIDENNQKA
PLTYVRFQTVLKSMGLPKRPVPSPSLENMKGVITPCTDKHENYGIPTLEELGQDPELAIPEVFPGGEQEALKR
LDKYMDKPGWVCSFEKPQTSPNALTPSTTVLSPYVRFGCLSARTFWWRLSDVYQGKKHSQPPVSLHGQLLWRE
FFYTAALGVPNFNKMEGNPVCVQVDWDDKPDHLAAWREARTGFPFIDAIMTQLRQEGWIHHLARHAVACFLTR
GDLWISWEEGQKVFEELLLDADWALNAGNWQWLSASAFFHQYFRVYSPVAFGKKTDKHGDYIKKYLPLLKKFP
DQYIYEPWKAPRSIQERAGCIVGKDYPRPIVEHEIISKKNIQRMKAAYAKRSPQTAESPSKGKGVKRKRESIK
DMLTKKKKKSD
>Coccinella_sept__XP_044750690.1
MTDNETSDITSHIHHHRMASIKDKHTVHWFRKGLRFHDNPAMLEGLKNSKTFRCVFVLDPWFAGSSNVGINKW
RFLLQCLDDLDNNLKNLNSRLFVVRGQPADALPKLFKEWGTTCFSFEEDPEPFGQVRDKNIIALCNELGITVI
QRPSHTLYDLDKIIEKNGGTAPITYHQFLEVISSMGSPSEPLPTPFDSVSSNQLNTPFTNDHDHEFGVPTLEQ
LGFDTKTLSAAVWNGGESEALRRLERHLERKAWVASFGMPKMTPQSLLSSQTGLSPYLRFGCLSTRLFYYRLT
ALYKKIKNAFPPLSLHGQLLWREFFYCAATRNPNFDKMLGNPICVQIPWDQNAEALAKWANGQTGFPWIDAIM
TQLRQEGWIHHLARHAVACFLTRGDLWISWEEGMKVFEELLLDADWSVNAGTWMWLSCSSFFQQFSHCYCPVK
FGRKADPNGDYIRKYICVLRNMPIKYIHDPWTAPDNVQKAAKCVIGKDYPLPMVNHSLVSKINIQRMKQVYRQ
LKSYSENNDEFGTQITENCKNSCQYVQTDK
>Coccinella_sept__XP_044764764.1
MAPNVLKDLAKEIFAKNIATQRSEAGKSIKEFKFKKDRCRLLSKYEDLREDSKGIIYWMSRESRVNDNWALLF
AQKLCFKYEVPLHVCFFLDDYKELYPTTRQAGFLRKGLEHVRKDLEKLNIPFYLLKKSPLELVDIIQANDIGC
LVCDFFPLRIVTNWQEKLKELLPSNVAIVQVDAHNIVPCWIASDKLERAARSIRPKILKQLPTYLTEFPAVSK
QKEKKELVIKPGLEWSKNDKLDIEFVEEITWAEPGEEGGIEMLRTFLLERLQYYGISSNEPSKKHQSNLSPWL
HFGQISAQRIALEVSKLKKTWTTQCERFLEEAIIRKEICDNFCLYNPNYDSFEGADKWAKETLNLHRDDKRDF
LYTPEQFENCLTHDPIWNSAQFQLVTEGKLQGYMRMYWCKKILEWTESPEKAIKTALWLNDKYSIDGSDPNGF
VGVMWSICGIHDQGFKERPVFGKIRFMVDYSLSRKYDIKTYCAKYRPNAKVSGNITKYLQTSNKKNEDKECDS
KSPNKTEGKNGKNKETETEDKVKPAKQSSDKSCGKKRTVKSETHEESKKKRKV
>Colius_stri__KFP26438.1
RFLLQSLEDLDNSLRKLNSRLFVVRGQPTDVFPRLFKEWGVTRLTFEYDSEPFGKERDAAIVKLAKEAGVEVV
IENSHTLYDLDRIIELNGQKPPLTYKRFQAIISRMELPKKPVSTVISQQMETCKVDIQENHDDVYGVPSLEEL
GFPTDGLAPAVWQGGETEALARLDKHLERKAWVANYERPRMNANSLLASPTGLSPYLRFGCLSCRLFYYRLWE
LYKKVKRNSTPPLSLYGQLLWREFFYTAATNNPKFDRMEGNPICIQIPWDRNPEALAKWAEGKTGFPWIDAIM
TQLRQEGWIHHLARHAVACFLTRGDLWISWESGVRVFDELLLDADFSVNAGSWMWLSCSAFFQQFFHCYCPVG
FGRRTDPSGDYVKRYLPKLKGFPSRYIYEPWNAPESVQKAAKCIIGVDYPKPMVNHAETSRLNIERMKQIYQQ
LSRYRGLCLLASVPSCVEDLSGPVSDSASGQGCSSSTVLRLSQADQASPKRKHEGAEELCTEELSKRAKVTCL
PAPEIPGKS
>Colius_stri__XP_010202269.1
MSHRTVHLFRKGLRLHDNPTLLAAVESSQILYPVYILDREFVTSVMHIGALRWHFLLQSLQDLQKSLCQLGSC
LLVLQGEYEPVLKAHIQKWNITQVTLDAEMEPFYKEMESNIRRLGEELGFEVLSLVGHSLYDTKRILELNGGS
PPLTYKKFLHILSLLGDPEVPVRNLTAEDFQRCRPPDLGLAECYRVPLPVDLNIPLESLSPWKGGETEGLQRL
EQHLTDQGWVAHFTKPKTIPNSLLPSTTGLSPYFSVGCLSVRTFFYRLSNIYAQAKHHSLPPVSLQGQLLWRE
FFYTVASATPNFTRMVGNPICLQIPWYEDAEKLHKWKTAQTGFPWIDAIMTQLQQEGWIHHLARHAVACFLTR

GDLWISWEEGMKVFEEMLLDADYSINAGNWMWLSASAFFHQYTRIFCPVRFGKRTDPEGKYIRKYLPILKNFP
PKYIYEPWTASEEEQKQAGCIIGQDYPFPMVNHKEVSDHNLQLMKQVREEQQRTAQLTRGESCCRDCHQPQGS
>Colius_stri__XP_010202766.1
MRFLLQCLKDLDANLRKLNSRLFVIRGQPADVFPRLFKEWNIAKLSIEYDSEPFGKERDAAIKKLASEAGVEV
IVRISHTLYDLDKIIELNGGQPPLTYKRFQTLISRMEPLEMPVETITPEVMEKCTTPVSDDHDEKYGVPSLEE
LGFDTDGLPSAVWPGGETEALTRLERHLERKAWVANFERPRMNANSLLASPTGLSPYLRFGCLSCRLFYFKLT
DLYKKVKKNSSPPLSLYGQLLWREFFYTAATNNPRFDKMEGNPICVQIPWDKNPEALAKWAEGRTGFPWIDAI
MTQLRQEGWIHHLARHAVACFLTRGDLWISWEEGMKVFEELLLDADWSVNAGSWMWLSCSSFFQQFFHCYCPV
GFGRRTDPNGDYIRRYLPVLRGFPAKYIYDPWNAPESVQKAAKCIIGVNYPKPMVNHAEASRLNIERMKQIYQ
QLSRYRGLGLLATVPSNPNGNGNGGLMGYSPGESISGCGSSGGAQMGTGDGHTVVQPCALGDSHSGGASGIQQ
QGYCQASSILHYAHGDSQQSHLLQAGRTALGTGISAGKRPNPEEETQSVGPKVQRQSTS
>Colius_stri__XP_010206669.1
MAGTTRTAICLLRCDLRAHDNQVLHWAQSNADFVIPLYCFDPRHYVGTHCYGFPKTGPHRLRFLLESVKDLRE
TLKKKGSTLVVRKGKPEDVVYDLITQLGSVSAVAFHEEATQEELDVEKGLCQVCSQHRVKVHTFWASTLYHRD
DLPFRPIARLPDVFTHFRKAVESEAKVRPTLQLAEQLKPLTPGVEEGPIPTIEDLGQKDPVAEPRTAFPCSGG
ETQALMRLQYYFWDTNLVTSYKETRNGLVGMDYSTKFAPWLALGCISPRYIYKQIQKYEKERTANESTY
>Colius_stri__XP_010206798.1
GEKEEEEKEAVHEARRRTAPSVREFKYNKKRVRLVSQGDDLKEDARCILYWMSRDQRVQDNWAFLYAQRLALK
QELPLHVCFCLVPKFLDATIRHYDFMLRGLQEVAKECEELNIPFHMLLGYAKDVLPTFVVEHGVGGLVTDFCP
LRLPRQWVEDVRQRLPEDVPFAQVDAHNIVPCWLASSKQEYSAWTFRGKINTQIPEFLTEFPPIIHHPYPPSC
LAKPIDWEACYSSLQVDRTVKAVEWATPGTAAGLAVLQSFISQRLKSFGAHRNDPNKEALSNLSPWFHFGQVS
TQRAILEVQKQRSKYKESVDVFVEEAVVRRELSENFCYYNDKYDSVQAAYSWAQTTLKLHAKDKRPFLYKLEE
LEQGTTHDPLWNAAQLQMVREGKMHGFLRMYWAKKILEWTHSPEEALQFAIYLNDRYELDGRDPNGYVADAGC
LWSICGIHDHGWTERAVFGKIRYMNYAGCKRKFKVDQFERRYAPQTLSQ
>Colocasia_esc_hypothetice_MQM01560.1
MASTNPTSLLPCPPTPTQEAAEAPPEDHPHAAAPPPPPTASISLSLSTLLSLSGHKPSAPPVGALPSKVRIPS
QISAVSLSSSSAASSSKPSRTATRAAAISAFPLQAPLRYLTRRPAEPSHPAAGRRCTVVWFRSDLRVHDNEAL
TAASNDSLSLLPVYLFDPRDYGKSSSGFDKTGPYRATFLLESVRDLRNSLRARGSDLVVRVGRPETVLVELAR
EVGADAVYAHQEVSHDEVRMEEKMAKVMEEEGVEVKYFWGSTLYHMEDLPFDLEHMPSNYGGFREKVQGLGVR
KTIEALDQIKGMPRRGDVEPGDIPTLMDLGLTPPSTMGQDGKPASNSGLVGGETEALDRLKRFAAEFQAHPNK
GDKDGNCGSIYGANFSCKISPWLAMGCLSPRFMFDELKKTAARTISAASSQVNGGKPGNNGMNWLMFELMWRD
FFRFITKKYSSTKKKIEAVPATACTGALV
>Colocasia_esc_MQL88944.1_Taro_02151
MLGGGAGGVGSTSGGGGGTTTSIVWFRRDLRLEDNPALAAGVRSGYVVALFIWAPEEEGHYYPGRASRWWLSQ
SLRHLDCSLRSLGTALITKRSLDAASTLVEVVRSTGASQVFFNHLYDPLSLVRDHRLKELLSAQGITVRSFNA
DLLYEPWEVNDDEGQPFSTFAEFWNKCLSMPYDPAAPVLPPKRIMSGDVSRCSSDNLVFEDDSEKGSNALLAR
AWSPGWRNADKALAAFLSGPLIEYSVNRKKADSATTSLLSPHLHFGEVSVRKAFHLIRMKQLVWANDGSKAGE
ESINLFLKSIGLREYSRYMSFNHPCSHEKPLLAHLKFFPWVVDEGYFKAWRQGRTGYPLVDAGMRELWATGWL
HDRIRVVVSSFFVKVLQLPWRWGMKYFWDTLLDADLESDALGWQYISGSLPDGRELDRIDNPQFEGYKFDPNG
EYVRRWLPELARLPTEWIHHPWDAPESVLQAAGIELGSNYPLPIVEISEAKARLQEAIVEMWQLEAASRAAME
NGTEEGLGDSSDLAPIDFPQGIQMEVEHEPVRINNPAALARRHEDQMVPSMTFSLVRDEEESYVDLGNNREDN
RPEVPSNLGFDPEPQRDTGNQAGATIPQNNNIPHFNLIRLQDAADSTSESSSSWTGRDGGVVPVWSPPTVSSH
SEQFMGEDSAVGSSGYVRRHPPSRQLMNWSHLSHNR
>Colocasia_esc_MQL99100.1_Taro_03181
MAKLEGLVLPGRIRVLKEAATPPGKEKKKGPVVYWMFRDQRCRDNWALIHAVDLANRSGAPVAVAFNLFHRFL
GAHARQLGFMLRGLRELQARLAALRIPFFLFRGESGDTIPRFLGDCGASMLVTDFSPLREVRRSKDDICGRVG
ASVAVHEVDAHNVVPLWSASGKLEYSARTIRTKIHKLLPEYLVEFPELPTPTAEWAAEEPPAIDWDGLISDVL
REGAEVPEMGWCEPGEAAAREVLMGSSEGFLTRRLRGYADDRNNPLKPKGLSGLSPYLHFGHISAQRCALEAR
AFKKSHPQSVDAFLEEMIVRRELADNFCFYQPHYDSLQGAWEWARKTLTDHARDKRDHVYTKEELENAKTADP
LWNASQLEMVHHGKMHGFMRQIVLSTILIQVLIIRMYWAKKILEWTSGPEEALAIAIYLNDKYELDGRDPNGY
VGCMWSICGVHDQGWRERAVFGKIRYMNFAGCKRKFDVDGYIAYVKRLVGETKKRKLDSPTTKPKQTTITKSR
RLSKAEG
>Colocasia_esc_MQM06337.1_
MPWPARLGLQSLLGQLPSSAFLPRRCSGWILVRKDPNSCLLEFLVELKCTKVPMLPFGFQLAISCLGFASTTN
CYFGCQYLVPDFRHLYVECLPENASKLLIMGSHSRTIVWFRRDLRIEDNPALTTAGKDGSVLPVFIWCPKEEA
QFYPGRVSRWWLKQSLIHFGKSLRSLGVPLILMRAEDTFSALLQCINATGSTRVFFNHLYDPVSLVRDHTIKS
QLAEIGINVTTFNGDLLYEPWEVYDEQGLAFTTFDAFWEKCKSLPVEPTFHLPPWRLVPPDGSEAVQGCSIEE
LNLEKETEKSSNALLSRGWSPGWSNADKLLNEFMENHLIDYSRNMMKVEGATTSLLSPYLHFGELSIRKVYQS
VRMKQMQWLKEENSVAAESVKFFLKSLGLREYSRYLCFSFPFTHEKSLLGNLRHYPWQADEVQFKAWRQGRTG
YPLVDAGMRELWATGWIHNRTRVIVSSFCVKFLLLPWTWGMKYFWDTLLDADLESDILGWQYISGSLPDGHQL

DRLDSPEVQGWKFDPDGEYVRQWLPELTRVPTEWIHHPWDAPVSVLRAAGVELGLNYPKPIVDISVARERLDK
AIAMMWELDRTSKAEKKDETSEVVDDNLAGFKSLDIPKVVVKREVTYTSSSNDQRVPSIQNLKSSCNPQCSKD
IDHEKHNQDNRNDSIAKPEKSHMDEDLRSTAESSSARKRSMSESLCAVPTSSFSSSEAKFHDGYGLSKLQVPV
ESTSMCLWQGNCNTDRMVTVCLSRWWTLTSEFQPGFIGCYWRHWFGRVPHMV
>Colocasia_esc_MQM06510.1_Taro_03933
MESNALLWFRKGLRIHDNPALDHARRGATRRLFPVFVLDPRYLEPDPTAFSPGSARCGINRIQFLLESLADLD
GSLRRLGSRLLVLRGGDPADVLIRVLKEWKIGKLCYEADTEPYGQARDNRVREFASASGIEIFSPVSHTLFDP
ADVIQKNGGKPPLTYQSFVKLVGQPPSPIAQRVTSLPPVGDVGEYEILGVPTLQELGYGDIGQVEFSPFRGGE
SEALKRLKESLTSKEWVANFEKPKGDPSAFIKPATTVLSPYLKFGCLSSRYFYQSIQEIYKNAKRHTSPPVSL
VGQLLWREFFYTVAFGTPNFDQMRENKICKQIPWSNDEKLLASWREGQTGFPWIDAIMVQLQKWGWMHHLARH
SVACFLTRGDLFIHWEKGRDVFERLLIDSDWAINNGNWLWLSCSSFFYQYHRIYSPTKFGKKYDPTGKFIRHF
LPVLKDMPKEYIYEPWTAPLSVQKKANCIIGKDYPRPIVSHDTASKECKKKMGEAYAFNNLLGDANWKRLREK
SEKDKLDPGRRPRLKQKRLG
>Colocasia_esc_MQM16981.1_Taro_04994
PETHHEAEKRPRHLPKHPRVVASLLVTSFFPSPPSHTPLSLSLSPITTSLLPVPCHAMRVLVSPSPASLFFFF
SSTVKPCLTPRRPPSSTSLSAPSFAMMSSSSSTASSSTAPLPATRPGAAAVQMVPGLSAEEVAAAADEAFARY
TSAAGAGRRKEGAGVAVVWFRSDLRVLDNEALCRAWAASGSLLPVYCVDPRMFEETHYFGFPRTGAHRAQFLL
ECLADLRKNLIKKGLNLLVQHGKPEDILPAIAKAFGAHTVFAHKETCSEELLVERLVRDRLQQVTVILDGSPS
QSIKPKLQLFWGSTMYHIDDLPFGTGSLPDIYTQFRKSVEYKCKVRSCYRLPTSLGPLPSHGMDEIGGWGNIP
SLEQLGLVETKVDKGMCFIGGESAALARDLIKIYKETRNGMIRPDYSTKFSPWLAFGCLSPRYVYEEVKRYEK
ERLANESTYWVLFELIWRDYFRFLSKKYGNSIFHLGGPRNVVSKWSQDQMLFESWRDGRTGYPLIDANMKELS
ATGFMSNRGRQIVCSFLVRDMGIDWRMGAEWFEKCLLDYDPCSNYGNWTYGAGVGNDPREDRYFSIPKQAQTY
DPDGEFVAYWLPELQALPKERRHSPGSSYIKQVVPLKFGSFNQKQGHASTEREKQKRLYNRKA
>Colossoma_macr__XP_036413904.1
MAASRTVVCLLRNDLRLHDNEVFHWAQRNADHIVPLYCFDPRHYLGTHHFNFPKTGPFRLRLLLDSVKDLRAT
LKKKGSNLIVRRGKPEEVVGDLIKQLGSVSAVAFHEEVTSEEKRVEKKVKHVCSQNKVKVQTFWGSTLYHRDD
LPFCHIGGLPDVYTAFRKAVEAQSRVRPVLPSAEHIRPLPLGLEEGPIPTFDDLEQTEPLDDTRSAFPCRGGE
SEALARLKHYFWETNAVAAYKETRNGLIGVDYSTKFAPWLALGCISPRYIYEQIKRYEAERTANQSTYWVIFE
LLWRDYFRFVALKYGDRIFHLNGLQDKHVPWKTDMELFNAWKEGRTGVPFVDANMRELFLTGFMSNRGRQNVA
SFLTKDLGLDWRLGAEWFEYLLVDYDVCSNYGNWLYSAGVGNDPRENRKFNMIKQALDYDSNGDYVRQWVPEL
KGIMGGDVHTPWALSSASLSHAQVALDKTYPSPIVKASEWSRHINMKPSGAGSSKGKKGSSHTPRQHRDRGID
FYFSKSKNL
>Colossoma_macr__XP_036415715.1
MPHRTLHLFRKGLRLHDNPTLLGALESSSVVFPVYVLDRAFEEEVMRVGFLRWRFLLQSLLDLHGRLSVVGSR
LYVLRGSHRAVLRELVVRWGITQISFDSETEPHHTVLQRELREMAEELGLTVNTCVSHTLYDPRRIIDANGGE
APLTYKTFLRVLSDIGEPDKPVRDITTEDFQRCYTPDEAECEQYCVPSLSELGLEGPDEAQAEVLWPGGETHA
LQRLHKHLESQGWVENSKPQAVPNSLWPNTTGLSPYFSFGCLSVRTFYHTLSRVYARARNHALPPVALQGQVL
WREFFYTAATHTPNFTRMEGNPICLQIHWNHDPDSLQKWRTARTGFPWIDAIMTQLRQEGWIHHLSRHAVACF
LTRGDLWISWEEGMKVFEEYLLDADYSVNAGNWMWLSASAFFHQYNRIFCPVRFGRRTDPEGHYIRKYVPVLK
NFPSRFIYEPWTAPEEVQLQAGCIIGEDYPLPMVCHAEASERNLALMKEARSRQENTAKLTRDVAEEPVDVCV
KRELGEKDQPSKRFSSDPQSRPCSWGQETPRLPEITGQAM
>Colossoma_macr__XP_036423969.1
MVVNSVHWFRKGLRLHDNPALQEALNGADTVRCVYILDPWFAGSANVGVNRWRFLLESLEDLDTCLRKLNSRL
FVVRGQPADVFPRLFKEWNVTRLTFEYDPEPYGKDRDGAIVKIAQEFGVETIVRNSHTLYSPDRIIEMNSNSP
PLTFKRFQAIVNRLELPKKPLPTITQEQMASCRTQIADNHDEHYSVPSLEELGFKNQGDVTPVWKGGETEALE
RLNKHLDRKAWVASFERPRINAHSLFANPTGLSPYLCFGCLSSRVFYYHLRELYMKLRRKSSPPLSLFGQLLW
REFFYTAATNNPNFDRMEGNPICVQIPWDHNPEALAKWAEGRTGFPWIDAIMTQLRQEGWIHHLARHAVACFL
TRGDLWISWESGTKVFEELLLDADWSVNAGSWMWLSCSAFFQQFFHCYCPVGFGRRTDPSGDYIRRYIPKLKD
YPNRYIYEPWNAPESIQKAANCVVGVDYPKPMINHAESSRLNIERMKQVYQQLSHYRGLSLLASVPTIQEEAE
PPLSDDSQASSSGVPSSSTQTQPQSQQTQTQPQPQPPDPDPQPPATASVPPAQRKRGRTPEPRTSLNPKVQHT
SQAKASEQPPQTTDKQ
>Colossoma_macr__XP_036440474.1
MSLAKASQVSAMLRDLMLGLEDPEGFFCLCLSALGERDTRALFLDLIKPLASGHIQLYSQLTTIFFNYFSQGD
EEALEVAIALSLHETTYRDDPPQSSSSDQLAKEALGDSQAGDPKKPIKKECPLKDTRPQRTTTMIKRENNSDS
SSCPADDESKNHDNMIHSVKPKMSRRHRQRKKQQLMQNPSAPRPVLLWFRRDLRMWDNPALIGCLELGAPIIP
VFLWNAAEEEGPGVTVATGGASKYWLHQALVCLNRSLEERSSHLVFQRAEPSSLAALQALVAETGAGAVVATA
LYEPWLRDRDDSVWAVLEKQGVKCHIYHSYCLRDPYTVSTKGVGLRGIGSVSHFMSCCQQNSGPGIGTPLDAP
CILPTPLSWPQGCPLTQLGLARMPRRKDGTEIDWAVNIRQAWDFSEEGARARLETFLKDGVYRYEKESSRADA
PNTSSLSPYLHFGQLSARSLLWDARGARCRPRKFQRKLAWRDLAYWQLSLFPHLPWESLRPPYKALCWSLDCA
HLKAWQRGQTGYPLVDAAMRQLWLTGWMNNYMRHVVASFLIAYLHINWQAGYRWFQDTLVDADVAIDAMMWQN

GGMCGLDHWNFVMHPVDAALTCDPYGNYVRQWCPELKALPDDFIHKPWKCPSSVLCRAGVVFGQNYPERIVVD
LEERRAQSLQDVTLVRKRFSEYVCQQTGCDLVPLPAKLVQDAMGSGNDIVRHGEKEFLLPVITRMEFKHEPED
TDTHDNPYSAVLKGYVSRHRDEKIAFLNERDFTASVMYENAQRRERLERDHCILEGLQCPPTNKGKGQHRNVR
PASGAVQKR
>Colossoma_macr__XP_036440837.1
MVVNTVHWFRKGLRLHDNPSLRDSIAGADTLRCVYILDPWFAGSSNVGINRWRFLLQCLEDLDASLRKLNSRL
FVIRGQPTDVFPRLFKEWKITRLSYEYDSEPFGKERDAAIKKLASEAGVEVTVRISHTLYDLDKIIELNGGQS
PLTYKRFQTLISKMDAVETPAETITAEIMGKCATPISDDHDEKFGVPSLEELGFETEGLSSAVWPGGETEALT
RLERHLERKAWVANFERPRMNANSLLASPTGLSPYLRFGCLSCRLFYFKLTDLYRKVKKNSIPPLSLYGQLLW
REFFYTAATNNPRFDKMEGNPICVQIPWDRNPEALAKWAEGRTGFPWIDAIMTQLRQEGWIHHLARHAIACFL
TRGDLWISWEEGMKVFEELLLDADWSVNAGSWMWLSCSSFFQQFFHCYCPVGFGRRTDPNGDYIRRYLPILRG
FPAKYIYDPWNAPEGVQKLAKCIIGVHYPKPMVNHAEASRINIERMKQIYQQLSCYRGLGLLATVPSNSGGNG
DGAGAGSGDSAQESSAAVHLTPANPREDLSASTFSCSHSDQQHGIIVQPQNSFSGTGRVGSHRQDSKQTLRGR
GTVPMKRHNEEPASSSGCKIQRQNSN
>Colossoma_macr__XP_036450149.1
MKHKCIHWFRKGLRLHDNPALKAALHDCEHIYPVFILDPWFPKNAQVGINRWRFLVEALKDLDSSLKKLKSRL
FVVRGTPREVFPKLFEEWKVTRLTFEVDTEPYSQIRDKEVMDLAKQHGVEVITKISHTLYNIERIIEQNNGKP
PLTYVRFQTVLKDMGPPKRPVPAPTREDMKGILTPSSDKHDEIYGIPTLEELGQDSSTLGPDLYPGGEQEALR
RLDEYMQRTGWVCSFEKPQTSPNALSPSTTVLSPYVKFGCLSARTFWWRLAEVYQGRKHSVPPVSLHGQLLWR
EFFYTAAVGIPNFSKMEGNPNCVQVDWDSNPEHLAAWREARTGFPFIDAIMTQLWQEGWIHHLARHAVACFLT
RGDLWISWEEGQKVFEDLLLDADWSLNAGNWQWLSASAFFHQYFRVYSPIAFGKKTDKNGDYIKKYLPVLKKF
PPEYIYEPWKAPKSVQQQAGCIVGKDYPRPIVDHDVVSKRNIQRMKAAYAKHSRDPTHSQTESKGQKRKSASI
KEMFTKKAKK
>Colossoma_macr__XP_036453411.1
MLCTQRRASAGRRAVNLIASAALLSSLELRAALLTFLPRRAASMYARRAELKRKAEALSSAVEGKKQKAEEKS
GGERGERGGRQAGWLQLEVDELRARNTGCKFNAKRVRFLSENQKVPQGCGGVLYWMARDQRVQDNWAVVYAQQ
LALAEKLPLHICFCLVPRYLDAAYRQYAFMIRGLQKVAKECKSLDIQFHLLRGEPELTLPCFVKSWNFGAVVT
DFNPLRLPLQWTEKVKKELPADVPFMQVDAHNVVPCWEASPKLEYGARTIRGKITKLLPEFLTEFPPVDVHPH
PSTKTAKAVDWEEVLSSVEVDRTVDEVKWAQPGTAGGMAMLESFIQHRLRLFATERNNPNSAALSQLSPWLHS
GQLSAQRVVKEVQRWGKNARESVASFTEELVVRRELADNFCFYNKNYDSIDGAYDWAKKTLKDHAKDKRPYLY
TQEQLENARTHDQLWNAAQRQLLLEGKMHGFMRMYWAKKILEWTSSPEEALAIAIYLNDHYALDGCDPNGYVG
CMWSICGIHDQGWAERAVFGKVRYMNYAGCKRKFDVGQFERRYTVKTA
>Columba_livia_AYE53929.1_cryptochro
MGAAAPGPAPAVCCSGHWFRRGLRLHDNPALHEALRDAASLRCIYILDPWFAASSAVGINRWRFLLQSLEDLD
NSLRKLNSRLFVVRGQPTDVFPRLFKGWGVTRLTFEYDSEPFGKERDAAIIKLAKEAGVEVVIENSHTLYDLD
RIIELNGHKPPLTYKRFQAIISRMELPKKPVSTIMSQQMEACKVDIQENHDDVYGVPSLEELGFPTDGLAPAV
WQGGETEALARLDKHLERKAWVANYERPRMNANSLLASPTGLSPYLRFGCLSCRLFYYRLWELYKKVKRNSTP
PLSLYGQLLWREFFYTAATNNPKFDRMEGNPICIQIPWDRNPEALAKWAEGKTGFPWIDAIMTQLRQEGWIHH
LARHAVACFLTRGDLWISWESGVRVFDELLLDADFSVNAGSWMWLSCSAFFQQFFHCYCPVGFGRRTDPSGDY
VKRYLPKLKGFPSRYIYEPWNAPESVQKAAKCIIGVDYPKPMVNHAETSRLNIERMKQIYQQLSRYRGLCLLA
SVPSCVEDLSGPVTDSASGQGCSTSTAMRLSQADQSSPKRKHEGAEELCTEELYKRARVTDLPTAEIPGKSV
>Columba_livia_NP_001302444.1_cryptochro
MGVNAVHWFRKGLRLHDNPALRECIQGADTVRCVYILDPWFAGSSNVGINRWRFLLQCLEDLDANLRKLNSRL
FVIRGQPADVFPRLFKEWNITKLSIEYDSEPFGKERDAAIKKLASEAGVEVIVRISHTLYDLDKIIELNGGQP
PLTYKRYQTLISRMEPLEMPVETITPEVMEKCTTPVSDDHDEKYGVPSLEELGFDTDGLPSAVWPGGETEALT
RLERHLERKAWVANFERPRMNANSLLASPTGLSPYLRFGCLSCRLFYFKLTDLYKKVKKNSSPPLSLYGQLLW
REFFYTAATNNPRFDKMEGNPICVQIPWDKNPEALAKWAEGRTGFPWIDAIMTQLRQEGWIHHLARHAVACFL
TRGDLWISWEEGVKVFEELLLDADWSVNAGSWMWLSCSSFFQQFFHCYCPVGFGRRTDPNGDYIRRYLPVLRG
FPAKYIYDPWNAPESIQKAAKCIIGVNYPKPMVNHAEASRLNIERMKQIYQQLSRYRGLGLLATVPSNPNGNG
NGGLMGYSPGESISGCGSTGGTQLGTGDGHAVVQPCALGDSHTGASGVQQQGYCQASSILHYAHGDNQQSHLL
QAGRTALGTGISAGKRPNPEEETQSVGPKVQRQSTN
>Columba_liv_XP_005509179.2_cry_DASH-l
MSGTAICLLRCDLRAHDNQVLHWAQSSADFVVPLYCFDPRHLLRTQRCGFPKTGPHRLRFLLESVKDLRETLK
KKGSTLVVRKGKPEDVVRDLITQLGSVSAVAFHEEATQEELDVEQGLRQVCSQHGVKIQTFWGSTLYHRGDLP
FRPIARLPDVYTHFRKAVESEAKVRPTLRMADQLKPLAPGVEEGCIPTMEDLGQKDPVTDPRTAFPCSGGETQ
ALMRLQYYFWDTNLVASYKETRNGLVGMDYSTKFAPWLALGCISPRYIYEQIRKYEKERTANQSTYWVLFELL
WRDYFRFVALKYGRRIFSLRGLQSKEVSWKKDLELFNCWKEGKTGVPFVDANMRELAATGFMSNRGRQNVASF
LTKDLGLDWRMGAEWFEYLLVDYDVCSNYGNWLYSAGIGNDPRDNRKFNMIKQGLDYDGNGDYVRLWVPELQG
IKGADIHTPWALNSAALAQAGVTLGETYPPPVVTAPEWSRHINQRPQGRNPHPRGRTGPAHPPAPHKDRGVDF
YFSRKKDAR

>Columba_liv_XP_005510394.1_cry-1
MPHRTIHLFRKGLRLHDNPTLLAALESSETIYPVYVLDRRFLASAMHIGALRWHFLLQSLEDLHKNLSRLGAR
LLVIQGEYESVLRDHVQKWNITQVTLDAEMEPFYKEMEANIRRLGAELGFEVLSRVGHSLYDTKRILDLNGGS
PPLTYKRFLHILSQLGDPEVPVRNLTAEDFQRCMSPEPGLAERYRVPVPADLEIPPQSLSPWTGGETEGLRRL
EQHLTDQGWVANFTKPRTIPNSLLPSTTGLSPYFSMGCLSVRTFFQRLSNIYAQAKHHSLPPVSLQGQLLWRE
FFYTVASATQNFTQMAGNPICLQIHWYEDAERLHKWKTAQTGFPWIDAIMTQLRQEGWIHHLARHAVACFLTR
GDLWISWEEGMKVFEELLLDADYSINAGNWMWLSASAFFHHYTRIFCPVRFGKRTDPEGQYIRKYLPVLKNFP
TKYIYEPWTASEEEQRQAGCIIGRDYPFPMVNHKEASDRNLQLMRRVREEQRGTAQLTRDDADDPMEMKRDCS
EENTARGKVARGRE
>Columba_liv_XP_005513288.1_photolyase
MRRSRGKRKAEAAKLPRVSRRRKEEEEDAVQEARRRTAPSVREFKYNKKRVRLVSQGSDLKEDARCILYWMFR
DQRVQDNWAFLYAQRLALKQELPLHVCFCLVPKFLDATIRHYDFMLKGLQEVTEECAELNIPFHLLLGYPKDV
LPAFVVEHGVGGLVTDFSPLRLPRQWVEDIRERLPEDVPFAQVDAHNIVPCWVASPKQEYSAWTFRGKINSQL
PEFITEFPPVICHPYPPSCPAEPIAWEACYSSLQVDRTVKEVEWATPGTAAGLAVLQSFIAERLKYFGSHRNN
PNKEALSNLSPWFHFGQVSTQRAILEVQKQRGKYKESVDVFVEEAVVRRELAENFCYYNENYDSVQGAYDWAQ
TTLKLHAEDKRPFLYKLQELEQGTTHDPLWNAAQLQMVQEGKMHGFLRMYWAKKILEWTRSPEEALQFAIYLN
DRYELDGRDPNGYVGCLWSICGIHDHGWTERAVFGKIRYMNYAGCKRKFDVGQFERRYTPHTPSQ
>Colwellia_psy_WP_011042217.1_photolyase
MLLWLRNDLRTHDNPALHYFLSRNALSQHDSAVPAKAIFFICEQQWQQHDWSPIKIDFIKRHAHALVKELALL
NIELELIELDNFTQQKTYLHSYCQLHNITEVVANSEVEFNEQQRDKALYLELKASGIPLTLLEADVIVPKGKV
LNQSGIMYKVFTPFKRAWLTYIQQGGFEYLGKVSVKSSAVNKADNQEEDQGSISSTWPLATVFEQQILPKFYQ
QKISTYKLNRDIPSIKGTSGISPYLAAGVISPRFVLRLLLNKHPDLLVASDSEEFSWLNEIIWREFYRHLLFH
EQRLCKHQCYKENYQEMLWHNDAALFDAWCQGRTGYPLVDAAMRQLNQTGWMHNRLRMVVASFLTKHLLIDWR
LGEKYFMQHLIDGDLASNNGGWQWAASTGCDAQPYFRIFNPIRQSERFDPKGVFIRKYIPELNNISDKAIHFP
HQYIKDNELNIYWPAIVEHKEARLKALAFYKV
>Condylura_cris__XP_004675775.1
MGVNAVHWFRKGLRLHDNPALRECIQGADTIRCVYILDPWFAGSSNVGINRWRFLLQCLEDLDANLRKLNSRL
FVIRGQPADVFPRLFKEWNITKLSIEYDSEPFGKERDAAIKKLATEAGVEVIVRISHTLYDLDKIIELNGGQP
PLTYKRFQTLISKMEPLEIPVETITSEVIEKCTTPLSDDHDEKYGVPSLEELGFDTDGLSSAVWPGGETEALT
RLERHLERKAWVANFERPRMNANSLLASPTGLSPYLRFGCLSCRLFYFKLTDLYKKVKKNSSPPLSLYGQLLW
REFFYTAATNNPRFDKMEGNPICVQIPWDKNPEALAKWAEGRTGFPWIDAIMTQLRQEGWIHHLARHAVACFL
TRGDLWISWEEGMKVFEELLLDADWSINAGSWMWLSCSSFFQQFFHCYCPVGFGRRTDPNGDYIRRYLPVLRG
FPAKYIYDPWNAPEGIQKVAKCLIGVNYPKPMVNHAEASRLNIERMKQIYQQLSRYRGLGLLASVPSNPNGNG
GLMSYSPGENIPACSSSGSCPQGSGILHYAHGDSQQTHLLKQGRSPLGTGLSSGKRPNQEEETQSSKVQRQST
N
>Condylura_cris__XP_004682938.1
MATAAVTAAAVSPAPAAVAEGASSVHWFRKGLRLHDNPALLAAVRGARCVRCVYILDPWFAASSSVGINRWRF
LLQSLEDLDTSLRKLNSRLFVVRGQPADVFPRLFKEWGVTRLTFEYDSEPFGKERDAAIMKMAKEAGVEVVTE
NSHTLYDLDRIIELNGQKPPLTYKRFQAIISRMELPKKPVGSVTSQQMEGCRADIQENHDETYGVPSLEELGF
PTEGLGPAVWQGGETEALARLDKHLERKAWVANYERPRMNANSLLASPTGLSPYLRFGCLSCRLFYYRLWDLY
KKVKRNSTPPLSLFGQLLWREFFYTAATNNPRFDRMEGNPICIQIPWDRNPEALAKWAEGKTGFPWIDAIMTQ
LRQEGWIHHLARHAVACFLTRGDLWVSWESGVRVFDELLLDADFSVNAGSWMWLSCSAFFQQFFHCYCPVGFG
RRTDPSGDYVRRYLPKLKGFPSRYIYEPWNAPESVQKAAKCVIGVDYPRPIVNHAETSRLNIERMKQIYQQLS
RYRGLCLLASVPACVEDLSNPVAEPSSSQAGSTSSAGPRPLPSGAASPKRKLEAEEEAPGEELSKRARVAGLP
TPEAPGKDV
>Coregonus_sp._'balchen'_CAB1313618.1_unnamed_pr
MVGNTIHWFRKGLRLHDNPSLKESIRGADTLRCVYILDPWFAGSSNVGISRFLLQCLEDLDASLHKLNSCLFV
IRGQPTDVFPRLFKEWQINRLFYEYDSEPFGKERDAAIQKLASEAGVEVTLNRGQSPLTYKCFQVLISHMDAV
EMPAETITAEVMRKCATPISDDHDDKFGVPSLEELGFETEGLATAVWPGGETEALTRLERHLERKAWVANFER
PRMNVNSLLASPTGLSPYLRFGCLSCRLFYFKLTDLYRKVKKNSSPPLSLYGQLLWREFFYTTATNNPCFDKM
EGNLVCVQIPWDRNPEALAKWAEGRTGFPWIDAIMTQLRQEGWIHHLARHAVACFLTRGDLWISWEEGMKVFE
ELLLDADWSVNAGSWMWLSCSSFFQQFFHCYCPVGFGRRTDPNGDYIRRYLPILKGFPAKYIYDPWNAPESVQ
KAAKCVIGVHYPKPMVHHAEASRLNVERMKQIYQQLSCYRGLGLLATVAANPNNGGHGAGVTSRMREPLKQQQ
GPEADQQLVPEEEKEEGIPATLLSSRVDQRLNIASSFPDSSTLQEHIYLRGVSSNRNGRGEETASIPQTLSDF
CEVSWLNGTSDHMMPRELIAASLSVTFSLCGESQTQCSNHKPTVTLSPVLLER
>Coregonus_sp._'balchen'_CAB1322736.1_unnamed_pr
FLLESLEDLDSSLRKLNSRLFVVRGQPANVFPSLFKEWNVTRLTFEYDSEPYGKERDGAIIKMAQEFGVETIV
RNSHTLYNLDRIIELNNCPPLTVKNHPPPLTFKRFQALVNRLELPMKPLPTITQEQMDSCRTKIADNHDEHYS
VPSLEELGFKTQGLNPAVWKGGESEALERLNKHLDRKAWVANFERPRINMCSLIASPTGLSPYLRFGCLSCRV
FYYNLRELYMKLRKHCSPPLSLFGQLLWREFFYAAGTNNPNFDRMEGNPICVQIPWDHNPEALAKWAEGSTGF

PWIDAIMTQLRQEGWIHHLARHAVACFLTRGDLWISWESGMRVFEELLLDADWSVNAGSWMWLSCSAFFQQFF
HCYCPVGFGRRTDPSGDYIRRYIPKLKDYPNRYIYEPWRAPESVQKAAKCIVGVDYPQPMINHAEGSRLNIER
MKQVYQQLSHYRGLSLLASVPTIQEEAEPPPTDDSQASSSTCDSPPHTSSAQSNTLKRGRPSEPQSTQTCTHT
KHLHTSPSKDQEQQWEEQSEHQPVDLQ
>Coregonus_sp._'_CAB1354074.1
MMAKQTGTQAKHYQCLEDLHRSLQALGSRLYVLQGPYQGTVMRLVAQWGITQLSMDTEIEPHYTQLDQQHRIM
ARTCPSPAAEEYSEGDYRAPSGWVANFSKPRTIPNSLLPSTTGLSPYLSLGCLSVWAFYHKLSKIYAQSKNHS
LPPVSLQGQVLWREFFYTVASATANFIKKEGNSICL
>Coregonus_sp._CAB1313746.1_unnamed_pr
MPVSGGEDPMSQVRQMLRELLVGRENADGFFCLCVSVLGHNDTRTHFLPLIQPLATVHNGLHTTLTSIYFEYF
SKDEDDELAVALALSLLEVKGQQQTDTKSLFPDPKPQGQTDTQATATYRPQNSSVQSQSVSHNGSSSQPPPAP
LPKGVSYAQLAAAGGRRQTQAQHQDSTSPRGSSGHGSSPQTERQRGPTQTQRQTDIKWSNLTQDVCASIASSL
TSNLNQTKDDVNVEEYDVDQSEKPKRSKNRRQRRKGYGQQVVGVPRCPSAPPPVLLWFRRDLRLHDNPAVIGS
LEAGGPVIPVFIWCPEEEEGPGVTVAMGGACKFWLHQALSCLSSALERIGGHLVFLRPDVEKEVEREGVGSSL
RALRSLVRETGAQTVLASALYEPWLRERDQIVVSALQRDRVEVNMVHSYCLRDPYTVTTEGVGLRGIGSVSHF
MSCCQMNPGPGLGVPLDPPISLPSPSVWPRGCPLEELGLARMPCRKDGTTIDWAANIRSSWDFSEEGAQSRLE
AFLNDGVYKYEKESGRADAPNTSCLSPYLHFGQLSARWLLWDAKGARCRPPKFIRKLAWRDLAYWQLTLFPDL
PWESLRPSYKARIGERGHLKAWQRGRTGYPLVDAAMRQLWLTGWMNNYMRHVVASFLIAYLHLPWQEGYRWFQ
NGGMCGLDHWNFVMHPVDAAMTCDPYGSYVRKWCPELAALPDDLIHKPWKCPASMLRRAGVVLGQSYPERVVT
DLEERRSQSLQDVALVRRRCGEYVDPCSGCDLVPLPPRLVSEALGGGVVNTGGQFLLPVITRMEFKHQSDNPD
ADAASNPYNAVLKGYVSRRRNETIAFLNQTDFTASVINEGAERRERQEKDQRRMEGLPHPLAARGRGRRTPAA
KDRFSTVPGGVATSHR
>Coregonus_sp._CAB1317817.1_unnamed_pr
MHRSLLLIPASKHIWKFVTLESQRHCYYSSLTKLVGLSSINNRTTMPAKKRKATFAKMDNAKQQKLQAVKEGE
REREKGWLQGVVAEQRKEKQDLKFNKKRLRFLSQTQKIKQGSEGVVYWMNRDHRVQDNWALIHAQQLALEEKL
PLHICVCLDCSALDIQFHLLQGSAGELLPGFVRDWILGAVVTDFSPLRTPLQWVEEVDAHNVVPCWVASDKLE
YAARTIRTKITKLLPEFLTEMPLVDKHPHPAVRTAKPVDWAEVLSSLEVDRTVEEVDWAKSGTAAGIAMLESF
IDLRLKNFATQRNNPNSSAVSQLSPWIRFGHLSAQRVVLQVKRSGKSAAESVSSFTEELVKTLKDHAKDIRPY
VYTRKQLEKAQTHDKLWNAAQYQMVIEGKMHGFLRMYWAKKILEWTSSPEGALSIAIYLNDRYELDGQDPNGF
VGCMWSICGIHDQGWAERPIFGKIRYMNYKGCTRKFDVAQFERKYCPKDL
>Coregonus_sp._CAB1341255.1_unnamed_pr
MDKTRRGEPKEKGLEREKGKSGGQCGERDEGSHRKKPGQEEEKKRGRREMETALIGLHPSSPLVPGCKADPSP
RPHSSVLAARLSFTVLLPCLLDSLVDQSDRPSGTINRLVDIREPSVDPQKPSRTDLVKPINHQNRLDRLVDHQ
DFYSEAIRTIRPSSGSIDQSDRPWTLSPSDRLVNMSTSRTVICLLRNDLRLHDNEVFHWAQRNAEHIVPLYCF
DPGHYLGTANYNLPRTGPFRLRFLLDSIQDLRTSLIQRGSTLVVRRGRPEEVVSDLIKQLGSVSAVAFHEEMK
VKVHTCWGSTLYHRDDLPFNHLSRLPDVYTQFRKAVETQGRVRPVLPTLDQLKPLPPGSGLDEGPIPSPEDLE
QTDTDAVATYKETRNGLMGMDYSTKFSPWLAIGCISPRYIYQQIKKYESERTANQSTYWVVFELLWRDYFKFV
GAKYGDRLFDIKGLQDKSIPWKKDMNLFNAWKEGRTGVPFVDANMRELALTGFMSNRGRQNVDHDVSSNYGNW
LYSAGIGNDPRENRKFNMIKQGLDYDNNVCFSLTNNTVELPQLYLKGDYVRFWVQELQGIRGGDVHTPWTLSS
ASLSHALVTLDETYPSPIVMVPEWSRHANKKPTGAGPSARGKKGPSHTPKQHKDRGIDFYFSKSKNF
>Coregonus_sp._CAB1352867.1_unnamed_pr
MLEKLDQDPEELFPGGEQEALRRLDQHMERKEWVCGFEKPQTSPNSLSPSTTVLSPYMTFGCLSARTFWWRLT
DVYQGKKHSQPPVSLHGQLLWREFFYTAGLGIPNFDKMEGNPVCTQARTGFPFIDAIMTQLRQEGWIHHLARH
AVACFLTRGDLWISWEEGQKVFEELLLDGDWSLNAGNWQWLSASTFFHQYFRVYSPIAFGKKTDKNGDYIKNY
LPLLKKYPAQYIYEPWKAPRSVQEAAGCIVGKDYPRPIVAHEVISKKNIQRMKAAYAKRSPHSSEESPGKEKG
LFPLGTDIGSASPNLNHRIG
>Coryphaena_hipp__AZB52138.1
ESFIDVRLKLFGTQRNNPNAAALSQLSPWIRFGHLSAQRVALQVKHAGKNASESTSAFIEELVVRRELTDNFC
FYNKKYDSVEGAYEWAKKTLKDHCTDKRPYIYTRQQLEKAKTHDKLWNAAQYQMVTEGKMHGFLRMYWAKKIL
EWTTSPEEALSIALYLNDRYELDGQDPNGFVGCMWSICGIHDQG
>Coryphaenoides_rupe__KAG7255536.1
FLLQSLEDLDASLRKLNSRLFRPAHDVFPRLFKEWGDQRLSYEYDSEPFGKERDAAIKKLACEAGVEVTVRIS
HTLYDLDKIIELNGGQSPLTYKRFQTLISRMEPVEVPADAITADAMWKCTTPLSGRPRRQVRRPLPGGSWVSF
DTEGLPSAVWPGGETEALTRLERHLERKAWVANFERPRMNANSLLASPTGLSPYLRFGCLSCHLFYFKLTDLY
RKVKKNSSPPLSLYGQLLWREFFYTAATNNPCFDKMEGNPICMQIPWDRNAEALAKWAEGQTGFPWIDAIMTQ
LRQEGWIHHLARHAVACFLTRGDLWISWEEGMKVFEELLLDADWSVNAGSWMWLSCSSFFQQFFHCYCPVGFG
RRTDPNGDYIRRYLPYIYEPWNAPECVQKAAKCVVGVHYPKPMVNHAEASRLNIERMKQIYQQLSCYRGLGLL
ATVPSNTNGSGEASSGMLGFPMEPAVAATTQNSASAPSGYQMVAAAPSQGDWQSAVMTYAPDEAQGGLGGAQQ
QGYSVPGNSLMYYTQSAQQVLPAVTQGARHHVQSIGKRHSEDAGSGSSSKFQRQCSH
>Coryphaenoides_rupe__KAG7259523.1

MCTPARTVICLLRNDLRLHDHEVLHWAQSNAEHIVPLYCFDPRHYLGTSVYNLPKTGPFRLRFLLDSVKDLRS
SLVASEERAVERAVKDVCVQMKVKVHTCWGATLYHRDDLPFPHLARLPDVYTQFRKAVETQSRVRPLIVSPDQ
LRPLPPGLEEGALPTLEDLGQSEPVADPRSAFPCRGGESQALARLQHYFWDTDAVATYKETRNGLIGTDYSTK
LAPWLAMGCLSPRYVHHQIQQYESQRTANQSTYWVIFELLWRDYFKFVGVKYGNRLFHIRGLQDKSIPWKKDM
ALFNAWKEGRTGVPFVDANMRELAMTGFMSNRGRQNVASFLTKDLCLDWRMGAEWFEYLLVDHDVCSNYGNWL
YSAGLGNDPRENRKFNMIKQGLDYDSNGDYVRRWLPELRGVGGGDVHTPWTLSSAALAHAQVSLGDGYPVPVV
MAPEWSRHAGRKPSDSGSSTRGRKGPSHTPKRHGDRGVDFYFSKSKNL
>Coryphaenoides_rupe__KAG7269861.1
MPSDKRKSAPCGEAPHAKKQKKFPPLTEERRERPEGWLQTEVEKQRAAEAGVQCNHKRTRFISDAQKVKQGSE
GILYWMTRDHRVQDNWALIHAQQLAMEEEQPLHVCVCLLVPESALSTLRHYSFMLKGLEEVAKECKALDIQFH
LLRGAPAEVLPGLVSDRGLGAVVTDFLPLRTPRQALESLKKSLPDDVPLVQVDAHNIVPCWVASPKLEYSART
IRGKITKLLPEFLTDFPVVGKHLYTATRTDKPVDWAEALGSLRLDGAVGEVAWARPGAATGTAMLESFIDLRL
RHFATHRNDPNSAALSQLSPWIRFGHLSAQRVALQVQRFGKKFSQSVAPFIEELVVRRELTDNFCFYNDNYDN
VKGAYEWAQKTLKDHAKDKREYYQMVTEGKMHGFLRMYWAKKILEWTASPEQALSIALYLNDRYSLDGQDPNG
FVGCMWSICGIHDQGWAERPVFGKIRYMNYKGCLRKFDVAEFETKYCPKKL
>Coryphaenoides_rupe__KAG7271508.1
MKRLSCSTGGGGGGGGGGGGGGCSTSSSGPECHAYSAGMAPACSIHWFRKGLRLHDNPALMSALRDCQELYPLF
ILDPCVHNKEAMGANRWRFLLGALRDLDGGLRKLNSRLFVGAPQDVLPGLFREWGVSRLTYEYDTEPYSRRRD
QAVGALARDHGVEVVYRISHTLHNIDRIIEENNGKAPLTYVRFQAVLKNLGPPKRPIPAPNLQDMKDVRTPCP
AESGDEQQQKYGLPTLEELGHDLSCLQEALFPGGEEEALRRLEEHMERTAWVCSFEKPQTSPNSLSPSTTVLS
PYVTFGCLSARTFWWRLTDVYQGKKHSAPPVSLHGQLLWREFYYTASVGVPNFHRMLGNPVCTQIDWDTNPEY
LAAWREVFEELLLDGDWALNAGNWLWLSASAFFHQYFRVYSPVAFGKKTDKHGDYIKKYLPVLKKFPAEYIYE
PWKAPRSVQEVAGCIVGKDYPRPIVEHEVISKKNIQRMKTSYAKRSQGVSESPQPQKEE
>Coryphaenoides_rupe__KAG7282122.1
MVVNSVHWFRKGLRLHDNPSLQEALIGADSVCCVYILDPWFAGSANVGLGINRWRFLLESLEDLDNSLRKLNS
RLFVVRGQPTDVFPRLFKEWNVTRLTFEYDPEPFGKERDGAIIKVAHELGVETIEWNVTRLTFEYDPEPFGKE
RDGAIIKVAHELGVETIVRNSHTLYNLDRIVEMNNNNPPLTFKRFQTIVNRLDLPRRPLPTVTAQQMERCKTR
ISKDHDRHYSVPSLEELAGLSPFLRFGCLSCRLRKRCSPPLSLFGQLLWREFFYTAATNNPSFDRMEGNPICV
QIPWDQNPEALAKWAEGRTGFPWIDAIMTQLRREGWIHHLARHAVFEELLLDADWSVNAGSWMWLSCSAFFQQ
FFHCYCPVGFGRRTDPSGDYIRRYIPILKDYPNRYIYEPWSAPLSVQKAANCVVGVDYPKPMINHAEASRLNI
ERMKQVYQQLSHYRGLNSPVHEPAARPLVPDSSTARTSSPAADADASHAAAHTQPPQTPPSSSASTHTSTFPA
FSPTPAIASVTKEPATVFLPPPQIPFPSSHAAASSQSPARDPAATPGPTPVPDLSHVTNPFVALSPRPVTLAA
AGQHAAFLGHRKKGQPRKHRRTQRSRGLLGGAPAGKADGGAKAAGRAGEGEEEHMEEDVVFSRTG
>Corythaeola_cris__NXC13099.1
KVDYDVCSNYGNWLYSAGIGNDPRDNRKFNMIKQGLDYDGNGDYIRLWVPELQGIKGADIHTPWALNSAALSQ
AGVTLGETYPQPVVTAPEWSRHTNQRPQGRSPHPRGRRGPAHTPVQHRDRGIDFYFSRKKDV
>Corythaeola_cris__NXC14474.1
MGVNAVHWFRKGLRLHDNPALRECIQGADTVRCVYILDPWFAGSSNVGINRWRFLLQCLEDLDANLRKLNSRL
FVIRGQPADVFPRLFKEWNIAKLSIEYDSEPFGKERDAAIKKLASEAGVEVIVRISHTLYDLDKIIELNGGQP
PLTYKRFQTLISRMEPLEMPVETITPEVMEKCTTPVSDDHDEKYGVPSLEELGFDTDGLPSAVWPGGETEALT
RLERHLERKAWVANFERPRMNANSLLASPTGLSPYLRFGCLSCRLFYFKLTDLYKKVKKNSSPPLSLYGQLLW
REFFYTAATNNPRFDKMEGNPICVQIPWDKNPEALAKWAEGRTGFPWIDAIMTQLRQEGWIHHLARHAVACFL
TRGDLWISWEEGMKVFEELLLDADWSVNAGSWMWLSCSSFFQQFFHCYCPVGFGRRTDPNGDYIRRYLPVLRG
FPAKYIYDPWNAPESVQKAAKCIIGVNYPKPMVNHAEASRLNIERMKQIYQQLSRYRGLGLLATVPSNPNGNG
NGGLMSYSPGESISGCGSTGGHLFPSSPGAQLGTGDGHTVVQPCPLGDSHTGASGIQQQG
>Corythaeola_cris__NXC18833.1
DNWAFLYAQRLALKQELPLRVCFCLVPKFLGATIRHYGFMLRGLQEVAEECAELNIPFHLLLGYAKDVLPPFV
VEHGVGGLVTDFCPLRLPRQWVEDVRERLPEDVPFAQVDAHNIVPCWVASPKQEYSASTIRGKIHAQLPEFLT
EFPPVICHPYPLSCLAEPIAWEASYSSLQVDRTVKEVEWATPGTAAGLAVLQSFIAKRLKSFGSHRNNPNKEA
LSNLSPWFHFGQVSTQRAILEVQKHRGKYKESVDMFVEEAVVRRELAENFCYYNENYDSVQGAYSWAQTTLKL
HAKDKRPFLYELQELEQGTTHDPLWNAAQLQMVREGKMHGFLRMYWAKKILEWTRSPEEALRFAIYLNDRYEL
DGRDPNGYTGKLQEGGCLWSICGIHDHGWTERAVFGKIRYMNYAGCKRKFDVGEFERRYAPDM
>Corythaeola_cris__NXC19319.1
MPHHTIHLFRKGLRLHDNPTLLAALESSETVYPVYILDRKFMTTVMHIGALRWHFLLQSLEDLQKNLCQLGSC
LLVIQGEYESVLRDHVQKWNITQVTLDAEMEPFYKEMEANIRRLGEELGFEVLSLVNHSLYDTKRILDLNGGT
PPLTYKRFLHILSLLGDPEVPVRNLMVEDFQRCSPPDPGLAECYRVPLPVDLKIPLESFSPWRGGETEGLQRL
EQHLTDQGWVASFTKPRTIPNSLLPSTTGLSPYFSLGCLSVRTFFYRLSNIYAQAKHHSLPPVSLQGQLLWRE
FFYTVASATPNFTKMAGNPICLQISWYEDAERLHKWKTAQTGFPWIDAIMTQLRQEGWIHHLARHAVACFLTR
GDLWISWEEGMKVFEELLLDADYSINAGNWMWLSASAFFHQYTRIFCPVRFGKRTDPEGQYIRKYLPILKNFP

SKYIYEPWTASEEEQKQAGCIIGQDYPFPMVNHKEASDHNLQLMKQVREEQYGTAQLTRDDTDDPMEMKVKRD
HSEDNISKGKVARMRE
>Corythaeola_cris__NXC19504.1
RFLLQSLEDLDNSLRKLNSRLFVVRGQPTDVFPRLFKEWGVTRLTFEYDSEPFGKERDAAIVKLAKEAGVEVV
IENSHTLYDLDRIIELNGHKPPLTYKRFQTIISRMELPKKPVSTVISQQMETCKVDIQENHDDVYGVPSLEEL
GFPTDGLAPAVWQGGETEALARLDKHLERKAWVANYERPRMNANSLLASPTGLSPYLRFGCLSCRLFYYRLWE
LYKKVKRNSTPPLSLYGQLLWREFFYTAATNNPKFDRMEGNPICIQIPWDRNPEALAKWAEGKTGFPWIDAIM
TQLRQEGWIHHLARHAVACFLTRGDLWISWESGVRVFDELLLDADFSVNAGSWMWLSCSAFFQQFFHCYCPVG
FGRRTDPSGDYVKRYLPKLKGFPSRYIYEPWNAPESVQKAAKCIIGVDYPKPMVNHAETSRLNIERMKQIYQQ
LSRYRGLCLLASVPSCVEDLSGPVTDSASGQGCSTSTAVRLSQADQASPKRKHEGAEELCTEELYKRAKVTGL
PTPEIPGKSL
>Corythaixoides_conc__NXJ87972.1
MGVNAVHWFRKGLRLHDNPALRECIQGADTVRCVYILDPWFAGSSNVGINRWRFLLQCLEDLDANLRKLNSRL
FVIRGQPADVFPRLFKEWNIAKLSIEYDSEPFGKERDAAIKKLASEAGVEVIVRISHTLYDLDKIIELNGGQP
PLTYKRFQTLISRMEPLEMPVETITPEVMEKCTTPVSDDHDEKYGVPSLEELGFDTDGLPSAVWPGGETEALT
RLERHLERKAWVANFERPRMNANSLLASPTGLSPYLRFGCLSCRLFYFKLTDLYKKVKKNSSPPLSLYGQLLW
REFFYTAATNNPRFDKMEGNPICVQIPWDKNPEALAKWAEGRTGFPWIDAIMTQLRQEGWIHHLARHAVACFL
TRGDLWISWEEGMKVFEELLLDADWSVNAGSWMWLSCSSFFQQFFHCYCPVGFGRRTDPNGDYIRRYLPVLRG
FPAKYIYDPWNAPESVQKAAKCIIGVNYPKPMVNHAEASRLNIERMKQIYQQLSRYRGLGLLATVPSNPNGNG
NGGLMSYSPGESISGCTSTGGHLFPSSPGAQLGTGDGHTVVQPCPLGDSHTGASGIQQQGYCQASSILHYAHG
DNQQSHLLQAGRTALGTGISAGKRPNPEEETQSVGPKVQRQSTN
>Corythaixoides_conc__NXJ92683.1
KVDYDVCSNYGNWLYSAGIGNDPRDNRKFNVIKQGLDYDGNGDYIRLWVPELQGIKGADIHTPWALNSAALSQ
AGVTLGETYPQPVVTAPEWSRHTSQRPQGRSPHPRGRRGPAHTPVQHKDRGIDFYFSRKKDV
>Corythaixoides_conc__NXJ93935.1
RFLLQSLEDLDNSLRKLNSRLFVVRGQPTDVFPRLFKEWGVTRLTFEYDSEPFGKERDAAIVKLAKEAGVEVV
IENSHTLYDLDRIIELNGHKPPLTYKRFQAIISRMELPKKPVSTVISQQMETCKVDIQENHDDVYGVPSLEEL
GFPTDGLAPAVWQGGETEALARLDKHLERKAWVANYERPRMNANSLLASPTGLSPYLRFGCLSCRLFYYRLWE
LYKKVKRNSTPPLSLYGQLLWREFFYTAATNNPKFDRMEGNPICIQIPWDRNPEALAKWAEGKTGFPWIDAIM
TQLRQEGWIHHLARHAVACFLTRGDLWISWESGVRVFDELLLDADFSVNAGSWMWLSCSAFFQQFFHCYCPVG
FGRRTDPSGDYVKRYLPKLKGFPSRYIYEPWNAPESVQKAAKCIIGVDYPKPMVNHAETSRLNIERMKQIYQQ
LSRYRGLCLLASVPSCVEDLSGPVTDSASGQGCSTSTAVRLSQADQASPKRKHEGAEELCTEELYKRAKVTGL
PSPEIPGKSL
>Corythaixoides_conc__NXJ95526.1
MPHHTIHLFRKGLRLHDNPTLLAALQSSETVYPVYILDRKFMTTAMHIGALRWHFLLQSLEDLQKNLCQLGSC
LLVIQGEYESVLRDHVQKWNITQVTLDAEMEPFYKEMEANIRRLGEELGFEVLSLVNHSLYDTKRILDLNGGA
PPLTYKRFLRILSLLGDPEVPVRNLVVEDFQRCRPPDPGLAERYRVPLPVDLKIPLENFSPWRGGETEGLQRL
EQHLTDQGWVASFTKPRTIPNSLLPSTTGLSPYFSLGCLSVRTFFYRLSNIYAQAKHHSLPPVSLQGQLLWRE
FFYTVASATPNFTKMAGNPICLQISWYEDAERLHKWKTAQTGFPWIDAIMTQLRQEGWIHHLARHAVACFLTR
GDLWISWEEGMKVFEELLLDADYSINAGNWMWLSASAFFHQYTRIFCPVRFGKRTDPEGQYIRKYLPILKNFP
SKYIYEPWTASEEEQKQAGCIIGQDYPFPMVNHKEASDHNLQLMERVREEQYRTAQLTRDDTDDPMEMKVKRD
HSEDNVSKGKAARMRE
>Corythaixoides_conc__NXJ96048.1
DNWAFLYAQRLALKQELPLRVCFCLVPKFLDATIRHYGFMLRGLQEVAEECAELNIPFHLLLGYAKDVLPPFV
VEHGVGGLVTDFCPLRLPRQWVEDVRERLPEDVPFAQVDAHNIVPCWVASPKQEYSASTIRGKIHSQLPEFLT
EFPPVISHPYHPSCPAEPIAWEASYSSLQVDRTVKEVEWATPGTAAGLAVLQSFITKRLKSFGSYRNNPNKEA
LSNLSPWFHFGQVSTQRAILEVQKHRGKYKESVDTFVEEAVVRRELAENFCYYNENYDSVQGAYSWAQTTLKL
HAKDKRPFLYELQELEQGTTHDPLWNAAQLQMVREGKMHGFLRMYWAKKILEWTRSPEEALQFAIYLNDRYEL
DGMDPNGYTGKLQEGGHRAGRGCLWSICGIHDHGWTERAVFGKIRYMNYAGCKRKFDVGEFERRYAPGM
>Crassostrea_gig_ANJ02841.1_cry_1
MNTNREEVVVHWFRHGLRLHDNPSLIDGLSECDRFYPVFIFDGEVAGTKTAGYNRFRFLLECLQDLDKNLKAA
GTRLYCFQGQPTDILERLIEEWGVTKVTFEADPEPIWQERDRLVRELLDKKNVQCVEKVSHTLWDPYEIIENN
GGSPPLTFSLFNLVTSTIGPPPRPVEDPDFTDISLPVSQNHDKQFGIPSLEDLNVRPECEEQNKRLVEWLGGE
SKALELLAIRMKHEEKAYENGYVMPNQYHPDLLSPPLSLSAHLRFGCLSVRKFYWSIHDKFEEVKPSMGAPVS
LSAQLMWREYFYTMAINNINYDKMETNPICLNIPWYDNPEHEEKWTQGETGYPWIDAIMKQLRYEGWVHHVAR
HAVSCFLTRGDLWLNWEVGLKVFYKYLLDADWSVCAGNWMWVSSSAFEKVLQCPNCFCPVRYGKRMDPSGEYV
RRYLPVLKDMPLRYLFEPWKAPLPVQQKAKCIVGVDYPKPMVDHQKASKECIQNMKAVKDALMGREIPHCAPS
EEIEARRFSWLPDHTPSGGHCTANFLCDGLKDMN
>Crassostrea_gig_AQM57602.1_cry_2

MSKSSKRKHVVHWFRKGLRLHDNPSLREALKGSSSYRCVYILDPWFAGSSQVGINKWRFLLQCLEDLDTSLRK
LNSRLFVLRGQPTDLFPKIFKEWNITTLSFEEDPEPFGKERDGAIQMLAKEAGVEVIVKTSHTLYDLQKIIAM
NGGSPPLTYKRFQSVLAKMEAPSEPEETINSGFLVKTKTPIAEDHDDKYGVPTLEELGFDTEGLGPAVFHGGE
AEALTRLERHLERKAWVASFERPKMSQQSLFPSQNVLSPYLRFGCLSARLFYWKLRELYRKVKKRKDPPLSLH
GQLLWREFFYTVATNNPNFDRMKDNPLCVQIPWDKNPEALAKWAEGKTGFPWIDAIMMQLRQTGWIHNLARHS
VACFLTRGDLWISWEEGMKVFEELLLDADWSVNAGMWMWLSCSSFFQQFFHCYCPVGFGKRADPTGDFIRTYL
PVLKGYPAKYIYEPWTAPESVQRAAKCIIGEDCPVPMVNHAEVSKLNTGRMKQVYQQLAVYASIASVPKQIHS
EEPYSKHEKAMHSGNHPSRVAMLENTDRGNHSQMSA
>Crassostrea_gig_AQM57607.1_cry-like_p
MESPTRKEILHNFQTGVIDAEECFCMIVSLDGYDSTRQHFLKNIDFLRLTNPKKYNELFTVFANYFEKPPSGA
FLDPIGCSVENGYLTSDFGYELEVELASALSLQDQSESLNSKASYVSVVQEPISKQRNTSSNSERNQPKNVNN
SYSKNVKDDNDSVTSSVKSKKRKNKGSTNRPVVYWFRRDLRLYDNPALFEAASMNVPVILVFLWSESEEDPEG
VVAAGGATKLWLHHALNHLDKSISDRYNNRIIYRKTQSCQREILSLIEETGAKALLINDVYEPFLKQRDDKIC
SELQRKGIECKRFHSYLLHEPGSVSAESVGMRGVGSVTHFMECCRQSDAQPIGHPLDYPPTLPKPDQFPSSSS
LHDLELAKMPRRKDGSIIDWAAPIVRQWDFGEEGAWKALELFLSEGVRKYEKESCRTDHLNTCRISPYLHFGQ
ISPRAVLEEARHMKSPKFLRKLAWRDLSYWLLTLWPDLPSQPTRVHYRDQAWSRDAGHLKAWQRGRTGFPLVD
AAMRQLWLEGWINNYLRHVVASFLISYLRLHWVEGYRWFQDTLLDADVAINAMMWQNGGMSGLDQWNFVMHPV
DAALTCDPDGAYVRKWCPEIAALPNDFIHQPWKCPPSILRRCGIKLGETYPNRVISDLEGAREQSLTDVVNVR
KKHPEFVDRRTGNDLVPLPDGLCVPVITRKEFKYKLHHPEAKDNPHTAVLRGYRSRKRDEAIAFANERDFMAS
AMNESVKLSERRLKATQYEAL
>Crassostrea_gig_XP_011414696.2_cry-1_X1
MKTCAIHWFRKGLRLHDNPALQAACKVADDVKPVFILDPWFANNANVGVNRWRFLLQTLQNLDENLKKINSRL
YIIKGKPADVFPKLFKNWGVSHLTFEEDIEPYALTRDSAIKKLAEEHNVKVTSCVSHTLFDPQRIISKNGGKA
PLTYQRLQTVLSSLGSPPKPVDSPSECKTKTESDHDKKYGVPSLEDLGKSEKECGPLLFPGGETEALRRLESM
MGKKNWVCTFEKPKTAPNSLEPSTTVLSPYLKFGCLSPRMFYYKLQEVYNKAKHTSPPVSLLGQLLWREFYYC
VAVDTPNFDKMEGNPVCKQIPWDTNESYLKAWKEGRTGYPFIDAVMTQLRQEGWIHHLARHSVACFLTRGDLW
INWEEGMKVFEEYLLDADWSLNAGNWMWLSASAFFHQYFRVYSPIEFGKKTDKDGDYIRKYVPQLSKYPTAYI
YEPWKAPLKVQEKAGCIVGKDYPKPIVEHDKIRKKNIERMAQAYNASKTEGGAGAKRKSDSSSSQKKKKAKVK
>Crassostrea_gig_XP_011422754.3_photolyase
MIRALRTFLYTHTHSTSKSCRRTFTLKAALLPPKHLEPFRLNSLCISDLSYSYPTVYWNANCFYLGVRKMSDK
KKSKRKADTEELAKESSKKQKTEVTDSQGQGQNGLIKQITESRKKLCESVAEFKFNKKRIKVLSKVQDFPDGK
NGVVYWMSRDQRVQDNWAMLYAQRLALKMEVPLHVCFCLVPKFLDATIRHYRFILKGLEEVEKECQELGISFH
LLIGHAKDVLPSFVKDHQIGGVITDFSPLRVPAEWVSAVSKALPSDIPLCQVDAHNVVPCWEASPKLEYGART
IRNKIHNQLSQYLTEFPPVSKHPHKPKAASQPVDWSKAEDSLEVDRKVTEVDWAVPGSTAGLNMLESFCHERL
KYFGSDRNNPNKNALSNLSPWIKFGQISVQRCILTVRQYRSKYKESVESYIEEAIIRRELSDNFCFYNKNYDS
IDGAYDWAKQTLKVHADDERKYVYTKEQLEFAKTHDDLWNAAQKQMVLEGKMHGFLRMYWAKKILEWTPSPKV
ALETAIYLNDKYSLDGRDPNGYVGCMWSICGIHDQGWKERDIFGKIRYMNYAGCKRKFDVAAFERKYKSKSIQ
>Crassostrea_gig_XP_011441094.2_cry_DASH-l
MGTKGKTIICLFRNDLRIHDNEVLQWANRNADFVLPMYIFDPRHFGGTYHFGFPKTGPHRTKFLLESIQDLRK
NLKIRGSGLAVRKGKPHEELKKMIDMLGQSSVHSVVFHEEVTQEELDVEKSIKKHCGVNIKTFWGHTLYHSED
LPFSPSQTPDVYTQFRKRVESQAPVRKCLDMPKSFRPLPEGVEEGEIPTNKDLGVEDPVTDPRSVVDFLGGES
VAIDRLHHYLWETDAIATYKETRNGLLGADYSTKFSVWLALGSLSPRHIFWEVKRYENERTSNNSTYWVLFEL
IWRDYFRYVAMKYGNQIFYEGGIQNKNIRWKQDKQFFRAWKEGKTGVPFVDANMRELAATGFMSNRGRQNVAS
FLAKDLHLDWRLGAEWFESMLLDHDVCSNYGNWLYSAGIGNDPREDRKFNVVKQGFDYDSEGDYVRTWVPELK
NVHGGSVHIVWTLSRGILEKAGVTLGETYPNPVHVAPEWSRHYSKASGSGGARGGSSHRPQKGIDFYFKGGNQ
RR
>Crassostrea_virg__XP_022288154.1
MRRAFRTLLYTHSCSNHATICRTLTETSCLIQRFPKINRDRSLHHSCLSPQQLRLSDWINYCSNSQKRKMSDK
KSQKRKMGEIEESIKESPKKQKTEVTDGQGKGQTSLVLQIKEARENLCSSVAEFKFNKRRVRVLSKVQDFPDG
KNGVLYWMSRDQRVQDNWAMLYAQRLALKMEVPLHVCFCLVPKFLDATIRHYRFILRGLEEVEKECKELGISF
HLMIGHAKDVLPTFVKDNQIGGVITDFSPLRVPAEWVSGVTKTLPSDIPLCQVDAHNLVPCWEASPKLEYGAR
TIRNKIHNQLNKYLTEFPPVSKHPHKPKIPSEPVDWSKAEDSLEVDRKVKEVDWAVPGSTAGLNMLESFCRER
LKFFGADRNNPNKNALSNLSPWVKFGQISVQRCILTVRQYRSKYKESVESYIEEAVIRRELSDNFCFYNEHYD
SIEGAYDWAKQTLKVHADDKRDHIYSKEQLEFAKTHDDLWNAAQKQMVLEGKMHGFLRMYWAKKILEWTPSPK
IALETAIYLNDKYSLDGRDPNGYVGCMWSICGIHDQGWKERDVFGKIRYMNYAGCKRKFDVAAFERKYKSKLL
>Crassostrea_virg__XP_022291740.1
MNHNQQEVVVHWFRHGLRLHDNPALIEGLAECDKFYPVFIFDGEVAGTKTAGYNRYRFLLECLRDLDDNLQAA
GTRLYCFQGQPTDIFERLIEEWGVTKVTFEADPEPIWQERDRLVKDLLDKRNVQCVERVSHTLWDPHEIIQNN
GGNPPLTFSLFNLVTSTIGVPPRPVENPDFTDISLPVSQNHDKQFGIPSLEDLNIRPECEEQNNRFMEWKGGE
TKALELLEIRMKHEEQAYEKGYVMPNQYFPDLLSPPLSLSAHLRFGCLSVRNFYWRIHDKFKEVKPSVQALVS

LSAQLLWREYFYTMAVNNMNYDKMENNPICLNIPWYDNAEHEEKWTKGETGYPWIDAIMKQLRYEGWIHHVAR
HAVSCFLTRGDLWLSWEVGLKVFYKYLLDADWSVCAGNWMWVSSSAFEKVLQCPNCFCPVRYGKRMDPSGEYI
KRYLPVLKDMPLRYLFEPWKAPLPVQQKAKCIVGVDYPRPMVDHQVASKDCMKNMKAVKDALMGKEIPHCAPS
EDMEARRFSWLPDHTPSGGKCTANFLCDGLMNMD
>Crassostrea_virg__XP_022292416.1
MSTGSHAGGVLPPEQIGYIPDKLKKAEKRIEAFPYDTEAWSVLIRDAQMKPIEVARQVYERLVTQFPNAGKYW
RIYIEQELKAKNFERVEKLFQRCLVKILSIDLWKLYLNYIKDTKGKLPSYREKMAQAYDFALDKMGMDIMSYQ
IWVDYINFLKSVEAVGSYAENQQITAVRKVFQRGVVNPMINIEGLWKDYCQYEQNINPLIAKKMTEDRGREYI
NARRVAKEYEAVTRGLNKNLPSVPPQNNPDEAQQVDLWKKYIAWEKSNPLRTEDHALITKRVMFAYEQCLLCL
GHHPDIWYEAATYLEQSSKILTEKGDQNAGKMFADEAGSIYERAVTTLMKNNMLVYFAYADFEESRMKYEKVH
GIYKKLLVVQDIDPTLAFIQYMKFARRAEGIKSARQIFKMAREENRTRYQVFVAAALMEYYCSKEKTVALKIF
ELGLKKYGGIPEYLLCYMDFMSHLNEDNNTRVLYERVLSSGQVPPEKSIEIWSRFLAFESEVGDLASIQKVEK
RRAQAIEKVQEFEDKDTALLIDRYKYLDLYPCTTSELKAVGYFDLARQQVVTLPSASVTKVVLEDEESKNKPQ
YPKPDVEQMIAFKPRQIVPVGAHPVPGGEFPPPPAAANLISQLPPPDCFHGPFVIMDKFIEHFNQMVLPEAPV
GTENGIDGSFKLDPGTQLSIDFALGRKRKVTEGEDSDEEGSEKHVVHWFRKGLRLHDNPSLREALKGAATYRC
VYILDPWFAGSSQVGINKWRFLLQCLEDLDTSLRKLNSRLFVLRGQPTDLFPKIFKEWNITTLSFEEDPEPFG
KERDSAIQMLAKEAGIEVIVRTSHTLYDLQKIISMNGGSPPLTFKRFQSVLSKMEPPCEPEETVTSSFLEKKK
TPITEDHDDKYGVPTLEELGFDTEGLGPAVFHGGEAEALTRLERHLERKAWVASFERPKMSQQSLFPSQNVLS
PYLRFGCLSARLFYWKLRELYRKVKKRKIPPLSLHGQLLWREFFYTVATNNPNFDRMKDNPLCVQIPWDKNPE
ALAKWAEGKTGFPWIDAIMIQLRQTGWIHNLARHSVACFLTRGDLWISWEEGMKVFEELLLDADWSVNAGMWM
WLSCSSFFQQFFHCYCPVGFGKRADPTGDFIRTYLPVLKGYPAKYIYEPWTAPESVQRAAKCIIGKDYPVPMV
NHAEVSKLNTGRMKQVYQQLALYASIAAVPKQIHSEEPCSNSKAMHSGNHPSRVAMIENTDHGNHSQMSAEEG
DNQHQFLAA
>Crassostrea_virg__XP_022332471.1
MGTKGKTVICLLRNDLRIHDNEILQWANRNADFVLPLYVFDPRHFGGTYHFGFPKTGPHRTKLLIESVQDLRK
NLKLRGSGLAVRRGKPHEELKKVIDKLGQSSVHSVVFHEEVTQEELDVEKSIRKCCGVDIKTFWGHTLYHRDD
LPFSPAQTPDVYTQFRKRVESQAPVRKCLDMPKAFKPLPEGVEEGEVPTNRDLGVEDPVHDPRSVVDFVGGES
VAIDRLHHYLWETDAIATYKETRNGLLGADYSTKFSVWLALGSLSPRHIFWEVKRYENERTSNNSTYWVLFEL
IWRDYFRFVAMKYGNQIFFEGGIQNKSIRWKQDKQLFQAWKEGRTGVPFVDANMREMAATGFMSNRGRQNVAS
FLTKDLRLDWRLGAEWFESMLLDHDVCSNYGNWLYSAGIGNDPREDRKFNVVKQGFDYDSEGDYVRTWIPELK
NVHGGSVHIVWTLSRGALEKAGVTLGETYPNPVHVAPEWSRHYSKASGGGGGGARGSSHKQQRGIDFYFKGGN
KRNMAEESLSERQEFELQALQAIYTGDVVDLRVQDAWKVRRPPEIQITIKPQESFGNQGEHHVQVDLLVKCPE
RYPEELPEFSFIKTKGLSNTQLSNLKSEVDSIARENKGEEMLLQIAQHVQQFLHANNKPPAKSFYEEMMLNKR
KQEEQKEEEQRKELERLKKTEEKELQTLEIEIQRRKEALKEENKKRKEETKQVDERPQSVPSSPSSDSVPNVS
PVGTPGMVRRPRVSVSPSPLIVHNNTETRENKTRRQRRTSTPCRDHLEEHTCQNHTGGIVVLAFSTKGERTIH
RGACLGHGSSGNTVYVGIDTTSGELVTISEWVLKWRHYGRKKILRKEDGDEDKEGAACLKQVNSIEQELLSLI
RLHHPNLVHYLAIKYQQEPGKITLCLLMEYCSGTSLSVSIRRKRPIHVTHIKYYTEEILNALAYLHNKDVVHK
NLRASSIFLDATGKIKVADYSIERRLADLFYLLEQSRPGVHFDDNRPSQCRATKKGDSYGLGLVLLSLAIGEE
VDESNLEIPTHFPSYFQDFLNKCLMKEDKSRWSVHQLLEHSFIKMALPLHLPITRHETQQEDESQDEDSSDEE
QPVITPYEASGQSRLTNEFEILKSLGKGGFGDVIKVRNKLDRRFYAIKRIPLNPKSKQFNKKITREVKLLSRL
NHENVVRYFNSWIELSDDPAHSESSSSVSTTPKSTPKTPEKLTNTLDLSDDVEKLAPQTVEESVEWSRSYSAI
INPGWDSEEEDEADVFGTSFMLTDMSEDVVFVHSVDNDELSIDQVDGENVKAGEESTLSPRLQYLYIQMEYCE
KSTLRNCIDAGLYQDMNRVWRLFREIIEGLVHIHEQGMIHRDLKPVNIFLDSNDQVKIGDFGLATTSIISKSN
ILDVKMLTLSAAGDNTESYSETTGDGSLTGKVGTALYVSPEMMTGASKIVYTQKVDLYSLGIIFFEMCYKPLP
TGMERVKILGNLRLPEINFPEDFDQYQLKGQTHIIRWLLNHDPTKRASTKDLLLSEHLPPPQMEEEELDEILR
STIADPLSKAYRHMLTALFSQNVNAADDLLFDSEIYKTKFSTNLPLVQEMVQEQMVRIFHHHGAVRVTTPLLM
PKCELYTKTDLYTRFMDHCGHLVSLPFDLRIPFVRYVARHNCTSVKRFCIDKVFREKKVYGQHPRELTECAFD
IITPNLGSLFPDAEVVLLVQEIISEFSVLQQRNYYIKINHMLVLQAILQHCGVTGDQMNDVLHILSGAKLDSH
YRNISKSLTSLGLSDQTVSTLLGILEMEGNYGKVASYLRCITKTRGEAASKAKQGLHELEVILSNLDTLGLKL
PVTVSLGFVYNPQQFSGMIFQVMVDSKKKHHSPDILAAGGCYDQLIEKFCSPLSHPSVESSIHGVGVSIAFEK
IVQAMLDYKELTSTSSYDILVCTTIGHKPMLKERMAVLRDLWAAGLRAEIYLESMQTQEEVQEFCRSSGVSFL
VILKDDDPVAKVRSIEKDRITEKKFPSNALVVDFLQQKLQASSKQEQSDPPQVPPSKTLGMVTSSSDTGLSNH
GNNILINFNFVIENGKLASKSRYKYESQILAKVSSTLSWLPKRSIDVIAVEFSAPVLKCIVAFLEVDGTDQEF
EDSIGTVIEKQQRHYKKYIHKICDQIYHLKFEKRCNYIVLYGLKDDSIKLLT
>Crassostrea_virg__XP_022335390.1
MKTCAIHWFRKGLRLHDNPALLAACKAADDVKPVFILDPWFPKNANVGINRWRFLLQTLQNLDDNLKKVNSRL
YVVKGKPNEVFPKLFKHWGVSHLTFEEDTEPYAVSRDSEIQKIAKEHNVEVTLXVSHTLFDTQRTISKNGGKA
PLTYQRLQTVLSSMGSPPKPVESPTKCQTKTEPDHDKKYGVPSLEELGKSPEDCGPXCIPGGEIEALRRMESM
IQKKSWICSFEKPKTEPNSLEPSTTVLSPYLKFGCLSXRLFYYKLQEVYSKAKHTSPPVSLHGQLLWREFFYT
VAADTANFDKMEGNPVCKQIXWDTNEEYLKAWKEGRTGYPFIDAIMTQLRQEGWIHHLARHSVACFLTRGDLW

ISWEEGMKVFEEYLLDADWSLNAGNWMWLSASAFFHQYFRVYSPIEFGKKTDKDGNYIRKYVPQLSKFPKAFI
YEPWKAPLDIQKKAGCIIGKDYPKPIVNHDQMRKKNIERMANAYNSNKTDGGGEKRKSEASSVQKKKKRKVE
>Crassostrea_virg__XP_022341701.1
MEYEHGKEILHNFQTGGIDAEECFCMIVSLDGYDATRHTFLRNIDFLRLTNPEKYKELVSIYATYFEKPPSET
FLDPVGCSVENGYLTSDFGYELEVELASALSLEDQKGNQPDKPSYVNVVNHTVHNKPNNGSIPKRNANELQYK
DSKETNHKQLLNRPTSSQQHKKNKKKGGVAGQHMQPVIVWFRRDLRLYDNPALSEAASRNVPIILVFIWSESE
EDPQDILAAGGATKLWLHYALNELNRSISDKYTNKIIYRKTDSCQKEIMSLFEETGAKTLYINDVYEPFLKQR
DDKFVXELQRKGIECKRFHSYLLHDPNSVSVECVGMRGVGSVTHFMECCRLSTTQPIGRPLDYPGTLPKPNQF
PPSLSLHELELAKMPKRKDGSMINWAAPIVEKWNFGEDGAWEALELFLSEGVRKYEKESCRADHLNTCRISPY
LHFGQISPRAVLEEARHMKSPKFLRKLAWRDLSYWLLSLWPDLPSQPTRVHYRDQAWSSNAAHLKAWQRGRTG
FPXVDAAMRQLWLEGWINNYLRHVVASFLISYLRLHWIEGYRWFQDTLVDADVAINAMMWQNGGMSGLDQWNF
VMHPVDAALTCDPDGAYVRQWCPEISALPNDFIHQPWKCPPSILRRCGIKLGETYPHRALTDLEGARDQSLAD
VVNVRKRHPEFVDRRTGNDLVPLPDGSCVPVITRKEFKYKLHHPEARDNPHTAVLRGYRSRNRDEAIAFANER
DFMASAMNESIKLSERKLKSTQYQAL
>Crateromorpha_mey_CAZ66368.1_cry
MTYNTLHIFTDDALRINDNNSLLSNLENTKNLYTVFYYGHIKKINHIPPHRVIFLLESLKNLKERLDEYGIPL
YFIDEPMFQSLRMLISKWNINRVTTEPVVSIVGKRDQLSLRNFLSAFGVMLRTYNSSTLYDTVKVPVNITRSE
FFRIITQMEPELSLPEELTEFLSKFSSFPDPFINKKIPSLSEFGITNETMSSIDTKFRGGETAAILQLEKLIA
NRCEPNNLPKVAQLNQYDAISPAIKFGCISVRTIYNRVSKLEPKYNEVKNQIYDGLRNRDYCILVGGNCPNID
NQGSIYTYILPWDVKQDASTRFQTGRTGYPFIDAAIAQLKREGFIHNSVKNILVRTLTCDLMWIGWHEGVRMF
YKWSLDYNAAICALSWMHGSKSTWLLEEISISQINPIEEAKEIDKDGDYIRKYLPELKDYPSEYIHTPWLAPL
DQQIESECVIGQDYPYPNYCDVEERVQQCRKRLQIFYNIMPIAKKRRTLSLLKKRTRININNNDSVINIRAQL
ECKATKLSQ
>Crocodylus_porosus_XP_019407380.1_PREDICTED:
MAASRSFASSVPALAGPCRAVHWFRRGLRLHDNPALQAALRDAASVRCIYILDPWFAASSAVGINRWRFLLQS
LEDLDNSLRKLNSRLFVVRGQPTDVFPRLFKEWGVTRLTFEYDSEPFGKERDAAIIKLAKEAGVEVVVENSHT
LYDLDRIIELNGNKPPLTYKRFQAIISRMDLPKKPVSSITSQQMEKCKAEIQENHDDMYGVPSLEELGFPTDG
LAAAVWQGGEMEALARLDKHLERKAWVANYERPRMNANSLLASPTGLSPYLRFGCLSCRLFYYRLWELYKKVK
RNSTPPLSLYGQLLWREFFYTAATNNPKFDRMEGNPICIQIPWDRNPEALAKWAEGKTGFPWIDAIMTQLRQE
GWIHHLARHAVACFLTRGDLWISWECGVRVFDELLLDADFSVNAGSWMWLSCSAFFQQFFHCYCPVGFGRRTD
PSGDYVRRYLPKLKGFPSRYIYEPWNAPESVQKAAKCIIGVDYPKPMVNHAETSRLNIERMKQIYQQLSRYRG
LCLLASVPSCAEDLSGPVTDPASVQGCSTSTALKPSQSGQASPKRKHEGLEETCTEDLYKRVKVTGLHGPEIP
SKSL
>Crocodylus_por_XP_019388427.1_photolyase
MFPKKQKQAAGADAGPSGSCKRGRQDAQAAPGAESLAEVVEQARRRAAPSVREFKFNKKRVRLLSQESCLQDG
ALGILYWMSRDQRVQDNWAFLYAQRLALKQQLPLHVCFCLVPKFLEATIRHFGFLLKGLREVAEECGELGIPF
HLLQGYAKDVLPGFVTRHGIGGVVTDFSPLRVPLQWVEDVKERLPQDVPLVQVDAHNIVPCWVASSKQEYGAR
TIRPKIHAQLPEFLTEFPPVVQHPFPAAAPAQPIDWDACYASLEVDRTVSEVKWAAPGTAAGLAVLQAFVTRR
LPGYSEYRNNPNKAALSNLSPWFHFGQVSVQRAVLEVRKSRDRHRESVDSFVEEAVIRRELADNFCFYNKSYD
QLEGAHDWARDTLRLHARDKREYLYDLQQLEEGKTHDQLWNAAQIQMVREGKMHGFLRMYWAKKILEWTRSPE
DALRFAIYLNDRFELDGRDPNGYVGCMWSICGIHDQGWAERNVFGKIRYMNYAGCKRKFNVGQFEHKYRP
>Crocodylus_por_XP_019400341.1_cry-1_X2
MGVNAVHWFRKGLRLHDNPALCECIQGADTVRCVYILDPWFAGSSNVGINRWRFLLQCLEDLDANLRKLNSRL
FVIRGQPADVFPRLFKEWNITKLSIEYDSEPFGKERDAAIKKLASEAGVEVIVRISHTLYDLDKIIELNGGQP
PLTYKRFQTLISRMEPLEMPVETITAEVMEKCTTPVSDDHDEKYGVPSLEELGFDTDGLPSAVWPGGETEALT
RLERHLERKAWVANFERPRMNANSLLASPTGLSPYLRFGCLSCRLFYFKLTDLYKKVKKNSSPPLSLYGQLLW
REFFYTAATSNPRFDKMEGNPICVQIPWDKNPEALAKWAEGRTGFPWIDAIMTQLRQEGWIHHLARHAVACFL
TRGDLWISWEEGMKVFEELLLDADWSVNAGSWMWLSCSSFFQQFFHCYCPVGFGRRTDPNGDYIRRYLPVLRG
FPAKYIYDPWNAPEAVQKAAKCMIGINYPKPMVNHAEASRLNIERMKQIYQQLSRYRGLGLLATVPSNPNGNG
NGGLMGYSPGENVSGCSSAGSYFQASSILHFPHGDNQQSHLLQQGRTSLSSSISAGKRPNPEEETQSIGPKVQ
RQSTN
>Crocodylus_por_XP_019402505.1_cry-2-like
MKHSCIHWFRKGLRLHDNPALLAAMKDCSDLYPIFILDPWFPKNMQVSVNRWRFLIESLKDLDESLKKLNSRL
FVVRGHPAEVFPGLFKAWKVTRLTFEVDTEPYSKLRDAEVVRLAAAHGVQVIQKVSHTLYDTDRIIAENNGKV
PLTYRQLQAVLAGLGSPKQPMLAPTLETLKDCCTPARDNHDPKYEIPTLEELGQDPKEAGPRLYPGGESEALS
RLDFHMKRMTWVCNFKKPDTEPNTLSPSTTVLSPYIKFGCLSVRTFWWKLAEIYRGKKHSSPPVSLHGQLLWR
EFFYTAGAGIPNFNRMEGNPICVQVDWDDNLEYVKAWKEGRTGYPFIDAIMTQLRTEGWIHHLARHAVACFLT
RGDLWVSWEEGQKVFEELLLDADWSLNAGNWQWLSASAFFHQFFRVYSPVAFGKKTDKSGAYIKKYLPILRKF
PAEYIYEPWKAPRSMQEQAGCIIGRDYPRPIVEHEAVSKRNIMRMKAAYAQRSHSKSAQVEKEGTKKGGKRKL
PAGPSVVELLTKKPKAKSS

>Crotophaga_sulc__NWS70718.1
KVDYDVCSNYGNWLYSAGIGNDPRENRKFNMIKQGLDYDGNGDYVRLWLPELQGLKGADIHSPWALTSAALSQ
AGVTLGETYPQPVVTAPEWSRHMNQSLQGSSPHPRGRRAPAHTPAPHRNRGIDFYFSRKKDV
>Crotophaga_sulc__NWS73616.1
MGVNAVHWFRKGLRLHDNPALRECIQGADTVRCVYILDPWFAGSSNVGINRWRFLLQCLEDLDANLRKLNSRL
FVIRGQPADVFPRLFKEWNIAKLSIEYDSEPFGKERDAAIKKLASEAGVEVIVRISHTLYDLDKIIELNGGQP
PLTYKRFQTLISRMEPLEMPVETITPEVMEKCATPVSDDHDEKYGVPSLEELGFDTDGLPSAVWPGGETEALT
RLERHLERKAWVANFERPRMNANSLLASPTGLSPYLRFGCLSCRLFYFKLTDLYKKVKKNSSPPLSLYGQLLW
REFFYTAATNNPRFDKMEGNPICVQIPWDKNPEALAKWAEGRTGFPWIDAIMTQLRQEGWIHHLARHAVACFL
TRGDLWISWEEGMKVFEELLLDADWSVNAGSWMWLSCSSFFQQFFHCYCPVGFGRRTDPNGDYIRRYLPVLRG
FPAKYIYDPWNAPESVQKAAKCIIGVNYPKPMVNHAEASRLNIERMKQIYQQLSRYRGLGLLATVPSNPNGNG
NGGLMGYSPGESISGCGSTAGQSNSFTQLGTGDGHTVVQPCALGDSHTGGSGIQQQGYCQASSILHYAHGDNQ
QSHVLQAGRTALGTGISAGKRPNPEEETQSVGPKVQRQSTN
>Crotophaga_sulc__NWS77795.1
RFLLQSLEDLDNSLRKLNSRLFVVRGQPTDVFPRLFKEWGVTRLTFEYDSEPFGKERDAAIVKLAKEAGVEVV
IENSHTLYDLDRIIELNGQKPPLTYKRFQAIISRMELPKKPVSTVMSQQMESCKVDIQENHDDMYGVPSLEEL
GFPTDGLAPAVWQGGETEALARLDKHLERKAWVANYERPRMNANSLLASPTGLSPYLRFGCLSCRLFYYRLWE
LYKKVKRNSTPPLSLYGQLLWREFFYTAATNNPKFDRMEGNPICIQIPWDRNPEALAKWAEGKTGFPWIDAIM
TQLRQEGWIHHLARHAVACFLTRGDLWISWESGVRVFDELLLDADFSVNAGSWMWLSCSAFFQQFFHCYCPVG
FGRRTDPSGDYVKRYLPKLKGFPSRYIYEPWNAPESVQKAAKCIIGVDYPKPMVNHAETSRLNIERMKQIYQQ
LSRYRGLCLLASVPSCMEDLSGPVTDSASGQGCSTSTPVRLSQTEQASPKRKHEGAEELCTEELYKRAKVTGL
PAPEIPGKSL
>Crotophaga_sulc__NWS78428.1
DNWAFLYAQRLALKQELPLHVCFCLVPKFLDATLRHYSFMLKGLQEVAEECAELNIPFHLLLGCAKDVLPAFV
VEHGVGGLVTDFCPLRLPRQWVEDVKERLPEDVPFAQVDAHNIVPCWVASPKQEYSARTIRGKIHAQLPEFLT
EFPPVIRHPHSPSCPAEPIAWEACYSSLQVDSTVKEVEWATPGTAAGLAVLQSFIAERLKFFGSHRNDPNRAA
LSNLSPWFHFGQVSTQRAILEVQKHRSKYKESVEAFVEEAVVRRELAENFCYYNENYDNVQGAYDWAQTTLKL
HAKDKRPFLYGLQELEQGTTHDPLWNAAQLQMVWEGKMHGFLRMYWAKKILEWTRSPEEALRFAIYLNDRYEL
DGRDPNGYVGCLWSICGIHDQGWAERAVFGKIRYMNYAGCKRKFDVGQFERRYAPRT
>Crotophaga_sulc__NWS78782.1
MPHRTIHLFRKGLRLHDNPTLVAALESSEVVYPVYILDRTFLTSAMHIGNLRWHFLLESLEDLQKNLCQLGSC
LLVIQGEYESVLRDHVQQWNITQVTLDAEMEPFYKDMEANVRRLGEELGFEVLSRVGHSLYDTKRILDLNGGT
PPLTYKRFLHILSLLGDPEAPVKTPTAEDFQRCRPPAPGLAECYRVPLPVDLEVPLENLSPWRGGETEGLRRL
EQHLTDQGWVASFTKPRTIPNSLLPSTTGLSPFFSMGCLSVRTFFYRLSNIYAQAKHHSLPPVSLQGQLLWRE
FFYTVASATPNFTKMVGNPICLQISWYKDAEKLHKWKMAQTGFPWIDAIMTQLRQEGWIHHLARHAVACFLTR
GDLWISWEEGMKVFEELLLDADYSINAGNWMWLSASAFFHQYTRIFCPVLFGKRTDPEGQYIRKYLPVLKNFP
SKYIYEPWTASEEEQKQAGCIIGEDYPFPMVNHKEASDHNLQLMRQVREEQHRTAQLTRDDTDDPMEMNVKRE
HSEEHPSKGKVAKMTE
>Crypthecodinium_coh_QDO16439.1_6-4_photol
MSAGVWFRKCLRLHDNAALTKAAESSDSVIPFFILDPTFDRSKIGVNRFHFLLESLRDLDEQLRERYNSRLLV
FRGKPAEVLSELFEGSGEFRLGSLFYERDIEPYARARDAAVEDLAKKFKVPTMTFSGHTILDVDLVVQAPGFK
PPIAMSSIQNLVKKHGPIPKATGAPKYLPALKVKGGGHKYDVPDLAELYEEEPTERHLMPGGEREALARLKRS
CSDKDYIATFEKPKTESTGRQGAPWEPSTTGLSPYIKFGCISVRKVWHAIDDCLKGRKHSQPPMSLHGQLLFR
EMFYVLGASVPNLDKDKGNRMCKEIPWGHNQKVLSAWEEGRTGYPFIDALMRQLVQTGWMHHLGRHAVSCFLT
RGDLWQNWTAGRDIFDKYLLDSDWAINNGNWLWLAGVAPWSAPYFRIYDPCPGPKTSLNAVHSADFIRHFVPE
LKGMPDRFLLTPWKAPDDVQKAAQCILGKDYPKPIVDHKSAREGNLSRFKAALESNRTGSSSKLPEKFGQRKT
DPGPMRVAYSDKSATSVLESGQASGSISTSGGGRRVSAPEEDDDGRAKLKTGQEAGALKRPAAVAAGGARKRP
ATAATAAAATVTRGKSQ
>Crypthecodinium_coh_QDO16452.1_CPD_photol
MATTSAAKKLFVDVALVDSRRCRKINSIPFPDRPGPVVYWMSRDQRSEDNWALLYAQGLAKQQKVPLHVVFCL
VPKFLDATIRQFDFMLRGLREVEQDLISKDIPFHLKMGLASNEVPAFCKEQEASAVVCDMAPLRVPMQWTNDV
GNALADLNIPLVQVDAHNVVPVWAASDKQEYAARTIRRKIMDSLPTYLKDFPPLEKHPDPPAKDKWPKPVDWD
LAEKTLEVDRTVLPVKQFLPGMFEGKKVLEAFCGSKLSKFADKRNDPNSDISSNLSPYLHFGQLAPQRAALAA
RKAAEKDKKAYAKGCESFIEELVVRRELADNFCFYNPKYDKIEGASAWAQETLKAHQSDKREHIYTAQQLEEG
KSHDDLWNAAQLQLVREGKMHGFLRMYWAKKILEWMPTPAEALAEAIRLNDRYELDGRDPNGYVGCMWSICGI
HDMGWTERPIFGKIRFMNYAGCKRKFNIPNFVKRYPGATKSVLTFPEAKKRKTD
>Crypthecodinium_coh_QDO16498.1_cry_DASH,_
MAGRLRCLWFRVDLRIDDNPALQAACRGSGNLLPVFVFDPKNFNVPTLAGARKSSARRAQFLLESVECLRKSL
EKRGSGLAVGLGSAEDVFSKLCGDAQDIFLQESVCSEERSEEARVEKRLKGSKLQRVWGGTLYLPEECGQQPN
NTPTLFTSFKNKAEGRGTIRQPIEPPKKLPELPQLPEDVANSLSYLPSLEDLGYSRDEAEAAKRSDPRGVLDF

RGGEEAALARMQKWIWEDDRLQEYKEIRNGMLGEGFSSKLSPWLALGCMSPRRIYKEVQHYESERVKNKSTYW
LIFELEWRDFFVYMAKTQGDKIFYKGGVVGDKAPWKNPKGALQRWKDGKTGDPLVDANMRELAATGFMSNRGR
QNVASYLIFNLEVDWRHGAAHFEEHLLDYDPCSNWGNWVAAAGLTGQRINKFNTKKQLNDYDPERRYVRHWLE
ESKSSPTEMEVDEEGASSAGRRWAPKSQHDEKGKGKGKD
>Crypturellus_undu__NWJ00429.1
DNWAFLYAQRLALKQELPLHVCFCLVPKFLDATIRHYSFMLRGLQEVAKECTELNIPFHVLLGYAKDVLPAFV
VRHGVGGLVTDFYPLRVPQQWVEDVRERLPEDVPFAQVDAHNIVPCWVTSSKQEYSAWTIRSKIHRQLPEFLT
EFPPVIQHPYPPSTPAEPIAWEACDSSLQVDRTVKEVAWATPGTAAGLAVLQSFIAERLESYGSYRNNPNKAA
LSNLSPWFHFGQISTQRAVLEVRKHHRKYQESVDIFVEEAVVRRELSDNFCYYNKNYDSVQGAYDWAQTTLKL
HAKDKRPFLYKLQELEKGNTHDPLWNAAQLQMVQEGKMHGFLRMYWAKKILEWTRSPEEALQFAIYLNDRYEL
DGRDPNGYSRDADSVCVCPAGCLWSICGIHDHGWTERAVFGKIRYMNYAGCKRKFDVGQFERRYGPRK
>Crypturellus_undu__NWJ00893.1
MPHRTIHLFRKGLRLHDNPTLLAALESSEAVYPVYILDRPFMASVMNIGALRWHFLLQSLENLQKNLLQLDSC
LLVIQGEYESVLRDHVKKWNITQVTLDAEVEPFYKEMEAKIRSLGEELGFKVLSLINHSLYDTQRILDLNDGT
PPLTYKRFLHILSLLGDPEVPVRSPAAEDFQRCRPSDLCLTECYRVPLPVDLKIPTESLSPWKGGETEGLQRL
EQHLANQGWVASFTKPRTIPNSLLPSTTGLSPYFSLGCLSVRTFFYRLSNIYAQSKHHSLPPVSLQGQLLWRE
FFYTVASATPNFTKMVGNPICLQINWYEDAERLHRWKTAQTGFPWIDAIMTQLRQEGWIHHLARHAAACFPTR
GDLWINWEEGMKVFEEMLLDADYSINAGNWMWLSASAFFHQYTRIFCPVRFGKRTDPQGHYIRKYLPILKNFP
SKYIYEPWTASEEEQKEAGCIIGQDYPFPMVNHKEVSDHNLLLMKQVREEQYRTAQLTRDDADDPMEMKMKRD
HSEESIKKGKVARTTE
>Crypturellus_undu__NWJ02641.1
RFLLQCLEDLDANLRKLNSRLFVIRGQPADVFPRLFKEWSIAKLSIEYDSEPFGKERDAAIKKLATEAGVEVI
VRISHTLYDLDKIIELNGGQPPLTYKRFQTLISRMEPLEMPVETITPEVMEKCTTPVSDDHDEKYGVPSLEEL
GFDTDGLPSAVWPGGETEALTRLERHLERKAWVANFERPRMNANSLLASPTGLSPYLRFGCLSCRLFYFKLTD
LYKKVKKNSSPPLSLYGQLLWREFFYTAATNNPRFDKMEGNPICVQIPWDKNPEALAKWAEGRTGFPWIDAIM
TQLRQEGWIHHLARHAVACFLTRGDLWISWEEGMKVFEELLLDADWSVNAGSWMWLSCSSFFQQFFHCYCPVG
FGRRTDPNGDYIRRYLPVLRGFPAKFIYDPWNAPESVQKAAKCVIGVNYPKPMVNHAEASRLNIERMKQIYQQ
LSRYRGLGLLATVPSNPNGNGNGGLMGYSPGENISACSSTGGAQLGTGDAHAVDVQTCALGDSHAGASGIQQQ
GYCQASSILHYAHGDNQQSHLLQTGRTSLGTGISAGKRPNPEEETQSIGPKIQRQSTN
>Crypturellus_undu__NWJ08543.1
LLKVDYDVCSNYGNWLYSAGVGNDPRGDRRFNTIKQGLDYDGSGDYVRLWVPELQELKGGDIHAPWALSGASL
AQAGITLGETYPQPVVTAPEWSRHIGPRPHRRGPHPKGREGPAHPPRQHKDRGIDFYFSRKKRL
>Crypturellus_undu__NWJ09111.1
CIYILDPWFAASSAVGINRWRFLLQSLEDLDNSLRKLSSRLFVVRGQPTDVFPRLFKEWGVTRLTFEYDSEPF
GKERDAAIVKLAKEAGVEVVIENSHTLYDLDRIIELNGHKPPLTYKRFQAIISRMELPKKPVSSIVSQQMETC
KVDIQENHDDVYGVPSLEELGFPTDGLAPAVWQGGETEALARLDKHLERKAWVANYERPRMNANSLLASPTGL
SPYLRFGCLSCRLFYYRLWELYKKVKRNSTPPLSLYGQLLWREFFYTAATNNPKFDRMEGNPICIQIPWDRNP
EALAKWAEGKTGFPWIDAIMTQLRQEGWIHHLARHAVACFLTRGDLWISWESGVRVFDELLLDADFSVNAGSW
MWLSCSAFFQQFFHCYCPVGFGRRTDPSGDYVKRYLPKLKGFPSRYIYEPWNAPESVQKAAKCIIGVDYPKPM
VNHAETSRLNIERMKQIYQQLSRYRGLCLLASVPCVEDLSGQVADPTLGQSSSTSTVMRVPQSDQASPKRKHE
GAEELCTEELYKRAKVGGLPAPEIPGKSL
>Ctenocephalides_fel_XP_026474410.1_LOW_QUALIT
MEVDSKEEVVPGKHAVHWFRRGLRLHDNPALLEALKGADTLRCVYILDPWAARGAGANALNKWRFLLESLTDL
DASLKSRLDCRLHVVRGQPADALPALLERWGVTLLSYETDPEPYGRARDRKLAARCRDNGVEIVEYESHTLYP
LDSIIEHNGGSAPVTFEQFLGVLTTMGPPDPPAGPVRRDRLFTTRTRVPTDPDYDQSYAVPTLSELGFEANSS
EPPVWRGGEAEALARLERHAEKRTLALNSSPNFDTLPPGFLLADGAGLSPYLRFGCLSARLCYHQLADLAPAT
KPLLQPPPAVMPLLWREFLYCCGADNPRFDRVQGNPLCLQTPWDRDPTALAKWANGQTGFPWIDAAMSQLRSE
GWIHHLARFALIDFLTKGALFISWEEGMKVFEDLLLDADWSTNAGSWLWLSCSSFFRKPAPSVCPVALGKLVD
PDAEYIRKYVPALRGFRSARLVHAPWLASREEQRRAACRLGAQYPLPLLVDHARTRKENARRTRLACETLPGY
RNRERRLSESFQDESDIDCNCEPVIPKQQIAQDEPIMDTNIHLPDIRQEIKSLPEFESTINSLFI
>Cuculus_canorus_XP_009568372.1_PREDICTED:
MTAGRKPGTDWFRRGLRLHDNPALQEALRDASSLRCIYILDPWFAASSAVGINRWRFLLQSLEDLDNSLRKLN
SRLFVVRGQPTDVFPRLFKEWGVTRLTFEYDSEPFGKERDAAIIKLAKEAGVEVVIENSHTLYDLDRIIELNG
HKPPLTYKRFQAIISRMELPKKPVSTVMSQQMETCKVDIQENHDDVYGVPSLEELGFPTDGLAPAVWQGGETE
ALARLDKHLERKAWVANYERPRMNANSLLASPTGLSPYLRFGCLSCRLFYYRLWELYKKVKRNSTPPLSLYGQ
LLWREFFYTAATNNPKFDRMEGNPICIQIPWDRNPEALAKWAEGKTGFPWIDAIMTQLRQEGWIHHLARHAVA
CFLTRGDLWISWESGVRVFDELLLDADFSVNAGSWMWLSCSAFFQQFFHCYCPVGFGRRTDPSGDYVKRYLPK
LKGFPSRYIYEPWNAPESVQKAAKCIIGVDYPKPMVNHAETSRLNIERMKQIYQQLSRYRGLCLLASVPSCVE
DLSGPVTDSASGQGCSTSTAMRLSQPDQASPKRKHEGAEELCTEELYKRAKVTGLPTPEIPGKSL
>Cuculus_can_XP_009555905.1_cry-1,_par

VNAVHWFRKGLRLHDNPALRECIQGADTVRCVYILDPWFAGSSNVGINRWRFLLQCLEDLDANLRKLNSRLFV
IRGQPADVFPRLFKEWNIAKLSIEYDSEPFGKERDAAIKKLASEAGVEVIVRISHTLYDLDKIIELNGGQPPL
TYKRFQTLISRMEPLEMPVETITPEVMEKCATPVSDDHDEKYGVPSLEELGFDTDGLPSAVWPGGETEALTRL
ERHLERKAWVANFERPRMNANSLLASPTGLSPYLRFGCLSCRLFYFKLTDLYKKVKKNSSPPLSLYGQLLWRE
FFYTAATNNPRFDKMEGNPICVQIPWDKNPEALAKWAEGRTGFPWIDAIMTQLRQEGWIHHLARHAVACFLTR
GDLWISWEEGMKVFEELLLDADWSVNAGSWMWLSCSSFFQQFFHCYCPVGFGRRTDPNGDYIRRYLPVLRGFP
AKYIYDPWNAPESIQKAAKCIIGVNYPKPMVNHAEASRLNIERMKQIYQQLSRYRGLGLLATVPSNPNGNGNG
GLMGYSPGESVSGCGSTGGAQLGTGDGHTVVQPCALGDSHTGGSGIQQQGYCQASSILHYAHGDNQQSHVLQA
GRTALGTGISAGKRPNPEETQSVGPKVQRQSTN
>Cuculus_can_XP_009556633.1_cry_DASH-l
MTAICLLRCDLRAHDNQVLHWAQGIAGFVVPLYCFDPRHYLGTHCHSFPKTGPHRLRFLLESVRDLRETLKKK
GSTLVVRKGKPEDVVHDLITQLGSVSAVAFHEEATQEELDVEKGLCQVCSQHGVKIQTFWGSTLYHRDDLPFR
PIARLPDVYTHFRKAVESGAKVRPTLQMADQLKPLPPGVEEGCIPTMEDLGQKDPVTDPRTAFPCSGGETQAL
MRLQYYFWDTNLVASYKETRNGLVGMDYSTKFAPWLALGCISPRYIYEQIQKYEKERTANQSTYWVLFELLWR
DYFRFVALKYGKRIFSLRGLQGKEISWKKDLQLFDCWKEGKTGVPFVDANMRELAATGFMSNRGRQNVASFLT
KDLGLDWRMGAEWFEYLLVDYDVCSNYGNWLYSAGIGNDPRENRKFNMIKQGLDYDGNGDYVRLWVPELQGIK
GADIHTPWALSKAALLQAGVTLGETYPEPLVTAPEWSRHVNQSLQGRSPHPRGRRAPAHTPVPHKNRGIDFYF
SHKKDA
>Cuculus_can_XP_009557709.1_cry-1-like
MPHRTIHLFRKGLRLHDNPTLLAALESSEVVYPVYILDRTFLMSAMHIGNLRWHFLLQTLEDLQKNLCQLGSY
LLVIQGEYESVLRDHVQQWNITQVTLDAEMEPFYKEMEVNIRRLGKELGFEVLSRVGHSLYDTKRILDLNGGT
PPLTYKRFLHILSLLGDPEMPVQTLTAEDFQRCCPPAPGLAERYRVPLAVDLKIPSENLSPWRGGETEGLRRL
EQHLNDQGWVASFTKPRTIPNSLLPSTTGLSPYFSTGCLSVRTFFYKLSNIYAQAKHHSLPPSSLQGQLLWRE
FFYTVASATPNFTKMAGNPICLQISWYEDAEKLHKWKTAQTGFPWIDAIMTQLHQEGWIHHLARHAVACFLTR
GDLWISWEEGMKVFEELLLDADYSINAGNWMWLSASAFFHQYTRIFCPVRFGKRTDPEGQYIRKYLPVLKNFP
SKYIYEPWTASEEEQKQAGCIIGEDYPFPMVNHKEASDHNLQLMRQVREEQQRTAQLTRDDTDDPMEMKVKRE
HSEERTSKGKVAKMTEQTEIPAGISCWEPENGKEGEPEAS
>Cuculus_can_XP_009563639.1_photolyase
MGRVPAFPGLWERTGHRDGYQHSPGSGMGIGVGTSIPRALRVDRGLGTVPFSLVATTLLRASILAVLRMRRGQ
RKRKAEATEVPPVSRRRKEEEEALQERRRAAPSVREFKYNKKRVRLVSQGSELKEDARCILYWMSRDQRVQDN
WAFLYAQRLALKQQLPLHVCFCLVPKFLDATIRHYGFMLKGLQEVAKECAELNIPFHLLLGYAKDVLPTFVVE
HGVGGLVTDFCPLRLPRQWVEDVRQRLPEDVPFAQVDAHNIVPCWVASPKQEYSARTIRGKIHAQLPEFLTEF
PSVIRHPHPPSCPVEPIAWEACYSSLQVDRSVEEVEWATPGTAAGLAVLQSFITERLKFFGSHRNDPNKAALS
NLSPWFHFGQVSTQRAILEVQKHRSKYKESVDAFVEEAVVRRELAENFCYYNENYDSVQGACDWAQTTLKLHA
KDKRPFLYELEELEQGTTHDALWNAAQLQMVREGKMHGFLRMYWAKKILEWTHSPEKALQFAIYLNDRYELDG
RDPNGYVGCLWSICGIHDQGWAERAVFGKIRYMNYAGCKRKFDVGKFERRYAPCMLSQ
>Cucurbita_mos_bluelightPa_XP_022960062.1
MDPNPQILENPEPISSEDQSPPVPVHSPPFAVASLTLSLSTVIPSNFLVQPRFSGLFSRQPNKDNVPTQAASL
SHLPISCSSLCPPKISLKSTVSANPLQIPLSLGPRRPSEPSNGAGMRRATIVWFRNDLRIHDNECLNSANNDS
ISVLPVYCFDPRDYGKSSSGFDKTGPYRATFVIESVSDLRKNLQARGSNLVVRIGKPETVLAELAKEIGADGI
YAHYEVSHDEMETEERIESAMKEENVEVKYFWGSTLYHIDDLPFKMEDMPSSHGAFRERVQGLSVRKTIEALD
KMKGLPSRGDVEPGDIPSLSELGLNQPVAMSKEGWLAPNTSQVGGETEALHRLQKYAAECRSQPPKATNNGAQ
NSIYGATFSNKISPWLTMGCISPRSVFDELNKTVSRKHDGGNGTGTNWLMFELLWRDFFRFITKKYNSTKKQP
NPSPATACTGALA
>Cucurbita_mos_XP_022922402.1_photolyase
MASTPSNSVQSGRIRVLKDVTGSLDRPVGPVVYWMFRDQRVKDNWALMHAVDEANRAKVPVAVAFNLFDQFLG
AKSRQLGFMLRGLQQLQYDLEETLQIPFFLFQGEAEHTIPNFIRECEASLLVTDFSPLREVRKCKEEICKRVD
ESVKVHEVDAHNVVPIWIASEKLEYSAKTLRGKINKKLPEYLIDFPALLIPTRKWPSTDKFIDWDRLIDDNLR
KGADVPEIEWCKPGEKAAMEVLMGSKDGFLTKRLKSYAIDRNNPLKPRGLSGLSPYLHFGQISAQRCALEARN
VRKLNPQAVDMFLEELIVRRELADNYCYYQPHYDSLLGAWEWARKTLMDHASDKREHVYTREQLEKAQTADPL
WNASQLEMVFHGKMHGFMRMYWAKKILEWTRGPEEALEICIYLNDKYEIDGRDPNGYVGCMWSICGVHDQGWK
ERPIFGKIRYMNFAGCKRKFDVDGYIAYVKRVVGEVKKRKAGEVVVDRKAKELRC
>Cucurbita_mos_XP_022935871.1_cry_DASH,_
MNTLRISFSSFPLLKTLPNSSSLKPAQIAANSAHRRIFVMNSSSKLDSRSSSSSICQVPGLESEEMDRIAEQM
FRRYESPSSSSVKRGKGVAIVWFRNDLRVLDNEALYKAWISSEAVLPVYCVDPRLFGSTCYFGFPKTGALRAQ
FIVECLADLKRNLINRGLNLLIQHGKPEEILPSLAKALGAHTVYAQMETCSEELYVERMVSKGLKTVVLSPTS
EKSAKPSSARSPTLLLVWGTTMYHIDDLPFDTNSLPDVYTQFRKSVEAKCAIRDCIRLPALLGPPASIDNWGC
VPSLDKLELQPPSVVKGMRFIGGESAALSRIYEYFWKKDLLRIYKETRNGMLGPDYSTKFSPWLASGSISPRL
IHEEVKRYEKEREANQSTYWVLFELIWRDYFRFLSVKYGNSLFHIGGPRKVESKWSQDKNLFESWRDGRTGYP
LIDANMKELSTTGFMSNRGRQIVCSFLVRDMGIDWRMGAEWFETCLLDYDPCSNYGNWTYGAGVGNDPREDRY

FSIPKQAQTYDPEGEYVAYWLPQLRMLPKDKRHFPGKMLYMEQVVPLKFGNAARPQSQDYARRKNFGGRQAKD
FKR
>Cucurbita_mos_XP_022936701.1_cry-1_
MSGGGCSIVWFRRDLRVEDNPALTAAVRSGAVVAVFIWAPEEEGHYYPGRVSRWWLKQSLALLDSSLRSLGTF
LVTKRSTNTISALLDVVKATGASQLFFNHLYDPLSLVRDHRAKEVLSAQGIGVRSYNADLLYEPWDVKDANGN
PFTTFAGFWERCLSMPCDPEAPLLPPKRIISGDASRRCPCDTLVFEDESEKGSNALLARAWSPGWSNADKALT
TFINGPLIEYSKNRRKADSATTSFLSPHLHFGEVSVRKVFHLVRMKQVLWANEGNEAGEESVNLFLKSIGLRE
YSRYMSFNHPYSHERPLLGHLKFFPWVVDEGYFKAWRQGRTGYPLVDAGMRELWATGWLHDRIRVVVSSFFVK
VLQLPWRWGMKYFWDTLLDADLESDALGWQYISGTLPDSREFDRIDNPQLEGYKCDPYGEYVRRWLPELSRLP
TEWIHHPWNAPEYVLQAAGIELGSNYPLPIVGLDAAKARLEEALSEMWQQEAASRAAIENGTEEGLGDSSESI
PIAFPQEITMEEEDVEPARMNAHTIRCYEDQMVPSMTSSVRLGDEPSLNIQSTAEDGRAEVPINANLNQELTR
NVVNPRVRPNDPTQTRLPYTPGIGLRTAAEDSTAESYSSSDVRRERDGGVVPVWSPPSSSYTEQFVVEENGIG
TSSSFLQGHQQSHQIINWRRLSQTG
>Cucurbita_mos_XP_022958759.1_cry-1
MGCNKTIVWFRRDLRIEDNPALTAAARDGFVYPVYIWCPKEEGQFYPGRVSRWWLKQSLAHLKQSLKSLGSDL
VLMKTQSTIFSLLECINAIGATKVAFNCLYDPISLVRDHNIKEKLVELGISVQSYNADLLYEPWDVYDENGNA
FTTFKDYWGKCLLLQKEFISTLPPWKLQHAAGSVGSCCIEELGLENESEKSSNALLARAWSPGWSNADKALAE
FVENHLLEYAKNRQQLGGSSTSLLSPYLHFGEVSVWKVFQKVRMKQILWAREENAVGEQSTNLFLRAIGLREY
SRYICFNFPFTHERSLLSSLKFFPWHASQNNFKAWRQGRTGYPLVDAGMRELWATGWIHNRIRVIVSSFAVKV
LLLPWKWGMKYFWDTLLDADLESDILGWQYISGSLPDGHELERLDDPQIQGSKYDPDGEYIRHWLPELARMPT
EWIHHPWDAPQTVLKVSGVELGLNYPTPIVDLDLAANRLREAIIKMREIEAAAGANSNGTNEVVMDNADRIQS
LGTANVVAEPKTCATYSSNDQKVPMIQTSKVDNPLSRKRSKPMEEKGEFQYNIRNDVQSEAGTSKPDEDLCST
AESSSSKKPSTSRTSFSVPQFCSSSKGLPESSEGTTDR
>Cucurbita_mos_XP_022959592.1_(6-4)DNA_p
MRAAIQPFSLHCSTSNKFMASNSSSLLWFRKGIRIHDNPALEYAAKGANCLYPVFVIDPHYMKPNPSAFSPGS
SRAGLNRIQFLLESLVDLDMNLKKLGSRLLVLHGEPGEVLIRCLEEWNVKKLCFEYDTDPYYQALDSRVQNHA
SAAGVEVFSPVSHTLFNPADIIQKNGGRPPLSYQSFLKLAGEPSWARAPLSTTVSSLPPVGDTRRSQILDVPT
IRDLGYEDMGKVEWTPFRGGETEALKRLEESLSNKDWVAKFEKPKGDPSAFLKPATTVLSPYLKFGCLSSRYF
YQRVQDIYKNVKGHTTPPVSLVGQLLWREFFYTAAFGTPNFDRMKDNKICKQIPWKDDNELLAAWRDARTGFP
WIDAIMVQLRKWGWMHHLARHSVACFLTRGDLFVHWEKGRDVFERLLIDSDWAINNGNWMWLSCSSFFYQYNR
IYSPVTFGKKYDPNGDYIRHFLPVLKDMPKQYIYEPWTAPLSIQKKAKCIIGKDYPEPVVSHDIATKDCRRKM
AEAYELNKRLNGMISEDDLENLKRSSEEEQSQKVQTKRKRQTIIDWIQAP
>Cyanidiococcus__yan_1,3-beta-gluc_KAF6001014.1
MSLHFAFVLTTKATLPVLPYRQVGSAARARRRYREHKLVHLASVRHPLVMHGVSTLAEGSLERGGTFWSAQNL
GLEPAPMFIGADLGKNAEFGGKGGGVVLLWFRSDLRLDDNPALSAALEEGSSVLPVYCFDPRQFGKTSFGFEK
TGRYRARFLIESVADLRNALKKKGNNLLIRIGKPEEVLPDLCRKYEIKKVLFHQEVTFEELECEEAVTRRLEE
MKIEVRPFWTNTLYAVEDLPFPVEETPDVYTEYRLAVEQKSRVRDPLSAPEEVRAIPRRVDYGTVPSLQELGL
GDIPKSSEHGHMNATSSNIHNFRGGESEAQKRLAQYVQEAKLALTAGGDVERAAAHLGADFSCKISPWLALGC
VSPRRIFGEIRDQALNGSDGVQKSTTFFELVWRDFFRLITYKYSTSRLRKSEKAAGMPNRAPVAAAAS
>Cyanidiococcus_yan_KAF6000401.1_cry
MFRRGMQSWGLVWFRNDLRLRDHEAFVTACKEHDGRVIPIYVFDGRQVGPGAKTAVGGFSKCSTPRAQFLLDA
VQDLEDTLRQRYHGMQLIIYQNAYPEEVIPALITRLGSEKRVHITAVYYHQEVCVEEKAVEEALKVSLTNLYG
HENATAVACRALRGSSTLYHPDDLPPTASVAALPPVFTTWRKLVERCGRVRAPLAMVEPSQVHPLPFSIPCDR
VPSSVRDLIGENCEQERFRQQLTWSLPTFSDMRMDARNAFPFRGGETASQERLQAYTFGSDAIARYKETRNGL
VGTEYSTKWSPYLAIGNITPRQIYHTLRQYEEMRPEKKGVSTYWAVFELEWRDFFRFAAMRYGNALFWRSGLR
AVIQFELDKLSKHRRWRQDPVMLKTWQCGLTGFPFVDANQRELLATGWMSNRGRQNVASFLAKDLELDWRLGA
EWFESLLLDYDPCSNYGNWQYCAGVGLDPRADRHFDVIRQARMYDPDGTFMAQWLPELASCTNWFDIPRIHSQ
YPHYPRPVVSQLNRGRKQIDAGFADRPKQVASEGSSQDRRRKPLAGARQREPRWMRRTHSGPADTMDTKTSSS
EAKQTVLDHFGWSSKQRR
>Cyanidiococcus_yan_KAF6001522.1_F1559_0025
MLPRQALWIVQWKPVLESRQGSVLAWPWLHRVRIGVTLHCRSKRCRQHVNGTSQLPHLRFCLSRSSVFTSVCA
QDRMPSRVEVKTESSVVEPERVHVANTKPIRPERRYVLYWVQASPRIPENHALEYAIERANSLRKPLVACFGV
TDRYPEANIRHYRFLLEGLYELMHDLRSKRGIRLHVLYGSPDQVAAQCARDAACEVITDLGYMRVQRRWRESL
AETLDCRLTLVESECVVPVALASEKHEFAARTLRPRIWKHIQRFCVPLSVQNLHHKDLDVSVDYGPYAVLFGQ
ASMTGAGTQHPVDSALEKLESLDRSVPSVSKYFRGGTSEAMRRLELFCTHKLAHYGQGRNHPEKDDTSCLSAY
LHFGQISPITIALAVWARKEAPRESRDAFIEELIVRRELSFNHCWYRPDDYDRLDSLPDWIHRTKEEHRQDAR
EHLYDLETLERARTHDPYWNAAQQEMVLTGRMHGMMRMYWAKKVIEWTPSWECAYDWLIYLNNKYELDGRDAN
SFVGVLWSFGLHDRAHAERPIFGKLRWMSREGLERKFPMHAYLERVNALARGMSWEEATASGLASRDAQTKSK
KRPRR
>Cyanidiococcus_yan_KAF6001604.1_F1559_0040

MQPLECRLATFVAKPASLLRRHSSAEKSWLAQERASLFQNKTQRGPLSFQKFGTSTRRAYLNMRKTKRRSSET
STTEARRPHPSGLASGRSAAGPSSASSSTTIIWLRGRGDLRVYDNPLLVHAQREKQPLIIIFTWSQADATGPF
SYGDAQRAFMFSVIDELHRDLKRRYIQDIHYVRLRDQACASDYVELFDYLGKTLRANVLLLARRYEPPWRQLD
DEIEHALKTMNRWKVFSYNMHLLHEPGAVGYTGDSPATFGHFGTLLPFYRAWERNAGHPPQPSEAVSWLPEAP
QNLPWPETFSATIGDVNASEVIPECRELSTSSFSLVPSLQSTGLHPVGAGVARWDITVMRSWDVREEAVRAQL
RQFLQDRLPRYESSKSRADRQSVSRLSPFLALGVVSVREMYHETRDALRRLPEHKRSKTFARRLIWRELAYWT
LHHFPEAAMEPIRSHYTTQEWNEDPELIAAWQQGRTGYPLIDAGMRQLWQTGWMPQNIRMAVASFLIEYLNIH
WMHGLQWFHHTLVDLDIAINAMMWQNAGRSGLDMWNFVIHPVTSSRTSDPTGAYVRQWIPELSQLPNEFIHAP
WAAPESVRQAAAVRLGDTYPERIVNDLAEARRRSKRAVLEMRCRTEPHFFNDDGYDRIRVPILETGQSRLEHT
GRSAGPHLYVWRHVFTRREFRGAPFGTLLSEQAASTKAPQLQPVALQDTPAAQGKIAEEAS
>Cyanidiococcus_yan_KAF6002947.1_cry-1_
MPFDANALEESWQPGIDTVQLRLQHFLESVFPAYGDTRRRQRPQGGTSRLSPYLRFGELSPRRLYHAVRAFVL
QCEPSDAIIRAARTLLKNLCMREYGYHLLFYRPSVVDRPLVPEFEALPYARDEMKRYLEAWVSGHTGYPLVDA
AMRELRHTGWLHANMRFLVASFFTKYLLLPWQLGARYLYKTLIDGDLASNSLGWQWVTGCNNDVFPLNCLVNP
VVMGMRIDPDGEYCTNLVPRTEAYT
>Cyanidiococcus_yan_KAF6004024.1_cry-2
MWVVMNGSSAVHWFRKGLRLHDNPALLDAVKNPATRYVLPVFCLDPVFLQPELVGVNRIQFLLECLQDLDRQL
RQRRSRLFVLRGDPLEQFPAFFRRYKTTLLTYEFDTEPYAKHRDERVRQLCTDLGIDVRTRATHTLYDPELVR
CKLGERPTPLTYKSFYSFVTGTLGRPPASMPAPTTADDDIHTPDIELDAYKVPTLAELRAYDGLSVTTSFRGG
ETEALAIMERFLTERQADVRAFSKPQTSPTAWPKASTTQLSPYFKFGCLSPRLFIERVQELHPLEEPPVSLIG
QIWWREFFTYVAYVTPNFHQMRGNRICRQISWREDRDDPLYRAWEEARTGYPWIDACMTQLRKEGWLHHLARH
AVACFLTRGDLWVSWERGRETFDRLLVDADYSLNSANWLWLSCSAFFHQYYRVYSPIGFPQKYDPNGDFVRHF
LPVLREMPAKYIYTPWEAPLAVQRKAHCIIGEDYPSPIVDHATASRENMERMRKAVSESGPQRPPKRKRDDTG
ASTSSPIP
>Cyclospora_cay_XP_022589613.1_photolyase
MPRGQPEGERICTGDKRRLEESATQLPGAGKHPQKKGCSETSSSLCWLKDSSVDHRRVRCLTPARTQPNTLGK
TVVCLLQRDLRLQDNWALLFAQEAALTLGTPLYVLHLVIPGHTFQPTIRHVSFHLSSLEEFATDLANQSIGFR
CIPISTADDDKASTCEARSCIESTVTELKPCLMVCDLMPLRRPSELVKQAANVCVTKGQCPLYQVDSHNIVPV
WLASDKQEYSARTFRVRVTRFLREFACEIPRIGQHPHKLQTEDGQTLKIPDILNRLKLDHSVRLPSQWLPGSK
AGLAALQEFCTRPRLLAYASRNDPLERGQSGLSPWLHFGQLSSQRCLLTINSLGPGTKIGALAAAARESFIEE
LVVRRELAENFIFYNSKYDEIQGAPKWAQDTLNAHRGDKREHIYSLSHFEGAKTHEALWNAAQLQLVHDGKMH
GFMRMYWAKKILEWAASPESALKISLHLNDKYSLDGTDPNGVVGCMWSIAGVHDQGWAERPIFGKVRYMNLAG
CKRKFSVDEFIRRTSVVAATALKVAKKG
>Cygnus_atratus_XP_035395284.1_cryptochro
MAAAAAAPSRSVHWFRRGLRLHDNPALQAALRGAASLRCIYILDPWFAASSAVGINRWRFLLQSLEDLDNSLR
KLNSRLFVVRGQPTDVFPRLFKEWGVTCLTFEYDSEPFGKERDAAIVKLAKEAGVEVVIENSHTLYDLDRIIE
LNGNKPPLTYKRFQAIISRMELPKKPVSSVTSQQMETCKVDIQENHDDVYGVPSLEELGFPTDGLAPAVWQGG
ETEALARLDKHLERKAWVANYERPRMNANSLLASPTGLSPYLRFGCLSCRLFYYRLWELYKKVKRNSTPPLSL
YGQLLWREFFYTAATNNPKFDRMEGNPICIQIPWDKNPEALAKWAEGKTGFPWIDAIMTQLRQEGWIHHLARH
AVACFLTRGDLWISWESGVRVFDELLLDADFSVNAGSWMWLSCSAFFQQFFHCYCPVGFGRRTDPSGDYVKRY
LPKLKGFPSRYIYEPWNAPESVQKAAKCIIGVDYPKPMVNHAETSRLNIERMKQIYQQLSRYRGLCLLASVPS
CVEDLSGPVADSASGQGSSTSAAVRLPQSDQASPKRKHEGEELCTEELYKRAKVTGLPASEIPGKSL
>Cygnus_atr_XP_035400705.1_photolyase
MPRGSGKRKASGGGEAAKRREEEEEEEEAAGRLEAALREARRAAAPSVREFRYNKKRVRLVSRGPELREDAKC
ILYWMSRDQRVQDNWAFLYAQRLALKQELPLRVCFCLVPKFLGATIRHYGFMLRGLQEVSKECTELNVPFHLL
LGYAKDVLPPFVVQQGVGGLVTDFCPLRVPRQWVEDVRERLPEDVPFVQVDAHNIVPCWVTSPKQEYSARTIR
GKIHSQLPEFLTEFPPVIRHPHPPSCPTEPIAWDACYSSLQVDRSVAEVEWATPGTSAGLAVLQSFIAERLKS
FGSQRNDPNKAALSNLSPWFHFGQVSTQRAILEVQKHRRAHKESVDAFVEEAVVRRELADNFCYYNENYDSLQ
GAYDWAQTTLKLHAKDKRPFLYELQQLEQATTHDPLWNAAQLQMVREGKMHGFLRMYWAKKILEWTRSPEEAL
RFAIYLNDRYELDGRDPNGYVGCLWSICGIHDQGWKERDVFGKIRYMNYSGCKRKFDVDQFERRYAR
>Cygnus_atr_XP_035410259.1_cry_DASH-l
MARAAVCLLRCDLRAHDNQVLHWAHNNADYIIPLYCFDPRHYVGTHCYGFPKTGPHRLRFLLESVKDLRETLK
KKGSTLVVRKGKPEDVVSDLITQLGCVGVVAFHEEATQEELDVEKGLCQVCGQHGVKVQTFWAATLYHRDDLP
FGPIARLPDVYTQFRKAVESEAKVRPTLQLADQLKPLAPGVEEGCIPTMEDLGQKDPGTDPRTAFPCSGGETQ
ALMRLQYYFWDTNLVASYKETRNGLIGMDYSTKFAPWLALGCISPRYIYEQIRKYEKERTANRSTYWVLFELL
WRDYFRFVALKYGRRIFSVRGLQSKEVPWKKDLQLFDCWKEGKTGVPFVDANMRELAATGFMSNRGRQNVASF
LTKDLGLDWRMGAEWFEHLLVDYDVCSNYGNWLYGAGVGNDPRDNRKFNMIKQGLDYDGNGDYVRLWVPELRG
VKGAAAHTPWALSSAALSQAGVTLGETYPQPVVTAPEWSRHISQRPQGRSPHPRGRRGPARTPAQHKDGGIDF
YFSRKKDV
>Cygnus_atr_XP_035425030.1_cry-1-like

MPRRTIHLFRKGLRLHDNPALLAALESSEVVYPVYILDRKFMTSVMHIGALRWHFLLQSLEDLQKNLCRLGSR
LLVIQGEYELVVRDHVQKWNITQVTLDAEMEPFYKEMEANIRCLGEELGFKVLSVVGHSLYDTKRILDLNDGA
PPLTYKRFLHILSLLGDPEAPVRNLTAEDFQRCRPPDLDLAECYRVPLPADLKIPPESISPWRGGETEGLQRL
EQHLADQGWVANFTKPRTILNSLLPSTTGLSPYFSMGCVSVRTFFYRLSNIYAQAKHHSLPPVSLQGQLLWRE
FFYTVASATPNFTKMAGNPICLQISWYEDAERLHKWKTAQTGFPWIDAIMTQLRQEGWIHHLARHAAACFLTR
GDLWISWEEGMKVFEELLLDADYSINAGNWMWLSASAFFHQYTRIFCPVRFGKRTDPEGQYIRKYLPVLKNFP
SKYIYEPWTASEEEQKQAGCIIGKDYPFPMVDHKEASDRNLQLMKHIREEQYRTAQLTRDDTDDPMAMKLKRD
HSEENVTKAKAARMTEQT
>Cygnus_atr_XP_035425130.1_cry-1
MGVNAVHWFRKGLRLHDNPALRECVQGADTVRCVYILDPWFAGSSNVGINRWRFLLQCLEDLDANLRKLNSRL
FVIRGQPADVFPRLFKEWNIAKLSIEYDSEPFGKERDAAIKKLASEAGVEVIVRISHTLYDLDKIIELNGGQP
PLTYKRFQTLISRMEPLEVPVETITPEVMEKCTTPVSDDHDEKYGVPSLEELGFDTDGLPSAVWPGGETEALT
RLERHLERKAWVANFERPRMNANSLLASPTGLSPYLRFGCLSCRLFYFKLTDLYKKVKKNSSPPLSLYGQLLW
REFFYTAATNNPRFDKMEGNPICVQIPWDKNPEALAKWAEGRTGFPWIDAIMTQLRQEGWIHHLARHAVACFL
TRGDLWISWEEGMKVFEELLLDADWSVNAGSWMWLSCSSFFQQFFHCYCPVGFGRRTDPNGDYIRRYLPVLRG
FPAKYIYDPWNAPESIQKAAKCIIGVNYPKPMVNHAEASRLNIERMKQIYQQLSRYRGLGLLATVPSNPNGNG
NGGLMGFSPGESISGCGSTGGAQLGTGDGQTVGVQTCALGDSHAGTSGIQQQGYCQASSILHYAHGDNQQSHL
LQAGRTSLGTGISAGKRPNPEEETQSVGPKVQRQSTN
>Cynoglossus_semi__XP_008306150.1
MARSSRTVICLLRNDLRLHDNELFHWAQRNAENIVPLYCFDPRHYVGTYNYNFPKTGPFRLRFLLESIRDLRN
TLISKGSNLVVRRGKPEDVVAELIQQLGSISTVVFHEEVTSEELNVEKKIKDVCAQMRVKVHTCCGSTLYHRD
DLPFRHPSELPDVYTQFRKAVETRGRVRSVFPTPDQLKPPPPGLDEGVIPTAEDLQQTEPLSDPRSAFPCTGG
ESQALARVQHYFWDTNAVASYKETRNGLMGVDYSTKFAPWLAIGCISPRYIYHQIKQYETERTANQSTYWVIF
ELLWRDYFRFVAVKYGNRLFQVKGLQDKSVQWKQDMRLFTAWKEGRTGVPFVDANMRELALTGFMSNRGRQNV
ASFLTKDLGLDWRMGAEWFEYLLIDYDVCSNYGNWLYSAGVGNDPRENRKFNMIKQGLDYDSNGDYIRQWVPE
LQRIVGADVHTPWTLSSAALSHAQVTLGETYPTPIVVAPEWSRHVNKKSSASGPSPRGKKGPSHTPKQHRDRG
IDFYFSKSKNL
>Cynoglossus_semi__XP_008309446.1
MAVNSVHWFRKCLRLHDNPALQEAVNAAHTLRCVYILDPWFAGAANVGINRWRFLGEALEDLDNSLKKFNSRL
FVIRGQPTDVFPRLFKEWKVTRLTFEYDPEPYGKERDGAIIKMAQEFGVETVVRNSHTLYNLDRIIEMNNNSP
PLTFKRFQTIVSRLELPRRPLPVITQQQMEKCCTKISENHEQLYSIPSLEELGFRTERLPPAVWRGGESEALD
RLNKHLDKKVWVANLEHSRINTCSLYASPTGLSPYLRFGCLSCRVLYYNLRELYMKLRKHSSPPPSLFGQLLW
REFFYTAATNNPNFDRMEGNPICVQIPWDQNPEALAKWAEGRTGFPWIDAIMTQLRQEGWIHHLARHAVACFL
TRGDLWISWESGMKVFEELLLDADWSVNAGSWMWLSCSAFFQQFFHCYCPVGFGRRTDPSGDYIRRYVPILKD
YPNRYIYEPWNAPESVQKAANCVVGVDYPKPMINHAEGSRLNIERMKQVYQQLSHYRGLSQLASVPTIQEEAE
PPMTDESQTSSGPDSPPAGGALTEAAGCSILPDSSTLCPTVVTAHQSSDCPQTSAAVTSTSASVNADPLKPPS
PCSSSSSPCGTTSPPSTSAQTLTKRKGPAHKIRRSMRQRGHQGAEERREDEEDKGREERMEDDDDENEESMEQ
DATGEMSGHRQ
>Cynoglossus_semi__XP_024911595.1
MVMTHNCIHWFRKGLRLHDNPALVSALRDCKDLYPVFVLDPHLYNSDHMGINRWRFLVGCLHDLDCSLRKLKS
RLFVLRGHPEDVFPELFKKWDVTKLTYEYDTEPYSLSRDGKVTELAERHGVEVVYKISHTLYDMDRVIEENNG
KAPLTYNRMQAILKSLGPPKKPVPAPTLEDMNEIKTPYTENHEEEYGIPPLGELCEDTAAPGEEKFPGGEEEA
LRRLDEHMKRTAWVCKFEKPQTSPNSLSPSTTVLSPYVTFGCLSARTFWWRLAGGYQGNKHSSPPVSLHGQLL
WREFYYAASVGIPNFNKMVGNPVCTQVDWDSNPELLAAWREARTGFPFIDAIMTQLRQEGWIHHLARHAVACF
LTRGDLWINWEEGQKVFEKLLLDGDWALNAGNWLWLSASAFFHQFFRVYSPVAFGKKTDKNGDYIKKYLPLLK
KLXYIYEPWKAPCSVQQAAGCIVGKDYPQPIVKHEEIHKKNIQRMKLAYAKRSSDAAVSPNKKQGLKRKASSV
ADLLQSKGKKK
>Cynoglossus_semi__XP_024915112.1
MARNSIHWFRKGLRLHDNPALREAVRGADTVRCVYFLDPWFAGSSNVGVNRWRFLLQCLEDLDASLRKLNSRL
FVIRGQPANVFPRLFKEWKISRLTFEYDSEPFGKERDAAIKKLAMEAGVEVNVKISHTLYDLDRIIELNGGQP
PLTYKRFQTLISRLDPPEMPAETMTDSTMGGCVTPISDDHGDKYGVPSLEELGFDTEGLPTAVWPGGETEALT
RIERHLERKAWVANFERPRMNANSLLASPTGLSPYLRFGCLSCRLFYFKLTDLYRKVKKNSSPPLSLYGQLLW
REFFYTAATNNPRFDKMEGNPICVRIPWDKNPEALAKWAEAKTGFPWIDAIMTQLRQEGWIHHLARHAVACFL
TRGDLWISWEEGMKVFEELLLDADWSVNAGSWMWLSCSSFFQQFFHCYCPVGFGRRTDPNGDFIRRYLPVLRG
FPAKYIYDPWNAPESVQAAAKCVIGVHYPKPMVHHAEASRLNIERMKQIYQQLSRYRGLGLLASVPSTNGNGN
GGMMAYSPGEQQPGTNGNSNTVHLPAVSGSSVATSNGSGSILLNFDSEEQTRPNSVGQRQQPLQQ
>Cynoglossus_semi__XP_024921971.1
MTVISRSLSPTPAKSSLKLLACTSSSLSKVLSLLVEKFTCKAAMLSTKRKASSAAKAPKAKQQKGPPVKEEEK
KKKKPESGGWLQNLVMQKRTKQKEMEFNQKRLRFIRDAKIKRGSAGVVYWMLRDQRVQDNWALIFAQKLAARK
NLPLHICFCLMVPKSTLSTLRHYSFMLKGLKEVAKECKALNVQFHLLHGSAAEVLPGFVSSHSLGAVVTDFSP

LREPLQWLEDVEKRLPTDIPIIQVDAHNIVPCWVASPKLEYAARTIRGKITKHLTEFLTGFPLVERHPFTARE
KAKKIDWDQTLSSLKVDRTVKETNWAQPGTCGGMAMLESFIDFRLKLFGNQRNDPNINALSQLSPWIHFGHLS
AQRVALQVQKNGKSAAESVTSFIEELVVRRELADNFCFYNKKYDRVEGAHEWAQKTLRDHAKDERPYLYTLQE
LEKAKTHDNLWNAAQYQMVREGKMHGFLRMYWAKKILEWTSSPEEALSIALYLNDRYELDGQDPNGVVGCMWA
ICGVHDQGWAERPVFGKIRYMNYKGCTRKFNVAQFEQKYCPQKCQKLRRF
>Cyprideis_toro__CAD7224866.1
MGLYGEILSHILEWDCMARFFLILEWNSVVADFSPLKIALQWIEGVKGALDKMDVPLIQVDAHNVVPVWETSD
KQEYAARTIRRKIHDRLKEFGTEFPPLVPHPAKEAAERIDWQALYDWLEVDESIGPVKWATPGTAAGLRVLEE
FLTKRMKKYEKRNDPNEEALSNLSPWFHYGQVAPQRAILEVSLSRKANPKAADSFIEEALVRRELSDNFCFYN
PRYDSIEGAADWARKTLNDHKNDVREYLYSFEEFEGSRTHDDLWNAAQIQLVREGKMHGFLRMYWAKKILEWT
TSPEEALAFAIKLNDKYELDGRDPNGFVGCMWSICGIHDQGWTERPIFGKIRYMNYAGCKRKFDVVKFVNKYG
AKAYPYNKGGTRKVAAGGSTSAAASTSRKRASTPAKSTAAKKAKK
>Cyprideis_toro__CAD7229706.1
FLLEALEDLDRSLRKLNSRLFVIRGQPADVLPKLYKDWQITHLTMEAEPEPFGRARDKNIIAISREYGVHVSI
KTSHTLYRLDSIIERNGSVPPVTYRQFQLILQSMDPPPPPVAAVTMDTVGSSKTPLRQDHEDEYGVPTLEELG
FEVEAKDHSSALWVGGESEALSRLERHLERKAWIASFGNPKFSPQSLMASQTGLSPYLRFGCLSCRLFFQELT
KLYRQVRKSPPPVSLYGQLLWREFFYTAATKNPKFDRMVGNPICLQIPWDKNPQALAKWATGNTGFPWIDAIM
RQLRIEGWVHHLARHALACFLTRGDLWLSWEDGMKVFEELLLDSDWSINAGTWMWLSCSSFFQTFFHSYCPVK
FGRKADPSGDYIRKYVPVLKNFPTEYIHEPWIAPLEVQQQAKCVIGRNYPLPMVNHAEASRRNFERLRQVYRS
LPRPGGFSVFRMDQKPSARPFRAAPPPPLPARGAHLISAKLQQAASILSTPSSTTKHEPPKFFVDLLTGDEPS
VAAQTNSSSSIRSRSQATSARKKVPPPRPPPPKKKVTPVSGISAKSDVLVDIGSDGGSIHSSGSDAGSGPQFP
PSSMSVVDAFDPFSPVSWGQRSSFSTVSVASLSNSAPQRSSSLYSRPPAAPSSGAPSSVLSDLRDIFQSSLAS
SNHPSPTVFGNGTVTQGVNWTATSSNATGVQSRTTSVKRDSPVVTRKAFEDETLITSASNAVADLPKWKPTVI
RARPISTPPVSTTVQTSTVSSTTSASITSATSIGENMNGSRTPELTAKSWSVSTDSLATASSSTLSAGPSLPP
RPKTSEIPYAVALYDYDSGNPEDLVFKAGDTILLHHWINEDWLEGSISGVTGMFPANFIQAGVLVHVTGKINK
EWLTGSYRGFRGQFPVSFVSHVPKRFRES
>Cyprideis_toro__CAD7232001.1
MGLIGYNRMRFLCECLRDLDAGFRLYGGRLFVVESKMPHVLQQLSKEFRIEKITWDQDCEALYHPRDDAVKET
CETLGIAWIERLGHTLWNPEDILEANGEEPPLTQAAFLQVATSLGHPDRPRGKVDLSDVSFLKSDGVSDGLKK
ELKLFDGVPDPADFGFRATRPHETPYWMGGECRGLDLLSRRLEQEARAFQDGICRVTQTRPNILAPATSLSPY
LRFGCVSVRRMYWEIQDLYEEVRGGASTAHVHLIDQLIWREFFYVMSARNPHYDKMEENPICLNIRWDDDPVA
LACWEEGMTGYPLIDAFMRQLKEEGWIHHLGRNLVAQFLTRGAMWLSWVPGLRHFLRYQLDADWSVCSGNWMW
ISSSAFEKVLDCSHCLSPVTFGKRLEPSGEFVRRYVPELQNFPTEWIYQPWQAPESVQEKSGCVIGKDYPLPI
VDYAQASQRCRYVSVFQKNVIMNLSFYLRIPE
>Cyprinodon_variegatus__DASH_XP_015255163.1
MSASRTVICLLRNDLRLHDNELFHWAQRNSEHIVPLYCFDPRHYVGTYNFNLPKTGPFRLRFLLESVRDLRNT
LLSKGSNLVVRKGKPEEVVADLIKQLGSVSSVAFHEEVASEELNVEKKVKEVCAQLKVQVHTCWGSTLYHRED
LPFPHISRLPDVYTEFRKAVESKSRVRPVLPTPERLNPLPPGLEEGAIPTAEDLQQTEPVSDPRSAFPCAGGE
SSVLARLQHYFWDTDAVATYKETRNGLIGVDYSTKFSPWLALGCISPRYIYHQIKKYERERTANQSTYWVIFE
LLWRDYFKFVGVKYGNKMFQLKGLQGKSVPWKMDTKLFDAWKEGRTGVPFVDANMRELALTGFMSNRGRQNVA
SFLTKDLGLDWRLGAEWFEYLLIDHDVCSNYGNWLYSAGIGNDPRENRKFNMIKQALDYDNNGEYVRRWVPEL
GAIRGPDVHTPWNLSSASLSHAGVSLGETYPTPMVTAPEWSRHFGKKPSGAGPSPKGKKGPSHHPKQHRDRGI
DFYFSKSKNL
>Cyprinodon_vari__XP_015226684.1
MGVNTIHWFRKGLRLHDNPALRASIQRADTLRCIYILDPWFAGSSSVGINRWRFLLHCLEDLDASLRKLNSRL
FVIRGQPTDVFPRLFKEWNITQLSYEYDSEPFGKDRDAAIQKLASEAGVEVIVRISHTLYNLDEIIEVNGGQP
PVTYKRFQGLINRMDDVEQPAETISSEMLKNCITLVTDDHDDKFGVPSLEELGFETEGLSTAIWPGGETEALK
RLERHLERKAWVANFERPRMNSNSLLATPTGLSPYLRFGCLSCRHFYFRLTDLYKKVKKNSNPPLSLYGQLLW
REFFYTTATNNPCFDKMEGNPVCLQIPWDRNPEALAKWAEGRTGYPWIDAIMTQLRQEGWIHHLARHAVACFL
TRGDLWISWEEGMKVFEELLLDADWSVNAGSWMWLSCSSFFQQFFHCYCPVGFGRRTDPGGDYIRRYLPVLRG
FPAKYIYDPWNAPEEVQKAAKCIIGTNYPKPMVNHGESSRVNIERMKQIYQQLSCYKGLGLLATVPANPNSNS
SIGDVSTGSAHYQKGFFEIHPSQEETSQTERRQSTLKRHHEKTEPECSFKSWKQRK
>Cyprinodon_vari__XP_015226847.1
MSDKKRKAPSAAEQGPSAKQPKEEKAAGWLESQVKQQREEKKDMKFNKKRQRFLTDSQKMKQGSEGVLYWMLR
DQRVQDNWALIRAQQLAAKEEVPLHVCYCLLVPKSELSTLRHYAFLLKGLEQVAKACKKLNIQFHLLHGAPGE
VLPGFVSDQGFGAVVTDFSPLREPQQWLNAVKKKLSKDIPLIQVDAHNIVPCWVASPKLEYSARTIRGKITNL
LPEFLTDFPPVEKHAHTATRTAKPVDWEQTLSSLDVDRTVGEPEWAKPGSKAGMAMLESFIDVRLKLFATQRN
DPNMAALSQLSPWIRFGQLSAQRVALQVRRCGKNAGESVAAFIEELVVRRELTDNFCYYNEKYDQVEGAYEWA
QKTLKDHAKDKREYIYTREQLENAKTHDKLWNGAQYQMVTEGKMHGFLRMYWAKKILEWTRSPEEALSIALYL
NDRYELDGQDPNGFVGCMWSICGIHDQGWAERAVFGKIRYMNYKGCLRKFDVARFERKYRPKTA

>Cyprinodon_vari__XP_015227078.1
MAHVCIHWFRKGLRLHDNPALIAALKDCKELYPVFIMDPNLHSTEVGINRWRFLIGALKDLDSSLRKLNSRLF
VLRGKPEEVFPKLFSKWKVTKLTYEYDTEPYSLSRDEKVTSLAKETGIDVIYKISHTLFNIERIIEENDGKPP
LTYKRLQAIVAKLGPPKKPIPAPTLDDMKDVKTPCSEKDEKKYGIPTLEEFGLDSTSLGEELFPGGEQEALRR
LDEHMKKTGWVCSFEKPQTSPNSLSPSTTVLSPYVTFGCLSARTFWWRLTEVYRGKKHSDPPVSLHGQLLWRE
FFYTCSLGIPNFNKMKGNTVCTQVDWDTNQDYLAAWREARTGFPFIDAIMTQLRQEGWIHHLARHAVACFLTR
GDLWISWEEGQKVFEELLLDGDWALNAGNWQWLSASAFFHQFFRVYSPIAFGKKTDKNGDYIKKYLPLLEKFP
PQYIYEPWKAPRSVQQAAGCIVGKDYPHPIVQHEQVSKKNIQRMKEAYARRSTASSESPIKKQGEKRKSLSVV
DMLQKKARKE
>Cyprinodon_vari__XP_015239296.1
MVVNSVHWFRKGLRLHDNPALQEALSGADTVRCVYVLDPWFAGAANVGINRWRFLLESLEDLDNSLKKLNSRL
FVVRGQPTEVFPRLFKEWNVTRLTFEYDPEPYGKERDAAIIKMAQEFGVEAVVRNSHTLYNLDRIIEMNNNNP
PLTFKRFQTIVSRLELPRRPLAPISQQQMNRCLSNISDNHDQLYSIPSLVELGFRTEGLPTAVWRGGESEALD
RLSRYLDKNVNLGREQEPHISLKSTLMGTFLXSPPLLSHVIVSCPVAVRYSDALKQDQTDLLLCQLCKRSSPP
LSLYGQLLWREFFYTLATNNPNFDRMEGNPICVQIPWDQNPEALAKWAEGRTGFPWIDAIMTQLRQEGWIHHR
ARHAVACFLTRGDLWISWESGMKVFEELLLDADWSVNAGSWMWLSCSSFFQQFFHCYCPVGFGKRTDPSGDYI
RRYVPILKDYPNRYIYEPWNAPEALQKAVNCVVGVDYPKPMINHAEGSRLNIERMKQVYQQLSHYRGLSLLAS
VPTIQEEAEPPMTDESQTSSGPDSPLRDLVEVEAAGCSTVPDSSTSIPHSDQEDTSHHHLFLDHCTSSGQPPS
PTSSPTRGATSRHKPGSPSCSCLSPSTSPSSDPLSQRQRGCQSCPVEEERMQEEVDEEKMEEEAAPQQ
>Cyprinodon_vari__XP_015239459.1
MIVMSYRSPWENRGTERVAGDQAVVRQMLREVLVGREDPECFFAMCLSVLGNQETRSEFLSIIKPLSTANIRL
HSVLRSLYQDYFTKTEDDELEIALALSLLEMKENQMSAPNQLVLQAGNDQSSLPLSSKPTSQEKSFSKQEDVP
GREKSEFTEQSRSKTKETFLDIENDKSLSFKSGDNQSLGSSQPPYIPGSSASFTIPTTASKSDQMIRGDGNVS
EKPKRPKSRRQRRKAAAQQVVGLIRSPSAPPPVLLWFRRDLRLCDNPALIGSLEVGAPVIPIFIWSPEEEEGP
GVTVAMGGACKYWLHQALSCFRASLEQIGSHLVFFKASNDVTEVGSSLNTLKTLVKETGAQSVMANALYEPWL
KERDDAVSSALQKEGVEFKMFHSYCLRDPFSVSTEGVGLRGIGSVSHFMSCCRQNPGAAVGVPLDPPVSLPSP
AHWPQGVPLDALGLARMPRRKDGTIVDWAANIRKSWDFSEDGAHARLEAFLHDGVYRYEKESGRADAPNTSSL
SPYLHFGQLSPRWLLWDAKGARCRPPKFQRKLAWRDLAYWQLTLFPDLPWESLRPPYKALRWNTDRSHLKAWQ
KGNTGYPLVDAAMRQLWLTGWMNNYMRHVVASFLIAYLHLPWQEGYRWFQDTLVDADVAIDAMMWQNGGMCGL
DHWNFVMHPVDAAMTCDPYGSYVRKWCPELSDLPDELIHKPWKCPASMLRRAGVVFGQTYPERIVIDLEERRT
KSLQDVALVRKQFQQYVDKRSGCDLVPLPKRLVSEALGLSHRDGAVVTEGKQFLLPVITRMEFKHQLEDPDAD
AASNPYNAVLKGYVSRKRDETIAFLNERDFTASVMYEGAQRRERLERDHRRLEGLPPPPSARGKARRTPTAKD
RFSVVPGGAIVSKN
>Danaus_ple_ple_OWR51555.1_KGM_210113
MQHTVYDFNSVVKKNNGSIPLTYQKFLSLVSDVQVKDIIQISKGVSDECKASDYDSQGYDIPSLEEFGVNESE
LSECKYPGGESEGLKRLDVYMAKKQWVCNFEKPKSSPNSIEPSTTVLSPYISHGCLSAKLFYHKLKQVENGSK
HTLPPVSLMGQLMWREFYYTAGSGTENFDKMVGNSVCTQIPWKKNDAHLKAWAEGKTGYPFVDAIMRQLKQEG
WIHHLARHMVACFLTRGDLWISWEEGAKVFEDFLLDYDWSLNAGNWMWLSASAFFYKYYRVYSPVAFGKKTDK
DGLYIRKYVPELKKYPSEFIYEPWKAPKGVQKTAGCVIGEGYPNRIVDHDKVHKDNIQKMNSAYKVNKEKKAM
KRPRQ
>Danaus_ple_ple_XP_032522602.1_cry-1
MLGGNVIWFRHGLRLHDNPSLHSALEDASSPFFPIFIFDGETAGTKMVGYNRMRYLLEALNDLDQQFRKYGGK
LLMIKGRPDLIFRRLWEEFGIRTLCFEQDCEPIWRPRDASVRALCRDIGVSCREHVAHTLWNPDTVIKANGGI
PPLTYQMFLHTVEIIGNPPRPVDDVDLNGVNFGSLPESFYREFVVFDKAPKPEDLGVFLENEDIRMIRWVGGE
TAALKQMQERLAVEYETFCRGSYLPTHGNPDLLGPPISLSPALRFGCLSVRRFYWSLQDLFQQVHQGRLASTQ
FITGQLIWREYFYTMSVNNPNYAQMSGNPICLDIPWKEPENDELQRWKEGRTGFPFVDAAMRQLRTEGWLHHV
VRNTVASFLTRGTLWLSWEHGLQHFLKYLLDADWSVCAGNWMWVSSSAFEALLDSGECACPVRLGRRLEPTGH
YVRRYVPELARMPGEYIYEPWRAPLEVQEAAGCVIGRDYPAPVVDHTAAAARNRANMQELRRLLEKAPPHCCP
SSEDEVRQFMWLGDDSQPELTTT
>Danaus_ple_ple_XP_032529216.1_photolyase
MKIKFLTHFTTKMASSAKKPKLLLPIKNESSTINVDDFMKQIHKKREETAKSILDFNFNKSRLRIISQEQMVS
DDCEGIVYWMSRDSRVQDNWAFLYAQELALKNKVPLHVCFCLIAKYLDASVRQFHFLIKGLEKVAADCDKLNI
SFHLLEGNGAEVLPQWVIDHRIGAVVCDFNPLRVPLGWVEGAKKKLKKDVPLIQVDAHNVVPCWVASNKQEYS
ARTIRNKINSKLDEYLTEFPPVIKHPHSSSFKPEPIDWDKAIETREADKSVGPIGWAGPGYDNAVKTLKSFLD
KRLKVFATKRNDPTQDALSNLSPWFHFGQISAQRVALCVKEYKTKYTESVNSYLEEAIVRRELADNFCFYCEH
YDSIKGASQWAQKTLDDHRNDKRTHIYTLEQFCKAETHDDLWNSAQIQMVKEGKMHGFLRMYWCKKILEWTSS
PEEALKYAIYLNDHYSVDGRDPSGYVGCMWSICGVHDQGWAERAVFGKIRFMNYDGCKRKFNVPAFVCRYGGK
VHKYNNLTDKQKKK
>Danaus_ple_XP_032511499.1_cry-1-like

```
MSVAETLPLRARSPTAQKSSQPAGVPKEKHTVHWFRKGLRLHDNPALREGLVDATTFRCVFIIDPWFASSSNV
GINKWRFLLQCLEDLDKNLRKLNSRLFVVRGQPADALPKLFREWGTTALTFEEDPEPYGRVRDHNIMTKCREV
GIQVTSRVSHTLYKLDDIIEKNGGKAPLTYHQFQALIASMPPPPSAEPTISLETLNRAVTPISDNHDERFGVP
TLEELGFDTEGLKPPIWIGGENEALLRLERHLERKAWVASFGRPKMTPESLLSSQTGLSPYLRFGCLSTRLFY
YQLSELYKRIKQERPPLSLHGQILWREFFYCAATRNPNFDRMEGNPICVQIPWEKNQEALKKWANGQTGFPWI
DAIMIQLRNDGWIHHLARHAVACFLTRGDLWISWEEGMKVFDELLLDADWSVNAGMWMWLSCSSFFQQFFHCY
CPVRFGRKTDPNGDFIRKYIPVLKNMPTRYIHEPWVCPEEIQKSIRCIIGKDYPMPIVDHTKASEINLERIKQ
VYAQLAKFKPQGALIPQMLQRPNVLQSSPSPTSIIANINQSNYLCSQSSDVPTPTNQTTNQFKEDAVFLKPTV
NNIKSNVDKQQQFKQVVIVQEDKHSENQRHSVGNKYIVNEINKNINDIPVKQNNYDFKALTLNLNKFSNEPLT
FLNQTPNKNESFGQDVNNVIDVYSTSKPKFYFTDNGVITHNENAQTFKRDSYSDNYNKESTGSNRVGEVHSNN
PQTDKISSEKKN
>Danio_rerio_BAA96850.1_cryptochro
MVVNSVHWFRKGLRLHDNPALQEALNGADTVRCVYILDPWFAGSANVGVNRWRFLLESLEDLDTSLRKLNSRL
SVVRGQPTDVFPRLFKEWNVTRLTFEYDSEPYGKERDAAIIKMAQEYGVETVVRNSHTLYNPDRIIEMNNHSP
PLTFKRFQAIVNRLELPRKPLPTITQEQMARCRTQISDNHDEHYGVPSLEELGFRTQGDSLHVWKGGETEALE
RLNKHLDRKAWVANFERPRISGQSLFPSPTGLSPYLRFGCLSCRVFYYNLRDLFMKLRRRSSPPLSLFGQLLW
REFFYTAGTNNPNFDHMEGNPICVQIPWDHNPEALAKWAEGRTGFPWIDAIMTQLRQEGWIHHLARHAVACFL
TRGDLWISWESGMKVFEELLLDADWSVNAGSWMWLSCSAFFQQFFHCYCPVGFGRRTDPSGDYIRRYIPKLKD
YPNRYIYEPWNAPESVQKAANCIVGVDYPKPMINHAESSRLNIERMKQVYQQLSHYRGLSLLASVPTIQEEAE
PPMSDDSQASSSSTGQASSPPHLSITAPSTPPLSESSSPNSSPTASTSVPHTQRKRVRPSETPSKQKTKVKHT
SQARGLELKMDDKQ
>Danio_rerio_NP_991249.1
MSASRTVICLLRNDLRLHDNEVFHWAQRNAEHIIPLYCFDPRHYQGTYHYNFPKTGPFRLRFLLDSVKDLRAL
LKKHGSTLLVRQGKPEDVVCELIKQLGSVSTVAFHEEVASEEKSVEEKLKEICCQNKVRVQTFWGSTLYHRDD
LPFSHIGGLPDVYTQFRKAVEAQGRVRPVLSTPEQVKSPPSGLEEGPIPTFDSLGQTEPLDDCRSAFPCRGGE
TEALARLKHYFWDTNAVATYKETRNGMIGVDFSTKFSPWLALGCISPRYIYEQIKKYEVERTANQSTYWVIFE
LLWRDYFKFVALKYGNRIFYMNGLQDKHVPWKTDMKMFDAWKEGRTGVPFVDANMRELALTGFMSNRGRQNVA
SFLTKDLGLDWRLGAEWFEYLLVDHDVCSNYGNWLYSAGIGNDPRENRKFNMIKQGLDYDNNGDYVRQWVPEL
RGIKGGDVHTPWTLSNSALSHAQVSLNQTYPCPIITAPEWSRHVNNKSSGPSSSKGRKGSSYTARQHKDRGID
FYFSKNKHF
>Danio_rerio_NP_001070765.2_cryptochro
MVVNTVHWFRKGLRLHDNPSLRDSILGAHSVRCVYILDPWFAGSSNVGISRWRFLLQCLEDLDASLRKLNSRL
FVIRGQPTDVFPRLFKEWNINRLSYEYDSEPFGKERDAAIKKLANEAGVEVIVRISHTLYDLDKIIELNGGQS
PLTYKRFQTLISRMEAVETPAETITAEVMGPCTTPLSDDHDEKFGVPSLEELGFDTEGLSSAVWPGGETEALT
RLERHLERKAWVANFERPRMNANSLLASPTGLSPYLRFGCLSCRLFYFKLTDLYRKVKKNSSPPLSLYGQLLW
REFFYTAATNNPRFDKMEGNPICVQIPWDKNPEALAKWAEGRTGFPWIDAIMTQLRQEGWIHHLARHAVACFL
TRGDLWISWEEGMKVFEELLLDADWSVNAGSWMWLSCSSFFQQFFHCYCPVSFGRRTDPNGDYIRRYLPVLRG
FPAKYIYDPWNAPESVQKAAKCIIGVHYPMPMVHHAEASRLNIERMKQIYQQLSCYRGLGLLAMVPSNPNGNG
ENSTSLMGFKTGDMTKEVTTPSGYQMPPTSQGEWHGRTMVYSQGDQQTSSIMTSQGFGNNGSTMCYRQDAQQI
TGRGLHSSIIQTSGKRHSEESGPTTVSKVQRQCSS
>Danio_rerio_XP_005168334.1
MLPEAIWSPSEVFSSWRKSVAMSHRTIHLFRKGLRLHDNPSLLGALASSSALYPVYVLDRVFLQGAMHMGALR
WRFLLQSLEDLDTRLQAIGSRLFVLCGSTANILRELVAQWGITQISYDTEVEPYYTRMDKDIQTVAQENGLQT
YTCVSHTLYDVKRIVKANGGSPPLTYKKFLHVLSVLGEPEKPARDVSIEDFQRCVTPVDVDRVYAVPSLADLG
LQVEAEVLWPGGESHALQRLEKHFQSQGWVANFSKPRTIPNSLLPSTTGLSPYLSLGCLSVRTFYHRLNSIYA
QSKNHSLPPVSLQGQVLWREFFYTVASATPNFTKMEGNSICLQIDWYHDPERLEKWRTAQTGFPWIDAIMTQL
RQEGWIHHLARHAVACFLTRGDLWISWEEGMKVFEEFLLDADYSVNAGNWMWLSASAFFHKYTRIFCPVRFGR
RTDPQGEYLRKYLPVLKNFPSQYIYEPWKAPEDVQLSAGCIIGKDYPRPIVSHIEASQRNLALMRQVRTEQQT
TAELTRDVADDPMEAGLKRELREEEGLLEEAESQCTSKRFSGSSDHKSRPCSWTPETLQLSELSGEVM
>Danio_rer_NP_957358.1_CPD_photol
MSANKNNLKRQMKSTISAGGKQPKLTGEKGKESGWLLKEVAELRRAAQGCEFNKKRLRYLSDTQKVKQSSQGV
LYWMSRDQRIQDNWALIYSQQLALAEKLPLHICFCLVPKFLDATYRQYAFMLKGLQEVVKECKSLDIEFHLLS
GEPVHNLPAFVKSWNIGAVVTDFNPLRISLQWIDTVKKHLPSDIPFIQVDAHNVVPCWEASTKLEYGARTIRG
KITKHLQEFLTDMPLVDTHPYCASRAAKIIDWEEVLSSLEVDHNVCEVEWAQPGTTGGMFMLESFIDQRLHIF
ATHRNNPNSDAVSHLSPWIHAGQLSAQRVVMQVKRKKNASESVASFIEEIVVRRELADNFCFYNQNYDNISGA
YDWAKKTLQEHAKDRRQYLYTKKELESAETHDQLWNAAQRQLLLEGKMHGFLRMYWAKKILEWTASPEEALSI
AIYLNDRLSLDGCDPNGYVGCMWSICGIHDQGWAERPIFGKIRYMNYAGCKRKFDVEQFERKYAAIKKNPNLN
AKISV
>Danio_rer_XP_005155462.1_cry_circad
```

MSHNTIHWFRKGLRLHDNPALIAALKDCRHIYPLFLLDPWFPKNTRIGINRWRFLIEALKDLDSSLKKLNSRL
FVVRGSPTEVLPKLFKQWKITRLTFEVDTEPYSQSRDKEVMKLAKEYGVEVTPKISHTLYNIDRIIDENNGKT
PMTYIRLQSVVKAMGHPKKPIPAPTNEDMRGVSTPLSDDHEEKFGIPTLEDLGLDTSSLGPHLFPGGEQEALR
RLDEHMERTNWVCKFEKPKTSPNSLIPSTTVLSPYVRFGCLSARTFWWRLADVYRGKTHSDPPVSLHGQLLWR
EFFYTTAVGIPNFNKMEGNSACVQVDWDNNPEHLAAWREARTGFPFIDAIMTQLRQEGWIHHLARHAVACFLT
RGDLWISWEEGQKVFEELLLDSDWSLNAGNWQWLSASTFFHQYFRVYSPIAFGKKTDKHGDYIKKYLPVLKKF
STEYIYEPWKAPRSVQERAGCIVGKDYPRPIVDHEVVHKKNILRMKAAYAKRSPEDKTINKGEKRKASPSIKE
MFQKKAKR
>Danio_rer_XP_009291670.1_uncharacte
MSAGEETKPVSEVRELLRELILGREDPQGFFCMCVSLLGDADTRRLFLDLIKPLSSEYEHLHSQLTSVFLEYF
SKDESEELELALTLSLYETKQIDPEDKLHNSDHQHHVPRSYKTYADVRTESSGADGSVVPENQPTAKCTLDTE
VHKTRNAIKHINGDKITKSQKRRLRKKQHLLKNPSGPRPVVLWFRRDLRMWDNPALIGCLELGAPVIPVFLWN
AMEEEGPGVTMSTGGASKYWLHQALVSLKRSLEERGSHLVTLKAEPSSLTALQGLMDETGAASVVATALYEPW
LKERDDALWETLEKRGVTCHIYHSYCLRDPYTVSTRGVGLRGIGSVSHFMSCCQQNPAGGLGSPLDAPTTLPS
PSAWPQGCPLADLGLARMPRRKDGTVIDWAVDIRKTWDFSEEGAHTHLEAFLRDGVYRYEKESCRADAPNTSC
LSPYLHFGQLSARQVLWAARGARCKSPKFQRKLAWRDLAYWQISLFPDLPWESLRPPYKALRWSSDHAHLKAW
QRGRTGYPLVDAAMRQLWQTGWMNNYMRHVVASFLIAYLHFPWQEGYRWFQDTLVDADVAIDAMMWQNGGMCG
LDHWNFVMHPIDAALTCDPCGTFVRQWCPELKALPDDLIHKPWKCPTSMLRRAGVVFGDSYPERIVIDLEERR
AQSLQDVASVRRRFRQFVDQRSGCDLVPVPSRLVQDALGSMEDIVKHEKNGFLLPVITRMEFKHQSEDPDRQD
NPYSAVLKGYVSRKRDETVAFLNERDFTASVMCESAQRRERLERDSCLLEGLPRPTASRGGARRTQTRDPYSK
VPGGVAVPRK
>Daphnia_mag_BAS19218.1_cry
MSSYESAPREKQVVHWFRKGLRLHDNPSLKDGLKGCSTYRCIFILDPWFAGSSNVDINKWRFLLESLEDLDQN
LPKLNSRLFVIRGQPAGVLPKLFKEWETTCLTFEEDPEPFGRVRDQNIMTMCKDFNIEVITRASHTLYHPQKI
IEKNGGKAPLTYRQFQNIIASVDPPPPPEADITFETIGRGYTPIDDSVDDRFSVPTLEELGFDTDGLMPAVWR
GGESEALTRLERHLERKAWVASFGRPKMTPQSLLASQTGLSPYLRFGCLSVRLFHQQLTNLYKKIKKAQPPLS
LHGQVLWREFFYCAATNNPNFDKMVGNPICIQIPWDSNAEALAKWANGQTGFPWIDAIMTQLREEGWIHHLAR
HAVACFLTRGDLWISWEEGMKVFEELLLDADWSVNAGTWMWLSCSSFFHQFFHCYCPVRFGRKVDPNGDFIKK
YQPILKNFPLQYIHEPWNAPESVQRAAKCIIGKDYPLPMVNHLEASQLNIERMKQVYQRLTQYRGTGVMSPTA
KSDNGVIHNVVNKNKRENSHAKQYRTEELRQNSTQRNQSNLN
>Daphnia_mag_XP_032780865.1_cry-2-like
MNKPVVIHWFRKGLRLHDNPALLSALEKVGESHYELRPVFILDPWFVKHARVGPNRWRFLIQSLQDLDNNLKK
IGSRLFVVKGSPLEGFKKLFKEWNVKKLTFEVDIEPYAKIRDEEIKQLAEKHSVTVITKVSHTIYDLEQVFKA
NGNKAPLTYVKFQSVVAKFGTPAKALDAPGNLPKQCQTLLLSEDYNVPLLEEMRVDLTGLGKELFPGGESEAL
IRLNKYMNQQEWVCKFSKPDTSPNSIEPSTTVLSPYLKFGCLSSRLMYHRIHDVIAGRKHTSPPTSLTGQLLW
REFYYTCGAYVPNFDRMEGNPICKQIPWKKDPEDKHYLAWKDGKTGYPFIDAIMTQLRTEGWIHHLARHAVAC
FLTRGDLWVSWELGQQVFEELLLDADWSLNAGNWMWLSASAFFHQYFRVYSPIAFGKKTDKHGDYIKKYLPVL
KKFPSEYIYEPWKAPLSVQQKAGCIIGKDYPKRIVEHEIAVKDNLAKMKEAYQGNKEEGPENEIAKRKKAPSK
DSSPKITKFLKKK
>Daphnia_mag_XP_032781741.1_photolyase
MSKPDSAIGSSKTPAAKKLKLETSDDEPSSSRTPSVDSGFLASVELKRKQCAGNILEFKFNKKRCRLLSKSMD
LGNFGGGVLYWMSREQRVQDNWALLYAQRLALKMKLPLHVCFCLVPTFLGATIRQFGFMLKGLEEVEAECQKL
QIEFHLLKGDSQICVPNLIKKLKLDAVVADFSPLRVPLSWIEKVKESIPEDVPFCQVDAHNIVPVWVASDKQE
IGARTIRKKIHDKLDEFLTDFPAVVTHPHPSQRKAKPVDWKAANAYLEVDRTVEEVDWITPGTKSGLLELSNF
CQKRLRLFGEKRNDPNVAALSNLSPWLHFGQLSAQRCILEVKEYKAKYAKSVDVYIEETLIRRELSDNFCFYN
PNYDNLKGAANWAQETLDVHKKDKRPALLTSQQLEEGKTHDDLWNASQIQLTRTGKMHGFLRMYWAKKILEWT
ETPEEALRLAIYLNDRYSLDGRDPSGYVGCMWSICGIHDMGWKQRDVFGKIRYMNYKGCQRKFDVVAFVQRFG
ARTYPIKGVKYE
>Daphnia_mag_XP_032785219.1_cry_DASH-l
MLKIVNMTNRVAVCLFRNDLRYYDNEVLAHAHKNTDYILPLYCFDPRHFAGTHHYGFPKTGSFRTRFLLESVE
DLRKKLLKHGSNLMIVHGKPEDVVSKIVKNLVGFKLTLLLHAEVTKEETDVEKSLQKVCQENKASFVNIWGST
LYHKGDLPFQISKVPDAYTAFRKDVEDQLKIRPEISMPEKLKPVPSFPSEISWGNLPSYEMLNSSEPLLNPSS
AFPFSGGETAALQRLKSYLWDTNAVAQYKETRNGLIGSDYSTKFSPWLALGCLSARKIHWELEKYEQQRTKNQ
STYWVRFELLWRDYFKFVSMKYGDRIFYPNGMKGKRQEWKKNMELFKAWQAGKTGVPFVDANMRELLETGWMS
NRGRQNVASFLVKDLHLDWRLGAEWFESLLLDHDVCSNYGNWNYVAGIGNDPRENRKFNMIKQSMDYDLEGNF
IRMWVPELREIPGSKIHSPWMLSSGALSAANVRLGQNYPNPVVVAPEWSRHQREGKESGHGGRGHGIPKAGNQ
RGIDFYFKNPGTKK
>Daphnia_mag_XP_032790260.1_cry-1-like
MNILWFRRGLRLHDNPALLSAIENSKEFLALYIFDTTFQDAGYKPYHMYGFLLECLNDLNESFQSVGTKLHIF
QGCPLEVFRHLRQSHCINKLCFIQDCEPIWHERDNAVKNLCLELDIEVYEHVAHTLWDPMDIIASNGGTPPLT

YEMFVHVAMSVGDPPKPAADPEWKGVKFVSIEPFETNRFTLFDGVPTLGAVGVPENPVGCRNRKIIGGETNAL
KHFEIRLRAEETAFRSGFYQPNQARPDLLGPPLSLSAAISVGAMSVRLFYWKIHELFDKVNKGSPPAWLGITG
QIIWRDYFYAMSRMNPKFDKEVDNPICLQIPWNENEELFEKWKNGQTGYPFIDAGMRQLNQEGWMHHSVRNAV
AMFLTRGDLWLNWDIGAEYMAYQLVDSDWSVNSGNWMWVSSSAFERLLDCSVCINSVLYGKRLEPSGDYIRRY
VPELANFEFEYIHEPWKAPIDIQRAANCIIGKDYPTRIVIHEEVLTRNQTWMKDFRRKFKETPAHCQPSSNSE
VYKFFCLPDDSLPF
>Daphnia_pul_EFX71237.1_DNA_photol
MSKPSSSKGASKPPATKKPKLESSDDEDSSASLVTDSSGFLANIHLKRKKCADNILEFKFNKKRCRLLSKSME
LGDCGGGILYWMSREQRVQDNWALLYAQRLALKMKLPLHICFCLVPTFLGATIRQFGFMLKGLEEVETECQKL
KIEFHLLKGESQICVPDLITKLKLDAVVADFSPLRVPLSWIDKVKESIPKNVPFCQIDAHNIVPVWVASDKQE
VGARTIRKKINDKLDEFLTEFPPVIVHPHPAKTKAKSVDWKAADAYLEVDRTVGEVDWITPGTKSGLQELFNF
CQRRLKMYAEKRNDPNLAALSNLSPWLHFGQISAQRCILEVKGYKTKHAKSVDAYMEETIIRRELSDNFCFYN
TNYDNLKGAANWAQETLEVHKKDKRPALLSAQQLEEGKTHDDLWNASQIQLTRTGKMHGFLRMYWAKKILEWT
ETPEEALRIAIYLNDRYSLDGRDPSGYVGCMWSICGIHDMGWKQRDVFGKIRYMNYKGCQRKFDVVAFVHRFG
ARAYPIKGVKYD
>Daphnia_pul_EFX77441.1_CRY-D
MNVLWFRRGLRIHDNPALLSALENSKDFIALFVFDTTFQDPGYKPYHMNGFLLECLHDLNESLESVGTKLHVF
QGCPLEVFRHLHNIKPINKLCFIQDCEPIFHERDIAAKNLCSELDIEVYEHVAHTLWDPMDIIASNGGTPPLT
YEMFVHVAMSVGDPPKPVADPEWKGVKFLTLEPFESNRFTLFGGVPTLSQIGVPENPVGCRGRRIFGGETNAL
KHFAIRLQAEETAFRSGFYQPNQARPDLLGPPLSLSAAISVGAISVRLFYWRIHEIFDKVNRGNPPAWLGITG
QIIWRDYFYAMSRMNPKFDKEVDNPICLQIPWVENEEFFEKWKNGQTGYPFIDAGMRQLNQEGWMHHSVRNAV
AMFLTRGDLWLNWDIGAEYMANQLVDSDWSVNSGNWMWVSSSAFERLLDCSVCINSVLYGKRLEPSGDYIRRY
VPELANFEFEYIHEPWKAPIDIQRTANCIIGQDYPAQMVVHEEVLPRNLEWMKEFRQKFKETPAHCQPSSNSE
VYKFFCLPDDSLPF
>Daphnia_pul_EFX82092.1_CRY-M
MSGYDSEPREKQVVHWFRKGLRLHDNPSLKDGLKGCSTYRCIFILDPWFAGSSNVDINKWRFLLESLEDLDQN
LRKLNSRLFVIRGQPAGVLPKLFKEWETTCLTFEEDPEPFGRVRDQNIITMCKDFNIEVITRASHTLYHPQKI
IEKNGGKAPLTYRQFQNIIASVDAPPPPESDITFESIGRGYTPMDESMDDRFSVPTLEELGFDTDGLMPAVWH
GGETEALTRLERHLERKAWVASFGRPKMTPQSLLASQTGLSPYLRFGCLSVRLFHQQLTNLYKKIKKAQPPLS
LHGQVLWREFFYCAATNNPNFDKMIGNPICVQIPWDSNAEALAKWANGQTGFPWIDAIMTQLREEGWIHHLAR
HAVACFLTRGDLWISWEEGMKVFEELLLDADWSVNAGTWMWLSCSSFFHQFFHCYCPVRFGRKVDPNGDFIKK
YQPVLKNFPLQYIHEPWNAPESVQRAAKCVIGKDYPLPMVNHLEVSQLNIERMKQVYQRLTQYRGTGLMSHSP
QSDHGIIINVGNKNKNENSHAKQFRTDELRQNAVQRNQSNLN
>Daphnia_pul_EFX85418.1_DNA_photol
MNNKPVVIHWFRKGLRLHDNPALLNALEKVGESHYELRPVFILDPWFVKNAKVGPNRWRFLVQSLQDLDDNLK
KIGSRLFILKGSPTETFKKVFKEWNVKKLTFEVDIEPYAKTRDEEIKKLADHHSVTVVAKVSHTIYDLEKVFK
ANGNKAPLTYVKFQSVVAKFGTPEKALNAPGKLPKQCQTLLLSDKYNVPLLEEMQVDLTGLGKELYRGGETEA
LARMEKYMSQQDWVCKFSKPDTSPNSIEPSTTVLSPYLKFGCLSPRLMYHRLHEIIDGRKHTSPPTSLTGQML
WREFYYTCGAYTPNFNRMVGNPVCKQIPWKVDPEDEHFVAWKNGRTGYPFIDAIMIQLRTEGWIHHLARHAVA
CFLTRGDLWVSWELGQQVFEELLLDADWALNAGNWMWLSASAFFHSYFRVYSPVAFGKKTDKHGDYIKKYLPV
LKKFPTEYIYEPWKAPLSVQQTAGCIIGKDYPKRIVDHDVVMKENLAKMKKAYQGKEEAPEGEPVKRKKSASN
ESPPKITKFFKKN
>Daphnia_pul_EFX86680.1_hypothetic
MIRILKMSNRVAICLFRNDLRYHDNEVIALAHKSADFVLPLYCFDPRHFEGTHHYKFPKTGIFRTQFLLESVE
DFRQTLVKRGSNLMIVHSKPEEALLKIFKSLTGLKVTLILQTEVTKEETDVEKRLQKICQEIKASYINCWGST
LYHKGDLPFQINHVPDSYTGFRKDVEEKLRIRPEISMPDKMKPVPTFAHEIPWGNLPTIEALNSTKPIPNSSS
AFPFNGGETAALLRLKSYLWDTNAVAQYKETRNGLIGSDYSTKFSSWLSHGCLSPRRIHWELEKYELQRTKNQ
STYWVRFELLWRDYFKFVSMKYGDRIFYPNGMKGRRQQWKKDMELFKAWQMGKTGVPFVDANMRELLATGWMS
NRGRQNVASFLVKDLLLDWRLGAEWFESLLLDHDVCSNYGNWNYVAGIGNDPRENRKFNMIKQSMDYDLEGNY
IRMWVPELREIPGSKIHSPWMLSSGALSAAKIRLGDNYPNPVVVAPEWSRHQKGGKDFGQGNPKGGTQRGIDF
YFKNPGGQK
>Darwinula_ste_CAD7244622.1_unnamed_pr
MEDFERVNHRQRTSGVIALHWFRHGLRLHDNPALSYAAANAKKLYVMFTFDGESAGTKLIGYNRMKFLLESLH
DLNCQLHSYGGQLLVFREKPLKVITRLHDVLGVNLVTFEQDCEPIWNERDEAVKEGCRELGIECREEVSHTLW
DPKEVVDMNGGVPPLTYTMFLQVTSAIGEPPHPEERLDFSSVKFGIVPVGLAAELNLFAEIPTPEDLNVKCSH
PADIEENSGSWIGGETQALLLFDMRLAVEADAFKRNFYLPNQARPDLLGPSRSLSPYLRYGCLSIRKMYWGIH
DLFAEIHKGKPPCHVHLTAQLIWRDFFYAMSVGNPNYDRVKENPICLQIPWRHKQKELQQWKDGKTGFPFVDA
VMRQLLREGWIHHVARNAVAQFLTRGGLWISWEDGLKVFLKHLLDADWSVCSGNWLWISSSAFETILDCSHCL
SPVAYGRRLEPTGDYIR
>Darwinula_ste_CAD7250301.1_unnamed_pr

MNEGWVMTNRQASDTATSSVRHRSAKHSSRPKHSVHWFRRGLRLHDNPSLRAGLENCITWRCIFILDPWFAGS
SNLGLNKWRFLLQCLEDLDQSLRKLNSRLFVIRGQPADVLPKYLKEWGTTCLTMEEDPEPFGRVRDHNINVLC
REMGIQVKSKVSHTLYKLEKIIEKNGGVAPLTYGQFQAVLSHMDPPPAPVPTITTQTIGNAYTPISDDHDEQF
AVPTLEELGFDTESLKPTLWPGGEMEALARLERHLERKAWVASFGRPKMTPQSLLASQTGLSPYLRFGCLSPR
LFHQQLSELYRKIRRCPPPLSLHGQLLWREFFYTAATNNPSFDKMQGNPICVQIPWDKNPEALAKWANGQTGY
PWIDAIMVQLREEGWIHHLARHAVACFLTRGDLWISWEEGMKVFEELLLDADWAVNAGNWLWLSCSSFFQQFF
HSYCPVRFGRKADPNGDYIRRYLPVLKNFPTAYIHEPWTAPEAVQKAAKCRIGQDYPLPMINHLDASRINMER
MKLVYQKLHNYRGGSVSPSSFHIPMKRVKLELEPWNGQALTT
>Darwinula_steve_CAD7245491.1_unnamed_pr
AGKSVESFPFIKKRVRILSGATEVKENSSGIVYWMSRDQRIQGLEEVSSECSTLGMNFHLLRGEAKSSLVKFV
KDYDIGAVVTDFAPLRVPLGWVEDVKKNLPNDVSLCQVDAHNIVPCWVASEKLEYGARTIRPKIHNKLSEFLT
EFPPVINHPYPPKIKAEAIDWDEVQKFLEVDRSVEEVQWATPGTRAGMQMLHEFIQKRLKLFGEKRNDPTVDA
LSNLSPWFHFGQVSVQRCILEVKKQGKSNSKSVEAFVEEAVVRRELADNFCFNNPNYDSIKGAYDWARKTLND
HKDDKRSYLYTRKELEEAKTHDDLWNAAQIQMVSEGKMHGFLRMYWAKKILEWTASPEEALTIAIYLNDKYEL
DGRDPNGYVGCMWSICGIHDQGWAERPVFGKIRFMNYQGCKRKFDVDSFVRKYKARAFSKK
>Dasypus_nove__XP_004473360.1
MGVNAVHWFRKGLRLHDNPALKECIQGADTIRCVYILDPWFAGSSNVGINRWRFLLQCLEDLDANLRKLNSRL
FVIRGQPADVFPRLFKEWNITKLSIEYDSEPFGKERDAAIKKLATEAGVEVIVRISHTLYDLDKIIELNGGQP
PLTYKRFQTLISKMEPLEIPVETITPEMIEKCTTPLSDDHDEKYGVPSLEELGFDTDGLPSAVWPGGETEALT
RLERHLERKAWVANFERPRMNANSLLASPTGLSPYLRFGCLSCRLFYFKLTDLYKKVKKNSSPPLSLYGQLLW
REFFYTAATNNPRFDKMEGNPICVQIPWDKNPEALAKWAEGRTGFPWIDAIMTQLRQEGWIHHLARHAVACFL
TRGDLWISWEEGMKVFEELLLDADWSINAGSWMWLSCSSFFQQFFHCYCPVGFGRRTDPNGDYIRRYLPVLRG
FPAKYIYDPWNAPEGIQKVAKCLIGVNYPKPMVNHAEASRLNIERMKQIYQQLSRYRGLGLLASVPSNPNGNG
GLMGYAPGENILGCSSSGSCAQGSSILHYAHGDNQQTHLLKQGRSSMGTVLSSGKRPSQEEETQSIGPKVQRQ
STN
>Dasypus_nove__XP_012380336.1
MRRPRMNANSLLASPTGLSPYLRFGCLSCRLFYYRLWDLYRKVKRNSTPPLSLFGQLLWREFFYTAATNNPRF
DRMEGNPICIQIPWDRNPEALAKWAEGKTGFPWIDAIMTQLRQEGWIHHLARHAVACFLTRGDLWVSWESGVR
VFDELLLDADFSVNAGSWMWLSCSAFFQQFFHCYCPVGFGRRTDPSGDYIRRYLPKLKGFPSRYIYEPWNAPE
SIQKAAKCIIGLWGNGKGWNGPTGMVQTSPLIVPVGWHSDYSPASLPGLLASVPSCVEDLSNPVAEHSSSQPG
SMSSTGPRPLPSGPASPKRKLEAAEEPPGEELSKRARVAELPSKDA
>Dendrobium_cat_blue-lighta_XP_028552950.1
METDPSPLQPQTPDSSEEQQKEITSSPSKSSPPIASLSLSLSTNLLLSKPTPPVATAQPFAALPSRLKIPTQI
FSLTRLSFSASSVSTPARPSNLSSSTPALLSSPSPFLRRRPADPSGGAAARRCTLLWFRCDLRTHDNEALTAA
SNESLSILPVYLFDPRDYGKSSSGFDKTGPYRATFLLQSVADLRRSLRARGSDLVVRIGRPESVLVELARLVG
ADAVYMHREVSHEEARTEERVEKAMESEGVEVKYFWGSTLYHIDDLPFELEQMPSNYGGFREKVQGLNVRKTI
EALDQIKGLPSRGDVEPGEIPTLMDLGLNPAPAVTQDGKPAVSASLAGGETEALERLRKFAVECCAQPNKGFR
NGAQDSIYGANFSCKISPWLAMGCLSPRFMYEELKRTANRAISSKTQKNVDASGGSGMNWLMFELLWRDFFRF
ITKKYSTKRKVGAEPVTACTVQSSFSTR
>Dendrobium_cat_XP_020678783.1_cry-1
MMDSQSRTIVWFRRDLRIEDNPALLSAARDGVTLPVFIWCPSEEGKFYPGRCSRWWLKQSLAHLDQSLKSIGA
PLVFIRAESTVSALLHCVNAIGATRVVYNHLYDPVSLVRDHAVKSQLVDHGIVVQSYNGDLLFEPWEVYDASG
HAFTTFNEFWNRCLSLPTEPAAFLPPWQLVPPSGIESLDSCSIEELGLEKESEKPSNALLGRGWSPGWSNADK
VLSVFVGENLLNYSKNRMKLEGTTTSLLSPYLHFGEISVRKIFQNVRMRQIQWTKENSDAVESVDFFLRSIGL
REYSRYLCFNFPFTHEMSLLGNLKHYPWRADQEQFKSWRQGRTGYPLVDAGMRELWATGWMHNRTRVIVASFF
VKFLQLPWTWGMKYFWDTLLDADLESDILGWQYISGSLPDGHELIRLDSPEVQGRKFDPDGAYIRSWIPELAR
MPTEWIHHPWDAPTSILKAAGVDLGSNYPKPIVDINIAREHLDDALSTMWELDRAAKAERFNGAGEVVADNVK
MQTFDIPTVVVRREASDITSSLDQRVPSFHNMDTNFNKKRSINLDYVMKKEVEFRNNEKGTVGEEDDLRSTAE
SSSAKRRSISEAQFSVPLPCYSESESKNQCGYDAFKPQFFRGSPSTQPVQKDNAMEAQYEEADVESCKEGLRS
CKRPA
>Dendrobium_cat_XP_020678980.2_photolyase
MAAAGAVHPGRVRLLNPIKTPPPPSPGSPAGPVVYWMFRDQRSRDNWALIHAIHLANHSSAPIAVAFNLFHRF
LNAHARQLGFMLRSLILLRLRLHELGIPFFLFRGDAVDTIPKFLCECGASALVTDFSPLRSVREWKAEICERV
GERVRVYEVDAHNVVPVWVASNKLEYSAKTIRAKIQRVLPEYLVEFPEISKPDLEWGLEDPKEIDWEELVAEV
LREGGEVPEIEWCEPGEDAAMEALMGAKEGFLTRRLKNYSTDRNNPLKPRALSGLSPYLHFGQISAQRCALEA
RKLKNSHPQAVETFLEELIVRRELADNFCYYQPHYDSFLGAWDWARRTLMDHAADKREHLYTRDELEKAKTAD
LLWNASQLEMVHHGKMHGFMRMYWAKKILEWTSGPEEALAIALYLNDKYEIDGRDPSGYVGCMWSICGVHDQG
WKERAVFGKIRYMNYAGCKRKFDVDGYIAHVKSLVVRSNKRKAENAKSPMPKISRT
>Dendrobium_cat_XP_020685617.1_(6-4)DNA_p

MESSSSSSNCMIWFRKGLRIHDNPALELAQKGSKHLFPVFVLDPYYIDPDSLASSPGSSRAGINRIQFLLESLV
DLDCGLRRLESRLLVLKGEPVQVIARLLKDWNIGKLFFEFDTEPYAQTRDNKVKDIASASGIEVFSPVSHTLF
DPAEVIRKNGGKAPLTYKSFVAIAGKPSAPLRSMYSKLPPIGDIAGYEILGVPSIHDLGYKDVKQEFSPFRGG
ETEALKRLKENLVNKKWVAEFEKPKGDPSEFIKPATTVLSPYLKFGCLSSRYFFQCVDDAYKTVKKHTQPPVS
LAGQLLWRDFFYTVAFGTPNFDRMQGNKICKQIPWRDDEKLFVAWRDAQTGYPWIDAIMIQLKKWGWMHHLAR
HSVACFLTRGDLFIHWEKGRDVFERLLIDSDWAINNGNWLWLSCSSFFYQYHRIYSPISFGKKYDPSGNFIRH
FLPVLKDMPKEYIYEPWTAPLSIQRQANCIIGRDYPKPVVPHDLASKECKRQIGAAYALNLSGETADAEEKLN
SLRRKLEEDDYDGIQNMKQKKRMSK
>Dendrobium_cat_XP_020695999.1_cry-1_isof
MPPASSTNGGNTSNNNISIVWFRRDLRVEDNPALAAGVRAGYVVSVFIWAPEEEGHYFPGRVSRWWLSQSLKH
LDSSLRSLGSNLITKRSFDTASTLVDVVNSTGATNLFFNHLYDPLSLVRDHRIKELLSSRGLTVRSFNADLLY
EPWEVADENGCPFSTFEPFWNKCLNMPYDPAAPLLPPKRIISGDVSRCPSDTLVFEDESERGSNALLARAWSP
GWRNANKALNAFINGPLIDYSVNRRKADGATTSLLSPHLHFGELSVRKVFHLVQIKLLVWINEENESGKESVN
LFLKSIGLREYSRYLSFNHPFSHERPLLSHLKFFPWVVDENYFKAWRQGRTGYPLVDAGMRELWATGWLHDRI
RVVVSSFFVKVLQLPWRWGMKYFWDTLLDADLECDALGWQYISGTLPDGREFDRIDNPQFEGYKFDPNGEYVR
RWLPELIRLPTEWIHHPWDAPEPVLQAAGIELGSNYPLPIVEISAAKERIQEALIEMWQIETASRTAMENETE
EGLGDSSDLPPFYYPQEIDMEADPEPTRANNLQTTTRRYIDQMVPSITSSFVRNDEDAISVDISSTREDGRAE
VPTNVNFDDQTHGATDQDGAAVVRNNAVLEFNPARLQNRLEFSEESSSSYTERDGGLVPVWSPSNLSEHFISE
ETSVRTSAYLQRHPQSHQMMNWRQISPPLRSWEVESTLHPNAIG
>Dendrobium_cat_XP_028549270.1
MRSLTSLFPQHSVVASTLCSPKTAGIMCSASSSYSATSSAVRLAAVHPVPSLTADEISAAASEAFARHSAAGL
KRSGNGVAIVWFRNDLRVVDNEALLKAWVGSSALLPVYFVDPRHFGSTHLFGFPKTGALRAKFLLECLHDLKK
NLKERGLDLLVKHGSPEDILPSIAKAFGAHTVFAHKETCSEELHVEKLVLKGLQQVILPQHERSNPKTVNPRL
QLIWGSTMYHIEDLPFDAENLPDVYTQFRKSVESKSSIRECNKLPVSLSPLPSSSLGEVGGWGSVPSLGDLGL
AEPKLDKGMHFIGGENAALGRVYEYFWKKALILAMNPL
>Dendronephthya_gig_XP_028409958.1_photolyase
MSRISRKRKPDGVEHEATSEKKKAKSSAESFNMKRVRILTGQKDVSSGYQGIVYWMWRDQRVQDNWAMIYAQK
IAMEHNMPLHVCFALPEKFREASIRQYGFMLKGLQEVEKELKKLNISFHMLLGDPSTTVSKFISSYNIAGVVT
DFSPLKETRSWLDQVVKTVPSDVAVFEVDAHNIVPCWEASPKLEYAARTIRPKITNQLPSFLTEFPPCVKHPI
DSKAHKDTNWKNVYKFIEVDQSVPEVSWAIPGTEAGMKRLMTFSEGNLKHFSTERNDPTKNSLSNLSPWFHTG
QISVQRAILAVSKQKSKWKESVEAFIEESVIRRELADNFCFYNENYDSVDGASEWAKKTLRDHVKDKREYIYT
KEQFETAKTHDRLWNAAQLQVVREGKMHGFLRMYWAKKILEWTKTPEDALEIALYLNDKYQLDGNDPNGFVGC
MWSICGIHDQGWSERSIFGKIRYMNYEGCKRKFDVAAFEDEYK
>Denticeps_clup__XP_028813092.1
MGGNAVHWFRKGLRLHDNPALRDALRGADTLRCVYILDPWFAGSSSVGVSRWRFLLQCLEDLDASLRKINSRL
FVIRGQPTDVFPRLFKEWKVTRLSYEYDSEPFGKERDAAIRKLANEAGVEVMVRISHTLYDLDKIIELNGGQS
PLTYKRFQSLISRMDAVEMPAETITADVMGRCTTPISDDHDDKFGVPSLEELGFEVDTLTTAVWPGGETEALT
RLERHLERKAWVANFERPRMNANSLLASPTGLSPYLRFGCLSCRLFYFKLTDLYRKVKKNCVPPLSLYGQLLW
REFFYTAATNNPRFDKMEGNPICVQMPWDRNPEALAKWAEGRTGFPWIDAIMTQLRQEGWIHHLARHAVACFL
TRGDLWISWEEGMKVFEELLLDADWSVNAGSWMWLSCSSFFQQFFHCYCPVGFGRRTDPNGDYIRRYLPILRG
FPSKYIYDPWNAPESVQKVAKCIIGVHYPKPMVNHAEASRINIERMKQIYQQLSCYRGLGLLATVPSNSSNNG
ESSMSGAAAGPSSVDSAQEGSAAAAVQQVTSAPQSEAAAQSSWHGDLRYPGTSTRQQHGNLVGGTDESQRQDS
KMAHAPASQRGKGTIAVKRHNEDLVARSSKVQRQSSH
>Denticeps_clup__XP_028822473.1
MSNARTVICLLRNDLRLHDNEVLHWAHKNAEHIVPLYCFDPLHYLGTHCFNFPKTGPFRLRFLLDSVKDLRTS
LEKKGSSLVVRRGEPHKVIGDLIRQLGSVSAVAFHEEATQEELKVERQIKDVCSQNKVRVQSFWGSTLYHRDD
LPFNHISRLPDVYTQFRKAVESQGRVRPVTPTPEQLRPLPTGLEEGPIPTFQDLEQTEALTDGRSAFPCCGGE
TEALARIQHYFWDTDAVASYKETRNGLIGVDYSTKFAPWLAMGCLSPRHIYHQIKKYEAQRTANQSTYWVIFE
LLWRDYFRFVAVKYGNQIFHLNGLQDKKVPWKKDMKLFNAWKEGRTGVPFVDANMRELALTGFMSNRGRQNVA
SFLTKDMGLDWRMGAEWFEYLLVDYDVCSNYGNWLYSAGIGNDPREDRKFNMVKQALDYDNNGDYVRLWVPEL
QGIKGGDVHTPWTLSSAALSHAGVCLNENYPSPVTVAPEWSRYVNKRPAGGAAPSARGKKGPSHTPRSHKDRG
IDFYFSRSKKL
>Denticeps_clup__XP_028833071.1
MYARLARLIQSARSGKVRRLHDRRLLHFPSLIQTLAFGLRDSGKTAFDRNHSIKRLACTMPPSSSERKRKAKG
SAAEASTGKKPKPSSAHQTEEAGGWLEARVATLRRQARGFKFNHKRVSILSDVQKIKQGSQGVVYWMSRDQRV
QDNWALIYAQQLAIEDKLPLHICFCLVPHFLDATFRHYAFMLKGLQEVAKECSALEIQFHLLHGSPGDTLPGF
VKDWNLGAVVTDFCPLRETLQWLDDVKKGLSSDVPFFQVDAHNIVPCWVASNKQEYGARTIRGKITKLLPEFL
TELPAVDQHPHSSSKTPKPIDWENTLSSLEVDRSVGEVDWAQPGTTGGFSMLESFIDQRLQYFATHRNNPNVA
ALSQLSPWIHFGQISAQRVVSLVQTHGKNREAVASFTEELVVRRELADNFCFYNKKYDSVQGAYEWAQKTLQD

HAGDCRKPLYTCEQLEQAQTYDKLWNAAQIQVVTEGKMHGFMRMYWAKKILEWTASPEQALSIAIYLNDRYSL
DGCDPNCYVGCMWSICGIHDQGWRERAVFGKVRYMNYEGCKRKFDVGQFERKYCSKKP
>Denticeps_clup__XP_028838111.1
MSHSCIHWFRKGLRLHDNPALLAALRDCRRLYPIFILDPWFAAAANVGINRWRFLVGALKDLDRSLRELDSRL
FVVRGKPSDVFPELFREWSVTRLTFETDTEPYSQQRDKEVLRLAEEHGVEVIQKISHTLYNIERIIEENNGKA
PLTYVRFQTVVKTIGPPQRPLPSPTLQEMNGKCTETHNEKYGIPTLEELGKDPQLAGPEIYPGGEQEALRRLD
KYMERTGWVCSFEKPNTSPNALSPSTTVLSPYLKFGCLSVRTFWWRLVGIYQGKKHSQPPVSLHGQLLWREFF
YTAGRGISNFDRMEGNPVCVRVDWDSNLEYLSAWREARTGFPFIDAIMTQLRQEGWIHHLARHAVACFLTRGD
LWISWEEGQKVFEELLLDADWALNAGNWQWLSASAFFHQYFRVYSPVAFGKKTDKNGDYIKKYLPILKKFPAE
YIYEPWKAPRSVQERAGCIVGKDYPRPIVEHEAVSKKNILRMKAAYAMHSPDTGGPSSKASAGHRLQFPLYEK
RI
>Denticeps_clup__XP_028841978.1
MVVNSVHWFRKGLRLHDNPALQEALNGADTVRCVYILDPWFAGSANVGINRWRFLLESLTDLDTSLRQLNSRL
FVVRGQPAEVFPRLFKEWNITRLTFEYDSEPYGKERDGAIIKMAQEFGVETVVRNSHTLYNLDRIIEMNNNSP
PLTFKRFQAILNRLELPRKPLPAITREQMDSCRTKIADNHDEHYSVPSLEELGFKNVDQTPAVWKGGETEAMD
RLNKHLDRKAWVANFERPRISVSSLMASPTGLSPYLRFGCLSCRVFYYSLRELYMKLRKRCSPPLSLFGQLLW
REFFYTAATNNPNFDRMESNPICVQIPWDHNPEALAKWAEGRTGFPWIDAIMTQLRQEGWIHHLARHAVACFL
TRGDLWISWESGMKVFEELLLDADWSVNAGSWMWLSCSAFFQQFFHCYCPVGFGRRTDPSGDYIRRYIPKLKD
YPNRYIYEPWNAPESVQKAANCVVGVDYPKPMINHAESSRLNIERMKQVYQQLSHYRGLSLLASVPTIQEEAE
PPMSDDSQASGSASDSGKVGWDPSEEQERQT
>Denticeps_clup__XP_028853227.1
MTHRTIHLFNKGLRLHDNPSLLAAMESSAAVYPVYVLDRQLMAASAHIGPLRWRFILQSLEDLHSSLQERGSR
LHVLQGPYLDVVRHLVARWGITQISFDSEVEPHCVQLEAGLRGLAGELGLSIISCVAHTLYDVKRIIKTNDGK
PPLTYKKFLHVLSFLGEPDKPARDITTADFQKCLTPRQDGDDEKDRVPTFEELGLEVEPEVLWPGGESHALKR
LEQHFQSQGWVANFSKPRTIPNSLLPSTTGLSPYLSLGCLSVRTFYHRLSSIYAQSKNHSLPPVSLQGQVLWR
EFFYTVASATPNFTKMEGNPICLQIGWYDNQEALQKWKRGQTGFPWIDAIMTQLRQEGWIHHLARHAVACFLT
RGDLWISWEEGMKVRRRVEWSVGGIFTALCVQVFEEYLLDADYSINAGNWMWLSASAFFHHYTRIFCPVRFGR
RTDPEGQYLRKYLPVLKNFPSKYIYEPWNAPEEVQLQAGCVIGKDYPLPMVSHQEVSERNLELMKQVRSEQGN
TAELTRDVADDPMEIGAKRHAQEEGPGADESAGRSCSSAASSRKRCSSGGEHGGRACSWTPEAVRLPEISSEV
M
>Dermacentor_silv__XP_037562602.1
MAAMSHAPAPAPAPHHQQQVTSAGGGGNSVGERHVVHWFRKGLRLHDQPALRVGLQGARTCRCVYILDPWFAGSS
NAGVNKWRFLLQSLEDLDARLRKLNSRLFVIRGQPADVFPKLFKEWRVTHLTFEQDPEPYGAIRDQKITALAH
EMGVSVVCEPSHTLYPLERILERHGGKTPLTYRQFQSVVVDMEPPAQPLPAPESLPRTPIDEDHDERFAVPTL
AELGFDMDNLKAAVWPGGETEALARLERHMERKQAWVASFGSPRMTPKALLASQTGLSPYLRFGCLSARLFYH
QLADLYRKIRNSNPPLSLQGQLLWREFFYCAATRNPNFDRMHNNPMCVQIPWDVNAEALAKWANGQTGYPWID
AIMRQLREEGWIHHVARHAVACFLTRGDLWLSWEEGMKVFDELLLDADWSVNAGSWMWLSCSSFFQQFFHVYC
PVRFGRKADPSGDYIRKYLPVLKHFPNNYIHEPWTAPESVQVAARCVVGRDYPLPMVNHQVASHVNLQRIRQV
YQQLTQCVKAPGLFPSLPSASPTECNKQNHFIDDRKLRTKNSRQEDEEESMEGVV
>Dermatophagoides_pte_XP_027196527.1_uncharacte
MVYLVLLQKDLRQFDNPALYHAVRRQKQVIVVYVMEKRLGLTSKWWLCRSLQTFFEELDPFWIPVLYLDNSDK
ISELFEYLSKYIEIERIHSNGLDFELPDDYDEIHDSFQPNLIFKPEDMIKEYKIFTPFWKSIHANCSPVKPLP
IPEYSRKNYVDLEFDEYIDLPKMDDSDRWNQMDQYWKIGEKAAQERFESFVKTKLSDYDHGRDFFCEDGTSKL
SPHLRFGEISVRQIYERLNTKSTERFCSELGWREFAYHVNRYHPDMTKIPLNPKYRDFWPEFSDDHLEAWKTG
NTGFPVIDAAMRDISTTGWMHNRLRMLVASFLVKNLLIPWQIGEEWFYEHLVDADPAINPFSWQWVAGCGTDS
VPYFRIFNPMLQGEKFDSKGIYVRKWLPEFADFPLKQNIHQYIIKQPQLCPKNYPKPIIDFKSSRERTLSTFK
NIVAPKVIQLKKPRLV
>Desmodus_rotu__XP_024414631.1
MKMAKEAGVEVVTENSHTLYDLDKIIELNGQKPPLTYKRFQAIISRMELPRKPVGSVTSQQMERCPAEIQENH
DETYGVPSLEELGFPTEGLGPAVWQGGETEALARLDKHLERKAWVANYERPRMNANSLLASPTGLSPYLRFGC
LSCRLFYYRLWDLYKKVKRNSTPPLSLFGQLLWREFFYTAATNNPRFDRMEGNPICIQIPWDRNPEALAKWAE
GKTGFPWIDAIMTQLRQEGWIHHLARHAVACFLTRGDLWVSWESGVRVFDELLLDADFSVNAGSWMWLSCSAF
FQQFFHCYCPVGFGRRTDPSGDYIRRYLPKLKGFPSRYIYEPWNAPESIQKAAKCIIGVDYPRPIVNHAETSR
LNIERMKQIYQQLSRYRGLCLLASVPSCMEDLSNPVAEPSSSQTGSTSSAGPRPLPTGPASPKRKLEAAEEPP
GEELSKRARVAELPAPELQSRDV
>Desmodus_rotu__XP_024435149.1
MGVNAVHWFRKGLRLHDNPALKECIQGADTIRCVYILDPWFAGSSNVGINRWRFLLQCLEDLDANLRKLNSRL
FVIRGQPADVFPRLFKEWNITKLSIEYDSEPFGKERDAAIKKLATEAGVEVIVRISHTLYDLDKIIELNGGQP
PLTYKRFQTLISKMEPLEIPVETITSEVIEKCTTPLSDDHDEKYGVPSLEELGFDTDGLPSAVWPGGETEALT
RLERHLERKAWVANFERPRMNANSLLASPTGLSPYLRFGCLSCRLFYFKLTDLYKKVKKNSSPPLSLYGQLLW

REFFYTAATNNPRFDKMEGNPICVQIPWDKNPEALAKWAEGRTGFPWIDAIMTQLRQEGWIHHLARHAVACFL
TRGDLWISWEEGMKVFEELLLDADWSINAGSWMWLSCSSFFQQFFHCYCPVGFGRRTDPNGDYIRRYLPILRG
FPAKYIYDPWNAPEGIQKVAKCLIGVNYPKPMVNHAEASRLNIERMKQIYQQLSRYRGLGLLASVPSNPNGNG
ALMGYSPGEHIPGCSSSASCTQGSGILHYAHGDSQQTHLLKQGRSSMGTGLSSGKRHSQEEDTQSIGPKVQRQ
STN
>Dianemobius_nig_BAF45421.1_cry_precur
MDNRSNVHKKVAVHRFRHGLRLHDNPALLDAVKDCDAFLPIFIFDGESAGTKLVGYNRMKFLLESLQDIDSQL
KKYGGNLYLFHGTPLCVFQYISQTIGLHKLCFEQDCEPIWQHRDDLVKKFCKENGIKCIERVSHTLWNPHDVI
KTNGGIPPLTFEMFVHTVSVIGPPPRPVEDVEWSVVNFGVLPMSSIPSDIKVFKNFPTPEDFGISSEVGNMNR
IIQWIGGESQALRHLQERLKVEENAFREGYCLPNQARPDLLGPPTSQSAALRFGCLSVRKFYWSIQDMYSSIC
GPSPNQNITSQLIWREYFYTMSVGNEYYAEMDRNPICLNIPWKNDYGSDFNKWKEGKTGYPFIDAIMRQLIQE
GWIHHVARNAVACFLTRGDLWISWEEGLNFFLQYLLDADWSVCAGNWMWVSSSAFEQLLDCSHCMCPVNYGRR
LDPWGQYIKRYIPELKNYPVEYLYEPWKAPLHVQETAGCIVGKDYPERIIDHQIASEKNRSYMDEIRNRLMNP
PPHCRPSSEKETRQFMWFPDDCSEHSSQ
>Dianemobius_nig_BCX29417.1_cry_2,_par
FGRVRDQNIMAMCREMGISVISRVSHTLYRLESIIEKNGGKAPLTYHQFQTVVASMDSPPPAEPRISARTTDG
VHTPVTDDHDDRFGVPTLEELGFDTEGLLPPVWTGGESEALARLERHLERKAWVASFGRPKMTPQSLLASQTG
LSPYLRFGCLSTRLFYYQLTDLYKKIKKACPPLSLHGQLLWREFFYCAATNNSNFDRMNGNPICVQIPWDKNP
EALAKWATGQTGFPWIDAIMTQLREEGWIHHLARHAVACFLTRGDLWISWEEGMKVFEELLLDADWSVNAG
>Dicentrarchus_labr__AFP33464.1
MVVNTIHWFRKGLRLHDNPSLRDSIRGADSLRCVYILDPWFAGSSNVGINRWRFLLHCLEDLDNSLRKLNSRL
YVIRGQPTDVFPRLFKEWQINRLSYEYDSEPFGKERDAAIQKLACEAEVEVMVQVSHTLYNLVKIIELNDGHP
PLTYKRFQALINRMDAVELPAETITLEVIRNCATPISEDHDDKFGVPSLEELGFETEGLTTAIWPGGETEALM
RLERHLERKAWVANFERPRMNANSLLASPTGLSPYLRFGCLSCRLFYFRLTDLYRKVKKNNSPPLSLYGQLLW
REFFYTTATNNSCFDKMEGNPVCVQIPWDRNPEALAKWAEGQTGFPWIDAIMTQLRQEGWIHHLARHAVACFL
TRGDLWISWEEGMKVFEELLIDVDWSVNAGSWMWLSCSSFFQQFFHCYCPVGFGRRTDPNGDYIRRYLPILRG
FPAKYIYDPWNAPEEVQKAAKCIIGVHYPKPMVNHAEASRVNIERMKQIYQQLSSYRGLGLLATVPVHINNGG
NGKRVDAGTRQGSAGSCETPSIQEGMSSAERGQFTLKRRREEVPLESSSKSWRHSK
>Dicentrarchus_labr__CBN81995.1
MSTFRTIICLLRNDLRFQDNELFHWAQRNAEYIVPLYCFDPRHYVGTYNYNLPKTGPFRLRFLLDSIRDLRNT
LLSKGSNLVVRQGKPEEVVADLIKQLGSVSAVAFHEEVTSEELNVEKGVKDVCAQMKVKVHTCWGSTLYHRDD
LPFHHISRLPDVYTQFRKAVETQSRVRPVFPTPEQLKPLPSGLEEGAIPTAEDLQQTEPLTDPRSAFPCSGGE
SQVLARLKHYFWDTDAVATYKETRNGLIGVDYSTKFAPWLAMGCISPRYIYHQIKQYEKERTANQSTYWVIFE
LLWRDYFKFVGVKYGNRLFQVKGLQDKSIPWKKDMKLFNAWKEGQTGVPFVDANMRELAMTGFMSNRGRQNVA
SFLTKDLGLDWRMGAEWFEYLLIDHDVCSNYGNWLYSAGIGNDPRENRKFNMIKQGLDYDSNGDYVRQWVPEL
QGIKGADVHTPWTLSTAALSHAHVSLGETYPNPIVIAPEWSRHVNKKPSGTGPSQRGKRGPPHTPKQHRDRGI
DFYFSRSKNL
>Diceros_bic_KAF5926500.1_HPG69_0011
MRQTMRLNLAGVTSLLDLLLGTVPFLHNKRCTVGADPFLLQCLEDLDANLRKLNSRLFVIRGQPADVFPRLFK
YVIVLIILNNATVCKIKNNYLIYTPKFYSSKEWNITKLSIEYDSEPFGKERDAAIKKLATEAGVEVIVRISHT
LYDLDKIIELNGGQPPLTYKRFQTLISKMEPLEIPVETITSEVMEKCTTPLSDDHDEKYGVPSLEELGFDTDG
LPSAVWPGGETEALTRLERHLERKAWVANFERPRMNANSLLASPTGLSPYLRFGCLSCRLFYFKLTDLYKKVK
KNSSPPLSLYGQLLWREFFYTAATNNPRFDKMEGNPICVQIPWDKNPEALAKWAEGRTGFPWIDAIMTQLRQE
GWIHHLARHAVACFLTRGDLWISWEEGMKVFEELLLDADWSINAGSWMWLSCSSFFQQFFHCYCPVGFGRRTD
PNGDYIRRYLPVLRGFPAKYIYDPWNAPEGIQKVAKCLIGVNYPKPMVNHAEASRLNIERMKQIYQQLSRYRG
LGLLASVPSNPNGNGGLMGYSPGENIPGCSSSGSCSQGSGILHYAHGDSQQTHLLKQGRSSMGTGLSSGKRPS
QEEDTQSIGPKVQRQSTN
>Diceros_bicornis_minor_KAF5920599.1_hypothetic
MAAAAVVTGAGGAPAAAAAAAGADGASSVHWFRKGLRLHDNPALLAAVRGARCVRCVYILDPWFAASSSVGIN
RWRFLLQSLEDLDTSLRKLNSRLFVVRGQPADVFPRLFKEWGVTRLTFEYDSEPFGKERDAAIMKMAKEAGVE
VVTENSHTLYDLDRIIELNGQKPPLTYKRFQALISRMELPKKPVGSVTSQQMESCRAEIQENHDETYGVPSLE
ELGFPTEGLGPAVWQGGETEALARLDKHLERKAWVANYERPRMNANSLLASPTGLSPYLRFGCLSCRLFYYRL
WDLYKKVKRNSTPPLSLFGQLLWREFFYTAATNNPRFDRMEGNPICIQIPWDRNPEALAKWAEGKTGFPWIDA
IMTQLRQEGWIHHLARHAVACFLTRGDLWVSWESGVRVFDELLLDADFSVNAGSWMWLSCSAFFQQFFHCYCP
VGFGRRTDPSGDYVRRYLPKLKGFPSRYIYEPWNAPESVQKAAKCIIGVDYPRPIVNHAETSRLNIERMKQIY
QQFSRYRGLCLLASVPSCVEDLSNPVAEPSSSQAGSMSSAGPRPPPSGPASPKRKLEAAEEPPGEELGKRARV
AEPPAPELPGRDV
>Didymodactylos_carn__CAF0784413.1
VNQIPETSTGIVYWMSRDQRVQDNWAMLYAQRLALKQQFALHVCFCLVPTFLQATIRAYRFMLKGLQECEQEC
KILNIQFHLLMGQAENVLPQFVEKNNIGTVVCDFAPLRVPRSWVENVAKKITTIPVVQVDAHNVVPCWKASPK

LEYAARTIRNKLHQQMDEYFTEFPPLIKHPHSTKHPAEIIDWQKADDCLQVDRVVDEVDWAKPGYQGGIEQLE
SFINTRILHFALGRNDPNKGAISMLSPWLHYGQISPQRALLIISKLRSKYKDSCDSFIEETFVRRELSDNYCY
YQENYDNLNGAWDWAKKTLNDHRNDKRTHLFTRDQFEYAQTYDDLWNASQVQMVKEGKLHGFLRMYWCKKILE
WTKTPEEAIEIAIYLNDKYNLDGRDPNGYVGIMWSLCGIHDQGWRERPIFGKIRYMNYEGCKRKFDIKTFCEK
YKSL
>Didymodactylos_carn__CAF1148914.1
LFFIYSQALSWANNNCDNVLNVYCFDPREITAGTYKYDFPKCGIHRLKFLIETVICLKTLLEEKGSNLFVQRE
IPEIVILNLVQKFKQHYSITIIFHQEVTREETDVEAAIRKVAIEHKVNVKEFWGLTLFHPNDLPYTSPKFFPD
VYTTFRTMLETKNIRVRKLIDCSQQLKPLPDNIEILQVPNLSDFGHSVAEIHAKTVFPFSGGETSALNRLQSY
VWTKDLVQSYKKTRNSLVNVDHTTKFSAWLSNGSLSPRKIYFEIKRYEKECGINDAGYWIIFEIIWRDFFRFI
ATKYGNRIFYPSGLSNKKYVWKQDVNLFEKWRTGHTGCPFVDANMREMLLTGWQSNRGRQNVASYLTKDLQLD
WQYGAEWFESILIDYDVCSNYGNWIYQAGVGNDPREYRHFNMIKQAFDYDPEGEFVRTWIPELAKLPNDLIHT
PWLASSIELKQANVELGVTYPRPIMIVPQWNNRVRSNVNYICFIILG
>Dimargaris_cri_RKP40130.1_DNA_photol
MWFRTDFRVSDNTALHYAIANSRHAKGQVIALYVVSPEEWLLHGVGPIKLDFWFRNLECLHEELALRDIPLVI
LTARRAADVPHIVTSFCRRMHALHLFFNIEYEVDELARDQRAIELLNSTGGRPAEALHANGSPPASTTPTGSP
PIHVHQFHDQCIVEPGTVLTNEDRPYSVYSPFRRKWYQVFAQNPDFQRLLPAPGIPSPMDRTLNFQKRHPALF
LTAKQFSSYWAELRGLLPVDLQLDPEASRQAGQRYGVGEKHAQTLLADFTRLVLSQYKERRDFFGDDAGTSRL
STYLASGIISPRQCLHAAVDHNDQRLECGDPGAVHWIQELIWREFYRHVLVAFPRVCKNRAFNPRGDGIQWND
NPEGYKRWCEGMTGYPIVDAGMRYLNAIGWLHNRLRMVVAMFLTKDLLIDWRLGEKHFNRYLIDSDFASNNGG
WQWSGSTGTDAQPYFRVFNPLLQSEKFDPSGRFIAQWVPELKPLYDKGIKRTADSKSKLPIHAPYERLSTTEF
KSLGYPKPIVEHHKAKDIAIKVFEAALKKKP
>Dinothrombium_tin_RWS08790.1_photolyase
MSQSNAKRRKVEHRQSAVKVKQEVCDENDSDGDNDPCSAIRKSRSLIARSINEFNFNKKRVRVLSDESEVCED
CKSIIYWITRDARVEDNWSMLYAQRLSLKFNVPLYVCFCLVPKFLNATKRQYNFLLKGLQEVERDCHNLNIPF
RLLIGEASQVLPLFAKQHDAGAIVTDFSPLRIKRDWIENLKVKLHKNIPICQVDAHNIVPCWVASPKLEYGAR
TIRKKIHDKLAEFLTHFPPVIRSPFECEIKSNNWDAALKSLQIDDETVDDVKWAVPGTEAGIRMLISFCNERL
KDYASKRNNPNYNCLSNLSPWFHFGHLSVQRAILEVKKYSNKYKESVDAFIEEAIIRRELAENFCYYNDKYDS
LEGAWDWAKITLKAHEKDKRPYIFSKEQLEKAQTLDPLWNAAQRQMLREGKMHGFLRMYWAKKILEWTKSPEE
ALEIAIFLNDKYNLDGRDPNGYVGCMWSICGVHDQGWAERPIFGKIRYMNYEGCKRKFDVSEFENKYKP
>Dinothrombium_tin_RWS10201.1_cry-2-like
MNGIYWFRKALRLHDNPALSEAVEKCETLFPLFILDPSIRSFVKVGANRWRFLVQSLQDLDRSLREANLRLIV
IRGTPKDLMPKLIDKWKINLIAYEMDTEPFAIKRDQEIDQIANKLKVELIKRVSHTLYDPNDILRNSASKSTP
FTYQGFLTTLSKMGSLSPPLPVPDFSHLHPIEKYLKNEPCELPTLEELGIKESECGPCLFPGGETEAIKRMEQ
TLSDHKWVVSFAKPNTSPNSLKPSTTVLSPYLKFGCLSCRRLYFKLKEIESKSSKCTQPPVSLVGQLLWREFF
YANAALTPNFDKMVGNPICKQIEWDKNDEHLKAWKEGRTGYPFIDAIMIQLKTEGWIHHLARHAVACFLTRGD
LWISWEDGLKVFEEYLLDADWSLNAGNWLWLSASAFFHQYFRVYSPISFGKKTDPNGDFIRKYIPVLRRFPKE
HIYEPWKAPKFVQQNCKCLIGVDYPKPIVDHETVRKINLSKMAKAYANNKAESIVEETGDVLMSPKKKKKI
>Dracunculus_med_VDN58570.1_unnamed_pr
MATNSKRIHQLNKQMIFNGGKYILYIMRCIRQKSSPALTFSCKRANLLNVPVISAFFYDYQNHNKAQRVFLME
GLIEVKKKMNSSGIPLICLKANNKTAVQIAADLSKWACEVVIDAGYLRHERLFEENLNNVLVDGNRRLTRVEG
NVVVPVTLASSLLEFGARTLRPKIWQHVADMLKEKWNDQPIVQCKDLKSVVDVKLDYMDLSVELKEALEQRNS
KNKFIGGENEASKTLDDFISKKINGYETKRNIPGTDYQSSLSPYLHYGMINPVDIIRRVNESKVNKASKDAFI
EELLVRRELSHNFVYYAMDNYDKMHGYLRMYWAKKVIEWTPSYQHALDFLIEQNDKYELDGNDPNGYAGVFWN
FGLHDRAHAERKIFGKLRYMNAEGLRRKYKNNIEKFVEINYRLANRQLDEPLPKKSKKS
>Dreissena_poly__KAH3736653.1
MDEAPKRNVLHWFRKGLRLHDNPSLREALDGANSFRCVYILDPWFAGVSQVGINRWRFLLQCLEDLDSSLRKL
NSRLYVVRGQPADVFPKLFKEWNINVLSFEEDPEPFGRSRDSAISALAQEIGVEMIVRISHTLYDLQKIISEN
NGTAPLTFKRFQSVLSRMDPPAKPEDRISLDTICQCRTPIALDHDDKYGVPTLDELGFDTSGLSSKVFRGGES
EALQRLHRHLERKAWVASFEKPKMTSQSLFPSQTTLSPYLRFGCISPRLFYWKLQELYRKIKKQNDPPLSLHG
QLLWREFFYTAATNNPNFDKMEGNAICVQVPWDKNPEALAKWAEGKTGFPWIDAIMVQLREVGWVHHLGRYAI
ACFLTRGDLWISWEEGFKVFDELLLDADWSVNAGMWLWLSCSSFFQQFLNCYCPVGFGKQADPTGDFIRHYLP
VLKGFPPQYIYEPWNAPESVQVAAKCIVGKDYPVPMVNHAEASKLCMERMKQVYQMLLKRRAKVQLPKQIAYK
ATEQEVSEDKPTREIGVEWFQEGFHVAALIESEIGVEWLQKGYYVTALLESEIGVGFHKGFHIGFHVGFHKGF
HEGFHEGFHKGFHKGFHKGFHNGFRKDFMWLPFLN
>Dreissena_poly__KAH3738683.1
MATSTMPPAQTSSDSVEVKKVAIHWFRHGLRIHDNPALLETVEEGWELYPVFIFDGEVAGTRTAGYNRMRFLI
ESLRDLDETFNNYGGRLYVFTGDLMNILGNLIKEWNVSRLSFDADVEAIWHKRDQDVRRLCSELDVEVVERVS
HVLWDPKDILENNGGKPPLTYSLFNQVARAVGDPPKTVDDPDFSKVTFKVKSNHDKLFGIPDVETLGVFPECE
QQNQRHNNWVGGETRALQLLSQRMQVEKMAFSVGMVMPNQTFPNVSGPPLSMSAHLRFGCLSIRKLYWVLRNA

FEKSHPGKGFSNSAIGQLLWREYFYTMSVNNINYDKMEENPICLNIPWYNDQEKLKKWENGETGFPWIDAIMK
QLKHEGWIHHVCRHATSTFLTRGDLWLNWVDGLKVFTKYLLDADWSVCAGNWMWMSSSAFEKVLQCPKCFCPV
RYGRKMDPDGVYIRRYLPVLKDMPLRYLFEPWKAPKSVQEKANCIIGVDYPLPMVEHGKASKQCYNMMMEVKN
KLLATGKEMELCGPANTEEVAQFVWMPGNHAGTCHQGEEEANPSMLCDGLMGM
>Dreissena_poly__KAH3779326.1
MSKRQEIDKILTEFTSGSIAADECFCLILSLCGLQEIESRFLTLIQGLRPRYGELYNRLCNVYMGYFDIKPSQ
IPSFLPDLDRAVENGYYATDYGSNNIGFELDMELALKLSEQEYTALNNGYSKAQTSKSPGFSSKPPGFSSNPP
GFSSKPPGFSYAGALKTGSNPMPVTFSAAKPLNNQLDNVKHTYTPVKTVDQLACNSKDKTEASGTAEDSIGQN
SDVTEKPKQKRKKKKNRVDRPLSNPSVIFWFRRDLRVYDNPALVAASQSGAPVIPIFLWSDREEGPQKTLATG
AATKYWLYMALPKLNSDLNNQFNNSIIYRKADNYLNELLDLFKSSGAKTLIINDVYEPFLKSRDDKICSVLKS
KGIKCERHHSYLLHEPGTIKTESLCMRGMGSVTHFMEMCRQCSTSPIGLPIDPPECLPAGTYRPESMPLEMLD
LGKLPKRKDGTVIDWAKIIRESWDFSETGAWEALQLFLNDGVRRYEKESSRGDQLNTCRISPYLHFGQISPRT
VLCEGRHMKSPKFLRKLAWRDLSYWMLSVLPDLPSEPSRPQYIHQRWSQNKTHLKAWQKGNTGYPLVDAPMRQ
LWLTGWMNNYMRHVVASFLISYLHISWVEGYLWFQDTLLDADVAINAMMWQNGGMSGLDQWNFVMHPVDAAMT
CDPKGDYVRKWVPELAKLHEDFIHQPWKAPPSILRRAGIELGVNYPHRIITELEEAREQSLRDVVEVRQKFPE
MIDPKTGNDLVRLPSGVRVPVITRKEFKYKTSNPDGNENPHNAVLRGYRGRKRDELVEFFNQRDFLASTMKEC
TSRHENGLKQMDLSFL
>Dreissena_poly__KAH3805941.1
MSSSSSKEEKKRKLENGGSDEPSPKKTQVEDLASRISEARKSLCSSVADFKFNKKRVKVLSKSEDIPNECDGI
VYWMSRDQRVQDNWAFLYAQKLALKLEVPLHVCFCLVPKFLEATIRHFTFMLEGLKEVEKECTELGISFHLLI
GHAKDRLPQFVAENKIGGVVTDFSPLRVPMAWVDDVGKALPKNIPFCQVDAHNLVPCWEASPKLEYGARTIRN
KIHNQLAGYLTEFPPVCKHKHTAPDSKSVDWAAAYASLEVDLSVKPVDWAKPGSSHGIKMLESFCKDRLKDFA
TDRNNPNKNALSNLSPWIHFGQLSAQRCILTVRQYKAKHKESVDAYIEEAVIRRELGDNFCYYNKKYDSIEGA
YDWAKKTLQDHEKDKRDHVYTREQLERGKTHDPLWNAAQNQMVLEGKMHGFLRMYWAKKILEWTPSPSEALSY
SIYLNDKYSLDGRDPNGYVGCMWSICGIHDQGWAERAVFGKIRYMNYQGCKRKFDVTKFEMRYRKQTTEK
>Dromaius_novaehollandiae_XP_025957051.1_cryptochro
MPHRTIHLFRKELRLHDNPTLLAALESSEVVYPIYILDRKFMTSVMNIGALRWHFLLQSLENLQKNLLQLDSC
LLVIQGEYGAVLRDHVKKWNITQVSLDAEVEPFYKEMEANIRSLGEELGFEVLSLIGHSLYDAKRILDLNGGT
PPLTYKRFLHILSLLGDPEAPVRNLAAEDFQRCRPPDLCLAECYRVPLPVDLKIPTECLSPWKGGETEGLQRL
EQHLMNQGWVASFTKPRTIPNSLLPSTTGLSPYFSLGCLSVRSFFYRLSNIYAQSKHHSLPPVSLQGQLLWRE
FFYTVAAATPNFTKMSGNPICLQINWYEDAERLHKWKTAQTGFPWIDAIMTQLRQEGWIHHLARHAAACFLTR
GDLWISWEEGMKVFEEMLLDADYSINAGNWMWLSASAFFHQYTRIFCPVRFGKRTDPEGQYIRKYLPILKNFP
SKYIYEPWTASEEEQKEAGCIIGQDYPFPMVNHKEVSDHNLQLMKQVREEQYRTAQLTRDDADDPMEMKIKRD
HSEENVKKGKVARTTEQT
>Dromaius_novaehollandiae_XP_025963309.1_cryptochro
MAAAASATPRGPCRSVHWFRRGLRLHDNPALQAALRDAVALRCIYILDPWFAASSAVGINRWRFLLQSLEDLD
NSLRKLSSRLFVVRGQPTDVFPRLFKEWGVTRLTFEYDSEPFGKERDAAIVKLAKEAGVEVVIENSHTLYDLD
RIIELNGHKPPLTYKRFQAIISRMELPKKPVSSIISQQMATCKVDIQENHDDVYGVPSLEELGFPTDGLAPAV
WQGGETEALARLDKHLERKAWVANYERPRMNANSLLASPTGLSPYLRFGCLSCRLFYYRLWELYKKVKRNSTP
PLSLYGQLLWREFFYTAATNNPKFDRMEGNPICIQIPWDRNPEALAKWAEGKTGFPWIDAIMTQLRQEGWIHH
LARHAVACFLTRGDLWISWESGVRVFDELLLDADFSVNAGSWMWLSCSAFFQQFFHCYCPVGFGRRTDPSGDY
VKRYLPKLKGFPSRYIYEPWNAPESVQKAAKCIIGVDYPKPMVNHAETSRLNIERMKQIYQQLSRYRGLCLLA
SVPCVEDLSGQVADPALGHSSSTSTVMRLPQSDQTSPKRKHEGAEELCTEELHKRAKVGGLPAPEIPGKSL
>Dromaius_novaehollandiae_XP_025973731.1_cryptochro
MGVNAVHWFRKGLRLHDNPALRECIQGADTVRCVYILDPWFAGSSNVGINRWRFLLQCLEDLDANLRKLNSRL
FVIRGQPADVFPRLFKEWNIAKLSIEYDSEPFGKERDAAIKKLASEAGVEVIVRISHTLYDLDKIIELNGGQP
PLTYKRFQTLISRMEPLEMPVETITPEVMEKCTTPVSDDHDEKYGVPSLEELGFDTDGLPSAVWPGGETEALT
RLERHLERKAWVANFERPRMNANSLLASPTGLSPYLRFGCLSCRLFYFKLTDLYKKVKKNSSPPLSLYGQLLW
REFFYTAATNNPRFDKMEGNPICVQIPWDKNPEALAKWAEGRTGFPWIDAIMTQLRQEGWIHHLARHAVACFL
TRGDLWISWEEGMKVFEELLLDADWSVNAGSWMWLSCSSFFQQFFHCYCPVGFGRRTDPNGDYIRRYLPVLRG
FPAKYIYDPWNAPESVQKAAKCIIGVNYPKPMVNHAEASRLNIERMKQIYQQLSRYRGLGLLATVPSNPNGNG
NGGLMGYSPGENISACGSTGGAQLGTGDGHTVGVQTCALGDSHTGASGIQQQGYCQASSILHYAHGDNQQSHL
LQTVLLEENIPDKEDKGQNYDTLYWN
>Dromaius_nov_XP_025949274.1_photolyase
MVEEVLFLFTGIVCHHATLARLVIPSATAWSRPWRLQYLSQHCPPTRTVLRMPKKKGKRKAGQGSEPEAEPDA
TELPRLSPKRNKEEAAMGKLQEAVREARRKAAPSVQEFKYNKKRVRLISQRSDLKDNAECILYWMSRDQRVQD
NWAFLYAQRLALKQELPLHVCFCLVPKFLDATIRHYGFMLRGLQEVAKECTELNIPFHLLXGYAKDVLPTFVV
RLGVGGLVTDFCPLRVPQQWVEDVRERLPEDVPFVQVDAHNIVPCWVTSPKQEYNARTIRSKIHSQLPEFLTE
FPPVIQHPYSPSTPAEPIAWEACYSSLQVDCTVKEVEWATPGTASGLTVLQSFIAKRLKSFGSHRNNPNKAAL
SNLSPWFHFGQVSTQRAILEVQKHHKKYKESVDIFVEEAVVRRELADNFCYYNKNYDSVQGAYDWAQTTLKLH

AKDKRPFLYELQELEKGNTHDPLWNAAQLQMVREGKMQFAVYLNDRYELDGRDPNGYVGCLWSICGIHDHGWP
ERAVFGKIRYMNYAGCKRKFDVVQFERRYAPCKLDK
>Dromaius_nov_XP_025952431.1_cry_DASH-l
MSAAGRTVLCLLRSDLRAHDNEVLHWAQSNADCVVPLYCFDPRHYVGTHCHGFPKTGPHRLRFLLESVRDLRE
TLKKKGSTLVVRKGKPEDVVHDLITQLGSVSAVAFHEEATKEELDVEKGLCQVCGQHGVKIQTFWGSTLYHRD
DLPFRPITRLPDVYTEFRKAMETEAKVRPTLHMADQLKPLAAGVEEGCIPTMEDLGQKDFVTDPRTAFPCSGG
ETQALMRLQYYFWDTNLVASYKETRNGLIGMDYSTKFAPWLALGCISPRYIYEQIRKYEKDRTANQSTYWVLF
ELLWRDYFRFVALKYGRRFFSLRGLQGKEVPWKKDLQLFDCWKEGKTGVPFVDANMRELAATGFMSNRGRQNV
ASFLTKDLGLDWRMGAEWFEYLLVDYDVCSNYGNWLYSAGIGNDPRDSRKFNMIKQGLDYDGSGDYVRLWVPE
LQGIKGGDIHAPWALSSASLSQAGITLGETYPRPVVTAPEWSRHISQRPQGRGPHPKGRKGPAQTPMQHKDRG
IDFYFSRKKDL
>Dromiciops_glir__XP_043823907.1
MGVNAVHWFRKGLRLHDNPALKECIEGADTVRCVYILDPWFAGSSNVGINRWRFLLQCLEDLDANLRKLNSRL
FVIRGQPADVFPRLFKEWDIAKLSFEYDSEPFGKERDAAIKKLASEAGVEVIVRISHTLYDLDKIIELNGGQP
PLTYKRFQTLISKMEPLATPVETLTPEMMNKCVTPLSDDHDEKYGVPSLEELGFDTDGLPSAVWPGGETEALT
RLERHLERKAWVANFERPRMNANSLLASPTGLSPYLRFGCLSCRLFYFKLTDLYKKVKKNSSPPLSLYGQLLW
REFFYTAATNNPRFDKMEGNPICVQIPWDKNPEALAKWAEGRTGFPWIDAIMTQLRQEGWIHHLARHAVACFL
TRGDLWISWEEGMKVFEELLLDADWSINAGSWMWLSCSSFFQQFFHCYCPVGFGRRTDPNGDYIRRYLPVLRG
FPAKYIYDPWNAPEGIQKVAKCVIGVNYPKPMVNHAEASRLNIERMKQIYQQLSRYRGLGLLASVPSNPNVNG
GLMGYTPGENISAGSSSSGAVPATANDGQTLKGCVPPEPTTGASGTQQQGRSTLSGNKRPRQEEDPQSAGPKV
QRQSTN
>Dromiciops_glir__XP_043826737.1
MAATIVVTVTAASAVPAPALAPAPTPTPTPTPTPTPTPTPTPTPAPEEGASSVHWFRKGLRLHDNPALQAALR
GARCVRCVYILDPWFAASSSVGINRWRFLLQSLEDLDISLRKLNSRLFVVRGQPTDVFPRLFKEWGVTRLTFE
YDSEPFGKERDAAIVKMAKEAGVEVVTENSHTLYDLDRIIELNGQKPPLTYKRFQAIISRMELPKKPVSCVTS
QQMERCQAEIRENHDDAYGVPSLEELGFPTDGLGPAVWQGGETEALARLDKHLERKAWVANYERPRMNATSLL
ASPTGLSPYLRFGCLSCRLFYYRLWELYKKVKRNNSPPLSLYGQLLWREFFYTAATNNPKFDRMEGNPICIQI
PWDRNPEALAKWAEGKTGFPWIDAIMTQLRQEGWIHHLARHAVACFLTRGDLWVSWESGVRVFDELLLDADFS
VNAGSWMWLSCSAFFQQFFHCYCPVGFGRRTDPSGDYVRRYLPQLKGFPSRYIYEPWNAPESVQKAAKCIIGV
DYPKPIVNHAETSRLNIERMKQIYQQLSRYRGLCLLASVPSCMEDLSTPMAEVSVGQASSVSGAGPKALPCGP
ASPKRKLDTTEEASGEEHSKRARGMAALSIAELAVKDA
>Dromiciops_glir__XP_043854557.1
MAPKKRSHSTSGEPEKMESQENKAKRKPLQKHQFSKSNMVQKEEEDKKEAEKKGGAEGLQEVVRQSRLRTAHS
VLEFRFNKQRVRLISQDCHLQDHSQAFVYWMSRDQRVQDNWAFLYAQRLALKQKLPLHVCFCLAPCFLGATIR
HYDFMLRGLEEVAEECEKLCIPFHLLLGLPKDVLPAFVQTHGIGGIVTDFSPLLHHTQWVKDVQDALPKQVPF
VQVDAHNIVPCWVASDKQEYGARTIRHKIHDRLPHFLTEFPPVICHPYPSNIQAEPVDWNACRAGLQVDRSVK
EVSWAKPGTASGLTMLQSFIAERLPYFGSDRNNPNKDALSNLSPWFHFGQVSVQRAILEVQKHRSRYPDSVAN
FVEEAVVRRELADNFCFYNKNYDKLEGAYDWAQTTLRLHAKDKRPHLYSLEELESGKTHDPLWNAAQMQMVKE
GKMHGFLRMYWAKKILEWTRSPEEALEFAIYLNDRFQLDGRDPNGYVGCMWSICGIHDQGWAEREVFGKIRYM
NYAGCKRKFDVAEFERKYSPAD
>Drosophila_mel_BAA12067.1_photolyase
MDSQRSTLVHWFRKGLRLHDNPALSHIFTAANAAPGKYFVRPIFILDPGILDWMQVGANRWRFLQQTLEDLDN
QLRKLNSRLFVVRGKPAEVFPRIFKSWRVEMLTFETDIEPYSVTRDAAVQKLAKAEGVRVETHCSHTIYNPEL
VKAKNLGKAPITYQKFLGIVEQLKVPKVLGVPEKLKKMPTPPKDEVEQKDSAAYDCPTIKQLVKRPEELGPNK
FPGGETEALRRMEESLKDEIWVARFEKPNTAPNSLEPSTTVLSPYLKFGCLSARLFNQKLKEIIKRQPKHSQP
PVSLIGQLMWREFYYTVAAAEPNFDRMLGNVYCMQIPWQEHPDHLEAWTHGRTGYPFIDAIMRQLRQEGWIHH
LARHAVACFLTRGDLWISWEEGQRVFEQLLLDQDWALNAGNWMWLSASAFFHQYFRVYSPVAFGKKTDPQGHY
IRKYVPELSKYPATCIYEPWKASLVDQRAYGCVLGTDYPHRIVKHEVVHKENIKRMGAAYKVNREVRTGKEEE
SSFEEKSETSTSGKRKVRRATGSAPKRKR
>Drosophila_mel_NP_523653.2_photorepai
MFTLASYWRESFKIVLPLQAMKRTKAQKAGPSKKAAKNEKASSEPKSDQESSDEEASTSKALLVSKPDYQNFE
QFLTHLEHQRVCTAANIQEFSFRKKRVRVLSKTEDVKESSLGGVVYWMSRDGRVQDNWALLFAQRLALKLELP
LTVVFCLVPKFLNATIRHYKFMMGGLQEVEQQCRALDIPFHLLMGSAVEKLPQFVKSKDIGAVVCDFAPLRLP
RQWVEDVGKALPKSVPLVQVDAHNVVPLWVASDKQEYAARTIRNKINSKLGEYLSVFPPVVRHPHGTGCKNVN
TVDWSAAYASLQCDMEVDEVQWAKPGYKAACQQLYEFCSRRLRHFNDKRNDPTADALSGLSPWLHFGHISAQR
CALEVQRFRGQHKASADAFCEEAIVRRELADNFCFYNEHYDSLKGLSSWAYQTLDAHRKDKRDPCYSLEELEK
SLTYDDLWNSAQLQLVREGKMHGFLRMYWAKKILEWTATPEHALEYAILLNDKYSLDGRDPNGYVGCMWSIGG
VHDMGWKERAIFGKVRYMNYQGCRRKFDVNAFVMRYGGKVHKKK
>Drosophila_mel_NP_732407.1_cry

MATRGANVIWFRHGLRLHDNPALLAALADKDQGIALIPVFIFDGESAGTKNVGYNRMRFLLDSLQDIDDQLQA
ATDGRGRLLVFEGEPAYIFRRLHEQVRLHRICIEQDCEPIWNERDESIRSLCRELNIDFVEKVSHTLWDPQLV
IETNGGIPPLTYQMFLHTVQIIGLPPRPTADARLEDATFVELDPEFCRSLKLFEQLPTPEHFNVYGDNMGFLA
KINWRGGETQALLLLDERLKVEQHAFERGFYLPNQALPNIHDSPKSMSAHLRFGCLSVRRFYWSVHDLFKNVQ
LRACVRGVQMTGGAHITGQLIWREYFYTMSVNNPNYDRMEGNDICLSIPWAKPNENLLQSWRLGQTGFPLIDG
AMRQLLAEGWLHHTLRNTVATFLTRGGLWQSWEHGLQHFLKYLLDADWSVCAGNWMWVSSSAFERLLDSSLVT
CPVALAKRLDPDGTYIKQYVPELMNVPKEFVHEPWRMSAEQQEQYECLIGVHYPERIIDLSMAVKRNMLAMKS
LRNSLITPPPHCRPSNEEEVRQFFWLADVVV
>Dryobates_pube__KFV67658.1
RFLLQSLEDLDNSLRKLNSRLFVVRGQPTDVFPRLFKEWGVTRLTFEYDSEPFGKERDAAIVKLAKEAGVEVV
IENSHTLYDLDRIIELNGHKPPLTYKRFQAIISRMELPKKPVSTVISQQMETCKVDIQENHDDVYGVPSLEEL
GFPTDGLAPAVWQGGETEALARLDKHLERKAWVANYERPRMNANSLLASPTGLSPYLRFGCLSCRLFYYRLWE
LYKKVKRNSTPPLSLYGQLLWREFFYTAATNNPKFDRMEGNPICIQIPWDRNPEALAKWAEGKTGFPWIDAIM
TQLRQEGWIHHLARHAVACFLTRGDLWISWESGVRVFDELLLDADFSVNAGSWMWLSCSAFFQQFFHCYCPVG
FGRRTDPSGDYVKRYLPKLKGFPSRYIYEPWNAPESVQKAAKCIIGVDYPKPMVNHAETSRLNIERMKQIYQQ
LSRYRGLCLLASVPSCVEDLSGPVTDSASGQGCSTSTALRLSQAEQASPKRKHEGAEELCTEELYKRAKVTEL
PAAELPGKS
>Dryobates_pube__KFV69614.1
DNWAFLYAQRLALKQELPLHVCFCLVPKFLDATIRHYSFMLRGLQEVAKECAELNIPFHLLLGYAKDVLPTFV
VEHGVGGLVTDFCPLRLPRQWVEDVRERLPEDVPFAQVDAHNIVPCWVASPKQEYSARTIRGKIHAQLPEFLT
EFPPIIRHPYTSSCLAQPIAWEVCYSSLQVDHTVKEVEWATPGTAAGLAVLQSFITERLESFGSHRNDPNKAA
LSNLSPWFHFGQVSTQRAILEVQKHRDKYKESVDVFVEEAVVRRELAENFCYYNENYDSVQGAHDWAQTTLKL
HSKDKRPFLYTLEELEHGTTHDPLWNAAQLQMVREGKMHGFLRMYWAKKILEWTRSPEEALQFAIYLNDRYEL
DGRDPNGYVGCLWSICGIHDQGWAERAVFGKIRYMNYAGCKRKFDVGQFERRYAP
>Dryobates_pube__KFV74926.1
RFLLQCLEDLDANLRKLNSRLFVIRGQPADVFPRLFKEWSIAKLSIEYDSEPFGKERDAAIKKLASEAGVEVI
VRISHTLYDLDKIIELNGGQPPLTYKRFQTLISRMEPLEMPVETITPEVMAKCTTPVSDDHDEKYGVPSLEEL
GFDTDGLPSAVWPGGETEALTRLERHLERKAWVANFERPRMNANSLLASPTGLSPYLRFGCLSCRLFYFKLTD
LYKKVKKNSSPPLSLYGQLLWREFFYTAATNNPRFDKMEGNPICVQIPWDKNPEALAKWAEGRTGFPWIDAIM
TQLRQEGWIHHLARHAVACFLTRGDLWISWEEGMKVFEELLLDADWSVNAGSWMWLSCSSFFQQFFHCYCPVG
FGRRTDPNGDYIRRYLPVLRGFPAKYIYDPWNAPESVQKAAKCIIGVNYPKPMVNHAEASRLNIERMKQIYQQ
LSRYRGLGLLATVPSNPNGNGNGGLMSYSPGESISGCGSTGALFSASPGAQLGTGDGHTGVQSCPLGDSHTGA
SGIQQQGYCQASSILHYAHGDNQQPHLLQAGRTALGTGISAGKRPNPEEETQSVGPKVQRQSTN
>Dryobates_pube__XP_009894705.1
MSHRSIHLFRKGLRLHDHPALLAALQSSQLLYPLYILDQQFLSSVMHMGALRWHFLLQSLQDLQESLCQLGSC
LLVVQGQYEAVLRELVQQWNISQVTLDAEVEPFYKEMEARIRCLGEELGFEVLSLVGHSLYDTKRVLELNGGS
PPLTYKRFLHILSLLGDPELPVRTLTAEDFQRCQAPAPGLAELXRVPLPGDLQMPPESLSPWRGGETAGLRRL
EQHLADQGWVTAFAKPRTIPNSLLPSTTGLSPYFSLGCLSVRTFFHRLAKIYAQAKHHSLPPVSLQGQLLWRE
FFYTVASATPNXXXXXXXXCQQISWAEDAERLHRWKTVRRGSPGIDAIMTQLRQEGWIHHLARHAAACFLTRG
DLWISWEEGMKVPPCSAVVFLLGDLGEPGCGRRKYLPILRNFGSKHIYEPWTASEEEQKQAGCIIGQDYPFPM
VNHKEVSEHNLQLMKQVREEQHRTAQLTRGETEDPMELKARCDHSEEDLPKGKVAKVAEXSPAGLSCWGPTRW
QRGQPEAT
>Dryobates_pube__XP_009897427.1
GAAGPAFCLLRCDLRVHDNQVLHWAQSNADFVIPLYCFDPRHYLRTHCYGFPKTGPHRLRFLLESVKDLRETL
KKKGSTLVVRKGKPEDVVSDLITQLGSVSAVAFHEEATQEELDVEKGLCQVCSQHGVKVHTFWASTLYHRDDL
PFRPIARLPDVYTHFRRAAESEAKVRPTLRMADQLKPLAPGVEEGCIPTMEELGQKDPVTEPRTAFPCSGGET
QALTRLQYYLWDTNLVASYKETRNGLVGMDYSTKFAPWLALGCISPRYIYEQIQKYEKERTANQSTYWVLFEL
LWRDYFRFVALKYGTRIFSLRGLQNKEMPWKKDLRLFDCWKDGRTGVPFVDANMRELAATGFMSNRGRQNVAS
FLTKDLGLDWRMGAEWFEYLLVDYDVCSNYGNWLYSAGLGNDPRDSRKFNMVKQGLDYDGSGDYVRLWVPELQ
GIKGADIHTPWVLSSAALSQAGVTLGETYPEPVVTAPEWSRHISQRPQGRGPHPRGRRGPTHMPAQHKDRGID
FYFSRKKDAR
>Dryodora_gla_AQX17841.1_putative_p
MSAVGVHWFRYGLRLHDNAPLLKAISSCSTLIPLYIIDTDYFTPSSCGINRMGFLLDSLKALDEDLREKGSKL
FVAKGKPCDILRTIIQQNKVEILTFERDTEPYNRAMDESVFSMAESLKIDVSALWGHTMFDPEYLLGLNNGSA
PLTMTSFQKLMYVTGHPPKPLDPPGKITPPPEGPLRCEGVHLFPNPPSLEDLNEFNFKQEDKTTWFVAGEKEA
MKVMEKFFAQKRRTANFEKPKTSPTALEPDTTALSPYMSRGSLSSRLFYHRLKDTVKDFKNASKPPVSLQGQL
YWREMAYLIGFSTPNFSQMKGNPICKQIPWQTGPDAKILLEKWESGQTGFPAVDAVMNQLKTEGWMHHLARHL
VACFLTRGDLWVSWEMGRDVFDKHLVDADWSINNFSWHWLSCSAFFHQYFRCYSPIAFFKKTDPNGAYIKKHV
PMLSKFPAKYIYEPWTAPKATQEACGCVIGRDYPKPMVDHSTVNKINMGKMKKAYDAAKPLDRGKKRETDGKD
SPAKRKQSTLSNFSEKRINC

>Dunaliella_sal_AAX56342.1_6-4_photol
MLRCAQPKYAPLKRQAFNQKLNRLVAAARTPSKSSSTSRMASTSSGQQGRSILWFRKGLRLHDNPALRDACTG
SAAVFPIFIIDPYFLQKSNNKVGVNRYQFLLESLSDLNSSLTSLGSQLLVLRGTPEEVIPRVLRDWSIKKLCY
EIDTEPYAKARDARVDDMAREAGVEVKKHWSHTLYDTDMLVRENKGKAPLTMQAFEKLVDRVGHPLTALPAPT
ARLPPVDVSLPGIKDAEVGVPTWQEMGFKEAPTAIFKGGETEALKRLEHYMKDTKWXASFEKPSTDPSAFTEP
STTALSPYLKFGCLSARFFHQRLLDVYRLHPKHSQPPMSLRGQLLWREFFYTLGSHTPNFDRIAGNPICRQIT
WDTNPALLKAWRDGATGYPWIDAAMTQLREWGWMHHLARHSVACFLTRGDLYLSWESGKEVFEELLLDADYFI
NAANWMWLSASAFFAQYFRVYSPVVFGKKYDKEGAYIRKFLPVLKDMPAKYIYEPWTAPKEVQQRANCIIGRD
YPAPIVDHAVASKECIARMGAAYKATNTGGSAGKASPAKAASSGDAGTSASAGAPSSSKKTTGKRAASADQGG
KRQKTLEESMTKKRCQ
>Dunaliella_sal_ABK76277.1_CPD_photol
MPKRKAQHPPEDDPSAGNAGPSSQPSSANHQLVDQRRVSELLSGNNSPTPPVVYWMSRDMRVSDNWALLQGCQ
EALQRLQLASHADTAAAAAAATAAHSSPALAQPPLVVAFNLVPQFLGAGARSFCFMLKGLRELETKLRQFNIP
FYLLRGQPEDTIPQLVDALRAGLVVSDYSPLRLSKQWKQQVAAKLQAKGVGFQVVDAHNVVPAKIASNKREYA
ARTIRPKIEKLLPEFLIEFPNQGLPPIMPTAGQGPHPPTPIDWPALLAEVTEAGAAVPEVDWITPGEAAARAA
LDGSQGFLTTPRIAQYHVKRNDPSCTTGLSNLSPYLHFGQLSAQRAALEASKLRSRHREAVDRYLEELIVRRE
LADNFCEHCPDYDKLVPGTAYDWALKSLEKHKRDPRPITYTRQQLESGHTGDDLWNAGQMELVRQGKMHGYIR
MYWAKKILEWSGTPEEAVENAIYLNDKWSLDGRDPSGYTGVMWSVAGVHDRAWIDRPIYGKIRVMTYDGCKGK
FDVPAYVAYVESLGRARQ
>Dunaliella_sal_DNA_photolyase_KAF5834002.1
MAMEPPYPAAPQHPTSAPMPPLASFMVARGHAPYLSNRQPVALSRSRRSSPATTWNHPSQQRCFPASPSSLAY
HTRSGAGSSKQGCVASNTAAAQPPWLFCLLQRIPGLLLRVLRGQATNGPQAVLQRATMAGAGLLFGLGMLAKL
PKRGNAAGDGHGRRAAPLHAGIVWMRSDLRVSDHEALSAAAQECSSLVPLYCFDPAEYGKDGSSFERTGPGRA
RFTIESVVALRQELRALGSDLIVRVGRPQDVLPCLAAQVGAQAVYCHSDVASEAQQVESRVHEALRCMGVQLK
TFWGGSTLFHADDLPFARTAVPANYAHFSQALAQVPVRAPTPTPTLLQAMPAGCCINPGDVPTLTQLGLTKEA
ELIPPSALLGPGRLRGGEGEALKHLRAFILDVKAAAGRQQEQQQGAAMKKELPGSGGSGPNLQGMCIGPTFSC
KISPWLALGCISPRTVYHEMRHQLGLLGSLGGLSKEGNVPTGLEHRALGSNWLMFELLWRDFFRFKSAPPALP
G
>Dunaliella_sal_KAF5839248.1
MAAEGAKRRLIVWLRNDLRLTDNTLLHEAASLCSGATGQAKYDEVLPVYIFDPRSYAVLKTGDYKTGVYRALF
VVQSVKDLKARLRQAIGSDLLVLVGKPEEVIQCLVRGCQSPQVLSSQEVTSEELKVEAALERALKPVGGRLTR
LWSNTLYHMDDLQSPQGPLGARGIKGMPDGFTSFREKVERGSRVRQALPAPSKGSLPLPSSASQLQVPSSALL
DRGQSGGAGDGGPSLQALLQADPSFESLPWAEGAAPQPPGTHPQAVLKFQGGETAALARLKYYLWDSDLLSTY
FDTRNGMLGGDYSTKFAPWLAAGCISPRTIYHEIKKYEAQRIANKSTYWVIFELIWRDFFRFFAAKHEDRIFY
ESGTIGKKLPWVKDPSRYERWAQGKTGLPLVDANMRELAATGVCNIVNVNHLHTHLFASHRENAFMGRHPV
>Echeneis_nauc__XP_029353429.1
MTHTCIHWFRKGLRLHDNPALKSALRDSKELYPVFILDPHLHDSTRVGINRWRFLIGSLKDLDCSLRKLNSRL
FVVRGKPEEVLPELFDKWKVTKLTYEYDTEPYSLSRDQKVSALAKEHGVEVTYKISHTLYDIDRIIEENNGKA
PLTYNRMQAVVKTLGAPKKPVPAPTLDDIKDVKTPCSDKHEKYGIPALEELSRDTAALGEDLFPGGEQEALRR
LDEQMKRTGWVCTFEKPQTSPNSLSPSTTVLSPYLTFGCLSARTFWWRLTEVYRGKKHSDPPVSLHGQLLWRE
FFYTASVGIPNFNKMEGNPVCTQVDWDTNPEYLAAWREARTGFPFIDAVMTQLRQEGWIHHLARHAVACFLTR
GDLWISWEEGQKVFEELLLDGDWALNAGNWQWLSASTFFHQYFRVYSPVAFGKKTDKSGDYIKKYLPLLKKFP
EQYIYEPWKAPRSIQQAAGCIVGKDYPHPVVQHEVISKKNIQRMKSAYAKRSADPNESPSKKQGVKRKATSVV
DMLIKKVRRKSL
>Echeneis_nauc__XP_029378125.1
MVVNSVHWFRKGLRLHDNPALQEALNGADTVRCVYILDPWFAGAANVGINRWRFLLEALEDLDNSLKKLNSRL
FVIRGQPTEVFPRLFKEWKVTRLTFEYDPEPYGKERDGAIIKMGQEFGVETIVRNSHTLYNLDRIIEMNNNSP
PLTFKRFQTIVSRLELPRRPLLTITQQQMDECRTKIADNHDQLYSIPSLEELGFRTEGLPPAMWQGGESEALD
RLNKHLDKKVWVANLEHPRVNTCSLYASPTGLSPYLRFGCLSCRVLYYNLRELYMKLRKRCSPPLSLFGQLLW
REFFYTAATNNPNFDRMEGNPICVQIPWDQNPEALAKWAEGRTGFPWIDAIMTQLRQEGWIHHLARHAVACFL
TRGDLWISWESGMKVFEELLLDADWSVNAGSWMWLSCSAFFQQFFHCYCPVGFGRRTDPSGDYIRRYIPILKD
YPNRYIYEPWNAPEFVQKAANCVVGVDYPKPMINHAEGSRLNIERMKQVYQQLSHYRGLSLLASVPTIQEEAE
PPMTDESQTSSGPDSPPRDPVDSEAAGSSTAPDSSTVCASSATAPCSDLEHLLNSHQTGIRCTPSHTQLCAAV
TSTSASHQSCLAASPSPSTPIQSPLSRSKTPPPNSCTTLLPSASPATTVAQTFPKRKSLARKVRRSQRHRGRQ
SCTPACKEGERRVKEEQKDESGGEERMEQTVEMDEERMEEEESGEIATPQQ
>Echeneis_nauc__XP_029378273.1
MGVNTIHWFRKGLRLHDNPSLRDSIRGADTLRCIYILDPWFAGSSNVGINRWRFLLHCLEDLDASLRKLNSRL
FVIRGQPTDVFPRLFKEWQINRLSYEYDSEPFGKERDAAIQKLANEAAVEVMVQISHTLYDLDKIIELNGGQP
PLTYKRFQALINRMDAVELPAETITCEVIKKCATPIGDDHDDKFGVPSLEELGFETEGLTTAVWPGGETEALM
RLERHLERKAWVANFERPRMNANSLLASPTGLSPYLRFGCLSCRLFYFKLSDLYRKVKKNSTPPLSLYGQLLW

REFFYTTATNNPCFDKMDGNPVCVQIPWDRNPEALAKWAEGRTGFPWIDAIMSQLRQEGWIHHLARHAVACFL
TRGDLWISWEEGMKVFEELLLDADWSVNAGSWMWLSCSSFFQQFFHCYCPVGFGRRTDPNGDYIRRYLPILRA
YPAKYIYDPWNAPEEVQKAAKCIIGVHYPKPMVNHAEASRINIERMKQIYQQLSCYRGLGLLATVPANPTNGP
SGSNVTGVNTGTSQGPGGSSEEQYVQEGTSRTERGQVTQKRRREEAPSESSSKSWRPSK
>Echeneis_nauc__XP_029378353.1
MAPGAEDSKVLVSRLLREVLEGREDPEGFFAVCVSVLGQQQTRSQFLSLIRPLSSADSPLHRVLTSVYQGYYQ
TEDNELELALALSISDMKDYQISAPSQESRLQQPKDSSNQISSVRLTPKSEARGGSHAQLAVAAGKRNTSSSQ
SGAWGSVCPPKTAQETELQKGARADKHDKETSGKGPTVCVFKPSASFSGLDMGQMTKDGDLNELEKPKRPKNR
RQRRKACGQQVIGLPSSLSAPRPILLWFRRDLRLCDNPALIASLEVGAPVIPVFIWSPEEEEGPGITVAAGGA
CKYWLHQALFCFCSSLERIGSHLIFLKANAEGNAKGSSLCALKDLIKETGARTVLANALYEPWLKERDEEVVS
ALQKDGVECKLFHSYCLRDPYSVSTVGVGLRGIGSVSHFMSCCRQNPGSALGVPLDPPASLPTPSQWPQGVSL
DILGLACMPRRKDGTTIDWSANIRKSWDFSEEGAHAKLEAFLNDGVYRYEKESGRADAPNTSCLSPYLHFGQL
SPRWLLWDAKGAHCRPPKFQRKLAWRDLAYWQLTLFPDLPWESLRPSYKALRWSSDRGHLKAWQRGQTGYPLV
DAAMRQLWLTGWMNNYMRHVVASFLIAYLHLPWQEGYRWFQDTLVDADVAIDAMMWQNGGMCGLDHWNFVMHP
VDAAMTCDPCGTYVRKWCPELKDLPNEFIHKPWKCPTSMLRRAGLTLGQTYPHRVITDLEERRSQSLQDVTLV
RKKFGQYVDKHTGCDLVPLPSRLVSEALGLSHSNGGAVEAGKQFLLPVITRMEFKHQLEDPDADAGSNPYNAV
LKGYVSRKRDETIAFFNERDFTASVIYEAAQRKERLESDYRKMEGLPQPLALRGKARRTPTAKDRFSIVPGGA
VTSPW
>Echeneis_nauc__XP_029380225.1
MLLHFCRSSTFSCALLKLVRHSDQRPTSLPPLFLKSVTSETIMSGRKRKASPGPDEEAATAKQQKVTAKKEKE
RTEGWLQGLVKEQRSENKDMKFNKKRLRYISDREKIKQGSEGILYWMLRDHRVQDNWALIHAQRLALKENLPL
HVCVFLVVPKSKLSTLRHYSFMLKGLEEVAKECEALDIQFHLRLGSPGELLSGFVSDHNVGAVVTDFSPLREP
MQWLEEIKTRLPEDIPLIQVDAHNIVPCWVASPKLEYAARTIRGKITKLLPEFLTDFPLVEKHPYATTITTKP
VDWDKTLASLQVDRTVGEPEWAKPGTQGGMAMLETFIDVRLKVFATQRNDPNASALSQLSPWIRFGHISAQRV
ALQVQCGGKSAGQSVSSFIEELVVRRELTDNFCFYNKKYDSVEGANDWAQKTLKDHAKDKRPYHYTCQQLEEA
ETHDKLWNAAQYQMVKEGKMHGFLRMYWAKKILEWTSSPKEALSIALYLNDRYELDGQDPNGFVGCMWSICGI
HDQGWAERPIFGKIRYMNYKGCTRKFDVAQFERKYCPKKL
>Echeneis_nauc__XP_029385143.1
MSASRTVICLLRNDLRLHDNELFWWAQRNAEHIVPLYCLDPRHYVGTYNYNLPKTGPFRLRFLLESIRDLRNT
LLNKGSNLVVRRGKPEDVVAELIKQLGSVTTVAFHEEVTSEELNVEKKVKDVCAQMKVKVQTCWGCTLYHRDD
LPFHHISSLPDVYTQFRKAVETQSRVRPAIPTPEQLKPLPPGLEEGAIPTALDLEQPEPLTDPRSAFPCSGGE
SQALARLKHYFWDTDAVATYKETRNGLIGVDYSTKFAPWLAMGCISPKYIYHQIKQYERERTANQSTYWVIFE
LLWRDYFKFVSAKYGNRLFQVEGLQDKSVPWKKDMKLFDAWKDGRTGVPFVDANMRELAMTGFISNRGRQNVA
SFLTKDLGLDWRMGAEWFEYLLIDHDVCSNYGNWLYSAGIGNDPRENRKFNMIKQGLDYDSNGDYVRQWIPEL
QGIRGADVHTPWTLSTAALSHAQVSLGDTYPTPIVIAPEWSRHVNKKSGSTGPSPKGKKGPSHTPRQHRDRGI
DFYFSRSKNL
>Echinococcus_gr_KAH9284864.1_photo-lyas
MPAKLSTEDFSKWVERININRVESCGKSVASYPFAACRVRLVSGTNEFKSVAETNLDDISGGVGSCRGCIVYW
MSRDQRIQDNWALLFAQRIALKFSVPLHVCFNLVSSSALRTRRHASFLLEGLAEIEKECKDLEIGFHLLPVPV
TKSKSGVKRSADGNVVHHGSDTGKAEPIVDVLKQLDARVVVTDFSPLRDDLRAVQFVSSHLSQDISLYQVDAH
NVVPAWFASDKLEYSARTIRGKLHDKAKHLFTDFPPVIHHPIPSKWEAVDWKAIKQSLVGLVDESVEPVTWAK
GGAKAGLSQLFSFLHHRLRAYATHRNDPTKKALSNLSPWLHFGHISAQRCLWEVNLRRSLHKESTDAFMEEAF
VRRELSDNFCYYNPNYDSIKGAWSWAQETLRKHANDLRKPSYSEETMESATTEDDLWNAAQRQLRREGKMHGF
LRMYWAKKILEWHAEGPEKALQLGFRLDNRYSLDGTDPSGYVGVMWSICGVHDQGWKERPIFGKIRYMNFAGC
KRKFDVAAFIRRYPRRMCTLYTLAHK
>Echinops_telf__XP_004708484.1
MAAAVGTAALAATAPAPNKDGASSVHWFRKGLRLHDNPALLAAVRGARCVRCVYILDPWFAASSSVGINRWRF
LLQSLADLDARLRTLHSRLFVVRGQPADVFPRLFKEWGVTRLTFEYDSEPFGKERDAAIMKLAKEAGVEVVTE
NSHTLYDLDRIIELNGQKPPLTYKRFQAIISRMELPRKPAGAPTGLQMESCQAEIPENHAETYGVPSLEELGF
PTEGLGPAVWQGGETEALARLDKHLERKAWVASYERPRMNANSLLASPTGLSPYLRFGCLSCRLFYYRLWDLY
KKVKRNSTPPLSLFGQLLWREFFYTAATTNPRFDRMEGNPICIQIPWDRNPEALAKWAEGKTGFPWIDAIMTQ
LRQEGWIHHLARHAVACFLTRGDLWVSWESGVRVFDELLLDADFSVNAGSWMWLSCSAFFQQFFHCYCPVGFG
RRTDPSGDYIRRYLPKLKGFPSHYIYEPWNAPESVQKAAKCIIGVDYPRPIVNHAESSRLNIERMKQIYQQLS
RYRGLCLLASVPSCVEDLSNPVAEPSVSQALSMSSTGPRLPTSRPTSPKRKLEAAKAPPGEDLSKRARVAKAP
RPEPPGKAV
>Echinops_telf__XP_045148331.1
MGVNAVHWFRKGLRLHDNPALKECIQGADTIRCVYILDPWFAGSSNVGINRWRFLLQCLEDLDANLRKLNSRL
FVIRGQPADVFPRLFKEWDITKLSIEYDSEPFGKERDAAIKKLATEAGVEVIVRISHTLYDLDKIIELNGGQP
PLTYKRFQTLISKMEPLEIPVETITSEVIEKCVTPLSDDHDEKYGVPSLEELGFDTDGLPSAVWPGGETEALT
RLERHLERKAWVANFERPRMNANSLLASPTGLSPYLRFGCLSCRLFYFKLTDLYKKVKKNSSPPLSLYGQLLW

REFFYTAATNNPRFDKMEGNPICVQIPWDKNPEALAKWAEGRTGFPWIDAIMTQLRQEGWIHHLARHAVACFL
TRGDLWISWEEGMKVFEELLLDADWSINAGSWMWLSCSSFFQQFFHCYCPVGFGRRTDPNGDYIRRYLPVLRG
FPAKYIYDPWNAPEGIQKVAKCLIGVNYPKPMVNHAEASRLNIERMKQIYQQLSRYRGLGLLASVPSNPNGNG
GLMGYSPGENTTGCSSGGGCPPGNGILHYAHGDSQQAALLKQGRSPLGTGLSSGKRPSQEEDTQSVGPKVQRQ
SSN
>Echinostoma_cap_VDP70089.1_unnamed_pr
MLGGLKEVEQECRSLNIPFHLINAADFLDSEEEDPGLGVRIRWSPDYVPSQMAGSYLYDAAVARAICSLVNSL
PTGCLVVDFSPLRAPSAWVERVAASVPDRVPVCQTTYAVKVFKVQAASDVDAHNIVPVWCGSDKLESHARTIR
PKLFSKTSKYLTEFPPIIAHPFRSEFDITRHPTIDWQTVLDRYCGDKSVKAVSWAIPGTRAAFEVLRSFIDER
LKGFDSQRNNPANPALSGLSPWFHFGQIAPQRAILEVSTVQKKYGRSVDIFIEEAFNRRELAENFCFYNPLYD
SLQGARQWAQETLTQHATDTRPASYTRDELESAKSGDDLWNAAQRQLVHTGKMHGFLRQYWAKKILEWCAEGP
QTAIDWAIYLNDRFSLDGSDPNGYVGIMWAICGIHDQGWNERTIFGKVRTMTYNGCKKWFSIPAFVERFTEHN
VTSK
>Ectocarpus_sil_CBJ30497.1_cry_1
MSSSKEPSSIHWFRKGLRLHDNRALLEACNGTASLYPLFVLDSDPASPESRAGPLRTAFLLESLQDLDEQLRA
KGSRLFVVRGKPEEVLPQLFEEWNVKKLTFEADSEPRSRARDREVGSLAKEAGVQVLIRGTSTLRNLQNYHML
MGKKQGTYLKTYGAFLKLHDNAGPVPDCAPDISGDLPAPGASPDDAEYDRFSIPSLSDLGVEPLAKALKFRGG
EREALARLERVMAREDWISKFEKPKTSPNSIEPSTTVLSMYISHGCLSTRRFWHALCKVYTKKGGSKPPVSLK
GQLLWKEFNYFSGYSIPSFDKMVGNPVIRQIPWDKDEEKLMAWKEARTGFPWIDAAMTQLKDEGWIHHLARHA
VACFLTRGDLWQSWEDGAAVFDELLLDADWSINNFNWQWLSCSAFFYQYFRCYSPIAFGKKTDQNGDYIKKYL
PALRKFPSKYIYEPWTAPLQVQRGCGCIIGKDYPKPIVDHADTSKANMAKMNDAYDAHQEAQAAESHDDGGSG
SGGGGSGGGSGGGARSRARGAQPASSKRVKR
>Ectocarpus_sil_CBJ48325.1_cry_2
MMASMMSAGGTAAGGSAAALKGAAGIVWFKYSDLRLEDHEPLALAHRESAQVAHVFCIDDRWFGQTRRGTSKM
SAARCRFLLESVADLRARLRERGSDLLVRRGHPEDVIPALAARLGGGSVAGVTLFAHTDVCSEEADVHAAVKS
ALAAASSAGRGGGAAVAVKEAWGNTLHDVADLPFDFPSGVPEIFTQFRKSVESKYSCKMRPPVPLPSPFRPVP
TGTTQDDGEASAPGAIPTTEELGLGTAPERDARSVFPFNGGETAGLRRVQSYIWDEDRLREYKVTRNGLLGSG
FSSKFSPWLALGCLSPRTIVKEIRKYETDRIANDSTYWLIFELLWRDFFRYSAVKNGNSIFHLGGPRRDTGRQ
RWLDDAGSLEAWKEGETGYPLIDANMRELKASGFMSNRGRQVVASFFTRDLQMDWRLGAEHFEEYLLDHDPAS
NWGNWNYVAGVGSDPREGRYFNIEKQAKTYDAEGAYMRHWLPELAALPTTALQTPGGITTMLRHMHAIPTSVY
PDPIAPLKFSKAGGGRGGGRGGDGGRGGRGGGGRGGGGKRGGGGKGGNSGRKFKASRLQT
>Ectocarpus_sil_CBN78764.1_conserved_
MALLETPDWFKAERVRCLTDASVPRDQGNCVVYWMSRDQRAEDNWAMLYAKNLAQEGGVPLVVAFVLGAWQVT
EPKTTLRYAGFMLKGLEETEGDLRKKKIPFHLLQAAEPRDVVPAFAKELGALAIVTDMCPLRDPTRRAREVAE
ELNKSGDGTPLFQVDAHNVVPVWATSDKQETMARTIRPKIHARPDFLGAIPELSPNPQGTKLPAATDWKAAQT
SLDLDRSVPEISWLKPGAKGASDNLQSFIETRMKGFADLSNNPNEDVCSHMSPYFNLGQMSAQAAVMRVKASR
RHPDGVKAFVEQGVVRRELSDNFCFYNDNYDNLSGAAGWARESLEAHANDQREWTYSVQELEEAKTHEDLWNA
AQLQLVRDGKMHNFLRMYWAKKILEWSPSPHDALERCLFFNDKYELDGRDPNGFTGCAWSVMGIHDMGWKERE
VFGKIRYMNYAGCKRKFDVQEFVSKYAGAGEAAVAAGGTVAPAKRKPSAAKGRSSAAKKGKAVSGRKG
>Egretta_garz__KFP11678.1
RFLLQCLEDLDANLRKLNSRLFVIRGQPADVFPRLFKEWNIAKLSIEYDSEPFGKERDAAIKKLASEAGVEVI
VRISHTLYDLDKIIELNGGQPPLTYKRFQTLISRMEPLEMPVETITPEVMEKCTTPVSDDHDEKYGVPSLEEL
GFDTDGLPSAVWPGGETEALTRLERHLERKAWVANFERPRMNANSLLASPTGLSPYLRFGCLSCRLFYFKLTD
LYKKVKKNSSPPLSLYGQLLWREFFYTAATNNPRFDKMEGNPICVQIPWDKNPEALAKWAEGRTGFPWIDAIM
TQLRQEGWIHHLARHAVACFLTRGDLWISWEEGMKVFEELLLDADWSVNAGSWMWLSCSSFFQQFFHCYCPVG
FGRRTDPNGDYIRRYLPILRGFPAKYIYDPWNAPESVQKAAKCIIGVNYPKPMVNHAEASRLNIERMKQIYQQ
LSRYRGLGLLATVPSNPNGNGNGGLMGYSPGESISGCGSTGGAQLGTGDGHTVVQPCALGDSHTGGSGIQQQG
YCQASSILHYAHGDNQQSHLLQAGRTALGTGISAGKRPNPEEETQSVGPKVQRQSTN
>Egretta_garz__KFP13269.1
DNWAFLYAQRLALKQELPLHVCFCLVPKFLDATIRHYGFMLRGLQEVAEECAELDIPFHLLLGYAKDVLPAFM
VEHGVGGLVTDFCPLRLPRQWVEDVRERLPEDVPFAQVDAHNIVPCWITSPKQEYSARTIRGKIHAQLPEFLT
EFPPVVRHPYPPSCQAQPIAWEACYSSLQVDRTVKEVEWATPGTAAGLAVLQSFIAERLKSFGSHRNDPNKAA
LSNLSPWFHFGQVSTQRAILEVQKHRGKYKESVDAFVEEAVVRRELAENFCYYNDNYDSVQGAYDWAQTTLKL
HAKDKRPFLYKLQELEQGTTHDPLWNAAQLQMVREGKMHGFLRMYWAKKILEWTRSPEEALQFAIYLNDRYEL
DGRDPNGYVGKRCLWSICGIHDQGWAERAVFGKIRYMNYAGCKRKFDVGQFERRYAP
>Egretta_garz__KFP23211.1
RFLLQSLEDLDNSLRKLNSRLFVVRGQPTDVFPRLFKEWGVTRLTFEYDSEPFGKERDAAIVKLAKEAGVEVV
IENSHTLYDLDRIIELNGHKPPLTYKRFQAIISRMELPKKPVSTVMSQQMESCKVDIQENHDDVYGVPSLEEL
GFPTDGLAPAVWQGGETEALARLDKHLERKAWVANYERPRMNANSLLASPTGLSPYLRFGCLSCRLFYYRLWE
LYKKVKRNSTPPLSLYGQLLWLGVFYTAATNNPKFDRMEGNPICIQIPWDRNPEALAKWAEGKTGFPWIDAIM

TQLRQEGWIHHLARHAVACFLTRGDLWISWESGVRVFDELLLDADFSVNAGSWMWLSCSAFFQQFFHCYCPVG
FGRRTDPSGDYVKRYLPKLKGFPSRYIYEPWNAPESVQKAAKCIIGVDYPKPMVNHAETSRLNIERMKQIYQQ
LSRYRGLCLLASVPSCAEDLSGPVTDSASGQGCSTSTSTVRLSQADQASPKRKHEGAEELCAEELYKRAKVTC
LPAPEIPGKS
>Egretta_garz__XP_009632356.1
MPHRTIHLFRKGLRLHDNPTLLAALESSEVVYPVYVLDRKFMTSVMHIGALRWHFLLQSLEDLQKNLCQLGSC
LLVIQGEYESVLRDHIQKWKITQVTLDAEMEPFYKEMEANIRRLGEELGFEVLSLVGHSLYDTKRVLDLNGGT
PPLTYKRFLHILSLLGDPEVPVRNLTAEDFQRCSPPDPGLAEHYGVPLPVDLKISPESLSPWRGGETEGLQRL
EQHLTDQGWVASFTKPRTIPNSLLPSTTGLSPYFSMGCLSVRTFFYRLSNIYAQAKHHSLPPVSLQGQLLWRE
FFYTVASATPNFTKMAGNPICLQISWYEDAERLHKWKTAQTGFPWIDAIMTQLRQEGWIHHLARHAVACFLTR
GDLWISWEEGMKVFEELLLDADYSINAGNWMWLSASAFFHQYTRIFCPVRFGKRTDPEGQYIRKYLPVLKNFP
SKYIYEPWTASDEEQKQAGCIIGQDYPFPMVNHKEASDHNLQLMKQVREEQSRTAQLTRDDADDPMEMKVKHG
HAEENGSKGKVARMTEQTEIPSGISPNGKRGEPEAS
>Egretta_garz__XP_035753346.1
MQHSSIHWFRKGLRLHDNPALLAAATDCRRLHPLFILDPSRGRAGANAQRFLLDALQDLDGSLREMGSRLFVV
RGCPEEVFPRLFHAWGTTRLTFEVDTEPSTRQRDAAVAKLAAQHGVEVIREVSHTLYDTQRVLALNDSKAPLT
YKRLQSLLAALGPPEKPAPALTQEHLQGCRTPCQVSHDTDYKVPTLEELGQDPAELGPHLYPGGETVALARLD
TFMERTAWVCGFKKPETEPNSLSPSTTVLSPYIKFGCLSVRTFWWRLDEVYQGRKHSQPPVSLHGQLLWREFF
YTAGASIPNFDRMVGNPVCLQVDWDDNPQHLRTWREGQTGYPFIDAIMTQLRTEGWIHHLARHAVACFLTRGD
LWISWEEGLKVFEELLLDADWSLNAGNWLWLSGSAFFHQYFRIYSPIAFGKKTDRDGAYIRKYLPILKDFPAE
YIYEPWKAPRAVQERAGCLVGTHYPQPIVEHGAASKRNLERMKAARAQKGKKPPQMHRVPPAALPPPWLPSSP
PGTKREPPAGPSTAESQRKKPKNEQR
>Egretta_garz__XP_035755535.1
DLRAHDNQVLHWAQGNADFVVPLYCFDPRHYVGTHCYGFPKTGPHRLRFLLESVKDLRETLKKKGSTLVVRKG
KPEDVVRDLITQLGSVSAVAFHEEATQEELDVEKGLCQVCSQHGVKIQTFWASTLYHRDDLPFRPIARLPDVY
THFRKAVESEAKVRPTVRMADQLKPLAPGVEEGCIPTMEDLGQKDPVTDPRTAFPCSGGETQALMRLQYYFWD
TNLVASYKETRNGLVGMDYSTKFAPWLALGCISPRYIYEQIQKYEKERTANQSTYWVLFELLWRDYFRFVALK
YGKRIFSLRGLQSKEVPWKKDLQLLDCWKEGKTGVPFVDANMRELAATGFMSNRGRQNVASFLTKDLGLDWRM
GAEWFEYLLVDYDVCSNYGNWLYSAGIGNDPRENRKFNMIKQGLDYDGNGDYVRLWVPELQGIKGADIHTPWA
LNSAALSQAGVTLGETYPQPVVTAPEWSRHLSQRPQGRSPHPRGRRGPAHTPMQHKDRGIDFYFSRKKDAR
>Eimeria_max_XP_013335797.1_DNA_photol
MSNLHWCLPDSGLPRGDGVLLTAGRLRASGTWLRGTVDSRRVRCVTTPLTEPKSSGESVVCVLQRDLRLQDNW
ALLFAQDAALSLEKPLHVLHLVAPGCSFQRTTRHLQFHLQGVQELAVGLTSHNIPFHCFPLLSAEASAAAAEA
TDRLKRALQQLNPRLVVCDLMPLRLPAAFVKELGFVCKEVMRCPLYQTDAHNVYAARTFRPRVMSLLEEFGCD
FPELQRHPHTSSALQGGIANILQQQPQVDEAVQLPPQWVPGAAGGFAALKAFCSQQRLAAYPSRNNPLLNTQS
GLSPWLHFGQIAAQRCLLAVRALGPATKIGAAAAAGRESFIEELVVRRELAENFTFYNGAYDKIEGAPRWAQE
TLEAHRGDKREYVYSLAQFEAAKTHDVLWNAAQLQLVHVGKMHGFLRMYWAKKILEWSPSAQEALSAALHLND
KFSLDGTDPNGIVGCMWSVAGVHDQGWAERPVFGKIRYMNLSGCRRKFPVDEFVQKVQASTRKLVKETHPKTR
NKQKEPTARVNATLALQPSPNKSTKKEKRQ
>Electrophorus_elec__XP_026872983.2
MVVHAVHWFRKGLRLHDNPALQEALAGADTARCVYVLDPWFAGAAKVGVNRWRFLLESLEDLDASLRKLNSRL
FVVQGQPADVFPRLFKEWNVTRLTFEYDSEPYGKDRDGAIIKMAQEFGVETIVRHSHTLYNPDRVLEMNGGNP
PLTFKRFQALVNRLELPKKPLPTVTAEQLAGCRTQLADDHDERYSVPSLEELGFKNPGKGASVWKGGETEALE
RLGKHLDRKAWVANFERPRIKAHSLFASATGLSPYLCFGCLSCRVFYHHLRELYLKLRRRSSPPPSLFGQLLW
REFFYTAATNNPNFDRMEGNPICVQIPWDHNPEALAKWAEGRTGFPWIDAIMTQLRQEGWVHHLARHAVACFL
TRGDLWISWEEGVKVFEELLLDADWSVNAGSWMWLSCSSFFQQFFHCYCPVGFGRRIDPNGDYIRRYVPQLKD
YPDRYVYEPWNAPEAVQRASGCVVGVDYPKPMINHAESTRLNIERMKQVYQQLSHYRGLSLLASVPTIQEEAE
PLLSDDSQAGSSALPTGSGCRSFVSLSTEGGDTPDSSRPARLTPVQRLQ
>Electrophorus_elec__XP_026888640.2
MVTNTVHWFRKGLRLHDNPSLRDSIVEADTLRCIYILDPWFAGSSSVGINRWRFLLQCLEDLDANLRKLNSRL
FVIRGQPTDVFPRLFKEWKITRLSYEYDSEPFGKERDAAIQKLAREAGVEVTVRISHTLYHLDKIIEMNGGQS
PLTYKRFQTLISRMDAVETPAETITADIMGRCTTPISDDHDDRFGVPSLEELGFDTEGLPSAVWPGGETEALT
RLERHLERKAWVANFERPRMNANSLLASPTGLSPYLRFGCLSCRIFYFKLTDLYRKVKKNSAPPLSLYGQLLW
REFFYTAATNNPRFDKMEGNPICVQMPWDRNPQALAKWAEGRTGFPWIDAIMTQLRQEGWIHHLARHAVACFL
TRGDLWVSWEEGVKVFEELLLDADWSVNAGSWMWLSCSSFFQQFFHCYCPVGFGRRTDPNGDYIRRYLPVLRA
FPAKYIYDPWNAPESVQKLAKCMIGVHYPKPMVNHAEASRINIERMKQIYQQLSCYRGLGLLASVPSNSSCNG
DGAGGGPGDSSQEVSGAAHLTPANPLEGMSAGILFSSHGDQQPGISGLPQTGGRVSPRQEPKSHQGGGSVPV
KRHSEDPPSSNGCKIQKRNTD
>Elephantulus_edwa__XP_006896803.1

MATTVVTATTGPLAPAPGTEGASSVHWFRKGLRLHDNPALLAAVRGARCVRCVYILDPWFAASSSVGINRWRF
LLQSLEDLDKSLRALNSRLFVVRGQPADVFPRLFKEWRVTRLTFEYDSEPFGKERDAAIMKMAKEAGVEVVTE
NSHTLYDLDRIIELNGQKPPLTYKRFQAIISRMELPKKPVGSVTSQQMEGCRAEIQENHDETFGVPSLEELGF
PTEGLGPAVWRGGETEALARLDKHLERKAWVANYERPRMNANSLLASPTGLSPYLRFGCLSCRLFYYRLWDLY
KKVKRNSTPPLSLFGQLLWREFFYTAATNNPRFDRMEGNPICIQIPWDRNPEALAKWAEGKTGFPWIDAIMTQ
LRQEGWIHHLARHAVACFLTRGDLWVSWESGVRVFDELLLDADFSVNAGSWMWLSCSAFFQQFFHCYCPVGFG
RRTDPSGDYIRRYLPKLKGFPSRYIYEPWNAPESIQKAAKCIIGVDYPRPIVNHAETSRLNIERMKQIYQQLS
RYRGLCLLASVPSCVEDLSNPVTEPSSSQAVSVTSAGQRQLPSGPASPKRKLETAEEPPGEEVSKRARVAELS
SPELPSNDV
>Elephantulus_edwa__XP_006897613.1
MGVNAVHWFRKGLRLHDNPALKECIQGADTIRCVYILDPWFAGSSNVGINRWRGFCVVIPFRVVSSNSRHPPA
LSGSRNRKLADPLEWDITKLSIEYDSEPFGKERDAAIKKLATEAGVEVIVRISHTLYDLDRIIELNGGQPPLT
YKRFQTLIRKMEPLEIPVETVTSEMVEKCTTPLSDDHDEKYGVPSLEELGFDTDGLPSAVWPGGETEALTRLE
RHLERKAWVANFERPRMNANSLLASPTGLSPYLRFGCLSCRLFYFKLTDLYKKVKKNSSPPLSLYGQLLWREF
FYTAATNNPRFDKMEGNPICVQIPWDKNPEALAKWAEGRTGFPWIDAIMTQLRQEGWIHHLARHAVACFLTRG
DLWISWEEGMKVFEELLLDADWSINAGSWMWLSCSSFFQQFFHCYCPVGFGRRTDPNGDYIRRYLPVLRGFPA
KYIYDPWNAPEGIQKVAKCLIGVNYPKPMVNHAEASRLNIERMKQIYQQLSRYRGLGLLASVPSNPNGNGGLM
GYSPGENIPGCSSSGSCSQGSGILHYAHGDSQQTHLLKQGRGSLGMGFSGGKRPSQEEEMQSIGPKVQRQSTN
>Elysia_chl_RUS74619.1_EGW08_0176
MFLSASGIFATQLSGNRLIGLCFTIPRAVFALKTLNWRKQTGVHKSFPLTSSISSVRIATKNSSVRKRPTVWQ
TRPSAFFTSSIMGEDNPSSSEKKTEKSSSKKEKVVKEEKVSKTKGKEEVKDEGPKTKSEKKERPARTVEKRKL
EKHQEEEATAKKSKIEDQNGATEPAEDVGNTDNEEFINKINQRRIDVCEDVSKFKFNKKRVRVISEEEDFCED
SNGVVYWMSRDQRVQDNWAFLYAQRLAMKLEVPLHVCFCLVPKYLDATIRMYGFMMKGLAQVEKECRELNIPF
HLLCGEASKSIPAFLKEHNIGGVVTDFSPLRVNVKWVDDLKNALPADVPLCQVDAHNLVPCWEASPKLEYGAR
TIRNKIHNQLGQFLTEFPPLVKHPHPPKKMPQITDWKKADGSLKVDRTVLEVAWAKPGTQAGYKMLQSFCEKR
LKHFATARNDPNKQALSNLSPWIHFGQISVQRCILTVRLYRSKSSDSVNAFIEEAVIRRELADNFCYYNKHYD
SVKGAYDWAKKSLALHKDDKRPYLYTRDELEESKTHDDLWNAAQYQLVSEGKMHGFLRMYWAKKILEWTTSPE
EALAFAIYLNDRFSLDGRDPNGYVGCMWSICGIHDQGWAERAVFGKIRYMNYQGCKRKFDVPGFVLRYKKKKL
EAKK
>Elysia_chl_RUS76899.1_EGW08_0153
MSQKCHRVIHWFRKGLRLHDNPALIEACKDASELYPVFVLDPWFVTNSNVGANRWRFLAQTLDDLDKQLKLLK
LRLFVVRGNPETELEKLCKKWKITKLTYEVDTEPYAVARDERVGKTMEDLGVSVIPCVSHTLYDVHSIVKING
GKAPLTYQSFQTAASRLGPPPQALPAPTLQDLPGCRNLASDAHGKEFEIPSLTELRVCESDLGPNLYPGGETE
ALDRMERHLKKVNWICSFEKPKTEPNSLLPSTTVLSPYLKFGCLSARLFYHKIINVYKGKKHSQPPVSLIGQV
LWREFYYTVAAVTPNFDKMEGNPVCKQIPWQENADHLNAWKQGRTGYPFIDAVMTQLRQEGWIHHLARHAVAC
FLTRGDLWVSWEEGQKEFEKLLLDADWSLNAGNWMWLSASAFFHQYFRVYSPVAFGKKTDPNGDFIRKYIPVL
KKFPAKFIFEPWKAPASMQQAAGCIIGKDYPKPIVDHDVVRQKNIQKMAAAYAKSKSSTSETNKNGHKELSSK
KRQSDDVGDGPSKKKKKS
>Elysia_chl_RUS77009.1_EGW08_0152
MNSNTDNNSNSEQSSATPTPSPTSKKHALFWFRKGLRLHDNPALIAAIHGAATYRCVYILDPWFAGASQVGIS
KWRFLLESLEDLDSSLRKLNSRLYVVRGQPADVLPRLFQEWSITTFAFEEDPEPYGKERDAALSALAREFSIQ
VIAKPSHTLYDPKNVIAANGNSPPLTYKRFQSILSTLPPPEKPCETLSALAITNGTTPVSHDHNDKYSVPSLD
ELGFDIDSLVPATFRGGESEALSRLNRHLERKAWVASFERPKMSPQSLYPSGTGLSPYLRFGCLSPRTFYWKL
TELYKRVKKGVEPPLALHGQLLWREFFYTVATNNPNFDRMVGNSICVQIPWEHNPEALAKWAEGMTGFPWIDA
IMVQLRREGWIHHLARHAVACFLTRGDLWISWEEGMKVFDEILLDADWSVNAGMWMWLSCSAFFQQFFHCYCP
VGFGKRADPSGDFVRHYLPVLKAMPTQYIYEPWTAPESVQKAAKCIVGKDYPLPMVQHSVVSQINLERMRQVY
KRLVLKSSARNRMFMGLPKMPKVDVILNKVKMFLSSSSDEEVSQKHRRRPSKHINDC
>Elysia_chl_RUS80278.1_EGW08_0119
MQVTRTLSPKAMAMNAKYGICLFRNDLRIHDNEVLNVANQKSDFLIPLYCFDPRHYKGTYHFGFPKTGKYRAS
FIIDSVVDLRSAIQSLGSKLIVRHGKPENVIQELIRNLNLKDVTVFLQEESMDEEKRVERAIQTKCNVAIESL
WGHTLYHVEDLPFQPKHLPDVYTEFRKRVENRAVIRKCFPTPSSLKPLPPDIDEGKMPTLADLELSEPEKNAQ
SAFPFPGGESSGLQRLHDYLWGTDSVSSYKETRNGMIGSDYSTKFSTWLAHGCISPRKIYWEIKKYEKERTSN
QSTYWVIFELIWRDYFRFVGLKFGNKLFYPGGIKGEDVRWRSDKEQFNAWKEGRTGVPYVDANMREMAATGFM
SNRGRQNVASFLTKDLKLDWRLGAEWFESLLLDHDVCSNYGNWLYSAGLGNDPRENRKFNVVKQGLDYDADGD
YVRLWVPELAAVKTGKVHCVWTLSPSVLQAAGVTLGETYPRPIVTAPEWSRHVNRPGSSGGGRGKGGASGQSR
GRGQGSSSHRGHGSNSQYPGQKRGIDFYFSGSS
>Elysia_chl_RUS86048.1_EGW08_0062
MDTESSVSGSSSCDNNGKEHVTVHWFRHGLRLHDNPALLEGLKDCREFYPIFILDGHVAGVSTAAFPRMQFLF
ETLTDLDNNLKQFGTRLYFLRGDPVEVFEHLFEEWGVTRLTFEQDPEPIWQDRDIKVKDLCQSRGVECIEKVS
HTLWDPHRIIEENGGHPPLTFAAFNQVAEIVGLPDKPVPNPDFQGIALPFIADHDQKYGIPSLSSLGVEPESK

EQASPCCRYLGGETKALKLLLSRLENEKRAFALDLSLPNQLYPDLVGMPMSLSPHLRFGSLSIRKLYWALRCS
YAQIHPNSPIPASITSQLVWREYFYCMSVNNPKYNCMEGNPICLKINWYNDEEKFKCWKEGRTGFPWIDACMR
QLVQEGWIHHVCRHAVSCFLTRGDLWIDWQKGLEVFDRYLLDADWSVCAGSWMWVSSSAFEKILQCPRCICPV
RYGRRMDPSGSYIRRYVPELKDMPMMYLCEPWKAPLKVQEEANCVIGVDYPAPMVDHKKAAQECKEKMERIKN
LCKGVPHVAPTNETEVLSYMWLGKNQDEGMQASLHSTCSHLSSISL
>Emiliania_huxle_XP_005763851.1
GGIVYWMGRDQRVQDNWALLHAAELAEAHSLPLSVVFCLPPPAAGSPSTGPGSTLREYGFMLKGLREVSTELA
ALRVPFCLLHGGSPPELLSQFCAQHEVAVVVADYSPLREPRGWKEALIAARPATPVLEVDAHNVVPVWVASDK
REVGARTIRKKITEKLPQWLVDIPALPPRAAADPQPPAGLAAAAAAMDWVALEAGLQLDRAVAETSPTHPSGA
AAALAAVDAFCDERLKLFADKRNDPNIAACSDLSPYLHFGQLSAQRMALCVKQHAKARSDSVASFLEESIVRR
ELSDNFCHYEPAYDSLDGAAGWARESLELHASDKREHVYSLEQLEGAQTHEDLWNAAQRQLAARGKMHGFMRM
YWAKKILEWTASPAEALRIGLYLNDKYSIDGRDPNGYVGVGWSVMGVHDMGWAERAVFGKIRYMNYNGCKRKF
DVKQYVAEWSGKKAAAGSTSAGAKSASAGRAKAGGKAMKQTTLGGGSTAAADDEPRAKAPPPKKPKLAIPI
>Emiliania_hux_XP_005763563.1_putative_C
MRTRFVWWVRNDLRLHDNPCLARILAHKGEAEARRSRDASESFHVLPVFLFDPRQYRRTARGCDKTGPFRAKF
LYDSVLDLRQQLRGIGSDLLVAPRGEGDTHTTILCQEQVTSEELRVDRALRSALPAGSCQFKPVWGGSLSELP
YRQDLSDLPDGFTPFRNKAGRPVESRCEVRAPIKRPGNGDLPLPPADALAAAAPLSCTALPPLPEIGLSEAAL
GEASRTLRRDAAPDPRGVMPFPGGEAAALARHYLAISLAAPWLKHYLWDSDALATYFETRNGMLGADYSSKFA
PWLAHGCLSPRQVAHECRRYEGARVQNKSTYWMGGPIGSHQSWRSDAQLLQRWKDGELGVPLVDANMRELKAT
GFMSNRGRQNVASYLALDLQLDWREGAEHFEALLLDYDVCSNWGNWVSAAGLTGGRVNRFNIVKQSKDYDTDG
AYVRHWLPELKDVPSQFVHEPWKMGRAEQERFGCRIGTHGDAASDYPNPPKISPLASYHGFRHDIMES
>Emiliania_hux_XP_005767880.1_putative_C
MVAVVYWFRKALRLHDNPALIAAAEGASSLYPVYCLDPKTCSRARGAWTNPNRMRFLLESLADLDASLRKLNS
RLIVLHGDPVEALPAACKEWSASRLAYELDYEQYSLRRDKAVRSQAEQAGLEVVTRVGHTLVDPEALLLLLSRG
APVTSYDKFRGLLAQELKARPVTTLPPPASLPPPGRTAAPHAIPSLEELGLEPMGDAAVIARGGEGAALRAME
AHLRRRDWIASFEKPKTSPTEMLAGGPFDAGAGRSTTVLSPYLTFGCLSSRLLYERVAAVYAEAAPRHSQPPV
SLHGQLLWREFYVCCMHATPNYRRMVGNPICRQISWGEDAALVAAWREGRTGYPWIDAAMTQLRREGWIHHLA
RHAVACFLTRGDLWQSWETGAAVFEEYLLDADEAINFGNWMWLSCSCFFYQYFRCYSPVAFPQKYDKQGAYVR
HWLPQLARLPAKYIYEPWKAPIAVQKEAGCIIGVDYPKPIVDHQAAAAATAAAPASARPLLAPPPPSPRRHPH
DPRPHAPATRSAGGVQREYGEDGRGVRGPQGGQGGRGARRRQWQAGCRGRQAGGGGWQAQARGERRRRKAEEA
LLVTAHPRASPQGSASICALWVALRGE
>Emiliania_hux_XP_005784167.1_photolyase
IVWLRAGDMRLTDNPALHAAAESGNPVIPVFIQPPRCEEGGWPLAGAAAYWLHHSLVALQHSLSRLGSAVVVR
RAADCGGSSAAALAALAAESGSRRVYYNASFEPWLVRRDEALEQELASQGVRAKRCAGNVLYHPEDARPDERT
AAHGFGSVGFFLAAVATLPPPPPPLPPPRRLKPPAAWPHSLPLSELGYGRLPVRPDGTVEDWAAGIRRSWAVG
EIGAQRALEAFVSGGIQRFEGRERFRADQANTAAISPHLRFGELSARAVLHSVRERASAPTFLRRLAWRDLAY
WALWRFPHLPSRPFRPWFEAQAWSSDQPALEAWQAGATGFPLVDAAMAQLWATGWMPNYMRHVVAGFLVEFLN
LDWRRFSLPVPRHGLRWFDYTLVDADTAINAYMWQNGGHSGMDQWNFVMHPVFAAKSCDPEGDYVRRWLPQLA
ALPVEYIHCPWEAPFALRAAAGVVLGGNYPKRILDDLEAARRASHAAVMAVR
>Engystomops_pust__KAG8563182.1
MSAAVSSCVFGHARECRVVRSRWLQNILCNMERVRMERTGLEAEVEAAMRQLLAGQVQADEFFCVLLSLLGEH
RVRALLPNIIQSLSSSSPALHSRLMSIHSQYYLQGPTDPYDEELEIALALSLQEASPCTAVPREDVCSRSMLS
FAEAAKKTVKKLNPEQKSSVYSKNCKPNDTEPRGALSEGVVGCHISDRKKHNVELAYNGQPEPEDCVLHNYED
VYPIESPATKPNKTKKNRRGRRKRNVPVPGSPVAMKPAIVWFRRDLRLYDNPALIAALELGVPVIPVFLWSMS
EETGHNFTLATGGATKFWLHHALYQLDQSIIQMYGSHIVLRVNSSCAEELVSLVLQSGAETVLVNALYEPWLK
ERDDLIAEILERKRVTFRKCHSYCLYEPYSVSTEGVGLRGIGSVSHFVSCCKKNASAAIGTPLQAPSNLPLPS
LWPGSVELDSLELAKMPRRKDGSEIDWAATIRKSWDFSEDGAYTCLGNFLEDGIKHYDKESGRADKPYTSHIS
PYLHFGQISPRTVLHEANFTKKSVPKFLRKLAWRDLAYWLLVLFPDMHVEPVRPAYRSQRWSSDKRHLRAWQK
GMTGYPLVDAAMRELWLTGWMCNYSRHVVASFLVAYLHLHWIHGYRWFQDTLVDADVAINAMMWQNGGMSGLD
HWNFVMHPVDAALTCDPNGSYVRQWCPELAGLPDEYIHKPWKCAPSQLKRAGTSAVCIFFINLFSASSSVDQ
>Engystomops_pust__KAG8564697.1
MEAAGTAAVSSVHWFRKGLRLHDNPALVAALRGARCVRCVYILDPWFAASSSGGVNRWRFLLQSLEDLDSSLR
KLGSRLFVVRGQPADVFPRLFKEWGVNRLTFEYDSEPFGKERDAAIMKLAKEVGVEVIVENSHTLYDLDKIIE
LNGNSPPLTYKRFQAIISRMELPRRPVPTITRQQMEYCRGEIKSTHDDTYGVPSLEELGFPCENQGVAVWPGG
ETEALARLDRHLERKAWVANYERPRMNANSLLASPTGLSPYLRFGCLSCRLFYYRLRELYQKVKKNSPPPLSL
FGQILWREFFYTAATNNPKFDQMEGNPICVQIPWDRNPEALAKWAEGKTGFPWIDAIMTQLRQEGWIHHLARH
AVACFLTRGDLWNSWECGVKVFDELLLDADFSVNAGSWMWLSCSAFFQQFFHCYCPVGFGRRTDPSGDYVRRY
LPVLKGFPSRYIYEPWNAPESVQKEAKCIIGVHYPKPIVNHAEASRLNIERMKQTYQQLSRYRGLCILASVPS
CAEDIGGPITDPTAAHHTSMEAAPKLSLCSPDSPKRKHEESAEGAFKKVRVQSIAEAERRAKDF
>Engystomops_pust__KAG8568157.1

MPHRSIHWFRKGLRLHDNPALLAAMKDCEELHPIFILDPWFPRNMKVSVNRWRFLIEALKDLDENLKMINSRL
FVVRGKPAEVFPQLFKKWKVTRLTFEVDTEPYSRQRDAEVGRIATEHNVEVIQKVSNTLYDVDRVIAENNGKP
PLTYVRLQTVLASIGPPKRPCSAPTKENMKDCRTPWKGSYEEIYGIPTLEELGQDPTKLGPHLYPGGESEALR
RLDLNMKRTAWVCNFKKPETEPNSLTPSTTVLSPYMKFGCLSTRTFYWRLAEIYHGKKHSSPPVSLHGQLLWR
EFFYTAGAGIPNFNKMEGNSVCVQVDWDENKDYLEAWREARTGYPFIDAIMTQLRTEGWIHHLARHAVACFLT
RGDLWISWEEGQKVFEELLLDADWSLNAGNWQWLSASAFFHQFFRVYSPVAFGKKTDKNGDYIKKYLPILKKF
PPEYIYEPWKSPRSVQERAGCIIGKDYPKPIVEHETVSKHNIQRMKAAYARRSGTSGEQAPKSPSNKKGVKRK
APSVADLFKKK
>Engystomops_pust__KAG8573280.1
MRMMAAPGRIVICLLRNDLRYHDNEVLLWAHRNADYIVPLYCFDPRHYVGTHYYNFPKTGPHRLKFLLESIKD
LRNTLKKIGSNLLLRRGHPENVIEDLIKQIGPVSAVALQEEATKEELDVEKSLKQVCTRHGVKYHTIWGSTLY
HREDLPFRHISGLPDIYTQFRKAVEAQCKVRPTLQMPEKLKPLPGGLEEGSVPTAEDFGEQDPLTDPRTAFPC
SGGESQALQRLQHYFWDTNLVASYKDTRNGLIGMDYSTKFAPWLALGCISPRYIFEQIRKYEKEQTANQSTYW
VIFELLWRDYFRFVALKYGRRIFFLRGLQDKEVPWKKDLKQFEAWKEGRTGVPFVDANMRELAMTGFMSNRGR
QNVASFLTKDLGLDWRMGAEWFEYLLVDYDVCSNYGNWLYSAGIGNDPRENRKFNMIKQGLDYDGNGDYIRLW
VPELQEIKGGDVHTPWALSNAALAHANVTLGDSYPFPIVMAPEWSRHVNQKPDSGAASSRRGRGPSHMPKQHK
NRGIDFYFSRNKNV
>Engystomops_pust__KAG8579692.1
MGVNAVHWFRKGLRLHDNPALMECIQGADTVRCVYILDPWFAGSSNVGINRWRFLLQCLEDLDANLRKLNSRL
FVIRGQPADVFPRLFKEWKISRLSIEYDSEPFGKERDAAIKKLASEAGVEVIVRISHTLYDLDKIIELNGGQP
PLTYKRFQTLISKMEPLETPVETITGEVMEKCTTPVLDDHDEKYGVPSLEELGFDTEGLPSAVWPGGETEALT
RLERHLERKAWVANFERPRMNANSLLASTTGLSPYLRFGCLSCRLFYFKLTDLYKKVKKNSSPPLSLYGQLLW
REFFYTAATNNPRFDKMEGNPICVQIPWDRNPEALAKWAEGRTGFPWIDAIMTQLRQEGWIHHLARHAVACFL
TRGDLWISWEEGMKVFEELLLDADWSVNAGSWMWLSCSSFFQQFFHCYCPVGFGRRTDPNGDYIRRYLPILKG
FPPKYIYDPWNAPEGVQKVAKCIIGVNYPKPMVNHAEASRLNIERMKQIYQQLSRYRGLGLLASVPSNPNGNG
NGGLMSYSPGDSMPGCSNNGGPQIGGGEGNSASNSNNQGESHSGAGGIQGYWQGSSILHYNHGDSQQSYLIQD
VGNRRVT
>Engystomops_pust__KAG8581634.1
MPKSKSESSGGEKSDTSQEDASKRKSITTAKRKSQENKGKSKIISDLSSSETSDSDAKKKKMQEKNDAEASGS
LSEAVKKSRLGAATSVSDFKFNKKRVRLVSPEADLKDDALGVVYWMSRDQRVEDNWAFLYAQRLALKQKLPLH
VVFCLVPKFLEATIRHFGFMLKGLQEVAEDCKGLNIPFHLLIGYAKDVLPNFVKEHGIGGVVTDFSPLRVPMQ
WVSDVCERLPKDVPLVQVDAHNIVPCWVASNKQEYGARTIRRKIHDQLSQFLTEFPPVTTHPYNSKLEAEPID
WDKCYASLEVDRTVKEVEWAKPGAKAGMDMLHSFITERLKFFNADRNNPNRQALSNLSPWFHFGQLSVQRAIL
EVQKYRSKYKESVDGFVEEAVVRRELADNFCYYNKNYDKVEGAYDWAKNTLKDHAKDKRTHLYTLQQLENGKT
HDPLWNAAQLQMVHEGKMHGFLRMYWAKKILEWTSSPEEALRFTIYLNDRFELDGRDPNGYVGCMWSICGIHD
QGWAERAVFGKIRYMNYQGCKRKFDVDQFERRYHPKKFAG
>Engystomops_pust__KAG8589654.1
MHIHIAELKGFASSLHVWRTCTHSTEDLQELTTFTMPHRTIHIFRKGLRLHDNPTLLAALESSDVIFPLYILD
KNFITSNTNVGSNRWNFLLQSIEDLHCNLQKLNSCLFVIKGDYKQVLRDYVQKWNITQVTVDHEIEPYYRDMD
ENITAMGQELGFQFLSLVSHTLYDIRRIITENGGRAPLTYKRFLHILSRLGDPEPPSREITEEHFMKCTTPAD
AFVTEEHRVPLPEDLDVPRSKSTNWIGGESQGLRRLEQHLEKQGWVSQFKKPQTIPNSLLPSTTGLSPYFSVG
CLSVRVFFQKLSTIYAQAKNHSLPPVSLQGQLLWREFFYTVASYTPNFTKMAGNPICLQIDWYKDEDKLNKWT
KAQTGFPWIDAIMTQLREEGWIHHLARHAVACFLTRGDLWISWEEGMKVFEELLLDADYSINAGNWMWLSASA
FFHHYTRIFCPVRFGKRTDPDGNYIRKYLPVLKDFPAKYIYDPWTAPEDIQKQAKCIIGQDYPFPMVDHKVVS
EHNLQLMKLVREQQHKTADLTQDAADDPMEMDIKEYFRKDKEPNSMENPNKKRRCAEDMNVEQVKTHELCKVK
VC
>Eolophus_rose__NXD62675.1
RFLLQSLEDLDNSLRKLNSRLFVVRGQPTDVFPRLFKEWGVTRLTFEYDSEPFGKERDAAIIKLAKEAGVEVV
IENSHTLYDLDRIIELNGHKPPLTYKRFQAIISRMELPKKPVSTVISQQMETCKVDIQENHDDVYGVPSLEEL
GFPTDGLAPAVWQGGETEALARLDKHLERKAWVANYERPRMNANSLLASPTGLSPYLRFGCLSCRLFYYRLWE
LYKKVKRNSTPPLSLYGQLLWREFFYTAATNNPKFDRMEGNPICIQIPWDRNPEALAKWAEGKTGFPWIDAIM
TQLRQEGWIHHLARHAVACFLTRGDLWISWESGVRVFDELLLDADFSVNAGSWMWLSCSAFFQQFFHCYCPVG
FGRRTDPSGDYVKRYLPKLKGFPSRYIYEPWNAPESVQKAAKCIIGVDYPKPMVNHAETSRLNIERMKQIYQQ
LSRYRGLCLLASVPSCVEDLSGPVTDSASGQGCSTSTAVRLSQADQPSPKRKHEGAEELCTEELFKRAKVTGL
PAPEVPGKSL
>Eolophus_rose__NXD65032.1
KVDYDVCSNYGNWLYSAGVGNDPRDNRKFNMVKQGLDYDGNGDYVRLWVPELQGIKGADIHTPWALSSAALSQ
VGVTLGETYPQPIVTTPEWSRHISQRPVSTGKEISRHPRGRRGPAHLPVQHKDRGIDFYFSRKKDA
>Eolophus_rose__NXD71355.1

DNWALLYAQRLALKQELPLYVCFCLVPKFLEATIRHYGFMLRGLQEVAEECAELNIAFHLLRGYAKDVLPAFV
VEHGVGGLVTDFSPLRLPWQWVQDVRERLPEDVPFAQVDAHNIVPCWIASPKQEYSASTIRGKIHAQLPEFLT
EFPPVVRHPYPPSCPAEPIAWEACYSSLEVDRTVKEVEWATPGTAAGLAVLQSFIKKRLKYFGSHRNNPNKAA
LSNLSPWFHFGQVSTQRAILEVQKHQHKYKESVDMFVEEAVVRRELAENFCYYNENYDSVQGAYEWAQTTLKL
HAKDERPFLYKLQELEQGTTHDPLWNAAQLQMVREGKMHGFLRMYWAKKILEWTRSPEEALQFAIYLNDRYEL
DGRDPNGYVGKGNADALSPHPAGCLWSICGIHDHGWTERAVFGKIRYMNYAGCKRKFDVGQFECRYPPAL
>Ephemera_dan_KAF4518708.1_(6-4)-phot
MSSSGTVIHWFRKGLRLHDNPALLSAIEKIDDKCFELRPIFILDPWIVTKLRVGTNRWRFLQQSLQDLDSKLR
AIGSRLYVLRGKPENVFPKIFKDWKVKRLTFELDTEPYARERDARIEKLAKEAGVPVAQKVSHTLYDTERVIM
TNMGKPPLTYQKMVSLAEQMGRPLLPKSAPESLPDECKVGAAGLPVNETDWPNTDVPSLEELGVDPAKLQPCL
YPGGETEALRRMEEHLAKKAWICKFEKPETSPNSLKPSTTVLSPYIKFGCLSARTFYHGSAHSKPPVSLVGQM
LWREFFYTVSAATPNFDRMQGNPICCQIPWGSNPTHLKAWTEGQTGFPFIDAVMRQLRQEGWIHHLARHSVAC
FLTRGDLWISWEEGMKVFEEFLLDADWALNAGNWMWLSASAFFHQYFRVYSPIAFGKKTDKDGSFIRKYVPEL
AKYPAEYIYEPWKSSLSVQRAAGCIIGQDYPRPIVNHDEASKRNKSRMAEAYKKNKQNKTDEGSSNAKKRGAG
NDRGFAKKFKQ
>Ephemera_dan_KAF4519522.1_B566_EDAN0
MASPEKRVKVGDNTKSPGSTSTSNVGDFIANIENEREKAASSILEFKFNKKRVRILSKASDVAVNASGIVYWM
SRDQRVQDNWALLFAQKLAMKNKIALHVCFCLLPKFLDATIRHFKFMLDGLVEVQTECSKLGLQFHLLQGDAA
SVLPSFVKTHKIGALVADFSPLRTHLDWTESVKKTLPSDVDAHNLVPCWVASDKLEYGARTIRNKITSKLPEF
LTQFPPVVKHPYPASFKAEPIDWSELESSLEVDTSVGPVTWAAPGTQAGLAVLHEFCQKRLKNFGTKRNDPTI
NVLSNLSPWFHFGQISVQRAILTVNKFKSKYAESVAAFVEESVVRRELSDNFCFYNPKYDSIDGTNAWAKTTL
IQHKKDKRPYLYTQEELDSAHTHDDLWNSAQIQLVKEGKMHGFLRMYWAKKILEWTVSPEEALRIAIYLNDRY
SLDGRDPNGYVGCMWSICGIHDQGWAEREVFGKIRYMNYAGCKRKFDVSAFVARYGGKVHFNKSNGQQTLKVT
KGKSK
>Ephemera_dan_KAF4522540.1_Chryptochr
MAGMNKLPRISVLWFRHGLRLHDNPALLDAIKNSDEFYPIFIFDGESAGTRLVGYNRMRFLLEALADLDRQLR
AVGGQLYLLQGSPSRLFQRLWEERSLTKLCFEQDCEPIWHARDAKVRATCIDRCIHCNEHISHTLWDPKEVIR
TNGGSPPLTYQLFLHTVSVIGDPPRPVADPDWSNVKFGKLPEIITQEFKMFACVPSPEHFGLTPPATSPVVRW
VGGETQALKHLQERLQVEEAAFMRGVILPNRASPDLLAPPTSQSAALRFGCLSVRREGWMHHVARNSVACFLT
RGALWQSWEHGLQHFLKHLIDADWSVCAGNWMWVSSSSFEQLLDCSQYVCPVNYGRRRYVPELRRYPVEYLYE
PWKAPLETQQNAGCIIGEDYPERIVDHTKALKQNRKYMQKLRDEMMEATPDHCCPSNEDEVLSFMQLPEACFE
ELNMSQA
>Ephemera_dan_KAF4523712.1_cry_2
MNCVLNKDNTTSSNTVSEKHTVHWFRKGLRLHDNPSLHEGLKDATSLRCVFILDPWFAGSSNVGINKWRFLLQ
CLEDLDRSLRKLNSRLFVIRGQPADALPKLFKEWGTTCLTFEEDPEPFGRVRDQNIMTLCRELGLSVISRISH
TLYDLEKILEKNGGKAPLTYYQFQNVVASMDPPPEPEHTVDSFGDTNTPVSDDHDEKYGVPSLEELGFVVDGL
LPPVWRGGESEALARLERHLERKAWVASFGRPKMTPQSLLASQTGLSPYMRFGCLSTRLFYYQLTDLYKKIKK
TVPPLSLHGQLLWREFFYCAATKNPNFDKMVGNPICVQIPWDKNPEALAKWANGQTGFPWIDAIMTQLREEGW
IHHLARHAVACFLTRGDLWISWEEGMKVFEELLLDADWSVNGGMWMWLSCSSFFQQFFHCYCPVRFGRKADPN
GDFIRQVRRYLPVLKKFPTRYIHEPWNAPESVQKAAKCVIGKDYGMPMVNHAVASRINIERMKQVYQQLSKYH
GAGLLETVPSSQMNGINLYENITLTTNSSDTTVLYKSNVNRQKSKQKRHH
>Eptesicus_fusc__XP_028005951.1
MVVNAVHWFRKGLRLHDNPALKECIRGADTIRCVYILDPWFAGSSNVGINRWRFLLQCLEDLDSNLRKLNSRL
FVIRGQPADVFPRLFKEWNITKLSIEYDSEPFGKERDAAIKKLATEAGVEVIVQISHTLYDLDKIIELNGGQP
PLTYKRFQTLISKMEPLEIPVETITLEVIEKCTTPLSDDHDEKYGVPSLEELGFDTDGLPSAVWPGGETEALT
RLERHLERKAWVANFERPRMNANSLLASPTGLSPYLRFGCLSCRLFYFKLTDLYKKVKKNSSPPLSLYGQLLW
REFFYTAATNNPRFDKMEGNPICVQIPWDKNPEALAKWAEGRTGFPWIDAIMTQLHQEGWIHHLARHAVACFV
TRGDLWISWEEGMKVFEELLLDADWSINAGSWMWLSCSSFFQQFFHCYCPVGFGRRADPNGDYIRRYLPVLRG
FPAKYIYDPWNAPEGIQKVAKCLIGVNYPKPMVNHAEASRLNIERMKQIYQQLSRYRGLGLLASVPSNPNGNG
GLMGYSTGENIPGCSSSGRRSSMGTGLGSGKRPSQEEDTQSIGHKVQRQSTN
>Eptesicus_fusc__XP_028009374.1
MAATAVTAAAAAAPAPAAGTEGASSVHWFRKGLRLHDNPALLAAVRGARCVRCVYILDPWFAASSSVGINRWR
FLLQSLEDLDTSLRKLNSRLFVVRGQPADVFPRLFKEWGVTRLTFEYDSEPFGKERDATIMKMAKEAGVEVVT
ENSHTLYDLDRIIELNGQKPPLTYKRFQAIISRMELPKKPVASLTRHQMESCPAEIQENHDETYGVPSLEELG
FPTEGLGPAVWQGGETEALARLDKHLERKAWVANYERPRMNANSLLASPTGLSPYLRFGCLSCRLFYYRLWDL
YKKVKRNSTPPLSLFGQLLWREFFYTAATNNPRFDRMEGNPICIQIPWDRNPEALAKWAEGKTGFPWIDAIMT
QLRQEGWIHHLARHAVACFLTRGDLWISWESGVRVFDELLLDADFSVNAGSWMWLSCSAFFQQFFHCYCPVGF
GRRTDPSGDYIRRYLPKLKGFPSRYIYEPWNAPESIQKAAKCIIGVDYPRPIVNHAETSRLNIERMKQIYQQL
SRYRGLCLLASVPSCMEDLSNPVAEPSSSRTGSMSSAAAALWLREQSLGARTATMAAESNRQKS
>Equus_asin__XP_014707833.1

MGVNAVHWFRKGLRLHDNPALKECIQGADTIRCVYILDPWFAGSSNVGINRWRFLLQCLEDLDANLRKLNSRL
FVIRGQPADVFPRLFKEWNITKLSIEYDSEPFGKERDAAIKKLATEAGVEVIVRISHTLYDLDKIIELNGGQP
PLTYKRFQTLISKMEPLEIPVETITSEVIEKCTTPLSDDHDEKYGVPSLEELGFDTDGLPSAVWPGGETEALT
RLERHLERKAWVANFERPRMNANSLLASPAGLSPYLRFGCLSCRLFYFRLTDLYRKVKKNSSPPLSLYGQLLW
REFFYTAATNNPRFDKMEGNPICVQIPWDKNPEALAKWAEGRTGFPWIDAIMTQLRQEGWIHHLARHAVACFL
TRGDLWISWEEGMKVFEELLLDADWSINAGSWMWLSCSSFFQQFFHCYCPVGFGRRTDPNGDYIRRYLPVLRG
FPAKYIYDPWNAPEGIQKVAKCLIGVNYPKPMVNHAEASRLNIERMKQIYQQLSRYRGLGLLASVPSNPNGNG
GLMGYSPGENIPGCSSSGSCSQGSGILHYAHGDSQQTHLLKQGRSSLGPGLSSGKRPGPEDDTQGIGPKVQRQ
STT
>Equus_asin__XP_014716667.2
MAAAAAAAVTVAAAAPAAAAGTEGASSVHWFRKGLRLHDNPALLAAVRGARCVRCVYILDPWFAASSSVGINR
WRFLLQSLEDLDTSLRKLNSRLFVVRGQPADVFPRLFKEWGVTRLTFEYDSEPFGKERDAAIMKMAKEAGVEV
VTENSHTLYDLDRIIELNGQKPPLTYKRFQAIISRMELPRKPVGSVTSQQMESCRADIQENHDETYGVPSLEE
LGFPTEGLGPAVWQGGEMEALARLDKHLERKAWVANYERPRMNAASLLASPTGLSPYLRFGCLSCRLFYYRLW
DLYRKVKRNSTPPLSLFGQLLWREFFYTAATNNPRFDRMEGNPICIQIPWDRNPEALAKWAEAKTGFPWIDAI
MTQLRQEGWIHHLARHAVACFLTRGDLWVSWESGVRVFDELLLDADFSVNAGSWMWLSCSAFFQQFFHCYCPV
GFGRRTDPSGDYIRRYLPKLKAFPSRYIYEPWNAPEAVQKAAKCIIGVDYPRPIVNHAETSRLNIERMKQIYQ
QLSRYRGLCLLASVPSYVEDLSNPVAEPSSSQAGSVSSAGPRPPPSGPASPKRKLEAAEEPPGEELSKRARVA
EPPSRAV
>Equus_caba__XP_005606624.1
MGVNAVHWFRKGLRLHDNPALKECIQGADTIRCVYILDPWFAGSSNVGINRWRFLLQCLEDLDANLRKLNSRL
FVIRGQPADVFPRLFKEWNITKLSIEYDSEPFGKERDAAIKKLATEAGVEVIVRISHTLYDLDKIIELNGGQP
PLTYKRFQTLISKMEPLEIPVETITSEVIEKCTTPLSDDHDEKYGVPSLEELGFDTDGLPSAVWPGGETEALT
RLERHLERKAWVANFERPRMNANSLLASPAGLSPYLRFGCLSCRLFYFRLTDLYRKVKKNSSPPLSLYGQLLW
REFFYTAATNNPRFDKMEGNPICVQIPWDKNPEALAKWAEGRTGFPWIDAIMTQLRQEGWIHHLARHAVACFL
TRGDLWISWEEGMKVFEELLLDADWSINAGSWMWLSCSSFFQQFFHCYCPVGFGRRTDPNGDYIRRYLPVLRG
FPAKYIYDPWNAPEGIQKVAKCLIGVNYPKPMVNHAEASRLNIERMKQIYQQLSRYRGLGLLASVPSNPNGNG
GLMGYSPGENIPGCSSSGRRSSLGPGLSSGKRPGPEEDTQGIGPKVQRQSTT
>Equus_caba__XP_023509705.1
MAAAAAVTAAAAAPAAAAAAAGAEGASSVHWFRKGLRLHDNPALLAAVRGARCVRCVYILDPWFAASSSVGIN
RWRFLLQSLEDLDTSLRKLNSRLFVVRGQPADVFPRLFKEWGVTRLTFEYDSEPFGKERDAAIMKMAKEAGVE
VVTENSHTLYDLDRIIELNGQKPPLTYKRFQAIISRMELPRKPVGSVTSQQMESCRADIQENHDETYGVPSLE
ELGFPTEGLGPAVWQGGETEALARLDKHLERKAWVANYERPRMNAASLLASPTGLSPYLRFGCLSCRLFYYRL
WDLYRKVKRNSTPPLSLFGQLLWREFFYTAATNNPRFDRMEGNPICIQIPWDRNPEALAKWAEAKTGFPWIDA
IMTQLRQEGWIHHLARHAVACFLTRGDLWVSWESGVRVFDELLLDADFSVNAGSWMWLSCSAFFQQFFHCYCP
VGFGRRTDPSGDYIRRYLPKLKAFPSRYIYEPWNAPEAVQKAAKCIIGVDYPRPIVNHAETSRLNIERMKQIY
QQLSRYRGLCLLASVPSCVEDLSNPVAEPSSSQAGSVSSAGPRPPPSGPASPKRKLEAAEEPPGEELSKRARV
AEPPSRAV
>Erinaceus_europaeus_XP_007519157.1_PREDICTED:
MAAVAVTAAATAAAAGPGPVADGASSVHWFRKGLRLHDNPALLAAVRGARSVRCVYILDPWFAASSSVGINRW
RFLLQSLEDLDTSLRKLNSRLFVVRGQPADVFPRLFKEWGVTRLTFEYDSEPFGKERDTAIMKMAKEAGVEVV
TENSHTLYDLDRIIELNGQKPPLTYKRFQAIISRMELPRKPVGSVTSQQMESCRAEIQENHDETYGVPSLEEL
GFPTEGLGPAVWQGGETEALARLDKHLERKAWVANYERPRMNANSLLASPTGLSPYLRFGCLSCRLFYYRLWD
LYKKVKRNSTPPLSLFGQLLWREFFYTAATNNPRFDRMEGNPICIQIPWDRNPEALAKWAEGKTGFPWIDAIM
TQLRQEGWIHHLARHAVACFLTRGDLWVSWESGVRVFDELLLDADFSVNAGSWMWLSCSAFFQQFFHCYCPVG
FGRRTDPSGDYIRRYLPKLKGFPSRYIYEPWNAPESVQKAAKCIIGVDYPRPIVNHAETSRLNIERMKQIYQQ
LSRYRGLCLLASVPSCVEDLSNPMAEPSSSQAGSASSAGPRSLTSGPASPKRKLEAAEEPPGEELSKRARVAE
LSIPEPPSKDV
>Erinaceus_eur_XP_016040514.1_cry-1
MGVNAVHWFRKGLRLHDNPALRECIQGADTVRCVYILDPWFAGSSNVGINRWRFLLQCLEDLDANLRKLNSRL
FVIRGQPADVFPRLFKEWNITKLSIEYDSEPFGKERDAAIKKLATEAGVEVIIRISHTLYDLDKRNTTSQLHL
LDAPVVPLRMLPCGAGIWTQGLSHKTSALSSFDTDGLPSAVWPGGETEALTRLERHLERKAWVANFERPRMNA
NSLLASPTGLSPYLRFGCLSCRLFYFKLTDLYKKVKKNSSPPLSLYGQLLWREFFYTAATNNPRFDKMEGNPI
CVQIPWDKNPEALAKWAEGRTGFPWIDAIMTQLRQEGWIHHLARHAVACFLTRGDLWISWEEGMKVFEELLLD
ADWSINAGSWMWLSCSSFFQQFFHCYCPVGFGRRTDPNGDYIRRYLPVLRGFPAKYIYDPWNAPEGIQKVAKC
LIGVNYPKPMVNHAEASRLNIERMKQIYQQLSRYRGLGLLASVPSNPNGNGGLMAYSPGENIPVCSSSGSCSQ
GSGILHYAHGDSQQTHLLKQGRSSVGTGFSNGKRPSQEEDMQSPKVQRQCTN
>Erpetoichthys_calabaricus_XP_028649658.1_cryptochro
MVVNSIHWFRKGLRLHDNPALLAALNGADTVRCVYILDPWFAGSTNVGVNRWRFLLESLEDLDTSLRKLGSRL
FVIRGQPADVFPRLFKEWNITRLTFEYDSEPFGKERDAAIIKFAEELGVETVVKNSHTLYNLDRIIELNNNSP

PLTYKRFQGIISRLEPPKKPVASLTKEQMQKCTTDIPETPYECFRVPSLDELGFKTATLSPSIWKGGETEALL
RLEKHLERKAWVASFERPRINVNSLIASPTGLSPYLRFGCLSCRTFYYTLLELYKKVKGNRTPPLSLFGQLLW
REFFYTAATNNPKFDRMEGNPICVQIPWDKNPKALAKWAEGQTGYPWIDAIMIQLKQEGWIHHLARHAVACFL
TRGDLWISWECGMKVFDELLLDADFSVNAGSWMWLSCSAFFQQFFHCYCPVGFGRRTDPSGNFIRHYIPKLKD
FPNRYIYEPWNAPDTVQKAARCIIGIDYPKPMISHAEASRLNIERMKQIYQHLSHYKGLSLLASVPTVPEEID
SLALEDCHKHSSMPGKVSPLGKRDRPPDLEIDEKCTDPKVSCSHLMKGNFEQ
>Erpetoichthys_calabaricus_XP_028671785.1_cryptochro
MVVNTVHWFRKGLRLHDNPSLWDSLKGADSVRCVYILDPWFAGSSNVGISRWRFLLQCLEDLDANLRKLNSRL
FVIRGQPADVFPRLFKEWNISRLSFEYDSEPFGKERDAAIKKLASEAGVEVIVRISHTLYDLDKIIELNGGQP
PLTYKRFQTLISRMEPLETPVESITADVMGKCTTPVSDDHDEKFGVPSLEELGFDTEGLPSAVWPGGETEALT
RLERHLERKAWVANFERPRMNANSLLASPTGLSPYLRFGCLSCRLFYFKLTDLYKKVKKNSSPPLSLYGQLLW
REFFYTAATNNPRFDKMEGNPICVQIPWDKNPEALAKWAEGRTGFPWIDAIMTQLRQEGWIHHLARHAVACFL
TRGDLWISWEEGMKVFEELLLDADWSVNAGSWMWLSCSSFFQQFFHCYCPVGFGRRTDPNGDYIRRYLPVLRG
FPAKYIYDPWNAPESIQKAAKCVIGLHYPKPMVNHAEASRLNIERMKQIYQQLSCYRGLGLLASVPSNPNGNS
SGGVMGLSTNEIIQGTHSSVGTVMGTGENQVGGGLTFQHGDSHPVLHHEGYGSEILLYSQRGSQQTESVILQP
VGRGTAVSTLTTVKRPNPEETTQGIGRKVQRQSSN
>Erpetoichthys_cal_XP_028650777.1_photolyase
MSNSTTFRCCLQSCWKVQSLPPTTLYTFAAWLYCATSKIIEMQRKSSTKYSVKQLKAKKSKLLEDDHHDDYAC
KRQKLPEVTGGGKRLSDAICISRRETASSVAEFKFNKKRVRILSETEDIVDGSDGILYWMSRDQRVHDNWALL
FAQRLALKQKQPLHVCFCLVPRFLEATIRHFGFMLCGLQEVEKECTELDISFHLLIGYAKDVLPEFVKSNHIG
AVVTDFSPLRVPLQWLQDVKDRLPEGIPFVQVDAHNIVPCWVVSDKQEYAARTIRKKIHDKLPEFLTEFPPVV
LHPHCTKIKTKEVDWEKARASLEVDHTVKEVDWAKPGTLGGLAVLESFISERLKIYSSDRNDPNKEALSNLSP
WFHFGQLAVQRAVLEVQKFRSKYRESVESFIEESVVRRELSDNFCFYNPRYDSLDGAFEWARKTLEDHTKDKR
THLYSRAELEEGRTHDPLWNAAQLQMVHEGKMHGFLRMYWAKKILEWTHSPAEALEISIYLNDRYELDGRDPN
GYVGCMWSICGTHDQGWSERAVFGKIRYMNYAGCKRKFDVNQFEYRYNHKKFTP
>Erpetoichthys_cal_XP_028659921.1
MCAARTVICLLRNDLRFNDNEILYWAQANAEYVVPLYCFDPRHYLGTHTFNFPKTGPFRLKFLLESVKDLRET
MQKKGSNLVVRKGKPEEVIQELIQKLKFVTAVTFQDEVTMEEKVVEYGVKQVCSQLNIKVQTFWGSSLFHRDD
LPFHHISQLPDVFTYFRKAVESQGIVRPTFAMPSQMKPLPEGVEEGDIPSHETFGQICPLLDPRSAFPCEGGE
TKALERLMHYFWDKNLIATYKETRNGLIGIDYSTKFAPWLALGCISPRYIYEQVKKYEKERMANQSTYWVIFE
LLWRDYFRFVALKYGRRIFFPKGLQDKAVAWKKDGKLFDTWKEGKTGVPFVDANMRELALTGFMSNRGRQNVA
SFLTKDLGLDWRMGAEWFEYLLTDYDVCSNYGNWLYSAGIGNDPRENRKFNMIKQGLDYDGDGEYVRLWVPEL
KEIKGGDIHTPWALSSASLSHFNVHLNETYPSPIIVQPEWSRHTNQKPANSSGHRRGRGGSHNLPKQHKDRGI
DFYFSGNKHSE
>Erpetoichthys_cal_XP_028666189.1_cry-1-like
MKHNSIHWFRKGLRLHDNPALLAALKDCAEMRPIFILDPWFPKNMKVGINRWRFLIESLKDLDCSLRKMNSRL
FIVRGKPVEVFPRLFKEWRVTRLTFEIDTEPYAQLRDSQVAEIAKEHGVEVIRKVSNTLYDTERIILENNGKP
PLTYVKLQDLLSRLGPPKKPAPSLKGEDLKGIHTPYTDYHDTEFGIPTLEELGLNPAATGPHLFPGGETEAIR
RLGEYMQKSVWVCNFKKPETEPNSLTPSTTVLSPYVKFGCLSVRTFWWKIAEVYHGKKHSSPPVSLHGQLLWR
EFFYTAGVGIPNFDHMEGNSVCLQVDWDSNEEYLAAWREGRTGYPFIDAIMTQLRTEGWIHHLARHAVACFLT
RGDLWISWEEGQKVFEEFLLDADWSLNAGNWQWLSASAFFYQFYRIYSPIAFGKKTDKNGDYIRKYIPILRKF
PVDYIYEPWKAPRSIQERAGCIIGKDYPKPIVDHDTVSKKNIQKMKAAYTRRSATSSKSGENCLKGPKKRSLS
MANVFTKKISQKTC
>Escherichia_col_WP_001583322.1_photolyase
MTTHLVWFRQDLRLHDNLALAAACRNSSARVLALYIATPRQWATHNMSPRQAELINAQLNGLQIALAEKGIPL
LFREVDDFVASVEIVKQVCAENSVTHLFYNYQYEVNERARDVEVERALRNVVCEGFDDSVILPPGAVMTGNHE
MYKVFTPFKNAWLKRLREGMPECVAAPKVRSSGSIEPSPSITLNYPRQSFDTAHFPVEEKAAIAQLRQFCQNG
AGEYEQQRDFPAVEGTSRLSASLATGGLSPRQCLHRLLAEQPQALDGGAGSVWLSELIWREFYRHLMTYYPSL
CKHCPFIAWTDRVQWQSNPAHLQAWQEGKTGYPIVDAAMRQLNSTGWMHNRLRMISASFLVKDLLIDWREGER
YFMSQLIDGDLAANNGGWQWAASTGTDAAPYFRIFNPTTQGEKFDREGEFIRRWLPELRDVPGKAVHEPWKWA
QKAGVKLDYPQPIVDHKEARLRTLAAYEEARKGA
>Esox_lucius_XP_010881675.1_cryptochro
MVVNTIHWFRKGLRLHDNPSLKESIRGADTLRCVYILDPWFAGSSNVGISRWRFLLQCLEDLDASLRKLNSRL
FVIRGQPTDVFPRLFKEWQISRLSYEYDSEPFGKERDAAIRKLASEAGVEVTVKVSHTLYDLDKIIELNGGQS
PLTYKRFQTLISHMDLVEMPAETITAEVMRKCATPLSDDHDDKFGVPSLEELGFETEGLVTAVWPGGETEALT
RLERHLERKAWVANFERPRMNANSLLASPTGLSPYLRFGCLSCRLFYFKLTDLYRKVKRNGSPPLSLYGQLLW
REFFYTTATNNPRFDKMEGNPVCVQIPWDRNPEALAKWAEGRTGFPWIDAIMTQLRQEGWIHHLARHAVACFL
TRGDLWISWEEGMKVFEELLLDADWSVNAGSWMWLSCSSFFQQFFHCYCPVGFGRRTDPNGDYIRRYLPILKG
LPAKYIYDPWNAPEGVQKAAKCEIGVHYPKPMVNHAEASCLNIERMKQIYQQLSCYRGLGLLATVPANSNNGG
NSAGGIPVPVPGPSPEEIHNDGAVQAGRGQFAVKRRNEDLIPGVSSKARRQTSN

>Esox_lucius_XP_019896110.1_cryptochro
MVVNSVHWFRKGLRLHDNPALQEALNGADTVRCVYILDPWFAGSANVGINRWRFLLESLEDLDSSLRKLNSRL
FVVRGQPANVFPSLFKEWSVTRLTFEYDSEPYGKERDGAIIKMAQEFGVETIVRNSHTLYNLDRLIEMNSGSP
PLTFKRFQALVNVLELPAKPLPTVTKEQMDSCRTKISGNHDQHYSVPSLEELGFKTQGLGPAVWKGGESEALE
RLNKHLDRKAWVANFERPRVSMCSLMASPTGLSPYLRFGCLSCRVFYYNLRELYMKLRKHCSPPLSLFGQLLW
REFFYTAGTNNPNFDRMEGNPICVQIPWDHNPEALAKWAEGRTGFPWIDAIMTQLRQEGWIHHLARHAVACFL
TRGDLWISWESGMRVFEELLLDADWSVNAGSWMWLSCSAFFQQFFHCYCPVGFGRRTDPSGDYIRRYIPKLKD
YPDRYIYEPWRAPESVQKAAHCIVGVDYPKPMINHAEGSRLNIERMKQVYQQLSHYRGLSLLASVPTIQEEAE
PPLTDDSQASGSTCESPPHTSSAQPNTLKRGRPSDSPGAPKATQSKHQHTTAPREEQEPDQGEWDEQQHLAEP
MDSQ
>Esox_luc_XP_010900290.2_photolyase
MHRSWWLLRTSHHVWKCAAIESRIPYHNLNKLIGLSSINPRTNMPAEKRKAPSNKMGNAKKQKLQVAKEGMGE
SEEVNEHQNQDLKFNKKRMRFLSQTEKMKQGSEGVVYWMNRDHRVQDNWALILAQQLALEGKVPLHICVCLVL
PQNSKLATLRHVGFMLRGLEENAKECRALDIQFHLLQGSAGEVLPGFVADWNLGAVVTDFSPLREPLRWLEDV
KKGIPLDIPFIQVDAHNVVPCWVASDKQEYAARTIRTKINKLLPEFLTEMPTVGKHPHPAAQTAKPVDWAGLM
SSLELDRTVEEVGWARPGTAAGLAMLESFIDQRLKNFATLRNNPNSSALSQLSPWIRFGHLSAQRVVLRVKSC
EKKARESVPTFIEELVVRRELTDNFCFYNSNYDSVKGASDWAQKTLKDHAKDPRPYVYSQEQLEKAQTHDKLW
NAAQYQMVTEGKMHGFMRMYWAKKILEWTSSPEEALSIAIYLNDRYELDGQDPNGFVGCMWSICGIHDQGWGE
RPVFGKIRYMNYKGCTRKFDVAQFERKYCPKTA
>Esox_luc_XP_028972045.1_cry_DASH_X
MSTSRTIIYLLRNDLRLHDNEVLHWTQRTADYVVPLYCFDPRHHLGTANYNLPCTGPFRLRFLLDSIQDVRAS
LLSRGSNLVVRRGRPEEVVGDLIKQLGSVSAVAFHEEVAPEDQKVERGVRDVCSQMKVKVYTCWGSTLYHRDD
LPFNHLSRLPDVYTQFRMAVESKGRVRPVLPTPGQLKPLPPGSGLDEGPIPSPEELAQTEAVRDPRSAFPFSG
GESQALARLQHYFWDTDAVATYKETRNGLIGPDYSTKFSAWLALGCISPRYIYQQIKKYESERTASQNTYWVI
FELLWRDYFKFVGAKYGDRMFDIKGLQDKSIPWKTDMKLFNAWKEGRTGVPFVDANMRELALTGFMSNRGRQN
VASFLTKDLGLDWRMGAEWFQYLLVDHDVSSNYGNWLYSAGIGNDPRENRKFNMIKQGLDYDNNGEYVRQWVP
ELQGIRGGDVHTPWTLSSSSLSHSQLTLEETYPSPIVMAPEWSRHVNRKLSGAGASARGRKGPSHTPKQHKDR
GIDFYFSKSKNF
>Esox_luc_XP_034149143.1_cry_circad
MAHTCIHWFRKGLRLHDNPALLAALQDCKEIYPVFVLDPHSPVNVNVGINRWRFLIGALKDLDCSLRKLNSRL
FVVRGKPEEVFPKLFQEWKVTRLTYEYDTEPFSLCRDKDVVRLAEEHGVEVIFKVSHTLYNIERIIEENNGKA
PLTFSRLQTLVKSIGPPRRPVPAPTSQDMKDIKTPCPEQYEQIYGMPKLEQLDQDPERLTVELFPGGEQEALL
RLDQHMERKEWVCGFEKPQTSPNSLSPSTTVLSPYMTFGCLSARTFWWRLTDVYQGKKHSQPPVSLHGQLLWR
EFFYTAGVGIPNFNKMEGNPACTQVDWDSNPEYLAAWTQARTGFPFIDAIMTQLRQEGWIHHLARHAVACFLT
RGDLWISWEEGQKVFEELLLDGDWSLNAGNWQWLSASTFFHQYFRVYSPIAFGKKTDKNGDYIKKYLPLLKKF
PAQYIYEPWKAPRSVQEAAGCIVGKDYPRPIVDHEVISKKNIQRMKGAYAKRSPHSSKESPSSKEKGGKRKAS
SVLDMLMKKKKTV
>Eudromia_elegans_NXA36424.1_CRY2_prote
CIYILDPWFAASSAVGINRWRFLLQSLEDLDNSLRKLSSRLFVVRGQPTDVFPRLFKEWGVTRLTFEYDSEPF
GKERDAAIVKLAKEAGVEVVIENSHTLYDLDRIIELNGHKPPLTYKRFQAIISRMELPKKPVSSIVSQQMETC
KVDIQENHDDMYGVPSLEELGFPTDGLAPAVWQGGETEALARLDKHLERKAWVANYERPRMNANSLLASPTGL
SPYLRFGCLSCRLFYYRLWELYKKVKRNSTPPLSLYGQLLWREFFYTAATNNPKFDRMEGNPICIQIPWDRNP
EALAKWAEGKTGFPWIDAIMTQLRQEGWIHHLARHAVACFLTRGDLWISWESGVRVFDELLLDADFSVNAGSW
MWLSCSAFFQQFFHCYCPVGFGRRTDPSGDYVKRYLPKLKGFPSRYIYEPWNAPESVQKAAKCIIGVDYPKPM
VNHAETSRLNIERMKQIYQQLSRYRGLCLLASVPCVEDLSGQVADPAVGQSSSTSTVMRVPQTDQASPKRKHE
GAEELCAEELYKRAKVGGLPAPEVPGKSL
>Eudromia_ele_NXA31107.1_PHR_lyase
DNWAFLYAQRLALKQELPLRVCFCLVPRFLDATIRHYGFMLRGLQEVAKECTELNIPFHVLLGYAKDVLPAFV
VRHGVGGLVTDFCPLRVPQQWVEDVRERLPEDVPFAQVDAHNIVPCWVTSPKQEYSAWTIRSKIHRQLPEFLT
EFPPVIQHPYPPSTPAEPVAWEACDSSLQVDRTVKEVAWATPGTAAGLAVLQSFIAERLASFGSHRNDPNKAA
LSNLSPWFHFGQVSTQRAILEVQKHHGKHKESVDVFVEEAVVRRELAENFCYYNKNYDSVQGAYNWAQTTLKL
HAKDKRPFLYKLQELEQGNTHDTLWNAAQLQMVQEGKMHGFLRMYWAKKILEWTHSPEEALQFAIYLNDRYEL
DGRDPNGYAPLRAGMLTLCVCPAGCLWSICGIHDHGWTERAVFGKIRYMNYAGCKRKFDVGQFERRYSPRK
>Eudromia_ele_NXA35803.1_CRYD
LLKVDYDVCSNYGNWLYSAGVGNDPRDNRRFNMIKQGLDYDGSGDYVRLWVPELQELKGGDIHAPWALSSASL
SRAGITLGETYPQPVVTAPEWSSHISHRPQGRGPHPKGRKGPAHPPTQHQDRGIDFYFSRKKHQ
>Eudromia_ele_NXA37922.1_CRY1
RFLLQCLEDLDANLRKLNSRLFVIRGQPADVFPRLFKEWSIAKLSIEYDSEPFGKERDAAIKKLATEAGVEVI
VRISHTLYDLDKIIELNGGQPPLTYKRFQTLISRMEPLEMPVETITPEVMEKCTTPVSDDHDEKYGVPSLEEL
GFDTDGLPSAVWPGGETEALTRLERHLERKAWVANFERPRMNANSLLASPTGLSPYLRFGCLSCRLFYFKLTD

LYKKVKKNSSPPLSLYGQLLWREFFYTAATNNPRFDKMEGNPICVQIPWDKNPEALAKWAEGRTGFPWIDAIM
TQLRQEGWIHHLARHAVACFLTRGDLWISWEEGMKVFEELLLDADWSVNAGSWMWLSCSSFFQQFFHCYCPVG
FGRRTDPNGDYIRRYLPVLRGFPAKFIYDPWNAPESVQKAAKCVIGVNYPKPMVNHAEASRLNIERMKQIYQQ
LSRYRGLGLLATVPSNPNGNGNGGLMGYSPGENISACSSTGGAQLGTGDAHAAGVQTCTLGDSHAGTSGIQQQ
GYCQASSILHYAHGDNQQSHLLQTGRTSLGTGISAGKRPNPEEETQSIGPKVQRQSTN
>Eudromia_ele_NXA43208.1_CRY1_
MPHRTIHLFRKGLRLHDNPALLAALASSDTLYPVYILDRGFVASATTMGPLRWHFLLQCLEDLRASLLQRGSC
LLLLQGEHERVLREHVSKWNITQVTLDAQVEPCYKEMEAKIRSLGQEMGFKVLSLTSHTLYDVQRILDLNGGT
PPLTYKRFLHVLSLLGDPEVPVRNPTAEDFQRCRPPDLCLAECCRVPLPADLNIPMESLSPWKGGETEGLQRL
ERHLMNQGWVASFTKPRTVPNSLLPSTTGLSPYFSLGCLSVRTFFYRLSHIYAQSKHHSLPPVSLQGQLLWRE
FFYTVASATPNFTKMVGNPICLQINWREDAERLHKWKTAQTGFPWIDAIMTQLRQEGWIHHLARHATACFLTR
GDLWISWEEGMKVFEEMLLDADYSINAGNWMWLSASAFFHRYTRIFCPVRFGKRTDPHGHYIRKYLPILKNFP
SKYIYEPWTASEEEQKEAGCIIGQDYPFPMVNHKEVSDHNLLLMKRVREEQRRTAQLTRDDADDPMEMKLKRD
HSEENVKKGKMAKTTE
>Euphausia_sup_ANW48376.1_cry_1
MTNTGCVVHWFRHGLRFHDNPALLHGLKLVDDNIKFLAVFIDDGHCAGLKNTGYNRARFLSESLEDLDDTLKK
YKSQLYCFQGKPEEIFRILHKEIGIRRLTFEQDCEAIWNERDKVVNKMCQELNIDVVEEVAHTLWNPYDIIEL
NGGTPPLTYEMFLQVSSTAGKPPKPCSRPQWEIVNFQEINDQLALKLKMHPKVPTPEMFGVYREGGENMQYTG
GETSALKHLKERLLVEENAFRDGYILPNQVNADILGAPMSMSAALRFGCLSVRKFYWSVQNTYEKLYAGESPP
TYSLTAQLIWREFFYCMSVNNPKFDQMKDNPICIQIPWYDNKEHLKAWTDGQTGYPFIDACMRQLKREGWLHH
IGRTAVSCFLTRGDLWISWEEGLKVFYKYLIDADWSVSAGNWMWVSSSAFERQLDCSTCICPVNYGRRVEPTG
DYIRRFVPELSSIPLEYLFEPWLAPLRVQEQNGCIIGKDYPTRIVIHKNASKENRRMMQDISAKLEMATPHVC
PSNNVETRTFLRLPDQCQHNVC
>Euphausia_sup_CAQ86665.1_cry
MTRGGEKHVVHWFRKGLRLHDNPTLKAGLKGATTFRGIFIIDPWFAGSSNVGINKWRFLLQCLEDLDTTLRKL
NGRLFVVRGQPAHVLPQLFKTWGTTCLTFEKDPEPFGKVRDANITHIAREMGIQVIIKTSHTLYKLEKIISKN
GGKLPLTYKTFQNVLSTMEPPPLPASPVTVRDVGDAFTPIDEDHDEKYGVPTLEELGFETENLAPSIWKGGES
EALARLEHHLERKAWVASFGRPKMTPQSLYPSRTGLSPYLRFGCLSARRFFAELNDLYRKIKKSPAPLSLHGQ
LLWREFYYTAATNNPKFDHMEGNPICVQIPWDKNAEALAKWAHGRTGFPWIDAIMSQLRKEGWIHNVARHAVA
CFLTRGDLWVSWEEGMKVFDELLLDADWSVNAGSWMWLSCSSFFQQFFHCYCPVRYGRKADPNGDYIRTYLPV
LKNFPTKYIHEPWTAPENVQKQSKCVIGRDYPMPMVDHVKQSQANLMRMKQVYQQLSHYRVTPPKITSLLKSC
PPPLPVICDKSKNTKNNKNEITRESNYTQQMQPA
>Euplokamis_dun_AQX17844.1_putative_p
MNGLGESVGLHWFRKDLRLHDNRALLKAIKTSSTLIPLYILDTKYLSPAYRGVNYQSWILDTLRCLNDELRAK
GSRLFVVIGNPINVLLSVIEKFQVKSITWERDTEPYNKDIDDRLYDICRDDEIDAHGFWGHTLYDPDYLLELN
EGAAPLTMTGFLNIIALAGPPLKPVAAPSTVPGPPTDIYCGDIFETIPTLSDLKSYGYNPLDKTTKFVAGEKE
AFRRMEKFLAQRSRVATFDKPKTNPTSLEPDTTALSPYISRGSLSTRYFYSKLLEALGESTESTRPILKGSLE
GQLYWREMAYLIGYSTPNFNQMVGNPVCKQIPWLEGKEAKMLLHKWEMGKTGYPAIDAAMNQLRIDGWLHHLG
RHLVACFLTRGDLWVHWELGRDIFNRYLLDSDWSITNFSWHWLSCSAFFHQYFRCYGPTTFFKRTDQTGAYVR
KFVPILKNFPDNYIYEPWKAPIEVQEAAGCIIGKDYPMPIVDHDVESKRNMGKMRMAYLNAQLLNLNFMY
>Euprymna_sco_AGJ94014.1_cry-1
MDVKMKKKIAVHWFRRGQRIHDNPALIDALKDCDEFYPIFIFDGKVAGTEICGYNRWRFLLENLKDLDDTFSQ
FGGRLYCFHGQPVDIFKNMFEEWGVNYITAEEDPEPIWKERDDSARELCEESGITCKFFTSHTLYSPQDIISK
NGGTPPLTLELFQLVISSLGDPMRPIPEPNLEGVNMPVPENFEKFALPNLSYFGIEPECEEQKKPINVFVGGE
KRALALLKARLEKEKLFFEQGSCLPNHQENPELLAKAISLSPYLRFGCVSIRKTYWGICDTYKQVCHKETPSE
VICQLHWREYFYVMCVGNINFDRIEGNPICLKINWAKNDELLKKWEFGQTGYPWIDAIMNQLRFEGWNHHVGR
HAVSCFLTRGDLWISWEEGLKVFLKYQLDADWSVCAGNWMWVSSSAFEKALQCPTCYSPIMYGMRMDRNGEFV
RTYLPVLKDMPLKYLFCPWKAPLGVQEKANCIIGKDYPEPICSHRDMSRENMAKMYKIKEELLKGHIPHCAPT
CEMEVWKFVWLPPIEHHEFAHNL
>Euprymna_sco_AGJ94015.1_cry-2
MSGEREKNVIHWFRKGLRLHDNPALKEALEGATTIRCVYTLDPWFAGSSQVGINKWRFLLNCLEDLDASLRKL
NSRLFVVRGQPADVFPQLSKEWNITTLSFEEDPEPFGKDRDAAICTLVRDAGIEVIIKTCHTVYDSKAILEKN
GGRPPLTYNKFQKILEEMDLPPRPLETVTVGDFGDCRTPLSVDHDEKYGVPTLEELGFDTEGLGQSNFHGGET
EALARLDRHLERKAWVASFEKPKMTPQSLFPSHTALSPYLRFSCLSPRLFYWKXNRTIXXDQKGRTIPPLSLH
GQLLWREFFYTAASNNPRFDQMVGNPVCVQIPWDINQEALAKWAEGMTGFPWIDAIMKQLRKEGWIHHLARHS
VACFLTRGDLWISWEDGMKVFEELLLDADWSVNAGMWMWLSCSSFFQQFFHCYCPVGFGKRIDPNGDFIRHYL
PILKRFPAKYIYEPWNAPESVQKTAKCIIGKDYPVPMVNHSEVSRINMERMRQVYHRLTMRSTATIPKQIHSR
EGQQYTKHSKPMDVCFYGTSKHDKRYEQHFMLKSY
>Euroglyphus_may_OTF77403.1_photolyase

MATLVLLQRDLRQFDNPALYNAVRRRKPIIVVHVMEKRLGSTSKWWLCKSLRLFRQQLEEWSIPLLHLDDSAN
FSDMIDYLNDHIEIEQIYSNGLDFELPDNFVEIHESFLANLIFIPEDMTKEFKIFTPFWKSIRKNCSPVKPLP
NPDWCKKNHVNIEFDEYNELPMMMADDRWNQMESYWEIGEKAAQKRLELFVKTKLSDYDHGRDFFHKDGTSKL
SPHLRFGEISVRQIYEKLNNKSNERFCSELGWREFAYHVYRNHGDMAKIPLNPKYCDFWPEYNDDHLNAWRNG
QTGYPVIDAAMRDLSTTGWMHNRLRMLVASFLVKNLLIPWQIGEEWFYDHLVDADPAVNPFSWQWVAGCGTDS
VPYFRIFNPMLQGEKFDPQGIYVRKWLPEFADFPMKRNIHQDIIKQPNLRPENYPQPIIDFKSSRERTLSTYK
AIVAAKVIQLKKPRLP
>Euryarchaeota_arc_MBF56040.1_photolyase
MWFRQDLRISDNPALLEAVSSGEIIPIYILDDINSQSEKIGDASRVWLHYSLLELNRSLNGNLRFFKGAAKEI
ISELIRETDSKKIVWNRCYEPWRIKRDTYIKKDLKESGIEVKSCNGSLLWEPQEICKSDGTHYRVFTPFYRRG
CLNAVPPRIPQNTPLNIKFAEILNLKRDCSLEELELLPKHNWYISMVKNWKIGEQGGLDDLDDFLKNGIVNYK
MGRNIPAKKSVSRLSPRLHFGELSPNQVWYAAQQIIEPGDSNLDSFLSELGWREFAYHLHFHYPSLANENLNP
KFNSFAWKTETNNIEAWQTGMTGYPIVDAGMRELYQTGYMHNRLRMIVGSFLVKNLLQHWHHGRDWFHNCLVD
ADLCVNSASWQWIAGCGADAAPYFRIFNPITQGEKFDPEGEYTKKWVPELINIPKKFLFCPWQAPAEILEKSG
IQLGTTYPMPIVDLKVSRNLALEAFSKLPKTA
>Euryarchaeota_arc_MBQ60325.1_CMA66_0727
MKLAVVWFRNALRIHDNPVLSWAYESENADFILPIYIFEEEIQQKSTQNMGEQRLRFQFDCVENLHQNLKNKL
DLDLHVFSGDCLEVLESIREQLQPFEVILLKEYSTNPRDRKQSELIGKKLINTNPTWSIKSFPSTQTILDIEA
IVGSPGYKPPKSMKDMVRLFKIEFGDYENIIFETNDPNPSSNERKQDVSVNSKYQVSLDKIRSSLSTAGYFRG
GENEAITRLEKKVISQNDYVNSFNKPKSISTNIQGNPMEPTTTGLSPYISTGCLSIRRLWNSCSEIQQSGEHT
VPPVSIQGQIMFREMFYLLSRSVDNWDQDSGNPQCKEIEWGELNHALLDSWDSGQTGFPLIDAMMRQLNATGW
MHHLGRHAVSCFLTRGQLWQNWTHGRDIFERKLIDSDWALNNGNWLWLAGVAPFSMPYYRIYNPCPDSKSSLN
VETKDAEFIRYWVPELAEFPSKYIFEPHLAPLHIQESAGCLIGTDYPNPIIDRKESRKKNLQSFKHSLEKLR
>Eurydice_pul_AGV28717.1_cry_2
MKMTGTNGSGKAKPKNVVHWFRKGLRIHDNPALRLGLRNCTTFRCIFILDPWFAGSSNVGVNRWRFLLQSLED
LDRSLRTFNSRLFVIRGQPAKALPELFKEWDTTILTFEEDPEPFGKARDANVVAMAKEMGIKVIVRTSHTLYK
LDRIIETNGGKPPLTYKSFQNILSHIGPPSKPDSPVNREEINGGITPVSDDHDDKFGVPSLEDLGFDTDHLPN
PVWKGGETEALTRLEHHLERKAWVASFGRPKMTPQSLYASPTGLSPYLRFGCLSPRQFYVKLNELFQKIKKLP
PPLSIHGQLLWREFYYCAATNNPKFDHMEGNPICVQIPWDKNPEALAKWANGQTGYPWIDAIMTQLRQEGWVH
NVARHAVACFLTRGDLWVSWEEGMKVFDEPLLDADWAVNAGSWMWLSCSSFFQQFFHCYCPVRYGRKADPNGD
YIRTYLPVLKNFPSRYIHEPWTAPEGVQRAAKCIIGIDYPMPMVDHSKQSQHNIERMKQVYQQLTHYRGTAPP
FSKSMKNRNHINIEFPPPRFEFEKPFPKKDKREVKTAVD
>Eurydice_pul_AGV28718.1_6-4_photol
MSVICWFRKGLRLHDNPALTAAIDQGMELFPVFVLDPWFVANARVGPNRWRFLHESLTDLDNSLKAIGSRLFI
LKGKPEEALVKYYSKFDVKAITWEYDSEPYSKERDQRIEEISESKGVKVIIKHGHTLYEAERIFKANGNKVPL
TYQAMQKSMSNLGAPPKPKPAPSQLPSHCEPKNKALMTSTKFNIPSLEDLNIDVSELLPPLYPGGESEALKRM
KKCLSKKQWICNFEKPKTSPNSLEPSTTVLSPYLKFGCLSSRLFYYELAKVYEGNKHSQPPVSLHGQLLWREF
FYAVGALSPNFDRMEGNPICKQIPWSNDQKLLEAWKHVRLQGRTGYPFIDALMTQLKREGWIHHLGRHAVACF
LTRGDLWQSWEEGMKVFEEFLLDADWSLNAGNWMWLSASAFFHQYFRVYSPVAFGKSTDPDGDYIRKYIPALS
KFPTKYIYEPWKAPLSDQKAAGCIIGQDYPRPIVDHDIARKKNLQKMSNAYKADKEKRGADDTSNKAKKKK
>Eurypyga_heli__KFW12078.1
DNWAFLYAQRLALKQELPLHVCFCLVPKFLDATIRHYDFMLRGLQEVAEECAELNIPFHLLLGYAKDVLPAFV
VEHGVGGLVTDFCPLRLPRQWVEAVREGLPEDVPFAQVDAHNIVPCWVASPKQEYSARTIRGKIHTQLPEFLT
EFPPVIHHPYPPSCPAEPIAWEACYSSLEVDHTVKEVEWATPGTAAGLAVLQSFITERLGSFGSHRNDPNKAA
LSNLSPWFHFGQVSTQRAILEVQKLKGKYKESVDAFVEEAVVRRELAENFCYYNENYDSVQGAYDWAQTTLKL
HAKDKRPFLYKLQELEQGTTHDPLWNAAQLQMVREGKMHGFLRMYWAKKILEWTRSPEEALRFAIYLNDRYEL
DGRDPNGYVGKRCLWSICGIHDQGWAERAIFGKIRYMNYAGCKRKFDVGQFERRYAP
>Eurypyga_heli__XP_010145866.1
MPHRTIHLFRKGLRLHDNPTLLAALESSGVVYPVYILDREFMTSAMHMGALRWHFLLQCLEDLQKNLCQLGSC
LLVIQGEYESVLRDHVQKWNITQVTLDAEVEPFYKEMEANIRHLGEELGFEVLSVVSHSLYDTKRILDLNGGT
PPLTYKRFLHILSLLGDPEVPVRTLTAEDFRSCRPPDPGLAERCRVPLAADLKILPESTSPWRGGETEGLQRL
EQHLTDQGWVASFTKPRTIPNSLLPSTTGLSPYFSLGCLSVRTFFYRLSNIYAQAKHHSLPPVSLQGQLLWRE
FFYTVASATPNFTKMAGNPICLQIGWYEDAERLHKWKTAQTGFPWIDAIMTQLRLEGWIHHLARHAVACFLTR
GDLWISWEEGMKVFEELLLDADYSINAGNWMWLSASAFFHQYTRIFCPVRFGKRTDPEGQYIRKYLPVLKNFP
SKYIYEPWTASDEEQAQAGCIIGRDYPFPMVNHKEASDRNLQLMKQVREEQCGTAQLTR
>Eurypyga_heli__XP_010146875.1
MQHSSIHWFRKGLRLHDSPNAQRFLLDALRDLDGNLQELGSRLFVVRGCPEEVFPRLFHAWGTTRLTFEVDTE
PPARRRDAAVAELAARHGVEVIQEVSHTLYDTERVLALNDGKAPLTYKRLQSLLALLGPPEKPAPALTQEHLR
GCCPPCQASHDADYGVPTLEELGQDPTEVGPHLYPGGETAALARLDTLMKRTTRGCSFKKPETLCRQREHSQP
PISLHGQLLWREFFYTAGSSIPNFDRMVGNPICLQVDWENNPEHLRAWREGRTGYPFIDAIMTQLRTEGWIHH

LARHAVACFLTRGDLWVSWEEGLKVFEELLLDADWSLNAGNWLWLSSSAFFHRYFHVYSPVAFGKKTDRDGAY
IRKYLPILKDFPAEYIYEPWRAPRAVQERAGCLVGTHYPQPIVEHGVASKRNLGRMKAAHARKGNRFPKKYPG
PPTAQPSPQLPSPPPGIKREPEASPAAAKSQRKKPKQR
>Eurypyga_heli__XP_010147964.1
VLHWAQSNADFVIPLYCFDPRHYLGTHCYGFPKTGPHRLRFLLESVKDLRETLKKKGSTLVVRKGKPEDVVRE
LITQLGSVSAVAFHEEATQEELDVEKGLCQVCSQHGVKIQTLWASTLYHRDDLPFRPIARLPDVYTHFRKAVE
SAAKVRPMLRMADQLKPLALGVEEGCIPTMEDFGQKDPVTDPRTAFPCSGGETQALMRLQYYFWETNLVASYK
ETRNGLVGMDYSTKFAPWLALGCISPRYIYEEIQRYEKERTANASTYWVLFELLWRDYFRFVALKYGKRIFSL
RGLQSKDIPWKKDLQLFDCWKEGKTGVPFVDANMRELAATGFMSNRGRQNVASFLTKDLGLDWRMGAEWFEYL
LVDYDVCSNYGNWLYSAGVGNDPRDNRKFNMIKQGLDYDGN
>Eurypyga_heli__XP_010154061.1
IIELNGHKPPLTYKRFQAIISRMELPKKPVSTIISQQMETCKVDIQENHDDVYGVPSLEELGFPTDGLAPAVW
QGGETEALARLDKHLERKAWVANYERPRMNANSLLASPTGLSPYLRFGCLSCRLFYYRLWELYKKVKRNSTPP
LSLYGQLLWRGVFFTPATNTFDRMEGNPICIQIPWDRNPEALAKWAEGKTGFPWIDAIMTQLRQEGWIHHLAR
HAVACFLTRGDLWISWESGVRVFDELLLDADFSVNAGSWMWLSCSAFFQQFFHCYCPVGFGRRTDPSGDYVKR
YLPKLKGFPSRYIYEPWNAPESVQKAAKCIIGVDYPKPMVNHAETSRLNIERMKQIYQQLSRYRGLCLLASVP
SCVEDLSGPVADSASGQGCSSSTTARLSQADQASPKRKHEGAEELCAEELYKRAKVTGLPAPETPGKSL
>Eurypyga_heli__XP_010159206.1
FLLQCLEDLDANLRKLNSRLFVIRGQPADVFPRLFKEWNIAKLSIEYDSEPFGKERDAAIKKLASEAGVEVIV
RISHTLYDLDKIIELNGGQPPLTYKRFQTLISRMEPLEMPVETITPEVMEKCTTPVSDDHDEKYGVPSLEELG
FDTDGLPSAVWPGGETEALTRLERHLERKAWVANFERPRMNANSLLASPTGLSPYLRFGCLSCRLFYFKLTDL
YKKVKKNSSPPLSLYGQLLWREFFYTAATNNPRFDKMEGNPICVQIPWDKNPEALAKWAEGRTGFPWIDAIMT
QLRQEGWIHHLARHAVACFLTRGDLWISWEEGMKVFEELLLDADWSVNAGSWMWLSCSSFFQQFFHCYCPVGF
GRRTDPNGDYIRRYLPVLRGFPAKYIYDPWNAPESIQKAAKCIIGVNYPKPMVNHAEASRLNIERMKQIYQQL
SRYRGLGLLATVPSNPNGNGNGGLMSYSPGESISGCGSTGGAQLGTGDGHTVVQPCALGDSHTGASGIQQQGY
CQASSILHYAHGDNQQSHLLQAGRTALGTGISAGKRPNPEEETQSVGPKVQRQSTN
>Eurytemora_aff_XP_023320251.1_photolyase
MASSSKKAKLDGISGLLMEERVELASSVSEFKFNKKRVRVLQGVDGDMKRECKSVMYWMWRDRRVQDNWALLY
SQKIALELNVPLQVLVCVPQSLGELTLRHYTFMLEGLKEVAEECKELNIGFSLALGEPSDIITKSYLTKHKIG
LLVVDYSPLRYHKAWLDCIQEKIDTPIHQVDAHNIVPVWITSDKQEYAARTIRPKITKNLAEYLTGFPPVIEH
PVDSISEISGLDFDEVYKGLKIDRTGWGVEPVKTFTPGTRAGLCNLEEFCAKRIKEYGAKRNDPNVSALSNLS
PWVNNGQISMQRAVIYVKKHGKSQSESVASFVEEGVVRRELSDNFCEYNPDYDTLKGASDWAKKSLNEHKKDK
REYIYTREEFEEGRTHDDLWNAAQLQLVREGKLHGFLRMYWAKKVLEWTESPESALVEALRLNDRYAVDGNDP
NGYVGVMWSITGIHDQGWAERAVFGKIRFMNYAGCKRKFDINKFIIKYGAKVHKKK
>Eurytemora_aff_XP_023327943.1_cry-1-like
MSKVEPGGKGFPEQGRPESKPTLKKTPEGLVRMRAQAEKDVINILWFRNGLRLHDNGSLHNATEDKTAKLLPV
FIFDGETPVTKECEYNKMKFLLECLEDLNEQFIEAGAKLYCIQGNPEEVLRKLKHRVQINRVCFDQDCEAIWL
ERDNNIRNYCVSHKIEVFEHIGATLWDPLEIIEANGGTPPLTYSHFCHVTKGIGEPKRPLPNIDLSTVSFYEI
PDSILAELKTFPGVPTPEDLGFYKGDEEKIYKGGERTALKYFNRRMAVEKEAFLDGSFLPNRRDPDILNPPKS
LSPDLKFGCISVKLFYWSTIDAFNQVNEKKSSSYLIVSQLIWREFYYAMSANNPFFAEMERNPVCINIPWKED
EQALEKFNAARTGYPFIDAGIRQLKKEGWIHHVLRNALSMFLTRGDLWLNWVHGEKLFRTYLIDGDWAVNAGN
WLWVSSSAFEKSLNYSYSLDPRVYGRHVDSHGKYIKRYIPELANYPVEYIYTPWKAPLEIQKKAGCIIGKDYP
EPMIDHDEAVAQNRQMMLSLQKDLLTQEKGQPNHVRPSGDLEINLFFRIHD
>Eurytemora_aff_XP_023329370.1_cry-1-like
MSRNKSSKRKHSVHWFRKGLRLSDNPALLRAIRRCETFRCIFILDPWFAGSSNVGTNKWRFLLQCLEDLDNGL
RKLNSRLFVVRGQPADVLPTLFKAWGTTYFTFEEDPEPFGRVRDQNIMAMCKEMGIVVTKEHSHTLYNLEKII
YRNGGKAPLTYKTFQTIIESMDPPSKPVPTLTLLMLGTGITPLSDDHDEKYGVPTLEELGFDCDHLKQSVWEG
GETEALSRLERHLERKAWVASFGRPKMTPQSLLASQTGLSPYLRFGCLSTRLFYHALSDLYRRIKKCDPPLSL
HGQLLWREFFYCASTKNPNFDKMVGNPICVQIPWDKNDEALAKWANAQTGFPWIDAIMTQLREEGWIHHLARH
AVACFLTRGDLWVSWEEGMKVFDELLLDADWSVNAGSWLWLSCSSFFQQFFHCYCPVKFGRKADANGDYIRRY
LPVLKNYPTRYIHEPWTAPDAVQKSARCIIGQDYPKPICHHPVVSKINMERMKQVYQQLAHYRQTGQTGPGIP
QDLLNQLPKTPKIPKVPNVTGGSMGPPNPPSNEKKKDGYDCMDQPESSSQIQEQNWIGSCESKFSDQTWIRSK
SQDWVGNPEAKVQDQTWIGSSTPIPRASVLTDLQTTIDNKECTRRAEEQASILSQTGGVIVYEQYNGANSQYI
PYIQERKNSSQSTSIHQTSDCLPVLSVLTVNYSRLSACIICSICKLFQIVCLYYVF
>Eurytemora_aff_XP_023333098.1_cry_DASH-1
MSASLKTAVYLFRNDLRLHDNECWVLANKTADYVIPLYCFDPAHFKKTWHFGLPKTGAIRAKFLIETVNDLKT
NLKTKNTDLVVKNENPVSALKSLVEACRLSAPVTSLIYQKEVTFEEVNLENDLKKFCGNLDIKVHEVWGSTLF
HLEDLPFKINRVPDTYTNFRKEVEGRSKVRRIVSDPQVIKPWPDSKIDGGSVPTLQVLGLEEPGLENRTAFPF
SGGETSALGKTIFILGENYSTKFSPWLAFGSLSPRRIYSEIKRYEDERVSNKSTYWVIFELLWRDYFRFVCLK
FGDKVFYRSGILGRDQAWIQDTNKFKAWVEGRTGVPFVDANMRELKETGWMSSLDWRFGAEWFESQLLDHDVC

SNYGNWNYAAGIGNDPRENRKFNMIKQGMDYDADGEFVRTWVPELRKIPGGKIHVPWTLNDKELTSAGVDLGV
NYPKPLVLAPEWGRHSQKTNKPPSYRDPPKGQRPINAFFKPEYGGGGVQGSGGREGGGVQGSGGRGGGGGKWG
PGRGKAPRGGRVQF
>Eurytemora_aff_XP_023343976.1_uncharacte
MVYRNVVWTALYEPILKERDSRLEKRLAGAGVHVDIEHSYLLHRPDQVTVAGVGARGIGSVTHFMECCKSNPG
DSIGKPIDPPRYLNNPTTWPTSHSLPQLRLYRKPVKADGSVVDWAKEIRASWKFGEDGGYQNLRKPIRLPYAN
QPWSQNKAHLKAWQRGNTGFPLVDASMRQLWKIGWMNNYMRHVVASFLISYLRISWIEGYNWFQDTLLDADVA
INAMMWQNGGMSGLDQWNFVMHPVDAAFTCDPQGEFVRTWIPELAGLPDKLIHQPWKCPKGVLSRAGVRLGEN
YPNRIIENLEDAREQSLQDVTEVRRKHSRGFIDPQHGRDMTGIPCTLLGINGVKQNSILMVPLITRKEFIYRT
GCPDAKDNPYNAVLKGYVGRARDEEIERTNKVDFTASTMLEFKERKARLDKLNGVVEEDEGRRGGRRGGGIQR
RTKTHDKFTKV
>Eurytemora_aff_XP_023343983.1_cry-1-like
FNCSPVHLFTCSLVHLFIRDWDARLLTWEEDTEPYAVSRDAKIKNIAKDLNVEVKTFCGHTLYNPEAIIAANK
GDVPLTYQKFQGLLSKLGSPAKPLELPAVLPTESKPEDIDESKYDVPSLEDILKRAGKKSDDLGLNKFPGGES
EALKRLEDKICEKTRGWVLKFEKPNTSPNSLEPSTTVLSPYLKFGCLSPRTMYWQLQSIYKTGKHTEPPVSLH
GQLLWREFFYTVGANTRNFDKMEGNQICKQIDWDSNPEKVAAWAEARTGFPWIDAIMTQLRTEGWIHHLARHS
VACFLTRGDLYQSWERGQEVFEELLLDADWSLNAGNWMWLSASSFFYQFFRVYSPVAFGKKTDPTGEYIRKYL
PQLKKYPDKYIYSPWEAPLAVQKAAGCIIGQDYPRPIVDHTVISKINIGRMKKAYDAGKAGKETPKKKEGKEP
PKKKQKTMDGFLAKK
>Evechinus_chlo__ACO51511.1
GKMHGFLRMYWAKKILEWTTSPEEALEIAIYLNDRYSLDGRDPNGYVGCMWSICGIHDQGWGERPVFGKIRFM
NYKGCKRKFDVDTFINRYKKY
>Exaiptasia_dia_KXJ19326.1_cry-1
MQQLSSILAMXLICDPKFLVFKGMARQVSCHWFRKDLRLHDNPALCESLANSDEFYAIYVLSPFFNKSIVSQR
KKNFLLQSLKVLEENLQKLGSKLIVVQGNLLQVFPFILKHFGITKLTFETACDRYGRQNEKVVEHLARELGIQ
VSSFASHTLFEVDEVVQVCGDSIPVLFEEYLDVVAQLQTPSKPYPKVCKTPAYNIEKLLDETLVAQMYHLNDV
QMSGELVGGEDNGLMKLEEYLEQGNTGFPLIDAIMRQLRQEGWINSTAQDIVCRFLTTKGLWISWDKGFKTFD
ALLLNSQWSLVAGSCMLSSQSSFFKKTIPLGFCPVKEAKRLDPNAEYINFVPSMGMDTSTLSILAHNTPHMLI
FTEGGKPEYTEKKTLLERELGNGTTGFPWIDAIMRQLRQEGWIHNFAREAVGCFLTRGCLWISWEEGFKAFDE
MQLDAEWGMNANCWMWLSCSSYVQGSVPWYCPVEVAKKFDPDAQYIRRYVPEVRRLPKKYIYEPWKAPQRVQE
AARCVVGFDYPEPMCDHIKQREICVQKLSEHSKRLTTPIVTGEIVHPLNNDVLKHCDGDYHATERE
>Exaiptasia_dia_XP_020903321.1_cry_DASH
MASKSRAVICLLRNDLRYHDNEVLSWAYKNAEFVVPLYCFDPRHFKCTWHFGFPKTGPFRAKFLVESVKDLRE
QLKTKRSNLVVRCGKPEEVVLELISRVNSETVKVEAIAFQSEPTKEELDVENAIQSGCGVPVHKIWGSTLYHI
DDLPFEIKRVPDVYTEFRKRVEAQSRVRSSIKMPETFKPLPPTVSEGLVPSLEELGIKESKDEPRSAFPFTGG
ETTGLQRLNYYLWDTNLVSKYKETRNGLIGHEYSTKFSTWLALGCLSPRKIYEEVKRYEKQRTSNQSTYWVIF
ELLWRDYFKFVACKFGDRLFHLSGLRGKNQEWHKDDTLFKAWKEGRTGVPFVDANMRELAATGFMSNRGRQNV
ASFLTKDLKLDWRLGAEWFEYLLVDHDVCSNYGNWLYSAGVGNDPRQDRKFNMIKQGLDYDPKGEYIRQWIPE
LAGIPGGDVHTPWRLSQTALSSAMVSLGETYPMPVVTAPEWXRHTSAKTKTGPKGSPQGRVAKRGIDFYFSSN
SNGGKR
>Exaiptasia_dia_XP_020904995.1_cry-1
MEVHVAHDHSIRRRAINSLHWFRKDLRLHDNPALREAVLNCDTFRVVYIFDTRNAQNSTIGINLWRFLLQSLE
ELDGSLRKLNSRLFVVRGQPADVLPRLFEQWRITKLTFTEDTEPFGKERDSAIRMLAREAGIDVVVKRSNTLY
DLQEIISRNNDKPPKTYKSFLNVLQRMSPPDLPVNTITHNSVGLGFTPFDESLIDQYGIPTLQELGLDASKLC
SEVWQGGEAEALNRLDRHLERKAWIASFEQPKINSNSLIATTNGVSPYLRFGCLSPRLFYHRLSDLFRKLKRK
VPPISLYGQLLWREFFYCVSTNNPNFDKMVHNPLCLQVEWRDDGRVEEDLERWAKGQTGFPWIDAVMAQVRQE
GWIHPLARYAAASFLTRGDLWISWERGVKVFEEFLLDADWSINAGNWMWHSCSAFSQHFFYPQCPIGFGKRLD
ANGDYISKYLPVLKGFPPNYIHEPWKAPPHVQKLARCIIGKDYPSPMLDHEEKRKENLARIVNILEYTTEDKP
EISYKEIDDKEPEQDLDFLALSSSG
>Exaiptasia_dia_XP_020910442.1_photolyase
MATSEPPSKKAKKESKGSXEEIDIMKHYNEKRKGVCDSVLDFKFNKKRVRLLTSTTDMKDDCKGILYWMWRDQ
RVQDNWALLYAQRLALKQQVPLHVCFCLPKQFLDATIRKYGFMIKGLQFVEKELEDLDIKFHLLLGEPDQVLP
GFVKEFGFGGIVCDFCPLRLPMKWVNEFVKSLPKDVPLCQVDAHNIVPCWEASPKLEYGARTIRPKIHKVLQS
FLTEFPPVIKHPHQTEVKSKGADWSAAIDFIEVDSSVPEVDWAKPGTAAGLNMLESFCKVRLKNFGASRNDPT
KNAISNLSPWFNTGQISNQRTILKIREYRGKYKDSVESFIEESVVRRELSENFCYYNDKYDSIEGTNDWAKKT
LKDHASDKREYLYDREALETAKTHDNLWNAAQRQLTREGKLHGFLRMYWAKKILEWTKTPETALADAIYFNDK
YALDGIDPNGYVGCMWSICGIHDQGWAERPVFGKIRYMNYKGCQRKFDVAEFIRRYKP
>Exaiptasia_dia_XP_020915067.1_cry-1_X1
MAAKRVHHTIHWFRKGLRLHDNPALREAYDTSLTVRPVYILDPDLVKCGQIGVVRWRFILQSLEDLDQSLRQI
NSRLLVIQGRPSEVFADLFKKWEITKLTFEVDTDXXAKAXDEEVIKLASQANVEVVQKVSHTLFDLDAILAKN

LGTAPLTYKSFQAVVSTLGAPDEPLPAVDRYITHGCKLPIDDEHDRYSIPTIDDLGLSIPEESSEVLYPGGEV
EALRRMNEYMAKQDWVCKFEKPSTSPNSIEPSTTVLSPYLALGCLSVRLFYKKIQEIYSKAKKHSQPPVSLHG
QLLWREFFSTCAHNTPNFNKMEGNRVCLQIPWDKNDDHLKAWSTGCTGFPFIDAIMIKLRKEGWIHHLARHAV
ACFLTRGDLWISWEEGLKVFENLLIDHDWSLNAGNWMWLSASAFFHAYFRVYSPIAFGKKTDPNGDFIRKYLP
VLKRFPAKYIYDPWNSPLSVQQAAGCVIGKDYPKPIVDHXKAVTKNLARMKQAREAKYGSKQAANVKKSPPKR
KRSSEKTNGKVRKITDFTKS
>Falco_cher__XP_005442448.1
MSGVARTAICLLRCDLRAHDNQVLHWAQSNADFVIPLYCFDPRHYLGTHCFGFPKTGPHRLRFLLESVKDLRE
TLKKRGSTLVVRKGKPEDVVCDLITQLGSVSAVAFHEEATQEELDVEKLLCEVCCQHGVKTHTFWGSTLYHRD
DLPFRPIARLPDVYTHFRKAVESEAKVRPTLQMADQLKPLAPGVEEGCIPTMEDLGQKDPVTDPRTAFPCSGG
ETQALMRLQYYFWDTNLVASYKETRNGLVGMDYSTKFAPWLALGCISPRYIYEQIQKYEKERTANQSTYWVLF
ELLWRDYFRFVALKYGRRIFSLRGLQNKEVPWKKDLQLFDCWKEGKTGVPFVDANMRELAATGFMSNRGRQNV
ASFLTKDLGLDWRMGAEWFQYLLVDYDVCSNYGNWLYSAGIGNDPRDNRKFNMIKQGLDYDSNGDYVRLWVLE
LQGIKGADIHTPWALNSAALSQVGVTLGKTYPQPVVTAPEWSRHINQRPQERSPHPRGRRGPAHTPRQHKDRG
IDFYFSRKKDA
>Falco_cher__XP_005443744.1
MPRGQRKRKAEGTELPGVSRRRKEEEEAVQEARRRAAPSVREFKYNKKRVRLVSQGSDLKENARCILYWMSRD
QRLQDNWAFLYAQRLALKQELPLHVCFCLVPKFLDATIRHYGFMLRGLQEVAEECAELNIPFHLLLGYAKDVL
PVFVVEHGVGGLVTDFCPLRLSRQWVEDVRERLPEDVPFAQVDAHNIVPCWVTSPKQEYSARTIRGKIHAQLT
EFLTEFPPVIRHPYPPSCPAEPIAWEACYSSLQVDRTVKEVEWATPGTTAGLAVLQSFITERLKSFSSHRNDP
NKAALSNLSPWFHFGQVSTQRAILEVQKHRDKYKESVDAFVEEAVVRRELAENFCYYNENYDSVQGAYDWAQT
TLKLHAKDKRPFLYKLQELEQGATHDPLWNAAQLQMVREGKMHGFLRMYWAKKILEWTRSPEEALQFAIYLND
RYELDGRDPNGYVGCLWSICGIHDQGWAERAIFGKIRYMNYAGCKRKFDVGQFERRYAPHTCSQ
>Falco_cher__XP_014135100.2
MDRFLLQSLEDLDNSLRKLSSRLFVVRGQPTDVFPRLFKEWGVTRLTFEYDSEPFGKERDAAIVKLAKEAGVE
VVIENSHTLYDLDRIIELNGHKPPLTYKRFQAIISRMELPKKPVSTVISQQMEACKVDIQENHDDVYGVPSLE
ELGFPTDGLAPAVWQGGETEALARLDKHLERKAWVANYERPRMNANSLLASPTGLSPYLRFGCLSCRLFYYRL
WELYKKVKRNSTPPLSLYGQLLWREFFYTAATNNPKFDRMEGNPICIQIPWDRNPEALAKWAEGKTGFPWIDA
IMTQLRQEGWIHHLARHAVACFLTRGDLWISWESGVRVFDELLLDADFSVNAGSWMWLSCSAFFQQFFHCYCP
VGFGRRTDPSGDYVKRYLPKLKGFPSRYIYEPWNAPESVQKAAKCIIGVDYPKPMVNHAETSRLNIERMKQIY
QQLSRYRGLCLLQSVPSCVEDLSGPVTDSASGQGCSTSTAMRLSQADQASPKRKHEGAEELCAEELYKRAKVT
GLPAPEIPGKSL
>Falco_cher__XP_014141780.1
MEPLEMPVETITPEVMEKCTTPVSDDHDEKYGVPSLEELGFDTDGLPSAVWPGGETEALTRLERHLERKAWVA
NFERPRMNANSLLASPTGLSPYLRFGCLSCRLFYFKLTDLYKKVKKNSSPPLSLYGQLLWREFFYTAATNNPR
FDKMEGNPICVQIPWDKNPEALAKWAEGRTGFPWIDAIMTQLRQEGWIHHLARHAVACFLTRGDLWISWEEGM
KVFEELLLDADWSVNAGSWMWLSCSSFFQQFFHCYCPVGFGRRTDPNGDYIRRYLPVLRGFPAKYIYDPWNAP
ESIQKAAKCIIGVNYPKPMVNHAEASRLNIERMKQIYQQLSRYRGLGLLATVPSNPNGNGNGGLMGYSPGESI
SGCGGTGAAQLGTGDGHTVVQPCALGDTHTGASGIQQQGYCQASSILHYAHGDNQQSHLLQAGRTALGTGIST
GKRPNPEEETQIVGPKVQRQSTN
>Falco_cher__XP_027658934.1
MQHSSIHWFRKGLRLHDNPALLAAATDCCCLHPLFILNPSNSQAGTNAQRFLLDALRDLDGSLQKIGSRLFVV
RGCPEEVFPSLFHAWGTTLLTFEVDTEPSARQRDATVAKLAAQHGVEVIQEVSHTLYDTERVLALNKGKAPLT
YKRLQSLLATLGPPEKPAPALTQEHLQGCHTPCQASHDTDYGVPNLEELGQDPTNVGPHLYPGGETAALTRLD
ALMERTAWVCSFQKPRTAPTSLSPSTTVLSPYLKFGCLSVRTFWWRLDQVYQGQEHSQPPTSLHGQLLWREFF
YTAGASIPNFDRMAGNPVCLQVDWDNNPQHLHAWREGQTGYPFIDAIMTQLRTEGWIHHLARHAVACFLTRGD
LWVSWEEGLKVFEELLLDADWSLNAGNWLWLSGSAFFHQYFRVYSPVAFGKKTDRDGAYIRKYVPILKDFPAE
YIYEPWKAPRAVQERAGCLVGTHYPQPIVEHGVASKRNLGRMKLAHARKGNKSPVRHQAHFTSSCHPSCLSLP
QAPSESHWLVPQWPNHKGKSPRPRGSEPHKDVQAVVSYGSCTTCPVLTHFAQPPCINPFPSPSLRLATWLLPP
PQKSTPTPSTCPKKG
>Falco_cher__XP_027672562.1
MLHRTIHLFRKGLRLHDNPALLAALESSEAIYPVYILDRKFMTSVMHIGALRWHFLLQSLEDLQKNLSQLGSC
LLVIQGEYESVLRDHVQKWNITQVTLDAEMEPFYKEMEANIRRLGEELGFEVLSLVGHSLYDTKRILDLNGGS
PPLTYKRFLHILSLLGDPEVPVRNITAEDFQRCRAPELCLAECYRVPLPVDLKIPPESLSPWKGGETKGLQRL
EQHLTDQGWVASFTKPRTIPNSLLPSTTGLSPYFSMGCLSVRTFFYRLSNIYAQAKHHSLPPVSLQGQLLWRE
FFYTVASATPNFTKMAGNPICLQISWDEDAERLHKWKTAQTGFPWIDAIMTQLHQEGWIHHLARHAVACFLTR
GDLWISWEEGMKVFEELLLDADYSINAGNWMWLSASAFFHQYTRIFCPVHFGRRTDPEGQYVRKYLPVLKNFP
SKYIYEPWMASEEEQKQAGCIIGRDYPFPMVNHKEASDHNLQLMKQVREEQYGTAQLTRDDTDDPMEMKPGCK
AAPGLSDGCERLAAGSWRC
>Fasciola_hepa__THD28195.1

MASENSFLIKIEKKRVNTAKSVLDFKFAKSRIRHLHGPEEFPGLVDESESAPMSVLYWMIRDQRVQDVAMGRL
TTQLSTTCALSVVCEIPRHYWENKHVLWSSEQKETDFRGGMNNWAMLYAQRLALKFRVPLHVCFCLAPCYQAD
TLRHLSFMLSGLVEVEQECRSLNIPFHLVNADNHLGPDENRKSGMQARWSPNYTTSSSAQIYDAAVAQAVGSL
VKSLHVGCLVVDFSPLRAPSAWVESVVRALPEDIPVCQVDAHNIVPVWCGSDKLESHARTIRPKLFSKTVKYL
TEFPPVIIHPFQNEMDTNCQSMIDWQSIKANHFVDSSVDAVTWATPGTKAAFEVLRSFIEERLKGFDAQRNNP
ANPALSGLSPWFHFGQISPQRSILEVSAVRKSYVRSADIFIEEAFNRRELAENFCFYNRLYDCLQGAREWAQE
TLMKHAKDKRLVAYTRKQLENAETADDLWNAAQRQLVHTGKMHGFLRQYWAKKILEWCAEGPQTAIDWAVYLN
DRFSLDGTDPNGYVGMCGVLLFAFCFRTNNLLAVSHLAFDKLCQTFTYT
>Fasciolopsis_busk__KAA0185550.1
MLYAQRLALKFQVPLHVCFCLVPSYQADTLRHLSFMLGGLMEVEQECHSLNIPFHLINAGDYLTPDEDLISGV
VARWSPDFTTSSIPQLYAQAVSRAICRLVDSLYTGCLVVDFSPLRAPSAWVESVIQNLPENIPVCQVDAHNVV
PVWCGSNKLETHARTIRPKLFSKTAKYLTEFPPVIVHPFQNELSINLRPSMTDWQSIRAGRYVDSSVDVVAWA
NPGTKAAFEVLHSFIKDRLKGFDAQRNNPANPSLSGLSPWFHFGHIAPQRSILEVSAVRKLYGRSADIFIEEA
FNRRELADNFCFYNPLYDCLQGAREWAQETLMAHSKDKREVAYSREQLENAKTTDDLWNAAQRQLVHTGKMHG
FLRQYWAKKILEWCAEGPQTAIDWAVYLNDRFSLDGTDPNGYVGIMWAICGIHDQGWNERKIFGKVRCMTYNG
CKKWFSIPEFVMRFPE
>Felis_catu__XP_003989258.1
MGVNAVHWFRKGLRLHDNPALKECIQGADTIRCVYILDPWFAGSSNVGINRWRFLLQCLEDLDANLRKLNSRL
FVIRGQPADVFPRLFKEWNITKLSIEYDSEPFGKERDAAIKKLATEAGVEVIVRISHTLYDLDKIIELNGGQP
PLTYKRFQTLISKMEPLEIPVETITSELIEKCTTPLSDDHDEKYGVPSLEELGFDTDGLPSAVWPGGETEALT
RLERHLERKAWVANFERPRMNANSLLASPTGLSPYLRFGCLSCRLFYFKLTDLYKKVKKNSSPPLSLYGQLLW
REFFYTAATNNPRFDKMEGNPICVQIPWDKNPEALAKWAEGRTGFPWIDAIMTQLRQEGWIHHLARHAVACFL
TRGDLWISWEEGMKVFEELLLDADWSINAGSWMWLSCSSFFQQFFHCYCPVGFGRRTDPNGDYIRRYLPVLRG
FPAKYIYDPWNAPEGIQKVAKCLIGVNYPKPMVNHAEASRLNIERMKQIYQQLSRYRGLGLLASVPSNPNGNG
GLMGYSPGENIPGCSSSGSCSQGSGILHYTHGDSQQTHLLKQGRSSMGTGLSGGKRPSQEEDTQSIGPKVQRQ
SVN
>Felis_catu__XP_023095565.2
MAAAVVTAAAAAPAPAAGADGASSVHWFRKGLRLHDNPALLAAVRGARCVRCVYILDPWFAASSSVGINRWRF
LLQSLEDLDTSLRKLNSRLFVVRGQPADVFPRLFKEWGVTRLTFEYDSEPFGKERDAAIMKMAKEAGVEVVTE
NSHTLYDLDRIIDLNGQKPPLTYKRFQAIISRMELPKKPVGSVTSQQMESCRAEIQENHDEAYGVPSLEELGF
PTEGLGPAVWQGGETEALARLDKHLERKAWVANYERPRMNANSLLASPTGLSPYLRFGCLSCRLFYYRLWDLY
KKVKRNSTPPLSLFGQLLWREFFYTAATNNPRFDRMEGNPICIQIPWDRNPEALAKWAEGKTGFPWIDAIMTQ
LRQEGWIHHLARHAVACFLTRGDLWVSWESGVRVFDELLLDADFSVNAGSWMWLSCSAFFQQFFHCYCPVGFG
RRTDPSGDYIRRYLPILKGFPSRYIYEPWNAPESIQKAAKCIIGVDYPRPIVNHAETSRLNIERMKQIYQQLS
RYRGLCLLASVPSCVEDLSNPVAEPSSSQTGNVSSAGPRALPSGPASPKRKLEAAEEPPGEELSKRARVAGLP
APELPSRDV
>Ficedula_albi__XP_005039905.1
MGVNAVHWFRKGLRLHDNPALRECIQGADTVRCVYILDPWFAGSSNVGINRWRFLLQCLEDLDANLRKLNSRL
FVIRGQPADVFPRLFKEWNIAKLSIEYDSEPFGKERDAAIKKLASEAGVEVIVRISHTLYDLDKIIELNGGQP
PLTYKRFQTLISRMEPLEMPVETITPEVMKKCTTPVSDDHDEKYGVPSLEELGFDTDGLPSAVWPGGETEALT
RLERHLERKAWVANFERPRMNANSLLASPTGLSPYLRFGCLSCRLFYFKLTDLYKKVKKNSSPPLSLYGQLLW
REFFYTAATNNPRFDKMEGNPICVQIPWDKNPEALAKWAEGRTGFPWIDAIMTQLRQEGWIHHLARHAVACFL
TRGDLWISWEEGMKVFEELLLDADWSVNAGSWMWLSCSSFFQQFFHCYCPVGFGRRTDPNGDYIRRYLPVLRG
FPAKYIYDPWNAPESIQKAAKCIIGVNYPKPMVNHAEASRLNIERMKQIYQQLSRYRGLGLLATVPSNPNGNG
NGGLMGYSPGESISGCGSTGGAQLGTGDGHTVVQSCTLGDSHSGTSGIQQQGYCQASSILHYAHGDNQQSHLL
QAGRTALGTGISAGKRPNPEEETQSVGPKVQRQSTN
>Ficedula_albi__XP_005040598.1
MSRAAGTAICLLRCDLRAHDNQVLHWAQHNADFVVPLYCFDPRHHLGTHCYGLPKTGPHRLRFLLESVKDLRE
TLKRKGSTLVVRKGKPEDVVRDLITQLGSVTAVVFHEEATQEELDVEKGLCQVCRQHGVQIHTFWGSTLYHRD
DLPFRPIDRLPDVYTHFRRALECGAKVRPTLRMPDLLKPLAPGLEEGNIPTMEDFGQKDPVTDPRTAFPCSGG
ETQALMRLQYYFWDTNLVASYKETRNGLVGMDYSTKFAPWLALGCISPRHIYEQIQKYERERTANQSTYWVLF
ELLWRDYFRFVALKYGRRIFSLKGLQSKEIPWKKDLQLFERWKEGRTGVPFVDANMRELSATGFMSNRGRQNV
ASFLTKDLGLDWRMGAEWFEYLLVDYDVCSNYGNWLYSAGIGNDPRDSRKFNMIKQGLDYDGNGDYVRLWVPE
LQAIKGADIHTPWALSSAALSQAGVTLGETYPQPVVTAPEWSRHIHQRPGGSPHPRGGRGPAQHKDRGIDFYF
SRKKDAC
>Ficedula_albi__XP_005047216.1
PLGPAPALCRSVHWFRRGLRLHDNPALQEALRDATSLRCIYILDPWFAASSAVGINRWRFLLQSLEDLDNSLR
KLNSRLFVVRGQPTDVFPRLFKEWGVTRLTFEYDSEPFGKERDAAIIKLAKEAGVEVVIENSHTLYDLDRIIE
LNGHKPPLTYKRFQAIISRMELPKKPVSTVISQQMETCKVDIQENHDDVYGVPSLEELGFPTDGLAPAVWQGG
ETEALARLDKHLERKAWVANYERPRMNANSLLASPTGLSPYLRFGCLSCRLFYYRLWELYKKVKRNSTPPLSL

YGQLLWREFFYTAATNNPKFDRMEGNPICIQIPWDRNPEALAKWAEGKTGFPWIDAIMTQLRQEGWIHHLARH
AVACFLTRGDLWISWESGVRVFDELLLDADFSVNAGSWMWLSCSAFFQQFFHCYCPVGFGRRTDPSGDYVKRY
LPKLKGFPSRYIYEPWNAPESVQKAAKCIIGVDYPKPMVNHAETSRLNIERMKQIYQQLSRYRGLCLLASVPS
CVEDLSGPVTDSALGQGCSTSTAVRLSQAEQASPKRKHEGAEEPCPEELYKRAKVADLPASEIPGKSL
>Ficedula_albi__XP_005059365.1
MLHRTIHLFRKELRLHDNPVLLAALQSSEALYPVYILDRAFLTSSMHIGALRWHFLLQSLEDLHKNLCQLGSC
LLVIQGEYETVLRDHIQRWNITQVTLDAEMEPFYKEMEANIQRLGAELGFEVLSLVSHSLYDTQRILDINGGS
PPLTYKRFLHILSLLGDPEVPVRNLTAEDFQRCSAPDADLAESYRVPLPEDLKISPENLSPWRGGETEGLQRL
EQHLTDQGWVASFTKPRTIPNSLLPSTTGLSPYFSMGCLSVRTFFYRLSNIYAQAKHHSLPPVSLQGQLLWRE
FFYTVASATPNFTQMAGNPICLQICWYKDAERLHKWKMAQTGFPWIDAIMTQLRQEGWIHHLARHAVACFLTR
GDLWISWEEGMKVFEELLLDADYSINAGNWMWLSASAFFHQYTRIFCPVRFGKRTDPQGNYIRKYLPILKNFP
SKYIYEPWTASEEEQKQAGCIIGQDYPFPMVNHKEASDHNLQLMRQVREEQHRTAQLTRDDADDPMEIKVKRD
HSEENISKGKVARTTE
>Ficedula_albi__XP_016156003.1
MRRGRGKRKAELTEVPPASRRRTEEEEAIENARRRAAPSVREFKYNKKRVRLVSQGSELKDDARCILYWMCRD
QRVQDNWAFLYAQRLALKQELPLHVCFCLVPKFLEATIRHYRFMLRGLQEVAQECAELNISFHLLLGYAKDVL
PTFVAEHGVGGLVTDFSPLRLPRQWVEDVRERLPEDVPFAQVDAHNIVPCWVASPKQEYSARTIRGKIHAQLP
EFLTEFPPVVRHPHPPSCPAQPIAWEACYSSLQVDHTVKEVEWATPGTAAGMAVLKSFIAERLKSFSTHRNDP
NKAALSNLSPWLHFGQVSTQRAILEVQKHRRNYKDSVDAFVEEAVVRRELAENFCYYNENYDSVQGAYDWAQT
TLKLHAKDKRPYLYSLQELEQGTTHDPLWNAAQLQMVWEGKMHGFLRMYWAKKILEWTRSPEEALQFAIYLND
RYELDGRDPNGYVGCLWSICGIHDQGWAERPVFGKIRYMNYAGCKRKFDVERFERRYAPTRSQ
>Firmicutes_bac_HDS30348.1_photolyase
MESERLRIIRPGEIARGPVVYWMSRDQRVSNNHSLVYAQSFAIREKVPLAVVFCLADNFLVATLRQYDFMLRG
LTETALSLGEKNISFHLLRGNPGTEIPKFVKENKIGMLVGDFDPLKIKREWKNEVAKKISVPFHEIDSHNVVP
CRAVSGKQEYGAYTIRPKIQRLLKKFMVLVPKIVRHPVSFDRNPENIEWEKIIAGLKIDRSVPPVEWIKPGEK
NARAMLKKFLIHGISRYSAERNDPNADAQSNLSPYIHFGQISAITVALETIKKSPDDENRRAFLEELIVRREL
SDNFCWHNENYDNIGGIPGWAKASLREHKSDKREYIYMEREFESASTHDRLWNAAQIEMVKRGKMHGWMRMYW
SKKILEWTKSPDEAIRIAIYLNDKYELDGRDPNGYAGILWSIGGLHDRPWGEREIFGKVRYMNYNGARRKFDV
ERYIERFGA
>Fistulifera_sol_GAX13915.1_photolyase
MIINPDPVHHIPKSELPEWFIPSRSRLLTDPNLTSKSSNAKDGSVVYWMQRDMRARDNWALCLASYFAAQKGL
PLRVVYAFAPPPKRLDPMPSLQDLPMTRRHGNFLLGGLELVHKELEEKKIPLHFVMAESHESVGEEVCRLLLE
ELRACLVVSDFSPLRQFREWMELQATPLLNNAAVPFYQVDTHNIVPVWTAAPNRQVGARTLRPKINNVIKTYL
KEFPVMAGNTQDVELPKFERDVYEAYLKIDETVKDVDWAIPGTDAGLQQFRKFCQVGLPKYNEHRNDPVQPHI
CSNLSAWINHGHISFQRLTMDVYKLNKYPTGVAGFVEEGVVRRELSDNYVYYEPNHYDSLDAAADWAKESLQL
HTADPREHLYSTEELEQGKTHDELWNAAQLQLVREGGMHGFMRMYWAKKILEWTESPEIALKTSQYFNDRYAL
DGKDPNGFVGVGWSIMGIHDQGWQERPVFGKIRYMNYNGCKRKFSIPQYVARYEGALRNAETVPSVKVLGSSA
KSSKGAPKKRVTNTTLDVFLKKKKH
>Fistulifera_sol_GAX23745.1_cry
MHDVSIHWYRNGLRFHDNPTLLDACQQSKTMLPLYVIDPDAPFAQTQGLRPACIRANFVMESIQEVDKKLRNM
NSQMIVILGKPKQVIPEVVALLGASALFYEQEAAAPIRKADAEILQELKSKVGTDCDCKGYPTHSLHPMELYL
SKCKGNVAPSSYGAFTKIFNSLKVPKEVEEVTQVPPLPEDALRKLKDKFGEQLRLPSLEDLGYENTKEKLKNR
AKGGIDFIGGEDAGLALLRYMMSRKQWVATFEKPNTSPNALKVDTTGLSPYVKHGCVSPRTFYHQLSKVYAQF
NSKEISKPPVSLHGQLMWREYNYLMGYSTPNFHQMADNPVMRQIPWDDDPQLLSVWENSQTGYPFIDALMTQF
RETGFLHHLGRHAVACFLTRGDLWQSWEKGAEVFEYYLVDADWSINNFNWQWLSCSAHFYQYFRCYSPVAFGK
KTDPDGTYIRKWLPMFKDMPSKFIYEPWEAPLAVQEKCGVIIGKDYPYPIVDHKIVSKDNMSRMKEAYDSQKN
GLPMPESQPVETNEKRITFADSSNSEDASQEPDSKRRKK
>Fistulifera_sol_GAX28242.1_photolyase
MSTVAKKASCQAVYYWFRNDLRLHDHPALTAAIQRASQLQVPLIPVYCFDPRYFGSEAYSEYGSWKCSPRRAQ
FVLESVSDLQQRLMNAGSNLVVVTRQTPEDYFMTCKTTHHPKAFLFYQEEVCREETTIAKKVTQHFDRQNVQA
IWGSTLYELSELPYYDAPESMPDTFTPFRNKVEKKCVIPKPLPAPTKLPPFPADLTTQPASKLTLADIGYEGM
LEKLSHDPRGVMEFVGGETAGLARVKDYIWTKDLLRHYFDTRNGMLGADYSSKFAPWLAHGCISPRFVAQQCQ
RYEQERVENKSTYWLIFELLWRDFFKFFAWKHGDTLFQPGGTIQSTKRWKTFEKSLQAWKEGKTGYPLVDANM
RELNATGFMSNRGRQNVCSFLCLELMQDWRLGADYFESTLLDYDVYSNWGNWCSGAGMTGGRINRFNIVKQSK
DYDQHGEYVKHWLPELKNVPANLVHEPWKMTQFQQTEYNCRLGVDYPHPIVKPFDATPKQGPDTKGGRANGQK
GKLDTNNPNRSRGQRRDMKSLNKGSYRVEG
>Fragilariopsis_cyl_OEU14018.1_FAD_bindin
WMQRDMRTIDNWALNLAQHHAEKSNLPLRVVHVMTTPPPSSDDDDDEPPPLEQLRTTERHGRFLLGGLQCVHN
ELNKKNVPLHIIHNSYDTQHQQHPNVLENFLNDYNPSVVVCDTNVLRHVRRWNESPVLKNALDAINVPLYQVD
AHNIVPVWYASPKREVGARTLRSKLHRLVDECLQNKDHKNGSIPAFSHEQFLDWDDSVKDVLTKDQKPGTAGG

RKQFDDFCNTGLRHFSMLRNDPNNDQISSSLSPWFNHGHLSFATCLRYLKTHNRDAEGKASFIEEGFVRRELS
DNFLWYAPDTYDTLEAGAQWAQDSLELHTSDPREYIYTLNEFESGKTHDDLWNAAQIQMMNTGKMHGFLRMYW
AKKILEWTSTPADALQYAQYFNDKYSLDGRDPNGFCGVAWSIFGNHDMGWKEREIFGKIRFMNYKGCKRKFKV
DEFVRK
>Fragilariopsis_cyl_OEU16032.1_FAD_bindin
MDLIGCGVCSGFFYQYFRCYSPIVFQKKNDPNGNYIRKYVPELKDLPSKYIYAPWEAPTAILDKAGVKLGKNY
PRPVVDHKVISKENMNRMNLAYVDHKAREAASVAAAKEKKPSKKKQRTS
>Fragilariopsis_cyl_OEU23746.1
MKPSRTISTTSSFSTRVELFFKNPDDLKDRIRFLQSEGISRFNIVNKDKKDTMNVWIDSIREVYPESNICAHY
SLKYNKVPRKGIAEQRDPFLKDLEEDYCNADEILLISGSGKKTTQWNTIEALKAVQDIKSTSAESSSKKTKPK
ISVAYNPYFPSSSDQEEENRRLEEKFATGCVSKIYLQFGTDLNSLKNGIEFCYSKAKELNYDDISIAGSLFLP
TKQLIAQQKFRPWNGVFLSQEFLSGPEHATAIVSEMIKIYMKNNIELLWEAPGIRTEKDMGMVHDIMERAVGS
KDDEITGNKETVSSSTTGTSNSDIDISATKRPKLMINNARSSLEKKNIIRSDDPCLLIFGTHDVRLQDNKAVE
EATRRHKIVLPVFLWTKEDSDGMWSAKGAIAVCLQDALYSLETSLQSFDLHLECRLCTNTDDGPEHGVTEIRD
LIDKVGAKAVFWNKEFTPEGRKRGDFRKDFLIKRGVQVYETQSSLLYDPDKIELSSDFDGGHFATLMPFLNNC
RKNFGPPPRPTPRVDTFRLLEETSLPTYTDSISTSTVNDLNIVKVTGNHKWDEPIRKRFPMSEKAANDAMEVF
ARNGMKKYEMERSRADQDGATSQLSPHLRIGTLSPNQLYWRIEDSGLSPKTLARRLIWRDLAYYQLSVFPDMR
TRCIRSHYEDMEWVDGPDEEERRFHAWKTGMTGFPIVDAGMRELYATGWMTQSIRMVVASFLVEYLRVNWTKG
CEWFHYTLVDADSAINAMMWQNAGRSGIDQWNFILSPTTASQDSTGAYTQRWVPELADLPTSALIHRPWEGSI
DVLLQAKVVLGRTYPHRIVTDLKAERQLSVDSTLSMRRKSQEYNDSRGYDLIQLPNGSKTIVFTKKEYRINEN
GGLLKNDPTSVRRKKTTRRKRK
>Fragilariopsis_cyl__OEU15165.1
MASRGVVGLTTQPASRISYSNGQHGGLVFYWFRLGDLRLHDNPGLERAAAYCRETQSNLIPLFCFDPRIFGAA
AITEFGSMKCAPRRAKFVIESIDDLKRSLEERGSKLLISSEKPEELFSRILKDASSETHNKIIYQQEVCSEEI
EIANNVEKLFKSSEAVWGSTLYDLKDLPYDDSLEDMPNGFTAFRNKVEKNCSIRGPLPIPKDLNTISNNQDLT
SMIDQYSIELPSLQDLGYTLDQIDHANFQDPRGVMTFRGGETAGLARVKEYLWDKDLLKTYFDSRNGMLGSEY
SSKLSPWLAHGNISPRYLASECKKYEETRVANKSTYWLVFEVLWRDYFKYFAKKHGDKIFSAGGITGSSPEWK
HYEKNLQAWIEGKTGFPLVDANMRELGATGFMSNRGRQNVCSFLALELNLDWRYGADYFESALLDHDVHSNWG
NWCAGAGMTGGRINKFNIVKQGKDYDQHGEYVRHWLPELKDVPTNIIHEPWKMSQFQQVGSNCRLGVDYPNPI
VKPYSPNPKNSGRSKGDNSKPSPNNNRGRGQRKDMKSMKKGSYDIRG
>Frankliniella_occ_KAE8746400.1_cry_2,_par
MCSSESESESEFITSPSAGSAATSIRPSPALSAATASAAAPRTKHSVHWFRKGLRLHDNPSLRHGLKGATTLRCI
YFLDPWFAGSSNVGINKWRFLLQCLEDLDRSLRKFNSRLFVIRGQPADALPKLFKEWGTTNLTFEEDPEPFGR
VRDQNIMAMCKELSISVVSKVSHTLYELEKIIEKNGGKPPLTYHQFQNVVASMDPPPQAMPTITSRNMQDCYT
PIEDDHDEKYGVPTLEELGFDTEGLKPAVWLGGETEALARLEHHLERKAWVASFGRPKMTPQSLLASQTGLSP
YLRFGCLSTRLFYYQLTDLYKKIKKAVPPLSLHGQLLWREFFYCAATRNPNFDKMQGNPVCVQIPWDHNLEAI
AKWANGQTGFPWIDAIMTQLREEGWIHHLARHAVACFLTRGDLWISWEEGMKVFEELLLDADWSVNAGMWMWL
SCSSFFQQFFHCYCPVRFGRKADPNGDYIRRYLPILKNFPTRYIHEPWSAPENVQRAAKCVVGKDYPKPMVNH
GTASRINIERMKQVYTQLSKYRGS
>Frankliniella_occ_KAE8747724.1_6-4_Photol
MSKATAVHWFRKGLRLHDNPALVAALKSEIELRPVYIFDPWYEKNVLCGPNRWRFLHQSLTDLDKSLRDIGSR
LFVFRGTPEEVFTSLFSEWKVQRLTFEIDIEPYALERDEQIIKIARQEDVEVIQKISHTLYNTELVLKANMDK
PPLTYQKLVSLLQSLGEPPEAVAAPKELLSEQRVSSKLLDSHDYDIPTLEDLGVDQSQLGPCLFPGGETEGLA
RLGRCLERKEWISTFEKPKTIPNSLSPSTTVLSPYIRFGCVSSRLTYHKINEILKVYKKHSKPPVSLIGQMYW
REFYYVVASATPNFDKMIGNKVCCAVPWDENPEFVEKWAQAQTGYPFIDACMRQLRQEGWIHHLARHAVACFL
TRGDLYQSWEVGQKVFEELLLDADWALNAGNWMWMSASAFYHQFYRVYSPVAFGKKTDVLGQYIR
>Frankliniella_occ_XP_026276018.1_photolyase
MSSNKEPPKKKIKVNDSPSSPSVSAQSNLKQRLMLDRKETAESILNFKFNKKRVRILSKATEVPSDCKGIAYW
MFRDERVQDNWAFLFAQKLALKNKVPLHVCFCLLTKFLDGTIRQFKFLLKGLREVQKECEELNIEFHMLYGSG
GDVMPGFVEKNKIGALVIDFMPLRNVMAYADQLKKALPKDVPLCQVDAHNIVPCWEASNKLEYGARTIRPKIH
KQLPEYLTQFPPVIKHPYSGPLKAEKVDWDRCEAHLEVDRSVDEVEWAVPGYSGGIATLESFINKRLKFFGTK
RNDPTQDALSNISPWLHFGQVSAQRCILVVKALRSKYPESVDGYIEEAIIRRELSDNFCFYNPKYDSIEGTND
WAKKTLNDHKKDKRPYLYTREELRDSKTHDDLWNSAQIQLVKEGKMHGFLRMYWAKKILEWTASPEQGLADAI
YLNDRYSLDGRDPSGYVGCMWSVCGIHDQGWGEREVFGKIRYMNYQGCKRKFDISAFVARYGGKVYTKSATMD
KFLTKGKKK
>Frankliniella_occ_XP_026282294.1_cry-1
MATSDNNMCDKATVLWFRRGLRLHDNPALIKALSTKNVFYPIFIFDGETAGITKESSYNRVRFFLESLKDLDR
QLRAKKGKLHFIKGDPVKVFEAIHNIVPLELVSFEQDPEPMWESRDSKAKAYFDQHTIKWTEEISHTLWNPLD
IIKANGGVPPLTYEMFLHILSVVGDPPRPVEDPEWEGVKFGFLPLNIGNEVTVYHSIPTPEQLGYLQTRSSDQ
QINRWIGGEQTALIKLQDRLKVEERAFLDGIYLPNQARPNLLGPSSSQSAALSFGCLSIRKFYWAVHDMFNKI

YLGHTQLNQSITGQFIWREFFYTMSIKNPYYGEMERNPLCLNLPWTKNREDLAKKWENGLTGYPFIDAVMRQL
HQEGWVHHVARNAVACFLTRGDLWISWEVGLRLFLRLLLDADWSLCAGNWMWVSSSAFEQLLDCSSCVCPVNY
GRRLDPQGEYIRRYIPELRNYPIEYLYEPWKAPLKVQEMAGCYIGQEYPERIVDHAVVSEKNRKMMENIRLTL
VEESTPHCCPSNEEEVRQFMWLPESCSDYLCANRK
>Fulmarus_glac__KFV89387.1
RFLLQCLEDLDANLRKLNSRLFVIRGQPADVFPRLFKEWNIAKLSIEYDSEPFGKERDAAIKKLASEAGVEVI
VRISHTLYDLDKIIELNGGQPPLTYKRFQTLISRMEPLEMPVETITPEVMEKCTTPVSDDHDEKYGVPSLEEL
GFDTDGLPSAVWPGGETEALTRLERHLERKAWVANFERPRMNANSLLASPTGLSPYLRFGCLSCRLFYFKLTD
LYKKVKKNSSPPLSLYGQLLWREFFYTAATNNPRFDKMEGNPICVQIPWDRNPEALAKWAEGRTGFPWIDAIM
TQLRQEGWIHHLARHAVACFLTRGDLWISWEEGMKVFEELLLDADWSVNAGSWMWLSCSSFFQQFFHCYCPVG
FGRRTDPNGDYIRRYLPVLRGFPAKYIYDPWNAPESVQKAAKCIIGVNYPKPMVNHAEASRLNIERMKQIYQQ
LSRYRGLGLLATVPSNPSGNGNGGLMGYSPGESISGCGSTGAQLGTGDGHTVVQPCALGDSHTGASGIQQQGY
CQASSILHYAHGDNQQSHLLQAGRTALGTGISAGKRPNPEEETQSVGPKVQRQSTN
>Fulmarus_glac__KFW08398.1
DNWAFLYAQRLALKQELPLHVCFCLVPKFLDATIRHYGFMLRGLQEVAKECTELNIPFHLLLGYAKDVLPAFV
VEHGVGGLVTDFCPLRLPRQWVEDVREQLPDDVPFAQVDAHNIVPCWIASPKQEYGARTIRGKIHAQLPEFLT
EFPPVIRHPYLPSCPAEPIAWEACYSSLQVDRTVKEVEWATPGTAAGLAVLQSFIVERLKSFGSHRNDPNKAA
LSNLSPWFHFGQVSTQRAILEVQKHRGKYKESVDAFVEEAVVRRELAENFCYYNENYDSVQGAYDWAQTTLKL
HAKDKRPFLYDLQELEQGTTHDPLWNAAQRQMVQEGKMHGFLRMYWAKKILEWTRSPEEALRFAIYLNDRYEL
DGRDPNGYVGKLQDRERAIFGKIRYMNYTGCKRKFDVGQFERRYAP
>Fulmarus_glac__KFW11500.1
HLAYFVPRIIELNGHKPPLTYKRFQAIISRMELPKKPVSTVMSQQMETCKVDIQENHDDVYGVPSLEELGFPT
DGLAPAVWQGGETEALARLDKHLERKAWVANYERPRMNANSLLASPTGLSPYLRFGCLSCRLFYYRLWELYKK
VKRNSTPPLSLYGQLLWREFFYTAATNNPKFDRMEGNPICIQIPWDRNPEALAKWAEGKTGFPWIDAIMTQLR
QEGWIHHLARHAVACFLTRGDLWISWESGVRVFDELLLDADFSVNAGSWMWLSCSAFFQQFFHCYCPVGFGRR
TDPSGDYVKRYLPKLKGFPSRYIYEPWNAPESVQKAAKCIIGVDYPKPMVNHAETSRLNIERMKQIYQQLSRY
RGLCLLASVPSCVEDLSGPVADSASGQGCSTSTAMRLSQADQASPKRKHEGAEELCTEELYKRAKVTCLPAPE
IPGKS
>Fulmarus_glac__XP_009574953.1
VLHWAQSNADFVIPLYCFDPRHYLGTRCYGFPKTGPHRLKFLLESVKDLRETLKKKGSTLVVRKGKPEDVVCD
LITQLGSVSAVAFHEEATQEELDVEKGLCQVCSQHGVKIQTFWASTLYHRDDLPFRPIARLPDVYTHFRKAVE
SEVKVRPTLCVADQLKPLAPGVEEGRIPTMEDLGQKDPVTDPRTAFPCSGGETQALMRLQYYFWDTNLVASYK
ETRNELVGMDYSTKFAPWLALGCISPRYVYEQIQKYEKERAANQSTYWVLFELLWRDYFRFVALKYGKRIFSL
RGLQSKEVPWKKDLQLFDCWKEGKTGVPFVDANMRELAATGFMSNRGRQNVASFLTKDLGLDWRMGAEWFEYL
LVDYDICSNYGNWLYSAGIGNDPRDNRKFNMIKQSLDYDGNGDYVRLWVPELQAIKGADIHTPWALNSAALSQ
AGVTLGETYPQPVVTAPEWSRHISQRPQGRSPHPRGRRGPAHTPVQHKDRGIDFYFSRKKDI
>Fulmarus_glac__XP_009575143.1
RCRPPDLGLAECYRVPLPVDLKIPPESLSPWRGGETEGLQRLEQHLTDQGWVTSFTKPRTIPNSLLPSTTGLS
PYFSLGCLSVRTFFYRLSNIYAQAKHHSLPPVSLQGQLLWREFFYTVASATPNFTKMAGNPICLQISWYEDAE
SLHKWKTAQTGFPWIDAIMTQLHQEGWIHHLARHAVACFLTRGDLWISWEEGMKVFEELLLDADYSINAGNWM
WLSASAFFHQYTRIFCPVRFGKRTDPEGQYIRKYLPILKNFPSKYIYEPWTASEEEQKQAGCIIGQDYPFPMV
NHKEASDHNLQLMKQVREEQYRTAQLTRDDTDDPMEMKVKRDHSEENVSKGKVARMTEQTEIPSGITRGEPKN
DKRGEPEAS
>Fulmarus_glac__XP_009580326.1
TRLTFEVDTEPPAHRRDTAVAELAARHGVEVIQEVSHTLYDTERWGRTGYPFIDAIMTQLRTEGWIHHLARHA
VACFLTRGDLWVSWEEGLKVFEELLLDADWSLNAGNWLWLSGSAFFHRYFHVYSPIAFGKKTDRDGAYIRGGL
PGGFDPSPXRAVQEQAGCLVGTHYPRPIVEHGAASKRNLGRMKAARAQK
>Fundulus_hete__XP_012708076.2
MGVNSIHWFRKGLRLHDNPALRDSIRGTGTLYCIYILDPWFAGSSSVGINRWRFLLHCLEDLDASLRKLNSRL
FVIRGQPTDVFPRLFRDWKITRLSYEYDPEPFGKERDAAIQKLASEAGVEVNVRISHTLYNLEKIIELNGGQP
PLTYKRFQAVINRMDAVELPAETITSEIMKNCIPLVTDDHDDKFGVPSLEELGFETEGLSTAVWPGGETEALR
RLERHLERKAWVANFERPRMNSNSLLATPTGLSPYLRFGCLSCRLFYFKLTDLYKKVKKNSTPPLSLYGQLLW
REFFYTTATNNPCFDKMEGNPVCLQIPWDRNPEALAKWAEGRTGYPWIDAIMTQLRQEGWIHHLARHAVACFL
TRGDLWISWEEGMKVFEELLLDADWSVNAGSWMWLSCSSFFQQFFHCYCPTGFGRRTDPSGDYIRRYLPVLRG
FPAKYIYDPWNAPEEVQKAAKCIIGTDYPKPMVNHAEASRINLERMKQIYQQLSSYKGLGLLATVPVNGGVNT
VSSQGPRGSFERPLLQEGTSQTVERRPSTQKRRHEETETECSFKSWKQSK
>Fundulus_hete__XP_012722956.2
MVVNSVHWFRKGLRLHDNPALQEALSGADTVRCVYVLDPWFAGAANVGINRWRFLLESLEDLDNSLKKLNSRL
FVVRGQPTEVFPRLFKEWNVTRLTFEYDPEPYGKERDGAIIKMAQEFGVETVVRNSHTLYNLDRIIEMNNNNP
PLTFKRFQSIVSRLELPRRPLAPIGQQQTNGCLGKISDNHDQLYSIPSLEELGFRTEGLPAAVWRGGESEALD

RLSRYLDKNVWVANTRAKTCSLYASPTGLSPYLRFGCLSCRVLYYNLREIYMKLCKRSSPPLSLYSQLLWREF
FYTAATNNPNFDRMEGNPICVQIPWDQNPEALAKWAEGRTGFPWIDAIMTQLRQEGWIHHRARHAVACFLTRG
DLWISWESGMKVFEELLLDADWSVNAGSWMWLSCSSFFQQFFHCYCPVGFGRRTDPSGDYIRRYVPILKDYPN
RYIYEPWNAPESLQKAVNCVVGVDYPKPMINHAEGSRLNIERMKQVYQQLSHYRGLSLLASVPTIQEEAEPPM
TDESQTSSGPDSPPRDLTEVEASGCSTAPDSSTSVTHPDQEDASHHHPFHYHCTSSGQPSSPNSSPAHSPAQQ
AKPSPASSSCLFVAHKGRQSQRQRGCQCCLAEEEEERMQEEVEVDEERMEEEQTAALQQ
>Fundulus_hete__XP_012724202.2
MAHVCIHWFRKGLRLHDNPALVYALKECKELYPVFILDPYLYNTQVGINRWRFLLGALKDLDCSLRKLNSRLF
VLRGKPEDVFPKLFDKWKVTKLTYEYDTEPYSLSRDEKVTSLAKEHGVDVSYKISHTLYNTERIMEENDGKPP
LTFKRLQAIVQKLGPPKKPIPAPTLENMKDVETHCSEENEKKYGIPTAEELGLDLTSLGEELFPGGEQEALRR
LDEHMKRTGWVCSFEKPQTSPNSLSPSTTVLSPYVTFGCLSARTFWWRLTEVYRGRKHSDPPVSLHGQLLWRE
FFYTCSVGIPNFNKMEANPVCTQVDWDTNPECLAAWREARTGFPFIDAIMTQLRQEGWIHHLARHAVACFLTR
GDLWISWEEGEKVFEELLLDGDWALNAGNWQWLSASAFFHQFFRVYSPVAFGKKTDKNGDYIKKYLPILKKFP
PQYIYEPWKAPRSVQQAAGCIVGKDYPHPIVQHEQISKKNIQRMKEAYAKRSSDSNESPSKKQGVKRKAPSVI
DLLQKKKRKV
>Fundulus_hete__XP_012735645.2
MSASRTVICLLRTDLRLHDNELFHWAQRNAEHIVPLYCFDPRHYVGTYNFNMPKTGPFRLRFLLESLRDLRAT
LLTKGSNLVVRKGKPEEVVADLIKQLGSVSSVAFHEEVASEELTVEKKLKEVCAQLKVKVHTCWGSTLYHRED
LPFPHISRLPDVYTEFRKAVESQSRVRPTLPTPERLSPLPPGLEEGAIPTAEDLQQTEPASDPRSAFPCSGGE
SAALARLKHYFWDTDAVATYKETRNGLIGVDYSTKFSPWLALGCVSPRYIYHQVKKYERERTANQSTYWVIFE
LLWRDYFKFVGIKYGNKMFQVKGLQGKSVPWKMDTKLFDAWKEGRTGVPFVDANMRELAQTGFMSNRGRQNVA
SFLTKDLGLDWRLGAEWFEYLLIDHDVCSNYGNWLYSAGIGNDPRENRKFNMIKQALDYDSNGEYVRRWVPEL
GSIRGADVHTPWNLSSAALSNAGVSLGETYPSPVVTAPEWSRHFSKKPSGAGPSTRGKKGPSHTPKQHRDRGI
DFYFSRSKDL
>Fundulus_hete__XP_012737566.2
MPPSAAGDQAAVRQMLREVLVGREDPEGFFAMCVSVLGHQETRSQFLAIIKPLSTANIRLHSVLSALYQQYFS
KPEDDELELALALSLLEMKDNPMPTPNQSPLQPGIRDDPSSVLLSSKPKSQGRTCTQPQDVHCRGKSALPVQS
SSKSKENLPETKTHKGLHVESADHQSLGTSQDLRVPVSSASFSVPNATSKSDQMMQEGDVNPSEKPRRPKSRR
QRRKAAAQQIVGLACSPSAPPPVLLWFRRDLRLCDNPALIRSLELGAPVIPVFIWCPEEEEGPGVTVAMGGAC
KYWLHQALSCFCASLEQIGSRLIFLKAGGEGNERRSSLHTLKTLVKETGAQTVLANALYEPWLKERDDVVLSA
LRKEGIDCEMFHSYCLRDPFSVSTEGVGLRGIGSVSHFMSCCRQNPGPAIGVPLDPPVSLPSPALWPQGVPMD
SLDLARMPRRKDGTIIDWAANIRKSWDFSEDGAHARLEAFLHDGVYRYEKESGRADAPNTSSLSPYLHFGQLS
PRWLLWDAKGARCRPPKFQRKLAWRDLAYWQLTLFPDLPWESLRPPYKALRWSTNRSHLKAWQKGKTGYPLVD
AAMRQLWLTGWMNNYMRHVVASFLIAYLHLPWQEGYRWFQDTLVDADVAIDAMMWQNGGMCGLDHWNFVMHPI
DAAMTCDPYGSYVRKWCPELAGLPDELIHKPWACPASMLRRAGVAFGQTYPERIVTDLEERRSKSLQDVALVR
KQFQQYVDKRSGCDLVPLPKRLVSKTLGLSHRDGAVVTEGKEFLLPVITRMEFKHQLEDPDADAASNPYNAVL
KGYVSRKRDETIAFLNERDFTASVMYEGAQRQERLERDRRRMEGLPPAPSPRGRARRTPTAKDKFSIVPGGAI
ASTN
>Fundulus_hete__XP_035993809.1
MLLAAWRRPSSISRFTAAFTGGCPKFPPRPPCGSSPSAARSPLPRLAQLHGRAPGSSLFFFFFFFFSLPSTSS
FSGSGSATMAEKKRKASSTAGGKEPGTKRPKLEKEKEKEEKAAGWLQSLVKQQREDKKDMKFNKKRQRVLSDA
AKIKQGSEGVLYWMLRDQRVQDNWALIRAQQLAAKEELPLHVCFCLLVPKSPLSTLRHYAFLLKGLQEVAKEC
KRLNVQFHLLHGAPGEVLPGFVSDQGFGAVVTDFSPLREPQQWLKDVKKKLSKDTPLIQVDAHNIVPCWVASP
KLEYSARTIRGKITNLLPEFLTDFPVIEPHPHTATRTAKPVDWEKTLASLDVDRTVGEPQWAKPGSDAGMAML
ESFIDVRLKLFATQRNDPNMAALSQLSPWIRFGQLSAQRVALQVRRCGKNASESVAPFIEELVVRRELTDNFC
FYNEKYDKVEGAYEWAQKTLRDHAKDKREYLYTREQLEKAQTHDKLWNGAQYQMVTEGKMHGFLRMYWAKKIL
EWTRSPEEALSIALYLNDRYELDGQDPNGFVGCMWSICGIHDQGWAERPVFGKIRYMNYKGCLRKFNVAQFEK
KYCPKKP
>Gadus_morh__XP_030205469.1
MSARTVICLLRQDLRLHDHEVFDWAQSHAEHIVPLYCFDPRHYLGTAVYNLPRTGPFRLRFLLDSIRDLRSAL
LKKGSTLVVRRGRPEEVVADLVRSLPFVSAVVFHQEVASEERAVEQAVMEVCAQMKVKVHTCWGSTLYHRDDL
PFPPARLPDVYTQFRKAVETQSRVRPLFLSPDQVRPLPPGLEEGDVPTLEELGQTEPVSDPRSAFPCSGGESQ
VLARLKHYFWDTDSVASYKETRNGLVGVDYSTKFSPWLALGCISPRYIYHQVRQYESERTANQSTYWVIFELL
WRDYFKFVGVKYGNRLFHLKGLQDKSVPWKKDMTLYNAWKDGRTGIPFVDANMREMAMTGFMSNRGRQNVASF
LTKDLGLDWRMGAEWFEYLLVDHDVCSNYGNWLYSAGLGNDPRENRKFNMIKQGLDYDADGDYVRRWVPELQG
LGGGEVHAPWNLGPSALNHAQVTLGTSYPHPVVMAPEWSRHTGRKAGGRQSDSCPSARGKKGPSHTPKQHKDR
GIDFYFSKSKNL
>Gadus_morh__XP_030209048.1
MVVNTIHWFRKGLRLHDNPALEDCIQGADTVRCVYILDPWFAGSSNVGISRWRFLLQSLQDLDASLRKLNSRL
FVIRGQPTDVFPRLFKEWEIRRLSYEYDSEPFGTERDAAIKKLASEAGVEVTARISHTLYDLDRIIELNGGQS

PLTYKRFQTLISRMAPVEVPADAISSTWKCSTPLADDHDDKFGVPSLEELGFDTEGLLSAVWPGGETEALTRL
ERHLERKAWVANFERPRMNANSLLASPTGLSPYLRFGCLSCRLFYFKLTDLYHKVKKNSSPPLSLYGQLLWRE
FFYTAATNNPSFDKMEGNPICMQIPWDRNAEALAKWAEGQTGFPWIDAIMTQLRQEGWIHHLARHAVACFLTR
GDLWVSWEEGMKVFEELLLDADWSVNAGSWMWLSCSSFFQQFFHCYCPVGFGRRTDPNGDYIRRYIPILKGFP
AKYIYDPWNAPESVQKAAKCIVGVHYPKPMVNHAEASRLNIERMKQIYQQLTCCRGLGLLAAVPSSATGGSEA
ASGLLGFTVEGAHGASTPSGYQSAVTPSQGDWQSAVMTYSQDEAQSTSGPQQTAPGYSPPGSSLMYYTPPALP
AVTQGVRNPLQTSGKRPSEDHGAGTGSKVPRQCSH
>Gadus_morh__XP_030219084.1
MASNKRKAASSAKPPAAKKQKPAPFKLRRGRGWRAGWXKMWPNKEERPDLSCNPKRIRFISDTQSIKHGCEGI
LYWMSRDHRIQDNWALIHAQQLAVEEKLPLHICVCLLVPKSELSTLRHYNFMLKGLEEVAKECKELDIQFHLL
RGAPGEVLPGFVSDHGLGVVVTDFSPLRSPLKALESLRKSLPEDIPLIQVDAHNIVPCWVASIKLEYAARTIR
GKITKLLPEFLTEFPVVRKHSYTATRPAKPVDWSEALGSLQLDRTVGEVDWALPGSAAGSAMLESFIDLRLKH
FANHRNDPNSAALSQLSPWIRFGHLSAQRAALQVQRCGKAFSESVAPFIEELVVRRELTDNYCFYNDNYDNIK
GAYEWAQKTLKDHAKDKREYLYTQADLEKAKTHDKLWNGAQYQMVSEGKMHGFLRMYWAKKILEWTSSPEQAL
SIALYLNDRYSLDGQDPNGFVGCMWSICGVHDQGWAERAVFGKIRFMNYKGCLRKFDVAKFETKYCPKKL
>Gadus_morh__XP_030221581.1
MNVKRTIAGAADNIASLSNTLQQLVLGGEDPGGFFGICVSSMGHSETLSVFPSLIQPLASVDPSQHSTLIAIY
VEYFSKDEEDELEVALALSLLDVKPQQKPACRPEPRPPVWQGGYPAKVGGSIPGGATKSKTTTLPGLSYAQSA
ATGGRGRRPSENLPKTGSGVPSSTPPTHPSRGRAPLPTDTHSTTSQTTRHKHVPGLPAPPTFHQSDRRQDAVN
DDARDPLDTEGTCPDQSQKPKRSKNRRQRGKGCGQRAVVGLPHSPSATPPVLLWLRRDLRLHDNPALIGSLQA
GAPVVPVFIWSPEEEEGPGVTMATGGACRYWLHQALACLQGALQLLGSHLVLLKADPGSSLGALLGLVGETGA
RTVLATALYEPWLRERDQRVEAGLRRAGVAWRMVHSYCLRDPYSVSTQGVGLRGLGSVSHFISCCEQNPGPAL
GPSLDPPVSMPTPSQWPPGDPLDALGLARMPRRKDGTVIDWAANIRASWDFSEQGAHARLEAFLQDGVYRYEK
ESGRADSPNTSCLSPYLHWGQISPRWLLWDAKSARCRPQKFQRKLAWRDLAYWQLSLFPALPWESLRPAYKAL
RWSSDRGHLKAWQRGRTGYPLVDAAMRQLWLTGWVNNYMRHVVASFLIAYLHLPWQEGYLWFQDTLLDADVAI
DAMMWQNGGMCGLDHWNFVMHPVDAALTCDPGGSYVRKWCPELAALPDDIIHKPWRCPASILRRAGVAFGHTY
PEPIVTDLEARRGQSLQDVAQVRSTLSEYVDERSGCDLVPLPPRLVAEALGSAGGDQLRTGGEQFLLPVITRM
EFKHRRGDPGEDAASNPYDAVLKGYVNRKRDETVAFLNQRDFSASVMNEGAQRRERLDSDMRILEGLPRAPPG
RGRARPTPAAQGQTS
>Gadus_morh__XP_030222357.1
MVVNSVHWFRKGLRLHDNPSLQEALNGADTVCCVYILDPWFAGSANVGVNRWRFLLESLEDLNNSLRKLNSRL
FVVRGQPTDVLPRLFKEWNVTRLTFEYDPEPYGKERDGAIIKVAHEMGVETIVRNLHTLYNLDRIIEMNNNSA
PLTFKRFQTIVNRLDLPRRPLPTVTPQQMERCKTLIAKDHDQHYSVPSLEELGFKTEGLPPAVWRGGESEALE
RLNKHLDRKAWVANFEHPRGVCSLFASPAGLSPFLRFGCLSCRVFYYNLRELYMKLRKRCSPPLSLFGQLLWR
EFFYTAATINPNFDRMEGNPICVQIPWDQNPEALAKWAEGRTGFPWIDAIMTQLRQEGWIHHLARHAVACFLT
RGDLWISWESGMKVFEELLLDADWSVNAGSWMWLSCSAFFQQFFHCYCPVGFGRRTDPSGDYIRHYIPILKDY
PNRYIYEPWNAPLSVQKAAHCVVGVDYPKPMINHAEASRLNIERMKQVYQQLSHYRGLSLLASVPTIQEEAEP
PMTDESQTSSGPDSPVHEPAACPLVPDSSRACMSSPAPGLNVSAAHTPSTSTREQAGVGAAAPPQAYASTSTD
PSTFPVLSSAAAITKLSIDVPAAVFLHPPPIPFPSSNPATGQTPARDPASSPASPNLSPASKPFRALSPRPVT
LAGHHAPLLGQRKKGPLRKHRRSQRARGVPGCVPGPPVEGGKAMDGAGGEGEECWREECMDEDMIQDSPNDQQ
>Gadus_morh__XP_030233389.1
MAHVCIHWFRKGLRLHDNPALMSALRGCKEIYPLFILDPCIHNKESMGVNPWRFLMGSLQDLDGSLRKLNLRL
FVVRGKPQDILPRLFQKWGVSRMTYEYDTEPYSRSRDLKVSELAKEHGVEVVYKISHTLHDVDRIIEENNGKA
PLTYGRFQTVLKTLGPPKRPIPSPTVEDIKDVKAPCVESHEEQQYGLPSLEELGHDLSCLQEAQFPGGEEEAL
RRLEESMERTGWVCSFEKPQTAPNSLSPSTTVLSPYLTFGCLSARTFWWRLADVYQGKKHSAPPVSLHGQLLW
REFFYTASVGVPNFDRMLDNPVCTQIDWDTNPEYLSAWKEARTGFPFIDAIMTQLRREGWIHHLARHAVACFL
TRGDLWISWEEGKKVFEGLLLDGDWALNAGNWLWLSASAFFHQYFRVYSPIAFGKKTDKHGEYIKKYLPVLKK
FPVEYIYEPWKAPLSVQKAAGCIVGKDYPSPIVEHEVISKQNIQRMKTSYGKRSQGVSESPQPMKAEKRKGPS
VLDMLKNKKKK
>Galbula_dea__NXI34715.1
MPHHTIHLFRKGLRLHDNPSLLAALESSELLYPLYILDRQLITSAMHMGALRWHFLLQSLEDLQRSLCQLGSC
LLVIQGEYESVLRDCVQEWHITQVTLDEDLEPFYKEMEAKIRSLGEELGFEVLSRVGHTLYDTRRILDLNGGA
PPLTYKRFLQVLAQLGDPEVPVREPTAEDFQRCQPPDLGLAERYRVPLPGDLELTPESPSPWRGGETEGLQRL
EQHLTEQGWVRSFTKPRTIPNSLLPSTTGLSPYFSMGCLSARSFFHRLANICAQAKHHSLPPVSLQGQLLWRE
FFYTVASVTPNFTRMAGNPICLQISWCEDAERLHRWKMAQTGFPWIDAIMTQLRQEGWIHHLARHAVACFLTR
GDLWISWEEGMKVFEELLLDADYSINAGNWMWLSASAFFHHYTRIFCPVRFGRRTDPEGHYIRKYLPVLQNFA
SKYIHEPWTAPEEEQKQAGCIIGQDYPFPMVNHKEASEHNLQLMKQVREEQYRTAQLTR
>Galbula_dea__NXI35518.1
CIYILDPWFAASSAVGINRWRFLLQSLEDLDNSLRKLNSRLFVVRGQPTDVFPRLFKEWGVTRLTFEYDSEPF
GKERDAAIVKLAKEAGVEVVIENSHTLYDLDRIIELNGHKPPLTYKRFQAIISRMELPKKPVSTVTTQQMEAC

KVDIQENHDDMYGVPSLEELGFPTDGLAPAVWQGGETEALARLDKHLERKAWVANYERPRMNANSLLASPTGL
SPYLRFGCLSCRLFYYRLWELYKKVKRNSTPPLSLYGQLLWREFFYTAATNNPKFDRMEGNPICIQIPWDRNP
EALAKWAEGKTGFPWIDAIMTQLRQEGWIHHLARHAVACFLTRGDLWISWESGVRVFDELLLDADFSVNAGSW
MWLSCSAFFQQFFHCYCPVGFGRRTDPSGDYVKRYLPKLKGFPSRYIYEPWNAPESVQKAAKCIIGVDYPKPM
VNHAETSRLNIERMKQIYQQLSRYRGLCLLASVPSCVEDLSGPVTDSASGQGCSTSTAIRLAQADQASPKRKH
EGAEELCTEELYKRAKVTSLPAPEIPGKS
>Galbula_dea__NXI41525.1
VDYDVCSNYGNWLYSAGVGNDPRGSRKFNMVKQGLDYDGNGDYIRLWIPELQGLKGADIHTPWALNSAALSQA
GVTLGQTYPQPVVTAPEWNRHISQRPQGRSSQPRGRRGPAHTALQHKDRGIDFYFSHKKDV
>Galbula_dea__NXI42337.1
RFLLQCLEDLDANLRKLNSRLFVIRGQPADVFPRLFKEWKIAKLSIEYDSEPFGKERDAAIKKLASEAGVEVI
VRISHTLYDLDKIIELNGGQPPLTYKRFQTLISRMEPLEMPVETITPEVMEKCTTPVSDDHDEKYGVPSLEEL
GFDTDGLPSAVWPGGETEALTRLERHLERKAWVANFERPRMNANSLLASPTGLSPYLRFGCLSCRLFYFKLTD
LYKKVKKNSSPPLSLYGQLLWREFFYTAATNNPRFDKMEGNPICVQIPWDKNPEALAKWAEGRTGFPWIDAIM
TQLRQEGWIHHLARHAVACFLTRGDLWISWEEGMKVFEELLLDADWSVNAGSWMWLSCSSFFQQFFHCYCPVG
FGRRTDPNGDYIRRYLPVLRGFPAKYIYDPWNAPESVQKAAKCIIGVNYPKPMVNHAEASRLNIERMKQIYQQ
LSRYRGLGLLATVPSNPNGNGNGGLMSYSPGENISGCGNTGGAQLGTGDGHTVVQPCALGDTHTGASGIQQQG
YCQASSILHYAHGDNQQSHLLQAGRTALGTGISAGKRPNPEEEIQSVGPKVQRQSTN
>Galbula_dea__NXI43595.1
DNWAFLYAQRLALKQELPLHICFCLVPKFLDATIRHYGFMLKGLQEVAKECAELNIPFHLLLGYAKDVLPTFV
VEHGVGGLVTDFCPLRLPRKWVEDVREQLPEDVPFAQVDAHNIVPCWVTSQKQEYSARTIRGKIHAQLPEFLT
EFPPVICHPYPPSCPAEPIAWEACYSSLQVDRTVKEVEWATPGTAAGLAVLRSFITERLKSFGSHRNNPNKAA
LSNLSPWFHFGQVSTQRAILEVQKQRSKYKESVDAFVEEAVVRRELAENFCYYNENYDSVQGAYNWAQTTLKL
HAKDKRPFLYELQELEQGTTHDPLWNAAQLQMVWEGKMHGFLRMYWAKKILEWTCSPKEALRIAIYLNDRYEL
DGRDPNGYVGKLQDRGMGWWWGCLWSICGVHDHGWAERAIFGKIRYMNYAGCKRKFDVGQFERRYTP
>Galdieria_sul_cryDASH_fa_XP_005708318.1
MSAFATLNCKYDNYYFRNIKGYSSFQKRSTYVKLTRGNSSLFWRKQIREAKVFRLYSKIRGDLHRVPESLNGG
TAILWFRNNLRLSDNSCLDLANTAEAVLPLYVFDKRSLVRNRLKQQRCGPFRYSFVKESVEQLQNNLRGLFSD
LLVEVGTAAEVIRELCAKYSINHIVAPKMIKVSQVATELHLNACFVWDCTLLSFDELIVNEYQHSFETFYNSV
ISKGLPKIFELDNSQRPFLKPLPFGYKPSFAIPLPHLVNDLGVENLCTPYEWPFPEPRAVYPFRGGEDSAKER
LTDYFVKKNGLQLIADLENYSGVMDSSTKLSPWISIGCVSVRQVYWEGYRSYMKNMDSQVANLWLKRFLWRDF
LYLLCLQEGSAVFRDRTVNEDAPEFPLYKQSATPYETICNQAIQTWLEGRTGYPYIDAHIKELITTGYISFRG
RLNVSSFLVHELDGIWQLGAEFFEHYLIDHDPVINWGYWYHLYEKKQIGVESIQVDTIEEANKYDPDGFYVKK
WLNQLVLLPPPFCHEPYLLSEEEQNQFQVILGSDYPEPVLFPTVSEEIERARQFLSSSS
>Galdieria_sulph_XP_005706429.1
MKTTKEGSPVAILSFVSHIGFSVCKISLSCKHSVTLLPCTKNGQRLTLGGRRSKAKGYERIVATGTDALSFAS
PEQTKQPKNHKSDNQRHLKTAPLFSPLGENFGESAGQAYKGSKIIVWFRNDLRLHDNPALFRAAEEGSLILPV
YCFDPRQFGKTSFGFEKTGRYRAQFLIDSVEDLRKSFRAKGSDLIVRLGRPEEVLPELCRQTGCKRVFCHREV
TYEDLIVEEDVGDALESIGVEMTLLWSNTLYQAEDLPFQVENMPDIYTKFRESVETGGKIREPLELSEAFPPR
PRCEPGEIPTLTELGLDASPERIPGESNPRSIHGFRGGESESLKRMEDYLSEMRSTEISSTTAGAYLGADFSC
KISPWLALGCISPRKIYHEVNGSSVPEDVRKTTYFELVWRDFFRFITQKYGNIRLEKSRHSSSSKRKVPSGNQ
FVATCG
>Galdieria_sul_XP_005703413.1_photolyase
MNCPVSNQRVRVLNNKPVQESREFVLYWMQAAVRLTYNYALQYAVFSANQLGKPLLICFGLTESFPEANARHY
AFLLEGLQDVQRQLAERKLSMVVFHSEPEQMVEKLFHKAVQVVVDMNYLRVTKNWRQRVASNIDCSFIQVETE
CVVPVELVTDKEEYAARTIRPKLWKHANKFLIPWADTIHVENQLVDIPQIDELTVLDLQGEAKELVERLNVDK
TVPPVSHFVGGETEAKRRLAEFCRSKLRDYEKKRNDPSLNCGKVVFLVFVCWRAKYLCLVSHMSPYLHFGHIS
PVEIVCQVQQAQASKTCKDAFIEEVFIRRELSFNFCYYCDKYDAFDCLPTWAKQTMSEHSKDPRQFSYTLEQL
ETAVTHDPYWNAAQLEMVQTGKMHNYMRMYWGKKVIEWTTNPEEAFRILLYLNNKYELDGRDPNSFTGVAWCF
GKHDRAHMERPITGKLRYMSAEGLRRKFNIESYVRQYLTSNQQNKILSVFYGNNNNNNPHKRKRTTK
>Galdieria_sul_XP_005706285.1_photolyase
MAIPSLKQWQRNILFAFHNDCSPSCNTPRARTLLASHIIQKHIHMDTGTHIKGPTIVWFRKSLRLHDNPTLQR
AVQLASELFPLFILDPYYINPDKIGFQRFRFMLECMKDLDHSLRKYQSKLLVIYGEPVNVLENCCKTWKCSYL
CFEKDSDIYSRNRDEKVLQRMKGLGVQCFVESGHTLYDLDMLVAKLNGNSPPTQMTSFLKFIEQIGPPPKPIE
TVRNIPPLRKELLSSISISKVPEIEEIPGYELITEPTPCPFQGGESKALERMQQALNREQGKWIVRFSKPHTS
PVSLDPPSTTVLSPYIKCGALSVRLFFWELKRVEDSFSVKTKPPVSLIGQLYWREHFYLLGYTIPHFDQIQGN
PLCKTIPWEHRQEWFQAWEQAQTGYPFIDAAMTQLKQWGWLHHLTRHVVACFLTRGDLWISWEKGKETFEKYL
IDGDWSINAANWMWLSCSAFFTRYFRVYSPIAFPKKYDPHGNYIRYFLPVLRNMPNKYIYEPWKAPLKVQKEV
NCILGKDYPLPIVDHEEMKKRNIQKMIQVFQNKS
>Galdieria_sul_XP_005707133.1_photolyase

MWLSSERKVTKKKRYKNATYTTQLVEHIEGTLTMEVNNNLNSIMSSEEEECNSPAKKSHGNRVSKSKQESCKD
PEMISYGDSHKLDIQSSVKAQVQIESAGTGQRESSLVWFRGHDLRLDDNPALLAALERKTKCYAIFLWSPNSF
GRWYPSKPWLWWLRSSLLVLERELIKLGVKLLCRNGLAEEELPSVVREIGATVIFWNRAYEPIYLKAEERVIS
MFSGSTVKLVSFKSELFVEPWELQNDHSPFFPDFHSYMRAWMLQLPPPEPLSISKQYCTSLPYTELENQVGQL
DLLSKEDIEAFESKNNWKPGCRQAQRTLKAFLSDSFDKFGAARYRRSLTGTSKLSPHLKFGELSVKRIFYMVR
KRVANNRENWYLYSQAAKAFLKNLCLREYAYHILYYYPNAIDTPLQSVFNLFPWERDYSLLDRWSVGHTGYPM
VDAAIRELRSTGWIHNILRFLLASFLTRYLLLPWQLGAHFLYSHLIDGDLAANTLGWQWTAGCNTDSFPLSCL
VNPIGMGRKYDSSGTYVRQWIPELASLQDKYIHCPWQAPEDVLANANITLGKTYPFPIVDVSFARSRAKEALR
IMRQVVDGQHLPKSLSSPTVNDVIEEWPDEEIGEESNRMLEETSMTEQGSSALSLLPSLWQLLQYDSPPSLPV
SSLEHLLFNSENEDDELMLNDLSFQKEDETAYFHLESRDDSSSYMTNKLDQLTAAQSNISNFVGSYSPSMGQQ
HVSQVFLPTWMSTPYSAQERRSYSEDVHLETAVYAQNNFHRRIPSAETNLNYLYQPFLDNSTQETVHMGPFYG
NPVSHKLESYPSEHRMTDVSPSSSMLTFPSTKRKVKATAHSSAPMEESTQKWNDISSHPDDSPEIITINMEGM
QESRRETTGTTKRVRQTQLKRKQLMTAHSSHNRTSLSQKTSSSDWRQPPQTSEERRAMLEIVAKDNQHEGQLF
AQFLLQYYELTDNVDRRISRDFVRVRNMKDLFHQWCPDKKKLLKIYKIKSFLSDILNLEVTGEWDRHLHGGVR
GPYVYGICPVEQKEQTMEAP
>Galemys_pyre__KAG8513992.1
FLLQCLEDLDANLRKLNSRLFVIRGQPADVFPRLFKEWNITKLSIEYDSEPFGKERDAAIKKLATEAGVEVIV
RISHTLYDLDKIIELNGGQPPLTYKRFQTLISKMEPLEIPVETITSEVIEKCITPLSDDHDEKYGVPSLEELG
FDTDGLSSAVWPGGETEALTRLERHLERKAWVANFERPRMNANSLLASPTGLSPYLRFGCLSCRLFYFKLTDL
YKKVKKNSSPPLSLYGQLLWREFFYTAATNNPRFDKMEGNPICVQIPWDKNPEALAKWAEGRTGFPWIDAIMT
QLRQEGWIHHLARHAVACFLTRGDLWISWEEGMKVFEELLLDADWSINAGSWMWLSCSSFFQQFFHCYCPVGF
GRRTDPNGDYIRRYLPVLRGFPAKYIYDPWNAPEGIQKVAKCLIGVNYPKPMVNHAEASRLNIERMKQIYQQL
SRYRGLGLLASVPSNPNGNGGLMGYSPGENIPGCSSSGSCPQGSGILHYAHGDSQQTHLLKQGRSSLGTGLSS
GKRPSQDEDTQSPKVQRQSTN
>Galemys_pyre__KAG8519553.1
GRSGSGPGVEAAMATAVVTAAAATSAPAPLADGASSVHWFRKGLRLHDNPALLAAVRGARCVRCVYILDPWFA
ASSSVGINRWRFLLQSLEDLDTSLRKLNSRLFVVRGQPADVFPRLFKEWGVTRLTFEYDSEPFGKERDAAIMK
MAKEAGVEVVTENSHTLYDLDRIIELNGQKPPLTYKRFQAIISLMELPKKPVGSVTSQQMEGCRAEIQENHDE
TYGVPSLEELGFPTEGLGPAVWQGGETEALARLDKHLERKAWVANYERPRMNANSLLASPTGLSPYLRFGCLS
CRLFYYRLWDLYKKVKRNSTPPLSLFGQLLWREFFYTAATNNPRFDRMEGNPICIQIPWDRNPEALAKWAEGK
TGFPWIDAIMTQLRQEGWIHHLARHAVACFLTRGDLWVSWESGVRVFDELLLDADFSVNAGSWMWLSCSAFFQ
QFFHCYCPVGFGRRTDPSGDYVRRYLPKLKGFPSRYIYEPWNAPESIQKAAKCIIGVDYPRPIVNHAETSRLN
IERMKQIYQQLSRYRGLCKQPLPTSGPHPCTPRHPPQPTEGKKGLLASVPACTEDLSNPVAEPSSSQAGSTSS
AGPRPLPSGAASPKRKLEAAEEPPGEELSKRARVAGLPAPEAPSKDV
>Galendromus_occ_XP_028966480.1_photolyase
MRLSISALYLRIRLYSSKMPKTDERVTAVNHFEHEKNILYLMRRDKRIFDNHCVELGFKLSYKSKTKFYTGVE
FSKLKINERQKTFVIEGLQEMEEACREHNIYFDLIDNLEKFVEKRDIDCIIMDFSPLRECEAYRNEVEEMCSD
KELSLYVVDAHNMVPCKLLDVYKRTSKAVKIQLFNHWDDYLSDFKPLEKHLYNKKSETSTQSNDFPKKSVKNI
FKGGYSHGMDTVEHFFDKKFAIYAKNRNNPDVDALSNLSPWISSGQISSQKVIYLATKRFGKKNENYISFINE
IFAVKENSEHFCLHEKNYDNIDGALQWAKDSLNLHRNDKRERIYDLETLEMGKTKDQAWNAAQRQLLAEGKLH
GYCRMYWAKQLLKWTKTPEEAIKTACYLNDTFAIDGNCPNGFLGPMWSICGSMDQGFKERPVTGKIRPMNAFK
APLYISKWSNYSPSSSKSSSTSSSSKK
>Galeopterus_vari__XP_008561553.1
FLLQCLEDLDANLRKLNSRLFVIRGQPADVFPRLFKEWDITKLSIEYDSEPFGKERDAAIKKLATEAGVEVIV
RISHTLYDLDKIIELNGGQPPLTYKRFQTLISKMEPLEIPVETITSEVIEKCTTPLSDDHDEKYGVPSLEELG
FDTDGLSSAVWPGGETEALTRLERHLERKAWVANFERPRMNANSLLASPTGLSPYLRFGCLSCRLFYFKLTDL
YKKVKKNSSPPLSLYGQLLWREFFYTAATNNPRFDKMEGNPICVQIPWDKNPEALAKWAEGRTGFPWIDAIMT
QLRQEGWIHHLARHAVACFLTRGDLWISWEEGMKVFEELLLDADWSINAGSWMWLSCSSFFQQFFHCYCPVGF
GRRTDPNGDYIRRYLPVLRGFPAKYIYDPWNAPEGIQKVAKCLIGVNYPKPMVNHAEASRLNIERMKQIYQQL
SRYRGLGLLASVPSNPNGNGGFMGYSPGENIPGCSNSGSCSQGSGILHYAHGDCQQSHLLKQGRSSTGTSLSS
GKRPSQEEDTQSIGPKVQRQSAN
>Galeopterus_vari__XP_008568740.1
MGGAGGASRGRAEVAGVVSSVMAAAVMTAAAAVPAPAPAAGADGASSVHWFRKGLRLHDNPALLAAMRGARCV
RCVYILDPWFAASSSVGINRWRFLLQSLEDLDTSLRKLNSRLFVVRGQPADVFPRLFKEWGVTRLTFEYDSEP
FGKERDAAIMKMAKEAGVEVVTENSHTLYDLDRIIELNGQKPPLTYKRFQAIISRMELPKKPVGLMTCQQMDS
CRAEIQENHDETYGVPSLEELGFPTEGLGPSVWQGGETEALARLDKHLERKAWVANYERPRMNATSLLASPTG
LSPYLRFGCLSCRLFYYRLWDLYKKVKRNSTPPLSLFGQLLWREFFYTAATNNPRFDRMEGNPICIQIPWDRN
PEALAKWAEGKTGFPWIDAIMTQLRQEGWIHHLARHAVACFLTRGDLWVSWESGVRVFDELLLDADFSVNAGS
WMWLSCSAFFQQFFHCYCPVGFGRRTDPSGDYIRRYLPKLKGFPSRYIYEPWNAPESIQKAAKCIIGVDYPRP

IVNHAETSRLNIERMKQIYQQLSRYRGLCLLASVPSCVEDLSHPVAEPSSSQPGSISSAGPRPVPSGPASPKR
KLEAAEEPPGEDLNKRARVAESPAPELPSKDV
>Gallus_gallus_NP_989575.1_cryptochro
MAAAASPPRGFCRSVHWFRRGLRLHDNPALQAALRGAASLRCIYILDPWFAASSAVGINRWRFLLQSLEDLDN
SLRKLNSRLFVVRGQPTDVFPRLFKEWGVTRLTFEYDSEPFGKERDAAIIKLAKEAGVEVVIENSHTLYDLDR
IIELNGNKPPLTYKRFQAIISRMELPKKPVSSIVSQQMETCKVDIQENHDDVYGVPSLEELGFPTDGLAPAVW
QGGETEALARLDKHLERKAWVANYERPRMNANSLLASPTGLSPYLRFGCLSCRLFYYRLWELYKKVKRNSTPP
LSLYGQLLWREFFYTAATNNPKFDRMEGNPICIQIPWDKNPEALAKWAEGKTGFPWIDAIMTQLRQEGWIHHL
ARHAVACFLTRGDLWISWESGVRVFDELLLDADFSVNAGSWMWLSCSAFFQQFFHCYCPVGFGRRTDPSGDYV
KRYLPKLKGFPSRYIYEPWNAPESVQKAAKCIIGVDYPKPMVNHAETSRLNIERMKQIYQQLSRYRGLCLLAS
VPSCVEDLSGPVTDSAPGQGSSTSTAVRLPQSDQASPKRKHEGAEELCTEELYKRAKVTGLPAPEIPGKSS
>Gallus_gallus_XP_040508605.1_cryptochro
MRHRTIHLFRKGLRLHDNPALLAALQSSEVVYPVYILDRAFMTSSCLLVIQGEYKSVVRDHVQKWNITQVTLG
AEMEPFYKEMEANIRGLGEELGFQVLSLMGHSLYNTQRILELNGGTPPLTYKRFLRILSLLGDPEVPVRNPTA
EDFQRCSPPELGLAECYGVPLPTDLKIPPESISPWRGGESEGLQRLEQHLADQGWVASFTKPKTVPNSLLPST
TGLSPYFSTGCLSVRSFFYRLSNIYAQAKHHSLPPVSLQGQLLWREFFYTVASATPNFTKMAGNPICLQIRWY
EDAERLHKWKTAQTGFPWIDAIMTQLRQEGWIHHLARHAAACFLTRGDLWISWEEGMKVFEELLLDADYSINA
GNWMWLSASAFFHHYTRIFCPVRFGRRTDPEGQYIRKYLPILKNFPSKYIYEPWTASEEEQKQAGCIIGRDYP
FPMVDHKEASDHNLQLMKQAREEQHRIAQLTRDDADDPMEMKLKRDHSEESLTKTKAARMTEQT
>Gallus_gal_NP_989576.1_cry-1
MGVNAVHWFRKGLRLHDNPALRECIRGADTVRCVYILDPWFAGSSNVGINRWRFLLQCLEDLDANLRKLNSRL
FVIRGQPADVFPRLFKEWSIAKLSIEYDSEPFGKERDAAIKKLASEAGVEVIVRISHTLYDLDKIIELNGGQP
PLTYKRFQTLISRMEPLEMPVETITPEVMQKCTTPVSDDHDEKYGVPSLEELGFDTDGLPSAVWPGGETEALT
RLERHLERKAWVANFERPRMNANSLLASPTGLSPYLRFGCLSCRLFYFKLTDLYKKVKKNSSPPLSLYGQLLW
REFFYTAATNNPRFDKMEGNPICVQIPWDKNPEALAKWAEGRTGFPWIDAIMTQLRQEGWIHHLARHAVACFL
TRGDLWISWEEGMKVFEELLLDADWSVNAGSWMWLSCSSFFQQFFHCYCPVGFGRRTDPNGDYIRRYLPVLRG
FPAKYIYDPWNAPESVQKAAKCVIGVNYPKPMVNHAEASRLNIERMKQIYQQLSRYRGLGLLATVPSNPNGNG
NGGLMSFSPGESISGCSSAGGAQLGTGDGQTVGVQTCALGDSHTGGSGVQQQGYCQASSILRYAHGDNQQSHL
MQPGRASLGTGISAGKRPNPEEETQSVGPKVQRQSTN
>Gallus_gal_XP_040534601.1_photolyase
MPRGNGKGRKERDAGREEEEAVGTLEAAVREARRRTAPSVRDFRYNKQRARLVSRGSELKEGAECILYWMCRD
QRVQDNWAFLYAQRLALKQELPLRVCFCLVPAFLDATIRHYGFMLRGLREVAKECAELDIPFHVLLGCPKDVL
PSFVVEHGVGGLVTDFCPLRVPRQWVEEVKERLPEDVPFAQVDAHNIVPCWVASPKQEYSARTIRAKIHSQLP
EFLTEFPPVIRHPHPPPNPPEPIAWDACYSSLQVDRTVTEVAWATPGTAAGLAMLQSFITERLKSFGSQRNDP
NKAALSNLSPWFHFGQVSTQRAILEVQKHRRVYKESVDAFVEEAVVRRELAENFCYYNENYDSVRGAYDWAQS
TLKLHAKDKRPFLYKLPQLEQATTHDPLWNAAQLQMVREGKMHGFLRMYWAKKILEWTRSPEEALQFAIYLND
RYELDGMDPNGYVGCLWSICGIHDQGWKERDVFGKIRYMNYAGCKRKFDVDQFERRYAHCK
>Gambusia_affi__PWA17548.1
MLPKDISTLSGTAAISLRLVLKPPFFITQTRTAAPEMDQQPAEGSAGFWSSATTEQPSPAGTPLLLLLRFLNP
SHLLTAPITAPRLARLPSQARRPKLSFASFGGATMSGKKRKAPTAEGGKEPSAKQPKVEKAEKAAGWLQNLVK
QQREDKKDMKFNKKRQRFLTDTEKIKQGSEGVLYWMQRDQRVQDNWALIRAQQLAAKEELPLHVCFCLVVPKS
ELSTLRHYAFLLKGLQEVAKDCKRLNVQFHLLRGAPGEVLPGFVSDRGLGAVVTDFSPLREPQQWLNDVKKKL
SKDIPLIQVDAHNIVPCWVASPKLEYSARTIRGKITNLLPEFLTDFPPVEKHQQTATRTAKPVDWEETLASLD
VDRTVGEPQWATPGSEAGMAMLESFIDVRLKLFATQRNDPNMAALSQLSPWIRFGQLSAQRVALQVRRCGKNA
SESVAAFIEELVVRRELTDNFCFYNEKYDKVEGAYEWAQKTLKDHAKDKREHIYTREQLEKAQTHDKLWNGAQ
YQMVAEGKMHGFLRMYWAKKILEWTRSPEEALSIALHLNDRYELDGQDPNGFVGCMWSICGIHDQGWAERPVF
GKIRYMNYKGCLRKFNVAQFERKYCPKKA
>Gambusia_affi__XP_043953409.1
MAHVCIHWFRKGLRLHDNPALMAALKDCKELYPVFILDPYLHHTQVGINRQRFLIGALKDLDCSLRKLNSRLF
VLRGKPEDVFPKLFNMWNVTKLTYEYDTEPYSLRRDEKVASLAKEHGVDVIYKISHTLYNIERIIEENNGKPP
LTYKRLQAIVQTLGPPKKPIPAPTLENMRDVKTPHSEKYEDKYRIPTSEDLQLDSRFLGEELFPGGEQEALRR
LDEHMKRTGWVCSFEKPQTSPNSLSPSTTVLSPYITFGCLSARTFWWRLTEVYRGRKHSDPPVSLHGQLLWRE
FFYTCSVGIPNFNKMNQNPVCTQVDWDTNQQNLAAWREARTGFPFIDAIMTQLRQEGWIHHLARHAVACFLTR
GDLWISWEEGQKVFEDLLLDGDWALNAGNWQWLSASAFFHQFFRVYSPIAFGKKTDKNGDYIKKYLPLLKKFP
PQYIYEPWKAPRSVQQAAGCIVGKDYPHPIVQHEQISKKNIQRMKEAYAKRSSDNNESPIKKQGVKRKTPSVI
DMLQKKIRK
>Gambusia_affi__XP_043961334.1
MSASRTVICLLRNDLRLHDNEVLHWAQRNAEHIVPLYCFDPRHYVGTYNFNLPKTGPFRLRFLLESVLDLRNI
LLSKGSNLVVRKGKPEEVVADLIKQLGSVSSVAFHEEVASEELNVENKVKEVCAQLKVKVHTCWGSTLFHRED
LPFHHISRLPDVYTEFRKAVESQSRVRPVLLTPERLNPLPPGLEEGAIPTAEDLQQTEPVTDPRSAFPCTGGE

SSALARIKHYFWDTDAVATYKETRNGLIGVDYSTKFSPWLALGCISPRYIYHQIKKYERERTANQSTYWVIFE
LLWRDYFKFVGIKYGNKMFQVKGLQGKSIPWKMDTKLFDAWKEGRTGVPFVDANMRELALTGFMSNRGRQNVA
SFLTKDLGLDWRLGAEWFEYLLIDHDVCSNYGNWLYSAGIGNDPRENRKFNMIKQALDYDNNGEYVRRWVPEL
GSIRGADVHTPWNLSSAALSHSGVSLGETYPNAIITAPEWSRHFSKKPGGAGPSPRGRKGPSHTPKQHRDKGI
DFYFSRSKNL
>Gambusia_affi__XP_043966449.1
MAPNSIHWFRKGLRLHDNPALREAVRGAGTVRCVYFLDPWFAGSSNVGVNRWRFLLQCLEDLDASLRKLNSRL
FVIRGQPANVFPRLFKEWKISRLTFEYDSEPFGKERDAAIKKLAMEAGVEVIVKISHTLYDLDKIIELNGGQP
PLTYKRFQTLISRLDPPEMPVETLSDALMGGCVTPVSEDHGEKYGVPSLEELGFDTEGLPSAVWPGGETEALT
RIERHLERKAWVANFERPRMNANSLLASPTGLSPYLRFGCLSCRLFYFKLTDLYRKVKKNSSPPLSLYGQLLW
REFFYTAATNNPRFDKMEGNPICVRIPWDKNPEALAKWAEAKTGFPWIDAIMTQLRQEGWIHHLARHAVACFL
TRGDLWISWEEGMKVFEELLLDADWSVNAGSWMWLSCSSFFQQFFHCYCPVGFGRRTDPNGDFIRRYLPVLRG
FPAKYIYDPWNAPESVQAAAKCIIGVHYPKPMVHHAEASRLNIERMKQIYQQLSRYRGLGLLASVPSTNGNGN
GGMMAYSPGEQQSGTNSNNSHLPGVSGSSVATGNGSGSILFNFDNEEQTGPRSVGQQQQQRLHTLPQSQHQQQ
HAGYHSVPDASQNITSSRLYHEFAVPQHPGLFLHTRGSGITGKRERESEREALGEEEPASCSVNKMQRQSAET
S
>Gambusia_affi__XP_043980279.1
MPHSSAGDQAAVRQMLREVLVGREDPEGFFAMCVSILGHQETRSQFLSIIKPLSTANIRLHSVLSSIYQEYFS
KTEDDELELALALSLLEMKDQQISTPNQLPPHPGVGGNASSVLRNSNAKSQENGCTQQEDVPGREKSAFVAQS
MSGSKENFQENQTYKSTYAEVVDNKSHGTSQPLYVSDSSTSFSMPNTASVSGQVMKEGVLNLSKKPRCSKNRR
QRRKAAAQQIVGLTRSPSAPPPVLLWFRRDLRLCDNPALIRCLELGAPAIPVFIWSPEEEEGSGVTLAMGGAC
KYWLHQALSCFCASLERIGSHLIFLKASGEGNEDGSSLHTLKTLIKETGAQTVMANALYEPWLKERDDVVFSA
LQKEGVECKMFHSYCLRDPFSVSTEGVGLRGIGSVSHFMNCCRQNPGASIGVPLDPPVSLPSPTCWPQGVPLD
MLGLARMPRRKDGTIIDWAENIRKSWDFSEDGAHARLEAFLHDGVYRYEKESGRADAPNTSCLSPYLHFGQLS
PRWLLWDAKGARCRPPKFQRKLAWRDLAYWQLTLFPDLPWESLRPPYKALRWSTDCRHLKAWQKGKTGYPLVD
AAMRQLWLTGWMNNYMRHVVASFLIAYLHLPWQEGYRWFQDTLVDADVAIDAMMWQNGGMCGLDHWNFVMHPV
DAAMTCDPYGSYVRKWCPELADLPDELIHKPWKCPASMLRRAGVVFGQTYPERIVINLEEQRSKSLQDVALVR
KQFQQYVDKRSGCDLVPLPKRLVSEALGVSHWDGAVVTEGKQFLLPVITRMEFKHQLEDPDADAASNPYNAVL
KGYVSRKRDETIAFLNETDFTASVMYEGARRQERLERDYRRMEGLPPAPSNRRRARRTPTAKDKFSVVPGGAI
ASRN
>Gambusia_affi__XP_043981785.1
MVVNSVHWFRKGLRLHDNPALQEALSGADTVRCVYVLDPWFAGAANVGINRWRFLLESLEDLDNSLKKLNSRL
FVVRGQPTEVFPRLFKEWNVTRLTFEYDPEPYGKERDGAIIKMAQEFGVETVVRNSHTLYHLDRIIEMNNNNP
PLTFKRFQAIVSRLELPRRPLAPVSQQQMNRCLGKISENHDQLYSIPSLEELGFRTEGLPPPVWRGGESEALD
RLSRYLDKNVWVANTRVKTCSLYASPTGLSPYLRFGCLSCRLLYYNLREIYMKLCKRSSPPLSLYGQLLWREF
FYTAATNNPNFDRMEGNPICVQIPWDQNPEALAKWAEGRTGFPWIDAIMTQLRQEGWIHHRARHAVACFLTRG
DLWISWESGMKVFEELLLDADWSVNAGSWMWLSCSAFFQQFFHCYCPVGFGRRTDPSGDYIRRYVPILKDFPN
RYIYEPWNAPESLQKAANCVVGVDYPKPMINHAEGSRLNIERMKQVYQQLSHYRGLSLLASVPTIQEEAEPPM
TDESQTSSGPDSPPRVLADIEAAVCLMAPDSSTSAPVPDQDNASLHQLFQDHCTSSGWPSSPASSPPQSPAPR
SKPGSPSSSCPSPSTSPSLLPRRRKGVAHKGRRSQRQRERPSCPMAKEEDEEERMQEEADEERMEEETAAAAQ
Q
>Garra_andr__ADL62680.1
SSNVGINRWRFLLQCLEDLDASLRKLNSRLFVIRGQPTDVFPRLFKEWKITRLSYEYDSEPFGKDRDAAIKKL
ATEAGVEVFVRISHTLYDLDKIIELNGGQSPLTYKRFQTLISKMDPVEIPAETITAEVMGKCATPVSDDHDDK
FGVPSLEELGFETDGLSSAVWPGGETEALTRLERHLERKAWVANFERPRMNANSLLASPTGLSPYLRFGCLSC
RLFYFKLTDLYRKVKKNSLPPLSLYGQLLWREFFYTAATNNPHFDKMEFNPICVQIPWDRNPEALAKWAEGRT
GFPWIDAIMTQLRQEGWIHHLARHAVACFLTRGDLWISWEEGMKVFEELLLDADWSVNAGSWMWLSCSSFFQQ
FFHCYCPVGFGRRTDPNGDYIRRYLPILRGFPAKFIYDPWNAPESVQKVAKCIIGVHYPKPMVNHAEASRINI
ERMKQIYQQLSCYRGLGLLATVPPNTTNNGDGNRAGPSSGDNTQETNQITVHPSPANSHSEFSPFPVSCDQQS
INIVQPQPGHTGVGMVGQARKQSQRGRGASTLKPHNEHRASVVLTKIQRHDSN
>Garra_andr__ADL62683.1
EALNGADTVRCVYILDPWFAGSANVGVNRWRFLLDSLEDLDTSLRKLNSRLFVVRGQPTDVFPRLFKEWNVTR
LTFEYDSEPYGKERDAAIIKMAQEYGVQTVVRNTHTLYNPDRIIEMNNHSPPLTFKRFQAIVNRLELPRKPLP
TITQEQMARCRTQISDNHDEHYGVPSLEELGFKTQRDSSHVWKGGETEAMERLNKHLDRKGWVANFERPRIIT
AQSLFASPTGLSPYLRFGCLSCRVFYYNLRELFMKLRRRSSPPLSLFGQLLWREFFYTAGTNNPNFDRMEGNP
ICVQIPWDHNPEALAKWAEGRTGFPWIDAIMTQLRQEGWIHHLARHAVACFLTRGDLWISWESGMKVFEELLL
DADWSVNAGSWMWLSCSAFFQQFFHCYCPVGFGRRTDPSGDYIRRYIPKLKDYPNCYIYEPWNAPESVQKAAN
CIVGVDYPKPMINHAESSRLNIERMKQVYQQLSHYRG
>Garra_andr__ADL62684.1

LHVLTVLGEPEKPVRDVSAEDFLRCMTPAEADHEKTYAVPSLADLGLEVEFDVLWPGGETHALQRLDKHFQSQ
GWVTSFSKTRTVPNSLLPSSTGLSPYLSLGCLSVRTFYHQLSSVYAQSKNHSLPPLSLQGQVLWREFFYTVAS
STPNFTKMEGNPICLQIQWYHDPEALEKWRTAQTGFPWIDAIMTQLRQEGWIHHLARHAVACFLTRGDLWISW
EEGMKVFEELLLDADYSINAGNWMWLSASAFFHKYTRIFCPVRFGRRTDPQGQYL
>Garra_andr__AYN44210.1
MSHNSIHWFRKGLRLHDNPALLAALKDCRHIYPLFLLDPWYPNNTHIGINRWRFLIEALKDLDSSLKKLNSRL
FVVSGPPAEVLPKLFEKWKITRLTFEVDTEPYSQSRDKEVMKLAEENGVEVIPKISHTLYNIDRIIEENNGKP
PMTYVRFLSVVNAIGSPKKPVPAPTKEDMKGVSTPFFEDHEEEFRIPTLEDLGLDTSFLGPNLFPGGEQEALR
RLDEHMERTTWVCKFEKPQTSPNSIIPSTTVLSPYIRFGCLSARTFWWRLADVYRGKQHSEPPVSLHGQLLWR
EFFYTTAVGIPNFNRMEGNRHCVQVDWDNNPEHLAAWREARTGFPFIDAIMTQLCQEGWIHHLARHAVACFLT
RGDLWISWEEGQKVFEELLLDADWSLNAGNWQWLSASAFFHQFFRVYSPIAFRKKTDKYGDYIKKYLPVLKKF
PVEYIYEPWKAPRSIQERAGCVVGKDYPRPIVEHEVVHKKNIQRMKAAYAKRSPEGKDDKTTTKGIKRKAPSI
MDMFEKKAKR
>Garra_andr__AYN44213.1
MSASRTVTCLLRNDLRLHDDEVFHWAQRNADHIIPLYCFDPRHYQGTYHFNFPKTGPFRLRFLLDSVKDLRAT
LKKRGSTMLVRQGKLEEVVSDLIQQLGSVTAVAFHEEVASEEKSVEKNLKDICCQNKVKVQTFWGSTLYHRDD
LPFSHIRGLPDIYTQFRKVVEGQGRVRPVLSTLVQVKYLPLGLEEGNIPTFEALGQTEPVEDCRSAFPSRGGE
TEALARLKHYFWDTNAVATYKETRNGMIGVDYSTKFSPW
>Garra_andr__AYN44215.1
MSANKRNLKHQKESPDSAGGKQPKLTGGKGRESGWLLKEVTKLRKAAQGCEFNKKRLRYLSDTQKIKQGSDGV
LYWMARDQRVQDNWALIYAQQLALAEKLPLHICFCLVPRYLDATYRQYAFMLKGLREVAKECKSLDIQFHLLS
GEPGQNLPSFVKNWKFGAVVTDFNPLRIPLQWIETVKKHLPSDIPFIQVDAHNVVPCWEASEKLEYGARTIRG
KITKLLPEFLTEIPLVDTHPHAASRTAKPVDWEEVLSSLEVDRSVGEVDWAQPGSSGGMAMLESFIDQRLRLF
ASHRNNPNYDALSHLSPWIHAGQLSAQRVVKQVKREKNASESVLSFTEELVVRRELADNFCFYNHNYDSISGA
YEWAKKTLQDHAKDRRQYLYTKEQLENGKTHDQLWNAAQRQLVLEGKMHGFLRMYWAKKILEWTASPEEALSI
SLYLNDRLSLDGCDPNGYVGCMWSVCGIHDQGWAERPIFGKIRYMNYAGCKRKFDVAQFERKYAVIKETPQKD
TKKSI
>Gavialis_gangeticus_XP_019374878.1_PREDICTED:
MAASRSFPSSVPAPAGLCRAVHWFRRGLRLHDNPALQAALRDAASVRCIYILDPWFAASSAVGINRWRFLLQS
LEDLDNSLRKLNSRLFVVRGQPTDVFPRLFKEWGVTRLTFEYDSEPFGKERDAAIIKLAKEAGVEVVVENSHT
LYDLDRIIELNGNKPPLTYKRFQAIISRMELPKKPVSSITSQQMEKCKAEIQENHDDMYGVPSLEELGFPTDG
LAAAVWQGGEMEALARLDKHLERKAWVANYERPRMNANSLLASPTGLSPYLRFGCLSCRLFYYRLWELYKKVK
RNSTPPLSLYGQLLWREFFYTAATNNPKFDRMEGNPICIQIPWDRNPEALAKWAEGKTGFPWIDAIMTQLRQE
GWIHHLARHAVACFLTRGDLWISWECGVRVFDELLLDADFSVNAGSWMWLSCSAFFQQFFHCYCPVGFGRRTD
PSGDYVRRYLPKLKGFPSRYIYEPWNAPESVQKAAKCIIGVDYPKPMVNHAETSRLNIERMKQIYQQLSRYRG
LCLLASVPSCAEDLSGPVTDPASVQGCSTSTALKPSQSGQASPKRKHEGLEEMCTEDLYKRAKVTGLHGPEIP
SQSL
>Gavialis_ganget_XP_019358991.1
MFPKKQKQAAGADAGLSGSRKRGRQDAEAAPGAESLAEAVEQARRRAAPSVREFKFNKKRVRLLSQESHLQDG
ALGILYWMSRDQRVQDNWAFLYAQRLALKQQLPLHVCFCLVPKFLEATIRHFGFLLKGLREVAEECGELGISF
HLLQGYAKDVLPGFVTRHGIGGVVTDFSPLRVPLQWVEDVKERLPQDVPLVQVDAHNIVPCWVASNKQEYGAR
TIRPKIHAQLPEFLTEFPPIVQHPFPAAAPAQPIDWDACYASLEVDRTVSEVKWATPGTAAGLVVLQAFVARR
LPGYSEYRNNPNKPALSNLSPWFHFGQVSVQRAVLEVQKSRDRHRESVDSFVEEAVIRRELADNFCFYNKSYD
QLEGAHDWARDTLRLHARDKREYLYDLQQLEEGKTHDQLWNAAQIQMVREGKMHGFLRMYWAKKILEWTRSPE
DALRFAIYLNDRFELDGRDPNGYVGCMWSICGIHDQGWAERNVFGKIRYMNYAGCKRKFNVGQFEHKYRP
>Gavialis_gan_XP_019364513.1_cry-2-like
MKHSCIHWFRKGLRLHDNPALLAAMNDCSDLYPIFILDPWFPKNMQVSVNRWRFLIESLKDLDESLKKLNSRL
FVVRGHPAEVFPGLFKAWKVTRLTFEVDTEPYSKLRDAEVVRLAAAHGVQVIQKVSHTLYDTDRIIAENNGKV
PLTYRQLQAVLVGLGSPKQPVLAPTLETLKDCCTPGRDNHDPKYEIPTLEELGQDPKEAGPCLYPGGESEALS
RLDFHMKRMTWVCNFKKPDTEPNTLSPSTTVLSPYIKFGCLSVRTFWWKLAEIYRGKTHSSPPVSLHGQLLWR
EFFYTAGAGIPNFNRMEGNPICVQVDWDDNPEYVKAWKEGRTGYPFIDAIMTQLRTEGWIHHLARHAVACFLT
RGDLWVSWEEGQKVFEELLLDADWSLNAGNWQWLSASAFFHQFFRVYSPVAFGKKTDKSGAYIKKYLPILRKF
PAEYIYEPWKAPRSMQEQAGCIIGRDYPRPIVEHEAVSKRNIMRMKAAYAQRSHSKAAQVEKEGTKKGGKRKL
PAGPSVVELLTKKPKAKSS
>Gavialis_gan_XP_019370873.1_cry-1_X1
MGVNAVHWFRKGLRLHDNPALCECIQGADTVRCVYILDPWFAGSSNVGINRWRFLLQCLEDLDANLRKLNSRL
FVIRGQPADVFPRLFKEWNITKLSIEYDSEPFGKERDAAIKKLASEAGVEVIVRISHTLYDLDKIIELNGGQP
PLTYKRFQTLISRMEPLEMPVETITAEVMEKCTTPVSDDHDEKYGVPSLEELGFDTDGLPSAVWPGGETEALT
RLERHLERKAWVANFERPRMNANSLLASPTGLSPYLRFGCLSCRLFYFKLTDLYKKVKKNSSPPLSLYGQLLW
REFFYTAATSNPRFDKMEGNPICVQIPWDKNPEALAKWAEGRTGFPWIDAIMTQLRQEGWIHHLARHAVACFL

TRGDLWISWEEGMKVFEELLLDADWSVNAGSWMWLSCSSFFQQFFHCYCPVGFGRRTDPNGDYIRRYLPVLRG
FPAKYIYDPWNAPEGVQKAAKCMIGINYPKPMVNHAEASRLNIERMKQIYQQLSRYRGLGLLATVPSNPNGNG
NGGLMGYSPGENVSGCSSAGAQMGTSDGHTVSVQPCALGESHVGSNGIQQQGYFQASSILHFPHGDNQQSHLL
QQGRTSLSSGISAGKRPNPEEETQSIGPKVQRQSAN
>Gavia_stel__KFV47743.1
HLAYFVYRIIELNGHKPPLTYKRFQAIISRMELPKKPVSTVMSHQMEACKVDIQENHDDVYGVPSLEELGFPT
DGLAPAVWQGGETEALARLDKHLERKAWVANYERPRMNANSLLASPTGLSPYLRFGCLSCRLFYYRLWELYKK
VRKLLLPHLCLTVGLSLLLLQKRLLATNNPKFDRMEGNPICIQIPWDRNPEALAKWAEGKTGFPWIDAIMTQL
RQEGWIHHLARHAVACFLTRGDLWISWESGVRVFDELLLDADFSVNAGSWMWLSCSAFFQQFFHCYCPVGFGR
RTDPSGDYVKRYLPKLKGFPSRYIYEPWNAPESVQKAAKCIIGVDYPKPMVNHAETSRLNIERMKQIYQQLSR
YRGLCLLASVPSCVEDLSGPVTDSASGQGCSTSTTMRLSQADQASPKRKHEGAEELCNEELYKRAKVTCLPTP
EIPGKS
>Gavia_stel__XP_009805516.1
GFMLRGLQEVAKECAELNIPFHLLLGYTKDVLPGCVVEHXGGLVTDFCPLRLPRQWVEDVRERLPEDVPFAQV
DAHNIVPCWVASPKQEYSARTIRGKIHAQLPEFLTEFPPVIRHPYPPSCPAEPIAWEACYSSLQVDRTVKEVE
WATPGTAAGLAVLQSFISERLKSFSSHRNDPNKAALSNLSPWFHFGQVSTQRAILEVQKHRGKYKESVDAFVE
EAVVRRELAENFCYYNENYDSVQGAYDWAQTTLKLHAKDKRPFLYELQELEQGTTHDPLWNAAQLQMVREGKM
HGFLRMYWAKKILEWTRSPEEALRFAIYGWAERAVFGKIRYMNYAGCKRKFDVGQFERRYAPRTLSQ
>Gavia_stel__XP_009810059.1
FLLQCLEDLDANLRKLNSRLFVIRGQPADVFPRLFKEWNIAKLSIEYDSEPFGKERDAAIKKLASEAGVEVIV
RISHTLYDLDKIIELNGGQPPLTYKRFQTLISRMEPLEMPVETITPEVMEKCTTPVSDDHDEKYGVPSLEELG
FDTDGLPSAVWPGGETEALTRLERHLERKAWVANFERPRMNANSLLASPTGLSPYLRFGCLSCRLFYFKLTDL
YKKVKKNSSPPLSLYGQLLWREFFYTAATNNPRFDKMEGNPICVQIPWDKNPEALAKWAEGRTGFPWIDAIMT
QLRQEGWIHHLARHAVACFLTRGDLWISWEEGMKVFEELLLDADWSVNAGSWMWLSCSSFFQQFFHCYCPVGF
GRRTDPNGDYIRRYLPVLRGFPAKYIYDPWNAPESVQKAAKCIIGVNYPKPMVNHAEASRLNIERMKQIYQQL
SRYRGLGLLATVPSNPNGNGNGGLMGYSPGESISGCGSTGGAQLGTGDGHTVVQPCALGDSHTGASGIQQQGY
CQASSILHYAHGDNQQSHLLQAGRTALGTGISAGKRPNPEEETQSVGPKVQRQSTN
>Gavia_stel__XP_009813817.1
MPHHTIHLFRKGLRLHDNPALLAALESSQVVYPVYILDRKFMTSVMHIGALRWHFLLQSLEDLQKNLCQLGSC
LLVIQGEYESVLRDHIQKWHITQVTLDAEMEPFYKEMEANIRRLGEELGFEVLSLVGHSLYDTKRILDLNGGT
PPLTYKRFLHILSLVGDPEVPVRNLTAEDFQRCRPSDPGLAECYRVPLPVDLKIPPESLSPWRGGETEGLQRL
EQHLTDQGWVASFAKPRTIPNSLLPSTTGLSPYFSLGCLSVRTFFYRLSNIYAQAKHHSLPPVSLQGQLLWRE
FFYTVASATPNFTKMAGNPICLQISWYEDAERLHKWKTAQTGFPWIDAIMTQLHQEGWIHHLARHAVACFLTR
GDLWISWEEGMKVFEELLLDADYSINAGNWMWLSASAFFHQYTRIFCPVRFGKRTDPEGQYIRKYLPVLKNFP
SKYIYEPWTASEEEQKQAGCIIGQDYPFPMVNHKEASDHNLQLMKQVREEQYRTAQLTRDDTDDPMEMKIKRD
HSEENVSKGKVARMTEQTETPSGITCWEPKNGKRGEPEAS
>Gavia_stel__XP_009817663.1
REHSQPPVSLHGQLLWREFFYTAGASIPNFDQMVGNPICLQVDWDDNPQHLHAWREGRTGYPFIDAIMTQLRT
EGWIHHLARHAVACFLTRGDLWVSWEEGLKVFEELLLDADWSLNGXWLWLSGSAFFHRYFHVYSPITFGKKTD
RDGAYIRKYLPVLKDFPTEYIYEPWKASRAVQERAGCLVGTHYPQPIVEHGAASKRNLGRMKAARARKALPPP
RLPSSPPGTEQEPPSVPSVAKSQRKKPKIEQH
>Gavia_stel__XP_009820255.1
VLHWAQSNADFIIPLYCFDPRHYLGTHCYGFPETGPPFLLESLKDLRETLKKKGSTLVVRKGKPEDVVHDLIT
VLGSVSAVAFHEEATQEELDVEEGLCQVCSQHGVKIQTFWASTLYHRDDLPFRPIARLPDVYTHFRKAVESEA
TVRPTLCMADQLKPLAPGVEEGCIPTMEDLGQKDPVMDPRTAFPCSGGETQALMRLQYYFWDTNLVASYKETR
NGLVGMDYSTKFAPWLALGCISPRYICEQIQKYEKERTANQSTYWVLFELLWRDYFRFVALKYGKRIFSLRGL
QSKEVPWKKDLQLFDCWKEGKTGVPFVDANMRELAATGFMSNRGRQNVASFLTKDLGLDWRMGAEWFEYLLVD
YDVCSNYGNWLYSAGIGNDPRDNRKFNMIKQGLDYDGNGDYVRLWVPELQGIKGADIHTPWALNRAALSQVGV
TLGKTYPQPVVTAPEWGRHINQRLQGRSPHPRGRRGPAHMPTQHKDRGIDFYFSRKKDV
>Geobacter_sul_WP_010943456.1_photolyase
MNCGRIRSLLQGGEATAGPVIYWMSRDQRVADNWALIHAQKLALARSAPLGVLFCLAPRFLGATARQYRFMLK
GLEQVRAALNRLDIPFFLVTGDPKGAVAAFTRRHRVSYLVTDFDPLRVKREWKRQVAGEISIPFDEVDAHNIV
PCWITSQRQEWGAYTIRPKIHRLLPDFMEPFPPLQRHPFPWQGALPSDAEWRETFTGMTLDESVPEVSWLASG
EEAAQAALARFLEDGLAGYATRRNNPAVMGQSGLSPWLHFGQLSAQRVAQAAFAAAAPIESRDAFLEELIVRR
ELADNFCYYNDAYDRFDGFPEWAQRTLNRHRHDPRPQCYEHDVLEQGQTHDSLWNAAQLEMVRWGRMHGYLRM
YWAKKLLEWTSSPEDALMIAIQLNDRYQLDGRDPNGYAGIAWSIGGVHDRPWAERPVFGTIRFMSRDGCRRKF
DTDAYERRVIISPATCAGIALCK
>Geococcyx_cali__NWH59420.1
KVDYDVCSNYGNWLYSAGIGNDPRENRKFNMIKQGLDYDGNGDYVRLWVPELQGIKGADIHTPWALNSTALSQ
AGVTLGETYPQPIVTAPEWSRHINQSLQGRSPHPRGRKAPAHTPVPHRNRGIDFYFSRKKDV

>Geococcyx_cali__NWH60086.1
RFLLQCLEDLDANLRKLNSRLFVIRGQPADVFPRLFKEWNIAKLSIEYDSEPFGKERDAAIKKLASEAGVEVI
VRISHTLYDLDKIIELNGGQPPLTYKRFQTLISRMEPLEMPVETITPEVMEKCSTPVSDDHDEKYGVPSLEEL
GFDTDGLPSAVWPGGETEALTRLERHLERKAWVANFERPRMNANSLLASPTGLSPYLRFGCLSCRLFYFKLTD
LYKKVKKNSSPPLSLYGQLLWREFFYTAATNNPRFDKMEGNPICVQIPWDKNPEALAKWAEGRTGFPWIDAIM
TQLRQEGWIHHLARHAVACFLTRGDLWISWEEGMKVFEELLLDADWSVNAGSWMWLSCSSFFQQFFHCYCPVG
FGRRTDPNGDYIRRYLPVLRGFPAKYIYDPWNAPESVQKAAKCIIGVNYPKPMVNHAEASRLNIERMKQIYQQ
LSRYRGLGLLATVPSNPSGNGNGGLMGYSPGESISGCGSTAGQS
>Geococcyx_cali__NWH62918.1
QAKHHSLPPVSLQGQLLWREFFYTVASATPNFTRMVGNPICLQISWYEDAEKLHKWKTAQTGFPWIDAIMTQL
RQEGWIHHLARHAVACFLTRGDLWISWEEGMKVPFWF
>Geococcyx_cali__NWH65292.1
DNWAFLYAQRLALKQELPLRVCFCLVPKFLDATIRHYGFMLKGLQEVAKECAELNIAFHLLLGYAKDVLPAFV
VEHGVGGLVTDFCPLRLPRQWVEDVKERLPEDVPFAQVDAHNIVPCWIASPKQEYSARTIRGKIHAQLPEFLT
EFPPVIRHPHSPSCPTEPIAWEACYSSLQVDSTVKEVEWATPGTAAGLAVLQSFITERLKFFGSHRNDPNKAA
LSNLSPWFHFGQVSTQRAILEVQKHRSKYKESVEAFVEEAVVRRELAENFCYYNENYDNVQGAYDWAQTTLKL
HAKDKRPFLYKLQELEQGTTHDPLWNAAQLQMVREGKMHGFLRMYWAKKILEWTRSPEEALQFAIYLNDRYEL
DGRDPNGYLMLCLCLAGCLWSICGIHDQGWAERAIFGKIRYMNYAGCKRKFDVGQFERRYMPH
>Geococcyx_cali__NWH67064.1
RFLLQSLEDLDNSLRKLNSRLFVVRGQPTDVFPRLFKEWGVTRLTFEYDSEPFGKERDAAIIKLAKEAGVEVV
IENSHTLYDLDRIIELNGHKPPLTYKRFQAIISRMELPKKPVCTVMSQQMETCKVDIQENHDDVYGVPSLEEL
GFPTDGLAPAVWQGGETEALARLDKHLERKAWVASYERPRMNANSLLASPTGLSPYLRFGCLSCRLFYYRLWE
LYKKVKRNSTPPLSLYGQLLWREFFYTAATNNPKFDRMEGNPICIQIPWDRNPEALAKWAEGKTGFPWIDAIM
TQLRQEGWIHHLARHAVACFLTRGDLWISWESGVRVFDELLLDADFSVNAGSWMWLSCSAFFQQFFHCYCPVG
FGRRTDPSGDYVKRYLPKLKGFPSRYIYEPWNAPESVQKAAKCIIGVDYPKPMVNHAETSRLNIERMKQIYQQ
LSRYRGLCLLASVPSCVEDLSGPVTDSASGQGCSTSTAMRLSQTDQASPKRKHEGAEELCTEELYKRAKVTGL
PTPEIPGKSL
>Geotrypetes_seraphini_XP_033784381.1_cryptochro
MAANSVHWFRKGLRLHDNPALQAALRGAQEARCIYILDPWFAASSSVGIIRWRFLLQSLEDLDNSLRKLNSRL
FVVRGQPADVFPRLFKEWGVTRLTFEYDSEPFGKERDAAIMKMAKESGVEVIVENSHTLYDLDRIIELNGQKP
PLTYKRFQAIISRMELPRKPVCSITQQQMETCRADIHENHDDTYGVPSLEELGFSTEELGPAVWHGGETEALA
RLDKHLERKAWVANYERPRMNANSLLASPTGLSPYLRFGCLSCRLFYYRLRDLYKKVKRNSLPPLSLYGQLLW
REFFYTAATNNPKFDRMEGNPICVQIPWDKNPEALAKWAEGKTGFPWIDAIMTQLRQEGWIHHLARHAVACFL
TRGDLWNSWEFGVKVFDELLLDADFSVNAGSWMWLSCSAFFQQFFHCYCPVGFGRRTDPSGDYIRRYLPKLKA
YPSRYIYEPWNAPEAVQKTAKCIVGVDYPKPIVNHAEASRLNIERMKQVYQQLSRYRGLCMLASVPSCAEDLS
NPLVDPASGQGCSTGTVSNVLHCGQTSPKRKHEGAEDLQSEDKFKRTKVTNLPVPEIFTKDF
>Geotrypetes_ser_XP_033808009.1_cry-1_X1
MGVNAVHWFRKGLRLHDNPALRECIQGADTVRCVYILDPWFAGSSNVGINRWRFLLQCLEDLDANLRKLNSRL
FVIRGQPADVFPRLFKEWKIAKLSIEYDSEPFGKERDAAIKKLASEAGVDVIVRISHTLYELDKIIELNGGQP
PLTYKRFQTLISRMEPLEMPVETITSEVMETCTTPVSDDHDEKYGVPSLEELGFDTEGLPSAVWPGGETEALT
RLERHLERKAWVANFERPRMNANSLLASPTGLSPYLRFGCLSCRLFYFKLTDLYKKVKKNSSPPLSLYGQLLW
REFFYTAATNNPRFDKMEGNPICVQIPWDKNPEALAKWAEGRTGFPWIDAIMTQLRQEGWIHHLARHAVACFL
TRGDLWISWEEGMKVFEELLLDADWSVNAGSWMWLSCSSFFQQFFHCYCPVGFGRRTDPNGDYIRRYLPVLRG
FPAKYIYDPWNAPDSVQKGAKCIIGINYPKPMVNHAEASRLNIERMKQIYQQLSRYRGLGLLASVPSNPNGNG
NGGLMSYSPGESTTTGYCQGSSILQYAHGDSQQNHLLQGRHILSTGVISGKRPNPEEETQSIGPKVQRQSIG
>Geotrypetes_ser_XP_033814121.1_photolyase
MPRKAKRARSPEEAASSDGGGETLLEGVARSRLRAGSSVLAFAFNKKRVRLVSSSSELKEASGGISYWMSRDQ
RVQDNWAFLYAQRLALQQKLPLHVCFCLVPKFLDATIRHYGFMLKGLQEVAEECKELHIPFHLLIGLAKDVLP
QFINEHDIGGLVTDFSPLRVPLQWVDDVRKNLPDDVPFVQVDAHNIVPCWFASSKQEYGARTIRKKIHDQLPQ
FLTEFPAVSRHPHNSKFTAEQSVDWDACYASLQVDRTVGEVDWATPGGTAGLRVLQSFIGERLKFFNTDRNNP
NKAALSNLSPWFHFGQVSVQRAVLEVQKYGGKFRESVDAFIEEAVVRRELADNFCFYNETYDKLEGAYAWARN
TLKDHTKDRRTHIYTLEQLEAGKTHDPLWNAAQLQMVHEGKMHGFLRMYWAKKILEWTSTPETALKYAIYLND
RFELDGRDPNGYVGCMWSICGIHDQGWAERAVFGKIRYMNYAGCKRKFDVEQFERRYHPKKFSH
>Gigantopelta_aegi__XP_041350836.1
MYKLRKQLLEWRITTLTFEEDPELYGKERDVAITSLTKEHDVETITRISHTLYDLQKILENNSGKHPLTYKRF
QSIISGMDQPPHAEETIDADLIGSSVTPISENHNKKYGIPTLKELGFDVELVEPSSFRGGETEALIRLFRHLE
KKGKTTILAWVANFEKPKLSPLSPFPSQTGLSPYLRFGCLSARHFYWNLRDLYKNIKKDNEPPMALHGQLLWR
EFFYMAATNNPKFDQMVGNPICVQIPWDKNSEALAKWAEGKTGFPWIDAIMNQLKQEGWIHHLCRHAVAYFLT
RGDLWLSWEEGMKVFDELLLDAGWSVNAGMCMWLSGSSFFHQFFHCYCPVGFGKQADPSGDYVRHYLPVLKSF

PTKYIYEPWTAPESVQKAANCIIGKDYPCPMVNHAEVSQVNMQRMKQVYQYLTIKAKSKFQGVSFFFLFFSFF
FN
>Ginkgo_bil_AXV45366.1_cry-1
MSTNCSIVWFRRDLRVEDNPALAAGVRAGPVIPLFIWSPEEEGHYYPGRVSKWWIKQSLVHLDSSLRSLGAPL
ITKRSTDSISALLQVIESTGATQLFYNHLYDPLSLVRDHKLKQDLSTRGILVRSFNADLLYEPWEVNDEEGRA
FSTFEAYWNKCLNMPYDPSPPLLPPKRIIQGDVSSCPSENLGLEDESEKGSNALLARAWSPGWSSADKALTAF
INGPLIEYAKNHRKADSATTSLLSPHLHFGELSVRKAFHLVRIKQVLWANEGNTSGEQSVNLFLKSIGLREYS
RYLSFNFPCSHERPLLAHLNFFPWRVDKGYFKSWRQGRTGYPLVDAGMRELWATGWLHDRIRVVVSSFFVKVL
QLPWRWGMKYFWDTLLDADLECDALGWQYISGSLPDGREIDRIDNPQIEGYKFDPAGEYVRRWLPELARLPTE
WIHHPWDAPEFVLHAAGIELGSNYPRPIVEISAAKDRLQEALTEMWQCEAASRAALENGIEEGLGESLENNND
LLPEHMDLDREPVRNNTEIAARSQEDQMVPSMTDDLRRAEVPYLLNQDVSSVAPPERPEVPSNMNPGIASTAP
SEQYLGRFTRPMTRISNEGLPYVSDNDRLIQVLDLQNESQSTAESSSTLREHDEVSGGGIVPVWSQSMSGHSQ
QFPEEGTVPGSNDSDNQRHLQSHPMVTWRPWSQSRQAHIHGRPPSWELENAVQSNAMV
>Glaucidium_bras__NXL29402.1
RFLLQSLEDLDNSLRKLNSRLFVVRGQPTDVFPRLFKEWGVTRLTFEYDSEPFGKERDAAIVKLAKEAGVEVV
IENSHTLYDLDRIIELNGHKPPLTYKRFQAIISRMELPKKPVSTVISQQMETCKVDIQENHDDVYGVPSLEEL
GFPTDGLAPAVWQGGETEALARLDKHLERKAWVANYERPRMNANSLLASPTGLSPYLRFGCLSCRLFYYRLWE
LYKKVKRNSTPPLSLYGQLLWREFFYTAATNNPKFDRMEGNPICIQIPWDRNPEALAKWAEGKTGFPWIDAIM
TQLRQEGWIHHLARHAVACFLTRGDLWISWESGVRVFDELLLDADFSVNAGSWMWLSCSAFFQQFFHCYCPVG
FGRRTDPSGDYVKRYLPKLKGFPSRYIYEPWNAPESVQKAAKCIIGVDYPKPMVNHAETSRLNIERMKQIYQQ
LSRYRGLCLLASVPSCVEDLSGPVTDSASGQGSSTSTAMRVSQADQASPKRKHEGAEELCTEELYKRAKVTGL
PASEIPGKSL
>Glaucidium_bras__NXL31451.1
DNWAFLYAQRLALKQELPLHICFCLVPKFLDATIRHYDFMLRGLQEVAEECAELNIPFHLLLGYAKDVLPAFV
VEHGVGGLVTDFCPLRLPRQWVEDVRERLPDDVPFAQVDAHNIVPCWVASPKQEYSARTIRGKIHAQLPEFLT
EFPPVIRHPFPPSCPAEPIAWEACYSSLQVDRTVKEVEWATPGTATGLSVLQSFIAERLKSFGSHRNDPNKAA
LSNLSPWFHFGQVSTQRAILEVQKHRGKYKESVDAFIEEAVVRRELAENFCYYNENYDSVQGAYDWAQTTLKL
HAKDKRPFLYKLQELEQGATHDPLWNAAQLQMVQEGKMHGFLRMYWAKKILEWTRSPEEALQFAIYLNDRYEL
DGRDPNGYVGKADAPSPHPAGCLWSICGIHDQGWAERAIFGKIRYMNYAGCKRKFDVGQFERRYAPRT
>Glaucidium_bras__NXL38379.1
MGVNAVHWFRKGLRLHDNPALRECIQGADTVRCVYILDPWFAGSSNVGINRWRFLLQCLEDLDANLRKLNSRL
FVIRGQPADVFPRVFKEWNIAKLSIEYDSEPFGKERDAAIKKLASEAGVEVIVRISHTLYDLDKIIELNGGQP
PLTYKRFQTLISRMEPLEMPVETITPEVMEKCTTPVSDDHDEKYGVPSLEELGFDTDGLPSAVWPGGETEALT
RLERHLERKAWVANFERPRMNANSLLASPTGLSPYLRFGCLSCRLFYFKLTDLYKKVKKNSSPPLSLYGQLLW
REFFYTAATNNPRFDKMEGNPICVQIPWDKNPEALAKWAEGRTGFPWIDAIMTQLRQEGWIHHLARHAVACFL
TRGDLWISWEEGMKVFEELLLDADWSVNAGSWMWLSCSSFFQQFFHCYCPVGFGRRTDPNGDYIRRYLPVLRG
FPAKYIYDPWNAPESIQKAAKCIIGVNYPKPMVNHAEASRLNIERMKQIYQQLSRYRGLGLLATVPSNPNGNG
NGGLMGYSPGESISGCGSTGGAQPGAGDGHTVVQPCALGETHTGASGIQQQGYCQASSILHYAHGDNQQSHLL
QAGRTALGTGINAGKRPNPEEETQSVSGPKVQRQSTN
>Gloeobacter_kil_WP_023172026.1
MPSLAIVWHRRDLRLQDNPALARAAEQFQQVLAVFVIDPAIIGRDDTAPARVHFLRESLAELQRAYANLGGRL
VVCRGEPAEQLVVLARTLGAEAVFFNDDIEPYARTRDQKVIEALAGAGVAAHPCAEILLHPAAEVLTGQGKPY
TVFTPFWRNWSAKPKPRPFAAPERLSAPSVETGTFISLDELGKPFSGKLLVTPGEQAGIKLLDHFCEAALYRY
DERRDHPAEPGTSLLSAHLKFGTIGIRAVWQRTIESWRKAPADRERANLAVWQQELGWREFYKYELAHFPELA
ERPFRRAFEQFPYDHDRERFERWCRGETGFPFVDAAMRQLNTVSWMHNRLRMVVASFLTKDLLLPYQWGERYF
MQKLVDGDLSANNGGWQWAASVGTDPKPLRIFNPSTQARRYDSEATYIRRWLPELAPVDTAMLVESERFSPLF
RSRYGYPQAIVEHKSQQQIFKERYRQIHSTEKQADTSTSD
>Gloeobacter_kil_WP_023172734.1_DNA_photol
MTHTLLWFRKGLRLHDNPALQMAIQKATRLMPVFVLDPHFIDPDRVGINRMAFLLESLVDLDRRLRTLGSRLL
VLRGQPEEVLAHAFSTWQVGRLCFERDTEPYARNRDERLRSLAQKCGIEVVSPTGHTLFDPDAVLKAGGGRAP
LTYSAFLRVISRLGTPAQPQPTPAHLPPPIAEICESPDYAIPTLTELGYTDLEAVNHRYPGGETAGLERLGDY
LADRARVAHFAKPDTDPTAFDPPATTVLGAHLKFGCLSARTFYYEVQAIYRQARSHTEPPVSLLAQILWREFF
YVLGYATPNYDRMAGNPICRQIAWDDNPAYLAAWSEGRTGYPWIDAAMHQLEREGWLHHLSRHAVACFLTRGD
LWLSWEAGQAVFERLLVDQDWSLNASNWMWLSASAFFHAYHRVYSPISFAKKYDPAGRFVRHYLPALERYPDA
FIYEPWKAPLSVQREAGCIVGRDYPEPIVDHTEARTRNIERMRMAFTADPSTLPT
>Gloeobacter_kil_WP_023173156.1_DASH_famil
MSETILIWYRNDLRLHDHEPIYRALQQRAQVVPLYCFDPRAFGRTPHGFAKTGAHRARFLLQSVAALKDALEQ
LGSGLVVRLGEPEAVIPALVKELNISAVYYHQEVTSEERAVESALEIALKPLNVALRAFWGTTLFHLEDLPFD
VAQLPELFTDFRKRVEESPTVREALPAPGQLSKLPEVDPGALPTLATWGLEEPATDPRQVIEFTGGEPAGLAR
LDHYFWQANLLKNYKQTRNGMLGGDYSSKFSPWLAHGCLSPRRIYEQVRLYESQRLKNDSTYWLIFELLWRDF

FRLVALKHGNRLFRASGLRGLVIDWQDDPERFERWRTGQTGFPLVDANLRELAATGYMSNRGRQNVASFLTKN
LGIDWRQGAEWFESQLIDYDVTSNWGNWNYTAGVGNDARGFRFFNILKQARDYDPEGDYVKHWLPELAHVPAP
KIHEPWKLLPVEQQRFGVRLGVDYPLPMVDLFQSAAANEALYNAAGERRAGRRRRAGR
>Gonapodya_pro_KXS11844.1_M427DRAFT_
MPPTSTALSALAAKTTSASSTVLHWFRTDLRIHDNTALLLASKAAQSLTRGESRTNLACLFVVSPVEWRGHGC
APVKVDFWVRNVEALRDTLAERGIPLVVKMNDDKKGWKAVSEVVKEVCKELSVEELFYNVEYEVNEQRRDKMV
TEAVAKLGVKVTPCHDQCVVEPGKVVTKEGKVYTVYTPFKNSWHRYVSENKAVLAVRDPPSSNNHKFSSTASS
YIDSHSTIPSAPSLPAYSATPLFSQPSLTHLTFLRTLYPAGETAAQTRLSAFTASRIDAYHESRDLPTAPGTS
SLSPYLALGVLSARQCVSAASAANGGRRDTGKQGAVVWIQELIWREFYRHVLAGFPHVCKNRAFKPQYEGVQW
TESWSRDVVLPAFPVPSTTSTPPPPLPPAPPGTPNPLFTAWTQGRTGYPLVDAAMRYLLHTGWMHNRLRMIVS
SFLVKDLLVDWRLGEAWFCANLIDHDLASNNGGWQWAAGTGTDAQPYFRIFNPSNQAEKFDKDGKFVRMWVKE
LEWVKDGVLLRGPWEAAAKGSVVVTKKKMEECGYPKPLVDHAQARLAAIAAFKLAVQGADGGGGDSDSGEEVV
PKSKKRAAGASAGITGFLITKPKKQKK
>Gonapodya_pro_KXS17061.1_cry/photol
MTSTRPHDFYAIAKLPARRPPHPPPTSLPPIATLPTLPSTDPSTLTGPDGSFSVSLLAELGHDEPPVDERSPH
MGGETEALRRFEAWMKREKEVAKVKLPGWGVDQLHSSARLPSRLRAITLNHISPIPRRTALPFLPHLAILDTS
CNFRTTKMGRTGSVAWVGFYLDIGRRQLKQEGWNHHLVRHAVAFFLIRGDLYISWERGAEVEELLLDAYHALN
LLRLLHDLLPCLPPVSFAKKYDKKGIFAKRYVKEVAGLPEKYVWEPWTAPASVQALAKCKVGTDYPASIVPHD
FERKTNMDRMAAAYKTGKRPVPESVGSVAAQGSSSAAGDVQDTLAGFTQGFGESDDEAAQDVARPAKRRAGTE
DEEEEARENERPKEKKKKAKS
>Gonapodya_proli_KXS15620.1
MSTASSTASRPKIAIILLNGAPRLHDNPLWLLRSPRRLGIPTATHALPLFCLDPRRYDLEMMYTGKEGADGPK
TRWFGFPKCGKWRSKFIIESVQDIRTSLKSLGSDLCLVPGRIETVVPQLVESCRSNHPTGADVVGVWISKEIS
SEELVVERKLAQNLSALFPSVPLFAEWTSTVFPSEFTFSHSNAWPASSAVNPVSNLFGGSTFLQLPDVFNQFR
KLVESRAGEPPEPYQRPASVPPLPDWLTLEGIGPENKGIGKPGHDDVSKFGHIPSSLLEVPTHESSAFPFRGG
ESSALGRLNHYISSGLITTYKQTRNGLLGEAFSTKFSPWLADGSLSARKIWHQVRQHENTHLESEGSAWILFE
LLWREFFKFHAVKYGDGIFRIGGVHAGTYKETFAKAWKRSVKSGWDMFSVVNADSRWALPNNEAFKSWADGRT
GIPWVDAGMRELKETGFLSNRSRQNVASFFLQSVPTISSSQSANGQVAPNWLPAAELFESLLIDHDPHSNYGN
WQYLAGVGTDPRGWRKFNMMKQAAEYDARAEWVSTWVTELAGIKKSLEERGGTTEQFHKPWTVNGGRAVDEVG
YPRPIAVEREWSHVRFPEVKLGERRSALVANSPSLRQDESSLEERPRDGFGRARSARSNQKRRVDAPWNGGR
>Gouania_will__XP_028290364.1
MSASRTIICLLRNDLRLHDNELLHWAQRNAEYIVPLYCFDPRHYVGTYHFNLPKTGPFRLRFLLESVKDLRNT
LVNKGSNLVVRRGKPETVIAELIKQLGSVSGVAFQEEVTAEELNVEKKVREVCAKLNVKVHTCWGSTLFHRDD
LPFHHSSSLPDVYTQFRKAVETQSRVRPLLQTPEKLNPLPVGLEEGSVPTAEDLEQTEAVTDPRSAFPCSGGE
THALARLKHYFWDTDLVATYKETRNGLIGVDYSTKFAPWLAMGCISPRYIYHQIKQYETERTANQSTYWVIFE
LLWRDYFRFVALKYGNRMFHVKGLQEKSVPWKRDMKLFDAWKEGRTGVPFVDANMRELAMTGFMSNRGRQNVA
SFLTKDLCLDWRMGAEWFEYLLIDYDVCSNYGNWLYSAGIGNDPRENRKFNMIKQGLDYDSNGDYVRQWLPEL
QTIRGADVHTPWILSSAALSHAQVSLGDTYPTPVIVAPEWSRHFNKKPGGGGPSQRGKKGPSHTPKQHRDRGI
DFYFSRSKNL
>Gouania_will__XP_028304438.1
MVVNSVHWFRKGLRLHDNPTLQEALNGADTVRCVYILDPWFAGAANVGINRWRFLLEALEDLDSSLKKLNSRL
FVIRGQPTDVFPRLFKEWRVTRLTFESDPEPYGKERDAAIIKMAQEFGVETVVRNSHTLYNLDRIIEMNNNSP
PLTFKRFQTIVSRLELPRRPMPPITQQMDKCHTKISDNHDQLYSIPSLEELGFRTVGLPPAVWRGGESEALD
RLNKHLDKKVWVANLEHPRSNTCSLYASPTGLSPYLRFGCLSCRILYYNLRELYMKLRKRSNPPLSLFGQLLW
REFFYTAATNNPNFDRMEGNPICVQIPWDQNPEALAKWAEGRTGFPWIDAIMTQLRQEGWIHHLARHAVACFL
TRGDLWISWESGMKVFEELLLDADWSVNAGSWMWLSCSAFFQQFFHCYCPVGFGRRTDPTGDYIRRYIPILKD
YPNRYIYEPWNAPEAIQKAANCVVGVHYPKPMINHAEGSRLNIERMKQVYQQLSHYRGLSLLASVPTIQEEAE
PPMTDESQTSSGAESPTRDSADCEVAGCSTAPDSSTASPAFTVALKQVQEDASTAASPPHNRPCTSAVPPHPI
PSMADSSPWSISKPSSPSSSCPPSFPSPSASCAAPTHTISFSASSCLGPWRKGLSRKARRGQRQRGGRPSCSP
AACEGEREEVGGEERMEEGVEQEDDRMELEEKTGEPQ
>Gouania_will__XP_028304738.1
MVVNTIHWFRKGLRLHDNPALRDSINGSDSLRCIYILDPWFAGSSNVGINRWRFLLQCLEDLDASLRKINSRL
FVIRGQPTDVFPRLFKEWEITRLSYEHDSEPFGKERDAAIQKLACEAGVEVMVRISHTLYNLDRIIEINGGQP
PLTYKRFQGLINRMDAVDSPAETITYDVIKNCTPPIRDDHDDKFGVPSLEELGFETEGLSMAVWPGGETEALM
RLERHLERKAWVANFERPRMNANSLLASPTGLSPYLRFGCLSCRLFYFKLTDLYKKVKKNGTPPLSLYGQLLW
REFFYTTATNNPCYDKMEGNPVCVQIPWDRNPEALAKWAEGRTGFPWIDAVMTQLRQEGWIHHLARHAVACFL
TRGDLWISWEEGMKVFEELLLDADWSVNAGSWMWLSCSSFFQQFFHCYCPVGFGRRTDPNGDYVRRYLPILRG
FPAKYIYDPWNAPEEVQKAAKCVIGVHYPRPMVNHAEASRINIERMKQIYQQLSCYRGLGLLATVPNNPSNSA
SGSNGEGVSTRTSQTPDDSYKHEASSTNKRGQSVLKRRREEIVSETSCKSWRPGK
>Gouania_will__XP_028306298.1

MSSSAADQTLLRQMLGQLLGGREDPEGFFAMCVSVLGHRETRSRFPSFIQPLCASHGSLHAQLTSIYLRYFSK
SEDDELGMALALSLVENTNPQSAQRSDEISAPPLGSESRSRGKEPENKAKDRAMMNVEESLKTQEKVSRSKKR
RQRRKGVVQQIVGLPGSPSAAAPVLLWIRRDLRLCDNPALIGCLELGAPVIPVFIWSPEEEEGPGPTVAVGGA
CKFWLHQALSCFCASLEYIGSHLIFLNATGKANELRASLHVLKDLIMETGARTVLANSLYEPWLKERDDEVLS
ALQTEGVECRMFPSYCLREPYSVSTRGVGLRGIGSVSHFMSCCRQNPGAAVGVPLDPPASLPTPSSWPQGVPL
DTLGLARMPRRKDGSVIDWAANIRTSWDFSEDGAHSRLEAFLHDGVYRYEKESGRADAPNTSCLSPYLHFGQL
SPRWLLWDAKGACCRPPKFQRKLAWRDLAYWQLTLFPDLPWESLRPPYKALRWSSDGGRLKAWQRGKTGFPLV
DAAMRQLWLTGWMNNYMRHVVASFLIAYLHLPWQEGYRWFQDTLLDADVAIDAMMWQNGGMCGLDHWNFVMHP
VDSAMTCDPCGTFVRKWCPELTDLPDELIHKPWTCPASMLRRAGVLIGQTYPERIVTDLEEQRSQSLRDVALV
RRQFPQFVDKRSGCDLVPLPPRLVSEALGGTREDVSGDFLLPVITRMEFKHQQDDPDADSASNPYNAVLKGYR
SRKRDETIAFLNERDFMTSVMFEGTQRKERLEKDQCRLEGLPPPPPSASRGRARRTPHAKDQFSIVPGGAVTS
LK
>Gouania_will__XP_028328731.1
MLHIIRSRSYFASVTLLKQTIKNPKSRLSFIVTSSMSGRKRKAPSAPAAKPASKQQKTEKKKKEEEEKSVDSA
PEISEKPPTEFNKRRIRLISDTEKIKQGSEAVLYWMLRDHRVQDNWALIHAQQLALEEELPLHVCFCLVVPKS
KTSTLRHYSFMLKGLQEVSKECKQLNIQFHLLHGAPDEVLPGFVSEQRFGAVVTDFSPLREPIRWLEMVNKAL
PQDIPLIQVDAHNVVPCWVASPKQEYAARTIRGKITKLLPEFLTGFPLVEKHSYTAARTAKPVDWEETLNSLN
VDRTVAESKWAEPGTKAGLLMLKSFIDVRLKTFGTLRNDPNSPSLSQLSPWIRFGHLSAQHVALQVQRNGKKF
GSSVAPFLEELVVRRELTDNFCFYNDKYDKVEGAYEWAQKTLREHAKDKRPYIYTLQQLENAETHDKLWNAAQ
YQMVSEGKMHGFLRMYWAKKILEWTSSPEEALSFALYLNDRYELDGQDPNGFVGCMWSICGIHDQGWAERPIF
GKIRYMNYKGCLRKFDVASFEKKYSPKKL
>Gouania_will__XP_028328938.1
MAHSCIHWFRKGLRLHDNPALMAALRDCKELYPLYILDPNYTYGGKKRIRFLMGALKDLDCSLQKLNSRLFVV
RGKPEEVFPELFSKWNITKLTYEYDTEPYGRSCVGKVTTLAQEHGVEVIYKISHTLYDIDRIIEENNGKPPLT
YNRLQSLVKTLGPPKKPIPAPTMEDIKDVKTPCFEQHNKKYKIPSLEDMGVDSTSMEEEQFPGGEQEALRRLD
EHMKMTAWVCSFEKPQTSPNSLRPSTTVLSPYTTFGCLSARTFWWRLSEVYRGKKHSDPPVSLHGQLLWREFF
YTASVGIPNFNKMEGNPVCTQVDWDTNSDYLSAWKEARTGFPFIDAIMTQLRMEGWIHHLARHAVACFLTRGD
LWISWEEGQKVFEEFLLDLDWALNAGNWQWLSASTFFHQYFRVYSPIAFGKKTDKNGDYIKKYLPILKKFPPN
YIYEPWKAPLSIQQAAGCIVGKDYPRPIVQHEVVSKKNIQRMKVAYAKRSPHITDSPEKKKGTKRKAPTVVEM
LMKKNRRQ
>Gracilariopsis_cho_PXF42490.1_photolyase
MSGNTSPAETTRVHKLNNNDVNKAGTFVLYWMSNSLRSHYNYALAHASYLCEKYEMPLRVAHVFDAVAQDGKP
LPERHAAFQLESLSDVQKDMHERNVPFVVINGSHKARDAVPALAEDAVAVVTDTSYLRKGRSDRHAVATALPI
PLYAVEADVVVPVQTVSSKSEYAARTIRPKITRALKDYLVPMKNVELTNQTNCDLKRWVSSANPDLSLLTLED
LDESLKGMEGLDRGAPRVRFFKGGQEEAQKRLTTFLDEKLREYGGGRNEPAKQLQSDLSPYLRSGNISPVDIA
LHTKAKATKKASWKESEESFLEELIVRRELAVNACWFNADAYDVYEQIVPNFARESLALHKSDKRPKIFTYEE
LEAAVTEDPYWNAAQLEMVVKGKMHGYMRMYWVKRIIGWVEDPADAMDYAIRLNNRWELDAVDPNSYAGVIWC
FGLHDRGWTERPIWGKVRYMNESGLKRKFNMPAYIAYVDKMVASEGLPRHIAELRKKHKMGGQQQTIDGSRKR
LRSRKATSSSSKSGLRGSESKSVKRIKM
>Gracilariopsis_cho_PXF43553.1_cry-1
MSAFHATVPSAKWFRKPSVVWYRGNDLRVEDHPALLAAAQRGGPVVPLFIWDSSDGFGAAMGATKQWWLRESL
THLQHDLRRLGVQLYTRKGRSTEELRAFLHETGADAVFWNRCYEPDLLKRDEELREELAQEGLTAVSFKAELL
VEPWELTNSKTSPRFETFHTYMCAWMTVAPPPQPFPCPSRLQPISKIVPSTGIDLLGFVIPDDVANKLGKVWT
PGSAHAKRQLDRFLKEIFPAFGDSRCRRHFEGTSRLSPHIRFGELSPRRMYHATRLRVLRDQQNLQKASIGQS
AEPNPYAVKHPALSDVKSLTGENNKFRKQDIHGLSAPCSSPVGGQNASLRDMSTIRRPQTRNNKADQASATPT
GRRRGVLPQISRSGRAFLKNLCLRDFSYHVLYHFPDLNSQPLIPEFAKFPWAEDNGLFARWRAGATGYPIVDA
AMRQLHQSGWVHNGMRFLLACFLTKYLLLPWSRGLEQFYDLLIDGDRSANALGWQWTTGSNTDAFPITALVNP
VKQAHKHDPNGDYIRRWLPELARLPVEYIHEPWKAPFEVLDKCGVQLGNTYPKRIVLISDARARATKAMTTMK
RLFAGNIVWRRYLKYDEEDLINEWPEQKPNAVRVDESNNPNGKGFLLPSLWALLQCEQPPSYISGSSTAIEPL
ISMDTTACLVDGALAIPLDDQPESIEQAFISTHEFVSTDPHLSSDGLLSPEVRDVDTPSVPHVPSDEQSHALF
HGENIKSEDVQQLQDPMKYTAVGSESYKDEARIQNQVEDEFGSSLANQGEDKKRTPDKGQPSAPAENIVCDTV
SAVPDQNRRITASPPASHGSQASQEQPARNQPVLGLEQYTTQASDPAALTPHSAAQRPFAISHSNALQSQSSI
HARIRPNNKEPHEQQIQFQQSQIHAPFHIGDLRGHQSFSQVLLSSQQYHGTDAHIAKFQPSQHGSQHPMVPVH
NVMDPTYAARFSTGRVHLNPMAGVPMPANPVMMGTVPTGMGSVPSGSEFGTSHLLNGGDTLNAQQAHAVRAHP
FFGYRQYVPMVNAHPSSTGERPELVGPSAAHSQKFSVPAIFPVSYDIYGNGAFDPSVFGGSLSQPQFPSHVYA
PQSQAVPIGVRHANGTSHPLLFPVGPRTELQQPQTSSVLSQEVTNAGAVAPNRHEPGAVGGSSSMLNGTTRKS
VNQNRLAEFEETCATPSRIYSARCRKGGQPSTSKGPGSANGGYVTPSTNVDNTSGLNISQQSRPNTKPIPKQR
QPPRSKRLRQSQGLSSRGKHDAKKGSKERSQRNGSESTHKAQAQSVGAATTPKSRQKILESVLTTEGHEYRGF
ARYLSQTYEFTGNTDRQTSRDYVRLCNLKDDYHKKCNSEKHKLKIYRIKHFFSKVLKLEVTGEWDRHNHGGVR
GPYVYGIKLKQQANSKSK

>Gracilariopsis_cho_PXF45941.1_cry_DASH
MRRGPRRVSNRGRDLHRGGSRRGRAKNSWFSHPRASVDPFSGPRVIEQAAPKSLPPESASILWLRNDLRVCDH
EALALANTSGTMVPVFIFDTSKFGIKNASPWGFQRNGPFRTVFMIESVKDMQQNLRLRGNDMLVRQGDPVTEI
LDIAKALAESGFDSVNVVAHKETTWEETKDEDALQAGLKRLAQETGKDMEAYWLWGSTMHHVDDVPFNPGGPG
LPETFTAYRKLVEGKKTPVREEVPTPERLNPFPMELRLRSDRFPSLRNDLQVEGLCDPLDHAFPHPLGAMDFV
GGESNGLDRMNEYIWDINGLECYKETRNDSGKRNFSSKFSPWLALGCISPRTLYWQVREYEQKKVANDSTYWM
LFELMTRDYFRWVSASVGTKLFAVNGYSGRSVGEPSLFDVNPAAVRPIHRTRLQKWIEGKTGAPYIDASMREL
ALTGFMSNRGRQNVASFLIHDLEFPDWRAGAEYFESVLIDHDVASNWGNWAYLAGVGSDPRGGRKFNVVKQSK
QYDPEGWFITRWCPELIDIPPPMIHEPHLLSSEALKEVGITEGETYPTPIVRLPTAPSSQKEQ
>Gracilariopsis_cho_PXF46329.1_(6-4)DNA_p
MPSPAVLHWFRRDLRLTDNRALNEAIRVAKKTKSPLIPLYILDPNLIPQNCSAVRMRFLRESVSQLRSSLLKH
GLNLRVVRGQATQVIPDLAGRWRVRDTFWESEYDPVSRERDAQVARDLKGSGVNVRTFPGFLLYDPDDVLRHC
NGLAPRTMTGMLKLVEILGDPRTPDSFANGELVEFEEDPFLYEDRAQGMCDIPSLADLGYQDPENRWDVFEGG
EKAGLKRLHRFVKQKNGRIVADFAKPKTNPAAFSHRETTVLSPYLALGCVSCRVFHERLREVEQRYALQEMPQ
TTLWGQLIWREHFWLLAYSVKNFHQMRGNVVCRQINWRADKQSEELLRTWAEARTGFPWIDALMTQLHKEGFI
HHLGRHSLACFLTRGDLWISWERGFKVFEKYLIDYDYALNSANWMWLSCSAFFVMYYRVYSPVTYGKKWDKDG
NFIRHYLPVLRTMPSKYIYEPWKAPKELQRRVGCIIGKDYPEPIVDHRTASQCNIARMKESFEKGEFGKLEDE
TESRVIASANNSIITSDDVREPYRKRRRR
>Gracilariopsis_cho_PXF48820.1
MPPPISCSPSTHSVFRVLGSPSCNADHLNHSLPDTAATVHSASIPSSPSPSPAAPIVVWFRGHDLRVHDHAAL
DAAIASGAPVVALYVLERGAETDGASSYVRRLSASRTQHHSHSEACSSITTSCHSATASHHPLYGTAYTATSP
STDEDHHHDHDHDEHCEPWGPLSPNGGFALGRVQRWYLHHSLRVLREQLQHLGVTLVLRRVDCAEETSRQVLS
VVRLLRAATVFWNKRYKPRAYPVDEAVKSALRKLNVASHDFDSETLIPPSTDYGRFNDFQGYTRFWINHMRLQ
PPPQPEPPLDPHAVVAYPRQLVFQLLSASSHYCSTPNRLPPFAAPVLPSVSDLELLDGLDVHGDHSPGDVSSI
GCVAAARALHRFLNGDRFARFASDVARRDGMVTGTDLATSRLSPHVRFGEISPRVLFWSVVNAGATAQMNGDA
QGLCAARVFLKNMSLREFGYYMLRRYPSAAWKPIMPEFEVFPWEDDSDGELTRAWKNGTTGFPIVDAAMRQLI
REGWLHNRMRFLAASFFCKYLLLPWPVGAAHMVRTLVDGDEACNSLGWQWTAGCNSDSFPFSTLVNPLSLHTH
TQSRTRAAAYVRKYVPELAHLPDSLVFTPWKASLEECAKYNITLLPLRNYTDISQSLQFNARSGDRDMAYYPS
RIVTGPQARYRARRAMEVMRRIFTAQKQCRTLLVDHTQPFPGDEINPLQLESYGMVLDVDHQGDGLSNKKSIS
LQETTSFVSTITTQRKNTRSRDEQDSLLVTPARKRMRTQTGEEDRTPHVDAVQLRTPSTRRPHIVQTEVPVPK
AITERADTKATGPNMLARLGDVGSVADQNVHIQIPKTTPAASRERISETPVGGGHRNPKRPRSPSLNAVTLES
TPQLSDPSGSKTPKLTDDRLSVSSLLSPNHSLPTHPVSQEPRPNHRRAVAAELNFAMSGLPRLAQAASIQCAA
RFTPHAGTMPNMSSEAVVSPHLSTCPTTPALSHGRHPSPEGEAPRSKGPNKLDANTEMSAPDSRVSVGFSVTT
GRKDSTPQLAQPPVNSRTAERPSLTVPSRMIRSSETFSNHSSYVVAPAPAGNQGVVVGMSRSMMQGNEGANSV
AFNQATAAPTHMFHPSNIPVALMGTHIGSPQNQGTHMLMPFTPAAGHIPMQPITHHPGAVQAQGTPQGYVNMR
AMGAPAQQTPGAPFTWYPTMHPYMGFRPAVLPQLQHPLMSSGHPALANPYQLDRTNVRSRPMQQNMAVPVPSQ
NTGAQSPRRVCPTTPLERQGIARRMAAMDYTDETYGGKHWEQWQAIALHLLNQYEFSEDTDRDTTRAYVRLCV
LKDELRDANPSGPRVTVNHCKEVFRILNLPVTGEWDRRGHGGVRGPYVYGCMRKNGQYSSRR
>Gracilinanus_agil__XP_044525951.1
MAPKKRSLGDNDEQEKTETQENKAKRKPLQKHQFSKSNVVQKEEENKKEGEKKGGAEGLHEVVKQSRLQTASS
VLEFRFNKQRVRLLSQDCHLQDHSQAFVYWMSRDQRVQDNWAFLYAQRLALKQKLPLHVCFCLAPCFLGATIR
HYDFMLRGLEEVAEECEKLHIPFHLLLGLPKDVLPAFVQAHGIGGIVTDFSPLLHHTQWVKDVQDGLPKQVPF
VQVDAHNIVPCWIASDKQEYGARTIRHKIHDRLPHFLTEFPPIICHPYPSNIQAEPVDWNACRAGLQVDRSVK
EVSWAKPGTASGLTMLQSFISQRLPYFGSDRNNPNKDALSNLSPWFHFGQVSVQRAILEVQKHRSRYPDSVAN
FVEEAVVRRELADNFCFYNKNYDKLEGAYDWAQTTLRLHAKDKRPHLYSLEQLESGKTHDPLWNAAQMQMVQE
GKMHGFLRMYWAKKILEWTRSPEEALEFAIYLNDRFQLDGRDPNGYVGCMWSICGIHDQGWAEREIFGKIRYM
NYAGCKRKFDVAEFERKYSPAD
>Gracilinanus_agil__XP_044533177.1
MGVNAVHWFRKGLRLHDNPALKECIEGADTVRCVYILDPWFAGSSNVGINRWRFLLQCLEDLDANLRKLNSRL
FVIRGQPADVFPRLFKEWNIAKLSIEYDSEPFGKERDAAIKKLASEAGVEVIVRISHTLYDLDKIIELNGGQP
PLTYKRFQTLISKMEPLATPVETITPEVMHKCVTPLSDEHDEKYGVPSLEELGFDTDGLPSAVWPGGETEALT
RLERHLERKAWVANFERPRMNANSLLASPTGLSPYLRFGCLSCRLFYFKLTDLYKKVKKNSSPPLSLYGQLLW
REFFYTAATNNPRFDKMEGNPICVQIPWDRNPEALAKWAEGRTGFPWIDAIMTQLRQEGWIHHLARHAVACFL
TRGDLWISWEEGMKVFEELLLDADWSINAGSWMWLSCSSFFQQFFHCYCPVGFGRRTDPNGDYIRRYLPVLRG
FPAKYIYDPWNAPEGIQKVAKCLIGINYPKPMVNHAEASRLNIERMKQIYQQLSRYRGLGLLASVPSNPNGNG
SLMGYTPGENIPGCSSGGGAPVGASDGQILQACVLPEPPTGTSGVQQPGYSQGSGISHYSHEDNQQAYMLKQG
RSSLGVGGGKRPRQEEETQSINPKVQRQSTN
>Gracilinanus_agil__XP_044536012.1
MTVTAAATAPTPAPAPATASAPFPGPAPEEGASSVHWFRKGLRLHDNPALQAALRGARCVRCVYILDPWFAAS
SSVGINRWRFLLQSLEDLDISLRKLNSRLFVVRGQPTDVFPRLFKEWGVTRLTFEYDSEPFGKERDTAIVKMA

KEAGVEVVTENSHTLYDLDRIIELNGQKPPLTYKRFQAIISRMELPRKPVNSVTSQQMQRCQAEIQENHDDAY
GVPSLEELGFPTDGLGPAVWQGGETEALARLDKHLERKAWVANYERPRMNATSLLASPTGLSPYLRFGCLSCR
LFYYRLWELYKKVKRNNTPPLSLYGQLLWREFFYTAATNNPKFDRMEGNPICIQIPWDRNPEALAKWAEGKTG
FPWIDAIMTQLRQEGWIHHLARHAVACFLTRGDLWVSWESGVRVFDELLLDADFSVNAGSWMWLSCSAFFQQF
FHCYCPVGFGRRTDPSGDYVRRYLPQLKGFPARYIYEPWNAPEPVQKAAKCVIGVDYPRPIVNHAETSRLNIE
RMKQIYQQLSRYRGLCLLASVPSCLEDLSSPMVEASMGQAGAIGGSGLKPLPCSPASPKRKLEATEEAPGEEH
SKRARVMVAAALLPVPELAGRDV
>Grus_ame_NWH19278.1_CRY1
MPHRTIHLFRKGLRLHDNPTLLAALESSEAVYPVYILDRKFMTSVMHIGALRWHFLLQSLEDLQQNLCQLGSC
LLVIQGEYESVLRDHIQKWNITQVTLDAEMEPFYKEMEANIRCLGEELGFEVLSLVGHSLYDTERILDLSGGT
PPLTYKRFLHILSLLGDPEVPVRNLMAEDFQRCRSPDPALAECYRVPLPVDLKIPLETLSPWKGGETEGLRRL
EQHLTDQGWVASFTKPKTIPNSLLPSTTGLSPYFSVGCLSVRTFFYRLSNIYAQAKHHSLPPVSLQGQLLWRE
FFYTVASATPNFTKMAGNPICLQISWYEDAERLQKWKMAQTGFPWIDAIMTQLRQEGWIHHLARHAVACFLTR
GDLWISWEEGMKVFEELLLDADYSINAGNWMWLSASAFFHQYTRIFCPVRFGKRTDPEGQYIRKYLPILKNYP
SKYIYEPWTASEEEQKQAGCIIGQDYPFPMVNHKEASDHNLQLMKQVREEQYRTAQLTRDDTDDPMETKVKRD
HSEENVSKGKVARMTE
>Grus_ame_NWH19651.1_CRYD_
KVDYDVCSNYGNWLYSAGIGNDPRDNRKFNMIKQGLDYDGNGDYVRLWVPELRGIKGADVHTPWALNSAALSQ
AGVTLSETYPQPVVTAPEWSRHISQRLQGRSPHPRGSRGPARTPVQHRGIDFYFSHKKDV
>Grus_ame_NWH23856.1_PHR_lyase
DNWAFLYAQRLALKQELPLHVCFCLVPKFLDATIRHYGFMLRGLQEVAEECAELNIPFHLLLGYAKDVLPTFV
VEHGVGGLVTDFCPLRIPRQWVEDVRERLPEDVPFAQVDAHNIVPCWVASPKQEYSARTIRGKIHAQLPEFLT
EFPPVIRHPYPPSCPAEPIAWEACYSSLQVDRTVKEVEWATPGTAAGLAVLQSFIMERLKSFGSHRNDPNKAA
LSNLSPWFHFGQVSTQRAILEVQKHRGKYKESVDVFVEEAVVRRELAENFCYYNENYDSVQGAYDWAQTTLKL
HAKDKRPFLYKLQELEQGTTHDPLWNAAQLQMVREGKMHGFLRMYWAKKILEWTRSPEEALRFAIYLNDRYEL
DGRDPNGYVGKVDALSPSPAGCLWSICGIHDQGWAERAIFGKIRYMNYAGCKRKFDVGQFERRYAPRM
>Grus_ame_NWH23941.1_CRY1
RFLLQCLEDLDANLRKLNSRLFVIRGQPADVFPRLFKEWNIAKLSIEYDSEPFGKERDAAIKKLASEAGVEVI
VRISHTLYDLDKIIELNGGQPPLTYKRFQTLISRMEPLEMPVETITPEVMEKCTTPVSDDHDEKYGVPSLEEL
GFDTDGLPSAVWPGGETEALTRLERHLERKAWVANFERPRMNANSLLASPTGLSPYLRFGCLSCRLFYFKLTD
LYKKVKKNSSPPLSLYGQLLWREFFYTAATNNPRFDKMEGNPICVQIPWDKNPEALAKWAEGRTGFPWIDAIM
TQLRQEGWIHHLARHAVACFLTRGDLWISWEEGMKVFEELLLDADWSVNAGSWMWLSCSSFFQQFFHCYCPVG
FGRRTDPNGDYIRRYLPVLRGFPAKYIYDPWNAPESVQKAAKCIIGVNYPKPMVNHAEASRLNIERMKQIYQQ
LSRYRGLGLLATVPSNPNGNGNGGLMGYSPGESISGCGSTGGHLFPASPGAQLGPGDGHTVVQPCALGESHTG
ASGIQQQGYCQASSILHYAHGDNQQSHFLQAGKRPNPEEETQSVGPKVQRQSTN
>Grus_americana_NWH22854.1_CRY2_prote
RFLLQSLEDLDNSLRKLNSRLFVVRGQPTDVFPRLFKEWGVTRLTFEYDSEPFGKERDAAIIKLAKEAGVEVV
IENSHTLYDLDRIIELNGHKPPLTYKRFQAIISRMELPKKPVSTVMSQQMETCKVDIQENHDDVYGVPSLEEL
GFPTDGLAPAVWQGGETEALARLDKHLERKAWVANYERPRMNANSLLASPTGLSPYLRFGCLSCRLFYYRLWE
LYKKVKRNSTPPLSLYGQLLWREFFYTAATNNPKFDRMEGNPICIQIPWDRNPEALAKWAEGKTGFPWIDAIM
TQLRQEGWIHHLARHAVACFLTRGDLWISWESGVRVFDELLLDADFSVNAGSWMWLSCSAFFQQFFHCYCPVG
FGRRTDPSGDYVKRYLPKLKGFPSRYIYEPWNAPESVQKAAKCIIGVDYPKPMVNHAETSRLNIERMKQIYQQ
LSRYRGLCLLASVPSCVEDLSGPVTDSASGQGCSTSTVMRLSQADQASPKRKHEGGEELCTEELYKRAKVTGL
PAPEIPGKSL
>Gryllus_bim_BAX56238.1_cry_1
MTELSEVRKNASVLWFRHGLRLHDNPALQETLKDNDVFLPIFIFDGKTAGTNIIGYNRMKFLLESLQDIDNKL
QEVGGKLSLIRGNPVEVFHFISEKFNLRKICFEQDCEPIWQERDNAVKKFCLENKIECFEKVSHTLWDPYLVQ
KTNGGVPPLTFQMFLHTISVLGSPPRPIGDVDWTLVEFGTLTPLPFPHNLHIFQHFPTPEDFGIYPEEKNGDR
IIQWIGGETQALIHLKERLKVEEDAFRKGYYLPNQARPDLLGPPSSQSAALRFGCLSVRRFYWSIQDIFTKIH
GNNQVPNQHITSQLIWREFFYTMSVGNKYYDEMERNPICLNIPWKENTKGHLELWEQGKTGYPFIDAVMRQLV
QEGWIHHVARNAVACFLTRGVLWISWEAGLKFFLKYLLDADWSVCAGNWMWVSSSAFEQLLDCSHCMCPVNYG
RRLDPWGEYIKRYIPELRNYPVEYLYEPWKAPLHVQEQAGCIVGKDYPQRMIDHLQASEKNRQYMEDIRNQLM
NPPPHCRPSNEKETREFMWCHDNCFDH
>Gryllus_bim_BAX56244.1_cry_2_tran
MTDVYQQLPLAAARGAGGPPGYGAPPAKHTVHWFRKGLRLHDNPSLREGLKGASTFRCIFILDPWFAGSSNVG
INKWRFLLQCLEDLDQSLRKLNSRLFVVRGQPADALPKLFKEWGTTNLTFEEDPEPFGRVRDQNIMAMCREMG
ISVVSRVSHTLYRLESIIEKNGGKPPLTYHQFQTVVASMDSPPAAEPRITARAIEGIHTPLHDDHDDRFGVPT
LEELGFDTEGLLPPVWTGGESEALARLERHLERKAWVASFGRPKMTPQSLQASQTGLSPYLRFGCLSTRLFYY
QLTDLYKKIKKACPPLSLHGQLLWREFFYCAATNNPNFDRMNGNPICVQIPWDRNPEALAKWATGQTGFPWID
AIMTQLREEGWIHHLARHAVACFLTRGDLWISWEEGMKVFEELLLDADWSVNAGMWMWLSCSSFFQQFFHCYC

PVRFGRKADPNGDYIRKYLPVLKNFPAKYIHEPWNAPESVQRAARCIVGKEYSLPMVNHAVASRINIERMKQV
YQQLSKYRGPPGLLAAVPSSQQPNRQSNTNNPCSKYGSGGLGRIKQEEGDIVSDQEGKENNHKHHHTLHSLNQ
QHN
>Guillardia_the_XP_005829195.1_GUITHDRAFT
MQRLLELSSRDIPSAREDKRPVIVWFRQDLRLHDNPALLFAAQSGRNVIPVFVHVEQDDENWLLGGAAKFWLH
QSLTKLSESLERKYGSKLIIRAGSDTLAGLLDVMMETGASTLCFNRVYEPWKLARDAKIVETLTMNGLTCSSY
KAVVLFEPLEVVPDSKEGLMYGFGSVGFFLNACDGKEISDPLPPPEQLRPPRAWPGSLSIGDLRLYVMPCRRN
GQVVDWAKGMREFWEFGEEGALKALEEFVRFGCHHFEGRQRFRADRKYTAVISPYVRFGELSPRRIFHEIVVR
HRYKAKTFLRRLAWRDLAYWCLWKFPTMADHPLRPQYAHQTWDYDQKQLRAWQRGRTGFPLVDAAMRQLWVTG
WMPNYMRHVVAGFLIEHMNMHWVDGEKWFHDTLVDADVAINAYMWQNGGHSGLDQWNFVMHPVFAAKSCDPEG
DYVRRWVPELSKLPPEYIHCPWEAPVTLLASSGVALGRNYPHRIIADLEGARTRSLAAVNAVRCGVGKAMVLP
DGHERMILPDGR
>Guillardia_the_XP_005833658.1
MPVSVQLLEALDDACKEEDQEKICDVLDRLQETDLDVDFIVKHVGKKVKLLRTHSSAEVKKKAVALTQFWKEV
VKKKVESSGKGKGRAAVKVKEEEVKQEVDNAAKIGKDSIERSGKIAVLPERTRAINHKNVESCPKGKPVVYWM
SRDQRVQDNWAMLYAQQQALEAGAPLAVCFNLVDSFLGAGLRQFGFMLRGLKEVEEELKELNIPFFLLRGDCV
KTIPKFVKDNNVGLVVPVWVASDKQEVGARTLRPKIHRLLPKFHQNFPAVMSHPHPWKTSPPPTDFEAALKSL
KCDESIPEVTWAKPGSAAGLENLSKFLMQKFKNFNTKRNDPSEEVLSDMSPWLHFGQVAPARCALEAKSFSKM
HSESYASYIEELVVRRELSDNFCHYNKKYDSLEGCAGWAQETLKVHAGDKRPHVYSLAKLEEAKTHDDLWNAA
QLELVHRGKMHGFMRMYWAKKILEWTSSPEEALKFAILLNDKYNLDGRDPNGFVGCMWSIGGVHDMG
>Guillardia_the_XP_005838416.1_induced_by
MSGGRVVLWFRNDLRLTDNVLIAEASKRFLASNSALSVLPVYCFDPRFFAQSQYGPRKTGVLRAKFLLESVAD
LKKRLQGVGSDLLVLSGKPEVIIPRLMQSGEDTTVLAQEEVTDEELRVDRAVKRAIAPLGGKLHLLWGHTLFH
RDDLPYRQGLTDMPDVFTPFKEACERKSKVRKCFPYPVKGNLGVVPEDAKKLDEGLPSLESLGFSAQEVEGAA
KPDPRSVLQFVGGESAGLDRIQHYIWKQDCLKDYFETRNGMIGADYSSKFSAWLAHGCISPRFIYEEVQRYES
QRVKNKSTYWLIFELIWRDFFRYICMKHGNEVFKIGGIAHRSWKWEGEGEAFQRWVDGETGQPLVDANMRELK
YTGFMSNRGRQNVASFLTQNLNVDWRLGAAYFEETLLDHDVTANWGNWMFAAGLSGGRINKFNILKQSKDYDE
DGEYCRLWCPELRNVPVVKVHSPWQMSEKEQLDCNGQDVGQRAALPETRLREESR
>Gymnodraco_acut__XP_034056367.1
MSTSRTIICLLRNDLRLHDNELFHWAQRNADHIVPLYCFDPRHYVGTYNYNLPKTGPFRLRFLLDSIRDLRHT
LLHKGSNLVVRRGKPEEVVADLIKQLGSVSTVAFHEEVSSEELNVEKRVKDVCAQMKVKVHTCWGSTLYHRDD
LPFNNMSRLPDVYTQFRKAVESQSQXDPXSQHLSSXRPLPQGLEEGGIPTAEDLEQTEPVTDPRSAFPCTGGE
SQALARLKHYFWDTNAVATYKETRNGXIGVDYSTKFSSWLALGCISPRYIYHQIQQYEKERTANQSTYWVIFE
LLWRDYFKLVGVKYGIRLFQIKGLQEKSIPWKKDMKLFDAWKEGRTGVPFVDANMRELAMTGFMSNRGRQNVA
SFLTKDLCLDWRMGAEWFEYLLIDHDVCSNYGNWLYSAGIGNDPRGENRKFNMIKQGLDYDNNGDYVRQWVPE
LQGIMGSDVHTPRTLSTASLSHANVCLGETYYPAPIVMAPEWSRHANKKPSGAGPSARGKKGPSHTPKQHRDR
GIDFYFSKSKNL
>Gymnodraco_acut__XP_034071310.1
MLHSICRSSSVFRVVTHSYLDTTSVLSSKNKAFTCQAIMSEKKRKSTSAAAAAVKEPGAKQQKLDPTKEKGWL
QDSLKQQRTKNKQMKFNKERLRFISNTERIKQGSEGVLYWMTRDHRVQDNWALIHAQQLALKEKLPLHVCFCL
FVPKSELSTLRHYSFMLKGLKEVEKKCRSLDIQFHLLHGSAGDVLPGFVSDRELGAVVTDFSPLREPLQWLED
VKKTLPKDIPLIQVDAHNIVPCWVASPKLEYAARTIRGKLTKLLPQFLTDFPLVEKHPYTTARTAKLIDWEKT
LASLQLDRDVGEPEWAMPGTKGGVAMLESFIDERLKLFATQRNDPNIAGLSQLSPWIRFGHLSAQRVALQVQS
SGKCAGPSVATFIEELVVRRELTDNFCFYNEKYDSVEGTHDWAQKSLKAHAKDKREYLYTREQLEKAKTHDKL
WNASQYQMITEGKMHGFLRMYWAKKILEWTSSPEEALSIALYLNDRYSLDGQDPNGFVGCMWSIGGIHDRAWG
ERKVFGKVRYMNYKGCQRKFDVARFEKKYCPKNL
>Gymnodraco_acut__XP_034081741.1
MVINTIHWFRKGLRLHDNPSLKDSLLGADTVRCVYILDPWFAGSSNVGINRWRFLLQSLEDLDSSLRKLNSRL
FVIRGQPTDVFPRLFQEWNISRLSFEYDSEPFGKERDAAIKKLACEAGVEVTVRISHTLYDLDKIIELNGGQS
PLTYKRFQTLISRMDAVEEPADSITADIMGKCRTPLSENHDDKFGVPSLEELGFDTEGLSSAVWPGGETEALT
RLERHLERKAWVANFERPRMNANSLLASPTGLSPYLRFGCLSCRLFYFKLTDLYKKVKKNSSPPLSLYGQLLW
REFFYTAATNNPCFDTMESNPICVQIPWDRNPEALTKWAEGRTGFPWIDAIMTQLRQEGWIHHLARHAVACFL
TRGDLWISWEEGVKVFEELLLDADWSVNAGSWMWLSCSSFFQQFFHCYCPVGFGRRTDPNGDYIRRYLPILRG
FPAKYIYDPWNAPEGVQNAAKCIIGVHYPKPMVNHAETSRLNIERMKQIYQQLSCYRGLGLLASVPSISNGNG
ETSSEGMGLSADGTHNAAAAPSGHQMGIPSQGDWQSGVNELMQGETQTSSSSQQQGYGGNSSSMLCYSQGVQK
IQKGPEHHVSTLSGGKRPCEDPRNGRGSKIQRQSTH
>Gymnodraco_acut__XP_034088101.1
MAHTCIHWFRKGLRLHDNPALMAALRDCKELYPVFILDPYIYNNTLVGINRWRFLIGCLKDLDCSLRKLNSRL
FVVRGKPEEVFPKLFEKWKVTKLTYEYDTEPYSLSRDKTVTTLAKEHGVEVVYKISHTIHDMDRIIEENNGKA
PLTYNRFRTILKTIGNPKKPIPAPAIEDTKDVKTPCSENHDKNDGIPTLEDLGHDTTTLGEEKFPGGEQEALR

RLDENMARKDWVCKFEKPQTSPNSLSPSTTVLSPYVTFGCLSARTFWWRVTEVYQGKKHSEPPVSLHGQLLWR
EFYYAAGVGIPNFDKMEGNPVCTQVDWDTNHEYLAAWREARTGLPFIDAIMTQLRQEGWIHHLARHAVACFLT
RGDLWINWEEGQKVFEYLLLHGDWALNAGNWQWLSASAFFHQYFRVYSPVAFGKKTDKNGDYIKKYLPLLKKF
PAQYIYEPWKAPRSVQQAAGCIVGKDYPQPIVQHEVISKKNIQRMKLAYAKRSAESAESPNQSPSKKQGVKRK
AKSVIDMLQKKDKK
>Gymnodraco_acut__XP_034089551.1
MAPSAAASEDRKALVRRMLIDVLMGREDPEGFFAMCVSAMGPQETRSQFLALIQPLSTANSSLHSGLTSIYEQ
YFSKTEDDELQLALALSLLEVKNDQLSTPSQDSPLQPPRDRPNQRSPAQKSTVCQPQGNSNTKPADGVGRREH
TITTQTEARVKASPLQTFRETELQKNNVDKYKMGTPGTSLARCVSSPSGLFLKQNTIEKGNQMRNEGDFNQPE
TPKRSKNRRQRRKGAGQKVVGLPCSPSAQPPVLLWFRRDLRLGDNPALIGSLEVGSPIIPVFIWSPEEEEGPG
ITVAMGGACTYWLHQALSCFSSSLERIGSHLVFLKANGSSLCTLKELVRETGARTVLANALYEPWLKERDDDV
ESDLQKDGVECKMYHSYCLRDPYSVSTEGVGLRGIGSVSHFMNCCRQNPGSALGAPLDPPVSLPTPAYWPQGV
SLNVLGLGRMPRRKDGTTIDWAANIRKSWDFSEGGACARLEAFLNDGVYRYEKESGRADAPNTSCLSPYLHFG
QLSPRWLLWDAKGARCRPPKFQRKLAWRDLAYWQLTLFPDLPWESLRPPYKALRWSSDRDHLKAWQRGRTGYP
LVDAAMRQLWLTGWMNNYMRHVVASFLIAYLYLPWQEGYRWFQDTLVDADVAIDAMMWQNGGMCGLDHWNFVM
HPVDAAMTCDPYGSYVRKWCSELAELPDELVHKPWKCPAPMLRRAGLVFGQTYPERIVTDLEERRSRSLQDVA
LVRKEFGQYVDKRSGCDLVPLPQRLVSEALGLSHRDDGVVTEGQQFLLPVITRMEFKHQLEDPDADAVSNPYN
AVLKGYVSRKRDETIAFLNERDFTASVMYEGVQRKERLESDYRRIEGLPGLPAPRGGARRTPTAKDRFSVVPG
GAVSSLR
>Haemaphysalis_long__KAH9369986.1
MSLASQQQATPHQQAPGPGGAERHVVHWFRKGLRLHDQPALRLGLQGARTCRCVYILDPWFAGSSNAGLNKWR
FLLQSLEDLDARLRKLNSRLFVIRGQPADVFPKLFKEWRVTHLTFEKDPEPYGAIRDQSISALANEMGVTVVC
EPSHTLYPLERILERHGGKAPLTYRQFQGVLVSMEPPPAPVPAPDALPRTPIDDDHDERFAVPTLAELGTFSI
RNERPLTRTHTVHLHAGFDTDNLKDAVWPGGETEALTRLERHLERKAWVASFGSPKMTPKSLLASQTGLSPYL
RFGCLSARLFYHQLADLYRKIKKSNPPLSLQGQLLWREFFYCAATRNPNFDRMHNNPICVQIPWDVNAEALAK
WANGQTGYPWIDAIMRQLREEGWIHHVARHAVACFLTRGDLWLSWEEGMKVFDELLLDADWSVNAGSWMWLSC
SSFFQQFFHLYCPVRFGRKADPSGDFIRRYLPMLKHFPSKYIHEPWTAPESVQVAARCVVGRDYPLPMVNHQD
ASHVNLQRIRQVYQQLTQCAKAPGKLLRSVLAKFQRSTAIIDVAFNDIYASLQR
>Halcyon_sene__NXD76476.1
DNWAFLYAQRLALKQELPLRVCFCLVPKFLDATIRHYGFMLKGLQEVAKECAELNIPFHLLLGYAKDMLPAFV
VEHGVGGLVTDFCPLRLPRQWVEDIRDRLPEDVPFAQVDAHNIVPCWVTSPKQEYSARTIRGKIHAQLPEFLT
EFPPVIRHPYSPSCPTEPITWEACYSSLQVDRTVTEVEWATPGTAAGLAVLQSFIAERLKSFGSHRNDPNKAA
LSNLSPWFHFGQVSTQRAILEVQKHRGKYKESVDAFVEEAVVRRELAENFCYYNENYDSVQGAYDWAQTTLKI
HAKDKRPFLYELQELEQGTTHDPLWNAAQLQMVREGKMHGFLRMYWAKKILEWTRSPEEALRFAIYLNDRYEL
DGRDPNGYVGKLWGCLWSICGIHDQGWAERPVFGKIRYMNYAGCKRKFDVGQFEHRYSPRP
>Halcyon_sene__NXD77464.1
KVDYDVCSNYGNWLYSAGIGNDPRDNRKFNMLKQGLDYDGDGDYVRLWVPELRGIKGADIHAPWALTSAALSQ
AGVILGETYPRPVVTAPEWSRHITQRPQGRSPRAGGRRGPAHPATQRKDRGIDFYFSPKKDA
>Halcyon_sene__NXD80752.1
RFLLQSLEDLDNSLRKLNSRLFVVRGQPTDVFPRLFKEWGVTRLTFEYDSEPFGKERDAAIVKLAKEAGVEVV
IENSHTLYDLDRIIELNGHKPPLTYKRFQAIISRMELPKKPVSTVIRQQMETCKVDIQENHDDVYGVPSLEEL
GFPTDGLAPAVWQGGETEALARLDKHLERKAWVANYERPRMNANSLLASPTGLSPYLRFGCLSCRLFYYRLWE
LYKKVKRNSTPPLSLYGQLLWREFFYTAATNNPKFDRMEGNPICIQIPWDRNPEALAKWAEGKTGFPWIDAIM
TQLRQEGWIHHLARHAVACFLTRGDLWISWESGVRVFDELLLDADFSVNAGSWMWLSCSAFFQQFFHCYCPVG
FGRRTDPSGDYVKRYLPKLKGFPSRYIYEPWNAPESVQKAAKCIIGVDYPKPMVNHAETSRLNIERMKQIYQQ
LSRYRGLCLLASVPSCVEDLSGPVTDSASGQGCSTSTVMRLSQADQASPKRKHEGTEELCTEELYKRAKVTGL
PAAEIPGKNL
>Halcyon_sene__NXD85396.1
MGVNAVHWFRKGLRLHDNPALRECIQGADTVRCVYILDPWFAGSSNVGINRWRFLLQCLEDLDANLRKLNSRL
FVIRGQPADVFPRLFKEWNIAKLSIEYDSEPFGKERDAAIKKLASEAGVEVIVRISHTLYDLDKIIELNGGQP
PLTYKRFQTLISRMEPLEIPVETITPEVMEKCTTPVSDDHDEKYGVPSLEELGFDTDGLPSAVWPGGETEALT
RLERHLERKAWVANFERPRMNANSLLASPTGLSPYLRFGCLSCRLFYFKLTDLYKKVKKNSSPPLSLYGQLLW
REFFYTAATNNPRFDKMEGNPICVQIPWDKNPEALAKWAEGRTGFPWIDAIMTQLRQEGWIHHLARHAVACFL
TRGDLWISWEEGMKVFEELLLDADWSVNAGSWMWLSCSSFFQQFFHCYCPVGFGRRTDPNGDYIRRYLPVLRG
FPAKYIYDPWNAPESVQKAAKCVIGVNYPKPMVNHAEASRLNIERMKQIYQQLSRYRGLGLLATVPSNPNGNG
NGGLMGYSPGESISGCGSTGGHVFPASPGAQLGTGDGHTVVQPCALGDSHTGASGGQQQGYCQASSILHYAHG
DNQQSHLLQAGRTSLATGISTGKRPNPEEETQSVGPKVQRQSTS
>Halcyon_sene__NXD88195.1
MSHRTIHLFRKGLRLHDNPTLLAALESSEVLYPVYILDRKFMTSVMHIGPLRWHFLLQSLEDLQKNLGQLGSC
LLVLQGEYESVLRDHIQEWNITQVTLDAEMEPFYKEMEANIRRLGEELGFEVLSLVGHSLYDTKRILDLNGGT

PPLTYKKFLHILSLLGDPELPVRNLTAEDFQRCGAPDPGLAEQYRVPLPVDLKMPLENLSPWRGGETEGLQRL
EQHLTDQVSPYCTWVLLVALAGLSLVSSSYMCLSPEAFTLYLVQAKHHSLPPVSLQGQLLWREFFYTVASATP
NFTQMVGNPICLQIRWYEDAERLHKWKMVMRG
>Haliotis_rubr__XP_046546140.1
MSHKTIHWFRKGLRIHDNPALLAAVKDGEALWPIFILDPWFAKHARVGINRWRFLLQSLKDLDENLKKVNSRL
FVIQGKPAEVFPGLFKQWEITRLTFEVDTEPYAVARDEEVQQLANEHGVEVVTEVSHTLFDVKKTIQKNGGSP
PLTYQRLQTLISQMGAPPSPVNTLTLEEMKDCRTPEADDTEYRVPTLEELDKCQDDLGPNLYPGGETEALVRM
ERYLKKTNWVCSFEKPKTEPNSLQPSTTVLSPYLKFGCLSARTFYFRLLDVYRVNKKHTQPPVSLHGQLLWRE
FFYTVASGTANFDQMEGNSVCTQVPWDDNKELLEAWTQARTGYPFIDAIMTQLRQEGWIHHLARHAVACFLTR
GDLWISWEEGQKVFEELLLDADWSLNAGNWMWLSASAFFHQYFRVYSPIAFGKKTDKNGDYIRKYLPQLKKLP
AEYIYEPWKSPLSVQKAAGCIIGKDYPKPIVDHDLVRKANIKKMASAYAKRKEMAGTTKKSAQSPAKKRKSSA
EEDGKKKKFKSGSKKMTDFFNK
>Haliotis_rubr__XP_046567787.1
MSVNNIKAYNTEGQPYLSEHTMEERPEVFNKWEKGMTGFPWIDACMRQLRSEGWIHHVCRHAVACFLTRGDLW
ISWEEGLRTFLTYLLDADWSVCAGNWMWVSSSAFERILQCSKCFCPVGYGRRIDPSGEFVRRYIPELKDMPLV
FLYEPWKAPLEIQEKAKCVIGKDYPSPIVDHRKVSKQNKEMMDAVMHVLKDKKISHCAPTSVEEVKTFVWVPE
HTFCEMETDHKKV
>Haliotis_rubr__XP_046571771.1
MWSCTKLIHCGTGWLRSRQGLHLTHRLSSSSVLFLGRLSSAMSDKGKSSDLKRKAEEDSGSAEKKVKVEASKK
SGDLLSDVIESRKSVCESVCKFKFNKKRVRVLSSAEEFPDDCDGVVYWMSRDQRVQDNWAFLYAQRLALKMEV
PLHVCFCLVPRFLEATIRQFGFMLKGLQEVEQECKTLNISFHLLIGYAKDVLPDFVKSNNIGGVVTDFSPLRT
PMTWVTDVKERLPKDIPMCQIDAHNVVPCWEASPKLEYGARTIRKKNHDKLPEFLTAFPPVCKHPHDAKVKSE
LVNWEAAYASLEVDRSVKEVDWATPGTKGGLKMLESFCKERLKYFNADRNNPNKNALSNLSPWIHFGQISVQR
CILTVKQYRSKGQESVNAFIEEAVVRRELADNFCFYNEHYDTIEGAYDWAKTSLKLHAGDKRPYLYSRQQLED
GRTHDKLWNAAQLQMVNEGKMHGFLRMYWAKKILEWTCSPEDALKDAIFLNDKYNLDGRDPNGYVGCMWSICG
IHDQGWKEREVFGKIRYMNYKGCTRKFDVPAFERKYKVKKI
>Haliotis_rubr__XP_046575178.1
MNRKMASVTGTIICLFRNDLRVHDNEALHWAIKNGTHVLPLYCFDPRHYKGTYHFNFPKTGANRFKFLLESVA
DLRQSLQSKGSNLIVRVGKPEEVVPQLIECVGKNMVQGLVYQGEVTEEELTVEQGLNKKCGVKVHTVWGHTLH
HKEDLPFNPKNVPDVYTQFRKRVEEQAPVRSVIPTPDRLKPLPPGIEEGTIPTPHDLGVQESTTDPRTAFPFA
GGETTGVARLQNYLWDTDHVAQYKETRNGMIGADFSTKFSPWLAHGCVSPRFIYSEIKKYEAKRTANQSTYWV
IFELLWRDYFRFVALKYGNKIFFSGGILGKDIPWKKDMRQFEAWKSGTTGVPYIDANMRELLATGFMSNRGRQ
NVASFLTKDLKLDWRLGAEWFESMLIDHDVCSNYGNWLYSAGIGNDPRQDRKFNIVKQGLDYDAQGDYVRLWV
PELSKVTGGDVHTVWALSNTVLSHAEVSLGETYPKPLLVAPEWGRHSAKSRGRPQGKGDKPQRNRGIDFYFKS
S
>Haliotis_rubr__XP_046585585.1
MSRPRKNTVHWFRKGLRLHDNPALREGIQDSTSYRCIYILDPWFAGASQVGINKWRFLLECLEDLDKSLRQLG
SRLFVIRGQPTEVFPKVFKEWNITHLSFEEDPEPFGKDRDASICSLATECGVKVIIRTSHTLYKLEDVIKMNN
STHPLTYKRFLSVLAQMKPPPMPEETIKPDLIGQSVTPVTLDHDEKYGVLKLEELGFDIDGIHPVIFRGGESE
ALTRLYRHLERKAWVASFEKPKMSPQSLFPSQTGLSPYLRFGCLSPRLFYWKLRELYKKVKKGNEPPLALHGQ
LLWREFFYTVATNNPNFDTMIGNPMCVQIPWDRNPEALAKWAEGQTGFPWIDAIMVQLRQEGWIHHLARHSVA
CFLTRGDLWISWEEGMKVFDELLLDADWSVNAGTWMWLSCSSFFQQFFHCYCPVGFGKRADPSGNFVRHYLPV
LKAFPTKYIYEPWLAPESVQKAARCIVGKDYPLPIVNHAVVSRINLERIRQVFKRLTVKANLSIPRDLGSPIF
KYTKEASQTAIKTHPSAEEGTAH
>Haliotis_rufe__XP_046326325.1
MSRDQRVQDNWAFLYAQRLALKMEVPLHVCFCLVPRFLEATIRQFGFMLTGLQEVEQECKTLNISFHLLIGYA
KDVLPDFVKDNNIGGVVTDFAPLRTPMAWVTDVKERLPKDIPMCQIDAHNIVPCWEASPKLEYGARTIRKKIT
DKLPEFLTAFPPVCKHPHNAAVKSEPVKWEAAYASLEVDRSVKEVDWAIPGTKGGLQMLESFCKDRLKYFNAD
RNNPNKNAVSNLSPWIHFGQISVQRCILTVKQFRSKGTESVNAFIEEAVVRRELADNFCFYNKHYDSIEGAYD
WAKTSLKLHAGDKRPYLYSRQQLEDGKTHDKLWNAAQLQMVNEGKMHGFLRMYWAKKILEWTRSPEDALKDSI
FLNDKYNLDGRDPNGYVGCMWSICGIHDQGWKEREVFGKIRYMNYKGCTRKFDVPAFERKYKVKKI
>Haliotis_rufe__XP_046347788.1
MDYSGEEEHCRKHVTAMWFRHGLRFHDNPTLIKALKTTKTFYPIFIFDGKVAGTSLIAYPRMTFLLECLHDLD
QTCKKYGSRLYVFQGSPVEVFSQIKKDWGITQIAFEQDTEPIWFERDKTVKDFCEESHIEYVEEIGHTLWDPR
EVIELNKGSPPVTYGLFNQVTSILGPPPRPVADPDFTGINLPVTPDHDQFRIPTGEELGVKKENDVLECQKQY
IGGETQALKLLAGRIPVEEKAFKMGFVMPNQARPNIVDPPLSMSPHLRYGCLSVRKFFWEIRDAHFRVCPNKP
LPEGATSQLIWREYFYTMSVNNIAYDTMKENPICLNIPWKKDPEMFNTWEKGMTGFPWIDACMRQLRSEGWIH
HVCRHAVACFLTRGDLWISWEEGLRTFLTYLLDADWSVCAGNWMWVSSSAFERILQCSKCFCPVGYGRRIDPS
GEFVRRYIPELKDMPLVFLYEPWKAPLETQQKANCVIGKDYPKPMVDHRKVSKENKEMMDGVMHFLKDKKISH
CAPTNIEEVKSFVWVPQHTFCEMETDLKKL

>Haliotis_rufe__XP_046351244.1
MNGIMASRTGTIICLFRNDLRLHDNEALSWAVKNGTHVLPLYCFDPRHYRGTYHFNLPKTGAHRLKFLLESVA
DLRQSLQSRGSNLMVRVGKPEEVVPQLIDSIGKTLVQGLVYQGEVTEEELTVEQGLDKKCGVKVHTVWGHTLH
HKEDVPFNPKNVPDVYTQFRKRVEDQAPVRSIIPTPDMLKPLPPGIEEGTIPTPCDLGVEESTKDPRTAFPFA
GGETTGVARLQNYLWETDNVSQYKETRNGMIGADFSTKFSPWLAHGCVSPRFIHSEIKKYEAKRTANQSTYWV
IFELLWRDYFRFVALKYGNKIFFSGGIVGKNIPWKRDMRQFEAWKRGTTGVPYIDANMRELLATGFMSNRGRQ
NVASFLTKDLKLDWRLGAEWFESMLIDHDVCSNYGNWLYSAGIGNDPRQDRKFNIVKQGLDYDAQGDYVRLWV
PELSKVMGGEVHTVWALSNTVLSHAEVSLGETYPKPLLVAPEWGRHSAKSRGGTRGRGDKPRQNRGIDFYFKN
S
>Haliotis_rufe__XP_046364930.1
MLYGCTDRVISAMSHKTIHWFRKGLRIHDNPALLAAVKDGEELWPIFILDPWFAKNARVGINRWRFLLQSLKN
LDENLKKVNSRLFVIQGSPTEVFPGLFKQWEITRLTFEVDTEPYAVARDEEVQQLAHQHGVEVVTEVSHTLFD
VKKTIQKNGGSPPLTYQRLQTLISQMGAPPSPANTLTKEEMKDCRTPQADDTEKYSVPALEDLDKCVDELGPN
LYPGGETEALTRMERYLKKTNWICSFEKPKTEPNSLQPSTTVLSPYLKFGCLSARTFYFRLLDVYRVNKKHTQ
PPVSLHGQLLWREFFYTVGSGTLNFDRMEGNSVCTQVPWENNKELLEAWTQARTGYPFIDAIMTQLRQEGWIH
HLARHAVACFLTRGDLWISWEEGQKVFEELLLDADWSRTGGNWMWLSASAFFHQYFRVYSPIAFGKKTDKNGD
YIRKYLPQLKKFPAQYIYEPWKSPQSVQKAAGCVIGKDYPKPIVDHDNVRKANIKKMAAAYAKRKEMATKEKS
TSSPAKKRKSSAMDDGTKKKSRSGDKKMTDFFNK
>Haliotis_rufe__XP_046370956.1
MSRPRKNTVHWFRKGLRLHDNPALREGIQDSFSYRCIYILDPWFAGASQVGINKWRFLLECLEDLDKSLRQLG
SRLFVIRGQPTEVFPKVFKEWNITHLSFEEDPEPFGKDRDASICSLATDCGVKTIIRTSHTLYKLEDVIKMNN
STHPLTYKRFLSVLAQMKPPPMPEETIKPDLIGHSVTPVTLDHDEKYGVLKLEELGFDIDGVHPVIFRGGESE
ALTRLYRHLERKAWVASFEKPKMSPQSLFPSQTGLSPYLRFGCLSPRLFYWKLRELYKKVKKGNEPPLALHGQ
LLWREFFYTVATNNPNFDTMIGNPMCVQIPWDRNPEALAKWAEGQTGFPWIDAIMVQLRQEGWIHHLARHSVA
CFLTRGDLWISWEEGMKVFDELLLDADWSVNAGTWMWLSCSSFFQQFFHCYCPVGFGKRADPSGNFVRHYLPV
LKAFPTKYIYEPWLAPESVQKAARCIVGKDYPLPIVNHAVVSRTNLERIRQVFKRLTVKANLSIPRDLGSPIF
KYTKGLTDVDVEASQAGIKAHPHTEEGTAH
>Halobacillus_sp._KGW1_WP_062514897.1
MTKRAVWFRRDLRLQDNTALAKALEHTKDGDELLLFFHIHPALNESFTARHDYFFQTLASFVEKAEDAGAPVH
FITGDTEDAFENLIETAGIDMVYFNRDETGFGYERDKQVEKYVTSLDVDVYHYLDHHIHGADEVKKQDGGFYK
VFTPYLKKWKQLPKPAVQKVDDELLKKAAVDERASFKEGESAYADLLEKIKADFGGVGEADAWARLEQFLDAD
IYDYDERRDFPAVDGTSLMSRYLRTGAISVRSVYHQIHDQVDFRKKTEGVETYISELAWRDFYNMIYHFYPNA
HDEEVVEKYQGIPWNNDEDVLERWKEGRTGFPIIDAAMRQLNESGWMHNRLRMAVASFLTKDLLMDWRKGERY
FQEKLVDYDPASNIGGWQWAASTGTDPVPYFRVFNPTRQSERFDPHGRFIKEWLPELRDVPKKYIHEPAKMPE
NEQEKAKCMIGEDYPEPMVDHKRMRERAIQLFKDKS
>Halobacillus_sp._WP_062515076.1_DASH_famil
MQNGALVWFRNDLRVHDHHPLHQAVQSGKPVIGMYVFDPAAYEQGEDGIRKTDRHRARFLIESIHDLRKNLGA
LGIPLIVRCRRATEAIHEIKEKIKIDAVYVHEEIGREEQTVEENVRQALPDTAFHVEHGHNLYLPDDLPFSIQ
ELPDTFSQFRKRLEKSGTAVRKAIAPPAPEGQAELPVPEGDIPTLETLGFDPADEAPRYPGGSSEARKRLIDY
IFTKDRLKIYKQTRNGMLKEDDSSKFSPYLANGCLSPRVVYEQIKAYERTNGANESTYMLYFELLWRDYFHLV
HRKYGDRIFYRSGLSGKAIPWERDEKLLQAWIDGKTGYPLVDAGMRELKETGFLSNRGRQNVASFFTKNLGLD
WRIGARWFESQLVDYDVSSNYGNWLYIAGVGNDAVPFRAFNVEKQAKDYDPDGSYLRHWLPELAALPADWIHQ
PREMGMIEQQQYGIVLGEHYPLPVVDLYESMRKRKEAFAAADR
>Halobacterium_hub_WP_059056769.1_DNA_photol
MQLFWHRRDLRTADNRGLAAAAESGEVVPVFCFDDVVLDHASPPRVAFMLDALADLRARYRDLSSDLLVRRGD
PSAVLSDLAEEYSADRVVWNHDYSGLARERDEAVRAALGDADVGHEQYHDAVFHEPGAIRTNAGDPYSVYTYF
WKKWRDREKPDPVPEPSAGDFADVSGEELPTLADLGFAEPDAALPDAGTEAARERLEDFCEDAVFRYEEARDY
PAEAATSRLSQDLKFGTIGVRELYERTEDAMAEADNDEERASVEEYQGQLAWREFYAQVLYFNPGVVSQNYKE
YEQPIEWREDDAELAAWKRGETGYPIVDAGMRQLREEAYMHNRVRMIVASFLTKDLLQDWREGYDHFREHLVD
HDTANDNGGWQWAASTGTDAQPYFRIFNPTTQGERYDPDAEYITEYVPELRDVPAEKIHAWPELDADERAALA
PDYPDPIVDHGERREQAISMFERARGDD
>Halteria_gra_TNV80968.1_FGO68_gene
MHRIIVWLRNDLRVHDNSALNWAINKGIKGTTKQIIPVYTFDPRFMRNKVEKYGIYKCGLTRARFLQESVQNL
RANLQDLGSQLLVTQELPEVFLPKLILKGQQNSIVFSQEVCYEEARIEQSVATEIKRNSKVHIERVWGSTIHH
IDDLQMDPENVASSVYTKFRQNTERDIVRDCLPTSKLGQLPFIEGDVPKAIQDAMRFAPSLMDLGFSEEEANT
PKDPRSSLEFVGGEDAGLERVKYYLWETKMIQKYKDTRNALMGADFSSKLSPWMACGNLSPRYVYWETKKFEQ
RFKENDSTRHFISELFWRDFCHWYAYCHGNGIFYEYGPLDKKGVPQWRVDQSVIQRWRQGLTGMPLVDALMRE
MNQSGFMSNRGRQIVASYFSLDLKQDWRYGAHHFEEKLIDHDVHSNYASWNMIAGIGPGKTNLFNVVKQSYDF
DADGEFIRTWVPELRNVPTDYIHEPWKMPFKLQQQCETVLGTNYPNPIPSRYTTKPSHQSGGSGLGYVKDY
>Hamiltosporidium_mag_TBU05018.1_CWI36_0685

MENRIIEMIENKNTKNILYLMQRDKRIHDNYSVLYSMELGNALSSEYFIGTILDEIKLNERQRTFVIEGLKEI
EDECKEYNIYFYLIDKLEEFCAQYNIDCIVTDFSPLKEFDKFYEKLRSLCKKHKYSFYVVDSHNMVPCKLIPT
YYKSSKAVKTRLYQKYNQYFKDFDTIKFYTNNDSKRCKEIKNNFPKTTVTNGFKGGYSNGMKEVESFFENRFE
NYDKYRNKPDYDVLSNLSPWIVSGQISAQKVVWLACHKFSNKNENLDTFLNEIFVWKETAEHFCLYNKDYDNI
NGALPWAYSTLMVHQEDERNNIYTLEQLERATTHDDLWNAGQKEMLITGKMHGYVRMYWAKQILKWIQDPKKA
VETAVQLNDKYSIDGNSPNGYLGVMWCICGSMDYGFAERPIIGKIRPMNAFKAPKYVAKWANKKI
>Helicoverpa_arm_ADN94465.2_cry_2
MSAAAETLPAPNARKHTPPATAAAPQPPSARRTNGKHIVHWFRKGLRLHDNPALREGLLDAATFRCVFIIDPW
FASSSNVGINKWRFLLQCLEDLDSSLRKLNSRLFVVRGQPADALPKLFREWGTTALTFEEDPEPYGRVRDHNI
MSKCREVGITVTSRVSHTLYKLDQIIERNGGKAPLTYHQFQALIASMPPPPPAEAPISAQMLNGATTPLNDDH
DDRFGVPTLEELGFETEGLKPPVWIGGESEALARLERHLERKAWVASFGRPKMTPQSLLASQTGLSPYLRFGC
LSTRLFYYQLTELYKRVKRVRPPLSLHGQILWREFFYCAATRNPNFDRMEGNPICVQIPWEKNQEALAKWASG
QTGFPWIDAIMIQLREEGWIHHLARHAVACFLTRGDLWISWEEGMKVFDELLLDADWSVNAGMWMWLSCSSFF
QQFFHCYCPVRFGRKTDPNGDFIRRYIPALKNMPTRYIHEPWVAPEAVQQSARCIIGRDYPMPMVDHSKASQV
NIERIKQVYAQLAKYKPQGTLNPNAVQRPNVMQSSPSPNSIITSINQSNYLCSQTPDPQPSPQIIPYKDNDVV
FQKPMNHRSMKPSFKQVVIVQKKQNTNVIQTVSNCTPQSKENYAVNGQMDNSYKTPINESLQSAKQENMTLKT
>Helicoverpa_arm_XP_021184238.1_cry-1_X1
MLGGSVLWFRHGLRLHDNPSLHSALEEKGFPFFPIFIFDGETAGTKLVGYNRMRYLLEALDDLDSQFKKFGGR
LIMLKGKPNVVFRRLWEEFGIRKLCFEQDCEPVWRARDDSVKSACKEIGVVCREHVSHTLWEPETVIKANGGI
PPLTYQMFLHTVATIGDPPRPVPNIDFTGVKFGSLPECFYQEFTVYDKTPKPEDLGVFLENEDIRMIRWVGGE
TTALKQMQQRLSVEYETFLRGSYLPTHGNPDLLGPPISLSPALRFGCLSVRSFYWAVQDLFRQVHQGRLTTNS
ASHFITGQLIWREYFYTMSVNNPNYGQMAGNPICLDIPWKNPEGDELQRWVEGRTGFPFVDAAMRQLRTEGWL
HHAARNTVASFLTRGTLWLSWEHGLNHFLKYLLDADWSVCAGNWMWVSSSAFEALLDSGECACPVRLGQRLDP
SGEYVRRYVPELARMPVEYIYEPWKAPIDVQERATCVIGKDYPAPVVNHLVAAQRNKNAMKWLSRTVADRLQK
DSWLDIVGELRHMLQKAPPHCCPSSEDEIRQFMWLNE
>Helicoverpa_arm_XP_021185587.1_photolyase
MRFPYLPSLIRTVNMASAPKKIKLSQTKLTSKSSSENKESNSNVEEFMKKLQDKREDTAKSILEYKFNKKRVR
IISLEQMVPDKCEGVVYWMSRDSRVQDNWAFLFAQKLALKNEVPLHVCFCLIAKYLDASVRQFDFLVKGLEKV
AADCKKLNISFHLLEGSGAEVLPQWVVKHNIGAVVCDFNPLRVPLGWLDGVKKKLKKGVPLIQVDAHNIVPCW
VASDKQEYSARTIRNKINSKLDEYLTEFPPVIKHPYTAKFEPEPIDWDEAIVSREADKNVGPVEWARPGYDNA
IKMLKSFLDQRLKVFATKRNDPTMNALSNLSPWFHFGQISVQRVALCVQQQKSKCTESVNSFLEEAIVRRELA
DNFCFYCEHYDSIKGASNWAQKTLDDHRKDKRTHIYTLEQLANSETHDDLWNSAQIQLVKEGKMHGFLRMYWA
KKILEWTPSVFGKIRYMNYDGCKRKFDIKAFIARYGGKVHKYVPKK
>Helicoverpa_arm_XP_021188646.1_cry-1
MTKVPSVIHWFRLDLRIHDNLALRNAINEAENRKHHLRPVYFIDPDIKSKVGINRLRFLVQSLQDLNENLKKL
NTRLYIIRGNAVEELPKLFKKWQVKYLTSQVDIDPIYVKQDEIIDKIAEKSDIFIVRRVQHTVYDVHSVLKKN
NGSVPLTYQKFLSLVQDVQVKECIEITKAVSDDCKPKDFDSKQYDVPNLDELDIDETALQPLKYPGGETEGVK
RLHMYMAKRDWVCKFEKPNSSPNSIEPSTTVLSPYISHGCLSAKLFYHKLQEALSGRKHSEPPVSLLGQLMWR
EFYYTAGAGTENFDKMVGNPVCTQIPWGKNDEHLKAWAEGRTGYPFVDAIMRQLKQEGWIHHLARHMVACFLT
RGDLWISWEEGAKVFEDYLLDYDWSLNAGNWMWLSASAFFYKFFRVYSPVAFGKKTDKEGLYIRKYVPELKKY
PTAFIYEPWKAPKNVQTTAGCIIGKDYPKRIVDHDTIHKENCQKMSVAYKLNKERKALKRPLT
>Hemibagrus_wyck__KAG7320415.1
MVVNTIHWFRKGLRLHDNPSLKESIRGADTVRCVYILDPWFAGSSNVGINRWRFLLQCLEDLDASLRKLNSRL
FVIRGQPTDVFPRLFKEWNITRLSYEYDSEPFGKERDAAIKKLASEAGVEVTVRISHTLYDLDKIIELNGGQS
PLTYKRFQTLISRMEAVETPAEAITAEVIGSCTTPLSDDHDEKFGVPSLEELGFDTEGLSSAVWPGGETEALT
RLERHLERKAWVANFERPRMNSNSLLASPTGLSPYLRFGCLSCRLFYFKLTDLYRKVKKNSSPPLSLYGQLLW
REFFYTAATNNPRFDKMEGNPICVQIPWDRNPEALAKWAEGRTGFPWIDAIMTQLRQEGWIHHLARHAVACFL
TRGDLWVSWEEGMKVFEELLLDADWSVNAGSWMWLSCSSFFQQFFHCYCPVGFGRRTDPNGDYIRRYLPVLRG
FPAKYIYDPWNAPESVQKSAKCVIGVHYPKPMVHHAEASRLNIERMKQIYQQLSCYRGLGLLAMMPPRTLLHP
LGGDITPALYRPLGNVTVRTLVMPQAQKSRDSTVLKQVPTCDHTVLELTDTHIH
>Hemibagrus_wyck__KAG7329621.1
MVVNSVHWFRKGLRLHDNPALQEALNGADTARCVYVLDPWFAGAANVGVNRWRFLLESLEDLNRSLRKLNSRL
FVVRGQPADVFPRLFKEWNVTRLTFESDSEPYGKERDGAIIKMAQEFSVETIVRHSHTLYNPDRIIEMNNNNP
PLTFKRFQAIVSRLELPKKPLPTITPEQMANCSTQVSENHDEHYAVPSLEELGFKTQGAGVPVWRGGETEALE
RLNKHLDRKAWVANFERPRIKAHSLFASPTGLSPYLCFGCLSSRVLYYHLRELYMKLRRRSSPPLSLFRQLLW
REFFYTAATNNPSFDRMEGNPICVQIPWDRNPEALAKWAEGRTGFPWIDAIMCQLRQEGWIHHLARHAVACFL
TRGDLWISWESGMKVFEELLLDADWSVNAGSWMWLSCSAFFQQFFHCYCPVGFGRRIDPSGDYIRRYIPELKD
YPSRYIYEPWNTPESIQKAANCIVGVDYPKPMINHAESSRLNIERMKQVYQQLSHYRGLSLLASVPTIQEEAE
PSMSDDSQASSSGAISTSHQPQLQSEPQPSQTDPDPNPDQDPDPEPSSLSAPQAVTEQMKVKEN
>Hemibagrus_wyck__KAG7329789.1

MAERKRNLKRKGEPLKGAEKLSAARGAEREAGWLQEKVAEIRRNSTECKFNMKRLRFLSDAQKVRQGCGAVLY
WMARDQRVQDNWALVYAQQLALEEKLPLHVCFCLVPRYLDSTYRQYAFMLKGLQEVAKECTSLDIQFHLLSGE
PELTLPGFVKHWDIGGVVTDFSPLRLPLQWTERVKKELPPDVPFIQVDAHNVVPCWEASNKLEYAARTIRGKI
TKLLPEFLTEFPPVEAHPHTSTKTAKKVNWEEVMSSLEVDRSVGEVVWAQPGMAGGVAMLESFIDKRLRLFAT
ERNNPNADALSQLSPWIHSGQLSAQRVLLEVQRWGKRLTESVASFKEELLVRRELSDNFCFYNDNYDNINGAY
DWAKKTLQDHAKDSRPYLYTREQLEHAETYDQLWNAAQRQLVLEGKMHGFMRMYWAKKILEWTSSPEEAVNIS
IYLNDRYALDGCDPNGYVGCMWSICGIHDQGWAERHIFGKVRYMNYAGCKRKFDVGQFERRYTGKKA
>Hemiprocne_coma__NXG55160.1
RFLLQSLEDLDNSLRKLNSRLFVVRGQPTDVFPRLFKEWGVTRLTFEYDSEPFGKERDAAIVKLAKEAGVEVV
IENSHTLYDLDRIIELNGHKPPLTYKRFQAIISRMELPKKPVSTVMSQQMETCKVDIQENHDDVYGVPSLEEL
GFPTDGLAPAVWQGGETEALARLDKHLERKAWVANYEKPRMNANSLLASPTGLSPYLRFGCLSCRLFYYRLWE
LYKKVKRNSTPPLSLYGQLLWREFFYTAATNNPKFDRMEGNPICIQIPWDRNPEALAKWAEGKTGFPWIDAIM
TQLRQEGWIHHLARHAVACFLTRGDLWISWESGVRVFDELLLDADFSVNAGSWMWLSCSAFFQQFFHCYCPVG
FGRRTDPSGDYVKRYLPKLKGFPSRYIYEPWNAPESVQKAAKCIIGVDYPKPMVNHAETSRLNIERMKQIYQQ
LSRYRGLCLLASVPSCVEDLSGPVTDSASGQGCSTSTAMRLSQADQASPKRKHEEAEELCTEELYKRAKVTGL
PAAEIPGKSL
>Hemiprocne_coma__NXG58024.1
MGVNAVHWFRKGLRLHDNPALRECIQGADTVRCNGNLFLEAAFSDGSSVVLVIRWKKFLLQCLEDLDANLRKL
NSRLFVIRGQPADVFPRLFKEWNIAKLSIEYDSEPFGKERDAAIKKLASEAGVEVIVRISHTLYDLDKIIELN
GGQPPLTYKRFQTLISRMEPLEMPVETITPEVMEKCTTPVSDDHDEKYGVPSLEELGFDTDGLPSAVWPGGET
EALTRLERHLERKAWVANFERPRMNANSLLASPTGLSPYLRFGCLSCRLFYFKLTDLYKKVKKNSSPPLSLYG
QLLWREFFYTAATNNPRFDKMEGNPICVQIPWDKNPEALAKWAEGRTGFPWIDAIMTQLRQEGWIHHLARHAV
ACFLTRGDLWISWEEGMKVFEELLLDADWSVNAGSWMWLSCSSFFQQFFHCYCPVGFGRRTDPNGDYIRRYLP
VLRGFPAKYIYDPWNAPESIQKAAKCVIGVNYPKPMVNHAEASRLNIERMKQIYQQLSRYRGLGLLATVPSNP
NGNGNGGLMGYSPGESVSGCGSTGGHLFSSSPGAQLGTGDGHTVVQPCALGDSHTGASGIQQQGYCQASSILH
YAHGDNQQSHLLQAGRTTLGPGISAGKRPNPEEETQSIGPKVQRQSTS
>Hemiprocne_coma__NXG60909.1
DNWAFLYAQRLALKQELPLHVCFCLVPKFLDATIRHYGFMLRGLQEVAEECAELNIPFHLLLGYAKDVLPTFV
VEHGVGGLVTDFCPLRLPRQWVEDVRERLPEDVPFAQVDAHNIVPCWVASPKQEYSARTIRGKIHAQLPEFLT
EFPPVIRHPYPASCLAEPIAWEACYSSLQVDRTVKEVEWATPGTAAGLAVLQSFIKERLKSFGSHRNDPNKAA
LSNLSPWFHFGQVSTQRAILEVQKHRGKYKESVDAFVEEAVVRRELAENFCYYNENYDSLQGSANDWAQTTLN
LHAKDKRPFLYKLQELEQGTTHDPLWNAAQLQMVQEGKMHGFLRMYWAKKILEWTRSPEEALQFAIYLNDRYE
LDGRDPNGYRANTPACPAGCLWSICGIHDQGWAERAIFGKIRYMNYAGCKRKFDVGQFERRYAPCT
>Hemiprocne_coma__NXG63048.1
KVDYDVCSNYGNWLYSAGVGNDPRDNRKFNMIKQGLDYDGNGDYVRLWVPELQGIKGADIHTPWALNSAALSQ
VGVALGDTYPQPIVTAPEWSRHVSQRPQGRSPHPRGRRGPAHSPMQHKGRGIDFYFSRKKDV
>Hemiprocne_coma__NXG64473.1
MSHHTIHLFRKGLRLHDNPTLVAALESSEVIYPVYILDGKFMTSVMHIGTLRWQFLLQSLEDLQKNLCQLGSS
LLVIQGEYESVLRDHVQRWNITQVTLDAEMEPFYKEMEANIRRLGDELGFEVLSLVGHSLYDTQRILELNGGS
PPLTYKRFLHILSLLGDPEVPVRSVTAEDFQRCRPPDPGLAECYKVPLPVDLKIPLESLSPWRGGETEGLQRL
ERHLTDQGWVTSFTKPRTIPNSLLPSTTGLSPYFSLGCLSVRTFFYRLSNIYAQAKDHSLPPVSLQGQLLWRE
FFYTVASATPNFTRMAGNPICLQISWYEDAEQLHKWKTAQTGFPWIDAIMTQLRQEGWIHHLARHAVACFLTR
GDLWISWEEGMKVFEELLLDADYSINAGNWMWLSASAFFHQYTRIFCPVRFGKRTDPQGHYIRKYLPILKNFP
SKYIYEPWTASEEEQKEAGCIIGQDYPFPMVNHTEASAHNLQRMQQVREEQHRTAQLTRDDTDDPMEMKVKRD
HSEANVSRGKVARMTE
>Hemiprocne_coma__NXG65597.1
MQHSSIHWFRKGLRLHDNPALLAAATDCHRLHPLFILHPSSIRAGANARRFLLDALQDLDSSLQKIGSRLFVA
RGCPEEVFPRLFRAWGTTRXGGRGGVEHKAVSHGVEVIREVSHTLYDTERVLALNDGKAPLTYKHLQSLLASL
GPPEKPAPVLTQEHLQGCCTPCQASHDINYGIPTLEELGHDPTEVGPQLYPGGETAALARLDMFMERTAWVCS
FRKPETEPTSLHPSTTVLSPYLTLGCLSVRTFWWQLDGVYQGQQKHSQPPVSLHGQLLWREFFYTAGASIPNF
DRMVGNPVCLQVNWDDNPQHLCAWREGRTGYPFIDAIMTQLRTEGWIHHLARHAVACFLTRGDLWISWEEGLK
VFEELLIDADWSLNAGNWLWLSGSAFFHRYFHVYSPIAFGKKTDRDGAYIRKYLPILKDFPTEYIYEPWKAPQ
AVQEHARCLVGTHYPRPIVEHGAARERNLERMK
>Henneguya_sal_KAF0990424.1_HZS_876
MRRALSKLGCVPSRKKSSSHKKHGESIDESKTVEYYRSKREHCCKSPLECDFNKSRIKILNGCTEIRDLESTV
MYWMNRDQRVQDNWALIFAQRLAAKYEIPLRVCFCLDKSYMNYSFRHYDFLIKGLELVHIELSHLNIPFHVVL
GDPVTNVLDLVSQNNVSALITDFCPIDPPKRWINQLRRSLPNDVSLCLVDAHNIIPAWITSDKREYAARTIRP
KIRKLLDEYLTEFPPIIKHQWNKSAIEFPAFVKDSYYDFVKENEYVDPIDWAQPGYAQGLVCLEDFIIHRLPE
FAFFRNNAASEGTSNLSPWLHFGHLSAQRAILRVMDFIDEYKKHVDVFVEEALIRRELSENYCLYTEEPSDPS

SAPTWAIETLNKHMEDKRPYIYNLDQLEKCSTHDPIWNAAQNQLKTKGKMSGYLRMYWAKKILEWSRNASAAL
ANSLYLNDKYSIDGNDCNGIVGCMWAIYGVHDRAWAERKVFGKVRFMSEVGCNRQFNVKEYIEKYGINV
>Hepatospora_eri_ORD99805.1_PHR
MEQTKKVKVSSTFKDVSMDKRVEKLNDVEDNKNVLYFMGRDKRIHENHSVILGYQISDKLKSELYLGCNLDQI
KKNDRQLKFIVEGLKSMEIDAKKYNLNFYLIDSLKMFIKENNVGVIIIDFSPLREYVKRDKEIKKICKDLNIS
LYRVDSHNLVPAKLLDVYKRTGASVKIHLNKKMSEFLVNYPKLKPHKYNKVVDVKNEYPNHNYKFSYILKGGY
EKGMEEYQSFIKNKFEKYPSERNNPDKNVLSNLSPYLNTGFISPIEIIIRTLEKYHDTEKCEIFLNEVFIWRE
TSEHFVLHEKNYDNIDGALKWAKETLLEHALDKRPKAYTKEQLRNGETQDELWNAAQHQMVREGKMHGYCRMY
WAKTLLRWFEPKEALKIAIEFNDEYQMDGNDPNGYTGIMWSICGSMDRAFKEKEFFGKIRSMKSFKCPKYLKK
YSK
>Hermetia_ill_CAD7078538.1_unnamed_pr
MYVKIETERLNFIRLNQAKLRSEEYIHLRDAIDTEGNTANIGRLIILPATYIGSPRHMHEYAQDAMTYIRHCG
RPDLFMTFTCNPKWIEIVQLLLPGQTSSDRPITARVFRQKLRSLMNYIVKQRVLGDTRCWMYSIEWQKRGLPH
AHILIWLVERIQTDQIDDIITAEIPDHEADPDLHDVVITNMIHGPCGAINTESSCMVDGNFYLTDILQISRFH
ILHAMKRSATGVTKTPSNSSQKSAKPNNQDGFSPSTESGFLKTIEESRVKTASSVLEFHFQKKRVRVLTEATE
VKESAGSILYWMSRDCRVQDNWAFLFAQKLALKNRLPLHVCFCLVPKFLDATIRHYKFMLKGLQEVCQECKDL
DINFHLLLGSGGEKIPEFVKEMEVGAVVCDFSPLRVPLKWLDDVKNNLQEDVPLCQVDAHNIVPVWVTSDKQE
YAARTIRNKINGKLDVYLTEFPPVIKHPFVDREYNDVDWPGAFNSLECNREVEEVDWAQPGYKAACRVLETFC
SSRLKLFNSKRNDPTVNALSNLAPWFHFGHISVQRCVLAVRDYKSKYPESVAAFCEEAIVRRELSDNFCFYNE
HYDSLKGLASWAEKTLSDHRKDKRTYIYTTDELENAKTHDDLWNSAQIQLVKEAKIHGFLRMYWAKKILEWTE
SPEKALQVAIYLNDKYSLDGRDPNGYVGCMWSIGGIHDQGWREREIFGKIRYMNYDGCKRKFDVPAFVARYGG
KVYSKKK
>Hermetia_ill_XP_037903861.1_LOW_QUALIT
MSTTNAGKQTIVHWFRKGQRIHDNPPLKKACALVNANPEKYVLRPIFILDPGIPRWMTVGANRFRFLQESLVQ
FNDNLKAINSRLYVVRGTPSDVFPRLFEEWRVKVLTFESDIEPYAKKRDAEVRKLADECDVEVIVENSHTIYD
PYKVLMLNSGRPIINYRSFQAVIAKLPAAEPVPTPDPIKCSPPLKDFIEEKNGVCYDVPSLEQIGVDVNSLGP
SKFPGGETEALSRMEHHLKNTEWICSFEEPNTFPNSLEPSTTVLSPYLKFGCLSSRLFYKXLTEILAKNKKHS
KPPVSLVGQLLWREFYYSAAAGEPNFDKMEGNRICYQIPWTSNPEYLEAWTYGRTGYPFIDAIMRQLRQEGWI
HHLARHAVACFLTRGDLWISWEEGQKVFEELLLDADWALNAGNWMWLSASAFFYQYFRVYSPVVFGKKTDKDG
KYIRKYVPELKNYPASLIYEPWKATVAQQKLYGCVVGKDYPKRIVVHEEVYKKNLEKMSKAYKVNKELLAKGK
EETGYGVSSKASGVKRKYEEDEDEC
>Hermetia_ill_XP_037923019.1_cry1-like_
MSAPQVERTNPNIEYNNVRPINGERHTVHWFRKGLRLHDNPALREGLIGATTFRCVFVIDPWFAGSSNVGINK
WRFLLQCLEDLDRSLRQLNSRLFVIRGQPADALPKLFKEWGTTCLTFEEDPEPFGKVRDKNVIEMCRELKIEV
IVAVSHTLYKLDTIIERNAGRAPLTYHQFQAIIAEMKPPAKPEEAINSENIGNVYTPMSEDHDDKYNVPTLYE
LGFDTEGLKPPVWVGGETEALARLERHLERKAWVASFGRPKMTPQSLLASQTGLSPYLRFGCLSTRLFYHQLS
GKFQNLYKKIKKGNPPLSLHGQLLWREFFYCAATRNPNFDKMEGNPICVQIPWDKNCEALAKWANGQTGFPWI
DAIMTQLREEGWIHHLARHAVACFLTRGDLWISWEEGMKVFEELLLDADWSVNAGMWMWLSCSSFFQQFFHCY
CPVRFGRKADPNGDYIRRYLPVLKNFPTKYIHEPWNAPESVQRAAKCIIGKEYSLPMVNHAVASRTNMERLKQ
VYQQIAKYRPQDDESSMKGGSAIPGMLAAARAQCGTTMNIVSSPSPTTVITNINNSENNYICQQQQQAPNSGH
VNLVTNNHQQTARSRYIITKREYDFSKQESLTVTSTSGYIQHPEQPPHQKSGYDKSILGENFQYNENIKNQQE
RNNQHYNRMTISDLNNEQNIKSEGLTADVLTYRKQYINHSVVVHYGYNSNECTNELSYDNYRNEYPGSNHDIT
LQIYHQQQQHHQQQQLQQQHQMQHQPQHDSLNNLSNVHQQLQQQMKTEANEIKAPNFKCDLDENSK
>Hermetia_ill_XP_037923127.1_cry1
MYNNSCDGNTVGPSVLWFRHGLRLHDNPALLAALENKHKGSQFLPIFIFDSESAGTKLIGYNRMRFLLTALAD
LDQQFKAQGGQLYFLQGKPSEIFRRLWEECAISTICYEQDCEPIWRERDSSVENLCHDIGVNCIEKISHTLWN
PQAVIQANGGIPPLTYEMFLHTVETLGLPSRPVPNPNWNEVKFFKLPEKMLKEFQAFSKPPNPEDFDVYFENC
DSDVTEAWIGGETKALENLRYRLKVEENAFQRGFYLPNHTHPDILGPSRSLSPHLRYGCLSIRRFYWDLHDLF
EEVKRMNGAHSPSGPHITGQLIWREYFYTMSVNNINYAQMKNNDICLNIPWAVPKGDEFERWKAGKTGFPLID
AAMRQLLSEGWLHHILRNTVASFLTRGALWLSWELGLQHFLKYLIDADWSVCAGNWMWVSSSAFEKLLDSSLC
TCPLSLARRLDPNGDYIKRYIPELRCLPKEYIHEPWNTPLEVQEACGCVIGVNYPEPMLDLQTASMKNAQAMR
FLRDTLIAGGAPKEGPPHCRPSNEHEIRQFFWLVE
>Hermissenda_cra_AWY10932.1_non-photor
MKESLGQDRLGHKNKKHVLFWFRKGLRLHDNPALYSALVEGGVASTYRCVYILDPWFAGASQVGINKWRFLLE
SLEDLDNSLRKLNNRLFVARGQPADILPRLFQEWGITTLAFEEDSEPFGKERDVALSTMAREFHIQVISKSSH
TLYNPKTVIAANGNSPPLTYKRFQSILSSLDPPAQPCETVSGQVVKDSVTTISDDHDDRFSVPTLDELGFDTD
TLGPAKFQGGESEALARLYRHLERKAWVASFERPKMSPQSLYPSGTGLSPYLRFGCLSPRTFYWKLTELYKKV
KKGIDPPLALHGQLLWREFFYTVSTNNSKFDRMMGNSICVQIPWEHNPEALAKWAEGMTGYPWIDAIMVQLRK
EGWIHHLARHAVACFLTRGDLWISWEEGMKVFDEMLLDADWSVNAGMWMWLSCSAFFQQFFHCYCPVGFGKRA

DPSGDFVRQYLPVLKNMPTQYIYEPWTAPESVQKAAKCVVGKDYPLPMVAHAEQSHINLERMRQVYKRLVLKS
TASKRIFLDLPKIPKVGMFLSKAKMFLSSSSDEEGSSKPRRRKQIDSTAA
>Hermissenda_cra_AWY10935.1_photorecep
MGSTDNENRNVVTVHWFRHGLRLHDNPALLEGLENCQEFYPIFIFDGSVAGTKTAGYCRMQFLLESLKDLDDS
LRAKGSRLYVMEGDPLQIFPNLFEEWKVNMLTFEQDPEPIWQERDDNVKDLCDSYNVEWIERVSHTLWDPHSI
LKANGGSPPLTYAMFCQVAEIVGQPPRPVEDLDFSNVILPVSDNHDRLFGLPTLEKLEVVADCDAQKSPFCRY
LGGEAKALVLLESRLDKERKTFSIGQAQANQMYPDLTGMPMSLSPHLRFGSLSIRKFYWALRDAFAEIHPESE
IPLQVTSQLVWREYFYCMSVNNPFYNRMAENPICLNIDWYTDDTSLEKWTEGKTGYPFIDACMRQLKQEGWIH
QVCRHAVSCFLTRGDLWIDWQKGLEVFDRYLLDADWSVCAGNWMWVSSSAFEKVLQCSRCICPVRYGRRMDPN
GDYIRRYVPELKNIPLTYLYEPWKAPIAVQEGANCIIGTDYPPPIVDHRAASRDCKQKMEIIKAKYKDTPHVA
PANEDEVIALIWMTKDKEENRYSCDHSEERCNADAI
>Hermissenda_cra_AWY11209.1_6-4_photol
MAQHRSIHWFRKGLRLHDNPGFLKACEQASEIYPVFILDPWFLKSGHCRVGSNRWRFLFQALEDLDNSLKSLN
LRLFVVRGNPCRQLEILCKKWKISRVTFEVDTEPYAVTRDEDIERRLTELGIEVHKCVSHTLFDTAKVVKANG
GSPPLTYQRFQTVLSK
>Hermissenda_cra_AWY11212.1_cry_dash
MASTSSRSAAIVLYRNDLRIHDNEALFLANKNHSQVLPVYCFDPRHFQGTYHYNFPKTGRHRAKFLLESVADL
KSSLQSRGSDLVVRLGKPEDVIPELITNLKLGKDTLVLLQKEVTDEEVQVEKALQSSVGVTVSTVWGHTLHHL
DDLPFQPRNLPDIYTQFRKSVEDNTPVRKCFQLPDKLKPLPSNVDPGVLPTIQKLGLSEPEPHPKSSFPFNGG
ESTALSRLHHYLWGSDKVATYKDTRNGMIGADYSTKFSSWLAHGCISPRQIYWDIKKYESERTANQSTYWVIF
ELLWRDYFRLVALKFGNKIFFSSGIKGGNVNWKKNALQFKAWQEGHTGVPYIDANMRELEATGFMSNRGRQNV
ASFLTKDLLLDWRLGAEWFESMLIDHDVCSNYGNWLYSAGIGNDPRENRKFNVVKQGLDYDADGAYVRTWVPE
LSKVKSGQVHCVWTISQMGLDAAGVSLGETYPHPVVMAPEWSRHVNRPGSSSSRGRGRGQGRGAGRHHDNSRS
GQQRGMDFYFKNSNPR
>Herpetotheres_cach__NXK06702.1
RFLLQSLEDLDNSLRKLSSRLFVVRGQPTDVFPRLFKEWGVTRLTFEYDSEPFGKERDAAIVKLAKEAGVEVV
IENSHTLYDLDRIIELNGHKPPLTYKRFQAIISRMELPKKPVSTVISQQMEACKVDIQENHDDVYGVPSLEEL
GFPTDGLAPAVWQGGETEALARLDKHLERKAWVANYERPRMNANSLLASPTGLSPYLRFGCLSCRLFYYRLWE
LYKKVKRNSTPPLSLYGQLLWREFFYTAATNNPKFDRMEGNPICIQIPWDRNPEALAKWAEGKTGFPWIDAIM
TQLRQEGWIHHLARHAVACFLTRGDLWISWESGVRVFDELLLDADFSVNAGSWMWLSCSAFFQQFFHCYCPVG
FGRRTDPSGDYVKRYLPKLKGFPSRYIYEPWNAPESVQKAAKCIIGVDYPKPMVNHAETSRLNIERMKQIYQQ
LSRYRGLCLLASVPSCVEDLSGPVTDSASGQGCSTSTAMRLSQADQASPKRKHEGAEELCAEELYKRAKVTGL
PAPEIPGKSL
>Herpetotheres_cach__NXK08078.1
KVDYDVCSNYGNWLYSAGIGNDPRDSRKFNMIKQGLDYDSNGDYVRLWVPELQGIKGADIHTPWALNSAALSQ
VGVTLGKTYPQPVVTAPEWSRYINQRAQGRSPHPRGRRGPAHTPRQHKDRGIDFYFSRKKDA
>Herpetotheres_cach__NXK08244.1
MQHSSIHWFRKGLRLHDNPALLAAATDCCRLHPLFILNPSSSQAGTNAQRFLLDALRDLDGNLQEVGSRLFVV
QGCPEEVFPRLFHAWGTTRLTFEVDTEPSARQRDATVAKLAAQHGVEVIQEVSHTLYDTQRVLALNNSKAPLT
YKHLQSLLATLGPPEKPAPTLTQEHLQGEHGVDLGWVEHEAASASPRSPLSGVMAVTPPLTHHPNFGAASSPS
QNMTPVCPPIQAWVCSFKKPRTEPTSLSPSTTVLSPYLKFGCLSVRTFWWRLDQVYQGQREHSQPPISLHGQL
LWREFFYTAGTSIPNFDRMVGNPVCLQVNWDNNPQHLHAWKEGQTGYPFIDAIMTQLRTEGWIHHLARHAVAC
FLTRGDLWVSWEEGLKVFEELLLDADWSLNAGNWLWLSGSAFFHRYFHVYSPITFGKKTDRDGAYIRKYIPIL
KDFPAEYIYEPWKAPRAVQEQAGCLVGTHYPWPIVEHGAASKRNLGRMKLAR
>Herpetotheres_cach__NXK08557.1
MGVNAVHWFRKGLRLHDNPALRECIRGADTVRCVYILDPWFAGSSNVGINRWRFLLQCLEDLDANLRKLNSRL
FVIRGQPADVFPRLFKEWNIAKLSIEYDSEPFGKERDAAIKKLASEAGVEVIVRISHTLYDLDKIIELNGGQP
PLTYKRFQTLISRMEPLEMPVETITPEVMEKCTTPVSDDHDEKYGVPSLEELGFDTDGLPSAVWPGGETEALT
RLERHLERKAWVANFERPRMNANSLLASPTGLSPYLRFGCLSCRLFYFKLTDLYKKVKKNSSPPLSLYGQLLW
REFFYTAATNNPRFDKMEGNPICVQIPWDKNPEALAKWAEGRTGFPWIDAIMTQLRQEGWIHHLARHAVACFL
TRGDLWISWEEGMKVFEELLLDADWSVNAGSWMWLSCSSFFQQFFHCYCPVGFGRRTDPNGDYIRRYLPVLRG
FPAKYIYDPWNAPESIQKAAKCIIGVNYPKPMVNHAEASRLNIERMKQIYQQLSRYRGLGLLATVPSNPNGNG
NGGLMGYSPGESISGCGGTGGQSKSFRAQLGTGDGHTVVQPCALGDTHTGASGIQQQGYCQASSILHYAHGDN
QQSHLLQAGRTALGTGISTGKRPNPEEETQSVGPKVQRQSTN
>Herpetotheres_cach__NXK11489.1
DNWALLYAQRLALKQELPLHVCFCLVPKFLDATIRHYGFMLRGLQEVAEECAELNIPFHLLLGYAKDVLPVFV
VEHGVGGLVTDFCPLRLSRQWVQDVRERLPEDVPFAQVDAHNIVPCWVASSKQEYSARTIRGKIHAQLPEFLT
EFPPVIRHPYPPSCPAEPIAWEACYSSLQVDRTVKEVEWATPGTTAGLAVLQSFITERLKSFSSHRNDPNKAA
LSNLSPWFHFGQVSTQRAILEVQKHRDKYKESVDAFVEEAVVRRELAENFCYYNENYDSVQGAYDWAQTTLKL

HAKDKRPFLYKLQELEQGTTHDPLWNAAQLQMVQEGKMHGFLRMYWAKKILEWTRSPEEALRFAIYLNDRYEL
DGRDPNGYVGKLQDGYMGWGGCLWSICGIHDQGWAERAIFGKIRYMNYAGCKRKFDVSQFERRYAPPT
>Herpetotheres_cach__NXK11544.1
MLHRTIHLFRKGLRLHDNPALLAALESSEAVYPVYILDRKFMTSVMHIGALRWHFLLQSLEDLQKNLCELGSC
LLVIQGEYESVLRDHVQKWNITQVSLDAEMEPFYKEMEANIRRLGEELGFEVLSLVGHSLYDTKRILDLNGGT
PPLTYKRFLHILSLLGDPEVPVRNLTAEDFQRCRAPDLGLAECYRVPLPVDLKIPPESLSPWKGGETKGLQRL
EQHLTDQGWVASFTKPRTIPNSLLPSTTGLSPYFSMGCLSVRTFFYRLSNIYAQAKHHSLPPVSLQGQLLWRE
FFYTVASATPNFTKMAGNPICLQISWYEDAERLHKWKTAQTGFPWIDAIMTQLHQEGWIHHLARHAVACFLTR
GDLWISWEEGMKVFEELLLDADYSINAGNWMWLSASAFFHQYTRIFCPIRFGKRTDPEGQYIRKYLPVLKNFP
SKYIYEPWMASEEEQKQAGCIIGRDYPFPMVNHKEASDHNLQLMKQVREEQYRTAQLTRDDTDDPMEIKVKCD
HSEENVSKRKVARMTE
>Heterocephalus_glab__XP_004844852.1
MAVNAVHWFRKGLRLHDNPALKECIRGADTIRCVYILDPWFAGSSNVGINRWRFLLQCLEDLDANLRKLNSRL
FVIRGQPADVFPRLFKEWNITKLSIEYDSEPFGKERDAAIKKLATEAGVEVIVRISHTLYDLDKIIELNGGQP
PLTYKRFQTLISKMEPLEIPVETITSEVIEKCVTPLSDDHDEKYGVPSLEELGFDTDGLPSAVWPGGETEALT
RLERHLERKAWVANFERPRMNANSLLASPTGLSPYLRFGCLSCRLFYFKLTDLYKKVKKNSSPPLSLYGQLLW
REFFYTAATNNPRFDKMEGNPICVQIPWDKNPEALAKWAEGRTGFPWIDAIMTQLRQEGWIHHLARHAVACFL
TRGDLWVSWEEGMKVFEELLLDADWSINAGSWMWLSCSSFFQQFFHCYCPVGFGRRTDPNGDYIRRYLPVLRG
FPAKYIYDPWNAPEGIQKVAKCLIGVNYPKPMVNHAEASRLNIERMKQIYQQLSRYRGLGLLASVPSNPNGNG
GLMGYAPGESIPGSSGSGSCAHGSGILPCAHTDGQQAHLLKPGRNCVGPVLSSGKRPSQEEDAQSIGPKLQRQ
STD
>Heterocephalus_glab__XP_004852021.1
MAAAVGTGTGAAPTPATGAEGACSVHWFRKGLRLHDNPALLAAVRGARCVRCVYILDPWFAASSSVGINRWRF
LLQSLEDLDTSLRKLNSRLFVVRGQPADVFPRLFKEWGVTRLTFEYDSEPFGKERDAAIMKMAKEAGVEVVTE
NSHTLYDLDRIIELNGQKPPLTYKRFQAIISRMELPKKPVGAVSSQQMKSCRAEIQENHDDTYGVPSLEELGF
PTEGLGPAVWQGGETEALARLDKHLERKAWVANYERPRMNANSLLASPTGLSPYLRFGCLSCRLFYYRLWDLY
KKVKRNSTPPLSLFGQLLWREFFYTAATNNPRFDRMEGNPICIQIPWDRNPEALAKWAEGKTGFPWIDAIMTQ
LRQEGWIHHLARHAVACFLTRGDLWVSWESGVRVFDELLLDADFSVNAGSWMWLSCSAFFQQFFHCYCPVGFG
RRTDPSGDYIRRYLPKLKGFPSRYIYEPWNAPESVQKAAKCIIGVDYPRPIVNHAETSRLNIERMKQIYQQLS
RYRGLCLLASVPSCVEDLSHPVAEPTLSQAGSSSSTGPRSLPDGPASPKRKLEAAEEPPGEELSKRARVAELP
APEPTSKDA
>Himantopus_hima__NXN64272.1
KVDYDVCSNYGNWLYSAGIGNDPRDNRKFNMIKQGLDYDGSGDYVRLWVPELQGIKGADVHTPWALSSAALFQ
AGVTLGETYPQPVVTAPEWSRHINQRPQGRSPHPRGRSGPAHTPMQHRDRGIDFYFSRKKDV
>Himantopus_hima__NXN65466.1
RFLLQSLEDLDNSLRKLNSRLFVVRGQPTDVFPRLFKEWGVTRLTFEYDSEPFGKERDAAIIKLAKEAGVEVV
IENSHTLYDLDRIIELNGHKPPLTYKRFQAIISRMELPKKPVSTVMSQQMETCKVDIQENHDDVYGVPSLEEL
GFPTDGLAPAVWQGGETEALARLDKHLERKAWVANYERPRMNANSLLASPTGLSPYLRFGCLSCRLFYYRLWE
LYKKVKRNSTPPLSLYGQLLWREFFYTAATNNPKFDRMEGNPICIQIPWDRNPEALAKWAEGKTGFPWIDAIM
TQLRQEGWIHHLARHAVACFLTRGDLWISWESGVRVFDELLLDADFSVNAGSWMWLSCSAFFQQFFHCYCPVG
FGRRTDPSGDYVKRYLPKLKGFPSRYIYEPWNAPESVQKAAKCIIGVDYPKPMVNHAETSRLNIERMKQIYQQ
LSRYRGLCLLASVPSCVEDLSGPVADSASGQGCSTSTAMRLSQADQASPKRKHEGAEELCTEELYKRAKVTGL
PAPEIPGKSL
>Himantopus_hima__NXN66078.1
MPHRTIHLFRKGLRLHDNPTLLAALESSEAIYPVYVLDRKFMTSVMHLGVLRWHFLLQSLEDLQKNLCQLGSC
LLVIQGEYESVLRDHVQKWNITQVTLDAEMEPFYKEMEANIRRLGEELGFEVLSLVGHSLYDTKRILDLNGGT
PPLTYKRFLHILSLLGDPEVPVRNLTAEDFQRCRPPDPGLAECYRVPLPVDLEIPLESLSPWRGGETEGLRRL
EQHLTDQGWVASFTKPRTIPNSLLPSTTGLSPYFSMGCLSVRTFFYRLSNIYAQAKHHSLPPVSLQGQLLWRE
FFYTVASATPNFTKMAGNPICLQISWYEDAERLHKWKTAQTGFPWIDAIMTQLRQEGWIHHLARHAVACFLTR
GDLWISWEEGMKVFEELLLDADYSINAGNWMWLSASAFFHQYTRIFCPVRFGKRTDPEGQYIRKYLPILKNFP
SKYIYEPWTASEEEQKQAGCIIGRDYPFPMVNHKEASDRNLQLMKQVREEQYGTAQLTRDDTDDPMQMKVKRH
HSEENVSKGKVARMTE
>Himantopus_hima__NXN67465.1
RFLLQCLEDLDANLRKLNSRLFVIRGQPADVFPRLFKEWNIAKLSIEYDSEPFGKERDAAIKKLASEAGVEVI
VRISHTLYDLDKIIELNGGQPPLTYKRFQTLISRMEPLEMPVETITPEVMEKCTTPVSDDHDEKYGVPSLEEL
GFDTDGLPSAVWPGGETEALTRLERHLERKAWVANFERPRMNANSLLASPTGLSPYLRFGCLSCRLFYFKLTD
LYKKVKKNSSPPLSLYGQLLWREFFYTAATNNPRFDKMEGNPICVQIPWDKNPEALAKWAEGRTGFPWIDAIM
TQLRQEGWIHHLARHAVACFLTRGDLWISWEEGMKVFEELLLDADWSVNAGSWMWLSCSSFFQQFFHCYCPVG
FGRRTDPNGDYIRRYLPVLRGFPAKYIYDPWNAPESVQKAAKCIIGVNYPKPMVNHAEASRLNIERMKQIYQQ

LSRYRGLGLLATVPSNPNGNGNGGLMGYSPGESISGCGSTGGHLFPASLGAQLGTGDGHTVVQPCALGETHTG
ASGIQQQGYCQASSILHYAHGDNQQSHLLQAGRTALGTGISAGKRPNPEEETQSVGPKVQRQSTN
>Himantopus_hima__NXN68464.1
DNWAFLYAQRLALKQEMPLHICFCLVPKFLDATIRHYSFMLRGLQEVAEECAELNIPFHLLLGYAKDVLPAFV
VEHGVGGLVTDFCPLRLPRQWVEDVRERLPEDVPFAQVDAHNIVPCWVASPKQEYSARTIRGKIHAQLPEFLT
EFPPVIRHPYPPSCPAEPIAWEACYSSLQVDRTVKEVEWATPGTAAGLAVLQSFIAERLKSFASHRNDPNKAA
LSNLSPWFHFGQVSTQRAILEVQKHRGKYKESVDAFVEEAVVRRELAENFCYYNENYDSVQGAYDWAQTTLKL
HAKDKRPFLYELQELEQGTTHDPLWNAAQLQMVQEGKMHGFLRMYWAKKILEWTRSPEEALRFAIYLNDRYEL
DGRDPNGYVGKLQDGGMGRGGCLWSICGIHDQGWAERAIFGKIRYMNYAGCKRKFDVGQFERRYGPRT
>Himantopus_hima__NXN69851.1
MQHSSIHWFRKGLRLHDNPALLAAATDCCRLHPLFILDPSSNRAGANARRFLLDALRDLDGSLREMGSRLFVV
RGCPEEVFPRLFCAWGTTRLTFEVDTEPPARQRDATVAELAARHGVEVIQKVSHTLYDTERVLALNDGKAPLT
YKRLQSLLASLGPPEKPAPALTQEHLQGCRTPCQVSHDTDYTVPTLEELGQDPTELGPHLYPGGETAALARLD
AHMERTAWVCGFKKPETEPTSLSPSTTVLSPYLKFGCLSVRTFWWRLDQVYQRQREHSQPPVSLHGQLLWREF
FYTAGASIPNFDRMVGNPVCLQVDWDDNPQHLRAWREGRTGYPFIDAIMTQLHSEGWIHHLARHAVACFLTRG
DLWVSWEEGLKVFEELLLDADWSLNAGNWLWLSGSAFFHRYFHVYSPVAFGKKTDWDGAYIRKYLPVLKDFPT
EYIYEPWKAPRAVQERAGCLVGTHYPRPIVEHRVVSERNLGRMKAARAQK
>Hippocampus_come__XP_019722428.1
MSASRTIICLLRNDLRIHDNEVFHFAQKHADHIVPLYCFDPRHYAGTYKYNLPKTGPFRLRFLLESLRDLRNT
LVKRGSNLVVRQGKPEEVVADLIQQLGSVSVVAFHQEVTSEELSVEKGVKDVCARMKVQVHTCWGSTLYHRDD
LPFQHISRLPDVYTQFRKEVESKSQVRPELPTSDRLKPLPGGLQEGDIPTPADLKQTEPLNDPRSAFPCGGGE
SQALRRLQHYFWDTDAVAAYKETRNGLVGLDYSTKFSPWLAVGCISPRYIFHQIRRYERERTANQSTYWVIFE
LLWRDYFKFVALKYGNRLFQVKGLQDKTIPWKTDVTLFNAWKEGRTGVPFVDANMRELAMTGFMSNRGRQNVA
SFLTKDLGLDWRMGAEYFEYLLIDHDVCSNYGNWLYSAGIGNDPRENRKFNMVKQALDYDSNGDYVRLWLPEL
QKIRGADVHTPWTLSKAALSHADVVLGDAYPIPVVVAPEWSRHIGKKQNDTGPYPRGRKGPSHNPKQHRDRGV
DFYFSRSKNL
>Hippocampus_come__XP_019722745.1
MAPNSIHWFRKGLRLHDNPALKEAVQGAGTVRCVYFLDPWFAGSSNVGVNRWRFLLQCLEDLDANLRKLNSRL
FVIRGQPANVFPRLFKEWKISRLTFEYDSEPFGKDRDAAIKKLAMEAGVEVIVKISHTLYDLDKIIELNSGQP
PLTYKRFQTLISRLDPPEMPVETLSDSLMVNCITPILEDHGDKYGVPSLEELGFDIEGLPTAVWPGGETEALT
RIERHLERKAWVANFERPRMNANSLLASPTGLSPYLRFGCLSCRLFYFKLTDLYRKVKKNSSPPLSLYGQLLW
REFFYTAATNNPRFDKMEGNPICVRIPWDKNPEALAKWAEAKTGFPWIDAIMTQLRQEGWIHHLARHAVACFL
TRGDLWISWEEGMKVFEELLLDADWSVNAGSWMWLSCSSFFQQFFHCYCPVGFGRRTDPNGDFIRRYVPVLRA
FPAKFIYDPWNAPESVQVAAKCIIGVHYPKPMVHHAEASRLNIERMKQIYQQLSRYRGLGLLASVPSTNGNGN
GGMMAYSPGEQQPGTNSSNNNNTPHSYHAVPENNAAVTNSRPYHEFAVPQHPGLAHIRGGITGKREREMEREC
SGEEDPSSCSGHKMQRHSAETT
>Hippocampus_come__XP_019733281.1
MRHCVTRTSFFSFPTKSLAAIMSDKKRKATKAAAEPSTKQQKLASTKEVKKEKAAGWLQDLVKQHRSDNKDMK
VNKKRVRFISDMQEAKEESEGVLYWMLRDQRVQDNWALIHAQRLAMKKKLPLHICACLFVPKSELSTLRHYSF
MLKGLEEVEKECRSLDIQFHLLHGSAGKVLPGFVSERGFGAVVTDFSPLREPLKWLDDVKKALPNDVPLIQVD
AHNIVPCWVASPKLEYAARTIRSKITKLLPEFLTDFPVVEKHPYTSTKNAKPVDWAKTIASLQVDRTVGEPEW
AQPGTTSGMVMLESFIDVRLKLFGTQRNDPNVAALSQLSPWIRFGHLSAQRVALQVQRGGKNASQSVASFIEE
LVVRRELTDNFCFYNDKYDSVEGAYEWAQKSLRDHAKDKRPYVYTREQLERAKTHDKLWNAAQYQMVSEGKMH
GFLRMYWAKKILEWTCSPEEALSIALYLNDRYELDGQDPNGFVGCMWSICGIHDQGWAERDVFGKIRYMNYKG
CLRKFDVPKFEKKYCPKGL
>Hippocampus_come__XP_019733726.1
MVVNSVHWFRKCLRLHDNPALQEALNGANTVRCVYILDPWFAGAANVGINRWRFLLEALEDLDCSLKKLNSRL
FVVRGQPAEVFPRLFKEWKMTRLTFEYDPEPYGKERDSAIIKMAQEFGVETIVRNSHTLYNLDRIIEMNNNSP
PLTFKRFQTIVSRLELPRRPLPSINQKQMDKCHTKITDNHDQIYSIPSLEELGFRTEGLPPAVWRGGESEALD
RLSKHLDKKVWVANLEHPRGNMCSLYASPTGLSPYLRFGCLSCRVLYYNLRELYMKLRKRCSPPLSLFGQLLW
REFFYTAATNNPNFDRMDGNPICVQIPWDQNPEALAKWAEGRTGYPWIDAIMTQLREEGWIHHLARHAVACFL
TRGDLWISWESGMKVFEELLLDADWSVNAGSWMWLSCSAFFQQFFHCYCPVGFGRRTNPTGDYIRRYIPILKD
YPNRYIYEPWNAPESVQKAANCVVGVDYPKPMINHAESSRLNIERMKQVYQQLSHYRGLSLLASVPTIQEEAE
PLMTDESQTSSGPDSPSVVHDKATGCVAAPDSSTVCTSSHTVLNPELESDEMRCLSQTPVNCSDSQMHPSATI
TSTCADHSAGVESPSSLVPPPAQSPLARSQTCTPSLVCSTLPPSPSPATAQTQTSSFGPRRKSFTRKVRRGQR
QRGRHSCHAVAREGERRGEEEETEPGEENEKMEESVEMEEERMEEETYAGH
>Hippocampus_come__XP_019743950.1
MPQTGEEDGELAVVRRLLREVLLGREDPEGFFAMCLSILGHQETRARFLPAVQPLSSANRFLHSTLVSIYEEY
FTKAEDDELELAVTLSLLETQVHPRTRLQKYDGVSNQSGHSKEGGGTTPEPAHQQETFLNMSGTCQTVSAKQD
MGSKSDMMEDQCEKTNHSKSRRQRRKAAAQHVVGLPDSPSGAAPVLLWVRRDLRLCDNPALVGSLELGAPVIP

VFIWSPKEEEGPGMTVAMGGACKYWLHQALSSFRSSLERIGSHLVFLEVNGSSTLRTLKELVRETGARTVLAN
ALYEPWLKERDDEVVSRLQKDGVRCKMFHSYCTRDPYSVSTEGVGLRGIGSVSHFMSCCRQNPAGTAFGAPLD
PPMSLPVPSSWPQGIPLDTLGLARMPRRKDGTTIDWAENICKSWDFSEEGAHARLEAFLHDGVYRYDKESGRA
DAPNTSTLSPYLHFGQLSPRWLLWDAKGARCRPAKFQRKLAWRDLAYWQLTLFPDLPWESLRPPYKELRWSSN
RNHLKAWQRGRTGYPLVDAAMRQLWLTGWMNNYTRHVVASFLIAYLHLPWQEGYRWFQDTLVDADVAIDAMMW
QNGGMCGLDHWNFVMHPVDAAMTCDPNGSYVRKWCPEIAELPDELIHKPWKCPTSMLRRAGVVLGQTYPERIV
TNLEERRSRSLQDVALVRRQHAEYVDTRSGCDLVPLPPRLVSEALGLAQPTGGKQFLLPVITRMEFKHQMDDP
DMDAATNPYNAVLKGYVSRKRDETVAFLNERDFTASVLHEGAQRRERLESDHRRMEGLPPKRLPTQGRARRST
NTNDRFSILPSGTVLTTHR
>Hippoglossus_sten__KAF5157511.1
MSRTVVCLLRNDLRLHDNELFHWAQRNAENIVPLYCFDPRHYVGTHNYNLPKTGPFRLRFLLQSIKDLRNTLL
NKGSNLVVRRGKPEDVVAELIKQLGCVSTVAFHEEVTSEELNVEKKVKDVCAQMKVTVHTCWGSTLHHRDDLP
FHQMSRLPDVYTEFRKAVEARSRVRPVFPAPEQLKPLPPGLDEGAIPTAQDLQQTEPLTDPRSAFPCSGGESQ
ALARLKHYFWDTDAVATYKETRNGLIGVDYSTKFSPWLAIGCISPRYIYHQIKQYERERTANQSTYWVIFELL
WRDYFKFVGVKYGNRLFQVKGLQDKSVPWKKDMKLFNAWKEGHTGVPFVDANMRELAMTGFMSNRGRQNVASF
LTKDLGLDWRMGAEWFEYLLIDHDVCSNYGNWLYSAGIGNDPRENRKFNMIKQGLDYDNNGDYVRQWVPELQG
IRGSDVHAPWTLSPAALSHAHVTLDDTYPTPIVMAPEWSRHVNKKSGATGPSPEERKARLTPPNSIRTEA
>Hippoglossus_sten__KAF5168753.1
GDSPENLRLHTDLLTCTQPRNASVSGPDIDSSSGRPLQNKMAPNSIHWFRKGLRLHDNPALQEAVRGADTVRC
VYFLDPWFAGSSNVGVNRWRFLLQCLEDLDANLRKLNSRLFVIRGQPANVFPRLFKEWKISRLTFEYDSEPFG
KERDAAIKKLAMEAGVEVNVKISHTLYDLDKIIELNGGQPPLTYKRFQTLISRLDPPETPAETLSEMLMGGCV
TPISDNHGDKYGVPSLEELGFDVEGLPPAVWPGGEAEALTRIERHLERKAWVANFERPRMNANSLLASPTGLS
PYLRFGCLSCRLFYFKLTDLYRKVKKNSSPPLSLYGQLLWREFFYTTATNNPRFDKMEGNPICVRIPWDKNPE
ALAKWAEAKTGFPWIDAIMTQLRQEGWIHHLARHAVACFLTRGDLWISWEEGMKVFEELLLDADCSVNAGSWM
WLSCSSFFQQFFHCYCPVGFGRRTDPNGDFIRRYLPVLRGFPAKFIYDPWNAPESVQAAAKCIIGIHYPKPMV
HHAEASRLNIERMKQIYQQLSRYRGLGLLASVPSANGNGGMMAFSPGEQQQPGTNSNNSHCKSIFYK
>Hippoglossus_sten__XP_035011753.1
MAVNSVHWFRKGLRLHDNPALQEXLSRADTVRCVTSGPGVVRKRQRRINRWRFLLESLEDLDSSLKKLNSRLF
VIRGQPTDVFPRLFKEWKVTRLTFEYDPEPYGKERDGAIIKMAQEFGVETIVRKSHTLYNLDRIIEMNNNSPP
LTFKRFQTIVSRLELPRRPLPAVTQQQMDKCRTKITENHDQLYSIPSLEELGFRTVGLPSAVWRGGESEALDR
LNKHLDKKVWVANLDHPRVNTCSLYASPTGLSPYLRFGCLSCRVLYYNLRELYMKLRKRCSPPLSLFGQLLWR
EFFYTAATNNPNFDRMDGNPICVQIPWDQNPEALAKWAEGRTGFPWIDAIMTQLRQEGWIHHLARHAVACFLT
RGDLWISWESGMKVFEELLLDADWSVNAGSWMWLSCSAFFQQFFHCYCPVGFGKRTDPSGDYIRRYIPILKDY
PSRYIYEPWNAPESIQKAANCVVGVDYPKPMINHAEGSRLNIERMKQVYQQLSHYRGLSLLASVPTIQEEAEP
PMTDESQTSSGPDSPPRVPAPSEAAGCSTAPDSSTVYVSYAPRPELEGTQGPHPSQTPCTSSGSHPQPPAAVS
WSFAPPSPAHSPLSRSKASSPSSSCPTSSPAPSPAGAPGQTFPKRKGLARQTRRNPRQRGRPSCATSGREGER
REEEEEEREDTGGDERMEEDAEHEVERMEEETSAHQQ
>Hippoglossus_sten__XP_035012548.1
MPPSAAGAQDSKALVRQLLREVLVGREDPEGFFAMCVSVLGQQETRTQFQSLIQPLSTANRSLHCSLTSIYLE
YFSKTGDDDLEFALALSVLEMKDPQLSTCSEESTLQPLGAKANQSSSVQLTSASQPQGSSSRTQRADVGGGRK
KTQSVQKGPRGVMCLPQTTQETELQKSTHVDKCNKETPGTSQSVCVSKPYASFFKQEAVLKGDQEAVLKGDQM
TEGGDSSNQSEKPKRSRNRRKRREGGGQQVVGMPTSPSASPPVLLWFRRDLRLCDNPALIGSLENGAPVIPVF
IWTPEEEEGPGITVAMGGACKYWLHQALSCFSSSLERIGSHLVYLKANGVGNGVGSSLRTLTELIKETGARAV
LANALYEPWLKERDDAVVSALQKVGVQCRMFHSYCLRDPYSVSTEGVGLRGIGSVSHFMSCCKQNPESALGVP
LDPPAFLPTPANWPQGVSLDALELALMPRRKDGTTIDWAVNIRKSWDFSEEGAHARLEAFLQDGVYRYEKESG
RADAPNTSSLSPYLHFGQLSPRWLLWDAKGARCRPPKFQRKLAWRDLAYWQLTLFPDLPWESLRPPYKALRWS
TDRGHLKAWQRGRTGYPLVDAAMRQLWLTGWMNNYMRHVVASFLIAYLHLSWQEGYRWFQDTLLDADVAIDAM
MWQNGGMCGLDHWNFVMHPVDAAMTCDPYGSYVRRWCPELADLPDELIHKPWKCPASILRRAGVIFGQTFPDR
IVTDLEERRSQSLQDVAVVRREFVQYVDKRSGCDLVPLPPRLVSEALGLSHRNGGQVTEVKQFLLPVITRMEF
KHHQEDPDADAASNPYNAVLKGYVSRKRDETIAFLNERDFTASVLYEGAQRKERLESNYRRMEGLPQPPAPQG
RARRTPTAKDRFSLVPGGAVTSLR
>Hippoglossus_sten__XP_035020365.1
MAHTCIHWFRKGLRMHDNPALLAALRDCKQLYPVFLLDPHLHNNMCINRWRFLVGALKDLDCSLRKLNSRLFV
VRGRPEDVLPKLFDKWKVTKLTYEYDTEPYSLGRDTAVTALAKEHGVQVIYKISHTLYDIERIIEENNGKAPL
TYTRMQAIVKTLGPPKKPVPAPTMEDMAEVKTPCSESHEQQYGIPALEELCPDAAALGEEPFPGGEQEALRRL
DEHMKRTRWVCEFEKPQTSPNSLSPSTTVLSPYVTVGCLSARTFWWRLADVYQGKKHSDPPVSLHGQLLWREF
FYTASVGIPNFNKMVGNPVCTQIDWDTNSEYLAAWREARTGFPFIDAIMTQLRQEGWIHHLARHAVACFLTRG
DLWISWEDGQKVFEDLLLDGDWALNAGNWQWLSASTFFHQYFRVYSPVAFGKKTDKNGDYIKKYLPLLKKFPA
QYIYEPWKAPRSVQQAAGCIVGKDYPHPIVQHEVISKKNIQRMKSAYAKRSSDKTDSPTKKQGVKRKGPSVVD
MMKKKEKRKKSPSS

>Hippoglossus_sten__XP_035025301.1
MFCIWRSSPSAQRRPLTLTYQRSPRPLLSLFTGSFSSVSTMSDKKRKAAPASKERSAKQKKLAPTKQQQEKQQ
EKQQRTGGWLQDLIMQQRSEEKEIKFNKKRLRILSDAEKMKQGSEGVLYWMSRDQRVQDNWALIHAQKLAVKK
NLPLHVCFCWFVPKSELSTLRRYSFMLKGLKEVAEECKALDIQFHLLHGSAGDVLPGFVSDHSLGAVVTDFYP
LREPLQWLEDVKEKLPEDILLKQVDAHNIVPCWVASPKQEYAARTIRGKITKLLPDFLTDFPPVEKHPVPATR
TAKPIEWEKTLASLPVDREVEVPEWAKPGTKAGMAMLESFIDVRLKLFENKRNDPNVSALSQLSPWIRFGHIS
AQRVALQVQSSGQKAGQTVASFIEELVVRRELTDNYCYYNKKYDSVEGAYEWAQKTLKDHAKDKRPYLYTREQ
LEKAKTHDKLWNGAQYQLITEGKMHGFLRMYWAKKILEWTSSPEEALSIALYLNDRYSLDGQDPNGFVGCMWS
ICGIHDQGWAERPIFGKIRYMNYKGCTRKFDVPQFERKYCPKNL
>Homarus_ame_AWC08575.1_cry_2
MTGEASKVKAKHLVHWFRKGLRLHDNPALRAGLKNAHTFRCIFILDPWFAGSSNVGINKWRFLLQSLEDLDSS
LRKLNSRLFVIRGQPAHVLPELFREWGTTCLTFEKDPEPFGKARDANIVAIAREMGINVIVKTSHTLYKPEKI
IDKNGGKPPLTYKSFQNILMSMDLPLLPQSTITLEDLEDKSTPLSDDHDEKYGVPTLEELGFETETLHQTGWK
GGESEALTRLEHHLERKAWVASFGRPKMTPQSLYPSRTGLSPYLRFGCLSCRRFFAELNDLYRKIRKSPPPLS
LHGQLLWREFYYTAATNNPKFDHMEGNPICVQIPWDKNPEALAKWANGQTGYPWVDAIMTQLRKEGWIHNVAR
HAVACFLTRGDLWVSWEEGMKVFDELLLDADWSVNAGSWMWLSCSSFFQQFFHCYCPVRYGRKADPNGDYIRM
YLPVLKNFPTKYIHEPWTAPESVQRAAKCVIGRDYPMPMVDHIKQSQNNIERMKQVYQQLAHYRGKINPAIKS
PTVDHYPSPPPEFEKKRSSKTNKNDLQYDSNYRVQVQA
>Homarus_ame_KAG7173815.1_cry_DASH-l
MKANTNVDEVVPVYCFDPRHFQETYHFCFSKTGNHRAKFLLQSVTREEKDVERALRERCKAAGAQIHSFWGST
LYHKNDLPFAVNNIPDTFTGFRKAVEAQSRVLGPIVMPEKLKPLPSGIDLGDLPTLERLGVKDAPTDRRTAFP
FSGGETSGLERVKRYFWDSNNVAKYKETRNGLLGEEYSTKLSPWLAHGCLSPRYIFAEIKKYESARIANQNGQ
TGVPFVDANMRELKETGWMSNRGRQNVASFLIKDLGLDWRLGIGNDPRENRKFNMVKQGLDYDPKGEFIRTWV
PELAEIRGAMVHTPWKMSLHDLGKAGVSLGEYYPNPVVTSRTTKHKNAWTNQGREPKQRGMDFYFKSNNKS
>Homarus_ame_KAG7176338.1_cry2-like
MLKKMTTIHWFRKGLRLHDNPALLASVEENCELRPLFILDPWFVTNARVGSNRWRFLAQTLADLDIKLRDLGS
RLFIARGTPEELFPKLFKDWNVQKLTWEVDTEPYSCIRDEKIEKLAKDHNVKVVCRVGHTLYSTEKIIQANLG
KTPLTYQSMQSLVSRMGHPPKPVDSLSSLPGSCRINNAMAVNAKYNLPTLAELGVDEGSLQPCLYPGGETEAL
RRLDKYMMRKTWVCKFEKPKTSPNSLQPSTTVLSPYLKFGCLSPRTMYFRLLEVYKNNSHTQPPVSLLGQLLW
REFFYTVGAKTPNFNRMEGNPICRQIPWVRNEKLMKAWEFVFEELLLDADWSLNAGNWMWLSASAFFHQYFRV
YSPIAFGKHTDKHGEYIRKYLPQLSKYPDNYIYEPWKAPLSTQKAAGCIIGQDYPKPIVKARHHLQTETLQTR
R
>Homarus_ame_KAG7176479.1_cry1-like_
MIKASVHWFRHGLRLHDNPALLESIKDTEKFYAVFIHDGHTAGTNITGYNRLNFLTETLNSLDEQLREVGSQL
FVFKGNPTEIFRILHKEAGITRITCEQDCEAIWNKRDGDVRNLCQELGMDFVERISHTLWDPFKIIENNGGQP
PLTYEMFLQVAQTLGLPPRPCPDPDWSNVLFGEISDELAAKLKLCPHVPKPEELGCTKKNGECPVYVGGEHPA
LLHLADRLRVEEEAFQDGYILPNQVNPDLLGPPMSMSAALRFGCLSVRKFYWDIQDVYVKMYEGESPPSHSLT
AQLIWREYFYCMSANNPKFDQMKENPICIDIPWYDSKEKLSAWEEGRTGYPFIDACMRQLHKEGWIHHVCRTA
VACFLTRGDLWISWEAGLKVFYKYLIDADWSVSAGNWMWVSSSAFERQLDCSTCICPVNYGRRVEPTGDYIRR
YVPELASMPQEYIFEPWKAPRKLQKKVKCIIGRDYPERIVIHEEMMKEISLKLKREMPHCCPSNPDEIKKFLR
LPTTCYHNVC
>Homarus_ame_XP_042217820.1
MMSKADEVPAECDGIVYWMCRDQRVQDNWAMLFAQRLALKNKVSLHVAFCLIPKFLDATIRQYHFMLKGLEEV
ENECQKLGIEFHLLHGEAKDVLPKIIEEHHIGGLVTDFSPLRLPQEMLKDVKETIPKNIPMCKVDAHNIVPCW
LASDKQEYSAKTIRRKIQDKLPDFLTKFPPVAKHSHESKYKAKLVDWTAAEKSLEVDRTVKPVEWATPGTHAG
LDTLNQFCLKRLRKYATQRNNPNAKSLSNLSPWLHFGQISAQRCVLEVKRYRKQFKDAADDFIEEAVVRCELS
DNYCFYQKNYDCVEGAYNWAITTLNDHKKDTRQYLYSREAFIEAQTHDDLWNSAQIQLVTEGKIHGFMRMYWA
KKILEWTATPEEALETAIYLNDRYSLDGCDPNGYAGKFFT
>Homo_sapiens_NP_066940.3_cryptochro
MAATVATAAAVAPAPAPGTDSASSVHWFRKGLRLHDNPALLAAVRGARCVRCVYILDPWFAASSSVGINRWRF
LLQSLEDLDTSLRKLNSRLFVVRGQPADVFPRLFKEWGVTRLTFEYDSEPFGKERDAAIMKMAKEAGVEVVTE
NSHTLYDLDRIIELNGQKPPLTYKRFQAIISRMELPKKPVGLVTSQQMESCRAEIQENHDETYGVPSLEELGF
PTEGLGPAVWQGGETEALARLDKHLERKAWVANYERPRMNANSLLASPTGLSPYLRFGCLSCRLFYYRLWDLY
KKVKRNSTPPLSLFGQLLWREFFYTAATNNPRFDRMEGNPICIQIPWDRNPEALAKWAEGKTGFPWIDAIMTQ
LRQEGWIHHLARHAVACFLTRGDLWVSWESGVRVFDELLLDADFSVNAGSWMWLSCSAFFQQFFHCYCPVGFG
RRTDPSGDYIRRYLPKLKAFPSRYIYEPWNAPESIQKAAKCIIGVDYPRPIVNHAETSRLNIERMKQIYQQLS
RYRGLCLLASVPSCVEDLSHPVAEPSSSQAGSMSSAGPRPLPSGPASPKRKLEAAEEPPGEELSKRARVAELP
TPELPSKDA
>Homo_sap_NP_004066.1_cry-1

MGVNAVHWFRKGLRLHDNPALKECIQGADTIRCVYILDPWFAGSSNVGINRWRFLLQCLEDLDANLRKLNSRL
FVIRGQPADVFPRLFKEWNITKLSIEYDSEPFGKERDAAIKKLATEAGVEVIVRISHTLYDLDKIIELNGGQP
PLTYKRFQTLISKMEPLEIPVETITSEVIEKCTTPLSDDHDEKYGVPSLEELGFDTDGLSSAVWPGGETEALT
RLERHLERKAWVANFERPRMNANSLLASPTGLSPYLRFGCLSCRLFYFKLTDLYKKVKKNSSPPLSLYGQLLW
REFFYTAATNNPRFDKMEGNPICVQIPWDKNPEALAKWAEGRTGFPWIDAIMTQLRQEGWIHHLARHAVACFL
TRGDLWISWEEGMKVFEELLLDADWSINAGSWMWLSCSSFFQQFFHCYCPVGFGRRTDPNGDYIRRYLPVLRG
FPAKYIYDPWNAPEGIQKVAKCLIGVNYPKPMVNHAEASRLNIERMKQIYQQLSRYRGLGLLASVPSNPNGNG
GFMGYSAENIPGCSSSGSCSQGSGILHYAHGDSQQTHLLKQGRSSMGTGLSGGKRPSQEEDTQSIGPKVQRQS
TN
>Hondaea_fer_GBG27575.1_cry-1
MPSAQTKILWLRKTLRIHDNPTLKACMEDASTVLPVYVLDKWHSNPQNVGDVRFEFIMESLRNLDEKFKKCGV
DAGVIFVRGEPEKVLPKIWEAVDAKAIGFDTSEEAEKHSQKTDEDVAGLANKADVKVIDEPAHWMHPPGLYER
HMNGGEVPTTMSSFETLFSQCGSVREPLSAPKSIPYDKGTADKALDAIRKALDVEDLTVPKSGDNKFYKGLKR
EQRKLRFPGGEDEALKRLEKVVEKQPDWVASFEKPKTAPTGLEPQTTVLSPYLNAGCLSLRRAWHAVKKAEKD
AKGKPTQPPTSLAGQLLWREHWLTIGYFTEEDISKMKGNPLARQIDWDWDNDAKELFAKWEAGETGYPFIDAI
MTQLREEGWIHHLARHMVACFLTRGDCFVSWEKGFDVFDRLLIDADWSVNSCNWMWLSCSAFFYQYFRCYSPI
SFGKKWDPDGDYIRKYVPALKDMPKKYIYEPWKAPREVQEKAKCIIGKDYPERIIEDHTATSKENMNRIKEDY
DLSSSTVK
>Hondaea_fer_GBG30516.1_cry_DASH
MGGAYRPVIVWFRNDLRLTDNAVLHLPEIQRATHLVGIYCLDDRFENARTKRFKEQAVAELEERFHKRFGAPL
ECYRGKPEKIIPDVVRKLRKLGTGSQDGFVVASEEVASYEKRVETKLQEQLAPLGFSLKLAWTYSLFHPDDLP
FDVRSGDLPEPFTSFRKRIEDSQDAVVRQVLDVPTLKLGAIPLDDPNAGLDCVPGAWTPSQDGGGVGKSPDQS
ADATSHVLPFEDGPLKSGHPDHWLTFRGGESAANARLEYFVEHGLSKYKTKRNESVGWDYSTKLSPWLAAGNI
SPRQVYHRIRAYEEEHGESVHTYWCSVFEPLWRDFMRFQGIKYGNKLFFELGPAGEMPSGAKWSYGDRETAKR
FKSWQEGRTGIPWVDGHMRELVHTGFMSNRGRQNVASFLVHNLHVDWREGAQFFQDQLIDHDVTSNWCNWASA
AGVGTRGARLNRFNMDKQARDYDKLAVHARLWVPEVADVRPEAVHDLALRSRGSSMESFRSKKGQQRQEQQNK
EQEQEQQEVKESGKGAEQQISAPYPPPIVRSMGGSRFENTRRLDRAELDATAAKVRHAVENAGCQVRCAMPPE
LRDKSSFGDVDLIVATFQDKEGGNEMILKAHEQAIDAAASALCSVARKGDAVTQMSLLSEEGFQVDLEFVPSA
SFALAVAVHAHGDFCWLLGRALQPLGLKLTTHGLFVRRAVATLLEDFLLSRDPHAVANFLGVCPSFFDGETSK
TSDTLIEIEDRRACRAFINGGDVIQRHPYLAGPLVGQCLRELCISHGNGNWKAYLQWLQATPREDILNAIDAQ
VNALQRRANCDVARLSSS
>Hondaea_fer_GBG31063.1_photolyase
MKVEVQPERCQWLKKDEDAVAEGKNDGGPVVYWMHREKRAEDNWALLKAQSLALAKNASLAVVMLVPTKYMHW
NLRHYSFGLKGLAETQKALHAKNIPMYVSLSENPAKDLVSTCKDLKARALVMDFFPMRPKRNWDAEVTKSVDI
PVCLVDAHNVVPCWEASDKREYAARTIRPKINRKKDDFFDAFPELSKMPSSNTDGKLKETDFDKMLEELKSDH
KLDTSVKPVNDRFPPGPSGAKKILDRFLDAKRLGKYSECRNDPNERVLSDMSPYMNYGFIAPQRVLMEARAYA
KKNGKIPSKEIDGFEEEALVRRELTDNYCHYVPEYDSLDAAYDWARETLKEHAKDKRPHIYSFEELAEARTND
ELWNAAQVMARDEGKMHGWLRMYWAKQLLTWTETPEEALDYGFRLNDIYNLDGGDPNSIVGVAWSIMGIHDQG
WREREIFGKIRCMTYKSSRTKFDVDKLAKKYPGPGEAKSGKRKASQDKGDEDDKDSKDKDSKNKSKKGSSSNK
KQKSGKK
>Hyalella_azt_XP_018012197.1_photolyase
MASVEKNSKGLELMDIVHKKRLKFAGSIADFKFCKKRVQVRSKSLDVPESFHSIIYWMSREQRVQDNWAFLFA
QRLALKNKVPLKVVFCLVPKFMDATLRHYSFMIQGLKEVEFDCKQLGIPFHLLMGQAKDVLPAFVTKNNVGGV
VTDYSPLRTPRQWVADVLNKLPKDIPLCQVDGHNVVPVWEASPKQEYAARTIRGKITSKLDAYLTEFPPLIAH
PFQDKEHEIPTTDCAAVEESLEVDTSVGPVTWARGGTSHAFAVLASFCSQRLKKFADKRNDPNEDALSNLSPW
IHFGQISAARCVLEVQQQNKSCRSSVEAYVEECVIRRELSDNFCHYQPHYDSLLGTSDWARITLDDHRKDKRE
KIYSLAQFDRAKTHDDLWNAAQVQLVTEGKMHGFLRMYWAKKILEWTPDPDTALAYALYLNDRYSLDGRDPNG
FVGCLWSIGGLHDQGWREREVFGKIRYMNYAGCNKKMNIKAFVSKYRVREDMPGATNVKNDLQVTSTAFTVNK
KKREDEDSVQSEAPKKKAKTGSRNSKKIVSFSDDDE
>Hyalella_azt_XP_018013167.1_deoxyribod
MRQLRQTGYMHNRLRMVTACFLVKDLGLDWRRGEAWFAQHLNDFDLAANNGGWQWAASTGCDAQPYFRIFNPV
SQSEKFDPEGKFIKRYVPELARLPTAAIHAPWRAKPLELTEAGVVLGRDYPEPLVDHDEARRLTLARFAVAKA
PRPR
>Hyalella_azt_XP_018021492.1_cry-1-like
MALDRTKSPGQNSSFGLKMSPNKTVSPVMRSTHRTILGDNNLAQPECSGSSGPSMSSEKNSSTIKVRSGKHIV
HWFRRGLRLHDNPALRDSIFNCETFRCIYILDPWFAGSSNVGVNKWRFLLQCLEDVDNSLRNLNSRLFVVRGQ
PANVLPQLFKEWNTTVLSFEEDPEPFGRARDASIIGIAREMGIEVVVRTSHTLYKLDEIIEKKGGKPPLTYKT
FQNILAMMDPPPAPVPPIVAGDLQHAFTPIQPDHDDKYGVPNLEHLGFETEQLPPTAWKGGETEALQRLKHHL
ERKAWVASFGRPKMTPQSLFACPTGLSPYLRFGCLSARKFYTELNELYTKIKKVPAPVSLHGHLLWREFFYTA
ATNNPKFDHMKGNPICVQIPWDKNPEALAKWAHGQTGFPWIDAIMTQLRAEGWIHNVARHAVACFLTRGDLWV

SWEEGMKVFDELLLDADWSVNAGSWMWLSCSSFFQQFFHCYCPVRYGRKADPNGDFIRTYLPVLKNFPTKYIH
EPWMAPESVQSSARCIIGQHYPLPMVDHATQSQTNIERMKQVYRQLAHYRATMSSRSGGDSKSRCKHQQTSAP
NRSLCSNRVLTTV
>Hyalomma_asia__KAH6938626.1
MMDAEKLIGLGKELGLSGKALLQWVEKERERESEEYALRREAAKEDHERKLERQAGERQLLELRLRMQELQQT
SQVVPAEPSDRDASMQGRATSQCLSVALPPVSIRLKSQEAKPAFRPAPLCNPKLRGAQTRGRHCAVSSRRWSA
GKTRQRGARRVQRDCPRGSSRDVAGVLQWEPAAASGHVEDAVLVSVARRIVVPSLFSRSAPSTQTTAAGLGPR
HRNRVRLYASPHGPDQPTGPDYAYVAMAAMSHAPAPAPPHHQQQSTSVGGNSNGERHVVHWFRKGLRLHDQPA
LRIGLQGARTCRCVYILDPWFLLQSLEDLDNRLRKLNSRLFVIRGQPADVFPKIFKEWRVTHLTFEQDPEPYG
AIRDQKITALAHEMGVSVVCEPSHTLYPLERILERHGGKTPLTYRQFQSVVVDMEPPAQPLPAPESLPRTPID
EDHDERFAVPTLAELGFDTDNLKAAVWPGGETEALQRLERHMERKAWVASFGSPRMTPKALLASQTGLSPYLR
FGCLSARLFYHQLADLYRKIRNSNPPLSLQGQLLWREFFYCAATRNPNFDRMHNNPMCVQIPWDVNAEALAKW
ANGQTGYPWIDAIMRQLREEGWIHHVARHAVACFLTRGDLWLSWEEGMKVFDELLLDADWSVNAGSWMWLSCS
SFFQQFFHVYCPVRFGRKADPSGDYIRKYLPVLKHFPNNYIHEPWTAPESVQVAARCVVGRDYPLPMVNHQVA
SHVNLQRIRQVYQQLTQCVKTPGLFPSLPSASPTDSNKQNNNFIDDRKLRAKNSRVLFHILCFLHYFKMA
>Hydra_vul_XP_002166544.2_cry_DASH-l
MAGFSSRKTIIYWIRNDLRLHDNECLNFACRDGSHVLPLYCFDPDHYKLTHHFGFLKTGIHRAKLILDAVSGL
RNSLQKKGSNLVISKHRPKEAIEKLILECEQTAPVTSIVFQKEITKEETDVEASVFKVCDSKGIQVLSFWGST
LYHIEDIPFQINQIPNTYTLFRKGVEKCPVRKLNPIPDKISSLPNLKNFPLGEVPLLNELGFDKNEYEIDVRS
VFPFNGSEVDAVKRLNEYLWGNSAILTYKETRNGLLGKDYSTKFSPWLALGSLSPRMIYHKIKDFEEQKKIKE
NDSTYWVVFELIWRDYFKFICSKHGNKVFFKGGLKGGKGPWTWKQDLNLFMKWANGDTGIPFVDANMKELLAT
GWMSNRGRQNVASFLVKDLNLDWRMGAEWFESLLIDSDVCSNYGNWNYIAGIGNDPRSNRKFNVIKQAADYDE
EGDYVRTWLPVLKKLGICHTPWRLSASELNAGGVDLGKNYPHPIIVAPEWAKHALKMNSKNCFHEHRGKQNGQ
KRSIDFYFKSGKTGE
>Hymenolepis_dim_VDL14025.1_unnamed_pr
MSSTVQNNKDGFAKWMDQIDKNRVIDCGKSIAEYPFASCRIRKICGLNFFKELSNDELKGVTKSAGTKGPVVY
WMNRDQRVQDNWALLFAQRIAMKFSVPLDICFNLLSSPAIQTRRHANFLLEGLTEIEQASLLLIIHSICDSLS
FNYIEECKDLNIGFHVLFETGAAKPKTGGKRKADGETAANSVVNKKLTPLINFLSQVGARVIITDFSPLRDDL
HLLDIIKDQLPEDIPFYQIDAHNVIPVWFASDKMEYAARTIRPKLHEKAKALFTNFPPVVTHPCVKQTGPVNW
SKIKEFLNSRVIETVEAVDKYKGGSKAGFFQLYTFLHNRLSSYGKDRNDPTKDALSNLSPWFHFGKSDLPVLF
LSFGSERSLRMFGTEHCIRLWFRREIPIPCLLTAFTIARTSSGCCLLFSRPPLLPSSLLPPHLCNMLVGKILR
IFIPLHIRFNPAIGSETLRKHSNDIRQPAYSEEKMESASTGDELWNAAQRQLVYEGKIHGFLRMYWAKKILEW
HAGGPEKALQLGIHLLHTYSSFLLGVMWSICGIHDQGWVERPVFGKIRFMNFAGCKRKFDVTAFIKKYPPPVN
KHFKA
>Hymenolepis_dim_VDL61458.1_unnamed_pr
MRVLHWFRKGLRLHDNPALLASLNPPKKTSDEPIEILFLYIYDTTGKEYGMRGYRQAQFLLESLVDLDQSLKK
LGKNLRLLVVEGDPIMEVPALCKNLDVKYVTFEADIEPYSIVRDNEVTKKLEEEGVTVSRTHGHTMWDPEFLK
SHHQPPQTATKDSNSKNLGIPMTYQSFCALIRRVSPQGPSKPLPSFEEYAKSCTAPNLSELELGKVHIGPPSS
LEQLKIEFDPIPELGDHVSDDEGEKWEMTFPGGEIEGLDRLQRMVAQRPDWVCSFAKPETAPTSLPISTTGLS
PYIRFGCVSARHAWWVIAEAMRPASKASKTKPPTQPPVSLHGQLLWREFFNLVASVTPNFDRIEGNPICRPID
WNVDENLLSAWREARTGFPFIDAAMIQLHRHGWMHHLARHAVACFLTRGQLWQSWTAGADIFSSLLLDADWAL
NSANWMWLSASAFFHQYYRIYCPVSFGRKTDQDGEFIRHFIPALRNMPPEYIYDPWTAPMSVQKKAGCIIGVD
YPDPIVDHQEAKAENLERMKAAYADREVSPTPEQKRKITFGFGKAAMKNAKKMKK
>Hyphobacterium_ind_WP_109260360.1_DNA_photol
MSSSPRAPVIVWFRQDLRLSDNPALEAARQTGQAVLPLFILDDETPGQWRRGGASRWWLHHSLAALSGALEAI
GSHLLLRRGPAGAVLKAFIEETGATAVFWNRCFEPFNCERDKSIKAELSSSGLDVQSFNASLLAEPWTVRNKS
GDPFRVFSPFWRNIRSELDPGYPLKAPEHLPSPAEWPAGDPLQSWNLLPRNPDWAEGFNTVWTPGEAGARKRL
EDFLASGARQYAGGRDRPDKPYTSGLSPHLHFGEVSPRQIVSATESVRDDIGDRNAEKFLSELGWREFAYSLL
FNFPDLPEANYQAKFDRFPWRQDSAALKAWQRGQTGYPMVDAGMRELWATGWMHNRVRMITASFLIKHLMIDW
REGEKWFWDTLVDADLANNSASWQWVAGSGADAAPYFRIFNPVTQGEKFDPDGDYIRKWVPELARLPGKYLNQ
PWNAGPLILKEAGIELGKTYPAPIIDHKAARERALAAFQSLKETA
>Ichthyophthirius_mul_XP_004034973.1_IMG5_10828
MNRVQQYIWEQDLLKNYKNTRNGMVGADYSSKFSIWLANGCISPRYIYQEVKKYEKERIKNDSTYWIIFELLW
RDYFKFVAIKYKNKLFYKNGIQGLDINWNQEINYFELWKNGKTGYPLVDANMRELNSTGFMSNRGRQNVASFL
NKNMWVDWRMGAEYFESILLDYDPESNYGNWQYSAGIGNDGRQFRVFNITKQSNDYDPEGIYIKLWIPELKNV
PKNKLFEPWKLNKEEQKEYGVIIGKDYPEPILDYFETMSCSFSGEFKALEKSFIDELSEHLSPIPLASNAYIY
KYTQGHKLIDLPRGGYIIKTSVGNIQYGIPPETIKDSITLKLDVPTNFIIPSKRFDIIANLNVAEFEFPAYYN
YFIHKKKINLICDIETKECIRNAFQETLCGPISHENLAEDFYYTYPVSGYPDFKKERKILSGAIHDSQYDSNV
DSLLSFQVFENNKIILKEDDNIVEIQKYREQNSNSFYFKFIDNGQTVANILDELDVFQGFKVKIAEEFYKVDD
VCQEQKQQTIQQQINPQIKKITDIVQSSSLEKRGSKFNIPINIEARNTFRPPDFGVTVLGNSHGFDPCGHTTG

FVLWIYGKGIMVDPPPFSNDYLKNMNIMPQTISAVIITHCHADHDSGAFEKILLQSKIEVITTRTIMNSFVRK
YSNITAMSEEKIKKLFVFRPVIIGSSMKINGGLFNFFYSFHVIPCIGFDVEVMGKSIYFSADTFYDPIRLKKY
FEWGYLQKERYSQLALINFDKYDLILHEAGVPPIHTPLSSFKHLSQQARENLYILHIAEKDLKNQEGFKTMKE
GIENTIVIYQRNTNQQIDHLKTLDLISNIDIFSSLTVKNVRYLLDCLQIEKYDKGELIFPENSTGNKFYVIKS
GLVKCFSKSNQNFESFIGPGDYFGEISLIDEMKQRKASAKAEKKTTLLSLERHDFQFLFSEGSNILNKLKKLT
QARKQFVIVYVEKNNFLNSLDKNQKVSLEAIIKEKQVQKDECIWKQGQIPNLVVIIKSGKLLFIKQDNLAKIC
DSGEFIGESLCIIKKQPAVTSLKAIKDIQSNQIIINDEDPLVCSCLIEEPTTLCF
>Ichthyophthirius_mul_XP_004037245.1_IMG5_05753
MDNYWKNKELEIITLEQMGFEKSGKDFPSIQFNEKIVKTYDKTRDIPSLNGTSRMGVHLRFGTVSIRDLVRRS
KSLNQVYINELIWRDFYMMILDQFPRVEKYNFKQKYDKLEWRNNPEEFKAWCEGKTGYHIVDAGMRQLNCSGY
MHNRLRMITASFLTKHLLIDWRWGERYFAKKLLDYDLASNSGGWQWSAGTGTDSQPYFRIFNPDSQQKKFDPN
YTFIKTWVKDLNTKSYPKPIVEHTFARNRCLETFKKV
>Ictidomys_trid__XP_005330087.1
MAAAVVTAAATVPTPATSADGASSVHWFRKGLRLHDNPALLAAVRGARCVRCVYILDPWFAASSSVGINRWRF
LLQSLEDLDTSLRKLNSRLFVVRGQPADVFPRLFKEWGVTRLTFEYDSEPFGKERDAAIMKMAKEAGVEVVTE
NSHTLYDLDRIIELNGQKPPLTYKRFQAIISRMELPKKPVGAVSSQQMESCKAEIQEDHDDTYGVPSLEELGF
PTEGLGPAVWQGGETEALARLDKHLERKAWVANYERPRMNANSLLASPTGLSPYLRFGCLSCRLFYYRLWDLY
KKVKKNSTPPLSLFGQLLWREFFYTAATNNPRFDRMEGNPICIQIPWDRNPEALAKWAEGKTGFPWIDAIMTQ
LRQEGWIHHLARHAVACFLTRGDLWVSWESGVRVFDELLLDADFSVNAGSWMWLSCSAFFQQFFHCYCPVGFG
RRTDPSGDYIRRYLPKLKGFPSRYIYEPWNAPESIQKAAKCIIGVDYPRPIVNHAETSRLNIERMKQIYQQLS
RYRGLCLLASVPSCVEDLSHPVAEPGSSQAGSISSTGPRPLSSGQASPKRKLEAAEEPPGEELSKRARVAELP
TPELPSKDA
>Ictidomys_trid__XP_013212794.1
MGVNAVHWFRKGLRLHDNPALKECIQGADTIRCVYILDPWFAGSSNVGINRWRFLLQCLEDLDANLRKLNSRL
FVIRGQPADVFPRLFKEWNITKLSIEYDSEPFGKERDAAIKKLATEAGVEVIVRISHTLYDLDKIIELNGGQP
PLTYKRFQTLISKMEPLEIPVETITSEVIEKCTTPLSDDHDEKYGVPSLEELGFDTDGLSSAVWPGGETEALT
RLERHLERKAWVANFERPRMNANSLLASPTGLSPYLRFGCLSCRLFYFKLTDLYKKVKKNSSPPLSLYGQLLW
REFFYTAATNNPRFDKMEGNPICVQIPWDKNPEALAKWAEGRTGFPWIDAIMTQLRQEGWIHHLARHAVACFL
TRGDLWISWEEGMKVFEELLLDADWSINAGSWMWLSCSSFFQQFFHCYCPVGFGRRTDPNGDYIRRYLPVLRG
FPAKYIYDPWNAPEGIQKVAKCLIGVNYPKPMVNHAEASRLNIERMKQIYQQLSRYRGLGLLASVPSNPNGNG
GLMGYAPGENIPGCSSSGRRSSMGTGLSSGKRPSQEEDTQSIGPKVQRQSTN
>Indicator_macu__NXN14763.1
DNWAFLYAQRLALKQELPLHVCFCLVPKFLDATIRHYSFMLRGLQEVAKECAELNIPFHLLLGYAKDVLPTFV
VEHGVGGLVTDFCPLRLPRQWVEDVRERLPEDVPFAQVDAHNIVPCWVASPKQEYSARTIRGKIHAQLPEFLT
EFPPIIRHPYTSSCLAQPIAWEVCYSSLQVDRTVKEVEWATPGTAAGLAVLQSFITERLKSFGSHRNDPNKAA
LSNLSPWFHFGQVSTQRAILEVQKHRGKYKESVDVFVEEAVVRRELAENFCYYNENYDSVQGSAYDWAQTTLK
LHSKDKRPFLYKLEELEHGTTHDPLWNAAQLQMVQEGKMHGFLRMYWAKKILEWTHSPEEALKFAIYLNDRYE
LDGRDPNGYVGCLWSICGIHDQGWAERAVLGKIRYMNYAGCKRKFDVRQFERRYAPRA
>Indicator_macu__NXN16040.1
MQHSSIHWFRKGLRLHDNPALLAAATDCCHLHPLFILDPSSSRGGPNARRFLLDALQDLDRSLREIGSRLFVA
RGHPEEVLPRLFHAWGTTRLSFEVDTEPSAQRRDASVAELAAQHGVELIQEVSHTLYDTKRILALNNGKAPLT
YKHLQSLLASLGPPQKPTPALTREQLQGCCTSNHDTNYEVPTLEELVQDPTELGPHLYPGGETAALARLDTFM
EKRAWVCSFKKPETAPSSLSPSTTVLSPYLALGCLSARTFWWRLDEVYQGQQEHSQPPVSLQGQLLWREFFYT
AGASIPSFDQMLGNPICLQVDWDDNPQHLHAWREGRTGYPFIDAIMRQLRTEGWIHHLARHAAACFLTRGDLW
ISWEEGLKVFEELLLDADWSLNAGNWLWLSGSAFFHRYFHIYSPVAFGKKTDRDGAYIRKYLPILKDFPSEYV
YEPWKAPLAVQEQAGCLVGTHYPQPIVEHRAASKRNLERMRAARAQK
>Indicator_macu__NXN16223.1
MAHHSIHLFRKGLRLHDNPTLLAALQSSQLLYPLYILDQDFLTSVLHMGALRWHFLLQSLEDLQQSLCQLGSC
LLVAQGDYEAVLRELVQQWNITQVTLDAEVEPFYKEMEAKIRCLGEELGFEVLSLVGHSLYDTKRVLDLNGGS
PPLTYKRFLHILSLLGDPELPVRNLTAEDFQRCQPPAPGLAERYRVPLPADLKVPAQSLSPWRGGESEGLQRL
ERHLADQGWVTTFTKPKTIPNSLLPSTTGLSPYFSLGCLSVRTFFYRLAKIYAQAKHHSLPPVSLQGQLLWRE
FFYTVASATPNFTRMAGNPICLQISWAEDAERLHRWKTAQTGFPWIDAIMTQLRQEGWIHHLARHAAACFLTR
GDLWISWEEGMKVFEELLLDADYSINAGNWLWLSASAFCPQAHRILCPVRFGRRTDPQGHYIRKYLPILKNFT
SKHIYEPWTASEEEQKQAGCIIGQDYPFPMVNHKEVSDHNLELMRQAREEQHRRAQLTR
>Indicator_macu__NXN17905.1
RFLLQSLEDLDNSLRKLNSRLFVVRGQPTDVFPRLFKEWGVTRLTFEYDSEPFGKERDAAIVKLAKEAGVEVV
IENSHTLYDLDRIIELNGHKPPLTYKRFQAIISRMELPKKPVSTVISQQMETCKVDIQENHDDVYGVPSLEEL
GFPTDGLAPAVWQGGETEALARLDKHLERKAWVANYERPRMNANSLLASPTGLSPYLRFGCLSCRLFYYRLWE
LYKKVKRNSTPPLSLYGQLLWREFFYTAATNNPKFDRMEGNPICIQIPWDRNPEALAKWAEGKTGFPWIDAIM
TQLRQEGWIHHLARHAVACFLTRGDLWISWESGVRVFDELLLDADFSVNAGSWMWLSCSAFFQQFFHCYCPVG

FGRRTDPSGDYVKRYLPKLKGFPSRYIYEPWNAPESVQKAAKCIIGVDYPKPMVNHAETSRLNIERMKQIYQQ
LSRYRGLCLLASVPSCVEDLSGPVADSASGQGCSTSTVLRLSQAEQASPKRKHEGAEELCTEELYKRAKVTEL
PAAELPAKSL
>Indicator_macu__NXN18583.1
SGEKSHQGLAQLFLPFSSSVLKVDYDVCSNYGNWLYSAGLGNDPRDNRKFNMIKQGLDYDGNGDYVRLWVPEL
QGIKGADIHTPWALSSAALSQAGVTLGETYPEPVVTAPEWSRHISQRPQGRGPHPRSRRGPTHMPAQHKDRGI
DFYFSRKKDV
>Ischnura_eleg__XP_046383944.1
MNPAAIQFIKTENVSTSAGQGQISDILLKLDRDRNKVASSAAAYDFNLKRVRLMSETEKIPDRMQGVVYWMSR
DARVQDNWAMLFSQRIALKHRVGLHVCFCLVPKFLDATIRHFKFLLGGLRVVESDLKKLNIQFHLLRGKADDV
LPEFVEKNSIGAVVTDFSPLRTPLSWVKSFKEKLNPSIPFWQVDAHNIVPMWVTSNELEYAAYTIRGKIKHHL
PEFLTHFPPVISHPYPGTVKAEPVDWAAAEASLQVDQSVKEVSWAVPGTSAGLNYLQSFVANRLQLYATKRNE
PTVKAQSNLSPWIHFGQISIQRCVLEVQEKRPKCPESVDGWVEQAVIRRELADNFCYFNPNYDNLNGADQWAK
DTLNNHRKDKREYVYSLAQFEAARTHDDLWNASQIQMMREGKMHGFLRMYWAKKILEWSESPDDALSISIYLN
DRYNLDGRDPNGYVGIMWSICGIHDHGFPERPVLGRIRWMSYEGCKRKFDVMSFVSRYGAKKYRYEGKK
>Ischnura_eleg__XP_046393096.1
MGNDNPEKHTVHWFRKGLRLHDNPSLREGLKGATTFRCVFILDPWFAGSSNVGINKWRFLLQCLEDLDRSLKK
LNSRLFVIRGQPADALPKLFKEWGTTFLTFEEDPEPFGRVRDQNIIAMCKENGIEVCSKVSHTLYKLENIISK
NAGKPPLTYHQFQKVVASMDRPECAAPPITAPFVGSAYTPVLDDHDDKYGVPTLEELGFDTEGLLPPVWQGGE
SEALARLERHLERKAWVASFGRPKMTPQSLLASQTGLSPYLRFGCLSTRLFYYQLTDLYQKIKKARPPLSLHG
QILWREFFYCAATQNPNFDKMAGNPICVQIPWDKNQEALAKWANGQTGFPWIDAIMAQLREEGWIHHIARHAV
ACFLTRGDLWISWEEGMKVFEELLLDADWSVNAGMWMWLSCSSFFQQFFHCYCPVRFGRKADPNGDFIRRYIP
ALKNFPTKYIHEPWTAPESVQKAAKCIIGKDYSVPMVNHAGASRVNMERMKQVYQQLSKYRGAGSEHRPSAKE
CFVGLLATVPIAPSAWTSQSSKETKENRPATQNTKNDVNAQKSLSTSPKLIISSDIASREA
>Ischnura_eleg__XP_046403167.1
MVGSAIHWFRKGLRIHDNPALLAVTEKVDGENCIIRPVFILDPWFVKNMRVGPNRWRFLQQSLMDLHESLKKM
GSRLYVLKGKPEEVLPIAFRVWNVKLLTFELDIEPYARERDERIEALAKDAGVRVMQKISHTLFDTEAVIKAN
LGRPPLTYQKLISVVSGLGAPPNPVTMPDKFPKECFVGNEKADVHGEIPGWPEFNVPSLKDLGVKEEELGPNL
HHGGETEALKRLSKNIAKKEWICKFEKPNTSPNSLQPSTTVLSPYLKFGCLSSRLFYYKLKEVTKGQPRVSQP
PVSLIGQLLWREFYYVVGSATPNFDRMKGNPVCCQVPWNNNPEHLEAWTMGRTGYPFIDAIMTQLRLEGWIHH
LARHAVACFLTRGDLWISWEEGQKVFEELLLDADWALNAGNWMWLSASAFFHQFFRVYSPVAFGKKTDKLGDY
IRKYVPKLAKFPPQYIYEPWDAPLSVQKAAGCIIGVDYPKRIVIHESVSKANIQRMSAAYKSNKERKAGEEDE
GAPKKSKGNTKKRASDSDPLSSSANKKSKTKGNKSIANYFEK
>Ischnura_eleg__XP_046405998.1
MSELDFPKKTERGSALIWFRHGLRLHDNPALHEALKDCSQFYAVFIFDGESAGTKLVGYNRMKFLLESLADLN
DQLERRGGKLYLLKGTPSTIFKRLHEEVGLAKICFEQDCEPIWKERDNSVKTTCEELGMKCVEKVSHTLWDPK
EIIQTNGGKPPLTYQLFIETVPLIGEPRRPVQDVEWTNIKFGILPDHLLDEFEAFSSIPSPEAFGLEPEVKDK
SPVVRWIGGETHALHQLTKRLQVEENAFKNGVYLPNQANPDLIGPPTSLSAALRFGCLSVRRFYWEIHDVWMK
IRGKDCPPSDSITGQLIWREYFYTMSVDNPFYAEMERNPICLTIPWVDDDENLQKWAKGETGYPFIDASMRQL
LLEGWIPHAARNAVACFLTRGDLWISWEKGLSVFLYHLLDADWSVCAGNWMWVSSSAFERLLDCTMCVSPVSY
GRRLDPWGTYIKRYVPELSGFPPEYIHEPWKAPIVVQESAGCIIGHDYPERIVDHAVASKRNQKYMEAIRNSL
MSDFPAPHCCPSNEDETRHFLMMDDAEIDHVH
>Ixodes_sca_XP_029833167.1_cry-1
MSTSQTSTTAESGGRNVVHWFRKGLRLHDQPSLRVGLQGARTCRCIYILDPWFAGSSNAGLNKWRFLLQCLED
LDARLRKLNSRLFVIRGQPADVFPKLIKEWRVTHLTFEKDPEPYGAIRDQNITALAHEMGVTVVCEPSHTLYP
LERILERHGGKAPLTYRQFQGVLVAMEPPPAPVPAPEALPRTPIDEDHDDRFAVPTLAELGFDTDNLKDAVWP
GGETEALARLERHLERKAWVASFGSPKMTPKSLLASQTGLSPYLRFGCLSARLFYHQLADLYRKIKKSNPPLS
LQGQLLWREFFYCAATRNPNFDRMHNNPICVQIPWDVNAEALAKWANGQTGYPWIDAIMRQLREEGWIHHVAR
YAVACFLTRGDLWLSWEEGMKVFDELLLDADWSVNAGSWMWLSCSSFFQQFFHLYCPVRFGRKADPSGDFIRR
YLPVLKHFPNKYIHEPWSAPEQVQVAARCVVGRDYPLPMVNHQDASRVNLQRIRQVYQQLTQCTKAPGLFPSV
PSGSPTSADKASNKFIEDLKLTGKNAMQDDEESMEGVV
>Klebsormidium_nit_Photolyasec_GAQ87857.1
MASVQECRGALVGAGPLTVLVVGPAGAGKSSTVNAVFGRHFLAAKPAEASAGRFKLEECVQTINGSKVVVLEA
SGLSKAKDVEELRKSLSKKRKTVDLVLLVEALQQAGSVDVASEMGAKFGAQLLNAAVPVFACGHVQEEADEES
KALYAKRSAAVLAAFGANGAEVPSRVIPLELEGASVCEEGLKVVLEEMRRVRAQKTPAFVPATTMDADLKDLS
LVMDHNMSVASFNAAPAFLPKQAPVKVVRAPTKEAAPAPPAKLAEAAPKAPVQQSFPAPPPNVAGAASISIAV
QSAASMLAPALQAAAATQVKKLRNLRGGVPMIAPRSPIDCPAIKVTKTLVPARDAAASKTTMLSSAASSFAPV
AAAVLAPSKPAANNKLMGLSAFPDMQKLRGGDFAGPAGAAAARNVSIVWFRNDLRVHDNEALAMAQQASSSIV
PVYCFDPRDYGKSSSGFDKTGPYRAKFLIECVADLRKNLRERGSELVVRIGKPEEVLADLARKTGAKTLFAHQ
EVTHEELQSERKVCAALKDAGVQAKFFWGSTLYHIDDLPFKLESMPSNYAGFREQVKDLKVRKTAAALKTLKG

LPKLSSVEVGKVPTLQDLGLDASKSTRHETGAQALIGGESEALDRLKSFVADATAGAKKAADNLNGANFSCKI
SPWLAMGCLSPRRMYEDLAASGGARAITATAQKSPSEDANGLSWLTFELLWRDFFRFITKKYGTANKSASTAA
SPASAMAVAV
>Klebsormidium_nit_GAQ77897.1_photolyase
MVSILRSASCPTAKAVSNCSAKWALRQLAPAQPIIARHVSKPARLNLHPTFTPARSSVRGIPQPSGPSFPHRR
AEGSFRSRAAQFKSNQVGEGFASFGSLGATSNLETVSTPSLPSSFRGSPLQGSWTLVGFVRVRSAGGHRQGRG
FCTQAAAREFVIKETDMASTKETPVILWFRSDLRLHDNEALVRAAEASDSVVPVFCFDPRIYGRTYHFGFQKT
GPLRAQFIWDAVSDLRQQLRQRGADLLVRVSNPEDVIPSLAKSVGAQRVFASLETCSEEKAVERRMLHKLQEG
NSRGFLELVWGGVTMYHIDDLPFPAAAVPDVYTQFRKGVESRSKVREPLKIPKLKPISKEVLGKIQAAGGPGE
MPRLEALGVDPQAMDPRGVLPFKGGETAALERLNDYFWTKDCLKEYKETRNGMLGGDYSSKFSAWLAHGCISP
RYIHSEVRRYERERVENKSTYWLLFELIWRDYFRFISIKFGDKLFKLGGLRDVRGKEWGQNKQLFDTWREGRT
GYPLVDANMKELAATGFMSNRGRQIVCSFLTRDMKLDWRLGAEWFETCLLDYDPCSNYGNWTYGAGVGNDPRE
DRYFSIPKQAQNYDSEGAYVAYWLPEIAKLPTYKRHAPNTLTREEQDKYDVEIGPGGYPAPVVPLLHGQPGGG
GRGGGFSGRGRGGGRDGRSGRGRGGRDGRAAKF
>Klebsormidium_nit_GAQ81945.1_photolyase
MASKRKAPSRGAEGADVKELLEKAVKKGKNAAGKAVESTAGAVKDAAGGAAKAVKKATEKVAEVPKAAAKAVL
PSSAKDEGAGPSSKTPPLSRLPADLNKLVDPKRVMTLREGEVTGTGPVLYWMSRDQRSADNWALLYAIEQAHK
QGSSVAVIFNLVPRFLHAGARHFGFMLRGLRVMEKNLKEKDIPFFIVQGEPVDTIVQFVAKVGTSLLVTDFSP
LRIAREWKGGVCSLVNIPVHQVDAHNVVPTWVCSDKQEYGARTIRTKVHRNLPEFLVEYPELPSNPNKWALEP
PAPIDWEALLKIVLSNKAVPEIDWAVPGEDAGMEWLLKRFLGKGIAKYDADRNDPTIPTGVSGMSPYLHYGNV
AAQRAALEARKYRKSHPKAVDAFLEELIVRRELSDNYCYYQPNYDNLQGAFPWARESLQKHAADPREYVYTKE
QLEKGQTYDELWNASQHEMVHHGKMHGFMRMYWAKKILEWTPSPEEALAIAIELNDKYEIDGRDPNGYVGCMW
AIAGVHDQGWGERPIFGKIRYMNYAGCKRKFKVDKYVAYVNKLMSDLKTKGKSSAAREKKPV
>Klebsormidium_nit_GAQ88501.1_photolyase
MEPVTVVWFRRDLRLEDNPALISAARMGTVVPVFIWAPEEEGQFQPGRCSRWWLKQSLLHLDVTLQKMGAPLI
LRRCADSATMLLDIVKETGATQVFYNHLYDPVSLVRDHRVKQSLVQHGVVVHTFNADLLYEPWEVYDEQGHPF
TVFENYWNKCLNMPFEPDAPLLAPKKLSGIEIEVPSLSLDGIGLEDPLEASSNSLLARAWAPGWSNADRALES
FLRGALASYATARTKTDTTTTSLLSPHLHFGELSVRKIFHESRKLQLLWSRDGASVAVTSVDLFLRSIGLREY
SRYLSFNFPFTHERSLLANLKSFPWAIDEKAFKAWRQGRTGYPLVDAGMRELWATGWLHNKIRVVASSFCVKF
LQLPWRWGMKYFWDTLLDADLESDVLGWQYISGSLPDGHELDRMENPHVEGYKYDPEGEYVRRWLPELSRLPT
EWVHHPWDAPSNVLRAAGVELGSNYPQPIVDVEAARERLAASLSAMWASEAALKAGGLEEGLGETPAVAVGAG
ISGFGRSSAAGSSRRDQMVPDMAPAPSLAAEEAAAHILGHQEAYQHELSSPTPPERLDASSAERPVDQAQPLP
GFRELNQDGAGLMREPQLPLRRGSESDILSTAESSSYPRPSEGGANAPKVPDPKNSWPYASPDLASGLQAAAD
AARAQTYTGIPFNKKHLLELQDGTPLEDPLMKRSRMRQMASPPIQGGR
>Klebsormidium_nit_GAQ91785.1_photolyase
MSSLMWFRKGLRLHDNPALLAAIEGAKHVYPVFILDPYFLRPDPTAPSPGSTRVGINRIQFLLQSLQDLDQGL
QARGSRLLLVHGDPVEIIPSMITQWRVNRLCFERDTEPYAKERDARVKELAAELGIKELVTPSTHTLFDPDET
IARNKGHVPTSYGQFCKAIGTPAEPLEAPTSIPGPDPEKKGIPTISVPTLTELGYGESDQEFLQQIVKGGETE
ALARLEVMMAKTDWVAKFEKPKTDPSAFAEPATTVLSPYLKFGCLSPRLFYSKLMAIYQKKTGHSKPPQSLEG
QLLWREFFYTVGHATPRFDCMVGNSLCKQVPWTDDDALLAAWRDAKTGYPWIDAIMTQLRQQGWMHHLARHAV
ACFLTRGDLFVYWEKGRDVFDRLLIDGDWSINNGNWLWLSASAFFSQYFRIYSPITFGQKYDKSGKYIRRFLP
VLKDFPAQYIYEPWKAPLDVQKAAKCVIGVDYPGPIVDHAEASKQCKERMAAAYALAKQSTDLPSQEAVEELR
RTVNPGGVKRKEQYGAAGSMAPAGKKSKGDIRKYLKK
>Labrus_berg__XP_020487077.1
MSKDKCNCECPYTFRAFPKQDTVEGGDQMMNMGDLTENEKPKRSKNRRQRRKGACHHVVGLPPSPSARPPVLL
WFRRDLRLCDNPALNGSLEVGAPVIPIFIWSPEEEEGPGITVAMGGACKYWLHQALSCFCSSLECIGSRLVFL
KAKAAKNEVGPTLHCLKELVKETGATTVFANALYEPWLKDRDDEVVSAFKKDGVEVKMFHSYCLKDPYSVSTE
GVGLRGIGSVSHFMSCCKQNPGSALGVPLDPPVSLPTPAQWPQGVSLDGLGLARMPRRKDGTTIDWAANIRKS
WDFTEKGAHARLEAFLQDGVYRYEKDSGRADAPNTSCLSPYLHFGQLSPRWLLWDAKGARCRPPKFQRKLAWR
DLAYWQLTLFPDLPWESLRPPYKALRWSVDHGHLKAWQRGRTGYPLVDAAMRQLWLTGWMNNYMRHVVASFLI
AYLYLPWQEGYRWFQDTLVDADVAIDAMMWQNGGMCGLDHWNFIMHPVDAAMTCDPCGSYVRTWCPELADLPE
ELIHKPWKCPTSMLRRAGVVFGQTYPERIITDLDERRNHSLQDVALVRKEFSQYVDKRSGCDLVPLPPRLVSE
ALGLSHRDGGVVKEGKQFLLPVITRMEFKHQLEDPDADAASNPYNAVLKGYVSRKRDETIAFLNERDFTASVM
YEGTQRRERLESDYRRMEGLPTPPAARGRARRTPTAKDRFSVVPGGAVTSLR
>Labrus_berg__XP_020497213.1
MVVNSVHWFRKGLRLHDNPALQEALNAADTVRCVYILDPWFAGAANVGINRWRFLLEALEDLDNSLRKLNSRL
FVVRGQPTDVFPRLLKEWNVTRLTFEYDPEPYGKERDGAIIKMAQEFGVETIVRNSHTLYNLDRIIEMNNNSP
PLTFKRFQTIVNRLELPRRPLPPISQQQMDKCHAKIADNHDQLYGIPSLEELGFRTVGLSPAIWHGGESEALD
RLNKHLDKKVWVANFEHPRVNMCTLYASPTGLSPFLRFGCLSCRVLYYNLRELYMKLRKRSSPPLSLFGQLLW
REFFYTAATNNPNFDRMDGNPICVQIPWDQNPEALAKWAEGRTGFPWIDAIMTQLRQEGWIHHLARHAVACFL

TRGDLWISWESGMKVFEELLLDADWSVNAGSWMWLSCSAFFQQFFHCYCPVGFGRRTDPSGDFIRHYIPILKD
YPNRYIYEPWNAPDSVQKAANCVVGVHYPKPMINHAECSRLNIERMKQVYQQLSHYRGLSLLASVPTIQEEAE
PPMTDESQTSSGPDSPPRSPAGAEAAGCSTAPDSSTVCTPAAYAQNEDLEDFENQTPCTSSNTYPSAAITSTS
TSACLPPSTATPPSPAQSPLSWSKPSTPSSSCPSLPPPPSPATTPTQTSRKDLSRKVRRSQRQQGDCSAQQQQ
QGRKRGGQRRRERMRGGRREWKRASSRRRREWRMRHVERQQDIISDTR
>Labrus_berg__XP_020497566.1
MNSWSKVKRIIVTRQSKATSEDFCLQGHLIIVVGDTPSPTFPPSLGILKTAGDMVVNTIHWFRKGLRLHDNPS
LRDSMRGADTLRCIYILDPWFAGSSNVGINRWRFLLHCLEDLDASLRKLNSRLFVIRGQPTDVFPRLFKEWKI
NRLSYEYDSEPFGKERDATIQKLASEAKVEVMVRISHTLYDLAKIIELNDGQPPLTYKRFQALVSRMEAVEHP
AETITPDVIKNCVTPISDDHDHKFGVPTLEELGFDTEGLSEAVWPGGETEALIRLERHLERKAWVANFESPRM
NANSLLASPTGLSPYLRFGCLSCRLFYSKLTDLYIKVKENNSPPLSLYGQLLFREFFYTSATNNPCFDKMEGN
PVCVQVPWDRNPEALAKWAEGQTGFPWIDAIMTQLRQEGWVHHLARHAVACFLTRGDLWISWEEGMKAFEELL
IDADWSVNAGSWMWLSCSSFFQQFFKCYCPVGFGRRTDPSGDYIRRYLPVLRGFPAEYIYDPWNAPEEVQKAA
KCIIGVHYPKPMVNHAEASRINIERMRQIYQQLSSYRGLGLLAAVPSNPSSNMRGVGAGSSQAPRGASENPLT
QETIPRTERGQMTQKRRYEEALPESCRKYLKQSK
>Labrus_berg__XP_020501533.1
MSTSRTVICLLRNDLRLHDNELFHWAQRNAEHIVPLYCFDPRHYVGTYNYNLPKTGPFRLRFLLESIRDLRNT
LLNKGSNLVVRQGKPEEVVADLIKQLGSVSTVAFHEEVTSEELNVESRLKDVCAQMKVKVHTCWGSTLFHRDD
LPFPHMSRLPDVYTQFRKAVETQSRVRPLLPTPEQLKPLPQGLEEGAIPTAEDLLQTEPMSDPRSAFPCSGGE
SPALARLKHYFWDTNAVATYKETRNGLMGVDYSTKFAPWLAMGCISPRYIYHQIQQYERERTANQSTYWVIFE
LLWRDYFKFVAVKYGNRLFQIQGLQDKSVSWKKDKKLFDAWKEGRTGVPFVDANMRELALTGFMSNRGRQNVA
SFLTKDLGLDWRMGAEWFEYLLIDHDVCSNYGNWLYSAGIGNDPRENRKFNMIKQGLDYDSNGDYVRQWVPEL
QGIRGADAHAPWTLSSAALSHAQVSLGETYPTPVVMAPEWSRHVNKKPSGAGPSPRGKKGPSHTPKQHRDRGI
DFYFSKSKNL
>Labrus_berg__XP_020506634.1
MSGKKRKAPTSAAKEPSAKQQKLAPCKEEKQERAGGWLQDHLKQQRTEKKDMKFNKKRLRFVSKAEDVKQGSK
GVLYWMSRDQRVQDNWALIHAQQLAVKENLPLHICFCLVVPKSELSTLRHFSFMLKGLKEVAEECKTLDIQFH
LLHGSAGEVLPGFVSDRGLGAVVTDFCPLREQMQWLEDVKNLLPKDIPLIQVDAHNIVPCWVASPKLEYAART
IRGKITKLLPEYLTDLPLVEQHPYTATRTAKPIDWDETLASLKVDTKVGETDWAKPGTKAGVAMLESFIDVRL
KLFDTKRNDPNAHALSQLSPWIRFGHLSAQRVALQVQHSGKTAGVTVASYIEELVVRRELTDNFCFYNKKYDS
VDGAYEWAQKTLKDHAKDKRPYLYTREQLEKAKSHDKLWNGAQYQMVSEGKMHGFLRMYWAKKILEWTSSPEE
ALSTALYLNDRYSLDGQDPNGFVGCMWSICGIHDQGWAERPVFGKVRYMNYKGCLRKFDVARFEKQYCPKNL
>Labrus_berg__XP_020513567.1
MAHTCIHWFRKGLRLHDNPALIAALRDCKELYPVFILDPYLHNKTSVGINRWRFLIGALKDLDCSLRKLNSRL
FVLRGKPEDVFPKLFNQWKVTKMTYEFDTEPYSLSRDKTVTTLAKDHGVEVIYKISHTLYDIERIIEENNGKA
PLTYTRMQAIVKTLGPPKRPISAPTVEDMKDVKTPSFKKHEEEYGLPTLEELGHNTSALFEEKFPGGEQEALR
SLDEHMQRTGWVCGFEKPQTSPNSLSPSTTVLSPHVTFGCLSARTFWWRLTDVYQGKRHSDPPVSLHGQLLWR
EFFYTASVGVPNFNKMEGNPVCTQVDWDTNPEYLAAWREARTGFPFIDAIMTQLRQEGWIHHLARHAVACFLT
RGDLWINWEEGQKVFEELLLDGDWALNAGNWQWLSASTFFHQYFRVYSPVAFGKKTDKNGDYIKKYLPLLKKF
PAAYIYEPWKAPHSVQQAAGCIVGKDYPRPLVEHEVISKKNIQRMKLAYAKRSADSESPSKKQGVKRKQPSVA
DFMKKKEKKK
>Ladona_fulv__KAG8231453.1
MRSRNPNENKVTLLWFRHGLRLHDNPALLEALRCSKEFYAVFIFDGESAGTRVIGHNRLKFLLESLSDINSQL
QSHGGKLYLLNGKPFEIFKTLKEEVGLQKICFEQDCEPIWKERDDSVKDLCQEIGIECVEEVSHTLWDPRVVI
ETNGGIPPLTYQMFLHTVAAIGPPPRPVQDADWTGVNFGILPKKVVDKFEAFTSVPSPEEFGLVDNTKSKGPK
VRWIGGESNALKNLSFRLEVENNAFCNGIYLPNQVNPDLIGPPTSQSAALSFGCLSVRKFYWAIHDLFNQVYG
ENLSNESITGQLIWREYFYTMSVDNPFYAEMKRNPICLDIPWHEDQENLESWKQGKTGYPFIDAAMRQLVQEG
WVHHAARNAVAFFLTRGDLWISWEHGLAFFLYYLIDADWSVCAGNWMWVSSSAFEKLLDCSQCVSPVSYGRRL
DPCGIYIKKYVPELSRFPIEYLHEPWKASLELQQSCGCIIGRDYPERIVDHNVASLRNRKYMEDICLSLMISP
PKHCCPSDEEETRRFLMITQEDVDHIH
>Ladona_fulv__KAG8231874.1
MEAAETHDDLWNSAQIQLSKEGKMHGFLRMYWAKKILEWTESPEEALSIAIYLNDRFSLDGRDPNGFVGCMWS
ICGIHDQGWAERAVFGKIRYMNYEGCKRKFDVAAFVARYGGKKYPYKGKK
>Ladona_fulv__KAG8233196.1
MAKGSVIHWYRKGLRVHDNPALLAATEKINGEYYEILPVFVLDPWIVKTMRVGPNRWRFLQQSLKNLDENLRK
IGSRLYVLRGKPEDVLPEAFKAWKVKAITFETDTEPYARERDERIETLAKKAGVSVIQKTSHTLYNPEAVLKA
NLGKPPLTYQKFLSVMNGMGPPPKPVMMPKNIPVGCLGYDAMKDKGSKLPGGPEYGVPSLSDLGVSEESLGPS
LFPGGETEALNRLSKFTAKKDWICKFEKPNTSPNSLQPSTTVLSPYLKFGCLSPRTFYYKIQEVIKGQSRVSQ
PPVSLIGQLIWREFYYVVSSATPNFDKMEGNPVCLQIPWDVNKEYLEAWSKGKTGYPFIDAIMTQLRSEGWIH
HLARHAVACFLTRGDLWISWEEGQKVFEELLLDADWALNAGNWMWLSASAFFHQFFRVYSPVAFGKKTDKLGD

YIRKYVPQLAKFPPQYIYEPWEAPLSVQKAAGCIIGDDYPKRIVIHETISKINIKRMSEAYKKNKESKIKAED
DVEQ
>Ladona_fulv__KAG8235753.1
MGSEKTEKHTVHWFRKGLRLHDNPSLREGLKGATTFRCVFILDPWFAGSSNVGINKWRFLLQCLEDLDRSLRK
LNSRLFVIRGQPADALPKLFKEWGTTFLTFEEDPEPFGRVRDQNITAMCKEIGIEVCSKVSHTLYKLEDIIKK
NNGKPPLTYHQFQMVVASMDPPEPAAPPIVKSFVGSAYTPISDDHDEKYGVPTLEELGFDTEGLLPPVWQGGE
TEALARLERHLERKAWVASFGRPKMTPQSLLASQTGLSPYLRFGCLSTRLFYYQLTDLYRKIKKAPPPLSLHG
QILWREFFYCAATHNSNFDKMAGNPICVQIPWDKNQEALAKWANGQTGFPWIDAIMTQLREEGWIHHLARHAV
ACFLTRGDLWISWEEGMKVGSHDKVFEELLLDADWSVNAGMWMWLSCSSFFQQFFHCYCPVRFGRKADPNGDY
IRRYLPVLKNFPTKYIHEPWMAPESVQRASKCLIGKDYSVPMIKKAPPPLSLHGQILWREFFYCAATHNSNFD
KMAGNPICVQIPWDKNQEALAKWANGQTGFPWIDAIMTQLREEGWIHHLARHAVACFLTRGDLWISWEEGMKV
GSHDKVFEELLLDADWSVNAGMWMWLSCSSFFQQFFHCYCPVRFGRKADPNGDYI
>Lampea_lac_AQX17843.1_putative_p
MSGVALHWFRKGIRLHDNVPLTKAIESCSTLIPIFILDTDFLDPKKVSANRLGFLLDSLKCLDEDLQERGSRL
IVAKGKPSEVIFSLIEEFKINKLTFERGTEPFNKDLDDELIGIVNANDIEVYSNWGHILFNPDHILALNNGKT
PVTMNVFLKLASSAPTPLKPIPAPEKIPPPPADFKPKNVEIFISTPTLSDLKEYGYDPEAMTTWFKAGEKEGI
KIMEDFLKQKQRVASFEKPKTKPTALLPDTTALSPYLSVGSLSVRLFYHKLLEVLKSFKRHSKPPVSLEGQLY
WRELTYLIGYSTPNFHQMRENPLCYQIPWTEGEAAQEALKKWEMGQTGYPAVDAAMNQLRSDGWMHHLARHLV
ACFLTRGDLWISWELGRDVFDKYLIDADWSINNFSWHWMSCSAFFYQFFRCYGPVSFFKKTDPNGDYIRKHVP
ILAKYPVKYIYEPWEAPKNVQEAAGCIIGQDYPRPIVEHKTVSQANMGKLKKAYDKNKADRAKPAKVNAEKLE
KPLAKSKLR
>Laodelphax_str_AVP27638.1_cry_1
MSDEMSSSVLWFRHGLRFHDNPALHAAIKKEDNFYPIFIFDGESAGTKVIGSNRMRFLLESLKDLDTQLQNVG
QRLFVFKGSPVKIFDWMAKSIKMKTLCFEQDCEPIWAERDNAVKNFCDGAGVKCVEKVSHTLWNPKEVIEANG
GVPPLTYQMFLYTVSTIGNPPRPEADVDWSKVKFGKLPEDIPDDIFLYAGVPTLEDFNMHAYDGGERMVRWVG
GETNAIANLRNRILVEEEAFRCGFYLPNQANPDLVAPPTSQSAALRFGCLSVRRFYWTLHDLFNEIHEGKLTS
NQNITGQLIWREYFYTMSVDNIHYGEMTRNPICLDIPWMPKTNKLHNEFLQRWKEGMTGYPFIDAAMRQLLQE
GWIHHVARNAVASFLTRGDLWLSWEAGLRHFMEHLLDADWSVCAGNWMWVSSSAFEQLLDCSHCVCPVNYGRR
LDPWGVYVKRYVPELRNFPVQFIYEPWKLPLEDQEKYNCVIGRDYPERIVEHKVASQINRKKMEHIRDSLMSK
VPHCCPSSAEEVRQFMWLPENCSDHVCVPN
>Laodelphax_str_AVP27639.1_cry_2
MTGEAVAKQQCGWSQDMVGGGGGRGGVQRGPEGGGGNKKQVGGQKHTVHWFRKGLRLHDNPSLREGLKNAATF
RCIFILDPWFTGASNVSINKWRFLLQCLEDLDNSLKKLNSRLFVVRGQPADVLPKIFKEWGTTNLTFEEDPEP
FGRVRDQNVMAICKEMGISVVSKVSHTLYRLDDIIEKNGGTAPLTYRKFQTVVASLDEPPQAEPTVTAKVVGA
AVTPIADDHDEKFGVPSLDELGFDTEGLLPPVWQGGESEALARLERHLERKAWVASFGRPKMTPQSLLASQTG
LSPYLRFGCLSTRLFYYQLSDLYKKIKKAAPPPSLHGQLLWREFFYCAATNNSNFDRMVGNPICVQIPWTNNP
EALAKWANGQTGFPWIDAIMHQLREEGWIHHLARHAVACFLTRGDLWVSWEKGMVVFDELLLDADWSVNAGMW
MWLSCSSFFQQFFHVYCPVRFGRLADPSGDFIRRYVPALKNFPSAYIHEPWNCPIELQKSAKCIIGEDFPLPM
VNHVQASRVNMERLKQVYSRLAEFRNNNMHLMRPLPSPSGPTTSNGVMTMLMDNCEISVNGLKHERNNAKEAI
DKGLFNTPFLPKHKDKLNQKRYIMHK
>Laodelphax_str_RZF47186.1_LSTR_LSTR0
MASKSDKSPPAKKQKLLDDVPSKSDTKSGSSLIKELKEKRNKTAASILQFKFNKNRVRILSEAKDVPDRAEGI
VYWMFRDERVQDNWALLFAQKLALKNKIPLHVCFCLKPRFMEATIRHYKFLLKGLEEVAEDCKKLNIQFNFLI
GDGERVLPGFVEKNSIGAVVVDFMPLKGPMSWAEQLKKTLPKDVPLCQVDAHNIVPCWIASDKQEYGARTIRN
KINNKLKEYLTEFPPVIKHTYGTVKDEVIDWEGGEKTLEVDRSVEPVKWLTPGYSGAIQMLDSFINKRLKKFA
TKRNDPVVDALSNLSPYFHFGQISVQRAILTVREHRSKCPESVDAFCEEAIIRRELADNFCFYNKNYDNINGS
FDWAKKTLNDHRKDKREYIYTKEELENAKTHDDLWNSAELQLLKEGKIHGFLRMYWAKKILEWTTSPEEALEI
SIYLNDKYSMDGRDPNGFVGCMWSICGIHDQGWAERNVFGKIRYMNYAGCKRKFDVAAFVARYGGKSYPYKKS
K
>Larimichthys_croc__KAE8291179.1
MSDKKRKPTSAASAAAKGSSAKLQKLTPMKEKAGGWLQDLMKQQRTEKKDMKFNKKRLRVISDTEKIKQGSEG
VLYWMSRDHRVQDNWALIHAQQLAVQENLPLHVCVCLLVPKSELSTLRHYSFMLKGLEEVAKECKALDIQFHL
LHGAAGDVLPGFVSDRSLGACGDGLLPPQRATAVVDAHNVVPCWEASPKLEYAARTIRGKITKLLPEFLTDFP
LVEKHPYTATRTAKPVDWEKNQASLDVDRTVREPEWAKPGTKAGVVMLESFIDVRLKLFETQRNDPNAAALSQ
LSPWIRFGHLSAQRVALQVQRSGKNAGQSVSSFIEELVVRRELTDNFCFYNKKYDSIEGAYEWAQKTLKDHAK
DERPYIYTREQLEKAKTHDKLWNAAQYQMLTEGKMHGFIRMYWAKKILEWTSSPEEALSIALYLNDRYSLDGQ
DPNGFVGCMWSICGIHDQGWAERPVFGKIRYMNYKGCLRKFDVAQFEQKYCPKNI
>Larimichthys_croc__KAE8296035.1
MVVNSVHWFRKGLRLHDNPALQEALNGADTVRCVYILDPWFAGAANVGINRWRFLLEALEDLDSSLKKLNSRL
FVVRGQPTDVFPRLLKEWKVTRLTFEYDPEPYGKERDGAIIKMAQEFGVETIVRNSHTLYNLDRVIEMNNNSP

PLTFKRFQTIVSRLELPRRPLPPITQQQMDKCRTKIADNHDQLYSIPSLEELGFRTEGLPPAIWRGGESEALD
RLNKHLDKKVWVANFEHPRVNTCSLYASPTGLSPYLRFGCLSCRVLYYNLRELYMKLRKRCSPPLSLFGQLLW
REFFYTAATNNPNFDRMEGNPICVQIPWDQNPEALAKWAEGRTGFPWIDAIMTQLRQEGWIHHLARHAVACFL
TRGDLWISWESGMKVFEELLLDADWSVNAGSWMWLSCSAFFQQFFHCYCPVGFGRRTDPSGDYIRRYIPILKD
YPNRYIYEPWNAPESVQKAANCVVGVDYPKPMINHAEGSRLNIERMKQVYQQLSHYRGLSLLASVPTIQEEAE
PPMTDESQTSSGPDSPPRDPEDAVDGYESQTPCNPSGLHTQPSAAITSTSACHPSSKAVPPTQSPLSRSKPSS
PSSSCHSSSPSPSPATPSSQTSSLGQRRKGITRKMRRSQRQRGRQSCTPTAKEGDRRSEEEEREERMEEDAEQ
DEERMEEETFGEAAGHQQ
>Larimichthys_croc__KAE8296365.1
MSPSAAAGAEDSKALVRRMLREVLVGREDPEGFFVMCLSALGHQETRSQFVSLIQPLSTANSSLHCTLTSIYR
EYFSKTEDDELELAMVLSLLEMKDHRLSTPSQESRCRQTGDRADRRKNINLPQGEPWVKVSPPQTARETDLEK
STHVYKYNTETLGTSQTVCVSTLSGSISKQDKFEQGYQKMNEGDSNQSEKPKRSKNRRQRRKGPCQQIVGLPC
SPSAPPPVLLWFRRDLRLRDNPALICSLEFGAPVIPVFIWSPEEEEGPGNTVAMGGACKYWLHQALSCFCSSL
ERIGSHLVFLKANHEGNKVGSSLSTLKELIKETGARTVLANALYEPWLKERDDAVVSSLQKDGVEFRMFHSYC
LRDPYSISTEGVGLRGIGSVSHFMSCCRQNPGSALGVPLDPPVSLPTPVPWPQGVPLDTLGLARMPCRKDGTA
IDWAANIRESWDFSEEGAHARLEAFLHDGVHRYDKESGRADAPNTSSLSPYLHFGQLSPRWLLWDSKGARCRC
PKFQRKLAWRDLAYWQLTLFPDLPWDSIRPPYKALRWSTDRSHLKAWQQGQTGYPLVDAAMRQLWLTGWMNNY
MRHVVASFLIAYLYLPWQEGYRWFQDTLVDADVAIDAMMWQNGGMCGLDHWNFVMHPVDAAMTCDPYGSYVRK
WCPELASLPDELIHKPWKCPASMLRRAGVVFGQTYPERIVTDLEERRSQSLQDVALVRKEFRHYVDKRSGCDL
VPLPPRLVSEALGLSHRDGDVVTAGTQFLLPVITRMEFKHQLEDPEADAASNPYNAVLKGYVSRKRDETIAFF
NERDFTASVMYEGTQRKERLENDYRRMEGLSQLPAPRGRARRTPTANDRFSIVPGGAVTSLR
>Larimichthys_croc__XP_010751564.3
MSTSRTVICLLRNDLRLHDNELFHWAQRNAENIVPLYCFDPRHYVGTYNYNLPKTGPFRLRFLLESIRDLRNT
LLNVGSNLIVRRGKPEEVVADLIKQLGSVSAVAFHEEVTSEELNVEKQVKDVCAQMKVKVHTCWGSTLYHRDD
LPFHHISRLPDVYTQFRKAVETQSRVRPVFTTPEQLKPLPSGLEEGAIPTADDLQQTEPVTDPRSAFPCSGGE
SQALARVKHYFWDTDAVATYKETRNGLIGVDYSTKFAPWLAMGCISPRYIYHQIKQYERERTANQSTYWVIFE
LLWRDYFKFVGVKYGNRLFQVKGLQDKTVPWKKDMRLFDAWNEGRTGVPFVDANMRELAMTGFMSNRGRQNVA
SFLTKDLGLDWRMGAEWFEYLLIDHDVCSNYGNWLYSAGIGNDPRENRKFNMIKQGLDYDNNGDYVRQWVPEL
QGIKGADVHTPWTLSPAALSHARVSLGDTYPTPMVTAPEWSRHFNKKPSGTGPSPRGKKGPPHTPKQHRDRGI
DFYFSRSKNL
>Larimichthys_croc__XP_010755355.2
MAHTCIHWFRKGLRLHDNPALMAALRDCKELYPVFILDPHLYNNTCMGINRWRFLIGSLKDLDCSLRKLDSRL
FVLRGKPEDVFPKLFNKWKVTKLTYEYDTEPYSLSRDQTVTALAKEHGVEVIYSVSHTLYDIDRIIEENNGKA
PLTFIRMQAIVKTLGPPKRPIPAPTMENMKDVKTPCSENHEKNYAIPTMEEVGQDTAALEEEKFPGGEQEALR
RLDEHMKRTGWVCNFEKPHTSPNSLSPSTTVLSPYVTFGCLSARTFWWRLTDVYQGKKHSAPPVSLHGQLLWR
EFYYTASVGIPNFSKMEGNPVCTQVDWDTNAEYLAAWREARTGFPFIDAIMTQLRQEGWIHHLARHAVACFLT
RGDLWINWEEGQKVFEELLLDGDWALNAGNWQWLSASAFFHQFFRVYSPVAFGKKTDKNGDYIKKYLPLLKKF
PAQYIYEPWKAPRSIQQAAGCIVGKDYPHPIVQHEVISKKNIQRMKLAYAKRSTDAAESPSKKQGVKRKSTSV
IDMMKKKKDRKK
>Larimichthys_croc__XP_019118656.1
MVVNTIHWFRKGLRLHDNPSLRESIRGADTLRCIYILDPWFAGSSNVGINRWRFLLHCLEDLDASLRKLNLRL
FVIRGQPTDVFPRLFKEWEITRLSYEYDSEPFGKERDAAIQKLACEAGVEVMVRISHTLYNLAKIIELNDGQP
PLTYKRFQALINHMDAVELPAETITLDVIKECAPPISEDHDDKFGVPSLEELGFDTEGLTTAVWPGGETEALI
RLERHLERKAWVANFERPRMNANSLLASPTGLSPYLRFGCLSCRLFYYKLTDLYRKVKRNNTPPLSLYGQLLW
REFFYTTATNNPCFDKMEGNPVCVQIPWDRNPEALAKWAEGQTGFPWIDAIMTQLRQEGWIHHLARHAVACFL
TRGDLWISWEEGMKAFEELLMDADWSVNAGSWMWLSCSSFFQQFFHCYCPVGFGRRTDPNGDFIRRYLPILRG
FPAKYIYDPWNAPEDVQKAAKCIIGVHYPKPMVNHAEASRINIERMKQIYQQLSCYRGLGLLATVPVNPTNGS
NGSNVGRVNTESSQRPGGSCENPSIQEGTSRTERGQSTQKRRREDASLESSSKSWKQSK
>Lasallia_pus_KAA6407800.1_cry-2_
MPAAKRRVIYWFRTDLRLHDSPALKAALDLKPECLYPIWCWDPHYVYRARVGSNRWQYLLDCQSDLSASITKL
NPKSKLFLIREAPQTLLPKLFKAWKISHLVFEKDTDAYARDRDERVSELAKQAGVEVVVRMGRTLYDPDDLVK
VNGGKPTMSITQVQHAADKLGEVARPIPAPTSIPDPGETSLNFEQTQPSQDPDINATQRSGNESSYTHLSGPN
GDFAIPTLEELGLKAATTSHRGGETVALAQLDRIIKDTVYTATFSKPATAPTAFSPQATTLLSPHHHFGSLSV
REFYWRVQAVLANYNGKAKASTVPTNLIGQLLFRDMYFGAQASLGYKYAQELGNSHCRFVPWHLPSKVDEKNG
LITAGYTVDAPQAEEWFQRWKHGRTGFPWIDALMRQLRFEGWIHHLGRHAVACFLTRGGCYVHWERGAEVFEE
WLVDHEVACNVGNWQWLSCTAFYSQFYRCYSPIVFPKKWDPHGDFVRHYVPELRDYPEKYIYEPWKAPISDQK
KAGCLIKGDGTDEEINGMNAYPKPMLDFSERRTVCIEAIKNAYHVNLYGNNPQVLDGTWRALFADDGEGPTEG
KSSKDGLKHDNERQVKHSADTQNGEAGVDENEGEAAEDGGDNKGGDKEGSKRKRGQGTLDGLFTRKKARLEMT
K
>Lasallia_pustul_KAA6416131.1

MLQKRAANGNGIKRKAIPPPDSPRKGPKNGITLYNESPKEEAYGIVQREFYPPEMTNARCHQYNNNELPRPIE
LLEAAQEDTKEARENIPVKDAVVHWYKCDLRTRDNKALHLAHRKARSKGVPLICMYIVSPQDFQAHLTSPVRV
DFILRTLAVLKEDLAELGIPLHVELIEKRKTIPDKIIEWCEKWGASHIYANAEYEVDELRREAMMTRKCLDKG
ISMTVVPDTCVVAPGELSSQQGRQYAVYSPWFRAWVAYLHQHPHQLDPYERPGQNPSSAHEKFKDIFEGKIPE
APENKKLNDEEKKRFHSMWPAGEQEALARLEMFLDQKIGKYKDSRNFPASNGTACTSVHFASGTLAARTAIRR
ARDVNSTTQLDKGNLDIASWISEVAWRDFYKHVLAHWPYICMNKPFKPEYTNIAWEYSPTLFHAWTTGHTGYP
IVDAAMRQLNTTGYMHNRCRMITASFLAKHLLLDWRLGEIYFMEHLIDGDWASNNGGWGFSASSGVDPQPYFR
IFNPLLQSEKFDAAGEYIRKWVPELREVPGKAVHDPYARGAAKVAEKEGYPRMVVEHRVARERCLERYKAGLG
RGTA
>Lateolabrax_macu__UBK24604.1
MAHTCIHWFRKGLRLHDNPALMAALRDCKQLYPVFILDPHLHNNTSVGYNRWRFLIGALKDLDCSLRKLNSRL
FVVRGKPEDVFPKLFNEWKVTKLTYEYDTEPYSLSRDKAVAALGQEHGVEVIYKISHTLHDIERIIEENNGKA
PLTYNRMQAIVKALGPPKRPIPAPTMEDIKDVKTPHSEDHEKKYGIPTLEELGQDTAALGEEKFPGGEQEALR
RLDEHMKRTGWVCSFEKPQTSPNSLSPSTTVLSPYMTFGCLSARTFWWRLTDVYQGKKHSDPPVSLHGQLLLR
EFFYTASVGIPNFNKMEGNPVCTQVDWDTNPEYLAAWREARTGFPFIDAIMTQLRQEGWIHHLARHAVACFLT
RGDLWISWEEGQKVFEELLLDGDWALNAGNWQWLSASTFFHQFFRVYSPVAFGKKTDKNGDYIKKYLPLLKKF
PAQYIYEPWKAPRSIQQAAGCIVGKDYPHPIVQHEVISKKNIQRMKLAYAKRSANPAESPSKKQGVKRKAPSV
VDMLKKKERKK
>Lateolabrax_macu__UBK24605.1
MVVNTIHWFRKGLRLHDNPSLMDSIRGADTLRCIYILDPWFAGSSNVGINRWRFLLHCLEDLDASLRKLNSRL
FVIRGQPTDVFPRLFKEWQITHLSYEYDSEPFGKERDAAIQKLASEARVEVIVRISHTLYDLVKIIELNDGHP
PLTYKRFQALINRMNAVELPAETITLEVIKNCATPISEDHDDKFGVPSLEELGFETEGLAKAVWAGGETEALV
RLERHLERKAWVANFERPRMNANSLLASPTGLSPYLRFGCLSCRLFYFKLTDLYKKVKKNSTPPLSLYGQLLW
REFFYTTATNNPCFDKMEGNPVCVQIPWGRNPEALAKWAEGQTGFPWIDAIMTQLRQEGWIHHLARHVVACFL
TRGDLWISWEEGMKVFEELLIDADWSVNAGSWMWLSCSSFFQQFFHCYCPVGFGRRTDPNGDFIRRYLPILKG
FPAKYIYDPWNAPEEVQKAAKCIIGLHYPKPMVNHAEASRINIERLKQIYQQLSCYRGLGMLATVPANHNNDA
NGKRVDAGTRQGSAGASENPSIPEGMSRIERGQSTQKRRREEVPLESSSKSWKQSK
>Lateolabrax_macu__UBK24609.1
IPSLEELGFRTEGLPPAIWHGGESEALDRLNKHLDKKVWVANFEHPRVNMCSLYASPTGLSPYLRFGCLSCKV
LYYNLRELYMKLRKRCSPPLSLFGQLLWREFFYTAATNNPNFDRMEGNPICVQIPWDQNPEALAKWAEGRTGY
PWIDAIMTQLRQEGWIHHLARHAVACFLTRGDLWISWESGMKVFEELLLDADWSVNAGSWMWLSCSAFFQQFF
HCYCPVGFGRRTDPSGEYIRRYIPILKDYPNRYIYEPWNAPQLVQKAANCVVGVDYPKPMINHAEGSRLNIER
MKQVYQQLSHYRGLSLLASVPTIQEEAEPPMTDE
>Lates_calc__XP_018519383.1
MSPSAVGADDSKSLVRKMLREVLVGREDPEGFFAMCVSLLGHQETRSRFLSLIQPLSTANSSLYSTLTSIYKK
YFSETEDDQLELALALSVLEMKDHQLSSASQESRLQQLQSSSAQLTSVSKPQGSSHTQLTDVAAENTYSAQTG
PLGMVCPPKTTQETELQRSTHVDKNNKETKGTCQTVCVSRPSASFSRQEMVVEGDQTVQEGDQSEKPKRSKNR
RQRRKGGSQQVVGLPHSPSAQPPVLLWFRRDLRLCDNPALIGSLEVGAPVIPVFIWSPEEEEGPGITVAVGGA
CKYWLHQALSCFCSSLERIGSHLVFLTANGEGNGVGSSLRSLKELIKETGARTVLANALYEPWLKERDDTVVS
ALQKDGVECRMLHSYCLRDPYSISTEGVGLRGIGSVSHFMSCCKQNPGSALGVPLDPPVSLPTPAHWPRGVSL
DTLGLARMPRRKDGTTIDWAANIRKCWDFSEEGAHARLEAFLHDGVYRYEKESGRADAPNTSSLSPYLHFGQL
SPRWLLWDAKGARCRPPKFQRKLAWRDLAYWQLTLFPDLPWESLRPPYKALRWSSDRGQLKAWQRGRTGYPLV
DAAMRQLWLTGWMNNYMRHVVASFLIAYLHLPWQEGYRWFQDTLVDADVAIDAMMWQNGGMCGLDHWNFVMHP
VDAAMTCDPYGSYVRKWCPELADLPDELIHKPWKCPASMLRRAGVVFGQTYPNRIITDLEERRSQSLQDVAVV
RKEFGQYVDKRTGCDLVPLPPRLVSEALGLSHRGGDVVAEGKQFLLPVITRMEFKHQLEDPDADAASNPYNAV
LKGYVSRKRDETIAFLNERDFTASVMYEGTQRRERLESDQRRMEGLPRPPAPRGRARRTPTAKDRFSIVPGGV
VASLR
>Lates_calc__XP_018519856.1
MVVNTIHWFRKGLRLHDNPSLRDSIQGADTLRCIYILDPWFAGSSNVGINRWRFLLHCLEDLDASLRKLNSRL
FVIRGQPTDVFPRLFKEWHITRLSYEYDSEPFGKERDAAIQKLAKEAAVEVMVRISHTLYDLDKIIELNGGQS
PLTYKRFQALINHMDAVELPVETITSEFIKKCATPISEDHDEKFGVPSLEELGFETEGLITAVWPGGETEALM
RLERHLERKAWVANFERPRMNANSLLASPTGLSPYLRFGCLSCRLFYFKLTDLYRKVKKNSTPPLSLYGQLLW
REFFYTTATNNPCFDKIEGNPVCVQIPWDRNPEALAKWAEGRTGFPWIDAIMTQLRQEGWIHHLARHAVACFL
TRGDLWISWEEGMKVFEELLLDADWSVNAGSWMWLSCSSFFQQFFHCYCPVGFGRRTDPNSDYIRRYLPILRG
FPAKYIYDPWNAPEEVQKAAKCIIGVHYPRPMVNHAEASRINIERMKQIYQQLSCYRGLGLLATVPANPNNGA
SGSNVAGVSTGTSQGPGGFSEDQSIQEGTSRTERGQSAQKRRHEEAPSENSSKSWRQSK
>Lates_calc__XP_018522462.1
MDCPWCVYRVTDMAHTCIHWFRKGLRLHDNPALMAALRDCKEIYPVFVLDPYFHTNTRVGINRWRFLIGALKD
LDCSLRKLNSRLFVVRGKPEDVFPKLFSKWKITKLTYEYDTEPYSLSRDEKVTALAKEHNVEVIYKISHTLYD
IDRTIEENNGKAPLTYNRMQAIVKTLGPPKKPIPAPTLEDVKDVMTPCSENHEKEYGIPVLEELSQDTAALGE

ELFPGGEQEALRRLDEHMKRTGWVCNFEKPQTSPNSLSPSTTVLSPYVTFGCLSVRTFWWRLTEVYQGKKHSD
PPVSLHGQLLWREFFYTASVGIPNFNKMEGNPVCTQVDWDTNPEYLAAWREAQTGFPFIDAIMTQLRQEGWIH
HLARHAVACFLTRGDLWISWEEGQKVFEELLLDGDWALNAGNWQWLSASAFFHQFFRVYSPIAFGKKTDKNGD
YIKKYLPLLKKFPAQYIYEPWKAPRSIQQAAGCIVGKDYPHPIVQHEVISKKNIQRMKLAYAKRSPAPTESPS
KKQGEKRKAPSVVDMLKKKTKRT
>Lates_calc__XP_018529316.1
MSTSRTVICLLRNDLRLHDNELFHWAQRNAEYIVPLYCFDPRHYVGTYNYNLPKTGPFRLRFLLESIRDLRST
LLNKGSNLVVRRGKPEDVVAELIKQLGSVSTVAFHEEVTSEELNVEKRVKDVCTQMKVKVHTCWGSTLYHRDD
LPFHHISRLPDVYTEFRKAVEARSRVRPLFPTPEQLKPLPPGLEEGAIPTAQDLEQTEPLTDPRSAFPCSGGE
SQALARLKHYFWDSDAVATYKETRNGLIGVDYSTKFAPWLAMGCISPRYIYHQNKQYERERTANQSTYWVIFE
LLWRDYFKFVGAKYGNRLFQLEGLQNKSVPWKKDMKLFNAWKEGQTGVPFVDANMRELALTGFMSNRGRQNVA
SFLTKDXGLDWRMGAEWFEYLLIDYDVCSNYGNWLYSAGIGNDPRENRKFNMIKQGLDYDNNGDYVRQWVPEL
KEIRGSDVHTPWTLSSAALSHAHVSLGETYPSPVVIAPEWSRHVNKKPSGTGPSPRGKKGPSHTPKQHRDRGI
DFYFSRSKNL
>Lates_calc__XP_018532761.1
MLLCIWRGSSFSCAPRRLLRPVIHNHQRPRPIFFFLTKSFTSETIMSDKKRKAPSAAAGKEPSAKQQKLAPSK
EEKKERAAGWLQELVKQQRSEKKEMKFNKKRLRFISDTEKVKQGSEGVLYWMLRDQRVQDNWALIHAQQLAVK
ENLPLHICFCLAVPKSELSTLRHYNFLLKGLEEVAKECKALDIQFHLLHGSPGEVLPGFVSDRNLGAVVTDFS
PLREPIKWLEDVKKSLSKDIPLIQVDAHNIVPCWVASPKLEYSARTIRGKITNLLPEFLTDFPLVEKHPHTAT
RTAKAIDWDKTLASLQLDRIVGVPEWAKPGTKGGMATLESFIDVRLKLFATQRNDPNAAALSQLSPWIRFGHL
SAQRVALQVQRSGKNAGQSVPSFIEELVVRRELTDNFCFYNKKYDSVEGAYEWAQKTLKDHAKDKREYLYKRE
QLEKAKTHDKLWNAAQYQMVTEGKMHGFLRMYWAKKILEWTSSPEEALSIALYLNDRYELDGQDPNGFVGCMW
SICGIHDQGWAERPVFGKIRFMNYKGCTRKFDVAQFERKYCPKNL
>Lates_calc__XP_018559545.1
MVVNSVHWFRKGLRLHDNPALQEALNGADTVRCVYILDPWFAGAANVGINRWRFLLEALDDLDSSLKKLNSRL
FVIRGQPTDVFPRLFKEWKVTRLTFEYDPEPYGKERDGAIIKMAQEFGVETIVRNSHTLYNLDRIIEMNNNSP
PLTFKRFQTIVSRLELPRRPLLPVTLQQMDKCHIKIADNHDQLYSIPSLEELGFRTEGLPPAVWRGGESEALD
RLNKHLDKKVWVANLEHPRVSTCSLYASPTGLSPYLRFGCLSCRVLYYNLRELYMKLRKRCSPPLSLFGQLLW
REFFYTAATNNPNFDRMEGNPICVQIPWDQNPEALAKWAEGRTGFPWIDAIMTQLRQEGWIHHLARHAVACFL
TRGDLWISWESGMKVFEELLLDADWSVNAGSWMWLSCSAFFQQFFHCYCPVGFGRRTDPSGDYIRRYIPILKD
YPNRYIYEPWNAPETVQKAANCVVGVDYPKPMINHAEGSRLNIERMKQVYQQLSHYRGLSLLASVPTIQEEAE
PPMTDESQTSSGPDSPPRGPIDSEAAGCSTAPDSSTVCASSTCALHPDLEDTPSSNLSQTPCPSSGSQTQPSA
AVTSTSASHPTSTAAPPSPASTPVQSPLSKSEPSSPSSSCLTLSLSLTPATTLAQTFHKRKGFTRKVRRSQRQ
RGQQSCREGERRAKEEEREDTGEEERMEEDVEQNEEKMEEETSGKMAGHQQ
>Latimeria_chalumnae_XP_014343736.1_PREDICTED:
VRGQPADVFPRLFKEWNVTRLTFEYDSEPFGKERDAVIIKVARESGVEVIMRDSHTLYNLNRIIELNGHKPPL
TYKRFQAIISRMELPRRPVAAVTQQQMETCKTDIGNDHDEQYGVPSLAELGFQVENDPAIWQGGETEALARLE
KHLDRKAQVANFERPWINANSLLANPTSLSPYLRFGCLSCRLFYYRLLELYKKIKGNSSPPLSLYGQLLWREF
FYTAATNNPRFDRMEGNPICVQIPWDSNPKALAKWAEGQTGFPWIDAIMTQLRQEGWIHHLARHSVACFLTRG
DLWISWECGMKVFEELLLDADFSVNAGSWMWLSCSAFFQQFFHCYCPVGFGRRTDPSGDFVRRYLPKLKNFPS
KYIYEPWNAPYSVQKAAKCIIGVDYPKPIVVHAEASRLNVERMKQIYQQLSHYKGLCLLASVPAYTEDQGSLH
MESQACSSSAGAAVLQYSKTSLKREMVQEEEEGCRRSKLQFLHIPEKPSKDQ
>Latimeria_chalumnae_XP_014347518.1_PREDICTED:
MGASSIHWFRKGLRLHDNPALREAVQGASTVRCVYILDPWFAGSSNVGINRWRFLLQCLEDLDASLRKLNSRL
FVIRGQPANVFPRLFKEWKITRLTIEYDSEPFGKERDAAIKKLASEAGVEVIVKISHTLYDLDKIIELNGGHP
PLTYKRFQTLVSRMEHPEMPVEAITASFMGKCITPVSEDHHEKYGVPSLEELGFDTEGLPSAVWPGGETEALT
RLERHLERKAWVANFEKPRMNANSLLASPTGLSPYLRFGCLSCHLFYFKLTDLYKKVKKNGSPPLSLYGQLLW
REFFYTAATNNPQFDKMEGNPICVRIPWDRNPEALAKWAEGKTGFPWIDAIMTQLRQEGWIHHLARHAVACFL
TRGDLWISWEEGMKVFEDLLLDADWSVNAGSWMWLSCSSFFQQFFHCYCPVGFGRRTDPNGDYIRRYLPVLRG
FPAKYIYDPWSAPESVQTAAKCIIGVHYPKPVVNHAEASRLNIERMKQIYQQLSRYRGLGLLASVPSNHNGNG
NGGMMVYSPVEPTLGTNAAHGFSIASGHSNTSILLSYSNDEQPGPSGIQGWSNYGNANGNSSGKRDREPEQDG
STDDETHMALVKVQRKSNDRTEELQGINLASY
>Leishmania_inf_XP_001463490.1_putative_D
MRQVETDSEFLAAAQGLTPPLSDSPVGEVNQSAATAEDGSHGGCMLASKPPWGSPAPPLALPEDAAELATDLA
QEVGALYTDAGVTSARCWEQAEQEMEADSGRRDDGVEFGDDAERDEQLLASLKGRHVRLQNTPDVVMAPLIIP
DRNGRVAADAQALLHCRGGESEASALEVAPRITARLLHRHSHLAEQDRLYLGPCTAPEAQLRPPDVMRAAPPS
SGTQRHGCCVLVVFSSTDLRVHDNQLLAFASVCARAAAAEAGSPVPVIGVCVLDYRTFAQPSTVGGFFRQSPQ
RAQFLLDTVAALRRKLEDTLHVPLLVRCGRPEEHVPRLAVELGAMDVLMTTQYAPHERRVQALMVRRLRAGTW
VSREEVSDEAVVAGAVGQGASATPVGAQCGSAAEEDDPLIAVVEHASCGVGGQHPYHCGSSLPLHCRAAAAPP
MVHSVWQSTLVHLDDLPTPLTVMKEGERWYHDDVTVSTIRPTEPYDKATALLAELPLTWQAAALLPTEDERYG

RAGPSVLRGALPRLEDLGYSAAAARGTDFAFQEVIATQSSHPDAGEDAALARLQNWLAQGGVTSLLRYGRERR
TNTKMYSQKLARVSPYIALGALSPRKYYEVLREFAQENQRDAFVQQQFREGLLRLSRRDYWHWMGLRFGDRLF
FSYGPHPEHTDDVPEWRHDRKVVQRWCDGLTGIPFADAAMRELVGTGFVAQEGRQALAWLLTRGYGQDWRVGA
EWMERCSLDYDPFVCYGNYAYSCGLMLDDFGEPVRNVYYLAHQHDQTGIYVKKWLPQLSKVPPVYIHRPHVLT
ERMQAMHGVYLGKNYPYPLKLWQGAQRSLSAAELTAYYPQGIVKGPGYAEALRYGSAVMQPEEYNAAVSPAYL
QRQEWATMLPASAFAGIEDSDEMAKHFSLLEAAAPRKSAPPAAVVAGGSTLKRVVA
>Leishmania_inf_XP_001468129.1_putative_D
MKRGRSRSSSTGSAAAEKRSHTVREEVALFLFRRDLRVVDNTGLQALCDEAARRFIPVLPAFFFNPIQCDKKR
NPYFGDAFFQFFCESLIDLDGAAQLNGGLVCLRGSDEDCLQRIRDSGYEVKVLGFNEDYTPFALARDRLLRAY
ADKQGIVCVTGPHDYSLRPLDEVVKNSEQPYSVFTPFYNKFTAEHARRVAVPLPVNVSKVQSMLVSRPKKCMG
HHLVDPALVYAHMPQVQDHGGRTEGLKRLACVERLKQYADARDDIAGDRTSHLSPHMKCGTVSTREVWHASVQ
ALGTGHPFTRQLVWREFYAMLAFTRPRLLQGQLNSFIGQQDIVKATQPKQNAPFQPSYDNFKWSWKAEHFEAF
KEGRTGVPLVDAAVRCLTATGWCHNRCRLVISNFAVKVLGIDWRECERWFATVAVDYDAANNNGGWLWSSGQG
ADAQPYFRTFNAFRQSERFDPDCKFIFQWVPELAKVPPSVVHHWEGYCARARGKATATHSRKRSGQGNAEEYP
TSYPAPIVDIKAATKAVVEEFKKYSKYKEN
>Lepeophtheirus_sal_XP_040563574.1_cry1-like
MTDQRKSHVCWFRNALRLHDNPALTEALKGSDEESTGFIPLFIFDGTSNGTDLIGYNRMSYLLESLKDLDEQF
KKFGGSFIIARGSPIQVLKEIHSKNPIMKITWEQDPENKGQKRDNKVRDFCANAKIKAIEKQGHTLFDPWHII
AANGGIPPTTFTTFSKIVDCIGVPNRPVPNADLSHANFISSNFSFSFDKFPTPEDLNIFPHSTFDDTKKVYMG
GELKALEDLERRMTHEIDHFRKGRYLPNRRSPDILCPPKSLSPDLRFGCLSVRKFYWGIIDANWEFQKAVGLN
IEINHQIVAPLLWREFFYTMAAKNQYFTEIQRNPMCIPIPWTSTTDNKQFDAFVKGKTGFPFIDAGLRQLYSQ
GWIHHVVRNAITCFLTRGDLWISWEEGFKLFFKYLLDADEAVCAGNWMWISNSAFEEVFNCQHCIDPVNFGRR
HDAHGEYVKRFIPELKSMPLEYIYEPWKAPHDVQVNVGCIIGEHYPSPVVNHKQISKLNTLRMKKLQLEFSHT
RSNPRPRSEYLHPSNSTEAIKFMSFERNCRTCPSNKSI
>Lepeophtheirus_sal_XP_040565695.1_deoxyribod
MPNSIFWFRRDLRLGDNPGLVEAAKLGKVLCVYIFEDRKQNAADFSELGSGTKAWLHYALDSLNKTLGGKLFL
ARGNPLDVLKDVAQKASSKNVFCNHIYEPMEMEMESHIIKELKSSAGITLRSFNSSLLWEPQNILQQNGKPYK
IFTHFYKKGCLNYGEPRSPLVEPSNLNLLQLNDTLTLEDLQLLSKDSPNLTVNEWNISEKGANERLESFLQNG
LDNYKIGRDFPSQHHVSRLSPYLRFGQISPNIIWYAARKVGTPGPDLDHFSSELGWREFSVYLLHHWPHIPRE
SFNPNFKGFPWKDDEKALAAWKEGRTGVPIVDAGMRELLQTGYMHNRLRMIVASYLVKNLMIHWHRGEDWFWD
KLLDADLASNSASWQWVVGCGADAAPYFRIFNPILQSEKFDPEGVFIRKYVPELTNVPTKHLFAPWEATDTVM
KNAGVIMGKTYPRPLVDFKKSRQAAQDAFDKMNNKG
>Lepeophtheirus_sal_XP_040573653.1_(6-4)DNA_p
MTTGVWFRNALRLHDNLPLIEAAKSSNPVICFYIFTPAVYNPDYMGMNRLYFLLQSLEDLKNRLKKQYKNGDL
IILNTSKSCKDVFSQLCKADVLKEVFYEYDSSPHGKGRDTQIQKEIHQVSFKVFHGHTFTKLPDVVAQKGFKN
PSTMSMMENIMIKEFGKGKGRSGINVPSPLDPPDEINFDISFLKKIKKRCPELEILEEVPKLKDLSKIFGPLF
DNVNVTSIEKNNFFHGGETEALERLERKVLDNTEFVCKFKKPETTCITTSASEFKEPSTTGLSPYIALGCLSV
RQLWHGVDQAYAKRKGFKIDSLATSLYGQLLFREMFYILNEAIGPVFALNDKNNPAISKYVEWDKKDPKLLEA
WENGQTGYPFIDALMRQMKSTGWMHHLGRHAVSCFLTRGQLYQNWTYGRDVFDRELVDSDYALNTGNWLWLSG
VATFSMPYFRVYSPCPTEDKKSALNFNAGGNDFIRHWVPELKKFPAKFLTKPWECPKMAQTSCGVIIGKDYPM
PIVPLKNDNLDKFKKSLQDNKSSSGPPNKKKKI
>Lepeophtheirus_sal_XP_040573654.1_deoxyribod
MESPMKKPKLNGDPFATERLEAYPSPMDFKFNEKRVKLLSGSENYSQRGKKPCIVYYMHRDQRVQDNWAFLYA
QRLALKHKLPLHVLTLISEKHPDHPGMTLRNLTFWLDGLREVSTELKTLNIPLHLLINQSLDESPGTSIVQWL
GMMSDVQALIVDFSPLRQHRKIVRDIIESIKTPDLPLYRVDAHNVVPVEVTSDKQEYAARTIRSKINKKLDEF
LTIFPPIIHHPHGVLSETEIHFGNQIGKNIEEDWSTLLNSLLIDHSVGPVDMYKGGSRAGFECLESFVTKRIQ
RYTDKRNDPNEDVLSRLSPWFHNGQISVQRAVLYVKKVANKYSSSFVEESIVRRELSDNFCYYNENYDSVKGA
ADWAQKTLSEHKKDKRTHIYSRSKLEKAKTHDDLWNAAQNQLVTEGKLHGYMRMYWAKKILEWTESPDVALAD
ALYLNDHYALDGNDANGFVGCMWSICGIHDQGWRERSIFGKIRYMNYDGCKRKFNIGSYINRYNPKNKNGFFK
PKLS
>Lepisosteus_oculatus_XP_006643183.1_PREDICTED:
MVVNSVHWFRKGLRLHDNPALQEALNISDTVRCVYILDPWFAASANVGINRWRFLLESLEDLDASLRKLNSRL
FVVRGQPADVFPRLFKEWNVTHLTFEYDSEPFGKERDAAIMKLARESGVETIVQESHTLYSLDRIIELNNNSP
PLTFKRFQAIVSRLGLPKKPLGTVTPQQMERCHTPISENHDECYRVPTLEELGFKTHGLSPSIWKGGETEAVR
RLNKHLERKAWVASFERPKINVYSLIASPTGLSPYLRFGCVSCRLFYYSLLELYKKVRKSSSPPLSLFGQLLW
REFFYTAATNNPKFDQMEGNPICVQIPWDRNPEALAKWAEGRTGYPWIDAIMTQLRQEGWIHHLARHAVACFL
TRGDLWVSWESGMRVFEELLLDADFSVNAGSWMWLSCSAFFQQFFHCYCPVGFGRRTDPSGDYIRRYIPKLKE
YPNRYIYEPWNAPEIVQKAANCVIGVDYPKPMINHAQASRLNIERMKQIYQQLSHYKGLSLLASVPSVPDDAR
SPVTDDPHGHSSSAPCSRHHSKNTSWRKRERPSDLESEDKCSRPKVQRPCVTGSRANDKP
>Lepisosteus_ocu_XP_006628580.1

MTHRTIHLFRKGLRLHDNPTLLGALESSAVLYPVFILDRAFMEEAMARGVLRWRFILQSLQDLQRSLAALGSR
LYVVEGRYDQVLREKVRQWGITQVTFDTEVEPFFTRLDDDIRALGQEMGFSVQSCVAHTLYDVQRIIKANGGE
APLTYKKFLHVLALLGAPETPARRITREDFRQCRTPTEEESEEKYRVPSLEDLGIVVESEALWVGGETEGLQR
LEQHMQNQGWIENFTKPRTIPNSLLPSTTGLSPYFSLGCLSVRMFYHRLSNIYAQSKNHSLPPVSLQGQVLWR
EFFYTVAAATPNFTRMAGNPICLQIDWYKDQEALDRWKTARTGFPWIDAIMTQLRQEGWIHHLARHAVACFLT
RGDLWISWEEGMKVFEEHLLDADYSVNAGNWMWLSASAFFHQYTRIFCPVRFGRRTDPEGHYLRKYLPVLKNF
PSQYIYEPWTAPQQVQLEAGCIIGKDYPLPMVNHREVSESNLALMREVRREQEKTAQLTRDTADDPMEVGKKR
ACQREAETDRSDAFLEKPERAKRFSHAEGEAKALACSWPSETLRLPALGREVM
>Lepisosteus_ocu_XP_006634324.1_cry_DASH-l
MSTIRTIICLLRNDLRFHDNELLHWAQGNAEQIVPLYCFDPRHYLGTHCYNFPKTGPFRLRFLLESVKDLRDT
LKKNGSNLLVKRGKPENVVSDLIKQLGSVTAVAFHEEVTKEEQDVETEVTRVCAQFKVRVHTCWGSTLYHRED
LPFNHIARLPDVYTQFRKAVETQSQVRPTIRMPEQLKPFPHNLEEGPIPTPEDLGQPNAVSDPRTAFPCIGGE
TQALARLKHYFWDTNLVASYKETRNGLIGMDYSTKFAPWLALGCISPRYIYEQIKQYEKERTANQSTYWVIFE
LLWRDYFRFVAVKYGNRIFTVKGLQQKSVSWKKDVKLFDAWKEGKTGVPFVDANMRELALTGFMSNRGRQNVA
SFLTKDLGIDWRMGAEWFEFLLVDYDVCSNYGNWLYSAGIGNDPRENRKFNMIKQGLDYDNNGDYVRLWVPEL
RGLKGGDVHAPWVLSSASLADAEVSLNQTYPVPMVVAPEWSRHTTSKASGGGLSQKGKRGPSHTPKQHRDRGI
DFYFSRNNKKLQ
>Lepisosteus_ocu_XP_006642290.2_cry-2-like
MDSLTGFGWKHLPGARPMMHRSIHWFRKGLRLHDNPALLASLRDCAELWPVFLLDPWFPKNARVSVNRWRFLL
RALQDLDGNLRKLGSRLFVVRGSPAEVFPRLFEQWKVTRLTFEVDTEPYARQRDAQVGKIAEEHGVEVIQKVS
HTLYDTERILVENNGKAPLTYNRLQALLKTLGAPKRPVPPPTAEDMKGVCTPCSERHDEEFGVPTLEELGQDP
RTAGPELYPGGETEALSRLDRHMQRTAWVCGFQKPNTEPNALSPSTTVLSPYLKFGCLSARTFWWRLTDVYRG
VRVSKRVLSLHGQYSRAEFSYPSENGLSQAACSSGWLVTSSAAARSVPQVSVRVCVSQGCTGFPFIDAIMTQL
RSEGWVHHLARHAVACFLTRGDLWISWQEGQKVFEELLLDADWALNAGNWQWLSASAFFHQYYRVYSPIAFGK
KTDKNGDYIRKYLPVLKKFPSAYIYEPWKAPRSVQEQAGCIVGKDYPRPIVDHDVVSKKNIQRMKLAYARRAQ
LGGEQEGTGKGMKRKGQSVADLLTKKQKRNSVEEKMTLSG
>Lepisosteus_ocu_XP_015207477.1_cry-1
MVVNTVHWFRKGLRLHDSPALRESLKGAASLRCVYILDPWFAGSSNVGINRWRFLLQCLEDLDASLRKLNSRL
FVIRGQPADVFPRLFKEWNINRLSFEYDSEPFGKERDAAIKKLASEADVEVIVRVSHTLYDLDKIIELNGGQP
PLTYKRFQTLISRMDPLETPAEPITAEVMGKCTTPVSDDHDEKFGVPSLEELGFDTEGLPSAVWPGGETEALT
RLERHLERKAWVANFERPRMNANSLLASPTGLSPYLRFGCLSCRLFYFKLTDLYKKVKKNSSPPLSLYGQLLW
REFFYTAATNNPRFDKMEGNPICVQIPWDKNPEALAKWAEGRTGFPWIDAIMTQLRHEGWIHHLARHAVACFL
TRGDLWVSWEEGMKVFEELLLDADWSVNAGSWMWLSCSSFFQQFFHCYCPVGFGRRTDPNGDYIRRYLPVLRG
FPAKYIYDPWNAPDSVQKAAKCVIGVHYPKPMVHHAEASRLNIERMKQIYQQLSCYRGLGLLASVPSNPNGSG
GAGMTGFSSGEGLHSSNAAGTSAGGVEGQAVTVLSYSHGESHRGLQQQGYQIPSGRSSLPSYSSGDSQAGSNL
LPQGYTRGSTVVHYTQGDSQQTSSVILQQGRGHLPSVLTTVKRPSPEESTQGIVPKVQRQSSN
>Lepisosteus_ocu_XP_015216926.1_photolyase
MSGRSPPSPQGGAAGKGAAKRKLQGGEGTGEKKAKKTAPEQGAGWLAEAVAQSRRQAGLSVQGFGFDKKRMRV
LSKASAMKGGSEGVLYWMSRDQRVQDNWALLYAQQLALEEQLPLHVCFCLVPCFLDATIRQFGFLLRGLEEVA
KECSALGLEFHLLRGSAGEVLPQFVQKQGLGAVVTDFAPLRLPLQWVEEVKERLPQDIPLIQVDAHNVVPCWV
ASDKQEYSAKTIRKKITSKLPDFLKEFPLVAEHPHRASKPAKPIDWEQARSSLAVDRSVKEVSWARPGAAAGL
EVLESFIDHRLKDFATQRNNPNGEALSNLSPWLHFGQVSAQRVVLEVRRHGRRWPQSVDVFVEEVVVRRELAD
NFCFYNKKYDRVDGAYEWAQKTLREHAKDRRPYLYSRGELEASRTHDKLWNAAQFQMVSEGKMHGFLRMYWAK
KILEWTASPEEALATAIYLNDRYELDGRDPNGYVGCMWSICGIHDQGWKEREIFGKVRYMNYAGCTRKFNVPK
FEIKYNPKRCDDEDGNRKRGGD
>Lepisosteus_ocu_XP_015223435.1_cry-2-like
MQGGHFALVYPFAPQAIRWSADRAHLKAWQRGRTGYPLVDAAMRQLWLTGWMNNYMRHVVASFLIAYLHLPWQ
EGYRWFQDTLVDADVAIDAVMWQNGGMCGLDHWNFVMHPVSAALTCDPYGSFVRKWCPELAALPDELIHKPWQ
CPASVLRRAGVTLGGDYPERIVADLEERRARSLRDVATARQQFAGEYVDRRSGCDLLPLPDKLVKEALGGGGG
GEVVQRGGRFLLPVITRKEFQHQTLEPGAQTNPFDAVLRGYVSRQRDEAAAFLRERDFTASVMSEGGRRLERQ
ERDRRVLEGLPPPPAQKSRARRTPRTDPFSVVPGGAPITPS
>Leptinotarsa_dec_XP_023017756.1_cry-2-like
MSGSTGQTSTGQEKHTVHWFRKGLRLHDNPSLREGLTGAKTFRCVFVLDPWFAGSSSVGVNKWRFLLQCLEDL
DRSLRKLNSRLFVIRGQPADALPKLFKEWGTTVLTFEEDPEPFGKVRDHNITALCRELGITEFFYFSITHVVS
GLNPPVWQGGESEALTRLERHLERKAWVASFGRPKMTPQSLLPSQTGLSPYLRFGCISTRLFYYQLTDLYKKI
KKAFPPLSLHGQLLWREFFYCAATKNPNFDKMHGNPICVQIPWDKNVEALAKWANGQTGFPWIDAIMTQLRQE
GWIHHLARHAVACFLTRGDLWISWEEGMKVFEELLLDADWSVNAGMWMWLSCSSFFQQFFHCYCPVKFGRKAD
PNGDYIRKYLPILNNMPLQYIHEPWLAPDSVQQAAKCIVGKSYPLPMVNHTNASRINIQRMKQVYQQLANYKV
METARYPVNTGYSETFQRQPSAVTVGNPDNGQAA
>Leptinotarsa_dec_XP_023024919.1_photolyase

MASLKPRSSSGKVVLEKLTKELFLKNIVESRESQGGTDEFDFNKTRCRVLTDNETVKDKSNGILYWMYRDCRI
QDNWAMVFAQRLAIKQKVPLFVCFTVKDAHQQYPTMRHFKFLIEGLKLLKKECESLNIGFYLLNSAPKDLAKS
IVDNNIGGVICDFSPLKHPKKLQEVLLEHLPDDVPVVQVDAHNIVPVWKASDKQEGMAKFLRTKITKQLPEYL
TGFPIIRRHKYSGKPNFKNEIESFDSAYSHYTPKWDVPEIKWGDGPGEKAGYSMLLDFILKNLRHYGATSNDP
SKDNSSKLSPWINFGQISAQRCALEVKSVDSIYKEQCDKYLEELIVRRELTDNYCFFNSNYDNINGAANWAKE
TLKLHSKDKRTWIYTREQLEKAETHDEMWNSAQLQAHHEGKIHNYMRMYWCKKILEWTESPEQAIEYGLWLND
TFCLDGTDPNGYVGVMWSICGVHDQGWREREIFGKIRYMVDYSLRRKYNMDAYCARFGRKILGDSKTKAAIIP
KEPKESKGKNETKKGKGIKRKAT
>Leptomonas_pyr_XP_015655211.1_putative_p
MAEVKRNRSRSPSSGTVRCEKKPRTEREEVALFIFRRDLRLVDNTGLQGLLDEAAQRSLRVLPAFFFNPIQCD
KAQNKYFGDSFFQFFCQSLEDLDGASQLNNHLVCLRGSDEECLQQVRRGGYDVTVLGYNEDFTPFARARDQLL
SAYADDHGVTCVVGRQDYTLRPLGEVVSGAERPYSVFTPFFNKFLCDHASRVAKPIATDVERVQSMLVRQPKK
YLEEYLVDPTRLCTHNPSIVEKGGRTEGLRRLANIKSMTNYGSVRDDIAHDQTSHLSPYMKCGAVSTREVWHA
SVQAVGLAHAFTRQLVWREFYAMLLHHHPRLAQGQLNAFIGQTEIVATTRPQHNAPFQAKYEDFQWSWKPQHF
DAFREGRTGVPLVDAAVRCLTATGWCHNRCRLVISNFAVKVLAIDWRVGERWYATVAVDYDVANNNGGWLWSS
GQGADAQPFFRTFNPFRQSERFDPDCEYIYKWVPELKNVPPEVVHSWDTYCAKLEHQKKGRGSKGSQKGKVKE
YDTAYPPPLVDIAKARTAVIDKFKSYTPKK
>Leptomonas_pyr_XP_015655956.1_putative_m
MLDTVAALRAKLEGELRVPLLVRCGRPEEHVPRLAAELGATDVFMTTQYAPHERRVQALAMRRLRQGVWVSRE
DVCGGARVTSAAAADPVGDDDALVAVVEHHTSQQHPYGGAAAVVAPPVVHSVWQTTLVHLDDLSTPLAAMKEG
ERWYHDDVTVSTIRPTAPYDSATQRLAELSTTWQHDALLPSQEERAGRSPPSVLRGALPTLEDLGYAADRADL
AFEEVIATESSHPAAGEEAALARVQDWLAEDGMSSLLRYGREHRTNTKMYSQKLARVSPYIAVGALSPRKYYE
LLRVHTHANMRDGFVQQQFREGVLRLSRRDYWHWMGLRFGDRLFFSYGPHPEQTDEVPDWRHDAKVVQRWCNA
LTGIPFADAAMRELVGTGFVAHEGRQALAWLLTRGYGQDWRLGAEWLERCSLDYDPFVCYGNFAYSSGLVLDD
FGEPVRNVHYLAHHHDQTGIYIKKWLPQLSKVPPVYIHRPHVLTARMQAIHNVHLGLNYPYPLKLWQGAQRTL
SAAELPSYYAQGIQRGPGYAEALRYGSAVMPPEELHAAVSPAYLRHRTWASMLPASAFAEGGEAGEEGGEAMA
VEMGAKSRVAALAGTQAAAMAKSSAGVHGSEKALV
>Leptosomus_disc__KFP99119.1
HLAYFVSRIIELNGHKPPLTYKRFQAIISRMELPKKPVSTVISQQMEICKVDIQENHDDVYGVPSLEELGFPT
DGLAPAVWQGGETEALARLDKHLERKAWVANYERPRMNANSLLASPTGLSPYLRFGCLSCRLFYYRLWELYKK
VKRNSTPPLSLYGQLLWREFFYTAATNNPKFDRMEGNPICIQIPWDRNPEALAKWAEGKTGFPWIDAIMTQLR
QEGWIHHLARHAVACFLTRGDLWISWESGVRVFDELLLDADFSVNAGSWMWLSCSAFFQQFFHCYCPVGFGRR
TDPSGDYVKRYLPKLKGFPSRYIYEPWNAPESVQKAAKCIIGVDYPKPMVNHAETSRLNIERMKQIYQQLSRY
RGLCLLASVPSCVEDLSGPVTDSASGQGCSTSTAMRLSQADQASPKRKHEGAEELCTEELHKRAKVTDLPAPE
IPGKS
>Leptosomus_disc__XP_009946273.1
NWAFLYAQRLALKQELPLRICFCLVPKFLDATIRHYSFMLRGLQEVAKECTELNIPFHLLLGYAKDVLPTFVV
EHGVGGLVTDFCPLRLPRQWVEDVRERLPEDVPFAQVDAHNIVPCWVASPKQEYSARTIRGKIHAQLPEFLTE
FPPIVHHPYSPSCPAEPIAWEACYSSLQVDHTVKEVEWATPGTAAGLAVLQSFIAERLKSFGSHRNDPNKAAL
SNLSPWFHFGQVSTQRAILEVQKHRGKYKESVDTFVEEAVVRRELAENFCYYNENYDSVQGAYDWAQTTLKLH
AKDKRPFLYKLQELEQGTTHDPLWNAAQLQMVREGKMHGFLRMYWAKKILEWTHSPEEALQFAIYLNDRYELD
GRDPNGYVGCRCLWSICGIHDQGWAERAIFGKIRYMNYAGCKRKFDVGQFERRYAPRMLSQ
>Leptosomus_disc__XP_009951143.1
MSHRTIHLFRKGLRLHDNPTLLAALESSEVFYPVYILDRKFMTSVMHIGALRWHFLLQSLEDLQKNLCQLGSC
LLVIQGEYEPVLRDHVQKWNITQVTLDAEMEPFYKEMEANIRRLGDELGFEVLSLVGHSLYDTKRILDLNGGA
PPLTYKRFLHILSLLGDPEVPVRNLRAEDFQRCRAPDPGLAECYRVPLPVDLKIPLKNLSPWRGGETEGLQRL
EQHLMDQGWVASFTKPRTIPNSLLPSTTGLSPYFSMGCLSVRTFFYRLSNIYAQAKHHSLPPVSLQGQLLWRE
FFYTVASATPNFTKMAGNPICLQISWYEDAERLHKWKTAQTGFPWIDAIMTQLRQEGWIHHLARHAVACFLTR
GDLWISWEEGMKVFEELLLDADYSINAGNWMWLSASAFFHQYTRIFCPVRFGKRTDPEGQYIRKYLPILKNFP
SKYIYEPWTASEEEQKQAGCIIGQDYPFPMVNHKEASDHNLQLMKQVREEQHRTAQLTRDDTDDPMETRVKRD
HSEENIKGKVARVTEHSEIPSGIPCWEPTNGTRGEPEAN
>Leptosomus_disc__XP_009956042.1
VLHWAQSNADFVIPLYCFDPRHYLSTHCYGFPKTGPHRLRFLLESVKDLRETLKKKGSTLVVRKGKPEDVVRD
LITQLGSVSAVAFHEEATQEELDVEQGLCQVCSQHGVKIQAFWASTLYHRDDLPFRPIARLPDVYTHFRKAVE
SEAKVRPTLRMADQLKPLAPGVEEGCIPTMEDLGQKDTVTDPRTAFLCSGGETQALMRLQYYFWDTNLVASYK
ETRNGLVGMDYSTKFAPWLALGCISPRYIYEQILKYEKERTANESTYWVLFELLWRDYFRFVALKYGRRIFSL
GGLQSKEVPWKNDLQLFDCWKEGKTGVPFVDANMRELAATGFMSNRGRQNVASFLTKDLGLDWRMGAEWFQYL
LVDYDVCSNYGNWLYSAGIGNDPRDNRKFNVIKQGLDYDGSGDYIRLWLPELRGIKGAAIHTPWALNSAALSQ
AGVTLGDTYPQPVVTAPEWSRHVDQRPQGRGPHPRGRRGPAHTPTQHRGTEFYFSRQKDAQ
>Leptosomus_disc__XP_009960183.1

FLLQCLEDLDANLRKLNSRLFVIRGQPADVFPRLFKEWNIAKLSIEYDSEPFGKERDAAIKKLASEAGVEVIV
RISHTLYDLDKIIELNGGQPPLTYKRFQTLISRMEPLEMPVETITPEVMEKCTTPVSDDHDEKYGVPSLEELG
FDTDGLPSAVWPGGETEALTRLERHLERKAWVANFERPRMNANSLLASPTGLSPYLRFGCLSCRLFYFKLTDL
YKKVKKNSSPPLSLYGQLLWREFFYTAATNNPRFDKMEGNPICVQIPWDKNPEALAKWAEGRTGFPWIDAIMT
QLRQEGWIHHLARHAVACFLTRGDLWISWEEGMKVFEELLLDADWSVNAGSWMWLSCSSFFQQFFHCYCPVGF
GRRTDPNGDYIRRYLPVLRGFPAKYIYDPWNAPESVQKAAKCIIGVNYPKPMVNHAEASRLNIERMKQIYQQL
SRYRGLGLLATVPSNPNGNGNGGLMGYSSGESISGCGSTGGAQLGTGDGHTVVQPCALGDSHTGASGIQQQGY
CQASSILHYAHGDNQQSHLLQAGRTALGTGISAGKRPNPEEETQSVGPKVQRQSTN
>Leptotrombidium_del_RWS31519.1_photolyase
MNSPFVRKISSFNLCTRFFTMSNKGRKRKANYSDTESLRVNIKEECVSDEVSSIHDSRTNIAESVEHFNFNKK
RVRILTKSKEIKENCECVVYWMSRDARVQDNWSMLYAQKISLKFKVPLRVCFCLVPTFLNATSRQYHFLIEGL
KEVERECIALNIPFHLLLGQPPQVLTHFVEKHKTGAVIADFSPLRIMKKWINELTENFPNDVPICQVDSHNIV
PCWVASDKLEYAARTIRKKINDKLPEFLTYFPPVIRSPFQCEICSNDWQKAELSLDVVKSDKVNWAIPGTTAG
LQMLKSFCETRLKGYATLRNNPNYDNLSNLSPWFHFGQISVQRAILEVKKYQNKFNDSVLAFIEEAVVRRELS
DNFCYYNENYDNLNGAWQWAQDTLKKHETDKREHIYSKEQLENAETFDPLWNASQIQLVKEGKMHGFLRMYWA
KKILEWTNSPSEALEISIYLNDKYNLDGRDPNGYVGCMWSICGVHDMGWKERPIFGKIRYMNFEGCKRKFNVN
EFINKYKK
>Limulus_pol_XP_013787452.1_CPD
MSPKPIKRKQVDEAGTAHKIQKSQPFKETELSSNILKDVEEARRKCAESVSEFNFIEKRARLISSSKDISKNC
KGIVYWMSRDQRVQDNWALLYAQKVALAVGCPLYVCFCLVPKFLDATIRHYQFILKGLQEVEKECVSLNIGFH
LLLGEAYKVLPNFIKKNQIGAVVVDFSPLRVPLKWVNDVKQMLPSEIVFWQVDAHNIVPCWEASEKLEYGART
IRRKIHDKLSTYMTEFPPVICHPHPCLKSQPVDWKAAEASLLVDQSVKEVKWATPGTKEGLKMLFNFCNIRLK
NFSDSRNDPTKNSLSNLSPWIHFGQISVQRCVLTVKQFRNKYSKSVDAYIEEAVVRRELADNFCFFNPKYDQV
EGAYDWAKTSLKLHAKDKREYIYNKEQFEKGQTHDPLWNAAQFQMVREGKMHGFLRMYWAKKILEWSKSPEEA
LEIAIYLNDKYELDGRDPNGYVGCMWSICGIHDQGWAERPVFGKIRYMNYQGCKRKFDVDAFIRKYPVKEKA
>Limulus_pol_XP_022247971.1_cry-2-like
MERNKYYRFFIPLFNFILLLMNSQLVFSNEFYLLESDNIQEDHFEKDERGNMFWDFGISGFIGRIKTVLLGIF
SFFGKDMKETSVHWFRKGLRLHDNPALMKTLEKCGNFRPIFILDPWFVKNMHVGPNRWRFLLQSLKDLDNSLN
KLHSRLFVIRGKPLEVFPKIFQQWNVKTLTYEVDTEPYAIERDEKVDQLAKRMGVTVKKEVSHTLYDIERLVM
KNNGSAPHTYQAMLTMLKKIGPPPKPVASLDSLKGCSTPVEENHDEKYNVPDLKELGIHESDCGPNLYSGGET
EALRIMNEKLSNKSWVCKFEKPKTQPNSLEPSTTVLSPYLKFGCLSVRTFYYRLLDVYNKYTGNCTKPPVSLE
GQLLWREFFYTVGYATPNFDQMKGNPLCKQIPWSNNPEFLEAWTKAQTGFPFIDAIMTQLRQEGWIHHLARHA
VACFLTRGDLWVSWEHGMKVFEEFLLDADWSLNAGNWMWLSASAFFHQYFRVYSPVAFGKSMDKNGDYIRKYL
PVLKKMPSEYIYEPWKAPLKVQQQIGCVIGKDYPKPIVDHDEARKSNLEKMNKAYKENKTSNEQKTTNKRSSE
ETVSSLLSPKKTKS
>Limulus_pol_XP_022256597.1_cry-1-like
MSAPQTKNLVHWFRRGLRLHDQPALLEGLAGCTSFRCVFILDPWFAGSSNVGVNKWRFLLQCLEDLDASLRKL
NSRLFVIRGQPADVFPRLFKEWNVTHLTFEEDPEPYGRVRDHSITTMAQELGIKVICRTSHTLYKLEKIIEKN
GGNAPVTYKEFQNIVASMEPPPGPKPPVTAETLGRAFSSISDDHDEKYGVPTLDELGFDTETLKPAVWQGGET
EALARLERHLERKAWIASFGRPKMTPQSLLPSQTGLSPYLRFGCLSARLFYQQLADLYKRIKKANPPLSLHGQ
LLWREFFYCAATSNPNFDRISNNPICVKIPWDVNPEALAKWANGQTGFPWIDAIMTQLREEGWIHHVARHAVA
MFSNSGRLVDILGRRHEGI
>Limulus_pol_XP_022257009.1_LOW_QUALIT
MFHLSKIQKLFGKVPVLLQLLVLKTYWNNKLWTGGESRALQRLEXRLIVERAAYKDGHYLPNQFNPDLLGPPM
SMSAALRFGSLXVRKFFWSIHDLYEEIHPNVVPPLGFMGQLMWREYFYTVSVNNLHYDTMTESPICLNIPWQR
NDEHLRLWELGKTGYPFIDAAMRQLRREEWIDHVARNAVACFLTHGDLWISWEDGLNVFLKYLLDADWSVCAG
NWMWVSSSAFENILQCTYCISPIHYGHRLDPTGEYIRQHVPELKNMPTKYVFQPWKAPLEVQQQAGCIVGRDY
PEPLVHHQIVSAKNREYMNRIRDRLLGKPVLPHCGPGSHSETRIFMWLPDKCLDNVHPCNL
>Lingula_ana_XP_013382193.1_photolyase
MCMLFSLRNYCAFQLKFYVRTRHIHLASIMSGKENTKKRKGNEEGPVSKKMKVKVEDDAEAGPSSQNSAGVKF
TDTSKDFLELINHAREAKAQSVSAFKFNKKRVRMLSTAKEFPEECDGVIYWMSRDQRVQDNWAMLYAQRLALK
MEVPLHVVFCLVPKFLEATMRQYGFMLKGLQEVEQECGRLSITFHLLTGYAKEVLPDFVKCNNIGGVVTDFSP
LRVPKQWVKDVTENLPKNIPLCQVDAHNIVPCWEASDKREYGARTIRNKIQSKLPEYLTEFPPVIRHPYAAKT
KAEPVDWSAAEASLQIDRTVTEVKWASPGTTAGLRTLETFCKERLKFFNADRNNPNKNVCSNLSPWIHFGQVS
AQRCALVVKQFKSKFKEGGDAFLEESIIRRELSDNFCYYVENYDSIECAYDWAKETLQKHASDKREYVYTCKQ
LEEGKTHDDLWNAAQLQMVKEGKMHGFLRMYWAKKILEWTTSPEEALKISIYLNDRYELDGRDPNGYVGCMWS
ICGIHDQGWGERPVFGKIRFMNYQGCKRKFDVPAFVRKYGGKKH
>Lingula_ana_XP_013384159.1_cry-1-like
MSSSRTHKTLHWFRKGLRLHDNPALLAACTKDCAEFRPIFILDPWFVKNARVGVNRWRFLLQALKDLDDSLKK
LKSRLFVLKGKPHEVFPKIFKEWGITRLTFEVDTEPYARVRDAEIIKLAKEHNVEVIQKISHTLYDTEKVIAR

NGGSAPLTYQRFQTVLSQLGAPPRTAETLTSEHLKGCITPVTADHDQDYGVPTLKDLKVDTADMGEVVFPGGE
TEALKRMERYLKKSNWICNFEKPKTEPNSLEPSTTVLSPYLKFGCLSARLFWHRLQEIYKQCKKHSQPPVSLH
GQLLWREFFYTAGLGVANFDRMEGNPVCRQIPWENKPEYIEAWTHARTGYPFIDAIMTQLRQEGWIHHLARHA
VACFLTRGDLWVSWEEGQKVFEELLLDADWSLNAGNWQWLSASAFFHQYFRVYSPIAFGKKTDPQGEYIKKYL
PMLKKFPKEYIYEPWKAPRAMQEKAGCIIGKDYPRPIVDHGEISKVNIKKMAAAYALNKDQNATEKGTPKKRK
SEADANEKSKSKKPKKGSSEKITNFLEK
>Lingula_ana_XP_013384752.1_cry-1
MGKQDKKEGVSIHWFRHGLRFHDNASLQEALKDVKECYLIFIFDGSVAGTDTAGYNRWRFLLECLEDLDNQLK
ELGGRLYTFQGDPVEILQKLFDEWGVTKLTFEQDPEPPWQKRDIAVKKLCREKNIECIEKVSHTLYNPQDILK
ANGGSPPLTYAMFCEVCKTLPAPERPQPVPSFSGVSLPVANDFSKFELPTLNDFGITPECSEQKKKKTPPYLG
GESRGLDLLELRRMQEKTSYKAGFILPHYVNPDLSGPPLSMSPHLRFGCVSVRRFYYTILDTFREVYKDHEQS
NELLTAQLMWREYFYVMSVNNSNFTKMVNNPICLQIDFNWDHEVVRKWEKGQTGYPWIDACMRQLVQEGWLHQ
VGRHAVSVFLTRGDAWQHWEMGLQVFFKYLLDADWSVCAGNWMWVSSSAFEKCLDCPKCFHPVRYGQRMDPVG
DYVRRYVPELSQFPLRYLFHPWRAPLEVQKKAGCIIGKDYPAPIVNHAEAAMKNGERMSLVREKYAKLPDIPH
VKPSNDEEVWRIAWLPRNLTSVPCVSSDKCDALDIDNLDGLSM
>Lingula_ana_XP_013407859.1_cry_DASH
MSGTAMKTVICLIRNDLRLHDNELLHWAYSHLQASHVLPLYCFDPRHFAGTYHFGFPKTGAHRLQFLLESVDD
LRKSLKARGSNLIIRQGKPEDVVPAIIKCLGQGNVIAVGFQEEATQEELDVEAALKKNCGVQIKTFWGSTLYH
KEDVPFKIQHVPDVYTQFRKAVEGQSSVRKLLEIPEQLKPPPPDLEEGEMPTMQTFGMQGEVKDERSAFPFCG
GETEALSRLKHYLWDSDSVAKYKETRNGMVGADYSTKFSTWLAHGSISPRKIFWEIKRYEKERTANQSTYWVI
FELIWRDYFKFVALKYGTRLFFLDGIMGKKIQWKQDPKLFQAWKTGNTGVPYVDANMREMAATGFMSNRGRQN
VASFLTKDLHLDWRMGAEWFESCLIDHDVCSNYGNWQYAAGIGNDPREDRKFNMVKQGMDYDPEGEYVRLWVP
ELSEIKGGNVHTPWALSKALLSKARVNIGETYPHPIVIAKEWAKHVGRQSDSRGRAPSGKGRQRGIDFYFKNP
SKP
>Lingula_ana_XP_013416713.1_cry-1
MTSTQKNVIHWFRKGLRLHDNPALVEALEGAVTFRCVYILDPWFAGSTQVGINKWRFLLQCLEDVDASLRKLN
SRLFVVRGQPADVFPKLFKEWKTTHLSFETDPEPYGKERDATIISLAQSAGVSVISRISHTLHDPKRIIDLNG
GTPPLTYKRFQTIFAGMEPPPKALDPITPDMVRGTLPVVSEDHDEKYGVPTLDELGFDTEGLAPAEWKGGEQE
GLARLVRHLERKAWVASFEKPKMTPQSLMSNQTGVSPYLRFGCLSVRLFHWKLTELYRRVKKRSDPPVSLFGQ
LLWREFFYVVATNNPKFDRMVGNPVCIQIPWDRNAIALAKWAEGKTGFPWIDAIMIQLRQEGWIHHLARHAVA
CFLTRGNLWISWEEGMKVFDEFLLDTDWSMNAGMWMWLSCSSFFQQFFQCYCPVGFGKMADPNGDFVRKYIPA
LKGFPAKYIYDPWNAPEHVQKMAKCIIGKDYPLPMVNHAEVSHINMERMKQVYQHIFFRISRTGMV
>Lingulodinium_pol_QDO16286.1_cry_DASH
MAAVVCAWLRNDLRAHDSPVLQGAAALARQRGLPALPVYVLDPRHFQRTSHGTLKTGPFRALFLLQALRALKR
RLRGLGSDLLVRIGRPEVVLPPLLPSGSVLLTQQEVTSEELGVDGRLRSALAGGVEWEYRWGSTLFHKEDLPF
HDDLRNAPDVFTSFKNKVEPELAARVNEVPSSYEGKRKPQSSIRVRPCLPEPAKGSLPLPALDPAELGFEPSW
ADLPYEEAVAEPTPHEGAALDFCGGEDTALERLKYYLWDSDLLATYFDTRNGMLGGDYSTKLAPWLALGCISP
RRVFEQVREYEGARVANKSTYWVIFELVWRDFFRFFAAKHGDAIFWRGGVAGRAEGWRRNDKLFDLWARGCTG
FPLVDANMRELLATGFMSNRGRQNVASFLALDLGLDWRRGADWFESYLVDYDVTANWGNWVHAAGLTTGRVNR
FNVVRQSKMYDPDGAYLRHWLPELRRVPAPRIHEPWLLQEAELQRYGAEAYPQPCLDPARFRETGGGGRRRR
>Lingulodinium_pol_QDO16287.1_CPD_photol
MRADRSNTSELSPYIRFGELSVRTAFWAARRRRERTDQWLFKEEVYKGLHRANATYLRRFLWRDLAYWFLWRF
PTLPQTSLRPQYEEQVWSGTRAQLRAWQRGSTGFPLVDAAMRQLWALGWMPNYLRHVVAQTLVEYLDVTWKEG
FRWFDWTLVDSDCAINAYMWQNCGHSGPDHWNFVMHPVHAAKSCDPEGHYVKRWLPCLAHLPVEYVHCPWEVP
ARGVRAAAGLLRSAYVARLVEDLDAARRAHAARVLQVRRDHPETISRTGHEWLRLGGG
>Lingulodinium_pol_QDO16374.1_CPD_photol
MPPRKKASAPKGKAAAAEDRKDDGSGDVSGGVVDWRRRRLLTPGVPAAGAAMEGKAVLYWMSRDQRVQDNWAL
LHARDLAKAAGGQLLVAFCLVPRFLEATIRQFDFMLRGLREVETELRELKIPFELLLCEYGSHAKSILGLCEQ
RGVGAVVCDMSPLRVPKAWTAEVAATLGKAGVSCVQVDAHNVVPVWQASSKQETAARTIRSKITLQYGEFLKE
IPAFEPMAKQEAGKPVDWEKAEASLVVDRTVAPVPGIEPGAKAAMQRLHEFCKKRLGMFAEQRNNPNADALSG
LSPYLHFGQISAQRCALAVRAAEEDQSAGAAVAKGCESFLEEAIVRRELSDNFCWYQEKYDSLEGAAGWAQET
LRVHEQDKREHLYSEEELEAARTHDDLWNAAQLQMVREGKMHGFLRMYWAKKILEWTSGPEEALRIAIRLNDR
YELDGRDPNGYVGCMWSICGIHDMGWTERPIFGKIRFMNYAGCKRKFSIPNFVSRYPSSLGASMPAKVVEEPP
AKRSKKA
>Lingulodinium_pol_QDO16378.1_6-4_photol
MTGVWFRKCLRLHDNAALIQATRESDHVFPFFILDPWFDRSRVGANRYQFLLDSLSDLDEQLRTKYGSRLLVL
RGRPESVFQELFEGKGPFRLQALYWEKDSEPYARERDARVEALAARLDVRTRSFAGHTLLLDLDTVVASPGYKP
PISMRDVLALVTRCGPIAEPQPEPCVPPLEVQGFEVPSIAEFYDELPTSRGFPGGEREALKRLDATCADVEYV
CSFEKPKTASTGRPRAPWEPSTTGLSPYFKFGCLSVRTAWSAIAACYRQRAHSQPPQSLHGQLLFREMFYLLG
ASVPNFDKAVENPMCKQIPWGEDPALLAAWSEGRTGYPFIDALMRQLRTTGYMHHLGRHAVACFLTRGDLWQH

WTRGRDVFDKLLLDADWAVNNGNWLWLAGVAPFSAPYFRIYDPCPGPKSSLNAEQAGEFVRHFVPELRAMPDR
FIYKPWTAPRAVQERAGCIVGVDYPAPVVDHAAAREANLGRFKAALAAQPGRELPEKFGPRRPGATSGRGQAE
AQVQGRVRGRQPEADADVEMLPSAGAQTKLRRWGKAPDRVLGA
>Lithobates_cate_PIO31814.1
VLLWAHRNADHIVPLYCFDPRHYLGTHHYNFPKTGPLRLKFLLESVKDLRNTLKKRGSNLLLKRGHPEDVIED
LIKQLGPVSAVAFQEEATKEELDVEKSIKQVCTRHGVKVQSIWGSTLYHRDDLPFRHISSLPDVYTQFRKAVE
TQCKVRPTLQMPEQLKSLPAGLEEGSVPTLEDFEQQDPLSDPRTAFPCSGGESQALQRLDYYFWDTNLVASYK
DTRNGLIGMDYSTKFAPWVVFELLWRDYFRFVALKYGRRIFFLREGRTGVPFVDANMRELAMSGFMSNRGRQN
VASFLTKDLGIDWRMGAEWFEYLLVDYDVCSNYGNWLYSAGIGNDPRENRKFNMIKQGLDYDGNGDYIRLWVP
ELQQIKGGDVHTPWALSGSALSHANVTLGKTYPLPIVMAPEWSRHINQRPFLQMIYVQTVIELSYGYCLNHNC
TYPFFSKPELVLFEGRNAGGIGYLVM
>Lithobates_catesbeianus_AAP13561.1_cryptochro
MEGPAVSSVHWFRKGLRLHDNPALLAALRGARCVRCVYILDPWFAASSSSSGGVNRWRFLLQSLEDLDSSLRK
LGSRLFVGRGQPADVFPRLFKEWGVTRLTFQYYSEPFGKERDAAIMKLAKEAGVEVIVESSHTLYDLDKIIEL
NGNSPPLTYKRFQAIVSRMELPRRPVPSITRQQMEKCRAEIKSTHDDTYGVPSLEELGFPRDNPGAAVWPGGE
TEALARLDRHLERKAWVAHYERPRMNANSLLASPTGLSPYLRFGCLSCRLFYYRLRELYQKVKKNSPPPLSLF
GQLLWREFFYTAATNNPKFDQMEGNPICVQIPWDKNPEALAKWTEGKTGFPWIDAIMTQLRQEGWIHHLARHA
VACFLTRGDLWNSWECGVKVFDELLLDADFSVNAGSWMWLSCSAFFQQFFHCYCPVGFGRRTDPSGDYVRRYL
PVLKGYPSRYIYEPWNAPESVQKEAKCIVGVDYPKPVVNHAEASRLNIERMKQTYQQLSRYRGLSPRLCAVDS
PKRKHEDLDGELCKKARLQCVQEMERAAKDFL
>Lithobates_cat_PIO14260.1_AB205_0057
ELLLDADWALNAGNWLWLSASAFFHQFFRVYSPVAFGKKTDKNGDYIKKYLPFLKKFPAEYIYEPWKAPRSIQ
ERAGCIIGKDYPKPIVVHETVSKQNIQRMKAAYARRSGGGEKETEKGSAKNGQKRKSPSVADLLKKKRS
>Lithobates_cat_PIO30423.1_AB205_0053
MLRFLLQCLEDLDANLRKLNSRLFVIRGQPADVFPRLFKEWKISKLSIEYDSEPFGKERDAAIKKLASEAGVE
VIVRISHTLYDLDKIIELNGGQPPLTYKRFQTLISKMEPLEMPTETITTEVMDKCTTPVSDDHDEKYGVPSLE
ELGFDVEGLPSAVWPGGESEALTRLERHLERKAWVANFERPRMNANSLLASTTGLSPYLRFGCLSCRLFYFKL
TDLYKKVKKNSSPPLSLYGQLLWREFFYTAATNNPRFDKMEGNPICVQIPWDRNPEALAKWAEGRTGFPWIDA
IMTQLRQEGWIHHLARHAVACFLTRGDLWISWEEGMKVFEELLLDADWSVNAGSWMWLSCSSFFQQFFHCYCP
VGFGRRTDPNGDYIR
>Lithobates_cat_PIO38983.1_AB205_0120
MESPGLPPLPDISGMKRQLLEGEIGAEEFFCLVMSTLGEHRTYRHLPHLIHNLTHTQPGLYGRLMDIHREYFH
QRPSDLPTYEEELEIAIALSLEEARSSAGPGCTIQDPVSNSSISFAEAAKKSPVLNSNGDQKPSTNAKCKKVH
DDLTTVELSKNLSKLRTADHLDSLYTGQPEPEDCVLNCDGMAVFQIQSPITKADKMKRSRRGRRKKNSLVPKN
PVLVKPVVVWFRRDLRLHDNPALIAALELGAPIIPVFLWSLSEETGQNFTLATGGASKYWLHHALFNLNLSLE
QSYGSHIVFRVNDSCVDELVGLVQETGAGTVLVNAVYEPWLKHRDDLLSETLKKRGVTFTRYHSYCLYEPYSV
STEGVGLRGIGSVSHFMSCCERNSSSPIGDPLEAPTSLPLPSCWPDSLELDRLDLAKMPRRKDGTVIDWAATI
KKSWDFSEDGAYICLGNFLEDGIKHYDKESGRADKPYTSHISPYLHFGQISPRTVLHEAYFTKKSVPKFLRKL
AWRDLSYWLLVLFPDMHVEPVRPAYKSQRWSSDRSHLRAWQKGMTGYPLVDAAMRELWLTGWMCNYSRHVVAS
FLVAYLHLHWIHGYRWFQDTLVDADVAINAMMWQNGGMSGLDHWNFVMHPVDAAMTCDPYGSYVRKWCPELAG
LPDEYIHKPWKCPPSQLRRAGIVLGQNYPHRIVVDLEERREQSLRDVVEVRQKHSEYVDKVSGSDMVSVPDQL
LAFTLGCADGDDNDDVIKSNTCQFLLPVITRKEFKYKTLQPNTKDNPYNTVLKGYVSRKRDETIAYMNERHFT
ASTINEGAQMYERRERTARIMEGLPQMRDPKNKSRRTPTSDPFSIVPPAYFHLAN
>Loa_loa_XP_020307232.1_photolyase
MYCRMESPRIQPLNKQNIVVGKYVLLLIRCIRSQQSPSFSFASGKANEHGVPVLVTYIYQPDRHNLAQRKFLL
EGLNCLENNLSRLHAPLLVIKANNNQEATKIVLKLSDAACEVITDAAYLREDRRLDENLNDKLIMKCRRFTKV
EGNVTIPVTVLCSKPAYNANTIRKVAWHFLDDFLLEKWNVTPKIHCKSWKSIVEYNLECTDISSEYTRAVADC
KIGSVLKGGEDAALQVMDYFIANNLKAYDKERNIPNGKKQSLLSPYMHFGMLNPITIVNEVKQSKAPKSAKDA
FLEEMVVRRELAHNFVYYYRDTYDTFDCLPEWAKKTMEEHRFDKREYIYNYKELEEGRTHDVYWNAAQFELVF
THKMSGYLRMYWAKKVIEWSPDYERAYAFLIEQNDKYELDGRDPNGYCGVMWNFGIHDRAHANRPVFGKIRYM
CADGLRRKFRNHIDDYVSINYRRAGRILELNSYVQSKATKTSEDFDPKRRRTCRTMKK
>Lophium_myt_KAF2488308.1_photolyase
MPPKRKTPAAPYTDPVGSAPNKRSRASKPAPKATESNGNDPPSFDHSRPEETSGIVQREFYPAELSNERCAMY
NSGEIPRPLAVLETTLSETRSAREKTEIGESVVHWFKRDLRLRDNKGLSLAAAKAKAGGVPLICVFLVSPQDY
QAHLTSAPRVDFEIRTLEVLKGDLGRLDVPLVVETVEKRRRLPGRLLEMAERWGAKHVFCNVEYEVDELRREE
SLVKLGLEKGIAFEAVHDDVVVPPGALVTGTGKQYAVYSPWFRSWIKHVHAHAELLKVWEGPGKNPSDARTKF
RTLFDSEMPEAPDNKKLEPEEKKKLERLWPAGEHEAIARLEKFLGEKVGKYSAMRNFPTANGTACVSVHHSVG
TLASRTSVRMARDANNTKRLDAGNEGIRVWISEVAWRDFYKHVLANWPYVCMNKPFKYEYTNIEWEYNTEHFE
AWCAGKTGYPIVDAAMRQLNSTGYMHNRCRMIVASFLAKDLLLDWRMGERYFMEHLIDGDFASNNGGWGFSAS

TGVDPQPYFRIFNPLLQSEKFDAEGDYIRKWVEELRNVEGKAIHDPYGRGAAGEAKKKGYPKQIVEHKFARER
ALTRYKAGIGRETA
>Lophium_myt_KAF2495633.1_DNA_photol
MAIKPRVIYWFRTDLRLHDSPALAAALDLKPECLWPIWCWDPHYVYRARVGPNRWQYLLDCQQDLSDSIARLN
PKSRLHVLREAPQTLLPKLFKAWKVTHLVFEKDTDAYARSRDEEVMSSAREAGVQVIVKYGKTLWDPDAVVKA
NGNKPTMTISQLRHAGEKVGDIPRPIPAPKSIPDPGNMPLDFAQTQPAPEPDLNSIYRTRKENSYEKLAGPNG
DFAVPTIEELGIKSATTPHRGGETGALAVLDNIIKNEQYTATFEKPKSAPTAFEPQSTTLLSPHLHFGSLSCR
EFYWRVQDVVDNFKGKASQPPVSLTGQLLFRDMYFAAQAKMGWKFSQTVGNDHCRFIPWHLRSKVDGPDGLVT
GEYNIDSVEAEEWFQRWKTGRTGFPWIDAVMRQLKQEGWIHHLARHSVACFLTRGGCYIDWERGAEVFEEWLI
DHETACNTGNWQWLSCTAFFSQFYRCYSPIAFPQKTDKNGDFVRHFVPELKDFPAKYIYEPWKAPIQDQRTAK
CIIKGDGTVQTIDELPAYPKPMFDFSTRRDICIGGMKNAYRVGLYGNSPKVVNGSWPSAFDDDAEGTTDGKNI
SEQDTLEVETHRQIQTDTNSKADSLKPMNKNKRKGTGTLEGFLRPQKAMK
>Lophium_myt_KAF2499533.1_cry_
MPPTPPPTPRILIYLLRHDLRLADNPIFHDLSRISRSSAYKTTASLDPPSPPICHSHSPPKSPTSARIQKTYE
ASGPALAEKLGVTHLLPMYVFPANQIEISGFLSEDGSGTSKSKSPYPEARSEVGGFWRTGPHRAKFIAESVWD
LKGKLERLDCESSLVTRVGLMGDVVDNIVSWYSQQRQGEVTGKVVGIWMTGEEGTEEATEERDVRRVAEDEGI
DFRIWKDEKYYVDDRDQPFTKISDLPNVYTTYRKPLEPLSSRPRPILPTPHRLPPLPDSLPPQQSPFSIPTTL
ATLKSALLAPLDADPTLGLPSPPQWAPKTTTAHPFLGGERAAHERLTHLASSGAMTAYKSTRNGMLGLDFSTK
LSAYLAYGCLTARQVHWAMVDFEAGTGGDGVGVAGYGRGENEGTAAIRFELLWRDYMRLCARKFGASLFDIDG
FRGAENTADDGEQPQPPKRWKYLDRSGGLGDDPAKTREALLRFLSGTTGIGLIDAAQRELFLTGYTSNRARQN
VASFLASHLGIDWRVGAEWYESMLIDYDMANNWGNWQYVAGVGNDPRQGRVFNPVKQALDYDAQGEYVKAWVG
ELRDVDLGEDEKGHVQVEKLMGLFQAWRLDSVEKERLGLNGVESVEHPLIRIPFSVTKSRGSDGGNSRGRGRG
GSYRGRGKGQGARRLGEMDKVARAGGAY
>Lottia_gig_XP_009045127.1_LOTGIDRAFT
MAGKVIICLLRNDLRIHDNEALLWAFKHGSQVLPIYCFDPRHFKGTYHFGFAKTGPHRLKFLIESVQDLRCRL
KSKGSGLIVRTGKPEEVIPELIKSVGPEFVSSLVYTQEVTQEEIDVEAALNKSCGVKIQTFWNSTLFHKDDLS
FSIKQLPDVFSQFRKRAETECSVRSVIPTPETFKPLPQGIEEGNIPTCEDLGVENVPQDSRTAFPYSGGETSG
LDRIKYYFWETDKVAKYKETRNGMIGADYSTKFAPWLAHGCLSPRFIYSEIKRYEKERISNQSTYWVIFELLW
RDYFRFVAMKYGNEIFFLNGIRRMHLPWKKDTNAFIAWKEGRTGVPYVDANMRELAATGFMSNRGRQNVASFL
TKDLKLDWRLGAEWFESMLIDHDVCSNYGNWQYSAGIGNDPRQDRKFNMVKQGLDYDPNGDYIRLWIPELNQV
KGGDVHTVWALNSTVLSRANVVLAQTYPKPLVIAPEWGRHMGKVGGATRGGGRGCGGQQQPTKRGMDFYFKNT
QPRQ
>Lottia_gig_XP_009045182.1_LOTGIDRAFT
MTSRSVHWFRKGLRLHDNPALLAALDKCVEFRAVFILDPWFVKNYKVGINRWRFLIESLTDLDNSLRKLNSRL
YIIRGKPESVFPALFKKWKITQLSYEIDTEPYALRRDKAIDILAEELNVKILKHVSHTLYDTNRIIEKNGGKA
PLTISKLQSIVKQVGNPSKPLDAPTLQHFKDIKIESDEDISENYKVPTLEELGQDPSLCGPVLYPGGETESLK
RLENSIAKPSWIASFEKPNTEPNSLSPSTTVLSPYIKFGCLSPRLFYWKIQEVYDKVKKHTAPPVSLHGQLLF
RELFYTVGYVTPNFDKMVDNPICTQIPWQNNEEYLKAWREGKTGYPFIDAIMTQLRQEGWIHHLARHAVACFL
TRGDLWCHWEEGQKVFEEYLLDADWSLNAGNWMWLSASSFFYQYFRVYSPVAFGKKTDPNGLYIKKYLPVLKK
LPSNYIYEPWKAPKSVQEAAGCIIGKDYPKRIVDHDIVRPINIERMHEAYAKHKASKSKTTTDTPFLQAASLT
GRNRVEKSEGIIST
>Lottia_gig_XP_009051269.1_LOTGIDRAFT
MNSKNSYLRKGTSVLWFRHGLRLKDNPALLAAVKSCNEFYPVFIFDGEVAGTKTAAYPRMKFLVESLNDLDRQ
FRALGGRLYCFRGDPVKIFTNLFVEWNVSKLTFEVDPEPIWQERDDKVKGLCERHNVDWEEFHSHTLWNPNEI
IENNGGHPPLTFDLFKQVSELVGSPARPEPDADFTHVNFGSKSDDDDNKFKIPSCEELGVYCEDEKQKKPYNN
YIGGETRGRKLLDSRMKVESLAFESGYCMPNQYKPDLTGPPLSLSPHLRFGTVSVREFYWKILDAYAEVYPNQ
EAPVSVIAMLIWREYFYTMSVNNLHFNTMKDNPVCLNIKWYKNQEHLDRWTNGKTGFPWVDACMNQLRSEGWL
HHVGRHMVSCFITRGDLWLSWEDGLKVFDKYLIDADWSVCAGNWMWVSSSAFEKMLQCPQCFSPSMYGRRMDP
TGEYIRRYVPEIARFPLHFLFEPWKAPLKVQQKAGCIIDKDYPAPMVNHQEVSIRNQKLMEEAKTLSRDQASH
CCPSNKKEVRDFVWLPDHTPAGGHCSGDGLCEGIECL
>Lottia_gig_XP_009060728.1_LOTGIDRAFT
MGSDKKSKEKVTKKSSKGTKSEDVPKDSKEKSPETDFSSKKRKLEEVSTTEKSSETSPSKKLKTDGDFVSRIA
AKRTNVCQSVTEFKFNKKRVRVLSQASDAPDENDGVVYWMSRDQRVQDNWAFLYAQRLALKMEVPLHVCFCLV
PKFLEATIRHFEFMLKGLEEVEEECRNLDISFHLLIGYAKDVLPEFVGKNKMGSVVTDFSPLRTPMSWVEDVK
NNLSKDVPFCQVDAHNIVPCWEASPKCEYGARTIRNKINNQLSTYLTEFPPLVQHKYKAKHIAQKVDWKAAIE
SLEVDRTVTLPDWCKPGTNGGLETLESFLKDRIKYFNSERNKPDKEVLSNLSPWFHFGQVSVQRSIIMVKEVK
SKYKESVEGFLEEAIVRRELADNFCFYNKNYDNIEGAYEWARNTLKDHWNDKREYLYTRQQLAESSTHDDLWN
AAQYQLVTEGKMHGFLRMYWAKKILEWTENPEVALKDSIYLNDKYSMDGRDPNGYVGCMWSICGIHDQGWKER
PIFGKIRYMNYQGCKRKFDVAAFVRKYKVKKPLPNIFNKK
>Lottia_gig_XP_009064438.1_LOTGIDRAFT

MNEQDNVPAKHSIHWFRRGLRLHDNPALIEAVKGATTFRCVYILDPWFASSSNIGVNKWRFLLECLEDLDSSL
RKLNSRLYVVLGQPVAVFPSLFKKWKITALTFEEETEPHGRNRDNVITSLAHDAGVEVIKKTSHTLYNLQDII
QINSGKPPLTFLRFQSLLCVMGPPNGTVNKLTEKLINNCENTVQSDHDEKYGVPDLATLGFDCANLNKIAFKG
GETEALCRLHRHLERKAWIASFETPKMTPESLMPSQTTLSPYLRFGCLSAREFYWQLRDLFKKLNRGEEPSLA
MYGQLLWREFFYLVARDNMKFDQMQGNPICVQIPWDKNSTALAKWTAGMTGYPWIDAIMTQLKKDGWIHHLAR
HAVACFLTRGDLWLSWEEGMKIFDEYLLDADWSINAGMWMWLSCSSFFAQFFHCYCPVNFGKRVDPSGDFIRH
YLPILRAYPQEYIFEPWTAPESVQKSAKCIIGKDYPLPMVNHHEVSQLNADRLRQVYHHLTLKVSSKFKKTDR
Y
>Loxodonta_africana_XP_003412157.1_cryptochro
MAAAVVTAGAAALVPIPSMDGASSVHWFRKGLRLHDNPALLAAVRGARCVRCVYILDPWFAASSSVGINRWRF
LLQSLEDLDTSLRKLNSRLFVVRGQPADVFPRLFKEWGVTRLTFEYDSEPFGKERDAAIMKMAKEAGVEVVTE
NSHTLYDLDRIIELNGQKPPLTYKRFQAIISRMELPKKPVGSVTSQQMESCRAEIQENHDETYGVPSLEELGF
PTEGLGPAVWQGGETEALARLDKHLERKAWVANYERPRMNANSLLASPTGLSPYLRFGCLSCRLFYYRLWELY
KKVKRNSTPPLSLFGQLLWREFFYTAATNNPRFDRMEGNPICIQIPWDRNPEALAKWAEGKTGFPWIDAIMTQ
LRQEGWIHHLARHAVACFLTRGDLWVSWESGVRVFDELLLDADFSVNAGSWMWLSCSAFFQQFFHCYCPVGFG
RRTDPSGDYIRRYLPKLKGFPSRYIYEPWNAPESIQKAAKCIIGVDYPRPIVNHAETSRLNIERMKQIYQQLS
RYRGLCLLASVPSCVEDLSSPVAEPSSSQAGSSNSAGPRPLPSGPTSPKRKLEAAEEPPGEELSKRARVAKLP
GPELPSKDV
>Loxodonta_afr_XP_003405361.1_cry-1
MGVNAVHWFRKGLRLHDNPALKECIQGADTIRCVYILDPWFAGSSNVGINRWRFLLQCLEDLDANLRKLNSRL
FVIRGQPADVFPRLFKEWDITKLSIEYDSEPFGKERDAAIKKLATEAGVEVIVRISHTLYDLDKIIELNGGQP
PLTYKRFQTLISKMEPLEIPVETITSEVIGKCTTPLSDDHDEKYGVPSLEELGFDTDGLPSAVWPGGETEALT
RLERHLERKAWVANFERPRMNANSLLASPTGLSPYLRFGCLSCRLFYFKLTDLYKKVKKNSSPPLSLYGQLLW
REFFYTAATNNPRFDKMEGNPICVQIPWDKNPEALAKWAEGRTGFPWIDAIMTQLRQEGWIHHLARHAVACFL
TRGDLWISWEEGMKVFEELLLDADWSINAGSWMWLSCSSFFQQFFHCYCPVGFGRRTDPNGDYIRRYLPVLRG
FPAKYIYDPWNAPEGIQKVAKCLIGVNYPKPMVNHAEASRLNIERMKQIYQQLSRYRGLGLLASVPSNPNGNG
GLMGYSPGENTPGCNSSGSCSQGSGILHYVHGDSLLKQGRSPTGTGVSSGKRPSQDEETQTLGPKVQRQSTN
>Lucifuga_dent__KAF7647002.1
XRLHDNPALKDSIRGADTLRCIYILDPWFAGSSNVGINRWRFLLQCLEDLDASLRKLNSRLFVIRGQPTDVFP
RLFKEWKITHLSYEYDSEPFGKERDAAIQKLANEAGVEVMVQISHTLYDLDKIIELNSGHPPLTYKRFQALIN
HMDAVELPAETITSEDMKKCVIPISEDHDDKFGVLSLEELGFETEGLTTSVWPGGEMEALMRLERHLERKAWV
ANFERPRMNSNSLLASPTGLSPYLRFGCLSCRLFHYKLTDLYRKVNKNSTPPLSLYGQLLWREFFYTTATNNP
CFDKMDGNPMCVQIPWDRNPEALAKWAEGRTGFPWIDAIMTQLRQEGWIHHLARHAAACFLTRGDLWISWEEG
MKVFEELLLDADWSVNAGSWMWLSCSSFFQQFFHCYCPVGFGRRTDPNGDYIRRYLPILRGFPAKHIYDPWNA
PKEVQKMAKCIIGVHYPKPMANHAEASRINIERMKQIYQQLSCYRGLGLLATVPANPNNNVSGSNAGGVNTRS
EGFSTQEITCQTDRGQSTQKRRREETTPENSYKTRQQSK
>Lucifuga_dent__KAF7650071.1
MKLRKRCSPPLSLFGQLLWREFFYTAATNNPNFDRMEGNPICVQIPWDQNPEALAKWAEGKTGFPWIDAIMTQ
LRQEGWIHHLARHAVACFLTRGDLWISWESGMKVFEELLLDADWSVNAGSWMWLSCSAFFQQFFHCYCPVGFG
RRTDPSGDYIRRYIPILKDYPNRYIYEPWNAPESIQKAASCVVGVDYPKPMINHAEGSRLNIERMKQVYQQLS
HYRGLSLLASVPTIQEEVEPPMTDESQTSSGPDSPPTDPCDSEAPGCSAAPDSSTACRSSVATAPHPDQDGAL
ASDLSQTACTSSAVTSTSMGHSPATPTHHVTEAPVSPSPIPEEIPLSTSKLSSASSPSLTLSLSPGPAAAQAQ
ASSLGPKRKGLPWKLRRSQQQRGRQGCTAAREKEDKKRAGEEEKMEEDAEQDVDRMEEETSGESRGNQ
>Lucifuga_dent__KAF7661351.1
MRVCASCVRQGCRNTVCRQILKLVALSKKRLILVLSCIKKSVTVQTTMPAEKRKAASSAKGPDAKQQKLVKER
AEGWLQDLMVQQRTEKKEMKFNRKRLRFISDTQKIKQESKGILYWMSRDHRIQDNCKCRSMRRNLLWKRTCLC
ISVLGAVVTDFSLLRQPLQWLEEVKKKLSKDVPLIQVDAHNIVPCWVASSKQEYSARTIRGKITNLLPEFLTD
FPLVEKHPFAATRTAKPVDWAQTLDSLQIDRTVGDVDWAKPGTSAGMAMLESFIDERLKLFGTQRNDPNCAAL
SQLSPWIRFGHLSAQRVALQVQRDGKNACQSIPPFIEELVIRRELTDNFCFYNKKYDRVEGDYEWAQKTLKDH
AKDERSHIYTREQLEQAKSHDKFWNAAQYQMVTEGKMHGFLRMYWAKKILEWTSSPEEALSISLYLNDRYELD
GQDPNGFVGCMWSICGIHDQGWAERPVFGKIRFMNYKGCLRKFDVARFERKYCPKNL
>Lucifuga_dent__KAF7666419.1
MEGNPVCTQVDWDTNPEYLAAWREAWTGFPFIDAIMTQLMQEGWIHHLARHAVACFLTRGDLWISWEEGQKVF
EQLLLDGDWALNAGNWQWLSASAFFHQFFRVYSPITFGKKTDKNGDYIKKYLPVLKKFPAQYIYEPWKAPASI
QQAAGCIVGKDYPCPIVEHEIISKKNIQRMKTAYAKRSADPKESPNKSKGEKCKGPSVVDMLTKKTRRM
>Lucifuga_dent__KAF7669269.1
MSTSRTVVCLLRNDLRVHDNEVFHWAQKNAEYIVPLYCFDPRHYKGTHNYNLPQTGPFRLRFLLQSVRDLRNT
LINKGSTLVVRHGKPEDAVAGLIKQLGSVSVVAFHEEVTSEELDVEKKVKDICAQMKVRVQTCWGLTLYHRDD
LPFNHTARLPDVYTQFRKAVETQSSIRPLFPTPGQLKPLPPELEEGAIPTVEDLQQTGCETDPRSAFPCSGGE
SQALHRLQHYFWDTDAVATYKETRNGLISVDCSTKFAPWLAMGCISPRYIYHQIKQYERERTANQSTYWVIFE

LLWRDYFKFVAVKYGSRMFQIKGLQDKSIPWKVDMKLFDAWKEGRTGVPFVDATMRQLAMTGFMSNRGRQNVA
SFLTKDLGLDWRLGAEWFECLLIDYDVCSNYGNWLYSAGIGNDPRENRKFNMIKQALEYDSNGDYVRKWLPEL
QGIVGADVHMPWTLSSAALSHAHVSLGESYPTPIVTAPEWSRHFNKKPSCTGPSARGKKGPSHTPKQHRDKHN
>Lytechinus_variegatus_XP_041453155.1_cryptochro
MPKRKHRSSRHSEKPGCLVHWFRKGLRLHDNPSLKEGLKAASGFRCIYILDPWFAGSCSKGVNRWRFLLECLE
DLDSSLRRLNSRLFLIRGQPADVLPRLFKEWKVTQLSFEEDSEPFGRTRDKAISTLAQEAGVKVISQVSHTLY
DPQEILALNDNEPPLTYKRFQDIISMMGIPVYPVDALEAEDVEGLETFIDPNHEDKYGIPTLEELGFDQEDVP
PPMWIGGETEAKQRLDRHLERKAWVANFERPRMSPASLMASPAGLSPYLRFGCLSPRTFYWKLTELYQKVRKT
THTPLSLHGQLLWREFFFTVACNNSMFDRMVDNPICIQIPWDKNTAMLNKWANGETGYPWIDAIMTQLRLEGW
IHPLARHAVACFLTRGDLWISWEEGMKVFDEFLLDADWSVNAGNWIWLSCSSFYQQFFHCYCPVKFGRRTDPN
GDYVRKYLPFLKNFPSKYIFEPWTAPLEVQEKAKCIIGKDYPLPIVDHAEASHRNIERMRKVYNNLSRHGGPG
ILGSVPSSKAASAPPKVGKPNVSSKLMPEHQQHQQQIQEAPVAQSPNNSILMPRPSKMRFMGMGMGHMQMPFS
RDTGSNMGEPSGIGGGGVGAGVSEVSVVPDVRGMEVSGLNVTDVTSHLPGSSSSTLSYQPSLVIQQHQQHQQH
PQQHPQHQQQQQHQQQQQQQHQMSQQHMPVTITGLSPIDVPGTSGSLLKELEGADWQSHWQFTNQ
>Lytechinus_var_XP_041452601.1
MPGGACIHWFRHGLRLHDNPALLEGMTLGTEFYPIFIFDNEVAGTSTSGFNRWRFLHDCLADIDEQLKAGGGR
LFVFQGDPCHVFKEMFLEWGVRYLTFESDPEPIWTERDCKVKALCKEMNVECIERVSHTLWNPDHIIEKNGGT
PPITYSMFMECVTEIGHPPRPMPDPIFTKVKMNVLADFEERFSLPSLEALGVRLECPEQEKKVWKGGETRALE
LFRVRILHEEEAFKGGYCLPNQYMPDLLGTPKSLSAYLRFGCLSVRKFYWKIHDTYSELKNEVSPSHLTAQVI
WREYFYTMSVGNIHFNKMKENPICLNIEWKKDDEKLEAWTEGRTGYPWIDACMKQLKYEGWIHQVGRHATACF
LTRGDLWISWEDGLRVFDKYLLDADWSICAGNWMWISSSAFEKFLQCPNCFCPVRYGRRMDPTGEYVRRYLPV
LKDMPIRYLFEPWKAPRAVQERAKCIVGKDYPMPIVEHKSASAANTEQMEKVINRLRDSGIVHCAPSTQKEVR
EFVWLPEKMAGGGSCRADQNCEGILGL
>Lytechinus_var_XP_041469260.1
MAGRMKIIICLLRNDLRYHDNEVLFWAHKNATNVIPLYCFDPRHYKSTYNFGFPKTGPHRLKFLLESVEDLRK
TLQSVGSGLVVRSGQPEDVVPQLIQQFGKDEVAAVALQEEVTSEELDVEAGLQQSCSKLGVQLKKFWGSTLYH
RQDVPFDPQGVPNVYTEFRKKVENRSHIRPPINMPNSLKGLPQGTEEGDIPTFSSFGVKDPGSDSRSAFPFRG
GESTGLARIEDYFWKSDCISQYKETRNGLIGSEYSTKFAAWLAHGCVSPRKIHAEVKRYEKERTANQSTYWVI
FELIWRDYFKYVAMKFGNKMFALNGLLDVQKQWTKNKRWFDAWSKGLTGVPFVDANMREVAATGFMSNRGRQN
VASFLAKDLCLDWRLGAEWFEQMLVDHDVTSNYGNWLYSAGVGNDPRESRKFNMIKQGLDYDPQGDYIRLWIP
ELKGIKGGAIHMPWTLSNAELNQAGVSLGETYPSPIVTAPEWSRHTSRPVSDKVKLISVILHI
>Lytechinus_var_XP_041481477.1
MPYNSTIHWFRKGLRIHDNPALLTAIQGTKVFRPVFILDPNFLESGKVGINRWRFLLEALRDLDYSFRALGSR
LFVVRGNPTTVFPELFKKWNVTRLTFDVDTEPYARQRDQEVIELAKKNGVEVVTKVSHTLYDTERTIKANKNK
PPMTYQRMVGLLSEIGAPALPELAPQISNFTGVTTPVKPDHDSVYGVPSLEDLGLDASGLGPRLYPGGETEGL
QRMELHLARKSWVCGFEKPKTSPNSLEPSTTVLSPYLKFGCLSPRKFYYAIKEVYAQKTNCTKPPVSLMGQLI
WREFFYTVAAGTPNFHQMEENPICLQVPWDDNPEFLAAWKEGRTGYPYIDAIMTQLRNEGWIHHLARHSAACF
LTRGDLWQSWVKGQEVFDELLLDADYSLNAANWMWLSSSAFFHQYYRVYSPIVFGKKTDQNGDYIRKYIPILE
RFPAQYIYEPWTAPRSVQEAAGCIIGRDYPRPIVDHSVVSKRNIGRMKDARACQPGKSAEKRPTDASNKNSNG
KVRKITSMLKKK
>Lytechinus_var_XP_041482616.1
MISFSYQGLKLGYQQQFCKQKKVVTLYRLFSSHSFCLIMGKDGETSKRSHEGQDDGEKGSVKKVKKEENEAEP
QDLQAKVANLRKAXGSSILNFKFNKKRVRILSDTMDTADDNQGIVYWMSRDQRVQDNWALLFAQRLAMKQEVP
LHVCFCLVPKFLDGTIRHYNFLLEGLKEVSQELHQLDIPFHLLTGYAKDVLPGFVKGHGLGAVVTDFSPLRTP
RQWVNDVKEELPKNIAFCQVDAHNVVPCWEASEKLEYGARTIRPKITKHLTTYLTEFPPVVCHPFKVKTKAEP
IDWEAAYASLDVDQTVKPVDWAKPGTSEGMKMLDSFTKERLRYFSSARNDPTKNVCSNLSPWIHFGQLSSQRA
ALIVRLYRSRYSESVASYLEESIVRRELSDNFCFYNDNYDSIEGTNEWAKKTLKDHAKDKREYVYSRDILERA
KTHDQLWNSAQKQMVREGKMHGFLRMYWAKKILEWTASPEEALEIAIYLNDRYSLDGRDPNGYVGCMWSICGI
HDQGWGERPVFGKIRFMNFKGCKRKFDVDTFINRYKKYSL
>Macrostomum_lig_PAA82046.1_BOX15_Mlig
SATPMERMQSCITDSNSPNDDHWMTLDGPAMSGGGVKHAVHWFRRDLRLHDNASLLAAARQADNLRCVFLLDC
QLERHQQHRYLAANKWRFLVDCLRDLDESLRRLGSRLFVVRGQPLEAFRRLLPLWGASHLSFEEDCTPLGRDR
DSAVTKLAHELGVTVLRGCSQTLYSQQVLMQASGGELPLTFAKFHRLLLTVGSPPAPCPSLQDEAATLPPPPP
PLTPQLHPGFHRGLSNSFSNNNKTSSSSNGNTCNAASFIDEDAFNLPTLGELGLDQDEANYQPTPVLIRGGET
EALARLTRHMERRSWVATYGGKPRYQSQLLMPSGTCLSSHLAFGCLSPKLVYAQLTDLYRKIKGGSSAQPPPL
HIYGELLWREYLYLTAHANPNFDRLEGNPMCLPIPWCRDQAAFGAWSTARTGVPWIDAIMTQIRLEGWAHPYC
RYSAVAFLTRGHLFQNWEDGAKLFEQLLIDAEWPTNNGLWLWMSGSAFFQAFGCAYSPVIAGRKLDPQGDYIR
HYLPQLARLPTRYLHEPWLAPPAVQEVAGCIIGRHYPAPMLPDLDEAAESCASRLRAVYAALLTQSGTPTAAS
AAAAAAAAGGSTLASMQPPPPPPPLTSRPSRPPFSQASSSSSTSSALAPPHLTMATGVQQQHQQHAEDFVLDM
LASPTIDDSMA

>Macrostomum_lig_PAA84477.1_BOX15_Mlig
QSKFKLEQLPKTKRRLDSANSSAEPEQQPKKKQRLDSAGSSAEPSSSTSTSPTQPLMEFSYASASVEEFRFAD
KRVRLLTKKVKDLLPDGRCVIYWMSRDQRVQDNWALIHAQRLALRSRLPLCVAFNLVPKFLDATIRQFDFLLR
GLEHVETECRRLDIDFRLLTGQPVDNLPRMVRDLSAAALVADFSPLRVPLNWANSVAAKLPTGVPMYQVDAHN
IVPVWLASDKLEYAARTIRPKIHRYLGEYLTEFPRIERHPYRLPEADRAKPVNWKAARDSLEVNMSVLPVKWA
EPGPVGASRTLAEFLQRRLRLFATGRNDPNVAALSNLSPWLHFGQISAQRCAFEAKKFRPSHKESVDAFIEES
VVRRELADNFCLHNSNYDSLEGAYDWARETLQRHSRDARPYLYTRQELEEGRTHDRLWNAAQLQMVREAKMHG
YLRMYWAKKILEWTESPAEALSISIYLNDKYNLDGRDPNGYVGCMWSICGVHDQGWGERAVFGKIRYMNYDGC
KRKFDVEKFSTLYCGAGPAAGSSRPSTSKAGDKKQQSTLLKYLSKPKNKKR
>Malus_dom_bluelightPb_XP_008359419.2
MDANRQIPENPESKSPEEQNPLAIVPSAAGLSPFATASLSLSLSTILPTHFFQQPKVSTLFSSQPTKVKVPTQ
ASSLAHLSLSTTANVTPPKLSFKSTIAANPLQSPLSLGPRRPLDPSNGAAIRRASIVWFRNDLRVHDNECLNS
ANNESVSVLPVYCFDPRDYGKSSSGFDKTGPYRATFLVESVADLRKNLQARGSDLVVRIGKPETVLVELAKAI
GADAIYAHREVSRDEVKEEEKIEAAMKEENVEVKYFWGSTLYHAEDLPFKLEDMPTKYGDFREKVKGLEVRKT
IEALDQMKGLPSRGDVEPGDVPTLMDLGLNPSATTSQDGRPAAIASVVGGETEALERLKKYAAECQAQPPKAS
KDGKHDSIYGANFSCKVSPWLVLGCLSPRSMFDELKKTSSRTISASSNRNDDGCSGMNWLMFELLWRDFFRFV
TCSSTKKQLNAAPATACTGALA
>Malus_dom_RXH69018.1_DVH24_0313
MSSGSSSLMWFRKGLRIHDNPALEYASKGSDFLYPVFVIDPHYMKPDPDAFSPGSSKAGLNRIRFLLESLSDL
DSNLKKLGSRLLVLKGEPSEVLNRCLKEWDVKRLCFEYDTEPYYQALDVKVKGYASAAGIEVFSPVSHTLFNP
ADIIRRNGGRPPLTYQSFVKLVGDPSWASSPLSITLSSLPPIGSFGRCEISEVPTVEELGYEKTQQDEHSPFK
GGESEALKKLRESIEKKEWVANFEKPKGDPSAFLTPATTVLSPYLKFGCLSSRYFYICLQDLYKNVKSHTSPP
VSLAGQLLWRDFFYTVAFGTPNFDRMRGNKICKQIPWNDDDELLQAWREARTGFPWIDAIMIQLRKWGWMHHL
ARHSVACFLTRGDLFVHWEKGRDVFERLLIDSDWAINNGNWLWLSCSSFFYQYNRIYSPTSFGKKYDPNGDYI
RHFLPVLKEMPKEYIYEPWTAPESIQKKAKCIIGRDYPKPVVAHDSASKECKRRMAEAYALNQKLTGLVSEED
LRNLRRKLEEDEKPEVKNKRQRKLIG
>Malus_dom_RXH73989.1_DVH24_0168
MSGGGCSVVWFRRDLRVEDNPALAAAVRAGGVVCVFIWAPDEEGPYYPGRVSRWWLKHSLAHLDSSLKSLGAS
LITKRSTDSVSSLLEVVVSTGATQLFFNHLYDPISLVRDHRAKEVLTAQGIAVRSFNADLLYEPWDVIDVHGR
PFTTFDAFWGRCLSMPYDPDAPLLPPKRIISGDASRSPSDTLVFEDESEKGSNALLARAWSPGWSNADKALTT
FINGPLLDYSNNRRKADGATTSLLSPHLHFGEVSVRKAFHLARMKQVVWANEGNKAGEESVNLFLKSIGLREY
SRYISFNHPYSHERPLLGHLKFFPWVLDQNYFKAWRQGRTGYPLVDAGMRELWATGWLHDRIRVVVSSFFVKV
LQLPWRWGMKYFWDTLLDADLESDALGWQYISGTLPDGREFDRIDNPQFEGYKFDPNGEYVRKWLPELARLPT
EWIHHPWNAPESVLQAAGIELGSNYPLPIVGIDAAKTRLQEALSEMWQHEAASRAAVENGTEEGLGDSSESVP
IAFSQDIQMEENNEPARNNPPGTRKYEDQMVPSITTSLVRAEEEETSLELQNLTEETRAEVPTDLIVNQEPRR
DILNQGFFQTIHNNALPQSNAAIGLQHAVEDSTAESSSSTRRERDGGVVPVWSPSSSSYSEQFGSEDNGIGTS
SYLQRHPQSHQIMNWRRLSQTG
>Malus_dom_XP_008369510.2_photolyase
MATNSATATVQPCRIRILKEGSKLADQISGPVVYWMFRDQRVRDNWALIHAIEHARKASVPVAVAFNLFDQFL
GAKARQLGFMLRGLQQLQSDLEETHQIPFFLFRGKAEETIPNFVGECGASLLVTDFSPLREVQKCKQEICKAV
SDFVTIHEVDAHNVVPIWAASEKLEYSARTIRSKINIKLPEYLVEFPKLQPPLTKWVGVNRLIDWDGLIDEVL
RKGAEVPEIDWCESGEKAAMEVLMGSKNGFLTKRLKGYSSDRNNPLKPGGLSGLSPYLHFGQLSAQRCALEAR
SRRKLCPEAVDAFLEELIVRRELADNFCFYQPHYDSLQGAWEWARKTLADHASDKREHIYTKEQLENAQTADP
LWNASQLEMVYYGKMHGFMRMYWAKKILEWTRGPEEALEISIYLNNKYEIDGRDPNGYVGCMWSISGVHDQGW
KERPIFGKIRYMNYAGCKRKFDVDGYISYVKRMLVETKKRKADSLLSRKAKEHCT
>Malus_dom_XP_028947742.1_cry-1-like
MSGNKTIVWFRRDLRIEDNPALAAAARDGSVFPVYIWCPKEEEQFYPGRVSRWWLKQSLAHLDQSLKSLGAEL
VLIKTQSTLAALIECIQAIGATKVVFNHLYDPVSLVGDHNIKGKLVELGISVQSFNADLLYEPWEVYAGKGQA
YTTFEVYWDKCLHIQRELVTRLPPWKLISATVIGTVAKCSLEELGLENETEKSSNALLRRAWSPGWSNADKAL
SEFFELHLLDYSNNRMKVGQNSTSLLSPYLHFGELSIRKVFQSARMKQILWAKEGNSTGEESVTLFLRAIGLR
EYSRYLCFNFPFTHERPLLSNLKFFPWQADQANFKAWRQGRTGYPLVDAGMRELWATGWIHNRIRVIVSSFSV
KVLLLPWRWGMKYFWDTLIDADLESDILGWQYISGSLPDGHELERLDSPEVQGSKFDPEGEYVRHWLPELSRL
PTEWIHHPWDAPDSVLKVSGVEFGFNYPKPIIEIDLARERLTSAIFKMWEMEAAAKAASSNGTDEVVVDNSIS
EDLPIPKVILKKTSPCPTYSSNDQKVPTCQNSKNNQFIRKRSKFMQDESPLPDNPHNPNEAGPSRTNEDTSST
AESSISKKQTTSRNSFSVPQSCSSSEGNPFMGCESSEMKQSWQEQSDMEQSSSKNGTIRDANLGMYLAGLFGG
REGMTDDSI
>Malus_dom_XP_028949158.1_cry_DASH,_
QTLCVCIVSVREVISALPLSPSSRLSLISPFDSPLIYSIHCYRQTMALTHCSFVVKFFNVSSASLSLLWPPNL
HVSNPTLCTRVSNPTAQNFLPLELKTRQWQMAYPHPWLAPHCLKKFIIRPFFPNPKPTISTERLNLFRPMSSS
SSSKPESPAAVSQVPGLDPVEMERVADQTLQRYSASSSAKRNGRGVAVVWFRNDLRLLDNEALYKAWVSSQEV

LAVYCVDPRIFGSTHYFGFPKTGALRAQFLIECLADLKKNLMKRGLNLLIQHGKPEEILPDLAKAFGAHTVYA
QKETCSEELNVERLVSKGLQRVALPSSSVQSSKPGSVNNPKLQLIWGTTMYHIDDLPFDTSSLPDVYTQFRKS
VEAKCTVRGCIKIPASLGPPPSVEDWGCVPSLDQFGLQSSIVSKGMKFVGGESAALGRVHEYFWKKDLVKIYK
HTRNGMLGPDYSTKFSPWLASGSLSPRFIYEEVKRYEKEREANDSTYWVLFELIWRDYFRFLSAKCGNSLFHL
GGPRKVEHRWSQEKTLFESWRDGHTGYPLIDANMKELSTTGFMSNRGRQIVCSFLVRDMGIDWRMGAEWFETC
LLDYDPCSNYGNWTYGAGVGNDPREDRYFSIPKQAQNYDPDGEYVAYWLPQLRSLPKDRRNFPGMAYIKPVVP
LKFGNASSRHQNQDRGSAAGGANFGGRQPRGQRR
>Manis_java__XP_017520962.1
MGVNAVHWFRKGLRLHDNPALKECIQGADTIRCVYILDPWFAGSSNVGINRWRFLLQCLEDLDANLRKLNSRL
FVIRGQPADVFPRLFKEWNISKLSIEYDSEPFGKERDAAIKKLATEAGVEVIVRISHTLYDLDKIIELNGGQP
PLTYKRFQTLISKMEPLEIPVETITSEVIENCTTPLSDDHDEKYGVPSLEELGFDTDGLPSAVWPGGETEALT
RLERHLERKAWVANFERPRMNANSLLASPTGLSPYLRFGCLSCRLFYFKLTDLYKKVKKNSSPPLSLYGQLLW
REFFYTAATNNPRFDKMEGNPICVQIPWDKNPEALAKWAEGRTGFPWIDAIMTQLRQEGWIHHLARHAVACFL
TRGDLWISWEEGMKVFEELLLDADWSINAGSWMWLSCSSFFQQFFHCYCPVGFGRRTDPNGDYIRRYLPVLRG
FPAKYIYDPWNAPEGIQKVAKCLIGVNYPKPMVNHAEASRLNIERMKQIYQQLSRYRGLGLLASVPSNPNGNG
GLMGYSPGENIPGCSSSGSCSQGSGILHYAPGDSQQTHLLKQGRSSMGTGLSSGKRPSQEEDTQSIGPKVQRQ
STN
>Manis_java__XP_017522607.1
MAAAVVTAAAAATAPADGADGASSVHWFRKGLRLHDNPALLAAVRGARCVRCVYILDPWFAASSSVGINRWRF
LLQSLEDLDTSLRKLNSRLFVVRGQPADVFPRLFKEWGVTRLTFEYDSEPFGKERDAAIMKMAKEAGVEVVTE
NSHTLYDLDRIIELNGQKPPLTYKRFQAIISRMELPKKPVGSMTSQQMESCRTEIQENHDDTYGVPSLEELGF
PTEGLGPTVWQGGETEALARLDKHLERKAWVASYERPRMNANSLLASPTGLSPYLRFGCLSCRLFYYRLWDLY
KKVKRNSTPPLSLFGQLLWREFFYTAATNNPRFDRMEGNPICIQIPWDCNPEALAKWAEGKTGFPWIDAIMTQ
LRQEGWIHHLARHAVACFLTRGDLWVSWESGVRVFDELLLDADFSVNAGSWMWLSCSAFFQQFFHCYCPVGFG
RRTDPSGDYIRRYLPRLKGFPSRYIYEPWNAPESIQKAAKCIIGVDYPRPIVNHAETSRLNIERMKQIYQQLS
RYRGLCLLASVPSCVEELSNPVAEPSSSQTGSMSSAGPRPSGPASPKRKLEAAEDPPGEEFSKRARVVELPTK
ELPSRDV
>Manis_pent__XP_036747204.1
MGVNAVHWFRKGLRLHDNPALKECIQGADTIRCVYILDPWFAGSSNVGINRWRFLLQCLEDLDANLRKLNSRL
FVIRGQPADVFPRLFKEWNISKLSIEYDSEPFGKERDAAIKKLATEAGVEVIVRISHTLYDLDKIIELNGGQP
PLTYKRFQTLISKMEPLEIPVETITSEVIEKCTTPLSDDHDEKYGVPSLEELGFDTDGLPSAVWPGGETEALT
RLERHLERKAWVANFERPRMNANSLLASPTGLSPYLRFGCLSCRLFYFKLTDLYKKVKKNSSPPLSLYGQLLW
REFFYTAATNNPRFDKMEGNPICVQIPWDKNPEALAKWAEGRTGFPWIDAIMTQLRQEGWIHHLARHAVACFL
TRGDLWISWEEGMKVFEELLLDADWSINAGSWMWLSCSSFFQQFFHCYCPVGFGRRTDPNGDYIRRYLPVLRG
FPAKYIYDPWNAPEGIQKVAKCLIGVNYPKPMVNHAEASRLNIERMKQIYQQLSRYRGLGLLASVPSNPNGNG
GLMGYSPGENIPGCSSSGSCSQGSGILHYAPGDSQQTHLLKQGRSSMGTGLCSGKRPSQEEDTQSIGPKVQRQ
STD
>Manis_pent__XP_036747632.1
MAAAVVTAVAAATAPADGADGASSVHWFRKGLRLHDNPALLAAVRGARCVRCVYILDPWFAASSSVGINRWRF
LLQSLEDLDTSLRKLNSRLFVVRGQPADVFPRLFKEWGVTRLTFEYDSEPFGKERDAAIMKMAKEAGVEVVTE
NSHTLYDLDRIIELNGQKPPLTYKRFQAIISRMELPKKPMGSVTSQQMESCRTEIQENHDDAYGVPSLEELGF
PTEVLGPTVWQGGETEALARLDKHLERKAWVASYERPRMNANSLLASPTGLSPYLRFGCLSCRLFYYRLWDLY
KKVKRNSTPPLSLFGQLLWREFFYTAATNNPRFDRMEGNPICIQIPWDRNPEALAKWAEGKTGFPWIDAIMTQ
LRQEGWIHHLARHAVACFLTRGDLWVSWESGVRVFDELLLDADFSVNAGSWMWLSCSAFFQQFFHCYCPVGFG
RRTDPSGDYIRRYLPRLKGFPSRYIYEPWNAPESIQKAAKCIIGVDYPRPIVNHAETSRLNIERMKQIYQQLS
RYRGLCLLASVPSCVEDLSNPVAEPSSSQTGSMSSAGPRPLPSGPASPKRKLEAAEDPPGEEFSKRARVVELP
TQELPSRDV
>Marchantia_pol_OAE26548.1_AXG93_3817
MAARTIVWFRRDLRVEDNPALVAAARAGIVIPVFIWSPEEDGQFHPGRVSRWWLKQSLIHLDASLKSLGSPLI
MRKSPDTLTVLLDIVQQVGATQVFYNHLYDPVSLVRDHRVKQGLSIHNIPVHTFNGDLLYEPWEVYDDEGQAF
TTFDDFWDKCMNMPFEPEAPLLSPRRLVGLPPGRISAGRIDDLGLENEYERSSNALLGRAWAPGWSSANKALE
VFMKGPLHDYATHRIKADNANTSQLSPHLHFGEVSVRKIFHEARTKQILWARENSYVGEQSVGMFLRAIGFRE
YSRYLSFNFPFTHERSLLANLKSFPWRIDENSFKSWRQGRTGYPLVDAGMRELWATGWLHNRIRVVVSSFCVK
FLQLPWRWGMKYFWDTLLDADLECDVLGWQYISGSLPDGHELDRMENPQIEGYKFDPEGEYVRRWLPELSRLP
TEWIHHPWDAPANVLRAAGVELGSNYPRPIVDISSARDQLQQSLAEMWEREAASKAALANGSEEGLGETVETP
GSCGPGVKMDVHRVVIRSSRAACSTASSRRDQLVPCMPTEDHKLAAASIMNWNQSPVDEEDEPVQPAFVPSQT
GGLLDRPSEVRAEVIAHPPSDSSMAEAVHVDNELNSTAESSAAVGRRSEESRGEVPVWPHSSSSRPQRPHLEG
LVPVVPEVRRATLNRRHLHSMQMVNLEARTSHKTSSLSA
>Marchantia_pol_PTQ29033.1_MARPO_0149

MVTVSCRFLLTLCGSLATSPASLRRSAVDFSSAVEFPGQGVCADFRCHLRGRSMSSTPKRKARVLQPSIKSAL
AGTKKEIEELEPAVKKLHQDVKKVAKSEGELDIVNVRSEFKVVEEVHKSRVRVLKEGDGTRKGPILYWMSRDQ
RSRDNWALLYAVQEAKKRGVSVAVVFNLVDNFLDAKARHFGFMLRGLRVVEKNLQAVEIPFFLFRGKAQETIP
AFAEECGASVVVMDFSPMRIGRVWREELCANSGPSVAVHEVDAHNVVPVWEASPKLEYGARTIRTKIHKLLPE
YLTDFPILKNSGQQWTSTPPPPVKWDELIDDVLSIGAEVPEVDWCVPGEDAAMEHLLGSKNGFLTKRLAKYEF
RNDPTKTSSLSNISPYLHYGQIAPQRAALECRNHRKQHLKMADSFFEELVVRRELSENFCYYQPNYDNVKGAW
DWAQKSLREHASDKREFVYTQEQLEKGKTHETLWNAAQLEMVHYGKMHGFMRMYWAKKILEWTESPEEALRIA
IYLNDKYELDGRDPNGYVGCMWSICGIHDQGWKERPVFGKIRYMNLAGCKRKFNVDGYVAYVDRLVSLAKKKA
KDSVKIAAAHKMS
>Marchantia_pol_PTQ32765.1_MARPO_0095
MPLVLKCTTILPPFAVSRIRIRTSVSRARHLCGVGESRTMGQHSLMWFRKGLRLHDNPALHKACEDASHVFPV
FVLDPFFLAPDPTAPSPGSRTAGVNRIHFLLQSLQDLDSSLKSRGSQLFLVHGNPTEVIPELLEKWSIKRLCF
EHDTEPYAQDRDKRIKEICEERGIELHSPVSHTLFNPAETILKNGGKPPLTYQAFCRTLGKPPKPVGDAPAAI
PEPSKDLMDVDVVPIPSLQDLGYADLNEEFTPFGGGETEGLRRLDKMLADKKWVAEFEKPKGNPTAFIKPATT
CLSPYLKFGCVSVRLFYARLLTVYSEMKKHSHPPVSLEGQLLWREFFYTVGYGTINYDRMVGNPICRQIPWKD
DEVLLSRWRDGQTGFPWIDAAMIQLRKWGWMHHLARHAVACFLTRGDLFVYWEKGRDVFDRLLVDSDWAINNG
NWMWLSASAFFAQYHRIYSPITFAKKHDPEGNYIKHFIPVLKDMPKAYIYEPWTAPLSVQKAANCIIGKDYPK
PVVDHAIANKKCRDRMGAAFALNKAAGSGQPTQEQVNALNEKLTAKLDEDSDASIAAESSDGEPPPSPKPKPK
SKRQRTLTEYKKK
>Marchantia_pol_PTQ48384.1
MANFDDLELILDSNVERASGGGVAEHFEFEKPLQSNGCSSHLRERVNTAETGSGDGEEEIVVASRDDLDSSKP
FMVPGAASLTMAMGSLLSLSHVSPPVHRLGFAPSKKVLKTDTSAKKTINSSPVVGASQMDVSMGSSITKTVKK
SVGSISPFPDYQRLHGSQFGEPRNPAGLRKATIVWFRNDLRVHDNEALVQASNDSMSIVPVYCFDPRDYGKSS
SGFDKTGPYRAKFLLECVSNLRDNLRERGSELIVRIGRPEEVLVSLAKTVGADALYAHLEVAHEEMNAEEKVS
AALKEEGVESKYFWGSTLFHVDDLPFKLEDMPSNYGGFRDNVQGVEVRATIEAPKHLKGLPVGGSVKAGEIPS
LQELGLNPSAINRPETPVSGARPGFSGNEPSDGELALNLALSLAKDFPQEEDEDEKHTAYGGALLIGGENEAL
QRLRSFVVEASSLSETKSRTADKAGESLYGANFSCKISPWLAMGCLSPRRMFEDLHQSNKKVAPASAAKCGVT
DDNGLNWLVFELLWRDFFRFITKKFGAGKKSSTPATACTGALSPA
>Marchantia_pol_PTQ49876.1_MARPO_0002
MLSSRGSKFLSSGSQLLASGSMQKSGASARPMHVMASKDGSGQKKGRQGTGTSVMWFRNDLRVSDNEALLRAW
QTSSAVLPVYCLDPRVFSTTHVFQFPKTGELRAQFILESVADLRKNLQRRGLDLVIRVGHPEEIVPSLAEAVG
AHTVFGHKETCSEETTVEGRLKGRLAKVNVPSEVPGKSVRHPQLDLVWGGTLYHIDDLPFSPNQLPDVYTQFR
KACETNSRIRPACKMPHQMGPLPDSVEKIGGCGPLPTLADVGLTPREQSPSGVMHFAGGETAALARLQDYFWV
KDKVRAYKETRNGMLGADYSTKFSPWLAHGCITPRTIGEELKKYEGERVANQSTYWVLFELLWRDYFRFLSIK
YGNSIFHLGGPRNVKGKSWGQDKLLFEAWRDGKTGYPLIDANMKELAQTGFMSNRGRQIVCSFLVRDMGLDWR
LGAEWFETCLLDYDPCSNYGNWTYGAGVGNDPREDRYFSIPKQAQTYDADGEYVAHWLPQLRHLPKTARHFPK
DAYLRPIVPLRHGQAPRNQPNSGRQPAFRRN
>Marine_Gro_OIR17544.1_BD935_0255
MNVGIIWFRNALRLNDNRVLVECLNSTNSQTILPLYILDKSDLEQNNNENKIKFLYESLIDLDANFKAKFGSN
LIVLNGKSKDIFRKLLDSDLLDLSEIFTDYSNKPDDIENENNLKSILAENASVKLHLISKVNSLTNVQEVVSQ
ENFKPPKTMKDMEKLFSNLYPKDEDGFYSIDKPLEIPENSKPIYENSSEIIKDYLFDVKKELSLLEKDSKSYF
KGGESEAIIRLERKVSSEEEYIRNFRKPRTISTNKPENPLEPETTGLSPYLSFGCLSPRLLWKETEKCYRNGE
HTQPPESMHGQLMFREMFYILSRAVENWDSDTNNSMCKKIEWGDFDKSKMELWEKGQTGFPYIDAMMRQLDAT
GWMHHLGRHAVSCFLTRGQLWQHWEQGRDVFDRKLVDADWALNNGNWLWLSGVAPFSMPYFRLYNPCPDGKSS
LNVEAKNAEFVRYWVPELKDYPSKYIFEPHLAPTPIQENARCIIGKDYPEPMVDRKVVAKENLAKFKASLATL
K
>Mastacembelus_arma__XP_026147489.1
MSAPRTIICLLRNDLRIHDSELFHWAQTHAEYIVPLYCFDPRHYMGTYNYNLPKTGPFRLHFLLESIRDLRNT
LLNKGGNLVVRRGKPEQVVAELIKQLGSVSTVAFHEEVTSEELNVEKKLKKACDQMEVKVHTFWGSTLYHRDD
LPFHHISGLPDVYTQFRRAVETRSRVRPLFPTPEQLKPLPPGLAEGAIPTAEDLQQTEPVSDPRSAFPCNGGE
TQALARLKHYFWDTNAVAMYKETRNGLIGVDYSTKFAPWLAVGCISPRYIYHQIKQYEKERTANQSTYWVIFE
LLWRDYFRFVGVKYGNRLFHVKGLQDKSVPWKKDMELFNAWKEGRTGVPFVDANMRELAMTGFMSNRGRQNVA
SFLTKDLGLDWRLGAEWFEYLLIDYDVCSNYGNWLYSAGVGNDPRENRKFNMIKQGLDYDSNGDFVRQWVPEL
QNIKGADVHTPWTLSTAALSHAHVSLGENYPTPIVVSPEWSRHVNNKLSGTGPSPKGKKGPCHTPKQHRDRGI
DFYFSRSKNL
>Mastacembelus_arma__XP_026166734.1
MVVNSVHWFRKGLRLHDNPALQEALNGASSLRCVYILDPWFAGAANVGINRWRFLLEALEDLDNSLKKLNSRL
FVVRGQPTDVFPRLFKEWKVTRLTFEYDPEPYGKERDGAIIKMAQEFGVETIVRNSHTLYNLDRIIEMNNNSP
PLTFKRFQTIVSRLELPRRPLLPITQQQMDKCHTKIADNHDQLYSIPSLEELGFRTEGLPPAVWRGGESEALD
RLNKHLDKKVWVANFEHSRVNTCSLYASPTGLSPYLRFGCLSCRVLYYNLRELYMKLRKHCSPPLSLFGQLLW

REFFYTAATNNPNFDRMEGNPICVQIPWDQNPEALAKWAEGRTGFPWIDAIMTQLREEGWIHHLARHAVACFL
TRGDLWISWESGMKVFEELLLDADWSVNAGSWMWLSCSAFFQQFFHCYCPVGFGRRTDPSGDYIRRYIPILKD
YPNRYIYEPWNAPESVQKAANCVVGVDYPKPMINHAEGSRLNIERMKQVYQQLSHYRGLSLLASVPTIQEEAE
PPMTDESQTSSGPDSPPRVAADSEATGCSTAPDSSTVCAFSTSILCPGLQDTANSLSCQTLCTSSSSHTEPSS
AIISSSANHPPSTSTPSSPVSTPVQSPLSKSKPLSPSLSCPTLSPSCIPATTPTQASGLGPKRKGLARKMRRS
QRQRGRQSCTLSAREGERRAEEEEREEVGGEERMEEDVELDEERMEEGTSGQTAHQQ
>Mastacembelus_arma__XP_026167449.1
MVVNTIHWFRKGLRLHDNPSLRDSIQGADTLRCIYILDPWFAGSSNVGINRWRFLLQCLEDLDASLRKLNSRL
FVIRGQPTDVFPRLFKEWQITRLSYEYDSEPFGKERDAAIQKLASEAGVEVIVRISHTLYDLDKLIELNGAQP
PLTYKHFQALINRMDAVELPVETITSEVLKTCATPISEDHDDKFGVPTLEELGFETEGLTTAVWPGGETEGLM
RLERHLERKAWVANFERPRMNANSLLASPTGLSPHLRFGCLSCRLFYFRLTDLYKKLKKNSTPPLSLYGQLLW
REFFYTTATNNACFDKMEGNPVCVQIPWDRNPEALAKWAEGQTGFPWIDAIMTQLRQEGWIHHLARHAVACFL
TRGDLWISWEEGMKVFEELLLDADWSVNAGSWMWLSCSSFFQQFFHCYCPVGFGRRTDPNGDYIRRYLPILRG
FSAKYIYDPWNAPEEIQKAAKCIIGVHYPKPMVNHAEASRINIERMKQIYQQLSCYRGLGLLATVPVNPNNAA
NGNNVTVVSTKTSQGPGRSTDDLPIQEGTSQTERRQSSQKRRHEEVSPKGNAKSWRQSK
>Mastacembelus_arma__XP_026169697.1
MFFCLWRRRSLKRLILSDPRSSQVLAFLQFSSSETIMSGRKRKAARVPAGEEPSAKQQKQQQQKQQQKQQKQQ
QKQQKQQQRAAGLVEQLRSEDKEMKFNEKRIRFMSDTEKIKQGSEGVLYWMSRDHRVQDNWALIHAHQLALKE
KLPLHVSFCLVVPKSELSTLRHYSFMFKGLEEVAKECKNLDIQFHLLHGPAGEVLPGFVSDHSLGAVVTDFSP
LREPLQWLEDVKKRLPKDIPLIQVDAHNIVPCWLASPKLEYSARTIRGKITNLLPEFLTDFPLVDKHPYTATR
TAKQINWDKTLASLQVDRTVGEPEWAKPGTKAGMAMLESFIDVRLKLFGTQRNDPNAAALSQLSPWIRFGQLS
AQRVALEVKRCGKNAGQSVSSFIEELVVRRELTDNFCFYNKKYDSVEGAYDWAQKTLKDHAKDKRPYLYTREQ
LENAKTHDKLWNAAQYQMVTEGKMHGFMRMYWAKKILEWTSSPEEALSIALYLNDRYELDGQDPNGFVGCMWS
ICGIHDQGWAERPIFGKIRYMNYKGCTRKFDVAQFERKYCPKKL
>Mastacembelus_arma__XP_026189555.1
MAHTCIHWFRKGLRLHDNPALMSALRDCKQLYPVFILDPYLHNNTCMSINRWRFLIGALKDLDCSLKNLDSRL
FVVRGKPEDVLPKLFTKWKVTKLTYEYDTEPYSLSRDQNVTALAKEHGVEVVYKISHTLYDTDRVIEENNGKA
PRTYKRMQAIVKALGPPKRPVPAPTTEDMKDVKTPCAENHEVKYGIPTLQELGLDAAAWTLELFPGGEQEALR
RLDQHMKRTGWVCSFEKPQTSPNSLSPSTTVLSPYVTFGCLSARTFWWRLTEVYQGKKHSDPPVSLHGQLLWR
EFFYTASVGIPNFNKMKGNPACTQVDWDTNPEFLAAWKEARTGFPFIDAIMTQLRQEGWIHHLARHAVACFLT
RGDLWVSWEEGQKVFEELLLDGDWALNAGNWQWLSASTFFHQFFRVYSPVAFGKKTDKNGDYIKKYIPLLKRF
PAQYIYEPWKAPHSVQQAAGCIVGKDYPRPIVQHEVVSKRNIQRMKLAYAKRSPDSAESPRENKSLSKKQGVK
RKIPSVIDMMKKKDRKKLTLV
>Mauremys_muti__KAH1171730.1
MAAAASTPVGLARSVHWFRRGLRLHDNPALQAALRDASSVRCIYILDPWFAASSAVGINRWRFLLQSLEDLDN
SLRKLGSCLFVVRGQPTDVFPRLFKEWRVTRLTFEYDSEPFGKERDAAIVKLAQEAGVEVVIENSHTLYDLDR
IIELNGHKPPLTYKRFQTIISRMELPKKPVSSITSQQMEKCKSEIQENHDDTYGVPSLEELGFPTDGLAPAVW
KGGETEALTRLDKHLERKAWVANYERPRMNANSLLASPTGLSPYLRFGCLSCRLFYYRLWELYKKVKRNNTPP
LSLYGQLLWREFFYTAATNNPKFDRMEGNPICIQIPWDRNPEALAKWAEGKTGFPWIDAIMTQLKQEGWIHHL
ARHAVACFLTRGDLWISWESGVRVFDELLLDADFSVNAGSWMWLSCSAFFQQFFHCYCPVGFGRRTDPSGDYV
RRYLPKLKGFPSRYIYEPWNAPESVQKAAKCIIGVDYPKPMVNHAETSRLNIERMKQIYQQLSRYRGLCLLAS
VPSCVEDLSSPVTDPASGQGSSTSTVQRLLHCGQTSPKRKHEGVEELCTEELYKRAKVTGLHISEIPIKNL
>Mauremys_muti__XP_044843194.1
MGVNAVHWFRKGLRLHDNPALRECIQGADSVRCVYILDPWFAGSSNVGINRWRFLLQCLEDLDANLRKLNSRL
FVIRGQPADVFPRLFKEWNIAKLSIEYDSEPFGKERDAAIKKLASEAGVEVIVRISHTLYDLDKIIELNGGQP
PLTYKRFQTLISRMEPLEMPVETITAEVMEKCTTPVSDDHDEKYGVPSLEELGFDTDGLPSAVWPGGETEALT
RLERHLERKAWVANFERPRMNANSLLASPTGLSPYLRFGCLSCRLFYFKLTDLYKKVKKNSSPPLSLYGQLLW
REFFYTAATNNPRFDKMEGNPICVQIPWDRNPEALAKWAEGRTGFPWIDAIMTQLRQEGWIHHLARHAVACFL
TRGDLWISWEEGMKVFEELLLDADWSVNAGSWMWLSCSSFFQQFFHCYCPVGFGRRTDPNGDYIRRYLPVLRG
FPAKYIYDPWNAPESIQKAAKCVIGVHYPKPMVNHAEASRLNIERMKQIYQQLSRYRGLGLLATVPSNPNGNG
NGNGNGGLMGYSPGENISGCSSASGAQMGSNDGHTVGIQTSSLEDSHAGSSGFQQHGYSQGSSILHYAQGDHQ
QSHLLQQGRTGSTGISTGKRPNPEKETQSIGPKVQRQSTN
>Mauremys_muti__XP_044852759.1
MKHISIHWFRKGLRLHDNPALLAAMTDCHTLYPIFILDPWFPKNMRVSVNRWRFLVESLRDLDESLQKLNSRL
FVVRGCPTEVFPVLFKEWKVTRLTFEIDTEPYSRQRDCEVVRLAAEHGIQVIQKVSNTLYDTDRVIAENNGKV
PLTYVRLQTLLAALGPPKRPVPAPTLENLKDCCTPCKDNHDAEYGIPTLEELGQDPEQAGPRLYPGGESEALC
RLDLHMNRTAWVCNFQKPQTEPNSLNPSTTVLSPYIKFGCLSVRTFWWRLAEIYQGRKHSNPPVSLHGQLLWR
EFFYTAGAGIPNFNKMEGNPVCVQVDWDNNPEHLRAWSEGRTGYPFIDAIMTQLRTEGWIHHLARHAVACFLT
RGDLWISWEQGQKVFEELLLDADWSLNAGNWQWLSASAFFHQFFRIYSPVAFGKKTDKGGEYIKKYLPFLRKF

PAEYIYEPWKAPRSVQEQAGCVIGRDYPKPVVVHEVVSKRNVERMKAAYARRSASATAQLEGGGGKKGAKRKT
PAGPSVAELLTKKPKT
>Mauremys_muti__XP_044861601.1
MSGAAPRTVICLLRNDLRYHDSEVLRWAQKNADYVIPLYCFDPRQYLKTYYYGFPKTGPHRLKFLLESVKDLR
QTLKKKGSNLVVRKGNPEDVVHDLITQLGTVAAVAFHEEATKEELDVEKALQRVCAQHGVKIQTFWASTLYHW
DDLPFRHISRLPDVYTQFRKAVEAQAKVRPTVQMPDQLKPLAPGVEDGCIPMPEEFGQKDPLTDPRTAFPCSG
GETQALMRLQHYFWDTNLVASYKETRNGLIGIDYSTKFAPWLALGCISPRYIYEQIRKYEKERTANQSTYWVI
FELLWRDYFRFVALKYGTRIFLLKGLQDKEVPWKKDPKLFDAWKEGRTGVPFVDANMRELAATGFMSNRGRQN
VASFLTKDLGLDWRMGAEWFEYLLVDYDVCSNYGNWLYSAGIGNDPRENRKFNMIKQGLDYDSNGDYVRLWVP
EIQCVKGGEIHTLWALSNAALSQAGIALGETYPLPVVLAREWSRHINQKPNKRDHPPREKKDPSDMPRRHKDR
GVDFYFSRNKDLS
>Mauremys_muti__XP_044870701.1
MPNMPHRTIHLFRKGFRLHDNPTLLAALESSEVVYPVYILDRKFLMSAMNMGTLRWHFLLQSLEDLQRNLTKL
GSCLLVIQGEYELVLRDHVKKWNITQVTLDAEIEPFYKEMERTIQVLGGEMGFDVLSLMGHTLYDIRRILDMN
GGTPPLTYKRFLHILSVLGDPDMPVRNLTTEDFQRCKPAPELPLAECYRVPLPEDLEIPRDSQSPWRGGETEG
LQRLEQHLKNQMSLLPLDCGAQRSLGGMSWGSSWNAVSLYWSLLMQGWIANFTKPKTIPNSLLPSTTGLSPYF
SLGCLSVRSFFYRLSNIYAQSKNHSLPPVSLQGQLLWREFFYTVASATPNFTKMAGNPICLQINWYQDAERLH
KWRMAQTGFPWIDAIMTQLHQEGWIHHLARHAVACFLTRGDLWISWEEGMKVFEEMLLDADYSINAGNWMWLS
ASAFFHQYTRIFCPVRFGKRTDPEGQYIRKYLPVLTNFPSKYIYEPWTAPEEAQKQAGCIIGQDYPLPMINHK
EASDHNLQLMKQVREEQYGTAQLTRDDMDDPMEMGLKRERSEENSKKGKRARNREQT
>Mauremys_muti__XP_044887525.1
MSQKSRNRQARGGEEITASPAGSGDAARTGAAAVPRGQLKGKRKVLAEPSGTEVLTVDKRRKEGEVAEGGGAG
SLGEAVRQARLRVAPSVQDFKYNKKRVRLVSQGSDLKDAAKGVVYWMSRDQRVQDNWAFLYAQRLALKQQLPL
HVCFCLVPKFLEATIRHFGFMLKGLQEVAEECQELDIPFHLLIGFAKDVLPAFVAGRGLGGVVTDFSPLRVPM
QWVEEVKERLPGDVPFVQVDAHNIVPCWVASDKQEYGARTIRRKIHDRLSEFLTEFPPVIKHPYSSAAPAEPI
DWNACHASLQVDCSVKEVTWATPGTAAGLAVLESFIGERLKSFGTDRNNPNRAALSNLSPWFHFGQVSGQRAI
LEVQKYRSKYKESVDGFIEEAVVRRELADNFCYYNRNYDKVEGAYDWAKTTLKLHAQDKRSHLYELEQLEEGK
THDPLWNAAQLQMVHEGKMHGFLRMYWAKKILEWTRSPEEALKFAIYLNDRYELDGRDPNGYVGCMWSICGIH
DQGWAERAVFGKIRYMNYAGCKRKFDVGQFERRYDPRKLGQ
>Maylandia_zebr__XP_004539639.2
MAHTCIHWFRKGLRLHDNPALMAALKDCKQLYPVFILDPYLHNNACVGINRWRFLIGALKDLDGSLRKLNSRL
FVVRGKPEDVLPKLFTKWKVTRLTYEYDTEPYSLQRDSKVTSLAKERGVEVIYKVSHTLYNIERIIEENNGKP
PLTYTKLQAIAKTIGPPKRPIPAPTMDDMKDVKTPSSENHEKEYGIPTLEELGLDTAPLGEDLFPGGEQEALR
RLDEHMKRTKWVCTFEKPQTSPNSLSPSTTVLSPYVTFGCLSVRTFWWRLTEVYRGNKHSDPPVSLHGQLLWR
EFFYTASLGIPSFNKMEGNSVCTQVDWDTNTDYLAAWREARTGYPFIDAIMTQLRQEGWIHHLARHAVACFLT
RGDLWISWEEGQKVFEQLLLDGDWALNAGNWQWLSASTFFHQYFRVYSPVAFGKKTDKNGDYIRKYLPLLKKF
PAEYIYEPWKAPRSIQQAAGCIVGKDYPHPIVQHEVISKKNIQRMKLAYAKRSPESPSKSKGVKRKAPSIEII
KKKAKVK
>Maylandia_zebr__XP_004546814.1
MSSSRTVICLLRNDLRLHDNELFHWAQRNAEHIVPLYCFDPRHYVGTYNYSLPKTGPFRLRFLLESIRDLRNT
LISKGSNLVVRRGKPEEVVADLVRQLGSVSSVAFHEEVTSEELNVEKRVKDVCAQMKVKVHTCWGSTLYHRDD
LPFPHMSRLPDVYTQFRKAVESEGRVRPVFSTPEKLNPLPPGLEEGAIPTAEDLQQTEPVIDPRSAFPCSGGE
SHALARLKHYFWDTDAVATYKETRNGLIGVDYSTKFSPWLAMGCISPRYIYHQIKQYEKERTANQSTYWVIFE
LLWRDYFRFVSVKYGNRIFQVKGLQDKSVPWKKDMKLFDAWKEGRTGVPFVDANMRELAATGFMSNRGRQNVA
SFLTKDLGLDWRMGAEWFESLLIDHDVCSNYGNWLYSAGIGNDPRENRKFNMIKQGLDYDNNGDYVRQWVPEL
QNIRGGDVHTPWSLSNAALAHAHVTLGDTYPAPIVTAPEWSRHINKKAGGTGPSPRGKKGPSHTPKQHRDRGI
DFYFSRSKNL
>Maylandia_zebr__XP_004553340.2
MSVTFYSKMPPSGADAQATVKWWLGEVLEGREDPEGFFAMCVSILGQVETRSQFLSLIEPLSTGDRLLHASLT
SIYQEYFTKTEDDELELVLALSLLDMKGQRGSVQLNSILKPEGSNCTNQTGSWNRESPPQTAREMEFQISTYP
NKQTLGTGQTVCVSEQEGDVDQSQKPKRSKNRRQRRKCTGQHLVGLPRSPSAPPPVLLWFRRDLRLCDNPALV
ASLEVGAPVIPVFIWSPKEEEGTGITVAMGGACKYWLHQALLCFCSSLEHIGSRLVVLKANGEMTSTESSLKA
LKELIKETGARTVLANALYEPWLKERDDLVVSALQKQGVECRMFHSYCLRDPYSVTTEGVGLRGIGSVSHFMS
CCRQNPGTAVGVPLDPPVSLPTPAHWPKGLCLDELGLARMPRRKDGTTVDWAANIRKTWDFSEGGAQARLEAF
LHDGVYRYEKESGRADAPNTSCLSPYLHFGQLSPRWLMWDAKGARCRPLKFQRKLAWRDLAYWQLTLFPDLPW
ESIRPPYKALRWSSDRGHLKAWQRGRTGYPLVDAAMKQLWQTGWMNNYMRHVVASFLIAYLYLPWQEGYRWFQ
DTLVDADVAIDAMMWQNGGMCGLDHWNFVMHPVDAAMTCDPYGTYVRKWCPELAELPDELIHKPWKCPASMLR
RAGVVFGQSYPERIITDLEERRNQSLQDVALVRREFEQYVDKRSGCDLVPLPPRLVSEALGLSHKDGAVVTQG
KQFLLPVITRMEFKHQHEDPDADATSNPYNAVLKGYVSRKRDETIAFLNKRDFTASVMHEAVQRKERLNSDYR
RMEGLPPSPSPRGRARRTPTAKDRFSIVPGGAVTSLK

>Maylandia_zebr__XP_004556263.1
MVVNSVHWFRKCLRLHDNPALQEALNGADAVRCVYILDPFFAGAANVGINRWRFLLEALEDLDSSLKKLNSRL
FVVRGQPTDVFPRLFKEWNVTRLTFEYDPEPYGKERDGAIIKLAQEFGVETIVRNSHTLYNLDRIIEMNNNSP
PLTFKRFQTIVSRLELPRRPLPPITQQQMDKCHTKIEDNHDQLYSIPSLEELGFRTDGLPLAVWHGGESQALD
RLGKHLDKKVWVTSLEHSRVKTCSLYASPTGLSPYLRFGCLSCRVLYYNLRELYMKVRKRCTPPLSLFGQLLW
REFFYTAATNNPNFDRMDGNPICVQIPWDQNPEALAKWAEGRTGFPWIDAIMTQLRQEGWIHHLARHAVACFL
TRGDLWISWESGMKVFEEMLLDADWSVNAGSWMWLSCSAFFQQFFHCYCPVGFGRRTDPTGDYIRHYIPILKD
YPNRYIYEPWNAPESLQKAANCVVGVDYPKPMINHAESSRLNIERMKQVYQQLSHYRGLSLLASVPTIQEEAE
PPMTDESQTSSGPDSPQRVPANTEEDVCYEAPDSSTVCPSSTSAPHPDQEYQAPCISSVILIGNKEKRP
>Maylandia_zebr__XP_004570537.1
MLHCVWRSTSPFSAPGRFLKLLPHHHQRPSSPALFFLTSFFSCQPTMSGTKRKASSATAGKDHRAKQQKLAPV
KQEKKEKVGGWLQSLVQLQRSEKKDVKFNKKRLRFLSDTEKIKQGSEGVLYWMLRDQRVQDNWALIHAQRLAV
KENLPLHVCFCLVVPKSELSTLRHYSFMLKGLEEVAKECEQLNVQFHLLHGAPEKVLPGFVSDHSFGAVVTDF
SPIREPLQWLEDVKKKLSKDIPFIQVDAHNIVPCWVASPKQEYSARTIRGKITNLLPEFLTDFPLVDAHPHTA
TRTAKGVDWDKTLASLDVDRNVKEPEWAKPGTKAGMDMLESFIDVRLKLFGSQRNDPNAPALSQLSPWLRFGQ
ISAQRVAWQVQRNGKNASQSVPAFIEELVVRRELTDNFCFYNKKYDSVEGAYEWAQKTLKDHAKDERPYLYTR
EQLEKAKTHDKLWNAAQYQMVTEGKMHGFLRMYWAKKILEWTSSPEEALSIALYLNDRYELDGQDPNGFVGCM
WSICGIHDQGWAERAVFGKIRYMNYKGCLRKFNVAQFERKYCPKSL
>Maylandia_zebr__XP_004570852.1
MVVNTIHWFRKGLRLHDNPSLKDSIQGSDTLRCIYILDPWFAGSSNVGINRWRFLLHCLEDLDASLRKLNSRL
FVIRGQPTDVFPRLFKEWQINRLSYEYDSEPFGKERDAAIQKLANEAGVEVTVRISHTLYNLDKIIELNGNQP
PLTYKRFQAVINRMDAVEMPAETITSEVLKNCVTPISEDHDEKFGVPSLEELGFETEGLTTAVWPGGETEALM
RLERHLERKAWVANFERPRMNANSLLASPTGLSPYLRFGCLSCRLFYFKLTDLYRKVKKNHTPPLSLYGQLLW
REFFYTTATNNPCFDKMDGNPICVQIPWDRNPEALAKWAEGRTGFPWIDAIMTQLRQEGWIHHLARHGVACFL
TRGDLWISWEEGMKVFEELLLDADWSVNAGSWMWLSCSSFFQQFFHCYCPVGFGRRTDPNGDYIRRYLPILRG
FPAKYIYDPWNAPDEVQKAAKCIIGVHYPKPMVNHAEASRINIERMKQIYQQLSCYRGLGLLATVPANSNFGG
NGTNPRAVCTVTNEGAGGSSKDPSVQVGMSQTERAQSVQKRRHEETPLESSSKSWKQSK
>Megalops_atla__KAG7454755.1
MVVNTIHWFRKGLRLHDNPSLRESIQGADTVRCVYILDPWFAGSSNVGISRWRFLLQCLEDLDASLRKLNSRL
FVIRGQPTDVFPRLFKEWNITRLSYEYDSEPFGKERDAAIRKLASEAQVEVTVCISHTLYDLDKIIELNGGQS
PLTYKRFQAVISRMGAVETPAEAITAEVMGNCTTPIGDDHDEKFGVPSLEELGFDTEGLASAVWPGGETEALT
RLERHLERKAWVANFERPRMNANSLLASPTGLSPYLRFGCLSCRLFYFKLTDLYKKVKRNSSPPLSLYGQLLW
REFFYTAATNNPRFDKMEGNPICVQIPWDRNPEALAKWAEGRTGFPWIDAIMTQLRQEGWIHHLARHAVACFL
TRGDLWISWEEGMKVFEELLLDADWSVNAGSWMWLSCSSFFQQFFHCYCPVGFGRRTDPNGDYIRRYLPILRG
FPAKYIYDPLNAPDSVQKAAKCVIGVQYPKPMVNHAEASRLNIERMRQIYQQLSCYRGLGLLATVPSNPNGSG
EGTGVTMAGPAVEVSQEGMASGCQMTVKPQTEWGAGALLYPQGDHQPSGGVQPQGLTCSSSAVCYAESQPGHS
AGLQQGHHTSALSTVKRHSSDALANISSKVQRQNSN
>Megalops_atla__KAG7456922.1
MQYYRFLFQPVLRNISQCVSPIRQRQRPNSVPFQIRLRLQQCVSMQEGGSKVTAKNGGEGKVASKRKGEPVEA
GGKKQPKVVPPKEGKKKREEGWLESEVTEARRAAQGVKFNEKRLRFLSEAQKVKQGTQGVLYWMSRDQRVQDN
WALIYAQQLAVTEELPLHVCFCLQPQSPDATLREYSFILKGLEEIAKECSVLGIEFHLLLGTAGEVLPGFVRD
WSLGAVVTDFSPLRIPLKWVEDVKKGLPTDVPFMQVDAHNVVPCWVASDKQEYSARTIRGKITKLLPEFLTQF
PLVDKHPHSASKPAKLVDWASTLSTVEVDRSVGEVEWARPGTAAGMAMLESFMDTRLRDYASERNNPNSDALS
QLSPWIHFGHISAQRAVLQVQRNGANRAQSVPAFVEELVVRRELADNFCFYNKNYDKVEGASDWARKTLMDHA
KDPRQYVYSKEQLEKAKTHDRLWNAAQYQMVTEGKMHGFLRMYWAKKILEWTSSPEEALAIAIYLNDRYELDG
RDPNGFVGCMWSICGIHDQGWKERPIFGKIRYMNYAGCTRKFNVQQFEKQYCPK
>Megalops_atla__KAG7467932.1
MVVNSVHWFRKGLRLHDNPSLQEALNGADTVRCVYILDPWFAGSANVGVNKWRFLLESLEDLDASLRKLNSRL
FVVRGQPADVFPRLFKEWNVTRLTFEYDSEPYGKERDAAIIKMAQEFGVEVRVKNSHTLYNLDRIIELNNNSP
PLTFKRFQAIISRLGLPKRPLATITKQQMDSCRTDISDNHNERYGVPSLEELGFETHGLSPAVWKGGETEALE
RLNKHLDRKAWVANFERLRINICSLMASPTGLSPYLRFGCLSCRLFYYNLRELYMKVRKHSSPPLSLFGQLLW
REFFYTAATNNPNFDRMEGNPICVQIPWDRNPEALAKWAEAKTGFPWIDAIMTQLRQEGWIHHLARHAVACFL
TRGDLWLSWESGMRVFEELLLDADYSVNAGSWMWLSCSAFFQQFFHCYCPVGFGRRTDPSGDYIRRYLPKLKD
YPNRYIYEPWNAPEPVQKAANCVVGVDYPKPMINHAEGSRLNIERMKQIYQQLSHYKGLSLLASVPSIQEEAE
PPMTDSSNPQSSTSVLSATRQSKQPTMKKRARPSELQRDDKCTQPKVPRASMAEEKPGDKQ
>Megalops_atla__KAG7473567.1
MSTKDEVKPVAHVKHFMRELLLGRQDPEEFFCLSLSLLGHQYTQEQFLDLIEPLAEGHEELHTCLVTIFLDYF
TKVQSGKGVEEEELELALTLSMQEMREGELAAQGPLSHAQRLSYAQRAIRGGRVAQKNGNSHCSRAPVPCDME
QAQRERCTEGLIGKITNIQNESVCVTGTEVRYPSLSRSDEDVPVGINSSQDEKSKRSRRRQRKRQQLAMSLS
PSSPRPVLLWFRRDLRLNDNPALIGSLEVGGPVIPVFLWCPEEEEGPGVTVAVGGASKYWLHHALLSLNHSLE

RLGSHLVTVKVETSSQEALQRLVTDTGAGTLLATALYEPWLKERDDLVFTALQRQGVKCQLYHSYCLRDPNSV
STEGVGLRGIGSVSHFMSCCQQNPGPGLGTPVDAPLCLPVPSAWPQGCHLSQLALARMPRRKDGTVVDWATNI
RSSWDFSEEGAQARLQAFLRDGIYRYEKESCRADAPNTSSLSPYLHYGQISPRSLLSESRSARCRPPKFQRKL
AWRDLAYWQLTLFPDLPWESLRPPYKALRWSSDRAHLKAWQRGRTGYPLVDASMRQLWLTGWMNNYMRHVVAS
FLIAYLHLPWQEGYRWFQDTLVDADVAIDAMMWQNGGMCGLDHWNFVMHPVCAALTCDPCGSFVRKWCPELAE
LPDEFIHKPWQCPTSLLRRAGVVLGQNYPERIVTDLEERRAQSLQDVAVVRRQFGQYVDRHSGCDLIPLPDRL
VKEALGRGDIVQSGSRFLLPVITRMEFKHQAHDTDAKSNPFDAVLKGYVSRKRDETIAFLNERDFTASVLSEN
AQRRDRQERDQRALEGLPHPPAAHSRARRTPSKDIFSVVPAGVTVRHK
>Megalops_atla__KAG7477630.1
MRLSPPYRQAVGRETGGRGRPKPRESDRDASAMTHRTIHLFRKGLRLHDNPTLVAALESSEALYPVYVLDRAF
LEGAMRTGALRWRFLLQSLRDLQRGLAALGSRLYVLRGRHEAVLRQLVRRWGVTQLTFDAEVEPFHAQLEGSL
RTLAQELGLSVQSRVAHTLYDVKRIIKANGGKPPLTYKKFLHVLSVLGEPDKPAREITHEDFEKCKTPIEEGS
EDEYQVPTLEDLGIEVDVEVLWPGGETQGLKRLEKHLESQGWVVNFSKPRTIPNSLLPSTTGLSPYFSLGCLS
VRSFYYRLSNIYAQSKNHSLPPVSLQGQVLWREFFYTVASATPNFTKMAGNPICLQINWYENQEALDKWRTAQ
TGFPWIDAIMTQLRQEGWIHHLARHAVACFLTRGDLWISWEEGMKVFEEHLLDADYSVNAGNWMWLSASAFFH
QYTRIFCPVRFGRRTDPDGQYLRKYLPVLRNFPSKYIYEPWTAPLEVQEEAGCIIGKDYPLPMVDHSEVSQRN
LALMRQVRSEQENTAQLTRDLADDPMEVGVKREHQDEQGGVTEDHSEKPERASKRFSPDGDCKALSCSWASDS
IRVPELSGEVM
>Megalops_atla__KAG7484496.1
MSPLIPAHLIGMCLMSSSGCPADMRHISVHWFRKGLRLHDNPALLAALQDCTEIYPVFVLDPGVPNQAHASIN
CWRFLTGALKDLDCSLKKLNSRLFVLRGKPEEVFPRLFQRWKVTRLTFELDTEPYSQDQDEKVVKLAKQHNVE
VIQKVSHTLFDTERIIAKNNGKPPLTFNRLQAVVQSLGAPEKPVPPPTLEDMKGVRTPCSEKDEESYKVPTLE
ELGLDPLSAGPELYPGGEQEALRRLDEHMKRTGWVCHFQKPETSPNALSPSTTVLSPYVRYGCLSVRTFWWRL
TDVYQGRKHSQPPVSLHGQLLWREFFYTTSVGIPNFNRMEGNPICLQVDWDSNPEHLAAWREARTGFPFIDAI
MTQLRQEGWIHHLARHAVACFLTRGDLWISWEEGQKVFEELLLDGDWALNAGNWQWLSASAFFHQYYRVYSPI
AFGKKTDKSGEYIKKYLPILKKFPADYIYEPWKAPRSVQERAGCIVGKDYPRPIVDHEVVSKENMQRMKMAYS
KRSKTFNESNGKAKGEKRKAASVVDMLMKKKVKVN
>Megalops_atla__KAG7488658.1
MSAARTIICLLRNDLRLHDNEVFHWAQRNAEHIVPLYCFDPRHYLGTYCYNFPKTGPFRLGFLLDSVKDLRAT
LQKKGSNLLVRQGKPEEVVGDLIRQLGSVSAVAFQEEVTQEELDVEKGVKDVCSQLKVKVQTFWGSTLYHGED
LPFNHISRLPDVYTEFRKAVESRCRVRPVLPTPDLLKPLPPGLEEGPIPTHQDLEQLDAPADSRSAFPCSGGE
SQALARLQHYFWDTDAVASYKETRNGLIGVNYSTKFAPWLAMGCISPRYIYEQIKKYERERTANQSTYWVIFE
LLWRDYFKFVAMKYGNKIFALEGLQGKSVPWKKDMTLFNAWKEGRTGVPFVDANMRELAQTGFMSNRGRQNVA
SFLTKDLGLDWRMGAEWFQYLLVDYDVCSNYGNWLYSAGIGNDPRENRKFNMIKQGLDYDNNGDYVRQWVPEL
RGIKGGDVHTPWTLSTAALSHAQVSLGETYPSPIVMAPEWSRHVNKKPSGAGPSGAGPSQGRKRGPSHTPKQH
RDRGIDFYFSRSKNL
>Megalops_cyprinoides_XP_036399197.1_cryptochro
MVVNSVHWFRKGLRLHDNPSLQEALNGADTVRCVYILDPWFAGSANVGVNKWRFLLESLEDLDASLRKLNSRL
FVVRGQPADVFPRLFKEWNVTRLTFEYDSEPYGKERDAAIIKMAQEFGVEVRVKNSHTLYNLDRIIELNNSSP
PLTFKRFQAIISRLGLPKRPLATVTKQQMDSCRADISDNHNERYGVPSLEELGFETHGLSPAVWKGGETEALE
RLNKHLDRKAWVANFERLRINICSLMASPTGLSPYLRFGCLSCRLFYYNLRELYMKVRKHSSPPLSLFGQLLW
REFFYTAATNNPNFDRMEGNPICVQIPWDRNPEALAKWAEAKTGFPWIDAIMTQLRQEGWIHHLARHAVACFL
TRGDLWLSWESGMRVFEELLLDADYSVNAGSWMWLSCSAFFQQFFHCYCPVGFGRRTDPSGDYIRRYLPKLKD
YPNRYIYEPWNAPEPVQKAANCVVGVDYPKPMINHAEGSRLNIERMKQIYQQLSHYKGLSLLASVPSIQEEVE
PPITDNSNPQSSTSVLSTTRQSEQPTMKKRARPSELQRDDKCTQPKVPRASMAEEKPGDKQ
>Megalops_cyprin_XP_036386825.1
MTHRTIHLFRKGLRLHDNPTLVAALESSEALYPVYVLDRAFLEGAMRMGALRWRFLLQSLRDLQRGLAALGSR
LYVLQGRHEAVLRELVRRWGVTQLTFDAEVEPFYARLDDSLRALAQELGLSVQSCVAHTLYDVKRIIKANGGK
PPLTYKKFLQVLSVLGEPDKPAREITRQDFEKCKTPTEEGSENEYQVPTLEDLGIEVDVEVLWPGGETQGLKR
LEKHLESQGWVVNFSKPRTIPNSLLPSTTGLSPYFSLGCLSVRSFYYRLSNIYAQSKNHSLPPVSLQGQVLWR
EFFYTVASATPNFTKMAGNPICLQINWYENQEALDKWRTAQTGFPWIDAIMTQLRQEGWIHHLARHAVACFLT
RGDLWISWEEGMKVFEEYLLDADYSVNAGNWMWLSASAFFHQYTRIFCPVRFGRRTDPDGQYLRKYLPALKNF
PSKYIYEPWTAPPEVQEEAGCIVGKDYPLPMVDHSEVSQRNLALMRQVRSEQENTAQLTRDLADDPMEVGVKR
EHQDDQGSVTQDHSEKPERASKRFSPDSDCKALSYSWASDGMRLPELSGEVM
>Megalops_cyp_XP_036377802.1_cry_DASH
MSAARTIICLLRNDLRLHDNEVFHWAQRNAEHIVPLYCFDPRHYLGTYCYNFPKTGPFRLSFLLDSVKDLRAT
LQKKGSNLLVRQGKPEEVVGDLIRQLGSVSAVAFQEEVTQEELDVEKGVKDVCSQLKVKVQTFWGSTLYHGED
LPFNHISRLPDVYTEFRKAVETRCRVRPVLPTPDLLKPLPPGLEEGPVPTHQDLGQLDAPADSRSAFPCSGGE
SQALARLQHYFWDTDAVASYKETRNGLIGVNYSTKFAPWLAMGCISPRYIYEQIKKYERERTANQSTYWVVFE
LLWRDYFKFVAMKYGNKIFALEGLQGKSVPWKKDMTLFNAWKEGQTGVPFVDANMRELAQTGFMSNRGRQNVA

SFLTKDLGLDWRMGAEWFQYLLVDHDVCSNYGNWLYSAGIGNDPRENRKFNMIKQGLDYDNNGDYVRQWVPEL
RGIKGGDVHTPWTLSTAALSHAQVSLGETYPSPIVMAPEWSRHVNKKPSGAAPSQGRKRGPSHTPKQHRDKGI
DFYFSRSKNL
>Megalops_cyp_XP_036379574.1_cry_circad
MRHITIHWFRKGLRLHDNPALLAALQGCTEIYPVFVLDPGVPNQAHASINCWRFLIGALEDLDCSLKKLNSRL
LVLRGKPEEVFPRLFQRWKVTRLTFEVDTEPYSQHRDEKVVKLAKQHNVEVIQKVSHTLYDTERIIAKNNGKP
PLTFNRLQAVVQSLGAPEKPVPPPMLEDMKGVRTPCSEKDEESYKVPTLEELGLDPLSAGPELYPGGEQEALR
RLDEHMKRTGWVCHFQKPETSPNALSPSTTVLSPYVRYGCLSARTFWWRLTDVYQGRKHSQPPVSLHGQLLWR
EFFYTTSVGIPNFNRMEGNPVCLQVDWDRNPEHLAAWREARTGFPFIDAIMTQLRQEGWIHHLARHAVACFLT
RGDLWISWEEGQKVFEELLLDGDWALNAGNWQWLSASAFFHQYYRVYSPIAFGKKTDKSGEYIKKYLPILKKF
PAEYIYEPWKAPRSVQERAGCIVGKDYPRPIVDHEVVSKKNMQRMKMAYSKRSETFNESNGKAKGEKRKAASV
VDMLMKKKVKVN
>Megalops_cyp_XP_036386216.1_cry_circad
MAPNSIHWFRKGLRLHDNPALREAVRGAATVRCVYFLDPWFAGSSNLGVNRWRFLLQCLEDLDASLRKLNSRL
FVIRGQPANVFPRLFKEWKISRLTFEYDSEPFGKERDAAIRKLAMEAGVEVIVKTSHTLYDLDKIIELNGGQP
PLTYKRFQTLISRMDPPELPVEPLSDAFMGRCVTPVGDDHGDKYGVPSLEELGFDTEGLPSAVWPGGETEALT
RIERHLERKAWVANFERPRMNANSLLASPTGLSPYLRFGCLSCRLFYFKLTDLYRKVKKNSSPPLSLYGQLLW
REFFYTAATNNPRFDKMEGNPICVRIPWDWNAEALAKWAEARTGFPWIDAIMTQLRQEGWIHHLARHAVACFL
TRGDLWISWEEGMKVFEEMLLDADWSVNAGSWMWLSCSSFFQQFFHCYCPVGFGRRTDPNGDFIRRYLPVLRG
FPAKYIYDPWNAPHSVQTAAKCIIGIHYPKPMVNHAEASRLNIERMKQIYQQLSRYRGLGLLASVPSNHNGNG
NGNGNGHGNGSGHGSGNGAMMAYSPGEQQPGISTPHGATVSGVNGSSSILLNFGGDERPGPSGMQQHGYHTVS
DQGQGVAGGHRLYQPSSSHEFAVPQHPGRLGHAAGSGTGKREREAERESGADSDPPSSALKVQRPNSEKSGDL
QDVNMVSF
>Megalops_cyp_XP_036391499.1_photolyase
MSTKDEVKPVAHVKHFMRELLLGRQDPEEFFCLSLSLLGHQYTQEQFLDLIEPLAEGHEELHACLVTIFLDYF
TKVQSGKGVEEEELELALTLSMQEMREGELAAQGPLSHVQDLSYAQRAIRGGKVVQRNGNSHCSRAPVPCATE
QAQTERYTEGLIGKITNCQNENMCVTVTEVMYPPLSRSDEDVPVEINSSQDEKSKRSRRRQRKRQQLAMSLS
PSSPRPVLLWFRRDLRLNDNPALISSLEVGGPVIPVFLWCPEEEEGPGVTVAVGGASKYWLHHALLSLNHSLE
RLGSHLVTVKVETSSQEALQRLVADTGAGTLLTTALYEPWLKERDDLVFTALLRQGVKCQFYHSYCLRDPNSV
STEGVGLRGIGSVSHFMSCCQQNPGPGLGTPVDAPLCLPVPSAWPQGSHLSQLGLARMPQRKDGTVVDWATNI
RSSWDFSEEGAQARLQAFLQDGIYRYEKESCRADVPNTSSLSPYLHYGQISPRSLLSGSRSARCRPPKFQRKL
AWRDLAYWQLTLFPDLPWESLRPPYKALRWSTDRAHLKAWQRGRTGYPLVDASMRQLWLTGWMNNYMRHVVAS
FLIAYLHLPWQEGYRWFQDTLVDADVAIDAMMWQNGGMCGLDHWNFVMHPVSAALTCDPCGSFVRKWCPELAE
LPDEFIHKPWQCPTSLLRRAGVVLGQNYPDRIVTDLEERRAQSLKDVAVVRKQFGQYVDRHSGCDLIPLPDRL
VKEALGGGDLMQSGSRFLLPVITRMEFKHQAHDADAKSNPFDAVLKGYVSRKRDETIAFLNERDFTASVLSEN
AQRRDRQERDQRALEGLPHPPATRSRARRTPSKDVFSVVPAGVTALHK
>Megalops_cyp_XP_036410274.1_CPD_photol
MQCYRFLIQPVLRSISQCVPPIRQRQRPNPVHFHVRLRLQWCASMQEGGSKVTAKNGGEGKAASKRKGEPVEA
GGNKQPKVVPPKEGKKKREQGWLESEVAEARRGAQGVKFNERRLRHLSEAQKVKQGTQGVLYWMSRDQRVQDN
WALIYAQQVAVTEELPLHVCFCLQPQSPDATLREYGFILKGLEEIAKECNVLGIEFHLLLGTAGEVLPGFVRD
WSLGAVVTDFSPLRIPLKWVEDVKKGLPSDVPFIQVDAHNIVPCWVASDKQEYSARTIRGKITKLLPEFLTQF
PLVDKHPHCASKPAKPVDWAHTLSTVEVDRSVGAVEWARPGTAAGMAMLESFVDARLRDYASERNNPNSDALS
QLSPWIHFGHISAQRAVLQVQRDGPNRAQSVPAFVEELVVRRELADNFCFYNKNYDKVEGASDWAQKTLKDHA
KDPRQYVYSREQLEKAKTHDKLWNAAQYQMVTEGKMHGFLRMYWAKKILEWTSSPEEALAIAIYLNDRYELDG
RDPNGFVGCMWSICGIHDQGWQERPVFGKIRYMNYAGCTRKFNVQQFEKQYCPK
>Melanotaenia_boes__XP_041824669.1
MSASRTVICLLRNDLRVHDNELLHWAQKNAEHIVPLYCFDPRHYVGTYNFNLPKTGPFRLRFLLESVRDLRNT
LLSKGSNLVVRRGKPEEVVADLIKQLGSVSSVAFHEEVASEELNVEKKVKEVCAQMKVKVHTCWGSTLYHRDD
LPFHHISRLPDVYTQFRKAVESQSRVRPVFPTPERLNPLPPGLEEGAIPSAEDLQQTEPVADPRSAFPCSGGE
SHALARLKHYFWDTDAVATYKETRNGLIGVDYSTKLSAWLALGCISPRYIYHQNKQYERERTANQSTYWVIFE
LLWRDYFKFVGVKYGNRMFQIKGLQDKSMPWKTDMKLFDAWKEGRTGVPFVDANMRELAMTGFMSNRGRQNVA
SFLTKDLGLDWRMGAEWFEYLLIDHDVCSNYGNWLYSAGVGNDPRENRKFNMIKQGLDYDSNGDYVRWWIPEL
KDIRGADVHTPWNLSLASLSHACVSLGDTYPTPIIIAPEWSRHFNKKPSGSGPTAKGKKGPSHTPKQHRDRGI
DFYFSRSKNL
>Melanotaenia_boes__XP_041847736.1
MTQISIHWFRKGLRLHDNPALMAALRDCKELYPVFILDPYLHNNTHVGINRWRFLLGALKDLDCSLRKLNSRL
FVVRGKPEDVFPKLFTRWSVTKLTYEYDTEPYSLSRDKKVTTLAKEHGIDVVYKISHTLYDIDRIIEENNGKP
PLTYNRLQAIVRMLGPPKRPIPAPTMEDMKDIKTPCSEKHEKKYGIPTLEELGMDMASFRDELFPGGEQESLQ
RLDEHMKRTGWVCSFEKPQTYPNSLSPSTTVLSPYITFGCLSARTFWWRLAEVYQGKKHSDPPVSLHGQLLWR
EFFYTASVGIPNFNKMEGNDVCTQVDWDKNPEYLAAWREARTGFPFIDAIMTQLRQEGWIHHLARHAVACFLT

RGDLWISWEEGQKVFEELLLDGDWALNAGNWQWLSASTFFHQFFRVYSPIAFGKKTDKNGDYIKKYLPLLKKF
PAQYIYEPWKAPRSVQQAAGCIVGKDYPRPILEHEVNSKKNIQRMKQAYAKRSSDGTESPTKKQGVKRKAPTV
IDMLLKKARKA
>Melanotaenia_boes__XP_041848176.1
MKISFIHVGFLCSSFLSASSRLLVLWPGQTLILSTTAFIGEPIMSGKKRKATSSAGSQEPDAKQPKVASEKEE
KPKKAAGWLQSLVKQQRADKKEMKFNKKRQRYITDTEKIKQGCKGVLYWMLRDQRVQDNWALIRAQQLAAKEN
LPLHVCFCLVVPKSELSTLRHYGFMLKGLEEIAKECKHLNIQFHLLHGAAGDVLPGFVSDRGFGAVVTDFSPL
REPLQWLKDIEKSLPKDVPLIQVDAHNIVPCWVASPKQEYSARTIRGKITNALSEFLTDFPPVEKHPHTAASS
AKPVDWDKTLASLQVDKAVGEPQWAKPGAEAGMNMLESFIDVRLKLFSTQRNNPNVAALSQLSPWLRFGHVSA
QRVALQVQRCGKSASQSIPPFIEELVVRRELTDNFCFYNKNYDRVEGAYEWAQKTLKDHAKDKREHLYTREQL
EKAKTHDKLWNAAQYQMVSEGKMHGFLRMYWAKKILEWTTSPEEALSIALYLNDRYELDGQDPNGFVGCMWSI
CGIHDQGWAERPVFGKIRYMNYKGCLRKFNVAEFERKYCPKKA
>Melanotaenia_boes__XP_041852008.1
MVVNSVHWFRKGLRLHDNPALQEALNGADTVRCVYILDPWFAGAANVGINRWRFLLEALEDLDSSLKRLNSRL
FVVRGQPTDVFPRLFKEWNVTRLTFEYDPEPYGKERDGAIIKVAQEFGVETVVRNSHTLYNLDRIIEMNNNNP
PLTFKRFQTIVSRLELPRRPLPPITKQQMDRCCTKIADNHDQLYSIPSLEELGFRTEDLPPAVWRGGESEALD
RLNKHLDKKVWVANVEHTRVNTCSLYASPTGLSPYLRFGCLSCRVLYYNLREFYMKLRKRCSPPLSLFGQLLW
REFFYTAATNNPNFDRMEGNPICVQIPWDQNPEALAKWAEGRTGFPWIDAIMTQLRQEGWIHHLARHAVACFL
TRGDLWISWESGMKVFEELLLDADWSVNAGSWMWLSCSAFFQQFFHCYCPVGFGRRTDPSGDYIRRYIPILKD
YPNRYIYEPWNAPESLQKAANCVVGVDYPKPMINHAESSRLNIERMKQVYQQLSHYRGLSLLASVPTIQEEAE
PLMTDESQTSSGPDSPPRDAADYEAAGCSTAPDSSTSVPHPDEEDAADEPSHPSSPACSPSSRSKPSSPSSSC
STSSMCPSPSAALTQTSSVGSRRKSVARKVRRSQRQRGRQCCLATPREEERGEAEEGERMEESVVVEEMTG
>Melanotaenia_boes__XP_041852192.1
MPPSAGSDQTVVRQMLREVLVGREDPEGFFAICVSVLGYGETRSQFPSLIKPLSTANISLHSVLTSIYQEYFS
KTEDDELELALALSLLEMKNPSPSTSIQEPQPLQPGVRINHSRSVQSNYVSKSQERNFTQLEDVIRRENTSSP
QFGFWNRENTHLDDNQTPGTAQTLCFPGSSASFSKQNTENQGAQTMEEGDLNQSEKPKRSKNRRQRRKRGGQQ
LVGLSRSPSAPPPVLLWFRRDLRLCDNPALVGSLELDAPVIPVFIWSPEEEEGPGVTVAVGGACKYWLHQALL
CFCGTLEYIGSHLIFLKASAEGNKVGSSLYTLKQLVKETGARKVLANALYEPWLKERDDAVVSALQKEGVECR
LFHSYCLRDPYSVSTEGVGLRGIGSVSHFMSCCRQNPGAAVGTPLDPPVSLPSPTHWPQGVPLDTLGLAWMPR
RKDGTIINWAANIQKSWDFSEDGAHARLDAFLHDGVYRYEKESGRADAPNTSCLSPYLHFGQLSPRWLLWDAK
GAHCRPQKFQRKLAWRDLAYWQLTLFPDLPWESLRPPYKALRWNTDRSHLKAWQRGKTGYPLVDAAMRQLWLT
GWMNNYMRHVVASFLIAYLHLPWQEGYRWFQDTLVDADVAIDAMMWQNGGMCGLDHWNFVMHPINAAMTCDPC
GSYVRKWCPELADLPDELIHKPWKCPASMLRRAGVVFGQTYPQRIITDLEERRSQSLQDVALVRKEFGQYVDK
RSGCDLVPLPPRLVSEALGLSHRDGIVVSEGKHFLLPVITRMEFKYQQEDPDADAASNPYNAVLKGYVSRKRD
ETIAFLNEKDFTASVMYEGAQRQERLEKDYRRMEGLPPSPSTRGRARRTPTAKDRFSIVPGGAVTSLK
>Melanotaenia_boes__XP_041852785.1
MVVNTIHWFRKGLRLHDNPSLRDSIRGADTLRCIYILDPWFAGSSNVGINRWRFLLHCLEDLDASLRKLNSRL
FVIRGQPTDVFPRLFKEWKIGRLSYEYDSEPFGKERDAAIQKLASEAGVEVIVRISHTLYDLDKIIELNGSQP
PLTYKRFQALINRMDAVDLPAETITSEVIKNCVTPIRDDHDDKFGVPSLEELGFETEGLTTAVWPGGETEALM
RLERHLERKAWVANFERPRMNANSLLASPTGLSPYLRFGCLSCRLFYFKLTDLYRKVKKNSTPPLSLYGQLLW
REFFYTTATNNPCFDKMEGNPVCVQIPWDRNPEALAKWAEGRTGFPWIDAIMTQLRQEGWIHHLARHAVACFV
TRGDLWISWEEGMKVFEELLLDADWSVNAGSWMWLSCSSFFQQFFHCYCPVGFGRRTDPNGDYIRRYLPILRG
FPAKYIYDPWNAPEEVQKAAKCMIGVHYPKPMVNHAEASRINIERMKQIYQQLSCYRGLGLLASVPVNPNHGA
NGNTGTSQGPGGTSEDPSAPEGMSRAERGQSTHKRRHEDVPSESNSKSWKQSK
>Melibe_leo_AWY10933.1_non-photor
MKEDINDNKNQSKKQVLFWFRKGLRLHDNPALYSAIDGAVTYRCVYILDPWFAGASQVGINKWRFLLESLEDL
DNSLRKLNSRLFVARGQPADVLPRLFQEWRITTLAFEEDSEPYGKERDSAISAMAREFNIQVISKSSHTLYNP
KTVIAANGNSPPLTYKRFQSILSTLDPPEQPCDTVGANVVKKSVTPVTDDHDDRFSVPTLDELGFDTDSLGPS
KFQGGESEALARLHRHLERKAWVASFERPKMSPQSLYPSGTGLSPYLRFGCLSPKTFYWKLTELYKKVKKGID
PPLALHGQLLWREFFYTVSTNNPKFDRMVGNSICVQIPWEHNPEALAKWAEGMTGYPWIDAIMIQLRKEGWIH
HLARHAVACFLTRGDLWISWEEGMKVFDEMLLDADWSVNAGTWMWLSCSAFFQQFFHCYCPVGFGKRADPSGD
FVRQYLPVLKNMPTRYIYEPWTAPESVQKAAKCIVGKDYPLPMVAHAEQSRINLERMRQIYKRLVLKSTASKR
IFLDLPKIPKVGMFLSKAKMFLSSSSDEEASTKPRRRKQVDSSAA
>Melibe_leo_AWY11207.1_photorecep
METMISESSNNVTIHWFRHGLRLHDNPSLLEGLENCSEFYPVFIFDGSVAGTKTAGYSRMQFLLETLRDLDEN
LKKKGSRLYVFMGEPVKIFRQLFEDWNATRLSFEQDPEPIWQDRDNNVKDLCDACNVEWIERVSHMLWDPHVI
LRANGGSPPLTYAMFCQVTEIVGLPPRPVEDPDFSKVTLPVSDHHDRLYGVPSLEKLGIVAECEAQLSPYCRY
LGGETKAAALLVSRLAKESKGFSLGQVQANQMYPDLTGMPMSLSPHLRFGSLSIRKLYWALRDTYSELYPGSS
VPMSVTSQLIWREYFYCMSVNNPNYNRMFENPICLNIDWYSDDALLRKWKEGKTGYPWIDACMRQLVQEGWIH
QVCRHAVACFLTRGYLWIDWQKGLETFDHYLLDADWSVCAGNWMWVSSSAFEKVLQCPRCICPVRYGKRMDPT

GTYVRRYVPELKNMPMNYLFEPWKAPESVQLEADCIIGTDYPSPMVDHRTASKECKEKMEKVKGKYRDDTPHI
APTNEDEVISLIWMRKQHGETQMCDHSDEKLT
>Melibe_leo_AWY11210.1_6-4_photol
MAHRSIHWFRKGLRLHDNPALLKAFEHASEVYPIFVLDPWFVSSPSCKVGINRWRFLLEALKDLDDKLKALNL
RLFVVRGSPDKELEKLCKKWNISRLTFEVDTEPYAVKRDEQVEAQMKNLGVEVIKCTSHTLFDTAKVVKTNKG
EAPLSYRGFQTILSKLGAPPKPLDPPTKEDVKDCQTRISDTHDEDYGVPTLNELGKDAESCGPLLYPGGETEA
LERMERHLKKTVQIYFS
>Melibe_leo_AWY11213.1_cry_dash
MFRSSVILQVFLKPLKVTGRLKMTARPSNVGIVLFRNDLRIHDNEAIFLANKNCSQVLPLYCFDPRHFQGTYH
YNFPKTGSHRAKFLLECIADLKANLQRKGSDLVVRLGKPENVIPELMTNLNLGTDTMVVMQKEVTDEELKVEK
ALKEKSKSKFETVWGHTLHHKEDLPFQIKHLPDVYTQFRKKVEGNTPIRKCFQLPDSLKPLPSNVEVGTIPTF
QDLHLSEPGRNPNSAFPFSGGESAALERLENYLWASDNVSTYKETRNGMIGAEYSTKFSSWLAHGCISPRKIY
WEIQKYEQERTANQSTYWVIFELLWRDYFRLVAMKFGNQLFFSSGIKGGHVQWKKNMLHFKAWQEGKTGVPYV
DANMRELAASGFMSNRGRQNVASFLTKDLELDWRLGAEWFESMLIDHDVCSNYGNWLYSAGIGNDPRENRKFN
VVKQGLDYDGQGEYIRLWVPELKEVKDGRVHCIWTASKTILDSSGVSLGETYPYPIVMAPEWSRHVNRPGSSH
SASGGGFSSGKGPRGGSARGRGGGRGRGHSDQSVGQKRGIDFYFKNSTPR
>Melopsittacus_undu__XP_030898891.1
MLRRQRKRKASAPEEIPRVSRKRTEQEEELHEARRRAAPSVREFKFNKKRVRLISQASDLKDGGRCILYWMSR
DQRLQDNWAFLYAQRLALKQELPLCVCFCLVPKFLEATIRHYGFMLRGLQEVAEECAELNIAFHLLRGYAKDV
LPAFVEEHGVGGLVTDFSPLRLPWQWVQDVREWLPEDVPFAQVDAHNIVPCWVASPKQEYSAYTIRRKIQAQL
PEFLTEFPPVVRHPYSPSCPAEPIAWEACYSSLEVDRTVKEVEWATPGTAAGLAVLQSFIRERLKYFGSHRND
PNKAALSNLSPWFHFGQVSTQRAILEVQKHQHKYKESVDMFVEEAIVRRELAENFCYYNENYDSVQGAYPWAQ
TTLKLHAKDERPFLYKLQELEQGTTHDPLWNAAQLQMVREGKMHGFLRMYWAKKILEWTHSPEEALQFAIYLN
DRYELDGRDPNGYVGCLWSICGIHDHGWTERAVFGKIRYMNYAGCKRKFDVRQFERRYAPCTLSQ
>Melopsittacus_undu__XP_030905675.2
MSGSARTAICLLRCDLRAHDNQVLHWAQSNAEFVVPLYCFDPRHYLRTYWYGFPKTGPYRLRFLLESVKDLRE
TLKKKGSTLVVRKGKPEDVVHDLITQLGSVSAVVFHEEATQEELDVENELCQVCSQHGVKVHTFWASTLYHRD
DLPFRPIARLPDVYTHFRKAVESEAKVRPTLRMADQLQPLAPGVEEGCIPTMEDFGQKDPDTDPRTAFPGSGG
ETQALMRLQYYFWDTNLVASYKEVRNGLVGMDYSTKFAPWLALGCISPRYIYEQIKKYERERTANQSTYWVLF
ELLWRDYFRFVALKYGRRIFSLRGLQSKEVPWKKDLQLFNCWKEGKTGVPFVDANMRELAATGFMSNRGRQNV
ASFLTKDLGLDWRMGAEWFEYLLVDYDVCSNYGNWLYSAGVGNDPRDNRKFNMVKQGLDYDGNGDYVRLWVPE
LQGIKGADIHTPWALSSAALSQAGVTLGETYPQPIVTAVEWSRHISQRPGRSLHPRGSRRPAHSPVQHKDRGI
DFYFSRKKDAR
>Melopsittacus_undu__XP_033918640.1
MCNPRAHHGSAQGAIHNRFLLQCLEDLDANLRKLNSRLFVIRGQPADVFPRLFKEWNIAKLSIEYDSEPFGKE
RDAAIKKLASEAGVEVIVRISHTLYDLDKIIELNGGQPPLTYKRFQTLISRMEPLEMPVETITPEVMEKCTTP
VSDDHDEKYGVPSLEELGFDTDGLPSAVWPGGETEALTRLERHLERKAWVANFERPRMNANSLLASPTGLSPY
LRFGCLSCRLFYFKLTDLYKKVKKNSSPPLSLYGQLLWREFFYTAATNNPRFDKMEGNPICVQIPWDKNPEAL
AKWAEGRTGFPWIDAIMTQLRQEGWIHHLARHAVACFLTRGDLWISWEEGMKVFEELLLDADWSVNAGSWMWL
SCSSFFQQFFHCYCPVGFGRRTDPNGDYIRRYLPVLRGFPAKYIYDPWNAPESIQKAAKCIIGVNYPKPMVNH
AEASRLNIERMKQIYQQLSRYRGLGLLATVPSNPNGNGNGGLMGYSPGESISGCSSAGGAQLGTGDGHTVVQP
CALGDSHTAGASGIQQQGYCQASSILHYAHGDNQQSHLLQAGRTAVGTGISAGKRPNPEEETQSVGPKVQRQS
TN
>Menidia_meni__CAG5853193.1
MAQNCIHWFRKGLRLHDNPALVAALRDCKELYPVFILDPYHHDTHVGINRWRFLIGALTDLDHSLRKLNSRLF
VVRGKPEDVFPKLFNKWNITKLTYEYDTEPSSLTRDKKVTSLAKEHGVDVIYKISHTLYDIDRVIEENNGKPP
LTYVRFQAVVKMLGAPKKPIPAPTMEDMKDVRTPCSEKHEKKYGIPTLEELGCVTSSLGEELFPGGECEALRR
LDEHMKRKAWVCSFEKPQTSPNSLSPSTTVLSPYITFGCLSVRTFWWRLTEVYKGSKHSDPPVSLHGQLLWRE
FFYTASVGIPNFNKMEGNPVCTQIDWDKNSEYLTAWKEARTGFPFIDAIMTQLRQDGWIHHLARHAVACFLTR
GDLWISWEEGQRVFEELLLDGDWALNAGNWQWLSASTFFHQYFRVYSPIAFGKKTDKNGDYIKKYLPILKKFP
AQYIYEPWKAPRSVQQAAGCIVGKDYPCPIVEHEVISKKNVQRMKESYAKRSSDGTESPKKKQGAKRQAPTVI
EMLQKKAKRTKLVRNSSV
>Menidia_meni__CAG5896000.1
MSATRTIICLLRNDLRVHDSELFHWAQRNAEHIVPLYCFDPRHYVGTYNYNVPKTGPFRLRFLLESVRDLRNT
LLNKGSNLVVRRGKPEEVVADLIKQLGSVSSVAFHEEVTSEELNVERKVKEVCAQLKVKVHTCWGSTLYHRDD
LPFHHMSRLPDVYTQFRKAVESQSRVRPVFPAPEKLNPLPPGLEEGAIPTAEDLQQPEAAADPRSAFPCSGGE
SQALARLKHYFWDTDAVASYKETRNGLIGVDYSTKFSSWLAMGCISPRYIYHQIKQYEMERTANQSTYWVIFE
LLWRDYFKFVGAKYGDRMFQIKGLQDKSVPWKMDKKLFDAWKEGRTGVPFVDANMRELAMTGFMSNRGRQNVA
SFLTKDLGLDWRMGAEWFEYLLVDHDVCSNYGNWLYSAGIGNDPRENRKFNMIKQGLDYDSNGDYVRQWVPEL

KSIRGADVHTPWNLSSATLMHAHVSLGETYPTPIVIAPEWSRHFNKKPSGTGPSPRGKKGPSHTPKQHRDRGI
DFYFSKSKNL
>Menidia_meni__CAG5896989.1
MTCAWTSSFIFCAPKRLWKLAIRPKRPSSSLSLLFDSLVGEAAMSGKKRKASTPAGGKDPGAKQPKLSSSSSS
SEEEKPKRAAGWLQSLVEQQRAEKKEMKFNKKRQRILTDAEKIKQGSEGVLYWMLRDQRVQDNWALIRAQQLA
AKKELPLHVCFCLDVPESELSTLRHYGFLLKGLEEVAKECKHLNMQFHLLRGAAGEALPGFVSDRSFGAVVTD
FSPLREPLGWLKSVKKSLPKDVPLIQVDAHNIVPCWVASPKLEYSARTIRGKITKLLPEFLTDLPLVERHPHT
ATKGAKPVDWEETLESLQVDRTVAEPAWAEPGAKAGMAMLESFIDVRLKLFGTQRNDPNVAALSQLSPWLRFG
QISAQRVALQVQRCGKSASQSIPPFIEELVVRRELTDNFCFYNKKYDSVEGAYEWAQKTLKDHAKDKREYIYT
REQLEKAKTHDKLWNAAQYQMVTEGKMHGFLRMYWAKKILEWTPSPEEALSIALYLNDRYELDGQDPNGFVGC
MWSICGIHDQGWAERPIFGKIRFMNYKGCLRKFNVAQFEKKYYPKKA
>Menidia_meni__CAG5958100.1
MPPSTSGDQALVRGMLKEVLVGREDPEGFFAICVSVLGHQETRSQFLSLIKPLSTANIRLHSALTSIYQGYFS
EIEDDELEHALALSLLEAKNQQLSTTSRESQPLHPGLNRSIQHNSPLKSQEKNSNRLDDTGGEKSPSFAQTAT
RHKVGSPQTTQKTELQKTELQKTSHAGNYDDRSLGTGRTLCAPGSLSGGGQMTEEEDLNQSEKPKRSKNRRQR
RKRAGQQVIGLPCSPSAHAPVLLWFRRDLRLCDNPALIGSLELGAPVIPVFIWSPDEEEGQGVTIAMGGACKY
WLHQALSCFCTALEGIGSHLTFLAANTDGDRVGSSLLSLKQLVKETGARTVLANALYEPWLKERDDEVVSSLQ
KEGVECRMFHSYCLRDPYSVSTEGVGLRGIGSVSHFMSCCRQNPGSTVGTPLEPPGSLPTPTLWPQGASLESL
GLARMPRRKDGTIVDWAANIRKSWDFSEDGAHARLDAFLHDGVYRYEKESGRADAPNTSCLSPYLHFGQLSPR
WLLWDAKGARCRPPKFQRKLAWRDLAYWQLTLFPDLPWESLRPPYKALRWSTDRSHLKAWQRGKTGYPLVDAA
MRQLWLTGWMNNYMRHVVASFLIAYLHLPWQEGYRWFQDTLVDADVAIDAMMWQNGGMCGLDHWNFVMHPVDA
AMTCDPCGTYVRKWCPELADLPDELIHKPWKCPASMLRRSGVAFGQTYPERIITDLEERRSRSLQDVAVVRKE
FGQYVDKRSGCDLVPLPPRLVSQALGLSHRDEAVTSNRKEFLLPVITRMEFKHQQEDPDADASSNPYNAVLKG
YVSRKRDETIAFLNERDFTASVMYEGTQRQERLERDHRRIEGLPPSPSPRGRARRTPTAKDRFSIVPGGAVTA
LK
>Menidia_meni__CAG5958302.1
MVVNTIHWFRKGLRLHDNPSLRDSIRGADSLRCIYILDPWFAGSSNVGINRWRFLLQCLEDLDASLRKLNSRL
FVIRGQPTDVFPRLFKEWKITRLSYEQDSEPFGKERDAAIQKLASEAGVEVIVRISHTLYDLDRIIELNAGQP
PLTYKRFLTLITRMDAVELPTETITSEIIQNCVTPTRDDHDDKFGVPSLEELGFETEGLSTAVWPGGETEALM
RLERHLERKAWVANFERPRMNANSLLASPTGLSPYLRFGCLSCRLFYFKLTDLYRKVKKNSSPSLSLYGQLLW
REFFYTTATNNPCFDKMEGNPVCVQIPWDWNPEALAKWAEGRTGFPWIDAIMTQLRQEGWIHHLARHAVACFV
TRGDLWISWEEGMKVFEELLLDADWSVNAGSWMWLSCSSFFQQFFHCYCPVGFGRRTDPNGDYIRRYLPVLRG
FPAKYIYDPWNAPEEVQKAAKCMIGVHYPKPMVNHAETSRINIERMKQIYQQLSCYRGLGLLASVPSNPSSGA
NGPRSGTARDPEGSSEDPSLQEGTSPTERGQFTQKRRHEDAPSASSSKSLKQSK
>Mercenaria_merc__XP_045159044.1
MDNTVDVDNILTDFKSGNLSADECFCMILSLIGYDAAKTHFLAHVETLRKEQPKLHNQLFNVYRGYFECLDEC
FNLPDKLDCSVENGFYATDSSENQDIGFDLDLEMALKLSLESAVDTPSYASKLMSSHTVNGVSDKCHEPERVR
KMTFSFTDNMSCEKKAIATQSLDTDSSHTFESSQKSKKKKNRKKSRVDRPLSNAPLIYWFRRDLRLYDNPALE
FAALSGAPVLPIFLWSDAEEGPTKAFANGGATKYWLHMALPTLNRDMIERYNNPIIFRKSTDYLTDIVDIIKQ
SGAKTLVMNDVYEPYLKERDDKICKVLRNKGVKCERFHSYLLHQPGAVKTESLCMRGMGSVTHFMECCRQSDP
EPLGVPVDPPNILPVGTDRPESVPVSELGLGRLPKRKDGTEIDWARVIRESWDFSENGAWEALQLFLNEGIRN
YEKESSRGDKLNTCRISPYLHFGQISPRTVLNEGRYLKSPKFLRKLAWRDLSYWLLSVFPDLPYAPTRPQYQH
QRWSDNKIHLKAWQKGNTGYPLVDASMRQLWLTGWMNNYMRHVVASFLISYLHITWVEGYLWFQDTLLDADVA
INAMMWQNGGMSGLDQWNFVMHPVDAAMTCDPKGDYVRKWIPELARMPEEFIHKPWKCPPSILRRAGVELGVT
YPHRIITNLEEAREQSLRDVVDVRQKFEGLIDPRTGNDLVQLPNGVLIPVITRKEFKYKTRNPESRDNPHSAV
LRGYRSRKRDELVEYLNQRDFMASTMKECSMRHDRGLKASQSGSFY
>Mercenaria_merc__XP_045167514.1
MSSKGRIVICMLRNDLRFHDNEVLAWANKNGNYLLPVYCFDPRHYGGTYKFGFPKTGPHRLKFLLESVKDLRN
TLKSHGSGLVVRNGKPEVVIPELIQKLGNDAVHAVAFHEEVTQEELDVERALKLQCENKVIQKIWGHTLFHRD
DLPFSVKQLPDVYTQFRKRVEGSCTVRQCIEMSEQLNPLPEGIEEGEIPSMQQLGIQGTSXLLLXAPDSRTSF
PFSGGETSGLERVRHFLWETDAVATYKETRNGMIGAEYSTKFSAWLAHGCISPRYIYYEIKRYEQERVANNST
YWVIFELLWRDYFRYVALKYGNKLFYLSGIQGKHLPWKKDKELFRAWKEGRTGVPYVDANMREVAATGFMSNR
GRQNVASFLTKDLKLDWRLGAEWFESMLIDHDVCSNYGNWLYSAGLGNDPREDRKFNMVKQGLDYDPEGDYVR
LWVPELKNVSGGNVHTVWTMSNNALSRADVSLGETYPNPVTIAPEWSRHYGKSGGASARGGARGGGRGGGHGQ
GGRHGSAGQKRGIDFYFKGS
>Mercenaria_merc__XP_045176200.1
MQVTSLLNGLAICSLLRNRKIWTGFQEQISSLIEMTHRSIHWFRKGLRLHDNPALLEACKNASHVWPVFVMDP
WFAETARVGVNRWRFLLQTLQNLDDNLKKINSRLFVVRGNPSDIFPKLFKEWGITKITYEVDTEPYAIERDSV
IDGLAKDSGIEVMRCVSHTLYDTERTISKNGGSPPLTYQRFQTLMSQMGDPVKPVTVPDDLIKGCKTPVDKDY
EKKYGVPTLEELGKSAKDCGPCLFPGGETEALERLDSLLKKTNWVCNFEKPKTEPNTLEPSTTVLSPYLKFGC

LSARMFYHRVNEVYKGKKHSQPPTSLLGQVMWREFYYTVAVGTPNFDKMVGNSVCTQVDWDTNPEFLEAWKMG
RTGYPFIDAVMTQLREEGWIHHLGRHAVACFLTRGDLWISWEEGMNVFEEYLLDADWSLNAGNWMWLSASAFF
HQYFRVYSPVAFGKKTDKNGDYIRKYIPKLAKFPEKYIYEPWNAPKAVQEKCGCVIGKDYPKPIVDHDVARKT
NISRMAAAYARRKAGKESNGAASTPATKKRKAESDASGKAKKKSKNKEITDFLKK
>Mercenaria_merc__XP_045196489.1
MEESAISMPPASTAVENSGHPASIHWFRHGLRLHDNPALLEALELGGDFYPIFIFDGEVAGTKTAAYNRMRFL
LESLEDLDESLKKYGGRLFVFHGDPVEILSGLFNEWSVEKLTFEQDPEPVWTARDNAVKKLCQDTGVEFVEKI
SHTLWDPKVIIENNGGCPPLTYMLFNQVATMVGDPPRPSVEPDFTKVSMPVSGDHDKKFGVPSMDKFGISPEC
EEQNDRINSWKGGETQALRLFAHRLQVEEIAFSCGNVMPNQSFPDLVGPPLSMSAHLRFGCLSIRKLYWALRD
AYTRVNLDKPFTISVVGQLMWREYFYTMSVNNINYNKMEGNPICLNVPWYKDDEKLKKWENGETGYPWIDAIM
KQLKHEGWIHHVGRHAVSTFLTRGDLWINWEEGLKVFDKYLLDADWSVCAGNWMWMSSSAFEKVLSCPKCFCP
VRYGRKMDPNGEYIKRYLPVLKDMPTRYLYEPWKAPKAVQKKANCIIGVDYPLPIVEHNKASKECYNMMMEVK
NKLVQQGKGMEHCRPTNMEEVRMFVWMPGHHKGVCDREVPLDEKDLCDGLTGM
>Mercenaria_merc__XP_045198496.1
MSSNQEGFIADKVKKAEKRVEVSPHDTEAWSVLIRDAQIKLIEQARPVYERLVSQFSNAGRYWKIYIEHELKS
KNYERVEKLFQRCLIKILNIDLWKLYISYIKDTKGKLPSYREKMAQAYDFALDKMGMDIMSHSIWHDYITFLK
SVDAVGSYAENQRITAVRKVFQRGVVNPMINIEALWKDYGTYENSINPLIAKKMIEDRGRDYMNARRVSKEYE
VVTRGLNRNLPSVHPTNSPEEATQVELWKKYIAWEKSNPLRTEDQALITKRVMFAYEQSLLCLAYHPDVWIEA
ANYLEQSSKILTEKGDQNAGKMFADEAAALFERAITTTMKANMLIYFTYADFEESRMKYEKVHSIYKRCVAVE
SIDPSLVYIQYMKFARRAEGIKSARQVFKLAREDKRSTYHVFVAAALMEYYCSKEKTVALKIFELGLKKFGDV
PDYIRAYVDFMSHLNEDNNTRVLFERVLSSGQIPPEKSISFTLDIKGEIPVLENGLDTPVKMDAGTLLSLEYA
SGRKRRITENEGSDDEDINTGPAPPSRDFASNFIIIDNVFSRNDGGLIKLIKHIRMDVTTKQPKRRVLHWFRK
GLRLHDNPALRAALEGAQTYRCVYILDPWFAGVSQVGVNRWRFLLQCLEDLDSSLRKLNSRLYVVRGQPAEVF
PRLFKEWNITVLSFEADPEPFGKNRDAAITAMAKEVGVEVVKRSSHTLYDLQRIIAENNGIPPLTFKRFQVVL
SRMSLPDKPEERITFETIGEAKTPITLDHDEQYGVPSLEELGFDTAELNSKVFRGGETEALQRLHRHLERKAW
VASFEKPKMTSQSLFPSQTTLSPYLRFGCISARLFYWKLQELYRKIKKDCDPPLSLHGQLLWREFFYTAATDN
PNFDRMSGNSICVQVPWDKNPEALAKWAEPGCLKMSI
>Mercenaria_merc__XP_045202658.1
MKFQGLRSIRLVINCRHIIESVIHPCANFSNTAYRLRYSPVLLPRYLSTMSGSESGKKRKLEADDKNSCKENG
EPDVKKPSVEDFQLKIIESRKSVCKSVAEFKFNKKRVRVLSKAQDFPEDSNGVLYWMSRDQRVQDNWAFLYAQ
KLALKLEVPLHVCFCLVPKFLEATIRHFTFMLEGLKEVEKECKELGISFHLLIGHAKDVLPSFLTEHSIGGLV
TDFSPLRVPRGWVEEVVKVLPKDIAFCQVDAHNLVPCWEASPKLEYGARTIRNKIHNQLSGFLTEYPPVCKHK
YRPKLESKPIDWDSAYASLEVNQIVGPVDWAKPGTSAGLQMLESFCKERLRYFGSERNNPNKNALSNLSPWIH
FGKISDSADTKIFSGAYDWAKQTLKAHEKDKREYVYSREKLEQGKTHDPLWNAAQNEMVNEGKMHGFMRMYWA
KKILEWTSSPSEALEIAIYLNDKYSLDGRDPNGYVGCMWSICGIHDQGWKEREVFGKIRYMNYNGCKRKFDVG
KYEAKHRRQTIAK
>Merops_nubi__KFQ18699.1
RFLLQCLEDLDANLRKLNSRLFVIRGQPADVFPRLFKEWNIAKLSIEYDSEPFGKERDAAIKKLASEAGVEVI
VRISHTLYDLDKIIELNGGQPPLTYKRFQTLISRMEPLEMPVETITPEVMEKCVTPVSEDHDEKYGVPSLEEL
GFDTDGLPSAVWPGGETEALTRLERHLERKAWVANFERPRMNANSLLASPTGLSPYLRFGCLSCRLFYFKLTD
LYKKVKKNSSPPLSLYGQLLWREFFYTAATNNPRFDKMEGNPICVQIPWDKNPEALAKWAEGRTGFPWIDAIM
TQLRQEGWIHHLARHAVACFLTRGDLWISWEEGMKVFEELLLDADWSVNAGSWMWLSCSSFFQQFFHCYCPVG
FGRRTDPNGDYIRRYLPVLRGFPAKYIYDPWNAPESVQKAAKCIIGVNYPKPMVNHAEASRLNIERMKQIYQQ
LSRYRGLGLLATVPSNPNGNGNGGLMGYSPGESISGCGSTGGAQLGTGDGHTVVQPCALGESQTGASGIQQQG
YCQASSILHYAHGDNQQSHLLQAGRTALGTGISAGKRPNPEEETQSVGPKVQRQSTN
>Merops_nubi__XP_008939625.1
NLVASYKETRNGLVGVDYSTKFAPWLALGCISPRYIYQQIQKYEKERTANQSTYWVLFELLWRDYFRFVALKY
GRRIFSSGGLQSKEVSWKKDLQLFDCWKEGRTGVPFVDASLRELAATGFLSNRGRQNVASFLTKDLGLDWRMG
AEWFQYLLVDYDVCSNYGNWLYSAGVGNDPREDRKFNVIKQGLDYDSNGDYVRLWVPELGGIQGAEVHAPWAL
DSAALSQAGVALGETYPRPVVTAPEWSRHISQRPQGRSPRGRGRRGPAHAPVRHRDRGMEFYFSGKKAA
>Merops_nubi__XP_008939780.1
VFDELLLDADFSVNAGSWMWLSCSAFFQQFFHCYCPVGFGRRTDPSGDYVKRYLPKLKGFPSRYIYEPWNAPE
SVQKAAKCIIGVDYPKPMVNHAETSRLNIERMKQIYQQLSRYRGLCLLASVPSCVEDFSGPVTDSASGQGCST
STAVRLSQAEQASPKRKHDGAEELCSEDLYKRAKVTGLPAAEVPGKSL
>Merops_nubi__XP_008942592.1
RKEEEEEVQEARRRAAPSVQEFKYNKKRVRLVSQGSDLKDDARCILYWMSRDQRVQDNWAFLYAQRLALKQEL
PLHVCFCLVPKFLDATIRHYSFMLRGLQEVAEECAELNIPFHLLLGYAKDVLPAFVVEHGVGGLVTDFCPLRL
PRQWVEDVKEQLPEDVPFAQVDAHNIVPCWVTSPKQEYSARTIRAKIHAQLPEFLTEFPPVIRHPYSPSSPAE
PIAWEACYSSLQVDRSVKEVEWATPGTAAGLAVLRSFLTERLKSFSSHRNDPNKAALSNLSPWFHFGQVSTQR
AILEVQKHRGKYKESVDAFVEEAVVRRELADNFCYYNENYDSVQGAYDWAQTTLKLHAKDKRPFLYELQELEQ

GTTHDPLWNAAQLQMVLEGKMHGFLRMYWAKKILEWTRSPEEALRFAIYLNDRYELDGRDPNGYVGCLWSICG
VHDQGWAERAVFGKIRYMNYAGCKRKFDVGQFERRYAPRPLSP
>Merops_nubi__XP_008947701.1
MSHHTIHLFRKGLRLHDNPTLLAALESSEVVYPVYILDRKFMTSVMHIGTLRWHFLLQSLEDLQKNLTQLGSC
LLVIQGEYESVLREHVQKWNITQVTLDAEVEPFYKEMEAKIQQLGEELGFEVLSLVSHSLYDTKRILELNGGT
PPLTYKRFLHILSLLGDPEVPVRNLTAEDFQRGGAPEPGLAEQYKVPLPVDLEIPLESLSPWRGGETEGLQRL
EQHLTDQGWVTSFTKPRTIPNSLLPSTTGLSPYFSMGCLSARTFFYRLSTIYAQAKHHSLPPVSLQGQLLWRE
FFYTVASATPNFTQMAGNPICLQIRWYEDAERLHKWKT
>Mesocentrotus_fra_ACO51512.1_photolyase
LALKQEVPLHVCFCLVPRFLEGTIRHFNFVLEGLKEVSQELHLLDIPFHLLIGYAKDVLPGFINDHGLGGVVT
DFSPLRTSRQWIKDVKEELPKNIPFCQVDAHNVVPCWEASNKLEYGARTIRPKITKQLTTYLTEFPPVICHPH
KAKAKAEPIDWEGAYASLEVDQTVKPVDWAQPGTSEGMKMLDSFVKERLRYFSSARNDPTKSVCSNLSPWIHF
GQLSSQRAALIVRLYRSRFSESVAGYIEESITRRELSDNFCFYNDNYDSIEGTNDWAKKTLKDHAKDKREYVY
SRETLERAKTHDQLWNSAQKQMVREGKMHGFLRMYWAKKILEWTTSPEEALEIAIYLNDRYSLDGRDPNGYVG
CK
>Mesocestoides_cor_VDD77140.1_unnamed_pr
MPSKLRPDGDSFIKWLKQINKSRFEHGDNWALLFAQRIALKFSVPLHVCFNVASSVALRTRRHLNFLLEGLAE
DCKDLAISFHLLLPLEAKSKTSLKRNADGSFACNETDHATLQPLVDFLKQRNACAVITDFSPLRDDRQAVEFV
SKRLPVDAHNVVPAWFASDKVEYAARTIRKKLHDKARHLLTEFPPVIRHPVTAKSVRERLLLQWAEPVDWVSV
KESLKELVDERVGEVNWAKGGSLAGFSQLYSFLHHRLHAYATGRNDPTKDALSNLSPWLHFGHISAQRCLWEA
KRLRTSHPQSVDAFIEEAFVRRELSDNFCLYNPHYDSIQGAWSWARETLKKHAGDARTPTYSSKAMEKAETGD
ELWNAAQRQLVQEGKLHGFLRMYWAKKILEWHDGGPEEALQLGFRLNDLYSLDGTDPNGYVGVMWSICGVHDQ
GWVERPIFGKIRYMNFAGCKRKFDVAAFIRRYPPKREPSSCK
>Mesocricetus_aura__XP_005064953.1
MAAAAVVAATVPALTMGADGASSVHWFRKGLRLHDNPALLAAVRGARCVRCVYILDPWFAASSSVGINRWRFL
LQSLEDLDTSLRKLNSRLFVVRGQPADVFPRLFKEWGVTRLTFEYDSEPFGKERDAAIMKMAKEAGVEVVTEN
SHTLYDLDRIIELNGQKPPLTYKRFQALISRMELPKKPVGAVTSQQMETCRAEIQENHDDTYGVPSLEELGFP
TEGLGPAVWQGGETEALARLDKHLERKAWVANYERPRMNANSLLASPTGLSPYLRFGCLSCRLFYYRLWDLYK
KVKRNSTPPLSLFGQLLWREFFYTAATNNPRFDRMEGNPICIQIPWDRNPEALAKWAEGKTGFPWIDAIMTQL
RQEGWIHHLARHAVACFLTRGDLWVSWESGVRVFDELLLDADFSVNAGSWMWLSCSAFFQQFFHCYCPVGFGR
RTDPSGDYIRRYLPKLKGFPSRYIYEPWNAPESVQKAAKCIIGVDYPRPIVNHAETSRLNIERMKQIYQQLSR
YRGLCLLASVPSCVEDLSHPVAEPSSSQAGSISNTGPRPPSSGPASPKRKLEAAEEPPGEELSKRARVTAMSA
QEPQTKDV
>Mesocricetus_aura__XP_040611116.1
MGVNAVHWFRKGLRLHDNPALKECIQGADTIRCVYILDPWFAGSSNVGINRWRFLLQCLEDLDSNLRKLNSRL
FVIRGQPADVFPRLFKEWNITKLSIEYDSEPFGKERDAAIRKLATEAGVEVIVRISHTLYDLDKIIELNGGQP
PLTYKRFQTLVSKMEPLEMPAETITSDVIGKCMTPLSDDHDEKYGVPSLEELGFDTDGLSSAVWPGGETEALT
RLERHLERKAWVANFERPRMNANSLLASPTGLSPYLRFGCLSCRLFYFKLTDLYKKVKKNSSPPLSLYGQLLW
REFFYTAATNNPRFDKMEGNPICVQIPWDKNPEALAKWAEGRTGFPWIDAIMTQLRQEGWIHHLARHAVACFL
TRGDLWISWEEGMKVFEELLLDADWSINAGSWMWLSCSSFFQQFFHCYCPVGFGRRTDPNGDYIRRYLPVLRG
FPAKYIYDPWNAPEGIQKVAKCLIGVNYPKPMVNHAEASRLNIERMKQIYQQLSRYRGLGLLASVPSNPNGNG
GLMGYAPGENVPSCSSGGSCSQGSGILHYAHGDSQQAHLLKPGRSSMGTGLSSGKRPSQEEESQSVGPKVQRQ
SSN
>Methanosarcinales_arc_NYT01758.1_photolyase
MNLKRVKVLREGSAKAGPVIYWMSRDQRVRDNWALIYAQSVALKRAVPLAVIFSLALRFLGATWRQYSFMLSG
LSLVERQLQELNIPFHLLSGKPAEVVPDFLMEEGAGVLVTDFDPLREKRSWKAAVARKTTIPFYEVDAHNIVP
CWQASPKQEYAAYTIRPKIGRALAEYLEDFPALERHPRTGEEGAGGGGNDWDGARRLLESEGRVPPVRWLSPG
EDAAWSCLQDFVNNRLDSYPEARNDPSVDGQSNLSPYLHFGQLSAQRVALEVQRSRARPESRAAFLEELIVRR
ELSDNFCFYNPHYDGFQGFPTWARETLDEHRDDAREYIYTSQELEEARTHDDIWNAAQMEMVRRGKMHGYLRM
YWCKKILEWSESPEVAQKTAIGLNDRYELDGRDPNGYVGVAWSVGGVHDRAWGERNIFGKIRYMSYKGIKSKF
DIQSYISRISSLPPE
>Methanosarcina_maz_WP_048036566.1_photolyase
MNPKRIRALKSGKHGDGPVVYWMSRDQRVEDNWALLFSRAIAKEANVPVMVVFCLTGEFLEAGIRQYEFMLKG
LQELEVSLSRKKIPFFFLRGDPGEKISRFVKDYNAGTLVTDFSPLRIKNQWIEKVISGISIPFFEVDAHNVVP
CWEASQKHEYAAHTFRPKLYALLPEFLEEFPELEPNSVTSELSAGAGMVETLSDVLETGVKALLPERALLKNK
DPLFEPWHFEPGEKAAKKVMESFIADRLDSYGALRNDPTKNMLSNLSPYLHFGQISSQRVVLEVEKAESNPGS
KKAFLDEILIWKEISDNFCYYNPGYDGFESFPSWAKESLNAHRNDVRSHIYTLEEFEAGKTHDPLWNASQMEL
LSTGKMHGYMRMYWAKKILEWSESPEKALEIAICLNDRYELDGRDPNGYAGIAWSIGGVHDRAWGERGVTGKI
RYMSYEGCKRKFDVKLYIEKYSAL
>Methanothermobacter_the_WP_010876537.1_photolyase

MIHAERIRNLNGEEPDLRGSYVVYWMQASVRSHWNHALEYAIETANSLKKPLIVVFGLTDDFPNANSRHYRFL
IEGLRDVRSNLRERGIQLVVERDSPPSVLLKYADDAAAAVTDRGYLDIQKEWVDEAAGALHIPLTQVESNVIV
PVETASDKEEYSAGTFKPKIKRHLKRFMVPLRMRTLKMDSLDLEPGPEFEDAVRDFRAPEDLEPSVFRGGTST
ALSIFSEFLREKLECFERYRNDPVKNCLSNMSPYLHFGQISPLYLALRASEAGECPEFLEELIVRRELSMNFV
HYSDSYSSISCLPEWAQRTLMDHVADPREYEYSLRELESASTHDPYWNAAQQEMVITGKMHGYMRMYWGKKIL
EWTDHPARAYDIALYLNDRYEIDGRDPNGFAGVAWCFGKHDRAWAEREIFGKVRYMNDRGLKRKFRIDEYVDR
IRGLMDE
>Microcaecilia_unicolor_XP_030056693.1_cryptochro
MVVNSVHWFRKGLRLHDNPALQAALRGAQTVRCIYILDPWFAASSSVGIIRWRFLLQSLEDLDNRLRKLNSRL
FVVRGQPADVFPRLFKEWGVNRLTFEYDSEPFGKERDAAIMKMAKESGVEVIVENSHTLYDLDRIIELNGQKP
PLTYKRFQAIISRMELPRKPVCSITQQQMETCRADIQENHDDTYGVPSLEELGFCTEGLGPAVWCGGETEALA
RLDKHLERKAWVANYERPRMNANSLLASPTGLSPYLRFGCLSCRLFYYRLRDLYKKVKRNNLPPLSLYGQLLW
REFFYTAATNNPKFDRMEGNPICVQIPWDKNPEALAKWAECKTGFPWIDAIMTQLRQEGWIHHLARHAVACFL
TRGDLWNSWESGVKVFDELLLDADFSVNAGSWMWLSCSAFFQQFFHCYCPVGFGRRTDPSGDYVRRYLPKLKA
YPSRYIYEPWNAPESVQKAAKCIVGVDYPKPIVNHAEASRLNIERMKQVYQQLSRYRGLCMLASVPSCSEDLS
NPLADPASGQGYKTGTGEKSTYKVHRYNSLELQEKDTQIQRDLKVDS
>Microcaecilia_uni_XP_030070014.1_cry-1
MGVNAVHWFRKGLRLHDNPALRECIQGADTVRCVYILDPWFAGSSNVGINRWRFLLQCLEDLDANLRKLNSRL
FVIRGQPADVFPRLFKEWKIAKLSIEYDSEPFGKDRDAAIKKLASEAGVEVIVRISHTLYDLDKIIELNGGQP
PLTYKRFQTLISRMEPLEMPVETITSEVMETCTTPVSDDHDEKYGVPSLEELGFDTEGLLSAVWPGGETEALT
RLERHLERKAWVANFERPRMNANSLLASPTGLSPYLRFGCLSCRLFYFKLTDLYKKVKKNSSPPLSLYGQLLW
REFFYTTATNNPRFDKMEGNPICVQIPWDKNPEALAKWAEGRTGFPWIDAIMTQLRQEGWIHHLARHAVACFL
TRGDLWISWEEGMKVFEELLLDADWSVNAGSWMWLSCSSFFQQFFHCYCPVGFGRRTDPNGDYIRRYLPVLRG
FPSKYIYDPWNAPDSVQKGAKCIIGINYPKPMVNHAEASRLNIERMKQIYQQLSRYRGLGLLASVPSNPNGNG
NGGLMSYSPGESTTTGAQMGGTDGHATGIQTVSQVEPNTGSSGIQHQGYCQGSSLHYAHGDSQQNHLLQGKST
LNTGVISGKRPNPEEEKQSIGPKVQRQSTV
>Microcebus_muri__XP_012628233.1
MGVNAVHWFRKGLRLHDNPALKECIQGADTIRCVYILDPWFAGSSNVGINRWRFLLQCLEDLDANLRKLNSRL
FVIRGQPADVFPRLFKEWNIAKLSIEYDSEPFGKERDAAIKKLATEAGVEVIVRISHTLYDLDKIIELNGGQP
PLTYKRFQTLISKMEPLEIPVETITSEVIEKCTTPLSDDHDEKYGVPSLEELGFDTDGLSSAVWPGGETEALT
RLERHLERKAWVANFERPRMNANSLLASPTGLSPYLRFGCLSCRLFYFKLTDLYKKVKKNSSPPLSLYGQLLW
REFFYTAATNNPRFDKMEGNPICVQIPWDKNPEALAKWAEGRTGFPWIDAIMTQLRQEGWIHHLARHAVACFL
TRGDLWISWEEGMKVFEELLLDADWSINAGSWMWLSCSSFFQQFFHCYCPASFGRRTDPNGDYIRRYLPVLRG
FPAKYIYDPWNAPEGIQKVAKCLIGVNYPKPMVNHAEASRLNIERMKQIYQQLSRYRGLGLLASVPSNPNGNG
GFMEYSPAENIPGCSSSGNCSQGSGILHYAHGDSQQTHLLKQGRSSMGTGFSGGKRPSQEEDTQSIGSKVQRQ
STN
>Microcebus_muri__XP_012645570.1
MKKARAKDHIWFLLQSLEDLDTSLRKLNSRLFVVRGQPADVFPRLFKEWGVTRLTFEYDSEPFGKERDAAIMK
MAKEAGVEVVTENSHTLYDLDRIIELNGQKPPLTYKRFQAIISRMELPKKPVGSVTSQQMESCRAEIQENHDE
TYGVPSLEELGFPTEGLGPAVWQGGETEALARLDKHLERKAWVANYERPRMNANSLLASPTGLSPYLRFGCLS
CRLFYYRLWDLYKKVKRNSTPPLSLFGQLLWREFFYTAATNNPRFDRMEGNPICIQIPWDRNPEALAKWAEGK
TGFPWIDAIMTQLRQEGWIHHLARHAVACFLTRGDLWVSWESGVRVFDELLLDADFSVNSGSWMWLSCSAFFQ
QFFHCYCPVGFGRRTDPSGDYIRRYLPKLKGFPSRYIYEPWNAPESIQKAAKCIIGVDYPRPIVNHAETSRLN
IERMKQIYQQLSRYRGLCLLASVPSCVEDLNHPVAEPSSSQAGSMSSTGPRPLPSGPASPKRKLEAAEEPPGE
ELSKRARVTELPTPELPSKDA
>Micromonas_comm_XP_002508013.1
VVWFRQDLRVRDNPALHAAARTGRPVVACFVWCPKEEGGWPMGGATRYWLHHALGSLQASLRARNAGGTSSPS
SSSSSSSSFDELAAVVRECGAKDVYCNRVYEPWKIARDRECERKFSAELNVRFRSFNAGVLYEPWDARPDATD
DACWNSGYGSVRFFLRGCARLGEPPPPLPPPPTMRVRLPADKRARSYTEHGGVQSNPGNGQSNGQSNAPKAST
ERFRADVRTTARISPYVRHGELSVREVYHSAKAIQVGSRKSRARSAAVFLRRLAWRDLAYWSLWRFPRLCDEP
LRPQYATQWWALPWDPVAWQFGQTGYPLVDAGMRELWATGYVPNYVRHVVAGFLIEYLNVDWRHGQLWFHDTL
VDADVAIQGFMWQNGGHSGMDQWNFVMHPVYAAKSADPDGEYVRRWCPELSGLPREYVHCPWEAPATTLAAAN
VALGRHYPKRIV
>Micromonas_com_XP_002507183.1_protein
MNRLVNEKRVRVLKEGKVGTGPVIYWCSRDQRVRDNWALIYACETANETGAPVVVVFSLVTRFLGAGARQFCF
MLKGLREMEQALLAKNIKFVLLEGDPSLTVPRFAKECGASLIVADQSPLRLGRTWRVAIASSVDCPLHEVDAH
NVVPVWEASQKLEVGARTLRGKLAKLYPEFLVEFPETPVVKETWPCAAAAALPTIGIDWEALIVRARNDGAAV
PEVTWAVPGEFAAHATLEHFLTRRLKYYEHRNDPAKPQALSGLSPYLHFGQISAQRCALEARRYAKSSNKAVD
VFLEELIVRRELADNFCWYSPKYDTIEGQKYEWAKETVREHSKDKRAYLYSLQQFENGETHDDLWNAAQLEMV
HGGKMHGFMRMYWAKKIMEWTESPDQALEFAIHLNDKYQLDGRDPSGYVGCMWSIVGVHDQGWSERPIFGKIR

YMAYSGCVKKFKVQDYIKRVDELVTAVKAGEKDTAVVNPGRFHIEVSRSLDCDSLAVTGSRVCSEDELQRFVR
VASANARSDTTRSLEALRALKSVDITAETLIKTGVGKELKKLSKQRENPSVSSAAAAIVQAWQKLLLP
>Micromonas_com_XP_002508365.1_protein,_p
RVVVWFRNDLRLQDNYAVKQAESIATRTAGCDVLPLYVFDPRTFAPSSWGSPKCGGHRGRFQLESVLNLKRNL
RAIGSDLLVAVGKPEEVIPKYLLDAPGAKNEEVCSEELRVDAKVKSAVKSAGNLNRLDKIWGSTLYHKDDLPF
RSDASDLPDVFTPFRNKVESKSEPREPVPPPAKGALPVPSDMTEAFAFEPTVADLPFANEDERAIAAAGAHPD
GVLPFEGGESAALARVRYYVWESEKIATYFETRNGMLGGDYSSKLAPWLAHGCVSPRQVVAEVRKFESQRVEN
KSTYWLIFELIWRDFFKFFALKHGNAIFFSEGTAGGKMGGAGYKGGAGPWRDDPAALAAWKEGKTGYPLVDAN
MRELAATGFMSNRGRQNVASWLALDAGVDWRLGAEWFENKLLDYDCSANWGNWVAAAGMTGGRVNKFNIAKQT
KDYDPEGEYIKYWVPELRKVPAKYIAEPRQMPGDVAQSAGCVIGVDYPAP
>Micromonas_com_XP_002508785.1_protein
MAAKRTILWFRKGLRVHDNPALVKACEGASAVQPVFVLDPWFIKPERVGANRLRFLLESLTDLDASLRARGSS
LLVLHGDPARVIPAALEAWRCDRLCYEFDTEPYAQKRDASVNEAARALGVEVHAPVSHTLWDLDRLLAKCPRG
EPPTAYASFLKIAHGVGPVPPVAPDVPSITPLSDGDALSDGKDLVDALRDVVTGSGIPSLERLGYEPIKAGEG
FPARGGETEGLARLRKMLARTTYIAEFKKPQTNPTELWTPVLGKEGKVKGAPPANPFEAAKKSSKEPKSTQLA
EAFMAPATTALSPYLKFGCVSPRTFWHELRAVLDVELGGKHSKPPESLEGQLLWREFYYLAGYGTPNYDRMAG
NRICRQIPWTWDEERLAAWEESRTGFPWIDACMMQLKQEGWMHHLARHAVACFLTRGDLFVHWEAGAAVFDRE
LVDADWALNNGNWMWLSCSCFFYQYFRVYGPVSFGKKYDKEGAYIRKYLPQLKDMPAKYIYEPWTAPIEVQRK
AGCVVGVDYPAPIVDHAVASKECIEKIAAAYNAHKNGTAGSIVAAGKKRKAGDGV
>Micropterus_dolo__XP_045889012.1
MLVLLVVNLEGERASGGSYPTLPQPLFTGMGHTCIHWFRKGLRLHDNPALMAALRDCRELYPVFILDPYLHNN
TCMGINRWRFLIGALKDLDCSLRKHNSRLFVVRGKPEDVLLKLFSKWKVTKLTYEYDTEPYSLSRDKTVTALA
KEHGLEVIYKISHTLYDIDRIIEENNGKPPITYNRMQAIVKTLGPPKRPIPAPTMEDMKDVKTPCSENHEKEY
GIPTLEELSQGTAALREEQFPGGEQEALRRLDEHMKRTEWVCSFEKPQTSPNSLSPSTTVLSPYVTFGCLSAR
TFWWRLTDVYHGKKHSAPPVSLHGQLLWREFFYTASVGIPNFNKMEGNPVCTQVDWDTNPEHLAAWTEARTGF
PFIDAIMTQLRQEGWIHHLARHAVACFLTRGDLWISWEEGQKVFEKLLLDGDWALNAGNWQWLSASTFFHQFF
RVYSPVAFGKKTDKNGDYIKKYLPLLKKFPAAYIYEPWKAPRNIQQEAGCIVGKDYPHPIAQHEVISKKNIQR
MKLAYAKRSADPAESPRKNQGVKRKTPSVVDMLKKKQRRK
>Micropterus_dolo__XP_045892992.1
MGVNTIHWFRKGLRFHDNPSLRDSIRGADTLRCIYILDPWFAGSSNVGINRWRFLLHCLEDLDASLRKLNSRL
FVIRGQPTDVFPRLFKEWQISRLSYEYDSEPFGKERDAAIQKLASEAGVEVMVRISHTLYDLAKIIELNDGQP
PLTYKRFQALINRMDAVELPAETITPEVVKKCATPTSDDHDDKFGVPSLEELGFETEGLTTAVWPGGETEALM
RLERHLERKAWVANFERPRMNANSLLASPTGLSPYLRFGCLSCRLFYFKLTDLYRKVKKNGTPPLSLYGQLLW
REFFYATATNNPCFDKMEGNPVCVQIPWDKNPEALAKWAEGRTGFPWIDAIMTQLRQEGWIHHLARHAVACFL
TRGNLWISWEEGMKAFDELLIDADWSVNAGSWMWLSCSSFFQQFFHSYCPVGFGRRTDPNGDYIRRYLPILRG
FPAKYIYDPWNAPEEVQKASKCIIGVHYPKPMVNHAEASRVNIERMKQIYQQLSCYRGLGMLATVPANPKNGG
NDCNLKDVNTWTSQSPGGSSENHSIHEGMSQTERGQSSQKRRREETPLQSTSKFWRQNK
>Micropterus_dolo__XP_045893137.1
MPPSAAAGAEDSKALVRKMLREVLVGREDPEGFFAICVSALGHQETRTQFMSLIQPLSTASSSLHSILTSIYK
EYFSKTEDKELEHALALSLLEMKDYRLSTPRQEPGDRLNQRSSSQLAVVSRPPQTPQETELQKSTRVDKFNKE
TPGTSQTVCVSRLSGSFSKQNTIEEGGHMMDGGDLNQSEKLKRSKNRRQRRKGANQQILGLPCSPLAPPPVLL
WFRRDLRLCDNPALMGSLEVGAPVIPVFIWSPEEEEGPGITVAMGGACKYWLHQALSCFCSSLERIGSHLVIL
KANGEGNKVGSSLRTLKDLVKETGARTVLANAVYEPWLKERDDAVVSALQKDGVKFRMFHSYCLRDPYSVSTE
GVGLRGIGSVSHFMSCCKQNPGSALGVPLDPPASLPTPAHWPQDVSLDALGLASMPHRKDGTTIDWAANIRKS
WDFSEGGAHARQETFLHDGVYRYEKESGRADAPNTSCLSPYLHFGQLSPRWLLWDAKGARCRPPKFQRKLAWR
DLAYWQLTLFPDLPWESLRPPYKALRWNSDRGHLKAWQRGRTGYPLVDAAMRQLWLTGWMNNYMRHVVASFLI
AYLHMPWQEGYRWFQDTLVDADVAIDAMMWQNGGMCGLDHWNFVMHPVNAAMTCDPYGSYVRKWCPELAALPD
ELIHKPWKCPASMLRRAGVVFGQTYPERIVTDLDERRSQSLQDVALVRKVFGQYVDKRSGCDLVPLPPRLVSE
ALGLSHRDGDVVTEGKQFLLPVITRMEFKHQLQDPDADAASNPYNAVLNGYVSRKRDETIAFLNERDFTASVM
HEGTQRKERLERDYRRMEGLTRPPASRGRARRTSTAKDRFSIVPAVAVTSLR
>Micropterus_dolo__XP_045893397.1
MVVNSVHWFRKGLRLHDNPALQEALNGADTVRCVYILDPWFAGAANVGINRWRFLLEALEDLDSSLKKLNSRL
FVVRGQPTDVFPRLLKEWEVTRLTFEYDPEPYGKERDGAIIKMAQEFGVETIVRNSHTLYNLDRIIEMNNNSP
PLTFKRFQTIVSRLELPRRPLPPITKQQMDNCHTKIADNHDQLYSIPSLEELGFRTEGLPPAVWRGGESEASD
RLNKHLDKKVWVANFEHPRVNRCSLYASPTGLSPYLRFGCLSCRVLYYNLRELYMKLRKRCSPPLSLFGQLLW
REFFYTAATNNPNFDRMEGNPICVQIPWDQNPEALAKWAEGRTGFPWIDAIMTQLRQEGWIHHLARHAVACFL
TRGDLWISWESGMKVFEELLLDADWSVNAGSWMWLSCSAFFQQFFHCYCPVGFGRRTDPSGDYIRHYIPILKD
YPNRYIYEPWNAPESVQKAANCVVGVDYPKPMINHAEGSRLNIERMKQVYQQLSHYRGLSLLASVPTIQEEAE
PPMTDESQTSSGPDSPPRGPADSEAAGCSTAPDSSTVCASSTYAPYPEPEDAVNIHPSQTPCPSSSSRTQPSA

AITSTASNPLSTPINSSPAHCPLSVSPAASCSTLSSSLSPASTATHSSLGHRRKGLACKVRRSQRQRGRLICT
PAAREGERRSEEEEEREEAEGEERMEEDVEQDEERMEEATSGETAGHQQ
>Micropterus_dolo__XP_045908458.1
MLHCIWRSSPFHCAPRRLLRFVAYRNQRPSSVFSCLTKSFTSEITMSAVKRKATFAAAAEEPSAKQQKLAPMK
EKKERAGGWLQDLVTKQRTEMKELKFNNKRLRFISDTKKIKQGSEGVLYWMSRDQRVQDNWALIHAQQLAMKE
SLPLHICVCLHVPKSELSTLRHYSFMLKGLEEVAEECKTLDIQFHLLHGSPGEVVPGFVSDCSLGAVVTDFSP
LREPLQWLEDVKKMLPKDIPFIQVDAHNIVTCWEASPKLEYAARTIRGKINKLLPEFLTQFPLVEKHPYTATR
TTKQVDWDKTLASLQVDKTVRETEWAKPGTKAGLAMLESFIDVRLKQFDTKRNDPNAAALSQLSPWIRFGHLS
AQRVALQVQSSGKSAGQSVSSFIEELVVRRELTDNFCFYNKKYDSVEGAYEWAQKTLKDHGKDKRPYLYTCEQ
LEKAKTHDKLWNAAQYQMITEGKMHGFLRMYWAKKILEWTSSPKEALSIALYLNDRYELDGQDPNGFVGCMWS
ICGIHDQGWGERPVFGKIRYMNYKGCLRKFDVAQFERKYCPKNL
>Micropterus_dolo__XP_045924268.1
MSTSRTIICLLRNDLRLHDNELFHWAQRNAEYIVPLYCFDPRHYVGTYNYNLPKTGPFRLRFLLQSIRDLRST
LLNKGSNLVVRHGKPEEVVADLIKQLGSVSAVAFHEEVCSEELNVEKRVKDVCALMKIKVHTCWGATLYHRDD
LPFHHISRLPDVYTQFRKAVETQSRVRPVFPDPEQLKPLPQGLEEGAIPTVEDLQQTEPMTDPRSAFPCSGGE
SQALARLKHYFWDTDAVATYKETRNGLIGVDYSTKFASWLAMGCISPRYIYHQIKQYERERTANQSTYWVIFE
LLWRDYFRFVGVKYGNRLFQVKGLQDKSVPWKKDMELFNAWKEGRTGVPFVDANMRELAMTGFMSNRGRQNVA
SFLTKDLGLDWRMGAEWFEYLLIDYDVCSNYGNWLYSAGVGNDPRENRKFNMIKQGLDYDNNGDYVRQWVPEL
HGIRGADVHTPWTLSTAALSHAQVSLGETYPTPIIIAPEWSRHANKKPSGMGPSPRGKKGPSHTPKQHRDRGI
DFYFSKSKNL
>Micropterus_salm__XP_038557715.1
MLVLLIVNLEGERASGGSYPTLPQPLFTGMGHTCIHWFRKGLRLHDNPALMAALRDCRELYPVFILDPYLHNN
TCMGINRWRFLIGALKDLDCSLRKHNSRLFVVRGKPEDVLLKLFSKWKVTKLTYEYDTEPYSLSRDKTVTALA
KEHGLEVIYKISHTLYDIDRIIEENNGKPPITYNRMQAIVKTLGPPKRPIPALTMEDMKDVKTPCSENHEKEY
GIPTLEELSQSTAALREEQFPGGEQEALRRLDEHMKRTEWVCSFEKPQTSPNSLSPSTTVLSPYVTFGCLSAR
TFWWRLTDVYHGKKHSAPPVSLHGQLLWREFFYTASVGIPNFNKMEGNPVCTQVDWDTNLEHLAAWTEARTGF
PFIDAIMTQLRQEGWIHHLARHAVACFLTRGDLWISWEEGQKVFEKLLLDGDWALNAGNWQWLSASTFFHQFF
RVYSPVAFGKKTDKNGDYIKKYLPLLKKFPAAYIYEPWKAPRNIQQEAGCIVGKDYPHPIAQHEVISKKNIQR
MKLAYAKRSADPAESPRKKQGVKRKTPSVVDMLKKKQRRK
>Micropterus_salm__XP_038561010.1
MPPSAAAGAEDSKALVRKMLREVLVGREDPEGFFAICVSALGHQETRTQFMSLIQPLSTASSSLHSILTSIYK
EYFSKTEDKELEHTLALSLLEMKDYHLSTPRQEPGDRLNQRSSSQLAVVSRPPQTPQETELQKSTCVDKFNKE
TPGTSQTVCVSRLSGSFSKQNTIEEGGHMMDGGDLNQSEKLKRSKNRRQRRKGANQQIVGLPCSPLAPPPVLL
WFRRDLRLCDNPALMGSLEVGAPVIPVFIWSPEEEEGPGITVAMGGACKYWLHQALSCFCSSLERIGSHLVIL
KANGEGNKVGSSLRTLKDLIKETGARTVLANAVYEPWLKERDDAVVSALQKDGVKFRMFHSYCLRDPYSVSTE
GVGLRGIGSVSHFMSCCKQNPGSALGVPLDPPASLPTPAHWPQDVSLDALGLASMPHRKDGTTIDWAANIRKS
WDFSEGGAHARQETFLHDGVYRYEKESGRADAPNTSCLSPYLHFGQLSPRWLLWDAKGACCRPPKFQRKLAWR
DLAYWQLTLFPDLPWESLRPPYKALRWNSDRGHLKAWQRGRTGYPLVDAAMRQLWLTGWMNNYMRHVVASFLI
AYLHMPWQEGYRWFQDTLVDADVAIDAMMWQNGGMCGLDHWNFVMHPVNAAMTCDPYGNYVRKWCPELAALPD
ELIHKPWKCPASMLRRAGVVFGQTYPERIVTDLDERRSQSLQDVALVRKVFGQYVDKRSGCDLVPLPPRLVSE
ALGLSHRDGDVVTEGKQFLLPVITRMEFKHQLQDPDADAASNPYNAVLKGYVSRKRDETIAFLNERDFTASVM
HEGTQRKERLERDYRRMEGLTRPPASRGRARRTSTAKDRFSIVPAVAVTSLR
>Micropterus_salm__XP_038574376.1
MLHCIWRSSPFHCAPRRLLRFVAYRNQRPSSVFSCLTKSITSEITMSAVKRKATSAAAAEEPSAKQQKLAPMK
EKKERAGGWLQDLVTKQRTEMKELKFNNKRLRFISETKKIKQGSEGVLYWMSRDQRVQDNWALIHAQQLAMKE
SLPLHICVCLHVPKSELSTLRHYSFMLKGLEEVAEECKTLDIQFHLLHGSPGEVVPSFVSDCSLGAVVTDFSP
LREPLQWLEDVKKMLPKDIPFIQVDAHNIVTCWEASPKLEYAARTIRGKINKLLPEFLTQFPLVEKHPYTATR
TTKQVDWDKTLASLQVDKTVGETEWAKPGTKAGLAMLESFIDVRLKQFDTKRNDPNAAALSQLSPWIRFGHLS
AQRVALQVQSSGKSAGQSVSSFIEELVVRRELTDNFCFYNKKYDSVEGAYEWAQKTLKDHGKDKRPYLYTCEQ
LEKAKTHDKLWNAAQYQMITEGKMHGFLRMYWAKKILEWTSSPKEALSIALYLNDRYELDGQDPNGFVGCMWS
ICGIHDQGWGERPVFGKIRYMNYKGCLRKFAVAQFERKYCPKNL
>Micropterus_salm__XP_038583812.1
MVVNSVHWFRKGLRLHDNPALQEALNGADTVRCVYILDPWFAGAANVGINRWRFLLEALEDLDSSLKKLNSRL
FVVRGQPTDVFPRLLKEWEVTRLTFEYDPEPYGKERDGAIIKMAQEFGVETIVRNSHTLYNLDRIIEMNNNSP
PLTFKRFQTIVSRLELPRRPLPPITKQQMDNCHTKIADNHDQLYSIPSLEELGFRTEGLPPAVWRGGESEASD
RLNKHLDKKVWVANFEHPRVNRCSLYASPTGLSPYLRFGCLSCRVLYYNLRELYMKLRKRCSPPLSLFGQLLW
REFFYTAATNNPNFDRMEGNPICVQIPWDQNPEALAKWAEGRTGFPWIDAIMTQLRQEGWIHHLARHAVACFL
TRGDLWISWESGMKVFEELLLDADWSVNAGSWMWLSCSAFFQQFFHCYCPVGFGRRTDPSGDYIRHYIPILKD
YPNRYIYEPWNAPESVQKAANCVVGVDYPKPMINHAEGSRLNIERMKQVYQQLSHYRGLSLLASVPTIQEEAE

PPMTDESQTSSGPDSPPRGPADSEAAGCSTAPDSSTVCASSTYAPYPEPEDAVNIHPSRHPAPHQAHAHSPLQ
P
>Micropterus_salm__XP_038584736.1
MSTSRTIICLLRNDLRLHDNELFHWAQRNAEYVVPLYCFDPRHYVGTYNYNLPKTGPFRLRFLLQSIRDLRST
LLIKGSNLVVRHGKPEEVVADLIKQLGSVSAVAFHEEVCSEELNVEKRVKDVCALMKIKVHTCWGATLYHRDD
LPFHHISRLPDVYTQFRKAVETQSRVRPVFPDPEQLKPLPQGLEEGAIPTVEDLQQTEPVTDPRSAFPYSGGE
SQALARLKHYFWDTDAVATYKETRNGLIGVDYSTKFASWLAMGCISPRYIYHQIKQYERERTANQSTYWVIFE
LLWRDYFRFVGVKYGNRLFQVKGLQDKSVPWKKDMELFNAWKEGRTGVPFVDANMRELAMTGFMSNRGRQNVA
SFLTKDLGLDWRMGAEWFEYLLIDYDVCSNYGNWLYSAGVGNDPRENRKFNMIKQGLDYDNNGDYVRQWVPEL
HGIRGADVHTPWTLSTAALSHAQVSLGETYPTPIIIAPEWSRHANKKPSGMGPSPRGKKGPSHTPKQHRDRGI
DFYFSKSKNL
>Mirounga_leon__KAF3814493.1
MEPLEIPVETITSEVIEKCTTPLSDDHDEKYGVPSLEELGFDTDGLPSAVWPGGETEALTRLERHLERKAWVA
NFERPRMNANSLLASPTGLSPYLRFGCLSCRLFYFKLTDLYKKVKKNSSPPLSLYGQLLWREFFYTAATNNPR
FDKMEGNPICVQIPWDKNPEALAKWAEGRTGFPWIDAIMTQLRQEGWIHHLARHAVACFLTRGDLWISWEEGM
KVFEELLLDADWSINAGSWMWLSCSSFFQQFFHCYCPVGFGRRTDPNGDYIRRYLPVLRGFPAKYIYDPWNAP
EGIQKVAKCLIGVNYPKPMVNHAEASRLNIERMKQIYQQLSRYRGLGLLASVPSNPNGNGGLMGYSPGENIPG
CSSSGSCSQGSGILHYAHGDSQQTHLLKQGRSSMGTGLSTGKRPSEEEDTQSIGPKVQRQSTN
>Mirounga_leon__XP_034868536.1
MAAAVVTAVAAAQASAAGVDGASSVHWFRKGLRLHDNPALLAAVRGARCVRCVYILDPWFAASSSVGINRWRF
LLQSLEDLDTSLRKLNSRLFVVRGQPADVFPRLFKEWGVTRLTFEYDSEPFGKERDAAIMKMAKEAGVEVVTE
NSHTLYDLDKIIELNGQKPPLTYKRFQAIISRMELPKKPVGSVTSQQMESCRADIQENHDDTYGVPSLEELGF
PTEGLGPAVWQGGETEALARLDKHLERKAWVANYERPRMNANSLLASPTGLSPYLRFGCLSSRLFYYRLWDLY
KKVKRNSTPPLSLFGQLLWREFFYTAATNNPRFDRMEGNPICIQIPWDRNPEALAKWAEGKTGFPWIDAIMTQ
LRQEGWIHHLARHAVACFLTRGDLWVSWESGVRVFDELLLDADFSVNAGSWMWLSCSAFFQQFFHCYCPVGFG
RRTDPSGDYIRRYLPILKGFPSRYIYEPWNAPESIQKAAKCIIGVDYPRPIVNHAETSRLNIERMKQIYQQLS
RYRGLCLLASVPSCVEDLSNPVAEPSSGQTGSMSSAGPRTLPSGPASPKRKLEAAEEPPGEELSKRARVAEVP
APELPSRDV
>Misgurnus_angu__BBI18597.1
MAPNSIHWFRKGLRLHDNPALQEAVRGADTVRCVYFLDPWFAGSSNLGVNRWRFLLQCLDDLDSSLRKLNSRL
FVIRGQPANVFPRLFKEWKISRLTFEYDSEPFGKERDAAIKKLAMEAGVEVIVKISHTLYNLDKIIELNGGQP
PLTYKRFQTLISRMDPPEMPEETLSNAMMGCCVTPVSEDHGDKYGVPSLEELGFDIEGLPSAVWPGGETEALT
RIERHLERKAWVANFERPRMNANSLLASPTGLSPYLRFGCLSCRLFYFKLTDLYRKVKKTSTPPLSLYGQLLW
REFFYTAATTNPRFDKMEGNPICVRIPWDKNPEALAKWAEAKTGFPWIDAIMTQLRQEGWIHHLARHAVACFL
TRGDLWISWEEGMKVFEELLLDADWSVNAGSWMWLSCSSFFQQFFHCYCPVGFGRRTDPNGDFIRRYLPILRG
FPAKYIYDPWNAPDCVQAAAKCIIGVHYPKPMVNHAEASRLNIERMKQIYQQLSRYRGLGLLASVPSTHNGNG
NGMAYSPGEQQSGSNTPAPAVSGSSVASGNRNGSILLNFGNQEHQGPSGIQQQ
>Misgurnus_angu__BBI18600.1
MSQNCIHWFRKGLRLHDNPALIAALKDCRHIYPLFLLDPWFPNNTKIGINRWRFLIEALEDLDSSLKKLNSRL
FVIRGSPSEVLPKLFEKWKITRLTFEVDTEPYSQSRDKEVMKLAEEHGVEVIPKISHTLYNIDRIIDENSGKP
PLTYVRFQTVVKNIGPPKKPVPAPTEQDMKDVSTPLSKNHEEEFGIPTLEDLGLDTSSLGPNLFPGGEKESLR
RLDEHMERTGWVCTFEKPNTSPNALTPSTTVLSPYVRFGCLSARTFWWKLTDVYRGKKHSQPPVSLHGQLLWR
EFFYTAAVGIPNFNTMKGNTICVQVDWDDNLEHLAAWREARTGFPFIDAIMTQLRQEGWIHHLARHAVACFLT
RGDLWISWEEGQKVFEELLLDADWSLNAGNWQWLSASTFFHQYFRVYSPVAFGKKTDKYGDYIKKYLPVLRKF
PAEYIYEPWKAPRSVQERAGCIVGKDYPRPIVEHEVVHKKNIQRMKAAYAKRSTDDGHDKTETKGIKRKTNAS
IKNMFAKKAKN
>Misgurnus_angu__BBI18601.1
MTHRTIHLFRKGLRLHDNPTLLAALESSSALYPVYVLETEEANRLGALRWRFILQSLEDLDGRLRALGSRLYV
LRGSTLAVLRELVTRWAVTQISYDTEVEPHYTRMDLDIRTLAQERGIKTHTCVSHTLYDVRRIIKANGGSPPL
TYKKFLHVWSVLGEPEKPVREITAEDFLRCPTPPDPDHERQFGIPSLEDLGLQVPSEVLWPGGETHALKRLQQ
HLKSQGWVANFSKPRTVPNSLLPSTTGLSPYISLGCLSVRTFYHRLSSIYAQSRNHSLPPVSLQGQVLWREFF
YTVASTTPNFIRMQGNPICLQIDWKHDPEALEKWRTAQTGFPWIDAIMTQLREEGWIHHLARHAVACFLTRGD
LWISW
>Misgurnus_angu__BBI18602.1
MSATRTVICLLRNDLRLHDNEVFCWAQRNAEHIVPLYCFDPRHYQGTYHYNFPKTGPFRLRFLLDSVIDLRAT
LKKQGSTLLVRHGKPEDVVSDLIKQLGSVTALAFQEEVASEEKNVEKKLKEVCSQNKVKVQTFWGSTLYHRDD
LPFSHIKRLPDVYTQFRKEVEGQGRIRPVFPTPEQIKSLPPGLEEGPILTFEALGQTEPVDNCRSAFPCRGGE
TEALARLKHYFWETNAVASYKETRNGLIGVDYSTKFSSWLALGCISPRYIYDQIKKYEAERTANQSTYWVIFE
LLWRDYFKFVGLKYGNRIFHINGLQDKHVPWKTNMTLFNAWKEGKTGVPFVDANMRELALTGFMSNRGRQNVA
SFLTKDLGLDWRLGAEWFEYLLVGNIHYICVGGLMVFGMITLCTVIF

>Misgurnus_angu__BBI18603.1
MSCCQQNPVSSPSTPLDSPGALPVPSSWPQGCPLVDLGLARMPRRKDGTEIDWAGNIRSSWDFSEEGAHACLE
AFLRDGVYRYEKESSRADSPNTSSLSPYLHFGQLSARWVLWDARGARCRPPKFQRKLAWRDLAYWQLCLFPDL
PWESVRPPYRALRWSSDRTHLKAWQRGGTGYPLVDAAMRQLWQTGWMNNYMRHVVASFLIAYLHLPWQEGYRW
FQDTLVDADLAIDAMMWQNGGMCGLDHWNFVMHPVDAALTCDPYGNYVRQWCPELKDLPNDLIHKPWKCPASM
LRRAGVILGENYPVRTVVDLEERRAQSLQDVTSVRRCFKEFVDQRSGCDLVPLPPRLVHEALDGREDVMICEG
SGFLLPVITRMEFKHQFEGPDGQSNPHSAVLKGYVSRKRDETIAFLNERDFTASVMSENAQRRERLERDCRLL
EGLPPPTASRGRARRTSTRDTYSRVPGGVTIPHK
>Molossus_molo__XP_036108654.1
MGVNAVHWFRKGLRLHDNPALKECIQGADTIRCVYILDPWFAGSSSVGINRWRFLLQCLEDLDANLRKLNSRL
FVIRGQPADVFPRLFKEWNITKLSIEYDSEPFGKERDAAIKKLATEAGVEVIVRISHTLYDLDKIIELNGGQP
PLTYKRFQTLVSKMEPLEIPVETITSDVIEKCTTPLSDDHDEKYGVPSLEELGFDTDGLSSAVWPGGETEALT
RLERHLERKAWVANFERPRMNANSLLASPTGLSPYLRFGCLSCRLFYFKLTDLYKKVKKNSSPPLSLYGQLLW
REFFYTAATNNPRFDKMEGNPICVQIPWDKNPEALAKWAEGRTGFPWIDAIMTQLRQEGWIHHLARHAVACFV
TRGDLWISWEEGMKVFEELLLDADWSINAGSWMWLSCSSFFQQFFHCYCPVGFGRRTDPNGDYIRRYLPVLRG
FPAKYIYDPWNAPEGIQKVAKCLIGVNYPKPMVNHAEASRLNIERMKQIYQQLSRYRGLGLLASVPSNPNGNG
GLMGYSPGENIAGCSNSGRRSSMGTGLSSGKRPSQEEDTQSIGPKVQRQSTN
>Molossus_molo__XP_036114156.1
MAATAVTATAAALAPAAGTDGASSVHWFRKGLRLHDNPALLAAVRGARCVRCVYILDPWFAASSSVGINRWRF
LLQSLEDLDTSLRKLNSRLFVVRGQPADVFPRLFKEWGVTRLTFEYDSEPFGKERDAAIMKMAKEAGVEVVTE
NSHTLYDLDRIIELNGQKPPLTYKRFQAIISRMELPKKPVGSVTSQQMGRCPAEIQENHDETYGVPSLEELGF
PTEGLGPAVWQGGETEALARLDKHLERKAWVASYERPRMNANSLLASPTGLSPYLRFGCLSCRLFYYRLWDLY
KKVKRNSTPPLSLFGQLLWREFFYTAATNNPRFDRMEGNPICIQIPWDRNPEALAKWAEGKTGFPWIDAIMTQ
LRQEGWIHHLARHAVACFLTRGDLWVSWESGVRVFDELLLDADFSVNAGSWMWLSCSAFFQQFFHCYCPVGFG
RRTDPSGDYIRRYLPKLKGFPSRYIYEPWNAPESVQKAAKCIIGVDYPRPIVNHAETSRLNIERMKQIYQQLS
RYRGLCLLASVPSCVEDLSSPVAEPTSSQTGSSGSAGPRPLPSGPASPKRKLEAAEEPPGEELSKRARVAELP
APELPSRDV
>Monodelphis_domestica_XP_007497436.1_PREDICTED:
MAATAVVTMTAAAPAPAPAPAPATASAPVPGPAPKEGASSVHWFRKGLRLHDNPALQAALRGARCVRCVYILD
PWFAASSSVGINRWRFLLQSLEDLDISLRKLNSRLFVVRGQPTDVFPRLFKEWGVTRLTFEYDSEPFGKERDA
AIVKMAKEAGVEVVTENSHTLYDLDRIIELNGQKPPLTYKRFQAIISRMELPRKPVNSVTSQQMERCQAEIQE
NHDDAYGVPSLEELGFPTDGLGPAVWQGGETEALARLDKHLERKAWVANYERPRMNAASLLASPTGLSPYLRF
GCLSCRLFYYRLWELYKKVKRNNTPPLSLYGQLLWREFFYTAATNNPKFDRMEGNPICIQIPWDRNPEALAKW
AEGKTGFPWIDAIMTQLRQEGWIHHLARHAVACFLTRGDLWVSWESGVRVFDELLLDADFSVNSGSWMWLSCS
AFFQQFFHCYCPXGFGRRTDPSGDYVRRYLPQLKGFPARYIYEPWNAPEPVQKAAKCIIGVDYPRPIVNHAET
SRLNIERMKQIYQQLSRYRGLCLLASVPSCLEDLSSPMVEASLGQAGAVSGPGLKPLPCSPASPKRKLEATEE
APGEEHSKRARVMVAVAARLPGSELAGKDV
>Monodelphis_dom_NP_001028149.1_DNA_photol
MQSRLQTASSVLEFRFNKQRVRLISQDCHLQDHSQAFVYWMSRDQRVQDNWAFLYAQRLALKQKLPLHVCFCL
APCFLGATIRHYDFMLRGLEEVAEECEKLHIPFHLLLGLPKDVLPAFVQAHSIGGIVTDFSPLLHHTQWVKDV
QDGLPKQVPFVQVDAHNIVPCWIASDKQEYGARTIRHKIHDRLPHFLTEFPPVICHPYPSNIQAEPVDWNACR
AGLQVDRSVKEVSWAKPGTASGLTMLQSFISQRLPYFGSDRNNPNKDALSNLSPWFHFGQVSVQRAILEVQKH
RSRYPDSVANFVEEAVVRRELADNFCFYNKNYDKLEGAYDWAQTTLRLHAKDKRPHLYSLEQLESGKTHDPLW
NAAQMQMVQEGKMHGFLRMYWAKKILEWTRSPEEALEFAIYLNDRFQLDGRDPNGYVGCMWSICGIHDQGWAE
REIFGKIRYMNYAGCKRKFDVAEFERKYSPAD
>Monodelphis_dom_XP_016281314.1_LOW_QUALIT
MGVNAGHWFRKRLRLPHNPALKGCIQGADTVCCVYIRDPWFGGSSNFGANEWKFLLQCLEDLDANLRKLNSRL
FVIRGQPADVFPRLFKEWNIAKLSIEYDSEPFGKERDAAIKKLASEAGVEVIVRISHTLYDLDKIIELNGGQP
PLTYKRFQTLISKMEPLATPVETITPEVMHKCVTPLSDEHDEKYGVPSLEELGFDTDGLPSAVWPGGETEALT
RLERHLERKAWVANFERPRMNANSLLASPTGLSPYLRFGCLSCRLFYFKLTDLYKKVKKNSSPPLSLYGQLLW
REFFYTAATNNPRFDKMEGNPICVQIPWDRNPEALAKWAEGRTGFPWIDAIMTQLRQEGWIHHLARHAVACFL
TRGDLWISWEEGMKVFEELLLDADWSINAGSWMWLSCSSFFQQFFHCYCPVGFGRRTDPNGDYIRRYLPVLRG
FPAKYIYDPWNAPEGIQKVAKCLIGVNYPKPMVNHAEASRLNIERMKQIYQQLSRYRGLGLLASVPSNPNGNG
SLMAYTPGENIPGCSSGGGAPVGASDGQILQACVLPEPPTGTSGVQQPGYSQGSGISHYSHEDSQQAYMLKQG
RSSLGVGGGKRPRQEEETQSINPKVQRQSTN
>Monopterus_albu__XP_020443658.1
MVVNSMHWFRKGLRLHDNPALQEALNGADTVRCVYILDPWFAGASDVGINRWRFLLEALEDLDNSLKKFHSRL
FVVRGQPTDVFPRLFKEWKVTRLTFEYDPEPYGKDRDGAIIKVAQEFGVETVVRNSHTLFNLDRIIEMNNNSA
PLTFKRFQTIVSRLELPRRPLPPVTYQQMDKCRTKMADNHDQLYSIPSLEELGFRTEGLLPAVWQGGESEALD
RLNKHLDKKVWVANFEHSRRNTCSLYASPTGLSPYLRFGCLSCRVLYYNLRELYMKIPWDQNPEALAKWAEGR

TGFPWIDAIMTQLRQEGWIHHLARHAVACFLTRGDLWITWESGMKVFEELLLDADWSVNAGSWMWLSCSAFFQ
QFFHCYCPVGFGRRTDPSGDYIRRYIPTLKDYPNRYIYEPWNAPESVQKAANCVVGVDYPKPMINHAEGSRLN
IERMKQVYQQLSHYRGLSLLASVPTIQEEAEPPMTDESQSSSGPDSPLRGPADTKAAPDSSTLCASFTSAPSD
LEDGHPSQTPCTSSGLHTWSSAAITCTSASHPPRTAASLSPATTPAQRPLSRSKPTSPPSPCPTLFPSPGPAT
MPTQTCALGPRRKGLARKMRRSQRQRGRPSSTPFATEGERRTEEERMEEDVEQDEEQMEEETSGETAGRQQ
>Monopterus_albu__XP_020445661.1
MAYQNCCLLHYLTHRVTNMAHTCVHWFRKGLRLHDNPALMAALRDCKELYPVFIVDPYLHNNTCVGINRWRFL
VGALKDLDCSLKKLNSRLFVVRGKPEDVLPRLFNKWKVTQLTYEYDTEPYSLSRDRTVAALAKERGVKVICKI
SHTIYDIDRIIEENNGKAPLTYNHMQAIVKAQSPPRRPIPAPTMEDMKDVKTPCSENHEMKYGIPTLEELGHG
TAALAEELFPGGEQEALRRLEEYMKRTEWVCSFEKPQTSPNSLSPSTTVLSPYVTFGCLSACTFWWRLTEVYQ
GKKHSKPPVSLHGQLLWREFFYTASVGIPNFNKMEGNPVCTQVDWDTNSEYLAAWREARTGFPFIDAIMTQLR
QEGWIHHLARHAVACFLTRGDLWISWEEGQKVFEEFLLDLDWALNAGNWQWLSASTFFHQFFRVYSPIAFGKK
TDKNGDYIRKYLPLLKKFPAKFIYEPWKAPRSVQQAAGCIVGKDYPSPIVQHELVSKKNIQRMKLAYAKRNST
ESQTKSPRKWHGVKRKAPSVIDMLNKKDRKNVGAN
>Monopterus_albu__XP_020457983.1
MSNAAPRTVICLLRNDLRVHDNELFHWVQTNAEYVVPVYCFDPRHYVGTYNYNLPKTGPFRLRFLLESVRDLR
NTLLSMGSNLVVRRGKPEDVIAELIKQLQSVTTVAFHKEVTSEELNVEKKLKEVCDQMEVQVHICWGSTLYHR
DDLPFHHMSGLPDVYTQFRKAVEIQSRVRPLFPTPRQLKPLPPGLEEGAIPTAADLQQKEPVTDPRSAFPCNG
GESQALARLKHYFWDTDAVATYKETRNGLIGVDYSTKFAPWLAMGCISPRYIYHQIKQYEKERTANQSTYWVI
FELLWRDYFKFVGVKYGNRLFQLKGLQDKSVPWKKDMELFNAWKEGRTGVPFVDANMRELAVTGFMSNRGRQN
VASFLTKDLGLDWRMGAEWFEYLLIDYDVCSNYGNWLYSAGIGNDPRENRKFNMIKQGLDYDSNGDYIRQWVP
ELQNIRGSDVHTPWTLSSAVLSHAHVSLGENYPTPIVIAPEWRRHINKPSGTGLSARGKKNSSHTPEPHRGRG
MDFYFSRSKNL
>Monopterus_albu__XP_020469337.1
MVVNTIHWFRKGLRLHDNPSLKESLLGADTVRCVYILDPWFAGSSNVGINRWRFLLQSLEDLDSSLRKLNSRL
FVIRGQPTDVFPRLFKEWKITRLSYEYDSEPFGKERDAAIKKLASEAGVEVTVRISHTLYDLDKIIELNGGQS
PLTYKRFQSLISCMDAVEVPAESITAEIMGKCTTPLSEDHDDKFGVPSLEELGFDTEGLSSAVWPGGETEALT
RLERHLERKAWVANFERPRMNANSLLASPTGLSPYLRFGCLSCRLFYFKLTDLYRKVKKNSSPPLSLYGQLLW
REFFYTAATNNPCFDKMENNPICVQIPWDRNPEALAKWAEGRTGFPWIDAVMTQLRQEGWIHHLARHAVACFL
TRGDLWISWEEGMKVFEELLLDADWSVNAGSWMWLSCSSFFQQFFHCYCPVGFGRRTDPNGDYIRRYLPILRG
FPAKYIYDPWNAPESVQKAAKCIIGVHYPKPMVHHAEASRLSIERMKQIYQQLSCYRGLGLLATVPSNSGGNG
NGNGETSSDGMGFPVEATHSATTASSYQMPVHSQGDWQSGVMMYLQGGPQTSISTQQQGCAGNSSSMMCYSQG
TQHIPAPVIQKGPEHHSTTQTTGKRHSKDSGNGKCSKVQKQSSH
>Monopterus_albu__XP_020479740.1
MFLCISRSLSFSSAPKRFVELVVPSHQRPIPALFFLNKLFTSETIMPNQKRKATPAAAAAATSEGSSTKRQKL
ATTKRDEGWLQDLVKQQRSENKELKFNLKRVRFISDTEKLKQGSEGVLYWMSRDQRVQDNWALIHAQQLAVKE
SLPLHVCLCLVVPKSELSTLRHYSFMLKGLEEVEKVLTXLSLNALTKAINSRDVLPGFVSDRNLGAVVTDFSP
LREPLQWLEDIKKRLTKDIPLIQVDAHNIVPCWVASPKLEYSARTIRGKITSLLPEFFTDFPVVEKHPYTATR
TAKSIDWDKTLASLHVDRTVGEPEWAKPGAKAGMAMLESFIDARLKLFGTKRNDPNAAALSQLSPWIRFGQIS
AQRVALQVNHSGKNSNTRQTVASFIEEVVVRRELTDNFCFYNKNYDSIEGAYEWAQKTLRDHAKDKRPYLYTR
QQLENAKTYDKLWNAAQCQMVTEGKMHGYLRMYWAKKILEWTSSPEDALSIALYLNDRYELDGQDPNGFVGCM
WSICGIHDQGWRERPVFGKIRYMNYKGCARKFDVAQFERNYCPKNL
>Monosiga_brevic_XP_001746666.1
MTAPGTAVRGAAAAVNPMINGHPLPWPPCLMTKKTCLQMMRSYGPQWAQSTIARASPRRHRLKRPRLPTYLIL
GTMARARPLRHRLNQWRQSRHPSAIYLTLIWRRPRQHPHSQHQRLQSTRWLQTCSTLAWGQSPPRARPRRRQL
LFNASRYKANHHSCAWCQFRCGHEYGHGCGVSPSTTPSGPIQPAGTTTTRGRLWRQGVRQDVRQGRWRIGSFG
WDWSLCSAPSACFLSFVRALVQDGMFDDLLSDSRFAKRNEKPSSLGAMLKTTRMANEDPIVVEVEAWCEGKDN
NIRALLGSLDIILWEGARWKPINIGVLTDNNQVRKAYQRACLVVHPDKLPPNNLPVIRTMAKAAKKAKVNTSA
DDDDAGHAQQNGSQGIPPGPKDFLMERVRAVTPGVDPKEGQCVLYWMSRDQRPTDNWALLYARHLAQERDVPL
RVVFCLVPKFLEATIRQFGFMLDGLQVVETHLRKKHIPFHLLTGYAKDVLPKFAEEQEACAVVCDMSPLRVPM
AWVKETGSKLKDMNVPLYQVDAHNIVPVWHASPKQEYAARTIRNKIHQKLDTCLQPFPELESNSNSVQLPDTV
DWKKARESLEINWDVKEVDWLKPGYEGGMKMLEEFINERLHRYADDRNDPNLDALSNLSPYYHFGQISVQRVV
LELRSKQRGKYAEGVKAYIEEAVVRRELSDNFCFYNHRYDSVEGASAWAQETLDVHSKDKREHLYTRKQLENA
ETADDLWNASQLQLVQEGKMHGFLRMYWAKKILEWTESPEKALEDAIYLNDKYELDGRDPNGYVGCMWSICGI
HDQGWGERPVFGKIRYMNYKGCKRKFDIAAFVKRYPPAAKNAAREAIHLRDSSFSHMCAASLMRWNTGIGPRP
SMRARRCK
>Monosiga_bre_XP_001745157.1_uncharacte
MAKAGNPRPVVVWFRNDLRVHDNEVLLQAAKASHNHVVPVYCFDIRQYSLVITHRSRRCGQFPKCGRPRARFL
IESVDDLRTRLQELGSGLVVRTGLPEEEVARVAAQVGATQVFAHQEVCSEEVAAEHRLKRQLEVPLSLHWGAV
TLCHLDDLDFGPRCKHLPSVFTQFRKRVEADMHVRPVVAAPARLAPLPSDLELGSIPTVEDLCPGQHEPDERA

VLPFKGGETAARARLQYYLWESNLLASYKDTRNGLVGGDYSSKFSPWLAHGNLTARWIYHEVKRYEQERTENT
STYWLIFELLWRDYFRFVALQHGTAIFKPGGVQHKDVPWRHSPADFEAWQNGQTGFPFIDANMRELAKTGFMS
NRGRQNVASFLTKDLQIDWRLGAEWFETLLLDHDPCSNYGNWNYAAGVGNDPRQGRHFNVIKQAKTYDPTAEY
VHLWVPELRGLDAPQAHVPFKLSSEELAQANIQLGSTYPRPVVDRLDGGRLPLKVRDETAYGPHVQLPQQGLI
KMILFDTLFTGLSW
>Monosiga_bre_XP_001747506.1_uncharacte
MSSSSISLHWFRKGQRLHDNPALWRALRGATQVYPVFVIDPHFAKPENVGVLRYNFLLESLKDLDEQLRGLGS
RLYVLRGKPEEQLPKKFKEWKVTRLTYELDTEPYARVRDAAINDLAKKHNVEVIAEAGHMLHDPESYLKKCGG
ASKDRQDKSYDVPTLKEMGYSPIKAPQMVLFPGGEREALKRLEHFIKKKNWIATFEKPKTNPAALDPDTTGLS
PYMKMGCLSVRKFWYDVQKVYDEKKDHSTPPESLHGQLLFRELFHLCGYAVKNFDKMKGNRICRQIDWDYSEK
FLDAWENSKTGYPWIDACMAQLRHEGWMHHLARHAVACFLTRGDLYQSWEHGARIFEKHLVDADWHLNNANWM
WLSCSSFFYQYFRVYSPIGFGQKYDKEGAFIRKYLPVLKDMPKKYIYEPWKAPKEVQQKAKCIIGKDYPEPIV
DHSDVSETNKDRMKACYDAHKRGGPIPGSVSRGATTDGAPTQKRPKHE
>Morone_saxa__XP_035507976.1
MAQTCIHWVPQGLRLHDNPALMAALRDCRKLYPVFIPRTPYLHNNTSVGINRWRFLIGALKDLDGSLRKLNSS
SGVDVRARSSLNVSAMLSTNLSVGECTVIKKKTESVSCDACLKGNFRGRRFKCLICYDYDLCASCYESGATTT
RHTTEHPMQCILTRVDYDLYYGGDTFSVDQPQSFTCPYCGKMGYTETSLQEHVTSEHAETSTEVGWVCSFEKP
QTSPNSLTPSTTVLSPYVTFGCLSARTFWWRLTDVYQGKKHSDPPVSLHGQLLWREFYYTASVGIPNFNKMEG
NSVCTQVDWDTNPEYLAAWTEARTGFPFIDAIMTQLRQEGWIHHLARHAVACFLTRGDLWISWEEGQKVFEEL
LLDGDWALNAGNWMWLSASAFFHQFFRVYSPVAFGKKTDKNGDYIKKYLPLLKKFPAAYIYEPWKAPRSVQQA
AGCIVGKDYPHPIVQHEVISKKNIQRMKLAYAKRSTDPAESPSKKQGVKRKAPSVVDMLTKKERKK
>Morone_saxa__XP_035520224.1
MVVNSVHWFRKGLRLHDNPALQEALNGADTVRCVYILDPWFAGAANVGINRWRFLLEALEDLDSSLKKLNSRL
FVVRGQPTDVFPRLLKEWQVTRLTFEYDSEPYGKERDGAIIKMAQEFGVETVVRNSHTLYNLDRIIEMNNNSP
PLTFKRFQTIVSRLELPRRPLPPITQQQMDKCRTKIADNHDQLYSIPSLEELGFRTEGLPPAIWQGGESEALD
RLNKHLDKKVWVANFEHPRVNTCSLYASPTGLSPYLRFGCLSCRVLYYNLRELYMKLRKRCSPPLSLFGQLLW
REFFYTAATNNPNFDRMDGNPICVQIPWDQNPEALAKWAEGRTGFPWIDAIMTQLRQEGWIHHLARHAVACFL
TRGDLWI
>Morone_saxa__XP_035520598.1
MKEQQLSTPGHQSRPQQPGDRLKQRSSLQLISVSQPQGSRHTQLVDAVGQRENTNSQTEPWVKVNPTQTTRET
ELQKNTQVYKYNKEAPETSQTVCVSFSKQEMVEEGDQVMGEGDLNKSDKPKRSKNRRQRRKGTGQQVVGLPCS
PSAPPPVLLWFRRDLRLCDNPALNGSLEVGAPVIPVFIWSPEEEEGPGITVAMGGACKYWLHQALSCFCSSLE
RIGSHLVFLRASGEGKEVGSSLRTLKQLVKETGARTVLANALYEPWLKERDNVVVSALQKDGVECRMVHSYCL
RDPYSISTDGVGLRGIGSVSHFMSCCKQNPGPALGVPLDPPVSLPTPGHWPQGVSLDTLGLARMPRRKDGTTI
DWAANIRKAWDFSEEGAHAQLEAFLQDGVYRYEKESGRADAPNTSCLSPYLHFGQLSPRWLLWDAKGARCRPP
KFQRKLAWRDLAYWQLILFPDLPWESLRPPYKALRWSSDRGHLKAWQRGQTGYPLVDAAMRQLWLTGWMNNYM
RHVVASFLIAYLYLPWQEGYRWFQDTLVDADVAIDAMMWQNGGMCGLDHWNFVMHPVDAAMTCDPYGSYVRKW
CPELADLPDELIHKPWKCPASMLRRAGVVFGQTYPERIITDLEERRSRSLQDVALVRKEFGQYVDKRSGCDLV
PLPPRLVSEALGLSQRDEGVVTAGKQFLLPVITRMEFKHQLEDPDADAASNPYSAVLKGYVSRKRDETIAFLN
ERDFTASVMYEGTQRKERLESDYRRMEGLPQPPAPRGRARRTPTAKDRFSIVPGGAVTSLK
>Morone_saxa__XP_035520960.1
MSTSRTIICLLRNDLRLHDNELFHWAERNAEYIVPLYCFDPRHYVGTYNYNLPKTGPFRLRFLLDSIRDLRNT
LLNKGSNLVVVRQGKPEEVVADLIKQLGSVSTVAFHEEVTSEELNVEKGVKDVCAQMKVKVHTCWGSTLYHRDD
LPFHHISRLPDVYTQFRKAVETQSRVRPVFPTPEQLKPLPSGLEEGAIPTAEDLQQTEPLTDPRSAFPCSGGE
SQALARLKHYFWDTDAVATYKETRNGLIGVDYSTKFAPWLAMGCISPRYIYHQIKQYEKERTANQSTYWVIFE
LLWRDYFKFVGVKYGNRLFQVKGLQDKSIPWKKDMKLFNAWKEGRTGVPFVDANMRELAMTGFMSNRGRQNVA
SFLTKDLGLDWRMGAEWFEYLLIDHDVCSNYGNWLYSAGIGNDPRENRKFNMIKQGLDYDSNGDYVRQWVPEL
QGIKGADVHTPWTLSTAALSHAHVSLGETYPNPIVTAPEWSRHVNKKPSGAGPSPRGKRGPSHTPKQHRDRGI
DFYFSRSKNL
>Morone_saxa__XP_035521005.1
MVVNTIHWFRKGLRLHDNPSLRDSIRGADSLRCVYILDPWFAGSSNVGINRWRFLLHCLEDLDNSLRKLNSRL
FVIRGQPTDVFPRLFKEWQINRLSYEYDSEPFGKERDAAIQKLACEAGVEVMVQVSHTLYNLVKIIELNDGHP
PLTYKRFQALINRMDAVELPAETITLEVIRNCATPISEDHDDKFGVPSLEELGFETEGLTTAIWPGGETEALM
RLERHLERKAWVANFERPRMNANSLLASPTGLSPYLRFGCLSCRLFYFRLTDLYMKVKKNNSPPLSLYGQLLW
REFFYTTATNNSCFDKMEGNPVCVQIPWDRNPEALAKWAEGQTGFPWIDAIMTQLRQEGWIHHLARHAVACFL
TRGDLWISWEEGMKVFEELLIDVDWSVNAGSWMWLSCSSFFQQFFHCYCPVGFGRRTDPNGDYIRRYLPILRG
FPAKYIYDPWNAPEEVQKAAKCIIGVHYPKPMVNHAEASRVNIERMKQIYQQLSSYRGLGLLATVPVHINNGG
NGKRVDAGTRQGSAGSCETPSIQEGMTSAERGQFTQKRRREEVPLESSSKSWRHSK
>Morone_saxa__XP_035526384.1

MLHCIWRSSSFPSARRTLSSPTSVFSCLRKPFNSQTIMSDKKRKARSAAKEPSAKQQKLAATKEEKKGRAGGW
LQELLEQQRTEKKEMKFNDKRLRFLSDTEKIKQGSEGVLYWMLRDHRVQDNWALVHAQRLAVEENLPLHVCVC
LHVPESELSTLRHYSFMLKGLEEVAKECKALDIQFHLLHGSAGDVLPGFVSDRSLGTVVTDFSPLREPLQWVE
DLKKRLPKDIPLMQVDAHNIVPCWVASPKLEYAARTIRGKITKLLPEFLTDFPLVEKHPYTATRTAKPVDWDK
TLTSLQLDRAVGETEWAKPGTKAGMAMLESFIDVRLKLFDTQRNDPNAPALSQLSPWIRFGHLSAQRVALQVQ
HSGKSAGQSVSSFIEELVVRRELTDNFCFYNKKYDSVEGAYEWAQKTLKDHAKDKRPYLYTREQLEKAKTHDK
LWNAAQYQVVTEGKMHGFLRMYWAKKILEWTSSPEEALSIALYLNDRYELDGQDPNGFVGCMWSICGIHDQGW
AERPIFGKIRYMNYKGCLRKFNVAQFEKKYCPKNL
>Mucor_lus_KAF1806586.1_FB192DRAFT
MTASKSVQVCFFRNVLRVHDNQSLFHALKAKPDADILPVVCLDPRMTDISILKNQGDNPVPKTWFFKLDRTKG
FRSKFYYESIMDLKQQLVKRGSDLMILYGRPEEVFPRLEKYLSTKKYQLENVHAPKEYAYEELLVEKALSKSM
KGNVKYYHDSMMVHPDDLDFAGERTYKVYTHFRKRIEKMDHPVRAPLDIPEKLPAFPEAVWDFEHAKDGFKDL
EALYKDIHVKKDSRSAFPWKGGEQSALDRLNSYVFKTHAVDEYKHTRNGMIGTEYSTKFSAFLAHGCLSPRLI
WHQMDKFNQQRKRKRSVKAGGDDDGIYWVRFELLWRDFFRYLVEGYGSRVFMLHGFRNMEKSELAKEDDKKQV
DNKPKPKNSYTDKVWKSNDDQFNKWCNGQTGTPFVDACMRELLYTGFLNNRGRQNVASYLAKDLEIDWRIGAE
YFESMLLDHDVHSNYGNWQYVAGVGCDPREGFRHFNIIKQGKDYDPDGDYIKLWCPELEHLPTHFIHCPWLMT
KEDQTKYKCQIGRDYPEPMALFDTWIKHYPAAATNGAITNYFQDNKKKVKTEKK
>Musca_dom_XP_005177055.1_photolyase
MKRARGAEVENSKSAKKQTNDAAAKPSESSPGKSTSNFIDNFKQQRLECASDVLQFSFKKKRVRILSKDQEVR
EDCNGPVVYWMSRDMRVQDNWAFLYAQRLALKLELPLVVVFCLVPKFSNATIRHYKFLLGGLEEVASECEELQ
IPFQLLMGPAKERLLEFMKEHDGAAVVCDFAPLRVPLQWVDDVKVGLEKACKCPLIQVDAHNVVPVWVASEKQ
EYGARTIRNKINSKLPEFLTEFPPVVKHKYKLKVALPNVDRKKAYESLECDMTVDEVPGIRPGYKAACKQLEE
FCTKRIKLFGEKRNDPTINALSGLSPWFHFGQISVQRCVLAVRLNKNKYKESVEAFCEEAIVRRELADNFCYY
NKNYDNLKGLHDWAAKTLNDHRKDKRSPCYSLEEFETAHTHDDLWNSAQLQLVKEGKMHGFLRMYWAKKILEW
SESPEKALEIAILLNDKYSLDGRDPNGYVGCMWSIGGIHDQGWAERAIFGKIRYMNYQGCKRKFDVNAFVVRY
GGKAYHKK
>Musca_dom_XP_005178207.1_cry-1_X1
MSVNIHWFRHGLRLHDNPALLEAISDKNRGIKLLPVFIFDGESAGTKCVGYNRFKFLLDSLKDLDDQFRAISG
GNGGRLYLFQGNPVQIFRSLHEKFRVHKLCFEQDCEPIWNERDNSVKALCHDLGIEAVEKISHTLWDPQTVID
TNGGIPPLTYQMFLHTVQVIGLPPRPADDPDWTNVEFIQIEENLIKELRGFLEFPTPEQFKIFPEKLSYLAKT
KWIGGETQALLHLRERLNVEENAFRCGRYLPNQASPNILESPKSMSAHLRFGCLSVRKFYWDVHDLFRTVQLQ
AEGFGMQMFGGAHITGQLIWREYFYTMSVNNPYYDRMEGNAICLTIPWAPTNIEQLNRWTEGQTGFPLIDAAM
RQLLAEGWLHHTLRNTVATFLTRGGLWQNWEHGLRYFLKHLLDADWSVCAGNWMWVSSSAFERLLDSSLVTCP
VALAKRLDPLGTYIKQYVPELAKVPQLFIHEPWRMPLEEQEKAECLIGVHYPSPMIDLTLATERNMKAMRDLR
MSLTSAGAPDKTPPHCRPSNEEEVRHFFWLAD
>Musca_dom_XP_005182773.1_cry-1
MSEKSTLIHWFRKGLRTHDNPALFKIFEKSHADPQKYCIRPIFILDPSLLQWLRVGANRWRFLQRSLDDLNKQ
LMALNSRLYVVRGNPNNVFPRLFKEWQTELLTFEEDIEPFALKRDAEIKTMAREHKVEVETFWSHTVFNPYTV
MQKNMGKAPLTYQKFLGIIEKMKAPEPLERPEKLKRNNVCKDGWEVEDEKCYDVPLLADLVKNMDELGEEKFP
GGETEALKRLEASLSNVGWVTAFEKPNTAPNSLEPSTTVLSPYLKFGCLSSRLFHKKLSAILKANAKHSKPPV
SLMGQLYWREFYYTAATGEPNFDRMIGNSVCMQIPWQTNDAHLEAWTYGRTGYPFIDAIMRQLRQEGWIHHLA
RHAVACFLTRGDLWISWEEGQKVFEELLLDQDWALNAGNWMWLSASAFFYQYFRVYSPVAFGKKTDPTGAYIR
KYVPELAKYPAGAIYEPWKVALGDQRKYGCVIGTDYPHRIVNHDIVHKENIKRMSAAYKVNREVKTGKEDQED
GGGGKKRKRDTSAGGGNGPSGSKAKKKKK
>Mus_musculus_NP_034093.1_cryptochro
MAAAAVVAATVPAQSMGADGASSVHWFRKGLRLHDNPALLAAVRGARCVRCVYILDPWFAASSSVGINRWRFL
LQSLEDLDTSLRKLNSRLFVVRGQPADVFPRLFKEWGVTRLTFEYDSEPFGKERDAAIMKMAKEAGVEVVTEN
SHTLYDLDRIIELNGQKPPLTYKRFQALISRMELPKKPAVAVSSQQMESCRAEIQENHDDTYGVPSLEELGFP
TEGLGPAVWQGGETEALARLDKHLERKAWVANYERPRMNANSLLASPTGLSPYLRFGCLSCRLFYYRLWDLYK
KVKRNSTPPLSLFGQLLWREFFYTAATNNPRFDRMEGNPICIQIPWDRNPEALAKWAEGKTGFPWIDAIMTQL
RQEGWIHHLARHAVACFLTRGDLWVSWESGVRVFDELLLDADFSVNAGSWMWLSCSAFFQQFFHCYCPVGFGR
RTDPSGDYIRRYLPKLKGFPSRYIYEPWNAPESVQKAAKCIIGVDYPRPIVNHAETSRLNIERMKQIYQQLSR
YRGLCLLASVPSCVEDLSHPVAEPGSSQAGSISNTGPRALSSGPASPKRKLEAAEEPPGEELTKRARVTEMPT
QEPASKDS
>Mus_mus_NP_031797.1_cry-1
MGVNAVHWFRKGLRLHDNPALKECIQGADTIRCVYILDPWFAGSSNVGINRWRFLLQCLEDLDANLRKLNSRL
FVIRGQPADVFPRLFKEWNITKLSIEYDSEPFGKERDAAIKKLATEAGVEVIVRISHTLYDLDKIIELNGGQP
PLTYKRFQTLVSKMEPLEMPADTITSDVIGKCMTPLSDDHDEKYGVPSLEELGFDTDGLSSAVWPGGETEALT
RLERHLERKAWVANFERPRMNANSLLASPTGLSPYLRFGCLSCRLFYFKLTDLYKKVKKNSSPPLSLYGQLLW
REFFYTAATNNPRFDKMEGNPICVQIPWDKNPEALAKWAEGRTGFPWIDAIMTQLRQEGWIHHLARHAVACFL

TRGDLWISWEEGMKVFEELLLDADWSINAGSWMWLSCSSFFQQFFHCYCPVGFGRRTDPNGDYIRRYLPVLRG
FPAKYIYDPWNAPEGIQKVAKCLIGVNYPKPMVNHAEASRLNIERMKQIYQQLSRYRGLGLLASVPSNSNGNG
GLMGYAPGENVPSCSSSGNGGLMGYAPGENVPSCSGGNCSQGSGILHYAHGDSQQTHSLKQGRSSAGTGLSSG
KRPSQEEDAQSVGPKVQRQSSN
>Myripristis_murd__XP_029904240.1
MAHTCIHWFRKGLRLHDNPALVAALRECKELYPVFVLDPYSYNSTHVGINRWRFLIGALKDLDCSLRKLNSRL
FVVRGKPEEVFPKLFNKWNVTKLTYEYDTEPYSLSTDKKVTTLAKEHGVEVIYKISHTLYNIDRIIEENNGKA
PLTYNRLQSVVKTLGPPKRPVPAPTIDDMKGVKTPYTDKHEKKYGIPTLEELGYDTASLSEEKFPGGEQEALK
RLDEHMKRTGWICSFEKPQTSPNSLSPSTTVLSPYVTFGCLSARTFWWRLTDVYQGKKHSDPPVSLHGQLLWR
EFFYTASVGIPNFNKMVGNPVCTQIDWDTNTEYLAAWREARTGFPFIDAIMTQLRQEGWIHHLARHAVACFLT
RGDLWISWEEGQKVFEELLLDGDWALNAGNWQWLSASAFFHQYFRVYSPIAFGKKTDKNGDYIKKYLPLLKKF
PAQYIYEPWKAPRSVQQAAGCIVGKDYPHPVVEHEVISKKNIQRMKIAYAKRSPDLTESPSKKTGKKRKAPSV
VDMLKKKK
>Myripristis_murd__XP_029909413.1
MPPAAGVDDTKVLVRKMLRELLMGREDPEGFFGLCVSLLGHQDTRSQFPALIQPLSTANRSLHSTLTSIYLDY
FSQDEDHELEMALSLSLLDVPAQYSSATKESRAQGPTDSRSLSAGIILSGSIQQSSASQPLGSSYAQLAASGG
RKKHNTDSLRPEKLQKNTHSDKDVQGGTSQNVCVSQLHTSSPKQDTGTEEDRLVQEESLEKTEKPKRSKNRRQ
RRKGGGQQVVGLPRSPSGPPPVLLWFRRDLRLCDNPALIGSLELGAPVIPVFIWSPEEEEGPGVTVAVGGACK
YWLHQALSCFRSSLERIGSHLVFLETDREGKDVGSSLQALKELVDETGARTVLANALYEPWLKERDDAVVSAL
QRDGVECRMVHSYCLRDPYSVSTEGVGLRGIGSVSHFMSCCRQNPGSGLGAPLDAPLSLPTPTHWPQGVCLDR
LGLARMPRRKDGTTIDWAANIRSSWDFSETGAHARLEAFLQDGVYRYEKESGRADAPNTSCLSPYLHFGQLSP
RWLLWDAKGARCRPPKFQRKLAWRDLAYWQLTLFPDLPWESLRPPYKALRWSSVRGHLKAWQRGRTGYPLVDA
AMRQLWLTGWMNNYMRHVVASFLIAYLHLPWQEGYRWFQDTLVDADVAIDAMMWQNGGMCGLDHWNFVMHPVD
AAMTCDPYGSYVRKWCPELAELPDELIHKPWKCPASMLRRAGVVFGQNYPERIVTDLEERRSQSLQDVALVRK
EFAEYVDKRTGCDLVPLPPRLVSEALGLSQKGGGVVTTGKLFLLPVITRMEFKHQSDDPDADAASNPYNAVLK
GYVSRKRDETIAFLNERDFTASVMYEGAQRRERLESDYRRMEGLPRPSAPRGRARRTPTAKDRFSIVPGGVVT
SPR
>Myripristis_murd__XP_029910364.1
MVVNSIHWFRKGLRLHDNPSLRDSIRGADTLRCVYILDPWFAGSSNVGISRWRFLLQCLEDLDASLRKLNSRL
FVIRGQPTDVFPRLFKEWQISRLSYEYDSEPFGKERDAAIQKLATEAGVDVMVQISHTLYDLDKIIELNGGQP
PLTYKRFQALISRMDAVEMPAETITSEVMRKCATPISEDHDDKFGVPSLEELGFETEGLSTAVWPGGETEALT
RLERHLERKAWVANFERPRMNANSLLASPTGLSPYLRFGCLSCRLFYFKLTDLYRKVKKNSSPPLSLYGQLLW
REFFYTTATNNPCFDKMEGNPMCVQIPWDKNPEALAKWAEGRTGFPWIDAIMTQLRQEGWIHHLARHAVACFL
TRGDLWISWEEGMKVFEELLLDADWSVNAGSWMWLSCSSFFQQFFHCYCPVGFGRRTDPNGDYIRRYLPILRG
FPAKYIYDPWNAPEEVQRAAKCIIGVHYPKPMVHHAEASRINIERMKQIYQQLSCYRGLGLLASVPANPNSGT
NGSNAGGANTGTGTCPAASNEDPAMQEGTSQTGRDGFTSSLAQTFLFTVPQTQQTRYKISLHLSVYCVS
>Myripristis_murd__XP_029930453.1
MLPRLCGRHVLKLLSQTKQSSCSPFTSLTKLSTSPTTMSAVKRKAPTSTKSPDAKQQKLAPHKKEEKKERVEG
WLQAHVLQQRRDKKEMKLNKKRVRYISDTQKIKQGSEGVLYWMSRDHRIQDNWALIHAQKLAVAENLPLHICV
CLVVPKSELSTLRHYTFMLKGLEEVAKECKTLDIQFHLLHGTAGQVLPGFVSDQGLGAVVTDFSPLREPLQWL
EDVKKQLPKDIPFIQVDAHNVVPCWVASGKLEYSARTIRGKITNLLPEFLTDFPPVVKHPCTATRTAKPVDWT
KTLASLQVDKTVGEVSWATPGTRAGMAMLESFIDVRLKLFGTQRNDPNRAALSQLSPWIRFGHLSAQRVALEV
RRLGKNAGESVSSFIEELVVRRELTDNFCFYNKNYDSVKGAYEWAQKTLKDHAKDPRPYVYTREQLEKAKTHD
KLWNAAQYQMVSEGKMHGFLRMYWAKKILEWTSSPEEALSIALYLNDRYELDGQDPNGFVGCMWSICGIHDQG
WAERPIFGKIRYMNYKGCLRKFDVAGFEKKYCPKNL
>Myripristis_murd__XP_029935360.1
MSTSRTIICLLRNDLRLHDNEVFHWAQRNAEYIVPLYCFDPRHYLGSYNYNLPKTGPFRLRFLQESVRDLRNT
LLKKGSTLLVRRGKPEDVVADLIKQLGSVSAVALHEEVASEERDVEKGVKDICAQMKVKVHTCWGSTLYHRDD
LPFPHIARLPDVYTQFRKAVETQSRVRPLIPAPEQLRPVPPGLEEGAIPTAEDLGQKEPLSDPRSAFPCSGGE
SQALARLKHYFWDTDAVAKYKETRNGLIGVDYSTKFAPWLAMGCISPRYIYHQINQYERERTANQSTYWVIFE
LLWRDYFKFVGVKYGNRMFHIKGLQDKSVPWKMDMKLFSAWKEGRTGVPFVDANMRELAMTGFMSNRGRQNVA
SFLTKDLGLDWRMGAEWFEYLLVDHDVCSNYGNWLYSAGIGNDPRENRKFNMIKQGLDYDSNGDYVRQWVPEL
QGIRGADVHTPWTLSSAALSHAQVSLGETYPTPIVMAPEWSRHVNKKPSGAGPSARGKKGPSHTPKQHRDRGI
DFYFSKSKNL
>Mytilus_edul__CAG2201599.1
MSKGDAKELNMPPQDFNNRHGNVSVHWFRHGLRLHDNPALLEALKNCTEFYPIFIFDGEVAGTKHVAFNRMRF
LIESLEDLDRNLRKFGGRLYVFHGQPVEILTNLFKEWGVTKLTFEQDPEAVWKQRDDAVKELCERKEIECVER
NYRKNDGQPPLTYSLFNLVASALGDPPRPVSYPVFHNIDLPVYPDHEKKFGIPKPQTVGVFPDCKEQNNRINE
WKGGESKALDLLEKRLALEKKAYEDGYVLPNQYIPDLLGEPMSMSAHLRFGCLSVRRFHWTIHDLFEKVKPKE
SKPDALTCQLIWREYFYVMSANNINYDKMEGNPICLNIPWYRNDEILKKWEMGQTGYPWIDAIMNQLRHEGWI

HHVGRHAVACFLTRGDLWISWVDGLKIFLKYLIDADWSVSSGNWMWVSSSAFEKVLQCPKCFCPVGYGRRMDR
TGEYIRRYLPVLKDMPLRYLFEPWKAPLAVQQKAHCIVGKDYPHPIVNHKEASNECASAMNKVIESFKGKEVP
HCAPSDDLEVRRFVCLPDFVSTGGKCTANQLCPGIENL
>Mytilus_edul__CAG2210639.1
MPRNNKKQILHWFRKGLRLHDNPALKEALKGADTYRCVYILDPWFAGSTQVGIAKWRFLLQCLEDLDAALRKL
NSRLFVIRGQPTDVFPRIFREWNITTLSFEEDPEPFGKERDAAIRHLAKEAGVEVIVETSHTLYELQKIISYN
NGVPPLTYKRFQAILSKMDPPKQPEDTITRQFIGHTKTPISEDHDDKFGVPSLEELGFDTEGLGPTIFRGGET
EALARLERHLERKAWVASFEKPKMTTQSLFPSQTALSPYLRFGCLSSRTFYWKLRELFRKVKKSSDPPLSLHG
QLLWREFFYSAATNNPNFDQMTDNPICVQVPWDKNPEALAKWAEIDAMDSYAWGGYDHSVACFLTRGDLWISW
EEGMKVFDELLLDADWSVNAGTWMWLSCSSFFQQFFHCYCPVGFGRRADPSGDFIRKYLPVLKAFPPKYIYEP
WNAPESVQKAAKCIIGKDYPSPMVNHAEVSKVNTERMKQVYQHLALRASAAASIPKPLHEEFAKHGKGHKSGN
HPSKMPMMAVGNNYTHSQTSEENHPPQFRGYNMTTN
>Mytilus_edul__CAG2223839.1
MLRNIKRLFDHTHSSLICTTLKETCFKLQTTQQSLTPFKLSSEKLKSVLFNSQLFYSEIYLKFAHHNASVRQP
QVRFFSSLPNLDRMAEKKALSAKRKVDSDENPPTEKKQKLETSDIKSDNFIEKISTSRSNVCKSISEFKFNKK
RVRVLSKAKDFPEDSQGVVYWMSRDQRVQDNWAFLYAQKLALKMEVPLIVCFCLVPKFLEATIRHYHFMMEGL
KEVEQDCNKLEISFHLLIGYAKDVLPQFVTDNDIGGVVTDFSPLRMPMQWVDDVTTAIPKNIPICQVDAHNIV
PCWEASPKLEYGARTIRNKIHNQLSGYLTEFPPVCVHPFTPKEKPQSIDWVKIEASLTVDRTVGPVDWAVPGS
TAGLQMLESFCKERLKYFSKDRNNPNKIALSNLSPWIHFGQISVQRSILIVKQYRSKYKEGVEAYIEEAIIRR
ELADNFCFYNKNYDSIEGAYDWAKTSLKLHEKDKRKYLYTREKLEKGQTHDDLWNAAQIQMTIEGKMHGFLRM
YWAKKILEWTGSPTEALETAIYLNDRFNLDGRDPNGYVGCMWSICGIHDQGWKEREIFGKIRYMNYDGCKRKF
DVAAFVRKYRGKKL
>Mytilus_edul__CAG2240703.1
MASKSRIAICLLRNDLRLHDNEALSWAFKNADFVVPLYCFDPRHYKGTWHFNLPKTGSHRLKFLLESVSDLRN
SLRLKGSNLIVRVGKPEVIVPELVKTLSDVQNLVFHEEVTYEELKVEKALNHSGIDVKTFWGHTLYHKSDLSF
SVNNLPDVYTQFRKSVEAKCPVRKIIDVDDVKPVPEGLEEGQIPTMEDLGVKSVPVDPRGALNFKGGETVALD
RLNHYLWETDKVATYKETRNGMIGADYSTKFSIWLAHGCISPRKIYWEIKRYEQERTANQSTYWVIFELLWRD
YFRYVALKYGNQIFYEGGVQNKRIPWKQDKAMFEAWKDGRTGVPYVDANMREMAATGFMSNRGRQNVASFLTK
DLKLDWRMGAEWFESLLIDHDVTSNYGNWLYSAGIGNDPREDRKFNMVKQGLDYDPDGEYVRLWVPELEKIKD
GSVHHVWTLPNSVLSRAEVSLGETYPNPIVIAPEWSRHTNKKSGRNQGPPQKQNKGMDFYFKGSQKH
>Mytilus_edul__CAG2248635.1
MSSVWFSLGRESQEILNQYSNGSCEADECFCMIMSIEGYDKTRNHFINFIENLKITKPILHNGLVTVFKNYFS
ETEALHSKSNEAVGCSVENGFLTSDFGFELDMELAKSLSLSDSNPKKTSGNTPSLYDSSNEPSGSASHSITKK
PKNNKSTPDSLNGNIEKNDGHTKTSIPGLQVANLSNHKHKKKKKGNALPRQISNPPVIYWLRRDLRMYDNPA
LVAAASSNAPVILVFIWSDHEEDPLDVVATGGATKLWLHYALKFLNQSFVEKFGNGILYYNTKTEDSHNIMKQ
IIQQTGANTLFINDVYEPFLKERDDALCNKLTKSGIQCNRFHSYLLHEPSSINTESLGMRGIGSVTHFMECSR
QSSVEPIGHPIESPGILPKCTSLPNSQTLDELELAKMPRRKNGSVIDWAAPIVKSWNFSEDGAWDALGSFLTE
GIKKYEKESCRADHVNTCRISPYLHFGQISPRAILTEAKHMKSPKFLRKLAWRDLSYWLLSLWPDLPSQPTRP
QYRHQRWSKNKSHLKAWQRGNTGFPLVDAAMRQLWLTGWMNNYMRHVVASFLISYLHLHWIEGYRWFQDTLLD
ADVAINAMMWQNGGMSGLDQWNFVMHPIDAAMTCDPDGTYVRQWCPELASVPKEFIHQPWKCPPSIMKRCKVE
LGNQYPNRVITDLEVAREQSLQDVVEVRKKFPDFVDRRSGNDLVPVDNGRLLIPVITRMEFKYKTTNPEAKDN
PHTAVLRGYRSRKRDEAIAFANERDFLASTMNECVQRQERHQKALKQGNF
>Mytilus_edul__CAG2250086.1
MTKRSIHWFRKGLRLHDNPALLAACENAVDVKPIFILDPWFASSARVGVNRWRFLIQTLQNLDQSLKNTILGT
FVTCCKGYPRRSFPELFKQWETTHLTFEFDIEPYAKVRDAAVKALAEKSGVEVMTCVSNTLYDTDKVIKANGG
NPPLTYQRLQTVLSKMGAPPKPVEGPKEIKTKIAANHDTEYGVPTMADLGKDDSECGPLLFPGGESEGLKRLD
TMMKKQYYYYSLYYLYNWVCKFEKPKTEPNTLSPSTTVLSPYLKFGCVSARTFYHDVQNVYRQNKNHTQPPTS
LLGQLFWREFYYVIASVSPNFDKMEGNPICTQVDWDDNKEYLNAWREGKTGYPFIDAIMIQLKTEGWIHHLAR
HAVACFLTRGDLWCHWEEGMNVFEEWLLDADWCLNAGNWMWLSASAFFHQYFRVYSPIVFGKKTDKDGNYIRK
YLPQLKHFPTQYIYEPWTAPLKVQEKAGCVTEKRKTESSEDGTPKKRRKTEGKNSKITDFVKKSK
>Mytilus_gallopr_VDI43893.1
MASKARIAICLLRNDLRLHDNEALSWAFKNADFVVPLYCFDPRHYKGTWHFNLPKTGSHRLKFLLESMVETGV
PYVDANMREMAATGFMSNRGRQNVASFLTKDLKLDWRMGAEWFESQLIDHDVTSNYGNWLYSAGIGNDPREDR
KFNMVKQGLDYDPDGEYVRLWVPELEKIKDGSVHHVWTLPNSVMGRAEVSLGETYPNPIVIAPEWSRHTNKKS
GRNQGPPQKQNKGIDFYFKGSQKH
>Mytilus_gallopr_VDI68070.1_cryptochro
MADLGKDDSECGPLLFPGGETEGLKRLDTMMKKTNWVCKFAKPKTEPNTLAPSTTVLSPYLKFGCVSARTFYH
DVQNVYRQNKNHTQPPTSLLGQLFWREFYYVIASVSPNFDKMEGNPICTQVDWDDNKEYLNAWRE
>Mytilus_gal_VDH90443.1_cry

MRKFSQRRHLVINRILVLNIEKEHRSKIIEKNDGQPPLTYSLFNLVASALGDPPRPVSYPVFHNIDLPVYPDH
EKKFGIPKPQTVGVFPDCKEQNNRINEWKGGESKALDLLEKRLALEKKAYEDGYVLPNQYIPDLLGEPMSMSA
HLRFGCLSVRRFHWTIHDLFEKVKPKESKPDALTCQLIWREYFYVMSANNINYDKMEGNPICLNIPWYKNDEI
LKKWEMGQTGYPWIDAIMNQLRHEGWIHHVGRHAVACFLTRGDLWISWVDGLKIFLKYLIDADWSVSSGNWMW
VSSSAFEKVLQCPKCFCPVGYGRRMDRTGEYIRRYLPVLKDMPLRYLFEPWKAPLAVQQKAHCIVGKDYPHPI
VNHKEASNECASAMNKVIESFKGKEVPHCAPSDDLEVRRFVCLPDFVSTGGKCTANQLCPGIENL
>Mytilus_gal_VDI01404.1_cry
MPRNNKKQILHWFRKGLRLHDNPALKEALKGADTYRCVYILDPWFAGSTQVGIAKWRFLLQCLEDLDAALRKL
NSRLFVIRGQPTDVFPRIFREWNITTLSFEEDPEPFGKERDGAIRHLAKEAGVEVIVKTSHTLYELQKIISFN
NGVPPLTYKRFQAILSKMDPPKQPEDTITRQFIGHTKTPISEDHDDKFGVPSLEELGFDTEGLGPTIFRGGET
EALARLERHLERKAWVASFEKPKMTTQSLFPSQTALSPYLRFGCLSSRTFYWKLRELFRKVKKSSDPPLSLHG
QLLWREFFYSAATNNPNFDQMTDNPICVQVPWDKNPEALAKWAEGKTGFPWIDAIMTQLRQVGWIHHLARHSV
ACFLTRGDLWISWEEGMKVFDELLLDADWSVNAGTWMWLSCSSFFQQFFHCYCPVGFGRRADPSGDFIRKYLP
VLKAFPPKYIYEPWNAPESVQKAAKCIIGKDYPLPMVNHAEVSKVNTERMKQVYQHLALRASAAASIPKPLHE
EFAKHGKGHKSGNHPSKMPMMAVGNNYTHSQTSEENQPQQFRGYNMTTN
>Mytilus_gal_VDI14359.1_photolyase
MEYGRTRPQHNSYLNHGYDEDQDEGSDVLAHSISDQSDGEMEDTNQTFQTETEQMRQRQETSRKKSVKQLMDS
ITSLLELDGTLSSPSWWKASSSRIPSRVYLRAEIVKNFSACYEHCDNISDCNKLEISFHLLIGYAKDVLPQFV
TDNDIGGVVTDFSPLRTPMQWVDDVTTAIPKNIPICQVDAHNIVPCWEASPKLEYGARTIRNKIHNQLSGYLT
EFPPVCVHPFTPKEKPQNVDWAKIESSLTVDRTVGPVDWATPGSTAGLKMLESFCKERLKYFSKDRNNPNKIA
LSNLSPWIHFGQISVQRSILIVKQYRSKYKEGVEAYIEEAIIRRELADNFCFYNKNYDSIEGAYDWAKTSLKL
HEKDKRKYLYTREKLEKGQTHDDLWNAAQIQMTIEGKMHGFLRMYWAKKILEWTGSPTEALETAIYLNDRFNL
DGRDPNGYVGCMWSICGIHDQGWKEREIFGKIRYMNYDGCKRKFDVAAFVRKYRGKKL
>Myxococcus_xan_WP_201420854.1_photolyase
MPEGFSWSELGVDSARVQVVKDLPLPSGRRDFVLYWCMVNHRAEQNHALDAAIGLGNNLGLPVVVYQAIRPDY
PHASDRLHAWALEGMMDMATGCAARGLPYWLELPRTSKEHRPRLAQLGRRAAAIVSDLFPTYIIPGHLRGAAK
ALDVPLFAVDASCVVPMQRIATRQIGAHTLRPKLKKLWPEYLDRAVPNRAVKAAATGRKLEPDFATSDAREAR
ESLDAFDFDHSVAPIQERGGRKAGLEALQAFVHQRLEGYDEGRNDPGLARQSNLSPFFHWGNLFAGEAARAAI
RARGAQDASVQGFLEELLVRRELGFNYCFHTPGPQQLSVASLPPWAKETLTRHQKDAREHRYSLKQLETARTA
DGLWNAAQRELVERGRIHNYLRMLWGKKILEWTPSPQEALQRIAFLNDKYAVDGRDPASVANFMWVLGLHDRP
FQERQVLGKVRPMSSLRTAAKYNLAPYLERWGRPEDPPVKLKRVRKTAGP
>Naegleria_fow_KAF0984536.1_FDP41_0004
MLQQSLARGGMNTTKVIGLWFRNDLRIHDHEAFSKAVQQARTEQKKILCLYVFDRSITTQPSPYSLSQLEESC
GPRRCKFLVESVQNLFSKLNGRMMLSVLPFQTLKEEEPSASRRTERPYSIEKVREFAAELIQKFNISDLYMNR
EFCMFEKEMEKEIEKVCKKKNVKVNWTWSNCLVHGDDLPYDNFPEDIPKTFSAFRKKIEQKLANHKDTIRKVF
EMEDDFRDFLLTSEEIQKHAQTLSMIDLNDFQDIEAFMKKHLFPHMSHDQFLSEIKQDQRGIIKFIGGEDTAL
ERCHSYINKNLKNYSTTRNNNLGDYSTKLSPWLALGCISARYIYHEIKKKTSKTEEKSSSASVLINELLWRDY
FKMLAWKMGRSFFFKKALQPSKNLEYEWKIPSDGYKNAQFQAWCQGKTGYPFVDACMKELLHTGFMSNRGRMV
VASFLVKDMNIDWRLGAEYFERNLLDFDASSNVGNWCWVAGVGCDFRPRYFNPIKQGMDHDKEGKYVKQWIPE
LEKVPHNAIHLPYLLLGENEKKILKVPKHYSNKENLVCSVALPSALKRKFEKGESSTTKKRK
>Naja_naja__KAG8125988.1
MNLVPSGCTGLVLSSLAPDHPKNGRHFAWPAGPQEHRKERERERRHRKPRGRSLAGNAASGRDPPRNAAREKC
AQAGQQAVDSSAPGTGAPKRLTGFDRQAGAQPRLLRARNPEARNRLSGKHRVEARARARSCFRQSATAGPLSV
ERLASSAHAAQQGGCAAGRRAKERGSGRSGAAAPDANILGNDLKCLSPLHPSAPSRGAGLRQRLGNMNRVQLK
RSAILKMALGGASEPLLQQQQEEEEEDSEGSSSSNNNSSKEPPFLLRAVQIAVVVCLYWFISITMVFLNKYLL
GSPSLRLDAPLFVTFYQCLVTVLICKLLSLLASCCPPGFVDFPSIRIDLKVSRSVLPLSVVFIGMITFNNLCL
KFVGVAFYNVGRSLTTVFNVLLSYLLLKQTTSVFALLSCGIIIGGFWLGIDQEGAEGTLSWAGIIFGILASLC
VSLNAIYTKKVLPAVDGSIWRLTFYNNFNACILFLPLLLFTIRGQPTDVFPRLFKEWGVTHLTFEYDSEPFGK
ERDAAIVKLAKEAGVEVTTENSHTLYDLDRIIELNGHKPPLTYKRFQAIISRMDLPKKPVSTITSQQMEMYKT
EIQENHDETYGVPSLEELGFPTEGLAPAAWVANYERPRMNANSLLASPTGLSPYLRFGCLSCRLFYYRLWELY
KKVKRNSTPPLSLYGQLLWREFFYTAATNNPKFDRMEGNPICIQIPWDRNPEALAKWAEGKTGFPWIDAIMTQ
LRQEGWIHHLARHAVACFLTRGDLWISWESGVRVFDELLLDADFSVNAGSWMWLSCSAFFQQFFHCYCPVGFG
RRTDPSGDYIRQYLPQLKGFPSRYIYEPWNAPESVQKAAKCIIGVNYPKPMVNHAETSRLNIERMKQIYQQLS
RYRGLCLLASVPSCIEDFSNITTDLSSAHGSCNKTTLRQSHCTSPKRKCYAGEENSEELHKRARIMHVSTSCG
>Naja_naja__KAG8136345.1
MVHEGKMHGFLRMYWAKKILEWTRSPEEALQFSIYLNDRYELDGRDPNGYVGCMWSICGIHDQGWAERAIFGK
IRYMNYAGCKRKFDVAQFERKYRPQRFTQ
>Naja_naja__KAG8138221.1
MGEKAWKYMDVQQSEYILNLKSSAMAVAAFVHSQVPTTILVHEKLRFLLQCLEDLDANLRKLNSRLFVIRGQP
ADVFPRLFKEWNISKLSIEYDSEPFGKERDAAIKKLATEAGLEVIRFQTLISRMEPLEMPVETITAEVMSKCA

TPVSDDHDEKYGVPSLEELGFDTDGLPSAAWVANFERPRMNANSLLASPTGLSPYLRFGCLSCRLFYFKLTDL
YKKVKKNSSPPLSLYGQLLWREFFYTAATNNPRFDKMEGNPICVQIPWDKNPEALAKWAEGRTGFPWIDAIMT
QLRQEGWIHHLARHAVACFLTRGDLWISWEEGMKVFEELLLDADWSVNAGSWMWLSCSSFFQQFFHCYCPVGF
GRRTDPNGDYIRRYLPILRGFPAKYIYDPWNAPEGVQKAAKCIIGINYPKPMVNHAEASRLNIERMKQIYQQL
SRYRGLVPSNGNGNGNGNGNGNGGLMGYSSSENLPGCVGTSGTQMGTSEGHTGNVQSCTLGETHKGTSGIHQQ
GRTSLSAGVCTGKRPNPEEGIQSIGPKVQRQSSN
>Nanorana_parkeri_XP_018410013.1_PREDICTED:
MEGPSVSSVHWFRKGLRLHDNPALLAALRGACCVRCVYILDPWFAASSASSGGVNRWRLLLQSLEDLDSNLRK
LGSRLFVVRGQPADVLPRLFKEWGVTRLTFEYDSEPFGKERDAAIMKLAREAGVEVIVENSHTLYNLDKIIEL
NGNSPPLTYKRFNAIISRMELPRRPVPSITRQQMETCRAEIKSTHDETYGVPSLEELGFPRDNPGAAVWPGGE
TEALARLDRHLERKAWVANYERPRMNANSLLASPTGLSPYLRFGCLSCRLFYYRLRELYQKVKKNSPPPLSLF
GQILWREFFYTAATNNPKFDQMEGNPICVQIPWDKNPEALAKWTEGKTGFPWIDAIMTQLRQEGWIHHLARHA
VACFLTRGDLWNSWECGVKVFDEMLLDADFSVNAGSWMWLSCSSFFQQFFHCYCPVGFGRRTDPSGDYVRRYL
PVLKGYPSRYIYEPWNAPMSVQKEAKCIVGIDYPKPIVNHAEASRLNIERIKQTYQQLSRYRGLCILASVPSC
VEDLSGPITDPTSSQQGSSDPAPKLSLCAADFPKHKHTDEEPCKKARLQSVQETERTTKDF
>Nanorana_par_XP_018408992.1_cry_DASH
MCAAARVIVCLLRNDLRFHDNEVLLWAHRNADHIVPLYCFDPRHYLGTHYYNFPKTGPLRFKFLLESVQDLRN
ALKKRGSNLLLRRGHPEDVIEDLIKQLGPVSAVAFQEEATKEELDVEKSVKQVCARHGVKVQSIWGSTLYHRD
DLPFRHISSLPDVYTQFRKAVETRCKVRPTLQMPDKLKSLPAGLEEGPVPTLEDFGQQDPLMDPRTAFPCSGG
ESQALQRLDYYFWDTNLVASYKDTRNGLIGMDYSTKFAPWLAFGCISPRYIFEQIRKYEKERTANQSTYWVIF
ELLWRDYFRFVALKYGRRIFFLRGLQDKEVPWKKDQQLFDAWKEGRTGVPFVDANMRELAMTGFMSNRGRQNV
ASFLTKDLGIDWRMGAEWFEYLLMDYDVCSNYGNWLYSAGIGNDPRENRKFNMIKQGLDYDGNGDYIRLWVPE
LQQIKSGDVHTPWALSSSALAHANVTLGTTYPPPIVMAPEWSRHINQKPDSGAAPAKRGKGSSYTPKQHKNKG
IDFYFSRNKDI
>Nanorana_par_XP_018410887.1_uncharacte
MKDFFRWLYNRVEDREERPQSTMERSGHPVLPDISCMKQQLMQGEIQAEEFFCMILSTMGDNTTHRHFPDLIQ
SLSKVYPGLHDQLMSIHRAYFLQRPSEQTYEEELELAVALSLQEAQSAAGSGCTIPYPVSSSRVSFAEAAKKS
SLPQSTGDPKPSSTAKWGNVRGDSANEELSKNLAKLQTTNHLDSLYSGQPEPEDCVLHCDGREIYPIQSPIKK
EDKMKRNRRGRRKKNSSVQRNPVRMKAVVVWFRRDLRLHDNPALIAALELGAPIIPVFLWSLSEEKGQNFTLA
TGGATKYWLHHALAQLNLALAQNYGSHIVFRVNDSCADELLHLVQETGAGTVLVNAVYEPWLKDRDDLISETL
KGQGVTFTKYHSYCLYEPYSVSSEGVGLRGIGSVSHFLSCCERNSSTPIGDPLEAPTSLPLPSCWPDSLELDR
LDLAKMPRRKDGTVVDWAATIRKSWDFSEDGAYTCLGNFLEDGIKHYDKESGRADKPYTSHISPYLHFGQISP
RTVLHEAYFTKKSVPKFLRKLAWRDLAYWLLVLFPDMHVEPVRWAYKSQRWSSDQSHLQAWQKGMTGYPLVDA
AMRELWLTGWMCNYSRHVVASFLVAYLHLHWIHGYRWFQDTLVDADVAINAMMWQNGGMSGLDHWNFVMHPVD
AALTCDPCGSYVRKWCPELAGLPDEHIHKPWKCPPSQLRRAGIALGQNYPHRIIVDLEERREQSLRDVVEVRQ
KHSEYVDKVSGSDMVSVSDQLLALTLGCPDGDDDLIKSHTGRFLLPVITRKEFKYKTLQPDTKDNPYNTVLKG
YVSRKRDETIAFMNERHFTASTINEGAQMYERRERTARLMEGLPQVRDPKNKSRRTPRSDPLSIIPPAYFHLA
N
>Nanorana_par_XP_018413653.1_LOW_QUALIT
MGVNAVHWFRKGLRLHDNPALRECIQGSDSVRCVYILDPWFAGSSNVGINRWRFLLQCLEDLDANLRKLNSRL
FVIRGQPADVFPRLFKEWKISKLSIEYDSEPFGKERDAAIKKLATEAGVEVILSISHTLYDLDKIIELNGGQP
PLTYKRFQTLISKMEPLEMPAETITAEVMDKCTTPVSDDHDEKYGVPSLEELGFDVEGLPSAVWPGGESEALT
RLERHLERKAWVANFERPRMNANSLLASTTGLSPYLRFGCLSCRLFYFKLTDLYKKVPXYIFNPPXXALNWXF
FYTAATSNPRFDKMEGNPICVQIPWDRNPEALAKWAEGRTGFPWIDAIMTQLRQEGWIHHLARHAVACFLTRG
DLWISWEEGMKVFEELLLDADWSVNAGSWMWLSCSSFFQQFFHCYCPVGFGRRTDPNGDYIRRYLPILKGFPP
KYIYDPWNAPEAVQKAAKCIIGVNYPKPMVNHAEASRLNIERMKQIYQQLSRYRGLGLLASVPSNPNGNGNGG
LMSYSPGDNMPGCSNNGGSLMGTSDGASTSNPNNNNQGESHTGIGGIQGYWQGSSILHYSHGDSQQSYLIQGR
NAMNSVMSFGKRPSPEETQSIGPKVQRQSSH
>Nanorana_par_XP_018420471.1_cry-1-like
MTHRSIHWFRKGLRLHDNPALLAAMKDCAQLHPIFILDPWFPRNMRVSVNRWRFLIEALKDLDENLKKINSRL
FVVRGKPAEVFPALFKKWKVTRLTFEVDTEPYAKQRDAEIERLAAEHNVEVIQRVSNTLYDVERVIAENNGKP
PLTYVRLQTVLQSIGPPKRPCKAPTKDDMKDCCTPWKPSYDEKYGVPTLEELGQDPSQVGPLLYPGGETQALQ
RLDLNMKRTAWVCNFKKPDTEPNSLTPSTTVLSPYMKFGCLSARLFYWRLAEIYQGKKHSAPPVSLHGQLLWR
EFYYVAGAGIANFDKMEGNSVCVQLHLLYSLLYWQQWRSARTGYPFIDAIMTQLRTEGWIHHLARHAVACFLT
RGDLWISWEEGQKVFEELLLDADWALNAGNWMWLSASAFFHQFFRVYSPVAFGKKTDKNGEYIKKYLPFLKKF
PAEYIYEPWKAPRSVQERAGCIIGKDYPKPIVVHETVSKQNIQRMKAAYARRSGASAAKTEGASPKNKKGERN
GSVLQH
>Nanorana_par_XP_018427648.1_photolyase
MPPKVKPSKSKKEESGSEKSDGSQKNGKMLAPKPKSSGTAKIPAKRKAEGSATGKSKAISGASSSESSDVDAK
KKKKPEKRDDAEGGDFFDSVKKSRLATAASVSDFKFNKKRVRLVSAEENLKDDAQGIIYWMSRDQRVEDNWAF

LYAQRLAAKQKLPLHVVFCLVPKFLNATIRHYGFMLKGLKEVAEDCKELNIPFHLLIGYAKDVLPDFVKENSV
GGVVTDFAPLRVPMQWVQEVSERLPKDVPLVQVDAHNIVPCWVASVKQEYGARTIRRKIHDQLSQFLTEFPPV
IKHPYDAMFKAEPIDWEACYASLEVDRTVKEVEWAKPGSKAGVEMLQSFITQRLKSFNSDRNNPNQQALSNLS
PWFHFGQLSVQRAILEVQKYRSKCKESVDSFVEEAVVRRELADNFCYYNKNYDKVEGAYEWAKKTLKDHEKDK
RTPIYSLEKLESGKTHDPLWNAAQLQMVHEGKMHGFLRMYWAKKILEWTNSPEEALKFAIYLNDRYELDGRDP
NGYVGCMWSICGIHDQGWAERAIFGKIRFMNYQGCKRKFDVAQFERRYHPKKFAQC
>Nasonia_vit_XP_008204431.1_cry-1_X1
MTGSQGNDIGCEISCRADIYQQPQKQLQQQHHQQQSQIQQPSPPLVNNDTKSDGLAIQGDGKKHTVHWFRKGL
RLHDNPSLREGLAGASTFRCVFVLDPWFAGSANVSINKWRFLLQCLEDLDRSLHQLNSRLFVIRGQPADALPK
LFREWGTTSLTFEEDPEPYGRVRDENITTLCKELGITVVQRVSHTLYKLDEIIEKNGGKPPLTYHQFQNVIAR
MDPPEYPAAAVTAACIGSAYTPLKDDHDDFFGVPTLEELGFDTEGLMAPVWVGGETEALARLERHLERKAWVA
SFGRPKMTPQSLLPSQTGLSPYLRFGCLSTRLFYYQLADLYKKVGFYIFKDKYICILRLHFKQLYGFWTFIQI
KKTIPPLSLHGQLLWREFFYCAATNNPNFDRMHGNPICVQIPWDKNVVALSKWANGQTGFPWIDAIMTQLREE
GWIHQLARHAVACFLTRGDLWISWEEGMKVFDELLLDADWSINAGMWMWLSCSSFFQQFFHCYCPVRFGRKAD
PNGDYIRRYLPVLKNFPTRYIHEPWNAPLSVQRAAKCIIGQEYALPMVNHSKSSRINVERMKRVYQQLSKYRA
NGVSLKGETIGLLSIVPTTPSLPFDQQQQQQDDDKKQQQNSLNVDNSPNNDCPASHLHQQQLQQHVHQHQYH
>Nasonia_vit_XP_031784876.1_photolyase
MAPCIFVTKLIAIKYLSKTNFTVTKATSSTNFKMSGPSTKKLRTSHTDLVKQIEEQRNQTADSVMTFKFNKKR
VRILTDSDEVSSESKGIVYWMFRDARVHDNWAMLFAQKIALKNKVPLHVCFCILPKFLDATIRHYKFLLEALE
EVEKDCKELNINFHLLHGEPNTAIINFVEKYKMGAVIADFFPLRLPLFWLEDIKKKLPKKIPLCQVDAHNIVP
CWVASEKLEYAARTIRNKINSKLDEFLTEFPPVIKHPHTSDQKFDKINWDTALDDVLVDKSVDKITWAKAGYK
GGIAELDKFLKIRLRIYDEKRNNPIFNALSNLSPWFHFGMISVQRCILEAKKYKSQYNKSVEAFMEEAIVRRE
LSDNFCFYNEHYDSLKGAYDWARETLNQHRNDKRDYIYTLNELENGLTHDDLWNAAEIQLVKEGKIHGFLRMY
WAKKILEWTETPDDALKWSIYLNDKYSMDGRDPNGYVGCMWSICGVHDQGWRERPVFGKIRYMNYKGCERKFD
VQAFVMKYGAKVQLTKDENKRKGKKK
>Nematostella_vec_XP_001623243.2_cry_DASH
MRITLTRLQKGIVTMATSKTQKTVICLLRNDLRYHDNEALLWAHKNADFVLPLYCFDPDHYKTTWRFSLPKTA
QYRAKFLLESVTDLRSTLQIHGSNLIIRQCQPLEAVTKLTELLKPVAPVTSIVFQQEVTYEELNVEKALVEFC
KKSGIHMHTIWGSTLFHKDDIPYKAKTVPDTYTQFRKGVENQSTVRNLIDMPKNLKPLPPVKGELGTIPDLKS
LLNDSEIKEVDQRSAFPFMGGEQEALSRLGSYLWGTDSVAKYKETRNGLLGENYSTKLSPWLANGSLSPRMVY
HRIKQYEEERVANHSTYWVLFELIWRDYFKFVCLKYGDRVFYRSGIMGKSLPWKHDKMTFKLWCEGKTGVPFV
DANMRELKETGWMSNRGRQNVASFLIKDLGLDWRYGAEWFESLLLDHDVCSNYGNWNYAAGIGNDPREGRKFN
MVKQGLDYDPDGDYIKTWVPELAKIPGAKVHVPWTLKPGELRSSGVELGVTYPNPIVIAPEWARHTSKTISGK
KPSNFNPRQKGVDFYFKSANNGPSGSR
>Nematostella_vec_XP_001636254.2_photolyase
MASDEPAAKRRKQEVTGPNKSELDQLDDIVKTYKEKRMSVCNSVTDFKFNKKRVRMLSKEGSISENQCGGVVY
WMWRDQRVQDNWALLYAQRLALKQQAPLHVCFCLPSNFLDATLRQYNFMIKGLQEVEKELKELEISFHLLLGD
PGKVLPEFVKSAGIGGIVVDFCPLRLPTQWVNDVVKAVPKDVPVCQVDAHNIVPCWHASPKLEYGARTIRPKI
HKVLTEFLTEFPPVIKHSHVSGEKTKTTDWDAVDTFIEVDRSVGEVDWAKPGTAEGLFMLESFCKDRLKYFHS
SRNDPTKRALSNLSPWFHTGQISPQRAILRVRDFRSKFRESVESFIEECIIRRELSDNFCYYNNEKYDSIEGT
NEWARKTLNDHAKDKREYLYARGKLEKAQTHDDLWNAAQRQLVREGKLHGFLRMYWAKKILEWTDSPETALSD
AIYFNDKYALDGIDPNGYVGCMWSVCGIHDQGWAERPVFGKIRYMNYKGCQRKFAVAEFVKRYKP
>Nematostella_vec_XP_032226079.1_cry-1_X1
MSGMDKFTTDSCNLVKAKKTSHSIHWLRKGLRIHDNPALRDAVLNWGTFRVVYILDTKSVASSNIGLNLWRFL
LQALEDLDDSLRKLNSRLFVIRGQPADVFPRLFREWGITRLTFEEDSEPFGKERDSAICMLAREAGVEVASHR
SHTLYHLQGIIDRNGGTPPLTYKKFLSVIEGIAPPDPPVPHIDPSAQELGHTPLSDNHDELYGVPTMEELGLE
TSKLFVEVWHGGETEALKRLDRHLERKAWIASFGKPKVTSDSLMASPSGVSPYLRFGCLSPRLFYYRLMDLYR
KVKGGPPPTSLYGQLLWREFFFVVSTNNPNFDKMESNPICLYIKWRTDKQVASDLQKWTDAQTGFPWIDAIMS
QLKQEGWIHYLARVAIASFLTRGCLWISWEAGMKVFAEHLLDADWSSNAGNWMWWSYSAFSQQFFNPPCPVEF
SKSLDASGDYVRKYLPVLKGFPARYIHEPWKAPLNVQKMAKCIIGKSYPSPMVDHEKAVSKNMEEMTSVYKSV
IQYRISVLSSEEKQIEETKSQFQMPETSMH
>Nematostella_vec_XP_032235657.1_cry-2
MVNSVHWFRKGLRLHDNEPLRRAIEGSDTFRGIFFLDKAAVKNAKVSPNGWRFLIESLRDLDQSLQKYNSRLF
IIQGQPIDVFPKLIKQWNISKLTFEYDSEPFPRQRDLAVKRIAEKAGVDVIVCSSHTLYDIEWMIGKNDGLPP
ITFKQFQKLLQGSGLPSHCVKTVDENLFGDCTTPLDNEHERLFGIPSLGDLGLSFTPEESIWKGGENAALKRL
ETVEKELLEGVSDKESYAPFPSSTKLSPYLRLGCLSARRVFHTASAINAKANNSSLAHGIQTSLLWRDFYFVL
ASRNQALDKMHDNNMCMQFPWVEREDWLDQWRHGKTGFPWIDAIIRQLRQEGWIHHLARQSIGCFLTRGCLWI
TWEEGFKMFDEFQLDAEWSLNACSWLSLSCSAFFNGTPSQWYCPVGIGKSLDPDGEYIRKYVPELKKMPTEYV
FEPWLAPLEVQETAGCIVGADYPNPLVDADVARKECIIRMREVYSGLVTKLSSAPGLGNNVIHWFRKDLRLHD
NPALLDGLKNCRAFYGVYILDPNNIKAAIVSANRWNFLLQCLADLDNNLKKCGTRLYVFRGQPTTVFPQMFNE

WNITKLTFESECEPFGQRRDVAIQSLAEEFGVKVSCKPSHSLYSIEQILEMNEGKPPVLFKDFEAIVRKLGPP
ENPVESITRKSFMNCICPITSTHSEKFGVPSLAELGIKPADVTCGDMWVGGEREALNRLGILEDKILGSNSSK
NCDGSSVLFPSRTQLSPYLRFGCLSPKLYYKKLTGAYVKMQFSPPPMSLYRQLIWREFFFTLASKNPNMDQVN
DNPLCIQISWEENKDHFLCWKQGNTGFPWIDAIMRQLQKEGWIHHLARLSVGCFLTRGCLWISWEEGLRAFEE
LQLDAEWSMNSGSWLWLSCSAFFHGQIPWFCPVEVGKKLDPSGSYIRKYVPELRSMPLKYLYEPWTAPDDIQR
AAMCVIGEDYPHPIVDHIEQRKACLQRLKTVCRHIQTTSFA
>Nematostella_vec_XP_032236034.1_photolyase
MLQLIWFRTDLRVQDNSALAAAMANGPTVALFLISPGQWLAHDDAPSKVDFWLRNLKELATELGKLNVPLLVR
EADDWSAAPQVIAELCQQLQISEVQVNEEYGVHESRRDQAVAEALDDLGVNFRSHLDQLFFKPGSVLTKSGSY
FQVYSQFRKVCYQRLHSALPTLIATPKAQAPLAIQSDAIPEHVAGFALPPAALRELWPAGEAEAARRLDDFAT
QQIDFYQSARDFPAQPGTSQLSAYLAAGVISPRQCLHAALQANQGEFDSGNVGVITWINELLWREFYKHILVG
YPRVSMHRAFRAETEAVAWRDAPLELKAWQEGRTGFPSSMQPCASCWPPAGCTTACA
>Nematostella_vec_XP_032241878.1_cry-1
MVLAMASKKTHQTVHWFRNGLRLHDNPALKEAFETSQTVRPLYVLDPDVLKNGNIGVVRWRFILESLADLDNN
LKKLNSRLFVVRGRPSEVFPKLFKEWKISKLTFEVDTTEPARKQDAEVLKIANKLGVDIEQRVSHTLYDLDRI
VNKNGGTAPLTYKKFQSIVSSLGPPAAPLPAIDKKMVAGCNTPTTANHDKIYGVPTLEEIGQEVPEESREVLY
PGGETEALERLEVYMKKEDWVCKFEKPNTAPNSIEPSTTVLSPYMTYGCLSARLFYTRLAEIYAKKKKHSQPP
VSLHGQLLWREFFTTAAYKTPNFNKMVGNSLCLQVPWDQNDEYLAAWAEARTGYPYIDAIMTQLRREGWVHHL
ARHSVACFLTRGDLWISWEEGLKVFERLLIDHDWNLNAGNWMWLSASSFFHAYFRVYSPVAFGKKTDPNGDYI
RKYIPKLSRYPPKYIYEPWNAPLAVQEKAGCVIGRDYPCPIVDHNKVVTRNMSRMKEARSLKYGKTDMGVDKQ
ASTPSKRKSKTTTDKPAKKTKKITDFLADSSE
>Neodiprion_lec_XP_015515077.1_cry-1_X1
MTDGEKAQHGGLKPFRRDGKKHTVHWFRKGLRLHDNPALREGLTGATTFRCVFVLDPWFAGSTNVGINKWRFL
LQCLEDLDRSLWKLNSRLFVIRGQPADVLPKLFKEWGTTDLTFEEDPEPFGRVLNQNITTLCEEMGISVVQRV
SHTLYKLDSIIEKNGGKAPLTYHQFQNVAAAMDPPPPPEALVTSEFTTGAYTPIMVNHNDVYGVPTLEELGFE
TENLLPPVWIGGETEALVRLEYHLARRYWVASFGRPKMTPQSLLASQTGLSPYLRFGCLSTRLFFYQLTDLYK
KIKKAVPPLSLHGQLLWREFFYCAATRNPSFDRMQGNPICVQIPWDKNAEALAKWANGQTGFPWIDAIMTQLR
EEGWIHHLTRHAVACFLTRGDLWLSWEEGMKVFDELLLDADWSVNAGMWMWLSCSSFFQQFFHCYCPVRFGRK
ADPNGDYIRRYVPALKTFPAKYIHEPWNAPQSIQQTAKCIIGKDYPLPMVDHGKSSRINIERMKQVYQQLNKY
RGNGLPGTRGETIEGLLNCTLATATLPQSEEDEKHKHQTTSNPSLSPARHQKTICSILSMVTLRQLDQTAEFP
SSEMD
>Neodiprion_lec_XP_015522462.1_photolyase
MDQFVAKRAKLSNFVNKLEEDRKNDSESVMKFKFNKKRVRVLTAIDKVKENCRGIVYWMFRDSRVQDNWAFLF
AQKTAIKNRLPLHVCFCVLPKFLGATIRHYKFHLAALKQVELECKNLNVNFHLLRGEPNDVILKFVEKYDMGA
VITDFFPLRLPLSWVDDLKKKLPEDIPLCQVDAHNIVPCWEASDKLEYSARTIRNKINSKLDEYLTQFPPVIK
HPYTTKQKFTENDWENALKDVEIDKSVKEITWAKPGYEEGILELERFIEKRLKLYDSKRNDPTLNALSNLSPW
FHFGMISVQRCILEVSKYKNSHKKSVESFMEEAIVRRELSDNFCFYNEHYDSLKGAKQWAVDTLDNHRKDKRE
YVYTLKEFENSLTHDDLWNSAQNQLLQDGKIHGFLRMYWAKKILEWTRTPEEALEWSIYLNDKYSMDGRDPSG
YVGCMWSICGIHDQGWAERSIFGKIRFMNYKGCQRKFDVKAFVARWGGKVHTKKK
>Neoseiulus_bar_AVI26872.1_DNA_photol
MRRSTRRARNVKPDLGRIKYESSDSELDSKEDVLDSKDFKVEELEVDSKNLKGDLKNVKADPDSNLSFADRVA
KSRHDTGASVSDFDFNEGRVRFLTEAQEVAKDSTAILYWMQRDQRVFDNWSLLYAQKLAIKKKLPMIVVFCFH
DRCDGPNATLRTFNFMVSGLEEVQSDCRSKNINFDVLKGLPGDVIPRYVVNQRIGAVVTDFYPIRESTAWVKA
VKDKLPPDVPLAEVDAHNIVPAWILHEKQVYQARQLRTKFAEHFPEFLTNFPDVMYHPHSFLAKTEKVDWKYL
MQDKKIDRSVDDVPGVHGGGKAALAALELFIKNSLKDYEDYKNDPARDVSPYLSHYFHFGHLSSQRAFFEVQK
FVHNRRYEASVSGFYEEAVVRKEMSDNYCLYNDKYDCLEGAYEWAISSLDAHRRDKRDYLYSEEDIESATTHD
DLFNAATKQLKTEGRMHRYMKMYWPKKILEWTPSPEKAIAISNRLLNKYAYDGRDPDSYLAVMWAIGGLHDRP
WAGRRVIGKIRCMMLEGCEKKFDVAAYVDRYSGV
>Neospora_can_XP_003881384.1_NCLIV_0441
MPHPTQTSPPHKAASPQTKGLKRSASAESEHKPEKKTKKAATLPETSRMHRGEQEWIKNGCVDERRVRLVTEG
IREPKADGKVVICYLQRDLRVQENWALLLAQEAARALNKPLVVIHLLVPGLAFQPTRRHLSFFIGGAREVEAE
LKSLNIGFELPIVAKKDPKGRLDEANKKIEEVFAALQPALAVCDFNPLRLPTQIVEALARVYGKGLSPLYQVD
THAVVPCWVASNKAETSARTMRPKLQAMLKEFAVPFPKVEPHPVPWTSKQLFPLDEDRVLSAVKPDPPEPLDS
WKPGTKAALRLLQSFATPQNIAKYGKARNDPLADCQSDLSPYIHFGHIAVQRCLMEVSKLKDTTGDRAVHEGV
RAFIDEVVVRSQLSDNFVFFNPHYDDIKGAPDWAKQTLEKHAKDKREPQYEFTALEEGKTYDSLWNAAQLQLV
RDGKMHGYLRMYWAKKLLEWTKSPQEALKTAIALNDKYHLDGTDPNGVTGCMWSIGGVHDQGFKERPVFGKIR
YMNYPGCERKFDVKAFVRKFPGAAENAVNAAKQGLIPFSGKMKKELQEIPKGRDSEQATATMMKKIEQHKAQN
TNAGPSSSKETKTDEPKKRQRKGSVDERAPAKSGAAKEEAEVEAEEEAGSDE
>Nephila_pili__GFT22944.1

MSKSQGNSIHWFRKCLRLHDNPALVEAVKKGGHFWPIFILDPWFVKNMRVGPNRWRFLMQSLNDLDESLKQFN
SRLFVIRGKPTEVFPDIVKKWKIELLTFESDTEPYAKARDEEIENLMKTLNVEIKKFCTNTLYNSEQIISYNG
KVPLTYQAFANTADKIGLPSKPVSSIKLLKELIAEKNLKTPIENDYEIKYRVPTLKELNVDESELNPCLYPGG
ETEALKRLELVTSKEDYICKFEKPNTSPNSLQPSTTVLSPYMKFGCLSARTFYYKVREVYLKSKKGYTKPPVS
LHGQLFWREFFYTVGSATPKFDQMVGNPVCTQIPWIENDTFLQAWANARTGFPFIDAIMTQLRKEGWVHHLAR
HAVACFLTRGDLWVSWEYGMKVFEELLLDADWSLNAGNWMWLSCSAFFHQYYRVYSPIAFGKKTDKNGDYIRK
YIPVLKRFPPEYIYEPWKAPKKLQEQLGCVIGKDYPSPIVDHDKARTENLRRMDAVYKAKKENKSNEKTSSTK
SKKSPNKQGKRSIDEDKEVKISKFFKNK
>Nephila_pili__GFT64976.1
MSSGKQKTLLDYENPAKKQKTDEVENDSQGFIKEIKQSRLSCATSIKDFKFNKKRVRVLSDAKDIAENAQSIV
YWMSRDQRVQDNWAFLYAQHLALKLDLPLCVCFCLVPKFLDATIRHYRFMLKGLEEVSEECESLNIHFHLLFG
EAKKVLPDFIKENNVGGIVTDFSPLRVPAKWVSDVLTKLPDDVPLCQVDAHNIVPCWIASDKLEYGARTIRKK
IHDKLKDFLTDFPPIIKQRETAKCKFQSIDWNKIENKLEVNMDVKEVAWAKPGTTAGMLQISEFCKKRLKDFG
NLRNDPTKSNLSNLSPWFHFGQISVQRAILTVLKYKGKYGSSVDAFVEEGVIRRELADNFCYYNKKYDKIEGA
YDWAKKTLKDHSKDKREYIYTKEEFENAKTHDLLWNAAQRQMVREGKMHGFLRMYWAKKILEWTNSPEEALEI
AIYLNDKYELDGRDPNGYVGCMWSICGIHDQGWAERAVFGKIRFMNYKGCQRKFDVNEFIRRYREK
>Nephila_pili__GFT83862.1
MTSHKQKNVVHWFRKGLRLHDQPALREGLSGATTLRCVFIIDPWFAGSSNVGINKWRFLLQCLENLDSNLRKL
NSRLFVIRGQPADVFPRLFKLWKTTHLTFEKDPEPYGRIRDHNTTTMAQENGVTVISRTSHTLYRLEKIVQKN
GNKSPLTYKQFQNILASMEPPPAPQPRITLEDMGSCYTPLSEDHDDKYGVPTLEELGFDTENLKPPVWVGGET
EALARLERHLERKAWVASFGRPKMTPQSLLPSQTGLSPYLRFGCLSARLFYQELTELYRKIKKVNPPLSLHGQ
ILWREFFYCAATNNPKFDHMIGNSMCVQIPWDVNSEALAKWTNGQTGFPWIDAIMTQLREEGWIHHVARHAVA
CFLTRGDLWISWEEGMKVFDELLLDADWSVNAGSWMWLSCSSFFQQFFHLYCPVRFGRKADPNGDYIRRYLPV
LKNFPTKYIHEPWIAPEKVQIAAKCIIGKDYPMPMVNHQEVSHINLERMKQVYQQLSHYRGANMYNNSYNQVN
QRESDDGYPKSAKRRAVEYPDESGQVHKRDISLK
>Nephila_pili__GFU12876.1
MVEHLSTKMSAESTKMGSFIHWFRNDLRLYDMPSINAALEKCTTFYPVYIFDENDIKEKQITYNKVRFILDAL
KDINGKLQPNGGRVYVFKGNPTDIFSDLFKKWNISYLTYEVDVQPAWKVLEKSVQQICQQHSIEIIKDVSHTL
WNLEDILKANRGQPPVTYEVFCQVVEALGPPSRPESYVNLEKVLMPKDDSFPIDLPNNPDDLGVFMECIQQSN
RIWIGGETHALKHLDARLKVEEEALESGIFMPNQAKPDLLGPSMSLSAALSLGCLSVRKFYWSLQDLHERVNS
DICVPDSLIGQLLWREYFYAMCMNNPFYATMVENPICLNIPWTTNEQHLQSWINGKTGYPFIDAAMRQMVQEG
WLHHVARNAVACFLTRGDLWISWEEGFKVFMKYLIDADYPVCAGNWMWVSSSAFENILQCPTCISPISYGRCF
EPTGDYIRKYVPELKNMPNEYIYEPWLAPLPEQEKAGCIIGKDYPQPIIDHQEAANTNKKRMDIIKESFYTKM
VPPHCSPSNPLETRVFMWLPENCIENLMHHRN
>Nestor_nota__KFQ46407.1
RFLLQCLEDLDANLRKLNSRLFVIRGQPADVFPRLFKEWNIAKLSIEYDSEPFGKERDAAIKKLASEAGVEVI
VRISHTLYDLDKIIELNGGQPPLTYKRFQTLISRMEPLEMPVETITPEVMEKCTTPVSDDHDEKYGVPSLEEL
GFDTDGLPSAVWPGGETEALTRLERHLERKAWVANFERPRMNANSLLASPTGLSPYLRFGCLSCRLFYFKLTD
LYKKVKKNSSPPLSLYGQLLWREFFYTAATNNPRFDKMEGNPICVQIPWDKNPEALAKWAEGRTGFPWIDAIM
TQLRQEGWIHHLARHAVACFLTRGDLWISWEEGMKVFEELLLDADWSVNAGSWMWLSCSSFFQQFFHCYCPVG
FGRRTDPNGDYIRRYLPVLRGFPAKYIYDPWNAPESIQKAAKCIIGVNYPKPMVNHAEASRLNIERMKQIYQQ
LSRYRGLGLLATVPSNPNGNGNGGLMGYSPGESISGCSSTGGAQLGTGDGHTVVQPCALGDAHTGASGIQQQG
YCQASSILHYAHGDNQQSHLLQAGRTALGTGISAGKRPNPEEETQSVGPKVQRQSTN
>Nestor_nota__KFQ48597.1
YFVSRIIELNGHKPPLTYKRFQAIISRMELPKKPVSTVISQQMETCKVDIQENHDDVYGVPSLEELGFPTDGL
APAVWQGGETEALARLDKHLERKAWVANYERPRMNANSLLASPTGLSPYLRFGCLSCRLFYYRLWELYKKVKR
NSTPPLSLYGQLLWREFFYTAATKFDRMEGNPICIQIPWDRNPEALAKWAEGKTGFPWIDAIMTQLRQEGWIH
HLARHAVACFLTRGDLWISWESGVRVFDELLLDADFSVNAGSWMWLSCSAFFQQFFHCYCPVGFGRRTDPSGD
YVKRYLPKLKGFPSRYIYEPWNAPESVQKAAKCIIGVDYPKPMVNHAETSRLNIERMKQIYQQLSRYRGLCLL
ASVPSCVEDLSGPVTESASGQGCSTSTAVRLSQTDQASPKRKHEGAEELCTEELFKRAKVTGLPAPEVPGKS
>Nestor_nota__XP_010013708.1
MVEGGRNRFLIPAGREGRHRRALPPFPEGRCFAGGCAGDIGAAPVPGKGGGRYQMMESRPRPHGTTLLSLSAR
VVLRMRRGKGKRKAEATKVPRVSRRRTEEEETELKEEVRCTLYWMSRAQRLQDNWAFLYAQRLALKQELPLHV
CFCLVPKFLEATIRHYGFMLRGLQEVAEECAELNIAFHLLRGYAKDVLPAFVVEHGVGGVVTDFSPLRLPRQW
VQDVRERLPEDVPFAQVDAHNIVPCWVASTKQEYSAWNIRGKIHAQLPEFLTEFPPVVRHPYPPSCPAEPIAW
EACYSSLEVDRTVKEVEWATPGTAAGLAVLQSFIAERLKSFVFHRNDPNKAALSNLSPWFHFGQVSTQRAILE
VQKHRHKYKESVDAFVEEAVVRRELAENFCYYNENYDSVQGAYAWAQTTLKLHAKDKRPFLYKLQELEQGTTH
DPLWNAAQLQMVQEGKMHGFLRMYWAKKILEWTHSPEEALQFAIYLNDRYELDGRDPNGYVGCLWSICGIHDH
GWTERAVFGKIRYMNYAGCKRKFDVRQFEHRYAPCALSQ
>Nestor_nota__XP_010017836.1

MLHRTIHLFRKGLRLHDHPVLLAALESSEALYPIYILDREFLTSVMHMGALRWHFLLQSLQDLRESLCQLGSS
LLVIQGEYEAVLRDHVQKWNITQVTLDAEVEPFYQEMEATIRRLGGELGFEVLSLGGHSLYDTKRILDLNGGT
PPLTYKRFLHVLSLVGDPEVPVRNLTAEDFQRCAPPDPGLAERYSVPLPGDLKIPQESFSPWKGGETEGLQRL
EQHLTDQGWVSSFAKPRTSPNSLLPSTTGLSPYLSTGCVSVRTFFYRLSNIYAQAKHHSLPPVSLQGQLLWRE
FFYTGASXXXXXXXXXXMAGNPICLQIRWHEDAEGLHRWKTAQTGFPWIDAIMTQLRQEGWVHHLARHAVACF
LTRGDLWISWEEGMKVFEELLLDADYSSNAGNWMWLSASAFFHRYTRIFCPVRFGKRTDPEGQYIRKYLPVLK
NFPSKYIYEPWTASAEEQKQAGCIIGQDYPFPMVNHKEASDHNLQLMKQVREEQCRTAQLTRGDTEDPMATKV
KRDHSEEHVSKGKVARTTEQTHLPAEFTCLEPQNGKRGEAEAS
>Nestor_nota__XP_010022925.1
VLHWAQSNADFVVPLYCFDPRHYLRTHCYGFPQTGAHLHCYGFPHKGLHRLRFLLESVKDLRETLKRKGSTLV
VRKGKPEDVVRDLIAQLGSVSAVVFHEEATQEELDVEKELRQVCSQHGVKIHTFWASTLYHRDDLPFRPIARL
PDVYTHFRKAVESEAKVRPTLRMADQLQPLAPGVEEGHIPTMEDLGQKDPETDPRTAFPGSGGETQALMRLQY
YFWDTNLVASYKEMRNGLVGMDYSTKFAPWLALGCISPRYIYEQIQKYEKERTANQSTYWVLFELLWRDYFRF
VALKYGRRIFSLRGLQSKEVAWKKDLQLFDCWKEGKTGVPFVDANMRELAATGFMSNRGRQNVASFLTKDLGL
DWRMGAEWFEYLLVDYDVCSNYGNWLYSAGVGNDPRDNRKFNMVKQGLDYDSNGDYVRLWVPELQGIKGADIH
TPWALSSAALSQAGVALGETYPQPVVTAPEWSRYINQRPVSTGKGRSPHARGRRGPARPPVQHKDRGIDFYFS
RRKDAR
>Neurospora_cra_XP_964834.2_photolyase
MAPSKRKASAPPQTSHVNGNPSADKKRKTTTDAPPTNPNTSSDPLRAPHPFYKDSETHGIVLRKFYPHEMSNA
RAQAYNDNELPRPIETLSAALAETAALRKSLPVRQAVVHWFKMDLRLHDNRSLWLASQKAKEAGVPLICLYVL
SPEDLEAHLRAPIRVDFMLRTLEVLKTDLEDLGIPLWVETVEKRKEVPTKIKELMKSWGASHLFCAMEYEVDE
LRREAKLVKLLAEGEKGEKMAADVVHDTCVVMPGALQSGSGGQYAVYSPWFRAWIKHIEENPECLEIYEKPGP
NPPGTKEKHENLFACSIPEAPEGKRLRDDEKARYHSLWPAGEHEALKRLEKFCDEAIGKYAERRNIPAMQGTS
NLSVHFASGTLSARTAIRTARDRNNTKKLNGGNEGIQRWISEVAWRDFYKHVLVHWPYVCMNKPFKPTYSNIE
WSYNVDHFHAWTQGRTGFPIIDAAMRQVLSTGYMHNRLRMIVASFLAKDLLVDWRMGERYFMEHLIDGDFASN
NGGWGFAASVGVDPQPYFRVFNPLLQSEKFDPDGDYIRKWVEELRDLPELKGGKGGEIHDPYGRGSEKVKKKL
EEKGYPRPIVEHSGARDRALDAYKRGLARDL
>Neurospora_cra_XP_965722.3_cry_DASH
MAPSKVVIYAMRRELRLSDNPIFHHLSNPESKHGFSHLLPVYVFPAQQIDLSGFVPKGSENPHPAPKSAVGGY
ARCGPYRAKFLAESVWDLKTSLQSIGSDLLVRAGPYKDVIQSLVEGLKAKECQVGAVWMTSHEGSEEKSEEKT
VASFCAKSGIDFKLWDDEKYLIHDRDTGITHLNDLPDVFTTYRKQIEPLREKARKTLPVPEKGALPAYPDIDM
IPSQQPPFNIPGTCEELVDAVVRPVKNFLKDLPDFPEKAESSHPFRGGETSAHKRIDHLVLSGGMKSYKDSRN
GLLGPDFSTKLSAYLAQGCVTARQIHHALVAYEDGTGTKYKGADGFGEGDNQGTETVRMELLWRDYMRLCHQK
YGDKLFRVEGFNGKHTDYEGEDKKYGWRTANTSIALPGQEPTPEKVSEILARFNAGTTGMGLIDASQRELIHT
GYTSNRTRQNVASFLAKHLEIDWRYGAEWYEMLLVDYDVSSNWANWQYVAGVGNDPRGAARIFNPVKQAFDYD
KDGTYVRTWVPEVAKFENLENVFQAWTASKEDLKTAGLEGNIMVTDPVKPIKFNLDHKPSKVKKRPFFRKRGT
KTRDAQGSAESPGSSDSHSGSGGSPDGSGGGNIPSESNCAAAGSGQAQQTHQGSGRSQSSSNHGGRSHSHQHN
QQNYHHSHRGNDYTRGGGGGRGGRGGRGGGGGGYSASQGYYGIGGGYRGGGRGRGGGGGFRGRYAPTGGLGGH
HHSEQQVASQFQTDA
>Nibea_albi__KAG8012485.1
MVVNSVHWFRKGLRLHDNPALQEALNGADTVRCVYILDPWFAGAANVGINRWRFLLEALEDLDSSLKKLNSRL
FVVRGQPTDVFPRLLKEWKVTRLTFEYDPEPYGKERDGAIIKMAQEFGVETIVRNSHTLYNLDRVIEMNNNSP
PLTFKRFQTIVSRLELPRRPLPPVTQQQMDKCRTKIADNHDQLYSIPSLEELGMHYRCSPPLSLFGQLLWREF
FYTAATNNPNFDRMEGNPICVQIPWDQNPEALAKWAEGRTGFPWIDAIMTQLRQEGWIHHLARHAVACFLTRG
DLILCLLLQVFEELLLDADWSVNAGSWMWLSCSAFFQQFFHCYCPVGFGRRTDPSGDYIRRYIPILKDYPNRY
IYEPWNAPESVQKAANCVVGVDYPKPMINHAEGSRLNIERMKQVYQQLSHYRGL
>Nibea_albi__KAG8012608.1
MSPSAAAGAEDSKALVRRVLREVLVGREDPEGFFVMCLSALGHQETRSQFVSLIQPLSTANSSLHSVLTSIYT
EYFLRSTEDDELELAMALSLLEMKDHCMSTPSQESRCRQTGVDRRKNIGLPQGEPCVKVSPPQTARETELEKS
THAYKYNTETPGTSQTVCVSTLSISKQDKLEQGYQKMDERDLNQSEKPKRSKNRRQRRKGPCQQIVGLPCSPS
ALPPVLLWFRRDLRLCDNPALICSLEFGAPVIPVFIWSPEEEEGPGITVAMGGACKYWLHQALSCFCSSLERI
GSHLVFLKANRQGNKFGSSLSTLKELIKETGARTVLANALYEPWLKERDDAVVSSLQKDGVEFRMFHSYCLRD
PYSVSTEGVGLRGIGSVSHFMSCCRQNPGSALGVPLDPPVSLPTPVHWPQGVPLDTLGLARMPCRKDGTTIDW
AANIRKSWDFSEDGARARLEAFLHDGVYRYDKESGRADAPNTSCLSPYIHFGQLSPRWLLWDAKGARCRPPKF
QRKLAWRDLAYWQLTLFPDLPWDSIRPPYKALRWSTDRGHLKAWQQGQTGYPLVDAAMRQLWLTGWMNNYMRH
VVASFLIAYLYLPWQEGYRWFQDTLVDADVAIDAMMWQNGGMCGLDHWNFVMHPVDAAMTCDPYGSYVRKWCP
ELASLPDELIHKPWKCPASMLRRAGVVFGQTYPERIVADLEERRSQSLQDVALVRKEFRQYVDKRSGCDLVPL
PPRLVSEALGLSHRDGDVVTAGKQFLLPVITRMEFKHQLEDPEADAASNPYNCCSERICEPQEG
>Nibea_albi__KAG8013990.1

MSDKKRKPASSSAAAAAKGSSAKQPKLTPAKEKAGGWLQDLMKQQRTEKKGMKVNKKRLRVLSDTGKIKQGSE
GVLYWMSRDHRVQDNWALIHAQQLAVEENLPLHVCVCLFVPKSELSTLRHYSFMLKGLEEVAKECKALDIQFH
LLHGAAGDVLPGFVSDRSLGAVVTDFSPLRERLQWLEDVKKKLPKDVPIIQVDAHNIVPCWEASPKLEYAART
IRGKITKLLPEFLTDFPLVEKHPYTATRTAKPVDWEKNQASLDVDRTVGEPEWAKPGTKAGVEMLESFIDVRL
KLFETQRNDPNAAALSQLSPWIRFGHLSAQRVALQVQRSGKNSGQSVSSFIEELVVRRELTDNFCFYNKNYDS
VEGAYEWAQKTLKDHAKDKRPYIYTREQLEKAKTHDKLWNAAQYQMVTEGKMHGFIRMYWAKKILEWTSSPEE
ALAIALYLNDRYSLDGQDPNGFVGCMWSICGIHDQGWAERPVFGKIRYMNYKGCLRKFDVARFEQKYCPKNL
>Nitrosopumilus_sp._RMW33708.1_photolyase
MAQYQKSLFIFRRDLRLADNTGLIAASQSSSEVIPIFIVDSAIMNSKERFSEFRLQFLKDCLLDLDSQLKEKK
SYLHIFVGNSEKIISKIIKASKIDAIFVNTDYTPFSKNRDKRIEKICKESKVDFISADDILLHNVELLKTGNG
EPYKVFSAFYSKAREFQYRKPQNHKFENLSKTKISSETSLEKIPEFNIKNNSKIMKGGRKAGLELLKNLKNLK
NYNKERNIPSINGTSHLSAHNKFGTCSIREIHQECIKNLGQSHTIISELHWRDFFTYIMYHSPYSYSKEFNKR
FQKIPWSKNQKNFSKWCQGMTGFPIVDAGMRELNNSGFMHNRVRMIVASFLTKDLHIDWRLGEKYFATKLIDY
DACVNIGNWQWSASTGCDAQPWFRIFNPWLQQKRFDPECTYIKKWVPELREMDAKVIHDLWKKHPEDSSYVKP
ILDHKTESQNAKEIFSSQ
>Nitrospira_len_WP_121988866.1_DNA_photol
MRGLVWFRRDLRLHDQPALTAACQECDEVLPLFVFDEPLLQSREFGSACVNFMLGCLHELSTSLAGRGLVLQW
RRGEPVEAVVQAARESKAEVVYWNRDYEPAAIERDRRVQLALAEQGIRTQTFTDHVVFETEEIRGATGDPLQR
YSAYRARWWAKWQAAKPSPLASPKPKASTVVPSRSAPRPFPSPVELGYDRVVPWIDPGEREAQRRLQEFLGNA
IHTYGDGRNRPGVDGSSRLSPHFRFGTLSVRTAVHEALAALAQGGRVSRADVLIWIDELVWREFFQQVLAAFP
WVVAGPFRAVAVPPVREPGLERDRLFQAWCQGRTGFPIVDAGMRQLHQTGWMHNRVRMIVASFLIKDLRIDWQ
SGERYFMQQLLDADVGANNGNWQWCAGTGTDAMPGYRIFNPALQSKKFDREGLYIRAYVPELAKVTGKWIHEP
HLMTPDEQDRAGCRIGVDYPAPLVDHAQARREYLDLGKQQVMR
>Nosema_bom_EOB11318.1_photolyase
MDKRLKILNGNPKTGSTLYVMRRDQRIYENYAIEYGYSLSYEKKSEFIIGIDFNTIIVNEFQKMIMFEGLEEL
SKDCLKFNLPLFSFDNLKFLIDKMKIKNIILDYSPLRGSLKYDEDVTKICKDLGIYCVQVDAHNVVPHNLLET
YKRTGKSVKNDLFKHFFDFLKEFKSLEKHKFNKSKGDFKLPSFTKHDYFIGGYKAGMVQVQYFFQNKFKRYAD
KRNSPDDNVLSDLSPYLHFGQISSQKVLLDAYDLFYNKDDKNLESFVNEIFVWKETSEHFCFHEKNYDNLEGA
LPWAKESLLKHADDKREKIYTKGILEEGKTKSELWNAAQTELREFNKIHGYVRMFWAKQMLKWTKSPEEALSL
AIWLNDKYSLDGNDPNGYTGIMWSICGTMDQGWKERDVIGKIRAMNEIKTKKYTKMWTNKKEKEAYLKQRKVK
RDKK
>Nothobranchius_furz__XP_015808759.1
MAHICIHWFRKGLRLHDNPALVAALRDSKELYPVFILDPYLHNSHVGINRWRFLIGALEDLDRSLRRLNSRLF
VLKGKPEDVFPKLFTKWKVTKLTYEYDTEPYSLRRDKAVAALAREHRVSVIYQISHTLYDIDRIIEENGGKPP
LTYNRLRELARKLGPPKKPIPAPTLEDMKDVTTPCSEKDQKKYGIPTLEELSLDTTSLGEELFPGGEQEALRR
LDEHMKRTGWVCSFEKPLTSPNSLSPSTTVLSPYVTFGCLSARTFWWRLADVYQGKKHSDPPVSLHGQLLWRE
FFYTASVGIPNFNKMEGNPVCTQVDWDTNPEYLAAWREARTGFPFIDAIMTQLRTEGWMHHLARHAVACFLTR
GDLWISWEEGQKVFEALLLDGDWALNAGNWQWLSASTFFHQFFRVYSPVAFGKKTDKNGDYIKKYLPVLKKFP
AEYIYEPWKAPRCVQQAAGCIVGKDYPRPLVKHEEVSKRNIQRMKRAYAQRSSNVPESPAKKQGVKRKNPSVL
EMLQKKIRKE
>Nothobranchius_furz__XP_015810621.1
MSASRTIVCLLRNDLRVHDNELFHWAQKNAEHIVPLYCFDPRHYVGTHNFNFPKTGPFRLRFLLESIRDLRNT
LLSKGSNLVVRRGKPEEVVADLIKQIGSVSSVAFHEEVTSEELGVERKLKEVCAQLKVKVHTFWGSTLYHRDD
LPFHHMSRLPDVYTQFRKEVESQSRVRPVFPTPERLNPLPPGVEGGDVPTAEDLQQTEPLTDPRSAFPCSGGE
SHALARLKHYFWDTDKVASYKETRNGLIGVDFSTKLSPWLANGCISPRYIYHQIKRYEEERTANQSTYWVIFE
LLWRDYFRFVGVKYGNKIFLIKGLQDKPVPWKKDTKLFDAWKEGRTGVPFVDANMRELALTGFMSNRGRQNVA
SFLTKDLGLDWRLGAEWFEYLLIDHDVCSNYGNWLYSAGIGNDPRENRKFNMIKQALDYDSNGDYVRQWVPEL
NAVRGADVHTPWNLSSAALSNAHVSLGETYPTPIVIAPEWSRHFNKKPSNSGPSPRGKRGPSHTPKQHRDRGI
DFYFSRSKNL
>Nothobranchius_furz__XP_015820104.1
MPSVADDLALVRHMLREVLVGREDPEGYFAMCLSILGHQETLSQFASIIKPLSTANIHLHSILTSIYQEYFSK
TEDDEMELALALSLLQMKDQQMATSNNEPTPLQFGVTDCPSCFHQPDVVSNHQGKNCLEVTPGREANSYSVLT
GTEEGQPHINQKTGQHKNTHINSDAKQTPETGQSPQISGSSASFSVPGMSHEGDHITEDLNTSDKPKRSRNRR
QRRKGTHQQLVGLPCSPSTPPPVVLWFRRDLRLCDNPALIRSLELGAPVIPVFIWSPKEEEGPGVTVAMGGAS
KYWLHQALSCLCASLEKIGSHLIFLRASGEGNEVGSSLHVLKQLVKTTGAQTVMANALFEPWLKERDDAAVAA
LQKGGVVCRMLQSYCLRDPYSVSTEGVGLRGIGSVSHFMSCCRQNPGSPIGVPLDPPVSLPTPAHWPQGVPLD
ALGLARMPRRKDGTTVDWAANIRKSWDFSENGAHARLDSFLNDGVYRYEQESGRADASNTSGLSPYLHFGQLS
PRWLLWDAKGACCRSPKFQRKLAWRDMAYWQLTLFPDLPWESLRPPYKALRWSTDRGHLKAWQQGKTGYPLVD
AAMRQLWQTGWMNNYMRHVVASFLIAYLHLPWQEGYRWFQDTLVDADVAIDAMMWQNGGMCGLDHWNFVMHPI
DAAMTCDPNGSYVRKWCPELSDLPDELIHKPWKCPASMLRRAGIVFGQTYAERVITDLEERRTRSLQDVALVR

REFGQYVDKRSGCDLLPLPPRLVSEALGRSHSDEAVVRGGRQFLLPLITRMEFKYQQEEPEADAASNPYNATL
KGYVSRRRDETVAFLNERDFKTSVMYESAQRQGRLERDYRRIEGLPSSPSPRGRARRTPTAKDRFSIVPGGAV
TLLK
>Nothobranchius_furz__XP_015820664.1
MVVNSVHWFRKGLRLHDNPALQEALNGADTVRCVYILDPWFAGAANVGINRWRFLLEALEDLDSSLKKLNSRL
FVVRGQPTDVFPRLFKEWNVTRLTLEYDPEPYGKERDGAIIKMAQEFGVETAVRNSHTLYNLDRIIEMNNNSP
PLTFKRFQTIVSRLELPRRPLAPITQQQMNRCPTQIPDNHDQLYSIPSLEELGFRTEGLPPAVWRGGESEALE
RLSRHLDKKVWVASTRVKTCSLYASPTGLSPYLRFGCLSCRVLYYNLREIYMKLCKRSTPPLSLYGQLLWREF
FYTAATNNPNFDRMEGNPICVQIPWDQNPEALAKWAEGRTGFPWIDAIMTQLRQEGWIHHRARHAVACFLTRG
DLWISWESGMKVFEELLLDADWSVNAGSWMWLSCSAFFQQFFHCYCPVGFGRRTDPSGDYIRRYIPILKDYPN
RYIYEPWNAPESLQKAANCVVGVHYPKPMINHAEGSRLNIERMKQVYQQLSHYRGLSLLASVPTIQEEAEPPM
TDESQTSSGPDSPTRILADVEAAGCSTAPDSSTSVSHPHHNKAQSHIRPGLDPCTSSARPTSPITNMSHIPTS
RTKPASLSSSCPSSSLSLCPGVLQNTSVGTRRKGAIRKVRQSQRQRGRPCCPSTAREDEEGEEEERMQEEEEE
ERMEDEETAAEMTGAL
>Nothobranchius_furz__XP_015822838.1
MAPNSIHWFRKGLRLHDNPALREAVQGAATVRCVYFLDPWFAGSSNVGVNRWRFLLQCLEDLDANLRKVNSRL
FVIRGQPANVFPRLFKEWKISRLTFEYDSEPFGKERDAAIKKLAMEAGVEVIVKISHTLYDLDKIIELNGGQP
PLTYKRFQTLISRLDPPEMPVETLSDTLMGSGITPISEDHGEKYGVPSLEELGFDTEGLPSAVWPGGETEALT
RIERHLERKAWVANFERPRMNANSLLASPTGLSPYLRFGCLSCRLFYFKLTDLYRKVKKNSSPPLSLYGQLLW
REFFYTAATNNPRFDKMEGNPICVRIPWDKNPEALAKWAEAKTGFPWIDAIMTQLRQEGWIHHLARHAVACFL
SRGDLWISWEEGMKVFEELLLDADWSVNAGSWMWLSCSSFFQQFFHCYCPVGFGRRTDPNGDFIRRYLPILRG
FPAKFIYDPWNAPESVQTAAKCIIGVHYPKPMVHHAEASRLNIERMKQIYQQLSRYRGLGLLASVPSTNVTGN
GGMMAYSPAEQHPGTSSSSSSHCYHSVPDSSQSITSSRLCHEFAVPQHPGLLHTRGSSITGKRERESEQNGSG
EEDQASCSVNKMQRQSAETS
>Nothobranchius_furz__XP_015829836.1
MHLKLQLNRKRLACFRTSFLFTFLAARSSFEATMSDRKRRAASSSSGKRPKLPSAPDREEGKEPAGWLLRAVQ
QQREDAKDQSFNRKRLRFISETEKVTQGSEGILYWMLRDQRVQDNWALIHAQQLAAQENLPLHVCFCLVVPKS
PLSTLRHYSFLLKGLEEVAKECKQLNIQFHLLHGAAGDLLPAFVSDRDFGAVVTDFSPLREHLQLLKDVQKKL
KKDVPLMQVDAHNVVPCWEASPKLEYAARTIRGKITSRLPEFLTDFPAVEKHPHTAVRTAKPVDWDEALASLD
VDRSVEEPQWAKPGTAAGMAMLESFIDVRLKLFNTHRNDPNAAALSQLSPWIRFGHLSAQRVALQVRRCGKNA
TESVASFIEELVVRRELTDNFCFYNENYDSVKGAYEWAQKTLNDHAKDKREYLYTREQLEQAQTHDKLWNAAQ
YQMVRDGKMHGFMRMYWAKKILEWTSSPEEALSIALYLNDRYELDGQDPNGFVGCMWSICGIHDQGWSERPVF
GKIRYMNYKGCLRKFDVAQFERKFCPKKP
>Notodromas_mon_CAD7273426.1_unnamed_pr
MSRGSSPPAKKLKLGESRPGGFLIQEISESRRKVGKSVLDFKFNKGRVKILTKATEVPEWAKGIVYWMSRDQR
VQDNWAMLFAQKLALKHKLPLHVCFCLVPKFLDATIRHFRFMLKGLQEVEKECRELEIPFHLLPGQANEVLVN
FVKSNKIGVVVTDFAPLRVPLKWVDDVKNNVPRDVSVVRVDAHNVVPVWIASEKQEYAARTIRPKIHRNLDSY
LTEFPPIQKHPYPPVVKPEAIDWENVENNLEVDRSVEDVEWAAPGTQAGLKQLESFILKRLKYYADKRNDPNV
KALSNLSPWFHFGQISVQRAVLEVKRHGKSFPKAVEGFVEEAVVRRELADNFCFYNQKYDRIAGAYDWARLTL
EAHRKDVREFLYTKEEFDDAKTHDDLWNAAQIQLKQEGKMHGFLRMYWAKKILEWTESPEQALEFAIYLNDRY
NLDGRDPNGYVGCMWSICGIHDQGWAERKVFGKIRYMNYKGCKGKFDVAAFVAKYRARAYPYKKK
>Notodromas_mon_CAD7273494.1_unnamed_pr
MENVSAHWFRHGLRLHDNPALLEGFSRASKVFAFFTFDGESAGTGWIGYNRMKFICESLADLDRSLRSRGGRL
WIFQKDVTTLLSAFKSRFSLTNLSFERDSEAVWQNRDCAVRQHCASSGIDLVEKVSLTLWDPDQILDANGGLP
PLTMVEFLSIAKKRGDPERPVDSENVDYSFFDLETAFANDKGMLDELCLFPGIPTPEDFGILPEPGQEIRLYI
GGETRADELLIKRLQASSVVWTGMIDFPDSGIFFTVEKNAMQMQITQPNHAFPNLIAPPMSLSPYLRYGCLSI
RKFYWLLLDMRRVGLSTTTCTSALGLVMQLMWREYFYCMSTRNPGFGRIEGNQICLPIPWSRDESSLNAWKLG
VTGFPFIDAAMRQVRREGWIHHVARNACAQFLTRGGLWISWEEGLRHFLFYLLDGDWSVCSGNWLWLSSSSFE
KMLDCSECFSPSMFGRRLDPAGDYVRKYVPELSGMPTEYLHEPWLAPLEVQKKAGCILGEHYPKPMIDFVKRS
KRNARRMANFRRQLGCAIPRHCAPSSEEEAREFMWSTAATSVFGHHSHSEVCRGTEVME
>Notodromas_mon_CAD7283181.1_unnamed_pr
KYQIGLKIASRIIDKNGGQAPMTYRQFHSVLSQMDPPHPPAGTVTQEYLDRHCPKSVSPIDEDHDDKYGIPTL
EELGFITSGLKQTLWPGGETEALTRLERHLERRAWVASFGRPRMTPQSLMANQTGLNPYLRFGCLSPKTFYHQ
MSELYRKIKKCPPPLSLHGQLLWREFFYCAATNNPKFDRMEGNPICVQIPWVKNPPALAKWANGQTGYPWIDA
IMTQLREEGWIHQLARHAVACFLTRGDLWLSWEEGMKVFEELLLEADWSVNAGSWMWLSCSSFFQQFFHSYCP
VKFGRKVDPNGDYIRRYLPVLKSYPTKYIHEPWKAPETVQKSARCIVGKDYPLPMLNHSEVSKINVERIRQVY
RRLSNFKNGPGRVPKAISCPLSTESCRSNGGHDDDAAAVIASASLTTPATIEV
>Numida_mele__XP_021232797.1
MQLLTSGTWRLGGRMRHRTIHLFRKGLRLHDNPALLAALQSSEVVYPVYILDRAFMTSAMHIGALRWHFLLQS
LEDLRNSLCQLGSCLLVIQGDYASVVRDHVQKWNITQVTLDAEMEPFYKEMEANIRALGEELGFEVLSLVGHS

LYNTQRVLELNGGAPPLTYKRFLRILSVLGDPEVPVRNLTAEDFQRCSPPELSLAERYGVPLPTDLKIPPESI
SPWRGGESEGLQRLERHLADQGWVARFTKPKTVPNSLLPSTTGLSPYFSMGCLSVRSFFYRLSNIYAQAKHHS
LPPVSLQGQLLWREFFYTVASATPNFTKMAGNPICLQIRWYEDAERLHKWKTAQTGFPWIDAIMTQLRQEGWI
HHLARHAAACFLTRGDLWISWEEGMKVFEELLLDADYSINAGNWMWLSASAFFHHYTRIFCPVRFGRRTDPEG
QYIRKYLPILKNFPSKYIYEPWMASEEEQKQAGCIIGRDYPFPMVDHKAASDHNLQLMKQVREEQYRTAQLTR
DDADDPMEMKLKRDRSEENLTKTKAARMTEQT
>Numida_mele__XP_021242261.1
MGVNAVHWFRKGLRLHDNPALRECIRGADTVRCVYILDPWFAGSSNVGINRWRFLLQCLEDLDANLRKLNSRL
FVIRGQPADVFPRLFKEWSIAKLSIEYDSEPFGKERDAAIKKLASEAGVEVIVRISHTLYDLDKIIELNGGQP
PLTYKRFQTLISRMEPLEMPVETITPEVMQKCTTPVSDDHDEKYGVPSLEELGFDTDGLPSAVWPGGETEALT
RLERHLERKAWVANFERPRMNANSLLASPTGLSPYLRFGCLSCRLFYFKLTDLYKKVKKNSSPPLSLYGQLLW
REFFYTAATNNPRFDKMEGNPICVQIPWDKNPEALAKWAEGRTGFPWIDAIMTQLRQEGWIHHLARHAVACFL
TRGDLWISWEEGMKVFEELLLDADWSVNAGSWMWLSCSSFFQQFFHCYCPVGFGRRTDPNGDYIRRYLPVLRG
FPAKYIYDPWNAPDSVQKAAKCVIGVNYPKPMVNHAEASRLNIERMKQIYQQLSRYRGLGLLATVPSNPNGNG
NGGLMSFSPGENISGCSTAGGAQLGTGDGQTAGVQTCALGDTHTGGSGVQQQGYCQASSILRYAHGDNQQSHL
MQPGRTSLATGVSTGKRPNPEEETQSVGPKVQRQSTN
>Numida_mele__XP_021250745.1
MGTTPGSHLPCGRAVSGGECWVCAQPAAAGCSGARFPHARGWGRSPRRARGRYQRGKRAAQRRSPGPRRGAAS
SCPRGGERVGAARGGHAHVTAHTPSPPSGPAPCCVNARSAAATWGRAAVRRLCVGLRMPRGSGKGRKERDAGT
EEEEAAAAEAVGTLEAAVREARRRAAPSVRDFRYNKQRARLVSRGSELKEGAECILYWMFRDQRVQDNWAFLY
AQRLALKQELPLRVCFCLVPSFLDATIRHYGFMLRGLQEVAKECAELDIPFHLLLGSPKDVLPGFVVKHSVGG
LVTDFCPLRVPRQWVEEVRDRLPEDVPFAQVDAHNIVPCWVTSSKQEYSARTIRAKIHSQLQEFLTEFPPVVR
HPHPPPNPPEPIAWDACYSSLQVDRTVTEVTWATPGTAAGLAMLQSFIAERLKSFGSQRNDPNKAALSNLSPW
FHFGQVSTQRAILEVQKHRHTYKESVDAFVEEAVVRRELAENFCYYNENYDSVRGAYDWAQSTLKLHAKDKRP
FLYELPQLEQATTHDPLWNAAQLQMVREGKMHGFLRMYWAKKILEWTRSPEEALQFAIYLNDRYELDGMDPNG
YVGCLWSICGIHDQGWKERDVFGKIRYMNYAGCKRKFDVDQFERRYAHCK
>Numida_mele__XP_021257408.1
MAAAPSPPRGFCRSVHWFRRGLRLHDNPALQAALRGAASLRCIYILDPWFAASSAVGINRWRFLLQSLEDLDN
SLRKLNSRLFVVRGQPTDVFPRLFKEWGVTRLTFEYDSEPFGKERDAAIVKLAKEAGVEVVIENSHTLYDLDR
IIELNGNKPPLTYKRFQAIISRMELPKKPVSSIVSQQMETCKVDIQENHDDVYGVPSLEELGFPTDGLAPAVW
QGGETEALARLDKHLERKAWVANYERPRMNANSLLASPTGLSPYLRFGCLSCRLFYYRLWELYKKVKRNSTPP
LSLYGQLLWREFFYTAATNNPKFDRMEGNPICIQIPWDKNPEALAKWAEGKTGFPWIDAIMTQLRQEGWIHHL
ARHAVACFLTRGDLWISWESGVRVFDELLLDADFSVNAGSWMWLSCSAFFQQFFHCYCPVGFGRRTDPSGDYV
KRYLPKLKGFPSRYIYEPWNAPESVQKAAKCIIGVDYPKPMVNHAETSRLNIERMKQIYQQLSRYRGLCLLAS
VPSCVEDLSGPVTDSAPGQGSSTSTAIRPPQSDQASPKRKHEGAEELCTEELYKRAKVTGLPALEIPGKSS
>Nyctibius_gran__NXQ79840.1
DNWAFLYAQRLALKQELPLHVCFCLVPKFLDATIRHYGFMLRGLQEVAEECAELNIPFHLLLGYAKDVLPAFV
VEHGVGGLVTDFCPLRLPRQWVEDVREQLPEDVPFAQVDAHNIVPCWVASPKQEYSARTIRGKIHSQLPEFLT
EFPPVICHPYPPSCLPEPIAWEACYSSLQVDRTVKEVEWATPGTAAGLAVLQSFIAERLKSFGSHRNDPNKAA
LSNLSPWFHFGQVSTQRAILEVQKHRGKYKESVDVFVEEAVVRRELAENFCYYNENYDSVQGAYDWAQTTLKL
HAKDKRPFLYELQELEQGTTHDPLWNAAQLQMVLEGKMHGFLRMYWAKKILEWTRSPEEALRFAIYLNDRYEL
DGRDPNGYVGKLQDGGMGWGGCLWSICGIHDQGWAERAVFGKIRYMNYAGCKRKFDVGQFERRYAP
>Nyctibius_gran__NXQ81244.1
CIYILDPWFAASSAVGINRWRFLLQSLEDLDNSLRKLGSRLFVVRGQPTDVFPRLFKEWGVTRLTFEYDSEPF
GKERDAAIIKLAKEAGVEVLIENSHTLYDLDRIIELNGHKPPLTYKRFQAIISRMELPKKPVSTVMSQQMEAC
KVDIQENHDDVYGVPSLEELGFPTDGLAPAVWQGGETEALARLDKHLERKAWVANYERPRMNANSLLASPTGL
SPYLRFGCLSCRLFYYRLWELYKKVKRNSTPPLSLYGQLLWREFFYTAATNNPKFDRMEGNPICIQIPWDRNP
EALAKWAEGKTGFPWIDAIMTQLRQEGWIHHLARHAVACFLTRGDLWISWESGVRVFDELLLDADFSVNAGSW
MWLSCSAFFQQFFHCYCPVGFGRRTDPSGDYVKRYLPKLKGFPSRYIYEPWNAPESVQKAAKCIIGVDYPKPM
VNHAETSRLNIERMKQIYQQLSRYRGLCLLASVPSCVEDLSGPVTDSAPGQGCSTSTAMRLSQADQASPKRKH
EGAEELCTEELYKRAKVTGLPTPEIPGKS
>Nyctibius_gran__NXQ83460.1
MPHRTIHLFWKGLRLHDNPTLLAALESSEAIYPVYILDRRFMTSVMHIGALRWHFLLQSLEDLQKNLRQLGSC
LLVIQGEYESVLRDHVQKWNITQVTLDTEVEPFYKEMEANIRRLGEELGFEVLSLVGHTLYDTKRILDLNGGT
PPLTYKRFLHILSLLGDPEVPVRNLTAGDFQRCQPPDPGLAECYRVPLPVDLGIPLESLSPWRGGETEGLQRL
EQHLTDQGWVASFTKPRTIPNSLLPSTTGLSPYFSMGCLSVRTFFYRLSNIYAQAKHHSLPPVSLQGQLLWRE
FFYTVASATPNFTKMAGNPICLQIRWYEDAERLHKWKTAQTGFPWIDAIMTQLRQEGWIHHLARHAVACFLTR
GDLWISWEEGMKV
>Nyctibius_gran__NXQ85569.1

MQHSSIHWFRKGLRLHDNPALLAAATNCHRLHPLFILDPSSSRASTNAWRFLLDALRDLDGSLQEMGSRLFVV
RGCPEEVFPRLFHAWETTRLTFEVDTEPSAHRRDATVAELAARHGVEVIQEVSHTLYDTKRVLALNDGKAPLT
YKHLQSLLASLGPPEKPAPALTQEHLQSCHTPCQASHDTDYGVPTLEELGQDPAEVGPHLYPGGETAALARLN
ALMKRTAWVRGFKKPETEPNSLSPSTTVLSPYLKFGCLSVRTFWWRLDEVYQAQREHSRPPVSLHGQLLWREF
FYTAGTSIPNFDRMVGNPVCLQVDWDDNPQHLHAWREGRTGYPFIDAIMTQLRTEGWIHHLARHAVACFLTRG
DLWVSWEEGLKVFEELLLDADWSLNAGNWLWLSGSAFFHRYFHVYSPVAFGKKTDRDGAYIRKYLPVLKDFPA
KYIYEPWKAPQAVQEQAGCLVGTHYPRPIVEHRAARERNLGRMK
>Nyctibius_gran__NXQ88889.1
KVDYDVCSNYGNWLYSAGIGNDPRDNRKFNMIKQGLDYDGNGDYVRLWLPELQGIKGADIHTPWALNSAALSQ
AGVTLGETYPQPVVTAPEWSRHINQRPQGRSPHPRGRRGPAHTPAQHKDRGIDFYFSRKKDV
>Nyctibius_gran__NXQ92069.1
NPALRECIQGADTVRCVYILDPWFAGSSNVGINRWRFLLQCLEDLDANLRKLNSRLFVIRGQPADVFPRLFKE
WNIAKLSIEYDSEPFGKERDAAIKKLASEAGVEVIVRISHTLYDLDKIIELNGGQPPLTYKRFQTLISRMEPL
EMPVETITPEVMEKCATPVSDDHDEKYGVPSLEELGFDTDGLPSAVWPGGETEALTRLERHLERKAWVANFER
PRMNANSLLASPTGLSPYLRFGCLSCRLFYFKLTDLYKKVKKNSSPPLSLYGQLLWREFFYTAATNNPRFDKM
EGNPICVQIPWDKNPEALAKWAEGRTGFPWIDAIMTQLRQEGWIHHLARHAVACFLTRGDLWISWEEGMKVFE
ELLLDADWSVNAGSWMWLSCSSFFQQFFHCYCPVGFGRRTDPNGDYIRRYLPVLRGFPAKYIYDPWNAPESVQ
KAAKCVIGVNYPKPMVNHAEASRLNIERMKQIYQQLSRYRGLGLLATVPSNPNGNGNGGLMGYSPGESISGCG
STGGAQLGTGDGHAVVQPCALGDSHTGASGIQQQGYCQASSILHYAHGDNQQSHLLQAGRTAFGTGISAGKRP
NPEEETESVRPKVQRQSTS
>Nymphon_str_KAG1657147.1_Deoxyribod
MIGIVWFRQDLRLSDNDSFFSSLPGVRSSNPLWLHNSLENLSSDLRAGSSNLILRKGGSLAVLSELVNETQAT
HLYWNRCYEPKISKRDAVIKATLRDQIEIRSFKGNLLIEPWQVLKADGSPYRVFTPYWKALTQKLILSVPLAT
PDNFKPYKKWPRSAKLSQLNFLPQHPEPSWDKKLMSNWKVGEAAAHDALHQFLEDGVQNYKEQRDFPAVGGTS
LLSPHLHFGEISPTQVYHYSKNHKAENPHHDHGVGHILRQIVWREFAYSLMYHFPQTITDPLFEKYKGFEWRE
SKEELKRWQQGKTGFPIIDAGMRELWQTGIMHNRVRMIVASFLCKNLLIHWHEGEAWFRDTLVDADIANNTMG
WQWVAGSGADAAPYYRIFNPILQSKKFDAKAEYIRRWVPELSHLDNKEIHQPSAAQAKKV
>Oceanites_ocea__NXF45076.1
CIYILDPWFAASSAVGINRWRFLLQSLEDLDNSLRKLNSRLFVVRGQPTDVFPRLFKEWGVTRLTFEYDSEPF
GKERDAAIIKLAKEAGVEVVIENSHTLYDLDRIIELNGHKPPLTYKRFQAIISRMELPKKPVSTVMSQQMETC
KVDIQENHDDVYGVPSLEELGFPTDGLAPAVWQGGETEALARLDKHLERKAWVANYERPRMNANSLLASPTGL
SPYLRFGCLSCRLFYYRLWELYKKVKRNSTPPLSLYGQLLWREFFYTAATNNPKFDRMEGNPICIQIPWDRNP
EALAKWAEGKTGFPWIDAIMTQLRQEGWIHHLARHAVACFLTRGDLWISWESGVRVFDELLLDADFSVNAGSW
MWLSCSAFFQQFFHCYCPVGFGRRTDPSGDYVKRYLPKLKGFPSRYIYEPWNAPESVQKAAKCIIGVDYPKPM
VNHAETSRLNIERMKQIYQQLSRYRGLCLLASVPSCVEDLSGPVADSASGQGCSTSTGMRLSQADQASPKRKH
EGAEELCTEELYKRAKVTCLPAPEIPGKSL
>Oceanites_ocea__NXF45918.1
MPHHTIHLFRKGLRLHDNPTLLAALESSEVVYPVYILDRKFMTSVMHIGALRWHFLLQSLEDLQKNLCQLGSC
LLVIQGEYESVLRDHIQKWNITQVTLDAETEPFYKEMEANIRRLGEELGFEVLSLVGHSLYDTKRILDLNGGT
PPLTYKRFLHILSLLGDPEVPVRNLTAEDFQRCKPPDLGLAECYRVPLPVDLKIPPESLSPWRGGETEGLQRL
EQHLTDQGWVTSFAKPRTIPNSLLPSTTGLSPYFSLGCLSVRTFFYRLSNIYAQAKHHSLPPVSLQGQLLWRE
FFYTVASATPNFTKMAGNPICLQISWYEDAEGLHKWKTAQTGFPWIDAIMTQLRQEGWIHHLARHAVACFLTR
GDLWISWEEGMKVFEELLLDADYSINAGNWMWLSASAFFHQYTRIFCPVRFGRRTDPEGQYIRKYLPILKNFP
SKYIYEPWTASEEEQKQAGCIIGQDYPFPMVNHKEASDHNLQLMKQVREEQYRTAQLTRDDTDDPMEMKVKRD
HSEENISKGKVARMTD
>Oceanites_ocea__NXF51252.1
KVDYDVCSNYGNWLYSAGIGNDPRDNRKFNMIKQGLDYDGNGDYVRLWVPELQSIKGADVHTPWALNSAALSQ
AGVTLGETYPQPVVTAPEWSRHINQRPQGRSPHPRGRRGPARTPMQHKDRGIDFYFSRKKDV
>Oceanites_ocea__NXF53842.1
MQHSSIHWFRKGLRLHDNPALLAAAADCCCLHPLFILDPSSGRAGATARRFLLDALQDLDGSLREVGSRLFVV
RGCPEEVFPHLFHAWGTTRLTFEVDTEPPARQRDAAVAELAARHGVEVIQEVSHTLYDTKRVLALNDGKAPLT
YKRLQSLLAFLGPPEKPAPVLTREHLQGCHTPRQVSHDADYRVPTLEELGEDPTKVGPHLYPGGERAALARLD
TLMERTAWVCGFKKPETEPTSLSPSTTVLSPYLKFGCLSVRTFWWRLDEIYQGREHSRPPVSLHGQLLWREFF
YTAGASIPNFDRMVGNPVCLQVDWDDNPQHLCAWREGQTGYPFIDAIMTQLRIEGWIHHLARHAVACFLTRGD
LWVSWEEGLKVFEELLLDADWSLNAGNWLWLSGSAFFHRYFHVYSPVAFGKKTDRDGAYIRKYLPVLKDFPTE
YIYEPWKAPRAVQERAGCLVGTHYPRPIVEHGAASKRNLGRMKAAR
>Oceanites_ocea__NXF54397.1
WFASENKCFFTIPCRFLLQCLEDLDANLRKLNSRLFVIRGQPADVFPRLFKEWNIAKLSIEYDSEPFGKERDA
AIKKLASEAGVEVIVRISHTLYDLDKIIELNGGQPPLTYKRFQTLISRMEPLEMPVETITPEVMEKCTTPVSD
DHDEKYGVPSLEELGFDTDGLPSAVWPGGETEALTRLERHLERKAWVANFERPRMNANSLLASPTGLSPYLRF

GCLSCRLFYFKLTDLYKKVKKNSSPPLSLYGQLLWREFFYTAATNNPRFDKMEGNPICVQIPWDRNPEALAKW
AEGRTGFPWIDAIMTQLRQEGWIHHLARHAVACFLTRGDLWISWEEGMKVFEELLLDADWSVNAGSWMWLSCS
SFFQQFFHCYCPVGFGRRTDPNGDYIRRYLPVLRGFPAKYIYDPWNAPESVQKAAKCIIGVNYPKPMVNHAEA
SRLNIERMKQIYQQLSRYRGLGLLATVPSNPNGNGNGGLMGYSPGESISGCGSTGGHLFPASPGAQLGTGDGH
TVVQPCALGDSHTGASGIQQQGYCQASSILHYAHGDNQQSHLLQAGRTALGTGISAGKRPNPEEETQSVGPKV
QRQSTN
>Oceanites_ocea__NXF54996.1
DNWAFLYAQRLALKQELPLHVCFCLVPKFLGATIRHYGFMLRGLQEVAKECAELNIPFHLLLGYAKDVLPVFV
VEHGVGGLVTDFCPLRLPRQWVEDVRERLPEDVPFAQVDAHNIVPCWVASPKQEYSARTIRGKIHAQLPEFLT
EFPAIVHHPYPPSCPSEPIAWEACYSSLQVDRTVKEVEWATPGTAAGLAVLQSFIAERLKSFGSHRNDPNKSA
LSNLSPWFHFGQVSTQRAILEVQKHRGKYKESVDAFVEEAVVRRELAENFCYYNENYDSVQGAYDWAQTTLKL
HTKDKRPFLYELQELEQGTTHDPLWNAAQLQMVREGKMHGFLRMYWAKKILEWTRSPEEALRFAIYLNDRYEL
DGRDPNGYVGKLQGCLWSICGIHDQGWAERAIFGKIRYMNYAGCKRKFDVGQFERRYAPH
>Oceanodroma_teth__NXH68236.1
DNWAFLYAQRLALKQELPLRVCFCLVPKFLDATIRHYGFMLRGLQEVAKECTELNIPFHLLLGYAKDVLPAFV
VEHGVGGLVTDFCPLRLPRQWVEDVRERLPEDVPFAQVDAHNIVPCWVTSPKQEYSARTIRGKIHAQLPEFLT
EFPPVIRHPYAPSCLAEPIAWEACYSSLQVDCTVKEVEWATPGTAAGLAVLQSFIVERLKSFGSHRNDPNKEA
LSNLSPWFHFGQVSTQRAILEVQKHRSEYKESVDAFVEEAVVRRELAENFCYYNENYDSVQGAYDWAQTTLKL
HAKDKRPFLYELQELEQGTTHDPLWNAAQLQMVREGKMHGFLRMYWAKKILEWTRSPEEALRFAIYLNDRYEL
DGRDPNGYVGKLQDGDMGLGGCLWSICGIHDQGWAERAIFGKIRYMNYAGCKRKFDVSQFEHRYAP
>Oceanodroma_teth__NXH68960.1
KVDYDVCSNYGNWLYSAGIGNDPRDNRKFNMIKQGLDYDGNGDYVRLWVPELQGIKGADIHTPWALNSAALSQ
AGVTLGETYPQPVVTAPEWSRHINQRPQGRSPHPRGRRGPAHMPVQHKDRGIDFYFSRKKDV
>Oceanodroma_teth__NXH74094.1
MQHSSIHWFRKGLRLHDNPALLAAAAGCCRLHPLFILDPSSSRAGANAQRFLLDTLRDLDGSLQEMGSRLFVV
RGCPEEVFPRLFHAWGTTRLTFEVDTEPPARRRDTAVAELAARHGVEVIREVSHTLYDTERVLALNDGKAPLT
YKRLQSLLASLGPPEKPAPALRQEHLRGCRTPCQVSHDADYGVPTLEELGQDPAEMGPHLYPGGETAALARLD
VFMERTAWVCGFKKPETEPTSLSPSTTVLSPYLTFGCLSVRTFWWRLDEVYQRREHSRPPVSLHGQLLWREFF
YTAGAGIPNFDQMVGNPVCLQVDWDDNPQHLRAWREGQTGYPFIDAIMTQLRTEGWIHHLARHAVACFLTRGD
LWVSWEEGLKVFEELLLDADWSLNAGNWLWLSGSAFFHRYFHVYSPIAFGKKTDRDGAYIRKYLPVLKDFPAE
YIYEPWKAPRAVQEQAGCLVGIHYPQPIVEHRAASERNLGRMKAAR
>Oceanodroma_teth__NXH75833.1
MPHRTIHLFRKGLRLHDNPTLLAALESSEVVYPVYILDRKFMMSVMHIGALRWHFLLQSLEDLQQNLCQLGSC
LLVIQGEYESVLRDHIQKWNITQVTLDAEIEPFYKEMEANIQRLGEELGFEVLSLVGHSLYDTKRILDLNGGT
PPLTYKRFLHILSLLGDPEVPVRNLTAEDFQRCRPPDPGLAECYRVPLPVDLKIPPESLSPWRGGETKGLQRL
EQHLIDQGWVASFTKPRTIPNSLLPSTTGLSPYFSMGCLSVRTFFYRLSNIYAQAKHHSLPPVSLQGQLLWRE
FFYTVASATPNFTKMAGNPICLQISWYEDAERLHKWKTAQTGFPWIDAIMTQLRQEGWIHHLARHAVACFLTR
GDLWISWEEGMKVFEELLLDADYSINAGNWMWLSASAFFHQYTRIFCPVRFGKRTDPEGQYIRKYLPVLKNFP
SKYIYEPWTASEEEQKQAGCIIGQDYPFPMVNHKEASDHNLQLMKQVREEQHRTAQLTRDDTDDPMEMKVKRD
HSEENISKEKVARMTE
>Oceanodroma_teth__NXH76752.1
HFAYFVPRIIELNGHKPPLTYKRFQAIISRMELPKKPVSTVMSEQMETCKVDIQENHDDVYGVPSLEELGFPT
DGLAPAVWQGGETEALARLDKHLERKAWVANYERPRMNANSLLASPTGLSPYLRFGCLSCRLFYYRLWELYKK
VKRNSTPPLSLYGQLLWREFFYTAATNNPKFDRMEGNPICIQIPWDRNPEALAKWAEGKTGFPWIDAIMTQLR
QEGWIHHLARHAVACFLTRGDLWISWESGVRVFDELLLDADFSVNAGSWMWLSCSAFFQQFFHCYCPVGFGRR
TDPSGDYVKRYLPKLKGFPSRYIYEPWNAPESVQKAAKCIIGVDYPKPMVNHAETSRLNIERMKQIYQQLSRY
RGLCLLASVPSCVEDLSGPVTDSASGQGCSTSTAMRLSQADQASPKRKHEGAEELCAEELYKRAKVTCLPAPE
IPGKT
>Oceanodroma_teth__NXH78450.1
RFLLQCLEDLDANLRKLNSRLFVIRGQPADVFPRLFKEWNIAKLSIEYDSEPFGKERDAAIKKLASEAGVEVI
VRISHTLYDLDKIIELNGGQPPLTYKRFQTLISRMEPLEMPVETITPEVMEKCTTPVSDDHDEKYGVPSLEEL
GFDTDGLPSAVWPGGETEALTRLERHLERKAWVANFERPRMNANSLLASPTGLSPYLRFGCLSCRLFYFKLTD
LYKKVKKNSSPPLSLYGQLLWREFFYTAATNNPRFDKMEGNPICVQIPWDRNPEALAKWAEGRTGFPWIDAIM
TQLRQEGWIHHLARHAVACFLTRGDLWISWEEGMKVFEELLLDADWSVNAGSWMWLSCSSFFQQFFHCYCPVG
FGRRTDPNGDYIRRYLPVLRGFPAKYIYDPWNAPESIQKAAKCIIGVNYPKPMVNHAEASRLNIERMKQIYQQ
LSRYRGLGLLATVPSNPNGNGNGGLMGYSPGESISGCGSTGGTQLGTGDGHTVVQPCALGDPHTGASGIQQQG
YCQASSILHYAHGDNQQSHLLQAGRTALGTGISAGKRPNPEEETQSVGPKVQRQSTS
>Ochotona_curz__XP_040836089.1
MMAATAVPAAAPAAAAGASSVHWFRKGLRLHDNPALLAAVRGARCVRCVYILDPWFAASSSVGINRWRFLLQS
LEDLDTSLRKLNSRLFVVRGQPADVFPRLFKEWGVTRLTFEYDSEPFGKERDAAIMKMAKEAGVEVVTENSHT

LYDLDRIIELNGQKPPLTYKRFQAIISRMELPRKPVGSVTSEQMESCRAEIQDNHDESYGVPSLEELGFPTEG
LGPAVWQGGETEALARLDKHLERKAWVANYERPRMNANSLLASPTGLSPYLRFGCLSCRLFYYRLWDLYKKVK
RNSTPPLSLFGQLLWREFFYTAATNNPRFDRMEGNPICIQIPWDRNPEALAKWAEGKTGYPWIDAIMTQLRQE
GWIHHLARHAVACFLTRGDLWVSWESGVRVFDELLLDADFSVNAGSWMWLSCSAFFQQFFHCYCPVGFGRRTD
PSGDYIRRYLPKLKGFPSRYIYEPWNAPESVQKAAKCIIGVDYPRPIVNHAETSRLNIERMKQIYQQLSRYRG
LCLLASVPSCVEDLSHPVAEPSSSQAGNVSSTGPRPLSSGPASPKRKLEAAEEPPGEELSKRARVAEVPAPEL
PSKAV
>Ochotona_curz__XP_040840948.1
MGVNAVHWFRKGLRLHDNPALKECIQGADTIRCVYILDPWFAGSSNVGINRWRFLLQCLEDLDANLRKLNSRL
FVIRGQPADVFPRLFKEWNITKLSIEYDSEPFGKERDAAIKKLATEAGVEVIVRISHTLYDLDKIIELNGGQP
PLTYKRFQTLISKMEPLEIPVETITSEVIEKCTTPLSEDHDEKYGVPSLEELGFDTDGLSSAVWPGGETEALT
RLERHLERKAWVANFERPRMNANSLLASPTGLSPYLRFGCLSCRLFYFKLTDLYKKVKKNSSPPLSLYGQLLW
REFFYTAATNNPRFDKMEGNPICVQIPWDKNPEALAKWAEGRTGFPWIDAIMTQLRQEGWIHHLARHAVACFL
TRGDLWISWEEGMKVFEELLLDADWSINAGSWMWLSCSSFFQQFFHCYCPVGFGRRTDPNGDYIRRYLPVLRG
FPAKYIYDPWNAPEGIQKVAKCLIGVNYPKPMVNHAEASRLNIERMKQIYQQLSRYRGLGLLASVPSNPNGNG
GLMGYSPGENIPGCSNSGSCPQGSGILHCGHGDSQQTHLLKQGRGSLGTGISSGKRPSQEEDTQSIGPKVQRQ
STN
>Ochotona_prin__XP_004585434.1
MMAATAAPAAVPAAAAGASSVHWFRKGLRLHDNPALLAAVRGARCVRCVYILDPWFAASSSVGINRWRFLLQS
LEDLDTSLRKLNSRLFVVRGQPADVFPRLFKEWGVTRLTFEYDSEPFGKERDAAIMKMAREAGVEVVTENSHT
LYDLDRIIELNGQKPPLTYKRFQAIISRMELPRKPVGSVTSEQMESCRAEIQDNHDESYGVPSLEELGFPTEG
LGPAVWQGGETEALARLDKHLERKAWVANYERPRMNANSLLASPTGLSPYLRFGCLSCRLFYYRLWDLYKKVK
RNSTPPLSLFGQLLWREFFYTAATNNPRFDRMEGNPICIQIPWDRNPEALAKWAEGKTGYPWIDAIMTQLRQE
GWIHHLARHAVACFLTRGDLWVSWESGVRVFDELLLDADFSVNAGSWMWLSCSAFFQQFFHCYCPVGFGRRTD
PSGDYIRRYLPKLKGFPSRYIYEPWNAPESVQKAAKCIIGVDYPRPIVNHAETSRLNIERMKQIYQQLSRYRG
LCLLASVPSCVEDLSHPVAEPSSSQAGNVSSTGPRPLSSGPASPKRKLEAAEEPPGEELSKRARVAVVPAPEL
PSKAV
>Ochotona_prin__XP_004589630.1
MGVNAVHWFRKGLRLHDNPALKECIQGADTIRCVYILDPWFAGSSNVGINRWRFLLQCLEDLDANLRKLNSRL
FVIRGQPADVFPRLFKEWNITKLSIEYDSEPFGKERDAAIKKLATEAGVEVIVRISHTLYDLDKIIELNGGQP
PLTYKRFQTLISKMEPLEIPVETITSEVIEKCTTPLSEDHDEKYGVPSLEELGFDTDGLSSAVWPGGETEALT
RLERHLERKAWVANFERPRMNANSLLASPTGLSPYLRFGCLSCRLFYFKLTDLYKKVKKNSSPPLSLYGQLLW
REFFYTAATNNPRFDKMEGNPICVQIPWDKNPEALAKWAEGRTGFPWIDAIMTQLRQEGWIHHLARHAVACFL
TRGDLWISWEEGMKVFEELLLDADWSINAGSWMWLSCSSFFQQFFHCYCPVGFGRRTDPNGDYIRRYLPVLRG
FPAKYIYDPWNAPEGIQKVAKCLIGVNYPKPMVNHAEASRLNIERMKQIYQQLSRYRGLGLLASVPSNPNGNG
GLMGYSPGENIPGCSNSGSCPQGSGILHCGHGDSQQTHLLKQGRGSLDTGISSGKRPSQEEDTQSIGPKVQRQ
STN
>Octopus_bima__KOG00441.1
MSNESGKHVIHWFRKGLRLHDNPALMEALKGATTLRCIYTLDPWFAGSSQVGINKWGFLLNCLEDLDDSLRKL
NSRLFVIRGQPANMFPQLFKEWNINTLSFEDDPEPFGKDRDNAICTLAREAGVEVIIRTCHTLYESQKILDLN
GGRPPLTYNKFQKILENMKMPPKPLDIITASDWGNCQTPLSSDHDDKYGVPTLEDLGFETEGLDLAHFHGGES
EALARLDRHLERKIKKKNIPPLSLHGQLLWREFFYTVASNNPRFDQMLGNPVCVQIPWDTNQEALAKWAEGMT
GFPWIDAIMKQLRKEGWIHHLARHSVACFLTRGDLWLSWEEGMKVFDELLLDADWSVNAGMWMWLSCSSFFQQ
FFHCYCPVGFGKRIDPNGDFIRHYLPILKRFPAKYIYEPWNAPESVQKTAKCIVGKDYPVPMVTHSEVSRINM
ERMRQVYHRLTMRTPAVISKPIHSRENLTYSRIHHNYPMVQHNVHSRPVLLSQN
>Octopus_bima__XP_014774572.1
MSVKDCNGVMKHEKKKKIAVHWFRHGQRLHDNPALVNALKDCDEFYPVFIFDGEVAGTKLCGFNRWRFLLENL
KDLDESFAEYGGRLYTFQGKPVEVFKNLQKEWGITHITAEIDPEPIWQERDDAVKEFCQKSGIECDFFTSHTL
WDPKLLLKKNGGTPPLTFELFQLVTSSLGPPLRPLDTPTFEGVKMPLPENHEKFAVPTLKSLGIDPEFEEQKS
PINVFIGGEKRALVLLKARLEKEAQSFRHGQCLPNHQEQPELLARAVSLSPYLRFGCVSIRKTYWDICDTYKR
IKKVEAPNEIVCQLYWREYFYIMSIDNINFDKIENNPYCLKINWQYNEEFLKKWEMGQTGYPWIDAIMNQLRF
EGWNHHVGRHAVSCFLTRGDLWVSWEDGLKLFLKYQLDADWSVCAGNWMWVSSSAFEKALQCPTCYSPVVYGM
RMDRNGDFVKTYVPVLKDMPLKYLFCPWKAPLEIQEKANCIIGKDYPEPIVMHRDASKQNMTKMYKVKEHLLH
QDVPHCGPTNETEVWKFAWLPPIEHHDLAHNI
>Octopus_bima__XP_014780460.1
MRHELFLQYLFILDNWAMLFAQRLALKEKTSLHVCFCVVVPFLEATKRHYDFLLNGLKEVEKECCNLDIQFHL
LKGDPSSEVSSLVKELNIGAVVTDFSPLRLPRKWLTDLTENLPKNVGLCQVDTHNIVPCWITSEKQEYGARTI
RNKIHSQMCNFLTEFPPVIKHPHKAKTEAKPTNWNTIIGKSDIDETVKPVDWAKPGTVAGYATLESFCNERLK
HFATERNDPTKNSLSNLSPWLRFGHISPQRCILVVKKFQKSYKNSVDAFVEEAVIRSELADNYCYYQKDYDNI
NGAYEWARNSLKQHAKDKREYVYSRKVFEEGKTHDQLWNAAQIQLVKEGKMHGFLRMYWAKKILEWSTTPSEA

LEIAIYLNDRYSLDGIDSNGYVGCMWSVCAIHDRAWGERKIIGKIRIMTYEGCKRKFDVDAYITKYCSKNSKK
I
>Octopus_sin_XP_029634730.1_cry-1
MSMNDCNGVMKHEKKQKIAVHWFRHGQRLHDNPALVNALKDCDEFYPVFIFDGEVAGTKLCGFNRWRFLLENL
KDLDESFAEYGGRLYTFQGDPIDVFKNLQKEWGITHITAEIDPEPIWLERDDAVKKFCQKSGIECDFFTSHTL
WDPQLLLKKNGGTPPLTFELFQLVTSSLGPPLRPLDTPNFEGVKMPLPENHDKFAVPTLKSLGIHPEFEEQKS
PINVFIGGEKRALVLLKARLEKEAQSFRHGQCLPNHQEQPELLARAVSLSPYLRFGCVSIRKTYWDICDTYKK
IKNVEAPNEIVCQLYWREYFYIMSIDNINFDKIENNPYCLKINWQYNEEFLKKWEMGQTGYPWIDAIMNQLRF
EGWNHHVGRHAVSCFLTRGDLWVSWEDGLKTFLKYQLDADWSVCAGNWMWVSSSAFEKALQCPTCYSPVMYGM
RMDRNGDFVKTYVPVLKDMPLKYLFCPWKAPLEIQEKANCIIGKDYPEPIVMHRDASKQNMAKMYKVKEHLLH
QDVPHCGPTNETEVWKFAWLPPIEHHDLAHNI
>Octopus_sin_XP_029646224.1_cry-1_X1
MSNESGKHVIHWFRKGLRLHDNPALMEALKGATTLRCIYTLDPWFAGSSQVGINKWGFLLNCLEDLDDSLRKL
NSRLFVIRGQPANMFPQLFKEWNINTLSFEDDPEPFGKDRDNAICTLAREAGVEVIIRTCHTLYESQKILDLN
GGRPPLTYNKFQKILENMKMPPKPLDIITANDWGNCQTPLSSDHDDKYGVPTLEDLGFDTEGLDLAHFHGGES
EALARLDRHLERKAWVASFEKPKMTPQSMFPSHTALSPYLRFSCLSPRLFYWKLTELYRKIKKKNIPPLSLHG
QLLWREFFYTVASNNPRFDQMLGNPVCVQIPWDTNQEALAKWAEGMTGFPWIDAIMKQLRKEGWIHHLARHSV
ACFLTRGDLWLSWEEGMKVFDELLLDADWSVNAGMWMWLSCSSFFQQFFHCYCPVGFGKRIDPNGDFIRHYLP
ILKRFPAKYIYEPWNAPESVQKTAKCIVGKDYPIPMVTHSEVSRINMERMRQVYHRLTMRTPAVISKPIHSRE
NLTYSKIYHRNPTTNGILLMGLNCNSAASNRKEVTQDI
>Octopus_sin_XP_029648244.1_photolyase
MAKRTANSKIEDEPADLKKSKNESLVEKIQESRIKCAKSVQDFKFNKKRVRLLNEVEEFLEGSQGVLYWMVRD
QRVQDNWAMLFAQRLALKEKTSLHVCFCVVVPFLEATKRHYDFLLNGLKEVEKECCNLDIQFHLLKGDPSSEV
SSLVKELNIGAVVTDFSPLRLPRKWLTDLTENLPKNVGLCQVDTHNIVPCWITSEKQEYGARTIRNKIHSQMC
NFLTEFPPVIKHPHEAKTEAKPTDWNTIIGKSDTDEAVKPVDWAKPGTAAGYATLESFCNERLKHFATERNDP
TKNSLSNLSPWLRFGHISPQRCILVVKKFQKSHKNSVDAFVEEAVIRSELADNYCYYQKDYDNINGAYEWARN
SLKQHAKDKREYIYSRKEFEEGKTHDQLWNAAQIQLVKEGKMHGFLRMYWAKKILEWSTTPSEALEIAIFLND
RYSLDGMDSNGYVGCMWSVCAIHDRAWGERNVIGKIRIMTYQGCKRKFDVDSYITKYCSKNSKKI
>Odontophorus_guja__NXJ03486.1
MPHRTIHLFRKGLRLHDNPALLAALQSSEVVYPVYILDRAFMTSVMHIGTLRWHFLLQSLEDLRNSLCQLGSC
LLVIQGEYVAVVRDHVQKWNITQVTLDAEMEPFYKEMEANIRGLGEELGFEVLSLVGHSLYDTQRILELNGGT
PPLTYKRFLHILSLLGDPEVPVRNLTAEDFQRCSPPELSLAEHYGVPLPVDLKIPPESISPWRGGESEGLQRL
EQHLANQGWVASFTKPKTAPNSLLPSTTGLSPYFSMGCLSVRSFFYRLSNIYAQAKHHSLPPVSLQGQLLWRE
FFYTVASATPNFTKMAGNPICLQIRWYEDAERLHKWKTVTRCTSVMAAFWLGAFGMADSCFLLQAQTGFPWID
AIMTQLRQEGWIHHLARHAAACFLTRGDLWISWEEGMKVFEELLLDADYSINAGNWMWLSASAFFHQYTRIFC
PVRFGRRTDPEGQYIRKYLPVLKNFPSKYIYEPWTASEEEQKQAGCIIGRDYPFPMVDHKEASSHNLQLMKQV
REEQYRTAQLTRDDADDPMEMKLKRDHSEENLTKTKAARMTE
>Odontophorus_guja__NXJ10722.1
MGVNAVHWFRKGLRLHDNPALKECIRGADTVRCVYILDPWFAGSSNVGINRWRFLLQCLEDLDANLRKLNSRL
FVIRGQPADVFPRLFKEWSIAKLSIEYDSEPFGKERDAAIKKLASEAGVEVIVRISHTLYDLDKIIELNGGQP
PLTYKRFQTLISRMEPLEMPVETITPEAMQKCTTPVSDDHDEKYGVPSLEELGFDTDGLPSAVWPGGETEALT
RLERHLERKAWVANFERPRMNANSLLASPTGLSPYLRFGCLSCRLFYFKLTDLYKKVKKNSSPPLSLYGQLLW
REFFYTAATNNPRFDKMEGNPICVQIPWDKNPEALAKWAEGRTGFPWIDAIMTQLRQEGWIHHLARHAVACFL
TRGDLWISWEEGMKVFEELLLDADWSVNAGSWMWLSCSSFFQQFFHCYCPVGFGRRTDPNGDYIRRYLPVLRG
FPAKYIYDPWNAPESVQKAAKCVIGVNYPKPMVNHAEASRLNIERMKQIYQQLSRYRGLGLLATVPSNPNGNG
NGGLMSFSPGESISGCSSTGGAPLGTGDGQTVGVQTCALGDSHTGGSGVQQQGYCQASSILRYAHGDNQQSHL
IQPGRASLGAGISAGKRPNPEEETQSVGPKVQRQSTN
>Odontophorus_guja__NXJ13662.1
CIYILDPWFAASSAVGINRWRFLLQSLEDLDNSLRKLNSRLFVVRGQPTDVFPRLFKEWGVTRLTFEYDSEPF
GKERDAAIIKLAKEAGVEVVIENSHTLYDLDRIIELNGNKPPLTYKRFQAIINRMELPKKPVSSIVSQQMETC
KVDIQENHDDVYGVPSLEELGFPTDGLAPAVWQGGETEALARLDKHLERKAWVANYERPRMNANSLLASPTGL
SPYLRFGCLSCRLFYYRLWELYKKVKRNSTPPLSLYGQLLWREFFYTAATNNPKFDRMEGNPICIQIPWDKNP
EALAKWAEGKTGFPWIDAIMTQLRQEGWIHHLARHAVACFLTRGDLWISWESGVRVFDELLLDADFSVNAGSW
MWLSCSAFFQQFFHCYCPVGFGRRTDPSGDYVKRYLPKLKGFPSRYIYEPWNAPESVQKAAKCIIGVDYPKPM
VNHAETSRLNIERMKQIYQQLSRYRGLCLLASVPSCVEDLSGPVTESAPGQGSSTSTAIRLPQSDQASPKRKH
EGAEELCTEELYKRAKVTGLPAPEIPGKSS
>Odontophorus_guja__NXJ15161.1
DNWAFLYAQRLALKQELPLRVCFCLVPAFLDATIRHYSFMLRGLQEVAKECADLDIPFHLLLGCPKDKLPSFV
VEHNVGGLVTDFCPLRVPRQWVEEVKERLPEDVPFAQVDAHNIVPCWVTSSKQEYSARTIRAKIHNQLPEFLT
EFPPVIRHPHPPPNPPEPIAWDACYSSLQVDRTVTEVAWATPGTAAGLAMLQSFITKRLKSFSSQRNDPNKAA

LSNLSPWFHFGQVSTQRAILEVQKHRRAYKESVDAFVEEAVVRRELAENFCYYNENYDSVQGAYDWAQSTLKL
HAKDKRPFLYELPQLEQATTHDPLWNAAQLQMVQEGKMHGFLRMYWAKKILEWTRSPEEALQFAIYLNDRYEL
DGMDPNGYTDLPSPCLAGCLWSICGIHDQGWKERDVFGKIRYMNYAGCKRKFDVEQFERRYTHCK
>Odontotermes_form__QLJ82960.1
MNAGSSEKHTVHWFRKGLRLHDNPSLREGLKSSTTFRCVFILDPWFAGSSNVGINKWRFLLQCLEDLDQNLRK
LNSRLFVIRGQPADALPKLFKEWGTTNLTFEEDPEPFGWVRDQNITAMCKELGITVISRVSHTLYQLETIIKK
NGGKAPLTYHQFQTIVASMETPPPTEPTINQHFLNGTYTPIGEDHDEKYGVPTLEELGFDTDGLLPPVWKGGE
SEALARLERHLERKAWVASFGRPKMTPQSLLASQTGLSPYLRFGCLSTRLFYYQLTDLYKKIKKAYPPLSLHG
QLLWREFFYCAATKNPNFDKMSGNPICVQIPWDRNAEALAKWANGQTGFPWIDAIMTQLRQEGWIHHIARHAV
ACFLTRGDLWISWEEGMKVFEELLLDADWSVNAGMWMWLSCSSFFQQFFHCYCPVRFGRKADPNGDYIRKYLP
VLKNFPTRYIHEPWNAPEAVQKAAKCIIGKEYSLPMVNHAVASRINIERMKQVYQQLSKYRGPGLLATVPTSQ
SRTSNTLNPTLGQKDFQKSSETKWEALPGVDINGYSLVICDEDFKTGIQSYCYKSQNKEEYE
>Oedothorax_gibb__KAG8176361.1
MSNPHGNSIHWFRKCLRLHDNPAIVDAAKSGGRFWPIFILDPIIAKNMKVGPNRWRFLVQSLRDLDSSLQKFN
SRLFVLRGKPKEVFPDIVKRWQVKCLTFESDTEPYAIERDREIENLMKELGVKVVTHATNLLYNPEQIIEYNG
KVPLTYQSFVNTAEKIGLPPQPSSSVEALEKLMSEKKLNTPPIEDTHDVEYGVPTLEELGVNESQLNPCKYPG
GETEALKRLQKYLPKEEYICKFAKPDTSPNSIEPSTTVLSPYVKFGCLSARLFYHEIRKVYLKSKKGYTKPPV
SLHGQLFWREFYYTAGFATPNFDKMVGNEVCTQIPWIENKEFLEAWTHARTGYPFIDAIMTQLRNEGWIHHLA
RHAVACFLTRGDLWVSWEHGQKVFEELLLDADWSLNAGNWMWLSASAFFHQYYRVYSPIAFGKKTDKNGDFIR
KYVPVLKRFPPEYIYEPWKAPRKLQEQLGCVIGKDYPNRIVDHDKVRVENLRRMDAAYKAKKDAKGTSSSPKT
PSPKKSTPKGKRSLETKKDSKITKFLKSK
>Oedothorax_gibb__KAG8188597.1
MSGKKKKTEDSGKPAVKKQKVEDSPEADLEFVKNVEDARKATAKSIKDFKFNKKRVRVISDAQDVPESAKSIV
YWMSRDQRVQDNWAFLKAQHLALKLDLPLCVCFCLVPKFLDATIRHYRFMLKGLKEVSEECESLNIHFHLLFG
EAKDVLPKFVATNKVGGIVTDFCPLRVPLKWLSDVASKLPDDVPLVQVDAHNIVPCWVASDKQEYGARTIRKK
IHDKLKEFLTDFPPVIKQKISAECKSEPINWEEVESTLEVNMDVGDVSWATPGTNAGLLKISDFCRNRLKHFG
DLRNDPTRNYLSNLSPWFHFGQISIQRTILIVQKLKSKYSASVDAFVEEAVVRRELADNFCYYNKKYDKVEGA
YDWAKNTLKDHAKDKREYIYTLKQFENSETHDLLWNAAQRQMVKEGKMHGFLRMYWAKKILEWTKSPEEALEF
AIYLNDKYELDGRDPNGYVGCMWSICGIHDQGWAERAVFGKIRFMNYKGCQRKFDVNDFISKYKKMK
>Oedothorax_gibb__KAG8197157.1
MTSQRQKNVVHWFRKGLRLHDQPALKEGLSGADTWRCVFILDPWFAGTSNIGINKWRFLLQCLENLDNNLRKL
NSRLFVIRGQPADVFPRLFKLWNVTHLTIEKDPEPFGRIRDHNTTTMAMENGVIVISRTSHTLYRLDKIIQKN
GGNSPLTYKSFQNILATMESPPAPIPKITLEDIGNCFTPIGEDHEEKYGVPSLDELGFDTENLKPSVWIGGET
EALSRLERHLERKAWVASFGRPKMTPQSLLPSQTGLSPYLRFGCLSPRLFYQELTELYRKIKKVNPPLSLHGQ
LLMREFFYCAATNNPKFDRMVGNPMCVQIPWDHNSEALKRWSNGLTGFPWIDAIMTQLREEGWIHLVARHAVA
CFLTRGDLWISWEEGMKVFDELLLDADWSVNAGQWMWLSCSSFFQQFFHLFCPVRFGRKADPNGDYIRKYLPV
LRNFPAKYIHEPWTAPEKIQIVAKCIIGKDYVMPMVNHQEVSHINLERMKQVYQQLSHYKGTNTRNGTYPLGD
NKEEGNPKSAKRQCVEFPENHDDAENRC
>Oikopleura_dio_CBY34235.1_unnamed_pr
MSNVSQVCPQLTSLLPTTSQQSTAEREQVLLSKQAMSSPPQKRKKTAKSEDDGVKIALFIFRRDLRLIDNTSL
IQAIKDGYRILPVFVFPPEQIDPEKNEWFSHPSVQFMCESLNDLGRQLKEEVDGKLHLFMGGNLETIKKIKEV
VNFSAIAYNEDYSVYARERDGEIEEWAESNNVEIIKEEDYGLLPIKDAWHHPKDGPPKPYRVLSIFFKHIQKF
YDIRKPESNFEFEASQFTQSKFSFKFSISKLNTLYNANEKLAERGGRLVGLIGLEKIKNKKDYNETRNFMHLK
AGTTKMSAHLKFGTVSIREMYWRTAELFGETHDLIRQLVFRDFYLKIYSHNPELQRGTALQAGVDERIPWSDD
EKLWTAWCEGMTGFPLVDAAQRQLKEIGWQHNRMRMLTGCVLTKYFMIDWRKGMKFFYQNLVDADVFNNTAGW
GWVSSTGPDAVPYFRPPFNPWIQSKKFDLEGKYIKQYIPELADVSPADLHKWGDASIRAKYKKNKKVQAYPAP
VVKHDEASKNAVYLYKKAFEEMKAAQNA
>Oikopleura_dioica_CAG5112545.1_Oidioi.mRN
MERNITRHRTLHWFRKGLRLHDNRPLLEAIRSSEAVYCVFCLDLEWMRANERIGNNRIRFLLESLKDLDDSLR
KIGTRLFVLKGNARAAIKTFCREYEITQMTYMRDAEVFYRQLEAEILEEVNRREIVTRSFHGHTLYDPQEVID
ANDGQVPLSLDEFYSVVGNMDVPAPPAPTITGEMYKSCAVTIEDNHQAIYGVPTLASLGLECPRSLTRWPGGE
TVALERMRFKLERKNLFEFDQGPSTSKAGDHEYEIPGTTEPWFETQPETTGLSPYINLGSMSPRTFWHGAQGA
PEKMRTTVQGQMIYREFFYTVAYTVNNFTKIEGNRICRDIKWSDPKNERVAEWIEKFKEGMTGYPWIDAAVRQ
MKSEGWITHISRFSLASFLTVGQMWCSWEIGQQLFEEFLLDADYALNAGNFLWVTGSAFSNQVPLRAMDPVKI
ARKWDTDGSFIRRYCPELKNLPTQYIYCPWKAPNDVQQQARTIIGVDYPEPMLNAATTRIQNLNKIKALNQQL
RPDSDSDRDSL
>Onchidium_ree_QDK59985.1_cry_1-like
MKDNPTELSMSQTPRKHSLFWFRKGLRLHDNPALIAAIEGASTYRCVYILDPWFAGASQVGINKWRFLLESLE
DLDSSLRKLNSRLFVVRGQPADVLPRLFQEWGITTLAFEEDPEPYGKERDAAISAMAREFNIQVIAKSSHTLY
DPKIVIAANGNSPPLTYKRFQSILSTLEPPHQPCETLTAQMVADVTTPITDDHDDRFSVPSLDELGFDTDSLG

PAQFRGGESEALARLYRHLERKAWVASFERPKMSPQSLYPSGTGLSPYLRFGCLSPRTFYWKLTELYKKVKKG
ADPPLALHGQLLWREFFYTVATNNPNFDRMVGNSICVQIPWEHNPEALAKWAEGMTGYPWIDAIMVQLRKEGW
IHHLARHAVACFLTRGDLWISWEEGMKVFDEMLLDADWSVNAGMWMWLSCSAFFQQFFHCYCPVGFGKRADPS
GDFVRHYLPVLKGIPTQYIYEPWTAPESVQKAAKCIIGKDYPLPMVQHSEASRINLERMRQVYKRLVLKSSAS
KRIFLDLKILSVFPLSFHF
>Oncorhynchus_gorb__XP_046149037.1
MSTSRTVICLLRNDLRLHDNEVFHWAQRNAEHIVPLYCFDPGHYLGTANYNLPRTGPFRLRFLLDSIQDLRTS
LIQRGSTLVVRRGRPQEVVGDLIKQLGSVSAVAFHEEVVPEDQQVERGVRDICSQMKVKVHTCWGSTLYHRDD
LPFNHLSRLPDVYTQFRKAVETQGRVRPVLPTLDQLKPLPPGSGLDEGLIPSPEDLEQTDTVRDPRSAFPCSG
GETQALARLQHYFWDTDTVATYKETRNGLIGLDYSTKFSPWLAIGCISPRYIYQQIKKYESERTANQSTYWVV
FELLWRDYFKFVGAKYGDRLFDIKGLQDKSIPWKKDMNLFNAWKEGRTGVPFVDANMRELALTGFMSNRGRQN
VASFLTKDLGLDWRMGAEWFHYLLVDHDVSSNYGNWLYSAGIGNDPRENRKFNMIKQGLDYDNNGDYVRHWIP
ELKGIRGGEVHAPWTLSSASLSHARVSLNDTYPSPIVMAPEWSRHTNKKPTGAGPSARGKTGPSHTLKQHKDR
GIDFYFSKSKNF
>Oncorhynchus_gorb__XP_046160399.1
MAHTCIHWFRKGLRLHDNPALVAALRDCKEIYPVFVLDPCAPNNVKIGINRWKFLIGALKDLDCSLRKLNSRL
FVVRGKPEEVFPKLFQKWKVTRLTYEYDTEPFSLRRDKEVVRLAEEHGVEIIYKVSHTLYNIDRIIEENNGKA
PLTYNRLQTLVSSIGPPKRPIPAPTSDDMKDVKTPCSEKHEEHYGISTLEKLYQDPESLTEELFPGGEQEALL
RLDQHMERKEWVCGFEKPQTSPNSLSPSTTVLSPYMTFGCLSVRTFWWSLTDVYQGKKHSKPPVSLHGQLLWR
EFFYTAGLGIPNFDKMEGNPVCTQVDWDSNPEYLAAWAEARTGFPFIDAIMTQLRQEGWIHHLARHAVACFLT
RGDLWISWEEGQKVFEELLLDGDWSLNAGNWQWLSASTFFHQFFRVYSPIVFGKKTDKNGDYIKKYLPHLKKY
PAQYIYEPWKAPRSVQEAAGCIVGKDYPRPIVEHKVIRKKNIQRMKAAYAKRSPHSSDETPSKEKGLFPLSTA
IGSASPNLNHSIG
>Oncorhynchus_gorb__XP_046164461.1
MPVSGGEDPMSQVRQMLRELLVGRENAEGFFCLCVSVLGHNDTRTHFLPLIQLLATDHNRLHTTLTSIYLEYF
SKDEDDELAVALALSLLEAKGQQQTNTKPLFPDPKLQGQTDTQATATYRPQNSSIQSQSVSPNGSSSQPPPAP
LPMGASYAQLAAVGGRRQTQAQHQDSTSPRRSSGHGSSPQTERQRGPTQTQKQTDIKWSNVTQDVCASIATLT
SNLNQTKDDVTVVECKVDQSEKPKRSKNRRQRRKGYGQQVVGVPRCPSAPPPVLLWFRRDLRLYDNPAVIGSL
EAGGPVIPVFIWCPEEEEGPGVTVAMGGACKFWLHQALSCLSSALEHIGSHLVFLQADEEREGIGSSLLALRS
LVRETGAQTVLASALYEPWLRERDQVVVSALQKDRVEVNMVHSYCLRDPYTVTTEGVGLRGIGSVSHFMSCCQ
MNPGPGLGVPLDPPISLPSPSVWPRGCPLEGLGLARMPCRKDGTTIDWAANIRSSWDFSEEGAQSRLEAFLND
GVYRYEKESGRADAPNTSCLSPYLHFGQLSARWLLWDTKGARCRSPKFIRKLAWRDLAYWQLTLFPDLPWESL
RPPYKALRWSNERGHLKAWQKGRTGYPLVDAAMRQLWLTGWMNNYMRHVVASFLIAYLHLPWQEGYRWFQDTL
VDADVAIDAMMWQNGGMCGLDHWNFVMHPVDAAMTCDPYGSYVRKWCSELAVLPDDLIHKPWKCPASMLRRAG
VVLGQSYPERVVTDLEERRSQSLQDVALVRRRFGEYVDPCSGCDLVPLPPRLVSEAMGGGMVNTGGQFLLPVI
TRMEFKHQSDDPDADAASNPYNAVLKGYVSRRRNETIAFLNQTDFTASVINEGAERRERQEQDQRRMEGLPRP
LAVEQRGKRTPAAKDRFSTVPGRVATSHR
>Oncorhynchus_gorb__XP_046204321.1
MVGNTIHWFRKGLRLHDNPSLKESIRGADTLRCVYILDPWFAGSSNVGISRWRFLLQCLEDLDASLRKLNSRL
FVIRGQPTDVFPRLFQEWQTSQLSYEYDSEPFGKERDAAIQKLASEAGVEVTVKVSHTLYDLDKIIELNGGQS
PLTYKRFQALISHMDAVESPAEIITAEVMRKCATPISDDHDDKFAVPSLEELGFETEGLATAVWPGGETEALT
RLERHLERKAWVANFERPRMNATSLLASPTGLSPYLRFGCLSCRLFYFKLTDLYRKVKKNSSPPLSLYGQLLW
REFFYTTATNNPRFDKMEGNPVCVQIPWDRNPEALAKWAEGRTGFPWIDAIMTQLRLEGWIHHLARHAVACFL
TRGDLWISWEEGMKVFEELLLDADWSVNAGSWMWLSCSSFFQQFFHCYCPVGFGRRTDPNGDYIRRYLPILKA
FPAKYIFDPWNAPESVQKAAKCVIGMHYPKPMVHHTEASRLNIQRMTQIYQQLSCYRGLGLLATVPANPNNGG
NGVGTSPEEIQHEAVAQAGRGHAAVKRSSEDLTPGGSNKAQRQTSN
>Oncorhynchus_gorb__XP_046216371.1
MHRSLLLIPASKHIWKFVTLESQRHCSFTKLVGPSSINNRTTMPAEKRKATFVKMDNTKRQKLQAVKEGKRER
VEGWLQDVVAEQRKEKKDLKFNKQRLRFLSQTQKIKQGSEGVVYWMNRDHRVQDNWALIYAQLLALEEKLPLH
ICFCLVVPQNSELATIRHFGFMLRGLEEVAKDCSALDIQFHLLQGSAGELLPGFVADWSFGAVVTDFSPLRTP
LQWVEEVKKGLPSDIPFIQVDAHNVVPCWVASDKLEYSARTFRNKITKLLKEFLTEMPLVDKHPHAALRTTKP
VDWAEALSSLEVDSTVEEVDWAKSGTAAGMAMLESFIDLRLKNFATQRNNPNSSAVSQLSPWIRFGHLSAQRV
VLQVKCCGKSAGESVSSFTEELVVRRELTDNFCFYNPSYDSVKGANDWAQKTLKDHAKDPRPYVYTREQLEKS
QTHDKLWNAAQYQMVIEGKMHGFLRMYWAKKILEWTSSPEGALSIAIYLNDRYELDGQDPNGFVGCMWSICGV
HDQKGKXPIFGNYYMNSGGCTRKFDVAQFERKYCPKDL
>Opisthorchis_vi_OON18721.1
MAPQRAVLEVAALQYKYGRSVDVFIEEAFNRRELADNFCFHTPLYDRLQGARQWAQDTLHKHAGDKRDVAFTR
AQLESSGTTDDLWNAAQRQLVQTGKMHGFLRQYWAKKILEWCADGPEVAIQWAIYLNDRYSLDGTDPNGYVGI
MWAICGVHDQGWPERRIFGKVRYMCYNGCRRWFSVPTFVSRFSDA
>Opitutae_bac_HAE11581.1_DCG39_0805

MSTSVVWFRKCLRLHDNPALSHACSDPSIDSLLPLFILDPSVMGPSFENLGSARLRFLIESLDDLDHRLKSKY
GGKLFLARARPLAVFENLHRVHRSGIGLVSSDYCSEPSGRDESVAIQKFFGKHSPKTDVRFHPSTHTLCDLEQ
TIASPVYDNPKSMRQMESLASSIFGLDGSGFLQFPEPLPEPRGMPPHPEEIGIDSHRSSSFSFHTVRALKQEL
SSKGFLTPTVFTIPFVGGETEALARMEKKVSSEPAFVNAFRKPRTSSTNSPNDPIEPSTTGLSPYLSTGCLSV
RRIWKEVEKCNRQGAHSKPPESLHGQLLFREMFYLLSRSVENWDQDDGNPMCKPIDWGDRDEEKFLAWEEGRT
GFPYIDAMMRQLETTGWMHHLGRHAVSCFLTRGQLWHHWKHGRDVFGRKLLDSDWALNNANWLWLAGVAPFSM
PYFRLYNPCPDRESSLNVETSTAKFIKFWVPELRNIPPRFIYEPHLAPVSVQKEAHCLVGSDYPNPIVDRKKS
AKENLAKFKLSLTA
>Oppiella_nov_CAD7637253.1_unnamed_pr
MHDDSPIKLDFYLRQIKAIQQELASIQIPLIIHTVPLWENIATEIDKLCHKLDIENVHANIECGVNELNRDFQ
VQTQLNQSNRDLVLYHDRTLFPVGSIRNKSNQPYQVFGAFKKYCYEQLSISIPQCFPIPETQALNDAIDVGEL
NNNSLPTLAELGFIFNPNQINKQWQIGEQYALNVLDDFIENKVRHYQLERQTGIPIVDAGMRQLLHTGWMHNR
IRMVVAMFLTKNLLIDWRLGEQWFMQHLIDGDLAANNGGWQWCASTGTDSAPYFRIFNPISQSQKFDAQGDYI
RQWVPELAHLDAKTIHEPYAKNPNIELNYPKPIVDLKQSRIRAIEAFKQI
>Orbicella_fav_XP_020602470.1_cry_DASH-l
MLLQMIRNLALRGCRVFTEVHVGFFAVRRSLFHSSRANQHREAGVTMETKAGKSVIYFVRNDLRVHDNECLYW
AQQNGDFVIPLFCFDKNIFGHSAMTWHYKFAKTATHRAKFILESVSDLRETLKKHGSDLVVRSEQTAKEAVSE
VINLCRQNNEGHISLVYQREITKEETDVEAELLELCKTENVTVKCFWGLTLYHLEDLPFASVRSLPDTYTGFR
KNVETKCRVRPLIPAPDSLKSLPVFVANDHIGRIPTLKELVKDQSAETTDARTAFPFSGGETAALDRLNHYLW
DTNLVASYKETRNGLIGSDYSTKFSPWLAVGSLSPRKVFHCIKQYETECTANQSTYWVIFELLWRDYFKFVSF
KYGDSIFYLSGIMKKRGLPWKRDMPAFDQWRYGQTGVPFVDANMRELLHTGWMSNRGRQNVASFLVKDLGLDW
RMGAEWFESLLLDHDVSSNYGNWNYSAGIGNDPRENRKFNMVKQALDYDPDGDFVRMWVPELAGLKGAKVHTP
WMLSTLEVKHASVTLGENYPRPMVCPPEWNRHTSKVKDTAKWPRTQRRGVDFYFKSPKEASSSKKRH
>Orbicella_fav_XP_020607172.1_photolyase
MMGRKMRGLGKNQQWKRKVTCYVTKKNAGIHNKCCCMCCYSVARTFFVIAELSSSFRSFYLFSRRRSHQQLFR
NNFRFVCMASEPPAKRTKKEIAETSDSNGGDESILATYMAKRQKVCKSVVDFKFDKKRVRLITSNVDMPETCK
GIAYWMWREQRVQDNWALLYAQRMAIKQEVPLVVCFCLPSKYLDAAFRQYSFMITGLQEVEKELTELNIPFHM
LLGEPDKVLPDFIKSKEIGGVVTDFTPMRTPMKWLENTAKELPKTVPVCQVDGHNIVPCWQASPKLEYGARTI
RPKIQGQLKQFLTEFPPVIKHPYVAKDAAKTVDWKAVDEFIEVDRSVKEVEWAKPGTKAGLEMLETFCHERLK
YFATDRNNPTKSALSNLSPWFHAGHISAQRAILRVREFRNKSKESVEAFIEEAVVRRELADNFCFYNLDHYDS
IKGTNAWAQKTLQDHAQDKREYLYTREQLENGQTHDDLWNAAQLQMVNEGKMHGFLRMYWAKKILEWTASPEE
ALQVSLYLNDKFELDGNDPNGYVGCMWSVCGIHDQGWGERPVFGKIRYMNYNGCKRKFDVAEFVRKYGPGKDK
SSVASTKSVLNRKRKAK
>Orbicella_fav_XP_020612047.1_cry-1-like
MLSEDSAPSKSLPASVKTNLYSYEAWRFCSFDLLIFPFLGHRSLHWFRKDLRLHDNPSLRECLADSKVFYGVY
FLECSAEDKMTVSPNRWHFLLQSLQDLDRGLAQCGSRLFVLKGRPMDMLPSLFKEWGITRLSFEVDCEPFRKS
RDAVITGLAKQSGVEVISRVSHTLYDAEVLLMSTEGRAPQVFDEFMEVALQLGRPQMPAPRVNRQLFGSCLTP
VARDHNQQYGVPTVEEIGFQKPAATSSWFYPGGEQEALTRMEAALQKMKENDFCDSPLTANSLMPSASHLSPY
LRLGCLSSRLLYQRMTEEYIKNKTVNPPPEVYSKLFWREFFFLVGSQVPGAHEMVNNSLSLQIPWEDSPEYLH
KWKQGMTGFPWIDAIMRQLHLEGWIHDVARKAVGSFLTRGCLWISWEEGFKVFDELQLDAEWSLNVGNWLWLS
GSTFVKEHVPWFCPVDVGKKIDPTGEYVRKYVPEVRNLPTNYIFEPWMAPRDLQESCGCVIGQDYPAPLVDHR
QQRVVCVQRLRDLAFKLSAIK
>Orbicella_fav_XP_020614721.1_cry-1-like
IDSIAPNYLLALLHNLCKFSLSLSIIKNNGGTPPLTYKKFLAIIRSLGPPEHPVPTLDVQLLTGCSMPVSEDH
EEKFGVPSLEELGLDIKNIPVEVWHGGEKEALVRLDRHLERKAWIASFEKPKVTPNSLFPSPTGVSPYLRFGC
LSPRLFYHRLTELYRKVKLGEPPISLYGQLLWREFFFTVAANNPHFDRMATNPMCLQIPWRKSPERLQKWEEG
KTGFPWIDAIMVQLRQEGWIHHLARHAVGCFLTRGDLWISWEEGMKVFDRWLLDAEWSLNAGNWMWLSYSAFF
QQFFNCLCPVGFGRKLDPNGDYVRKYLPVLKGFPAKYIHAPWTAPESVQKAARCIIGQDYPAPIVDHNKVSRV
NLEKLRNIFKALLCYKDSFAVSSEKQDDVPDRQEKLKEQMFLLEDKENE
>Orbicella_fav_XP_020629231.1_cry-1-like
MAAKTQRVHHTIHWFRKGLRLHDNQSLREACETSLTLRPVYFIDPDYVKHGNMGFNRWRFLIQSLNDLDNNLK
SLGSRLFVIQGKPELELPRLFKAWRITRLTFEVDHEPDGRQRDSKICELASEAGIEVETRVCHSLYDLDKVIE
KNGGAAPLTYKKLCSVVSSMGPPLKPVPAVDEQLFDGCSTPVDDENEYKLPTLEELGIEMPEESSTVLFPGGE
TEALRRLDEHMEKEDWVCKFEKPNTSPNSLQPSTTVLSPYVMFGCLSARLFYHRLSEVYSRAKKHSSPPVSLH
GQLLWREFFFTAAYATPNFNRMEGNPVCLQVPWDTNPEFVKAWSEARTGYPFIDAIMTQLRREGWIHHLARHA
VACFLTRGDLWISWEEGLKVFEKYLLDHDWSLNAGNWMWLSASAFFHAYFRVYSPVAFGKKTDPNGDYIKKYI
PVLKKYPPKYIYEPWTAPQSVQKAAGCVIGKDYPRPIVDHKVVVKTNITRMKKARADHYGESIDEDLDNKSKS
SPTKRKNNKQSPTSSKLRKITDFTKK
>Orchesella_cin_ODM91467.1_cry_DASH,_

CLVWFRNDLRFHDNEALLWAHEHAEFVIPVFCFDPRQFETTTHRYKFPKTGNYRAQFLIQSVEDLQKTLKEKG
STLLVFNKRPEVTLSSIYDVCKYDAVVFQKEVTSEETEVEEACELFCTKNKIQFRPIWGSTLYHKDDLPYTVK
NCPNTYTQFRKDVEGRSKIRLEQPMPKLLKHLPPELTEISEELPSFEKLGLVEPTVHANTAFPFTGGETSALN
RLQSYLWATGAIENYKTTRNGLLGSEYSTKFSPWLAFGCISPRRVFFEIRKYEKERVKNDSTYWVIFELLWRD
YFRFIAIKFGNCLFFSGGVKKIRYKWEEDMEKFRKWADGKTGVPFVDANMRELLHTGWMSNRGRQNVASFLTK
DLKLDWRLGAEWFEYLLVDYDVCSNYGNWNYSAGIGNDPREDRKFNMIKQATDYDPEGAYARTWVPELGGIQG
GKIHVPWVLRPGELGSIKLGVDYPNPMIVAQEWRRHFDKTKSPGWTVSTKNKQKGIDFYFKSNTSSHK
>Orchesella_cin_ODN01631.1_cry-1
MESTESSSSKGVDKTVSVSILWFRHGLRLHDSPAFIAATSNRDHLFLPLFIFDGESAGTKIVGFNRMRFLIES
LEDLNNSFSTKYGGSLTVLRGQPVDLFRKLSQKWKIAKICFEQDCEPIWKERDDGVKEFCKENGIEVVECISH
TLYDPAEILSANGGNPPYTYSSFMFVASTLGKPERPLSAPNPETEAGKINFFGMTGDDDDLALCQLHFPMTPE
VFGVSKEKAVGENMRKGGETEGIILLEKRLAIEKQAFEQGLMLPNHTNPDFINGASLSPHLRFGCVSVRTFYW
AVQDLFSAIVSGNTTCETITGQLFWREYFYVMSVPNPNFDKMKSNPICLNIPWAKDVIAKENLKKWKAGKTGY
PFIDASMRQLIEQGWLHHVARNATACFLTRNDLWISWEKGLKHFLKYLLDADWSVCAGNWLWVSSSAFENVLD
CTLCICPTRYAQRLDPEAKFIKRFIPELQNYPIPYVFEPWTAPLELQNQWGCMIGKDYPAPIVNHEIVSKRNK
AEMVSLQKMLAGGGLNEHPCPSNTNEARNFMWFPEICSHESSP
>Orchesella_cin_ODN02944.1_photolyase
MAGKSKAPEKKASSSSSKGTAKEDDAPAEKKKKEVEDFDGFNARIREQRKSEGSSQTFKFDKSRCRMITEDEN
LPASHSGVLYWMSRDQRVQDNWALIYAQRLALKEKVPLFVCFCLVPKFLQATLRQYAFMLKGLRSVQKELVDL
NIPFSLLFGEAKAVLPDFVKKHKIGGVVTDFSPLRTPRAWVDEVAKKLPEDVPLCQVDAHNIVPVWVTSEKLE
YAARTIRPKIHKHLPDYLTEFPDVISHPHKSSSKFENPFKNWDELMAFLKIDKTVGEVEWAKPGTAEAFQTLR
SCDVSKLDFRSAGQRDDPDSGLTAVLEA
>Orchesella_cin_ODN04604.1_cry-1
MDAFIFRFLLQCLEDLDQSLRKVNSRLFVIRGQPADVLPKCFKEWGTTCLTFEEDPEPFGRVRDQNIMAQCKE
LNIDVSVKSSHTLYKLDVIIDKNGGKAPLTYRHFLNVVSYLDPPPQPEPTISLDFMKNSYTPVNINHDDKYGV
PSLEELGFDTDNLRDPCWRGGEMEALARLERHLERKAYVATFGRPKMTPQSLMASQTGVSPYLRFGCLSPRLF
YYQLDDLYRKIKNAEPPLSLHGQVLWREFFYCAATKNPNFDRMVGNPICVQIPWDKNSKALAKWANAKTGFPW
IDAIMTQLREEGWIHHIARHAVACFLTRGDLWISWEEGMKVFEELLLDTDWSVNAGMWMWLSCSSFFQQFIHC
YCPVRFGRKADPNGDFIRKYLPVLKMLPTQYIHEPWMAPEAVQKASKCIIGKDYPMPMINHAVAYRINIERMK
QVYRSLPKYRPTDIHSFVQGTNNAGTPAPQMPIPLPEPDANFTTLDDSMINTAAANMDMNCDLSTKSLHDLLE
SAINDTFGTVGTLGS
>Orchesella_cin_ODN05388.1_cry-2
MGTKSPPVVLHWFRKGLRLHDNPALERAVKFAKDNQAVVRPVFILDTELPKSYKIGANRYRFLVESLEDLDQS
FRSLGTRLFVIRGKVQDVLENCFKTWNVKLITFENDTEPYSRARDTEFYKLAKKHGIPFEGFWSHTLYEPILL
IKTNKGQTPKTYQKFIDVMTKTGQPPKSISTITSIPAHFKDPLLSSSTYDTPTLTELKVNAEELGPHLFKGGE
TEALQRMDRSLANKHYVENFAKPNTSPNSIKPSTTVLSPYLKFGCLSCRLFYEKLQTFKGTKTKPPVSLLGQL
YWREFYYLVGAQTPNFIQMKGNSICKQIPWDDNPEYVQAWKEGRTGYPFIDAIMTQLRTEGWIHHLARHAVAC
FLTRGDLWQHWEKGQDVFEEYLLDADVYLNAGNWMWLSASAFFHQYYRVYSPIKFGQKTDTSGDYIRKYIPVL
AKYPPEYIYEPWKAPQSVQKAAGCIVGRDYPKRIVEHETIVKINLTKMNKAYAKPTSQPSPSKSDSKASLKRT
ADTMDSDDDYADEVHPANTTSTKLPRNSNSNTSTSKRQMSILDAFKAKH
>Orcinus_orca__XP_004264088.1
MAAAVATSAVAVPAPAARAEGASSVHWFRKGLRLHDNPALQAAVRGAHCMRCVYILDPWFAASSSVGINRWRF
LLQSLEDLDISLRKLNSRLFVVRGQPADVFPRLFKEWGVTRLTFEYDSEPFGKERDAAIMKMAKEAGVEVVTE
NSHTLYDLDKIIELNGQKPPLTYKRFQAIISRMELPKKPVGSVTSQQMEGCRAEIQENHDETYGVPSLEELGF
PTEGLGPAVWQGGETEALARLDKHLERKAWVANYERPRMNANSLLASPTGLSPYLRFGCLSCRLFYYRLWDLY
KKVKRSSTPPLSLFGQLLWREFFYTAATNNPRFDRMEGNPICIQIPWDRNPEALAKWAEGKTGFPWIDAIMTQ
LRQEGWIHHLARHAVACFLTRGDLWVSWESGVRVFDELLLDADFSVNAGSWMWLSCSAFFQQFFHCYCPVGFG
RRTDPSGDYIRRYLPKLKGFPSRYIYEPWNAPESIQKAAKCIIGVDYPRPIVNHAETSRLNIERMKQIYQQLS
RYRGLCLLASVPSCMEDLSNPVAEPSSSQAGGMSSAGPRPLPSGPASPKRKLGAAEEPPGEELSKRARVAGLP
PSELLSKDV
>Orcinus_orca__XP_004269459.1
MGVNAVHWFRKGLRLHDNPALKECIQGADTIRCVYILDPWFAGSSNVGINRWRFLLQCLEDLDANLRKLNSRL
FVIRGQPADVFPRLFKEWNITKLSIEYDSEPFGKERDAAIKKLATEAGVEVIVRISHTLYDLDKIIELNGGQP
PLTYKRFQTLISKMEPLEIPVETITSEVIEKCTTPLSDDHDEKYGVPSLEELGFDTDGLSSAVWPGGETEALT
RLERHLERKAWVANFERPRMNANSLLASPTGLSPYLRFGCLSCRLFYFKLTDLYKKVKKNSSPPLSLYGQLLW
REFFYTAATNNPRFDKMEGNPICVQIPWDKNPEALAKWAEGRTGFPWIDAIMTQLRQEGWIHHLARHAVACFL
TRGDLWISWEEGMKVFEELLLDADWSINAGSWMWLSCSSFFQQFFHCYCPVGFGRRTDPNGDYIRRYLPVLRG
FPAKYIYDPWNAPEGIQKVAKCLIGVNYPKPMVNHAEASRLNIERMKQIYQQLSRYRGLGLLASVPSNPNGNG
GLMGYSPGENIPGYSSSGSCTPGSGILHYAYGDSQQTHLLKQGRSSTCTGLSSGKRPSQEEDTQSIGPKVQRQ
STN

>Ordospora_col_TBU15134.1_photolyase
MDGRVEKKNEYMNKQNVLYMMRRDQRIHENYAVQLGYELSYEYRSQLYFGVEFFTLKMNARQRLFVLEGLAEL
SKEARKYNIELFIIKDLMAFVKTYTIGCIILDFCALRESILREQEIYRMCEEMKIELYVCDAHNIVPCKMLSK
YKRTPKAVRTDLLKFFWQYLVAFNKVEVHKINKIDKEVKKEIDEEVKKEIEIDFEDARTIRKFVSATEIEGYL
SRNKSNLERRNSISGGYSHGMKVMKNFFEGGRFKSFKSYRNDPDVDGNSGLSPWINFGQISAQEVVFFGCERF
EKTNENLETFINEAFVWREIAEHFVLHESNYDNIEGALPWARESLLEHACDKREIVFSQEQLESARTGYALWD
AAQCQLMYSGRIHGYVRMYWAKQLMWWTEDPRSALNIALYFNDTYAIDGNSPNGYLGVMWSICGSMDRAFKER
EVVGKIRTMKAFKAPVYINRWREY
>Oreotrochilus_mela__NXU71630.1
RFLLQCLEDLDANLRKLNSRLFVIRGQPADVFPRLFKEWNIAKLSIEYDSEPFGKERDAAIKKLASEAGVEVI
VRISHTLYDLDKIIELNGGQPPLTYKRFQTLISRMEPLEMPVETITPEVMEKCTTPVSDDHDEKYGVPSLEEL
GFDTDGLPSAVWPGGETEALTRLERHLERKAWVANFERPRMNANSLLASPTGLSPYLRFGCLSCRLFYFKLTD
LYKKVKKNSSPPLSLYGQLLWREFFYTAATNNPRFDKMEGNPICVQIPWDKNPEALAKWAEGRTGFPWIDAIM
TQLRQEGWIHHLARHAVACFLTRGDLWISWEEGMKVFEELLLDADWSVNAGSWMWLSCSSFFQQFFHCYCPVG
FGRRTDPNGDYIRRYLPVLRGFPAKYIYDPWNAPESVQKAAKCIIGVNYPKPMVNHAEASRLNIERMKQIYQQ
LSRYRGLGLLATVPSNPNGNGNGGLMGYSPGECVSGCGSTGGAQLGTGDGHTVVQPCALGESHTGASGIQQQG
YCQASSILHYAHGDNQQSHLLQAGRTTLGTGISAGKRPNPEEETRGVGPKVQRQSTS
>Oreotrochilus_mela__NXU79702.1
TRGSTTLLTVFSLSVPKVDYDVCSNYGNWLYSAGVGNDPREHRKFNMVKQGLDYDGKGDYVRLWVPELQSIKG
ADIHTPWALNTAALSQAGVTLGETYPQPVVTAPEWSRHINQKPQGRSPHPRGKRGPAHTPRQHRDRGIDFYFS
HKKDL
>Oreotrochilus_mela__NXU80590.1
DNWAFLYAQRLALKQELPLHVCFCLVPKFLDATIRHYGFMLRGLQEVTKECAELNIPFHLLLGYAKDVLPAFV
VEHGVGGLVTDFCPLRLPRQWVEDVKERLPEDVPFAQVDAHNIVPCWVTSPKQEYSARTIRGKIHAQLPEFLT
EFPPVIPHPYSPSCPAEPIAWEACYSSLQVDRTVKEVEWATPGTAAGMAVLKSFITERLKSFSSHRNDPNKAA
LSNLSPWFHFGQVSTQRAILEVQKHRRTYKESVDAFVEEAVVRRELAENFCYYNENYDSVQGAYDWAQTTLNL
HAKDKRPFLYKLQELEQGTTHDPLWNAAQLQMVQEGKMHGFLRMYWAKKILEWTRSPEEALQFAIYLNDRYEL
DGRDPNGYVGCLWSICGIHDQGWAERAIFGKIRYMNYAGCKRKFDVGEFERRYAP
>Oreotrochilus_mela__NXU81457.1
RFLLQSLEDLDNSLRKMNSRLFVVRGQPTDVFPRLFKEWGVTRLTFEYDSEPFGKERDAAIIKLAKEAGVEVV
IENSHTLYDLDRIIELNGHKPPLTYKRFQAIISRMELPKKPVSTIMSQQMETCKVDIQENHDDVYGVPSLEEL
GFPTEGLGPAVWHGGETEALARLDKHLERKAWVANYERPRMNANSLLASPTGLSPYLRFGCLSCRLFYYRLWE
LYKKVKRNSTPPLSLYGQLLWREFFYTAATNNPKFDRMEGNPICIQIPWDRNPEALAKWAEGKTGFPWIDAIM
TQLRQEGWIHHLARHAVACFLTRGDLWISWESGVRVFDELLLDADFSVNAGSWMWLSCSAFFQQFFHCYCPVG
FGRRTDPSGDYVKRYLPKLKGFPSRYIYEPWNAPEAVQKAAKCIIGVDYPKPMVNHAETSRLNIERMKQIYQQ
LSRYRGLCLLASVPSCVEDLGGPVTDSASGQGCSTSTAVRLLQADPASPKRKHEGAEELYSEELYKRAKMTGL
PAPEIPDKSS
>Ornithorhynchus_anatinus_XP_007666228.2_cryptochro
MAAVTVAGAAGPAPAAAAAGAAASSVHWFRKGLRLHDNPALQAALRGARSVRCVYILDPWFAASSSVGINRWR
FLLQSLEDLDTSLRKLNSRLFVVRGQPTDVFPRLFKEWGVTRLTFEYDSEPFGKERDAAIVKMAKEAGVEVVT
ENSHTLYDLDRIIELNGQKPPLTYKRFQAIISRMELPKKPVSCVTSQQMEGCKAEIQDNHDDTYGVPSLEELG
FPTDGLGPAVWQGGETEALARLDKHLERKAWVANYERPRMNANSLLASPTGLSPYLRFGCLSCRLFYYRLWDL
YKKVKRNSTPPLSLYGQLLWREFFYTAATNNPRFDRMEGNPICIQIPWDRNPEALAKWAEGKTGFPWIDAIMT
QLRQEGWIHHLARHAVACFLTRGDLWVSWESGVRVFDELLLDADFSVNAGSWMWLSCSAFFQQFFHCYCPVGF
GRRTDPSGDFIRRYLPKLKAFPSRYIYEPWNAPESVQKAAKCVIGVDYPRPIVNHAETSRLNIERMKQIYQQL
SRYRGLCLLASVPSCVEDLSSAAAESGLGQASGSNISTAPRPPPPPYGPASPKRKLEAAEGLPGEELCKRPKV
AGRPGPEPPGEDA
>Ornithorhynchus_ana_XP_028932146.1_photolyase
MAPKKRRANASKIQVSEEDDVGVQRETEEKGTTEKLTQTGGPRKRLTNVATVAQAEEKGSRKAEEGGKGEVGS
LQDAVPQARLRTAPSALAFRYDERRVRFISQGCHLKEDAKGVVYWMSRDQRVQDNWAFLYAQRLALQRKLPLH
VCFCLVPSFLGATIRHFGFMLGGLKEVAKECKQLDIPFHLLPGLAKDTLPTFVRDHDIGAVVTDFSPLRLPLQ
WVRDVRDGLPEVVPLVQVDAHNIVPCWVASDKQEYAARTIRRKIHDRLSNFLTEFPPLVRHPHPADFQAEPVD
WQACRAGLQVDRTVGEVSWAQPGTAAGLTVLQTFIDQRLRIFSSSRNDPNKAALSNLSPWFHFGQVSVQRAIL
EVRKQRHRYPDSVASFVEEAMVRRELADNFCFYNENYDKVEGAYDWARTTLRLHAKDKRTHLYSLEQLEAGKT
HDTLWNAAQLQMVQEGKMHGFLRMYWAKKILEWTRSPEEALEFAIHLNDRFQLDGRDPNGYVGCMWSICGIHD
QGWAEREVFGKIRYMNYAGCKRKFDVAQFEHKYSPRE
>Ornithorhynchus_ana_XP_028935073.1_cry-1
MGVNAVHWFRKGLRLHDNPALRDCVRGADTVRCVYILDPWFAGSSNVGINRWRFLLQCLEDLDANLRKLNSRL
FVIRGQPADVFPRLFKEWNIAKLSIEYDSEPFGKERDAAIKKLASEAGVEVIVRISHTLYELDKIIELNGGQP
PLTYKRFQTLISRMDPLAMPVETITAEVMGKCMTPLSDDHDEKYGVPSLEELGFDTDGLPSAVWPGGETEALT

RLERHLERKAWVANFERPRMNANSLLASPTGLSPYLRFGCLSCRLFYFKLTDLYKKVKKNSSPPLSLYGQLLW
REFFYTAATSNPRFDKMEGNPICVQIPWDRNPEALAKWAEGRTGFPWIDAIMTQLRQEGWIHHLARHAVACFL
TRGDLWISWEEGMKVFEELLLDADWSVNAGSWMWLSCSSFFQQFFHCYCPVGFGRRTDPNGDYIRRYLPVLRG
FPAKYIYDPWNAPEGIQKAAKCIIGVNYPKPMVNHAEASRLNIERMKQIYQQLSRYRGLGLLASVPSNPNANG
SGGLMAYSPGENIPGCSSGGGVQMGASESHLLQTCVLGESHLGPSGIQQQGRSSLTPGLSGGKRPCQEEESQS
IGPKVQRQSTD
>Orussus_abi_XP_012272812.1_photolyase
MTYKMNEKPSIKKQKTSTLQTIILNNRKNAAASVMTFKFDKKRIRIITNIKEVTKKCRGILYWMFRDARVQDN
WAFLFAQKIAIKNRVPLHVCFCILSKFLSSTTRQYRFLLKGLNEVAIECNQLNINFHLLHGEPSLAIAEFISK
YNIGGVVTDFSPLRLPIRWINDLKGMIPCEIPICQVDAHNIIPCWITSNKLEYSARTIRNKINSQIDQFLTEF
PPVIKHPYKASEQFEDINWETALNDVQAESIAELTWLTPGYKAGINQFETFLMDRLKDYYLKRNDPVENVTSN
LSPWFHFGMISVQRCIIEVQKYKCKFKQSVEAFMEEAIIRRELSDNFCFYNENYDNLQGANKWAIDTLNFHRN
DKREYIYDLHELEASLTHDDLWNSAQNQLMCMGKMHGFLRMYWSKKILEWTRTPEQALEWSIYLNDKYSIDGC
DPNGYVGCMWSICGIHDQGWKERNVFGKIRYMNYKGCQRKFNVAKFVADWGGKVHSKRKKKNA
>Orussus_abi_XP_012284945.1_cry-1_X1
MTGSLSELDQGPPTRGGRGKHTVHWFRKGLRLHDNPSLRDGLLGASTFRCVFVLDPWFAGSTNVGINKWRFLL
QCLEDLDCSLRKLNSRLFVIRGQPADALPKLFKEWGTTNLTFEEDPEPFGRVRDHNISTLCKELGISVVQRVS
HTLYKLDKIIDRNGGQSPLTYHQFQNIVASMEPPEPPVPTVTSACIGGAYTPLEDDHDDVYGVPTLEELGFSR
PGATGAQWNDKKSRCRPDGRSHYKRRFPGFDTEGLLPPVWVGGESEALARLERHLERKAWVASFGRPKMTPQS
LLPSQTGLSPFLRFGCLSTRLFYYQLTDLYKKIKKTAPPLSLHGQLLWREFFYCAATKNPNFDRMQGNPICVQ
IPWDKNGEALAKWANGQTGFPWIDAIMTQLREEGWIHPLARHAVACFLTRGDLWISWEEGMKVFDELLFDADW
SVNAGMWMWLSCSSFFQQFFHCYCPVRFGRKADPNGDYIRRYLPVLKNFPTRYIHEPWNAPLSVQRTAKCIIG
KEYSRPMVNHSKTSRINIERMKQVYQQLNKYRGTVLLPEAHLSSRGCSHPEGNQEESPTSLEASWKVDGNATC
R
>Orycteropus_afer_afer_XP_007944720.1
MAAAMATAAAAASAPTAEGASSVHWFRKGLRLHDNPALLEAVRGARCVRCVYILDPWFAASSSVGINRWRFLL
QSLEDLDTSLRTLNSRLFVVRGQPADVFPRLFKEWGVTRLTFEYDSEPFGKERDAAIVKMAKEAGVEVVTENS
HTLYDLDRIIELNGQKPPLTYKRFQAIISRMELPKKPVGSVTSQQMESCRAEIQENHDETYGVPSLEELGFPT
EGLGPAVWQGGETEALARLDKHLERKAWVANYERPRMNANSLLASPTGLSPYLRFGCLSCRLFYYRLWDLYKK
VKRNSIPPLSLFGQLLWREFFYTAATNNPRFDRMEGNPICIQIPWDRNPEALAKWAEGKTGFPWIDAIMTQLR
QEGWIHHLARHAVACFLTRGDLWVSWESGVRVFDELLLDADFSVNAGSWMWLSCSAFFQQFFHCYCPVGFGRR
TDPSGDYIRRYLPKLKGFPSRYIYEPWNAPESVQKAAKCIIGVDYPRPIVNHAETSRLNIERMKQIYQQLSRY
RGLCLLASVPSCVEDLSNPVAEPSSSQAGNLSSAGPRLLPGGPASPKRKLEAAEEPPGEELSKRARVAKLPSP
ELPSKDV
>Orycteropus_afer_afer_XP_007946421.1
MGVNAVHWFRKGLRLHDNPALKECIQGADTIRCVYILDPWFAGSSNVGINRWRFLLQCLEDLDANLRKLNSRL
FVIRGQPADVFPRLFKEWDITKLSIEYDSEPFGKERDAAIKKLATEAGVEVIVRISHTLYDLDKIIELNGGQP
PLTYKRFQTLISKMEPLEIPVETITSEVTEKCTTPLSDDHDEKYGVPSLEELGFDTDGLPSAVWPGGETEALT
RLERHLERKAWVANFERPRMNANSLLASPTGLSPYLRFGCLSCRLFYFKLTDLYKKVKKNSSPPLSLYGQLLW
REFFYTAATNNPRFDKMEGNPICVQIPWDKNPEALAKWAEGRTGFPWIDAIMTQLRQEGWIHHLARHAVACFL
TRGDLWISWEEGMKVFEELLLDADWSINAGSWMWLSCSSFFQQFFHCYCPVGFGRRTDPNGDYIRRYLPVLRG
FPAKYIYDPWNAPEGIQKVAKCLIGINYPKPMVNHAEASRLNIERMKQIYQQLSRYRGLGLLASVPSNPNGNG
GLMSYSPGENVPGCSSSASCSQGNGILHYAHGDSQQAHLLQQGRSSVGTGLSSGKRPSQEEETQSVGPKVQRQ
STN
>Oryctolagus_cuni__XP_002711467.1
MGVNAVHWFRKGLRLHDNPALKECIQGADTIRCVYILDPWFAGSSNVGINRWRFLLQCLEDLDANLRKLNSRL
FVIRGQPADVFPRLFKEWNITRLSIEYDSEPFGKERDAAIKKLASEAGVEVIVRISHTLYDLDKIIELNGGQP
PLTYKRFQTLISKMEPLEIPVETITSDVIEKCTTPVSDDHDEKYGVPSLEELGFDTDGLSSAVWPGGETEALT
RLERHLERKAWVANFERPRMNANSLLASPTGLSPYLRFGCLSCRLFYFKLTDLYKKVKKNSSPPLSLYGQLLW
REFFYTAATNNPRFDKMEGNPICVQIPWDKNPEALAKWAEGQTGFPWIDAIMTQLRQEGWIHHLARHAVACFL
TRGDLWISWEEGMKVFEELLLDADWSINAGSWMWLSCSSFFQQFFHCYCPVGFGRRTDPNGDYIRRYLPVLRG
FPAKYIYDPWNAPEGIQKVAKCLIGINYPKPMVNHAEASRLNIERMKQIYQQLSRYRGLGLLASVPSNPNGNG
GLMGYSPGENIPGCSSSGSCSQGSGILHYAQGDTQQTQLLKQGRSSMGTGLSSGKRPSQEEDTQSIGPKVQRQ
STN
>Oryctolagus_cuni__XP_008268424.1
MAAAAAVGPAAAAVPAPAASANGASSVHWFRKGLRLHDNPALLAAVRGARCVRCVYILDPWFAASSSVGINRW
RFLLQSLEDLDTSLRKLNSRLFVVRGQPTDVFPRLFKEWGVTRLTFEYDSEPFGKERDAAIMKMAKEAGVEVV
TENSHTLYDLDRIIELNGQKPPLTYKRFQAIISRMELPKKPAGSVTSQQLEGCRAEIQDNHDETYGVPSLEEL
GFPTEGLSPAVWQGGETEALARLDKHLERKAWVANYERPRMNANSLLASPTGLSPYLRFGCLSCRLFYYRLWD
LYKKVKRNSTPPLSLFGQLLWREFFYTAATNNPRFDRMEGNPICIQIPWDRNPEALAKWAEGKTGFPWIDAIM

TQLRQEGWIHHLARHAVACFLTRGDLWVSWESGVRVFDELLLDADFSVNAGSWMWLSCSAFFQQFFHCYCPVG
FGRRTDPSGDYVRRYLPKLKGFPSRYIYEPWNAPESVQKAAKCIIGVDYPRPIVNHAETSRLNIERMKQIYQQ
LSRYRGLCLLASVPSCVEDLSHPVAEPSSSQAGSVSGAAPRPLPSGPASPKRKLEAAEEPPGEELSKRARVAE
VPTPELPSKAV
>Oryza_sat_bluelightP_XP_015630924.1
MAAAASDSDSPAAAAARHHDDPTLPAFASFSLSLSLRTPASPSPAALASVPSTIHLPTQISTLAVCLHPSASA
SASPSSRRRLNAAAAASSLLAPLPASTPGLSRSFPSGAPAAAGRRRTLVWFRADLRLHDHEPLHAAVGASSSL
LPVFVFDPRDFGKSPSGFDRTGPYRAGFLLDSVADLRRGLRARGGDLVVRVGRPEVVIPELARAAGAEAVFAH
GEVSRDECRAEEKVSKAVEKEGIEVKYFWGSTLYHLDDLPFRLEDMPSNYGGFREAVKGLDVRKVLDAPEEVK
CVPMKNVLEPGEIPTLAELGLTAPPAMAHGSKAAVGSTLIGGEAEALERLKKFASECCMQPNKGDKDSTRDSI
YGANFSCKISPWLATGCLSPRFMYEELKKHASRAIPSGSTPKNGDGTSDAGTNWLMFELLWRDFFRFITKKYS
SAQKTSEVAPATGCTPTPALA
>Oryza_sativa_BAC78798.1_cryptochrome
MMAAEGIIVQSFNADLLYEPWEVVDDEGQSFTMFAPFWNRCLSMPYDPAAPLLPPKRINSGDLSMCPSDDLIF
EDDSEEGSNALLARAWSPGWQNRDKALTAFLNGPLIHYSVNRKKADSASTSLLSPYLHFGELSVRKVFHLVRM
KQLVWSNEGNRAAEESCTLFLRSIGLREYSRYLSFNHPCSHEKPLLAHLRFFPWVINECYFKIWRQGRTGYPL
VDAGMRELWATGWLHDRIRVVVSSFFVKVLQLPWRWGMKYFWDTLLDADLESDALGWQYISGSLPDGRELDRI
DNPQLEGYKFDPHGEYVRRWLPELARLPTEWIHHPWDAPASVLQAAGVELGSNYPLPIVGLDRANARLQEALS
EMWQLEAASRAAMDNGMEEGLGDSSEVPPIEFPRELQMEVDREPARVTANVLTTARRREDQMVPTMTSSLNRA
ETEISADFMNSVDSRAEVPTRVNFEPRTEREENFRTTAGNVARTNGIHEHNNFQQPQHRMRNVLAPSVSEASS
GWTGREGGVVPVWSPPAASDHSETFASDEADISSRSYLDRHPQSHRLMNWSQLSQSL
>Oryza_sat_XP_015614933.1_photolyase
MPPTSVSPPRTAPGPANPSPAHPSRVRVIHPGGGKPGGPVVYWMLRDQRLADNWALLHAAGLAAASASPLAVA
FALFPRPFLLSARRRQLGFLLRGLRRLAADAAARHLPFFLFTGGPAEIPALVRRLGASTLVADFSPLRPVREA
LDAVVGDLRREAPGVAVHQVDAHNVVPVWTASAKMEYSAKTFRGKVSKVMDEYLVEFPELPAVVPWDREQPEG
VDWDALIARVCSEAENVPEIDWCEPGEEAAIEALLGSKDGFLTKRIKSYETDRNDPTKPRALSGLSPYLHFGH
ISAQRCALEAKKCRHLSPKSVDAFLEELVVRRELADNFCYYQPQYDSLSGAWEWARKTLMDHAADKREHIYTR
EQLENAKTHDPLWNASQLEMVHHGKMHGFMRMYWAKKILEWTSGPEEALSTAIYLNDKYEIDGRDPSGYVGCM
WSICGLHDQGWKERPVFGKIRYMNYAGCKRKFDVDAYISYVKRLAGQSKKRNAEESPNPVVKLSKSQH
>Oryza_sat_XP_015623929.1_(6-4)DNA_p
MDAAATAATATAAAAMVWFRKGLRVHDNPALDAARRGGAAARLYPVFVLDPRYLRPDQAAPSPGSARAGVARV
RFLLESLSDLDARLRRLGSRLLLLRARDDGDVAGTVCAALKDWNIGKLCFESDTEPYALARDKKVMDFAAASG
IDVFSPVSHTLFDPAEIIEKNGGRPPMTYQSFVAIAGEPPEPIMEEYSELPPVGDTGEYELLPVPRVEELGYG
DISQEDLSLFRGGETEALKRMRESLHDKEWVAKFEKPKGDPSAFLKPATTVLSPYLKFGCLSSRYFYHCIQDI
YRSTKKHTNPPVSLTGQLLWRDFFYTVAFGTPNFDQMKGNKICKQIPWTENEELFPAWRDGRTGYPWIDAIMI
QLRKWGWMHHLARHSVACFLTRGDLFIHWEKGRDVFERLLIDSDWAINNGNWMWLSCSSFFYQYHRIYSPTSF
GKKYDPNGNYIRHFIPVLKDMPKEYIYEPWTAPLSIQKKANCIIGKDYPKPVVDHAIASKECKKMMGEAYASN
RLDDDKPDKGKSSNSSRRKLSAGSQVTPNSSKTKQLKRSS
>Oryza_sat_XP_015625483.1_cry-1_X1
MAGSERTVVVWFRRDLRIDDNPALASAARDGAVLPVFIWCPADEGQFYPGRCSRWWLKQSLPHLSQSLESLGCP
LVLIRAESTLEALLRCIDSVGATRLVYNHLYDPVSLVRDDKIKKELSALGISIQSFNGDLLYEPWEIYDDSGL
AFTTFNMYWEKCMELPIDASPSLAPWKLVPVPGLESVRSCSVDDLGLESSKDEESSNALLMRAWSPGWRNAEK
MLEEFVSHGLLEYSKHGMKVEGATTSLLSPYLHFGEVSVRKVYQLVRMQQIKWENEGTSEAEESIHFFMRSIG
LREYSRYLCFNFPFTHEKSLLGNLKHYPWKVDEERFKSWRQGMTGYPLVDAGMRELWATGWTHNRIRVIISSF
AVKFLLIPWTWGMKYFWDVLLDADLESDILGWQYISGSLPDGHELSRLDNPEVQGQKYDPDGVYVRTWIPELA
RMPTEWIHHPWDAPSCILEVAGVELGFNYPKPIVDLHIARECLDDSISTMWQLDTAEKLAELDGEVVEDNLSN
IKTFDIPKVVLRETSPCALPIDQRVPHASSKDHNLKSKVLKASNRSSICVDMIRSSKMEATSSVANSPVSRKR
SFCETAFHVPSYSSSAEVHSHIQDHGGSLVGPSRYLLQEAGRNYVDEVEDSSTADSGSSISRQRKAA
>Oryza_sat_XP_015644118.1_cry_DASH,_
MLHFLSSSSPLNPQFLLLPRQSARLRVLLSIPVSAMSSSSSSSSRGALAAAAVPSLSADEAGAAADEAFLRYT
SPSMRRSGGGGVAIVWFRNDLRVLDNEAVVRAWAASDAVLPVYCVDPRISAGSTHYFGFPKTGALRAQFLIEC
LEDLKRNLTKQGLDLLIRHGKPEDILPSIAKAVTAHTVYAHKETCSEELLVEHLVRKGLEQVVIPQGGASNQK
KPRNPKLQLIWGATLYHVDDLPFSVNNLPDVYTQFRKAVESKSSVRNCSKLPPSLGPPPGSGLDEIGGWGTVP
TLESLGLSMTKAEKGMHFVGGESAALGRVHEYFWKKDQLKVYKETRNGMLGPDYSTKFSPWLASGSLSPRYIC
EEVKRYEKQRIANDSTYWVLFELIWRDYFRFISAKYGNSIFHLGGPRNVESKWSQDQALFESWRDGRTGYPLI
DANMKELLATGFMSNRGRQIVCSFLVRDMGIDWRMGAEWFETCLLDYDPASNYGNWTYGAGVGNDPREDRYFS
IPKQAKTYDPDGEYVAYWLPELRSIAKERRNFPGASYIKQVVPLKFDGGHQKRDQQFNRQRRPGHMYRRQK
>Oryzias_latipes_XP_004070204.1_cryptochro
MVVNSVHWFRKGLRLHDNPALQEALNGADTVRCVYILDPWFAGAANVGINRWRFLLEALEDLDCSLKKLNSRL
FVVRGQPTDVFPRLFKEWNVTRLTFEYDPEPYGKERDGAIIKMAQEFGVETVVRNSHTLYNLDRIIELNNNSP

PLTFKRFQTIVSRLELPRRPLPAITQQQMDSCQRQIGDNHDQLYSIPSLEELGFRTERLPPAVWRGGESEALD
RLSKHLDKKVWVASVEHTRVNKCSLYASPTGLSPYLRFGCLSCRVLYYNLRELYMKLRKRCSPPLSLFGQLLW
REFFYTAATNNPNFDRMEGNPICVQIPWDQNPEALAKWAEGRTGFPWIDAIMTQLRQEGWIHHLARHAVACFL
TRGDLWISWVSGMKVFEELLLDAGWSVNAGSWMWLSCSAFFQQFFHCYCPVGFGRRTDPSGDYIRRYIPILKD
FPNRYIYEPWNAPESVQKAANCVVGVDYPKPMVNHAESSRLNIERMKQVYQQLSHYRGLSLLASVPTIQEEAE
PPMTDESQASSGPDSPHRDPADDEAAGSSTFPDSSTSAAPQDKEDSAHCYHAHQVSAHSPCTSSDSSAHGPHD
RSKPSSPSSSCHTMSPSPSPSTQTSPRRSSSSRKERRSQRQKARHVCSSSVRDNQREVEEERMEEEMEDSEER
MEEEAAGQQQ
>Oryzias_latipes_XP_004081133.1
MASSRIVICLLRNDLRLHDNELFFWAQKNADHIVPLYCFDPRHYVGTYNFNFPKTGPFRLRFLLDSVRDLRNT
LLSKGSNLVVRRGKPEEVVADLIKQLGSVSSVAFHEEVASEELNVEKKVKEVCAQMEVKVHTCWGSTLFHRDD
LPFPHMARLPDVYTEFRKAVESKSRVRPVFPTPDRLNSLPPGLEGGAIPTAEDLEQTEPETDPRSAFPCSGGE
SQALARLKHYFWDTDAVATYKETRNGLIGVDYSTKFSPWLAMGCISPRYIYHQIKKYEQERTANQSTYWVIFE
LLWRDYFKFVGVKYGNKMFFIKGLQDKSLPWKRDTKLFDAWKEGRTGVPFVDANMRELAMTGFMSNRGRQNVA
SFLTKDLGLDWRMGAEWFEYLLIDHDVCSNYGNWLYSAGIGNDPRENRKFNMIKQALDYDSNGDYVRQWIPEL
TAVRGGDVHTPWNLSSAALSRARVSLGETYPTPIVTAPEWGRHFNKKPSGPGPSQKGKKGPSHTPKQHRDRGI
DFYFSKSKNL
>Oryzias_latipes_XP_011474490.1
MPPSKSDQALVSEMLKEVLAGREDPEGFFAICLSVLGHRETYSVFQTLIKPLSTANTSVHSTLTLIYEEYFSK
TEDDDLELALALSLMEMEDQQPSTQHMQAEVRTNQSGPFQLNSASMSLEKSSIKPEDQVGRENDPVAKSGLKS
KESPNSESRDIAPQRGTHENQAQTKQDAFKEPQNLAKDQTLNQPEEPKRSKSRRKRRKGAASLVGLPGSPSAS
PPVLLWFRRDLRLCDNPALNAALEMDAPVIPIFIWSPEEEEGPGVTVAAGGASKYWLHQALACICTSLNHIGS
RLTFLKADGEEKCSLPTLKQLVKETGAKTLLANALYEPWLKERDDMVESTLQKEGVQCRIFPSYCLRDPYSVS
TVGVGLRGIGSVSHFMSCCKQNPGSAIGVPLDPPQSLPTPSCWPQGVPLEALGLARMPRRKDGTTIDWAANIR
KSWDFSEDGAHARLDNFLRDGVYRYEKESGRADAPNTSSLSPYLHFGQLSPRWLLWDAKGARCRSPKFQRKLA
WRDLAYWQLTLFPDLPWESIRPPYKALRWNTDRSHLKAWQRGKTGYPLVDAAMRQLWLTGWMNNYMRHVVASF
LIAYLHLPWQEGYRWFQDTLVDADVAIDAMMWQNGGMCGLDHWNFVMHPVDAALTCDPYGHYVRKWCPELADL
PDELIHKPWKCPASMLRRAKVVFGETFPERIVVDLEERRNQSLQDVASVRKEFGQFVDKRSGCDLVPLPPRLV
AEALGLSQRDGAVGKQFLLPVITRMEFKYQQDDPDAASNPYNAVLKGYVSRKRDETIAFLNQRDFTASVMHEG
TQRLERLERDYRRMEGLPPPPSTQGRARRTPTAKDRFSIVPGGAVTS
>Oryzias_lat_NP_001098271.1_CPD_photol
MSDKKKRKATATAGDAEPSAKQPREDKPTKAAGWLHSLIRQQREDKKDMKFSKKRQRFISETQKVRQGSGGVL
YWMLRDQRVQDNWALIRAQQLAAKESLPLHVCFCLVVPKSELSTLRHYSFLLKGLEEVQKECKHLNIQFHLLH
GAAGDVLPGFVTGHNFGAVVTDFSPLREPLQWLEAVKKGLPEDIPFIQVDAHNIVPCWVASPKLEYSARTIRG
KITNLLSEFLTDFPLVDKHPFSATKTAKAVDWDKTLASLKVDRTVGEPKLAKPGTEAGLAMLESFIDVRLKLF
GTQRNDPNAAALSQLSPWLRFGQLSAQRVALQVRKNSSPSVPAFIEELVVRRELTDNFCFYNKNYDSVTGAYE
WAQKTLKDHAKDKREYLYTREQFEKAQTHDKLWNAAQIQMVTEGKMHGFLRMYWAKKILEWSTSPEEALSIAL
YLNDRYELDGQDPNGFVGCMWSICGIHDQGWAERAVFGKIRFMNYKGCLRKFNVAQFERKYCSKKV
>Oryzias_lat_XP_004067451.1_cry-1
MAHVCIHWFRKGLRLHDNPALMAALRDCKELYPLFILDPYLYDQNLAGINRLRFLISSLQDLDCSLRKLNSRL
FVVRGKPEEVLPKLFTKWNVTKLTYEYDTEPYSRSRDKNVTMLAEEQRIQVIYKISHTLYDIDRIIEENNGKP
PLTYNRLRDIVKALGSPKKPIPAPTVEDMKNIAPFSEKHKPEYGIPSLEELGLDTSSLAEEIFPGGEQEALRR
LDTYMQRPGWVCSFEKPNTSPNSLSPSTTVLSPYVTFGCLSARTFWWRLAEVYQGKKHSDPPVSLHGQLLWRE
FFYTASVGIPNFNKMTGNPACTQVDWDENQEYLAAWREARTGFPFIDAIMTQLRQEGWIHHLARHAVACFLTR
GDLWISWEEGQKVFEKLLLDGDWALNAGNWQWLSASTFFHQFFRVYSPVAFGKKTDKNGDYIKKYLPILKKFP
PQYIYEPWKAPRSVQQAAGCIVGKDYPKPIIEHEVISKKNIQRMKQAYARRTSGSTESPTKKQGVKRKAPTVV
DLIQKKQKRS
>Oryzias_lat_XP_004069915.1_cry-1a
MTVNTVHWFRKGLRLHDNPALRDSIRGSDTLRCIYILDPWFAGSSNVGINRWRFLLHCLEDLDASLRKLNSRL
FVIRGQPTDVFPRLFKEWQITRLSYEYDSEPFGKERDAAIQKLASEAAVEVVVRISHTLYDLDRIIELNGGPP
PLTYKRFQALINRMDAVELPAERITLETMQSCVTPVSENHDDKFGVLSLEELGFETEGLPTAVWPGGETEALL
RLERHLERKAWVANYERPRMNANSLLATPTGLSPYLRFGCLSCRLFYFKLTDLYKKVKKNSSPPLSLYGQLLW
REFFYTTATNNPCFDKMEGNPVCVQIPWDRNPEALAKWAEGRTGFAWIDAIMTQLRQEGWIHHLARHAVACFL
TRGDLWISWEEGVKVFEELLLDADWSVNAGSWMWLSCSSFFQQFFHCYCPVGFGRRTDPNGDYIRRYLPVLRG
FPAKYIYDPWNAPEEVQKAARCIIGVHYPKPMVHHAEASRINIERMKQIYQQLSCYRGLGLLASIPTNPSSDA
DEGQRGSSEVPEGASRSGGGPSTQKRRHEDAPSASSSRSLKQSKLGGSCSR
>Oryzias_mela__KAF6716703.1
MATSRTVICLLRNDLRLHDNELFFWAQKNADHIVPLYCFDPRHYVGTYNFNFPKTGPFRLRFLLESVRDLRNT
LRSKGSNLVVRRGKPEEVVADLIKQLGSVSSVAFHEEVASEELNVEKKVKEVCARMKVKVHTCWGSTLFHRDD
LPFPHMSRLPDVYTEFRKAVESQSRVRPVFPTPDRLNPLPPGLEDGVIPTAEDLEQTEAETDPRSAFPCSGGE

NQALARLKHYFWDTDAVATYKETRNGLIGVDYSTKFSPWLAMGCISPRYIYHQIKKYEQERTANQSTYWVIFE
LLWRDYFKFVGVKYGNKMFFVKGLQDKSLPWKRDRKLFDAWKEGRTGVPFVDANMRELAMTGFMSNRGRQNVA
SFLTKDLGLDWRMGAEWFEYLLIDHDVCSNYGNWLYSAGIGNDPRENRKFNMIKQALDYDSNGDYVRQWIPEL
AAVRGADVHTPWNLSSASLSHAHVSLGETYPTPIVIAPEWGRHFNKKPSGPGPSRKGKKGPSHTPKQHRDRGI
DFYFNKSKNL
>Oryzias_mela__KAF6720364.1
MRLVGAAISFEVLCFPCRKGDKLSKRRTFLVQLFSLVSRPSSTLLYPRKSANMSAKKRKATSAAGEAEPSTKQ
PRQENPKKEAGWLHGLIKQQREDKKDMKFNKKRERFISETQKIRQGSGGVLYWMQRDQRVQDNWALIRAQQLA
TKESLPLHVCFCLVVPKSELSTLRHYSFLLKGLEEVEKECKHLNIQFHLLRGAAGDVLPDFVTGHNFGAVVTD
FSPLREPVQWLEAVKKGLPEDIPFIQVDAHNIVPCWVASPKLEYSARTIRGKITNLLPEFLTAFPLVDKHSFS
AAKSAKAVDWDKTLASLQVDRTVGEPKWAKPGTEAGLAMLESFIDVRLKLFGEQRNDPNAAALSQLSPWLRFG
QLSAQRVALQVRKNPSPSVPAFIEELVVRRELTDNFCFYNENYDNVKGAYEWAQKTLKDHAKDKREYLYTREQ
FEKAQTHDKLWNAAQNQMVTEGKMHGFLRMYWAKKILEWTTSPEEALSIALYLNDRYELDGQDPNGFVGCMWS
ICGIHDQGWAERPVFGKIRFMNYKGCLRKFNVAQFERKYCSKKV
>Oryzias_mela__KAF6735023.1
MPPSKDDHALVREMLKEVLTGREDPEGFFAICLSVLGHRETYSMFPTLIKPLSTANISVHSTLTLIYEEYFSK
TGDDDLELALALSLMEMEDQQLSTQHLQSEVRTNQSDQFRLNSASGFTNKDTKTKHNDPQNNNKAQTKQYAVK
ESTNLMEDQTSNQSEKPKSAKSRRKRRKGTVSIIGLPGSPSASPPVLLWFRRDLRLCDNPALNAALEMGAPVI
PVFIWSPEEEEGPGVTVAAGGASKYWLHQALACCCTSLNRIGSRLTFLEAEEQSSLRTLKQLVKETGARTLLA
NALYEPWLKERDDAGIGSVSHFTSCCKQNPGPAIGVPLDPPQSLPTPACWPQGVPLEALGLARMPRRKDGTII
DWAANIRTSWDFSEDGAHARLDAFLRDGVYRYEKESGRADAPNTSSLSPYLHFGQLSPRWLLWDAKGARCRSP
KFQRKLAWRDLAYWQLTLFPDLPWESIRPPYKALRWNTDRRHLKAWQRGKTGYPLVDAAMRQLWLTGWMNNYT
RHVVASFLIAYLHLPWQEGYRWFQDTLVDADVAIDAMMWQNGGMCGLDHWNFVMHPVDAALTCDPYGHYVRKW
CPELADLPDELIHKPWKCPASMLRRAKIVFGETYPERIVVDLEERRNQSLQDVASVRKEFGQFVDKRSGCDLV
PLPPRLVSEALGWSKRDGEAAKEGKQFLLPVITRMEFKYQRDDPDADAASNPYNAVLKGYVSRKRDETIAFLN
QQDFTASVMYEGAQRRERLERDYRRMEGLPPPPSARGRARRTPTAKDRFSIVPGGVVTS
>Oryzias_mela__XP_024136765.1
MVVNSVHWFRKGLRLHDNPALQEALNGADTVRCVYILDPWFAGAANVGINRWRFLLEALEDLDCSLKKLNSRL
FVVRGQPTEVFPRLFKEWNVTRLTFEYDPEPYGKERDGAIIKMAQEFGVETVVRNSHTLYNLDRIIEMNNNSP
PLTFKRFQTIVSRLELPRRPLPAITQQQMESCQRKIGDNHDQLYSIPSLEELGFRTERLPPAVWRGGESEALD
RLSKHLDKKVWVASVEHTRVNKCSLYASPTGLSPYLRFGCLSCRVLYYNLRELYMKLRKRCSPPLSLFGQLLW
REFFYTAATNNPNFDRMEGNPICVQIPWDQNPEALAKWAEGRTGFPWIDAIMTQLRQEGWIHHLARHAVACFL
TRGDLWISWVSGMKVFEELLLDADWSVNAGSWMWLSCSAFFQQFFHCYCPVGFGRRTDPSGDYIRRYIPILKD
FPNRYIYEPWNAPESVQKAANCVVGVDYPKPMVNHAESSRLNIERMKQVYQQLSHYRGLSLLASVPTIQEEAE
PPMTDESQTSSGPDSPHRDPADDEAAGSSTVPDSSTSAPPQDKEDPAHCCHSHQVSAHSPCTSTGSPAPLSCH
SPHDRSKPSSPSSSCHTMSPSPSPSTQTSPRRSSSSRKERRSQRQKARHACSSSARENKREVEEERMEEEVED
SEERMEEEAAEQLQ
>Oryzias_mela__XP_024137113.1
MVVNTVHWFRKGLRLHDNPALTDSIRGSDTLRCIYILDPWFAGSSNVGINRWRFLLHCLEDLDASLRKLNSRL
FVIRGQPTDVFPRLFKEWQITRLSYEYDSEPFGKERDAAIQKLASEAAVEVMVRISHTLYDLDRIIELNSGQP
PLTYKRFQALINRMDAVELPAERITSEVMQTCVTPVSENHDDKFGVPSLEELGFETEGLPTAVWPGGETEALL
RLERHLERKAWVANFERPRMNANSLLATPTGLSPYLRFGCLSCRLFYFKLTDLYRKVKKNSSPPLSLYGQLLW
REFFYTTATNNPCFDKMDGNPMCVQIPWDRNPEALAKWAEGRTGFSWIDAIMTQLRQEGWIHHLARHAVACFL
TRGDLWISWEEGVKVFEELLLDADWSVNAGSWMWLSCSSFFQQFFHCYCPVGFGRRTDPNGDYIRRYLPVLRG
FPAKYIYDPWNAPEEVQKAAKCIIGVDYPKPMVNHAEASRINIERMKQIYQQLSCYRGLGLLASVPANGGGGT
NTSEAPRGSAEDRDEGAPPSEREAATQKRRHEEAPSAGSSRSWKQSKLGSR
>Oryzias_mela__XP_024150891.1
MRCAYMAHVCIHWFRKGLRLHDNPALIAALRDCKELYPLFILDPYLHDNSRVGINRWRFLISSLQDLDSSLRK
LNSRLFVVKGKPEEVLPKLFTKWNVTKLTYEYDTEPYSLSRDKNVTTLAEERGIQVIYKISHTLYDIDRIIVE
NNGKPPLTYNRLRDIVRALGSPKKPIPAPTLEDMKNVASFSEKHKQEYGIPSLEELGLDTSSLTEEIFPGGEQ
EALRRLDKYMQRTGWVCSFEKPNTSPNSLSPSTTVLSPYVTFGCLSARTFWWRLAEVYQGRKHSDPPVSLHGQ
LLWREFFYTASVGIPNFNKMQGNSVCTQVDWDENQEYLAAWREARTGFPFIDAIMTQLRQEGWIHHLARHAVA
CFLTRGDLWISWEEGQKVFEELLLDGDWALNAGNWQWLSASTFFHQFFRVYSPVAFGKKTDKNGDYIKKYLPI
LKKFPPQYIYEPWKAPRSVQQAAGCIVGKDYPKPIVEHEVISKKNIQRMKQAYARRSSASSESPTKKQGLKRK
APTVVDLMQKKQKKS
>Ostreococcus_tau_OUS42345.1_cry_DASH,_
MGRTRVVIWFRNDLRLLDNACVARAATLASESSDVEVVPVYVFDETYFKPSKRGLARFGAGRGKFTLECVGDL
KTSLRALGSDLLVRCGKSRDVIAELTLTGANDRTIILTQTEVTSEETEMDVAVERATRERARGGAASATMERH
WGSTLYHIDDVPFDVTSGLSDLPDVFTPFRNKVESKCKVRDVIPAPTANALGHVPASVEGFEWMPDPSDLPFA
SSEIAMDCDKRIKDCLDERSVLDFKGGESNALARVKYYLWESDRLATYFETRNGMLGGDYSTKLAPWLALGCV

SPRHVVSEIRRYESERVENKSTYWVIFELIWRDFFKFFALKHGNKIFHLDGTAGRRASWKRDEKILKAWKTGT
TGYPLIDANMRELAATGFMSNRGRQNVASWLALDAGIDWRHGADWFEHHLLDYDTASNWGNWCAAAGMTGGRI
NRFNIAKQTKDYDPAGEYIKTWVKELAEVPAAYIADPNQAPRELRDRIGLNYPNKLALPRRDFTEMGSPPGPR
RGGGGGGRGRGRPGGSTPNRGTKARVASVYDTVYG
>Ostreococcus_tau_OUS44591.1
MPWRKDKNAFDAWTKGQTGYPMVDAGMRQLWQTGWMHNRVRMIVASFLTKHLLLPWQDGEAWFWDTLVDADPA
NNAASWQWTAGSGADAAPYFRVFNPISQGSKFDETGDYVRRFCPELKDLPDKYLHAPWEASEEILNKAGIQLG
KTYPKPIVDHSGARQRALDAYDTLKEKRDAA
>Ostreococcus_tau_OUS49163.1_FAD_bindin
MTLTGASALIARAGRARAARTARDRRRALATRRVSQKVQNDDVGVPSASSSDPDVRGAVWLARSHDLRMRDNE
AFISACAEARAVGGVVACVFAWSERDARESGGARRAWLAKALEALDRDLRRTYGGGVSMFKGEGAEGAMRAAR
AIRATRVFASKRYEPENAEADERASVEMAVRGVELVRTRGFLLFEPSEVRIDVAARDGYFGTLMPFVHAAERT
CGDPGKPKPAPVDVRMVDLGTESDHEWEQIGLEDLGVAPASDRRVDWSVGIRADWDISEGGAVETWSQFTAVG
LDRFEAEHGRADPEAGVTVSRLSPYLRFGMISPRTVYHDLVKKDGKNISKTFWHRLYRRELAYWQLHHWPDLP
KTSVRSHYENRAWLSGDEADEALRRWQRGETGFPVVDCGMRRLWRTGWMHQTERMITATFLVDYLGVHWTRGA
DWFMRTLVDADEAINSMMWQNAGKSGLDQWDFFEGTLAPDGSARAHDPHGDAIARWIPELAALPRGYLRHRPW
EASKATLAECDVELGSTYPRRMIVDLDRARQRMLDDVTTIRHAELVRAGGLVKPGSMEDLVVAPRAAVVRGAA
DLARVSTRKEFKRSLKALKASIAGIDLPSNPSSKPHGKSRANASRPKVSAASRPKSGPRSGRPPAPDRDAVRA
SRREAKRLDAAFRGRVFHHLPVDDLDDPDAAPPRVGRRPAR
>Ostreococcus_tau_XP_003078467.2_DNA_photol
MAPACSKKRVRALTSNTEPMASATAPVMYWMSRDQRVDDNWALLRACDLARERGAPVVIAFNLLTKYLGAGAR
QFGFMLRGLRELEAKARAAKATFAMTYGDEPAAAIDALAKKIGAKTIVCDFSPLRDGVRWRKDLAVLSEKRGA
HVEECDAHNVVPCWEASDKLEVGARTLRGRLAKRYPEFLKEFPAIPDDLVEYDGPAIDAVKWDDLLAEALKRG
EAVPEVTWAIPGETAARAVLDDFVANRMKLYEKRNDPSKPRALSGLSPWLHFGQISAQRCALEAKKAVGKASP
AAYDSFFEELVVRRELADNFCYYCPGQKYDEMEGQKYDWAKDTLRAHAGDKRPYIYTLEQLERAQTHDDLWNA
AQRELRYGGKMHGFCRMYWAKKILEWTESPEQALKWSIYLNDTYSLDGRDPSGYVGCMWSIVGVHDQGWKERE
VFGKIRYMAYDSTKKKFNIPDYIARVNALVKAAKSDFKTGEKSSAANPGLFSIDVSGVKRKADAMA
>Ostreococcus_tau_XP_022838904.1_DNA_photol
MSVASTSAAAKGRTLVWFRKALRVHDNPALSRGTLHATACQPVFVLDPWFCQPSRVGANRMRFLLQSLRDLDA
QLRARGSSLLVLHGEPRVVLPRACKKWRVDSVTWEHDIEPYAKIRDAAVRGALERAGVECHAASGHTLYDVDE
MLEKCKGAPPTTYQGFFKIVDKMGAPNAPIDAMEKMPGSFASSDEETKALVQGVADAYGIPTLEDLGYEPLGD
DEGFPGVGGETEGLRRLRLMLARKEWIGQFEKPSTNPTTRFHGLSQSGKTKPKSPFEIAAGRSKDGDASTSGA
EALMIPSTTALSPYMKFGCVSPRVFYHELTAVYKELEGKHSKPPTSLMGQLMWREFYYLVAAGTKNFDKMEGN
AICRQIPWKKDRELFAAWENAQTGFPWIDAAMTQLRREGWLHHLARHAVACFLTRGDLFIHWEWGRDAFDRDL
VDADWALNNGNWMWLSCSAFFYQYFRVYSPIAFAKKYDKDGQYVRHYLPVLKNMPSKYVYEPWLAPLDVQKKA
GCVVGVDYPAPIVDHSAASKRCVDDIATAYAAHKDANAVAGKKRKASD
>Otolemur_garn__XP_003781307.1
MWVGRGAPTGRSSGGLAVREAMAAAVATTAAQGPATSADGASSVHWFRKGLRLHDNPALLAAVRGARCVRCVY
ILDPWFAASSSVGINRWRFLLQSLEDLDTSLRKLNSRLFVVRGQPADVFPRLFKEWGVTRLTFEYDSEPFGKE
RDAAIMKMAKEAGVEVVTENSHTLYDLDRIIELNGQKPPLTYKRFQAIISRMELPKKPVGSVTSQQMESCRAE
IQENHDETYGVPSLEELGFPTEGLGPAVWQGGETEALARLDKHLERKAWVANYERPRMNANSLLASPTGLSPY
LRFGCLSCRLFYYRLWDLYKKVKRNSTPPLSLFGQLLWREFFYTAATNNPRFDRMEGNPICIQIPWDRNPEAL
AKWAEGKTGFPWIDAIMTQLRQEGWIHHLARHAVACFLTRGDLWVSWESGVRVFDELLLDADFSVNSGSWMWL
SCSAFFQQFFHCYCPVGFGRRTDPSGDYIRRYLPKLKGFPSRYIYEPWNAPESIQKAAKCIIGVDYPRPIVNH
AETSRLNIERMKQIYQQLSRYRGLCLLASVPSCMEDLSHPVAEPCSSQAGSINNTGPRPLLSGSASPKRKLEA
AEEPPGEELSKRARVTELPTPELPSKDA
>Otolemur_garn__XP_003783322.1
MGVNAVHWFRKGLRLHDNPALKECIQGADTIRCVYILDPWFAGSSNVGINRWRFLLQCLEDLDANLRKLNSRL
FVIRGQPADVFPRLFKEWNITRLSIEYDSEPFGKERDAAIKKLATEAGVEVIVRISHTLYDLDKIIELNGGQP
PLTYKRFQTLISKMEPLEIPVETITSEIIEKCTTPLFDDHDEKYGVPSLEELGFDTDGLPSAVWPGGETEALT
RLERHLERKAWVANFERPRMNANSLLASPTGLSPYLRFGCLSCRLFYFKLTDLYKKVKKNSSPPLSLYGQLLW
REFFYTAATNNPRFDKMEGNPICVQIPWDKNPEALAKWAEGRTGFPWIDAIMTQLRQEGWIHHLARHAVACFL
TRGDLWISWEEGMKVFEELLLDADWSINAGSWMWLSCSSFFQQFFHCYCPASFGRRTDPNGDYIRRYLPVLRG
FPAKYIYDPWNAPEGIQKVAKCLIGINYPKPMVNHAEASRLNIERMKQIYQQLSRYRGLGLLASVPSNPNGNG
SFMEYSPPENIPGCSSSGNCSQGSGILHYAPGDGQQPHLLKQGRSSMGTGLSGGKRPSQEEDMQSVGPKVQRQ
STN
>Owenia_fusi__CAC9497535.1
METKTKKPKNVLHWFRKGLRLHDNPSLMDSLKDADTFRCVYIIDPWFAGYSQVGINKWRFLLQCLEDLDLGLR
NLNSRLFVVRGQPADVFPKLFREWNITTLSFEQDPEPYGRERDCAICLMAREAMVEVIIRISHTLYDLNTIMH
RNNGCPPLTYKKFQKILETIKPPEYPIEAVTLKHVAGATTPIGADHEDKFSVPTLEELGFDTELLYPLVWRGG

EQEALRRLDRHLERKAWVASFEKPKMAPQSLLATQTGLTPYLRFGCLSARQFYWKLTELYRKVKKRQDPPLSL
HGQLLWREFFYTIATQNPYFDRMVGNPICVQIPWDRNPEAFAKWAEGKTGFPWIDAIMCQLRQEGWIHHLARH
AVACFLTRGDLWLSWEDGMKVFDELLLDADWSVNAGMWMWLSCSSFFQQFFHCYCPVNFGKRADPNGDYIKRY
LPILRGYSAQYIYEPWTAPMQVQKAAKCIVGQDYPLPMVNHVHSSTINLKRMKQVYQQVTYKANCSTATSQDT
VQVQFMKKRAQMLQQKDIYNEHPDPRAELPRGSTSNKQTYQPV
>Owenia_fusi__CAC9499959.1
MGRGQSGDDSITKPGCSILWFRHGLRLHDNPALQEAIKDCEKLYPVFIFDGQVAGTSTSGYNRTRFLLECLED
LDRQFKDAGGRLYVLYGKPVDVFREIHQEWGVTKICFEQDPEPIWQNRDALVRDFCKSAGIECVEKIGHTLWN
PKDIISENGGMPPLTYEKFNAITSLIGPPPRPTERHSFISVKLPVEPDHDERHSIPSLEKLGKEPELPEQLMR
INQWKGGESEARKLFKIRMRDEEEAFKKGFMMPNQFKPNMAAPPTSMAAHLRFGCISVREMYWAIHDTFEKVR
KEDSPTQLTGQLVWREFFYTMSVGNKDYDKMAPNPICLDIPWYENKEHLKLWEQGQTGFPFIDACQRQMLQEG
WVHHVGRHATSCFLTRGDLWISWEEGLKVYLKYMLDADWSVCAGNWMWVSSSAFEKVLQCPSCLDPVKYGMRM
DSKGEFVRRYVPELRDMPLRYLFSPWLSPLNIQKKANCIIGTDYPKPMVNHKEVAAKNADMMLKVKQAHENKV
TSICAPSNSDEVRKFMWLPTSQEKGLPSCTADDFCKAVENL
>Owenia_fusi__CAC9618788.1
MSHNTIHWFRKGLRLHDNLALLEGIRNSAEWRAVFVLDPWFVQNAKVGPNRWRFLMQCLEDLNNSLNKLGSRL
FVIRGNPNEVFPKLFKKWEITQLTFEVDTEPYAKQRDQTITDLAKKHQIDVLQRVSHTLYDTKQIIKKNGGSA
PGTYQRCLTVMSQLGAPPQPEPTLSKDDIKGCKLCMDACGFTADIPTLEELNVKENDLETCLYVGGESEALKR
LDKYMTRQQWVCNFEKPKTEPNSINPSTTVLSPYLKFGCLSARTFYHRLNEIYRHNKKHSGPPVSLEGQLYWR
EFFTTVGSDTPNFDKMVGNPICRQIPWDDNTQFLEAWKHGKTGYPFIDAIMVQLRREGWIHHLARHAVACFLT
RGDLWISWEEGQKVFEELLLDADWSLNAGNWMWLSASAFFHQYFRVYSPVAFGKKTDKNGDFIRKYLPVLQNF
PAQYIYEPWKAPLKVQEKFKCIIGKDYPKPIVDHDVVRQLNIKKMAAAYAKNKEAKEESPKKKKQKVK
>Owenia_fusi__CAC9662513.1
MISTVHRSYQSVQILRNLCCHLKVFRKMSETDESPTKKMKLQEGEPPDDFIERIQYQRTTKTSSVADFKFNKK
RVRMISSAENLDETSSGVVYWMSRDQRVQDNWALLYSQRLALKQKVPLYVCFCLVPKFLEATIRQFGFMLKGL
QQVEKDCKELSISFHLLIGYAKDVLPQFIEENNIGAMVTDFSPLRVPSQWVKDVSEQLGKTPFCQVDAHNVVP
CWEASPKLEYGARTIRSKINNQLSTYLTEFPPVIKHPHTAKNIQKPIDWSAAEASLEVDRKVTEVEWAEPGTA
AGLRTLESFIKKRLKYFDVDRNDPTKNALSNLSPWIHFGQISAQRCVLEVKKFRSRYGKSVEGYIEECVVRRE
LSDNFCYYNEKYDTIEGTNDWAKTSLELHKKDKREYVYSREQLETAKTHDPLWNAAQRQMVGEGKMHGFLRMY
WAKKILEWTASPEIALSDAIYLNDKYSLDGRDPNGYVGCMWSICGIHDQGWKEREVFGKIRYMNYAGCKRKFN
VAAFEAKYNKKKM
>Owenia_fusi__CAC9667714.1
MYPLPGRKRSPKKKQMLKMLLKHFEYLLKHFGVLRYTIKMIYHLKQLTVFLILLPLPSGLDPQQLPTMSDLGV
QEIAPDKRSAFPFSGGETSALDRLNKYLWETDSVAVYKETRDGMVGSDYSTKFSTWLANGCLSPRQIYWELKK
YESKRTSNQSTHSVVFELLWRDYFKYVGLKHGDKLFNLDGIMGKQIEWKQDQKLFEAWKEGRTGVPYVDANMR
EMAATGYMSNRGRQNVASFLTNDLKLDWRMGAEWFESMLIDHDVCSNYGNWQYSAGIGNDPSQDRKFNVVKQG
LDYDPQGEYVRLWVPELENIKTGDVHTPWALSTGQLADANLELGTTYPNPIVVAPEWSTQ
>Pandion_hali__NXS67412.1
CIYILDPWFAASSAVGINRWRFLLQSLEDLDNSLRKLNSRLFVVRGQPTDVFPRLFKEWGVTRLTFEYDSEPF
GKERDAAIVKLAKEAGVEVVIENSHTLYDLDRIIELNGHKPPLTYKRFQAIISRMELPKKPVSTVISQQMETC
KVDIQENHDDVYGVPSLEELGFPTDGLAPAVWQGGETEALARLDKHLERKAWVANYERPRMNANSLLASPTGL
SPYLRFGCLSCRLFYYRLWELYKKVKRNSTPPLSLYGQLLWREFFYTAATNNPKFDRMEGNPICIQIPWDRNP
EALAKWAEGKTGFPWIDAIMTQLRQEGWIHHLARHAVACFLTRGDLWISWESGVRVFDELLLDADFSVNAGSW
MWLSCSAFFQQFFHCYCPVGFGRRTDPSGDYVKRYLPKLKGFPSRYIYEPWNAPESVQKAAKCIIGVDYPKPM
VNHAETSRLNIERMKQIYQQLSRYRGLCLLASVPSCVEDLSGPVTDSASGQGCSTNTATRLSQAEQASPKRKH
EGAEELCTEELYKRAKVTGLPTPEIPGKSL
>Pandion_hali__NXS68492.1
LAQLLPFSSSVLKVDYDVCSNYGNWLYSAGIGNDPRENRKFNMIKQGLDYDGNGDYVRLWVPELQGIKGADIH
TPWALNSAALSQAGVTLGETYPQPVVTAPEWSRHINQRPHGRKPHPRGRRGPAHTPMQHKDRGIDFYFSHKKD
V
>Pandion_hali__NXS72903.1
DNWAFLYAQRLALKQELPLHVCFCLVPKFLDATIRHYGFMLRGLQEVAKECRELNVPFHLLLGYAKDVLPPFV
VEHGVGGLVTDFCPLRLPRQWVEDVRERLPEDVPFVQVDAHNIVPCWVASPKQEYSARTIRGKIHAQLPEFLT
EFPPIIRHPYPPSCPAEPIAWEACYSSLQVDRTVKEVEWATPGTAAGLAVLQSFIAERLKSFGSHRNDPNKAA
LSNLSPWFHFGQVSTQRAILEVQKHRGKYKESVDAFVEEAVVRRELAENFCYYNENYDSVQGAYDWAQTTLKL
HAKDKRPFLYELQELEQGTTHDPLWNAAQLQMVREGKMHGFLRMYWAKKILEWTHSPEEALRFAIYLNDRYEL
DGRDPNGYVGKLQEGGIEQGALSLMAKATVGCLWSICGIHDQGWAERAIFGKIRYMNYAGCKRKFDVGQFERR
YTPDT
>Pandion_hali__NXS73800.1

MQHSSIHWFRKGLRLHDNPALLAAATDCHRLHPLFILDPSSGRAGTNAWRFLLDALQDLDGSLREMGSRLFVV
RGCPEEVFPCLFHAWGTTRLTFEVDTEPSARRRDAAVAELAEQHGVEVIREVSHTLYDTERVLALNDGKAPLT
YKHLQSLLASLGPPEKPAPALTLEHLQGCHTACQVSHDAEYGVPTLEELGQDPTKAGPHLYPGGETAALTRLD
ALMDRTAWVCSFKKPETEPTSLSPSTTVLSPYLRFGCLSVRTFWWRLDKVYQGQREHSQPPVSLHGQLLWREF
FYTAGAGIPNFDRMVGNPVCLQVNWDDNPQHLHAWREGRTGYPFIDAIMTQLRTEGWIHHLARHAVACFLTRG
DLWVSWEEGLKVFEELLLDADWSLNAGNWLWLSGSTFFHRYFHIYSPVAFGKKTDRDGAYIRKYLPILKDFPV
KYIYEPWKAPQAVQEQAGCLVGTHYPQPIVEHGEASKRNLGRMKAARAQK
>Pandion_hali__NXS77093.1
MSHRTIHLFRKGLRLHDNPTLLAALECSEVVYPVYILDRKFMMSVMHIGTLRWHFLLQSLEDLQKNLCQLGSC
LLVIQGEYKSVLRDHIQKWNITQVTLDAEMEPFYREMEANIRHLGEELGFEVLSLVGHSLYDTKRILDLNSGT
PPLTYKKFLHILSLLGDPEVPVRNLMAEDFQRCRPPDPGLAECYRVPLPVDLKIPAESLSPWRGGETEGLQRL
EQHLTDQGWVASFTKPKTFPNSLLPSTTGLSPYFSMGCLSVRTFFYRLSNIYAQAKHHSLPPVSLQGQLLWRE
FFYTVASATPNFTKMAGNPICLQISWYEDAEKLHKWKTAQTGFPWIDAIMTQLRQEGWIHHLARHAVACFLTR
GDLWISWEEGMKVFEELLLDADYSINAGNWMWLSASAFFHQYTRIFCPIRFGKRMDPEGQYIRKYLPILKNFP
SKYIYEPWTATEEEQKQAGCIIGQDYPFPMVNHKEASDHNLRLMKQVREEQYRTAQLTRDDTDDPMEMKVKRD
HSEENVSKGKVARLTE
>Pangasianodon_hypo__XP_026791694.2
MTRLCYKILFCTCEVDFAAFLRHVFRIRCVSDVKRFSVIMPAKKRNLKRKGEPLKGPEKLSPGRGAGRGAGWL
QGKVAEIRRNSAECKFNMKRLRFLTDAQKVRQGCGAVLYWMARDQRVQDNWALVYAQQLALEENLPLHVCFCL
VPQYLEWTYRQYAFALRGLQEVAKECTSLNIQFHLLRGEPELTLPGFVKHWDIGAVVTDFNPLRLPLQWTEHV
KKELPSDVPFIQVDAHNVVPCWEASNKLEYAARTIRGKITKLLPEFLTEFPPVDMHPHLSTKTAKKVDWEEVM
SSLEVDRSVGEVLWAKPGMTEGIAMLESFIDQRLRLFATERNNPNANALSQLSPWIHSGQISAQRVLLEVRRW
GKRLTESVSSFTEELLVRRELSDNFCFYNDNYDNINGAYDWAKKTLQDHAKDARPYLYTREQLENADTYDQLW
NAAQRQIVLEGKMHGFMRMYWAKKILEWTASPEEAVNISIYLNDRYALDGCDPNGYVGCMWSICGIHDQGWAE
RPVFGKIRYMNYAGCKRKFDVGQFERKYAGKKS
>Pangasianodon_hypo__XP_034161949.1
MVVNSVHWFRKGLRLHDNPALQEALNGADSARCVYVLDPWFAGAANVGVNRWRFLLESLEDLDTSLRKLNSRL
FVVRGQPADVFPRLFKEWNVTRLTFEYDSEPYGKERDGAIIKMAQEFGVETIVRHSHTLYNPDRIIEMNNNNP
PLTFKRFQAIVSRLELPKKPLPTITPEQMASCSTQLSENHDKHYGVPSLEELGFKAQGKGAPVWRGGETEALE
RLNKHLDRKAWVANFERPRIKAHSLFASPTGLSPYLCFGCLSSRVLYYHLRELYMKLRRRSSPPLSLFRQLLW
REFFYTAATNNPSFDRMEGNPICVQIPWDKNPEALAKWAEGRTGFPWIDAIMCQLRQEGWIHHLARHAVACFL
TRGDLWISWESGMRVFEELLLDADWSVNAGSWMWLSCSAFFQQFFHCYCPVGFGRRIDPSGDYIRRYIPELKD
YPSRYIYEPWNTPEAIQKAANCIVGVDYPKPMINHAESSRLNIERMKQVYQQLSHYRGLSLLASVPTIQEEAE
PSMSDDSQASSSGAISASHQPPPQSEPQPSQTDPDPDPDQNPAPEPSASSAPAAMRKRGCSSEPQNSQNPKVQ
HTSQTKAEQLHTDSKQ
>Pangasianodon_hypo__XP_034162258.1
MAPNSIHWFRKGLRLHDNPALQEAVRGAGTIRCVYFLDPWFAGSSNLGVNRWRFLLQCLDDLDSSLRKLNSRL
FVIRGQPANVFPRLFKEWEISRLTFEYDSEPFGKERDAAIKKLAMEAGVEVIVKISHTLYNLDKIIELNGGQP
PLTYKRFQTLISRMDPPEMPVETLSSSLMGCCVTPVSEDHGDKYGVPSLEELGFDTEGLPSAVWPGGETEALT
RIERHLERKAWVANFERPRMNANSLLASPTGLSPYLRFGCLSCRLFYFKLTDLYRKVKKTSTPPLSLYGQLLW
REFFYTAATTNPRFDKMEGNPICVRIPWDKNPEALAKWAEAKTGFPWIDAIMTQLRQEGWIHHLARHAVACFL
TRGDLWISWEEGMKVFEELLLDADWSVNAGSWMWLSCSSFFQQFFHCYCPVGFGRRTDPNGDFIRRYLPILRG
FPAKYIYDPWNAPDSVQAAAKCIIGVHYPKPMVNHAEASRLNIERMKQIYQQLSRYRGLGLLASVPSTHNGNG
NGMAYSPGEQQSGTNTPAPAISGSSVANGNRSGSILLNFDSEEHQGPSRMQQQQKLGRVLHTAGRVTCKRERE
SERDTEGDDTLSTSHKLQRQIAEITTVYGTNGNQPGMKS
>Paragonimus_kell__KAF6776971.1
MLFDFLTNRLHLYDKHRNDPTKVAQSGLSPWLHFGQLAPQRVLLETRAVRNLHSKSADAFIEETFVRRELADN
FCFYNPRYDSLDGAYKWAQDTLQMHAKDKRNPAYTDAQMENAKTTGRPVERMSTSACSRGEDARISAHVLG
>Paragonimus_wes_KAA3677470.1_photolyase
MMSAVSGDLKLMAKTDLVSWTSNILQRRNSLGGSVRDFPFAKCRVRQLKGPNLFPELDGAITTDKIGMGGVLY
WMIRDHRVQDNWAFLYAQRLALKFGVPLHACFCLSPRYQADTLRHFAFMLEGLSEVEEECNQLNIPFHLVQAK
TTISTISTGMKRTLSDIGHSEGYSHELAVANSVLTLVRDLRIGCVVTDFCPLREPTAWIEMLLKLVPEEVPVC
QVDAHNVVPVWYASDKLEYAARTIRRKLHDKATGLWTEFPPLIAHPHLAKSTTSPQRIVWSDLRTHFPGDCQV
KPVKWAKGGSNAGLEVLFDFLSNRLHLYDKHRNDPTKVAQSGLSPWLHFGQLAPQRVLLEARAVRNVHAKSAD
AFIEEIFVRRELADNFCFYNPRYDSLDGAYKWAQDTLHLHAKDTRNPAYTDAQMETAKTKDDLWNACQHQLVH
MGKMHGFLRMYWAKKILEWHKSGPEAALRLALYLNDRYSLDGSDPNGVVGVMWSICGVHDQGWAERAIFGKIR
CMTYKGCQNKFSIPTFVAKQKNNAL
>Paralichthys_oliv__XP_019934037.1
MPPSAAGAEDSKALVRQLLREVLVGREDPEGFFAMCVSVLGQQETRTQFQSLIQPLSSANRSLHCRLTSIYLE
YFSKTEDDDLELALALSVLDVKDHQPSTCSEEYKLQQLGAKVNQSSSVQLTSASQPQGSSSRTQRADVGGGRK

KNHSSEKEPWGVMCPPQTTQETELQKSTHVDKCNKETPGTSQSVCVSKPYTSFFKQDAVLKGDQTTEEGDSIN
QSEKLKRSKNRRKRREGNGQQVVGMPTSPSAPPPVLLWFRRDLRLCDNPALIGSLENGAPVIPVFIWSPEEEE
GPGITVAMGGACKYWLHQALSCFSSSLERIGSHLVFLKANGVGNGVGSSLRTLKELVKETGARAVLANALYEP
WLKERDDVVVSALQKVGVQCRMFHSYCLRDPYSVSTEGVGLRGIGSVSHFMSCCKQNPESALGLPLDPPASLP
TPAHWPQGVSLDALELALMPRRKDGTTIDWAGDIRKSWDFSEEGAHAQLEAFLNDGVYRYEKESGRADAPNTS
SLSPYLHFGQLSPSWLLWDAKGARCRPRKFQRKLAWRDLAYWQLTLFPDLPWESLRPPYKALRWSTDRGHLKA
WQRGRTGFPLVDAAMRQLWLTGWMNNYMRHVVASFLIAYLHLPWQEGYRWFQDTLLDADVAIDAMMWQNGGMC
GLDHWNFVMHPVDAAMTCDPYGSYVRRWCPELADVPDELIHKPWKCPASMLRRAGVVFGRTYPDRIVTDLEER
RSQSLQDVAVVRREFGQYVDKRSGCDLVPLPPRLVSEALGLSHRNGGVVTEGKQFLLPVITRMEFKHQQEDPD
ADGASNPYNAVLKGYVSRKRDETIAFLNERDFTASVMHEGVQRKERLESNYRRMEGLPQPPAPQGRARRTPKA
KDRFSVVPGGAVTSLR
>Paralichthys_oliv__XP_019935864.1
MAHTCIHWFRKGLRLHDNPALLAALRDCKQLYPVFLLDPHLYNDTRMGINCWRFLLGALKDLDCSLRKLNSRL
FVVQGRPEDVLPKLFNKWKVTKLTYEYDTEPYSLVRDKTVTALAKEHGVEVIYKISHTLYDIERIIEENNGKA
PLTYNRMQAIVKTLGPPKKPVPAPTLEDMAEVKTPCPDSHEQQYEIPALEELCPDAAALGEELFPGGEQEALR
RLDEHMKQTAWVCKFEKPQTSPNSLSPSTTVLSPYVTFGCLSARTFWWRLTDVYRGKKHSDPPVSLHGQLLWR
EFFYTASVGIPNFNKMVGNPVCTQIDWDTNSEYLAAWREARTGFPFIDAIMTQLRQEGWIHHLARHAVACFLT
RGDLWISWEEGQKVFEELLLDGDWALNAGNWQWLSASTFFHQYFRVYSPVAFGKKTDKNGDYIKKYLPLLKKF
PAQYIYEPWKAPRSVQQAAGCIVGKDYPHPIVQHEVISKKNIQRMKSAYAKRSSDNTDSPTKKQGVKRKGPSV
VDMMKKKERRKSSSS
>Paralichthys_oliv__XP_019942880.1
MMSVSRTIICLLRNDLRLHDNELFHWAQRNAEHIVPLYCFDPRHYVGTYNYNLPKTGPFRLRFLLESIRDLRN
TLLNKGSNLVVRRGKPEDVVAELIKQLGSVSTVVFHEEVTSEELNVEKKVRDVCAKMKVKVHTCWGSTLHHRE
DLPFHHMSRLPDVYTEFRKAVESRSRVRPVFPAPEQLNPLPPGLDEGAIPTAQDLQQTEPLTDPRSAFPCSGG
ESQALARLKHYFWDTDAVATYKETRNGLIGVDYSTKFAPWLAIGCISPRYIYHQIKQYERERTANQSTYWVIF
ELLWRDYFKFVGVKYGNRLFQVKGLQEKSVPWKKDMKLFNAWKEGHTGVPFVDANMRELAMTGFMSNRGRQNV
ASFLTKDLGLDWRMGAEWFEYLLIDHDVCSNYGNWLYSAGIGNDPRENRKFNMIKQGLDYDNNGDYVRQWIPE
LQEIRGSDVHMPWTLSPAALSHAHVSLNETYPSPIVMAPEWNRHVNKKSSATGPSPRGKRGQSHTPKQHRDRG
IDFYFSRSKNL
>Paralichthys_oliv__XP_019957739.1
MVVNSIHWFRKGLRLHDNPSLRDSIRGADTLRCIYILDPWFAGSSNVGINRWRFLLHSLEDLDASLRKLNSRL
FVIRGQPTDVFPRLFKEWQITHLSYEYDSEPFGKERDATIQKLANKAAVEVMVQISHTLYDLDKIIELNGGQT
PRTYKSFEDLINCMGPVDVPAETITSEVIKKCAMPIGADHDVKFGVPSLEELGFETEGLTAAVWPGGETEALM
RLERHLERKAWVANSEYPRINANSLLASPTGLSPYLRFGCLSCRLFYFKLTDLYRKEKENSKPPLCFYGQLLL
REFFYTTATNNPCFDKMEGNPACVQIPWDRNPEALAKWAEGRTGFPWIDAIMTQLRQEGWIHHLAWRAVACFL
TRGDLWISWEEGMKVFEELLLDADWSVNAGSWMWFSCSSFFQQFFRCYCPVGLGRRTDPNVEYIRRYLPILRG
FPAKYIYDPWNAPEEVQKAAKCIIGEHYPKPMVNHAEASRINIERMKQIYQQPSCYSGLGLLATVPANPHNGV
RSSNTGTSRGPGGSDEQLYNLEGTSRTGRGQWTQKRRSEEAPSGSISKSWRQSK
>Paralichthys_oliv__XP_019958265.1
MRRRRKALWATQFYSSTTPCYKSPNTGGRVSVLSADVCCSQRLAVWWFTLRVKLVFRLHADRRTSGFIRCMFY
SWRSSSAPRRLLTLRNQRSPTPLLFTCSFTSVCTMSDKKRKAAPASKGPASKQQKVVPMKQEEKQQQQKPEGW
LQDLLMKQRSRVKGANFNKKRLRFISDTENMKQDSEGVLYWMSRDQRIQDNWALIHAQKLAVKKNLPLHVCFC
LFVPKSELSTLRHYSFLLNGLKEVAEECKALDIQFHLLHGAAGDVLPGFVSDRSLGAVVTDFSPLREPQEWLE
DIKKKLPKDILFKQVDAHNVVPCWEASPKQEYSARTIRGKITKLLPDFLTDFPPVEKHPVTATRTAKPIKWEK
TLASLSVDREVGEPEWAKPGTKAGLAMLESFIDVRLKVYDNQRNDPNVNALSQLSPWLRFGHISAQRVALQVE
SSGKKAGQNISSFIEELVVRRELTDNFCFYNKKYDRVEGAYEWAQKTLKDHAKDERPYFYTREQLEKAQTHDK
LWNGAQYQMVTEGKMHGFLRMYWAKKILEWTSSPEEALSIALYLNDRYELDGQDPNGFVGCMWSICGIHDQGW
AERPVFGKIRYMNYKGCLRKFDVLKFEKKYCPKNL
>Paralichthys_oliv__XP_019962786.1
IIEMNNNSPPLTFKRFQTIVSRLELPRRPLPPVTQQQMDKCRTKITNNHDQLYSIPSLEELGFRTVGLPSAVW
TGGESEALDRLNKHLDKKVWVANLEHPRVNTCSLYASPTGLSPYLRFGCLSCRVLYYNLRELYMKLRKRCSPP
LSLFGQLLWREFFYTAATNNPNFDRMDANPICVQIPWDQNPEALAKWAEGRTGYPWIDAIMTQLRQEGWIHHL
ARHAVACFLTRGDLWISWESGMKVFEELLLDADWSVNAGSWMWLSCSAFFQQFFHCYCPVGFGRRTDPSGDYI
RRYIPILKDYPNRYIYEPWNAPESVQKAANCVVGVDYPKPMINHAEGSRLNIERMKQVYQQLSHYRGLSLLAS
VPTIQEEAEPPMTDESQNSSGPDSPPRDPAHSEAAGCSTAPDSSTVYVSCAPHPELDDAQNPRPSQTPCTSSG
SHPPTPAAVSWTFVGHPPSTAAPPCPASAHSPLSRSRPPSPSSSCLTSSPAPSPAVTPVQTFPKRKGLARKVR
RSQRQRGRPSCATSGREGERTEEEEEEEEDTGEDERMEEDVDQEEGERMEEETSEAVRQGGV
>Parambassis_rang__XP_028257055.1
MAHTCIYWFRKGLRLHDNPALIAALRDCKEFYPVFILDPYLYHNTRVGNNRGRFLIGALKDLDCSLRNLNSRL
FVVRGKPEDVFPKLFNKWKVTKLAYEYDTEPYSLSRDEKVTKLAKEHGVEVIYKISHTLYDINRIIEENNGKT

PLTYNRLQAIVKTLGPPKRPVAAPTKEDMKDVKTPCSDSHEKEYGILTLEELHLDTTSLKEELFPGGEQEALR
RLDEHMKRTAWVCNFEKPQTSPNSLSPSTTVLSPYVTFGCLSARTFWWRLRDVYQGKKHSDPPVSLHGQLLWR
EFFYTASVGIPNFNKMEGNPVCTQVDWDTNPEYLAAWREGRTGFPFIDAIMTQLRQEGWIHHLARHSVACFLT
RGDLWINWEEGQKVFEELLLDGDWALNAGNWQWLSASTFFHQFFRVYSPVAFGKKTDKNGDYIKKYLPLLKKF
PAEYIYEPWKAPRSVQQAAGCIVGKDYPHPIVQHEVISKKNIQRMKQAYAKRSPDTSESSSKKQGLKRRTPSA
IDMFNKKKRVK
>Parambassis_rang__XP_028263365.1
MVVNSVHWFRKGLRLHDNPSLRESIQGAETLRCIYILDPWFAGSSSVGINRWRFLLHCLEDLDASLRKLNSRL
FVIRGQPTDVFPRLFKEWKITRLSYEYDSEPFGKERDAAIHKLASEAGVEVMGRISHTLYDLDKIIELNGGQS
PLTYKRFQALINRMDAVELPAETITSEVLKKCATPINDDHDDKYGVPSLEELGFETEGLTSAVWPGGETEALM
RLERHLERKAWVANFERPRMNANSLLASPTGLSPYLRFGCLSCRLFYFKLTDLYRKVKKNSTPPQSLYGQLLW
REFFYTTATNNSCFDKMEGNPVCVQIPWNRNPEALAKWAEGRTGFPWIDAIMTQLRQEGWIHHLARHAVACFL
TRGDLWISWEEGMKVFEELLLDADWSVNAGSWMWLSCSSFFQQFFHCYCPVGFGRRTDPNGDYIRRYLPILRG
FPAKYIYDPWTAPEDVQKVAKCIIGVHYPKPMVNHAEASRINIERMKQIYQQLSCYRGLGLLATVPANPNNGN
NGSNVGGVNTGASHGSGASTDPSTQEAASRTERGQPVQKRRREEAQPESGAKSFKQSK
>Parambassis_rang__XP_028282443.1
MLHCIRKSASFYFASRPSPVLFLLSSSVTSKTTMSAKKRKSAVSAAPGKEPSAKQQKVEEEEKKERVEGWLLS
LVEKQRSEKKEMKFNNNRLNFISDTVKIKQGSEGVLYWMLRDHRVQDNWALIHAQQLAVQENLPLHVCVCMVV
PKSKLSTLRHYSFMLKGLKEVAKECEHLNIQFHLLHGEAGDVLPGFVSDRGLGAVVTDFSPLREPLQWLEDVK
KRLPKDIPIIQVDAHNIVPCWVASPKLEYSARTIRGKITKLLPEFLTDFPLVEKHPCTATRTAKLVDWDETLA
SLKVDKTVGETEWAKPGTKAGLAMLESFIDVRLKIFGAQRNDPNAHALSQLSPWIRFGHISAQRVALQVRQSG
KNAAESVAPFIEELVVRRELTDNFCFYNKKYDSVEGASEWARKTLKDHSEDKRAYLYTREQLEKAETHDKLWN
AAQYQMVSEGKMHGFLRMYWAKKILEWTSSPEEALSIALYLNDRYELDGQDPNGFVGCMWSICGIHDQGWPER
PVFGKIRYMNYKGCLRKFNVAQFEKQYSPKNL
>Parambassis_rang__XP_028286007.1
MVVNSVHWFRKGLRLHDNPALQEALNGADTVRCVYILDPWFAGAANVGINRWRFLLEALEDLDSSLKKLNSRL
FVVRGQPTDVFPRLFKEWNVTRLTFEYDPEPYGKERDGAIIKMAQEFGVETVIRNSHTLYNLDRIIEMNNNSP
PLTFKRFQTIVSRLELPRRPLPPITQQQMDKCRTNIADNHDQLYSIPSLEELGFRTEGLPSAVWRGGESEALD
RLNKHLDKKVWVANLEHPRVNTCSLYASPTGLSPYLRFGCLSCRVLYYNLRELYMKLRKRCSPPLSLFGQLLW
REFFYTAATNNPNFDRMEGNPICVQIPWDQNPEALAKWAEGRTGFPWIDAIMTQLRQEGWIHHLARHAVACFL
TRGDLWISWESGMKVFEELLLDADWSVNAGSWMWLSCSAFFQQFFHCYCPVGFGRRTDPTGDYIRRYIPILKD
YPNRYIYEPWNAPEALQKAANCVVGVDYPKPMINHAEGSRLNIERMKQVYQQLSHYRGLSLLASVPTIQEEAE
PPMTDESQTSSDPDSPPRRPADVEAVGCSTAPDSSTACPSSTVALQEEEEEDTAHSHLAQTPFVSSGSPTQPS
MLAPSPLSWSKPSSSPSSSCPASSPSLSPSPALNQISCMGPRRKSITRKMRRSQRQRGRQSSSAPSAREGEKE
EVGGEARMEEETEQEEERMEETAGQQ
>Parambassis_rang__XP_028287972.1
MPPSATAAAQATVRAVLREVLVGREDPEGFFAICLSLLGHQETRSQFLSLIQPLSTGNRSQHSILVSIYQEYF
SNTEDDELEHALALSLLEVKDQQPSTPRQESQLLQPGDRPSQSSLDHLKLESRSQDGWKKNIYAAQTGAWSKK
CTTQTEREIKLQNSTHLDNHHNQTCGTGQTLCDSGNSAKQDTSEQIKAESAVNQSEKPKRYKNRRRRRKAVGQ
QHVGLPCSPSAALPVLLWVRRDLRLCDNPALTASLELGSPVIPVFIWSPEEEEGPGVTVAMGGACKYWLHQAL
SCFCSSLEHIGSHLVLLKANGHGKEAGSSLHTLKELIKETGAKTVLANALYEPWLKERDDVVVSALQKESVEC
RMFHSYCLRDPYAVSTEGVGLRGIGSVSHFMSCCRQNPGPTVGIPQDPPGSLPSPAHWPQGVSLDTLGLARMP
RRKDGTAIDWAANIRKSWDFSEEGAHARLETFLQDGVYRYEKESGRADAPNTSCLSPYLHFGQLSPRWLLWDA
REARCRPPKFQRKLAWRDLAYWQLTLFPDLPWESLRPPYKALRWSTDRGHLKAWQRGKTGYPLVDAAMRQLWL
TGWMNNYMRHVVASFLIAYLHLPWQEGYRWFQDTLVDADVAIDAMMWQNGGMCGLDHWNFVMHPVDAAMTCDP
YGSYVRKWCPELTDLPDELIHRPWKCPASMLRRAGVVFGQTYPQRIITDLEERRTRSLQDVASVRREFGQYVD
KHSGCDLVPLPPRLVSEALGLSHSDGAVVTEGKQFLLPVITRMEFKHQLEDPDGDAASNPYNAVLKGYVNRRR
DETIAFLNEKDFTASVMHEGTQRKERQEKNYRRMEGLPLAPSPGGRARRTPTAKDQFSVVPGGAVTSPR
>Parambassis_rang__XP_028288837.1
MSTSRTIICLLRNDLRLHDNELFHWAQRNAEHIVPLYCFDPRHYGGTYNYNLPKTGPFRLRFLLESIRDLRNT
LINKGSNLVVRHGKPEEVVASLIKQLGSVNTVAFHEEVTSEELNVEKKVKEVCAQMKVKVHTCWGSTLYHRDD
LPFHLISRLPDVYTQFRKATETQSRVRPVFPTPEKLNPLPTGLEEGAIPTAEDLQQTEPVTDPRSAFPCSGGE
SHALARLKHYFWDTDAVATYKETRNGLIGVDYSTKFAPWLALGCISPRYIFHQIKQYETERTANQSTYWVIFE
LLWRDYFKFVAVKYGNRLFQVNGLQDKSVPWKKDMKLFNAWKEGRTGVPFVDANMRELALTGFMSNRGRQNVA
SFLTKDLGLDWRMGAEWFEYLLIDYDVCSNYGNWLYSAGVGNDPRENRKFNMIKQGLDYDSTGDYVRQWVPEL
QSIRGADAHAPWTLSAAALSHAHVSLGETYPAPIVMAPEWSRHINQKASGSGPSPRGKKGPSHTPKQHRDRGI
DFYFSRSKNL
>Paramecium_tet_XP_001458001.1_(macronucl
MKERIKYLNDKKVNVKGKYVAYWIQASQRTKYNHALELAIQKANQEQIPLFCFFGLTKYPAANQRPYHFMLEG
LQQLKTSLADRKILFGVAKQSPDDLAISIAQNAKLLIVDCGYLRIQKQWRKKVADTIDCQFIQVETDVLVPVE

QASQKEEWAAKTIRPKIQSLTKYFAKELNEETLVKQMDKLPFQEYDISNITKVIDDLGVDKSVSIVQQFKGGE
IEAQKRLEEFLNKKLKNYAKNRNDPSLNATSNLSPYLHFGMISPLHIYLEAMKFPPSESRESFLEELIVRREL
SMNFCYYNDLYDKYEGLPDWAKNTLQEHAKDKRDYIYTLDQLEKAKTHDVYWNSAQLEAIHKGKISGYMRMYW
GKKVIEWTESPQQAFEYLVFLNDKYHLDGRDANGYTGVAWCFGKHDRPWTGRKIFGNIRYMIDSGLKRKFDTG
QYVMNVNQLKKQSLK
>Paramicrosporidium_sac_PJF19504.1_DNA_photol
MVKVAAVENAFVWFRNDLRVTHNPALLAASQYSATHGGRVYALLVTSPLEWRSIHNWGLPKASYYVRTVSVLR
RELSKRGIEMLLHTPTAKNATEYYKALASEIVQLCKSQNCSAVYMNAEYDGVALERDAAVASALDTAGILHHT
THDQCVVPVGKVLTQSGTPYKVFSQFKKTWIRYIEEHGLDMIDSEPTEIEKSSTILPDIELDLGYDMHEYPNW
DLNALHTTFPAGETEARKRLHEFLQKSVSGYHEDRNHFMEGGASKLSAPLAVGAISLTECLLAARIANKGALN
SGSPGLLTWMSELCWRDFYRHVLFHFPHVGRGASFRPEFEHLSWRGWPPNIEPAAEEHFQAWCEGRTGYPIVD
AAMRQLRREGWLCNRLRMVVAMFLTKDLRIHWRRGEQHFNQFLVDYDFSSNNGGWQWSASTGTDAQPYFRIFN
PLLQSEKFDPSGDYIRRYVDELSKVSAPSIHDPFKRCKGFDQLKYPRPIVDHSKVKEQVLALFTAATTVTTKR
PAD
>Paramormyrops_kingsleyae_XP_023657742.1_cryptochro
MVVNTIHWFRKGLRLHDNPSLRESIQGADTVRCVYILDPWFAGSSNVGINRWRFLLQCLEDLDTSLRKLNSRL
FVIRGQPTDVFPRLFKEWNISRLSYEYDSEPFGKERDAAIRKLASEAGVEVTVRISHTLYDLDKIIELNGGQS
PLTYKRFQSLISCMEAVETPAEPITADVVAKCATPVSDDHDDKFGVPSLEELGFNTEGLSTAVWPGGETEALT
RLERHLERKAWVANFERPRMNANSLLASPTGLSPYLRFGCLSCRLFYFKLTDLYKKVKKNSSPPLSLYGQLLW
REFFYAAATNNPRFDKMEGNPICVQIPWDRNPEALAKWAEGRTGFPWIDAIMTQLRQEGWIHHLARHAVACFL
TRGDLWISWEEGMKVFEELLLDADWSVNAGSWMWLSCSSFFQQFFHCYCPVGFGRRTDPNGDYIRRYLPVLRG
FPAKYIYDPWNAPEGVQKMAKCIIGVHYPKPMVHHAEASRLNIERMKQIYQQLSCYRGLGLLATVPSTPSGGN
DEAGAAMGFPLREGAQDSAAHAGYQVPVNPAGDWCAGMLPPPQGDNKPGSSSMAQQNVPAGLSMCYNQERPQS
QTAGSQPGEDLRPFHISPCIVTSNIK
>Paramormyrops_kingsleyae_XP_023661931.1_cryptochro
MVVNSVHWFRKGLRLHDNPALQEALNGADTVRCVYILDPWFAGSANVGINRCRFLLESLEDLDTSLRKLNSRL
FVVRGQPADVFPRLFKEWNVTRLTFEYDSEPYGKERDAMIIKMAKEFGIETVVRNSHTLYNLDKIIELNNNSP
PLTFKRFQAIVNRLELPKKPLPTVTRQQMDSCWTDVLVNHDERFGVPSLEELGFKTQRLSPAVWKGGEMEALD
RLKKHLDRKAWVANFERPQINVCSLIASPTGLSPYLRFGCLSSRLFYYELRDLYLKVRKHPNPPLSLFGQLLW
REFFYTAATNNQNFDRMEGNPICVQIPWDHDPESLAKWAEGRTGFPWIDAIMTQLRQEGWIHPLARHAVACFL
TRGDLWLSWESGMRVFEELLLDADWSINAGSWMWLSCSGFFQQFFHCYCPVGFGRRTDPSGEYIRRYIPKLKN
YPNRYIYEPWNAPETVQKAADCVVGKDYPKPMINHAESSRLNIERMKQVYQQLSRYKGLSLLATVPTIQEEVE
PPAMEDSGAHSSNDVCPAQPSRKALLRKRERPAEMQRAAACTQPKVQRISSAEGSADDQQ
>Paramormyrops_kin_XP_023686334.1
MSGEKTSVDVARKRRAESAAASGRKEPKLLRTEGWLVAEVDGLRRAASGAEVNTQRLRFLSQTEKVKQGSQGV
LYWMCRDQRVQDNWALLYAQQLALAEQLPLHVCFCLMPRFLDATIRQFGFMLEGLREVATECRTLEIQFHLLL
GAAAEVLPGFVKDWALGTVVTDFFPLRVPLQWVEDVKKGLPLDVPFIQVDAHNVVPCWLASDKQEYSARTMRG
KITKLLPKFLTEFPPVEKHPHCAQRPVASVDWDRARSFVKVDPTVGEVQWARAGTAAGMATLESFIEQRLHSY
AAHRNDPNCAALSHLSPWLHAGQISAQRAVLQVRRGGARGSPSVDAFVEEAVLRRELADNFCYYNRNYDCVEG
AYEWARMSLQDHAKDHRPYLYSRQELEGARTGDKLWNAAQNQLVLEGKMHGFMRMYWAKKILEWTSSPEEALS
ISLYLNDRYSLDGNDPNGYVGCMWSICGIHDQGWKERPIFGNIRYMNYNGCKRKFDVSAFEKQYGPKQLKIS
>Parasteatoda_tepi__XP_015913173.1
MSHENAIHWFRKCLRIHDNPALLEAIKNSKHFWPIFILDPWFVKNMKCGPNRWRFLAQSLQDLDDSLRKFNSR
LFVIRGKPMEVFPQIVKKWKIKLLTFESDTEPYAKVRDEEIQTLMKILKVEVCTFSTNTIYNPEKIIEFNGKV
PLTMQAYLNIVDKIGLPAKPVQSEEKIIQLTSDKERCSPLEDISDSKYDVPTLEELGVKVNDLYPCLYPGGET
EALKRLERSLSNEQYVCKFSKPDTFPNSIKPSTTVLSPYVKFGCLSARTFYYKIRDVYLKSKSGYTKPPVSLH
GQLFWREFFYTVGSVTPHFDQMIENSVCRQIPWMENKEHLEAWAHGRTGYPFIDAIMTQLRQEGWVHHLARHA
VACFLTRGDLWISWEHGMKVFEELLLDADWSLNAGNWMWLSASAFFHQYYRVYSPIAFGKKTDKNGDYIRKYV
PILKRFPPEYIYEPWKAPKKLQEQLGCVIGKDYPSPIVDHDKARVENLRRMDAVYKSAKESGGKKSASPKKSQ
KQSPKASSSHQTSALSSTPKSTKRANSQQQNKITKFLKLQ
>Parasteatoda_tepi__XP_015915881.1
MSNLKQKNVIHWFRKGLRLHDQPALKEGLQGASTWRCVFILDPWFAGSSNVGINKWRFLLQCLENLDNNLRKL
NSRLFVIRGQPADVFPRLFKLWKTTHLTFEKDPEPYGRIRDLNITTMAQENGVNVITRTSHTLYDLEKIIEKN
GGKSPLTYKQFQNILANMEQPPLPLPRITFEDLSNCYTPLSEDHEEKYGVPTLEELGFDTENLKPPVWLGGET
EALTRLERHLERKAWVASFGRPKMTPQSLLPSQTGLSPYLRFGCLSARLFYLELAELYRKIKKVNPPLSLHGQ
ILWREFFYCAATNNPKFDHMVGNPMCVQIPWDVNSDALAKWTSGQTGFPWIDAIMTQLREEGWIHHVARHAVA
CFLTRGDLWISWEEGMKVFDELLLDADWSVNAGSWMWLSCSSFFQQFFHLYCPIRFGRKADPNGDYIRRYLPV
LKNFPTKFIHEPWIAPEKVQHAAKCVIGKDYPMPIVNHQDVSHINLERMKQVYQQLSHYRGANMSNPPRPIQD
RDDEGYQKSVKRRAVEYSENSRWKAMKEA
>Parasteatoda_tepi__XP_042907496.1

MLKGLQEVEEECKSLNIHFHLLLGESKNVLPKFVKDNQVGGVVTDFSPLRVPQKWVSELASKLPPDVPLCQVD
AHNIVPCWVASDKQEYGARTIRKKIHDKLKEYLTDFPPVVKNKLTPECKFKPVDWNSVEEILEVNMDVDEVKW
AIPGTTAGLKQLSSFCKDRLKHFHDCRNDPTKNNLSNLSPWFHFGQLSIQRTILVVSKLRSKYPASVDAFVEE
AVIRRELSDNFCFYNKKYDQVDGAYDWAKKTLKDHSKDKREYIYTKEQFENAKTHDLLWNAAQRQLKKEGKMH
GFLRMYWAKKILEWTNSPEEALEFAIYFNDKYNLDGRDPNGYVGCMWSICGIHDQGWAERAVFGKIRFMNFKG
CQRKFDVNAFIQRYREK
>Parasteatoda_tepi__XP_042908256.1
MADGQLIHWFRNDLRLHDMPSLSLAMTKCSKLYPVYIFDKTGILEKRIGYNRLRYIIESLEDIDRTFKASGGR
LYVFCGEPTKIMKDLMTDWNITHLSYEDDIQPVWTDVESSIQDFCRERNVEIINKVSHTLYDPKDILKANRGN
PPVTYNVFCQVAQSLGPPPKPEPPLKFENIMLSNEEVLDSKIPSSPEDLGATMECPEQTNRIWRGGERKALEL
LNNRLQIEKDAAESGILMPNQAQPDLLSPSMSLSAPLSMGCLSVRKEENDSMLECWNEGKTGFPLIDAAVRQM
RQEGWIHHVARNAIACFLTRGDLWISWEEGFKTFMKYMIDADFAVCAGNWMWVSSSAFENILQCPSCVSPVTY
LRRFDRSGNYIKKYIPELKNMPTQFVFEPWLAPLSIQESAGCILGKDYPLPIVNHDMAATKSTEKMNAIKEKF
LTKSVPLHCTPSSPAETRIFMWLPENCLDNLIPQN
>Patiria_min_XP_038049378.1
MAKGEKKGKPATKRLKTADEGSSSSSSSLATSPPSKKLKTENGTSDDSEQEFQQVKSAPSEAQGIMEKLAALR
LRTAASVAAFKFNKKRVRVLSQVKDVPEGCHGIVYWMSRDQRVQDNWSMLYAQRLAMKLAVPLRVCFCLQPRF
LDATIRHFAFMLKGLAEVEQELKELDIPFHLLIGEAKDVLPNFVKDHNIGGLVTDFSPLRVPSKWVKEATESL
PDIPVCQVDTHNIVPCWEASPKLEYGARTIRPKITKLLPTFLTEFPPVVKHPHQAKPMPKPVDWDAADCSLDV
DRTVGEVSWAKPGTTAGLLMLEEFCLKRLKYFGADRNNPNKSALSNLSPWIHFGQISAQRAALVVRGYRSKYS
KSVEGYIEESVVRRELSDNFCFYNENYDSIQGTNDWAKQTLKVHAKDKREYVYSKERLEKARTHDDLWNAAQV
QLVKEAKMHGFLRMYWAKKILEWTPSPEEALEIAIYLNDKYSLDGRDPNGYVGCMWSICGIHDQGWAERPVFG
KIRYMNYNGCKRKFDVAQFVRKYKQKL
>Patiria_min_XP_038063798.1
MHVRYYENVELEGLPPSYSLTSKYHTVERKRAMAKYHQQSLHWFRKGLRLHDNPSLLAAVRDSQEFRAVFILD
PMFVKSGTVGINRWRFLLQSLQDLDDGLKGLGSRLYVLRGKPTEVFPDIFKKWKISHLTFELDTEPYATQRDE
AVEAIAEKHGVQITKKSSHTLYDIQSITRANGGKTPMTYQSLLSIVSKMAAPRKPDPALSKEDLQDCKLPVTS
DHDSQYGVPSLEELGLDPKDAGPVLFPGGETEAVRRMEEHLERQEWVCRFEKPKTEPNTLSPSTTVLSPYLKF
GCLSPRLFYHKIQDVYTRKKNHSKPPVSLHGQLLWREFFYTVAATTPNFHKMEGNPVCLQVPWDTNQEYLQAW
KEARTGYPFIDAIMTQLKQEGWIHHLARHAVACFLTRGDLWISWEEGQKVFEEWLLDADYSLNAGNWMWLSAS
AFFHQYFRVYSPIVFGKKTDKNGDYIRKYLPVLKKMPSQYIYEPWTAPRTVQEKAGCIVGTDYPRPIVEHSVV
MKRNIERMKKARAEKYGNNNGPQKAISSPKTSSGGTKRKSKSKDDAGSSNKRTKKMTDFF
>Patiria_min_XP_038072863.1
MKRQQREQESQSGHLTDTTSTDEDALPQQEAKVQKLENDVPKSHESLTDMPTRPALGLDNKDRKKETIAVHWF
RHGLRLHDNPSLIDAINLASELYPIFIFDGEVAGTKVCGFNRWRFLYESLEDIDRKLKEVGGRLYTFNGDPCT
VFNQLIEEWGVNLITFEQDPEPIWQDRDNAVKRLCEERGIRCIESVSHTLWDPNEVTSRNGGTPPVTYAMFQE
VVSTIGLPHRPSGEPDFSSVKLSICDNFDQFALPHFQDIAPKMEHPDQERQLWIGGETRALELFEVRVRREAA
AFKAGYCLPNQYMPDLLGPPMSLSPYLRFGCLSVRKFYWRIHDTYTELNNTISPSHLTAQLIWREYFYTMSVG
NKNFDRMNGNNICLKIDWSEDQEKLQKWTEGETGYPWIDACMKQLKVEGWIHQVARHAVACFLTRGDLWISWE
DGLKVFYQYLLDADWSICAGNWMWISSSAFEKVLQCPNCFCPVRYGRRMDPNGEFVRRFLPVLVNMPLRYLFE
PWKAPLAVQEKAGCIIGKDYPMPIVEHRAAASSNREKMNRTVQSLEEKFSHCTPSNEVEVREFSWLPETIRGV
EKCTADDLCEILGI
>Patiria_min_XP_038073890.1
MGKRKRTRGSSRRGVSLVHWFRNKGLRLHDNPALREALVGAKTFRCVYILDPWFARSPNQGVNKWRYLLQCLE
DLDASLRKLNSRLFLVRGQPSDVFPKLFQEWGVNRLSFEEDPEVFGIQRDAKIKTLAATFKVQVITRMSHTLY
DPKEILKMNNNSPPLTYKHFQYLISLMDQPPFPEDTITLSDIEGINTAITSNHDSKYGLSSLEELGFDPEDIP
PPVWIGGETVAKQQLDRHLERKAWVANFERPRMSSASLMACPTALGPYLRFGCLSPRLFYWRLTELYQKTKKT
NNIPLFLHGQLYWREFFFTLGYDNPKLDEMVGNPICIQIPWEKNEEALQRWRNGQTGFPWIDAIMTQLKQEGW
VHPVARHAVACFLTRGDLWISWEEGAKVFEDLLLDADWSVNAGNWIWLSCSSFYQQFFHCYTFCPIKFGQQTD
PNGDFIRKYIPALKNFSAKYICKPWTAPLEVQEQAKCIIGKDYPFPMVDHNEVMRRNTERMKNILQNVSQAPN
YSYNEYITSIPSLPQSSNSIWVPSMSQEPQPKMLRLSQDSMPMMTQDNMMSQNNMMSQNNMMPQDNMMPQENM
MSQDNMMSQDSMMSQDNMLSQDSMAMLSQDDVPSTSAYIVTNPSGMLTLTNPYACTMTQESMMAPQPSQNALG
TSWNDPNSLANQYYNQ
>Patiria_min_XP_038075073.1
MATKYRTVICLLRNDLRYHDNEVLHWAHQNADYILPLYCLDPRHYHGTWHFSFPKTGPHRLKFLLESLRDLKE
TLRQVGSNLVISRGKPETVIQDLIRQLGQENVKAVALQQEATREELDVEAAIEKSCQVPVHKFWGSTLYHKDD
IPFRIRDLPNVFTEFRKKTENRSDVRPTLPMPSKLKPLPPGIDPGHAPSVEEMGMKDPARDERTAFPYSGGEK
QALARIQHYFWDYDYVSSYKETRNGLIGADYSTKFSSWLALGCLSPRKIYEEIRKYEKERRANQSTYWVVFEL
LWRDYFKFVALKFGDRLFYLSGLLGNHREWRENKALFDAWREGRTGVPFVDANMRELAATGFMSNRGRQNVAS
FLTKDLKLDWRLGAEWFEHLLIDHDVCSNYGNWLYCAGVGNDPRQDRKFNMIKQGLDYDAQGDYVRLWVPELQ

GIKGGAVHTPWILSSSMLIQANVRLGHSYPLPVVKAPEWNKHMRPQERGKSGKGPGQRQQTRGIDFYFSSGKQ
GKFKK
>Pecten_max_XP_033735988.1_cry_DASH-l
MKHTAARGTSFITSQNLESHYQRTNRSSWNLTMSSKVIICLLRNDLRLHDNEVLHWAYKTAEYVLPIYCFDPR
HFQGTYHFNFPKTGAHRAKFLLESAADLQNTLKSKGSGLVIRMGKPEEIVPQIIQTVGQDKVSYLCYQQEVTS
EELDVESKLQKTCGVKVQTFWGHTLYHRDDLPFEIQQLPDVYTQFRKSVEGQARVRKSFTLPDTLKPLPPGVE
DGQLPTLKDLGLKDLTEDPRSVISFQGGESVALDRLNHYLWQSDSVSTYKETRNGLVGSDYSTKFSVWLAHGC
ISPRHIYWEIKRYEAERVANQSTYWVIFELLWRDYFRFVALKYGKKLFFEGGIQGKNIPWKQDQNLLKAWIEG
RTGVPFVDANMRELAATGFMSNRGRQNVASFLTKDLKLDWRMGAEWFESQLIDHDVCSNYGNWLYSAGIGNDP
REDRKFNMVKQGLDYDPEGDYVRLWLPELNKVSGGAVHTVWTLSPASLAKAEVSLGETYPNPIVVAPEWSRHI
GKSKEGAHGGGSSRPTTQKQNRGIDFYFKGSQ
>Pecten_max_XP_033739658.1_cleavage_s
MAQQGSTQTTGVLPPEQHGYIPDKLKKAEKRIEAHPYDTEAWSVLIRDAQMKTIEVARQVYERLVAQFANAGK
YWRIYIEQEIKAKNYERVEKLFQRCLVKVLSIDLWKLYLTYIKDTKGSLPSYREKMAQAYDFALDKMGMDIMS
YQLWVDYINFLKSVDAVGSYAENQRITAVRKVYQRGVVNPMINIEALWKDYCAYENNINPLIAKKMTEDRGRD
YMNARRVAKEYEAVTRGLNKSLPSIPPSNSPEEAQQVELWKKYISWEKGNPLRTEDHALITRRVMFAYEQSLL
CLGHHPDIWYEAASYLEESSKILTEKGDQNAGKMFADEAAAVYERAITTLMKNNMLVYFAYADFEESRMKYEK
VHSIYKRNLNVPDVDPTLSYIQYMKFARRAEGIKSARSVFKMGREDNRTKYQVFVAAALMEYYCSKEKNVALK
IFELGLKKYGECPEYVLCYMEFMSHLNEDNNTRVLYERVLSSGQIPPQKSIEIWSRFLAFESEVGDLACIQKV
EKRRAQAIEKVQEFEGKDTALLIDRYKYLDLYPCTTSELKAVGYYDLAKQQVVQLPSNSVTKVVMEEEEGKKA
PEYPKPDVEQMLPFKPRTVIPNGSHIVDGGVFPPPPSAANLMTQLPPPECFHGPFVVMDAFIEHMKKLVLPDV
PIPQENGIDTKPDTGTQFSIDLAMGRKRKITEGEESDEERKQILHWFRKGLRLHDNPALRDALKGSSTYRCVY
ILDPWFAGSSQVGINKWRFLLQCLEDLDCSLRKLNSRLFVVRGQPTNVFPHLFKEWNITTLSFEEDPEPFGKE
RDGAIKALASEAGVETIVRISHTLYDLQKIINLNYGVSPLTYKRFQMFLSKMDPPKQPVDTINAALLGKCRTP
LSEDHDEHYGVPTLEELGFDTEGLKPAEFRGGEMEALSRLERHLERKAWVASFEKPKMTAQSLFPSQTALSPY
LRFGCLAPRLFYWKLAELYRKVKKQSDPPLSLHGQLLWREFFYTAATNNPNFDQMKDNPICVKVPWDHNPEAL
AKWAEGMTGYPWIDAIMRQLKQTGWIHHLARHSVACFLTRGDLWISWEEGMKVFDELLLDADWSVNAGMWMWL
SCSSFFQQFFHCYCPVGFGKRADPTGDFIRSYIPELKNYPSKYIFEPWNAPQSVQKAAKCVIGKDYPVPIIRH
SDVSKINMERMKQVYQLLAQRASAVSSIPKHPEDLTNKTTMALTGAHSGNHPTRGPACCNAAAQQTNIEDNDQ
GIGSTFAVYTSAS
>Pecten_max_XP_033740162.1_cry-1-like
MEEEPQKQKIVAIHWFRNGLRIHDNPALCDALENCDEFYPIFIFDGEVAGTKFSGFNRMRFMHESLEDLDNTF
KKNGGRLYTFQGKARDVLEGLLHEWNVTRLTYEAEVEPIWETRDKEVEELCQEHGIQLISRISHTLWNPSEVI
EKNGGSPPVTYSHFELTTALLGPPERPVPVPNLTKVNKPVPDNFDQKFALPSVKDLGYEPECKEQMERINEWK
GGETKALQLLEERLKNERKPMTDKIMLPNQFLPDLVGPPTSMSPHLRFGCVSVRTFYWGIHDLYQEIHADKEV
PVSLTGQLVWREYFYTMSVSNIHYDKMKENPICLDIAWDEDAEKLEKWVMGKTGFPWIDAGMNQLRAEGWCHH
VVRHAVSCFLTRGDLWINWVQGCQVFFKYQLDADWSVCAGNWMWMSSSAFEKVFQCSTCFCPVGYGRRMDPKG
EYVRRYVPVLKDMPIQYLFEPWKAPYFIQEKANCIIGKDYPRPMLDHKQASMYCRSKMMEVHRRLASSSPIPH
CAPSNSKEANFFSWLPESEVLSLCNHTQHLWPENKSVGIRS
>Pecten_max_XP_033752615.1_LOW_QUALIT
MAKKSIHWFRKGLRIHDNPSLVAACENAADIKPIFILDPWFQKNGNVGINRWRFLLQSLQDLDSNLRKINSRL
FVIKGKPQEVLANLFKKWNITTLTYEVDTEPYAKLRDADVDDLARECGVEVIKCVSHTLYDTKRIVDKNGGST
PLTYKRLQTVVSMLGPPPKPVPEPSKCVTSVSPNHDQNYGIPTLEDLDKDLTDCGPCLFPGGETEALNRLDRM
MKKTNWVCTFEKPKTEPNSLQPSTTVLSPYLKFGCLSPRTMYYRLVDIYKHNKKHTAPPVSLDGQLLWREFFY
TVGIGHPKFDQMVDNPICIQIPWDSNEEFLDAWKQGRTGFPFIDAIMTQLKQEGWIHHLARHAVACFLTRGDL
YISWEEGKKVFEEYLLDADWSLNAANWMWLSASSFFYQYFRVYSPIAFGKKTDKDGSYIRKYVPVLKNMPTQY
IYEPWTAPMKVQEKAGCVIGRDYPKPIVEHDKIRPINIQRMAKAYKNRPDKADKSPGKGQKRKLEQNSPKPKK
SKTTSKNSKITDFVKGGK
>Pecten_max_XP_033760904.1_photolyase
MSRDQRVQDNWAMLYAQRLALKLKVPLVVCFCLVPKFLEATIRHYGFMLKGLQEVEQECRSLNINFHLLQGYA
KDVLPGFVEDNDFGGVVTDFSPLRVPVGWVNDVKERLPKKVPFCQVDAHNIVPCWEASPKLEYGARTIRNKIH
NQLSGFLTEFPPVCEHPHKSKISPQSVDWESVRGSLEVDQSVPEVSWATPGANGGLAMLESFCNDRLRYFSSD
RNNPNKNALSNLSPWIHFGQISVQRCILTVRKYRSKSSEGVNAFIEEAVIRRELSDNFCYYNKNYDSVEGAYD
WAKQTLKIHASDKREFLYTRDQLAAGKTHDDLWNAAQIQMVKEGKMHGFLRMYWAKKILEWTESPEVALETAI
YLNDRFSLDGRDPNGYVGCMWSICGIHDQGWKEREIFGKIRYMNYAGCKRKFDVPAFVIKNMAKKK
>Pediculus_hum_XP_002430545.1_photolyase
MSDNSSQNSGKHTVHWFRKGLRLHDNPSLREGIKNSVTFRCVFVIDPWFAGSSNVGINKWRFLLQCLEDLDRS
LRKLNSRLFVIRGQPADTLPKLFKEWGTTNLTFEEDPEPFGRVRDLNIMAMCKELGISVVSKSSHTLYKLEHI
IEKNGGNPPLTYHQFQTIIANIGPPPEPEETVTANLLEGSQTPLSDDHDNKYGVPTLEELGFETDKLKPTVWS
GGESEALARLERHLERKAWVASFGHPKMTPQSLLASQTGLSPYLRFGCLSTRLFYYQLVELYKKIKKAMPPLS

LHGQLLWREFFYCAATKNPNFDKMNENPICVQIPWDENAQALAKWANGQTGFPWIDAIMTQLREEGWIHHLAR
HAVACFLTRGDLWISWEEGMKVFDELLLDADWSVNAGMWMWLSCSSFFQQFFHCYCPVRFGRKADPNGDYIRK
YLPILKNFPTKYIHEPWLAPETEQRAAKCIIGKDFPLPMVNHSIVSKINIERMKQVYRQLSKYRSQGT
>Pelecanoides_urin__NXT31242.1
RFLLQSLEDLDNSLRKLNSRLFVVRGQPTDVFPRLFKEWGVTRLTFEYDSEPFGKERDAAIVKLAKEAGVEVV
IENSHTLYDLDRIIELNGHKPPLTYKRFQAIISRMELPKKPVSTVTSQQMETCKVDIQENHDEVYGVPSLEEL
GFPTDGLAPAVWQGGETEALARLDKHLERKAWVANYERPRMNANSLLASPTGLSPYLRFGCLSCRLFYYRLWE
LYKKVKRNSTPPLSLYGQLLWREFFYTAATNNPKFDRMEGNPICIQIPWDRNPEALAKWAEGKTGFPWIDAIM
TQLRQEGWIHHLARHAVACFLTRGDLWISWESGVRVFDELLLDADFSVNAGSWMWLSCSAFFQQFFHCYCPVG
FGRRTDPSGDYVKRYLPKLKGFPSRYIYEPWNAPESVQKAAKCIIGVDYPKPMVNHAETSRLNIERMKQIYQQ
LSRYRGLCLLASVPSCVEDLSGPVADSASGQGCSTSTAMRLSQADQASPKRKHEGAEELCTEELYKRAKVTCL
PAPEIPGKSL
>Pelecanoides_urin__NXT33719.1
MPHRTIHLFRKGLRLHDNPTLLAALESSEVIYPVYILDRKFMTSVMHIGALRWHFLLQSLEDLQKNLCQLGSC
LLVIQGEYESVLRDHIQKWNITQVTLDAEMEPFYKEMEANIRRLGEELGFEVLSLVGHSLYDTKRILDLNGGT
PPLTYKRFLHILSLLGDPEVPVRNLTAEDFQRCRPPEPGLAECYRVPLPVDLKIPPESLSPWRGGETEGLQRL
EQHLTDQGWVASFTKPRTIPNSLLPSTTGLSPYFSLGCLSVRTFFYRLSNIYAQAKHHSLPPVSLQGQLLWRE
FFYTVASATPNFTKMAGNPICLQISWYEDAERLHKWKTAQTGFPWIDAIMTQLHQEGWIHHLARHAVACFLTR
GDLWISWEEGMKVFEELLLDADYSINAGNWMWLSASAFFHQYTRIFCPVRFGKRTDPEGQYIRKYLPILKNFP
SKYIYEPWTASEEEQKQAGCIIGQDYPFPMVNHKEASDHNLQLMKQVREEQYRTAQLTR
>Pelecanoides_urin__NXT34709.1
MQHSSIHWFRKGLRLHDNPALLAAAADCHRLHPLFILDPSSSRTGTNARRFLLDALRDLDGSLREIGSRLFVV
RGCPEEVFPRLFRAWRTTRLTFEVDTEPPARRRDTAVAELAARHGVEVIREVSHTLYDTERVLALNDGKAPLT
YKRLQSLLASLGPPEKPAPALTREHLQGCRTPCQVSHDADFGVPTLEELGQDPAEVGPHLYPGGETAALARLD
AFMKRTAWVCGFKKPETEPTSLSPSTTVLSPYLKFGCLSVRTFWWRLDEVYQGREHSRPPVSLHGQLLWREFF
YTAGAGIPNFDRMVGNPVCLQVDWDDNPQHLRAWREGRTGYPFIDAIMTQLRTEGWIHHLARHAVACFLTRGD
LWVSWEEGLKVFEELLLDADWSLNAGNWLWLSGSAFFHRYFHVYSPIAFGKKTDRDGAYIRKYLPVLKDFPAK
YIYEPWKAPRAVQERAGCLVGTHYPRPIVEQR
>Pelecanoides_urin__NXT35300.1
KVDYDVCSNYGNWLYSAGIGNDPRDNRKFNMIKQGLDYDGNGDYVRLWVPELQAIKGADIHTPWALNSAALSQ
AGVTLGETYPQPVVTAREWSRHINQRPQGRSPHPRGRRGPAHTPVQHKDRGIDFYFSRNKDI
>Pelecanoides_urin__NXT37061.1
MGVNAVHWFRKGLRLHDNPALRECIQGADTVRCVYILDPWFAGSSNVGINRWRFLLQCLEDLDANLRKLNSRL
FVIRGQPADVFPRLFKEWNIAKLSIEYDSEPFGKERDAAIKKLASEAGVEVIVRISHTLYDLDKIIELNGGQP
PLTYKRFQTLISRMEPLEMPVETITPEVMEKCTTPVSDDHDEKYGVPSLEELGFDTDGLPSAVWPGGETEALT
RLERHLERKAWVANFERPRMNANSLLASPTGLSPYLRFGCLSCRLFYFKLTDLYKKVKKNSSPPLSLYGQLLW
REFFYTAATNNPRFDKMEGNPICVQIPWDRNPEALAKWAEGRTGFPWIDAIMTQLRQEGWIHHLARHAVACFL
TRGDLWISWEEGMKVFEELLLDADWSVNAGSWMWLSCSSFFQQFFHCYCPVGFGRRTDPNGDYIRRYLPVLRG
FPAKYIYDPWNAPESVQKAAKCIIGVNYPKPMVNHAEASRLNIERMKQIYQQLSRYRGLGLLATVPSNPSGNG
NGGLMGYSPGESISGCGSTGAQLGTGDGHTVVQPCALGDSHTGASGIQQQGYCQASSILHYAHGDNQQSHLLQ
AGRTALGTGISAGKRPNPEEETQSVGPKVQRQSTS
>Pelecanoides_urin__NXT42939.1
DNWAFLYAQRLALKQELPLHVCFCLVPKFLDATIRHYGFMLRGLQEVAKECTELNIPFHLLLGYAKDVLPAFV
VEHGVGGLVTDFCPLRLPRQWVEDVRQQLPDDVPFAQVDAHNIVPCWVASPKQEYSARTIRGKIHAQLPEFLT
EFPPVIRHPYLPSCPAEPIAWEVCYSSLQVDRTVKEVEWATPGTAAGLAVLQSFIMERLKSFCSHRNDPNKAA
LSNLSPWFHFGQVSTQRAILEVQKHRGKYKESVDAFVEEAVVRRELAENFCYYNENYDSVQGAYDWAQTTLKL
HAKDKRPFLYDLQELEQGTTHDPLWNAAQLQMVQEGKMHGFLRMYWAKKILEWTRSPEEALRFAIYLNDRYEL
DGRDPNGYVGKLQDGGCLWSICGIHDQGWAERAIFGKIRYMNYAGCKRKFDVGQFERRYAPH
>Pelodiscus_sinensis_XP_006115758.1_cryptochro
MSNMLHRTIHLFRKGLRLHDNPTLLAALESSEVVYPVYILDRKFLMSAMHIGTLRWHFLLQSLEDLHRSLTKL
GSCLLVIQGEYESVLRDHVEKWNITQVTLDAEIEPFYKEMEQAIQALGGEMGFDVLSSMGHTLYDIRRILDKN
GGTPPLTYKRFLHILSVLGDPNAPVRNLTAEDFQRCKPPSALGLVEGYQVPRPEDLGIPQQSQSPWRGGESEG
LQRLEQHLKNQGWVASFTKPKTIPNSLLPSTTGLSPYFSLGCLSVRSFFYRLSNIYAQSKNHSLPPVSLQGQL
LWREFFYTVASATPNFTKMVGNPICLQINWYQNAEGLHKWRMAQTGFPWIDAIMTQLHREGWIHHLARHAVAC
FLTRGDLWISWEEGMKVFEEMLLDADYSINAGNWMWLSASAFFHQYTRIFCPVRFGKRTDPEGQYIRKYLPVL
TNFPSKYIYEPWTAPEEAQKQAGCIIGQDYPFPMVNHKEASARNLQLMKQVREEQYGTAQLTRDDMDDPMEMG
LKHELSEENPKKGKRARNREQT
>Pelodiscus_sinensis_XP_006124563.1_cryptochro
SLSYTQTTAADCLFPHHRFLLQSLEDLDNSLRKLGSCLFVVRGQPTDVFPRLFKEWGVTRLTFEYDSEPFGKE
RDAAIIKLAKEAGVEVVIENSHTLYDLDRIIELNGHKPPLTYKRFQTIISRMELPKKPVSSITSQQMEKCKSE

IQDNHDDTYGVPSLEELGFPTDGLAPAVWKGGETEALARLDKHLERKAWVANYERPRMNANSLLASPTGLSPY
LRFGCLSCRLFYYRLWELYKKVKRNNTPPLSLYGQLLWREFFYTAATNNPKFDRMEGNPICIQIPWDRNPEAL
AKWAEGKTGFPWIDAIMTQLKQEGWIHHLARHAVACFLTRGDLWISWESGVRVFDELLLDADFSVNAGSWMWL
SCSAFFQQFFHCYCPVGFGRRTDPSGDYVRRYLPKLKGFPSRYIYEPWNAPESVQKAAKCIIGVDYPKPMVNH
AETSRLNIERMKQIYQQLSRYRGLCLLASVPSCVEDLSNPVTDSTAGQGSSTTTAQRLLHCGQTSPKRKHEGI
EELCAEELYKRAKVTGLHISEIPSKNL
>Pelodiscus_sine_XP_014427726.1
IVVQKEDTWGTDIINAPLYFQVLHWAQNNADYIIPLYCFDPRHYLRTYHYGFPKTGPYRLKFLLESVKDLRQT
LKKKGSNLVVRKGTPEDVVHEFITQLGSVTAVAFHEEATTEELDVEKALQRVCAHHGVKIQTFWASTLYHRDD
LPFSHISRLPDVYTQFRKAVESQGKVRPTVQMADQLKPLAPGVEDGCIPMPEEFGLKEPLADPRTAFPCSGGE
TQALMRLQHYFWDTNLVASYKETRNGLIGIDYSTKFAPWLALGCISPRYIYEQIRKYEKERTANQSTYWVIFE
LLWRDYFRFVALKYGTRIFSLKGLQDKEVPWKKDPKLFDAWKEGRTGVPFVDANMRELAATGFMSNRGRQNVA
SFLTKDLGLDWRMGAEWFEHLLVDYDVCSNYGNWLYSAGVGNDPRENRKFNMIKQGLDYDSNGDYVRLWVPEI
QCIKGGEIHTLWALSNAALSQAGIALGETYPLPVVLAREWSRHINQKPNKRDHPPRGKKDPSHTPKQHKDRGV
DFYFSRNKDVS
>Pelodiscus_sin_XP_025034720.1_cry-1
MPITHGKRWISSKMKLPAEVHQEFGAMTTEGRTITRTALQASLDAVDSAARTMASGVVMYRGAWLQVSGIPPD
VQTTVQDLPFDGLNYFRKINVGLFFLLHRFLLQCLEDLDANLRKLNSRLFVIRGQPADVFPRLFKEWNIAKLS
IEYDSEPFGKERDAAIKKLASEAGVEVIVRISHTLYDLDKIIELNGGQPPLTYKRFQTLISRMEPLEMPVETI
TAEVMEKCTTPVSDDHDEKYGVPSLEELGFDTDGLPSAVWPGGETEALTRLERHLERKAWVANFERPRMNANS
LLASPTGLSPYLRFGCLSCRLFYFKLTDLYKKVKKNSSPPLSLYGQLLWREFFYTAATNNPRFDKMEGNPICV
QIPWDRNPEALAKWAEGRTGFPWIDAIMTQLRQEGWIHHLARHAVACFLTRGDLWISWEEGMKVFEELLLDAD
WSVNAGSWMWLSCSSFFQQFFHCYCPVGFGRRTDPNGDYIRRYLPILRGFPAKYIYDPWNAPESIQKVAKCVI
GVNYPKPMVNHAEASRLNIERMKQIYQQLSRYRGLGLLATVPSNTNGNGNGGLMGYSPGENISGCSTTGGAQM
GSNDGHTGGIQTCALGDSHAGSSGIQQHGYSQGSSILHYAHGDNQQSHLLQQGRTGSTGISTGKRPNPEKETQ
STGLKVQRQSTN
>Pelodiscus_sin_XP_025039643.1_LOW_QUALIT
MNEGAAQAGSMKHCSIHWFRKGLRLHDNPALLAAMRDSRQLYPIFILDPWFPKNMRVSVNRWRFLVESLRDLD
ESLKKLNSRLFVVRGRPMEVFPGLFKEWKVTRLTFEIDTEPYSRQRDAEVVRLAAEHGVEVIQKVSNTLYDTD
RIIAENNDKVPLTYVRLQTLVAELGPPKRPVPAPTLENLKDCRTPCGDNHDAEYGIPTLEELGQDPTQAGPHL
YPGGESEALGRLDLHMNRTAWVCNFQKPQTEPNSLSPSTTVLSPHLKFGCLSVRTFWWRLADVYQGRKHSSPP
VSLHGQLLWSELXSRMEGTPVCLQVDWDDNPEHLRAWREGRTGYPFIDAIMTQLRTEGWIHHLARHAVACFLT
RGDLWISWEEGQKVFEELLLDADWSLNAGNWQWLSASAFFHQFFRVYSPVAFGKKTDKDGEYIKKYLPFLRQF
PAAYIYEPWKAPRSMQEQAGCVIGRDYPKPIVVHEVVSKRNVERMKAAYARRSASTTAQLEGGSGKKGAKRKL
PAAPSVAELLTKKPKTKS
>Pelodiscus_sin_XP_025045251.1_LOW_QUALIT
NSGCVPPQRWKSDSCWARGDSALGWESERGCKVRCEIPAGGTDRLLATGNSSRRLQGNIPERLRKGGVSLFTD
RFTPAGCCWEGWPELWSLQLAGVAAGGCRSKARLPGRLPACLRRGGAGRPGGGRAPPPRGRLTGKRKGPVEPS
GAAAVAVEKRRKEGEVTEAGGAGCLGEAVRQARLRAAPSVQEFKYNKKRVRLVSQGPDLKEDAQGIVYWMSRD
QRVQDNWAFLYAQRLALKQKLPLHVCFCLVPXHFGFMLKGLQEVAEECKELAIPFHLLVGFAKDVLPPFVTGR
GLGGXGAPLRVPAQWVEDVRERLPGDVPFVQVDAHNIVPCWVASDKQEYGARTIRRKIHDRLSEFLTEFPPVI
KHPYPPTAPAEPIDWSACRAGLQVDHTVKEVTWATPGTAAGLAMLESFIGERLKFFGTDRNNPNRAALSNLSP
WFHFGQVSVQRAILEVRKHRGQCKESVEGFVEEAVVRRDAAESRCALARQELQTGAGFSLWRQPESTLSRGKP
RPGPGRPHPRGSGFSSPPPELPGLPGAGCGSPGCPRWALPRLQLQMVHEGKMHGFLRMYWAKKILEWTRSPEE
ALKFAIYLNDRYELDGRDPSGYVGCMWSICGLHDQGWAERAVFGKIRYMNYAGCKRKFDVGEFERRYHPRKLG
Q
>Penaeus_mono__XP_037782690.1
MMTKTSVHWFRHGLRLHDNPALLESVKDAEKFYAIFIHDGETAGTEVSGYNRMQFLTEALYDLDAQLRAVGGQ
LHIFKGKPADVIKMLHTEIRITRLTFEQDCEAIWNKRDNQVRNQCKELGIEIVEKISHTLWDPFEIIETNGGQ
PPLTYEMFVQVSAVLGPPNRPVPDVNWNEVIFGEISDELAMKLQLCPHIPTPGELGWNKECDEQSAYVGGETA
ALAHLQKRLKVEENAFRDCYILPNQVNPDILGEPMSMSAALRFGCLSVRKFYWNIQDIYFELHKEIKPPFHSL
TAQLIWREFFYCMSANNESYNKMKGNPICIDIPWRKDDDHLQAWTEGRTGYPFIDACMRQLRKV
>Penaeus_mono__XP_037784691.1
MISCSRSVGGRVLKLQRNHFHQNVSKLREISSGKVTPGKMVSTNKSVAFCWLRNDLRYHDNEILTKANINADH
VVPVYCFDPRHFKGTYHFGFPKTGPHRAKFLKESVEDLKATLRNRGSDLIVRFGKPEEVLPFLIKSVAATTSH
LVFQEEVTKEEKDVERSVRDRCKDAGVNVHSFWGATLYHKTDLPFPIDKVPDTYTNFRKAVEAQSRVRGVCNM
PDKLKPLPENLDVGEIPTLSKLGVEELPMDHRTAFPFAGGETAGLARVKNYLWDTNNVANYKETRNGLLGEAY
STKFSPWLAHGCLSPRYIFNEIKKYESQRVANKSTYWVIFEMIWRDYFRFVCMKFGDRVFYPSGIMGKKVQWK
RNAQLFEMWKGKVKEIVIGILVYGVGGLCLCYLLFDFCLFXLDHDVCSNYGNWNYAAGIGNDPRENRKFNMIK

QGLDYDPEGNFIRVWVPELKNIKNGRIHTPWKMNIAELTNAGVSIGEDYPKPVVVAPEWGRHDHKMNNSNSNK
KQKGNWMPPGQKRGIDFYFSGNRK
>Penaeus_mono__XP_037785843.1
MSLLVNIHRTFQVIDRFSRGFIRSRTLNISTFYKVSCNIQCRDFASIRSMASDEPTPKKAKIDDDALNIHKRL
KKERYEEAEDVSDFKFNKKRVEVISKEGEVPDDCDGIVYWMSRDQRVQDNWALLFAQRLALKNKVSLHVVFCL
VPKFLDATIRHFHFMLRGLEEVEKECKELNIEFHLLLGEAKDVLPTFIKENKIGGLVTDFSPLRVPQQWLKDV
KKAIPSSIPMCKVDAHNIVPCWITSDKQEYSARTIRKKIHDKLPKFLTKFPPVAKHRYTSKYKSEGVDWVEAE
KSLEVDQTVKPVDWATPGTRAGLDLLNEFCLKRLRKFADSRNDPTINALSNLSPWIHFGQISAQRCVLEVKRY
SKQLSKSVEAYVEEAVVRRELSDNYCFYQKNYDKLDGAYEWARKTLEDHRNDKRPYVYTRETLEEGKTHDELW
NSAQIQLVTEGKMHGFLRMYWAKKILEWMKSPDDSLATAIYLNDKYSLDGRDPNGYVGCMWSICGIHDQGWGE
RQVFGKIRYMNYDGCKRKFKIDAYVARYGGKKHKYIPAV
>Penaeus_mono__XP_037792362.1
MTGKVNEPRPKHMVHWFRKGLRLHDNPSLRAGLRNAATFRCIFILDPWFAGSSNVGINKWRFLLQCLEDLDSS
LRKLNSRLFVIRGQPADVLPQLFREWGTTCLTFEKDPEPFGKARDANIVAIAREMGVNVVMKTSHTLYKPEKI
IEKNGGKPPLTYKSFQNILMNMDLPPLPQGTITSEEVEDKQTPISDDHDEKYGVPSLEELGFDTEQLQQTVWK
GGESEALTRLEHHLERKAWVASFGRPKMTPQSLYPSRTGLSPYLRFGCLSCRRFFMELNELYRKIKKSPPPLS
LHGQLLWREFYYTAATNNPKFDHMEGNPICVQIPWDKNPEALAKWANGQTGYPWVDAIMTQLRTEGWIHNVAR
HAVACFLTRGDLWVSWEEGMKVFDELLLDADWSVNAGSWMWLSCSSFFQQFFHCYCPVRYGRKADPNGDFIRT
YLPVLKNFPTKYIHEPWTAPESVQRAAKCIISRDYPMPMVDHIKQSQNNIERMKQVYQQLVHYRGTIHPAIKS
PKVDHYPPPPPDYEKPRSNKNGKNDIQRDSNYRVQVQA
>Penaeus_mono__XP_037792441.1
MTTIHWFRKGLRLHDNPALLAAIDESKELRPVFVLDPWFVRESRTGVNRWRFLAQTLVDLDTSLKQLGSRLFV
VRGKPEEVFPKLFKEWNVEKMTWEVDTEPYARIRDEKIEKLSKEHGVKVVCKVGHTLYDTEKIIRTNLGKTPM
TFQGLEGVLKKIGPPPKVVDKPTSLPEGCKISSEVAADPKYDPPTLKDLGVVESDLEPCLYPGGETEALKRLE
KYMERKSWVRSFEKPKTSPNSLQPSTTVLSPYLKFGCLSCRTMYYRLLEVYGSKPSQIAQESLLGQLTFREMF
YTVGATTANFDQMEGNSICRQIPWKRDEKMLEAWEYGKTGFPFIDAIMTQLRKEGWVHHLARHAVACFLTRGD
LWQPWEDGMKVFEELLLDADWSLNAGNWMWLSASAFFHRYFRVYSPIEFGRKTDPHGDYIRKYIPKLKKFPDN
YIYEPWKAPLSVQKAAGCIIGQDYPRPIVDHQKAREENLKRMGQAYQADENANVGPLDIQSVEVIVRE
>Penaeus_van_ROT83202.1_putative_c
MAKVREDSKKRDSSRFTFCSPSARGTSHVTLRAPSGTAPRKGGGGRGREGGDGRRRNSLTQTRRGGGRKDCIL
ARLVPRARLSMTTIHWFRKGLRLHDNPALVAAIDENKELRPVFVLDPWFVRESRTGVNRWRFLAQTLVDLDTS
LKQLGSRLFVVRGKPEEVFPKLFKEWNVEKMTWEVDTEPYARTRDEKIEKLSKEHGVKVVCKVGHTLYDTEKV
IRTNLGKTPLTFQGLEGVLKKIGLPPKVVDRPTSLPEGCKISSEVASDPKYDPPTLKDLGVVESDLEPCLYPG
GETEALKRLEKYMERKSWVRSFEKPKTIPNSLQPSTTVLSPYLKFGCLSCRTMYYRLLEVYGSKPSQIAQESL
LGQLTFREMFYTVGATTANFDQMEGNAICRQIPWKRDEKMLEAWEFGKTGFPFIDAIMIQLRKEGWIHHLARH
AVACFLTRGDLWQPWEDGMKVFEELLLDADWSLNAGNWMWLSASAFFHRYFRVYSPIEFGRKTDPHGDYIRKY
IPKLKKFPDNYIYEPWKAPLSVQKAAGCIIGQDYPRPIVDHQKAREENLKRMGQAYQAGK
>Penaeus_van_XP_027213306.1_cry_DASH-l
MISCSRSVGGRVLKLQRNHFHQNVTKLREASSIKVTPGKMVATNKSVAFCWLRNDLRYHDNEILTKANINADH
VVPVYCFDPRHFKGTYHFGFPKTGPHRAKFLKESVEDLRATLRNRGSDLVVRIGKPEEVLPFLIKSVAATTSH
LVFQEEVTKEEKDVERSVRDRCKDAGVNVHSFWGATLYHKTDLPFPINKVPDTYTNFRKAVEAQSRVRGVCNM
PDKLKPLPENLDLGEIPTLSKLGVEELPMDQRTAFPFSGGETSGLARVKNYLWDTNNVANYKETRNGLLGEAY
STKFSPWLAHGCLSPRYIFNEIKKYESQRVANKSTYWVIFEMIWRDYFRFVCMKFGDRVFYPSGIMGKKVHWK
RNAQLFEMWKDGRTGVPFVDANMRELKETGWMSNRGRQNVASFLLKDLGLDWRLGAEWFESQLLDHDVCSNYG
NWNYAAGIGNDPRENRKFNMVKQGLDYDPEGNFIRVWVPELKNIKNARIHTPWKMNIAELTNAGVSLGEDYPK
PVVIAPEWGRHDHKMNNSNSNKKQKGSWMPPGQKRGIDFYFSGNGK
>Penaeus_van_XP_027216548.1_cry-1-like
MTKTSVHWFRHGLRLHDNPALLESVKDCEKFYAIFIHDGETAGTEVSGYNRMQFLTESLCDLDEQLRAVGGQL
FVFKGKPTAVIKMLHAEIGITRLTFEQDCEAIWNKRDNQVRSQCKELGIEIVEKIAHTLWDPFEIIDCNGGQP
PLTYEMFVQVSAVLGPPERPVPDVKWDDVIFGEISDELAMKLQLCPHIPTPAELGWNRECDEQSAYIGGETAA
LAHLQKRLKIEENAFRDCYILPNQVNPDILGEPMSMSAALRFGCLSVRKFYWNIQDIYFELHKEIKPPFHSLT
AQLIWREFFYCMSANNEAYNRMKGNPICIDIPWRKDDEHLKAWTEGRTGYPFIDACMRQLRKEGWIHHVCRTA
VACFLTRGDLWISWEDGLHVFLKYLIDADWSVCAGNWMWVSSSAFERQLDCSTCICPVNYGKRIEPTGDYIRR
YVPELASLPQDYIFEPWLAPKSVQEAYKCIIGQDYPERIVIHAEASKANRKMMESIREKMAVKPPHCCPSNVK
ETRVFLRLPEACYHNVL
>Penaeus_van_XP_027226418.1_cry-1-like
MTGKVNEPRPKHMVHWFRKGLRLHDNPSLRAGLRNAATFRCIFILDPWFAGSSNVGINKWRFLLQCLEDLDSS
LRKLNSRLFVIRGQPADVLPQLFREWGTTCLTFEKDPEPFGKARDANIVAIAREMGVNVVMKTSHTLYKPEKI
IEKNGGKPPLTYKSFQNILMNMDLPPLPQGTITSEEVEDKQTPISDDHDEKYGVPSLEELGFDIEQLQQTVWK
GGESEALTRLEHHLERKAWVASFGRPKMTPQSLYPSRTGLSPYLRFGCLSCRRFFMELNELYRKIKKSPPPLS

LHGQLLWREFYYTAATNNPKFDHMEGNPICVQIPWDKNPEALAKWANGQTGYPWVDAIMTQLRTEGWIHNVAR
HAVACFLTRGDLWVSWEEGMKVFDELLLDADWSVNAGSWMWLSCSSFFQQFFHCYCPVRYGRKADPNGDFIRT
YLPVLKNFPTKYIHEPWTAPESVQRAAKCIISRDYPMPMVDHIKQSQNNIERMKQVYQQLVHYRGTIHPAIKS
PKVDHYPPPPPDYEKPRSNKNGKNDIQRDSNYRVQVQA
>Penaeus_van_XP_027232396.1_photolyase
MTEAEDVSEFKFNKKRVEVISKEGEVPDDCDGIVYWMSRDQRVQDNWALLFAQRLALKNKVSLHVVFCLVPKF
LDATIRHFHFMLRGLEEVEKECKDLNIEFHLLLGEAKDVLPTFIKEHKIGGLVTDFSPLRVPRQWLKDVKKAI
KPNVGLLQVDAHNIVPCWITSDKQEYSARTIRKKIHDKLPKFLTKFPPVAKHRYTSKYKSKGVDWVEAEKSLE
VDQTVKPVDWATPGTRAGLDLLNEFCLKRLRKFAESRNDPTINALSNLSPWLHFGQISAQRCVLEVKRYNKQL
SKSVEAYVEEAVVRRELSDNYCFYQKNYDNLDGAYEWARKTLEDHRNDKRPYIYTRETLEEGKTHDELWNSAQ
IQLVTEGKMHGFLRMYWAKKILEWMKSPDDSLATAIYLNDKYSLDGRDPNGYVGCMWSICGIHDQGWGERQVF
GKIRYMNYDGCKRKFKINAYVARYGGKQHRYIPPV
>Penelope_pile__NXC39494.1
DNWAFLYAQRLALKQELPLRVCFCLVPEGRDATLRHCAFMLRGLQEVAEECAELDVPFHLLLGCPRDVLPTLV
AQHGVGGLVTDFCPLRIPRRWVEEVRERLPEDVPFAQVDSHNIVPCWVTSPKQEYSARTIRGKIHSQLPEFLT
EFPPVVRHPHPLPGPAEPIAWDACYSQLQVDRTVTEVAWATPGTTAGLAVLQSFITERLEAFGSQRNDPNKAA
LSNLSPWFHFGQVSTQRAILEVQKHRRAYKESVDAFVEEAVVRRELAENFCYYNEHYDSVQGAYDWAQTTLKL
HAKDKRPFLYELPQLEQAATHDPLWNAAQLQMVQEGKMHGFLRMYWAKKILEWTRCPEEALHFAIYLNDRYEL
DGRDPNGYVGKLQGGMGCLWSICGIHDQGWKERNVFGKIRYMNYAGCKRKFDVDQFERRYAP
>Penelope_pile__NXC41228.1
MPHRTIHLFRKGLRLHDNPALLAALQSSDVLYPVFILDRAFLTSSMHIGALRWHFLLQSLEDLQNSLRRLGSC
LLVIQGEYGAVVRERVQEWSITQVTLDAEMEPFYKEMEANIRGLGEEMGFEVLSLAGHSLYDTERILELNGGT
PPLTYKRFLHVLSLLGDPEVPVRNLTAEDFQRCSPPEPGLAERYGVPLPADLKLPPESISPWRGGESQGLQRL
EQHLADQGWVASFTKPRTIPNSLLPSTTGLSPYFSMGCLSVRSFFYRLSNIYTQAKHHSLPPVSLQGQLLWRE
FFYTVASATPNFTKMAGNPICLQISWYEDAERLHKWKTAQTGFPWIDAIMTQLRQEGWIHHLARHAAACFLTR
GDLWISWEEGMKVFEELLLDADYSINAGNWMWLSASAFFHQYTRIFCPVRFGRRTDPEGQYIRKYLPVLKNFP
SKYIYEPWTASEEEQKQAGCIIGQDYPFPMVDHKEASDHNLQLMKRVREEQHRTAQLTRDDADDPMEMKLKRD
RSEEDITGAKAARMAE
>Penelope_pile__NXC42041.1
CIYILDPWFAASSAVGINRWRFLLQSLEDLDNSLRKLNSRLFVVRGQPTDVFPRLFKEWGVTRLTFEYDSEPF
GKERDAAIVKLAKEAGVEVVIENSHTLYDLDRIIELNGNKPPLTYKRFQAIISRMELPKKPVSSIVSQQMETC
KVDIQENHDDMYGVPSLEELGFPTDGLAPAVWQGGETEALARLDKHLERKAWVANYERPRMNANSLLASPTGL
SPYLRFGCLSCRLFYYRLWELYKKVKRNSTPPLSLYGQLLWREFFYTAATNNPKFDRMEGNPICIQIPWDKNP
EALAKWAEGKTGFPWIDAIMTQLRQEGWIHHLARHAVACFLTRGDLWISWESGVRVFDELLLDADFSVNAGSW
MWLSCSAFFQQFFHCYCPVGFGRRTDPSGDYVKRYLPKLKGFPSRYIYEPWNAPESVQKAAKCIIGVDYPKPM
VNHAETSRLNIERMKQIYQQLSRYRGLCLLASVPSCVEDLSGPVTDSASGQGSSTSTAIRLPQSDQASPKRKL
EGAEELCTEELYKRAKVTGLPAPEIPGKN
>Penelope_pile__NXC44637.1
RFLLQCLEDLDANLRKLNSRLFVIRGQPADVFPRLFKEWNIAKLSIEYDSEPFGKERDAAIKKLASEAGVEVI
VRISHTLYDLDKIIELNGGQPPLTYKRFQTLISRMEPLEMPVETITPEVMEKCTTPVSDDHDEKYGVPSLEEL
GFDTDGLPSAVWPGGETEALTRLERHLERKAWVANFERPRMNANSLLASPTGLSPYLRFGCLSCRLFYFKLTD
LYKKVKKNSSPPLSLYGQLLWREFFYTAATNNPRFDKMEGNPICVQIPWDKNPEALAKWAEGRTGFPWIDAIM
TQLRQEGWIHHLARHAVACFLTRGDLWISWEEGMKVFEELLLDADWSVNAGSWMWLSCSSFFQQFFHCYCPVG
FGRRTDPNGDYIRRYLPVLRGFPAKYIYDPWNAPESVQKAAKCIIGVNYPKPMVNHAEASRLNIERMKQIYQQ
LSRYRGLGLLATVPSNPNGNGNGGLMGFSPGESVSGCGSTGGFFSSSLLGAQLGTGDAQTAGVQTCALGDSHT
GASGIQQQGYCQASSILRYAHGDNQQSHLMQAGRTSLGTGISAGKRPNPEEETQSVGPKVQRQSTN
>Penelope_pile__NXC46129.1
QPISPAFSSVLKVDYDVCSNYGNWLYSAGIGNDPRDNRKFNVIKQGLEYDGNGDYVRLWVPELQGIKGADIHT
PWALSSATLSRAGVTLGETYPQPVVTAPEWSRHISQRPQGGSPHPQGRRGPAHTPMQCKDRGIDFYFSRKKDL
>Penicillium_sub_OKP02382.1_cry-1
MKSGSASNIVVLYWHRTDLRLHDSPALHAALALEPSVFIPVWTWDPHYVYRARVGPNRWRFLLECQSDLSKSY
TKLNPKQKLWLVREAPQTVLPKLWKKWGITHLVFEKDTDPYARERDEAIVRLAEEAGVEVVVRMGRTLFDPDD
LVRKNGGKPTMSMAQTEKAAEKINRGQPDRPVETPKSVPDAWAKDKMDLSDIEHEFANPTPDLNADHRGSKDK
QYSQIMGPNNDFGVPTMEEIGIDPALATTPHRGGESVALKMLADHISNEEYIGTFEKPKTSPAAFEPQATTLL
SPYLHFGSLSVRKYWWDVQDVMTKRRKANQPNAFVPVNLPGQLLFRDMYFAAQAAVGAPYARVHGNKIVRFID
WHLQSKPHRPAEKDGEALPERDYEVDSPEAEEWFQRWKTGRTGFPWIDALMRQLRSEGWIHHLGRHSVACFLT
RGGCYVSWERGAEVFEELLIDHETSANAGNWMWLSCTAFFAQFYRCYSPIAFGKKWDPNGDFIRRYVPELAKF
DKKFIYEPWKAPIADQKKWGCRVTNDDSAAGGQSYPKPMFDFDKRRQICLDSMKNAYDVGLHGNDEQVLDGSW
RKLFGENGGGEEPKKKRQRTGDEDE
>Penicillium_sub_OKP10124.1_photolyase

MSNSNGDSSPKTAPESKKAKPQDLRQPHPNAQQTEEFGIVLRDFYPPEMSNDRCKAYNDGTIERPIDTLEKAC
KDTADKRRAVTPGKAVVHWFKTDLRLQDNRALSTAFQIAKDNQIPVIGVYIVSPEDFEAHLTSPARVDFTLRT
LKQLQKDLSELDIPLYVETQEKRKNIPTRIVELCQQWEAKHLCANLEYEVDELRRDAKVVRRCADHDIDFTPS
HDTCVVTPGLLITGAGKQYAVYTPWFRSWLRFLKENPDYLEMSEDPGTNPGDARKQFADLFDCKIPDMPSSKS
LSEKEKKRFEELYPAGEHEATRRLEKFLKEKCGDYDKNRSMLPGENTSALSPYFASGAISARTAVVTAKRANK
NHLDRYDQGHMTWISEVAWRDFYRHVLVHWPFIWYGVLYYLLPPPSIHSRKSGLTDPPSMNKCFKPEATNIEW
EYDADQFQAWSEGKTGFPIVDAAMRQLKHCAWMHNRTRMVVSSFLSKDLMIDWRRGERFFMEHLIDGDFASNH
GGWGFGSSTGVDPQPYFRIFNPLRQSERFDPDGEYIRHWVPELRDVKGNAIHEPYSRGAGAIAQKNGYPRPIV
EHAASRDRALARYKSALQG
>Periophthalmus_magn__XP_033823666.1
MLREVLVGREDPEGFFALCVSVLGHHDTRAQFLTVIRPLSTANMSLHSALTAIYEEYFSNNEDDELQLAMALS
LLDAEETTTATKQSTTQEVIQSPPAEDASTVQNKTVPTMDNSFRPSSLTSKQHIYEPKRSKRRRQRSKGSGQQ
VVGLPTAPSPQPPVLLWFRRDLRLCDNPGMIGALEVGAPVIPVFIWAPEEEEGPGTTVAMGGACKFWLHQALS
CLHTSLQRIGSSLVFLKADSGSSLQTLRGLLQETGARTVVANALYEPWLKQRDDLVLSALHKDGIDLRMYHSY
CLRDPYSVSTVGVGLRGIGSVSHFISCCKQNPGPTLGAPLDPPVSLPTPSHWPSGVPLDMLELARMPRRKDGT
IIDWAANIRKSWDFSEEGAHAQLETFLQNGVYRYEKESGRADCCNTSGLSPYLHFGQLSPRWLLWDAKGAKCR
PPKFQRKLSWRDLAYWQLSLFPDLPWESVRPPYKALHWSSNRSHLKAWQRGRTGYPLVDAAMRQLWLSGWMNN
YMRHVVASFLIAYLHLPWQEGYRWFQDTLVDADVAIDAMMWQNGGMCGLDHWNFVMHPVDAAMTCDPCGSYVR
QWCPELADLPDELIHKPWKCPTSMLKRAGVVLGQSYPERIITDLEEHRTQSLKDVAQVRLQFKQYVDRATGCD
LVPLPPRLVSEALGIHGDVEVRGKQFLLPLITRMEFKHQVEEPDSDAGSNPYNAVLKGYVSRKRDETIAFLNE
RDFTASVMNEGVQRRERMDVNYRKMEGLPQVRQPRGRARRTPTAKDKFSIVPGGVVSTLR
>Periophthalmus_magn__XP_033823679.1
MVVNSVHWFRKGLRLHDNPALQEALNGADTVRCVYILDPWFAGAANVGINRWRFLLEALEDLDNSLKKLNSRL
FVIRGQPTDVFPRLFKEWNVTRLTFEYDPEPYGKERDGAIIKMAQEFGVETLVRNSHTLYNLDRIIEMNNNNP
PLTFKRFQTIVSRLELPRRPLPPITPQQMDKCHTKISDNHDQLYNIPSLEELGFRTEGLPTAVWRGGESEALE
RLNKHLDKKVWVASIEHPRVSTCSLYASPTGLSPYLRFGCLSCRVLYYNLRELYMKLRKRCSPPLSLFGQLLW
REFFYTAATNNPNFDRMEGNPICVQIPWDQNPEALAKWAEGRTGFPWIDAIMTQLRQEGWIHHLARHAVACFL
TRGDLWISWESGMKVFEELLLDADWSVNAGSWMWLSCSAFFQQFFHCYCPVGFGRRTDPTGDYIRRYIPILKN
YPNRYICEPWNAPESVQKAANCVVGVDYPKPMINHAEGSRLNIERMKQVYQQLSHYRGLSLLASVPTIQEEAE
PPMTDESQTSSGPDSPHKPPDIEAAGCSTAPDSSTTDLSTFSATVPYTALGDTPCPQNEPPTAISSTSTDPMT
AQPKPSSPASPCPNVAPSPGRTTSLGPRRKGMARKARRSHRQRVRQNCATATKEGDRRTDEESRDSTAAEDKM
EEEIEQDDEKMEEEMHGGESPGYQQ
>Periophthalmus_magn__XP_033823696.1
MVVNTIHWFRKGLRLHDNAALRDSIRGSDSLRCIYILDPWFAGSSNVGINRWRFLLQCLEDLDASLRKLNSRL
FVIRGQPTDVFPRLFKEWQINRLSYEYDSEPFGKERDAAIQKLASEAGVELMVRISHTLYNLDKIIELNQGQP
PLTYKRFQGLINHMEAVELPAETITADVIRTCLTPISDDHDDKYGVPSLEELGFETEGLTTAVWPGGETEALM
RLERHLERKAWVANFERPRMNANSLLASPTGLSPYLRFGCLSCRLFYFKLTDLYKKVKKNSTPPLSLYGQLLW
REFFYTTATNNPCFDKMDGNPVCVQIPWDRNREALAKWAEGRTGFPWIDAIMTQLRQEGWIHHLARHAVACFL
TRGDLWISWEEGMKVFEELLLDADWSVNAGSWMWLSCSSFFQQFFRCYCPVGFGRRTDPNGDFIRRYLPILRT
YPAKYIYDPWNAPEDVQKAAKCIIGLHYPKPMVNHAEASRINIERMKQIYQQLSSYRGLGLLATVPANLSIGT
NGNGQGPGGSVEEPSVLEGAPRTERTQATQKRRHEEPLTEGSAKSWRQSK
>Periophthalmus_magn__XP_033838200.1
MSQKRKATTDAGAEKPSKKPNLTFEKKADGWLQEQVLQQRAAKKEMKFNKKRLRFLTDTQKLKQGAEGVLYWM
NRDQRIQDNWALIHAQRLALKEALPLHVCFCLVVPKSKLSTYRHFSFLLKGLEEVSQELKTLGVEFHLLHGPP
GDVLPGFVSDRSLGVVVTDFSPLREPLQWLEDVKKKLPKDIPFIQVDAHNIVPCWEASPKLEYAARTIRGKIN
KVLSEFLTEFPLVEKHPHTATRTAKQVDWVKELESLQVDKTVGETEWAKPGMKAGMAMLESFIDVRLKLFAAQ
RNDPNAAALSQLSPWIRFGQISAQRVALQVKKKGGESAAPFIEELVVRRELTDNFCYYNKKYDSVEGAYEWAQ
KTLKDHAKDKRPYVYTRDQLEKAKTHDKLWNGAQFQMVSEGKMHGFLRMYWAKKILEWTSSPGEALSIALYLN
DRYSLDGQDPNGFVGCMWSICGIHDQGWAERPIFGKIRYMNYKGCQRKFDVARFEKKYCPKDL
>Periophthalmus_magn__XP_033841635.1
MSTARTIICLLRNDLRLHDNELFHWAQRHAEHVVPLYCFDPRHYEGTYNYNFPKTGPFRLRFLLDTIKDLRNT
LVSKGSNLIVRQGKPEDVVADLIKQLGTVSTVAFHEEVTSEELTVEKNVKSVCAQMKVKVQTCWGSTLYHRDD
LPYNHISRLPDVYTQFRKAVESQSSVRPVFPTPEKLKPIPPGLQEGAIPTAEDLKQTEPIDDPRSAFPCRGGE
SEALARIKHYFWDTNAVATYKETRNGLIGVDYSTKLAPWLALGCISPRYIYHQIKTYETQRTANQSTYWVIFE
LLWRDYFRFVAVKYGNQMFHIYGLQNKSVPWKKDMKLFNAWKEGRTGVPFVDANMRELAMTGFMSNRGRQNVA
SFLTKDLGLDWRMGAEWFEYLLIDYDVCSNYGNWLYSAGVGNDPRENRKFNMIKQGLDYDNNGEFVRQWVPEL
QAIRGSDVHTPWTLSTAALSHAQVSLGETYPVPIVIAPEWSRHMNKKQPGGASGSSQRGKKGPSHTPKQHKDR
GIDFYFSKGKGFP
>Periophthalmus_magn__XP_033845754.1

MSHTCIHWFRKGLRLHDNPALIAGLKDCKELYPVFVLDPYLYNKAFGLNRWRFLIGSLRDLDCSLKKLNSRLF
IVRGEPKDVFPTLFKKWKVSKLTYEYDTEPYSLSRDQTVTELAKEHGVEVIYKVSHTLFDINRIIEANNGKPL
LTYKRMQAIVSDLGSPKKPIPAPTVNDIKYTSTPSSKDHEEKFGVPTLDELGHDIASISEELFPGGEVEALKR
LDEHMKRTQWVCNFEKPQTSPNSLMPSTTVLSPYVTFGCLSARTFWWRLAEVYQGKKHSSPPVSLHGQLLWRE
FFYTASLGIPNFNKMKDNPVCTQVDWDNNPEFLAAWREARTGFPFIDAIMTQLRTEGWIHHLARHAVACFLTR
GDLWISWEEGQKVFEELLLDGDWALNAGNWQWLSASTFFHQFFRVYSPVAFGKKTDKNGDYIKKYLPLLKKFP
PQHIYEPWKAPRSVQQAAGCIIGKDYPKPLVEHEVVRKKNIQRMTLAYAKRSADKSESPSKKQGVKRKAPSVV
DMLNKKAKQK
>Periplaneta_ame_AGE89265.1_cry_2
MSEEKSAKHTVHWFRKGLRLHDNPSLREGLKCASTFRCVFILDPWFAGSSNVGINKWRFLLQCLEDLDQSLRK
LNSRLFVIRGQPADALPKLFKEWGTTNLTFEEDPEPFGRVRDQNIMAMCKELGITVISRVSHTLYKLESIIEK
NGGKAPLTYHQFQTVVASMGSPPPAEPTITSKYINGAYTPIGEDHDDKYGVPTLEELGFDXEGLLPPVWQGGE
TEALARLERHLERKAWVASFGRPKMTPQSLLASQTGLSPYLRFGCLSTRLFYYQLTDLYKKIKKACPPLSLHG
QLLWREFFYCAATKNPNFDKMNGNPICVQIPWDKNAEALAKWANGQTGFPWIDAIMTQLREEGWIHHLARHAV
ACFLTRGDLWVSWEEGMKVFEELLLDADWSVNAGMWMWLSCSSFFQQFFHCYCPVRFGRKADPNGDYIRKYLP
VLKNFPTRYIHEPWNAPESVQKAAKCIIGKEYSLPMVNHAVASRINIERMKQVYQQLSKYRGPGLLATVPTSQ
SQSSANLNPISTQKEYKKSSPENKWETSSDNEINCHSPSYRENIKSGIHFHRFQQHSQKQNREDCE
>Perkinsela_sp._KNH05154.1_DNA_photol
MNSFHFSIPLISAEMKNVLLWFGLHDLRLHDHYALKLACRKAQRTGGCVYPFFCFDLRKFAAASDLCEFFMAH
PRRAIFVKECIEDLRSSLNDLNQQLWVRTGYVENILPDLAEKLNCSDIFAHELHSPEDIAIRLKVENKIRERD
RAVNLRLAWGSTLIHIDELDAPVSDFPIEFSRYQDFFHRKKVRRTDGFNCRTPGGFPPQNPTGGDIPPAGTIP
SLEDLGYRQQADVTYDARGVLDFTGGETAGCQRVRTFFDRKIIENYSPGFAERERARLYKQRSSHLSPWISHG
CVSPRRVYELVLKYSHEQRGTKDWECKSFIMMLVRRDYWYFLSKRFGARLRAPEGPTPSACAIEPPRAGWRLD
PPLIARWCAGVTGVPFVDAAMRELTLTGFSSAQCRRALLWFLVRGLQQDWRVAAEWFQRTLIDYDPHTCWGNC
AYYSGLMFDIGTRMPLHTAEHHHLQFDGSGVFTRLWLPELHNVPTYFLHRPHAMTKHMQEIHNARLGVDYPVP
MKLWFDVEKDVSFVSKLPNYITAENSKLIGEGTAFGLGVVTEREKTLHLSR
>Perkinsus_mar_XP_002773871.1_photo-lyas
MSREIRSMDNWALLYAQNLALEGKQPLLVVYNLAIGYLGGGLRQHVFKVGGLRVLAKNLRDKGISFRVDYSKD
FPKALSKMVTMLGCSHVVTDFTPLKVNKAWEDAFIKEAERLGIRFDQVDAHNIVPCWVASDKLEYAARTIRPR
LWRNWDTYSTEFPTLHKHPYHYDGAHFEITSEACFDMLLSDNEQQLLAGLDHGVKPVTKVLPGEDAGLEHLYT
FIKNMKGYSQFRNDPTKNAISDLSPYFHYGMVSPQRAVMEVAKAKSIPKPDRDAFIEEAFIRRELADNFCYYN
ENYDRFEGFHNWAQKTLQDHEADKRKYVYSLSQLEKASTHDELWNACQNQMLKSGKMHGYLRMYWGKKILEWT
ENPRQALKFAIYLNDKYELDGRDPNGYVGCAWSIGGIHDQGWAERPIFGKIRYMTYDGCKRKFDIQKYINKWG
GIKEGKDDDNYGIKAYLKKRSTTDGESSNAASKRSKIITSLQ
>Perkinsus_sp._TEB20199.1_C9890_0096
MFHGDTVQVLTSLHGICKFQHIFCNRDFTVFAQERDRKIHEWCRKNSVTYVDCEDYDIVPSDRVLMNEGAPEA
RPYTVLSHYFNKFTKDVYTNKALARRPVSESISTSTFFRESLQMENYSLYNKDDLSKLYTKKTTLIQQGGRAH
GLSSLSRVDSVWQKYSNDRHYPGKQEATTRMSAHLKFGTVSIREFFYKVVDASGGSLQSDLLREVVFRSFYYK
IWTHRPTLQRDVSFHQHIDDQIPWRYPKDSAKDWEAWTLGMTGFPMADAGMRQLEVEGYVHGRPRMVLATVAT
RYLLFDWRECARFFATKLTDYDPILNAAGWGFGSSLGENAQNVWRAPMNPLLQSRNYDPECEYIKRWIPELKS
VSVKDIHDWMSITLRTHLNQ
>Petromyzon_mari_XP_032801146.1
MLVGAFCSRYRTQRERALLQPSSSAFRIDLIAKSSRYQNCPGRKRVGPRLAAAIVVCVCVCVCVWRSCTRLLS
ILLCTSSNGSLPTMATTSKQPIPTKKTEPELSNIGALPETSGGPPSKKPKVGATSKESDAGTTAAPTGSGNFE
EQISKTRAALASSVLEFRFNKKRVRAVSNADDMAANASGILYWMSRDQRVQDNWALLYAQRLALKQRLPLYVC
FCLVPKFMDATIRHFGFMLRGLQEVEKEFRSLDISFHLLKGYAKDVLPPFVNEKNIGAVVTDFLPLRVPRQWV
KDLKKALPKNVPLAQVDAHNVVPCWVTSDKLEYGARTIRRKIHDKLPQFFTEFPPAIKHTYPAKSLPEETDWE
AAWQSLEVDRSVGEVAWAQPGTSAGLEMLNSFINERLKLFSTDRNNPNSNALSNLSPWIHFGQISVQRCALEV
SKLKPKYRESVDMYIEEAIVRRELGDNFCFYNKNYDSLDGAYDWAKRSLDAHTQDNREYIYSLQQLEEAKTHD
PLWNAAQLQMVVEGKMHGFMRMYWAKKILEWTSSPEEALRIAIYLNDRYQLDGSDPNGYVGCMWSICGTHDQG
WAERAVFGKIRYMNYAGCKRKFDVGAYESRYSSKKLGFPAPAE
>Petromyzon_mar_XP_032805096.1_cry-1-like
MVLNSIHWFRKGLRLHDNPALREALQGADTVRCVYILDPWFAGASNVGINRWRFLLQSLEDLDASLHKLNSRL
FVIRGQPADVFPRLFKEWNISRLTFEYDSEPFGKERDAAIKKLASEAGVEVLVRISHTLYNLDRIIELNEGQA
PLTYKRFQALVSRMEPPERPVGTITSEVMGPCRTPLWEDHDERYGVPSLEELGFDTDGLTTAVWQGGESEALT
RLDRHLERKAWVANFERPRMNANSLLASPTGLSPYLRFGCLSCRLFHYKLTELYKKVKKNSSPPLSLYGQLLW
REFFYTAATNNPKFDRMEGNPICVQIPWDRNPEALAKWAEGRTGYPWIDAIMTQLRQEGWIHHLARHAVACFL
TRGDLWVSWEEGMKVFEELLLDADWSVNAGSWMWLSCSSFFQQFFHCYCPVGFGRRTDPNGDYIRRYIPLLRA
FPAKYIYDPWNAPESVQKTARCIIGVDYPKPMANHAEASRLNIERMKQIYQHLSRYRGLCLLASVPSNGNGGM

SGYGQGDGHSRPSSAAGFGTSDGSAGGNGGNDGATCSYQKDQQPCASGSLRQQPREMAAPAGVGSPGALDPLG
VLKRDRLCRMEPGEDPPRKKIPKHRSSESMAGN
>Phaeodactylum_tri_XP_002178889.1_cry-dash_f
MSSSRSKQSFWFLIFVHTVLILTFAAGAATAMRGASVSTVTKATSRASSQVVLHWFRHGDLRLLDNPALIHSS
KTAESCVPVFCFDDSVYGNDNRTPDTRAPHSNDRGQLKCGPRRAQFVLDSVQDLRRSLQSRGSALYVAHGKPA
QVFQRLVDAWPAVPAADTAAPNGSLLTIVCQREVVREENDAVRAVQSVLRRRFPQAKVQQIWGSTMYELDDLP
FATDLANMPDTFTPFRNKVEKNCQIGTPLPVPKQLSLPENFPSALKQGLEYLPTLKELGYTDAQIQQVETHDE
RGVLHFMVAKQPDSHDCLKDYFETRNGMLGPNYSTKFSPWLAHGNVSPRYVAAQCRKYEEERVENKSTYWVVF
ELLWRDFCKFFATKHGDAIFYPYGTTERTDRHKPWSTFGRNLQAWQEGRTGYPLVDANMRELVATGFMSNRGR
QNVASFLAINLNHDWRCGGDFFESHLLDYDVYSNWVNWCAAAGMTGGRLNRFNISKQSKDYDQHGDYVRHWLP
ELAKVPNEFVHEPWKMTSFQQMEYECKLGVDYPNPIVPPSRPNPHTDRNNRGRGGHQSKGNNRHGPPDKSRKA
NASGSNRHQKYEMKSLQPGSFRVKES
>Phaeodactylum_tri_XP_002179379.1_protein
MSNSDHGGKQRILAPAVQAHSLSRPAVVLFGTHDIRIHDNEALLLACHHNHVLPVFLWQVPVHHWGARGALQV
VLKEALHQLSLQLSQESINLPLVCGNTADSVSELCKIASEIGASAVYWNREMTPESREMERHRATSLKQLDIA
AVACQSALLYDVEKLELDEGFHGGHWGTLMPFKRACEKQLGKPDRPILMKESLACLYSVAPPPQDCKSVPIEE
LGFETVPPSTKWDEPIRERFPMIHYLAQRRLDHFLIKGLPLYESDRSRADMEYATSQLSVYLRIGIISPRELY
WRIEDSSLSPEAKKTFARRLIWRELAYYQLFCFPKMRDRSIRKHYEASEWVTGDEEKGRFNAWKRGLTGYPLV
DAGMRELYTTGYLTQSVRMVVASFLVEYLRVDWTKGAEWFHYTLADADSAINSMMWQNAGRSGIDQWNFVLSP
ENASQDPYGEYTRKWVPELSPLPLQYLQRPWQTFEGDLRMAGIVLGETYPHRIVQDLKGERQKSVESVLAMRR
RSQEKNDENGYDLIDLPSGIETVVFTKKEYRIDRLGKVLQGKPKTATSTVKRRKTKRTTKTDGRRKNRLPSSL
A
>Phaeodactylum_tri_XP_002180071.1_class_II_C
MLANRTRVLTSEGTEPKEGQSVVYWMQRDVRSVDNWALLWARDLAMQHDVPLHVVYALPPPASSDGSDNDRDL
PPALIQLPMTKRHGAFLLGGLECVYKELKEMKIPLYVCLPDSHEKVGETVCEAILHKYKAKIVVSDFSPIREY
RQWMELQAVPILEEAKVPFYQVDAHNIVPVWTATDKRQVGARTLRPRIHKVYNDYLQDYPDLKGNSHSVDQPK
FDRVEYESFLQMDESVESVDWAQPGTEAGMKQFEFFSKNGLKIFHEQRNDPVQKHVCSDMSPWINHGHISFQR
LALNVKALNKHANGAAAFIEEGVIRRELSDNMLYYSPNDYDSLETAAGWARESLQLHASDEREFVYSLSELEE
GRTHDDLWNAAQLQMVRDGKMHGFMRMYWAKKILEWSESPVGALRTAQYLNDKYELDGRDPNGFVGVGWSIMG
IHDQGWKEREVFGKIRYMNYNGCKRKFKVEEYVAQYKGAAENAANAVEETNGSSNKRKSLPSSSNSKQKTARK
>Phaeodactylum_tri_XP_002180095.1_cry_photol
MAKSEEKKHDVAIHWFRNGLRFHDNPCLLDACQKSESLLPIYVVDPEFPFAQTAGCRAGTIRANFLLESINEV
DEKLRKMGSQLVVVLGKSHEVLPEIVATTQAKALFYEQEAAAPVREQDAETIQAIKNRLKRDGKNYECKFEAY
ATHTLHPMERYLAQCKDHTAPSTYGSFTKIFNKMSVAKEVNEVKEVPSLPNKSVKLLEKSFAEALRMPTLKDL
GYAAAADDMKNSGKGGYAFAGGENAAIELLAKNMARSQWVATFEKPKTSPNDATRPSTTALSPYVKHGCISPR
RFYHELSKVYSKYNSKETSKPPVSLHGQLMWRDFNYLVGYSTPNFDKMIDNPIARQIPWDDDPDLLLAWKMSK
TGYPYIDAIMTQLRETGWIHHLARHSVACFLTRGDLWQSWEDGATVFEEYLIDADWSINNFNWQWLSCTAHFY
QYFRCYSPIAFGKKTDPNGDYIRKWLPQFKDMPAKYIYEPWEAPIELQKKVGVIVGENYPHPIVDHKLVSKNN
MSRMKEAYDAQKNREPMPANESHSSGLNRDTSPKRQRRN
>Phaethon_lept__KFQ64035.1
FLLQSLEDLDNSLRKLNSRLFVVRGQPTDVFPRLFKEWGVTRLTFEYDSEPFGKERDAAIVKLAKEAGVEVVI
ENSHTLYDLDRIIEMNGHKPPLTYKRFQAIISRMELPKKPVSTIISQQMEMCKVDIQENHDDVYGVPSLEELG
FPTDGLAPAVWQGGETEALARLDKHLERKAWVANYERPRMNANSLLASPTGLSPYLRFGCLSCRLFYYRLWEL
YKKVKRNSTPPLSLYGQLLWREFFYTAATNNPKFDRMEGNPICIQIPWDRNPEALAKWAEGKTGFPWIDAIMT
QLRQEGWIHHLARHAVACFLTRGDLWISWESGVRVFDELLLDADFSVNAGSWMWLSCSAFFQQFFHCYCPVGF
GRRTDPSGDYVKRYLPKLKGFPSRYIYEPWNAPESVQKAAKCIIGVDYPKPMVNHAETSRLNIERMKQIYQQL
SRYRGLCLLASVPSCVEDLSGPVTDSASGQGCSTSTAMRLSQADQASPKRKHEGAEELCAEELYKRAKVTGLP
APEIPGK
>Phaethon_lept__KFQ69809.1
DNWAFLYAQRLALKQELPLHVCFCLVPKFLDATIRHYSFMLRGLQEVAKECAELNIPFHLLLGYAKDVLPTFV
VEHGVGGLVTDFCPLRLPRQWVEDIRERLPADVPFAQVDAHNIVPCWVTSPKQEYSARTIRGKIHAQLPEFLT
EFPPVIRHPYPPSCPAEPIAWEACYSSLQVDHTVKEVEWATPGTAAGLSVLQSFITERLKSFGAHRNDPNKAA
LSNLSPWFHFGQVSTQRAILEVQKHRGKYKESVDVFVEEAVVRRELAENFCYYNENYDSVQGAYDWAQTTLKL
HAKDKRPFLYELQELEQATTHDPLWNAAQLQMVQEGKMHGFLRMYWAKKILEWTRSPEEALRFAIYLNDRYEL
DGRDPNGYVGKLQDGGCLWSICGIHDQGWAERAIFGKIRYMNYAGCKRKFDVGQFERRYAP
>Phaethon_lept__KFQ78546.1
WFASENKRFVTISCRFLLQCLEDLDANLRKLNSRLFVIRGQPADVFPRLFKEWNIAKLSIEYDSEPFGKERDA
AIKKLASEAGVEVIVRISHTLYDLDKIIELNGGQPPLTYKRFQTLISRMEPLEMPVETITPEVMEKCTTPVSD
DHDEKYGVPSLEELGFDTDGLPSAVWPGGETEALTRLERHLERKAWVANFERPRMNANSLLASPTGLSPYLRF
GCLSCRLFYFKLTDLYKKVKKNSSPPLSLYGQLLWREFFYTAATNNPRFDKMEGNPICVQIPWDKNPEALAKW

AEGRTGFPWIDAIMTQLRQEGWIHHLARHAVACFLTRGDLWISWEEGMKVFEELLLDADWSVNAGSWMWLSCS
SFFQQFFHCYCPVGFGRRTDPNGDYIRRYLPVLRGFPAKYIYDPWNAPESVQKAAKCIIGVNYPKPMVNHAEA
SRLNIERMKQIYQQLSRYRGLGLLATVPSNPNGNGNGGLMGYSPGESISGCGSTGGAQLGTGDGHTIIQPCAL
GDSHTGASGIQQQGYCQASSILHYAHGDNQQSHLLQAGRMALGTGISTGKRPNPEEETQSVGPKVQRQSTN
>Phaethon_lept__XP_010281210.1
MQHSSIHWFRKGLRLHDNPALLAAATNCRRLHPPFILHPSSNRAGINAQRFLLDALRDLDGSLQEMGSRLFVA
QGCPEEVFPHLFHAWGTTRLTFEVDTEPSACRRDHGVEVIREVSHTLYDTERVLALNDGKAPLTYKRLQSLLA
LLGPPEKPAPALTQEHLQGCLTPCQASHDGDYRVPTLEELGQDPAKVGPHLYPGGETAALARLDTLMERTAWV
CNFKKPETEPTSLSPSTTVLSPYLKFGCLSVRTFWWRLDEIYQGREHSQPPVSLHGQLLWREFFYTAGASIPN
FDRMAGNPVCLQVEWDDNPQHLHAWREGRTGYPFIDAIMTQLRTEGWIHHLARHAVACFLTRGDLWVSWEEGL
KVFEELLLDADWSLNTGNWLWLSGSAFFHRYFHVYSPITFGKKTDQDGAYIRKYLPILKDFPTKYIYEPWKAP
QAVQERAGCLVGTHYPQPIVEHGVASKRNLGRMKAARAQKDTKREPPAGPSTVKSQRKKPKIEQH
>Phaethon_lept__XP_010288559.1
MPHRTIHLFRKGLRLHDNPTLLAALESSEVVYPVYILDRKFMTSVMHIGALRWHFLLQALEDLQKNLCQLGSC
LLVIQGEYESVLRDHARKWNITQITLDAEMEPFYKEMEANIRCLGEALGFKVLSLVGHSLYDTRRILDLNGGT
PPLTYKRFLHILSLLGDPEMPVRNLTAEDFQRCSPPDPGLAECYRVPLAVDLKIPLESLSPWRGGETEGLQRL
EQHLTDQGWVASFTKPRTIPNSLLPSTTGLSPYFSMGCLSVRTFFYRLSNIYAQAKYHSLPPVSLQGQLLWRE
FFYTVASATPNFTKMAGNPICLQISWYEDAERLHKWKTAQTGFPWIDAIMTQLRQEGWIHHLARHAVACFLTR
GDLWISWEEGMKVFEELLLDADYSINAGNWMWLSASAFFHQYTRIFCPVLFGKRTDPEGQYIRKYLPILKNFP
SKYIYEPWRASEEEQKQAGCIIGQDYPFPMVNHKEASDHNLQLMKQVREEQYKTAQLTRGWELACARWRNAYP
KAVRLYF
>Phaethon_lept__XP_010293381.1
MSGAARTAICLLRCDLRAHDNQVLHWAQSNADFVIPLYCFDPRHYLGTHCFGFPKTGPHRLRFLLESVKDLRE
TLQKKGSTLVVRKGKPEDVVCDLITQLGSVSAVAFHEEATQEELDVEKGLCQVCSQHGVKIQTFWASTLYHRD
DLPFRPIARLPDVYTHFRKVVESEAKVRPTLHMADQLKPLAPGVEEGCILTMEDLGQKDPVTDPRTAFPCSGG
ETQALMRLQYYFWDTNLVASYKETRNGLVGMDYSTKFAPWLALGCISPRYIYEQIQKYEKERTANQSTYWVLF
ELLWRDYFRFVALKYGKRIFSLKGLQSKEVPWKKDLQLFDCWKDGKTGVPFVDANMRELAATGFMSNRGRQNV
ASFLTKDLGLDWRMGAEWFEYLLVDYDICSNYGNWLYSAGIGNDPRDNRKFNMIKQGLDYDGNGDYVRLWVPE
LQGIKGPDIHTPWALNSAALSQAGVTLGETYPHPVVTAPEWSRHINQRPPGRSPHPRGRRGPAHTLMQHKDRG
IDFYFSRKKDV
>Phalacrocorax_carb__KFW93274.1
RFLLQSLEDLDNSLRKLNSRLFVVRGQPTDVFPRLFKEWGVTRLTFEYDSEPFGKERDAAIIKLAKEAGVDVV
IENSHTLYDLDRIIELNGHKPPLTYKRFQAIISRMELPKKPVSTVMSQQMETCRVDIQENHDDVYGVPSLEEL
GFPTDGLVPAVWQGGETEALARLDKHLERKAWVANYERPRMNANSLLASPTGLSPYLRFGCLSCRLFYYRLWE
LYKKVKRNSTPPPSLYGQLLWREFFYTAATNNPKFDRMEGNPICIQIPWDRNPEALAKWAEGKTGFPWIDAIM
TQLRQEGWIHHLARHAVACFLTRGDLWISWESGVRVFDELLLDADFSVNAGSWMWLSCSAFFQQFFHCYCPVG
FGRRTDPSGDYVKRYLPKLKGFPSRYIYEPWNAPESVQKAAKCIIGVDYPKPMVNHAETSRLNIERMKQIYQQ
LSRYRGLCLLASVPSCVEDLSSPVTDSASGQGCSTSTAIRLSQADQASPKRKHEGAEELCTEELYKRAKVTCL
PAPEIPGKS
>Phalacrocorax_carb__XP_009499104.1
FLLQCLEDLDANLRKLNSRLFVIRGQPADVFPRLFKEWNIAKLSIEYDSEPFGKERDAAIKKLASEAGVEVIV
RISHTLYDLDKIIELDGGQPPLTYKRFQTLISRMEPLEMPVETITPEVMEKCTTPVSDDHDEKYGVPSLEELG
FDTDGLPSAVWPGGETEALTRLERHLERKAWVANFERPRMNANSLLASPTGLSPYLRFGCLSCRLFYFKLTDL
YKKVKKNSSPPLSLYGQLLWREFFYTAATNNPRFDKMEGNPICVQIPWDKNPEALAKWAEGRTGFPWIDAIMT
QLRQEGWIHHLARHAVACFLTRGDLWISWEEGMKVFEELLLDADWSVNAGSWMWLSCSSFFQQFFHCYCPVGF
GRRTDPNGDYIRRYLPVLRGFPAKYIYDPWNAPESVQKAAKCIIGVNYPKPMVNHAEASRLNIERMKQIYQQL
SRYRGLGLLATVPSNPNGNGNGGLMGYSPGESISGCGSTGGAQLGTGDGHTVVQPCALGDSHTGASGIQQQGY
CQASSILHYAHGDNQQPQLLQSGRTALDTGISAGKRPNPEEETQSVGPKVQRQSTN
>Phalacrocorax_carb__XP_009509490.1
MSGAARTTICLLRCDLRAHDNQVLHWAQSNADFVIPLYCFDPRHYLGTHCYSFPKTGPHRLRFLLESVKDLRE
TLKKKGSTLVVRKGKPEDVVCDLITQLGSVSAVAFHEEATQEELDVEKGLCQVCCQHGVKVQTFWASTLYHRD
DLPFRPIARLPDVYTHFRKAVESEVKVRPTLRMADQLKPLAPGVEEGCIPTMEELGQKDPVTDPRTAFPCSGG
ETQALMRLQYYFWDTNLVASYKETRNGLLGMDYSTKFAPWLALGCISPRYIYEQIQKYEKERTANQSTYWILF
ELLWRDYFRFVALKYGKRIFSLRGLQSKEVPWKKDLQLFDCWKEGKTGVPFVDANMRELAATGFMSNRGRQNV
ASFLTKDLGLDWRMGAEWFEYLLVDYDVCSNYGNWLYSAGIGNDPRDSRKFNMIKQGLDYDGNGDYVGLWVPE
LQGIKGADIHTPWALSSAALSQAGVTLGETYPQPVVTAPEWSRHITQRP
>Phalacrocorax_carb__XP_009513296.1
LYWMSRDQRVQDNWAFLYAQRLALKQELPLHVCFCLVPKYLDATIRHYGFMLRGLQEVAEECAELNVPFHLLL
GYAKDVLPTFVVEHGVGGLVTDFCPLRLRRQWVEDVSERLPEDVPFAQVDAHNIVPCWVASPKQEYSARTIRG
KIHTQLPEFLTEFPPVVRHPYPPSCSVEPIAWEACYSSLQVDRTVKEVEWATPGTAAGLAVLQSFITERLKSF

GSLRNDPNKAALSNLSPWFHFGQVSTQRAILEVQKHRGKYKESVDAFVEEAVVRRELAENFCYYNENYDSIQG
AYDWAQTTLKLHAKDKRPFLYKLQELEQGTTHDPLWNAAQLQMVREGKMHGFLRMYWAKKILEWTRTPEEALQ
FAIYLNDRYELDGMDPNGYVGCLWSICGIHDQGWAERAIFGKIRYMNYAGCKRKFDVGQFERRYTPHTLSQ
>Phalacrocorax_carb__XP_009513466.1
MPHRTIHLFRKGLRLHDNPTLLAALESSEVVYPVYILDRKFMTSAMQIGALRWHFLLQSLDDLQKNLCQLGSC
LLVIQGEYESVLRDHIQKWNITQVTLDAEMEPFYKEMEANIRHLGEELGFEVLSLLGHSLYDTKRILDLNSGT
PPLTYKRFLHILSLLGDPEVPVRNLSAEDFQRCRPRDPGLAECYRVPLPVDLKIPPESPSPWRGGETEGLQRL
EQHLTDQGWVASFTKPRTIPNSLLPSTTGLSPYFSMGCLSVRTFFYRLSNIYAQAKHHSLPPVSLQGQLLWRE
FFYTVASATPNFTKMAGNPICLQISWYEDAERLHKWKTAQTGFPWIDAIMTQLRQEGWIHHLARHAVACFLTR
GDLWISWEEGMKVFEELLLDADYSINAGNWMWLSASAFFHQYTRIFCPVRFGKRTDPEGQYIRKYLPILKNFP
SKYIYEPWTASEEEQKQAGCII
>Phascolarctos_cinereus_XP_020865444.1_cryptochro
MAATTVVKVPAAAAAPAPAPAPAPAPAPAPALTPTPTQTQTQTPAPAPEEGASSVHWFRKGLRLHDNPALQAA
LRGARCVRCVYILDPWFAASSSVGINRWRFLLQSLEDLDISLRKLNSRLFVVRGQPTDVFPRLFKEWGVTRLT
FEYDSEPFGKERDAAIVKMAKEAGVEVVTENSHTLYDLDRIIELNGQKPPLTYKRFQAIISRMELPRKPVSCV
TSQQMERCQAEIQENHDDTYGVPSLEELGFPTDGLGPAVWQGGEMEALARLDKHLERKAWVANYERPRMNATS
LLASPTGLSPYLRFGCLSCRLFYYRLWELYKKVKRNNTPPLSLYGQLLWREFFYTAATNNPKFDRMEGNPICI
QIPWDRNPEALAKWAEGKTGFPWIDAIMTQLRQEGWIHHLARHAVACFLTRGDLWVSWESGVRVFDELLLDAD
FSVNAGSWMWLSCSAFFQQFFHCYCPVGFGRRTDPSGDYVRRYLPQLKGFPSRYIYEPWNAPESVQKAAKCII
GVDYPKPIVNHAETSRLNIERMKQIYQQLSRYRGLCLLASVPSCMEDLSNPMAETSMGQGSGVSGTGPKPLPC
SPASPKRKLEAVEETSGEEHSKRARVMAALPVPDLAGKV
>Phascolarctos_cin_XP_020838097.1_cry-1_X1
MGVNAVHWFRKGLRLHDNPALKECIEGADTVRCVYILDPWFAGSSNVGINRWRFLLQCLEDLDANLRKLNSRL
FVIRGQPADVFPRLFKEWNIAKLSIEYDSEPFGKERDAAIKKLASEAGVEVIVRISHTLYDLDKIIELNGGQP
PLTYKRFQTLISKMEPLATPVETITPEAMHKCVTPLSEEHDEKYGVPSLEELGFDTDGLPSAVWPGGETEALT
RLERHLERKAWVANFERPRMNANSLLASPTGLSPYLRFGCLSCRLFYFKLTDLYKKVKKNSSPPLSLYGQLLW
REFFYTAATNNPRFDKMEGNPICVQIPWDRNPEALAKWAEGRTGFPWIDAIMTQLRQEGWIHHLARHAVACFL
TRGDLWISWEEGMKVFEELLLDADWSINAGSWMWLSCSSFFQQFFHCYCPVGFGRRTDPNGDYIRRYLPVLRG
FPAKYIYDPWNAPEGVQKVAKCLIGVNYPKPMVNHAEASRLNIERMKQIYQQLSRYRGLGLLASVPSNPNGNG
GLLGYAPGESIPACSSSGGGAPAGASDGQILQACALPEPPTGASGSQQAGYSQGSGISHYSREDSQQAYMLKP
GRSSLGGGKRHRPEEETQGVGPKMQRQSVN
>Phascolarctos_cin_XP_020841690.1_photolyase
MAPKKRSHSTSSEPEKTESQESKAKRKPLQKHQFSKSNMVQKEEEDKREGDKRGGTEGLQEVVRQSRLRTAPS
VLEFRFNKQRVRLISQDCHLQDRSQAFVYWMSRDQRVQDNWAFLYAQRLALKQKLPLHVCFCLAPCFLGATIR
HYDFMLRGLEEVAEECEKLCIPFHLLLGLPKDVLPAFVQTHGIGGIVTDFSPLLHHTQWVKDVQDALPRQVPF
VQVDAHNIVPCWVASDKQEYGARTIRHKIHDRLPHFLTEFPPVICHPYLSNIQAEPVDWNGCRAGLQVDRSVK
EVSWAKPGTASGLTMLQSFIAERLPYFGSDRNNPNKDALSNLSPWFHFGQVSVQRAILEVQKHRSRYPDSVTN
FVEEAVVRRELADNFCFYNKNYDKLEGAYDWAQTTLRLHAKDKRPHLYSLEQLESGKTHDPLWNAAQMQMVKE
GKMHGFLRMYWAKKILEWTHSPEEALEFAIYLNDRFQLDGRDPNGYVGCMWSICGIHDQGWAEREIFGKIRYM
NYAGCKRKFNVAEFERKYSSAD
>Phoenicopterus_rube_ruber_KFQ77349.1
IGALRWHFLLQSLEDLQKNLCQLGSFLLVLQGEYESVLRDHIQKWNITQVTLDAEIEPFYKEMEANIRRLGDE
LGFEVLSLVGHSLYDTKRILDLNGGTPPLTYKRFLHILSLLGDPEVPVRSLTAEDFQRCRPPEPRLAERYGVP
LPEDLKIPLESLSPWRGGETAGLQRLEQHLTDQGWVASFTKPRTIPNSLLPSTTGLSPYFSLGCLSVRAFFYR
LSNIYAQAKNHSLPPVSLQGQLLWREFFYTVASATPNFTKMAGNPICLQIGWYEDAERLHKWKTAQTGFPWID
AIMTQLRQEGWIHHLARHAVACFLTRGDLWISWEEGMKVFEELLLDADYSINAGNWMWLSASAFFHQYTRIFC
PVRFGKRTDPEGQYIRKYLPVLKNFPSKYIYEPWTASEEEQKQAGCIIGQDYPFPMVNHKEASDHNLQLMKQV
REEQHR
>Phoenicopterus_rube_ruber_KFQ82110.1
RFLLQSLEDLDNSLRKLNSRLFVVRGQPTDVFPRLFKEWGVTRLTFEYDSEPFGKERDAAIVKLAKEAGVEVV
IENSHTLYDLDRIIELNGHKPPLTYKRFQAIVSRMELPKKPVSTVTSQQMEMCKVDIQENHDDVYGVPSLEEL
GFPTDGLAPAVWQGGETEALARLDKHLERKAWVANYERPRMNANSLLASPTGLSPYLRFGCLSCRLFYYRLWE
LYKKVKRNSTPPLSLYGQLLWREFFYTAATNNPKFDRMEGNPICIQIPWDRNPEALAKWAEGKTGFPWIDAIM
TQLRQEGWIHHLARHAVACFLTRGDLWISWESGVRVFDELLLDADFSVNAGSWMWLSCSAFFQQFFHCYCPVG
FGRRTDPSGDYVKRYLPKLKGFPSRYIYEPWNAPESVQKAAKCIIGVDYPKPMVNHAETSRLNIERMKQIYQQ
LSRYRGLCLLASVPSCVEDLSGPVTDSASGQGCSTSTAMRLSQADQASPKRKHEGAEELCPEELYKRAKVTGL
PAPEIPGKS
>Phoenicopterus_rube_ruber_KFQ82669.1
RFLLQCLEDLDANLRKLNSRLFVIRGQPADVFPRLFKEWNIAKLSIEYDSEPFGKERDAAIKKLASEAGVEVI
VRISHTLYDLDKIIELNGGQPPLTYKRFQTLISRMEPLEMPVETITPEVMEKCTTPVSDDHDEKYGVPSLEEL

GFDTDGLPSAVWPGGETEALTRLERHLERKAWVANFERPRMNANSLLASPTGLSPYLRFGCLSCRLFYFKLTD
LYKKVKKNSSPPLSLYGQLLWREFFYTAATNNPRFDKMEGNPICVQIPWDKNPEALAKWAEGRTGFPWIDAIM
TQLRQEGWIHHLARHAVACFLTRGDLWISWEEGMKVFEELLLDADWSVNAGSWMWLSCSSFFQQFFHCYCPVG
FGRRTDPNGDYIRRYLPVLRGFPAKYIYDPWNAPESVQKAAKCIIGVNYPKPMVNHAEASRLNIERMKQIYQQ
LSRYRGLGLLATVPSNPNGNGNGGLMGYSPGESISGCGSTGGAQLGTGDGHTVVQPGALGDSHTGASGIQQQG
YCQASSILHYAHGDNQQSHLLQAGRTALGTGISAGKRPNPEEETQSVGPKVQRQSTN
>Photinus_pyr_XP_031344947.1_cry-1-like
MSAISGCMGPALSKGPEVHTVHWFRKGLRFHDNPALRESLKGAKTFRCVFVLDPWFAGSSNVGINKWRFLLTC
LEDLDRSLQKMNSRLFVVRGQPADVLPKLFKEWGTTLLTFEEDPEPFGRVRDQNIITICKEMGIAVSCKVSHT
LYHLEDIIDRNGGRAPLTYHQFQTIIASMDPPSFAEPPVSAKDVANIATPIEDDHVEKYGVPSLEELGFDTEG
LLPPVWLGGESEALARLERHLERKAWVASFGRPKMTPQSLLPSQTGLSPYLRFGCLSTRLFYYQLTDLYKKIK
KTVPPLSLHGQLLWREFFYCAATRNTNFDKMLGNPICVQIPWDKNAEALAKWANGQTGFPWIDAIMTQLREEG
WIHHLARHAVACFLTRGDLWLSWEEGMRVFEELLLDADWSVNAGMWMWLSCSSFFQQFFHYYCPVKFGRKADP
NGDYIKKYLPVLKNIPTQYIHEPWMAPDVIQRTSKCIIGKDYPLPIVNHASASRINIQRMKQVYQKLANYRNI
DTWNVPQKQNYIRFENNAVNGNVI
>Physcomitrium_pat_XP_024358644.1_cry_DASH,_
MLSTSVLPGNRHLRSSFLPAVFEFAASQNLRRAAGAWRVQKFAVTLTVSASGEKSKMTSRKGTGIAIVWFRND
LRISDNEALLKAWQSSQAVVPLYVVDPRLYGQTYHFGFPKTGELRAQFIMESVANLRDNLRQRGLDLIVRQGK
PEDVVPSIAKSIGAHTVYAHKETCSEETTVETGVKRGLANLEGGPKMQIRLDFVWGGTLYHLDDLPFSPSELP
DVYTQFRKGVEYNCKVRPALKMPTQMGPLPESLSEKIGELGDIPTMAELGLKYREQSPLGVMHFEGGETAALA
RLQDYFWNKDQLKIYKETRNGMLGANYSTKFSPWLAHGCLSPRTIHDEVKRYETERVANDSTYWVLFELIWRD
YFRFVSIKYGNAIFHLGGPRKVVGKRWSQNRELFEAWREGRTGYPLIDANMKELAATGFMSNRGRQIVCSFLV
RDLGLDWRMGGEWFETCLLDYDPCSNYGNWTYGAGVGNDPREDRYFSIPKQSATYDPEGEYVAHWLPSLKQSP
PKQRNFPGDAYLRPIVPLKFGMGSGGKPNYSRAGYKGKNVGKVGKR
>Physcomitrium_pat_XP_024361046.1_(6-4)DNA_p
MVAESFRCVGHSFFVKSLPSAALRTANCGRRRSFSVSCSNEKGNNNMGKRSLMWFRKGLRLHDNPALLRSCEG
ASHVYPVFVLDPWFLAPDPTAPSPGSKLVGVNRIRFLLQSLEDLDENLRKHGSRLLVLHGNPTTVIPELLMKW
QINELCFEFDTEPYAQDRDADIKKLATKYGVEVFSPVSHTLFNPIDTIRKNGGQTPLTYQAFLKLTGKPPLPV
TAPLQIPSPPEDLNDVHVVPVPKLEDLGYVNLDEEFSPHPGGETEALRRLDKSLVNQKWVCDFEKPKGNPTAF
IEPATTVLSPYLKFGCLSCRLFHQRLLAVYSQNKKHSSPPVSLEGQLLWREFFYTAGYGTPNFDRMLGNPICK
QIPWKDDDRLLAAWRDGQTGYPWIDAAMVQLRKWGWMHHLARHAVACFLTRGDMFVYWEKGRDVFDRLLIDSD
WAINNGNWLWLSASAFFHQYHRIYSPVTFGKKYDPDGLYVKHFLPVLKDMPKYYIYEPWTAPLAVQKKANCII
GVDYPRPMAVLNSCRPLHCQQGMPREDGHCLFTEPFASSTVRKQYDAQEKAWC
>Physcomitrium_pat_XP_024374871.1_photolyase
MPPKKKLKALSVVKEEVHSEPKGRSLKPPKVEEEGEVSELSRKRPKPSSTVTEEDKVTESRVEKLGKDAAAAA
SSNSERRPPLSLGVHPGRIQNLKLGENEHGPVVYWMSRDHRSRDNWALLHAVHQAREKGVPVAVAFNLVESFL
EARARHFGFMLRGLRVVEQNLKAVNIPFFLFRGNPVETIPEFLKKCNASLLVMDYSSLRIGRQWRNGVCENVA
SSVAVAEVDAHNVVPVWRASDKLEYGARTIRNKINNQLAEFLNEYPVLENPGKPWEAGAPDTIDWDALIAEVS
RFGSEVPEVTWCEAGEDAALEALAGKSKGFVNIRIRNYVNRNDPSKPTGLSGLSPYLHYGHISAQRCALEARK
VRKVHTKSVDAFLEELIVRGGLAENFCHYQPNYDNLKGAWGWAQESLRIHAKDKREVVYTESELEAGKTHDKL
WNAAQLEMVYYGKMHGFMRMYWAKKILEWTESPEEALRIAIYLNDKYELDGRDPNGYVGCMWSICGIHDQGWK
ERPVFGKIRYMNFNGCKRKFNVDGYIMNVNQMVAKTKKQLKEGSTTAAPSKPVAGKSDKL
>Physcomitrium_pat_XP_024380321.1_cry-1-like
MAACTIVWFRRDLRLEDNPALIAAARAGTVVPVFVWSPAEDGQFHPGRVSRWWLKQSLTHLELSLKKLGSPLI
LRKSPDTLSVLLEIAEATGATQVFYNHLYDPVSLVRDHRVKQGLSQRGIVVHTFNGDLLYEPWEVYDEEGQAF
TVYEAFWKKCMSMPFEPEAPLLPPRRLTGPIGKIVGCNAEELGLEDEFEKSSNALLARAWCPGWGFANKSLDS
FLRSPLIDYARDRQKADGASGTPTSLLSPHLHFGELSVRKIFHEVRKRQITWAREGNAGGEASVNMFLRALGF
REYSRYLSFHFPFTHERSLLANLKSFPWRADEGYFKAWRQGRTGYPLVDAGMRELWATGWAHNRIRVVVASFS
VKFLQLPWRWGMKYFWDVLLDADLECDVLGWQYISGSLPDGHELDRIENPEVEGYRFDPDGDYVRRWIPELAR
LPNEWVHHPWDAPPSALRAAGVELGTNYPRPIVEIGAARERLQASLAEMWERDAAMKAALANGLEEGLGETVE
VAGTGGPEHERMDVPRVMVHMQRDADMSCNSSRRDQLVPEIVPNQFHIRAHESIMNRSAAMVEDGEEAGRAAV
PMVFASVRRGMGGNYGGHHVEGNGGEVAQASAPIQWPTVTAVDYELDSTAESASVTGRGGSEGGTVPVWSQSV
SARTPIQVREGLVPEVRRGPGLSRRQLQASVQRVNLEGMTSNKQAEEEDFYVPKLVKWTQPRKRRVKQDG
>Physcomitrium_pat_XP_024385617.1_blue-light
MEARGADSLWGVGIRRELFRASIEGHHCPRCSSVRVLSGPSPPAFPHTPPPLLPVFVLSISFLSAMAGYGGLD
DLELILDRNLHRALAASRTNAPAPPWTNSGAAATGAEVEDDEEEAVASKEEVADAASFGNPLPSAASLSLALN
SVLTSSLILSKNNVVPQASQRGGVGPAISGAVPSQKKSHSPVPVMASSKVQVALDIPVATPITVSKKSANYVS
AFPEYEKLRGPDFKDPANGCGLRRASIVWFRNDLRVHDNEALVSANRDSLSILPVYCFDPKDYGKSSSGFDKT
GPYRANFLLECVANLRSSLRERGSDLIVRVGSPEAVLVDLAKSVGAEALYVHQEVTYEELQAEEKVAAALQEK
GIETKYFWGSTLFHLEDLPFKLQDMPSNYGGFREKVQNVAIRDTIEAPQQLKGLPACGNVKPGSIPSIEVLGL

NPATNLRKQGSVTVGGDGLLGGEEEALQRLQKFALEARSQTSNLKASKSQSESSGDSLYGANFSCKISPWLTM
GCLSPRRMFEDLKKSASSASAGAMSALAVKASGAGSDDNGLNWLVFELLWRDFFRFITKKYGTGKRLSEATPA
TASTASLVAA
>Physeter_catodon_XP_007125038.1_cryptochro
MAAAVATAALAAPAPAARAEGASSVHWFRKGLRLHDNPALQAAVRGAHCMRCVYILDPWFAASSSVGINRWRF
LLQSLEDLDTSLRKLNSRLFVVRGQPADVFPRLFKEWGVTRLTFEYDSEPFGKERDAAIMKMAKEAGVEVVTE
NSHTLYDLDKIIELNGQKPPLTYKRFQAIISRMELPKKPVGSVTSQQMEGCRAEIQENHDETYGVPSLEELGF
PTEGLGPAVWQGGETEALARLDKHLERKAWVANYERPRMNANSLLASPTGLSPYLRFGCLSCRLFYYRLWDLY
KKVKRSSTPPLSLFGQLLWREFFYTAATNNPRFDRMEGNPICIQIPWDRNPEALAKWAEGKTGFPWIDAIMTQ
LRQEGWIHHLARHAVACFLTRGDLWVSWESGVRVFDELLLDADFSVNAGSWMWLSCSAFFQQFFHCYCPVGFG
RRTDPSGDYIRRYLPKLKGFPSRYIYEPWNAPESIQKAAKCIIGVDYPRPLVNHAETSRLNIERMKQIYQQLS
RYRGLCLLASVPSCMEDLSNPVAEPSSSQVGSMNSAGPRPLPSGPASPKRKLGAAEEPPGEELSKRARVAGLP
PSELPSKDV
>Physeter_cat_XP_023973802.2_photolyase
ELLHAKGPAPVEKWTPGTQAGLQQLRAFCCPQQLVNYEKTRNDPLAGAQSNLSPYLHFGQLSQQRCLLEALLV
KRGTGGLESNRSSSSSGDASCRSRTSSHNGSSSGVDTGGSNRHPNASTRDDSRIKVEDRRSSSASSKPARPNI
SLGSRSRSGGSRNSTSSAPSGGFNRVNVITGVDAFVEELVVRCALSENFVTYCGDKYDKIEGAPRWAQESLRQ
HEKDPRVPLYDLRVLEAAETYDALWNASQQQVMKEGKMHGYLRMYWAKKILEWMKSPADALAGAILLNDKYSL
DGTDPNGYVGCMWSIAALHDQPFKERPIYGKVRYMNYAGCQRKFNVNAFIQLYTTSSNNSRTSTSAGITTKRR
QGSSSKEESKSIRRDGRTSSTREAEAPKYGAKRTAEKGAERLGKALLTSTPKELRQQY
>Physeter_cat_XP_023983171.1_cry-1_X1
MGVNAVHWFRKGLRLHDNPALKECIQGADTIRCVYILDPWFAGSSNVGINRWRFLLQCLEDLDANLRKLNSRL
FVIRGQPADVFPRLFKEWNITKLSIEYDSEPFGKERDAAIKKLATEAGVEVIVRISHTLYDLDKIIELNGGQP
PLTYKRFQTLISKMEPLEIPVETITSEVIEKCTTPLSDDHDEKYGVPSLEELGFDTDGLSSAVWPGGETEALT
RLERHLERKAWVANFERPRMNANSLLASPTGLSPYLRFGCLSCRLFYFKLTDLYKKVKKNSSPPLSLYGQLLW
REFFYTAATNNPRFDKMEGNPICVQIPWDKNPEALAKWAEGRTGFPWIDAIMTQLRQEGWIHHLARHAVACFL
TRGDLWISWEEGMKVFEELLLDADWSINAGSWMWLSCSSFFQQFFHCYCPVGFGRRTDPNGDYIRRYLPVLRG
FPAKYIYDPWNAPEGIQKVAKCLIGVNYPKPMVNHAEASRLNIERMKQIYQQLSRYRGLGLLASVPSNPNGNG
GLMGYSPGENIPGCSSSGSCTPGSGILHYAHGDSQQTHLLKQGRSSTCTGLSSGKRPSQEEDTQSIGPKVQRQ
STN
>Piaya_caya__NWH71253.1
MMHRTIHLFRKGLRLHDNPTLLAALESSEVVYPVYILDRTFLTSAMHIGNLRWHFLLQTLEDLQKNLCQLGSY
LLVIQGEYESVLRDHVQQWNITQVTLDAEMEPFYKEMEVNIRRLGEELGFEVLSRMGHSLYDTKRILDLNGGM
PPLTYKRFLHILSLLGDPEVPVQTLTAEDFQRCHPPAPGLAEHYRVPLPVDLKIPSESLSPWRGGETEGLRRL
EQHLTDQGWVASFTKPRTIPNSLLPSTTGLSPYFSMGCLSVRTFFYRLSNIYAQAKHHSLPPVSLQGQLLWRE
FFYTVASATPNFTKMAGNPICLQINWNENAEKLHKWKMAQTGFPWIDAIMTQLRQEGWIHHLARHAVACFLTR
GDLWISWEEGMKVFEELLLDADYSINAGNWMWLSASAFFHQYTRIFCPVRFGKRTDPEGQYIRKYLPVLKNFP
SKYIYEPWTASEEEQKQAGCIIGEDYPFPMVNHKEASDHNLQLMRQVREEQQRTAQLTRDDTDDPMEVKVKRE
HSEEHTLKGKVAKMTE
>Piaya_caya__NWH72433.1
RFLLQCLEDLDANLRKLNSRLFVIRGQPADVFPRLFKEWNIAKLSIEYDSEPFGKERDAAIKKLASEAGVEVI
VRISHTLYDLDKIIELNGGQPPLTYKRFQTLISRMEPLEMPVETITPEVMEKCATPVSDDHDEKYGVPSLEEL
GFDTDGLPSAVWPGGETEALTRLERHLERKAWVANFERPRMNANSLLASPTGLSPYLRFGCLSCRLFYFKLTD
LYKKVKKNSSPPLSLYGQLLWREFFYTAATNNPRFDKMEGNPICVQIPWDKNPEALAKWAEGRTGFPWIDAIM
TQLRQEGWIHHLARHAVACFLTRGDLWISWEEGMKVFEELLLDADWSVNAGSWMWLSCSSFFQQFFHCYCPVG
FGRRTDPNGDYIRRYLPVLRGFPAKYIYDPWNAPESVQKAAKCIIGVNYPKPMVNHAEASRLNIERMKQIYQQ
LSRYRGLGLLATVPSNPNGNGNGGLMGYSPGESISGCGSTGGHLFPASLGAQLGTGDGHTVVQPCALGDSHTG
GSGIQQQGYCQASSILHYAHGDNQQSHVLQAGRTALGTGISAGKRPNPEETQSVGPKVQRQSTN
>Piaya_caya__NWH73114.1
PKASPRFTGPAQLFLLFSSSVLKVDYDVCSNYGNWLYSAGIGNDPRENRKFNMIKQGLDYDSNGDYVQLWVPE
LQSIKGADIHTPWALSSAALAQAGVTLGETYPQPIVIAPEWSRHVNQSLQGRSTHPRGRRAPAHTPVPHRNRG
IDFYFSHKKDV
>Piaya_caya__NWH74562.1
DNWAFLYAQRLALKQELPLHVCFCLVPKFLDATIRHYGFMLKGLQEVAKECAELNIPFHLLLGYAKDVLPAFV
VEHGVGGLVTDFCPLRLPRQWVEDVKERLPEDVPFAQVDAHNIVPCWVASPKQEYSARTIRSKIHTQLPEFLT
EFPSVIRHPHPPSCPAEPIAWEACYSSLQVDRTVEEVKWATPGTAAGLAVLQSFITERLKFFGSHRNDPNKAV
LSNLSPWFHFGQVSTQRAILEVQKHRSKYKESVDAFVEEAVVRRELAENFCYYNENYDSMQGSAYDWAQTTLK
LHAKDKRPFLYELQELEQGTTHDPLWNAAQLQMVREGKMHGFLRMYWAKKILEWTHSPEEALRFAIYLNDRYE
LDGRDPNGYVANTSSLCLAGCLWSIGGIHDQGWAERAVFGKIRYMNYAGCKRKFDVGEFERRYAPCT
>Piaya_caya__NWH75916.1

HLTYFASRIIELNGHKPPLTYKRFQAIISRMELPKKPVSTVMSQQMETCKVDIQENHDDVYGVPSLEELGFPT
DGLAPAVWQGGETEALSRLDKHLERKAWVANYERPRMNANSLLASPTGLSPYLRFGCLSCRLFYYRLWELYKK
VKRNSTPPLSLYGQLLWREFFYTAATNNPKFDRMEGNPICIQIPWDRNPEALAKWAEGKTGFPWIDAIMTQLR
QEGWIHHLARHAVACFLTRGDLWISWESGVRVFDELLLDADFSVNAGSWMWLSCSAFFQQFFHCYCPVGFGRR
TDPSGDYVKRYLPKLKGFPSRYIYEPWNAPESVQKAAKCIIGVDYPKPMVNHAETSRLNIERMKQIYQQLSRY
RGLCLLASVPSCVEDLSGPVTDSASGQGCSTSTAMRLSQTDQASPKRKHEGTEELCTEELFKRAKVTGLHTPE
IPGKSL
>Picea_sit_ABK24574.1_unknown
MAPAYTSQNFRDLEMIMDNNIQRVISPFSSSHRHQHQQRLHRPTPPPEPEQQQQEVIVSECCAVEAQKPNPIA
PPNLLASSASISLALGSILSSNLLPSRTGLLPTTPKTLTARLGSARPVRPSSLPAPKKRTSPLPVQASTALQV
PAPPPATSHTKATGVSAFPDYHRLRQSGRDAGDPSNAAALRKAAIVWFRNDLRVHDNEALSSANKEALSVLPV
YCFDPRDYGKSSSGFDKTGPFRATFLLECVANLRANLRERGSDLVVRIGRPEEVLVELAKSIGADALYAHQEV
SHEEIATEEKIKSSLEEEGVETKFFWGSTLYHPEDLPFKLQDMPSNYGGFRERVQRLSVRQIIKDPEQLKGLP
TRGDVEPGKVPSLLDLGLNPNFGQDGKTAVNASLTGGESEALQRLKRFAVECREQPNKLGKDGSGESLYGANF
SCKISPWLAMGCLSPRYMFEELKKSEKISISSSLGKSSGADSGQGGLNWLKFELLWRDFFRFITKKYGSAKKQ
QDASPVTACTGALAV
>Picea_sit_ABR16130.1_unknown
MNLIQSHAQKRDGFVKEFALKLGVDVFTPISHTIFSPEEIIKKNGGKPPLTYQSFIKLIGLPPLPLGNGSPSI
PPVGIDNSNDLPIPTLTDLGFGDVSQEECNPFIGGETEALKRMDASIQDQKWVAAFEKPKGDPTAFLKPATTV
LSPYLKFGCLSSRLFYQRLQEVYRKVKKHTEPPVSLEGQLLWREFFYTVAYGTPKFDQMEGNPICKQIPWNDD
DSLLSAWRDGQTGYPWIDAVMVQLRKWGWMHHLARHAVACFLTRGDLFVHWEKGRDVFDRLLIDSDWAINNSN
WMWLSCSAFFSQYHRIYSPISFGKKYDPDGKYVRYFLPVLKDMPKEYIYEPWTAPIDVQRKAKCIIGKDYPEP
LVSHLGASKNCKEKIAAAYALNKQIKGERNQVQLQGVKKNSSDSRNTDDVIKQPPKKLRQKKLTSY
>Picea_sit_ABR17701.1_unknown
MSTNCSIVWFRRDLRVEDNPALAAGVRAGAVIVLYIWSPEEEGQYYPGRVSRWWLKQSLAHLDSSLRNLGARL
ITKRSTNSISAILQVVQATGATQLFYNHLYDPLSLVRDHKLKQDLSSRGILVRSFNSDLLYEPWEVNDEEGHA
FSTFQDYWNKCLNMPYDPLAPLLPPKRIVAGEVSNCPSDDLNLEDESEKASNALLARAWSPGWSSADKALTAF
INGPLLDYAINRQKADSATTSLLSPHLHFGELSVKKVFHLLRIRQVLWANEGNTSGEQSVNLFLKSIGLREYS
RYLSFNFPSSHEKPLLSHLNYFPWRVDSDYFKSWRQGRTGYPLVDAGMRELWATGWLHDRIRVVVSSFFVKVL
QLPWRWGMKYFWDTLLDADLECDALGWQYISGCLPDGREMDRIDNPQFEGYKFDPAGEYVRRWLPELARLPTE
WIHHPWDAPRAVLQAAGVELGSNYPLPIVEISTAKERLQEGLSEMWQREADARASLENGTEEGLGESLENANA
SLPDQMDVDREPVRNNTDLAARSQDDQLVPSISNAVQRAYVGYTLSQAASSSPSEQAEVPSNTDSGIASAASS
QQFLVQSTWSMTRGLIEDLPNVTNDYPQMQVLDEQNESHSTADSSSILRGRDDMSSGGIVPVWSQSLSQSC
>Pimephales_prom__KAG1937016.1
MVVNTIHWFRKGLRLHDNPSLLDSVRGADTLRCVYILDPWFAGSSNVGINRWRFLLQCLEDLDASLRKLNSRL
FVIRGQPTDVFPRLFKEWKISRLSYEYDSEPFGKDRDAAIKKLASEAGVEVFVRISHTLYDLDKIIELNGGQS
PLTYKQFQTLISRMDPVEISAETITAEVMGKCVTLISEDHDYKFCVPSLEELGFETEGLSTAVWPGGETEALT
RLERHLERKAWVANFERPRMNSNSLLASPTGLSPYLRFGCLIVKKNSVPPLSLYGQLLWREFFYTAATNNPHF
DKMEFNPICVQIPWDRNPEALAKWAEGRTGFPWIDAIMTQLRQEGWIHHLARHAVACFLTRGDLWISWEEGMK
VFEELLLDADWSVNAGSWMWLSCSSFFQQFFHCYCPVSFGRRTDPNGDYIRRYLPILRGFPAKFIYEPWNAPE
SVQKIAKCIIGVHYPKPMVNHAEASRINIERMKQIYQQLSCYRGLGLLATVPFSTANNGDGNGAGPSSADIPQ
ESTRITVHPSRASSQPDFQPFRVSCDQHPVNSVQPQPGVY
>Pimephales_prom__KAG1969196.1
MSANKRSLKRQRESASAGGKQPKSAGEKGTEYGGKQPKPAGERGREPGWLLQEVSELRRAAQGCEFNKKRLRY
LSNTQKIKQGSDGVLYWMSRDQRVQDNWALIYAQQLALAEKLPLHICFCLVPRFLDAAYRQYAFMLKGLQEVA
KECKSLDIQFHLLSGEPGQNLPTFVKNWNFGAVVTDFNPLRIPLQWIETVKKHLPSDIAFIQVDAHNVVPCWQ
ASEKLEYGARTIRGKITKLLPEFLTEIPLVDTHPHAASQTAKPIDWEEVLSSLQVDRGIGEVDWAQPGTSAGM
AMLESFIDKRLRLFATHRNNPNFDALSNLSPWLHTGQLSAQRVVRQVKREKNASESVASFTEELVVRRELADN
FCFYNHNYDDISGAYDWAKKTLQEHSKDTRQYLYTKQQLENGKTHDQLWNAAQRQLVSEGKMHGFLRMYWAKK
ILEWTASPEEALSIAIYLNDRLSLDGCDPNGYVGCMWSICGIHDQGWAERPIFGKIRFMNYAGCKRKFDVAQF
ERKYAAIKASV
>Pimephales_prom__XP_039510234.1
MSVGKESISQVRELLRELMLGREDPDGCFCLCLSLLGDTDTRTHFLDMIEPLSSGHEHLHSQLTATFLEYFSK
DEADELELALTLSLNETKHIKTDFRQQNSVHEPHMPLCNKSYAEMTTGSSGGDQPKVNQPITKWTLDTEVHKT
TCAIKNINKDNDDLKVTKSKRSQRRLRKKELLLKNPSGPRPVLLWLRRDLRIWDNPALIGCLELGAPVIPVFL
WNAVEEEGPGVTVASGGASKYWLHQALVSLNLSLEQLGSHLVTLKGEPSSLTALQGLIAETGAASVVATALYE
PWLKERDDSVWETLEKQGVNCHIYHSYCLRDPYAVTTRGVGLRGIGSVSHFMSCCQQNPGSGLGAPLDAPGAL
PAPSTWPQGCPLVDLELARIPRRKDGTEIDWAVNIRKSWDFSEKGAQAHLEAFLRDGVYRYEKESSRADAPNT
SSISPYLHFGQLSARHLLWDARGARCRSPKFQRKLSWRDLAYWQLCLFPDLPWESIRPPYKVLRWSSDRAHLK
AWQRGFTGYPLVDAAMRQLWQTGWMNNYMRHVVASFLIAYLHFPWQEGYRWFQDTLLDADVAIDAMMWQNGGM

CGLDHWNFVMHPVDAALTCDPYGTYVRQWCPELKALPDDLIHKPWKCPASMLRRAGVVLGGNYPERVVTNLEE
RRAQSLQDVASVRRRFRQFIDQRSGCDLVPLPARLVQEALGSMEDVVRHEGNGFLLPVITRMEFKHHSEDPDR
QDNPYNAVLKGYVSRKRDETIAFFNERDFTASVMCETAQRRERLERDCCLLEGLPQPTASRGRARRTPTRDPY
SKVPGGVPIPHR
>Pimephales_prom__XP_039522870.1
MSASRTVICLLRNDLRLHDNEVFHWAQRNAEHIIPLYCFDPRHYQGTHNFNFPKTGPFRLLFLLDSVKDLRAT
LKKRGSTLLVRQGKPEDVVGDLIKQLGSVSAVAFHEEVASEEKTVEKKLKDICCQNKVKVQTFWGSTLYHRDD
LPFRHIGGLPDVYTQFRKVVEGQGRVRPVLSTPEKVKSLPLGLEEGLIPTFEGLGQTEPVMDCRSAFPCRGGE
TEALDRLKHYFWDTNAVASYKETRNGMMGVDYSTKFSPWLALGCISPRYIYDQIKKYEAERTANQSTYWVIFE
LLWRDYFKFVGLKYGDRIFNINGLQDKQVSWKTDMTLFCAWKEGRTGVPFVDANMRELALTGFMSNRGRQNVA
SFLTKDLGLDWRLGAEWFEYLLVDHDVCSNYGNWLYSAGIGNDPRENRKFNMIKQGLDYDNNGDYVRQWVPEL
GGIKGGDVHTPWTLSNSALSHAKVLLNDTYPSPIVIAPEWSRHVNNKSSGPSSAKGKRGSSYTPRQHKDRGID
FYFSKKKGF
>Pimephales_prom__XP_039533080.1
MSQNSVHWFRKGLRLHDNPALIAALKDCRHIYPLFLLDPWYSTNTRIGINRWRFLMEALKDLDSSLKKLNSRL
FVVSGPPTEVLPKLFEKWEITRLTFEVDTEPYSRSRDEEVMKLAKEYGVEVIPKISHTLYNIDRIIDENNGKT
PLTYVRFQSVLKAIGPPKKPVPAPTKEDMSGVSTPISEDHEEKFGIPTLEDLGLDASSLGPDLFPGGEQEALR
RLDEHMQRPGWVCKFEKPQTSPNALTPSTTVLSPYVRSGCLSARTFWWRLSDVYRAKKHSEPPVSLHGQLLWR
EFFYTTAVGIPNFDRMEGNPHCVQVDWDNHPEHLAAWREARTGFPFIDAIMTQLRQEGWIHHLARHAVACFLT
RGDLWISWEEGQKVFEELLLDADWSLNAGNWQWLSASAFFHQYFRVYSPVAFGKKTDKNGDYIKKYLPLLKKF
PAEYIYEPWKAPRSIQERAGCIVGKDYPRPIVDHEVVHKKNIQRMKAAYAKRSPEDKPITKGVKRKATSTVDQ
LQKKAKR
>Pimephales_prom__XP_039538549.1
MSHRTIHLFRKGLRLHDNPGLQGALASSAVLFPVFVLDRALLEETTAVAPTRWRFILQSLEDLDARLRALGSR
LYVLRGSTATVLRDLVTRWSITRISYDTEVEPYHARVERDIQALAEERGLQTHTCVSHTLYDVQRIIQANGGS
PPLTYKKFLHVLSVLGEPEKPCRDVSAEDFLRCPTPADPELAYGVPSLADLGLEVQSETLWPGGETHALHRLH
THFQSQGWVSSYCKPRTVPNSLLPSTTALSPYLSLGCLSVRSFYHRLASVYAQSKNHSLPPVSLQGQLLWREF
FYTVAAATPNFTKMEGNPICLQIRWYHEPESLEKWRTARTGFPWVDAIMTQLRQEGWIHHLARHAVACFLTRG
DLWISWEEGMKVFEEFLLDADYSLNAGNWMWLSASAFFHKYTRIFCPVRFGRRTDPQGQYLRKYLPVLKNFPS
QYIYEPWKAPKDVQLSAGCIIGKDYPCPMVCHTEASQRNLGLMRQVRTEQQKTAELTRDVTDDPMEAGLKREL
QEEEELLEGAEPHRGVKRLSGSSDQKSRPSSWTPETVRLSEDVT
>Piptocephalis_cyl_RKP13396.1_photolyase
MSTRGKRSESTQTSTDPPRSKGAKRVKEDEANRTLVWFRRDFRLHDHMALARASASTPASQSHLLALYLISPE
EWIDHDEAPIKVDFWLRALPILRKDFAALHIPLIIRTLDRAKGQHADVILDVCKQYRISAVHWNREYEVDELA
RDASVQQLLQTHDRQVYQHHDQCIVPPGQVLTKAGTTSKVYSSFRNSWYTVVARNPSLLKESRFPRANEPSAL
RELHEAFEDGHIPESLDQFPLAESARERARRDFPVGEEEAKKRLHAFIQKKMGMYGENRSSLALQGTSILSPY
LHSGMVSARSCIREAKEANGNRLEGGMAGAVNWMAEVVWRDFFRHILVAHPKVSRNQPFKAEFTRFPWASDSG
DRSFQAWTQGRTGYPIVDASMRCLNTTGWLHNRSRMVVASFLCKDLLVDWRKGERYFMQHLLDGDLASNNGNW
QWVAASGTDSQPFFRIFNPARQSQRFDPDGAFIREWVKELRDCPKEDIHAPSASIAQRLKYPEPIVDHSKARD
RALKAFQQMRSAS
>Pityrogramma_aus_AAQ18175.1_type_II_CP
MEEEAEEVHADGPVIVSTEVNPGRVRLLKQASQEVVQGGPVVYWMSRDQRSRDNWALLYAAQCAQQQGGPLCV
AFNLVDSFLHAEARHFGFMLRGLRVVHHNLSSLGIPFFLLRGKAEENIPAFVERCGASILVMDFSPLRIGRVW
REGVCKAVPSSVSVFEVDAHNVVPVWVASDKQEYAARTIRGKIQRHLPEYLVEYPPLVAPIHTWTLEKPANID
WDLLIDEVAKKGAEGLEVKWCESREDAAMELLLGKKKGFLTTRIRNYADDRNDPSKPAGLSGLSPYLHYGQVL
KKKGFLTTRIRNYADDRNDPSKPAGLSGLSPYLHYGQVLAQRCALEGRKLRRSHTKAIDTFLEELVIRRELAD
NFCYYQPNYDSLQGAYEWARSTLLAHASDKREHLYTQEELEKGKTHDELWNASQLEMVHLGKMHGFMRMYWAK
KILEWTGGPEEALAVAIYLNDKYELDGRDPNGYVGCMWSICGIHDQGWQERPVFGKIRYMNYNGCKRKFNVDG
YIQYVKRMVAELKRAAKDPAKIPPSSTPAKKDILE
>Plakobranchus_ocel__GFO07232.1
MAAANLKYGICLFRNDIRVHDNEALFLANQRSDFLIPLYCFDPRHFKGTYHFGFPKTGHHRACFMIDSVQDLR
SSLQSLGSNLLVRQGKPEQVVLDLIRSLDLKNVTVFLQDESMDEEKRVENAIRSKSSVSVTSIWGHTLYHVED
LPFQPKHLPDVYTEFRKRVEGRASIRKCFTMPPSLKPLPTDLEEGNIPTMSDLGLPVLQKDERSVFPFKGGES
SGLNRLQDYLWGTDHVSTYKETRNGMIGSDYSTKFSPWLAHGCISPRKIYWEIKKYENERTSNQSTYWVIFEL
IWRDYFRFVGLKYGNQLFHPGGIRGNEVNWRSDNAQFSAWKEGKTGVPYVDANMRELAATGFMSNRGRQNVAS
FLTKDLKLDWRLGAEWFESLLLDHDVCSNYGNWLYSAGLGNDPRENRKFNVVKQGLDYDGDGDYVRLWLPELA
GVKTGKVHCVWTLSPSVLEAANVSLGETYPSPVVMAPEWSRHVNRPTSSMGSGKGRTGAPGSARGKGQGSSSY
RGHGSQAQYPGQKRGIDFYFSSSNKR
>Plakobranchus_ocel__GFO20146.1

MDSKETDSSQADEGKKDSHITVHWFRHGLRLHDNPALLEGLKNCKEFYPIFILDGHVAGVSTAAFPRMQFLFE
TLEDLDNNLQALGTRLYFLKGDPVEVFEKLFEEWDVTRVTFEQDPEPIWQDRDSRVKELCNLRGVECIEKVGH
TLWDPHVILQENGGNPPLTYAAFNQVVGIVGLPEKPLPNPDFEGISLPVGEDHDSKYGIPSLKSLGVEPESAE
QASPHGRYLGGETKALKLLLSRLEKERKAFELNLSLPNQLYPDLVGMPMSLSPHLRFGSLSIRKFYWALRCTY
AQVHPNSPIPKSITSQLVWREYFYCMSVNNPLYNRMEGNPICLRIDWYNDEEKFKLWKEGRTGFPWIDACMRQ
LVQEGWIHHVCRHAVACFLTRGDLWIDWQKGLEVFDRYLLDADWSVCAGSWMWVSSSAFEKVLQCPRCICPVR
YGRRMDPTGNYIRRFVPELKDMPLRYLCEPWKAPQKVQEDANCVIGVDYPLPMVNHKDASKECKEKMDRIKNL
CKDIPHMAPTNENEVLSYMWLDKSNSGAMEESLFEACSHLSSVAL
>Plakobranchus_ocel__GFO20945.1
MSQKCHKAIHWFRKGLRLHDNPAFIEACKDASELYPVFVLDPWFVTNANVGANRWRFLAQTLNDLDNNLKSLH
LRLFVVRGNPEIELEKLCKKWEITKLTYEVDTEPYAVARDKCVDLKMDNLGVSVVKCISHTLFDIHKVVKVNG
GKAPLTYQSFQTAAARMGPPAQAVASPTLQDLQGCRTPIADTHDKEFGVPTLKDLGICSEDLGPNLFQGGETE
ALERMERHLKKVNWICAFEKPKTEPNSLVPSTTVLSPYLKFGCLSARLLYHKIIQVYKGKKHSQPPVSLLGQV
LWREFYYTVAAVTPNFDKMEGNPVCKQIPWQENSEHLDAWKMGRTGYPFIDAIMIQLRQEGWIHHLARHAVAC
FLTRGDLWISWEEGQKEFEKLLLDADWSLNAGNWMWLSASAFFHQYFRVYSPIAFGKKTDPNGDYIRKYLPVL
NKFPAKFIFEPWKAPLTMQQAASCIIGKDYPKPIVDHDTVRQKNIQKMAAAYAKSKNGASNDVKDLATSKKRP
SSDASNSPVKKKKKA
>Plakobranchus_ocel__GFO43536.1
MDSAPENNAAEQSSGTLTPSPTLKKHALFWFRKGLRLHDNPALIAAIQGSATYRCVYILDPWFAGASQVGISK
WRFLLESLEDLDSSLRKLNSRLYVVRGQPADVLPRLFQEWSITTFAFEEDSEPYGKERDAAISALAREFNIQV
ISKSSHTLYDPKVVIAANGNSPPLTYKRFQSILSTLPPPEKPCETLSAFAVSSVTTPVSHDHNDKFSVPSLDE
LGFDTDSLVPATFRGGESEALARLNRHLERKAWVASFEQPKMKPQSLYPSGTGLSPYLRFGCLSPRTFYWKLT
ELYKRVKKGVDPPLALHGQLLWREFFYTVATNNPNFDRMVGNSVCVQIPWDHNPEALAKWAEGMTGFPWIDAI
MVQLRREGWIHHLARHAVACFLTRGDLWISWEEGMKVFDEMLLDADWSVNAGMWMWLSCSAFFQQFFHCYCPV
AFGKRADPSGDFVRHYLPVLKGMPTQYIYEPWTAPESVQKAAMCIVGKDYPLPMVQHSVVSQINLERMRQVYK
RLVLKSSARNRMFSGLPKMPKVDIFLTKVKMFLSTSSDEEVSQKQRRRSKHINDC
>Plakobranchus_ocel__GFO45498.1
MAEQEEFAISADLVNKLRSTSYRAQVAYRQIKTASNLTMAEEGSSKEEIKAAAGESSTKTKEKAAKPTKGEKK
EKSSSNEKDQKEVKDKQKPKNAERPVRNVEKRKAEKSLQEESPAKKLKAENGASEPEPTEDVDKMESDDFIKK
IDQRRKDVCEDVSKFKFNKKRVRVLSEEEDFSDESNGVVYWMSRDQRVQDNWAFLYAQRLAMKLEVPLYVCFC
LVPKFLDATHRMFDFMLKGLAHVEKECRNLNIPFYLLYGDASKAIPPFLKEHNIGGVVTDFTPLRVPAKWVED
LKNALPSDVPLCQVDAHNLVPCWEASPKLEYGARTIRNKIHNQLGQFLTEFPPLVKHPHPPKKMPSNIDWKKA
DATLQVDRSVAEVTWAKPGSEAAYKTLQSFCEKRLKHFATLRNDPNKQALSNLSPWIHFGQISVQRCVLTVRL
YRSKSSESVNAFIEEAVIRRELADNFCYYNKHYDSIKGAYDWAKKSLALHKGDKRPYLYTRDQLETSQTHDDL
WNAAQFQLVLEGKMHGFLRMYWAKKILEWTTSPEEALAISIYLNDKYSLDGRDPNGYVGCMWSICGIHDQGWA
ERAVFGKIRYMNYQGCKRKFDVPGFVLKYKKKKMEMKK
>Planoprotostelium_fun_PRP73543.1_PROFUN_025
MKHVTHNGNSNIMPPRVIYWFRTDLRLHDSPALSKALELEPECLYPVWTWDDHYVFHARVGVNRWQFLLDSQS
QLSENITKINPKSKLHVLRGPPLQILPIVVKQWKITHLVFEKDTDTYGRSRDDQVMEMMRKLKVEVISVSGRT
LWDVDEVLKRNKGKPPNTINALSKITEKMGPPEHPLPTPTSLPDPGDLDLDIESQSVDDAEDLNAEQREKPAV
CYDSIRGPKGDFSVPTFEELGLKPATSPWRGGESVALKMLTEYTDDIEKTAKFSKPDTPPTQFLPPATTNLSA
HLHFGTLSVRTFWYKVEESVAKFKGKKSSFPTNLPGQLMFRDMYFCCQAGWENFERMEGNKECRFIPWALRNS
YVLSHTYRTSHVISYDEEGRVNGQERDGEAEEWFTRWKEGRTGFPWIDALMRQLKHEGFIHHLGRHSVACFLT
RGHCYISWERGQEVFEEWLIDHEAACNSGNWQWLSASAFFHQYHRVYSPIAFPKKYDKTGRLVRHYCPELSKF
PDKYIYEPWRAPKAEQKAAGCIIGEDYPLPMFDEGERRKRCLDAMGHAYKGVGDERLRDGSWRELFPEVKVKK
EKVKEEKGGKVKEEKVKEEKGGKVKEEKEERGKKRKGTLDEYVSKKEEEEEEEEEEEEEEEEEEEEEEEE
EEEEERGGKRGKDKKEKEKLASMGPSKPKRRKR
>Planoprotostelium_fun_PRP73583.1_cry_DASH-l
MADNNQSFSLVWFRGDLRLHDNEPLTMAIKQSQGRILPFYCFDVRQFGPTYSFGFPKTGPFRAEFLIESVQCL
RDGLRKLGSDLIVRVGKPEEEIPKLIKSLNVPVTHLFGQKEITSEETDCEKSVHRALGKLDKKVESKLVWGGQ
TMHHIDDLPFQVEGGHPLKVFTHYRTMMESKNISVRQPFPSPSKLQSLPKTGKEDREVSVGDLPDLDQLMAIG
NFGKWLQDHVQRDPNYKGDEIVKEGPTSNSKKHRDPAQVMTGGEVAGLKRLHFYLWEGRHIDTYKSTRNGLLG
STYATKFSPWLATGCLSVRQIYHEIKKYESKHGANDSTYWVTFELMWRDYFKWVGLKHKNDIFKLYGLKGEKK
DEQREWKKDVSTFEMWARCKTGYPFVDANMRELTATGHMSNRGRQNVASFLTKDMKMDWRMGAEFFESFLIDH
DVTSNYCNWLYVAGVGNDPKENRYFNVVKQGHMYDSKGEYVCYWFPQLKKLPDADHMMQPFLYKGPEGTFSLD
RDYCEPVVKVNFKKEGGDHHRSHGKKDFKIKRGGHGGRTVRDGAPQHSSTH
>Planoprotostelium_fun_PRP75200.1_PROFUN_159
MLSVKCCYSLYRYGVIQVLCCVSASFKNGLFDSGSDDVLTMPGEENLLLRKTRGKNIMPATRGKRKERSPTES
EKDPHKEEASNAQKSSRSESEKKAPKLNQEESEKGRAKRYERVSNNTNNTSHPRLGVKAREKQGMYNKNAQKG
EPDPTAIGGSFEGTPLDELEKVMKKSEVEFKKAKNGNVLMWIRNKDFRAFERASQAAKSMGTHVIALHVISPG

DYKSHGRGARRIDFMMRNVKELRSELKEKNIPLVVRTITPRKTVPKEIISFCDEFNITKIYANIEYEVDELRR
DAVVVHLARESGKAAFFVHDCIIVPPGMLLTKEGKPYSVYGPWFKKWVETVNENDNLLETSEDPEANDESVRK
DKSLHSLFEEEIPSEVEGYECADAEKMAQLWPAGYHSARESLGRFLEGKKGKEADDIFQSSKVKEKKAAVENY
PEGRNRPDVDGTSRLSPYLAAGVISPREVVQKARKMNKGSISQGGKENGFSNWISEVAWRDFYAHVIVAWPRV
VMGRAYNLAMESVCWEYEEDQVKAWKEGNTGYPIVDAAMRQMRETGFMHNRCRMITAMFLVKDLMVDWKNEVG
EAYFMQHLMDADFASNNGGWQWSASTGTDPQPYFRIFNPFSQSEKSAQSYSDPDGAYIKRWVPELKSLSGKAL
HDPFHKLDKASFKKLGYPEPIVDHNEARKRALRRFKTPGEK
>Plasmodium_viv_SCO67762.1_photolyase
MKEQAGITSEPGEVPNERGRNTLLVSERGKEPNAHSEGPPTDPRGETHEEKQPLRQEVPTRSITTGEEEEKNK
NKKKKKKTEHVIKENTKSDIKGEKTNKESPKMNRPEDQNGKGANLDEETFVKKAPSDDPLLIPGEGRASKMGT
NKAQTSKGEEKSKLAKLTKLMQGRGSKQDEVGRMGNPVRGKNGSLGRGGGQVSGKDGSLESKNRLVERRNGSA
RTPKGSLGSSGGLKNSPAGCSGGPKNSPAGFSPNSAPRSTSKFVSKSAPKLASKLAPKLVSKVAPKLAPKLAP
KSTLNSAPSAKPHTPRSGPASAPTLNRFKCASLKKVPPSTPQKPKGKTNFPSVHPPLRKESSTPRGSEDQRKR
NPYETSREKKLTILAKRVRCLTSFESGAESGGGGDAAKMRCSAESSGGGDAAKMRCSTEGSGGGGDASNPPSI
PPGEEPNHASPTSIPHGEEPNHASPTSNPSSAANEQRDALLLLLTRDFRANDNWALTYAYELAKKKGLNLIAC
TYLNRKEKLTSRFINAKLKVLKNLEQALKQMNIPFYILPLFMIDEFKEFIKTHHIKVIACDLHVLNDERAFIK
SLCHICNKRKVKVFQVDSHNVIPIWVTSKGEECSVRTIKPKIQALLPTFLTEYVLLSPFEQKIKFPEPFDMEE
VASKLTVTNTCAVVSGAVFTEKKAQELLSHFCRQVLDKYNVKRNDPNSDAATVLLPYVNMGVISAQRCILEVH
KHAYSNPSINAVSGKESFTDDFIMRKELADNYCYYNRNYCTFEGGRDWAKESLKKHDADKREYLYDYEDFRTA
KTHSDLWNCCQLQLIDEGVMHEFLKVYWAKKILNWSANSQVALKYAMQLNEEFSIDGKTPSGYVSIMWSIMGV
HDQGWNERNIFGKVRFINSNGCKRNFDMNMFMSKYPKGKENASIVKKIPTITFASYLKKRKHGPAAPLQESTR
EKRGRGPAR
>Plasmopara_hal_XP_024571475.1_dna_photol
MKVLVWFRRDLRLHDNLALHAALQHIEKHKNKDDVEFIPLYITHRPQIMRCGAHRFQFVLESVEDLSRALEER
GSKLVVAKGDGLQVLQRLLPAWSITHLFFDRVCEPYAVERDNRALELAKSLGIKTHVTNGYNLYDLDQVISSQ
SNKAPKTYRAFLKAVGEQSLPSEPLPTPEKLPSPMRKHHELYQQVVEYWNNCEMSDDREKEDDPKEKKKQIEQ
IAGPKHDFTVPDLAFFDYETPEKHSFIYGGEQKALKTLDNYCKDEDRVARFEKPKTSPAQPPPTPSTTVLSPY
MFYGCLSPRTFYHRVQDIQKRRKKGSQPPVSLIGQLLWRDFYHCHGHDNPYFDKMEESPTCLQVNWRYHTIPE
NEEEMSDDEKLARSQFEAWRDGRTGFPWIDAIMIQLKEEGWMHHLARHAVACFLTRGDLYISWVRGLEVFQER
LIDHDWSVNAGNWLWLSSSYFFTSYFRVYSPISFGKKSDPEGLFIKKYIPALKKFPAKYIYEPWKAPKSIQQD
AGCVIGKDYPTPIVEHKSAMQRCKEGMKMSYANKEYGTPPSNKSNRRSKKRQREDSDEE
>Plasmopara_hal_XP_024580044.1_photolyase
MATVFRALEKLKLPGGLHRERIQWLYEGATLKSDGSYLLYWMQTSVRTKQNYALEYAVAAANELKVPLHVVYL
FPDRSVLPALQSSKGDEWKAEALQDANAHAFATERHALFALEGLADVHQRLVGRGLSFDVFHHQQNKKRLARN
ELLEICARKAALVVTDRPYLRPWRSALDNLVSSVEDQGANYGIVQIEGDVVVPVAEVTDKEEFSARTIRPKIM
KLVDRYVASLEVMVPDKVCKTEGQTLLAILEKPEQLKVLDVTSPVAVDKTLQLLDVDHSVKSVGWHFKGGEVA
ASACAKEFLTPDKLASYATERNEPSLDAGSDLSIYLRHGHICVVRIVLAAQKLKKVAKAKEGLASFLEELIVR
RELSLNLVTFNHQNYDSMRCLPKYAQTTLKDHIDDKREKIYSREELESYQTEDRFWNAVQRELVVTGKMHGYM
RMYWGKKLLEWTRTPEEGFHAALVLNNKYALDAPDANSYAGVAWTFGKHDQGWRERPIFGKVRYMNETGLKRK
FRMDAYVHKVGRLAAQAKKASKGGDLDDEKHSSSSVGHKKDASIGKWEQN
>Platynereis_dum_AEJ87227.1_putative_l
DGEVAGTKLVSYPRMKFLLECLKDLDDSLKKHGGRLYVVKGPSDVVIKQLIEEWGVTRVTCEIDPEPIWQPRD
KAVKDLCATKGVKWFDYNSHLLWDPKAVCDANGGRPPHTYKLFCQVTDLLGKPETPHPDPDFSHVQMPVSDDF
DDKFGLPTLKELGCEPECEEQEKPFNKWQGGETGALELLETRLMIERTAYKAGYIMPNQYIPDLVGPPRSMSP
HLRFGALSIRKFYWDLHNNYAEVCGGEWLGALTAQLVWREYFYCMSYGNPSFDKMEGNPICLQIPWYKDEEAL
EKWKQGQTGFPWIDACMRQLRYEGWMHHVGRHAVACFLTRGDLWISWVDGLEAFYKYMLDGDWSVCXRQLDVG
>Platynereis_dum_AGX93012.1_tr-cry
MWQQIFSPPNTVVEGKNIVHWFRKGLRLHDNPALLEALKGASTWRCIYILDPWFAGSSQAGISKWRFLLQCLE
DLDASLRKLNSRLFVVRGQPADVLPRLFVEWSVKVLSFEEDPEPFGRERDAAICALAQEAGVDVNIRTSHTLF
APQKVIERNDGQVPLTYKRFQSIIAGMEVPCQPENCPTTEMVMNCRSPTNDDHDETYGVPTLEELGFDTDGLR
PAVWKGGETEALSRLDRHFERKAWVASFEKPKMTPNSLLASPTGLSPYFRFGCLSPRLFYWRLTELYRKVKKR
QDPPLSLHGQLLWREFFYVVSTNNPKFDRMQSNPICVQIPWDKNPEALAKWAEAKTGLPWIDAIMTQLRQEGW
IHHLARHAVACFLTRGDLWISWEEGMKVFDEMLLDADWSTNAGSWMWLSCSSFFQQFFHTYCPVNFGKRTDPN
GDYIRRYLPVLKGFPARYIFEPWTAPESVQRAAHCIIGKDYPLPMVNHQEASRINMERMMQVYQQLSPRAGHL
GRLMLRGRSNLADPRLKTDKFGIPMTGNKPSKALVRPRLDTQYLSATATTAVTQSSSDSETPTNSSNQLNLRN
K
>Platynereis_dum__AGX93015.1
MPEVAKSSIHWFRKGLRLHDNPALLKAFENANEVRPIFILDPWFVKKGRVGINRWRFLIQTLRDLDTNLKELG
SRLFVVRGNPLDELPKLFKEWKVDHLTFEVDTEPYAKERDEKIEELASELGVKVSKIVSHTLYDTQRTIAKNG

GTAPTSYQRCLTVLSQLGPPAKPAPKLSAEHIKDCKTPIGSDHEEKYGVPSLAELEKKESDCGPSLFPGGETE
ALSRMEKYLKKTSWICKFEKPETEPNSLAPSTTVLSPLPEVWLRLSENILLES
>Platynereis_dum_AIE45779.1_cry,_parti
HYEHQKWSNDQSHLKAWQKGNTGYPLVDASMRQLWLTGWMNNYMRHVVASFLISYLRLHWIHGYEWFQDTLVD
ADVAINAMMWQNGGMSGLDQWNFVMHPVDAAMTCDPKGNYIRKWIPELAALPDEFIHIPWKCPPSILRRAGVS
LGGNYPQRIIIDLETSREQSLKDVTEVRRKFGRDWVDKITGNDLVPLPNGLKIPCITRKEFKYKTANPESREN
PHTAVLRGYRSRKRDVKIAFENKVDFLASTMNECVLRQER
>Platynereis_dum_AIE57497.1_CPD_photol
LCNFGGKLTARKSCHLRFRQSPGPVIYNKKKMSEPPAKKIKTEEEKVEKDFLQKIENQRRDTAKSVADFKFNK
KRVRVLSSQQEFPDENKGIVYWMSRDQRVQDNWAFLYAQKLALKAEVPLHVCFCLVPKFLEATIRHYGFMLKG
LREVEEECRKLDINFHLLIGYAKDKLPEFVKEQKIGGVVTDFSPMRVPAGWVNEVKKLLPKDVPFCQVDAHNI
VPCWEASPKLEYGARTIRSKIHKVLSTYFTEFPPVIRHPHKATAKPEPIDFEAVQESLDVDRTVGEVDWVTPG
TTGGLRNLESFIKQRLKYFNADRNDPNKNALSHMSPWYHFGQVSVQRGMLMVRQYRNKYKEGVEGYIEEGIIR
RELADNFCYYNENYDNIDGAYEWARKTLNDHKKDKRTHIYSREQLEKAKTHDDLWNAAQMQMVTEGKMHGFLR
MYWAKKILEWTESPEKALADAIYLNDRFNLDGRDPNGYVGCMWSICGIHDQGWKEREVFGKIRFMNYKGCQRK
FDVAAFVRKYRAKKI
>Platynereis_dum_ARB18112.1_CryDash
MSKPRVIICLLRNDLRYLDNEALLWAHRQGTHVLPIYCFDPRHYKGTYHFSLPRTGPHQLKFMLESIRDLKST
LQKHGSDLILRQGKPEVIVPAIIQQIGKENVSALVLQKEVTDEEVKVEEGLKKSCGVQLQTIWGATLYHLDDL
PFKPQRLPDVYTQFRKTVESQGRVRPLVEMPETLRPLPEGVTSEDVPTATQLGCQESTFDERSAFPFTGGESS
ALARLKSYAWETDAVAKYKETRNGLIGHEYSTKFSSWLAHGCISPRQIYWEIKNYESQRVANQSTYWVIFELL
WRDYFRFVGMKYGNKLFFEGGIFGKQIPWKRDENLFKAWKEGRTGVPFVDANMRELAATGFMSNRGRQNVASF
LTKDLKLDWRMGAEWFESMLIDHDVCSNYGNWLYSAGIGNDPREDRKFNMIKQGLDYDAEGDYVRLWVPELKG
IQGGQVHTVWTVGKGILERAGVSLGETYPMPIVQAPEWARHFGKTGGARAKSGRGPPPTKQNRGIDFYFKST
>Platysternon_megacephalum_TFK00652.1_Cryptochro
MPNMPHRTIHLFRKGLRLHDNPTLLAALESSEVSLEDLQRNLTKLASCLLVIQGEYESVLRDHVKKWNITQVT
LDAEIEPFYKEMERTIQVLGGEMGFDVLSLMGHTLYDIRWILDMNGGTPPLTYKRFLHILSVLGDPDMPVRNL
TAEDFQRCKPPPELRLAECYRVPLPEDLEIPRESQSPWRGGETEGLQRLEQHLKNQGWIANFTKPKTIPNSLL
PSTTGLSPYFSLGCLSVRSFFYRLSNIYAQSKNHSLPPVSLQGQLLWREFFYTVASATPNFTKMAGNPICLQI
NWYQDAERLHKWRMAQTGFPWIDAIMTQLHQEGWIHHLARHAVACFLTRGDLWISWEEGMKVFEEMLLDADYS
INAGNWMWLSASAFFHQYTRIFCPVRFGKRTDPEGQYIRKYLPVLTNFPSKYIYEPWTAPEEAQKQAGCIIDY
PLPMINHKEASDHNLQLMKQVREEQYGTAQLTRDDMDDPMEMGLKRERSEENSKKGKHARNREQT
>Platysternon_megacephalum_TFK04765.1_taste_rece
MGVNAVHWFRKGLRLHDNPALRECIQGADSVRCVYILDPWFAGSSNVGINRWRFLLQCLEDLDANLRKLNSRL
FVIRGQPADVFPRLFKEWNIAKLSIEYDSEPFGKERDAAIKKLASEAGVEVIVRISHTLYDLDKIIELNGGQP
PLTYKRFQTLISRMEPLEMPVETITAEVMEKCTTPVSDDHDEKYGVPSLEELGFDTDGLPSAVWPGGETEALT
RLERHLERKAWVANFERPRMNANSLLASPTGLSPYLRFGCLSCRLFYFKLTDLYKKVKKNSSPPLSLYGQLLW
REFFYTAATNNPRFDKMEGNPICVQIPWDRNPEALAKWAEGRTGFPWIDAIMTQLRQEGWIHHLARHAVACFL
TRGDLWISWEEGMKVFEELLLDADWSVNAGSWMWLSCSSFFQQFFHCYCPVGFGRRTDPNGDYIRRYLPILRG
FPAKYIYDPWNAPESIQKAAKCVIGVHYPKPMVNHAEASRLNIERMKQIYQQLSRYRGLGLLATVPSNPNGNG
NGGLMGYSPGENISGCSSASGAQMGSNDGHTVGVQTCSLEDSHAGSSGIQQHGYSQGSSILHYAQGDRQQSHL
LQQGRTGSTGISTGKRPNPEKETQSIGPKVQRQSTN
>Platysternon_megacephalum_TFK06320.1_suppressor
MKHISIHWFRKGLRLHDNPALLAAMTDCRKLYPIFILDPWFPKNMRVSVNRWRFLVESLKDLDESLQKLNSRL
FVVRGRPTEIFPVLFKEWKVTRLTFEIDTEPYSRQRDSEVVRLAAEHGIQVIQKVSNTLYDTDRVIAENNGKV
PLTYVRLQTLLAALGPPKRPVPAPTLENLKDCHTPCKDNHDAEYGIPTLEELGQDPEQAGPRLYPGGESEALC
RLDLHMNRMAWVCNFQKPQTEPNSLNPSTTVLSPYIKFGCLSVRTFWWRLAEIYQGRKHTNPPVSLHGQLLWR
EFFYTAGAGIPNFNKMEGNPVCVQVDWDNNPEHLRAWSEGRTGYPFIDAIMTQLRTEGWIHHLARHAVACFLT
RGDLWISWEQGQKVRHVARGSAEMVGGLQDLHG
>Platysternon_megacephalum_TFK15166.1_BTB/POZ_do
MAAAGSTPVGLARSVHWFRRGLRLHDNPALQAALRDASSVRCIYILDPWFAVSSAVGINRWRFLLQSLEDLDN
SLRKLGSCLFVVRGQPTDVFPRLFKEWGVTRLTFEYDSEPFGKERDAAIVKLAKEAGVEVVIENSHTLYDLDR
IIELNGHKPPLTYKRFQTIISRMELPKKPVSSITSQQMEKCKSEIQENHDDTYGVPSLEELGFPTDGLAPAVW
KGGETEALARLDKHLERKAWVANYERPRMNANSLLASPTGLSPYLRFGCLSCRLFYYRLWELYKKVKRNNTPP
LSLYGQLLWREFFYTAATNNPKFDRMEGNPICIQIPWDRNPEALAKWAEGKTGFPWIDAVMTQLKQEGWIHHL
ARHAVACFLTRGDLWISWESGVRVFDELLLDADFSVNAGSWMWLSCSAFFQQFFHCYCPVGFGRRTDPSGDYV
RRYLPKLKGFPSRYIYEPWNAPESVQKAAKCIIGVDYPKPMVNHAETSRLNIERMKQIYQQLSRYRGLCLLAS
VPSCVEDLSSPVTDPTSGQGSSTSTVQRLLHCGQTSPKRKHEGVEELCTEELYKRAKVTGLHISEIPSKNL
>Platysternon_meg_TFK04422.1

MSGAAPRTVICLLRNDLRYHDSEVLRWAQNNADYVIPLYCFDPRQYLRTYYYGFPKTGPHRLKFLLESVKDLR
QTLKKKGSNLVVRKGNPEDVVHDLITQLGTVAAVAFHEEATKEELDVEKALQRVCAQHGVKIQTFWASTLYHR
DDLPFRHISRLPDVYTQFRKAVEAQAKVRPTVQMPDQLKPLAPGVEDGCIPVPEEFGQKDPLTDPRTAFPCSG
GETQALMRLQHYFWDTNLVASYKETRNGLIGIDYSTKFAPWLALGCISPRYIYEQIRKYEKERTANQSTYWVI
FELLWRDYFRFVALKYGTRIFLLKGLQDKDVPWKKDPKLFDAWKEGRTGVPFVDANMRELAATGFMSNRGRQN
VASFLTKDLGLDWRMGAEWFEYLLVRPNSTCDWGGLSFSLQVADNRPPCMGLKGTELPLSSIPALLEEGSYNL
VRSF
>Platysternon_meg_TFK04899.1
MSQKSRNRQARGGEEISEDAATPGAAAAPRGQLKGKRKVLAEPSGAEALTVDKRRKEGEGAEGAGAGSLGEAV
RQARLRVAPSVQDFKYNKKRVRLVSQGSDLKDAAQGVVYWMSRDQRVQDNWAFLYAQRLALKQQLPLHVCFCL
VPKFLEATIRHFGFMLKGLQEVAEECQELDIPFHLLIGFAKDVLPAFVMGRGLGGVVTDFSPLRVPMQWVEDV
KERLPGDVPFVQVDAHNIVPCWVASDKQEYGARTIRRKIHDRLSEFLTEFPPVIKHPYPSTAPAEPIDWNACR
ASLQVDRSVKEVTWATPGLAAGLAVLESFIGERLKSFGTDRNNPNRAALSNLSPWFHFGQVSGQRAILEVQKY
RSKYKESVDGFIEEAVIRRELADNFCYYNRNYDKVEGAYDWAKTTLKLHAQDKRSHLYELEPLEEGKTHDPLW
NAAQLQMVHEGKMHGFLRMYWAKKILEWTRSPEEALKFAIYLNDRYELDGRDPNGYVGCMWSICGIHDQGWAE
RAVFGKIRYMNYAGCKRKFDVGQFERRYDPCKLGQ
>Plectropomus_leop__XP_042341943.1
MAHTCIHWFRKGLRLHDNPALMAALRDCKELYPVFILDPQLHNKTLVGINRWRFLIGALKDLDCSLRKLNSRL
FVVRGKPEEVFPKLFDNWKVTKLTYEYDTEPYSLSRDKTVTMLAREHGVEVIYKISHTLYDIERIIEENNGKA
PLTYNRMQAIVKTLGPPKKPIPAPTMEDLKDVKTPCSENHEKKYGIPKLEELGHSDTAGLEEEPFPGGEQEAL
RRLDEHMERTAWVCSFEKPQTSPNSLSPSTTVLSPYVTFGCLSARTFWWRLTHVYQGKKHSDPPVSLHGQLLW
REFFYTASVGIPNFNKMEGNPVCTQVDWDTNPDYLAAWKEARTGFPFIDAIMTQLRQEGWIHHLARHAVACFL
TRGDLWISWEEGQKVFEELLLDGDWALNAGNWQWLSASTFFHQFFRVYSPVAFGKKTDKNGDYIKKYLPLLKK
FPAQYIYEPWKAPRSVQQAAGCMVGKDYPHPIVQHEVISKKNIQRMKLAYSKRNSDPAESPSKKQGVKRKAPS
VVDMLKKKNKTM
>Plectropomus_leop__XP_042351757.1
MVVNTIHWFRKGLRLHDNPALRDSIWDADTLRCVYILDPWFAGSSNVGINRWRFLLQCLEDLDASLRKLNSRL
FVIRGQPTDVFPRIFKEWKITRLSYEYDSEPFGKERDTAIQKLASEAGVEIMVRTAHTLYNLDKIIELNDGQS
PLTYKRFQALINRMDAVELPAETITLDVIRKCATPISEDHDDKFGVPSLEELGFDTEGLTTAVWPGGETEALM
RLERHLERKAWVANFERPRMNANSLLASPTGLSPYLRFGCLSCRLFYFKLTDLYRKVKKNSSPPLSLYGQLLW
REFFYTTATNNPCFDKMEGNPVCVQIPWERNPEALAKWAEGRTGFPWIDAIMTQLRQEGWIHHLARHAVACFL
TRGDLWVSWEEGMKVFEELLIDADWSVNAGSWMWLSCSSFFQQFFHCYCPVGFGRRTDPNGDYIRRYLPILRG
FPATYIYDPWNAPEEVQKAAKCIIGVHYPRPMVNHAEASRINIERMKQIYQQLSCYRGLGLLATVPANPNNGA
NGSNVGGVNTGTSQDPGTSSEGVSTYKGMSRTDRGVSTQKRRHEEASSKGSSKLWKQSK
>Plectropomus_leop__XP_042352682.1
MPPSAAAGTEDGKVLVRKILREVLVGREDPEGFFAMCVSVLGHQETRSQFMSLIQPLSSANSTLHSALTSIYK
EYFSQTEDDELELALALSLLEMKDHNSQESRPQQPGDRLNQRRFVQLTSVSTPQASSLRKENTNSAQSGPWVK
MSPPHTTKETELQKSTHVETPGTKNVCVSTPSGSFSKQDTVEKHDQMIDEGDLNQSDKPKRSKNRRQRRKGTG
QHVVGLPVSPSATPPVLLWFRRDLRLGDNPALIGSLEVGAPVIPVFIWSPEEEEGPGVTVAMGGACKYWLHQA
LSCFSSSLERIGSHLVYLKANGASLRTLKELVKETGARTVLTNALYEPWLKERDDVVVSALQKDGVECRVFHS
YCLRDPYSVSTEGVGLRGIGSVSHFMSCCRQNPGSASGVPLEPPVSLPTPAHWPQGVSLDTLDLARMPRRKDG
TTIDWAANIRKSWDFSEEGACARLEAFLNDGVYRYEKESGRADAPNTSCLSPYLHFGQISPRWLLWDAKGARC
RSPKFQRKLAWRDLAYWQLMLFPDLPWESLRPPYKALRWSSNRHHLKAWQRGRTGYPLVDAAMRQLWLTGWMN
NYMRHVVASFLIAYLYLPWQEGYRWFQDTLVDADVAIDAMMWQNGGMSGLDHWNFVMHPVDAALTCDPCGSYV
RKWCPELADLPDELIHKPWKCPASMLRRAGVVLGQTYPERIVTDLEERRSRSLQDVAHVRKEFGQYVDKRTGC
DLVPLPPRLLSETLGLSHRDGGVVTEGKQFLLPVITRMEFKHQLEDPDADAASNPYNAVLKGYVSRKRDETIA
FLNERDFTASVMYEGVQRKERLESDYRRMEGLPRPPAPRGRARRTPTAKDRFSVVPGGAVASLR
>Plectropomus_leop__XP_042354039.1
MLHSIWRSSSFSTVAPRRLLRFLAHVQQRPSSVFSCLTKSYTSQTIMSGKKRKSESAAAKEPSAKQQKLAPMK
KDEKEKAVGWLKDLLKQQRTEGREMKFNKKRLRFISETEKIKQGSEGVLYWMLRDHRVQDNWSLIHAQRLALK
ENLPLHVCFCLVVPKSELSTLRHYNFMLKGLEEVAKECKTLDIQFHLLHGSAAEVLPGFVSDRSLGAVVTDFY
PLREPVQWLEGVKKALPKDIPLIQVDAHNVVPCWVASPKLEYGARTIRGKITKLLPEFFTDFPPVEKHPHTAT
RTAKPVDWDKTLASLQVDQTVGVPEWAKPGTKGGMAMLESFIDVRLKLFDTKRNDPNAVALSQLSPWIRFGHL
SAQRVALQVQHSGKSAGHSVASFIEELVVRRELTDNFCFYNKKYDSIEGAYEWAQKTLKDHAKDKRPYLYTRE
QLEKAKTHDKLWNGAQYQMVTEGKMHGFLRMYWAKKILEWTSSPEEALSIALYLNDRYELDGQDPNGFVGCMW
SICGVHDQGWAERPXFGKIRYMNYKGCMRKFDVAGFERKYCPKNL
>Plectropomus_leop__XP_042366222.1
MSASRTIICLLRNDLRLHDNELFHWAQRNAEYIVPLYCFDPRHYVGTYNYNLPKTGPFRLRFLLESIRDLRNT
LLSKGSNLVVRRGKPEEVVADLIKQLGSVSTVAFHEEVTSEELDVEKRVKDVCAQMKVNVHTCWGSTLYHRDD
IPFNHISRLPDVYTQFRKAVETQSRVRPLFPTPEQLKPLPQGLEDGAIPTAEDLQQTEPVADPRSAFPCSGGE

SQALARLKHYFWDTNAVAAYKETRNGLIGVDYSTKFAPWLAMGCISPRYIYHQIQQYERERTANQSTYWVIFE
LLWRDYFKFVAVKYGNRLFQIQGLQDKSIPWKKDMRLFNAWKEGRTGVPFVDANMRELALTGFMSNRGRQNVA
SFLTKDLGLDWRMGAEWFESLLVDHDVSSNYGNWLYSAGVGNDPRENRKFNMIKQGLDYDGNGDYVRQWVPEL
QKIRGADVHTPWTLSTASLSHAQVSLGETYPTPIVMAPEWSRHVNKKTSGTGPSPRGKKGPSHTPKQHGNRGI
DFYFSRSKNL
>Plutella_xyl_XP_011557520.2_cry-1
MSAAPAVVQAGEPPVVQPQAPPSDPAPRTAPPRHTIHWFRKGLRLHDNPAFREGLQDAVTFRCVFIIDPWFAS
SSNVGINKWRFLLQCLEDLDASLKKINSRLFVVRGQPADALPKLFKEWGTTALTFEEDPEPYGRVRDHNITAK
CREVGITVISRVSHTLYKLDKIIERNGGKAPLTYHQFQALIASMPPPPPAELAVGPAGLHGATTPVSEDHDER
FGVPTLEELGFEVENLKPPVWVGGESEALARLERHLERKAWVASFGRPKMTPQSLLASQTGLSPYLRFGCLST
RLFYYQLTELYKRVKRVRPPLSLHGQILWREFFYCAATRNPNFDRMEGNPICVQIPWEKNQEALAKWANGQTG
YPWIDAIMVQLREEGWIHHLARHAVACFLTRGDLWISWEDGMKVFDELLLDADWSVNAGMWMWLSCSSFFQQF
FHCYCPVRFGRKTDPNGDFIRRYIPVLKNIPTRYIHEPWAAPDEVQQAARCVVGRDYPLPMVDHGKASSINIE
RIKQVYAQLAKYKPQSNLNPQTVQRPNSMQMSPSPTSIITSINQSNFLCSQTPEPATDNFSATDQTELNDKNN
QTIDINSTDKQDNYDLKNLTIMNNDLQQEFNNTNEMYLQTQQFINKIIRSDLYDQQQQPVKLNYNEEKGQFFL
PAYNGNDELCLHPPNFETPSYTDLQPCNQNGTEESTSNKEGQGGGAATEKKDSYSSIDKTNPVDQNITTK
>Plutella_xyl_XP_037961567.1_cry-1
MTTARNTVVHWFRKDLRLHDNLALRNAINEAENRKHSLRPIYIMDPDIKQKVGSNRLRFLLQSLHDLDNNLKN
INSRLYVVKGNPIEILPKLFDKWQVKYLTIQLDIEPEYVEQDEIIEKISEEKDIFVVKRVQNTVYDVHSVLKK
NNGNVPMTYQKFLSLVLDVQVKECIEITKKVSDDCKPPKDGQEKKSANCYDVPTAADIDFDESELGPCKYPGG
ETEGVKRLHSYMVRRDWVCKFEKPNSSPNSLEPSTTVLSPYISHGCLSAKLFYHKLKEAENGRKHSEPPVSLL
GQLMWREFYYVAGAGTKNFDKMVGNAVCTQIPWGKNDEHLKAWAEGRTGYPFVDAIMRQLKQEGWIHHLARHM
VACFLTRGDLWISWEEGAKVFEEYLLDYDWSLNAGNWMWLSASAFFYKYFRVYSPVAFGKKTDKTGLYIRKYV
PELKKYPTEYIYEPWKAPKSVQTTAGCIIGEHYPKRIVEHETIHKENMQKMNAAYKVNKEKKSLKRPRS
>Plutella_xyl_XP_037964882.1_cry-1
MLGGSVLWFRHGLRLHDNPALLSAIEDRGQTFFPLFIFDGETAGTKIVGYNRMRYLLESLDDLDSQFKKHGGR
LLMVKGQPSTIIRRLWEEFGIRKLCFEQDCEPVWRERDDSVRRECSQTGIKCVQHVSHTLWEPDQVIRANGGI
PPLTYQMFLHTVGTMGEPPRPVEDADFTGITFGTLPDCFYQEFTVYDKTPKPEDLGVFLENEDIRMIRWVGGE
SQALLQATQRLQVEYETFSRGSYLPTHGSPDLLGPPISLSPALRFGCLSVRRFYWSLQEVFRRVHRGRLPAAH
FISGQLIWREYFYTMSVNNPQYGQMEANPICLDIPWRQPRGDELERWKSGRTGFPFIDAAMRQLLTEGWLHHA
VRNTVASFLTRGTLWLSWEHGLHHFLKYLLDADWSVCAGNWMWVSSSAFEALLDSGECADPVRLGQRLDPSGE
YVRRYVPELSQMPRNYVYEPWKAPLEVQERANCVIGRDYPAPIVNHLAAAASNRAAMQELRLLLQKAPPHCCP
SSDDEIRQFMWLHGETNQPDEVHSGI
>Plutella_xyl_XP_037969083.1_LOW_QUALIT
MSVLNNLILLAKRIQFVRMASSAKKIKLSAPSASSGSETGTNVEEFMKKIQAKREDTAKSILEYKFNKKRVRI
ISEEQLVPEGCEGIVYWMSRDSRVQDNWAFLFAQKLALKNEVPLHVCFCLIAKYLDASVRQFHFLLKGLEKVA
ADCKKLNISFHLLEGSGADALXQWVVDHKIGAVVCDFNPLRVPLGWLDGVKKKIKKDVPLIQVDAHNIVPCWV
ASDKQEYSARTIRXKINSKLDEFLTEFPPVIKTSLPSKFEPEPIDWXEAIESREADKTVGPVEWAKPGYDEAX
KQLKSFLDKRLKIFATKRNDPTQDALSNLSPWFHFGQISSQRTALCVKEYKSKHTESXNAFLEESIVRCELAD
NFCFYNEHYDSIKGASNWAQKTLDDHRKDKRTHIYTLDQLKNAQTHDELWNSAQIQLVKEGKQHGFLRMYWAK
KILEWTPSPEDALKYSIYLNDHYSIDGRDSNGYVGMLACGPVCGIHDQGWASAQCSGKIRFMNYQGWKRKFDV
NAFVARYXGKVHKYVPPKDAK
>Pocillopora_dam_XP_027043718.1_cry-1-like
MAAKQQRVHHTIHWFRKGLRLHDNLSLKEACETSLTLRPVYFIDPEYVKRGNMGFNRWRFLIQSLNDLDNNLK
SLGSRLFVIQGKPELELPKLFKAWKITKLTFEFDHEPEGRQRDAKVHELATKAGIEVETKVCHSLYDLDKIIA
KNGGTAPLTYKKLCSVVASLGSPLKPIPTVDKSTFDGCSTPVGKGNEYQLPTLEELEIETPEESSTVLFPGGE
SEALRRLDEHMQKEDWVNKFEKPNTSPNSLQPSTTVLSPYVVFGCLSARLFYHRLSDVYSRAKKHSNPPVSLH
GQLLWREFFYTAAYATPNFNKMEGNPVCLQIPWNNNQEFIQAWSEARTGYPFIDAIMTQLRREGWIHHLARHA
VACFLTRGDLWISWEEGLKVFEKLLLDHDWSLNAGNWMWLSASAFFHAYFRVYSPIAFGKKTDPNGDYIKKYI
PILKKYPPKYIYEPWTAPVSIQKTAGCVIGKDYPRPIVDHKEVVKINIAQMKKARTERDGDTGMEEESSEEKK
SSPGKKRKTNKEAPPSKRSRKITEFLKT
>Pocillopora_dam_XP_027044972.1_cry_DASH-1
MNRLLVQRGYFYTFTDIQVSFFSAGTNFLHSLRRYQHREVTPTMEATRGKSVVIYLMRNDLRVHDNECLQWAH
QNSDFVIPLFCFDKSILSPTAVTWHYKFAKTAMHRAKFILESVSDLRESLRELGSDLVVRTEQTAKEAVSDII
SLCQEKNEGHVALVYQREITKEETDVEAELMKVCEAKKVIVKSFWGLTLYHLEDLPFASVKFLPDTYTEFRKT
VEAKCRVRPLIPAPDCLKSLPAFLKDDHVGRMPALKELAKDYSEETADSRSAFPFSGGETAALKRLNQYLWDT
DLVARYKETRNGLIGSDYSTKFSPWLAVGALSPGKVFDCIKRYETECTANQSTYWVIFELLWRDYFKFVSLKY
GNSIFYLSGIMRKTGLPWKRDMMAFEQWQYGQTGVPFVDANMRELLYTGWMSNRGRQNVASFLVKDLGLDWRM
GAEWFESLLLDHDVSSNYGNWNYSAGVGNDPRENRKFNMIKQALDYDADGDFVRLWVPELAGLKGAKVHTPWL
LSSSELKHAGVTLGENYPKPMVCPPEWSRHTSKVKDSRKTPRAQHRGIDFYFKSPEGAGSSKKRY

>Pocillopora_dam_XP_027056122.1_cry-1-like
MSYATVQRKDVHAGSVDERSSAINDKEKHAIHWIRKDLRLHDNPSLLEAVKGSDTLRIIYVLDTKVDQNIGIG
ANLWRFLLQSLEDVDDSLRQLNSRLFVVRGQPADVFPRLFREWNTSLLTFEEDSEPFGREKDAAIRLLAQESG
VQVAIRRSHTLYDPQLIIKNNGGTPPLTYKKFLAIISSLGPPGHPVPTLDVHLLGSCSTPISDDHEEKYGVPS
LEELGLDIRKIHAAVWHGGEKEALVRLNRHLERKAWIASFEKPKVTPNSLFPSPTGVSPYLRFGCLSPRLFYH
RLTELYRKVKSSDPPISLYGQLLWREFFFTVAANNPHFDRMTTNPMCLQIPWKKNPENLAKWEEGRTGFPWID
AIMIQLQQEGWIHHLARHAVGCFLTRGNLWISWEEGMKVFERWLLDAEWSLNAGNWMWLSCSAFFQQFFNCIC
PVGFGRKLDPNGDFVRKYLPVLRGFPAKYIHAPWTAPESVQKAARCIIGVDYPYPMVDHSKVSCANLEKLRNI
FKALLCYKDSTAVSSEKQDNLHGQQEKLKQQMFLLEDKENE
>Pocillopora_dam_XP_027060408.1_uncharacte
MTSNINSLHWFRKGLRLHDNPSLRQAIEGSSTFRAVFFLDVDSLDEINLSKNKWRFLLECLHDLDKSLRRLNS
RLFIVRGQPIDVFPKLIRQWNITRVTYEYDSEPFPRRRDDSIKRLLESEGVEVLTSVSHTLYDIDEVIKSNHG
NVPVTFKQFECLVSKLKSPSNCVPVVDEQLLKNCKTPVEDNDENNYGVPEYDALDFVEEEAEPGQFAGGETEA
LRRLAVLEKEVAEPKLGKPQQESNSPFPKSSKLSPYLRFGCLSVRQLFHRMNQVYREANGKPAPMSVHLPLLW
REFYFVVASKHPNFDKMKNNSLCLQFPWEEQACKLEKFQQGKTGFPWIDAMMRQMLLEGWIPYLARQAVGCFL
TRGALWICWEEGFKIFEKYMLDAEWSINAGSWMWLSCSAFFAKHPQWTCPVGLGKHLDPQGNYVRKYIPELKD
FPAEYIYEPWDAPLELQVACNCVIGTDYPQPIVNHQQARKSCVDRLRGVYQNLVTKVSCNVGLGHNAMHWFRK
DLRLHDNPSLCEALANCRTFHAVYILDPVSARAAKVSANRWKFLVDCLRDLDKSLRECGSRLHVIRGQPTYVL
PKLFQDWNISKLTFECEIEPFGQRRDVAVAVLGEEHGVEVISKPSHTLYDPERIIKSNEGEAPMLIKTFENVI
RQMGPPEKPVDAVNKEMFKGCVCPVSNDHSTKFGIPSLDELGYSGCEVTTGDLWVGGEQEAMKRLTELEEKVL
EGNLKKSVEGLDALRPSRTQLSPYLRFGCLSPRLFHLRLTKAYMKVKCQPPPLSLYRQLVWREFFFTLASRNP
QMDRAVDNPLSFNIPWEENQDAFDAWKKGQTGFPWIDAIMRQLHAEGWIHHVARQAVGCFLTRGCLWISWEEG
FKLFEQLQLDSEWSLNAGNWLWVSCSAFFHGQIPWYCPVNVGKKLDPSGNFIRRYVPELKTMPTKYIHEPWMA
PLAVQKASNCIIGQDYPDRLCDHIERRKVCLERLKDVCHQIQGTNSSSRDIADVVD
>Podargus_stri__NXX18684.1
TQRAFLYAQRLALKQELPLHVCFCLVPKFLDATIRHYAFMLRGLQEVAEECAELNIPFHLLLGYAKDVLPAFV
VEHGVGGLVTDFCPLRLPRQWVEDIRDRLPEDVPFAQVDAHNIVPCWVTSPKQEYSARTIRGKIHAQLPEFLT
EFPPIICHPYPPSCPAEPIAWEACYSSLQVDRTVREVEWATPGTAAGLAVLQSFIRERLKSFGSHRNDPNKAA
LSNLSPWFHFGQVSTQRAILEVQKHRDKYKESVDAFVEEAVVRRELAENFCYYNENYDSVQGAYDWAQTTLKL
HAKDKRPFLYNLQELEQGTTHDPLWNAAQLQMVREGKMHGFLRMYWAKKILEWTHSPEEALQFAIYLNDRYEL
DGRDPNGYVGKLQEGGMGADAPPPCLTGCLWSICGIHDQGSAERAIFGKIRYMNYAGCKRKFDVGQFERRYAP
H
>Podargus_stri__NXX19603.1
FVLKVDYDVCSNYGNWLYSAGIGNDPRDNRKFNMIKQGLDYDGNGDYVRLWLPELQGIKGADIHTPWALSSAA
LSQAGVTLGETYPQPVVTAPEWSRHINQRPQGRSPHPRGRRGPAHTPVQHKGRGIDFYFSRKTDI
>Podargus_stri__NXX21976.1
RFLLQSLEDLDNSLRKLNSRLFVVRGQPTDVFPRLFKEWGVTRLTFEYDSEPFGKERDAAIVKLAKEAGVEVV
IENSHTLYDLDRIIELNGHKPPLTYKRFQAIISRMELPKKPVSTVTSQQMETCKVDIQENHDDVYGVPSLEEL
GFPTDGLGPAVWQGGETEALARLDKHLERKAWVANYERPRMNANSLLASPTGLSPYLRFGCLSCRLFYYRLWE
LYKKVKRNSTPPLSLYGQLLWREFFYTAATNNPKFDRMEGNPICIQIPWDRNPEALAKWAEGKTGFPWIDAIM
TQLRQEGWIHHLARHAVACFLTRGDLWISWESGVRVFDELLLDADFSVNAGSWMWLSCSAFFQQFFHCYCPVG
FGRRTDPSGDYVKRYLPKLKGFPPRYIYEPWNAPESVQKAAKCIIGVDYPKPMVNHAETSRLNIERMKQIYQQ
LSRYRGLCLLASVPSCVEDLSGPVADSASGQGCSSSTAMRLSQADQASPKRKLEGAEELCTEELYKRAKVTGL
PAPEVPGKSS
>Podargus_stri__NXX23758.1
MPHRTIHLFRKGLRLHDHPTLLAALGSSEALYPVYILDREFTTSAMHIGALRWHFLLQSLEDLQKNLCQLGSC
LLVIQGEYESVLRDHIQKWNITQVTLDAEMEPFYREMEANIRRLGEELGFEVLSLVGHSLYDTKRILDLNGGT
PPLTYKRFLHILSLLGDPEVPVRNVTAEDFQRCRAPDLGLAERYRVPLPVDLKIPLESLSPWRGGETEGLQRL
QQHLTDQGWVASFTKPRTIPNSLLPSTTGLSPYFSMGCLSVRTFFYRLSNIYAQAKHHSLPPVSLQGQLLWRE
FFYTVASATPNFTRMAGNPICLQISWHEDAERLHKWKTAQTGFPWIDAIMTQLRQEGWIHHLARHAVACFLTR
GDLWISWEEGMKVFEELLLDADYSINAGNWMWLSASAFFHQYRRIFCPVRFGKRTDPQGQYIRKYLPILKNFP
SKYIYEPWRASEEEQKQAGCIIGQDYPFPMVNHKEASDHNLQLMKQVREEQQRTAQLTR
>Podiceps_cris__KFZ48403.1
RFLLQCLEDLDANLRKLNSRLFVIRGQPADVFPRLFKEWNIAKLSIEYDSEPFGKERDAAIKKLASEAGVEVI
VRISHTLYDLDKIIELNGGQPPLTYKRFQTLISRMEPLEMPVETITPEVMEKCTTPVSDDHDEKYGVPSLEEL
GFDTDGLPSAVWPGGETEALTRLERHLERKAWVANFERPRMNANSLLASPTGLSPYLRFGCLSCRLFYFKLTD
LYKKVKKNSSPPLSLYGQLLWREFFYTAATNNPRFDKMEGNPICVQIPWDKNPEALAKWAEGRTGFPWIDAIM
TQLRQEGWIHHLARHAVACFLTRGDLWISWEEGMKVFEELLLDADWSVNAGSWMWLSCSSFFQQFFHCYCPVG
FGRRTDPNGDYIRRYLPVLRGFPAKYIYDPWNAPESIQKAAKCIIGVNYPKPMVNHAEASRLNIERMKQIYQQ

LSRYRGLGLLATVPSNPNGNGNGGLMGYSPGESISGCSSTGGAQLGTGDGHTVVQPCALGDSHSGASGIQQQG
YCQASSILHYAHGDNQQSHLLQAGRTALGTGISAGKRPNPEEETQSVGPKVQRQSAN
>Podiceps_cris__KFZ51847.1
RFLLQSLEDLDNSLRKLNSRLFVVRGQPTDVFPRLFKEWGVTRLTFEYDSEPFGKERDAAIVKLAKEAGVEVV
IENSHTLYDLDRIIELNGHKPPLTYKRFQAIISRMELPKKPVSTVISQQMETCKVDIQENHDDVYGVPSLEEL
GFPTDGLAPAVWQGGETEALARLDKHLERKAWVANYERPRMNANSLLASPTGLSPYLRFGCLSCRLFYYRLWE
LYKKVKRNSTPPLSLYGQLLWREYTPATNNPKFDRMEGNPICIQIPWDRNPEALAKWAEGKTGFPWIDAIMTQ
LRQEGWIHHLARHAVACFLTRGDLWISWESGVRVFDELLLDADFSVNAGSWMWLSCSAFFQQFFHCYCPVGFG
RRTDPSGDYVKRYLPKLKGFPSRYIYEPWNAPESVQKAAKCIIGVDYPKPMVNHAETSRLNIERMKQIYQQLS
RYRGLCLLASVPSCVEDLSGPVTDAASGQGCSTSTAIRLSQADQASPKRKHEGAEELCPEELYKRAKVTGLPA
PEIPGKS
>Podiceps_cris__KFZ58887.1
IGALRWHFLLQCLEDLQKNLRQLGSCLLVLQGEYEPVLRDHIQKWNITQVTLDAETEPFYKEMEANVRRLGDE
LGFEVLSLVGHSLYDTKRILDLNGGTPPLTYKRFLHILSLLGDPEVPVRNLTAEDFQRCRSPDPALAERYRVP
LPVDLKIPPESLSPWRGGETAGLQRLEQHLTDQGWVASFTKPRTIPNSLLPSTTGLSPYFSMGCLSVRTFFYR
LSNIYAQAKNHSLPPVSLQGQLLWREFFYTVASATPNFTKMVGNPICLQISWYEDAERLHKWKMAQTGFPWID
AIMTQLRQEGWIHHLARHAVACFLTRGDLWISWEEGMKVFEELLLDADYSINAGNWMWLSASAFFHQYTRIFC
PVRFGKRTDPEGQYIRKYLPVLKNFPSKYIYEPWTASEEEQKQAGCIIGQDYPFPMVNHKEASDHNLQLMKQV
REE
>Podiceps_cris__KFZ69140.1
DNWAFLYAQRLALKQELPLHVCFCLVPKGWWWDGVCRGADHPVVLSPSPQECAELNIPFHLLLGYAKDVLPVF
VVEHGVGGLVTDFCPLRLPRQWVEDVRERLPEDVPFAQVDAHNIVPCWVASPKQEYSARTIRGKIHAQLPEFL
TEFPPVIRHPYPPSCPAEPIAWEACYSSLQVDRAVKEVEWATPGTAAGLAVLQSFITERLKSFGSHRNDPNKA
ALSNLSPWFHFGQVSTQRAILEVQKHRDKYKDSVDSFVEEAVVRRELAENFCYYNENYDSVQGAYDWAQTTLK
LHAKDKRPFLYELQDLEQGATHDPLWNAAQLQMVREGKMHGFLRMYWAKKILEWTRSPEEALRFAIYLNDRYE
LDGRDPNGYVGKRCLWSICGIHDQGWAERAVFGKIRYMNYAGCKRKFDVGQFERRYAP
>Podilymbus_podi__NXL47854.1
FTQGQPTSSYCFLSLHKVDYDVCSNYGNWLYSAGIGNDPRENRKFNMIKQGLDYDGNGDYVRLWVPELQGIKG
ADVHTPWALNSAALSQAGVTLGETYPQPVVTAPEWSRHISQRPQGRSPHPRGRRGPAHTPMQHKDRGIDFYFS
HKKDA
>Podilymbus_podi__NXL49016.1
MGVNAVHWFRKGLRLHDNPALRECIQGADTVRCVYILDPWFAGSSNVGINRWRFLLQCLEDLDANLRKLNSRL
FVIRGQPADVFPRLFKEWNIAKLSIEYDSEPFGKERDAAIKKLASEAGVEVIVRISHTLYDLDKIIELNGGQP
PLTYKRFQTLISRMEPLEMPVETITPEVMEKCTTPVSDDHDEKYGVPSLEELGFDTDGLPSAVWPGGETEALT
RLERHLERKAWVANFERPRMNANSLLASPTGLSPYLRFGCLSCRLFYFKLTDLYKKVKKNSSPPLSLYGQLLW
REFFYTAATNNPRFDKMEGNPICVQIPWDKNPEALAKWAEGRTGFPWIDAIMTQLRQEGWIHHLARHAVACFL
TRGDLWISWEEGMKVFEELLLDADWSVNAGSWMWLSCSSFFQQFFHCYCPVGFGRRTDPNGDYIRRYLPVLRG
FPAKYIYDPWNAPESIQKAAKCIIGVNYPKPMVNHAEASRLNIERMKQIYQQLSRYRGLGLLATVPSNPNGNG
NGGLMGYSPGESISGCSSTGVFFFFPASPVAQLGTGDGHTVVQPCALGDSHSGASGIQQQGYCQASSILHYAH
GDNQQSHLLQAGRTALGTCISAGKRPNPEEETQSVGPKVQRQSTN
>Podilymbus_podi__NXL52186.1
RFLLQSLEDLDNSLRKLNSRLFVVRGQPTDVFPRLFKEWGVTRLTFEYDSEPFGKERDAAIVKLAKEAGVEVV
IENSHTLYDLDRIIELNGHKPPLTYKRFQAIISRMELPKKPVSTVISQQMETCKVDIQENHDDVYGVPSLEEL
GFPTDGLAPAVWQGGETEALARLDKHLERKAWVANYERPRMNANSLLASPTGLSPYLRFGCLSCRLFYYRLWE
LYKKVKRNSTPPLSLYGQLLWREFFYTAATNNPKFDRMEGNPICIQIPWDRNPEALAKWAEGKTGFPWIDAIM
TQLRQEGWIHHLARHAVACFLTRGDLWISWESGVRVFDELLLDADFSVNAGSWMWLSCSAFFQQFFHCYCPVG
FGRRTDPSGDYVKRYLPKLKGFPSRYIYEPWNAPESVQKAAKCIIGVDYPKPMVNHAETSRLNIERMKQIYQQ
LSRYRGLCLLASVPSCVEDLSGPVTDSASGQGCSTSTAMRLSQADQASPKRKHEGAEELCPEELYKRAKVTGL
PAPEIPGKSL
>Podilymbus_podi__NXL53871.1
MSHRTIHLFRKGLRLHDNPTLLAALESSEVIYPVYILDRKFMASVMHIGALRWHFLLQSLEDLQKNLRQLGSC
LLVLQGEYESVLRDHTQKWNITRVTLDAETEPFYKEMEANVRRLGDELGFEVLSLVGHSLYDTKRILDLNGGT
PPLTYKRFLHILSLLGDPEVPVRNLTAEDFQRCRSPDPALAERYRVPLPVDLKIPPESLSPWRGGETAGLQRL
EQHLTDQGWVASFTKPRTIPNSLLPSTTGLSPYFSMGCLSVRTFFYRLSNIYAQAKNHSLPPVSLQGQLLWRE
FFYTVASATPNFTKMVGNPICLQISWYEDAERLHKWKTAQTGFPWIDAIMTQLRQEGWIHHLARHAVACFLTR
GDLWISWEEGMKVFEELLLDADYSINAGNWMWLSASAFFHQYTRIFCPVRFGKRTDPEGQYIRKYLPVLKNFP
SKYIYEPWTASEEEQKQAGCIIGQDYPFPMVNHKEASDHNLQLMKQVREEQYGTAQLTR
>Podilymbus_podi__NXL54180.1
MPHSSIHWFRKGLRLHDNPALLAAAAADCRCLHPLFILDPSSDRAGANGRRFLLDALRDLDGSLRDVGSRLFV
VRGCPEEVFPRLFRAWGTTRLTFEVDTEPLARRRDDAVAELAAQHGVEVIREVSHTLYDTERVLALNDGKAPL

TYKHLQSLLASLGPPEKPAPALTREHLQDGCARPARRGHREDGQCPAPITLILGLLGLSPQPKTRPQCVPHMQ
AWVCGFKKPETEPNSLSPSTTVLSPYLKFGCLSVRTFWWRLDEVYQGREHSRPPVSLHGQLLWREFFYTAGAG
IPNFDRMVGNPVCLQVDWDDNPQHLRAWREGQTGYPFIDAIMTQLRTEGWIHHLARHAVACFLTRGDLWVSWE
EGLKVFEELLLDADWSLNAGNWLWLSGSAFFHRYFRVYSPIAFGKKTDRDGAYIRKYLPILKDFPTAYIYEPW
KAPRAVQERAGCLVGTHYPRPIVEHGAASKRNLGRMKAAR
>Pollicipes_poll__XP_037072142.1
MAPPVVIHWFRKGLRLHDNPALLAAAAYAERTGAHLRPIFILDPHFVRSGRVGANRWRFLAGALADLAASLAA
RGSRLFVCRGAPEAVLARLIPDWDVRHLTFEADTEPYAVARDARVTALARQLSCAVTAVTSHTLFEPARVCAA
NGGRAPLTYQKMVSLAGQLGAPPAAEPAPASLPAPGKCPGEHAPPTLHELGVDAERVPAELYPGGETEGLARL
ERHLARAAWCCRFQKPQTAPNSLAPSTTVLSPYLKFGCVSARLFYHRLAAVYRAGGTHTQPPVSLHGQLLWRE
FFYTVGASTPNFDRMEGNPICRQIPWQKNEEHLKAWTEGRTGYPFIDAIMTQLRQEGWIHHLARHAVACFLTR
GDLFISWEEGMKVFDELLLDSDWSLNAANWMWLSASAFFHQFFRVYSPVAFGKKTDPNGDYIKKYLPKLKKYP
SQYIYEPWKAPLSVQRAAGCVIGTDYPAPIVDHDTVRPRNIEWMAAAYKKGKPAGPPAAAAPVQKASAGSPSE
QPAAARKRPASGGNKPAQKRSGGGQNKAKARPAGQ
>Pollicipes_poll__XP_037072188.1
MGQDDPMMAKPVGVHWFRHGLRLHDNPALTDLLATCSTVYLMFIFDGESGGTRLIGYNRFKFLLESLEDLDGQ
LATHGGRLVTARGDPVDVLNMFRVDYGMTHLSFEQDCEPVWLPRDDRVRAWCQQQGVTIIERISHTLWDPAEV
IDANGGEPPLTFAMFHQVAMSIGLPPPPVQAAPLDSRQLARVDTDRPHGHPVELFPRVPAVADLKPLSPPAGE
HQKRYVGGETHALYLLGERIAYEEAALSRGIIMPNQYCPDLLGPPRSLSAYLRHGCLSVRRMYHMLQKVFRAI
MDVNGHSLSICLQLIWREFFYTMSVNNPYYDQMEPNPICLNIRWRSDARQLKQWTLGQTGYPFIDAAMRQLVQ
EGWIHHVARNAASCFLTRGDLWISWVDGLQVFYEHLLDADWSVSAGNWMWVSSSAFEKFLDTSDIISPVAYGR
MFEPTGEYIRRYVPELRRMPTEYVFSPWEAPLEVQREAGCVVGEDYPCRIVDHQVASRHNCKKMKRIAQQLDS
CPPHCMPSGHAEVKLFLGVPGASLEDVLAGR
>Pollicipes_poll__XP_037075374.1
MSNDETPKHTIHWFRKGLRLHDNASLLEGVTGSSTFRCIYILDPWSAGSSSVGINKWRFLLECLEDLDKSLQR
LNSRLFVIRGQPADVLPSLFKKWGTTCLSFGEDPEPFGRVRDRNVRKMAEEHQVRVVSRTCHTLYDLNRVMAK
MGGKSPLTYKQFQNVLLSMPPPPPPAAAITLEMLDDSYTPVSDDHDDKYGVPTLDELGFDTDSAQPAVWHGGE
TEALVRLERHMERKAWIASFGRPKMTPQSLKASLTGVSPYLRFGALSPRLFYRDLAHLYRKIKNSDPPLSVHG
QLLWREFFYVAATNNPNFDKMVGNPICVQIPWDRKPKALSKWATGQTGFPWIDAIMTQLRREGWIHHLARHAV
ACFLTRGDLWISWEEGMQVFEELLLDADWSVNAGTWMWLSCSSFSQQFFHCYSPVRFGRKVDPDGDYIRNYIP
VLNSMPSRYIHEPWLAPESVQKAARCVIGENYPLPIVSHAEASRINVERMRQVYTHLLGYKGGRVQLTGPLLP
IPLGEEGEEQAQKLAPAVRATGASYKQDLLPSARQTARRVRAAEPREA
>Pollicipes_poll__XP_037083138.1
MSGKRIAIALLRNDLRYLDNEVLHMAHSSSDFVVPLYCLDPRHFQTTYHFGFPKTGAHRARFLLHSLEDLRAQ
LTKHGSDLVIREGRPESAVPDLLSSLSGHRLSLVLQQEATDEELRVERALADACRRLSVPVHTVWGATLYHRD
DLPFKVQSVPDVYTQFRQAVESRASVRAPLTMPDRLRPLPAGVAPGPLPAPAALGLPDGPPPAPDARSAFPWR
GGEAAALDRLRHYLWQTDAVATYKETRNGLVGADYSTKFSPWLALGCLSPRHIHSEVRRYEAERTANKSTYWV
LFELIWRDYFRFVALKFGNRIFFETGLLNKKVPWQNNKVAFDQWKDGATGVPFVDANMRELKLTGWMSNRGRQ
NVASFLTKDLGLDWRLGAEWFESLLLDHDVCSNYGNWNYSAGIGNDPRENRKFNMIKQATDYDPQGEYVRLWV
PELASVKDGRVHMPWALPPAARRQLEYPDPMVVAPEWQRHASKTAPGRGRGRGGPQPGRGQRRGIDFYFSSSA
SGDRRH
>Pollicipes_poll__XP_037090097.1
MIAPEAETMSEPPNKKQKKSDDCPIDKQIEDKRKQLREKGWKFNKKRCRILTAEEVPEKARSVVYWMSRDQRV
QDNWALLFAQKLALKFKLPLHVCFCLVPKFLDATIRHYGFMLKGLREVETECSRLGIGFHLLIGPASTSLVKF
VADNGAGAVVADFAPLRVPRRWLDDVIEALPNDVSVSQVDAHNVVPCWVASDKQEYGARTIRRKIMDRLPEFL
TEFPPVLRHPHAADGKRQPVDWAAAERSLEVDRTVAEVEWARPGAAAALRQLDEFXRSRLRLFADKRNDPTVE
ALSNLSPWLHFGQLSAQRAVLEVRRHSSRHSKAVQAFVEEAVVRRELADNFCYYQPHYDSVEGAYGWAGDTLK
AHRKDKREYLYSREQLAAAQTHDDLWNAAQIQMVQEGKMHGFLRMYWAKKVLEWTPSPEEALATAIYLNDRFS
LDGRDPNGYVGCMWSICGIHDQGWAERPVFGKIRYMNYQGCKRKFKVDQFVSRYGGKVHKYVAPK
>Polyodon_spat__MBN3281058.1
MKHSSIHWFRKGLRLHDNPALLAALRDCAEIRPVFILDPWFPRNTRVGINRWRFLLDALKDLDGSLRKVNSRL
FVVRGKPTETFPRLFRQWKVTRLTFEMDTEPYSRQRDAEVVKLAEEHGVEVIQKVSNTLYDTERVIVENNGKP
PLTYKRLQTILSFLGLPKKPVPVPTLDDMKGEWDYCLLLMVLIDCNVRTPCSDSHEQEFGVPTLEELGQNPLK
AGPHLYPGGEGEALRRLDQHMQRVAWVCNFKKPETEPNTLTPSTTVLSPYVKFGCLSARTFWWRLTDVYRGAW
VCNFKKPETEPNTLTPSTTVLSPYVKFGCLSARTFWWRLTDVYRGKKHSEPPVSLHGQLLWREFFYTAGAGIP
NFHCMEGNPVCVQVDWDTNEEHLAAWREGRTGYPFIDAIMTQLRSEGWIHHLARHAVACFLTRGDLWISWEEG
MKVFEDLLLDSDWSLNAGNWQWLSASAFFHQFFRVYSPVAFGKKTDKKGDYIRHVKQNVWEWNEHFVFNLAGL
VCRQNERKYIPLLRKFPAEYIYEPWKAPLSIQERAGCIVGQDYPRPIVEHDVVSKRNVQRMKAAYARRAAPNS
ETGAVKRGEPESFLAVNCFKITP
>Polyodon_spat__XP_041110950.1

MVVNTVHWFRKGLRLHDNPSLRESIQGADSIRCVYILDPWFAGSSNVGINRWRFLLQCLEDLDASLRKLNSRL
FVIRGQPADVFPRLFKEWNINRLSFEYDSEPFGKERDAAIKKLASEAGVEVMVQRSHTLYDLDRIIELNGGQP
PLTYKRFQTLISRMETLETPVESITVEVMGKCMTPVSDDHDDKFGVPSLEELGFDTEGLPSAVWPGGETEALT
RLERHLERKAWVANFERPRMNANSLLASPTGLSPYLRFGCLSCHLFYFKLTDLYKKVKKNSSPPLSLYGQLLW
REFFYTAATSNPRFDKMEGNPICVQIPWDKNPEALAKWAEGRTGFPWIDAIMTQLRQEGWIHHLARHAVACFL
TRGDLWLSWEEGMKVFEELLLDADWSVNAGSWMWLSCSSFFQQFFHCYCPVGFGRRTDPNGDYIRRYLPVLRG
FPAKYIYDPWNAPESVQKIAKCVIGVHYPKPMVNHAEASRLNIERMKQIYQQLSCYRGLGLLASVPSNPTGNG
NGGMMGFSPGEGMHGGNTQASNPVGVEKGNAGMQQQGFQMSSSGSSMLAYPQGDSLSGNGLMLQGRRAHSSVL
TTVKRHSPEETAQGIGPKVQRQSSN
>Polyodon_spat__XP_041119980.1
MEEKPGKLRKTKNDNGNTGAGRVLDTVKIDQKVVEGGRQAGSSSGREAKAARKQEPGKRESAGETTRSGSSGD
ELSNTGTRKKRRLGARDVVASPPGGVGGLASSIKETRLQARMSVQGFEFNKKRVRLISEAVEMKDGSESIVYW
MSRDQRVQDNWALLYSQHLALSHELPLHVCFCLVPRFLDATIRHFGFMLRGLEEVAKVDAHNIVPCWVASEKQ
EYGARTIRKKIHDKLSEYLTEFPPVLRHPHGAGCEAKAVDWEQARSSLEVDCSVKEVDWARPGTEAGLAMLES
FIMERLKLFGSDRNNPNRPALSNLSPWFHFGQVSVQRAVLEVRKCSSKYLQSVNSFIEEAVVRRELADNFCYY
NKNYDCVEGASDWARKTLQDHAQDERSHLYSRAQLEAGKTHDPLWNAAQLQMTLEGKMHGFLRMYWAKKILEW
TSSPEEALETALYLNDRYELDGRDPNGYVGCMWSICGIHDQGWGERPVFGKIRFMNYAGCKRKFDVGQFERKY
HPEKFTV
>Polypterus_senegalus_XP_039605072.1_cryptochro
MVVNSIHWFRKGLRLHDNPALLAALNGADTVRCVYILDPWFAGSTNVGVNRWRFLLESLEDLDTSLRKLGSRL
FVIRGQPADVFPRLFKEWNTTRLTFEYDSEPFGKERDAAIIKFAEELGVETVVKNSHTLYNLDRIIELNNNSP
PLTYKRFQGIISRLEPPKKPVASLTKEQMQKCPTDIPETPYECFRVPSLDELGFKTATLSPSIWKGGETEALL
RLEKHLERKAWVASFERPRINVNSLIASPTGLSPYLRFGCLSCRTFYYTLLELYKKVKGNRSPPLSLFGQLLW
REFFYTAATNNPKFDRMEGNPICVQIPWDKNPKALAKWAEGQTGYPWIDAIMIQLKQEGWIHHLARHAVACFL
TRGDLWISWECGMKIFDELLLDADFSVNAGSWMWLSCSAFFQQFFHCYCPVGFGRRTDPTGNFIRHYIPKLKD
FPNRYIYEPWNAPDTVQKAAHCIIGIDYPKPMISHAEASRLNIERMKQIYQHLSHYKGLSLLASVPTVSEEID
LEYCNKHSSMPGKVSPLGKRDRPPDLEIDEKCTDPKVSCSHLMEGNFEQ
>Polypterus_senegalus_XP_039617556.1_cryptochro
MVVNTVHWFRKGLRLHDNPSLWESLKGADSVRCVYILDPWFAGSSNVGISRWRFLLQCLEDLDANLRKLNSRL
FVIRGQPADVFPRLFKEWNISRLSFEYDSEPFGKERDAAIKKLASEAGVEVIVRISHTLYDLDKIIELNGGQP
PLTYKRFQTLISRMEPLETPVESITADVMGKCTTPVSDDHDEKFGVPSLEELGFDTEGLPSAVWPGGETEALT
RLERHLERKAWVANFERPRMNANSLLASPTGLSPYLRFGCLSCRLFYFKLTDLYKKVKKNSSPPLSLYGQLLW
REFFYTAATNNPRFDKMEGNPICVQIPWDKNPEALAKWAEGRTGFPWIDAIMTQLRQEGWIHHLARHAVACFL
TRGDLWISWEEGMKVFEELLLDADWSVNAGSWMWLSCSSFFQQFFHCYCPVGFGRRTDPNGDYIRRYLPVLRG
FPAKYIYDPWNAPESIQKAAKCVIGLHYPKPMVNHAEASRLNIERMKQIYQQLSCYRGLGLLASVPSNPNGNS
NGGVTGLSPNEIIQGTHSSVGTGMGTGENQVVGGLTFQHGDSHPVLHHEGYGSGNSILLYSQRGSQQTESVIL
QPVGRGTAASTLTVKRPNPEETAQGIGPKVQRQSSN
>Polypterus_sene_XP_039620008.1
MKHNSIHWFRKGLRLHDNPALLAALKDCAEIRPIFILDPWFPKNMKVGINRWRFLIESLKDLDCSLRKMNSRL
FLVRGKPVEVFPRLFKEWRVTRLTFEIDTEPYAQLRDSQVVEIAKEHGVEVIQKVSNTLYDTERIILENNGKP
PLTYVKLQGLLSRLGPPKKPAPSLKGEDLKGIHTPYTDYHDTEFGIPTLEELGLDPAAAGPHLYPGGETEAIR
RLDEYMQKSVWVCNFKKPETEPNSLTPSTTVLSPYVKFGCLSVRTFWWKIAEVYHGKKHSSPPVSLHGQLLWR
EFFYTAGVGIPNFDHMEGNSVCLQVDWDSNEEYLAAWREGRTGYPFIDAIMTQLRTEGWIHHLARHAVACFLT
RGDLWISWEEGQKVFEEFLLDADWSLNAGNWQWLSASAFFYQFYRIYSPVAFGKKTDKNGDYIRKYIPILRKF
PADYIYEPWKAPKSIQERAGCIIGKDYPKPIVDHDTVSKKNIQKMKAAYSRRSATSSKSGENCLKGPKKRALS
MANLFTKKTSQKTS
>Polypterus_sen_XP_039609456.1
MCAARAVICLLRNDLRFNDNEVLYWAQANAEYVVPLYCFDPRHYLGTHTFNFPKTGPFRLKFLLESVKDLRET
MKKKGSNLVVRKGKPEEVIQELIQKLKFVTAVTFQDEVTMEEKVVESGVKQVCSQLNIKVQTFWGSSLFHRDD
LPFHHISQLPDVFTYFRKAVESQGIVRPTFAMPSQMKPLPEGVEEGDIPSHETFGQTCPLLDPRSAFPCEGGE
TKALERLMYYFWDKNLIASYKETRNGLIGVDYSTKFAPWLALGCISPRYIYEQVKKYEKERTANQSTYWVIFE
LLWRDYFRFVALKYGRRIFFPKGLQDKAVAWKKDCKLFDTWKEGKTGVPFVDANMRELALTGFMSNRGRQNVA
SFLTKDLNLDWRMGAEWFEYLLIDYDVCSNYGNWLYSAGIGNDPRENRKFNMIKQGLDYDGDGEYVRLWVPEL
KEIKGGDIHTPWALSSASLSHFNVHLNETYPSPIIVQPEWSRHANQKPANSSGHRRGRGGSHHLPKQHKDRGI
DFYFSRNKHSE
>Polypterus_sen_XP_039620627.1
MQKKSSTKDSVKQLKAKKSKLLEDDHHDDYACKRQKLPEVTGGGKRLSDAICISRRETASSVAEFKFNKKRVR
ILSKTEDIVDGSDGILYWMSRDQRVQDNWALLFAQRLALKQKLPLHVCFCLVPRFLEATIRHFGFMLHGLQEV
AKECTELDISFHLLIGYAKDVLPEFVKLNHIGAVVTDFSPLRVPLQWLQDVKDRLPESIPFAQVDAHNIVPCW
VVSDKQEYAARTIRKKIHDKLPEFLTEFPPVVLHPHCTKIKTKEVDWEKARASLEVDHTVKEVDWAKPGTQGG

MAVLASFISERLKLYSSDRNDPNKEALSNLSPWFHFGQVAVQRAVLEVQKFRSKYRESVESFIEESVVRRELS
DNFCFYNPRYDSLDGAFEWARKTLEDHAKDKRTHLYSRAELEEGRTHDPLWNAAQLQMVHEGKMHGFLRMYWA
KKILEWTHSPAEALEISIYLNDRYELDGRDPNGYVGCMWSICGTHDQGWSERAVFGKIRYMNYAGCKRKFDVN
QFECTYNYKKFTP
>Pomacea_canalic_XP_025115306.1_cryptochro
MMPELFKDWKITTLTFEIDTEPYAMQRDDAIEKLAKSCNVNVIKRVSHTLYDTEKTISKNGGSPPLTYQRLQT
VLKEMGAPPKPSNTLTSSHLSVHVCLDCTTPWKEEFDEKYGVPTLAELGKNEEDCGPLLFPGGEMEALSRMER
YLKKTNWICKFEKPQTEPNSLEPSTTVLSPYIKFGCLSVRTFYYRLLDIYNLNKVYTKPPVSLHGQLLWREFF
YTAGVGTANFNRMEGNPVCTQVPWDSNPEYLAAWSEARTGYPFIDAIMTQLRTEGWIHHLARHAVACFLTRGD
LWLSWEEGQKVFEELLLDADWYLNAGNWLWLSASAFFHQYFRVYSPVAFGKKTDKNGDYIKKYVPKLRKFPAQ
YIYEPWKAPKSVQAAAGCIIGQDYPKPVVDHDTISKVNIKRMAAAYAKRKEGQDFSGKSASPQKRRKESTDSS
SKKKPKKDD
>Pomacea_can_XP_025078907.1_photolyase
MRECLQVRLGWQIFCIFDTISPNFSSQHRRVSHLLLRKCKTKFVLGRAKPEISLQIFLQKPRSHNYKYPYFVN
IKLCSSSKMDKEGSKKTTRIKNQKRKSELDSSESTKKSRSESLPGSKPLDDVDFVQQIAQCRTAACESIEAFK
FNKKRVRVLSEAKDCPDEGKGVLYWMSRDQRVQDNWAFLYAQRLALKLKLPLYVCFCLVPNFREATIRQFGFM
LKGLAEVEKECRNLDISFYLLMGKAKDVLPEFIKNHHIGGVVTDFSPLRIPSAWVTELSALLPKDVPFCQVDA
HNIVPCWVASPKLEYSARTIRSKIHKVLNEYLTEFPPLIKHPHKSPTMPKSVNWEKAAEFLEVDRTVKEVDWA
QPGTTGAYHTLATFCNKRLKFYESDRNDPNKEALSDLSPWFHFGQISPQRCVLAVRGFRSRAKESVDAFIEES
VIRRELTDNFCFYNENYDSIKGAAKWAQDTLEAHRNDKRAYVYNRQQLEQAKTHDNLWNAAQIQLLKEGKMHG
FLRMYWAKKILEWTPSPEEALKEAIYLNDRYSIDGRDPNGYVGCMWSICGIHDQGWKERPIFGKIRYMNYEGC
KRKFDVASFVKKYSKL
>Pomacea_can_XP_025095473.1_cry-1-like
MTSLSPKKVVSIHWFRHGLRLHDNPALLESLKNADEFYAVFIFDGSVAGTATAAYPRMRFLLECLSDLDAGLR
KLGTRLYIIHGQPEEVFPRLFEEWGVSRVTFEQDPEPVWQDRDNKVKTLCKQWKVECIEKVSHTLWDPQSIIQ
ANGGSPPLTYAMFCQVTDIVGLPPRPCPQPDFKGVNLPVVDNKKDVMGIPTCEELGVYPESERQKNPLGGYVG
GEQRALELLSLRIEKERKAFEKGQCLPNQLNPDLTGLPLSLSPHLRFGSLSVRRFYWAIHDAFKEVSQCEEVP
TSITGQLIWREYFYCMSVNNPMYNCMEGNPICLDIDWYQNQEQFEKWSKGQTGYPWIDACMRQLKEEGWIHHV
CRHAVACFLTRGDLWIDWQMGLKVFDKYLVDADWSVCAGNWMWVSSSAFEKVLQCPKCICPVRYGRRMDPKGD
YVRRYVPELKNMPLLYLFEPWKAPAEIQEKAGCIIGCDYPPPMVDHKQASKMCMSKMEKIKNKCRGLPHIAPS
SAAEVRLFMWMDNKSLEESLAHCQKHVTEPETFSKATLPLGQ
>Pomacea_can_XP_025096987.1_cry-1-like
MKGKVTSPKKTSEGTMPKKNSIHWFRKGLRLHDNPALLEALKDSSSYRCVYILDPWFAGSSQVGINKWRFLLE
SLEDLDSSLRKLNSRLFVIRGQPADVFSRIFKEWNITQLTFEEDPEPYGKERDAAICALAAEIGVQVVTKPSH
TLYDLRHILEANNNQPPLTYRRFQSILSGLLPPPVPLETVTATMLKHTHTPVADDHDDKYGVPSLEELGFDTD
NLGPATFRGGESEALARIYRHLERKAWVASFERPKMSPQSLFPSQTGLSPYLRFGCLSPRLFYWKLTELYKKV
KKGQEPPLALHGQLLWREFFYTAATNNPNFDKMSGNSICVQIPWDHNPEALAKWAEGQTGFPWIDAIMVQLKK
EGWIHHLARHAVACFLTRGDLWISWEEGMKVFDELLLDADWSVNAGMWMWLSCSAFFQQFFHCYCPVNFGKRA
DPTGDFVRHYLPALKGFPTQYIYEPWTAPEAVQKAARCIIGKDYPLPMVDHTHVSRVNMERMRQVYKHLLVSS
SVSGYKRKLDPPKIPKVDMFLLKANMFTSTSSDDDHEQTSGMRLENKI
>Pomacea_can_XP_025105331.1_cry_DASH-l
MKQLSIALKNRWTRTVYTAFSSRVNAPGCFFSKHEKGGIMKTVIYLFRNDLRLHDNESLYWACQNADTVLPVY
CFDPRHFGKTWHFGFAKTGPHRLKFLVESVVDLRGNLQKHGSDLMVRQGKPEDVIPDIISQLGKDNVEALVYQ
KEVTQEELDVEEALKLKSGVKVHTVWGHTMVHIDDLPFKPQTLPNAYTQFRKKVEESNRMRKELPIPDKLKPL
PAKFDVGTIPTFDDFKIPMSVPVTHTVFPFPGGETSALARLQSYFWETDNVAKYKDTRNGMIGSDYSTKFSPW
LAHGCISPRRIYWEIKRYEQQRTANESTYWVLFELLWRDYFRFVALKYGNKIFKVRGIQDKDIPWKKDEKLFE
AWREGRTGVPYIDANMRELQATGFMSNRGRQNAASFLIKDLQIDWRIGAEWFESILIDHDVCSNYGNWLYIAG
IGNDPRENRKFNVIKQGLDYDANGDYVRLWVPELSKVKRGSVHVVWTLSPAALSSAQVSIGESYPAPLVMAPE
WRHHYGRPASDSVGKRGRGRGPEPSPKQRGINFYFHNSSQQ
>Porphyra_umb_BU14_0223s_OSX75752.1
MQGHDAAFVPAGSSAFGVKVTSSATGRNNGVKAVSTASTPASFSPLTASAHSVAIGATTGVAPTPMPVMLPSL
CDFGPFSESPAGRKGGKVLVWFRSDLRVHDNPALTHALEEAGTVVPVYCFDPRQFGKTSFGFEKTGRYRAKFL
IESVTGLRDSLRKLGSDLIVRSGRPEDTLPELCRRLGVSSVFYHQEVTYEEQEVEQALAESLKAAGVDFKPFW
ANTLYHSEDLPFAVQDMPDVYTAYRESVESGGEIRSPLPSPTEMPTVPSAVKAGPIPSLKELGLAEPVGEMAA
VGGVGTFTGGEAEAMRRLSSYIASVGEKAAAAGLNSRASTSSAAHMGADFSCKISPWLALGCVSPRQIYQDLR
SSAAAGAAVAKTTTFFELVWRDFFRFITLKYGSVRLAAKSGKKATTHVRALATAGR
>Porphyra_umbili_OSX68520.1
PAAARAAPFTPTDPTVAVLWLRADLRLADNALLRAGADASALLPVYVFDPAAWGAPSAAHTLYGTVKGGAHRA
AFVTAAVAEVRAGLAAVGSGLAVRVGDPAAVLADVVARLLAAGTPVRVVASKEFCAEEVAAERSVAAALAGLT
TPDGVPPVALQFVWEGTLTHLDDLGFNPLTDTPPVFTQYRKRMEGGPGGRDGLAVRPPAPAPTRLPPLPGCVD

ATLLGAVPSLGDLGLDAAAAAVDGRAALDVRGGEAAASARLQTWMVDGDHLRHYKETRNDSGTDAGSSKLAAW
LATGCVSPRTVYAAIRRYEAATGVRDDNTYWLVFELLTRDFFRWTAAAVGPTLFAWRGHGSDRSRGDFAARRF
APTDTARFDAWAAGATGVPFVDAGMRELAATGYLSNRLRQNVASFLINELGCPDWRAGAAHFEATLVDYEPAA
NWGNWAYIAGVGADPRGGRAFNTVKQGGVYDPAGAYTKLWVPELAALPPAQLWEVGKVDPATLAGYGVTLG
>Porphyra_umbili_OSX72831.1
MLYVLEPAATTAPHTGAVRLRFLLESLTALDGELRARGSSLHVARVADGADVAAEVAAAAARCGATTLVYEDD
DSPAGRQRDAAVGAAAAAAGLAVVTRSGHTLYPPALLLDAAGGVAPRTMAAFTSLVDRVGPPRPPLPTVGDDL
PPPPPPPPPLGDGEAVADGAVPSLGALGWDAAAAAGLRVRGGEAAALQRMEAFLGRRGGAVVRAFEKPKTSMV
AWRTPDTTVLSPYLAAGVLSPRTFYARLREVEAAGNGRHTRPPVSLVGQLLWREHFHLLAGSVPHFGRMAGNP
LCRQIPWDDPSTDAAAAARHAAWATARTGYPAVDACMAQLRKEGWVHHLGRHLLACFATRGDLWLSWEAGAAT
FEELLLDWDPALNAGNWMWLSASAFFNSYFRVYGPVTWMRKVDPEGKFIRHYLPALARLPTAYIYEPWKAPAG
VQAAAGVRIGDGGDYPAPIVDHAVVSKVNIDRMAKAFRSGAAAEKGGNKAPAVKVGGVGGWGGGANAKPAAAT
GAPSRGGRSAKAKADAAPVKTEESGGAGGLRTRSGRVSKKR
>Porphyridium_pur_KAA8493818.1_photolyase
MERSVVEKERISKLNAAPVRERGKYVLYWMQASVRALRNPALEYAVERANDADVPVLVVFGLFEKYPEASERH
FAFLLECISDAGKQLEERGIEFVLVHQVPTTVVKALAADAVSVVTDRGYLRLLREWRSQVATEIDCRMVQIEG
DVVVPVQLVSDKEETAARTIRPKIHKYMPKFLHPLERVTPKNTTGKAGMLASTLATLKEFELLDWRDPLAVLA
KLDVDRSVPRVTWITGGATHAEAMLADFLKHKLKDFAANRNEPAQDFGSNMSPYLHYGGISVVHLVMEALKHK
TSASKEGVDSFIEELVVRRELSMNLVFYNPHYDAYACIPNYARITLDEHRHDEREPTYTLQELEAGVTYDECW
NACQREMVVRGKMHGYMRMYWAKKIIEWTSSPEEAYERCLLLNNRWELDGRDPNGYAGVAWSFGKHDQGWAER
AIFGKVRYMNKQGLHRKFKMELYVNKIDKLVREVGLPPHLDEVSKQSTAHARKQQKTLSDVQPQKKKPKVK
>Porphyridium_pur_KAA8493823.1
MQPFGGRRDVARKTCGVVYAQTSVATMRMCDGAAIRPAFVSIPVTARKARASQFGAPWYPPRWQIQLAVACAY
RRSSSHIAMAASAAQTSKAAVAGKCVIAWFRADLRLRDNPMLMDAIRAGQGCVLPVYCFDPRCFGLTDEAKFP
KCAAPRAQFLRECVEDLRNNLRALGSDLIVRHGHPEVVLTQLAEQYQCSHVFGSKEVAWEETRVEKKLRANMS
KLEKPPKLSLTWGAVTMYHLDDLPMDIDQLPDVFSQFRRSVESRPAVEVRAPLLAPAKGALRPLPEGLEIGAL
PSMDELLEATIPEASQRQFQQDSRAVLSFRGGETAALARVQYYLWESDCVAKYKETRNGMVGGDYSSKFSAWL
AHGCVSARDIYHEIKSYERTRVANDSTYWLIFELIWRDYFRLLALRCGNDIFFLGGPRRRDKIQWKQDRKAFE
AWRTGNTGYPLVDANMRELNQTGFMSNRGRQNVASFLTKDLRIDWRWGAEWFESMLLDHDVHSNYGNWTYAAG
VGMDPREDRHFNVVKQAKTYDETGEYAKLWLPELADVPAEFIYEPYKIPASERSRFNGLDQYPRPVVNTFGKG
SFQAGGQGGRQPDIQSGKKHRGPSKPRANARREPKY
>Porphyridium_pur_KAA8496724.1
MVASILLILDGFLPLIFFRPCRLDSYSAEVTDCSDAPASSGSVMNGLWNKLQLRHPPLPSSCCLDALSLCTFD
QSPIQSDLLFEPIQSPPFVPAAVFTLHTSPRFCVRCRLMRRSSLHGMAILGWVAQTGLRSPGSLCPPKCCDRT
RRYRRTAFAPTRFARALQTRVSLSPSREAAVPTDGVATSVAAPKKVCVVWFRRDLRLDDNPALYAAVAEHKYD
LVVPLFIVGDDQDELPDVFAATSRSCRDPFAHTAHSVYLQSSLQSFSRRLKELGGAGLHVVQRGDQTVAAVLK
AVCDSAKSTAVFFNARYEHGCAQQDERTIAALSRQGIIVHRCMGDLLFDPARIATAQQRQTRRTTHQQDIEES
DKNRKFAHWGTLTWFKRLAESSTPSSHHAALVPPIHSFSALEIHTLKEQSERILHTPLRRWGTKVMQYWEPGE
QTAQKALREFIDNGGLSAYERSKSRADMTGAVSKLSMRLRCGELSPRTVQNAFEKANVRHRAPILSRRLYWRD
LAYFQLALFSDPLEPIRPQYREQTWPGTKEQADAWIRGQTGYPLVDAAMRELWETGWMSQNVRMIAASFLVHI
LGVHWHVGLRHFHNALVDADVAINTMMWRNAAGVGVDSWNFSLDPVSDSRFVDPTGAYVRRWIPELAHLPNEH
LHAPWLASSREGLNYPERIVTDLKSARERMMDFFRQAKIRHHATSSVSCNERGYDLVRVPGTKSETVLFTLPF
LRKAVPKAQGMTEATAQSVSSKSRKHGPKKPSKTASSRSSEKRALTRQLRDLS
>Porphyridium_pur_KAA8497133.1_(6-4)DNA_p_
MTQPVLFWFRKSLRVHDNLALKRAVEYAAQHSATLVPVFVIDPHFVNPDKVGVVRMNFLLESLQDLSSTLQQH
RAQQLLVLRGNPEQVFPDLLFARKGSESGISALFFENDTEPYAKQRDTRVGELARKADVHVESCYGHTLYDPD
WLLKRARGGRPPETMSAFTALMESVGPPSKPVSAEDVMKSLANTPQDDELLRKQLMSEAQPLSIPNLAELGYP
DASPSRLFKGGESAALEKLREFCARKKGECVRKFAKPETNPAAFEPPETTTLSPYMKFGCLSPREFYHRVESI
RNSKSTQPPVSLIGQLYWREHFTLLGYVTPNFDRMEGNPLCRRIDWDTTPEYVSAWESGKTGYPWIDACMRQL
VSEGWLHHLARHSVACFLTRGDLWQSWEVGKAVFEKHLIDADWSLNAANWMWLSCSAFFHQYFRVYSPSAFPK
KYSHVAAFIRKYVPELRAMPDAYLLEPWKAPEKVQREAGCLIGKHYPSRVVIHEDISKENIQRMKRAFELNKT
ERPSSTGTKRSREE
>Portunus_tritub_XP_045110316.1_LOW_QUALIT
MPMGKVAVHWFRHGLRFHDNPALLEALQDAKMFYAIFIFDGESAGTKVTGYNRQRYLTESLLDLDQQLRDVGS
QLFVCEGNPVDIFSTLHKEIGLTHLSFEQDCEAIWNKRDNAVRKLCSELGVTVIERISHTLWDPFEVIEANGD
QPPTTYEMFVQVIKVLGDPPKPSPNPDWENVLFGNISDEVAQKIKLFPQVPSVEEQGYTRIRECPVYIGGEKV
ALAHLQERLIVEENAFRDGYILPNQVNPDLLGPPMSMSAALRFGCLSVRKFYWDMQETYQKINRGEPPASHSL
TAQLIWREFFYCMSANNPKYSEMEGNPICIQIPWYKKPGELAAWEEGRTGYPFIDACMRQLRKEGWIHHVCRT
VVSCFLTRGDLWISWVDGLKVFYKYLIDADWSVAAGNWMWISSSAFERQLDCSVCVCPVEYGRRIEPTGDYIR

QYVPELANFPQEFIFEPWKASLAIQKEHNCIIGQDYPERIEXHTEASKSNSKIMKSIRKRLKKPPRHCSPSDS
EEIKQYLRLPQSCFHNVF
>Portunus_tritub_XP_045118419.1_cryptochro
MTGEANKPKPKHHSVHWFRKGLRLHDNPALRAGLKNAVTFRCIFILDPWFAGSSNVGINKWRFLLQSLEDLDT
SLRKLNSRLFVIRGQPAHVLPELFREWGTTCLTFEKDPEPFGKVRDANIVAIAREMGINVIVKTSHTLYKPEK
IIDKNGGKPPLTYKSFQNILMSMDLPPLPLSTITLDDLENKYTPLNDDHDEKYGVPSLEELGFDTESLQQTGW
KGGENEALTRLEHHLERKAWVASFGRPKMTPQSLYPSRTGLSPYLRFGCLSCRRFFKELNDLYKKIRKSPPPL
SLHGQLLWREFYYTAATNNPKFDHMEGNPICVQIPWDKNPEALAKWANGQTGYPWVDAIMTQLRNEGWIHNVA
RHAVACFLTRGDLWVSWEEGMKVFDELLLDADWSVNAGSWMWLSCSSFFQQFFHCYCPVRYGRKADPNGDYIR
TYLPVLKNFPTKYIHEPWTAPESVQRASKCVIGRDYPMPMVDHIKQSQNNIERMKQVYQQLAHYRGKINPAIK
SPTMDHYPSPPPDFEKKRNKTGKSDIQRDSNYRVQVQA
>Portunus_tritub_XP_045118530.1_cryptochro
MTTVHWFRKGLRLHDNPALLAAVKEGAELRPVFVLDPWFVKHARVGINRWRFLSQALRDLDDSLRKLKSRLFV
VRGSPEEVFPALFKKWDVQKITWEIDTEPYAQRRDEKVERLARESGVEVVCRHGHTLYRTEKIIEANLGKAPL
TYVSLEKVVSKLGHPPKPVDTLNDLPESCHVRGEAATDVSYDVPSLKELGVEESQLEPCLYPGGETEALARLK
IYMERKSWVCKFEKPKTSPTSLQPSTTVLSPYLKFGCLSPRVMYHQLIQIYKGSAHSKPPVSLLGQLLWREFF
YTVGAVTPNFDRMEGNTVCRQIPWVRDDKLMDAWTNARTGYPFIDAIMTQLRKEGWIHHLSRHAVACFLTRGD
LWQPWEDGMKVFEELLLDADWFLNAGNWMWLSASAFFNSYFRVYSPIAFGKNTDKHGDYIRKYLPQLAKYPEN
YIYEPWKAPLATQRAAGCIIGQDYPRPIVDHSIVMKRNLDRMAKAYKAGKEKKASSDNQSSPKKKMKK
>Portunus_tritub_XP_045130719.1_cryptochro
MAVSGLTFLKLISSGNKILSSMSEGSSRVAICWLRNDLRYHDNEILLKANINADQVLPVYCYDPRHFKGTYHF
NFPKTGSHRARFLLESVANLRNTLREKGSDLVVRLGQPEQILPLLVKSLSARECHIVYQEEVTKEEKDVEIKL
KENCRQFGVKFHPLWGLTLYHRADIPFPVSRVPDTYTGFRKAVEAQSLVRAPLVMPDSMKPLPPDIDVGQLPT
LAQLGVKEQSIDRRTAFPFSGGESHGLARLRQYLWETDNVFTYKETRNGLLGSDYSTKFSPWLAHGCLSPRHI
HAEVRRYEKERQANQSTYWVLFEMIWRDYFKFVCMKFGDRVFYPSGIMGKKIRWKKDHLMFQKWKEGRTGIPF
VDANMRELQETGWMSNRGRQNVASFLIKDMGLDWRLGAEWFESQLLDHDVCSNYGNWNYAAGIGNDPRENRKF
NMIKQAFDYDPKGDFVRCWVPELAGLKEAMIHTPWKLTGNQLTSAGIALGDTYPNPILVAPEWGRHQHKGMQG
KGPAFKGPRQRGIDFYFKSPPKV
>Portunus_tritub_XP_045137570.1_photo-lyas
MQRPRCIQRLLRHLAALTNLGHFPPASTFPAPISRSVSSNTMSNGEPQAKKHKPDPDSLDIHRRLEKARRDEA
ESILDFKFNKKRVEMLSKADEVPDCDGIVYWMCRDQRVQDNWAMLFAQRLALKYKVSLRVVFCLVPKFLDATI
RHYHFMLKGLEEVESECQKLDISFNLLLGEPVDVLPDFIKSHNIGGLVTDFSPLRVPRRWLKEVKKNIPQNIP
MCQVDAHNIVPCKKASDKQEYSAKTIRKKIHDKLSDFLTQFPPVIKHPYKSKEENEAVDWEAAEKSLEVDRTV
KSVEWAVPGTKAGLDTLNEFCLKRLRKYATQRNDPNVNALSNLSPWLHFGQVSAQRCVLEVKRYKKQLKEGVD
GFVEEAVVRRELADNYCYYQENYDRIEGAYQWAITTLNNHKKDPRPYLYSLETLMEARTHDELWNSAQIQLVK
EGKIHGFMRMYWAKKILEWTASPETALETAIYLNDRFSLDGRDPNGYVGCMWSICGIHDQGWGERDVFGKIRY
MNYDGCKRKFKIDGYVSRYGGKKHKYIPPSK
>Potorous_tri_Q28811.1_photolyase
MDSKKRSHSTGGEAENMESQESKAKRKPLQKHQFSKSNVVQKEEKDKTEGEEKGAEGLQEVVRQSRLRTAPSV
LEFRFNKQRVRLISQDCHLQDQSQAFVYWMSRDQRVQDNWAFLYAQRLALKQKLPLHVCFCLAPCFLGATIRH
YDFMLRGLEEVAEECEKLCIPFHLLLGLPKDVLPAFVQTHGIGGIVTDFSPLLHHTQWVKDVQDALPRQVPFV
QVDAHNIVPCWVASDKQEYGARTIRHKIHDRLPHFLTEFPPVICHPYTSNVQAEPVDWNGCRAGLQVDRSVKE
VSWAKPGTASGLTMLQSFIAERLPYFGSDRNNPNKDALSNLSPWFHFGQVSVQRAILEVQKHRSRYPDSVTNF
VEEAVVRRELADNFCFYNKNYDKLEGAYDWAQTTLRLHAKDKRPHLYSLEQLESGKTHDPLWNAAQMQTVKEG
KMHGFLRMYWAKKILEWTRSPEEALEFAIYLNDRFQLDGWDPNGYVGCMWSICGIHDQGWAEREIFGKIRYMN
YAGCKRKFDVAEFERKISPAD
>Powellomyces_hir_TPX57374.1_PhCBS80983
MVKTIALVLLRNDLRVHDSPPLFAAHNHPQTTHILPLYVFDPRQIDLSCVPLNFSHYKPFVPPLTHFYKFPRC
GGWKARFLVESVKGLGHGLREHGSGLAVGFGMPGDVVAEVVKELESKGDKLSGIFLSKEYTHEEVCVEEAIAA
KTPQVPLHTYHSSTLYHPDDLPFKAAETPLIYTQFRLRVEPIRDLIRGPLETPDRFKPLPSFPDVPLVIMSDA
IELSWFVEPKYLDRSDKSAVPFEGGEKQGLARLNSFISRDRKSPVSTYKATRNGLLGADYSSKFSVFLANGTL
SPRQIEAALKHYETTVEKNESTYWLWFELVWRDYFKYISWRFGKSLFFERGFANDTKKQIDWKKDQSTIDGWV
AGKTGVPFVDAAMREVASTGYMSNRSRQNVASFFCHDIYQHWLIGAEWFESVLIDHDVCSNYGNWQYVAGVGT
DPRSNRWFNVVKQGREYDPDGKFIRTWCHELRDVGGDVHKVPQAADGYPQPLVTNKAWDTAKPREMGKEGNRN
ERVGKIDNGKRRLGKSRKNKKQGGEQV
>Powellomyces_hir_TPX58310.1_PhCBS80983
MPLQKRKAELAICETAGGEEVVKKVRADMRGGAEAGGREEQAVEEPVGLSHAAADALSAATSATVEEPTMPQP
TCLVWFKSDLRVRDNPALYAATTFEPGTANINSGMKQIRVGGSATTAAASASNLPVIALFLINVAEWALHDMA
PLRADFILRNLECLKHELAQLHIPLIVRVVEPDDAANHIEDRHHPVAAPTAVLEVARRVHARHVFFNREFEVD
ETRRDKATQHMLEADGRTVHTFQDQCVMDPGSVRTKEGKVYKVYSMFRTTWITHVVRDRMWDKLTPDPRPNPK

GACGAGSAVAAYLARWSEVPTQLAGDVLRVDPAQAILARTHFPAGEAEAHARLATFLADRSPMYTTHRDLVAQ
AGTSSLSPYLSSGILTTRQALHAAMRANNGRIEGGSAGLAKWISELVWREFYRHILVAFPHVCKHQPFKPEYK
NVPWLNDPLAFDAWTRGRTGYPIVDAGMRQLNTLGWMHNRCRMITAMFLTKHLLHDWRRGEHYFMQHLIDGDF
ANNNGGWQWSASSGVDPQPYFRIFNPLLQSQKCDPDGTYIRHWVPELTHLHGKALHNPHAELPAAEFARLGYP
TPIVDHKFARQRCLKAFKTALGKM
>Powellomyces_hir_TPX58791.1_PhCBS80983
MLPPPQHPHACTAPISTPAPRPNVLLWIRAKTLRLHDSPALHHALSLHPSHLHIVFCFDPFYTTHVRVGTTRW
QFLLSAARDFDSALRKLGNRLIVPRGKPGKVLPVLWDRWGVTHLVYERDEEPYPKQRDGEVEEMAREKGIVCA
RVVGHTIWDPREVAKVNGGRPPLTYAAFLSLVEKKLPRPGPPIPPPVGPLPPHGGLAVLECEPCDVWPEGKII
FKHWRGPDGSCSVPTLTELGLPEAPPPLHRGGETEALRRLSAYLSSTTLVAAFEKPKTSPTQFSPSASTTVLS
PYLKFGCLSARLFRDRLLATYAKHTGRHTHPPVSLLGQLYWREFFYAVAAHTPNFDRMEGNPLCLQVPWRLRD
RTDADVDALRDFEAWRTARTGYPWIDAIMTQLRTEGWIHHLARHAVACFLTRGHLYISWERGAEVFEQLLIDH
DWSLNTANWLWLSASAFYTQYVRVYSPVAFPKKYDPSGEYVRKYVPVLRDVPAEWIYEPWKAPLSVQKRARCV
VGVDYPERICEMEEARKRNIERMRVAYKEGVRGSVDLQRSQTTSSIPTTPVRAIQHDDVPAMLHVDAHTLTSS
SNVPPLSAVDVPAASSVSGEKTSSPADAPRRSAGAKRTIDSFFAPIKKPKHGR
>Probosciger_ater__NWS41259.1
RFLLQSLEDLDNSLRKLNSRLFVVRGQPTDVFPRLFKEWGVTRLTFEYDSEPFGKERDAAIIKLAKEAGVEVV
IENSHTLYDLDRIIELNGHKPPLTYKRFQAIISRMELPKKPVSTVISQQMETCKVDIQENHDDVYGVPSLEEL
GFPTDGLAPAVWQGGETEALARLDKHLERKAWVANYERPRMNANSLLASPTGLSPYLRFGCLSCRLFYYRLWE
LYKKVKRNSTPPLSLYGQLLWREFFYTAATNNPKFDRMEGNPICIQIPWDRNPEALAKWAEGKTGFPWIDAIM
TQLRQEGWIHHLARHAVACFLTRGDLWISWESGVRVFDELLLDADFSVNAGSWMWLSCSAFFQQFFHCYCPVG
FGRRTDPSGDYVKRYLPKLKGFPSRYIYEPWNAPESVQKAAKCIIGVDYPKPMVNHAETSRLNIERMKQIYQQ
LSRYRGLCLLASVPSCVEDLSGPVTDSASGQGCSTSTAVRLSQADQPSPKRKHEGVEELCTEELFKRAKVTGL
PAPEVPGKS
>Probosciger_ater__NWS41534.1
DNWAFLYAQRLALKQELPLYVCFCLVPKFLEATIRHYGFMLRGLQEVAEECAELNIAFHLLRGYAKDVLPAFV
VEHGVGGLVTDFSPLRLPRQWVQDVRERLPEDVPFAQVDAHNIVPCWIASPKQEYSASTIRGKIHAQLPEFLT
EFPPVVRHPYPHSCPAEPIAWEACYSSLEVDRTVKEVEWAAPGTAAGLAVLQSFIKKRLKYFGSHRNNPNKAA
LSNLSPWLHFGQVSTQRAILEVQKHQHKYKKSVDVFVEEAVVRRELAENFCYYNENYDSLQGAYEWAQTTLKL
HAKDKRPFLYTLQELEQGTTHDPLWNAAQLQMVREGKMHGFLRMYWAKKILEWTRSPEEALQFAIYLNDRYEL
DGRDPNGYVGKRCLWSICGIHDHGWTERAVFGKIRYMNYAGCKRKFDVGQFERRYPP
>Probosciger_ater__NWS44089.1
KVDYDVCSNYGNWLYSAGVGSDPRDNRKFNMVKQGLDYDGNGDYVRLWVPELQGIKGADIHTPWALSSAALSQ
AGVTLGETYPQPIVTAPEWSRHINQRPVSTGKEISRHPRGRRGPAHPSVQHKDRGIDFYFSRKKDA
>Probosciger_ater__NWS47685.1
MGVNAVHWFRKGLRLHDNPALRECIQGADTVRCVYILDPWFAGSSNVGINRWRFLLQCLEDLDANLRKLNSRL
FVIRGQPADVFPRLFKEWNIAKLSIEYDSEPFGKERDAAIKKLASEAGVEVIVRISHTLYDLDKIIELNGGQP
PLTYKRFQTLISRMEPLEMPVETITPEVMEKCTTPVSDDHDEKYGVPSLEELGFDTDGLPSAVWPGGETEALT
RLERHLERKAWVANFERPRMNANSLLASPTGLSPYLRFGCLSCRLFYFKLTDLYKKVKKNSSPPLSLYGQLLW
REFFYTAATNNPRFDKMEGNPICVQIPWDKNPEALAKWAEGRTGFPWIDAIMTQLRQEGWIHHLARHAVACFL
TRGDLWISWEEGMKVFEELLLDADWSVNAGSWMWLSCSSFFQQFFHCYCPVGFGRRTDPNGDYIRRYLPVLRG
FPAKYIYDPWNAPESIQKAAKCIIGVNYPKPMVNHAEASRLNIERMKQIYQQLSRYRGLGLLATVPSNPNGNG
NGGLMGYSPGESISGCSSAGVFTVSPGAQLGTGDGHTIVQPCALGDSHTAGASGIQQQGYCQASSILHYAHGD
NQQSHLLQAGRTALGTGISAGKRPNPEEETQSVGPKVQRQSTN
>Protomyces_lac_XP_040725890.1_DNA_photol
MARTLLYLHRHDLRLHDNPILEQIRKGQYDSFLPVYIFPAHQIEVNPLLRAEHQGKAHKELRASFGTRRTGNV
RVSYLCESLWDLKRSYAKLDVNMLITAGKTDDVLKSILDKLQAHSAGQIEVWLQKEYTTEEREEEAAIMAVCK
SNKTVAYKTVEESTLVDISDLGYPIDKLPNYYTEFRKGVEPLDSVRLPFEAPKHLPPSPKITFENEYEISDDM
DKVIEQLCAPLGGRCDIPQNTAHPMKGGETAAIKRLEYYVTGGKSAPAASYKTTRNGLLGSDYSTKFSSFLSV
GSLSPKLIHHRLLQFEKEHNLEGDVNTYWIRFELLWRDYFKFVALKFGKRMFLKYGLKQAKLNDQWLGADSRE
AMRWKEGTTGVGIVDAAMRELKATGYLSNRARQIVASFLTKDLNVDWRIGAEYFEEKLIDHDPSANYGNWQYQ
AGVGNDPRTSRRFNPAKQAHDYDPDGAYIKTWCPEVAKATVSMNWVYFWTTPPKLQQTLGIAGLEGVRDPVKG
LCGDERERNSGRGVPGYRKKARRHEGRPRRGERTASYDS
>Protomyces_lac_XP_040727286.1_FAD_bindin
MAEWQQCPTIKRAKEIDESDPYRTLGEALEDNVLPETTKNVLHWFRSKDLRIQDNKALHAASQKAEEADAPLV
TLYVHSPAHLQWHGTSPARTDLILRTIKQMQADLKKDGIPLYILQTETRQSLVPDVLKFIADNDISHVFANFE
YEHDELMRDIKLARKLKDAKTSFEIMHDQCAVDPGTLTTGSGTPHKVFTPYHKAWLAEMSANGDSLLSLVPPP
SPNSEEQHAKLEDMGLFKSPMPTIPKEKTFKNDEERDRIRKLWPAGYEEAHRRLVKFLDTKAKDYHLKRSEPS
ADATSRMSPYFSSGTISVRETLALCRQRNKNSLDFSGSKGKGISGWVREIVFREYYRQLTYVFPHKSMNMPQN
LKMAAVAYVTGKEADDNYQKWCEGKTGFPLVDAGMRQLNHEAWMHNRARMNTASLLRTNMLIDYRRGERYFAE

HLIDWDMSNNSQGWEPSYTVFNPTTQGENNDPTAEYIRKWCPELKDLDNKKIFAPFERCTRQEFEALDYPEPV
VDWKETKVRAIEALKHATATGEH
>Protomyces_lac_XP_040727966.1_DNA_photol
MGAARVIYWFRTDLRLHDSPALHAALELKPEVLYPVWTWDPHYVYHARVSSNRWRYLLDCQADVDKRIKKLTG
SNGLLVLRGNPLNVLKQALQDWKITHLVFEQDTDTYALSRDTQVRAMAEKLGVKVVSKLGRTLWDPIELNKVH
GGKPVMTLAAVQKLGSQIGPVPRPIEAPTSLPSAGDTKLEAPKDEIQDAPDLNEPARGDGRVTSYDQVAGPNG
DYSVPTMEEINIKPAEGPYRGGETLALEALEAYMADKKKTATFEKPKTNPGVFHPPETTLLSPHLHFGSLSIR
TFYWRVMDVCKAYGSGASKPPASLEGQLLFRDMYFAAQCGIDNFEQTLGNAHCRYIPWRLLQEPDEEAEAWLE
AWTHGRTGFPWIDAIMRQLRRDGWIHHLARHSVACFLTRGHCYIAWEKGAQVFEELLIDHETACNVGNWQWLS
CTAFFSQYYRVYSPVAFGKKWDKSGALIREFVPELKNVPDKYIYEPWLMSKVDMKAAGCILGQDYPKRIFDEK
EQAKVALAGMKEAYDAKLYGDSPQIGKTKSPESPVKSGKKRGQQSLDGFAKKAKIEPAEEHEANEA
>Protopolystoma_xen_VEL28982.1
MLVSSCISGYTIGPARLGAHSWAKETLRLHAKDVRNPAYSSKQIEFAQTGDHLWNSAQLQMVREGKMHGFLRM
YWAKKILEWTAEGPEEALALAIRLNDKYSLDGRDPNGYTATNKVLLLQDACGQYAEFTTKDGPNVQYTAR
>Protopterus_anne__XP_043923143.1
MSGIRTVICLLRNDLRLHDNEVLYWANLNAENIVPLYCFDPRHYGVTHNYNFPKTGPFRLKFLLEGVKDLRET
LKKKGSNLLMRRGKPEDIIQDLIKELGSVTAVAFHEEVTAEELNVENNVKQVCFQLKVNVVTFWGSTLYHRDD
LPFRHISRLPDVFTHFRKSVETQAKVRSTLQMPEMLKPVPLGLEEGSIPTYEDFSERDPLTDPRTVFPFSGGE
GPALARLKHYFWDSNLVASYKETRNGLIGAEYSTKFAPWLALGCISPRYIYEELKKYEKERTENESTYWVIFE
LLWRDYFRFVALKCGRKIFFLKGLQDKEVSWKKDQRIFDTWKDGKTGVPFVDACMRELALTGFMSNRGRQNVA
SFLTKDLGIDWRMGAEWFEYLLVDYDVCSNYGNWLYSAGIGNDPRENRKFNIIKQGLDYDANGDYIRLWVPEL
KDIKGGDIHTPWTLSSAALTHAGVNLPETYPLPVVIAPEWSRYINQKTTGGARGANSGPEVRKGPSHTPRQHR
NRGIDFYFSHNKKHY
>Protopterus_anne__XP_043926648.1
MTDFAMSETIEEQVSTDGSVKSRSENSKTDLEVNTTGKTKKDEASKEKQKKAKRKGNTSESETDDQKLKSSMK
RKTEQSAKQKDKKTLQSEMSSKESLSGSGGKKSKIESDPANNGDLVEAVKKARLKAGSSVEDFKYNKQRVHLI
SENDDLKNGAQGIIYWMSRDQRVQDNWALLYAQRLALKQKWPLHVCFCLVPKFLDATIRHFGFMLKGLQEVAE
ECKDLNIPFHLLIGYAKNKLPQFIKDHNIGGMVTDFSPLRVPLQWVQDVKENLPKDVPFVQVDAHNLVPCWIA
SDKLEYGARTIRRKIHDKLPQFLTEFPPVICHPYTANFKEQPIDWGAAYESLEVDRSVTEVEWAKPGTLAGLR
MLESFIEERLKYFHTDRNNPNRTALSNLSPWFHFGQVAVQRAILEIRKHRKFRESVDGFIEEAVVRRELADNF
CFFNKKYDSLEGAYEWAKTTLKIHATDKRSHVYTLKQLEEGKTHDPLWNAAQLQMVREGKMHGFLRMYWAKKI
LEWTRSPEEALEWSIYLNDRYELDGRDPNGYVGCMWSICGIHDQGWAERPVFGKIRYMNYEGCKRKFDVTQFE
RKYSPKNLCP
>Protopterus_anne__XP_043936748.1
MTHYSVHWFRKGLRLHDNSALLAALQDCKEIYPIFILDPWFPKNMCVSVNRWRFLIESLKDLDESLRKLNSRL
FVIRGNPAEVFPCLFKDWKVTRLTFEVDTEPYARQRDAKVVKLAEEHGVEVIQRVSNTLYDTERVIIENNGKP
PLTYVRLQTILSSLGPPKKPVPAPSLELMRNCQTPCKDLHDARYDIPTLEELGKSSAEAGPHLYPGGETEALR
RLEHYMQRTSWVCNFQKPETSPNSLDPSTTVLSPYVKFGCLSARTFWWRLSETYQGRKHSQPPVSLHGQLLWR
EFFYVIGAAIPNFDRMVGNPVCTQVDWDDNREYLQAWAEGRTGYPFIDAIMTQLRTEGWIHHLARHAVACFLT
RGDLWISWEEGQKVFEELLLDADVSLNAGNWQWLSASAFFHQFYRVYSPVAFGKKTDKNGDYIKKYLPFLKKF
PAEYIYEPWKAPRSIQQQAGCIIGTDYPKPIVLHEQVRKRNIERMKAAYARHAATKKDSMESPKKGTKCTSDH
ESLTDALKKKKRKLNN
>Protopterus_anne__XP_043938505.1
MVVNSIHWFRKGLRLHDNPALQAAIRDADTVRCVYLLDPCFAGSSTVGINRWRFLLQSLEDLDNSLRKFASRL
FVVRGQPTDIFPKLFKKWNITRLTFEYDSEPFGKERDAAIMKLAKKAGVEVIMKNSHTLYDLNKIIELNGNSP
PLTYKRFQTIISKMALPKKPVETVTPEQMERCRTNISENHNEKYGVPSLEELGFSARNAPPVMWHGGESEALS
RLDKHLERKAWVANYEKPRMNANSLLASPTGLSPYLRFGCLSCRLFFYRLLELYKTVKKNGTPPISLYGQLFW
REFFYTAATNNARFDRMERNPICVQIPWDRNPEALAKWAEGKTGYPWIDAVMTQLRQEGWIHHLARHAVACFL
TRGDLWISWESGMKVFDELLLDADFSVNAGSWMWLSCSAFFQQFFHCYCPVGFGRRMDPSGDYIRRYLPHLKN
YPTKYIFEPWTAPESVQNEARCIVGVHYPCPMVNHGEASRLNVERMKQIYQQLSHYQGLCLLASVPACAEDVA
VPLAEACGSHCNMTTPLNDSTSSKRRRESSSSLETEDEELERTKSKYLCISE
>Protopterus_anne__XP_043943524.1
MGVNVVHWFRKGLRLHDNPSLKESIEGAESVRCIYILDPWFAGSSSVGINRWRFLLQCLEDLDANLRKLNSRL
FVIRGQPADVFPRLFKEWNINRLSFEYDSEPFGKERDAAIKKLASEAGVEVIVRISHTLYDLDKIIELNGGQP
PLTYKRFQTLVSRMEPLETPVETITAEAMGKCTTPVSDDHDEKYGVPSLEELGFDTEGLASAVWPGGESEALT
RLERHLERKAWVANFERPRMNANSLLASPTGLSPYLRFGCLSCRLFYFKLTDLYRKVKKNSSPPLSLYGQLLW
REFFYTAATNNSRFDKMEGNPICVQIPWDRNPEALAKWAEGRTGFPWIDAIMAQLRQEGWIHHLARHAVACFL
TRGDLWISWEEGTKVFEELLLDADWSVNAGSWMWLSCSSFFQQFFHCYCPVGFGKRTDPNGDYIRRYLPVLRG
FPAKYIYDPWNAPLSVQKSAKCIIGVNYPKPMVNHAEASRLNIERMKQIYQQLSRYRGLGLLASVPSNANGNG

GLLSYLPGENTPSCSNSSAGFGQGSTMLRYPQGDIQMQNNTLLQVRNPVYNNITNGKRPNSEEEMQGIEAKLL
KQSGI
>Pseudo-nitzschia_mul_VEU36634.1_unnamed_pr
MFEVFLGSPTTPTRSILQYIVAALLLPICASLSTTIPPTMTKGKVVNAMHWFRKGLRLSDNPALXACLEQXPK
NIYPVYVLDGNSYQLLRCSPLRANFLVECLQDLDNNLRTLGSRLYVLSGDPTVVLPEKWEEWEITDLSYEEDE
TGEPYALQRDEKIVNLAQISDIRLFTAQSETLYPLKDYVAKSGKKPVPGTMTAFQKLFGGMPTMKKVLSHPSK
DQFPRNENLDELSKLYLPPTHPTDLPWPRGIPKAEVDSLWDASDCVNLAPVVHGGETLARKALKKKLKDANWV
ATFEKPQTSCTSLEPSTTALGPYLSWGCLSPREVWFAIDKAIAKSSVTKVSKPPVSLHGQLLWRDFNNLMAHD
ANIHHPGSWNHMEGNKYCRQIPWDDDPKLVEAWKEGKTGFPWIDAAMRQLAQEGWIHHLGRHAVACFLTRGDL
WQSWEEGAKHFEAQLLDADYALNGFNWMWLSCSGFFYQYFRCYSPISYQQKNDPTAEYIRKYLPELKDVPKKY
IYSPWEAPPLVLENAGVRLGKDYPYPLVDHKIIVKENMGRMKQAYDDHKAVEAEKAQAAKKAAAASKKNSSSS
SAQSRKKRQKTK
>Pseudo-nitzschia_mul_VEU40361.1_unnamed_pr
MGGDSAVARDCFPKTTHWCYVLLVVAVAAAVTPAFSLALPPPLRKNDCCGFHPRRQRTAQTRTTRMQRQMLKR
DAETPSLVRRKRRLVWHRNRDLRFHDHPFYGGGDRGVPTTSVFVFDEPAPVAGKGGAIGSDPFVPTRPSTCLP
GDWGTVPNLGPHGMRVLLESVEDLRRQSFLPKRPQGQSQSQPQPEQPLVVRRGPTVPVLMGLLEELVTLDESN
ESSECEYEYEVCWNEEPGWHELELSGAVRDAILDRYGKGGCEPSSYGGKTHKRKQVVVTLRTALSCTLYHPDD
LPGAGSWTPMSKKEKRRLENQRKRQKQLLRRGGGGKDPPGSLPSEGLPGAAKAPRTTGPHDLVDLSVERWSGM
PRIMGDFRRIARERAGVRACDSHSSDALGAARDAVLHGADTDARVRSGDAATKEPPEAAGGGIDPGPMPTLES
LLEPLLLYVGDGEDYDHGKKPILGLPPAVIREACYNSIRIHQKNRGESPAGRGSPAEAGVANTTTTMIGGETA
ALKHLSDFCNHHLHRAERSLACVDDHQSSHLGQYLAFGCLSPRTVVGVADAAIAKRRQAQGGNDGGEPHPGSG
ETRKNHNKNNDSDNASRVRGHNARENDGTWLVSHMTMRDFFLYTCLASGKKFYRLEGIPISQKQAAAMEWKPF
FPEPQTRTPTPHGHKRGSTPLPPSPTRSGKEPSEALDLWRAWATGTTGLPLVDAGMRELLATGYTSNRVRQNA
ASVLAKDLALDWRAGAEWFQFLLADHCVAANWGNWLYFSGVGPDPKQRHFCTVSQALKYDPSGAYVRKWLPSL
RDPPEREEGREGVREGVRHPDFHLRPWDFDGSWPTPIVDPDSQYTWRELERLKTTGRLTE
>Pseudo-nitzschia_mul_VEU43758.1
MGKAAPTEFFRRLLLKSITNTKGTRRLSSQGRBTSTSQRLTPRTTATKTTRASSLFLPGYYGLSITYCWLFAA
VSTTSLSSAFAPQIFPSHRLSPSATKAYSTSKASAPTTIANPGQKAVSSISMPFPYVFDGDRSVPSSELPSWM
LPQRTRVLTETETVSNGKAKGKXDDCVVYWMQRDVRTVDNWGLLLAQHLAQEKGLPLRVVHVLASPSPLPDTG
GEDDETSSEDDTPPPLESLRTTERHGKFLLGGLKCVHDELKQKNVPFDVVHHTSETPEDESNAYVLEXFVNVH
KPSVVVCDTSVLRNPRRWNESPSFRDALDAMGVPLYQVDAHNVVPIWYASPKREVGARTLRSKLHKVVDECLQ
NKDYKKGLIPDFVGNEHVNLSKAMEKEASKDFDYDSHVRFLEWDDSVKSVLREKPGTEAGIKKFEAFTXTGLR
HFSMLRNDPNNDLVCSXLSPWFNHGHLSFATCMRILKGHNRDAEGKASFVEEGFVRRELSDNFLWYAPDTYDT
LEAGAQWAQDSLELHRHDPREYIYSLREFERAKTHDDLWNAAQLQVVRTGKMHGFLRMYWAKKILEWTSSPAD
ALRYAQYFNDKYALDGRDPNGFCGVAWSIFGNHDMGWKEREIFGKIRFMNYNGCKRKFKVDQFVKKHPPAAKN
AXKAAVNKEVASRTIHRNQIAIANTPAVPASSTSSDSEAEQTPKRRKTTLNKXRKRQKTSASADDSEDEVEDL
SGLSKSAASRKTVKVLKAYIQSKGVSIKDDNGKPLLKAGLVDALMSMS
>Pseudo-nitzschia_mul_VEU44083.1_unnamed_pr
MLSPIQHSTFLILSTILYFLSAFLVLSSRHLVFSFSSTQRMHSGSDGKNSHPHPQLLFSSPSPMLLSQMPIPV
RVELFLPNRTNKKKKKPPNGAVRANKDDRGLVDRIRLFEEHGITSFNIVNKDKTDKDSVVEWIETIRNASLKA
SNSTDVHVCSQFSLLHNRPPKTRNLRDQRKEHLLETLKKGYFGAADEILLVSGSGTKPLSSSPCSETPWNTAD
GIDAVNDYYENRGDNDVCQTTFPHISVSYNPYLSDPHLQDQENSRLERQLATGCLSKIYLPFGTDLDRLRTGL
EFCADAIHTAKRKHQRAGDVSLVGSFVLPTPRWMAHQRKFFRSSQKRVMLDPRFFKSDENQELTAIVVQVIRM
YEKHGFGELLLEASPSANTLSCTKDDLSFLFDILKRVSASRDDGLGPNRCNDSERSSSMGMNNFRNNSNGVDF
SSNSKGTKTGAESSQSPEILKKSLPSSLRQDWRSRSSSPCILLFGSHDVRLQDNKALEAAARSHKKLLPVFLW
TKEERDGDQWGCPRNTAVAVCLEEALKSLQDSLQSFGLPLIYCNCCGPSPRHDSREEQLHQQENSHGIRELKH
VVEAVGATAVYWNKDPTPEGRDRDAYRRRILEQHRLPRRIDVFEAQSSLLYDVSTIELPTGFRGGHFGTLMPF
LNHCRKQFGVPSRPTPYFETFRILEESSPPEAFDTIFDMHGSTMSTHLRDLKIIDTFKLSHKWDQPIRARFPM
SEKIAQSTVDSFVRNGMKRYETDRSRADISDATSRLSPHLRIGTMSPNQLYWRIEDSELPYKDTKTLGRRLIW
RDLAYFQLHCFPSMKKKCIRSHYEAMEWVETIEEDRRFRAWKTGTTGYPIVDAAMRELYATGWMTQSIRMVAA
SFLTEYLRINWTKGAEWFHHTLVDADSAINAMMWQNAGRSGIDQWNFVLSPTTASQDPTGAYTRRWVPELAEL
PTNRLVHCPWEATEDLLEEHGVVLGETYPHRIVVDLKKERKKSVENTLAVRRAAQDYNTDRGYDMIDLPNGQK
SIVFTKKEYRIDRNGNVLPSNKSNGNRRASNRMGNSRTGVVRHKKSSRNPKKAKTST
>Psilopogon_haem__NXG41087.1
SLEDLDNSLRKLNSRLFVVRGQPTDVFPRLFKEWGVTRLTFEYDSEPFGKERDAAIVKLAKEAGVEVVIENSH
TLYDLDRIIELNGHKPPLTYKRFQAIISRMDLPKKPVSTVISQQMETCKVDIQENHDDVYGVPSLEELGFPTD
GLAPAVWQGGETEALARLDKHLERKAWVANYERPRMNANSLLASPTGLSPYLRFGCLSCRLFYYRLWELYKKV
KRNSTPPLSLYGQLLWREFFYTAATNNPKFDRMEGNPICIQIPWDRNPEALAKWAEGKTGFPWIDAIMTQLRQ
EGWIHHLARHAVACFLTRGDLWISWESGVRVFDELLLDADFSVNAGSWMWLSCSAFFQQFFHCYCPVGFGRRT
DPSGDYVKRYLPKLKGFPSRYIYEPWNAPESVQKAAKCIIGVDYPKPMVNHAETSRLNIERMKQIYQQLSRYR

GLCLLASVPSCVEELSGPVTDSASGQGCSTSTAVRLSQADQASPKRKHEGAEELCTEELYKRPKVTGLPSAEV
PGKSL
>Psilopogon_haem__NXG46235.1
DNWAFLYAQRLALKQELPLRVCFCLVPKFLDATIRHYSFMLKGLQEVAKECAELNIPFHLLLGYAKDVLPTFV
VEHGVGGLVTDFCPLRLPRQWVEDVRERLPEDVPFAQVDAHNIVPCWVASPKQEYSARTIRGKIHAQLPEFLT
EFPPIIRHPYTSSCLAQPISWEVCYSSLQVDRTVKEVEWATPGTAAGLAVLQSFIAERLKSFGSHRNDPNKAA
LSNLSPWFHFGQVSTQRAILEVQKHRGKYKESVDAFVEEAVVRRELAENFCYYNENYDSVQGAYDWAQTTLKL
HIKDKRPFLYKLEELEHGTTHDPLWNAAQLQMVQEGKMHGFLRMYWAKKILEWTRSPEEALQFAIYLNDRYEL
DGRDPNGYVGCLWSICGIHDQGWPERAIFGKIRYMNYAGCKRKFDVGQFERRYAPRT
>Psilopogon_haem__NXG51452.1
KVDYDVCSNYGNWLYSAGLGNDPRDNRKFNMIKQGLDYDGNGDYVRLWLPELRGIKGADIHTPWVLSSADLSQ
AGVTLGETYPQPVVTAPEWSRHVSQRPQGRSPHPRGRREPTRRPVQHRGIDFYFSHKKDV
>Psilopogon_haem__NXG53569.1
MGVNAVHWFRKGLRLHDNPALRECIQGADTVRCVYILDPWFAGSSNVGINRWRFLLQCLEDLDANLRKLNSRL
FVIRGQPADVFPRLFKEWNIAKLSIEYDSEPFGKERDAAIKKLASEAGVEVIVRISHTLYDLDKIIELNGGQP
PLTYKRFQTLISRMEPLEMPVETITPEVMAKCSTPVSDDHDEKYGVPSLEELGFDTDGLPSAVWPGGETEALT
RLERHLERKAWVANFERPRMNANSLLASPTGLSPYLRFGCLSCRLFYFKLTDLYKKVKKNSSPPLSLYGQLLW
REFFYTAATNNPRFDKMEGNPICVQIPWDKNPEALAKWAEGRTGFPWIDAIMTQLRQEGWIHHLARHAVACFL
TRGDLWISWEEGMKVFEELLLDADWSVNAGSWMWLSCSSFFQQFFHCYCPVGFGRRTDPNGDYIRRYLPVLRG
FPAKYIYDPWNAPDSVQKAAKCIIGVNYPKPMVNHAEASRLNIERMKQIYQQLSRYRGLGLLATVPSNPNGNG
NGGLMSYSPGESISGCGSTGGAQLGTGDGHTSVQPCTLGESHTGASGIQQQGYCQASSILHYAHGDNQQPHLL
QAGRTALGTGISAGKRPNPEEETQSVGPKVQRQSTN
>Psophia_crep__NXI89661.1
KVDYDVCSNYGNWLYSAGIGNDPRDSRKFNMIKQGLDYDGNGDYVRLWVPELRGIKGADIHTPWALNSAALSQ
AGVTLSETYPQPVVTAPEWSRHISQRPQGRSHHPRGSRGPAHRPVRRRGIDFYFSRRKDV
>Psophia_crep__NXI89928.1
RFLLQCLEDLDANLRKLNSRLFVIRGQPADVFPRLFKEWNIAKLSIEYDSEPFGKERDAAIKKLASEAGVEVI
VRISHTLYDLDKIIELNGGQPPLTYKRFQTLISRMEPLEMPVETITPEVMEKCTTPVSDDHDEKYGVPSLEEL
GFDTDGLPSAVWPGGETEALTRLERHLERKAWVANFERPRMNANSLLASPTGLSPYLRFGCLSCRLFYFKLTD
LYKKVKKNSSPPLSLYGQLLWREFFYTAATNNPRFDKMEGNPICVQIPWDKNPEALAKWAEGRTGFPWIDAIM
TQLRQEGWIHHLARHAVACFLTRGDLWISWEEGMKVFEELLLDADWSVNAGSWMWLSCSSFFQQFFHCYCPVG
FGRRTDPNGDYIRRYLPVLRGFPAKYIYDPWNAPESIQKAAKCIIGVNYPKPMVNHAEASRLNIERMKQIYQQ
LSRYRGLGLLATVPSNPNGNGNGGLMGYSPGESISGCGSTGGAQLGPGDGHAVVQPCALGDSHTGASGIQQQG
YCQASSILHYAHGDNQQSHFLQAGRTALGTGISAGKRPNPEEETQSVGPKVQRQSTN
>Psophia_crep__NXI95798.1
CIYILDPWFAASSAVGINRWRFLLQSLEDLDNSLRKLNSRLFVVRGQPTDVFPRLFKEWGVTRLTFEYDSEPF
GKERDAAIVKLAKEAGVEVVIENSHTLYDLDRIIELNGHKPPLTYKRFQAIISRMELPKKPVSTVISQQMETC
KVDIQENHDDVYGVPSLEELGFPTDGLAPAVWQGGETEALARLDKHLERKAWVANYERPRMNANSLLASPTGL
SPYLRFGCLSCRLFYYRLWELYKKVKRNSTPPLSLYGQLLWREFFYTAATNNPKFDRMEGNPICIQIPWDRNP
EALAKWAEGKTGFPWIDAIMTQLRQEGWIHHLARHAVACFLTRGDLWISWESGVRVFDELLLDADFSVNAGSW
MWLSCSAFFQQFFHCYCPVGFGRRTDPSGDYVKRYLPKLKGFPSRYIYEPWNAPESVQKAAKCIIGVDYPKPM
VNHAETSRLNIERMKQIYQQLSRYRGLCLLASVPSCVEDLSGPVTESASGQGCSTSTVMRLSQADQVSPKRKH
EGAEELCTEELYKRAKVTGLPAPEIPGKS
>Psophia_crep__NXJ00149.1
MPYRTIHLFRKGLRLHDNPTLLAALESSEAIYPVYILDRKFMTSVTHIGALRWHFLLQSLEDLQQNLCQLGSC
LLVIQGEYESVLRDHIQKWNITQVTLDAEMEPFYKEMEANIRCLGEELGFEVLSLVGHSLYDTERILDLSGGT
PPLTYKRFLHILSLLGDPEAPVRNLTAEDFQRCRSPDPSLAECYRVPLPVDLKIPLESPSPWRGGETEGLRRL
EQHLTDQGWVTSFTKPRTIPNSLLPSTTGLSPYFSMGCLSVRTFFYRLSNIYAQAKHHSLPPVSLQGQLLWRE
FFYTVASATPNFTKMAGNPICLQISWYEDAERLHKWKTAQTGFPWIDAIMTQLRQEGWIHHLARHAVACFLTR
GDLWISWEEGMKVFEELLLDADYSINAGNWMWLSASAFFHQYTRIFCPVRFGKRTDPEGQYIRKYLPILKNYP
SKYIYEPWTASEEEQKQAGCIIGQDYPFPMVNHKEASDHNLQLMKQVREEQYRTAQLTRDDTDDPMEMKVKRD
HSAENVSKGKVVRMT
>Psophia_crep__NXJ02549.1
DNWAFLYAQRLALKQGLPLRVCFCLAPKFLDATIRHYGFMLRGLQEVAEECAELNIPFHLLPGYAKDVLPAFV
VEHGVGGLVTDFCPLRIPRQWVEGVRERLPEDVPFAQVDAHNIVPCWVTSPKQEYSARTIRGKIHAQLPEFLT
EFPPVIRHPYPPSCPAEPIAWEACYSSLQVDRTVKEVEWATPGTAAGLAVLQSFVTERLKSFGSHRNDPNKAA
LSNLSPWFHFGQVSTQRAILEVQKHRGKYKESVDAFVEEAVVRRELAENFCYYNENYDSVQGAYDWAQTTLKL
HAKDKRPFLYELQELEQGTTHDPLWNAAQLQMVREGKMHGFLRMYWAKKILEWTRSPEEALRFAIYLNDRYEL
DGRDPNGYVGKRCLWSICGVHDQGWAERAIFGKIRYMNYAGCKRKFDVGQFERRYAP
>Puccinia_str_KNE96768.1_PSTG_09905

MVKRAIQEEDDSKNLRASKRTNLAEEETPYKHLLRLIEEGEEEKQQPRKEAKDGVIVYWMRMRDLRLTDNRAL
SLASQLAHSRNLHLVVLHILSTQDFKAHDRSPRRIDFVLRNLIDLKSRLRVLDIPLHTLTIHERHSIPDQIIQ
ILKTWNASHLTANIEHEVDELRRDIQVLELAKKNQIQVRLVHDTCIVEPGKVLTKENKAYSVFGPWQRNWANL
VNSNLDEYIGKSEDCKPNNACIKDIKTLNDLFDSEVPASLPGFELSEQDSLVMNENWKAGEKAAEDLLNRFLN
TKLEPEKKEIQTTLTGKASPPKKKEESTIVQEYSTLRNRPDLPGTSRLSAYLAAGVISPRACIRASMQIAEKG
KKAVNGLQVDRRDLGVGVWQSEIGWRDFYQHVMAAWPRVSMGKAFNQKYEKMVWEFDDEIFQKWKDGLTGYPF
IDACMRQLKHLGYMHNRGRMCVAMFLTKDLMMDWRLGEKWFMENLVDGDLGSNNGGWQWSASTGTDPQPYFRI
FAPLSQSEKSDPNGDFIRHWVPELKSLNAKQIHAPFDKLSIEKFKSLKYPKPIVDHSVVRKRALARYKNPGCA
DNE
>Puccinia_str_KNF04081.1_PSTG_02786
MGVVGPRSLTIALFRNDLRIHDNPILTHSHLATVKEGDSTRKNKVSDYVLPLYVFDERQVELSGLDGYLRQGN
PPRTEVCGFWRTGSHRLNFLCQSVYELKDQLKKKGSDLLIRFGVVESTTIKIIEELQKEGFSVDHVYMAKEVA
FEEVGTEKRLANLLAKLPQKVPLSLFHTRSLVHPNDLPFTINKTPDVYTPFRSKVESLPADKLCRPLLPLPGK
LQPFPTLPDTISKASPESGYSGSLCEGDGCAEVFGRLVKPLLSNPDIPHDSKDVKGEYKADSRSAFPYKGGES
EALRRLDDYFFQGDQPLVRSYKTTRNGLLGQAYSTKFSPFLALGCISPRKIIHSLWDHEAKFGANKDTYWVLF
EILWRDYFIFISQKFGRTLFLIKGFEGKLDPKGATQKVSYWKPYKDEKMNQGQGKGKPTTINKNAIAWLKGKT
GVPFIDANMIELRETGFMSNRGRQNVASFLAKDLELDWRIGAEWFESELLDYDPCSNYGNWQYVAGVGNDPRT
SRQFNPIKQAKDYDAQGDYVKQWIPELKNFPTNRIHSPWLMNGQEWDKHLTGPSKGDNPDHKHIQKDYPRRPI
LEQQAWKKHYQRR
>Puccinia_str_KNF06174.1_PSTG_00683
MSSKPIKLLYWFRTDLRLHDSPALIKALDLGPVEFYPVWCWDPYYVVNTPVGPNRWQFLLDSMNDLSKSICKI
NERSKLLVIRGEPSITLPYIWSRWGITHLAFEEDDDNQHSRPRDESIIKAAKQAGLEVIPTPGHTLYNQEEVM
TAAKGNLPASYRGFLTAIEKLGPPSPALDPPATLPDPGPTTLDDPLPPEFLERWKKAWTDRQTEEKDVNKKNR
TAEDQAYRSPYPLSGPAGKFEIPTIEELGIQTPTSHHRGGETVALQKLEEYLKDKEKVLKFEKPKTSPAQFDP
PSTTTLSAHLKFGTLSARLFYERIKKLEADNPKIKISTPPESLIGQLLWRDFFHLQQSKIPNFHQIDGNRICR
SLDWKLKDRNRDKNLDSEAESHLDAWSNGMTGFPWIDAIMRQLQTEGWIHHLARHSVACFLTRGHLYISWERG
AEVFDDLLIDWDPSLNAGNWMWLSASAYFSQYFRVYSPIAFGKKFDPNGDYIRHFCPELKDFPKQYIYQPWEA
PISVQKKANCVIGERYPKPLVNEKEARESCLKQMKEGYQLNKYGIDSSSKPSTPHKSKTASPDKKGVNAATTS
KRKLEKNSPGKIGSGSAKQLKLDFKPSTSSD
>Pundamilia_nyer__XP_005722760.1
MSSSRTVICLLRNDLRLHDNELFHWAQRNAEHIVPLYCFDPRHYVGTYNYSLPKTGPFRLRFLLESIRDLRNT
LISKGSNLVVRRGKPEEVVADLIRQLGSVSSVAFHEEVTSEELNVEKRVKDVCAQMKVKVHTCWGSTLYHRDD
LPFPHMSRLPDVYTQFRKAVESEGRVRPVFSTPEKLNPLPPGLEEGAIPTAEDLQQTEAVIDPRSAFPCSGGE
SHALARLKHYFWDTDAVATYKETRNGLIGVDYSTKFSPWLAMGCISPRYIYHQIKQYEKERTANQSTYWVIFE
LLWRDYFRFVSVKYGNRIFQVKGLQDKSVPWKKDMKLFDAWKEGRTGVPFVDANMRELAATGFMSNRGRQNVA
SFLTKDLGLDWRMGAEWFESLLIDHDVCSNYGNWLYSAGIGNDPRENRKFNMIKQGLDYDNNGDYVRQWVPEL
QNIRGGDVHTPWSLSNAALAHAHVTLGDTYPAPIVMAPEWSRHINKKAGGTGPSPRGKKGPSHTPKQHRDRGI
DFYFSRSKNL
>Pundamilia_nyer__XP_005729928.1
MSVTFYSKMPPSGADAQATVKWWLGEVLEGREDPEGFFAMCVSILGHLETRSQFLSLIEPLSTGDRLLHTSLT
SIYQEYFTKTEDDELELALALSLLDMKGHPLQSPSQESQPLHSGDAQNQRGSVQLNSILKPEGSNCTNQTGSW
NRESPPQTAREMEFQISTYPNKQTLGTGQTVCVSEQEGDVDQSQKPKRSKNRRQRRKCTGQHLVGLPRSPSAP
PPVLLWFRRDLRLCDNPALVASLEVGAPVIPVFIWSPKEEEGTGITVAMGGACKYWLHQALLCFCSSLEHIGS
RLVVLKASTESSLKALKELIKETGARTVLANALYEPWLKERDDLVVSALQKQGVECRMFHSYCLRDPYSVTTE
GVGLRGIGSVSHFMSCCRQNPGTAVGVPLDPPVSLPTPAHWPKGVCLDELGLARMPRRKDGTTVDWAVNIRKT
WDFSEGGAQARLEAFLHDGVYRYEKESGRADAPNTSCLSPYLHFGQLSPRWLMWDAKGARCRPLKFQRKLAWR
DLAYWQLTLFPDLPWESIRPPYKALRWSSDWGHLKAWQRGRTGYPLVDAAMRQLWQTGWMNNYMRHVVASFLI
AYLYLPWQEGYRWFQDTLVDADVAIDAMMWQNGGMCGLDHWNFVMHPVDAAMTCDPYGTYVRKWCPELAELPD
ELIHKPWKCPASMLRRAGVVFGQSYPERIITDLEERRNQSLQDVALVRREFEQYVDKRSGCDLVPLPPRLVSE
ALGLSHKDGAVVTQGKQFLLPVITRMEFKHQHEDPDADATSNPYNAVLKGYVSRKRDETIAFLNKRDFTASVM
HEAVQRKERLNSDYRRMEGLPPSPSPRGRARRTPTAKDRFSIVPGGAVTSLK
>Pundamilia_nyer__XP_005733435.1
MTHTCIHWFSKGLRLHDNPALMAALKDCKQLYPVFILDPYLHNNACVGINRWRFLIGALKDLDGSLRKLNSRL
FVVRGKPEDVLPKLFTKWKVTRLTYEYDTEPYSLQRDSKVTSLAKERGVEVIYKVSHTLYNIERIIEENNGKP
PLTYTKLQAIAKTIGPPKRPIPAPTMDDMKDVKTPSSENHEKEYGIPTLEELGLDTAPLGEDLFPGGEQEALR
RLDEHMKRTKWVCTFEKPQTSPNSLSPSTTVLSPYVTFGCLSVRTFWWRLTEVYRGNKHSDPPVSLHGQLLWR
EFFYTASLGIPSFNKMEGNSVCTQVDWDTNPDYLAAWREARTGYPFIDAIMTQLRQEGWIHHLARHAVACFLT
RGDLWISWEEGQKVFEQLLLDGDWALNAGNWQWLSASTFFHQYFRVYSPVAFGKKTDKNGDYIRKYLPLLKKF
PAEYIYEPWKAPRSIQQAAGCIVGKDYPHPIVQHEVISKKNIQRMKLAYAKRSPESPSKSKGVKRKAPSIIEM
IKKKAKVK

>Pundamilia_nyer__XP_005747322.1
MVINTIHWFRKGLRLHDNPSLKESLLGADTVRCVYILDPWFAGSSNVGINRWRFLLQSLEDLDSSLRKLNSRL
FVIRGQPTDVFPRLFKEWKITRLSYEYDSEPFGKERDAAIKKLASEAGVEVTVRISHTLYELDKIIELNGGQS
PLTYKRFQTLISRMDPVEVPAESITAEIMGKCTTPLSEDHDEKFGVPSLEELGFDTEGLSSAVWPGGETEALT
RLERHLERKAWVANFERPRMNANSLLASPTGLSPYLRFGCLSCRLFYFKLTDLYRKVKKNSSPPLSLYGQLLW
REFFYTAATNNPCFDKMESNPICVQIPWDRNPEALAKWAEGRTGFPWIDAIMTQLRQEGWIHHLARHAVACFL
TRGDLWISWEEGMKVFEELLLDADWSVNAGSWMWLSCSSFFQQFFHCYCPVGFGRRTDPNGDYIRRYLPILRG
FPAKYIYDPWNAPEVVQKAAKCIIGVHYPKPMVHHAEASRLNIERMKQIYQQLSCYRGLGLLATVPSGSNGNG
NGNGNAEASSDSMRFSETSRDAAAPSTYQMPVHSQGDWQSGVMMYVQGDPQTSVGMHQHGPEHHPTTQTSGKR
HSEDSGNSKSSKVQRQSIH
>Pundamilia_nyer__XP_005747941.1
MVVNSVHWFRKCLRLHDNPALQEALNGADAVRCVYILDPFFAGAANVGINRWRFLLEALEDLDSSLKKLNSRL
FVVRGQPTDVFPRLFKEWNVTRLTFEYDPEPYGKERDGAIIKLAQEFGVETIVRNSHTLYNLDRIIEMNNNSP
PLTFKRFQTIVSRLELPRRPLPPITQQQMDKCHTKIEDNHDQLYSIPSLEELGFRTDGLPLAVWHGGESQALD
RLGKHLDKKVWVTSLEHSRVKTCSLYASPTGLSPYLRFGCLSCRVLYYNLRELYMKVRKRCTPPLSLFGQLLW
REFFYTAATNNPNFDRMDGNPICVQIPWDQNPEALAKWAEGRTGFPWIDAIMTQLRQEGWIHHLARHAVACFL
TRGDLWISWESGMKVFEEMLLDADWSVNAGSWMWLSCSAFFQQFFHCYCPVGFGRRTDPTGDYIRHYIPVLKD
YPNRYIYEPWNAPESLQKAANCVVGVDYPKPMINHAESSRLNIERMKQVYQQLSHYRGLSLLASVPTIQEEAE
PPMTDESQTSSGPDSPQRVPANTEEDVCYEAPDSSTVCPSSTSAPHPDQEYQAPCISSVILIGNKEKRP
>Pundamilia_nyer__XP_005752546.1
MLHCVWRSTSPFSAPGRFLKLLPHHHQRPSSPALFFLTSFFSCQPTMSGTKRKASSATAGKDHRAKQQKLAPV
KQEKKEKVGGWLQSLVQQQRSEKKDVKFNKKRLRFLSDTEKIKQGSEGVLYWMLRDQRVQDNWALIHAQRLAV
KENLPLHVCFCLVVPKSELSTLRHYSFMLKGLEEVAKECEQLNVQFHLLHGAPGKVLPGFVSDHSFGAVVTDF
SPIREPLQWLEDVKKKLSKDIPFIQVDAHNIVPCWVASPKQEYSARTIRGKITNLLPEFLTDFPLVDSHPHTA
TRTAKGVDWDKTLASLDVDRNVKEPEWAKPGTKAGMDMLESFIDVRLKLFGSQRNDPNAPALSQLSPWLRFGQ
ISAQRVAWQVQRNGKNASQSVPAFIEELVVRRELTDNFCFYNKKYDSVEGAYEWAQKTLKDHAKDERPYLYTR
EQLEKAKTHDKLWNAAQYQMVTEGKMHGFLRMYWAKKILEWTSSPEEALSIALYLNDRYELDGQDPNGFVGCM
WSICGIHDQGWAERAVFGKIRYMNYKGCLRKFNVAQFERKYCPKSL
>Pungitius_pung__XP_037306365.1
MPTSACEDSKVLVRRMLVEVLMGREDPEGFFAMCVSVLGHRETSSQFLPLIRPLATANGSLHATLTSIHKEYF
SKTQDDELELALSLSLVETNDHRLSGTSQESRPQPPADGANPPTSVSRGGIRSPQTGASVKASSPQTNHAPER
HKITRIDKYETDKPGTSQTARVSGPVSKQDTFKEGDKMMDDEALSPSEKPKRSKNRRQRRKGAGQQVVGLPRS
PSARPPVLLWFRRDLRLGDNPALTGSLEVGAPVIPVFIWSPEEEGPGITVAVGGACKYWLHQALSCFSASLE
RIGSHLVFLEANRSSLHTLKELVEETGAKTVLANALYEPWLKERDDVVESALRRDGVVCKMFHSYCLRDPYSV
STEGVGLRGIGSVSHFRNCCRQNPGSALGAPLDSPASLPTPAHWPVGVSLDALGLARMPRRKDGTMIDWAANI
RTSWDFSEEGASARLEAFLNDGVYRYEKESGRADAPNTSCLSPYLHFGQLSPRWLLWDAKGARCRPPKFQRKL
AWRDLAYWQLTLFPDLPWESLRPPYKALRWSSDRGHLKAWQRGRTGYPLVDAAMRQLWLTGWMNNYMRHVVAS
FLIAYLYLPWQEGYRWFQDTLVDADVAIDAMMWQNGGMCGLDHWNFVMHPVDAAMTCDPYGSYVRKWCPELAD
LPDELIHKPWKCPPAMLRRAGVVFGQTYAERIVTDLEERRSRSLRDVALVRKEFGQYVDKGSGCDLVPLPPRL
VSQALGLAHRDAGLVTEGKRFLLPVITRMEFKHQQEDPDADAASNPYNAVLKGYVSRKRDETVAFLNERDFTA
SVMHEGVQRRERLESNYRRIEGLPQPPAPRGTARRTPTAKDKFSVAPPR
>Pungitius_pung__XP_037306498.1
MTVNSVHWFRKGLRLHDNPVLQEALDGADTVRCVYILDPWFAGAANVGINRWRFLLEALEDLDSSLKKLKSRL
FVIRGQPTDVFPRLFKEWEVTRLTFEYDPEPYGKERDGAIIKMAQEFGVETIVRNAHTLYNLDRIIEANNNSP
PLTFKRFQAIVSRLELPRRPLPPINQQQMDRCRTNIADNHNQQYSIPSLKELGFRTEGLPPAVWQGGESEALD
RLNKHLDKKVWVANFEHPRVNTCSLYASPTGLSPYLRFGCLSCRVLYYNLRELYMKLRKRCSPPLSLFGQLLW
REFFYTAATNNPNFDRMEANPICVQIPWDQNPEALAKWAEGRTGFPWIDAIMTQLRQEGWIHHLARHAVACFL
TRGDLWISWESGMKVFEELLLDADWSVNAGSWMWLSCSAFFQQFFHCYCPVGFGRRTDPSGDYIRRYIPMLKD
YPNRYIYEPWNAPESVQKAANCVVGVDYPKPMINHTEGSRLNIERMKQVYQQLSHYRGLSLLASVPTIQEEAE
PPMTDESLTGSGPDSPPKGPADWEAAGCSIAPHLSTLYPSSTFAPYPDLEGAENIHPSQTLSGSPGSHTPSSV
AAAMSTWAPPSLASSSTMSSLSSSKSSSSTCPNVSPSTQPSSLGQRRKGLTRKVRRDQRQRGRKEEVRRAEIA
GREERMEEDVEQQEEKMEEATFEEISGHQQ
>Pungitius_pung__XP_037307515.1
MAHTCVHWFRKGLRLHDNPALMAALRDCKELYPVFIVDPHLHNNTCVGINRWRFLVGALKDLDCSLRRLNSRL
FVVRGKPEEVFPELFDRWKVTKLTYEYDTEPYSLSRDKAVATLAKEHGVEVIYKISHTLYDTERIIEENSGKA
PLTYNRMQAIVKTLGPPKRPIPAPTIEDLPDAMTPCSETHETKYEIPTLEELGQDTAALGGEKFPGGEQEALK
RLDEHMKRTGWVCGFEKPQTSPNSLSPSTTVLSPYVTFGCLSARTFWWRLADVYQGKKHSNPPVSLHGQLLWR
EFFYTASVGIDNFNKMEGNPVCTQVDWDTNPEYLAAWREARTGFPFIDAIMTQLRQEGWIHHLARHAVACFLT
RGDLWISWEEGQKVFEELLLDGDWALNAGNWQWLSASAFFHQYFRVYSPVAFGKKTDKNGDYIKKYLPLLKKF

PAEYIYEPWKAPRSVQQAAGCIVGNDYPQPIVKHEVISKKNIQRMKLAYAKRSGDPAESPIKKQGIKRKAPSV
VDMLKKKDRRK
>Pungitius_pung__XP_037307927.1
MVVNTIHWFRKGLRLHDNPSLRDSIRDADSLRCVYILDPWFAGSSNVGINRWRFLLQCLEDLDASLRKLNSRL
FVIRGQPTDVFPRLFKEWQTSRLSYEYDSEPFGKERDAAIQKLASEAGVEVMVRTAHTLYNLDKIIELNDGQS
PLTYKRFQALVNRMDAVELPAETITSEVIKKCATPLSKDHDDKFAVPSLEELGFETEGLTTAVWPGGETEALM
RLERHLERKAWVANFERPRMNANSLLASPTGLSPYLRFGCLSCRLFYFKLTDLYRKVKKNSTPPLSLYGQLLW
REFFYTTATNNPCFDKMEGNPVCVQIPWDRNPEALAKWAEGRTGFPWIDAIMTQLRQEGWIHHLARHAVACFL
TRGDLWVSWEEGMKVFEELLIDADWSVNAGSWMWLSCSSFFQQFFHCYCPVGFGRRTDPNGDFIRRYLPVLRG
FPAKYIYDPWNAPEEVQKAAKCIVGVHYPRPMVNHAESSRVNIERMKQIYQQLSSYRGLGLLATVPANPNSVA
NDGIFGVVNSGASRGPGGSSEGLSTHAASSFQTERGNSTQKRRREEDISLQSSAKSWRQSN
>Pungitius_pung__XP_037311500.1
MSTSRTIICLLRNDLRLHDNEPFHWAQRNAEYIVPLYCFDPRHYVGTYNYNLPKTGPFRLHFLLEGVKDLRNT
MLSKGSNLVVRRGKPEEVVADLIKQLGSVSTVAFHEEVTSEELNVEKRVKDVCARMKVKVHTCWGSTLYHRDD
VPFHHISRLPDVYTQFRKAVETQSRVRPLFPAPEQLKPLPEGLEEGAIPTAEDLEQTGPVTDPRSAFPCSGGE
SQALARLKHYFWDTDAVATYKETRNGLIGMDYSTKFAPWLAMGCISPRYIYHQIQQYEKERTANQSTYWVIFE
LLWRDYFKFVAVKYGNRLFQIKGLQEKSVPWKKDMTLFNAWKEGRTGVPFVDANMRELAMTGFMSNRGRQNVA
SFLTKDLGLDWRMGAELFEYLLIDHDVCSNYGNWLYSAGIGNDPRENRKFNMIKQGLDYDGNGDYVRRWVPEL
QGIRGADVHAPWTLSTAALLHAGVSLGETYPTPIVMAPEWSRHVNKKPSGAGPSPRGRKGPSHTPKQHGDRGI
DFYFSRSKNL
>Pungitius_pung__XP_037313207.1
MLLWVCRSATLSSDARRLLGLVVRSDRRPTSPLSLLSQSLPCQAVMSARKRKATSAAAEPSAKQQKKEEEGEK
NDERAAGWLKDLLKQLRKENKDMKFNKKRLRFISDTESIKQGSQGVLYWMLRDHRVQDNWAMVHAQQLALKEN
LPLHVCVCLLVPQSELSTLRHYSFMLKGLEEVAKECKALDIQFHLLHGSPGDVLADFVAEHSLGAVVTDFSPL
REPLRWLEEVKATLPNEIPLIQVDAHNIVPCWVTSPKLEYAARTIRGKITKLLPEFLTEFPLVEKHCCTATTT
AKPVDWDKKLASLQVDRAVGDTEWARPGTGGGLAMLESFIDVRLKLFDTKRNDPNSAALSQLSPWIRFGHLSA
QRVATQVQSSGKSAGHSVASFIEELVVRRELTDNFCFYNEKYDSIKGAYEWAQKTLKDHAKDKRPYLYTREQL
EEAKTHDKLWNGAQYQMVTEGKMHGFLRMYWAKKILEWTSSPEEALSIALYLNDRFSLDGQDPNGFVGCMWSI
CGIHDQGWKEREVFGKIRYMNYKGCLRKFDVAAFERKYRPKNH
>Pygocentrus_natt__XP_017540798.1
MPHRTLHLFRKGLRLHDNPTLLGALESSSVVFLVYVLDRAFEEEAMRVGFLQWRFLLQSLLDLRGRLSAVGSR
LYVLQGSHRAVLRELVVRWRITQISFDLETEPHHVALQRELGEMAQELGLTVHTCVAHTLYDPRRIIDANGGE
APLTYKKFLRVVSVIGEPDKPVRDITAEDFQRCYSPEEVECEQHCIPSLSELGLDGSDEAQAEVLWPGGETHA
LQRLHKHLESQGWVQNSKPQAVPNSLWPNTTGLSPYFSFGCLSVRTFYHTLSRVYARARNHALPPVALQGQVL
WREFFYTAAAHTPNFTRMEGNPICLQIRWNHDPDALQKWRTAQTGFPWIDAIMTQLRQEGWIHHLSRHAVACF
LTRGDLWISWEEGMKVFEEYLLDADYSVNAGNWLWLSASAFFHQYNRIFCPVRFGRRTDPEGRYIRKYVPVLK
NFPSRFIYEPWSAPEEVQLQAGCIIGEDYPFPMVCHAEASERNLALMKEARSCQENTAKLTRDVAEEPVDVRV
KRELGEEDQPSKRFSSDPQSRPCSWGQENPRLPEITGQAM
>Pygocentrus_natt__XP_017545666.2
MVVNTVHWFRKGLRLHDNPSLRDSIAGADTLRCVYILDPWFAGSSNVGINRWRFLLQCLEDLDASLRKLNSRL
FVIRGQPTDVFPRLFKEWKITRLSYEYDSEPFGKERDAAIKKLASEAGVEVTVRISHTLYDLDKIIELNGGQS
PLTYKRFQTLISKMDAVEMPAETITAEIMGRCATPISDDHDEKFGVPSLEELGFETEGLSSAVWPGGETEALT
RLERHLERKAWVANFERPRMNANSLLASPTGLSPYLRFGCLSCRLFYFKLTDLYRKVKKNSIPPLSLYGQLLW
REFFYTAATNNPRFDKMEGNPICVQIPWDRNPEALAKWAEGRTGFPWIDAIMTQLRQEGWIHHLARHAIACFL
TRGDLWISWEEGMKVFEELLLDADWSVNAGSWMWLSCSSFFQQFFHCYCPVGFGRRTDPNGDYIRRYLPILRG
FPAKYIYDPWNAPEGVQKLAKCIIGVHYPKPMVNHAEASRINIERMKQIYQQLSCYRGLGLLATVPSNSGGNG
DGAGAGPGDSTQESNAAVHLTPANPREDLSASTFSCSHSDQQPGISVQPQTSFSGAGRVGSHREDSKQTLRGR
GTFHMKRHSEEPASSNGCKIQRQNSN
>Pygocentrus_natt__XP_017554325.1
MVVNSVHWFRKGLRLHDNPALQEALNGADTVRCVYILDPWFAGSANVGVNRWRFLLESLEDLDTCLRKLNSRL
FVVRGQPADVFPRLFKEWNVTRLTFEYDPEPYGKDRDGAIIKISQEFGVETIVRNSHTLYSPDRIIEMNSNSP
PLTFKRFQAIVNRLELPKKPLPTITQEQMASCRTQIADNHDEHYSVPSLEELGFKSQPDVTPVWKGGETEALE
RLNKHLDRKAWVASFERPRINAHSLFANPTGLSPYLCFGCLSSRVFYYHLRELYMKLRRKSSPPLSLFGQLLW
REFFYTAATNNPNFDRMEGNPICVQIPWDHNPEALAKWAEGCTGFPWIDAIMTQLRQEGWIHHLARHAVACFL
TRGDLWISWESGTKVFEELLLDADWSVNAGSWMWLSCSAFFQQFFHCYCPVGFGRRTDPSGDYIRRYIPKLKD
YPNRYIYEPWNAPESVQKAANCVVGVDYPKPMINHAESSRLNIERMKQVYQQLSHYRGLSLLASVPTIQEEAE
PPLSDDSQASSSGVPSSSTQTQSQQTQTQPQPQPPDPDPQLPATASVPPAQRKRGRTPEHRTSLNPKVQHTSQ
AKASEQPPQTTNKQ
>Pygocentrus_natt__XP_017573179.1

MKAPLCETAELLPQVSKRWSPLKMASSRTVVCLLRNDLRLHDNEVFNWAQRNAEHIVPLYCFDPRQYLGTHHF
NFPKTGPFRLRFILDSVKDLRDILKKKGSNLIVRCGKPEEVVSDLIKQLGSVSAVAFHEEVASEEKRVEMKVK
HVCSQNKVKVQTFWGSTLYHRDDLPFCHIGGLPDVYTAFRKAVEAQSRVRPVLPLAEHIRPLPLGLEEGPIPT
FDDLEQREPLDDTRSAFPCRGGESEALARLKHYFWETNAVAAYKETRNGLIGVDCSTKLAPWLAVGCISPRYI
YEQLKKYEAERTANQSTYWVVFELLWRDYFRFVALKYGDRIFHLNGLQDKHVPWKTDMELFNAWKEGRTGVPF
VDANMRELFLTGFMSNRGRQNVASFLTKDLGLDWRLGAEWFEYLLVDYDVCSNYGNWLYSAGVGNDPRENRKF
NMIKQALDYDSNGDYVRQWVPELKGIMGGDVHTPWALSSASLSRAQVALDETYPSPIVKAPEWSRHINMKPSG
AGSSRGKKGPSHTPRQHRDRGIDFYFSKSKNL
>Pygocentrus_natt__XP_037391061.1
MLCVQRCASAGRRAGNLTTSAASPPSLELRAALLTFLHRRAASMSARRAELKRKAEALGSAAEGKKRKAEESG
GERGGERGGERGGERAERGERQAGWLQLEVAELRARNAACKFNTKRVRFLSENQKVPQGCGGVLYWMARDQRV
QDNWAVVYAQQLALAEKLPLHICFCLVPRYLDAAYRQYAFMIRGLRKVAKECKSLDIQFHLLQGEPERTLPGF
VKSWNIGAVVMDFNPLRLPLQWTEKVKKQLPADIPFMQVDAHNVVPCWEASPKLEYGARTIRGKITKLLPEFL
TEFPPVDIHPHSSTKTAKAVDWDEVLSSVEVDRTVGEVEWAQPGAAEGMAMLESFIQHRLRLFATKRNNPNSA
ALSQLSPWLHSGQLSAQRVVKEVQRWGKNARESVTSFTEELVVRRELADNFCFYNKNYDSIDGAYDWAKKTLK
DHAKDKRPYLYTQEQLENARTHDQLWNAAQRQLLLEGKMHGFMRMYWAKKILEWTSSPEEALAIAVYLNDHYA
LDGCDPNGYVGCMWSICGIHDQGWAERPVFGKVRYMNYDGCKRKFDVGQFERRYAVKKA
>Pygocentrus_natt__XP_037403264.1
MIFWQSMKHKCIHWFRKGLRLHDNPALKAALHDCEHIYPIFILDPWFPKNAQVGINRWRFLVEALKDLDSSLK
KLKSRLFVVRGVPRDVFPKLFKEWKVTRLTFEVDTEPYSQIRDKEVMDLAKQHGVEVITKISHTLYNIERIIE
ENNGKPPLTYVRFQTVVKAIGTPKRPVPAPTREDMKGILTPSSDKHDENYGIPTLKELGQDSSTLGPDLYPGG
EQESLRRLEEYMQRTGWVCSFEKPQTSPNALSPSTTVLSPYVKFGCLSARTFWWGLAEVYQGRKHSAPPVSLH
GQLLWREFFYTAAVGIPNFSKMEGNPNCVQVDWDSNPEHLAAWKEARTGFPFIDAIMTQLRQEGWIHHLARHA
VACFLTRGDLWISWEEGQKVFEELLLDADWSLNAGNWQWLSASAFFHQYFRVYSPIAFGKKTDKNGDYIKKFL
PVLKKFPPEYIYEPWKAPKSVQQQAGCIVGKDYPRPIVDHDVVSKRNIQRMKAAYAKRHGDPSSSQTESKGQK
RKSASIKEMFTKKAKK
>Pygoscelis_papu__KAF1442024.1
MPHRTIHLFRKGLRLHDNPTLLAALESSEVVYPVYILDRKFMTSVMHIGDLRWHFLLQSLEDLQKNLCQLGSC
LLVIQGEYESVLRGHIQKWNITQVTLDAEMEPFYKEMEASIRRLGEELGFEVLSLVGHSLYDTKRILDLNSGT
PPLTYKRFLHILSLLGDPEVPVRNLTAEDFQRCRPPDPGLAECYRVPLPVDLKISLESLSPWRGGETEGLQRL
EQHLTDQGWVASFTKPRTIPNSLLPSTTGLSPYFSMGCLSVRTFFYRLSNIYAQTKHHSLPPVSLQGQLLWRE
FFYTVVSATPNFTKMAGNPICLQINWREDAERLHKWKTAQTGFPWIDAIMTQLRQEGWIHHLARHAVACFLTR
GDLWISWEEGMKVFEELLLDADYSINAGNWMWLSASAFFHQYTRIFCPVRFGRRTDPEGQYLRKYLPILKNFP
SKYIYEPWTASEEEQKQAGCIIGQDYPFPMVNHKEASDHNLQLMKQVREEQYRTAQLTR
>Pygoscelis_papu__KAF1442728.1
WFASENKCFFTISSRFLLQCLEDLDANLRKLNSRLFVIRGQPADVFPRLFKEWNIAKLSIEYDSEPFGKERDA
AIKKLASEAGVEVIVRISHTLYDLDKIIELNGGQPPLTYKRFQTLISRMEPLEMPVETITPEVMEKCTTPVSD
DHDEKYGVPSLEELGFDTDGLPSAVWPGGETEALTRLERHLERKAWVANFERPRMNANSLLASPTGLSPYLRF
GCLSCRLFYFKLTDLYKKVKKNSSPPLSLYGQLLWREFFYTAATNNPRFDKMEGNPICVQIPWDKNPEALAKW
AEGRTGFPWIDAIMTQLRQEGWIHHLARHAVACFLTRGDLWISWEEGMKVFEELLLDADWSVNAGSWMWLSCS
SFFQQFFHCYCPVGFGRRTDPNGDYIRRYLPVLRGFPAKYIYDPWNAPESVQKAAKCIIGVNYPKPMVNHAEA
SRLNIERMKQIYQQLSRYRGLGLLATVPSNPNGNGNGGLMGYSPGESISGCGSTGGHLFPASPGAQLGTGDGH
TVVQPCALGDSHIGASGIQQQGYCQASSILHYAHGDNQQSHLLQAGRTALGTGISAGKRPNPEEETQSVGPKV
QRQSTN
>Pygoscelis_papu__KAF1450427.1
RFLLQSLEDLDNSLRKLNSRLFVVRGQPTDVFPRLFKEWGVTRLTFEYDSEPFGKERDAAIVKLAKEAGVEVV
IENSHTLYDLDRIIELNGHKPPLTYKRFQAIISRMELPKKPVSTVMSQQMETCKVDIQENHDDVYGVPSLEEL
GFPTDGLAPAVWQGGETEALARLDKHLERKAWVANYERPRMNANSLLASPTGLSPYLRFGCLSCRLFYYRLWE
LYKKVKRNSMPPLSLYGQLLWREFFYTAATNNPKFDRMEGNPICIQIPWDKNPEALAKWAEGKTGFPWIDAIM
TQLRQEGWIHHLARHAVACFLTRGDLWISWESGVRVFDELLLDADFSVNAGSWMWLSCSAFFQQFFHCYCPVG
FGRRTDPSGDYVKRYLPKLKGFPSRYIYEPWNAPESVQKAAKCIIGVDYPKPMVNHAETSRLNIERMKQIYQQ
LSRYRGLCLLASVPSCVEDLSGPVADSASGQGCSTGTAVRLSQADQASPKRKHEGAEELCTEELYKRAKVTCL
PAPEIPGKSL
>Pygoscelis_papu__KAF1670776.1
DNWAFLYAQRLALKQELPLHVCFCLVPKFLDATIRHYGFMLKGLQEVAEECAELNIPFHLLLGYAKDVLPTFV
VEHGVGGLVTDFCPLHLPRQWVEDVRERLPEDVPFAQVDAHNIVPCWVASPKQEYSARTIRGKIHAQLPEFLT
EFPPIICHPYPPSCPAEPIAWEACYSSLQVDRTVKEVEWATPGTAAGLAVLQSFIAERLKSFGSHRNDPNKAA
LSNLSPWFHFGQVSTQRAILEVQKHRGKYKESVDAFVEEAVVRRELAENFCYYNENYNSVQGAYNWAQTTLKL
HAKDKRPFLYELQELEQGTTHDPLWNAAQLQMVQEGKMHGFLRMYWAKKILEWTRSPEEALQFAIYLNDRYEL

DGRDPNGYVGKLQDGGAGWRVFDGQGCSCLWSICGIHDQGWAERAIFGKIRYMNYAGCKRKFDVGQFERRYAP
H
>Pygoscelis_papu__KAF1675079.1
KVDYDVCSNYGNWLYSAGIGNDPRDNRKFNMIKQGLDYDRNGDYVRLWVPELQGIKGADIHTPWALNSAALSQ
AGVALGETYPQPVVTAPEWSRHINQRPQGRSPHPRGRRGPAPPPMRHKDRGMDFYFSREKDV
>Pyrrhocoris_apt_cpd_photolyase_MW662130
MAPPVKKLKTTENTPAFDAFLNSIEADRTKVGPSILEFSFNKKRARVLSKAKDLPEWAEGIMYWMTREERIQD
NWSLLYAQRLAMKSKLPLHIYFCLRKTFMNAPIRHFKFLLKGLEEVANESEKLTIPFHMVISNEEGSNTVLDF
VMENKIGCVVIDFSPLRIGRSWAEKLKEALPEDLPLVEVDGHNIVPCWIASDKLEYGARTIRNKINSKLNDFL
TPFPPVIKHPYSGKLKSKEIDWQVAEASLEVDRSVDVVSWAKPGYINGIKMLYEFCNDRLKKFKDKRNDPLGN
AISNLSPWFHFGQISVQRAILTVKSFKSANKESVECFCEEAIVRRELADNFCYYNPNYDKLEGAYDWAKKTLN
DHRKDKREWLYTQEELEKSLTHDDLWNSAQIQLVTEGKMHGFLRMYWAKKILEWTESPEEALAITIYLNDRYS
LDGRDPNGFVGCMWSICGIHDQGWKERQVFGKIRYMNYAGCKRKFDVEAFVSRYGGKVVKKKR
>Pyrrhocoris_apt_cry-m_MW662132
MAEKHTVHWFRKGLRLHDNPSLRHGLKGAKTFRCIFILDPWFANASNVGINKWRFLLQCLEDLDRSLMKLNSR
LFVIKGQPADILPKLLKEWGTTCLTFEEDPEPFGRVRDQNIMAMCKGMNITVISHVAHTLYKLERIIEKNGGK
APLTYHQFQSVIARMEDPPIPDSPVTSAIIGEAVSPISDDHDDKYGVPTLEELGFDVEGLLPGVWPGGESEAL
SRLERHLERKAWVASFGKPKMTPQSLLPSQTGLSPYLRFGCLSTRLFYYQLNDLYRKIKKAIPPLSLHGQVLW
REFFYCAATKNPNFDRMIGNPICVQVPWDKNPEALAKWANGQTGFPWIDAIMTQLREEGWIHHLARHAVACFL
TRGNLWISWEEGMKVFEELLLDADWSVNAGMWMWLSCSSFFQQFFHCYCPVRFGRKADPNGDYIRKYLPILKN
FPTKYIHEPWNCPATVQKASKCIIGTDYPLPMLNHSLVSKHNIERMKQVYLQLMKFRQPGISLSLNSESSTER
TKKTEESIYEENIFAPPASTFRGSLNKK
>Pythium_oli_TMW55947.1_Poli38472_
MRRVLEASSGLRLPNGFHMERVQWLYDGLATSLTVPRPTSESFDGRPPPKHLVYWMQTSVRSKYNYSLEYAIA
VANALSLPLHVVYFLSDRSVVPPSYQPRDPNAFGFATERHAKFGLEGLACVQKKLAKRGLSFEVFHHRHVVSP
SDKPIEFWTDVDEASEEDPPSTLKPSRSMLLDRCARNAAAVITDRPYLRPWREALAQCVSDAKAQRREWGVVQ
VEGDVVVPCESASYKEEYAARTIRPKIQAQLKKFLVELDHQEVKEECKTVGKSIVTSAFQDDTVLVPLDVTDT
DKALQLLDVDRTVRGVSCFLGGEDVAVETGKTFLEKKLVRYATDRNEPSGDGGSNLSIYLRYGHISPVRIALN
AQKVKNAKEGKDSFLEELIVRRELSVNMGVFNASYDTMACLPDYAAKTLQDHAGDKREHLYTVEQLEAAKTFD
VYWNAAQLEMVYTGAMHGYMRMYWAKKVLEWTKTPELGFRIGLYLNNKYALDAPDPNSYTGIAWSFGKHDQGW
KERPIFGKVRYMNESGLKRKFRMDAYVNKVKKLQSGDKDSNAGILDLPKQIKSPAKPRKTKKLVEDEGDSPVS
ASSPTPKKRKTLDNFFSKK
>Pythium_oli_TMW57503.1_Poli38472_
MEEPNELLALASLPLADESDRRVQSSVVPSVELDVEVEAAQAAVNAQVPPSPQLSTHRTMRGLVWFRRDLRIH
DNHALHAALQHVPKGMTLIPLYIIHRPKVMLCGVNRFQFLIESVSSLSEAIAERGSRLIIARGDSVEVLKRVL
PAWDITHLFFDNVTEPFGIERDNRVRTVADALNIKVETTNGCTLYDLDAVIEKHSGRAPKTYTSFLRTISTFP
IPSKPLDTPQELPPPMCLPSELYQQIVDHWSAIAQDDAEIQKQVEIIAGPEQSFTIPEVTEFGYETPARHSFI
YGGERIALNILSEYCKNEGRVVKFEKPKTSPAETRPSSSTTSLSPYLYFGCISPRLFYHRVREIQERHQRASS
APPVSLDGQLLWREFFHCNGHANPFLDKMEENPICLQIDWRWHTIPEREEDMTEDDKLAKAQFEAWTEGQTGY
PWIDAIMIQLKEEGWMHHLARHSVACFLTRGDLYISWVRGLEVFQERLIDHDWCINGGNWLWLSASAFFSAYF
RVYSPSTFGKKWDPEGKFIRKYIPQLRNMPTKYIYEPWKSPVTVQRAAGCLIGKDYPFPIVDHKIAQQKCMAG
MKQSYAMRRYGVSPLESLGSPRAKRPREDSDTSDDASMYY
>Python_bivittatus_XP_007436636.1_cryptochro
MAALSGLVPRPRSGGCSVHWFRRGLRLHDNPALQAALREATSVRCIYILDPWFAASSAVGINRWRFLLQSLED
LDNSLRKLNSRLFVVRGQPTDVFPRLFKEWGVTHLTFEYDSEPFGKERDAAIVKLAKEAGVEVTTENSHTLYD
LDRIIDLNGHKPPLTYKRFQAIISRMDLPKKPVSTVTSQQMEMCKTEIQENHDDTYGVPSLEELGFPTEGLAP
AVWQGGETEALTRLDKHLERKAWVANYERPRMNANSLLASPTGLSPYLRFGCLSCRLFYYRLWELYKKVKRNS
TPPLSLYGQLLWREFFYTAATNNPKFDRMEGNPICIQIPWDRNPEALAKWAEGKTGFPWIDAIMTQLRQEGWI
HHLARHAVACFLTRGDLWISWESGVRVFDELLLDADFSVNAGSWMWLSCSAFFQQFFHCYCPVGFGRRTDPSG
DYIRRYLPQLKGFPSRYIYEPWNAPESVQKAAKCIIGIDYPKPMVNHAETSRLNIERMKQIYQQLSRYRGLCL
LASVPSCIEDFSNLTTDFSSAHGSYSSTTLRQSHCGQTSPKRKCDAEADHSEELHKRARIMALQISKNPQQEL
LPPEIGN
>Python_bivittat_XP_007441920.2
MTLHPCILLADNWAFLYAQRLALKQRLPLHVCFCLVPKFLDATIRHFGFMLRGLEEVAEECRTLDIPFHLLIG
YAKDTLPPFVAQRGIGGVVTDFSPLRLPMKWIQEVLEQLPSDVPFAQVDAHNIVPCWVASDKQEYGARTIRRK
IHDQLAEFLTEFPPVIKHPFPVGLQEEPISWDACYSSLQVDCSVKEVDWAKPGAASGLKVLEEFVKERLKHFA
SGRNNPNQAALSNLSPWFRFGQVAVQRAILEVYKHRTRYKESVEGFIEEAVVRRELADNFCFYNPNYDKIEGA
YDWAKTTLKIHAKDKRPHLYTLKQLEEGKTHDPLWNAAQLQMVQDGKMHGFLRMYWAKKILEWTRSPEEALQF
SIYLNDRYELDGRDPNGYVGCMWSICGIHDQGWAERAVFGKIRYMNYAGCKRKFDVGQFERKYGAHSFTQ
>Python_biv_XP_007432430.1_cry-1_X1

MGVNSVHWFRKGLRLHDNPALREVIQGADTVRCVYILDPWFAGSSNVGINRWRFLLQCLEDLDANLRKLNSRL
FVIRGQPADVFPRLFKEWNISKLSIEYDSEPFGKERDAAIKKLATEAGLEVIVRISHTLYDLDKIIELNGGQP
PLTYKRFQTLISRMEPLEMPVETITAEVMSKCTTPVSDDHDEKYGVPSLEELGFDTDGLPSAVWPGGETEALT
RLERHLERKAWVANFERPRMNANSLLASPTGLSPYLRFGCLSCRLFYFKLTDLYKKVKKNSSPPLSLYGQLLW
REFFYTAATNNPRFDKMEGNPICVQIPWDKNPEALAKWAEGRTGFPWIDAIMTQLRQEGWIHHLARHAVACFL
TRGDLWISWEEGMKVFEELLLDADWSVNAGSWMWLSCSSFFQQFFHCYCPVGFGRRTDPNGDYIRRYLPILRG
FPAKYIYDPWNAPEGVQKAAKCIIGINYPKPMVNHAEASRLNIERMKQIYQQLSRYRGLGLLASVPSNGNGNG
NGNGNGNGGLMGYSPGENLPGCAGAGAGGAQMGTSEGHTGNVQACTLGETHTGTSGIQQQGYSQGNSGILHYA
HGDSQKTLLMQQGRTSLSVGVCTGKRPNPEEGIQSIGPKVQRQSSN
>Ramazzottius_var_GAU94194.1_RvY_06016
MNKISGLLLRRRLLPDHLRRASKSATRTMPPLSKKFKKEDGEPSSSESIADVSSTADQPETTQTIDGVVSDIF
LPSANPLYEPPTLTSEQKKFINKINQSRKNWGSPMMFPYRMERVRPIIPATSPQPISPGQCVLYWMSRDTRVQ
DNWAFIHAQRMALQARVPLLACFCLVPKYLGAPLRHFQFLLGGLVEVQKECSQLNIPFHMLIGFAKDVLPQFL
KDKNVGLLVNDFSPLKGPMSWMNEAKERMDGTVAWDQVDAHNIVPCWTASDKEEYGARTLRSKIHKVKDRYLT
PFPAIVKHPYSMIEEMPQIDWVACEASLEVDRSIGAVDWAKAGTSHSLVTLHSFITKRLPNYGKRNDPNVPAL
SQLSPWFHYGQISVARAILETEQAAKDKPSLRAGFDTFFEEAVIRRELSDNFCFYNSNYDNIDGAKPWAKQTL
LAHMKDKRPILYTFKELEECRTADDLWNAAQMQMNREGKCHGFMRMYWAKKILEWTENPQQALEFSIKLNDKY
SLDGRDPNGYVGCMWSICGTHDQGWAEREVFGKIRYMNYNGCKRKFDVAGFVSKYSKYFPKPVAVKVKY
>Ramphastos_sulf__NXP71433.1
DNWAFLYAQRLALKQQLPLHVCFCLVPKFLDATIRHYSFMLKGLQEVAKECAELNIPFHLLLGYAKDVLPTFV
VEHGVGGLVTDFCPLRLPRQWVEDVRERLPEDVPFAQVDAHNIVPCWVASPKQEYSARTIRSKIHAQLPEFLT
EFPPIIHHPYTSSCLAQPIAWEVCYSSLQVDHTVKEVEWATPGTAAGLAVLQSFIAERLKFFGSHRNDPNKAA
LSNLSPWFHFGQVSTQRAILEVQKHRSKYKESVDVFVEEAVVRRELAENFCYYNENYDSMQGSAYDWAQTTLK
LHIKDKRPFLYKLEELEHGTTHDPLWNTAQLQMVREGKMHGFLRMYWAKKILEWTHSPEEALQFAIYLNDRYE
LDGRDPNGYVGCLWSICGIHDQGWPERAIFGKIRYMNYAGCKRKFDVGQFERRYAPCT
>Ramphastos_sulf__NXP71993.1
MGVNAVHWFRKGLRLHDNPALRECIQGADTVRCVYILDPWFAGSSNVGINRWRFLLQCLEDLDANLRKLNSRL
FVIRGQPADVFPRLFKEWNIAKLSIEYDSEPFGKERDAAIKKLASEAGVEVIVRISHTLYDLDKIIELNGGQP
PLTYKRFQTLISRMEPLEMPVETITPEVMAKCSTPVSDDHDEKYGVPSLEELGFDTDGLPSAVWPGGETEALT
RLERHLERKAWVANFERPRMNANSLLASPTGLSPYLRFGCLSCRLFYFKLTDLYKKVKKNSSPPLSLYGQLLW
REFFYTAATNNPRFDKMEGNPICVQIPWDKNPEALAKWAEGRTGFPWIDAIMTQLRQEGWIHHLARHAVACFL
TRGDLWISWEEGMKVFEELLLDADWSVNAGSWMWLSCSSFFQQFFHCYCPVGFGRRTDPNGDYIRRYLPVLRG
FPAKYIYDPWNAPESVQKAAKCIIGVNYPKPMVNHAEASRLNIERMKQIYQQLSRYRGLGLLATVPSNPNGNG
NGGLMSYSPGESISGCVMSLFSASPGAQLGTGDGHTGVQPCALGDSHTGASGIQQQGYCQASSILHYAHGDNQ
QPHLLQAGRTALGTGISAGKRPNPEEETQSVGPKVQRQSTN
>Ramphastos_sulf__NXP72287.1
MSHHTIHLFRKGLRLHDHPTLLAALRSSRRLYPVYILDLQFISSAMQMGTLRWHFLLQSLEDLQKSLCQLGSS
LLVIQGQYEAVLRERVRQWSITQVTLDAEVEPFYREMEANIRCLGQELGFEVLSLVGHSLYDTKRVLDLNGGD
PPLTYKRFLHILSLLGDPELPVRSITAEDFQRCQPLDPGLAERYRVPLPADLKMPPESLSPWRGGETEGLQRL
EQHLTDQGWVATFTKPRTIPNSLMPSTTGLSPYFSLGCLSVRTFFYRLAKIYAQAKHHSLPPVSLQGQLLWRE
FFYTVASVTPNFTRMAGNPLCLQISWAEDAERLHKWKTAQTGFPWIDAIMTQLRQEGWIHHLARHAVACFLTR
GDLWISWEEGMKVFEELLLDADYSINAGNWLWLSASAFYHQYRRILCPVRFGRRTDPEGHYIRKYLPILKNFG
SKHIYEPWTASEEEQKQAGCIIGRDYPFPMVNHKEVSDHNLQLMQQVREEQHRTAQLTRDDRDDPMEVKVRPE
CSEEDLPRGKVARVAE
>Ramphastos_sulf__NXP76113.1
RFLLQSLEDLDNSLRKLNSRLFVVRGQPTDVFPRLFKEWGVTRLTFEYDSEPFGKERDAAIIKLAKEAGVEVV
IENSHTLYDLDRIIELNGHKPPLTYKRFQAIISRMELPKKPVSTVISQQMETCKVDIQENHDDVYGVPSLEEL
GFPTDGLAPAVWQGGETEALARLDKHLERKAWVANYERPRMNANSLLASPTGLSPYLRFGCLSCRLFYYRLWE
LYKKVKRNSTPPLSLYGQLLWREFFYTAATNNPKFDRMEGNPICIQIPWDRNPEALAKWAEGKTGFPWIDAIM
TQLRQEGWIHHLARHAVACFLTRGDLWISWESGVRVFDELLLDADFSVNAGSWMWLSCSAFFQQFFHCYCPVG
FGRRTDPSGDYVKRYLPKLKGFPSRYIYEPWNAPESVQKAAKCIIGVDYPKPMVNHAETSRLNIERMKQIYQQ
LSRYRGLCLLASVPSCVEDLSGPVADSASGQGCSTSTAMRLSQADQASPKRKHEGTEELCTEDLYKRPKVTGL
PATEVPGKSL
>Ramphastos_sulf__NXP79048.1
VDQERSHQGLAQLFLLFSSPVLKVDYDVCSNYGNWLYSAGIGNDPRDNRKFNMIKQGLDYDGNGDYVRLWVPE
LQGIKGADIHTPWVLSSADLSQAGVTLGETYPQPVVTAPEWSRHISRRPQGRSPHPRGRRGPTHTSVQHKDRG
IDFYFSGKKDV
>Rhamdia_quel__QGW67268.1
MVVNAVHWFRKGLRLHDNPSLLECIQGADTLRCIYILDPWFAGSSNVGINRWRFLLQCLEDLDANLHKLNSRL
YVIRGQPTDVFPRLFEEWKITHLSYEFDSEPFGKERDAAIKKLANEAGVEVTVGISHTLYDPEKIIEMNGGQS

PLTYKRFQMVISKMDTVEMPVETITAEILSKCIMPISDDHDERFGVPSLEELGFETEGLPSAVWPGGESEALT
RLERHLERKAWVANFERPRMNANSFLASPTGLSPYLRFGCLSSRLFYFKLTDLYQKVKKNSAPPLSLYGQLLW
REFFYTAATNRPCFDKMEGNPICVQIPWDRNPEALAKWAEGRTGFPWIDAIMTQLRQEGWIHHLARHAVACFL
TRGDLWISWEEGVKVFEELLLDADWSVNAGSWMWLSCSSFFQQFFHCYCPVGFGRRTDPNGDYIRRYLPVLRS
FPAKYIYDPWNAPESVQKLAKCIIGTHYPKPMANHAEASRINIERMKQIYQQLSCYRGLSLLASVPSNANGN
>Rhamdia_quel__QGW67269.1
MVVNSVHWFRKGLRLHDNPALQEALNGADTVRCVYVLDPWFAGAANVGFNRWRFLLESLEDLDTSLRKLNSRL
FVVRGQPADVFPRLFKEWNVTRLTFEYDSEPYGKERDSTIIKMAQEFGVETIVRHSHTLYNPDRIIEMNNNNP
PLTFKRFQAIVSRLELPKKPLPTITPEQIASCSTQLSENHDKHYGVPSLVELGFKTQGQSAPVWKGGETEALE
RLNKHLDKKAWVANFERPRIKAHSLFASPTGLSPYLCFGCLSARLLYNHLREFYMKLRRRSSPPLSLFRQLLW
REFFYTAATNNPSFDRMEGNSICVQIPWDQNPEALAKWAEGRTGFPWIDAIMCQLRQEGWIHHLARHAVACFL
TRGDLWISWESGMKVFEELLLDADWSVXXXXXXXLSCSAFFQQFFHCYCPVGFGRRIDPSGDYIRRYIPELKD
YPSRYIYEPWNTPESIQKAANCIVGVDYPKPMLNHAESSRLNIERMKQVYQQLSHYRGLSLLASVPTIQEEVE
PSMSDDSQASSSGVISHHPQSQSEPQSSKTDPDPNPDQDPDPEPSASSAPAAMRKRGCSSEPQNFQNPKVQHT
SQSKAKQLHADGKQ
>Rhinatrema_bivittatum_XP_029438108.1_cryptochro
MASGRNESPKALPLAVNSVHWFRKGLRLHDNPALQAALRGAQTLRCIYILDPWFAASSAVGIMRWRFLLQSLE
DLDNSLRKLNSRLFVVRGQPTDVFPRLFKAWSVTRLTFEYDSEPFGKERDAAIIKMAKESGVKVIVENSHTLY
DLDRIIELNDQKPPLTYKRFQAIISRMELPRQPVCSITEPQMETCKAEIQQNHDETYGVPSLEELGFCTEGLG
PAVWRGGETEALARLDKHLERKAWVANYERPRMNANSLLASPTGLSPYLRFGCLSCRLFYYRLRDLYKKVKRS
SLPPLSLYGQLLWREFFYTAATNNSKFDRMEGNPICVQIPWDKNPEALAKWAEGKTGFPWIDAIMTQLRQEGW
IHHLARHAVACFLTRGDLWNSWESGVRVFDELLLDADFSVNAGSWMWLSCSAFFQQFFHCYCPVGFGRRTDPS
GDYVRRYLPKLKAFPSRYIYEPWNAPEALQKAAKCIIGVDYPKPVVNHAEASRLNIERMKQVYQQLSHYRGLC
MLASVPSSSQELSNPLAGSASGQGGTIGAAPRLPHGAQTSPRRKDEGTEDLKSEDRFKRTKVTNLPIPEFPAK
DL
>Rhinatrema_biv_XP_029455619.1_cry-1
MGVNAVHWFRKGLRLHDNPALRECIQGADSVRCVYILDPWFAGSSNVGINRWRFLLQCLEDLDANLRKLNSRL
FVIRGQPADVFPRLFKEWKITKLSIEYDSEPFGKERDAAIKKLASEAGVEVIVQISHTLYDSDKIIELNGGQP
PLTYKRFQTLISRMEPLEMPVETITSEVMETCSTPVSEDHDEKYGVPSLEELGFDTEGLPSAVWPGGETEALT
RLERHLERKAWVANFERPRMNANSLLASPTGLSPYLRFGCLSCRLFYFKLTDLYKKVKKNSSPPLSLYGQLLW
REFFYTAATSNPRFDKMEGNPICVQIPWDKNPEALAKWAEGRTGFPWIDAIMTQLRQEGWIHHLARHAVACFL
TRGDLWISWEEGMKVFEELLLDADWSVNAGSWMWLSCSSFFQQFFHCYCPVGFGRRTDPNGDYIRRYLPVLRG
FPAKYIYDPWNAPDSVQKGAKCIIGINYPKPMVNHAEASRLNIERMKQIYQQLSRYRGLGLLASVPSNPNGNG
NGGLMSYSPGENTTTGTQIGATDGHTIGIQMLSQIEPSMGSNGIQHQGRSALSTGVINGKRPNPEEETQSIGP
KVQRQSAS
>Rhinatrema_biv_XP_029472371.1_LOW_QUALIT
MSRMATTPQHIADDLGAEVPIMTSSRKRHMEETLRGSYAELASVVDTKLMRPNESSGGGNLLQAVQRTRLRAG
SSVLEFAFNKKRVRLISSNWDVKDDSQGIIYWMSRDQRVQDNWAFLYAQRLALKQNIPLHVCFCLEPTFLDST
IRQFGFMLKGLQEVVEECKELNIPFHLLIGYAKDVLPQFIIEHDIGGLVTDFSPLRVPLQWVQDVRENLPRRV
PFVQVDAHNIVPCWVASNKQEYAARTIRKKIHEQFPWFLTEFPALSKHPYNSKFTAMPVDWDACCASLQVDRS
VREVDWATPGTAAGLSVLQSFIGERLKFFCTDRNDPNKAALSNLSPWFHFGQVSVQRAVLEVKKYRSKFRDSV
DGFVEEAVIRRELADNFCFYNKNYDKVKGAYDWARKSLRDHVKDKRTHIYTLEQLEAGKTHDPLWNAAQLQMV
QEGKMHGFLRMYWAKKILEWTSTPEIALKYSIYLNDRFELDGRDPNGYVGCMWSICGIHDQGWAERDVFGKIR
FMNYAGCKRKFDVEQFERRYNSKKFTF
>Rhincodon_typ_XP_020390164.1_cry-1-like
METHSIHWFRKGLRLHDNPALKEALRGASTVRCVYILDPWFAGSSNVGVNRWRFLLQCLEDLDASLVELNSRL
FVIRGQPADVFPRLFKASSCELYAVSATSMHLIIELNGGQPPLTYKRFQTLISQMDSPEPPVETISERSLGKS
TILVLDDHNEKYGVPSLEELGFDTEGLPSAVWPGGETEALTRLERHLERKAWVANFEKPRMNALSLLASPTGL
SPYLRFGCLSCRLFYFKLTELYKRIKKTDSPPLSLYGQLLWREFFYTAATNNPCFDKMEGNPICVRIPWDKNP
VSLAKWAEGKTGYPWIDAIMTQLRQEGWIHHLARHAVACFLTRGDLWISWEEGMKVFEELLLDADWSVNAGSW
MWLSCSSFFQQFFHCYCPVGFGRRTDLNGDYVRRYLPVLKGFPAKYIYDPWNAPNSVQVAAKCIIGIHYPKPM
VIHAEASRLNIERMKQIYQQLSRYRGLGLLASVPSNQNGSGGTMYSLGDQITENNGIVQSHTNGAMVGKRELE
RDTAAGDDMESLAQKVRRQSDDNNEDEQNLNLVSY
>Rhincodon_typ_XP_020392441.1_LOW_QUALIT
MSKNDKQAHETTRCNANDSEYKKNIEKKSDKKKTAFSSLSNSLKTSTKVSMKKPNHNGSCREDSPKLQSKGKR
KVEDRAPRLIEGEMSNPRMNKKRKIEDQAEASPSRERNLLESINKSRLSTAKSVEEFNFNKKRIRLLSKTESI
RENSQGIIYWMSRDQRVQDNWAFLYAQWLALKHKLPLHVCFCLVPKFLDATIRHFGFMLKGLHEVAEECKQLN
ISFHLLIGFAKDVLPDFVKEHNIGGMVTDFSPLRLPLEWIENVRYNLPEDIPFIQVDAHNVVPCWVASDKQEY
SARTIRKKIHDKLPQFLTDFPPVIQHPFSSSLKAELIDWDAAAASLQVDRTVKEVDWAKPGTAAGLTRMESFI
NEQLKHFNTDRNNPSKTALSNMSPWFHFGQISVQRAVLEIRKYRNKFKDSVDSYIEEAVVRRELADNFCFYNK

QYDKIAGASDWAQKTLQMHSKDKRPYVYTLKELEDGRTHDSLWNAAQLQMIHEGKMHGFLRMYWAKKILEWTA
SPEEALQFSIYLNDRYELDGXDPNGYV
>Rhinopomastus_cyan__NXN91581.1
DNWAFLYAQRLALKQELPLRVCFCLVPKFLDATIRHYRFMLKGLQEVAKECAELNIPFHLLLGYARDVLPAFV
VKHGVGGLVTDFCPLRLPRQWVEEVKMQLPEDVPFAQVDAHNIVPCWVASPKQEYSAQTIRGKIHTQLPEFLT
EFPPVVRHPHSPSCPAEPIAWEACYSSLQVDHTVGEVAWATPGTAAGLAMLRSFIAERLKSFSSHRNNPNKAA
LSNLSPWFHFGQVSTQRAILEVQKHRGRYKESVEAFVEEAVVRRELAENFCYYNENYDSVQGAYSWAQTTLKL
HAKDKRPFLYELQELEQATTHDPLWNAAQLQMVREGKMHGFLRMYWAKKILEWTRTPEEALQFAIYLNDRYEL
DGRDPNGYVGKRCLWSICGIHDHGWTERAVFGKIRYMNYAGCKRKFDVGQFERRY
>Rhinopomastus_cyan__NXN98048.1
MPHRTIHLFRKELRLHDNPTLLAALESSEVIYPVYILDRKFLTSTMHMGTLRWHFLLQSLGDLRKNLSQLGSC
LLVIQGEYESVLRDHVRQWDITQVTLDAEMEPFYKEMEANIRRLGEELGFEVLSLVGHSLYDTKRILDLNGGT
PPLTYKRFIHILSLLGDPMVPVRNPTAEDFQSCSPPEPGLAERYAVPLPVDLKLPPESLSPWKGGETEGLQRL
EQHLTDQGWVTSFTKPRTIPNSLLPSTTGLSPYFSLGCVSVRTFFYRLSNIYAQAKHHSLPPVSLQGQLLWRE
FFYTVASATPNFTQMAGNPICLQISWYEDAEKLHKWKMAQTGFPWIDAIMTQLRQEGWIHHLARHAVACFLTR
GDLWISWEEGMKVFEELLLDADYSINAGNWMWLSASAFFHQYTRIFCPVRFGKRTDPEGQYIRKYLPILKNFD
SKYIYEPWTASEEEQKQAGCIIGQDYPFPMVDHKKASDHNLQLMKQVREEQYRTAQLTRDDADDPMEMKVKHP
IAEEKVPKVKAPRMAE
>Rhinopomastus_cyan__NXN99823.1
LQEWGVTRLTFEYDSEPFGKERDAAIIKLAKEAGVEVVIENSHTLYDLDRIIELNGHKPPLTYKRFQAIISRM
ELPKKPVSTVISQQMETCKVDIQENHDDLYGVPSLEELGFPTDGLAPAVWQGGETEALARLDKHLERKAWVAN
YERPRMNANSLLASPTGLSPYLRFGCLSCRLFYYRLWELYKKVKRNSTPPLSLYGQLLWREFFYTAATNNPKF
DRMEGNPICIQIPWDRNPEALAKWAEGKTGFPWIDAIMTQLRQEGWIHHLARHAVACFLTRGDLWISWESGVR
VFDELLLDADFSVNAGSWMWLSCSAFFQQFFHCYCPVGFGRRTDPSGDYVKRYLPKLKGFPSRYIYEPWNAPE
SVQKAAKCIIGVDYPKPMVNHAETSRLNIERMKQIYQQLSRYRGLCLLASVPSCVEDLTGPVTDASSGQGCST
STAVRLLQADQASPKRKHEGAEEPCAEELHKRPKVTGLPAPEIPGKN
>Rhinopomastus_cyan__NXO01642.1
MGVNAVHWFRKGLRLHDNPALRECIQGADTVRCVYILDPWFAGSSNVGINRWRFLLQCLEDLDANLRKLNSRL
FVIRGQPADVFPRLFKEWNIAKLSIEYDSEPFGKERDAAIKKLASEAGVEVIVRISHTLYDLDKIIELNGGQP
PLTYKRFQTLISRMEPLEMPVETITPEVMEKCTTPVSDDHDEKYGVPSLEELGFDTDGLPSAVWPGGETEALT
RLERHLERKAWVANFERPRMNANSLLASPTGLSPYLRFGCLSCRLFYFKLTDLYKKVKKNSSPPLSLYGQLLW
REFFYTAATNNPRFDKMEGNPICVQIPWDKNPEALAKWAEGRTGFPWIDAIMTQLRQEGWIHHLARHAVACFL
TRGDLWISWEEGMKVFEELLLDADWSVNAGSWMWLSCSSFFQQFFHCYCPVGFGRRTDPNGDYIRRYLPVLRG
FPAKYIYDPWNAPESVQKAAKCIIGVNYPKPMVNHAEASRLNIERMKQIYQQLSRYRGLGLLATVPSNPNGNG
NGGLMGYSPGESISGCGSTGGAQLGTGDGHTVVQPCALGDSHTGASGIQQQGYCQASSILHYAHGDNQQSHLL
QAGRAALGTGVSAGKRPSPDEETQSVGPKVQRQSTN
>Rhizoclosmatium_glo_ORY43053.1_putative_p
MKRAGSPTATNKSDAKKAKTSTESRCFVHWFRNDLRLADNTSLSWVVQEASRASKPVVCLFVISAAEWNQHEM
APAKVRWLLASLESLKENLKERGIPLVVRVSESRRGVPKVVVDTVSELGAEAVGWNWEYEVHEEWRDHTVMDS
LEKVGIKHNGMHDQCVIDTSRVRTKEGKPYTVFTPFKKTWIQTIVKNAHLVKLRDAPTENTAPISDTLAAKIE
TLSTAIPGPSDPLPAPYTESDNPPASVTEFINKTFAPSEKAAATRLATFIESRGKSYKDTRDFPGKDLTSKLS
PYLSLGLISPRVCLDAAVKANNGKYDSGNEGLVTWISEICWRDFYRNILIEFPRVCKNLPFKPETDNVEWNYS
RDNPLYIAWCEGKTGFPIVDAGMRQLATEGWMHNRVRMIVSSFLTKDLGVDWRLGESWFMRNLIDGDLASNNG
GWQWSASTGTDSQPYFRIFNPLLQSERFDPDGHYIRKYVKELSAVKGKEIHDPKGRLGSAYFAKLGYPKPIVD
HKTASQAFLAAFKKGIGK
>Rhizoclosmatium_ORY50797.1
MPVAVHWFRKALRLHDNPALAKAVAACSAPNSSLLPVYTLDPHFVKNSHIGTNRWLFFLESLQDVDRSLSESG
SKLLVLRGEPMTLFSTLFDTLIANGETVSLYFEADTSPYARNRDAAMLELCASKNIAVHTFNSHTLFNPSDVI
KVNGGITPIVYQSFLKVITEKMPAIQPATPTLTQVPPLSDEMKALLTYTETFGFDAFQVPSMEDIGKAVPGEN
LVSTHKGGEREALKRLEEYMKQKDLVCKFQKPETNPAAFQPASTTILSPYLKMGSLSARTFYHELMKVYRASK
IGYTKPPVSLLGQLYWREFYYTAGYGTPNFDKMLGNPICKQIPWRTDEQSNVDLEKWATGQTGFPWIDAIMIQ
LKQEGWIHHLARHSVACFLTRGDLYISWERGAKVFEKLLIDADWSLNTGNWLWLSASAFFHQYFRVYGPVSFP
KKYAPESVKYVRKYIPALKNFPEKYLFEPWKCPIADQKKAGCIIGKDYPKPMCDHDAVSKVNIGKMKVAYDEG
KVASPPKAPKGKKRPLDED
>Rhizoclosmatium_ORY50805.1
MTSCVLFRLTDLRLHDNVTLARAIADATTNNKTLLPMFVWDTRFLGLAPTQQHQSLPSRIGTTWHFGFPRLSA
QRQRFLLESLVDLKSQLQKLKSDLVVIVPPHNVALSDYISAQIVPSLQNGSDGGVSVFMGADSPCYEERILER
ELSECTGFSLKLVKDNHTMLSMSAVEGRVPDIYTQFRSNIERSASAKSDISFPAVALPLPSPTKLPSSPVNVV
MEHTLSLEDLESLLKSCPANKSQPSVSFPFKGGETAALARLKAYMSGPIAKYKETRNGLLGSDYSTKFSPYLA
WGCLSARTIIHAMRPLTAKAQDGTYWAWFELLWRDYFYFVARDYGNMLFYPTGLTDTPVKAGYTHAPTNPKFV

AWCEGRTGVPFVDANMRELKATGFMSNRGRQNVASFLVKDLGVDWRLGAEWFEAMLLDHDPCSNYGNWLYVAG
WGNDPRQATEGDGRYFNVLKQARDYDPKAEYVLAWCPELHGHPNPHAPWTRTGVFGSDNLYARRPICMGKGWD
RHLEVRAESGKKEGNGRHRVPKKF
>Rhizoctonia_sol_CAE6419686.1_unnamed_pr
MARRIAIALLRNDLRVSDNPILHAARNASGITHLLPLFVFDDRQVELSGVVGYREAQHGGDEPLPIAKTRICG
FWRTGRHRVSLASPRFLVQSVFDLKSQLKSAGSDLGVYLGRPENVVPHLIHQLQKQGDTIEGVWLQRESASEE
VSVERQLSRALQGMRTTLHLDSGARTLVHESDLPFQLPSQLPDVFTVFRKRVEGLNENMVRPVLPYPSKGEWK
PYPTLKDYTKDTSSRSIFALPNDMTEDHFIEQLTAPLVAEPVPGDTSHGTAYYEVPGAAFPFKGGESAARQRL
DYYLGIGGAEESCPATTYKETRNGLLGADYSTKFSPYLTFGCLSAREIAARCDDLEDRLREAGSLTDTARKNI
YWIKFELLWRDYMFFVNIKYGNNLFKPQGLLTGASPGKSEWTEWENRNDKLQAWFEGRTGVPFVDACMRELAS
TGYMSNRGRQNVASYLTKDMHFDWRIGAEFFESFLLDYDPASNYGNWAYVAGVGNDPRQGRKFNIIKQANDYD
PNGEYVKQWVPEVSGSELTGFQRHIPWRVWSGAEESRGSGSYPRSPIVEDSGWKRFYSTGDGKQGKHGERGSK
GGRGKGRGRKH
>Rhizoctonia_sol_CAE6534518.1_unnamed_pr
MASKRGRATSSNKPAKKQKTMTDFVKSNVKSKAASSEPEDAPASEIAYVQQVHGKIATAENAARVDTEPPLGR
LQNAISEHKDELRNISKGDSVMYWMRMEDMRIRDNRAFSAASNVAQDLGLPLVVLFIFSPDDYVSHDRSPRRI
DFCLRNLAVLKEKLNEMNIPLHVTSHTPRKTLPEEVIKLARKWNARRIYANIEYQVDELRRDMRLVHLGTMAS
IRTEYFSDRCVVEPGALATKEGKQYAVYSPWLKNWVATLDRDPSRLEESPMPKPNDEKVRFECHDRETMIKLY
PAGEDAAHEVLERFLHTKSRTSQLADASPLAPDAETSSNHSRIKDYMSDRDRADADTTSRISPYLASGVISAR
ECVRSAMNILGVKRVDAGKTNGPGVWVSEIAWRDFYTHVMAAFPRVSMGRPFHEKFADVKWETNAAHLKAWKE
GKTGVPIVDAALRQANTQGWMHNRTRMIAAMYLTKDLMLDWRLGEKHFMEQFIDGDLASNNGGWQWSASTGTD
PQPYFRIFNPYSQSEKADPSGNYIKYFVPELKDLSGKALHDPYGHLSPSEFKKLGYPKPLVDHKEARERALRR
YKNPGEE
>Rhizoctonia_sol_CEL59601.1_cry-2_OS=G
MTPRPRVLHWFRTDLRVHDAPALQAGLALKPEVWYPVWCWDPEYVYSHRVGVNRFNFLIESMNDLSQSLTRIN
DKSKLLVVRGSPYTVLPKLFRDWKITHLVYELDTGGYAQERDKRVQEIATQNKVQVVDVLGHSLYHPAEVIEK
NGGKPTTTITQWQKAASKLGSVPRPTAAPKKLIPPGDLHLGSLSREDHPVNREHDLNEKTRISPVTCFDTLTG
PKGDFAVPTLEELGYPNPTTTIRGGEKEGRARLFAFNKDDNGNRAALFEKPKTSPTIPGPFKASRTNNGHEDT
LQEDGDEFEDLPEGAQIDVTKPSTTLMSPYLKFGCVGVRECYWSWHDILVNAPKSAKKPENLLGQLEFRDMYY
AAELAVGGTAFARVQGNPICRYIPWRCPTVYSDDTNQIIPRPKDEQHPRAEEWLAAWTEGRTGFPWIDALMRQ
LREEGWMHHLGRHSVACFLTRGQCYISWERGAEVFDEYLIDWDPASNYGNWMWLSCSAFFSQFMRVYGVATWP
SKFDKTGALVRKYIPELQNFPNQYIYEPWLAPKSVQKEAGCIIGKDYPSPMLDEKEEKGRNIERMKAAYQAGL
YGDSPKVLDGTATIEVPNKPEDDDSNKAGTKRKADKSGGSKTRSTRTRKVSDDGQKSLDAYMKKK
>Rhynchosporium_agr_CZS96539.1_related_to
MSDSKVLIYLMRRDLRVSDNPVLNSLVTSKKHGFTHLLPLYVLSAQQLETSGFIADSNTKSPYREARSKVGGF
WRTGPHRVTFLAECIYDLKEGLEKVGSGLCLRVGTVGDVVDDMLRKFKEKGELKAGAVWMIGEEGVEEAGEES
QVKKACREADVEFKLWNDEKYLIDDRDVPFDNIDELSDVFTTYRKTVEPLRESPRKVLPTPTKGSLPPFPPTS
STPEQRSPFTIPNSLETLQSSLLKPLQAQDLVKNPPSYPKETKSAHPFLGGETQAHDRLNYLIKSGNVTNYKD
TRNGLLGHDFSTKFSAYLALGCITARQIHSNLLAFEDGTNDTFKGGNGYGKGENAGTKSIRFELLWRDYMRLC
TRKFGPKLFRLSGFKAEGEEKWSTPAKPAPGVSSQQVRDQIERFLNGTTGMGLIDASMREMYHTGYTSNRARQ
NVASFLTKHLKIDWRIGAEWYESMLVDYDLSSNWGNWQYVAGVGNDPRGDNRIFNPVKQAFDYDPEGDYIKAW
VPELQKLTEPAEVFQAWTVREDVRKEKGLQGLNWVENPLLRINFTVNKRGRNGGKRGGGGGRGEYGHGHGGGR
FNEQDGRRYYANNNRNGGGESGRGGGGYGGGGGYAGRGYGSSRGHLKGNGYGSGRGGGGREFRTGMMDKVTEY
EQSTQ
>Rhynchosporium_agr_CZS99641.1_related_to
MAKPRVIYWFRTDLRLHDSPALKAALDLNAEAFWPVWTWDPHYVYRARVGTNRWQFLIDCQNDVSKNITKINN
NSKLFLMREAPQTLLPKLFKAWRITHLVFEKDTDAYARERDAAIMEAAREAGVEVVVRSGRTLWDSDELVKKN
HGKPTMSISQVQAAGPKAGPIPRPIPSPKSIPDPGETSVDFEQHQPEQAPDFNSKYRNQNDKSYESIAGPKGD
FAVPTLEELGFPAATTSHRGGENNALKVLDELIANEKYTATFEKPNTAPTQFLPQSTTLLSPHLHFGSLSIRE
FYWRVQDVVDNFSGKSSQPPVSLTGQLLFRDMYFGAQAKLGYAFGQTMYNSHCRFIPWHLPSKVDHETKLVTG
EYHIDSPQAEEWFQRWKYGMTGFPWIDALMRQLRQEGWMHHLGRHAVACFLTRGGCYISWERGAEVFEELLID
HETACNAGNWQWLSCTAFFAQFFRCYSPIAFPQKWDKQGEFVRRYVPELAKLDKKYIYEPWKAPIADQKKAGV
SVKGDGRSKEVGVYPKPMFDFAERRTICLDGMKNAYKVAMYGDHEKVIDGSWREMFGDGAEGPTEGKSFVDAM
SGFGRKIRDGDDVDVENDGAVAEDERGAAEKGGESKGGDERKTGTKRKAAQATLDGIVKRAKRK
>Rhynchosporium_agr_CZT06608.1_probable_p
MLSKKAATNGKRKASPPVEPAPKKSKITSDSTDPLRQPHETAQEAEDNGIVLREFYPKEMNNARAKAYNSDQL
PRPIELLNNALKDTKAARQKILAKDAVVHWFKCDLRTMDNKSLHLASEKAKEKGVPLIAMYIVSPQDFQAHLT
SPVRVDFILRTLEVMKKDLAIMDIPLYVETVEKRRNVQGRILELLEEWGANHLFTNVEYEVDELRREARMVRA
CLEKGIAMDVVPDTCVVSPGELSSGTGKQYSVYSPWFRSWTAYVHSNPKVLDLFNAPDKNPPTAREKFAKLFE
QSIPDAPANKTLTDEEKKRFRSMWPAGEAEAHERLRKFCDDRIGKYGDKRNFPAQGATSSLSVHFASGTLSAR

TAIKTARDHNTTKKLDAGNSGIMTWVSEVAWRDFYKHVLAHWPYVCMNKPFKPEYTNITWEYNQEHFKAWTEG
KTGYPIVDAAMRQLNHCGYMHNRCRMIVGSFLAKHLLLDWRMGERYFMEHLIDGDFASNNGGWGFCASTGVDP
QPYFRIFNPLLQSEKFDLEGEYIRKWVPELEGVKGKAIHDPYSRGEGVRAKKMGYPQRIVDHKACRERALARY
KEGLGRDTA
>Rhyparobia_mad_AGA01579.1_cry_2
MSLEKSPKHTVHWFRKGLRLHDNPSLREGLKCAKTFRCVFILDPWFAGSSNVGINKWRFLLQCLEDLDRSLRK
LNSRLFVIRGQPADALPKLFKEWGTTNLTFEEDPEPFGRVRDQNIMAMCKELGISVITRVSHTLYRLENIIEN
NGGKAPLTYHQFQTVIASMGPPPPAEPNISPKFMNGAYTPIGDDHDDKYGVPTLEELGFDIEGLLPPVWQGGE
PEALARLERHLERKAWVASFGRPKMTPQSLLASQTGLSPYLRFGCLSTRLFYYQLTDLYEKIKKACPPLSLHG
QLLWREFFYCAATRNPNFDKMMGNPICVQIPWDKNAEALAKWANGQTGFPWIDAIMTQLREEGWIHHLARHAV
ACFLTRGDLWISWEEGMKVFEELLLDADWSVNAGMWMWLSCPSFFQQFFHCYCPVRFGRKADPNGDYIRRYLP
VLKNFPTRYIHEPWNAPESVPKAAKCIIGKEYSLPMVNHAVASRINIERMKQVYQQLSKYRGPGLLATVPTSQ
PQSANIMNQFSLNRGFKKSPNLEQKWEKSPTDSVVNGLSPSYGEDSVNGIQLHRYHMQNKQSQPQHQKQDIEE
CE
>Rodentolepis_nan_VDO07365.1_unnamed_pr
MCINISGHISPQRCLWEAGFLQNQHKESVDAFIEEAFIRRELADNFCFYNKHYDSLKGKYLCHITYFRAWSWA
QETLRKHSNDIRQPSYSEEKMESASTGDELWNAAQRQLLWEGKMHGFLRMYWAKKILEWHAGGPEKALKLGIY
LNDKYSIDGTDPNGYVGVMWSICGIHDQGWMERPVFGKIRFMNFTGCKRKFDVAAFIKKYSPPINKHLKA
>Rodentolepis_nan_VDO14864.1_unnamed_pr
MRVLHWFRKGLRLHDNPALLATINPPKKATEETIEILLLYVFDTSGKEYGLRGHRQAQFLLESLIDLDQSLQK
LGKNLRLLVVEGDPIKEVPAICKSLDIKYVTFETDIEPYSIARDNEVTKVLEEEGVTVSRIHGHTMWDPEFLK
SHHHPPPTATKDSNSKDLGVPMTYKSFCALVRRVSPQGPPKPLPSFKEYAKSCIAPNLPELKLDKVHIGPPSS
LEQLRIKFDPIPEIGDHVSDNEGEKFELNFPGGEIEGLNRLQRMVAQRADWVCSFSKPETAPTSLPISTTGLS
PYIRFGCVSARHAWWVIAEAMRPSSKASKTKPPTQPPVSLHGQLLWREFFNLVASVTPSFDQIEGNPICRPIE
WNVDETLLSAWREARTGFPFIDAAMIQLHRHGWMHHLARHSVACFLTRGQLWQSWTAGADVFSTLLLDADWVL
NSANWMWLSASAFFHQYYRVYCPVSFGRKTDQDGDFIRHFIPALRNMPSEYIYDPWTAPISVQKKAGCRDKSL
ETTIFIYCDTPGPGFRACPSHSERIDKLMRFYC
>Rotaria_soci__CAF3317211.1
MSKRKESMPMGKKKRVKRTSESDCNTTEKEDEFEEEEQQQQQQQKDELASKITNTKANTTVIDYFTIKPLQSA
IDHIRKSICSSITEFKFNKTRVRPINKINEIPQDSQTILYWMSRDQRLQDNWAMLYAQRLAIKQQLPLHVAFC
LVPTFLAASLRAYRFMLKGLREVEKECQQLNIIFHLLLGKAENVLPKFVEDNKVGTIITDFSPLRVPRQWVDN
VGKKITKIPVVQVDAHNVVPCWHASPKLEYGARTIRTKLHKHMNEFFTEYPPVIKHPHTSTTHSEMKTIDWET
VDDSLQVDRTVNEVSWATPGYTGGIAQLESFINERIMSFGTSRNDPTKNVLSNLSPWLHYGQISPQRALLIVA
KLRSKYKEACDEYIEETFVRRELSDNYCFYQDNYDNINGAWDWAKKTLNDHRKDKRTYEYSRDDLEYARTTDK
LWNAAQIQMIKEGKMHGFLRMYWAKKILEWTKTPEEAIEISIYLNDKYNLDGRDPNGYVGIMWSICGIHDQGW
RERPIFGKIRYMNYDGCKRKFDVKQFENKYSCL
>Rotaria_soci__CAF3324377.1
MKRLGIVWFRNDLRLHDNELLAWAHANNDYVNHMHCFDPRQITDKTYKCDFVKCDKYRLKFLIETIENLNTSL
ISNGSGLLTYRDTPENVFKQLIQQYKDKFEISIGFHQEVTQEETDVEKAIRQLARDNNVHVKEFWTTTLYHPD
DLPYNNPKAFPDVFTQFRVALEKQNVRPRSLTNIPDKFKPLPDGSIVTFIPALADYGYSNVTVHSSSVFPFTG
GESSALAHLHSYIWEKNLAKSYKQTRNSLTGCENSTKFSPWLANGSLSPRKICVELKKYENEQGAADAGYWII
FELLWRDFFKFTALKYGARLFYGRGLKSNPYDWKHDLQQFEAWRTGTTGVPFVDANMREFMNTGWMSNRGRQN
VASFLTKDLSIDWRYGAVWFESILIDHDVCSNYGNWVYVAGIGNDPRENRHFNMIKQAFDYDSSGEFVRTWCP
ELARLPNEYIHTPWLAPSHILKDAAVELGVNYPRPIIVVPQWNQQSQNWRTSLQNQHHTQQRGIDFYFKKPEK
RH
>Rotaria_sord__CAF0871498.1
MSKRKETMSKVRKKREKKTNESDNNTNEKEDDIEEEEDEEEQKNNLAQKISNTKTNTNLIDYFTIKPLQLAID
HIRKNICLNILDFNFNKNRIRPINNINIIPQDSQIILYWMSRDQRIQDNWAMLYAQRLALKQQLPLHIVFCLV
PTFLAAPIRAYRFMLKGLHEIEKECQQLNIIFHLLIGQAENILPKFIENYKVGTIITDFSPLRISRQWLENVA
KKITTIPIVQVDAHNVVPCWHASTKLEYGARTIRNKLHKHMNEFFTEYPPIIKHPYTSSNDYQIKSIDWQAVD
DSLQVDRTIKEVSWAIPGYTGGINQLETFINERVMNFALRRNDPNKDVLSNLSPWFHYGQISPQRALLIIAKL
RPKFKDACDSFIEEAFVRRELSDNYCFYQENYDTINGAWEWAKKTLNDHRNDKRTHIYSRDDLEYARTYDKLW
NASQIQMIKEGKMHGFLRMYWAKKILEWTKTPEEALDIAIYLNDKYNLDGRDPNGYVGIMWSICGIHDQGWKE
RPIFGKIRYMNYEGCKRKFDVKQYENKYASL
>Rousettus_aegy__KAF6464657.1
MAATVGTAAAAPSAPAAGTDGASSVHWFRKGLRLHDNPALLAAVRGARCVRCVYILDPWFAASSSVGINRWRF
LLQSLEDLDTSLRKLNSRLFVVRGQPADVFPRLFKEWGVTRLTFEYDSEPFGKERDAAIMKMAKEAGVEVMTE
NSHTLYDLDRIIELNGQKPPLTYKRFQAIISRMELPKKPVGSVTSQQMESCRAEIQENHDETYGVPSLEELGF
PTEGLGPAVWQGGETEALARLDKHLERKAWVANYERPRMNANSLLASPTGLSPYLRFGCLSCRLFYYRLWDLY
KKVKRNSTPPLSLFGQLLWREFFYTAATNNPRFDRMEGNPICIQIPWDRNPEALAKWAEGKTGFPWIDAIMTQ

LRQEGWIHHLARHAVACFLTRGDLWVSWESGVRVFDELLLDADFSVNAGSWMWLSCSAFFQQFFHCYCPVGFG
RRTDPSGDYIRRYLPKLKGFPSRYIYEPWNAPESVQKAAKCIIGVDYPRPIVNHAETSRLNIERMKQIYQQLS
RYRGLCLLASVPSCVEDLNNPVAEPSSSQPGSMNNAGPRPLPSGPASPKRKLEAAEEPPGEELSKRARVAELP
TSELPSKDV
>Rousettus_aegy__XP_016003549.1
MGVNAVHWFRKGLRLHDNPALKECIQGADTIRCVYILDPWFAGSSNVGINRWRFLLQCLEDLDANLRKLNSRL
FVIRGQPADVFPRLFKEWNIAKLSIEYDSEPFGKERDAAIKKLATEAGVEVIVRISHTLYDLDKIIELNGGQP
PLTYKRFQTLISKMEPLEIPVETITSEVIEKCTTPLSDDHDEKYGVPSLEELGFDTDGLPSAVWPGGETEALT
RLERHLERKAWVANFERPRMNANSLLASPTGLSPYLRFGCLSCRLFYFKLTDLYKKVKKNSSPPLSLYGQLLW
REFFYTAATNNPRFDKMEGNPICVQIPWDKNPEALAKWAEGRTGFPWIDAIMTQLRQEGWIHHLARHAVACFL
TRGDLWISWEEGMKVFEELLLDADWSINAGSWMWLSCSSFFQQFFHCYCPVGFGRRTDPNGDYIRRYLPVLRG
FPAKYIYDPWNAPEGIQKVAKCLIGVNYPKPMVNHAEASRLNIERMKQIYQQLSRYRGLGLLASVPSNPNGNG
GLMGYSPGENIPGCSSSGSCSQGSGSLHYAHGDGQQTHMLKQGRSSMGTGLSSGKRPSQEEDMQSIGPKVQRQ
STN
>Rozella_all_EPZ35776.1_DNA_photol
MKRSLEKGEMVLKKVSQRALMWFKSDLRVTDNPALRNASLYGEVIALYIISPQEWKSHDVAPIKVDFILRNLK
KLRESLNELNIPLVVKQINHVKETPNLILNHCKEWNINAVFINKEYEVDESKRDLKVQKTLEENGFLFDITHD
QCVVPPGRVLTKDGKPYSVFTPFKKAWIKILLAENSLIQVIPLPEPNIDQSLIIHQEKNEIPEKVEGFDISKK
YDADYVRGLWAAGKEAAHKKLEQFSKFKILDYKKNRDAPALDGTSVISPFLAIGALGIRECLNEALIANKGLL
DSGNEGAVTWISELCWREFYRHVLFHYPRVCMYQPFKLETKNIKWRDDPEGFEKWCKGETGYPIVDAGMRQLN
KTGWMHNRSRMIAAMFLTKDLLIDWRKGEKYFMENLIDGDFASNNGGWQWSASTGTDSQPYFRVFNPLLQSQK
NDPDGVFIKKWVPELKGLNKKQIHCPKDCLGASEFKKLNYPLPLVDHDMARKRAIDAFKNLKTDEEPKSAY
>Rozella_all_RKP18286.1_ROZALSC1DR
MERHPSVKTVSYERDYDPYSIKRDKELEDFFNEADINVRTFGTHVLYEPTQVLEKNGGVAPLTYQGFLKVVNK
LGKPGFPIPPPTKLPGFFDLQKILNCNEGIEFAGPNKTFDVPAIQELGFETPLYRSPHHGGESVALQMLDKYF
LDKTKVLTFEKPNTSPAAFDPASTTVLSPYVKYGCLSVRLFYQRLMDIYRNQSHSKPPVSLEGQLFWREFYYT
AGYATPNFHRMEGNPICLQTNWRLKEKDDVDEEASKHLEAWRDGTTGFPWIDAIMNQLRQEGWIHHLARHSVA
CFLTRGDLYISWERGAEIFEYLLLDADYFLNFGNWLWLSASAFFHQYFRVYSPIAFPKKYDPEGNFVRKYVPQ
LSQFPKKYIYEPWKAPLEVQRKANCIIGKDYPKPIVEHDVIV
>Rynchops_nige__NXN49257.1
DNWAFLYAQRLALKQELPLHVCFCLVPKFLDATIRHYGFMLKGLQEVAEECAELNIPFHLLLGYAKDVLPAFV
VERGVGGLVTDFCPLRLPRQWVENVRERLPEDVPFAQVDAHNIVPCWVASPKQEYSARTIRGKIHAQLPEFLT
EFPPIIRHPYPPSCPPEPIAWEACYSSLQVDCTVKEVEWATPGTSAGLAVLQSFITERLKSFSSHRNDPNKAA
LSNLSPWFHFGQVSTQRAILEVQKHRGKYKESVDAFVEEAVVRRELAENFCYYNENYDSVQGAYDWAQTTLKL
HAKDKRPFLYKLQELEQGTTHDPLWNAAQLQMVREGKMHGFLRMYWAKKILEWTRSPEEALQFAIYLNDRYEL
DGRDPNGYVGKLQDGGMGFGGGCLWSICGIHDQGWAERAIFGKIRYMNYAGCKRKFDVDQFERRYAP
>Rynchops_nige__NXN49677.1
MLHRTIHLFRKGLRLHDNPTLLAALESSEVLYPVYILDRKFMTSVMHIGALRWHFLLQSLEDLQKNLCQLGSC
LLVMQGEYESVLRDHVQKWNITQVTLDAEMEPFYKEMEAKIRCLGEELGFEVLSLVGHSLYDTKRILDLNGGT
PPLTYKRFLHILSLLGDPEVPVRNLTAEDFQRCRSPDPGLAEGYRVPLPVDLKIPLESLSPWRGGETEGLQRL
EQHLTDQGWVASFTKPRTVPNSLLPSTTGLSPYISMGCLSVRTFFYRLSNIYAQAKHHSLPPVSLQGQLLWRE
FFYTVASATPNFTRMAGNPICLQISWYEDAERLHKWKTAQTGFPWIDAIMTQLRQEGWIHHLARHAVACFLTR
GDLWISWEEGMKVFEELLLDADYSINAGNWMWLSASAFFHQYTRIFCPVRFGKRTDPEGQYIRKYLPILKNFP
SKYIYEPWTASEEEQKQAGCIIGRDYPFPMVNHKEASDHNLQLMRQVREEQPRTAQLTRDDTDDPMEMKVKRD
RPEENVSKGKVARTAE
>Rynchops_nige__NXN52096.1
MQHSSIHWFRKGLRLHDNPALLAAATDCCRLHPLFILDPSSSRVGANAWRFLLDALRDLDGRLREMGSRLFVV
RGCPEEVFPRLFHAWGTTRLTFEVDTEPAARQRDAAVAELAAQHGVEVIREASHTLYDTERILALNDSKAPLT
YKRLQSLLASLGPPEKPAPALTQEHLRGCCTPCQVSHDIDYGVPTLEELGQDPTKVGPHLYPGGETAALARLD
MHMERTAWVCGFKKPETEPTSLSPSTTVLSPYLRFGCLSVRTFWWRLDEVYQGRKHSQPPVSLHGQLLWREFF
YTAGASIPNFDRMVGNPVCLQVNWDDNPQHLRAWREGQTGYPFIDAIMTQLRSEGWIHHLARHAVACFLTRGD
LWVSWEEGLKVFEELLLDADWSLNAGNWLWLSGSAFFHRYFHVYSPIAFGKKTDRDGAYIRKYLPILKDFPTE
YIYEPWKAPLAVQERAGCLVGTHYPRPIVEHRAASERNMGRMKAARAQR
>Rynchops_nige__NXN56413.1
CIYILDPWFAASSAVGINRWRFLLQSLEDLDNSLRKLNSRLFVVRGQPTDVFPRLFKEWGVTRLTFEYDSEPF
GKERDAAIIKLAKEAGVEVVIENSHTLYDLDRIIELNGHKPPLTYKRFQAIISRMELPKKPVSTVMTQQMETC
KVDIQENHDDVYGVPSLEELGFPTDGLAPAVWQGGETEALARLDKHLERKAWVANYERPRMNANSLLASPTGL
SPYLRFGCLSCRLFYYRLWELYKKVKRNSTPPLSLYGQLLWREFFYTAATNNPKFDRMEGNPICIQIPWDRNP
EALAKWAEGKTGFPWIDAIMTQLRQEGWIHHLARHAVACFLTRGDLWISWESGVRVFDELLLDADFSVNAGSW
MWLSCSAFFQQFFHCYCPVGFGRRTDPSGDYVKRYLPKLKGFPARYIYEPWNAPESVQKAAKCIIGVDYPKPM

VNHAETSRLNIERMKQIYQQLSRYRGLCLLASVPSCVEDLSGPVADSASGQGCSTGTAMRLSQADQASPKRKH
EGAEELCAEELYKRAKVTGLPAPEIPGKS
>Rynchops_nige__NXN57093.1
WFASKNKCFFTIPCRFLLQCLEDLDANLRKLNSRLFVIRGQPADVFPRLFKEWNIAKLSIEYDSEPFGKERDA
AIKKLASEAGVEVIVRISHTLYDLDKIIELNGGQPPLTYKRFQTLISRMEPLEMPVETITPEVMEKCTTPVSD
DHDEKYGVPSLEELGFDTDGLPSAVWPGGETEALTRLERHLERKACFKLLQAWVANFERPRMNANSLLASPTG
LSPYLRFGCLSCRLFYFKLTDLYKKVKKNSSPPLSLYGQLLWREFFYTAATNNPRFDKMEGNPICVQIPWDKN
PEALAKWAEGRTGFPWIDAIMTQLRQEGWIHHLARHAVACFLTRGDLWISWEEGMKVFEELLLDADWSVNAGS
WMWLSCSSFFQQFFHCYCPVGFGRRTDPNGDYIRRYLPVLRGFPAKYIYDPWNAPESIQKAAKCIIGVNYPKP
MVNHAEASRLNIERMKQIYQQLSRYRGLGLLATVPSNPNGNGNGGLMGYSPGETISGCGSTGGAQLGTGDGHT
VVQPCALGDSHTGASGVQQQGYCQASSILHYAHGDNQQSHLLQAGRTTLGTGISAGKRPNPEEETQSVGPKVQ
RQSTN
>Rynchops_nige__NXN57354.1
LLKVDYDVCSNYGNWLYSAGIGNDPRDNRKFNMVKQGLDYDSNGDYVRLWLPELQGIKGADIHTPWALNGAAL
SQAGVTLGETYPQPVVTAPEWSRHINQRPQGRSPHPRGRRGPAHTPTQHKDRGIDFYFSRKKDV
>Saccharomyces_cer_GAX67612.1_photolyase
MKRTVISSSNAYASKRSRLDIEHDFEQYHSLNKKYYPRPITKTGANQFNNKSRAKPMEIVEKLQKKQKTSFEN
VSTVMHWFRNDLRLYDNVGLYKSVALFQQLRQKNAKAKLYAVYVINEDDWRAHMDSGWKLMFIMGALKNLQQS
LAELHIPLLLWEFHTPKSTLSNSKEFVEFFKEKCMNVSSGTGTIITANIEYQTDELYRDIRLLENEDHRLQLK
YYHDSCIVAPGLITTDRGTNYSVFTPWYKKWVLYVNNNKKSTSKICHLHIIEPLKYNETFELKPFQYSLPDEF
LQYIPKSKWCLPDVSEEAALSRLKDFLGTKSSKYNNEKDMLYLGGTSGLSVYITTGRISTRLIVNQAFQSCNG
QIMSKALKDNSSTQNFIKEVAWRDFYRHCMCNWPYTSMGMPYRLDTLDIKWENNPVAFEKWCTGNTGIPIVDA
IMRKLLYTGYINNRSRMITASFLSKNLLIDWRWGERWFMKHLIDGDSSSNVGGWGFCSSTGIDAQPYFRVFNM
DIQAKKYDPQMIFVKQWVPELISSENKRPENYPKPLVDLKHSRKRALKVYKDAM
>Sagittarius_serp__NXQ94764.1
MQHSSIHWFRKGLRLHDNPALLAAAADCRCLHPLFILDPSSGRAGTNAWRFLLDALRDLDGTLQEMGSRLFVV
RGFPEEVFPHLFHAWGTTRLTFEVDTEPSALRRDAAVAELAARHGVEVIKEVSHTLYDTERVLALNDGKAPLT
YKHLQSLLASLGPPEKPAPVLTWEHLQGCCTPIQVSHDTDYGVPTLEELGQDPSKVGPHLYPGGETVALARLN
ALMDRTDWVCSFKKPETEPTSLSPSTTVLSPYLKFGCLSVRTFWWRLDEVYQGREHSQPPVSLHGQLLWREFF
YTAGAGIPNFDRMVGNPICLQVNWDDNPQHLRAWKEGQTGYPFIDAIMTQLRTEGWIHHLARHAVACFLTRGD
LWVSWEEGLKVFEELLLDADWSLNAGNWLWLSGSAFFHRYFHIYSPVAFGKKTDRDGAYIRKYLPILKDFPAE
YIYEPWKAPRAVQERAGCLVGTHYPQPIVEHGTVSKRNLGRMKVARTQK
>Sagittarius_serp__NXQ99261.1
RFLLQSLEDLDNSLRKLNSRLFVVRGQPTDVFPRLFKEWGVTRLTFEYDSEPFGKERDAAIVKLAKEAGVEVV
IENSHTLYDLDRIIELNGHKPPLTYKRFQAIISRMELPKKPVSTIISQQMETCKVDIQENHDDVYGVPSLEEL
GFPTDGLAPAVWQGGETEALARLDKHLERKAWVANYERPRMNANSLLASPTGLSPYLRFGCLSCRLFYYRLWE
LYKKVKRNSTPPLSLYGQLLWREFFYTAATNNPKFDRMEGNPICIQIPWDRNPEALAKWAEGKTGFPWIDAIM
TQLRQEGWIHHLARHAVACFLTRGDLWISWESGVRVFDELLLDADFSVNAGSWMWLSCSAFFQQFFHCYCPVG
FGRRTDPSGDYVKRYLPKLKGFPSRYIYEPWNAPESVQKAAKCIIGVDYPKPMVNHAETSRLNIERMKQIYQQ
LSRYRGLCLLASVPSCVEDLSGPVADSASGQGCSINTAMRLSQADQASPKRKHEGAEELCTEELYKRAKVTGL
PAPEIPGKS
>Sagittarius_serp__NXR00476.1
DNWAFLYAQRLALKQELPLHVCFCLVPKFLDATIRHYGFMLRGLQEVAEECAELNIPFHLLPGYAKDVLPAFV
VEHGVGGLVTDFCPLRLPRQWVEDVRERLPEDVPFAQVDAHNIVPCWVASPKQEYSARTIRGKIHAQLPEFLT
EFPPVIHHPYPPSCPAEPIAWEACYSSLQVDRTVKEVEWATPGTAAGLAVLQSFIAERLKSFGSHRNNPNKAA
LSNLSPWFHFGQVSTQRAILEVQKHRGKYKESVDTFVEEAVVRRELAENFCYYNENYDSVKGAYSWAQTTLKL
HAKDKRPFLYELQELEQGTTHDPLWNAAQLQMVWEGKMHGFLRMYWAKKILEWTRSPEEALQFAIYLNDRYEL
DGRDPNGYVGKRCLWSICGIHDHGWTERAIFGKIRYMNYAGCKRKFDVGQFERRY
>Sagittarius_serp__NXR00810.1
FVLKVDFDVCSNYGNWLYSAGIGNDPRDNRKFNMIKQGLDYDGNGDYIRLWVPELQGIQGADIHTPWALNSAA
LSQAGVTLGETYPQPVVTAPEWSRHINQKPQGRSPHPRGRRGPAHTPMQHKDRGIDFYFSRKKDV
>Sagittarius_serp__NXR01154.1
MPHRTIHLFRKGLRLHDNPTLLAALESSEVIYPVYILDRKFMTSVMHIGTLRWHFLLQSLEDLQRNLCQLGSC
LLVIQGEYESVLRDHIRKWNITQVTLDAEMEPFYKEMEANIQRLGEELGFEVLSLVGHSLYDTKRILDLNGGT
PPLTYKRFLHILSLLGDPAVPVRNLTAEDFQRCRPPEPGLAECYRVPLPVDLKIPSESLSPWRGGETEGLQRL
EQHLTDQGWVASFTKPRTIPNSLLPSTTGLSPYFSMGCLSVRTFFYRLSNIYAQAKHHSLPPVSLQGQLLWRE
FFYTVASATPNFTKMAGNPICLQISWYEDAERLHKWKTAQTGFPWIDAIMTQLRQEGWIHHLARHAVACFLTR
GDLWISWEEGMKVFEELLLDADYSINAGNWMWLSASAFFHQYTRIFCPVRFGKRTDPEGQYIRKYLPVLKNFP
SKYIYEPWTATEDEQKQAGCIIGQDYPFPMVNHKEASDHNLQLMKQVREEQYRTAQLTRDDTDDPMEMKVKRD
HSEENASKGKAARMTE

>Salarias_fasc__XP_029951737.1
MSPSVTSVQPVVRDMLREVLVGREDPEGFFALCVSLMGHQETRSQFPVIIRPLSTSNRGLHATLTSIYQEYFS
KTEEDELELALALSRLESDNQQLSTSSQESQVMWPGKRNSCVSYPQPVSEPKSIENKCTQLADGLGSGRNTCP
AQTSGQSNKSPSQTQNPKENKAGEDEQMTREGDLNESQKAKRSRRRQRQKGAGQQLVGLPSSPSASPPVLLW
FRRDLRLCDNPALIGSLELGAPVIPVFIWSPEEEEGPGITVAMGGACKYWLHQALSHFRTSLEGIGTRLVFFK
ANGESHEAGSSLRILKKLVKETGAQTVLANALYEPWLKERDDAVASALQKEGVECRLFHSYCLRDPYSVSTEG
VGLRGIGSVSHFMSCCKQNPGSPVGVPLDPPASLPTPAHWPQGVALDTLELARMPRRKDGTTIDWAANIRKSW
DFSEEGAHARLDAFLHDGVYRYEKESGRADAPNTSCLSPYLHFGQLSPRWLLWDAKGARCRPPKFQRKLAWRD
LAYWQLTLFPDLPWESLRPPYKALRWSSDRGHLKAWQRGRTGYPLVDAAMRQLWLTGWMNNYMRHVVASFLIA
YLHLPWQEGYRWFQDTLVDADVAIDAMMWQNGGMCGLDHWNFVMHPVDAAMTCDPNGSYVKKWCPELAGLPDE
LVHKPWKCPASMLRRAGVVFGQTYPERIILDLEERRSQSLQDVALVRKQFGEYVDKRSGCDLVPLPPRLVSEA
LGLSHRHGGVVTEGKEFLLPVITRMEFKHQQEDPDADAASNPYNAVLKGYVSRKRDETIAFLNERDFTASVMF
EGAQRRERLENNQRRMEGLPLLAAPRGRARRTPTAKDKFSIVPGGAVASLK
>Salarias_fasc__XP_029952200.1
MAHTCIHWFRKGLRLHDNPALLAALRDCKVLYPLFIVDPDCTYGGNNRKRFLLGALKDLDCSLRKLNSRLFVV
TGKPEEVFPKLFKEWNVTKLTYEYDTEPYSLNRDSKVTALAKEHGVEVFYKVSHTLYDMDRIIEENNGKTPLT
YKSFQGIVKRLGPPKKPIPAPVLGDMKDVKTPCSEKHDEKYGIPTLEDVGVDDTSLSEELFPGGEQEALRRLD
EHMSRTGWVCNFEKPQTSPNSLSPSTTVLSPYVAFGCLSARTFWWRLSDVYRGKKHSDPPVSLHGQLLWREFF
YTASVGIPNFNKMVGNHVCTQVDWDTNAEYLAAWREARTGFPFIDAIMTQLRREGWIHHLARHAVACFLTRGD
LWISWEEGQKVFEDYLLDHDWALNAGNWQWLSASTFFHQFYRVYSPVAFGKKTDKRGDYIKKYLPVLKKFPEQ
YIYEPWKAPLSVQRAAGCIVGQDYPRPIVEHEVISKTNIQRMKQAYAKSSQNKSESPIKKTASSESPVKNKGV
KRKMTSIADMLVKKKSKGEVS
>Salarias_fasc__XP_029952233.1
MVVNTIHWFRKGLRLHDNPALRDSIRGAESLRCIYILDPWFAGSSNVGINRWRFLLQCLEDLDASLRKINSRL
FVIRGQPTDVFPRLFKEWKIKRLSYEYDSEPFGKERDAAIQKLASEAGVEVMVRISHTLYNLDKIIELNGGQP
PLTYKRFQALINRMDPVELPVETITFEVIKRCLTPISDDHDDKFGVPSLEELGFETEGLSTAVWPGGETEALM
RLERHLERKAWVANFERPRMNANSLLASPTGLSPYLRFGCLSCRLFYFKLTDLYRKVKKNSTPPLSLYGQLLW
REFFYTTATNNPCFDKMDGNPVCVQIPWDRNPEALAKWAEGRTGFPWIDAVMTQLRQEGWIHHLARHAVACFL
TRGDLWISWEEGMKVFEELLLDADWSVNAGSWMWLSCSSFFQQFFHCYCPVGFGRRTDPNGDYIRRYLPILRG
FPAKYIYDPWTAPDEVQKTAKCIVGVHYPKPMVNHAEASRINIERMKQIYQQLSCYRGLGLLAMVPTNPNNTA
TGSSAAGGSAGASHGPGVSSEDSSMQEGTSRTERGHSVQKRRREETLPETSRKSWKQGK
>Salarias_fasc__XP_029952418.1
MVVNSVHWFRKCLRLHDNPALQEALNGADTVRCVYILDPWFAGAANVGINRWRFLLEALEDLDSSLKKLNSRL
FVVRGQPTDVFPRLIKEWNVTRLTFEYDPEPYGKERDGAIIKMAQEFGVETVIRNSHTLFNLDRIIEMNNNSP
PLTFKRFQTIVSRLELPRRPLPTITQQQMDTCRTDIAADHDKLYSIPSLEELGMHHRFGFRTRGLAAAVWRGG
ESEALDRLNKHLDKKVWVANLEHPRVNTCSLYASPTGLSPYLRFGCLSCRVLYYNLRELYMKLRKRSSPPLSL
FGQLLWREFFYTAATNNPNFDHMQGNPICVQIPWDQNPEALAKWAEGRTGFPWIDAIMTQLRQEGWIHHLARH
AVACFLTRGDLWLSWESGMKVFEELLLDADWSVNAGSWMWLSCSAFFQQFFHCYCPVGFGRRTDPTGDYIRRY
VPVLKDYPTRYIYEPWNAPECVQKAANCLVGVHYPKPMINHAEGSRLNIERMKQVYQQLSHYRGLSLLASVPT
IQEEAEPPMTDESQNGSGPDSPGAALADHQAPGCPAAPDSSSRPAPPPPACRPPPDAAAAAAPSCSFRHNVLL
PAPAPAAAASSRRKGFSRKPRRSYRPRAARAGGPPPPPPPPRDEQRRDRRREQRVEEKMEQEDEEEEEEEEPE
EHMEEEGEELRQ
>Salarias_fasc__XP_029955718.1
MSTSRTIICLLRNDLRLHDNELFHWAQRNAEYIVPLYCFDPRHYVGTYNFNLPKTGPFRLHFLLNSIKDLRNT
LRSKGSNLVVRRGKPEEVVADLIKQLGSVSSVAFHEEVTSEELNVEKRVKEVCARMKVKVHTCWGSTLYHRDD
LPFHHISRLPDVYTQFRKAVETQSRVRPVFPTPETLNPLPPGLEEGDIPTAADLEQPEPVADSRSAFPCSGGE
SPALERLKHYFWDTNLVASYKETRNGLIGVDYSTKFAPWLAMGCISPRYIYHQIQQYERERTANQSTYWVIFE
LLWRDYFKFVAVKYGNRLFQIKGLQDKHVPWKLDMKLFDAWKEGRTGVPFVDANMRELAMTGFMSNRGRQNVA
SFLTKDLGLDWRLGAEWFEYLLIDYDVCSNYGNWLYSAGIGNDPRENRKFNMIKQGLDYDNNGDYVRQWVPEL
QRIRGGDVHTPWALSSAALSHAEVSLGETYPAPVVVAPEWNRHFNKKPSGVGPSARGKKGPSHTPKQHRDRGI
DFYFSKSKNL
>Salarias_fasc__XP_029970321.1
MLRGATRSSSFALFLKTGSSSALRSLITPTMSDKKRKSTSSSSGEQPGAKQQKREKKERSRGWLRSELQQQRA
DNKDMKFNEKRLRFISEARKVKQGSEGVLYWMWRDQRVQDNWALIHAQRLAVEESLPLHICFCLTVPESPLST
LRHYSFLLKGLEEVSKECRPLDIQFHLLRGAPGEVLPGFVSDRGFGAVVTDFSPLREAVQWLQDVRKALPEDL
PLIQVDAHNVVPCWVASAKLEYAARTIRGKITKLLPEFLTDFPPVEKHPHTAARTAKAVDWAEMLASLPVDRT
VGEPEWAEPGAQAGLAMLESFMDERLPLFGAQRNDPNVAALSQLSPWIRFGQLSAQRAALQVQRGGKSAGQSV
GPFLEELVVRRELTDNFCFYNQQYDSVEGAYEWAQKTLKDHAKDKRPYLYTREQLENAKTHDKLWNAAQYQMT
SEGKMHGFLRMYWAKKILEWTPSPEEALSIALYLNDRYELDGQDPNGVVGCMWSICGVHDQGWAERPVFGKIR
YMNYKGCLRKFNVAQFERKYCPKTA

>Salmonella_ent_GAS74637.1_photolyase
MTTHLVWFRRDLRLHDNLALAAACRDRSAQVLALYIATPEQWKAHDMAPRQAAFTCAQLNALQTALAEKGIPL
LFHEVADFAASVDIVKSVCLQHDVSRLFYNYQYEINERQRDANVEKSLPQVVCEGFDDSVILPPGAVMTGNHE
MYKVFTPFKNAWLKRIKDGIPECVSAPKVRESGALDAPLTPITLSYPQQDFDAQRFVADEKLAIAQLRQFCQQ
AAGEYEQQRDFPAIDGTSRLSASLATGGLSPRQCLNRLLAEQPQALEGGSGSVWLNELIWREFYRHLMTYHPA
LCRYQPFIRWTDRVQWQNDPAHLQAWQMGQTGYPIVDAAMRQLNATGWMHNRLRMITASFLVKDLLIDWRKGE
QYFMSQLIDGDLAANNGGWQWAASTGTDAAPYFRIFNPTTQGEKFDRDGEFIRQWLPELRDVPGKTIHDPWSW
AEKAQVTLNYPRPIVDHKQARLATLAAYEAARKE
>Salpingoeca_ros_XP_004989008.1_cry_DASH
MSSSSSKAVRPAVVWFRGSDLRIHDHEPLLEAAKASKGHVVPVYCFDPRQVGPAAKTRCGQFPKCGWQRTRFI
VECVEDLRRNLQRLGSNLVVRVGEPEEVIPDIVSITGATEVFGQKEVCSEETGVEHRLAKRLSVPLTLRWGAI
TLCHRDDLPYERSCSDLPGVFSQFRKAAEACVPIRPPRPPPPSLQPLPQPIAGDPGHVPPVTQLSPNAPAKAD
ERGVLTFVGGETVARTRVKHYLWDTDCIATYKDTRNGLVGADYSSKFSPWLAHGCVSARWIHSEVKRYERERV
KNNSTYWLVFELLWRDYFRFVALQHGTAIFKEGGVQRKGIAWRHSPLDLSAWQEGRTGFPFVDANMRELAATG
FMSNRGRQNVASFLTKDLLIDWRLGAEWFESLLIDHDPCANYGNWNYAAGIGNDPRQGRHFNVIRQAKTYDPA
AEYVHLWVPELKHLPAPFAHTPYQAPHGELEAAGVALEKSYPRPIVASLDNGRIPIRGGAAKMYSAGKPFKGP
KWMDRDGQEAGSNTSDGGGRSRSGKRRGRHNRKPRVQGGWS
>Salpingoeca_ros_XP_004994459.1_DNA_photol
MGLPSGGEGFLMERVRAITPTQELREGGKCVVYWMSRDQRAKDNWALLYARSLARSARVPLVVVFSLVPKFLD
ATIRHYGFMLRGLHQTAKHLHEKLVPFHLLQGSAATTVPAFAAQHEAAAVICDMSPLRVPLRWVKDVGQALEA
QNVPLLQVDAHNIVPVWVTSQKQEYAARTIRPKIHKHLDTYLQPFPELDANDKDTLGDMELPPVFDLEAQFDM
LEVDTSVKEVDWIEPGYEQGMAAAEAFGRDRAKKFDELRNNPNEDVCSNLSPYFHFGQISAAAVVLLLKSKYS
KKAAKGVQTFIEEAVVRRELSDNFCFYNRRYDSIDGAAVWARDTLDTHRHDKREYVYTREQLEQGKTHDDLWN
AAQLQMVERGKMHGFLRMYWAKKILEWTATPEDALQTALFLNDRYELDGRDPNGYVGCMWSIAGIHDQGWAER
AVFGKIRYMNYKGCKRKFDVARFVSRFPQAVANAAEVNGQASSQPTLKQASSRSKRGAGGAGANTAKRAKKTS
A
>Salpingoeca_ros_XP_004995002.1_DNA_photol
MSGGAVSIHWFRKGLRLHDNAALLAALKGAKQVYPVFVLDPHFAKPEFVGVVRYNFLLESLRDLDKSLRTLGS
RLYVLKGKPLQALEEKFKEWEVTRLTFERDTEPYAKLRDTQARELAEKHGIEVITTVGHTLHDPEQYIAKAGG
ADKIPLTYSSFGTLFRRLGKVPAALSAPTKEDFPQSSTLVRTDALDDHRFDVPSLKDMGYEWNDDEHEVRFPG
GETEALERMRRHLQRKAWIAHFEKPKTSPNTLEPSTTGLSPYLKFGCLSPRLFYHELARVYAEYRDHAKPPVS
LHGQLLWREFFHMCGYAVKNFDRMEGNRICRQIDWDTNDALLAAWENARTGYPWIDACMTQLRREGWLHHLAR
HAVACFLTRGDLYQSWEKGAQVFDRLLVDADWHLNSANWMWLSCSSFFYQYFRVYSPVAFGKKTDPSGAYIRK
YLPQLKAFPDKYIYEPWKAPPGVQRSCGCIIGKDYPEPIVDHAEVSAANKARIRACYDALKQAEKAKKTTTKD
AKSSGGASKRSSAQSSAKLKKTKTASSKR
>Salvelinus_alpi__XP_023826479.1
MPVSGGEDPMSQVRQMLRELLVGRENAEGFFCLCVSVLGHNDTRTHFLPLIQLLATDHNSLHTTLTSIYLEYF
SKDEDDELAVALALSLLEVKGQQQTDTKPLFPDPKLQGQTDTQATATYRPQNSSIQSQSVSPNGSSSQPPPAP
LPKGASYAQLAAVGGRRQTQAQHQDSTSPRRSSGHGSSPQTERQRGPTQTDIMWSNVTQDVCASIATSLTSNL
NQAKDDITVVECEVDQSEKPKRSKNRRQRRKGYGQQVVGMPRCPSAPPPVLLWFRRDLRLHDNPAVIGSLAAG
GPVIPVFIWCPEEEEGPGVTVAMGGACKFWLHQALSCLSSALEHIGSHLVFLRTDEEREGIGSSLLALRSLVR
ETGAQTVLASALYEPWLRERDQVVVSALQKDRVEVNMVHSYCLRDPYTVTTEGVGLRGIGSVSHFMSCCQMNP
GPGLGVPLDPPISLPSPSVWPRGCPLEGLGLARMPCRKDGTTIDWAANIRSSWDFSEEGAQSRLEAFLNDGVY
RYEKESGRADAXNTSCLSPYLHFGQLSARWLLWDTKGARCRPPKFIRKLAWRDLAYWQLTLFPDLPWESLRPP
YKALRWSNERGHLKAWQKGRTGYPLVDAAMRQLWLTGWMNNYMRHVVASFLIAYLHLPWQEGYRWFQDTLVDA
DVAIDAMMWQNGGMCGLDHWNFVMHPVDAAMTCDPYGSYVRKWCPELAVLPDDLIHKPWKCPASMLRRAGVVL
GQSYPECVVTDLEERRSQSLQDVALVRRRFGEYVDPCSGCDLVPLPPRLVSEAMGGGMVNTGGQFLLPVITRM
EFKHQSDDPDADAASNPYNAVLKGYVSRRRNETIAFLNQTDFTASVINEGAERRERQERDQRRMEGLPRPLAA
QGRGKRTPAAKDRFSTVPGGVATSHR
>Salvelinus_alpi__XP_023841416.1
MVGNTIHWFRKGLRLHDNPSLKESIRGADTLRCVYILDPWFAGSSNVGISRWRFLLQCLEDLDASLRKLNSRL
FVIRGQPTDVFPRLFQEWQTRQLSYEYDSEPFGKERDAAIQKLASEAGVEVTVKVSHTLYDLDKIIELNGGQS
PLTYKRFQALISHMDAVESPAETITAEVMRKCATPISDDHDDKFAVPSLEELGFETEGLATAVWPGGETEALT
RLESHLERKAWVANFERPRMNANSLLASPTGLSPYLRFGCLSCRLFYFKLTDLYRKVKKNSSPPLSLYGQLLW
REFFYTTATNNPRFDKMEGNPVCVQIPWDXNPEALAKWAEGRTGFPWIDAIMTQLRQEGWIHHLARHAVACFL
TRGDLWISWEEGMKVFEELLLDADWSVNAGSWMWLSCSSFFQQFFHCYCPVGFGRRTDPNGDYIRRYLPILKA
FPAKYIFDPWNAPESVQKAAKCVIGMHYPKPIVHHTEASRINIQRMTQIYQQLSCYRGLGLLATVPANPNNGG
NGVGTSPEEIQHEAGKGKAGRGHAAVKRSNEDLTPGGSNKAQRQTSN
>Salvelinus_alpi__XP_023841829.1

MVVNSVHWFRKGLRLHDNPALQEALNGADTVRCVYILDPWFAGSANVGINRWRFLLESLEDLDSSLRKLESRL
FVVRGQPANVFPSLFKEWNVTRLTFEYDSEPYGKERDGAIIKMAQEFGVETIVRNSHTLYNLDRIVEMNNGSP
PLTFKRFQALVNRLELPMKPLPTITQEQMGSCRTKIADNHDEHYSVPSLEELGFKTQGLDPAVWKGGESEALE
RLNKHLDRKAWVASFERARINMCSLIASPTGLSPYLRFGCLSCRVFYYNLRELYMKLRKNCSPPLSLFGQLLW
REFFYAAGTNNPNFDRMEGNPICVQIPWDHNPEALAKWAEGSTGFPWIDAIMTQLRQEGWIHHLARHAVACFL
TRGDLWISWESGMRVFEELLLDADWSVNAGSWRWLSCSSFFQQFFHCYCPVGFGRRTDPSGDYIRRYIPKLKD
YPNRYIYEPWRAPESVQKAANCIVGVDYPKPMINHTEGSRLNIERMKQVYQQLSHYRGLSLLASVPTIQEEAE
PPPTDDSQASSSTCESPPHTSSAQSNTLKRGRPSEPQSTQTCTHTKHQHTSTAKGDQERQQEQWEEQSEHQPV
DLQ
>Salvelinus_alpi__XP_023866165.1
MHRSLLLIPASKHIWKFVTLESQRHCSFTKLVGLSSINNRTTMPAEKRKATFAKMDSTKKQKLQAVKEGKGER
EEXWLQGVVAKQRKDKDLKFNKKRLRFLSQTQKIKQGSEGVVYWMNRDHRVQDNWALIYAQQLALEEKLPLHI
CFCLVITQNPELATIRHFGFMLRGLEEVAKDCSALDIQFHLLQGSAGELLPGFVEDWRFGAVVTDFSPLRTPL
QWVEEVKKGLPSDIPFIQVDAHNVVPCWVASDKLEYSARTIRNKITKLLXEFLTEIPLVDKHPHTALRTAKPV
DWAEALSSLEVDXTVEEVDWAKSGSAAGIAMLESFIDLRLKNFATQRNNPNSSAVSQLSPWIRFGHLSAQRVV
LQVKCCGKSAGESVSSFTEELVVRRELTDNFCFYNPSYDSVKGANDWAQKTLKDHXKDPRPYVYTREQLEKAQ
THDKLWNAAQYQMVIEGKMHGFLRMYWAKKILEWTSSPEGALSIAIYLNDRYELDGQDPNGFVGCMWSICGVH
DQGWAERPIFGKIRYMNYKGCTRKFDVAQFERKYCPKDL
>Salvelinus_alpi__XP_023867825.1
MAHTCIHWFRKGLRLHDNPALVAALRXCKEIYPVFVLDPCAXNNVNIGINRWKFLIGALKDLDCSLRKLNSRL
FVVRGNPEEVFPKLFQKWKVTRLTYEYDTEPFSLRRDKEVVRLAEAHGVEIIYKVSHTLYNIDRIIEENNGKA
PLTYNRLQTLVSSIGPPKRPIPAPTSDDMKDVKTPCSEKHEENYGISTLEKLYQDPESLTEELFPGGEQEALR
RLDQHMERKEWVCDFEKPQTSPNSLSPSTTVLSPYMTFGCLSVRTFWWSLTDVYQGKKHSQPPVSLHGQLLWR
EFFYTAGLGIPNFDKMEGNPVCTQVDWDSNSEYLAAWAEARTGFPFIDAIMTQLRQEGWIHHLARHAVACFLT
RGDLWISWEEGQRVFEELLLDXDWSLNAGNWQWLSASTFFHQFFRVYSPIAFGKKTDKNGDYIKKYLPHLKKY
PAQYIYEPWKAPRSVQEAAGCIVGKDYPRPIVEHEVISKKNIQRMKAAYAKRSPHSSDESPSKKEKGLFPLGT
AIGSASPPLTLTIV
>Salvelinus_alpi__XP_024000180.1
MSTSRTVICLLRNDLRLHDNEVFHWAQRNAEHIVPLYCFDPGHYLGTANYNLPRTGPFRLRFLLDSIQDLRTS
LIQRGSTLVVRRGRPEEVVGDLIKQLGSVSAVAFHEEVVPEDQQVERGVRDICSQMKVKVHTCWGSTLYHRDD
LPFNHLSRLPDVYTQFRKAVETQGRVRPVLPTVDQLKPLPLSGLDEGPIPXPEDLEQTDTVRDPRSAXPCSGG
ETQALARLQHYFWDTDAVATYKETRNGLIGLDYSTKFSPWLAIGCISPRYIYQQIKKYESERTANQSTYWVVF
ELLWRDYFKFVGAKYGARLFDIKGLQDKSIPWKKDMNLFNAWKEGRTGVPFVDANMRELALTGFMSNRGRQNV
ASFLTKDLGLDWRMGAEWFHYLLVDNDVTRSNYGNWLYXAGIGNDPRENRKFNMIKQGLD
>Sander_luci__XP_031137844.1
MSTARIIICLLRNDLRLHDNELFHWAQRNAELIVPLYCFDPRHYVGTYNYNLPKTGPFRLRFLLESIRDLRNT
LLNKGSNLVVRQGKPEEVVAELLKQLGSVSTVAFHEEVTSEELNVEKRVKDVCAQMKVKVHTCWGSTLYHRED
LPFHHISRLPDVYTQFRKAVETQSRVRPLFPNPEQLKPLPQGLEEGAIPTAEDLEQTEPVADPRSAFPCSGGE
SQALARLKHYFWDTDAVATYKETRNGLTGVDYSTKFAPWLAMGCISPRYIYHQIQQYERERTANQSTYWVIFE
LLWRDYFKFVGVKYGNRLFQIKGLQDKSVPWKKDMKLFNAWKEGQTGVPFVDANMRELAMTGFMSNRGRQNVA
SFLTKDLGLDWRMGAEWFEYLLIDHDVCSNYGNWLYSAGIGNDPRENRKFNMIKQGLDYDNNGDYVRLWVPEL
QGIRGADVHTPWTLSTASLSHAHVSLGETYPAPIVTAPEWSRHVNKKPNGTGPSPRGKKGPSQTPKQHRDRGI
DFYFSRSKNL
>Sander_luci__XP_031138961.1
MVVNTIHWFRKGLRLHDNPSLRDSIRDADTLRCVYILDPWFAGSSNVGINRWRFLLQCLEDLDASLRKLNSRL
FVIRGQPTDVFPRMFKEWQINHLSYEYDSEPFGKERDAAIQKLASEAGVEVTVRTAHTLYNLDKIIELNDGQS
PLTYKRFQALINRMDAVELPADTITSDVIKNCATPISEHHDDKFGVPSLEELGFETEGLTTAVWPGGETEALI
RLERHLERKAWVANFERPHMNANSLLSSPTGLSPYLRFGCLSCRLFYFKLDDLYRKVKKNSTPPLSLYGQLLW
REFFYTTATNNPCFDKMEGNPVCVLIPWDRNPEALAKWAEGRTGFPWIDAIMTQLRQEGWIHHLARHAVACFL
TRGDLWVSWEEGMKVFEELLIDADWSVNAGSWMWLSCSSFFQQFFHCYCPVGFGRRTDPNGDYIRRYLPILRG
FPAKYIYDPWNAPEEVQKAAKCIIGVHYPGPIVNHAEASRINIERMKQIYQQLSSYRGLGLLATVPANPNNGA
NGSNVRGVNKGTSQGPGGSSDGIFKYPLTSRTERGQKRRCEEAPTQSNCKSWRKSK
>Sander_luci__XP_031150246.1
MLHCLWRSSSFSSAPRRLLRLVTHSNHRPTSVFSSPSKSFTSQTIMSDKKRKSTSSAAAGGKEPSAKQQKLAP
MKEEEKKERVEGWLQDLVKQQRTEKKEMKFNKKRLRFMSDTEQAKQGSEGVLYWMLRDHRVQDNWALVQAQRL
ALEENLPLHICVCLVVPKSELSTLRHYSFMLKGLKEVAKECKTLDIQFHLLHGSAGDVLPGFVSDRGLGTVVT
DFSPLREPLQWLEDVKKTLPKDIPLIQVDAHNIVPCWVASPKLEYAARTIRGKITKLLPEFLTEFPLVEKHPY
TATRTAKPVDWDKILASLQVDRAVGEPEWAKPGTNGGMAMLESFIDVRLKLFDTQRNDPNAAALSQLSPWIRF
GHLSAQRVALQVQHSGKSAGHSVSSFIEELVVRRELTDNFCFYNKKYDCVEGAYEWAQKTLKDHAKDKRPYLY

TREQLEKAKTHDKLWNGAQYQMVTEGKMHGFLRMYWAKKILEWTSSPEEALSIALYLNDRYELDGQDPNGFVG
CMWSICGIHDQGWRERAVFGKIRYMNYKGCLRKFDVARFERKYCPKNL
>Sander_luci__XP_031153688.1
MVVNSVHWFRKGLRLHDNPVLQEALNGADTVRCVYILDPWFAGAANVGINRWRFLLEALEDLDSSLKKLNSRL
FVVRGQPTDVLPRLFKEWKVTRLTFEYDPEPYGKERDGAIIKMAQEFGVETIVRNAHTLYNLDRIIEANNNSP
PLTFKRFQTIVSRLELPRRPLLPITQQQMENCRTEIADNHDQLYSIPSLEELGFRTVGLPPAVWRGGETEALD
RLNKHLDKKVWVANFEHPRVNTCSLYASPTGLSPYLRFGCLSCRVLYYNLRELYMKLRKHCSPPLSLFGQLLW
REFFYTAATNNPNFDRMEGNPICVQIPWDQNPEALAKWAEGRTGFPWIDAIMTQLRQEGWIHHLARHAVACFL
TRGDLWISWESGMKVFEELLLDADWSVNAGSWMWLSCSAFFQQFFHCYCPVGFGRRTDPSGDYIRRYIPILKD
YPNRYIYEPWNAPESVQKAAKCVVGVDYPKPMINHAEGSRLNIERMKQVYQQLSHYRGLSLLASVPTIQEEAE
PPMTDESRTSSGPDSPPKGPADSEAADCSTAPDSSTVCAFSTYSPYPDLEGATNSHPSQTPCAYSVSHTQPSA
AITSASASQALSTAAPPSPASTPTQSSLSRFKPSSPSSSCPTMSPSLSPAMTMTQHSFSGQKRKGLVSKVRCI
QRQRGRQSCTPAAGEVERRAEEEEKEREEAGLEERMEQDVEQDEERMEEETAGHQQ
>Sander_luci__XP_031170258.1
MAHTCIHWFRKGLRLHDNPALMAALRDCKELYPVFILDPYLHNNTCVGINRWRFLVGALVDLDCSLRKLNSRL
FVVRGKPEEVFPKLFDKWKVTKLTYEYDTEPYSLSRDKTVTTLAKEHGVEVIYKISHTLYDIDRIIEENNGKA
PLTYNRMRAIVKTLGPPKRPIPAPIMQDTKDVKTPCSENNEELKKYGIPMLEELGQDTAALGQEQFPGGEQEA
LRRLDEHMQRTGWVCGFEKPQTSPNSLSPSTTVLSPYVTFGCLSARTFWWRLTDVYRGKKHSDPPVSLHGQLL
WREFFYTASVGIPNFDKMEGNPVCTQVDWDTNPDYLAAWREARTGFPFIDAIMTQLRQEGWIHHLARHAVACF
LTRGDLWISWEEGQKVFEELLLDGDWALNAGNWQWLSASAFFHQFFRVYSPVAFGKKTDKNGDYIKKYLPLLK
KFPAQYIYEPWKAPRSIQQAAGCIVGQDYPHPIVQHDVISKKNIQRMKLAYAKRADPAESPSNKQGVKRKAPS
VIDMMKKKDRKK
>Sander_luci__XP_035858845.1
MPHSVAAAEDSKALVRKTLREVLVGREDPEGFFAMCVSALGHQETRSQFLSLIQPLSTATNSSLHSILTSIYR
GYFSKTEDDELELALALSLLEMKDHQLSTPSQQSRLQQPGDRPNQRCSVQLNSVSQPQGSSHAQLADVENTNS
PQTGAWVKAGPPQTTRETEPQKSTHVDEYKSETPGTSQTVCVSRLSGLFSKQDTVEEGDQMMDEGDLNQIEKL
KRSKKRRQRCKGAGQQVVGLPCSPSAPPPVLLWLRRDLXLGDNPALIGSLEVGAPVIPLFIWSPDEEEGPGIT
VAMGGSCKYWLHQALSCFSSSLQSIGSHLVFLKANGSSLRTLKELVKETGARTVLATALYEPWLKDMDDVVVS
ALQKHGVECRMIHSYCLRDPYSVSTEGVGLRGIGSVSHFMSCCRQNPGSVLGFPLDSPGSLPTPAHWPQGVSL
GTLGLARMPRRKDGTTIDWAANIRKSWDFSEEGACAQLEAFLDWCVDMKESGRADAPNTSCLSPYLHFGQLSP
RWLLWDAKEARCRPPKFQRKLAWRDLAYWQLTLFPDLPWESLRPPYKDLRWSSDLGHLKAWQQGRTGYPLVDA
AMRQLWLTGWMNYMRHVVASFLIAYLYLPWQEGYRWFQDTLMDADVAIDAMKWQNGGMCGLDHWNFVMHPVDA
AMTCDPYASYVRKWCPELADLPDELIHKPWKCPASMLXRAGVVFGQTYPERIVTDLRERRSRSLQEVALVRXE
YGRYVDKCTGCDLVPLPPRLVSEALGLSHRDGGVVTEGKQFLLPVITXHAPSNPYNAVLKGYMSCKRDDNIAF
LNERDKCDVXGSSEKGERLERDYCRIEGLPQPPAPQGRARRTPTSKDRFSIVPGGAVSSLR
>Saprolegnia_dic_XP_008613356.1_SDRG_09216
MKLVDLAVAGVKAERLRWLHGSAALSSGSHVLYWMQTSLRTRYNYALHVAMAAATTLQKPLYVLYTLDTHEAG
MSERHMAFLLESLYDVNKSLQQTHHVPFSAVHCASGSSGPLDVVLAASRNASLVVTELQYLRSGRKLCTTFGE
QATAPVVQVESDVVVPVEVASNKEEYAARTLRPKITKHLPTYIVSLPSIAYAPPSPAPKSLQELQALSLSTHD
TWLDLTDASAVDTLLATAKDVDRSIPRVSTFLGGETSAQSVLTTFLSQKLLKYGTERNEPSGNGASNLSPYLH
YGNISPVDIALQVNATEGRGPAMAASKASFLEELIVRRELSMNFVWFNPRHYDSIKSLPPFATETLAIHADDA
RATIYSEAQLDTAMTHDAYWNAAQLDMIVNGKMQNYMRMYWGKKILEWTASPEEAYEICIRLNDKYNLDGYDP
NSYTGIAWVFGKHDTGWAERAIFGKVRYMNADGLKRKYDMQAYVNMIRHQCAAAKMTLQPKQLALECTKPKAK
RQKKA
>Sarcophilus_harrisii_XP_031794408.1_cryptochro
MGVNAVHWFRKGLRLHDNPALKECIEGADTVRCVYILDPWFAGSSNVGINRWRFLLQCLEDLDANLRKLNSRL
FVIRGQPADVFPRLFKEWNIAKLSIEYDSEPFGKERDAAIKKLASEAGVEVIVRISHTLYDLDKIIELNGGQP
PLTYKRFQTLISKMEPLATPVETITPEVMLKCVTPLSDEHDEKYGVPSLEELGFDTDGLPSAVWPGGETEALT
RLERHLERKAWVANFERPRMNANSLLASPTGLSPYLRFGCLSCRLFYFKLTDLYKKVKKNSSPPLSLYGQLLW
REFFYTAATNNPRFDKMEGNPICVQIPWDRNPEALAKWAEGRTGFPWIDAIMTQLRQEGWIHHLARHAVACFL
TRGDLWISWEEGMKVFEELLLDADWSINAGSWMWLSCSSFFQQFFHCYCPVGFGRRTDPNGDYIRRYLPVLRG
FPAKYIYDPWNAPEGIQKVAKCLIGVNYPKPMVNHAEASRLNIERMKQIYQQLSRYRGLGLLASVPSNPNGNG
GLMGYTSGENGPACNSGGGAPVGASDGQILQSCALPEPPAGASCIQQSDI
>Sarcophilus_harrisii_XP_031799863.1_cryptochro
MAATAVVTVTAAAPAPAPAPAPAPAEGASSVHWFRKGLRLHDNPALQAALRGARCVRCVYILDPWFAASSSVG
INRWRFLLQSLEDLDISLRKLNSRLFVVRGQPTDVFPRLFKEWAVTRLTFEYDSEPFGRERDAAIVKMAKEAG
VEVVTENSHTLYDLDRIIELNGQKPPLTYKRFQAIISRMELPRKPVSGVTRQQMERCQAEIREDHDEAYGVPS
LEELGFPTDGLGPAVWQGGETEALARLDKHLERKAWVANYERPRMNATSLLASPTGLSPYLRFGCLSCRLFYY
RLWELYKKVKRNNTPPLSLYGQLLWREFFYTAATNNPKFDRMEGNPICIQIPWDRNPEALAKWAEGKTGFPWI
DAIMTQLRQEGWIHHLARHAVACFLTRGDLWVSWESGVRVFDELLLDADFSVNAGSWMWLSCSAFFQQFFHCY

CPVGFGRRTDPSGDYVRRYLPQLKGFPSRYIYEPWNAPESVQKAAKCIIGVDYPKPMVNHAETSRLNIERMKQ
IYQQLSRYRGLCLLASVPSCMEDLSSPMVEASVGQPGLSSAGPKVPPCPPASPKRKHETPDEACGGEHGKRAR
VMVAVPGPELVGRDV
>Sarcoptes_sca_KAF7493271.1_Deoxyribod
MSNLVILQNDLRLHDNPALYYAAQEDLPIIVVYVIEKRIGTAALWWLTKSLTNFRERLQEYGIELLSVFDRNQ
FPDLIDYLQKDLSIEIKKIFSNGIDFDLPDKSLEKIHESLMPNNIYDFSKLPKEHKVFTPFWKFLQEKFEPEL
PVDDPKFSKSNKTMIQFDRFQEIPYEKTREDLDSHWKTGEKAALEQYRNFVKSRLVNYDVERDYFSRDGTSKL
SPHLRFGEISARKIYHDLKKNSRNKRFLSELGWREFGYQTYYFHPKMDSIPLNPKYANFFPKYNEDHLIAWKN
GTTGYPVIDAAMRQLKHTGWMHNRLRMVVASFLTKNLLIPWQQGEQFFYDCLVDGDRALNPMNWQWVAGCGFD
SVPYFRIFNPSLQGEKFDSDGTFVRYWVEELKNVSKSFNIYKDYEKIPSKSYPKPIINYSKSRIRALETLKNV
VNPKVVKVQKRSHKEEDSDED
>Scatophagus_argu__XP_046227599.1
MSASRTVICLLRNDLRLHDNELFYWAQRNAEYIVPLYCFDPRHYVGTYNYNLPKTGPFRLRFLLESISDLRDT
LLKKGSNLVVRRGKPEEVVADLIKHLGSVSAVAFHEEVTSEELTVEKQVKDVCAQLKVKVHTCWGSTLYHRDD
LPFHHMSRLPDVYTQFRKAVETQSSVRPVIPTPEQLKPLPQGLEEGSIPTAEDLHQTEPVTDPRSAFPCSGGE
SHALARLKHYFWDTDAVATYKETRNGLIGVNYSTKFAPWLALGCVSPRYIYHQIKQYERERTANQSTYWVIFE
LLWRDYFKFVGVKYGNRLFQVKGLQDKSVPWKNDMKLFNAWQEGRTGVPFVDANMRELALTGFMSNRGRQNVA
SFLTKDLGLDWRMGAEWFEYLLIDYDVCSNYGNWLYSAGIGNDPRENRKFNMIKQGLDYDNNGDYVRQWVPEL
QGIKGADVHTPWTLSTAALSHARVSLGETYPTPVVIAPEWSRHVNKKPMGPGPSPRGKKGPSHTPKQHRNRGI
DFYFSRSKDL
>Scatophagus_argu__XP_046237517.1
MTHTCVHWFRKGLRLHDNPALMAALRDCKELYPVFILDPNLHNDARVGINRWRFLIGALKDLDCSLRKLNSRL
FVVRGTPEDVFPKLFNKWKVTKLTYEYDTEPHSLSRDKTVTTLAKKNGVEVIYKISHTLYDIDRVIEENNGKA
PLAYNRMQAIVKTLGPPKRPIPAPTMENMKGVMTPCSENQDEKYGIPTLEELNLKTAGLGEEQYPGGEQEALR
RLDEHMKRKEWVCSFEKPQTSPNSLNPSTTVLSPYVTFGCLSARTFWWRLTDVYHGKKHSDPPVSLHGQLLWR
EFFYTASVGIPNFNKMEDNSVCTQVDWDTNPEYLAAWREARTGFPFIDAIMTQLRQEGWIHHLARHAVACFLT
RGDLWISWEEGQKVFEELLLDGDWALNAGNWQWLSASAFFHQYFRVYSPVAFGKKTDKNGDYIKKYLPTLKKF
PAEYIYEPWKAPRSIQQAAGCIVGKDYPRPIVQHEVISKKNIQRMKSAYAKRSAHIAESPNKKQGVKRKAPSV
LDMPKIKMRNK
>Scatophagus_argu__XP_046248946.1
MPPSVAGGAEDSKALVRKMLREVLVGREDPEGFFAMCVSALGHQETRSKFLSLIQPLSTANISLHSNLTSIYK
EYFSESEDDELELALALSLLETKDDQLSTPSRQSKLKRNPARLTSLPQTQGGSHTQSADVDSRVKNISSLRDG
PWVKLSTPQITGEASLQGTAHIEYNKETPGTSQTVHVSTLPGSIFKQEMGEEGEQMMDEGHSDQSENRKRSKN
RRQRRRGASQQVVGLPCSPSALPPVLLWFRRDLRLCDNPALTGSLELGAPVIPIFIWSPEEEEGPGITVAMGG
ACKYWLHQALSCFCSSLEHIGSHLVFFKATGQGNEVGSSLCILKGLVKETGARTVLANALYEPWLKERDDAVV
SALQKHGVECRMFHSYCLRDPYTVSTEGVGLRGIGSVSHFMSCCRQNPGSALGVPLDPPVSLPTPAHWPQGVS
LDTLGLARMPRRKDGTTIDWAANIRKSWDFSEEGAHARLEAFLHDGVYRYEKESGRADAPNTSCLSPYLHFGQ
LSPRWLLWDAKGARCRPPKFQRKLAWRDLAYWQLTLFPDLPWESLRPPYKALRWSSDRGHLKAWQRGRTGYPL
VDAAMRQLWLTGWMNNYMRHVVASFLIAYLHLPWQEGYRWFQDTLVDADVAIDAMMWQNGGMCGLDHWNFVMH
PVDAAMTCDPYGSYVRKWCPELSDLPDELIHKPWKCPASMLRRAGVVFGQTYPERIVTDLEERRSQSLRDVAL
VRKEFGQYVDKRSGCDLVPLPPRLVSEALGLSHRDGSVVTAGKQFLLPVITRMEFKHQLDDPDGDAATNPYNA
VLKGYVSRKRDDTIAFLNERDFTASVMYEGTQRRERLESDYRRIEGLPRPPAPRGRARRTPTAKDRFSIVPGG
TVTSLR
>Scatophagus_argu__XP_046250340.1
MVVNTIHWFRKGLRLHDNPSLRDSIQGADTLRCIYILDPWFAGSSNVGINRWRFLLQCLEDLDASLRKLNSRL
FVIRGQPTDVFPRLFKQWKITHLSYEYDSEPFGKERDAAIQKLACEAGVEVIVRISHTLYNLAKIIELNDGHP
PLTYKRFQALISHMDAVELPAETITLKVIKKCTTPISEDHDDKFGVPSLEELSFDTEGLTTAVWPGGETEALV
RLERHLERKAWVANSELPRMNANSLLASPTGLGPYLRFGCLSCRLFYFKLTDLYRKVKKNNTPPLSLYGQLLW
REFFYTTATNNPCFDKMEGNPVCVQIPWDRNPEALAKWAEGQTGFPWIDAIMTQLRQEGWIHHFARHAVACFL
TRGDLWISWEEGMKVFEELLIDADWSVNAGSWMWLSCSSFFQQFFHCYCPVGFGRRADPNGDYIRHYMPILRG
FPAKYIYDPWNAPEEVQKAAKCIIGVHYPKPMVNHAEASRINIERMKQIYQQLSRYKGLGLLATVPVNPNSGI
NGSNVGRVNTGTSQVPGGSCENPSTQDGTSQAERGPFPQKRRCVEAPLESSSKCWKQGK
>Scatophagus_argu__XP_046250381.1
MVVNSVHWFRKGLRLHDNPALQEALNGADTLRCVYILDPWFAGAANVGINRWRFLLEALEDLDSSLKKLNSRL
FVVKGQPTDVFPRLLKEWKVTRLTFEYDPEPYGKERDGAIIKMAQEFGVETIVRNSHTLYNLDRIIEMNNNSP
PLTFKRFQTIVSRLELPRRPLPPITKQQMDKCQTKIADNHDQLYSIPSLEELGFRTEGLPPAVWRGGESEALD
RLNKHLDKKVWVTNFEHPRVNTCSLYASPTGLSPYLRFGCLSCRVLYYNLRELYMKLRKRCSPPLSLFGQLLW
REFFYTAATNNPNFDRMEGNPICVQIPWDQNPEALAKWAEGRTGFPWIDAIMTQLRQEGWIHHLARHAVACFL
TRGDLWISWESGMKVFEELLLDADWSVNAGSWMWLSCSAFFQQFFHCYCPVGFGRRTDPSGDYIRHYIPILKD
YPNRYIYEPWNAPESVQKAANCVVGVDYPKPMINHAEGSRLNIERMKQVYQQLSHYRGLSLLASVPTIQEEAE

PPMTDESQTSSGPDSPPRGPADSEAAGCSTAPDSSTVCPSSTYAPQPDLEDTADNYLSQTTSSCSRSQPSAAI
TSTSAPPFSAQSPVSRSKPSSPSSSCLTSSSSLSPATTPTPTQASSLGQRRKGLARKVRRSQRQRGRQSCTPA
AREGDRRSEEDEREEVGEDERMEEDAEQDDERMEEETSGQAAGSQQ
>Scatophagus_argu__XP_046263365.1
MLHYIWRNSSFFFTPGSFLRLIAHSKQKPISSCLSKSLTSQTIMSGKKRKATSAAKESSAKQQKLAPMKEEKG
EKKERAGGDEEMEFNTKRLRFISDTEKMKQGSEGVLYWMFRDHRVQDNWALIHAQQLAVKEHLPLHVCVCLLV
PKSELSTLRHYSFMMKGLEEVAKECKTLDIQFHLLHGSAGEVLPGFVTTHSLGAVVTDFSPLREPLQWLEDVK
KKLPKDIPLMQVDAHNIVPCWVASPKLEYAARTIRGKITKVLPEFLTDFPLVEKHPYKATRTAKPVDWDKTLD
SLQIDRSVGEPQWAKPGTKAGMAMLESFIDVRLKLFDTQRNNPNVAALSQLSPWIRFGHLSAQRVALQVQRSG
KKTGLSVSSFIEELVVRRELTDNFCFYNKKYDSVEGAYEWAQKTLKDHAKDERPYIYTREQLEKAKTHDKLWN
AAQYQMVTEGKMHGFLRMYWAKKILEWTSSPKEALSIALYLNDRYELDGQDPNGFVGCMWSICGIHDQGWAER
PIFGKIRYMNYKGCLRKFDVPQFERKYCPKNP
>Schistocephalus_soli__VDL96751.1
SSLRVVSEQGWLGESSVYLFTNGPYAQIISIYEAPRRATHTHDSSTVVVRRGPPYAPRKGVAETDPDWKSIEA
SVLGHLDESVKAIKWAQGGSRHGFRELFTFLHERLALYATARNDPTKNALSQLSPWFHFGEFFLPHAYLPKFH
REWTPATCHLSVQRAILEAQRLRRKHKESVDAFIEEAFVRRELSDNFCLYNPHYDSIEGAWSWAQETLCKHAS
DERKPAYAMETMEKAQTGDDLWNAAQRQLLTEGKIHGFLRMYWAKKILEWHAGGPEAALAVGLRLNDRYSIDG
SDPNGFVVGAAASDAIGSTKPLPTATDELGVMWSICGIHDQGWSERAIFGKIRYMNYAGCKRKFNIAQFVARY
PS
>Schistocephalus_soli__VDL97200.1
MLAAWTEARTGFPFIDAAMTQLNRHGWMHHLARHAVACFLTRGQLWQSWTDGAAVFQSLLLDADWALNSANWM
WLSASAFFHQYYRVYCPVGFGRKTDPEGKFIRHFLPCLKNMPKQYIYAPWTAPLAVQKKAGCVIGVDYPVPIV
DHQEMRAANLVKMKAAYDACRKFAPSSANKGTDTEHNPSNKKTDTPHYPSNRKTDTSPTKLGRLTTDKSQASK
RRKL
>Schistocerca_gre_QVD39239.1_cry_2
MCAKTTQEHTVHWFRRGLRLHDNPSLRAGLIGATTFRCIFILDPWFAGSSNVGINKWRFLLQCLEDLDSSLRK
LNSRLFVIRGQPADALPKLFKEWGTTNLTFEEDPEPFGKVRDQNIMAMCREMGISVISIASHTLYSLDTIIEK
NGGKAPLTYHQFQNIVASMDSPPPAEPSITPACVKDKFTPISDDHDERFGVPTLEELGFDTDGLMPPVWQGGE
SEALARLERHLERKAWVASFGRPRMTSQSLLASQTGLSPYLRFGCLSTRLFYYQLTDLYKKIKKACPPLSLHG
QLLWREFFYCAATKNPYFDKMYGNPICVQIPWDRNPEALAKWATGQTGYPWIDAIMIQLREEGWIHYLARHAV
ACFLTRGDLWISWEEGMKVFDELLLDADWSVNAGMWMWLSCSSFFQQFFHCYCPVRFGRKADPNGDFIRKYLP
ILKNFPTRYIHEPWKAPESVQITAKCIIGKEFPLPIVDHQYASRINIERMKQVYQQLSKYRGPGLLATVPTIR
SPSVKGCQYDSKEDKTTPENRIVSANNDCSMYVETQQQMTPQETKCDWEFCRRNM
>Schistocerca_gre_QVD39240.1_cry_2
MAKGVAIHWFRKGLRIHDNPALLEAISACYNENLELCPVFVLDPWIIKKLRVGPNRWRFLQQTLCDLDANLRK
IGTRLYVLRGTPEETFKDKFQEWNVKVITFETDIEPYARERDERVIKIAETAGARVVQKVSHTLYDPEHIVQK
NMGKPPLTYQKLLSLVDNIGPPEKPVNSPSKLSERITLNSVKIDDHNHCVPTLIELGVNEKLLKPTIFPGGES
AALDRLNLKMKNKEWVRKFEKPNTSPNSLEPSTTVLSPYLKFGCLSARTFYYKLKEVIGTSPHTKPPVSLLGQ
LLWREFYYVVAAETPNFDKMVGNPICCQVPWNKNEEHLAAWAEGRTGYPFIDAIMIQLRTEGWIHHLSRHAVA
CFLTRGDLWISWEDGQKVFEELLLDADWALNAGNWMWLSASAFFHQFFRVYSPVAFGKKTDKLGEYIKKYVPV
LRKYPPEYIYEPWKAPLSVQKQAGCVIGEDYPKRIVIHEDVYKINISKMSEAYKRWKGAKSKQEDKISNTVKN
KKMKIT
>Schistosoma_man_XP_018648974.1_putative_D
MAVLAETVHEWLDGIHNKRLSTASSAENFKFHKSRIRHLSGPGTFPEKDDGFGQGGVLYWMSRDQRVQGNAIQ
GYTFFVFLVLLISKEIIFIAQPEDISSGCKHRWSDENALFNQTDRIEKNFLYETYVAKSVVDLAKLLNVGCLV
TDFCPLRAPRSWVKKVTDELPDNIPFCEVDAHNIVPVWCGSEKREYSARTIRSKLFQQSPKFLTDFPPIIKHT
CSDKITVLPPPVNWESILSDYVGDKSVKPVDWAVPGTQAGLEVLYEFISKRLKKFDPHRNDPAHPALSGLSPW
LHFGQIAPQRAVLEVVAVQKHFGRSADIFIEECFNRRELAENFCFHTPFYDSIKGAYDWARESLMTHSTDKRD
PAYSKTQMETAQTGDDLWNAAQRQLVLKGKMHWFLRQYWAKKILEWCAEGPESAIQIAIYLNDRYSLDGTDPN
GYVGIMWAICGVHDQGWPERPIFGKIRYMNYKGCLRKFSVSTFVSRYPEKLD
>Scleropages_formosus_XP_018619474.1_cryptochro
MVVNSVHWFRKGLRLHDNPALQEALKGADTLRCVYVLDPWFAGSANVGVNRWRFLLESLEDLDACLRKLNSRL
FVVRGQPADVFPRLFKEWGVTRLTFEYDSEPYGKERDAAIIKMAQEFGVETVVKKSHTLYDLDRIIEMNNSSP
PLTFKRFQAIVNRLGLPRKPLPTVTRQQMDGCWTDVSMNHDERFGVPTLEELGFKTQELSPAVWKGGETEALE
RLKKHLDRKAWVANFERPQINVCSLMASPTGLSPYLRFGCLSCRLFYYELRDLYLKVRKHSSPPLSLFGQLLW
REFFYTAATNNPNFDRMEGNPICVQIPWDHNPEALAKWAEGRTGFPWIDAIMTQLRQEGWIHHLARHAVACFL
TRGDLWLSWESGMRVFEELLLDADWSVNAGSWMWLSCSAFFQQFFHCYCPVGFGRRTDPSGDYIRKYIPKLRD
YPNRYIYEPWNAPESVQKAVNCVVGVDYPRPMINHAEVSRLNIERMKQVYQQLSHYKGLSLLASVPTIQEEAE
LPRSDDSQSHNSSDVQSDEPSKKASLGKRERPSELQRPPACTQPKVQRRSTAEGSSSKQQY
>Scleropages_for_XP_018586555.2_photolyase

MPGRRKQPAGKRGAEPAERPPEGGKRRREEGWMARQVAELRAAERAQPFNEKRLRFLSESRKVKQGSQGVLYW
MCRDQRVQDNWALIYAQRLARTEKLPLHVCFCLSPRFLEATVRQMGFMLNGLKEVEKECSTLEIQFHLLLGAA
GEVLPGFVKDRRMGAVVTDFFPLRLPLQWAEDVKKGLPSDVPFIQVDAHNVVPCWVASDKQEYSARTIRGKIT
KLLPEFLTEFPLVEKHPRCAPVLAKPVDWDKAWSSLEVDRSVGEVEWARPGSTAAMATLESFVDQRLQDFAAH
RNNPNREALSQLSPWLHSGQVSAQRVVLQVKRNGPRGPSVDAFVEEAVVRRELADNFCYYNKDYDCVDGAYEW
AQKTLRDHAKDPRPHLYSRKQLEEAKTGDRLWNAAQIQLVKEGKTHGFLRMYWAKKILEWMPSPEEALSTALY
LNDRYSLDGNDPNGYVGCMWSICGIHDQGWQERPVFGKIRYMNYAGCKRKFDVPAFERRYCSVKL
>Scleropages_for_XP_018586695.2
MRHNSVHWFRKGLRLHDNPALQAALRDCAELYPVFVLDPWFPKNARVGITRWRFLIGALADLDCSLRRLNSRL
FVVRGKPEEVFPRLFQKWKVTRLTFEVDTEPYALQRDKNIASLAAEHQVEVIQEVSHTLYDTERIILENNGKA
PLTFKRMQTLINTVGAPKRPCPPPTLQDMIDICTPSSEDHEEAYGVPSLAELGFDSLNAGPELYPGGEQEALR
RLEEHMSRTGWVCAFEKPQTSPNALTPSTTVLSPYLKFGCLSARTFWWRLDDVYRGRKHSEPPVSLHGQLLWR
EFFYTTSVGIPNFDCMVGNPVCVQVDWETNPEYLAAWREGRTGFPFIDAIMTQLRQEGWIHHLARHAVACFLT
RGDLWISWEEGMKVFEELLLDADWALNAGNWQWLSASAFFQQYFRVYSPVAFGKKTDKNGDYIRKYLPILKKF
PAEYIYEPWKSPRSLQERAGCIVGKDYPKPIVEHEVISKKNIQRMKMAYAKHSGSSSESSGKSKGEKRKVGSV
LDMLTKKNTKLD
>Scleropages_for_XP_018606994.1_cry_DASH-l
MCSAPHTVICLLRNDLRLHDNEVFHWAQKNARYIVPLYCFDPRNYLVTFCYNLPKTGPFRLRFLLDSLKDLRA
TLQKKGSNLFVRQGKPAEVISELIKQLESVSTVIFHEEVTHEELEVEKEIRNVCSQLKVKVHSCWGSTLYHRD
DLPFSHISRLPDVYTQFRKAVETQGEVRPVLSTPDMLKPFPAGLEEGSIPALKDLGQTDVLEDPRSAFPYHGG
ESQALARLQHYFWDTDAVARYKETRNGLIGVNYSTKFAPWLANGCISPRFIHDQIKKYERERGANQSTYWVIF
ELLWRDFFRFVAMKHGNKIFSLNGLQDKALPWKKDISLFNAWKEGRTGVPFVDANMRELAQTGFMSNRGRQNV
ASFFTKDLGLDWRMGAEWFEYLLVDYDVCSNYGNWLYSAGIGNDPRENRKFNVIKQGLDYDNNGDYVRQWVPE
LQGISGCKVHTPWTLSKDVLSKGQVTLDKTYPFPVVMASEWSHHANQNMDVSGPGPRGRRGLSHTPKHYGNRG
IDFYFSRNKNLK
>Scleropages_for_XP_018610422.2
MVVNTIHWFRKGLRLHDNPSLKESIQGADTVRCVYILDPWFAGSSNVGISRWRFLLQCLEDLDASLRKLNSRL
FVIRGQPTDVFPRLFKEWSIKRLSYEYDSEPFGKERDAAIRKLASEAGVEVVVRISHTLYDLDKIIELNGGQS
PLTYKRFQTLISRMEAVETPVESITAEVMGKCTTPVFDDHDDKFGVPSLEELGFNTEGLSSAVWPGGETEALT
RLERHLERKAWVANFERPRMNSNSLLASPTGLSPYLRFGCLSCRLFYFKLTDLYKKVKKNSSPPLSLYGQLLW
REFFYTAATNNPRFDKMEGNPICVQIPWDRNPEALAKWAEGRTGFPWIDAIMTQLRQEGWIHHLARHAVACFL
TRGDLWISWEEGMKVFEELLLDADWSVNAGSWMWLSCSSFFQQFFHCYCPVGFGRRTDPNGDYIRRYLPILRG
FPAKYIYDPWNAPESVQKSAKCMIGVHYPKPMVNHAEASRLNIERMKQIYQQLSCYRGLGLLATVPSNPSGNS
EAAGNMVGLPHGEAAQDSLGTIGHQVPANLVGDWSGAVMAHPQGESKPGGSTQQQRRGHHTSTMTTVKRPSED
STPGVVFKVQRQNSN
>Scleropages_for_XP_029109379.1_uncharacte
MMPATDSSRPAAHVSRVQAMVTQVLLGTMEPEELFWGSLSLLGLQGTQDTFLDLIEPLAQEHAEVHTHLTSVF
LDYFTRAKEDEALALALALSLQDAGAGKQMTTEESSGSRSQDVKCTRTLWLEANASAGPRDGWSSSSTQCSGD
LEHQHNLQPQSMTGAGTTGKSNSMSERANSCSLLPCTDNKNVPEEVTASCPGRAKSSRRKRRSGKQQLAISRF
PSFLKPILLWFRRDLRVSDNPVLIASLEMGVPIIPVFLWCPKEEEGPGITVATGGASKYWLHHALLCLNQSLE
KLGSHLVTVEAETSSLEALQGLAAETGASGVVAAALYEPWLKERDEAVFSALESKGVKCQLRHSYCLREPSSI
STEGVGLRGIGSVSHFLSCCQQNSKSALGTPVETPPGLPVPSTWPQGCPLAQLGLAKMPRRKDGTTVDWAANI
RSSWDFSEEGAQARLRAFLLDGIHRYEKESCRADAPNTSCLSPYLHFGQISPRLLLWASRSATCRPPKFRRKL
AWRDLAYWQLCLFPDLPWQSLRPAYRALRWSSDRTHLKAWQKGRTGYPLVDAAMRQLWLTGWMNNYMRHVVAS
FLIAYLHLPWQEGYRWFQDTLVDADVAIDAMMWQNGGMCGLDHWNFVMHPVSAGLTCDPRGTFARRWCPELAA
LPDELVHRPWKSPASMLRRAGVTLGVNYPERIITDLEERRAQSLRDVVRVRRNFPQYVDQLSGCDLVPLPQRL
VQEALGSGEVADRGGRFLLPLITRMEFKYQTQNPDCRDNPFDAVLKGYVSRKRDEATAFRNERDFAASVLSES
VQRRERQEREQRALDGIPRPATGRPGRTPRKDVFSAQGAGFTLLHN
>Scleropages_for_XP_029114945.1
MTGKHGESGTSTKTHKTIHLFRKGLRLHDNPSLVWALESSRVVYPVYVLDRIFLEGALRTGALRWRFLLQSLE
DLHIQLAVLGSRLYVVQGRFEAVLRHLIAKWGITQLTFDVEVEPFYARLDEDLGAMGKELGLSIHTCVAHTLY
NVSRIVEVNGGHPPLTYKKFLQVLSTMGEPDRPAREITAQDFLNCPTPVDASEEFRVPSLEDFGIVVESEVLW
PGGESHALKRLEKHFESQGWVANFSKPRTIPNSLLPSTTGLSPYLSLGCLSVRTFYHRLSNIYAQSKNHSLPP
VSLQGQVLWREFFCTVASATPNFTCMAGNPICLQIGWYEDTEALKKWRTAQTGFPWIDAIMTQLQQEGWIHHL
ARHAVACFLTRGDLWISWEEGMKVFEEYLLDADYSVNAGNWMWLSASAFFHQYTRIFCPVRFGRRTDPDGQYI
RRYLKVLQNFPSRYIYEPWTAPLEVQVEAGCVIGRDYPLPMVSHSEVSQKNLALMAQVRREQASTAQLTRDVT
DDPVEMGMKREHVEDQGFRDGISEQPERPCKRLSPEGDCPVTPCSWSHDGLSLPKLSGEVM
>Scophthalmus_maxi__AWP20602.1
MSTSRTVICLLRNDLRLHDNELFHWAQRNAEKIVPLYCFDPRHYVGTHNYNLPKTGPFRLRFLLESVRDLRDT
LLAKGSNLVVRRGKPEDVVAELIKQLGSVSTVAFHEEVTSEELHVEKKVKEICAQMRVKVHTCWGSTLYHRDD

LPFHHMSRLPDVYTQFRKAVETQSKVRPVFPAPEQLKPLPPGLEEGAIPMAQDLQQTEPLTDPRSAFPCSGGE
SQALARLKHYFWDTDAVATYKETRNGLIGVDYSTKFAPWLANGCISPRYIYHQIQQYERERTANQSTYWVIFE
LLWRDYFKFVGVKYGNRLFQVQGLQDKSVPWKKDMKLFDAWKEGRTGVPFVDANMRELAMTGFMSNRGRQNVA
SFLTKDLGLDWRMGAEWFEYMLIDHDVCSNYGNWLYSAGIGNDPRENRKFNMIKQGLDYDSNGDYVRQWIPEL
QGISGADAHTPWTLSPAVLSHARISLSETYPTPIVTAPEWSRHVNKKPSGSGPSPRGKKGPSHTPKQHRDRGI
DFYFSRSKNL
>Scophthalmus_maxi__KAF0041381.1
MVVNTVHWFRKGLRLHDNPSLRDSIRASDTLRCVYILDPWFAGSSNVGINRWRFLLQCLEDLDASLRKLNSRL
FVIRGQPTDVFPRLFKEWQVSRLSYEYDSEPFGKERDGALRKLADEAGVEVMVRISHTLYDLDKIIELNAGQP
PLTYKRFQVLLTCMDAVDLPTETITSEVIKKCTTPIVADHDDKFGVPSLEELGFETEGLTTAVWPGGETEALV
RLERHLERKAWVANFERPRMNATSLMASPTGLSPYLRFGCLSCRLFYFKLTDLYKKMKKNSTPPLSLYGQLLW
REFFYTTATNNPCFDKMEGNPMCVQIPWDRNPEALSKWVEGQTGFPWIDAIMTQLRQEGWIHHLARHAVACFL
TRGDLWIRWEEGMKVFEELLLDADWSVNAGNWMWLSCSSFFQQFFHCYCPVGFGRRTDPNGEYIRRYLPILRG
FPAKYIYDPWNAPEEVQKAAKCIIGVQYPKPMVNHAEASRANIERMRQIYQQLSSYRGLGLLATVPANPNNGA
GGSNVAGVNTGTGHGSGESSEELSIREGMPRTERSQSTQKRQREEEPSKSGSKSGRQSK
>Scophthalmus_maxi__XP_035484982.1
MAHTCIHWFRKGLRLHDNPALLAALRDCEELYPVFLLDPHLHNDARMGINRWRFLAGALRDLDCSLRKLNSRL
FVVRGRPEDVFPALFDKWRVTKLTYEYDTEPYSLSRDKTVTALAEEHGVEVVYKISHTLYDTERIIEENNGKA
PLTYNRMQAIVKSLGPPKKPVPAPTMEVMAEAKTPCSEDHEKKYGIPALEELGQGAAALSEEQFPGGEQEALR
RLDEHMQRTGWVCNFEKPQTSPNSLSPSTTVLSPYVTFGCLSARTFWWRLTDVYQGKKHSDPPVSLHGQLLWR
EFFYTASAGIPNFNKMVGNPVCTQVDWDTNSEYLAAWREAQTGFPFIDAIMTQLRQEGWIHHLARHAVACFLT
RGDLWISWEEGQKVFEELLLDGDWALNAGNWQWLSASTFFHQYFRVYSPVAFGKKTDKNGDYIKKYLPLLKKF
PAQYIYEPWKAPRSIQQAAGCIVGKDYPHPIVQHEVISKKNIQRMKLAYAKRSTDDTESPSKKQGTKRKTPSV
VDMLKKKERRK
>Scophthalmus_maxi__XP_035491623.1
MFCIWRRRKVVLTLPPPPRSPRSLAARRVPPRRGDSSSSLSTGQLHLGPPLTCEDVMSGKKRTAKSAPAGKQP
AAKLRKSAPTKEEEEKVERAGGWLQDLVMQQRSKEKEMKFNKKRLRVISDAEKMKPGSEGVLYWMSRDQRVQD
NWALIRAQKLAVKEDLPLHVCFCLSAPKSELSTLRHYGFMLKGLEEVAEECKALDIQFHLLHGSAGEVLPGFV
ADRSLGAVVTDFSPLKEPLQWLEDVKKALPNHIPLIQVDAHNVVPCWVASPKLEYSARTIRGKITNHLPEFLT
DLPLVEKHPHTATRTAKPIDWDKTLASLQIDRAVSEPQWATPGTKGGMAMLESFIDVRLKLFGSQRNDPNANA
LSNLSPWIRFGHLSAQRVALQVQRNGKHASQSVPPFIEELVVRRELTDNYCFYNKKYGSLEGAYEWAQKTLRD
HANDERPFLYTRQQLEEAQTHDKLWNAAQYQMVSEGKMHGFLRMYWAKKILEWMSSPEEALSTALYLNDRYSL
DGQDPNGFVGCMWSICGIHDQGWAERAVFGKIRYMNYKGCTRKFDVPRFERKFCPKNPPK
>Scophthalmus_maxi__XP_035497736.1
MPRPVVDADDSKASVRKWLREVLAGREDPEGFFAMCVSVLGHQETRSQFQSLIQPLSAANRSLHAALTSVYQG
YFHKTEDDELELALALSTLEMKDHQLSTPGTESRLQQPGDTPSQSSSVHLNSVSQPQGSSHTQQADVRGQRKK
KHLAQTGPWGMVCPPQITQETELQKSTNVDKGGKGTTGTSQSAYVSRPPASFSTQKTVLKSDQTMEEGDLNRT
EKPKRSRNRRKRREGGGQQVVGLPSSPSAPSPVLLWFRRDLRLCDNPALIGSLEVGAPVIPVFIWSPEEEEGP
GITVAMGGACKYWLHQALSCFRSSLECIGSHLIFLKANGEGHGVGSSLHTLRELIKETGARTVLANALYEPWL
KERDDVVVSALQEVGVQCKVFHSYCLRDPYSVSTEGVGLRGIGSVSHFMSCCRQNPGSALGVPLEPPGSLPTP
AHWPQGVCLDNLELARMPRRKDGTMVDWAFNIRKSWDFSEEGAHARLEAFLHDGVYRYEKESGRADAPNTSSL
SPYLHFGQLSPRWLLWDAKGARCRPPKFQRKLAWRDLAYWQLTLFPDLPWESLRPPYKNLRWSNDRGHLKAWQ
RGRTGYPLVDAAMRQLWLTGWMNNYMRHVVASFLIAYLHLPWQDGYRWFQDTLLDADVAIDAMMWQNGGMCGL
DHWNFVMHPTDAAMTCDPYGSYVRKWCPELADLPDELIHKPWKCPASMLRRAGVVFGQTYPDRIITDLEERRS
RSLQDVALVRKEFGQFVDKRTGCDLVPLPPRLVSEALGSSHRNGGVAAEAKQFLLPVITRMEFKHQLEDPDAD
AASNPYNAVLKGYVSRKRDETIAFLNERDFTASVMYEGTQRKERLESDYRRMEGLPTPPAPQGRARRTPKAKD
KFSLVPGGVVTSLR
>Scophthalmus_maxi__XP_035499212.1
MVVNSVHWFRKCLRLHDNPGLQEALNGADTVRCVYILDPWFAGAANVGINRWRFLLEALEDLDNSLKKLNSRL
FVIRGQPTDVFPRLFKEWKVTRFSFEYDPEPYGKERDGAIIKMAQEFGVEPVVRNSHTLYNLDRIIEMNNNSP
PLTFKRFQTIVSRLELPRRPLPPVTQQQMDQCRTKISDNHDQLYSIPSLEELGFRTAGLPAAVFRGGESEALD
RLNKHLDKKVWVANFEHPRVNTCSLYASPTGLSPYLRFGCLSCRVLYYNLRELYMKLRKRCSPPLSLFGQLLW
REFFYTAATNNPNFDRMDGNPICVQIPWDQNPEALAKWAEGRTGFPWIDAIMTQLRQEGWIHHLARHAVACFL
TRGDMWISWESGMKVFEELLLDADWSVNAGSWMWLSCSAFFQQFFHCYCPVGFGRRTDPSGDYIRRYIPVLKD
FPNRYIYEPWNAPESVQKAANCVVGVNYPKPMINHAESSRLNIERMKQVYQQLSHYRGLSLLASVPTIQEEAE
PPMTDESQTSSGPGSPLGDEFDTEAAPDSSTVEVCASSTFVSHPELNDPLTLCASSGSQQPPTPPAPLHSAAP
LSPTSPSALTPPPSMSKPSSPLSSSSSSCPAMSPAPSPAAGVLAHQTFPKRKGVARKMRRSISQRQRARLGGG
APSGREEVGGDERMEEETSGYQQ
>Scopus_umbr__NXX54069.1

YCKTVWFASGNKCFFTTPCRFLLQCLEDLDANLRKLNSRLFVIRGQPADVFPRLFKEWNIAKLSIEYDSEPFG
KERDAAIKKLASEAGVEVIVRISHTLYDLDKIIELNGGQPPLTYKRFQTLISRMEPLEMPVETITPEVMEKCT
TPVSDDHDEKYGVPSLEELGFDTDGLPSAVWPGGETEALTRLERHLERKAWVANFERPRMNANSLLASPTGLS
PYLRFGCLSCRLFYFKLTDLYKKVKKNSSPPLSLYGQLLWREFFYTAATNNPRFDKMEGNPICVQIPWDKNPE
ALAKWAEGRTGFPWIDAIMTQLRQEGWIHHLARHAVACFLTRGDLWISWEEGMKVL
>Scopus_umbr__NXX55203.1
MPHRTIHLFRKGLRLHDNPTLLAALESSEVVYPVYILDRKFMTSVMHIGALRWHFLLQSLEDLQKNLCQLGSC
LLVIQGEYKSVLRDHIQKWNITQVTLDAEMEPFYKEMEANIRRLGEELGFEVLSLVGHSLYDTKRILDLNGGT
PPLTYKRFLHILSLLGDPEVPVRNLTAEDFQRCRPPDPGLAECYRVPLPVDLKIPLESLSPWRGGESEGLRRL
EQHLTDQGWVASFTKPRTIPNSLLPSTTGLSPYFSMGCLSVRTFFYRLSNIYAQAKHHSLPPVSLQGQLLWRE
FFYTVASATPNFTKMAGNPICLQISWYEDAERLHKWKTAQTGFPWIDAIMTQLRQEGWIHHLARHAVACFLTR
GDLWISWEEGMKVFEELLLDADYSINAGNWMWLSASAFFHQYTRIFCPVLFGKRTDPEGQYIRKYLPILKNFP
SKYIYEPWTASEEEQKQAGCVIGQDYPFPMVNHKEASDRNLQLMKRVREEQYRTAQLTRDDTDDPMEMKVKHD
HSEENVSKGKVARMTE
>Scopus_umbr__NXX59662.1
MQHSSIHWFRKGLRLHDNPALLAAATDCRHLHPLFILDPSRGRAGPNARRFLLDALRDLDGSLREMGSRLFVV
RGRPEEVFPRLFHAWGTMRLTFEVDTEPPARQRDAAVAELAARHGVEVIREVSHTLYDTERVLALNDGKAPLT
YKRLQSLLASLGPPEKPAPALTWEHLRGCRTPCQVRHDADYGVLTLEELGQDPAEVGPHLYPGGETAALARLD
TLMERTAWVCSFKKPETEPTSLSPSTTVLSPYLKFGCLSVRTFWWRLAEVYQGREHSRPPVSLHGQLLWREFF
YTAGAGIPNFDRMVGNPVCLQVDWDDNPQHLRAWREGQTGYPFIDAIMTQLRAEGWIHHLARHAVACFLTRGD
LWVSWEEGLKVFEELLLDADWSLNAGNWLWLSGSAFFHRYFHVYSPVTFGKKTDRDGAYIRKYLPVLKDFPAE
YIYEPWKAPRAVQERAGCLVGTHYPRPIVEHRAASERNLGRMKAAR
>Scopus_umbr__NXX62443.1
RFLLQSLEDLDNSLRKLNSRLFVVRGQPTDVFPRLFKEWGVTRLTFEYDSEPFGKERDAAIVKLAKEAGVEVV
IENSHTLYDLDRIIELNGHKPPLTYKRFQAIISRMELPKKPVSTVMSQQMETCKVDIQENHDDVYGVPSLEEL
GFPTDGLAPAVWQGGETEALARLDKHLERKAWVANYERPRMNANSLLASPTGLSPYLRFGCLSCRLFYYRLWE
LYKKVKRNSTPPLSLYGQLLWREFFYTAATNNPKFDRMEGNPICIQIPWDRNPEALAKWAEGKTGFPWIDAIM
TQLRQEGWIHHLARHAVACFLTRGDLWISWESGVRVFDELLLDADFSVNAGSWMWLSCSAFFQQFFHCYCPVG
FGRRTDPSGDYVKRYLPKLKGFPSRYIYEPWNAPESVQKAAKCIIGVDYPKPMVNHAETSRLNIERMKQIYQQ
LSRYRGLCLLASVPSCAEDLSGPVTDSVSGQGCSTSTAVRLSQADQASPKRKHEGAEELCTEELYKRAKVACL
PAPEIPGKSL
>Scopus_umbr__NXX63304.1
DNWAFLYAQRLALKQELPLHVCFCLVPKFLDATIRHYGFMLRGLQGVAEECAELNIPFHLLLGYAKDVLPAFV
AEHGAGGLVTDFCPLRLPRQWVEDVRERLPEDVPFAQVDAHNIVPCWVTSPKQEYSARTIRGKIHAQLPEFLT
EFPPVIHHPYPPSCPAEPIAWEACYSSLQVDHTVKEVEWATPGTAAGLAVLQSFITERLKSFGSHRNDPNKAA
LSNLSPWFHFGQVSTQRAILEVQKHRGKYKESVDAFVEEAVVRRELAENFCYYNENYDSVQGAYDWAQTTLKL
HAKDKRPFLYELQELEQGTTHDPLWNAAQLQMVREGKMHGFLRMYWAKKILEWTRSPEEALRFAIYLNDRYEL
DGTDPNGYPTLSPCPAGCLWSICGIHDQGWAERAIFGKIRYMNYAGCKRKFDVGQFERRYAPTC
>Scopus_umbr__NXX63934.1
GEKIHQGLAQLFLLFSSSVLKVDYDVCSNYGNWLYSAGIGNDPRDNRKFNMIKQGLDYDGNGDYVRLWVPELQ
DIKGADIHTPWVLNSAALSQAGVTLGETYPQPVVTAPEWSRHITQRPQGRSPHPRGRRGPGRTPMQHKDRGID
FYFSRKKDV
>Scyliorhinus_cani__XP_038636618.1
MGGHSIHWFRKGLRLHDNPALLDALLGSDTIRCVYIVDPWFAGSSSVGINRWRFLLQCLEDLDASLRKLNSRL
FVVRGQPADVFPRLFKEWKISRITFEYDSEPFSKERDAAIKKLAIEAGVEVIVQISHTLYDLDMIIDRNGGQP
PLTYKHFQTLVSRMESIDMPVGTMTAETIGKCITPVLDDHDDKYGVPTLEELGFDTEGLPSAVWPGGETEALT
RLERHLERKAWVANFERPGMNSTSLLASPAGLSPYLRFGCLSCRLFYFKLTDLYKKVKKNSSPPLSLYGQLLW
REFFYVAATNNPRFDKMEGNPICVQVPWDWNPEALAKWAEGMTGFPWIDAIMTQLRQEGWIHHLARHAVACFL
TSGDLWISWEEGMKIFEELLLDADWSVNAGSWMWLSCSSFFQQFFHCYCPVGFGRRTDPNGDYIRRYLPVLRD
FPAKYIYDPWNAPESVQKAAKCIIGVHYPKPMVNHAEASRLNIERMKQIYQQFSRYRGLGLLASVPSNPNASG
GAMGYFPGESTHGFSSITGRSCLGNSPSSGKQPSAWKEMPGLGPKVQRQSIE
>Scyliorhinus_cani__XP_038663740.1
MVLNSVHWFRKGLRLHDNPALQRSLSGADTVRCIYILDPWLVASSNIRINRWRFLLQCLEDLDSSLRKLKSRL
FVVRGQPADVFPRLFKEWNMTQLTFEYDSEPFGKARDATVMKLAENAGIEVIVRNSHTLYEVDRIIEANDNKP
PLTFKRFQTIIKQLELPKAPVNPVTLDQMEKCTRTIQENHDQMYGIPSLEELGFDTSELGPAIWQGGETEALA
RLSKHLEQQTSIANYEKPKLNADFLLSSPTGLSPYLRFGCLSCRTFYYTLLRFYRMVKKQGNPPLSLYRKLLW
REFFYTAATNNPNFDKLEGNPICMQIPWDKNQQALTKWAHGQTGFPWIDAIMKQLRQEGWIHQLARRAVACFL
TRGDLWISWEEGMKVFDEMLLDADFSVNAGSWMWLSCSAFFQKFFQFYCPVDFGRRTDPSGDYVRRYLPVLRG
YPAKYIYEPWNAPIEVQKAANCIVGIHYPKPMINHREASRLNIERMKQIYPKLSHQERFCSCLPNHVKTAAIM

EKGNKPEDTEIEDGCKPAKIPCLQSPEGDGRNEEEAMAPEYVPEKVKKAEKKLEENPYDLDAWSILIREAQNQ
PIDKARKTYERLVTQFPSSGRFWKLFIEAENPYCQLLLGFELSIPEIFMVMYLIDYCFLD
>Scyliorhinus_torazame_GCB62468.1_hypothetic
MMLNSVHWFRKGLRLHDNPALQRSLSGADTVRCIYILDPWLVASSNIRINRWRFLLQCLEDLDSSLRKLKSRL
FVVRGQPADVFPRLFKEWNMTQLTFEYDSEPFGKARDATVMKLAENAGIEVIVRNSHTLYEVDRIIEANDNKP
PLTFKRFQTIIKQLELPKAPVNPVTLDQMEKCTRTIQENHDQMYGIPSLEELGFDTSELGPAIWQGGETEALA
RLSKHLEQQTSIANCEKPKLNADYLLSSPTGLSPYLRFGCLSCRTFYYTLLRFYRMVKKQDNPPLSLYKKLLW
REFFYTAATNNPNFDKLEGNPICMQIPWDKNQQALTKWAHGQTGFPWIDAIMKQLRQEGWIHQLARGAVACFL
TRGDLWISWEEGMKVFDEMLLDADFSVNAGSWMWLSCSAFFQQFFQFYCPVDFGRRTDPSGDYVRRYLPVLRS
YPAKYIYEPWNAPIEVQKAANCIVGIHYPKPMINHLEASRLNIERMKQIYPKLSHQERFCSCLQNHEKTAGIM
EKGNKPEDTEIEDGCKPAKIPCLQSPEGDGRNEEEAMFLDLTHNF
>Scyliorhinus_tor_GCB77202.1_scyTo
VAVSPRAPQQCVPSPSHMETHSIHWFRKGLRLHDNPALMEALRGASTVRCVYILDPWFAGSSNVGVNRWRFLL
QCLEDLDASLLELNSRLFVIRGQPADVFPRLFKEWNINRLTFEYDSEPFGKERDAAIKKLASEAGVEVIVKIS
HTLYDLDKIIELNGGQPPLTYKRFQTLISQLDSPEPPVETISEGRLGKCAVLVLDDHNEKYRVPSLEELGFDI
EGLHSAVWPGGETAALTRLERHLERKAWVASFERPRMNAFSLLASPTGLSPYLRFGCLSCRLFYFKLTDLYKK
IKKTNSPPLSLYGQLLWREFFYTAATNNPCFDKMEGNPICVRIPWDKNPVSLAKWAEGKTGYPWIDAIMTQLR
QEGWIHHLARHAVACFLTRGDLWISWEEGMKFFHCYCPVGFGRRTDLSGDYVRRYLPILKGFPAKYIYDPWNA
PDSVQVAAKCIIGVHYPKPMVVHAEASRLNIERMKQIYQQLSRYRGLGLLASVPSNQNGNGRTMYSLRDQLSE
NSGIARSHTVGAIGKRELEHDTTVGDEVESLAQKIRRHCDDELTRGASSV
>Selaginella_moe_bluelightPa_XP_024526689.1
MANAADGDGLVDLELIMDANLSSSSIAHATLGAAPPPEELEVELHSEVVDKPAQRHFIPCGNLASLSLALNTL
MPLSSKVGVLPRKLLAKPPANLPSQKKANSPIPVVPSSKLKFGLDVEPQQQQQQSSASKICSSATAFPDYQML
PRSGRRYGNPAAGAGMRRASIVWFRNDLRVHDNEALAAANKESLSVIPVYCFDPKDYGKSASGFDKTGPYRAK
FLVECVANLRDNLRERGSELVVRIGNPVEVLSTIAKAVGADGLYAHQEVSSEELGMEDKVTSALKDQNVDVKF
FWGSTLFHVDDLPFKVEDMPSNYGGFRDKVKDVQVRAATEAPKQLKGLPSQGDVKAGDIPSLQELGLSPVSAA
GQQEKHKRNRMIGGEVEALKRLKSFSLVSPPPSKGGKENSIYGANFSSKISPWLAMGCLSPRKMFEDLKKTTS
RLVPKPTPTSVAPSSAASGDSGMQWLVFELLWRDFFRFVTKKHGAAKKTQTEAVPAMACA
>Selaginella_moe_XP_002969486.2_cry-1_
MGSSTTIVWFRRDLRVEDNPALAAASKAGAVVPLFIWCAEEEGEFRPGRVSRWWLKQSLMHLNSSLTKLGCPL
LMQKSRGSSLNALLQIARDTKATQVFYNHLYDPVSLIRDHHVKHGLSQYGITVQTFNADLLYEPWELFDARGN
PYTHFQLFWQRYLGMDHEPDGPLLPPKRLSPPAEPGLGHLGLPAEELGLEDDFEKSSNAMLARTWTPGWSNAN
KSLDAFLAGPLADYGDNRGKVDGGTTSMLSPHLHYGELSIRKVFHAVRSKQLLWITDGRQPAERSASLFLRAL
GYREYSRYLCFHFPFTHERSLLGHLRHFPWRVDEGFFKAWRQGRTGYPLVDAGMRELWATGWLHNRIRVIVSS
FCVKFLQLPWRWGMKYFWDTLLDADLESDILGWQYISGSLPDGHELDRMEIPHIEGRRFDPDGNYVRKWLPEL
SRLPTEWIHEPWNAPPSVLCAAGIELGCNYPRPVVEIAAARERLQRGLRDMWEHNSNSISNGSGTPEPPEINS
DNGEQQIRDYGGSNGHQCHESQRRSNDQMVIERQNQQQQRDRCSDTSHPDQIVPTLLPDFNTLRSGSSRIHH
HHHHHHHHLQPQQHNFALAGPGEDFHPANHRQEQHPQQQFPQLQLQQPQQQHQQAVEMMDSDVNSTAESSSA
FRRNYDGYATAAAVWFPTHSNRRRVDFSAYL
>Selaginella_moe_XP_002972041.2_photolyase
MSQLSGKKEVAGALDAAAAGAKKQKLDADGGGFLGEGLRSSIGVNPRRVRVLSQGDESHSGPVVYWMSRDQRS
RDNWALLYAAEQARKRDTPVAVVFNLVESFLEAKARHFGFLLRGLRVVEKNLEKLGIAFFLLRGRPEDTIPAF
VEACNASILVLDFSPLRIGRTWRTGVVSRLGADTTTAVHEVDAHNVVPVWIASDKLEYAARTIRLKIQRQLPE
FLVDFPTLEPFSKPWPGVAQEKIDWDSLIDEVLRAGDEVPEVDWCEPGEDIALERLAGFLSSRLQRYSAERND
PSKLERLSDLSPYFHYGQLSPQRCAFEVRKARKLHMQSVDSFLEELIIRRELSDNFCYYQPNYDNIQGAWEWA
RNSLLEHANDKREHIYTRQQLEDGKTKDKLWNASQLEMVHRGKMHGFMRMYWAKKILEWTESPEDALAIAIYF
NDKYQLDGRDPNGYVGCMWSICGIHDQGWKERPIFGKIRYMNYEGCKRKFNVDGYIMYVNKLVAEVKKRRKSQ
ERTLEC
>Selaginella_moe_XP_024537592.1_cry_DASH,_
MADAVESMIQRYSSAGSSRNGKGVSIMWFRNDLRIVDNEALFKAWASSSSVLPVYCVDPRHFGTTHYFKFPKT
GELRAQFLLESLVDLKKNLKKRGLDLVIRSGNPEFILPLLVEAVGAHTIYAQEETCSEELQVEKAVKKSIGTK
AKLELVWGTTMYHLNDLPFTTARLPDVYTQFRKAVESSSRVRPCLKLPQTLGPLPDSLGEKVGDLDKVPNLSD
LGLRPREQSSLAAMQFTGGETAAIQRLCEYFWTKNCLRNYKETRNGMLGADYSTKFSPWLASGSISPRFIYEE
VKRYEKERTANNSTYWVLFELIWRDYFRFLSMKYGNSIFHLEGPRKVRTKSWSQDKTLFEAWRTGTTGYPLID
ANMRELYATGFMSNRGRQIVCSFLVRDMGIDWRMGAEWFESCLLDYDPCSNYGNWTYGAGVGNDPREDRYFSI
PKQAQTYDPDGSYVSHWIPQLQELPTDARHSPKVGYLKKIVPLRFSNPGRTYKTSANFRK
>Selaginella_moe_XP_024540344.1_(6-4)DNA_p
MALLWFRKGLRLHDNPSLEAACHGSRNVYPVFVLDPWFLAPDPRASSPGSARVGINRIQFLLESLQDLDRNLS
SRGSKLLLVHGNPIQVIPELLDKWRIRKLCFEFDTEPYALDRDAKIKEHAKDKGIELHCPVSHTIFNPDLLIA
KNGGKAPLTFQSFCKNLVPATKPIGNGPSAIPPTGDLHGIKVVPVPTLEELGYADFHEDFSPFRGGETVALTR

LEDSLANEKWVCEFEKPKGDPTAFIKPATTVLSPYLKFGCLSSRLVYSRVKEVYSRAKSFTSPPVSLEAQLLW
REFFYTAAYATANFDKMVGNPICKQIPWKDDDELLSAWRDGRTGYPWIDAAMTQLRKWGWMHHLARHAVACFL
TRGDMFVYWEKGRDVFDRLLIDADWSINNGNWLWLSASAFFNQYHRIYSPITFAKKYDRQGNYIKHFLPVLKD
MPQEFIYEPWKAPAEIQRKAKCIIGKDYPTPVLDHAAASKICRERMGAAYALSKSSHGNVSREQVDELQDTLT
DVPTSSTSKNKRQKQSRITDYSA
>Sepia_off_QHA94772.1_cry_6_vari
MNSCSNKDMDLEIKKKISVHWFRHGQRIHDNPALVDAVNDSDEFYPIFIFDGKVAGTELCGYNRWRFLLENLK
DLDESFSKFGGKLYCFHGQPVDIFQKLFKEWNVNYITAEEDPEPIWQERDESVKKLCEKSGVTCKFFTSHTLY
SPQEIIDKNGSTPPLTLELFQLVITSLGPPLRPLPQPNLQDINMPVPKDLKKFALPTLDFFGIEPEFEEQKNP
LNVFVGGETRALNLLKARLEKEKSSFEQDSCQPNHQEQPQLLAKAVSLSPYLRFGCVSIRRTYWGICDMYREV
HNKEPPSEVVCQLYWREYFYVMSVGNLNYDKMEGNPICLQINWQKNDEHLKKWELGQTGFPWIDAIMNQLRFE
GWNHHVGRHSVSCFLTRGDLWISWEDGLKVFLKYQLDADWSVCAGNWMWVSSSAFEKALQCPTCYSPVMYGMR
MDRNGEFVRMYVPVLKDMPLKYLFCPWKAPLEIQKNANCIIGKDYPEPICSHRDMSRVNMSKMYRVKEKLLKA
GIPHCAPTGEMEVWKFAWLPPIEHHDLAHNL
>Sepia_off_QHA94774.1_cry_123
MSGEREKNVIHWFRKGLRLHDNPALKEALKGANTIRCIYTLDPWFAGSSQVGINKWRFLLNCLEDLDASLRKL
NSRLFVVRGQPADVFPQLFKEWNITTLSFEEDPEPFGKDRDAAICTLVRDAGIEVIIKTCHTLYDSKAILEKN
GGRPPLTYNKFQKILAEMDLPPRPLETVTASDFGNCRTPLSVDHDEKYGVPTLEELGFDTEGLGQTNFHGGET
EALARLDRHLERKAWVASFEKPKMTPQSLFPSHTALSPYLRFSCLSPRLFYWKLTELYTKIKKKSNPPLSLHG
QLLWREFFYTAASNNPRFDQMVGNPVCVQIPWDINQEALAKWAEGMTGFPWIDAIMKQLRKEGWIHHLARHSV
ACFLTRGDLWISWEDGMKVFEELLLDADWSVNAGMWMWLSCSSFFQQFFHCYCPVGFGKRIDPNGDFIRHYLP
ILKRFPAKYIYEPWNAPESVQKTAKCIIGKDYPVPMVNHSEVSRINMERMRQVYHRLTMRSSATIPKQIHSRE
GLQYSKHSKPMDICFHGTSKHDKRYDQHFMLKSY
>Sepia_phar__CAE1232360.1
MSGEREKNVIHWFRKGLRLHDNPALKEALKGANTIRCVYTLDPWFAGSSQVGINKWRFLLNCLEDLDASLRKL
NSRLFVVRGQPADVFPQLFKEWNITTLSFEEDPEPFGKDRDAAICTLVRDAGIEVIIKTCHTLYDSKAILDKN
GGRPPLTYNKFQKILAEMDLPPRPLETVTASDFGNCRTPLSVDHDEKYGVPTLEELGFDTEGLGQTNFHGGET
EALARLDRHLERKAWVASFEKPKMTPQSLFPSHTALSPYLRFSCLSPRLFYWKLTELYTKIKKKSNPPLSLHG
QLLWREFFYTAASNNPRFDQMVGNPVCVQIPWDINQEALAKWAEGMTGFPWIDAIMKQLRKEGWIHHLARHSV
ACFLTRGDLWISWEDGMKVFEELLLDADWSVNAGMWMWLSCSSFFQQFFHCYCPVGFGKRIDPNGDFIRHYLP
ILKRFPAKYIYEPWNAPENVQKTAKCIIGKDYPVPMVNHSEVSRINMERMRQVYHRLTMRSSATIPKQIHSRE
GLQYSKHSKPMDICFHGTSKHDKRYDQHFMLKSY
>Sepia_phar__CAE1267008.1
MQARIICFFLETGLLILFFWCFSARKMDSSFNKDMDIEIKKKISVHWFRHGQRIHDNPALVDAVKDCDEFYPI
FIFDGKVAGTELCGYNRWRFLLENLKDLDESFSKFGGRLYCFHGQPMDIFQKLFKEWNVNYITAEEDPEPIWQ
DRDNSIKKLCEKSGVTCKFFTSHTLYSPQDIIAKNGGTPPLTLELFQLVITSLGPPLRPLPQPDLQDVNMPVP
KNFKKFALPTLEFFGIEPEFEEQKKPLNVFVGGETRALNLLKARLEKEKASFEQDSCQPNHQEQPELMAKAVS
LSPYLRFGCVSIRRTYWGICDMYTKVHNKEPPSEVVCQLYWREYFYIMSVGNLNYDKIENNPICLKINWQKND
EHLRKWELGQTGYPWIDAIMNQLRFEGWNHHVGRHAVSCFLTRGDLWISWEDGLKVFLKYQLDADWSVCAGNW
MWVSSSAFEKALQCPTCYSPVMYGMRMDRNGEFVRTYVPVLKDMPLKYLFCPWKAPLEVQKAANCIIGKDYPE
PICSHRDMSRVNMSKMYRVKEKLLKADIPHCAPTGEMEVWKFAWLPPIEHHDLAHNL
>Sepia_phar__CAE1268455.1
MLTDVDAFPQDANGGVLYWMVRDQRLQDNWAMLFAQRLALKEKTSLHVCFCLVSPFMEATERHYDFVIRGLQE
VEKPVDWMVAKKKLKMDTSVKPVDWAIPGERAGLKTLESFCNERLKYFATERNDPTKSSLSNLSPWLHFGAYD
WAKKSLKEHEKDKREYIYTQEQLENANTHDQLWNAAQIQLLTEGKMHGFLRMYWAKKILEWTANPAEALCVAI
YLNDRYSLDGMDSNGYVVLGHKWQGNMPGAEFADDAGVTFTRALLLGVCHSGDAERLPANPIPCEVLQLNDRE
KINSSFPLSIVLSSPLSFTR
>Seriola_lala_dorsalis_XP_023253166.1
MVVNSVHWFRKCLRLHDNPALQEALNGADTVRCVYILDPWFAGAANVGINRWRFLLEALEDLDSSLKKLNSRL
FVIRGQPTDVFPRLFKEWKVTRLTFEYDPEPYGKERDGAIIKMAQEFGVETIVRNSHTLYNLDRIIEMNNNSP
PLTFKRFQTIVSRLELPRRPLLPITQQQMEKCHTKIADNHDQLYSIPSLEELGFRTEGLPQAVWRGGESEALD
RLNKHLDKKVWVANLEHPRVNTCSLYASPTGLSPYLRFGCLSCRVLYYNLRELYMKLRKRCSPPLSLFGQLLW
REFFYTAATNNPNFDRMEGNPICVQIPWDQNPEALAKWAEGRTGFPWIDAIMTQLRQEGWIHHLARHAVACFL
TRGDLWISWESGMKVFEELLLDADWSVNAGSWMWLSCSAFFQQFFHCYCPVGFGRRTDPSGDYIRRYIPILKD
YPNRYIYEPWNAPEPVQKAANCVVGVDYPKPMINHAEGSRLNIERMKQVYQQLSHYRGLSLLASVPTIQEEAE
PPMTDESQTSSGPDSPPRDPVDNEAAGCSTAPDSSTVCASSTFALHPDLEDTLNCRPFQTPCTFSHTQASAAV
TSTSANHPLSLAASPSLASTPIQGSLSRSKPSSPSSSCPTLSSSSSPVLTLAQTFPKRKSLARKVRRSQRQRG
RQICTPSGREGERRAEEEERDDTGGDKRMEEDVEPDEERMEEETSEKLAGLQQ
>Seriola_lala_dorsalis_XP_023259636.1

MPPSAGGAEDSKTLVRKLLREVLVGREDPEGFFAMCLSVLGHQETRSQFPSLIRPLSTANCSMYSTLTGIYQE
YFSVVSECLLNTEDDELEVALALSVLEMKDHQMSAPSQESRLQQPKDRPNQSSSVQLTSVSQPQGSSHTQLAD
VAGRKKNSHSAQSGPWGMVCPPQTTRETEPQKSTHVDKYNKWTPGTSQTESVSKPSASFSKQELVIEGGQMTK
EGDLNQSGKPKRSKNRRQRRKAGGQQVVGLPCSPSAPQPVLLWFRRDLRLCDNPALIGSLEVGAPVIPVFIWS
PEEEEGPGITVAVGGACKYWLHQALSHFCSSLESIGSHLIFLKADGGGDGVGSSLCTLKELIKKTGARTVLAN
ALCEPWLKERDDVVVSDLQKDGVECRMFHSYCLRDPYSVSTEGVGLRGIGSVSHFMSCCRQNPGSALGVPLDS
PASLPTPAHWPQGVSLDTLGLARMPRRKDGTTIDWAANIRKSWDFSEEGAQAQLEAFLHDGVYRYEKESGRAD
APNTSCLSPYLHFGQLSPRWLLWDAKGARCRPPKFQRKLAWRDLAYWQLTLFPDLPWESLRPPYKALRWSSDR
GHLKAWQRGQTGYPLVDAAMRQLWLTGWMNNYMRHVVASFLIAYLHLPWQEGYRWFQDTLVDADVAIDAMMWQ
NGGMCGLDHWNFVMHPVDAAMTCDPYGSYVRKWCPELADLPDELIHKPWKCPASMLRRAGVVFGQTYPDRIIT
DLEERRSQSLQDVAVVRKEFGHYVDKRTGCDLVPLPPRLVSEALGLSHSNGGVVAEGKQFLLPVITRMEFKHQ
LEDPDADAASNPYNAVLKGYVSRKRDETIAFLNERDFTASVMYEGAQRKERLEKDYRRMEGLPQPAAPRGRAR
RTPTAKDRFSIVPGGAVTSLR
>Seriola_lala_dorsalis_XP_023263640.1
MSTSRTIICLLRNDLRLHDNELFHWAQRNAEYIVPLYCFDPRHYAGTYNYNLPKTGPFRLRFLLESIRDLRNT
LLNKSSNLVVRRGKPEDVVAELIKQLGSVSTVAFHEEVTSEELNVEKKVKDVCAQMKVKVHTCWGSTLYHRDD
LPFHHISRLPDVYTQFRKAVETQSMVRPAFPTLEQLKPLPSGLEEGAVPTAQELQQTEPLTDPRSAFPCSGGE
SQALARLKHYFWDTDAVATYKETRNGLIGVNYSTKFAPWLAMGCISPRYIYHQIKQYERERTANQSTYWVIFE
LLWRDYFKFVAVKYGNRLFQLKGLQDKSVPWKKDMKLFNAWKDGRTGVPFVDANMRELAMTGFMSNRGRQNVA
SFLTKDLGLDWRMGAELFEYLLIDHDVCSNYGNWLYSAGIGNDPRENRKFNMIKQGLDYDNNGDYVRQWVPEL
QGIRGADVHTPWTLSTAALSHAHVSLDETYPTPIVMAPEWSRHVNKKSSGTGPSPRGKRGPSHTPKQHRDRGI
DFYFSRSKNL
>Seriola_lala_dorsalis_XP_023267685.1
MAHTCIHWFRKGLRLHDNPALMAALRDCKELYPVFILDPYLHTNTHVGINRWRFLIGALKDLDCSLRKLNSRL
FVVRGKPEDVFPKLFNKWKVTKLTYEYDTEPYSLSRDKKVMELAKEHEVEVIYKISHTLYNIDRIIEENNGKA
PLTYNRMQAIVKTLGRPKKPIPAPTMEDIKDVKTPCSENHEKKYGIPALEELSQDTADLAEELFPGGEQEALR
RLDEHMKRTGWVCNFEKPKTSPNSLSPSTTVLSPYVTFGCLSARTFWWKLTEVYQGKKHSDPPVSLHGQLLWR
EFFYMASVGIPNFNKMEGNPVCTQVDWDANTEYLAAWREARTGFPFIDAIMTQLRQEGWIHHLARHAVACFLT
RGDLWVNWEEGQKVFEELLLDGDWALNAGNWQWLSASAFFHQFFRVYSPVAFGKKTDKNGDYIKKYLPLLKKF
PVEYIYEPWKAPRNIQQAAGCIVGKDYPHPIVQHEVISKKNIQRMKLAYAKRSSDHTESPSKKQGAKRKAPSV
VDMLTKKERRK
>Seriola_lala_dorsalis_XP_023270531.1
MLLCIWRSSSFSSAPRRLLLRLVIHSNLRPCLINSLFTKSFTSETIMSDRKRKATSAPAGKKPTAKQQKLVPLK
EEKKERAGGWLQEKLKQRRTEKKEMKFNKKRLRFVSDTEKIKQGSEGVLYWMSRDHRVQGEHTPSSKTIRHLV
WLHLQDVIDMPISVFLSSPFFTSQPVDWDKTLVSLQVDRTVGEPEWATPGTKGGIAMLESFIDVRLKLFDTKR
NDPNADALSHLSPWIRFGHLSAQRVALQVQHSGKKAGQSVSSFIEELVVRRELTDNFCFYNKKYDSVEGASEW
AQKTLEVHAEDNRPYLYTRQQLEEAKTHDKLWNAAQYQMVSEGKMHGFLRMYWAKKILEWTSSPKEALSIALY
LNDRYELDGQDPNGFVGCMWSICGIHDQGWRERAVFGKIRYMNYKGCTRKFDVAQFERKYCPKKL
>Seriola_lala_dorsalis_XP_023271280.1
MVVNTIHWFRKGLRLHDNPSLRDSIRGADTLRCIYILDPWFAGSSNVGINRWRFLLNCLEDLDASLRKINSRL
FVIRGQPTDVFPRLFKEWQITHLSYEYDSEPFGKERDTAIQKLANEAAVEVIVRISHTLYDLEKIMELNGGQP
PLTYKRFQALINHMDAVELPAGTITSEVIKKCATPIGDDHDDKFGVPSLEELGFETEGLTTAVWPGGETEALM
RLERHLERKAWVANFERPRMNANSLLASPTGLGPYLRFGCLSCRLFYFKLTDLYRKVKKNSTPPLSLYGQLLW
REFFYTTATNNPCFDKMEGNTVCVQIPWDRNPEALAKWAEGRTGFPWIDAIMTQLRQEGWIHHLARHAVACFL
TRGDLWVSWEEGMKVFEELLLDADWSVNAGSWMWLSCSSFFQQFFHCYCPVGFGRRTDPSGDYIRRYLPILRG
FPARYIYDPWNAPEEVQKAAKCIIGVHYPKPMVNHAEASRINIERMKQIYQQLSCYKGLGLLATVPDNPSNGP
RGSNAAGVNRGTSHGPGGPSEALSIQEGTSRTERGPSTQKRRYEEAPSKSSSKSWRQSK
>Siganus_gutt__BAL72538.1
MVVNTIHWFRKGLRLHDNPALRDSIRGADTLRCIYILDPWFAGSSSVGINRWRFLLDCLEDLDASLRKLNSRL
FVIRGQPTDVFPRLFKEWQITRLSYEYDSEPFGKERDAAVHKLASEAGVEVIVRISHTLYSLAKIIELNDGHP
PLTYKRFQDIISQMDAVEHPAETVTQRDLDKCTTPISKNHSDKFGVPSLEELGFETEGLSAAVWPGGETEALM
RLEQHLEWRESVLNHGCSHIDARSLLANPTVLCPYLRFGCLSCRLFYFRLTELHKKIKQNNIPPPYFYDQLLW
SEFLYTVSTNNPCFDKMEGNPVCIQIPWDRNPEALAKWAEGRTGFPWIDAIMTQLKQEGWIHSLAKYSVACFL
TRGDLWISWEEGMKAFEELLIDADWSMNAGSWMWFSCSSFFRQFFHCRCPVESGRRKDPQGDYIRRYLPVLRG
FPAKYIYDPWNAPEEVQKEANCIIGVHYPKPMVNHAEASCINIERMKQIYQQLSGYRGLALLATVPVHPNRGT
SGSSVARFNPGTGQVAEGSSENLSTQEGTSGTGRVQSAKKRRREEDSLESSSKKNPEAPVYERPKLSAKS
>Siganus_gutt__BAL72539.1
MVVNSVHWFRKGLRLHDNPALQEALNGADTVRCVYILDPWFAGSANVGINRWRFLLEALEDLDNSLKKLNSRL
FVVKGQPTDVFPRLLKEWKVTRLTFEYDPEPYGKERDGAIIKMAQEFGVETIVRNSHTLYNLDRIIEMNSNNP
PLTFKRFQTIVRRLELPRRPLAPITQQQMDKCRTKIADNHDQLYSIPSLEELGFRTEGLPAAVWRGGESEALD

RLNKHLDKKVWVANLEHPRVNTCSLYASPTGLSPYLRFGCLSCRVLYYNLRELYMKLCKHSSPPLSLFGQLLW
REFFYTAATNNPNFDRMEGNPICVQIPWDQNPEALAKWAEGRTGFPWIDAIMTQLRQEGWIHHVARHAVACFL
TRGDLWISWESGMKVFEELLLDADWSVNAGSWMWLSCSAFFQQFFHCYCPVGFGRRTDPSGDYIRRYIPILKD
YPNRYIYEPWNAPESVQKAANCVVGVDYPKPMINHAEGSRLNIERMKQVYQQLSHYRGLSKSTHQFLPV
>Silurus_meri__KAF7698971.1
MGVNAVHWFRKGLRLHDNPSLRECIVGADTLRCVYILDPWFAGSSNVGINRWRFLLQCLVDLDASLRKLNSRL
YVIRGQPTDVFPRLFKEWKITHLSYEYDSEPFGKERDAAIKKLASKAGVMVNVRISHTLYDLEKIIELNGGQS
PLTYKRFQALISRMDGVEMPTETITAETLGKCIMPIADDHDEKFGVPSLEELGFETDSLPTAVWPGGETEALT
RLERHLERKAWVANFERPRMNANSLLASPTGLSPYLRFGCLSCRLFYYKLTDLYRKVKKNSVPPLSLYGQLLW
REFFYTAATNSPCFDKMEGNSICVQIPWDRNPEALAKWAEGRTGFPWIDAIMAQLRQEGWIHHLARHAVACFL
TRGDLWISWEEGVKVFEELLLDADWSVNAGSWMWLSCSSFFQQFFHCYCPVGFGRRTDPNGDYIRQYLPVLKG
FPAKYIYDPWNAPESVQKLAKCIIGTHYPKPMVNHAEASRINIERMKQIYQQLSCYRGLGLLASVPSNASGNC
DGAGACLNSADSTQDSNAAGPDGLKSWRQDIKQRQSDRGAATLKRHGEDTGSSTGCKIQKL
>Silurus_meri__KAF7705967.1
MYAGFSVFFRLRRVLEVRRFAVIMSEKKQKNLKRKAEPMKGPEKLSAPRGAEREAGWLQDTVAEIRNSFECKF
NTKRLRFLSEAQKVRQGCGAVLYWMARDQRVQDNWALVYAQELALEEKLPLHVCFCLVPRYLESTYRQYAFML
RGLQEVAKECTSLNVQFHLLRGEPELTLPEFVKHWDVGAVVTDFNPLRLPLQWTERVKKQLPSDVPFIQVDAH
NVVPCWEASSKLEYAARTIRGKITKLLPEFLTEFPLVDAHPYTSTKTPKKVDWEEVMSSLVVDRSVGEVQWAE
PGTAGGVAMLESFIEQRLRIFATERNNPNANALSQLSPWIHSGQISAQRALLEVRRWGKRSPESVSSFTEELL
VRRELCDNFCFYNENYDNFSGAYDWAKKTLQDHTKDRRSYVYSQEQLENAETHDPLWNAAQRQLLLEGKIHGF
MRMYWAKKILEWTSSPEEAVNISIYLNDRYSMDGCDPNGYVGCMWSVCGIHDQGWAERPVFGKVRYMNYAGCK
RKFDVAQFEKRYAVKKA
>Silurus_meri__KAF7707629.1
MVVNSVHWFRKGLRLHDNPALQEALNGADTARCVYVLDPWFAGAANVGVNRWRFLLESLEDLDTSLRKLNSRL
FVVRGQPADVFPRLFKEWNVTRLTFEYDSEPYGKERDGAIIKMAQEFGVETIVRHSHTLYNPDRIIEMNNNHP
PLTFKRFQAIVSRLELPKKPLPTITPGQMAGRSSQLSENHNKRYGVPSLQELGFKPQGEAVPVWRGGETEALE
RLNKHLDRKAWAASFERPRIKAHSLFASPTGLSPYLCFGCLSSRVLYYHLRELYMKLRRRSSPPLSLFRQLLW
REFFYTAATNNPSFDRMEGNPICVQIPWDQNPEALAKWAEGRTGFPWIDAIMCQLRQEGWIHHLARHAVACFL
TRGDLWISWENGMKVFEELLLDSDWSVNAGSWMWLSCSAFFQQFFHCYCPVGFGRRIDPSGDYIRRYIPELKD
YPSRYIYEPWNTPESIQKAANCIVGVHYPKPMLNHAESSRLNIERMKQVYQQLSHYRGLSLLASVPTIQEEAE
PSMSDDSQASSSGSISMSHQPQPEFEPQPPQTDPDPDPDQDPDPEPSSAPAAVRKRGRSSEPQNSQNPKVQHT
SQTIAEQLRTNSKE
>Siniperca_chua__XP_044032540.1
MSTSRTIICLLRNDLRLHDNELFHWAQRNAEYIVPLYCFDPRHYAGTYNYNLPKTGPFRLRFLLESIRDLRNT
LLNKGSNLVVRRGKPEEVVADLIKQLGSVSTVAFHEEVTSEELNVEKRVKDVCAKMKVKVHTCWGATLYHRDD
LPFHHISRLPDVYTQFRKAAETQSRVRPVFPTPEQLKPLPQGLEEGAVPTVEDLQQTQPVTDPRSAFPCSGGE
SQALARLKHYFWDTDAVATYKETRNGLIGVDYSTKFAPWLAMGCISPRYIYHQIKQYERERTANQSTYWVIFE
LLWRDYFKFVGVKYGNRLFQVEARGNFLSGLQDKSVPWKKDMKLFNAWKEGQTGVPFVDANMRELAMTGFMSN
RGRQNVASFLTKDLGLDWRMGAEWFEYLLIDHDVCSNYGNWLYSAGIGNDPRENRKFNMIKQGLDYDNNGDYV
RQWVPELQGIRGADVHTPWTLSTAALSHAQVSLGETYPTPIVIAPEWSRHTNKKLNGTGPSPRGKKGPSHTPK
QHRDRGIDFYFSKSKNL
>Siniperca_chua__XP_044047054.1
MLCFCNCHLVGLRNRDMNLSRCPELPNISPKSSMAHTCIHWFRKGLRLHDNPALMAALRDCKELYPVFILDPY
LHNNTCVGINRWRFLIGALKDLDCSLRKLNSRLFVVRGKPEDVFPKLFSQWKVTKLTYEYDTEPYSLSRDKTV
TALSKEHGVKVIYKISHTLYDIDRIIEENNGKAPLTYNRMQAIVKTLGPPKRPIHAPSMEDMEDVETPCSENH
EKEYGIPTLEELCQDTAALREEQFPGGEQEALRRLDEHMKRTGWVCSFEKPQTSPNSLSPSTTVLSPYVTFGC
LSARTFWWRLTDVYQGKKHSDPPVSLHGQLLWREFFYTASVGIPNFNKMEGNPVCTQVDWDTNPEYLAAWREA
RTGFPFIDAIMTQLRQEGWIHHLARHAVACFLTRGDLWISWEEGQKVFEELLLDGDWALNAGNWQWLSASAFF
HQFFRVYSPVAFGKKTDKNGDYIKKYLPLLRKFPATYIYEPWKAPLRIQQAAGCIVGKDYPHPIAQHEVISKK
NIQRMKLAYAKRSTDSADTPSKKQGVKRRAPSVADMLKKKREKVSLHK
>Siniperca_chua__XP_044047888.1
MVVNTIHWFRKGLRLHDNPSLRDSIEGSDTLRCIYILDPWFAGSSNVGINRWRFLLHCLEDLDASLRKLNSRL
FVIRGQPTNVFPRLFKEWQITRLSYEYDSEPFGKERDAAIQKLASEAGVEVMVRISHTLYNLDKIIELNDGQP
PLTYKRFQALLNRMDAVELPAETIMSKVIKKCATPISDDHDDKFGVPSLEELGFETEGLTTAVWPGGETEALM
RLERHLERKAWVANFERPRMNANSLLASPTGLSPYLRFGCLSCRLFYFKLTDLYRKVKKNNTPPLSLYGQLLW
REFFYATASNNPCFDKMEGNPVCVQIPWDRNPEALAKWAEGRTGFPWIDAIMTQLRQEGWIHHLARHAVACFL
TRGDLWISWEEGMKAFEELLIDVDWSVNAGSWIWLSCSSFFQQFFHCYCPVGFGRRTDPNGDYIRRYLPILRG
FPAKYIYDPWNAPEEVQKTAKCIIGVHYPNPMVNHAEASRFNIERMKQIYQQLSCYRGLGLLATVPANPSNGG
NGSNVGGVNTGTSQGPGGSSENRSIHEGMPQTERGQSTQKRRCEEAPLECSSKAWRKNK
>Siniperca_chua__XP_044048297.1

MVVNSVHWFRKGLRLHDNPALQEALNGADTVRCVYILDPWFAGAANVGINRWRFLLEALEDLDSSLKKLNSRL
FVVRGQPTDVFPRLLKEWKVTRLTFEYDPEPYGKERDGAIIKMAQEFGVETIIRNSHTLYNLDRIIEMNNNSP
PLTFKRFQTIVSRLELPRRPLPPITQQQMDNCHTKIADNHDQLYSIPSLEELGFRTEGLPPAVWRGGESEASD
RLNKHLDKKVWVANFEHPRVNTCSLYASPTGLSPYLRFGCLSCRVLYYNLRELYMKLRKRCSPPLSLFGQLLW
REFFYTAATNNPNFDRMEGNPICVQIPWDQNPEALAKWAEGRTGFPWIDAIMTQLRQEGWIHHLARHAVACFL
TRGDLWISWESGMKVFEELLLDADWSVNAGSWMWLSCSAFFQQFFHCYCPVGFGRRTDPSGDYIRRYIPILKD
YPNRYIYEPWNAPESVQKAANCVVGVDYPKPMINHAEGSRLNIERMKQVYQQLSHYRGLSLLASVPTIQEEAE
EPMTDESQTSSGPVFLDSPSRGPADTEAAGCSTAPDSSTVCASSTYAPHPHLEDTANSHPSQTTCTSSGSHAQ
PSAAITSTSVSHPLSTAIPASLAQSLLSSSKPSSSCSTLSPSPNPATTTTQTSSLGPRRKGLAGNVRRSQRQR
GRQSCTPAAREGERRSEDEEGEEAEGEERMEEDIEQNVERMEEETSGETAGHQQ
>Siniperca_chua__XP_044050679.1
MRPSAASGAEHSKALLRKMLRDVLVGREDPEGFFATCVSALGHQETRSQFLSLIQPLFTANSSLHSILISIYK
EYFSETEDDELKLALALSLLEMKDHQLSTPSQESRLQQPGDRPNQRSSVQLPAISQPQGSSHTQLAVGGRKEN
TNSPQTEPWVNHNPQHTTRETEHQKSTHVDKYNKETPGTSQIVCVSRLSGLFSKQDTVEEGDQMMDEGDLNQS
EKPKRSKNRRQRRKGVGRQVVGLPCSPSAPPPVLLWFRRDLRLCDNPALIGSLEVGAPVIPVFIWSPEEEEGT
GTTVAMGGACKYWLHQALSCFCSSLERIGSRLVFLKANGEGNEVGSSLRTLKELVKETGARTVLANAVYEPWL
KERDDVVGSALQKDGVECKMFHSYCLRDPYSVSTEGVGLRGIGSVSHFMSCCRQNPGYALGVPLDPPVSLPTP
AHWPQGVSLDTLGLARMPRRKDGTTIDWAANIRKSWDFSEEGAHARLEAFLHDGVYRYKKESGRADAPNTSCL
SPYLHFGQLSPRWLLWDAKGVRCRPPKFQRKLAWRDLAYWQLTLFPDLPWESLRPPYKALWWSSDRGHLKAWQ
RGRTGYPLVDAAMRQLWLTGWMNNYMRHVVASFLIAYLHLPWQEGYCWFQNTLVDADVAIDAMMWQNGGMCGL
DHWNFVMHPVDASMTCDPYGSYVRKWCPELADLPDELIHKPWKCPASMLRRAGVVFGQTYPERIVTDLEERRG
QSLQDVALVRKEFGQYVDKRSGCDLVPLPPRLVSEALGLSHRDGGAVTKGKQFLLPVITRMEFKHQLEDPDAD
AFSNPYNAVLKGYVSRRRDETIAFLNERDFTASVIYEGTQSKERLESVYRRMEGLPRPPAPRGRARRTPSAKD
RFSIVPGGAVTSLM
>Siniperca_chua__XP_044074458.1
MLHCIWRSSSFSSKPRQLLRLAAHSNQRPSSVFSCLTKSFTSQTIMSGKKRKATSAAAAKDPSAKQQKLAPME
EEKEERAGGWLQDLVKQQRTKNKEMKFNKKRLRFISDTEKIKQGSEGVLYWMSRDHRVQDNWALVHAQQLALK
ENLPMHVCVCLDVPKSELSTLRHYSFMLKGLEEVAKECKTLDIQFHLLHGSAGEVLPGFVSDRCLGAVVTDFS
PLREPLKWLEDVKKRLPKDIPLIQVDAHNIVTCWEASPKLEYAARTIRGKINKLLPEFLTDFPLVEKHPFTAT
RTAKPVDWDKTLASLQVDRAVGMPEWAKPGTKAGIAMLESFIDVRLKQFDTQRNDPNAAALSQLSPWIRFGHL
SAQRVALQVQSRGKNAGQSVSSFIEELVVRRELTDNFCFYNKKYDSVEGAYEWAQKTLKDHAKDERPYLYTRE
QLEKAKTHDKLWNAAQLQMVTEGKMHGFLRMYWAKKILEWTSSPEEALSIALYLNDRYELDGQDPNGFVGCMW
SICGIHDQGWAERPVFGKIRYMNYKGCLRKFDVARFERKYCPKNL
>Soboliphyme_bat_VDP02910.1_unnamed_pr
MVDGRFAVDLSLVNVLNSQPVNSTGKFVLYWVQAAPRVAYNAALEMACSLANVHKVPLFAVFVLSAGYPSANA
RHYTFLLEGLRQFQQKLKEERRLRLDVLQGVASEVIPELAKNCCEMVVDMGYTRIQREWYTKVAAKISCRLTQ
VETNVVVPVKVASDKEEFAARTIRPKLWNVAKKYLIEFPTIMPNIISLDEQIWATTSSVVDLSQELTNIIDHM
KVSFEVAPVINIHGGHIAAVEMLEKFVDSRLKNYEKDRNIPGTAAQSFLSPYLHFGHLSPVEAILRVKRSQAP
AAPKNAFIEELFVRRELAVNFVYYNDSYDSIKCLPAWATETLKIHEKDKRPYVYSSEQLENAQTHDIYWNACQ
LEMVHTGKMHGYMRMYWGKKVLEWSSTVDAAYNWILNMNDKYELDGRDPNGFAGVAWIFGKHDRPHGERAIFG
KVRYMNEEGLRRKFRGSLEKYVSTCYALAGKATGIKQYMR
>Solanum_lyc_cryptochrome_DASH_NP_001318103.1
MDPKNQFSDNSEAQTPQEEQQQTPILPIASISLALSTILPTHFLNPPKISTLFNKPNKVKIPTQVSSLTNLSL
SASSLPPTNSKFKSTISANPLQNTLTLNPLRPSEPSNAAGLRRASIVWFRNDLRVHDNECLNAAHNESMSVLA
VYCFDPRDYGKSSSGFDKTGPYRASFLIDSVADLRKNLQARGSDLVVRIGKPETVLVELAKAVGAEAVYAHRE
VSHDEVKGEDKIDAVMKDEGLEVKYFWGSTLYHVDDLPFKLEQMPTNYGGFREKVQGLEVRKTIEALDQLRGL
PARGDVEPGEIPSLVDLGLNPSATMGQNGKSAANASLVGGENEALQRLRKFAAECQAQPSKENKDGTNDSIYG
ANFSCKISPWLAMGCLSPRSMFDELKKSTSRTISAASAKKDGGSGTGLNWLMYELLWRDFFRFITKKYSAAKQ
NSAAPVTTCVGAAA
>Solanum_lyc_NP_001234245.1_cry_2_
MESNYKTIVWFRRDLRIEDNPALAAAARNGSVLPVFIWCPKEEGQFYPGRVSRWWLKQSLIHLKQSLKSLGAE
LVLMKAQSTLSALTECVDAVGATKVVYNHLYDPVSLVRDHNIKQKLGDLGISVQSYNGDLLNEPWEVYDDDGK
VFTTFDAYWEKSLSIQNEPVSQLPPWRLTQAAGSVKMCSVEELGLENESEKSSNALLGKGWAPGWSNADKALT
EFVESNLLAYSKDRLRVGGNSTSLLSPYLHFGEVSVRKVFNSVRLKQILWTKEGNSVGKDSATIYLRAIGLRE
YSRYICFNFPFTHERSLLNNLRFFPWNADQAHFKAWRQGRTGYPLVDAGMRELWATGWVHNKIRVIVSSFFVK
FLLLPWQWGMKYFWDTLLDADLESDIIGWQYISGSLPDGHELERLDNPEVQGFNYDPEGEYVRHWLPELARMP
AEWIHHPWDAPLNVLKAAGVELGMNYPNPIIDVDVARDRLMQAIIMREKEAAVNTSHANGTVEVVFDNSENV
GDSASIPKDDVVKGKEPCPSSSSYDQRVPSMQNVGTYRKRPKPEEETKKLNDNKLSYKNERIKMSNVDGDLCS
TAESSSMKKQMTVSRNSFSVPRTITMSHDRKSFDDEASSHVKLQKEEEIDT
>Solanum_lyc_NP_001234577.1_cry_1b_

MSSGGCSIVWFRRDLRLEDNPALAAAVRAGSVIAVFIWAPEEEGYYCPGRVSRWWIKKSLAHLDSSLKKLGTS
LITKRSNDSVSSLLQVVKSTGATRVFFNHLYDPISLVRDNCAKETLSAEGVSVCSFNADLLYEPWEVVDDESR
PFSTFSDFWEKCLTMPYDPEAPLLPPKRIISGDASRCPSDNLVFESELEKGSNALLARAWSPGWSNADKALTT
FINGPLIEYSKNRSKADSATTSFLSPCLHFGEVSVRKVFHRIRTKQTLWANEGNKAGEESVNLFLKSIGLREF
SRYMSFYHPYSHERPLLGQLKYFPWLVDEGYFKAWRQGRTGYPLVDAGMRELWATGWLHDRIRVVVSSFSVKV
LQLPWTWGMKYFWDTLLDADLESDALGWQFITGTLPDGCEFLGIDNPQFEGYKFDPNGEYVRRWLPELARLPT
EWIHHPWDAPESVLQAAGIELGSNYPFPIVEIVAAKERLEEALSQMWQLEAAARSAIENGMEEGHGDSTDEFV
PIAFPQAMQIEMEANNVPVRNNNPTITALRRYGDQIVPSMSSSFFRNEDEETSVDIRNSVVDSRAEVPIISM
>Solanum_lycoper_NP_001296304.1
MIKQPFLLTKFTPFSSKSKHTLFTFHCNFSIKMASLTARTTPTVQNVPGLTPEEMERVCEQTFQRYESGGLGK
RKGKGVAIVWFRNDLRVLDNEALLRAWVSSEAILPVYCVDPRLFGTTHYFGMPKTGALRAQFIIECLNDLKRN
LVKRGLDLLIQHGKPEDIVPSLAKAYKAHTVYAHKETCSEEVKVEKMVTRNLQKLVSPSSGGIGNDPGSGNTT
KLELVWGSTMYHIDDLPFDCESLPDVYTQFRKSVEYKSKVRNCTKLPTSFGPPPEVGDWGHVPQVSELGLQQE
KVSKGMNFVGGESAALGRVHDYFWKKDLLKVYKETRNGMLGADYSTKFSPWLASGSLSPRFIYEEVKRYEKER
LSNDSTYWVLFELIWRDYFRFLSIKLANLLFQAGGPQKVNINWSQDQTMFDAWRRGQTGYPLIDANMKELAAT
GYMSNRGRQIVCSFLVRDMGIDWRMGAEWFETCLLDYDPCSNYGNWTYGAGVGNDPREDRYFSIPKQAQNYDP
EGEFVAYWLPELRALPREKRHSPGMMYLNPIVALKHGYTKKTGDSKTAFSSRRGRPEDNRRKRHGY
>Solanum_lyc_XP_004252323.1_(6-4)DNA_p
MASGANSLMWFRKGLRLHDNPALEYAAKGSKFLYPVFVIDPHYMDPDPTAFSLGSSKAGLNRIQFLLESLADL
DLSLKKVGSRLLVLKGDPGELLIRCLKEWSIGKLCFEYDTEPYYQALDEKVKGYVSGTGVEIFSPVSHTLYNP
ADIIHKNGGSPPLSYQSFLKLAGQPSWAATPLSTTISSLPRIGNTGSFAVSEVPTVRELGYEDLPEDEKTPFK
GGESEALKRLRESIANKEWVANFEKPKGNPSAFLKPATTVLSPYLKFGCLSSRYFYQCIQDILKCSKKHTSPP
VSLLGQLLWRDFFYTAAFGTPNFDQMKGNRICKQIPWKNDDKLLAAWRDSKTGFPWIDAIMVQLRKWGWIHHL
ARHSVACFLTRGDLFVHWERGRHVFERLLIDSDWAINNGNWLWLSCSSFFYQYNRIYSPISFGKKYDPAGNYI
RHFLPVLKDMPKEYIYEPWTAPISVQRKAKCIIGVDYPKPVVSHDSASKECKMRLGEAYALNKKLNGLVSEED
LNELRRKADNESTTLDSVSRKKKQKLID
>Solanum_lyc_XP_010325452.1_photolyase
MASAIPVVQSGRIRVVKQGSGPLVGPVVYWMFRDQRIRDNWALIHAVDQANKANVPVAIAFNLFDQFLGAKAR
QLGFMLRGLEKLQGNLESTLRIPFFLFQGEAIDTIPNFLKECGASLLVTDFSPLRDVRSWKEKICERVDESVT
VHEVDAHNIVPLWVASNKLEYSARTIRGKINKLLPEYLIELPAIEPLKIKWSSSNAPIDWPKLVSDVVRKGAE
VPELEWCEPGEDAAFEVLMGSKKGFLTTRLKTYSTDRNNPLKPQALSGLSPYLHFGQISAQRCALEANKVRKN
YTQAVDTFLEEMIVRRELSDNFCYYQPQYDSLLGAWEWARKTLMEHASDKREHIYTREQLEKAQTADVCFGQL
WNASQLEMVHYGKMHGFMRMYWAKKILEWTNGPEEALAITIYLNDKYHIDGRDPSGYVGCMWSICGVHDQGWR
ERPVFGKIRYMNYAGCKKKFNVDGYISYVKRLVGESKKRKAEVILDKKAKELRN
>Solea_sene__KAG7458369.1
MRKRTSPPLSLFGQLLWREFFYTAATNNPNFDRMEGNPICVQIPWDQNPEALAKWAEGRTGFPWIDAIMTQLR
QEGWIHHLARHAVACFLTRGDLWITWESGMKVFEELLLDADWSVNAGSWMWLSCSAFFQQFFHCYCPVGFGRR
TDPSGDYIRRYVPVLKDYPNRYIYEPWNAPESLQKAANCVVGVDYPKPMINHAEGSRLNIERMKQVYQQLSHY
RGLSLLASVPTIQEEAEPPMTDESQTSSGPDSPPRVPVHREPAICSPAPDSSIVCGSSSSSGDACSLQTLHSS
SQPSPAATSKSAAAAPPPPASSPVHSPLNLPSPSSSCPTLSPAPSLAATSAQTFPKRKGPAHKVCRSRRLRGS
RSREEGGGGEEEEEEEETGAEEKMEEDVEEEEKMEEED
>Solea_sene__KAG7481848.1
MKRTGWVCKFEKPQTSPNSLSPSTTVLSPYVTIGCLSARTFWWRLTEVYRGNKHSDPPVSLHGQLLWREFFYT
ASVGIPNFNKMEGNTVCTQVDWDTNSEYLAAWREARTGFPFIDAIMTQLRQEGWIHHLARHAVACFLTRGDLW
ISWEEGQKVFEKLLLDGDWALNAGNWQWLSASTFFHQFFRVYSPVAFGKKTDKHGDYIKKYLPLLKKFPAEYI
YEPWKAPRSVQQAAGCIVGKDYPQPIVQHEVISKKNIQRMKLAYAKRSADKTASPSKKQGVKRKTSVVDMLKK
NKR
>Solea_sene__XP_043877840.1
MVINTIHWFRKGLRLHDNPSLKDSLLGADTVRCIYILDPWFAGSSNVGINRWRFLLQSLEDLDSSLRKLNSRL
FVIRGQPTDVFPRLFKEWKINRLSYEYDSEPFGKERDAAIKKLASEAAVEVIVRTSHTLYDLDKIIELNGGQS
PLTYKRFQTLISRMDAVEVPAESITGDVMGKCTTPLSDEHDDKFGVPSLEELGFDTEGLASAVWPGGESEALT
RLERHLERKAWVANFERPRMNANSLLASPTGLSPYLRFGCLSCRLFYFKLTDLYRKVKKNSSPPLSLYGQLLW
REFFYTAATNNPCFDKMESNPICVQIPWDRNAEALAKWAEGRSGFPWIDAIMTQLRQEGWIHHLARHAVACFL
TRGDLWISWEEGMKVFEELLLDADWSVNAGSWMWLSCSSFFQQFFHCYCPVGFGRRTDPNGDYIRRYLPILRG
FPAKYIYDPWNAPESVQKAAKCIIGVHYPKPMVNHAEASRLNIERMKQIYQQLSCYRGLGLLASVPSNPSGHG
HGNEVSEASSDAMAFHAEATPTAAAPSSYQMPAHSQGDWQSGVMLYLQGDSQTNAGTHQQGYASTSTSSSMMC
YTPGAQQISGPLIQKGLDHHSISHTSGKRHIKESGNGKSSKIQRQSTH
>Solea_sene__XP_043878427.1
MICIWRSSLSSFSPRRLLKLIVDGNRRPRQSLVPLIEAYTCEPTMSGKKRKATSAAKVASAKQQKLVPTKEEK
QKAGGWLHDLVTQQRSENQEMKFNKKRCRFISDTEKIKQGSEGVLYWMLRDQRVQDNWALTYAQRLAVKENLP

LHICYCLDVPKSELSTLRHYNFMLKGLEEIAKECKTLDVQFHLLHGSAEEVVPGFVSDRSLGAVVTDFSPLRE
PLQWLEDMKKTLPKDIPLIQVDSHNIVPCWVASPKLEYAARTIRGKITNLLPEFLTEFPLVEKHPHTATRTAK
PVDWDKLLASLQVDRTVGEPEWAKPGTKAGMAMLESFIDVRLKLFDNLRNDPNANALSQLSPWIRFGHLSAQR
VALQVKHSGKSTGHSITSFIEELVVRRELTDNFCFYNKKYDSVEGAYEWAQKTLKDHAKDKRPYLYTREQLEK
AKTHDKLWNAAQYQMVSEGKMHGFLRMYWAKKILEWTSSPEEALSTALYLNDRYELDGQDPNGFVGCMWSICG
IHDQGWAERDVFGKIRYMNYKGCTRKFDVARFEQKYCPKNL
>Solea_sene__XP_043878908.1
MSASSRTIICLLRNDLRLHDNELFHWAQRNAENIVPLYCLDPRHYVGTYNFNFPKTGPFRLRFMLDSIRDLRK
TLVNKGSNLVVRRGKPEDVVAELIKQLGSVSTVAFHEEVTSEELSVEKNVKDVCARMKVKVHTCWGSTLYHRD
DLPFHHVSRLPDVYTQFRKAVETQSSVRPVFPTPEQLKPLPPGLDEGAIPTAVDLQQTEPVTDPRSAFPCTGG
ESQALARVQHYFWDTNAVASYKETRNGLIGADYSTKFAPWLAIGCISPRYIYHQIKQYERERTANQSTYWVIF
ELLWRDYFKFVAAKYGNRLFQVKGLQDKSVPWKKDMKLFNAWKEGRTGVPFVDANMRELAQTGFMSNRGRQNV
ASFLTKDLGLDWRMGAEWFEYLLVDHDVCSNYGNWLYSAGVGNDPRENRKFNMIKQGLDYDNNGDYVRQWVPE
LQGIRGGDVHTPWTLSSATLSHAHVSLGETYPTPVVMAPEWSRHANKKASASGPSPRGRKGPSHTPKQHGNRG
IDFYFSRSKNL
>Solea_sene__XP_043891818.1
MPPSAAGAEERRAYVRKLLREVLVGREDPEGFFAMCVSVLGHQDTRSHFLTLIQPLTTANSTLHSGLTSIYQD
YFSKTEDEELELALALSVLEMKDDQLPTPVQEPGDGQTDSRSVQLTSVGQPQGSRHTQLTDRSGGRKKGAWGM
ACPLKNPQETELQKNTQTLAACQTVCVASPCASLSKQETDLEGDERTEEGDSIQSGKTKRSRNRRKRRQAVGQ
QVVGLPCSPSAAAPVLLWCRRDLRLCDNPALIGSLEVGAPVIPVFIWSPEEEEGPGITVAMGGACKYWLHQAL
SHFRSSLEGIGSHLVFLKADGAGSGLGSSLQSLKELVKETGARTVMANALYEPWLKERDDEVVSALQKGGVQC
RMFHSYCLRDPYSVSTEGVGLRGIGSVSHFMSCCRQNPTSALGAPLDPPGSLPTPATWPQGVSLDTLELARMP
RRKDGSTIDWAANIRNSWDFSEEGAHARLEAFLQDGVYRYEKESGRADEPNTSCVSPYLHFGQLSPRWLLWDA
KGARCRPPKFQRKLAWRDLAYWQLTLFPDLPWESLRPPYKALRWSTDRRHLKAWQRGRTGYPLVDAAMRQLWL
TGWMNNYMRHVVASFLIAYLHLPWQEGYRWFQDTLVDADVAIDAMMWQNGGMCGLDHWNFVMHPVDAAMTCDP
CGNYVRKWCPELADLPDELIHKPWKCPVSMLRRAGVVIGQTYPERVISDLEEQRSRSLKDVAVVRKRFGQYVD
KRTGCDLVPLPPRLVTEALGPSHGNGGIVAEGKQFLLPVITRMEFKHQLADPDSDAASNPYNAVLKGYVSRKR
DETIAFLNERDFTASVLHEGAQRRERLESDQRRMEGLPRPPAPPSRARRTPKAKDKFSVVPGGAVTSLT
>Sorex_aran__XP_004619154.1
MKMAKEAGVEVVTENSHTLYDLDRIIELNGQKPPLTYKRFQAIIGRMELPRKPVGSVSRQQMESCRAEIQENH
DETYGVPSLEGLGFPTEGLGPAVWQGGETEALARLDKHLERKAWVANYERPRMNANSLLASPTGLSPYLRFGC
LSCRLFYYSLWDLYKKVKRSSTPPLSLFGQLLWREFFYTAATNNPRFDRMEGNPICIQIPWDRNPEALAKWAE
GKTGFPWIDAIMTQLRQEGWIHHLARHAVACFLTRGDLWVSWESGVRVFDELLLDADFSVNAGSWMWLSCSAF
FQQFFHCYCPVGFGRRTDPSGDYIRRYLPKLKGFPSRYIYEPWNAPEAVQKAAKCVIGVDYPRPMVNHAETSR
LNIERMKQIYQQLSRYRGLCLLASVPSCVEELSNPMAEPSGSQAGSSAGPRSVPGGPSSPKRKLEAAEEPPGE
ELSKRARVAGSSTPEPTSSGAAVCSAEAPGASVDAHLSSCHHHAQPATAGSTDRGSERVCMGAGWKKLLLQEP
LLHPSPLQLPSAQLKFLMNLQTGVSVSAGPVEPTHWVAATISGLFPILEGRLCPPVPESRVHFVILSISRSTD
CGPCLVLSPDMHATLTDDRQPALVSVRPQELGAGDSAQAAEKTQGDSVDLSQTP
>Sorex_aran__XP_012790734.1
MEPLAIPVETITSEVIEKCTTPLSDDHDEKYGVPSLEELGFDTDGLPSAVWPGGETEALTRLERHLERKAWVA
NFERPRMNANSLLASPTGLSPYLRFGCLSCRLFYFKLTDLYKKVKKNSSPPLSLYGQLLWREFFYTAATNNPR
FDKMEGNPICVQIPWDKNPEALAKWAEGRTGFPWIDAIMTQLRQEGWIHHLARHAVACFLTRGDLWISWEEGM
KVFEELLLDADWSINAGSWMWLSCSSFFQQFFHCYCPVGFGRRTDPNGDYIRRYLPVLRGFPAKYIYDPWNAP
EGIQKVAKCLIGVNYPKPMVNHAEASRLNIERMKQIYQQLSRYRGLGLLATVPSNPNGNGGFMGYSPGENASG
CSSSGSYFQGSGILRYAYGDSQQTHLLKQGRSSSCTGLSSGKRPSQEEDRQSPKVQRQTTN
>Spadella_cep_QNM38094.1_cry,_parti
SEVVDRQREDCVAIHWFRHGLRLHDCPAMVEAVERAGTLYPVFIFDGRVAGTWNSGYNRWRFLLECLEDLDQR
LRKSGGRLWIFHGDPREIFARLVPLWGVTHVSFELDLEPIWQERDCAVTSLLNDMGVEVIGRISYTLWDPREI
IKANGGSPPLTFGLFMLTTSTMGEPAKPVDEPDWSSVILPIDEAKLGEEWRNRNHVPNLDELGVAEDVPGQKD
NYVWEGGETRALVKKASRLENERKAVAKGQFQTNILRPDLVGASRSLSPYFRFGCLSVRAFYHEIMDIFRKEL
TDDTMMNRPTAVTAQLIWREYFYTMSINNPYFAEIDDNPVCLPIPWNENEDLLKAWKDGKTGFPWIDALQRQL
VLEGWTHHVGRHAAACFLTRGHLWQSWVPGALHFIKHQIDGDWAVGIGNWMWVSSSAFEKALDCAKCMCPINF
GRRMDQDGAFIKKYVPEVRNLPLRYLFSPWKAPENVQKEANCVVGKDYPFPIVDYKFVSKRNMQWMEDFKTQV
VERMADHVRPTTYSEEELRLCGLATHSSTCKACQDRSRPIEDDEEENYQDMD
>Sparganum_pro_VZI11874.1_unnamed_pr
MRVLHWFRKGLRLHDNPALLAALQPLAARPDGGAAPVELLFVFILDPCLPDHMDIGFRRCSFLLECLQALDKS
LASLNPKFRLLTLRGRPEEVLPKACLAWGVEHVTYEVDTDAYGRSRDVAVRDLLAQSNISVSEHSGHTLWDLD
WLRSKYTAPPPSPKRPTENGDGPTNSTSSQQLGVPLTYQSFCALTRRVAPQGPARPLPSFAEYVSQGIVSSPD
LEGLRGSFPDLGCSLGAPAAVADMDIGPIHYSRVGEGLSDSAAEKQGLLFPGGEAEALERLRRMPATAPTGLP
ISTTGLSPYLRYGCLSVRTAWWALREARRTAKTPASASQPPVSLHGQLLWREFFYLVSSVTPNYDRIVANPIC

RQIPWIRNEELLTAWAEARTGFPFIDAAMTQLHRHGWMHHLARHAVACFLTRGQLWQSWTDGAAVFQRLLLDA
DWALNSANWMWLSASAFFHQYFRVYCPVGFGRKTDPEGNFIRHFLPCLKNMPKQYIYEPWTAPLAVQKKAGCI
IGVDYPARIVDHQEMRAANLVKMKAAYDACRTAAHNPSKRGTDTSPTKPGQLGTGELSANKRKKSWIQSKFGL
SNLTAIQGKQPSAHTFAWDSKHLCNS
>Sparganum_pro_VZI21603.1_unnamed_pr
MTTATLKASQTVSRVQLGITSLFVKLVRKSQSRKLSIMASSMRTEEKQSFDDWVAGINLRRSEDAAKSVGEFG
FATCRVRLLSGNNKFVTFPESASGIKQLDASGAASLNSGADSASVLYWMNRDQRVQDNWALLFAQRVALKFSV
PLHVCFNLPSSSAISTRRHFNFQLAGLKEIATQCKDLNIGFTVLPLMSESSRGTKRTAEGQLVDTAKTGSTFS
GLLNLIRNLQVAALITDFSPLREDTAAVQTVSGLLPSNTSFFQVDAHNIVPAWHASDKLEYAARTIRRKLHEK
SQTLLTEFPPVRAHPIRGNVKEPDWESIEASVVGHLDESVKPIKWAQGGSRHGLRELFTFLHQRLSLYATARN
DPTKNALSQLSPWFHFGHLSVQRAILEAQRLRRKHKESVDAFIEEAFVRRELSDNFCLYNPHYDSIEGAWPWA
QETLRKHASDERKPAYDEEAMEKARTDDNLWNAAQRQLLRDGKIHGFLRMYWAKKILEWHAAGPEAALALGLR
LNDRYSIDGSDPNGFVGVMWSICGIHDQGWSERTIFGKIRYMNYAGCKRKFNVSQFETQYPS
>Sparus_aura__XP_030254933.1
MFGNSLAGNVTDPPAPQTGGRCWLTHRTVDVSRTTTAPSSLKTATMSTARTVICLLRNDLRLHDNELFHWAER
NAEYIVPLYCFDPRHYVGTRNYNLPKTGPFRLRFLMDSVRDLRNTLLNKGSNLVVRQGKPEDVVAALIKQLGS
VSTVAFHEEITSEELNVEKSVKDVCAQMNVKVHTCWGSTLYHRDDLPFHHMSRLPDVYTQFRKAVETQSEVRP
VFPTPEQLKPLPQGLEEGAIPTAEDLQQTEPVADPRSAFPCSGGESQALARIKHYFWDTDAVATYKETRNGLI
GVDYSTKFAPWLAMGCISPRYIYHQIQQYEKERTANQSTYWVIFELLWRDYFKFVAVKYGDRLFQIEGLQEKS
IPWKKDMKLFDAWKEGRTGVPFVDANMREVAMTGFMSNRGRQNVASFLTKDLGLDWRMGAEWFEHLLIDHDVC
SNYGNWLYSAGIGNDPRENRKFNMIKQGLDYDNNGDYVRQWVPELQGIKGADVHSPWTLSSAALSHAQVTLGE
TYPTPIVMAPEWSRHVNKKPSGAGPSPRGKRGPSHTPKQHRDRGIDFYFSKGKQQ
>Sparus_aura__XP_030258793.1
MFFCFWRSSFIPFAPRPLLRLVAQGSQRSAFVFTSRTIMSDKKRKSKSAAAGPRAKQQKVAAVKEEKRERAAG
WLQDFLQQQRTEEKEMKLNKKRIRVISATEKIKQGSEGVLYWMSRDHRVQDNWALIHAQRLAVKEKLPLHVCA
CLNVPKSELSTLRHYSFMLKGLEEVAEECKSLDIQFHLLHGTAGEVLPGFVSDRVLGAVVTDFSPLREPLQWL
EDIKKKLPKDIPLIQVDAHNVVPCWEASPKLEYSARTIRGKITKILSDFLTDFPPVEKHPYTATRTAKPVDWE
QTLASLEVDKTVGETEWAKPGTKAGTAMLESFIDLRLKIFDTKRNDPNAPALSQLSPWIRFGHLSAQRVAQQV
QHSGKKVGVTVSSYIEELVVRRELTDNFCFYNKKYDSVEGAHEWAQKTLRDHAKDERPYIYTREQLEKAKTHD
KLWNAAQYQMVTEGKMHGFLRMYWAKKILEWTSSPEEALSIALYLNDRYELDGQDPNGFVGCMWSICGVHDQG
WAERPIFGKIRYMNYKGCLRKFDVAQFERTYSPKNL
>Sparus_aura__XP_030271124.1
MAHTCVHWFRKGLRLHDNPALMAALRDCKELYPVFVLDPNLHNNTCVGINRWRFLIGALKDLDCSLRKLNSRL
FVVRGKPEDVFPTLFNEWKVTKLTYEYDTEPYSVRRDKTVMALAKEHGVEVIYKVSHTLYDIDRIIEENNGKP
PLTYNRMQAIVKTLGPPKKPIPAPTMENMKDVQTPCSESHEKKYGIPTLEELDQDAAALGEDKFPGGEQEALR
RLDEHMKKTGWVCGFEKPQTSPNSLSPSTTVLSPYVTIGCLSARTFWWRLTDVYQGKKHSDPPVSLHGQLLWR
EFFYTASVGIPNFNKMEGNSVCTQVDWDSNPEYLAAWREARTGFPFIDAIMTQLRQEGWIHHLARHAVACFLT
RGDLWINWEEGQRVFEELLLDGDWALNAGNWQWLSASTFFHQFFRVYSPVAFGKKTDKNGDYIKKYLPLLKKF
PAQYIYEPWKAPHSVQQAAGCIVGKDYPHPIVTHEDISKTNIQRMKLAYSKRSADPGASPSKKQGVKRKAPTV
VDMLKKKSKKK
>Sparus_aura__XP_030281971.1
MHHKTEDDELELALALSLLEMKDHRLSTPSQDSPPQQPGDRPNQRRFVQLASVTQPQGSSHAQLADVVGRGKN
TNSSRGAPWVKSSPPQTTRETGLQESAHVYKCNKETPGTSKTECVSRLSGSISKQATGEEDNQMMDVGDLTES
QKPKRSKNRRQRRKGAGQQVVGLPCSPSAQPPVLLWFRRDLRLCDNPALIGSLEAGAPVIPVFIWSPEEEEGP
GITVAMGGACKYWLHQALSCFSSSLESIGSHLVFLKANRERSGVGSSVHTLKELVKETGARTVVANALYEPWL
KERDDAVVSALQKDGVECKMFDSYCLKDPYSVSTEGVGLRGIGSVSHYMSCCSQNPGSALGAPLDPPGSLPAP
SNWPQGVSLDTLGLARMPRRKDGTTIDWAANIRKAWDFSEEGAHARLEAFLQDGVYRYEKESGRADAPNTSSL
SPYLHFGQLSPRWLLWDAKGARCRPPKFQRKLAWRDLAYWQLTLFPDLPWESLRPPYKALRWSSDRGHLKAWQ
RGRTGYPLVDAAMRQLWLTGWMNNYMRHVVASFLIAYLHLPWQEGYRWFQDTLVDADVAIDAMMWQNGGMCGL
DHWNFVMHPVDAAMTCDPCGSYVRKWCPELADLPDELIHKPWKCPASMLRRSGVVFGQTYPERVVTDLEERRT
QSLHDVAVVRKKFGQYVDKRSGCDLVPLPQRLVSEALGLSHSDGGVVTTGKQFLLPVITRMEFKHQLEDPDAD
AASNPYNAVLKGYVSRKRDETIAFLNERDFTTSVMYEGTQRRERLESDYRRMEGLPRPAAPRGRARRTPIAKD
RFSVVPGGAVTSLR
>Sparus_aura__XP_030282112.1
MVVNTVHWFRKGLRLHDNPSLRESIRGADSLRCIYILDPWFAGSSNVGINRWRFLLQCLEDLDASLRKLNSRL
FVIRGQPTDVFPRLFKEWQINRLSYEYDSEPFGKERDAAIQKLACEAGVEVTVLNSHTLYDLSRIIELNDGHP
PLTYKRFQGLINRMDAVELPAETVTLEVLRKCDTPVSDDHDDKFGVPSLEELGFDTDGLATAVWPGGETEALM
RLERHLERKAWVANFERPRMNANSLLASPTGLSPYLRFGCLSCRLFYFKLTDLYKKVKKDNNPPLSLYGQLLW
REFFYATATNNPCFDKMEGNPMCVQIPWDRNPEALAKWAEGRTGFPWIDAIMTQLRQEGWIHHLARHAVACFL
TRGDLWLSWEEGMKTFEELLIDADWSVNAGSWMWLSCSSFFQQFFHCYCPVGFGRRTDPNGDFIRRYLPVLKG

FPAQYIYEPWNAPEELQKSVKCIIGVHYPKPMVNHAEASRVNIERMKQIYQQLSCYRGLGLLATVPVNPHDGT
NRSNVGRVNTCGSSENLSMLGGTSRTERWHGAQKRRCEDSPLESSCKSWRQSK
>Sparus_aura__XP_030282812.1
MVVNSVHWFRKGLRLHDNPALQEALNGADTVRCVYILDPWFAGAANVGINRWRFLLEALEDLDNSLKKLNSRL
FVVRGQPTDVFPRLLKEWKVTRLTFEYDPEPYGKERDGAIMKMAQEFGVETIVRNSHTLYNLDRIIEMNNNSP
PLTFKRFQTIVSRLELPRRPLPPITQQMDKCRTKMADNHDQLYSIPSLEELGFRTEGLPPAIWQGGESEALD
RLNKHLDKKVWVANLEHPRVNTCSLYASPTGLSPYLRFGCLSCRVLYYNLRELYMKLRKRCSPPLSLFGQLLW
REFFYTAATNNPNFDRMDGNPICVQIPWDQNPEALAKWAEGRTGFPWIDAIMTQLRQEGWIHHLARHAVACFL
TRGDLWVSWESGMRVFEELLLDADWSVNAGSWMWLSCSAFFQQFFHCYCPVGFGRRTDPSGDYIRRYIPILKD
YPNRYIYEPWNAPESVQKAANCVVGVNYPKPMINHAEGSRLNIERMKQVYQQLSHYRGLSLLASVPTIQEEAE
PPMTDESQTSSGPDSPPRGPADSEAAGCSTAPDSSTVCASSTYALHPDLKDKGDNYPSKKPYTSSGSLTQSSA
AITSTFASHPPAPLSPAQSPLSRSKPSSPSSSCPTFAESPSPTTAPTQTSSLGQRRKGLAHKVRRSQRQRGRQ
SWTLAPREGGRKSEEEEREEAGGEERMEEDVEQDEERMEEEPSEETAGQQQ
>Sphaeramia_orbi__XP_029978740.1
MSTSRTIIYLLRNDLRLHDNEVFHWAQRNAECIVPLYCFDPRHYGGTYHYNLPKTGPFRLRFLLESIRDLRKT
LLNKGSNLVVRRGKPEDVVADLIKQLGSVSTVAFHEEVASEELNVEKKLKDVCAQMKVKVHTCWGSTLYHRDD
LPFHHMSRLPDVYTQFRKAVETQSRVRPVFPTPEQLKPVPPGLDEGAIPTAEDLEQTEPPTDPRSAFPCSGGE
SDALARLKHYFWDTNAVATYKETRNGLIGADYSTKFAPWLAIGCISPRYIYHQIKQYEMERTANQSTYWVIFE
LLWRDYFKFVGVKYGNRLFHIKGLQDKSVPWRKDMKQFNAWKEGRTGVPFVDANMRELAMTGFMSNRGRQNVA
SFLTKDLGLDWRMGAEWFEYMLIDYDVCSNYGNWLYSAGIGNDPRENRKFNMIKQGLDYDNNGEFVRQWVPEL
RGLKGSDVHTPWTLSTAALSHAHVTLGDTYPTPIIIAPEWSRHINSKAGGSGPSSRGKKGPSHTSKQHRDRGI
DFYFSRSKNM
>Sphaeramia_orbi__XP_029986858.1
MAHTCIHWFRKGLRLHDNPALMAALRDCKEIYPVFILDPYLHNDTCVGINRWRFLIGALKDLDCSLKKLNSRL
FVLRGKPEDVFPKLFDKWKVTKLTYEYDTEPYSLSRDKTVTKLAKEHGVEVIYKVSHTLYDIDRIIEENNGKA
PLTYNRMQAIVNSLGPPKRPVPAPSAEDMKDVKTPYSEDHEKNYGIPTLEELGLDTSSLTEELFPGGEQEALK
RIDEHMTRTAWVCSFEKPQTSPNSLMPSTTVLSPYVTFGCLSARTFWWRLTEVYQGKKHSSPPVSLHGQLLWR
EFFYTASVGIPNFNKMEGNPVCTQVDWDTNSEYLTAWREARTGFPFIDAIMTQLRQEGWIHHLARHAVACFLT
RGDLWISWEEGQRVFEELLLDGDWALNAGNWQWLSASAFFHQFFRVYSPIAFGKKTDKNGAYIKKYLPLLKKF
PPEYIYEPWKAPRSIQQAAGCIVGKDYPLPIVQHEIISKKNIQRMKLAYAKRSTDTADSPSKKKGEKRKAPSV
VDMLKKKGKRN
>Sphaeramia_orbi__XP_029991901.1
MVVNTIHWFRKGLRLHDNSSLRDSIRGADTLRCIYILDPWFAGSSNVGINRWRFLLQCLEDLDASLRKLNSRL
FVIRGQPTDVFPRLFKEWQITRLSYEYDSEPFGKERDAAIQKLASEAGVELMVKISHTLYNLDKIIELNGGQP
PLTYKRFQALINRMDPVELPAETITCEVIKRCATPISDDHDDKYGVPSLEELGFETEGLTTAVWPGGETEALM
RLERHLERKAWVANFERPRMNANSLLASPTGLSPYLRFGCLSCRLFYFKLTDLYRKVKKNRTPPLSLYGQLLW
REFFYTTATNNPCFDKMDGNPVCVQIPWDRNPEALAKWAEGRTGFPWIDAIMTQLRQEGWIHHLARHAVACFL
TRGDLWISWEEGMKVFEELLLDADWSVNAGSWMWLSCSSFFQQFFHCYCPVGFGRRTDPNGDYIRRYLPVLRG
FPAKYIYDPCNAPEEVQKAAKCIIGVHYPKPMVNHAEASRINIERMKQIYQQLSCYRGLGLLATVPANPNNGS
NDSAGSSQGPGASAEDTSTQEAPSRIDRGQSTQKRRREEALPESSSSKSWKQSK
>Sphaeramia_orbi__XP_029991988.1
MVVNSVHWFRKGLRLHDNPALLEALNGADTVRCVYILDPWFAGAANVGINRWRFLLEALEDLDSSLKKLNSRL
FVIRGQPTDVFPRLFKEWKVTRLTFEYDPEPYGKERDGAIIKMAQEFGVETVIRNSHTLYNLDRIIEMNNNSP
PLTFKRFQTIVSRLELPRRPLAPITRQQMDKCCSKISDNHDQLYNIPSLEELGFQTSGLPPAVWRGGESEALD
RLGKHLDRKVWVANIEHPRVSTCSLYASPTGLSPYLRFGCLSCRVLYYNLRELYMKLRKRCSPPLSLFGQLLW
REFFYTAATNNPNFDRMEGNPICVQIPWDQNPEALAKWAEGRTGFPWIDAIMTQLRQEGWIHHLARHAVACFL
TRGDLWISWESGMKVFEELLLDADWSVNAGSWMWLSCSAFFQQFFHCYCPVGFGRRTDPSGDYIRRYIPILKY
YPNRYIYEPWNAPESVQQAANCVVGVHYPKPMINHAEGSRLNIERMKQVYQQLSHYRGLSLLASVPTIQEEAE
PPMTDESQTSSGPDSPPRVPSGDEAAPDSSTACPSPTCSAAVQHPSPEETLSNQQTHAAISSTSSGPPPPLAA
APPSPSSPLSRSKPSSPSSSCPSLSPAPSPAAAPPPRASTLGTRRKGMARKMRRSQRQRGRQICAMTTREGGG
REEEEEEERDDEKMEEDAEPEEEERMEEGTSGGASGHQQ
>Sphaeramia_orbi__XP_029993416.1
MSPLAAASGDDSKALVRKMLREVLVGREDPEGFFALCVSVLGYQDTRSQFLSLIQPLSTANRSLHSTLTSIYK
EYFSKAEDDDLQLALALSLLDTEDQMSVCSQGSQHQQINGSSSQSSLLQATSVSHTQGGSYSQIAAIRSQRKN
ASSLQSGHHSKMSPQETAQNCDLDKDTQELKMSPMVCVSRQSSSFSKQDTCQGGVQMMEKGDSNELEKPKRSR
NRRQRRKGGDKQVVGVPCSPSGLPPVLLWFRRDLRLCDNPAIIGSLEVGAPVIPIFIWSPEEEEGPGMTVAIG
GACKYWLHQALSCLSSSLERIGSHLVFLKANGEGNDVGSSLHTLRELVKVTGARTVLANALYEPWLKDRDDKV
VSALQKDGVECKMYHSYCLRDPYSVSTKGVGLRGIGSVSHFMSCCKQNPGSAIGVPLDPPESLPTPAHWPQGV
SLDMLGLARMPRRKDGTVIDWAANIRKSWDFSEEGAQVRLEAFLQDGVYRYEKESGRADAPNTSCLSPYLHFG
QLSPRWLLWDAKAARCRPPKFQRKLAWRDLAYWQLTLFPDLPWESLRPPYKALRWSNDRGHLKAWQRGRTGYP

LVDAAMRQLWLTGWMNNYMRHVVASFLIAYLHLPWQEGYRWFQDTLVDADVAIDAMMWQNGGMCGLDHWNFVM
HPVDAAMTCDPNGSFVRKWCPELANLPDDLIHKPWKCPASMLRRAGVLFGQTYPERIVTDLEGQRSQSLQDVA
LVRKEYGQYVDKRTGCDLVPLPPRLVSEALGLSHQEEGAVTVGKQFLLPVITRMEFKHQLEDPDADAASNPYN
AVLKGYVSRKRDETIAFLNERDFTASVMYEGAQRKERMESDYRRMQGLPRPPAPRGRARRTPTAKDKFSIVPG
GVVTSLR
>Sphaeramia_orbi__XP_030015659.1
MPPCSCNWLRKGKQRAVTCVFLWTAKNTVHFFGRLRFASSDKIIIQCLLSCVWRNPSFSSAPRKFLKLVGHST
QSRRSPLSGLTISFTTETIMSDKKRKATNDTAPEQHSKQPKLATKKDEKVGGWIQDLVVQQRIEKKGMKFNKK
RLRFLSDTEKIKQGSEGVLYWMSRDQRVQDNWALIHAQRLAEEEKLPLHICFCLVVPKSELSTLRHFSFMLKG
LEEVSKECKKLDIQFHLLHGPAGDVLPGFVSDRGIGAVVTDFSPLREPLKWLDDVKKKLPKDIPFIQVDAHNI
VPCWVASPKLEYSARTIRGKITNLLPEFLTGFPPVEKHSYTATRTAKSVDWAKTLDSLQVDRTVGEPEWAVPG
TKAGMAMLESFIDVRLKLFGTQRNDPNVAALSQLSPWIRFGHLSAQRVALQVQRSGKNASQSIPSFIEELVIR
RELTDNFCFYNKKYDSVEGAYEWAQKTLKDHAKDKREYLYTREQLEKAKTHDKLWNGAQYQMVTEGKMHGFLR
MYWAKKILEWTTSPEEALSIALYLNDRYELDGQDPNGFVGCMWSICGIHDQGWAERPVFGKIRYMNYKGCLRK
FDVARFEKKYCPKNL
>Sphaeroforma_arcXP_014160934.1
MRFNFIISRFPARFNTYRSQSVKQSKCPWQFCSAHKQKVSNLDFYARALGINSLPQFQSNVCSSQLFYSTMGK
RARSGRDSVEDVREVKKLKSVKRTVEEKVNSARVRRLNGKQNIGKIVEVEEGHKTVIYWMERDVRVNDNWALL
YAEELAQKNKLTLSVVYVYPERYREEATVRQELFRLRGLQEVSKRLQGLNIPFHFLVGDVAKNIVDQIDKLDA
AVVVVDFHALRQKRKWRDSVAEKVCSIPVMEVDAHNVVPVWGTSEKCEYGARTIRTKIHKRLAEFGTEFPEVE
VQEGDVEDAEEVDWDEIMNNVDADHDVPEVKWCTPGEAGAMEALETFVNERLQLFARDRNDPNVHASSDLSCF
FNFGQLSAQRAFLVVQTFSGSVQKGRESFIEESIVRRELSDNFCFYKPDTYDTLEGAPGWAQESLEKHTEDKR
EYTYTSDEFAKGNTHDDLWNAAQLQMVEEGKMHGFLRMYWAKKILEWTQTPQTALKTALYLNDKYEIDGYDPN
GYVGCIWSIGGVHDQGWMERSVFGKVRYMNYNGCLRKFNVYEFVQQYPQAVKNCKAQGGTPTKEKHSKKKK
>Sphaeroforma_arc_XP_014156447.1_SARC_05172
MPGAIFWFRKGLRLHDNKALIAAVESRGKKVYPVYVLDPKFVNPKQMGLNRLRFLLECLLDLDYSLRTKHNSR
LIVLRGDPTVVIPEQIEAWDVDRLVYEDEPEPHNVERDEAIKAVLKESNSACLVYCTPGHTIWDPKYLLKINN
GKPPLTMQSFTTLAEKKADPPARPINAPTAEAMQLMLPRDVPETDKAEGDMYSVPVVENVKDFVALYSSLGVN
GDQCTIDEADEKENNAGTPIVKGGETEALKRLSIKVNNRHTWVATFAKPDTAPTAFDPPSTTILSPYLANGSI
SPRLFYYAILDCYEKNRKENGHAHTKSPTSLMAQLYWREFFTLCGRELPHFGAMVGNSICKQIPWSRDMALLR
AWEKSHTGFPWIDSVMTQLRTTGWIHHLARHSVACFLTRGDLWVHWEMGRDAFERHLLDADWSLNNANWMWLS
ASGFFHQFYRVYSPIAWPKKTDPNGDHVRHFVPVLKDMPKKYIYEPWLAPLTVQKKANCIIGKDYPYPVVDHN
TVKNANLTKMKDAYGRHSDGKINWEEDVAQNVSNADKVIQTTGIGEYEYVSKDTHNSGKRKAVAAPSEVDSDE
NTVKVEGQSSAPKKDKAKDINSKPTRGNRSKILRQTTLEF
>Spheniscus_mend__KAF1408180.1
WFASENKCFFTISSRFLLQCLEDLDANLRKLNSRLFVIRGQPADVFPRLFKEWNIAKLSIEYDSEPFGKERDA
AIKKLASEAGVEVIVRISHTLYDLDKIIELNGGQPPLTYKRFQTLISRMEPLEMPVETITPEVMEKCTTPVSD
DHDEKYGVPSLEELGFDTDGLPSAVWPGGETEALTRLERHLERKAWVANFERPRMNANSLLASPTGLSPYLRF
GCLSCRLFYFKLTDLYKKVKKNSSPPLSLYGQLLWREFFYTAATNNPRFDKMEGNPICVQIPWDKNPEALAKW
AEGRTGFPWIDAIMTQLRQEGWIHHLARHAVACFLTRGDLWISWEEGMKVFEELLLDADWSVNAGSWMWLSCS
SFFQQFFHCYCPVGFGRRTDPNGDYIRRYLPVLRGFPAKYIYDPWNAPESVQKAAKCIIGVNYPKPMVNHAEA
SRLNIERMKQIYQQLSRYRGLGLLATVPSNPNGNGNGGLMGYSPGESISGCGSTGGHLFPASPGAQLGTGDGH
TVVQPCALGDSHIGASGIQQQGYCQTSSILHYAHGDNQQSHLLQAGRTALGTGISAGKRPNPEEETQSVGPKV
QRQSTN
>Spheniscus_mend__KAF1420273.1
MPHRTIHLFRKGLRLHDNPTLLAALESSEVVYPVYILDRKFLTSVMHIGDLRWHFLLQSLEDLQKNLCQLGSC
LLVIQGEYESVLRDHIQKWNITQVTLDAEMEPFYKEMEANIRRLGEELGFEVLSLVGHSLYDTKRILDLNGGT
PPLTYKRFLHILSLLGDPEVPVRNLTAEDFQRCRPPDPGLAECYRVPLPVDLKISLESLSPWRGGETEGLQRL
EQHLTDQGWVASFTKPRTIPNSLLPSTTGLSPYFSMGCLSVRTFFYRLSNIYAQAKHHSLPPVSLQGQLLWRE
FFYTVASATPNFTKMAGNPICLQINWCEDAERLHKWKTAQTGFPWIDAIMTQLRQEGWIHHLARHAVACFLTR
GDLWISWEEGMKVFEELLLDADYSINAGNWMWLSASAFFHQYTRIFCPVRFGRRTDPEGQYIRKYLPILKNFP
SKYIYEPWTASEEEQKQAGCIIGQDYPFPMVNHKEASDHNLQLMKQVREEQYRTAQLTR
>Spheniscus_mend__KAF1453987.1
HLAYFVSRIIELNGHKPPLTYKRFQAIISRMELPKKPVSTVMSQQMETCKVDIQENHDDVYGVPSLEELGFPT
DGLAPAVWQGGETEALARLDKHLERKAWVANYERPRMNANSLLASPTGLSPYLRFGCLSCRLFYYRLWELYKK
VKRNSMPPLSLYGQLLWREFFYTAATNNPKFDRMEGNPICIQIPWDKNPEALAKWAEGKTGFPWIDAIMTQLR
QEGWIHHLARHAVACFLTRGDLWISWESGVRVFDELLLDADFSVNAGSWMWLSCSAFFQQFFHCYCPVGFGRR
TDPSGDYVKRYLPKLKGFPSRYIYEPWNAPESVQKAAKCIIGVDYPKPMVNHAETSRLNIERMKQIYQQLSRY
RGLCLLASVPSCVEDLSGPVADSASGQGCSTGTAVRLSQADQASPKRKHEGAEELCTEELYKRAKVTCLPAPE
IPGKSS

>Sphingomonas_sp._RZM33948.1_photolyase
MTTPVLLWLRQDLRLHDHPALVAATQEGPVIPVYILDDDAPGDWAIGGAQRWWLHHSLEAFAKSLDKKGSRLI
LRRGDDVATLRSLMTETGAERVFATRQYEPWWRKAEDALGDAICLHDGNQLARVESVTTGAGGQFRVYSSFWK
ALSQMMPPPEPVAMPRKIDAPETWPESDALESWELLPTKPDWSGGFSDWRPGEDAARKAVARFADVADDYDHD
RNIPSTEGTSRLSPHLHFGEISPRQVWHAVSGHGDGVTTFRKEVAWRDFTQGVVLALPDYGRRNGRPAFDALP
WRTGKAAASDLKAWQTGRTGYPIVDAGMRQLWATGWMHNRVRMITASFLVKHLLLDWREGERWFWDCLVDADY
GNNSVNWQWVAGTGVDSNMFGRIMAPLSQSEKFDAGDYIRQWVPELAKVTGDAIHDPDESGCRPRDYPAKIIG
HREARERALEAGRHAR
>Sphingomonas_sp._WP_128453466.1_photolyase
MPAILPHSLSSWSEAPRVRALNARDVRPDGRYVLCWLQQALRARDNPVIDAAIHLGNALGLPVIVYHGLREDY
PYASDRLHRFILGASRDLARECRQRGLACVQYVDRADAREKGLVYRLAADSAAVLVEDQPTFVARWQSERFAA
RIERAVFAVNAACLVPPAVLGDGIGSTTAFRRRHQPVRSDWQETTDAAPAVAPYDGPLPYTPDRLEACSDTDL
DHLVATAAIDHTLPVSTMFPAGREGALVHLERLMDTVLPTYAATRNDATNPVGASMLSPYLHFGVIGPRDVMA
TVEAADAPAASKEKFADELLTWREWFHYQARTLPAPERYDRIAEWAQETLEAHAVDPRPDIETLAALLHGETR
DETWNACQRQFLIDGWMHNNLRMYWGKRIIAMTPDPQTAWATACYLNDRLSLDGRDPSTYGNIAATFGGGSPG
REGPSVYGKVPTRSDGSTRHRPGGPAWLHAAATRSRPDPAVPSDLPTDLYLAGKSPL
>Spizaetus_tyra__NXJ47120.1
MPHRTIHLFRKGLRLHDNPTLLAALECSEVLYPVYILDRKFMTSVMHIGTLRWHFLLQSLEDLQKNLYKLGSC
LLVIQGEYESVLRDHIQKWNITQVTLDAEMEPFYKEMEANIRCLGEELGFEVLSLVGHSLYNTKRILDLNSGT
PPLTYKRFLHILSLLGDPEVPVRNLTAEDFQKCRPPDLGLAECYRVPLPVDLKITAESLSPWRGGETEGLQRL
EQHLTDQGWVASFTKPRTIPNSLLPSTTGLSPYFSMGCLSVRTFFYRLSNIYAQAKHHSLPPVSLQGQLLWRE
FFYTVASATPNFTKMVGNPICLQISWYEDAERLHKWKMAQTGFPWIDAIMTQLRQEGWIHHLARHAVACFLTR
GDLWISWEEGMKVFEELLLDADYSINAGNWMWLSASAFFHQYTRIFCPIRFGKRTDPEGQYIRKYLPILKNFP
SKYIYEPWTATEEEQKQAGCIIGQDYPFPMVNHKEASDHNLQLMKQVREEQYRTAQLTRDDTDDPMEMKVKRD
HSEENVSKGKVARMTE
>Spizaetus_tyra__NXJ48363.1
WFASENKYFFTIPCRFLLQCLEDLDANLRKLNSRLFVIRGQPADVFPRLFKEWNIAKLSIEYDSEPFGKERDA
AIKKLASEAGVEVIVRISHTLYDLDKIIELNGGQPPLTYKRFQTLISRMEPLEMPVETITPEVMEKCTTPVSD
DHDEKYGVPSLEELGFDTDGLPSAVWPGGETEALTRLERHLERKAWVANFERPRMNANSLLASPTGLSPYLRF
GCLSCRLFYFKLTDLYKKVKKNSSPPLSLYGQLLWREFFYTAATNNPRFDKMEGNPICVQIPWDKNPEALAKW
AEGRTGFPWIDAIMTQLRQEGWIHHLARHAVACFLTRGDLWISWEEGMKVFEELLLDADWSVNAGSWMWLSCS
SFFQQFFHCYCPVGFGRRTDPNGDYIRRYLPVLRGFPAKYIYDPWNAPESVQKAAKCMIGVNYPKPMVNHAEA
SRLNIERMKQIYQQLSRYRGLGLLATVPSNPNGNGNGGLMGYSPGESISGCGSTGGAQLGTGDGHTVVQPCAL
GDSHTGASGIQQQGYCQASSILHYAHGDNQQSHLLQAGRTALGTGISAGKRPNPEEETQTVGPKVQRQSTN
>Spizaetus_tyra__NXJ48371.1
QPSSLFSSSVLKVDYDVCSNYGNWLYSAGIGNDPRENRKFNMIKQGLDYDSNGDYVRLWVPELQGIKGADIHT
PWALNSAALSQAGVTLGETYPQPVVTAPEWSRHINQKPQGRSPHPRGRRGPAHTPMQHKDRGIDFYFSHKKDV
>Spizaetus_tyra__NXJ49940.1
MQHSSIHWFRKGLRLHDNPALLAAATDCRHLHPLFILDPSSSHAGTNAWRFLLDALRDLDGSLREMGSRLFVV
QGCPEEVFPRLFHAWGTTRLTFEVDTEPSARQHDATVAKLAERHGVEVIQEVSHTLYDTERVLALNDGKAPLT
YKGLQSLLASLGPPEKPAPTLTLEHLQGCHTPCQVSHDTEYGVPTLEELGQDPTEVGPHLYPGGERAALARLN
ALMDRTAWLCSFKKPETEPTSLSPSTTVLSPYLKFGCLSVRTFWWWLDEVYQGQREHSQPPVSLHRQLLWREF
FYTAGAGIPNFDRMVSNPICLQVNWDNNPQHLRAWREGQTGYPFIDAIMTQLRTEGWIHHLARHAVACFLTRG
DLWISWEEGLKVFEELLLDADWSLNAGNWLWLSGSAFFHRYFHIYSPVAFGKKTDRDGAYIRKYLPVLKDFPA
KYIYEPWKAPRTVQEQAGCLVGTHYPQPIVEHGAASKRNLGRMKAARTQK
>Spizaetus_tyra__NXJ51409.1
DNWAFLYAQRLAFKQELPLHVCFCLVPKFLDATIRHYGFMLRGLQEVAEECGELNIPFHLLLGYAKDVLPTFV
VEHGVGGLVTDFCPLRLPRQWVEDVRERLPEDVPFAQVDAHNIVPCWVASPKQEYSARTIRGKIHAQLPEFLT
EFPPVIRHPYPPSCPAEPVAWEACYSSLQVDHTVKEVEWATPGTAAGLAMLQSFIAERLKSFGSHRNDPNKAA
LSNLSPWFHFGQVSTQRAILEVQKHRGKYKESVDAFVEEAVVRRELAENFCYYNENYDSVQGAYDWAQTTLKL
HTKDKRPFLYKLQELEQGTTHDPLWNAAQLQMVREGKMHGFLRMYWAKKILEWTRSPEEALQFAIYLNDRYEL
DGRDPNGYVGKLQEGGTEQGALSLMAKATVGCLWSICGIHDQGWAERAIFGKIRYMNYAGCKRKFDVGQFERR
YTPRT
>Spizaetus_tyra__NXJ54151.1
CVYILDPWFAASSAVGINRWRFLLQSLEDLDNSLRKLNSRLFVVRGQPTDVFPRLFKEWGVTRLTFEYDSEPF
GKERDAAIVKLAKEAGVEVVIENSHTLYDLDRIIELNGHKPPLTYKRFQAIISRMELPKKPVSTVISQQMETC
KVDIQENHDDVYGVPSLEELGFPTDGLAPAVWQGGETEALARLDKHLERKAWVANYERPRMNANSLLASPTGL
SPYLRFGCLSCRLFYYRLWELYKKVKRNSTPPLSLYGQLLWREFFYTAATNNPKFDRMEGNPICIQIPWDRNP
EALAKWAEGKTGFPWIDAIMTQLRQEGWIHHLARHAVACFLTRGDLWISWESGVRVFDELLLDADFSVNAGSW
MWLSCSAFFQQFFHCYCPVGFGRRTDPSGDYVKRYLPKLKGFPSRYIYEPWNAPESVQKAAKCIIGVDYPKPM

VNHAETSRLNIERMKQIYQQLSRYRGLCLLASVPSCVEDLSGPVTDSASGQGCSTNTAMRLSQADQASPKRKH
EGAEELCTEELYKRAKVTGLPTPEIPGKS
>Sporisorium_sci_CDR99981.1_protein
MAASKCKDVSPPAKATKPEPHDEDEKVLDPKFAEHPDEDESETSKPPTKRQKKASPTKDDSRSAVINGGSKVA
NQYGSSAYAKAGDGWHRSDFNPSPKGRTDSKTPLDDLEAALKAHAPSKSNDESDAKNVVYWMRMHDLRIHDNR
ALAHASALASARRQKGKGGHLIALFTITPADYSAHDRGARRIDFVLRTLASLKNQFDDDLDIPFVVYTHHGAR
TKLGEKVFALCEKWNVSQLTGNIEYEVDELWRDISMVKSASDKKVAFSLFHDCYVVPPGRVLTNDGRPYSVFS
PWNRRWTDCISKDMSLIEASPDPQRNDKSIHKDSTLSALFNLDTLGDSYGIPDELPGFECKDRAYMAKLWPVD
GDAPRQVLDNFMKGKGGETALDRPANEPSNKEVGGNAKESRLGRYAEGRNLLNENGTSRISPYLAAGLVSARE
CLRRTKALTKDRLTVGRDSGAAMWNTEISFRDFYGHVLAAWPKVCMGHAFITKYEDVVWETDPSTLQAWKEGK
TGYPIVDAAQRQCIQQGYIHNRGRMISAMFLTKHLLHDWREGERHFSLNFIDQDFASNNGGWQWSASTGTDPQ
PYFRVFNSLSQSEKSDPQGDYIRHFVPELKNVKGNAVHDPFHRLSKAEFDKLGYPKPIVEHAEARQRALRRYK
NPGDK
>Sporisorium_sci_CDU23015.1_related_to
MTHNVLIAVLRNDLRLHDHPIFHLCSDPTPTSNPFAQPITHVLPVYVYDQRHIEVSAFPSLLKATSSGGGRGS
TQFAKTRELGIWRTGIHRTSFINESVFDLRDRLRGVGSDLALFAGTPESVIPALVREIRDKGDTVEGVFLGRE
INTEEVNVQKRLERVLGELQCPFKLFEGKSLIHSRDLGFPVKQLPDVFTHFKQVEGADMFRAPLPAPSKLKPF
PKVEIADAEGVYALSASKGLDTKEAVQKHLLQPLLDEPVLGHNELLSKVFSNGTINVPHSAFPYKGGETEALR
RLDHYFNGGKSSPGASYKETRNQMLGADYSTKFAAALAHGLLSPRLIAQKATELDSSTGNANNKAGGYWIIFE
LLWRDYFYFVGWKFDSHLFTLGGIKEQLSPKSAASKTYDWKSTTSLTSRTDPFIRWATAQTGIPLIDANMIEL
VQTGFMSNRGRQNVASFLTKDLGWNWQQGAEFFESWLVDYDPNSNWGNWQYVAGVGNDPRSSRQFNPIKQGKD
YDAKGEYVATWIPQLRGVPEQYRHHPWTQAGGGSVEGYPNRPMVEQQMWRKHYSNAGGAGGNGARNDGGGGGG
GGHQNNRS
>Sporisorium_sci_CDW98027.1_
MSKVARVLYWFCTDLRLHDSPALQSVLDLKPAALFPVWCWDPNYVYKHRVGINRFRFLLESMTALSRSITSTQ
SNPQLLVVRGEPTELLPELWKRWNITHLVIEKDPSAYDRRRDKLILKAAKKNNVEVVAVQGHHLYDPEEVVKR
NKGKPTMSMSTLQKIVADMGDVPKPIDAPKDLPLPTLKDFQAKSLSDTLSELAKSLEGLPHYTGKGREGLSSV
DLNSTELGGQRNKKVTCYDTINGKASELFSVPTLASLGMDAQKIRDTIKGSEPLALEKLAKICKDAEYVATFA
KPKTSPGQSAEDPSTTLMSPYLKFGCLSVRKFWWDVEEAKKKYKGGSKTGPPENFEWPVAVPRHNGDMILPRP
AGDPVSEARLSAYKLGQTGFPWIDALMRQLRLEGWMHHLGRHSVAAFLTRGQCWISWERGAEIFDKYLIDWDP
CSNPGNWMWLSCSAFFTQYFRLYGLATFPAKYDKTGALVRKYCPELAKFPDKYIYEPFNAPKDVQQKAGCIVG
KDYPFPMLDEKETKGENMERMKICYQANLHGTSKEVLEGKADEMLGKKHGFKDAPKEFDWNNRPGAKIPDWAK
APKGEEHTTEGAPKGGNGASTKKDEDGEAESDGDDSDDVVYEREEEQKEECGDKKRKAPVKEENGKSKKKK
>Steatornis_cari__NWX43228.1
DNWAFLYAQRLALKQELPLHVCFCLVPKFLDATIRHYGFMLKGLREVAEECTELNIPFHLLLGYAKDVLPTFV
VEHGVGGLVTDFCPLRLPQQWVEDVRERLPEDVPFVQVDAHNIVPCWVTSPKQEYSARTIRGKIHTQLPEFLT
EFPPVICHPYPSSCQAEPITWEACYSSLQVDRTVKEVEWATPGTAAGLAVLQSFIVERLKSFGSHRNDPNKAA
LSNLSPWFHFGQVSTQRAILEVQKHRGKYKESVDAFVEEAVVRRELAENFCYYNEKYDSMQGVYNWAQTTLKL
HAKDKRPFLYKLQELEQGTTHDPLWNAAQLQMVQEGKMHGFLRMYWAKKILEWTHSPEEALQFAIYLNDRYEL
DGTRGSADTLSPCPAGCLWSICGIHDQGWAERAIFGKIRYMNYTGCKRKFDVGQFERRYAPRM
>Steatornis_cari__NWX44729.1
KVDYHVCSNYGNWLYSAGIGNNPRDNRKLNMIKQGLDYDGNRDYVQLWVPELQGVKGVDIHTPWALNRTALSQ
VEVTLGETYPQPVVTAPDWSWHINQRPRGRSPHLRGRRGHAHTPAQHKDRGTDFYFSRKKDI
>Steatornis_cari__NWX45383.1
MGVNAVHWFRKGLRLHDNPALRECIQGADTVRCVYILDPWFAGSSSVGINRWRFLLQCLEDLDANLRKLNSRL
FVIRGQPADVFPRLFKEWNIAKLSIEYDSEPFGKERDAAIKKLASEAGVEVIVRISHTLYDLDKIIELNGGQP
PLTYKRFQTLISRMEPLEMPVETITPEVMEKCTTPVSDDHDEKYGVPSLEELGFDTDGLPSAVWPGGETEALT
RLERHLERKAWVANFERPRMNANSLLASPTGLSPYLRFGCLSCRLFYFKLTDLYKKVKKNSSPPLSLYGQLLW
REFFYTAATNNPRFDKMEGNPICVQIPWDKNPEALAKWAEGRTGFPWIDAIMTQLRQEGWIHHLARHAVACFL
TRGDLWISWEEGMKVFEELLLDADWSVNAGSWMWLSCSSFFQQFFHCYCPVGFGRRTDPNGDYIRRYLPVLRG
FPAKYIYDPWNAPESVQKAAKCVIGVNYPKPMVNHAEASRLNIERMKQIYQQLSRYRGLGLLATVPSNPNGNG
NGGLMGYSPGESISGCGSTGGHLFPASPGAHLGSGDGHTVVQPYALGDSHTGASGIQQQGYCQASSILHYAHG
DNQQSHLLQAGRTALGTGISAGKRPNPEDDTQNVGPKVQRQSTN
>Steatornis_cari__NWX51041.1
RFLLQSLEDLDNSLRKLNSRLFVVRGQPTDVFPRLFKEWGVTRLTFEYDSEPFGKERDAAIVKLAKEAGVEVV
IENSHTLYDLDRIIELNGHKPPLTYKRFQAIISRMELPRKPVSPVTSQQMEACKVDIQENHDDVYGVPSLEEL
GFPTDGLAPAVWQGGETEALARLDKHLERKAWVANYERPRMNANSLLASPTGLSPYLRFGCLSCRLFYYRLWE
LYKKVKRNSSPPLSLYGQLLWREFFYTAATNNPKFDRMEGNPICIQIPWDRNPEALAKWAEGKTGFPWIDAIM
TQLRQEGWIHHLARHAVACFLTRGDLWISWESGVRVFDELLLDADFSINAGSWMWLSCSAFFQQFFHCYCPVG
FGRRTDPSGDYVKRYLPKLKGFPSRYIYEPWNAPESVQKAAKCIIGVDYPKPMVNHAETSRLNIERMKQIYQQ

LSRYRGLCLLASVPSCVDDLGGPVADSASGQGCSTSTAVRLSQADQASPKRKHEGAEELCTEELYKRAKVTGL
PAPEIPGKSI
>Stegastes_part__XP_008276779.1
MAQTCIHWFRKGLRLHDNPALMAALRDCKELYPVFILDPHLHNNTCVGINRWRFLIGALKDLDCSLRKLNSRL
FVMRGRPEDVLPKLFQKWNVTKLTYEYDTEPYSLSRDKKVTELAKEHGVEVIYKISHTLYDIERIIEENNGKT
PLTYNRLQAIVKGLGSPKRPIPAPAMENMKDVKTPCSEKHEEKYGIPALEELGHDTTSLGEDLFPGGEQEALR
RLDEHMKRTGWVCNFEKPQTSPNSLSPSTTVLSPYVTFGCLSVRTFWWRLTDVYQGKKHSAPPVSLHGQLLWR
EFFYTASVGIPNFNKMKGNHVCTQVDWDTNPEHLAAWREARTGFPFIDAIMTQLRQEGWIHHLARHAVACFLT
RGDLWINWEEGQKVFEELLLDGDWALNAGNWQWLSASTFFHQFFRVYSPVAFGKKTDKNGDYIKKYLPLLKKF
PAQYIYEPWKAPRSIQQAAGCIVGKDYPRPIVEHEVISKKNIQRMKMAYAKRSPDTTESPSKKQGVKRKAPSV
VDMLKKKSKTN
>Stegastes_part__XP_008283888.1
MLLCVWRSSSRLLKHLIQPNQRPSPVVFSTDSLTSEATMSDKKRKATSAAAGKEPSAKQPKLAPAKEEKKKDA
GWLQSLVQQQRSEKNDMKFNKKRLRFISGVEKIKQGSEGVLYWMLRDNRVQDNWALIRAQRLAMKENLPLHVC
YCLVVPESQLSTLRHYNFMLKGLEEVAKECKHLNIQFHLLHGAPGEVLPGFVSDRSFGAVVTDFSPLREPIQW
LKDVKKGLSKEIPLIQVDAHNIVPCWEASPKLEYAARTIRGKITKVLSDFLTDFPLVEKHPHTATRTAKPVDW
DKTLASLSVDRTVGETEWAKPGTKAGMAMLESFIDVRLKLFDTQRNDPNAPALSQLSPWIRFGHISAQRVALQ
VQRSGRTAGVSVASFIEELVVRRELTDNFCFYNEKYDSVEGAYEWARKTLKDHAKDKRPYLYTKEQLEKAKTH
DKLWNAAQYQMVSEGKMHGFLRMYWAKKILEWTSSPVEALSIALYLNDRYELDGQDPNGFVGCMWSICGIHDR
GWGERPIFGKIRYMNYKGCLRKFNVAQFEKKYCPKNL
>Stegastes_part__XP_008289212.1
MVVNTIHWFRKGLRLHDNPSLRDSIRGADTLRCIYILDPWFAGSSNVGINRWRFLLHCLEELDASLRKINSRL
FVIRGQPTDVFPRLFKEWQITHLSYEYDSEPFGKERDAAIQKLASEAGVEVVVRISHTLYNLDKIIELNGGQP
PLTYKRFQALINRMDAVELPAETITSNVVKNCLMPVSDDHDDRFGVPSLEELGFETEGLTTAVWPGGETEALM
RLERHLERKAWVANFERPRMNANSLLASPTGLSPYLRFGCLSCRLFYFKLTDLYRKVKKNSTPPLSLYGQLLW
REFFYTTATSNPCFDKMEGNPMCVQIPWDRNPEALAKWAEGRTGFPWIDAIMTQLRQEGWIHHLARHAVACFL
TRGDLWIRWEEGMKVFEELLLDADWSVNAGSWMWLSCSSFFQQFFHCYCPVGFGRRTDPNGDYIRRYLPILRG
FPAKYIYDPWNAPEEVQKAAKCIIGVHYPKPMVNHAEASRVNIERMKQIYQQLSCYRGLGLLATVPANPNSSA
NGSSVGGVNPGTNQGARGSSEEPSLQEGTSQTDRGQSIQKRRREETPPESSSKVWKQSK
>Stegastes_part__XP_008295949.1
MSTSRTVICLLRNDLRLHDNELFHWAQRNAEHVVPLYCFDPRHYVGTYNYNLPKTGPFRLRFLLESIRDLRNT
LLNKGSNLVVRRGKPEEVVADLIKQLGSVGSVTFHEEVTSEELNVEKKVKEVCAKMKVKVHTCWGSTLYHRDD
LPFPHMARLPDVYTQFRKAVEAQSRVRPVFPTPEKLNPLPPGLEEGAIPSAEDLQQTEPVSDPRSAFPCSGGE
SHALARLKHYFWDTDAVATYKETRNGLIGVDYSTKFAPWLAMGCISPRYIYDQIKQYESKRTANQSTYWVIFE
LLWRDYFRFVAVKYGNRLFQLKGLQDKSVPWKKDMKLFNAWKEGRTGVPFVDANMRELAMTGFMSNRGRQNVA
SFLTKDLGLDWRMGAEWFEYLLVDYDVCSNYGNWLYSAGIGNDPRENRKFNMIKQGLDYDGNGDYVRQWVPEL
QAVRGADVHTPWTLSSAALSHAHVSLGDTYPTPIVTAPEWSRHINKKPSGAGPSPRGRKGPSHTPKQHRDRGI
DFYFSRSKNL
>Stegastes_part__XP_008303150.1
MVVNSVHWFRKGLRLHDNPALQEALNGADTVRCVYILDPWFAGAANVGINRWRFLLEALEDLDSSLKKLNSRL
FVVRGQPTDVFPRLFKEWNVTRLTFEYDPEPYGKERDGAIIKMAQEFGVETVVRNSHTLYNLDRIIEMNNNSP
PLTFKRFQTIVSRLELPRRPLPPITQQQMDKCRTKIADNHDLLYSIPSLEELGFRTQGLAPAVWRGGESEALD
RLNKHLDKKVKKKNNRPVFPSSCLHQLRKRCSPPLSLFGQLLWREFFYTAATNNPNFDRMDGNPICVQIPWDQ
NPEALAKWAEGRTGFPWIDAIMTQLRQEGWIHHLARHAVACFLTRGDLWISWESGMKVFEELLLDADWSVNAG
SWMWLSCSAFFQQFFHCYCPVGFGRRTDPSGDYIRRYIPILKDYPNRYIYEPWNAPEALQKAANCVVGVDYPK
PMINHAEGSRLNIERMKQVYQQLSHYRGLSLLASVPTIQEEAEPPMTDESQTSSGPDSPSRDLADSDAAGCST
APDSSTAAQHPDPDEAAQPPCVSSHQLQPGQPASPSSSSCPTSSPSPSPASVAQRRKGIARKARRNQRQRGRP
SYPPIARGEEREEAGGEERMEEDVEQEEERMEEEETAAQQQ
>Stegastes_part__XP_008304483.1
MPPPAADAQTVVRKMLRDVLAGREDPEGFFAMCVSVLGHQETRTQFPSLIQPLSTANRALHSILTSIYQEYFS
KTEDDELELALALSLLEMKDHQLSTPSQELQLLQPEDRPNQSSSVQLNSVSKSQGSNYTQPADVPDVRKNTDS
AQTGPWRNGSPPQTTRETELQKSTHLDKHKKQTPGPGQTVSFSKQDAVREGDQRMGTEDLIQSEKPKHSKNRR
QRRKGAGQQLVGLPCSPSAPPPVLLWFRRDLRLCDNPALMGSLEVGAPVIPVFIWSPEEEEGPGVTVAMGGAC
KYWLHQALSCFSSSLERIGSHLVFLKANGEGNEVGSSLLTLKELVKETGARTVLANALYEPWLKDRDDVVVSA
LKKDGVECRMFHSYCLRDPYSVSTEGVGLRGIGSVSHFMSCCKQNPASTLGVPLDPPVSLPTPSRWPQGAPLD
TLGLARMPRRKDGTTIDWAANIRKSWDFSEEGAHARLDAFLHDGVYRYEKESGRADAPNTSCLSPYLHFGQLS
PRWLLWDAKGARCRPPKFQRKLAWRDLAYWQLTLFPDLPWESLRPPYKALRWSSDRGHLKAWQRGKTGYPLVD
AAMRQLWLTGWMNNYMRHVVASFLIAYLHLPWQEGYRWFQDTLVDADVAIDAMMWQNGGMCGLDHWNFVMHPI
DAAMTCDPYGSYVRKWCPELADLPDELIHKPWKCPASMLRRAGVAFGQTYPERIITDLEGRRNQSLQDVALVR
KEFGQYVDKRSGCDLVPLPPRLVSEALGLSHGAVRTEGKQFLLPVITRMEFKHQLEDPDADAAANPYNAVLKG

YVSRKRDETIAFLNERDFTASVMYEGTQRKERLESDYRRMEGLPLLPSPRGRARRTPTAKDRFSVVPGGAVTS
LR
>Stegodyphus_dum_XP_035210488.1_photolyase
MLKVLCLHALKPRLRLLEYIKPKRGMSKQKKLTDYGNPAKKQKVEEIDEAETTEFLNNISSERLSCASSIKDF
KFNKKRVRVISEAKDIPKNAETIVYWMSRDQRVQDNWALLYAQYLAFKVDLPVCICFCLVPKFLDATIRHYRF
MLKGLKEVSEECESLNIHFHLLFGEAKDVLPNFVKSNKVGTIVTDFSPLRVPRKWLLDISKKLPSDVPLLQVD
AHNLVPCWIASDKQEYGARTIRKKIHDKLKEFLTGFPPVLKQKVTAKCMYEPTDWKNIEKHLDVDMNVGDVAW
AQPGTKGGLLKLYEFVKIRLKYFNDSRNDPTKDSLSNLSPWFHFGQISIQRSILVVLKYKSKYPASVDAFVEE
AVIRRELADNFCYYNSKYDEVEGAYDWAKKTLKDHSKDKREYLYSKEKLEDAKTHDPLWNAAQRQLIVEGKMH
GFLRMYWAKKILEWTQSPEEALEIAIYLNDKYSLDGRDPNGYV
>Stegodyphus_dum_XP_035216340.1_cry-1-like
MLVMCNLVHSEFMQWTKEACWIAIFYSFVDSDTNTVINCLFFILFYECNKDINLEMGESLKQTGTGIHLFRND
LRLHDMPSLSTILEVCSTLYPVYIFEDDDIQTQKIGYNRMRFILDALKDIDDKLHKVNGRLYVFQGKGIAILS
DLFKRWNVTHLSYECDVQPVWVETENNIKQICAESNIEIIKKVSHTLWDTEAIVKANGGSPPVTYSIFNHVIG
VLGLPEKPQSEVNFNEVIIPDDEKLDSKLLAKPEEFGINMECAEQINKIWIGGEIRALEHLEERLKIEKIAFS
SGYFMPNQINPDLLGPPMSMSAALSNGCLSVRRFYWSLSDLYKSVNSDSCLPDGLLPDGLMGQLIWREYFYAM
CMNNAHYSSMLDNPICLNIPWATNPSMLECWTDGKTGFPFIDAAMRQLRQEGWIHHVARNAVACFLTRGDLWI
SWEEGLKVFMKYLIDGDAPVCAGNWMWVSSSAFENVLQCPSCISPILYGRRFEPSGKYIRKYVPELKNMPHQF
LFEPWLAPLSVQEVANCIIGKDYPFPIVNHQQASKRNADMMTAIKESFLPKSVPLHCAPSNSAETRVFMWLPE
KCVENLLTCDN
>Stegodyphus_dum_XP_035217620.1_cry-1-like
MPVYKNLIQGKLFVIRGNPMDVLPEAVKRWNIKLLTYEHDTEPYAKQRDEDIDKMMEKLNVIVTKGYTNTLYS
PEQIIDYNGKVPLTYQSFLKSVEKIGLPAKPVPSFDMLKELKIAKGASTLIDSDYEEKYAVPTLEELRVNPEI
LYPCLYPGGESEGMKRLEHVLQDEEWICKFEKPNTSPNSLSPSTTVLSPYVKFGCLSARTFYFKIREVYMKSK
HGYSKPPVSLHGQLFWREFFYTVGSATPYFDQMIGNPICKQIPWKENKMLLEAWENAQTGYPFIDAIMTQLRQ
EGWVHHLARHAVACFLTRGDLWVSWVHGMKVFEELLLDADWSLNAGNWMWLSCSAFFHQYFRVYSPIAFGKKT
DKNGDYIRKYIPVLKRFPPEYIYEPWKAPKKLQEQLGCVIGKDYPARIVDHDEARVQNLRLMDAAYKGKSGNA
SAEVKISAPDLKNSRTSKPESELPPVTQSSKRQSLERKSEGEHSKFSKKKVKESLCNVRKRCKESDIKKYFIK
KN
>Stegodyphus_dum_XP_035227663.1_cry-1-like
MAQENGITVISRTSHTLYELEKIIEKNHGKSPLTYKQFQNILASMEPPPPPQPKITVEDVGNCYTPLSEDHDE
KYGVPTLEELGFDTENLKPPVWIGGETEALARLERHLERKAWVASFGRPKMTPQSLLPSQTGLSPYLRFGCLS
ARLFYQELAELYRKIKKVNPPLSLHGQILWREFFYCAATNNPKFDHMVGNSMCVQIPWDVNPEALAKWTNGQT
GFPWIDAIMTQLREEGWIHHVARHAVACFLTRGDLWISWEEGMKVFDELLLDADWSVNAGSWMWLSCSSFFQQ
FFHLYCPVRFGRKADPNGDYIRRYLPVLKNFPTKYIHEPWTAPEKVQLAAKCVIGKDYPMPMVNHQDVSHINL
VRMKQVYQQLSYYRGANMCNTLTTHSSQQRDDDGYPKIVKRKAVDYPDDNASFQEVLHSSINVLQQNFTFEST
DSQQQFFVL
>Stercorarius_para__NXG84728.1
LLKVDYDVCSNYGNWLYSAGIGNDPRDNRKFNMIKQGLDYDGNGDYVRLWLPELQGIKGADVHTPWALNGAAL
SQAGVTLGKTYPQPVVTAPEWSRHINQRPGRSPHPRGRRGPAHTPTQHKDRGIDFYFSRKKDL
>Stercorarius_para__NXG85121.1
CIYILDPWFAASSAVGINRWRFLLQSLEDLDNSLRKLNSRLFVVRGQPTDVFPRLFKEWGVTRLTFEYDSEPF
GKERDAAIVKLAKEAGVEVVIENSHTLYDLDRIIELNGHKPPLTYKRFQAIISRMELPKKPVSTVMSQQMETC
KVDIQENHDDVYGVPSLEELGFPTDGLAPAVWQGGETEALARLDKHLERKAWVANYERPRMNANSLLASPTGL
SPYLRFGCLSCRLFYYRLWELYKKVKRNSTPPLSLYGQLLWREFFYTAATNNPKFDRMEGNPICIQIPWDRNP
EALAKWAEGKTGFPWIDAIMTQLRQEGWIHHLARHAVACFLTRGDLWISWESGVRVFDELLLDADFSVNAGSW
MWLSCSAFFQQFFHCYCPVGFGRRTDPSGDYVKRYLPKLKGFPSRYIYEPWNAPESVQKAAKCIIGVDYPKPM
VNHAETSRLNIERMKQIYQQLSRYRGLCLLASVPSCVEDLSGPVTDSASGQGCSTSTAMRLSQADQASPKRKH
EGAEELCAEELYKRAKVTGLPAPEIPGKSL
>Stercorarius_para__NXG86034.1
MPHRTIHLFRKGLRLHDNPTLLAALESSEVLYPIYILDRKFMTSVMHIGTLRWHFLLQSLEDLQKNLRQLGSC
LLVIQGEYESVLRDHVQKWNITQVTLDAEMEPFYKEMEAKIRCLGEELGFEVLSLVGHSLYDTKRILDLNGGT
PPLTYKRFLHILSLLGDPEVPVRNLTAEDFQRCRPPDPGLAEGYRVPLPVDLKIPPESLSPWRGGETEGLQRL
EQHLTDQGWVASFTKPRTVPNSLLPSTTGLSPYFSMGCLSVRTFFYRLSNIYAQAKHHSLPPVSLQGQLLWRE
FFYTVASATPNFTRMAGNPICLQISWYEDAERLHKWKTAQTGFPWIDAIMTQLRQEGWIHHLARHAVACFLTR
GDLWISWEEGMKVFEELLLDADYSINAGNWMWLSASAFFHQYTRIFCPIRFGKRTDPEGQYIRKYLPILKNFP
SKYIYEPWTASEEEQKQAGCIIGRDYPFPMVNHKEASDHNLQLMRQVREEQPRTAQLTRDDTDDPMEMKAKRD
HSEENISKGKVARTAE
>Stercorarius_para__NXG93853.1

MQHSSIHWFRKGLRLHDNPALLAAATNCCCLHPLFILDPSSSRVGANAQRFLLDALRDLDGRLQEMGSRLFVV
RGCPEEVFPRLFRAWGTTRLTFEVDTEPPARQRDTAVAKLAAQHGVEVIRDVSHTLYDTERILALNNGKAPLT
YKHLQSLLASLGPPEKPAPALTQEHLRGCCTPCQVSHDIDYGVPTLEELGQDPTKVGPHLYPGGETAALARLD
VHMERTAWVCGFKKPETEPTSLSPSTTVLSPYLKFGCLSVRTFWSRLDEVYQGRKHSQPPVSLHGQLLWREFF
YTAGASIPNFDRMVGNPVCLQVDWDDNPQHLRAWREGQTGYPFIDAIMTQLHSEGWIHHLARHAVACFLTRGD
LWVSWEEGLKVFEELLLDADWSLNAGNWLWLSGSAFFHRYFHVYSPVAFGKKTDRDGAYIRKYLPILKDFPAE
YIYEPWKAPRAVQERAGCLVGTHYPWPIVEHRAASERNMGRMKAAR
>Stercorarius_para__NXG94507.1
DNWAFLYAQRLALKQELPLHVCFCLVPKFLDATIRHYGFMLKGLQEVAEECAELNIPFHLLLGYAKDVLPAFV
VEHGVGGLVTDFCPLRLPRQWVENVRARLPEDVPFAQVDAHNIVPCWVTSPKQEYSARTIRGKIHAQLPEFLT
EFPPIIRHPYPPSCPAEPIAWEACYSSLQVDRTVKEVEWATPGTSAGLAVLQSFITERLKSFGSHRNDPNKAA
LSNLSPWFHFGQVSTQRAILEVQKHRGKYKESVDVFVEEAVVRRELAENFCYYNENYDSVQGAYDWAQTTLKL
HAKDKRPFLYKLQELEQGTTHDALWNAAQLQMVQEGKMHGFLRMYWAKKILEWTRSPEEALQFAIYLNDRYEL
DGRDPNGYGRADVPSPCLAGCLWSICGIHDQGWAERAIFGKIRYMNYAGCKRKFDVDQFERRYSPRT
>Sterechinus_neum__ACO51513.1
REGKMHGFLRMYWAKKILEWTASPEEALEIAIYLNDRYSLDGRDPNGYVGCMWSICGIHDQGWAERPVFGKIR
FMNFKGCKRKFDVDTFINRYKKYNL
>Strongylocentrotus_pur_XP_785873.3_cry-1
MPKRKHRSSRRDREEKPGCLVHWFRKGLRLHDNPALKEGLKSASGFRCIYILDPWFAGSCSKGVNRWRFLLEC
LEDLDSSLRKLNSRLFLIRGQPADVLPRLFKEWKVTQLSFEEDSEPFGRTRDKAISTLAQEAGVKVISKVSHT
LYDPQEILALNNNEPPLTYKRFQDIISLMGIPIYPAEALEAEDVEGLDTFIDPNHEDKYGIPTLEELGFDPED
VPPPMWIGGETEAKQRLDRHLERKAWVANFERPRMSPASLMASPAGLSPYLRFGCLSPRTFYWKLTELYQKVR
KTTNTPLSLHGQLLWREFFFTVACNNRQFDHMVDNPICIQIPWDKNTALLNKWANGETGYPWIDAIMTQLRLE
GWIHPLARHAVACFLTRGDLWISWEEGMKVFDEYLLDADWSVNAGNWIWLSCSSFYQQFFHCYCPVKFGRRTD
PNGDYVRKYLPFLKNFPSKYIFEPWTAPIEVQEKAKCIIGKDYPLPIVDHAEASHRNIERMRKVYNNLSRRGG
PGILGSVPSSQAASAPPQLGKFNMSSKLMMTKQQHQQQTQETPMAPPAGNDSDLMPRPAKMRFMGTGMSHMQM
LLGGNTSTGIGGTGVVVGPGVSGVGAGVGVGGVSLGAGVSGIGPGVGVGPGVSGIGAGVSGIVSVGVPCVRGV
EVSGINVTDVTSHLPGSSGTNVLSYQPSLDMQQQHQRQQQQQQHQQQHQQQQQQLSQQIMPVDMATLSQMDVP
GTSGSLLKELEGTNWQTHWQFTNQ
>Strongylocentrotus_pur_XP_030838243.1_photolyase
MGKDGQTAKRSSEGQDNGETSAAKKVKKEEDGAEVQDLQTKVASLRNASGSSILNFQFNKKRVKILSDTMDIA
EDNRGIVYWMSRDQRVQDNWALLFAQRLAMKQEVPLHVCFCLVPKFLEGTIRHFNFVLEGLKEVSQELHLLDI
SFHLLIGYAKDVLPGFIKDHGLGAVVTDFSPLRTSSQWVTDVKEELPKNIPFCQVDAHNVVPCWEASNKLEYG
ARTIRPKITKQLSTYLTEFPPVICHPQKAKAKAEPIDWEGAYASLEVDQTVKPVDWAKPGTSEGMKMLDSFVK
ERLRYFSSARNDPTKSVCSNLSPWIHFGQLSSQRAALIVRLYRSRFSESVAAFIEESIIRRELSDNFCFYNDK
YDSIEGTNDWAKKTLKDHAKDKRDYVYSRETLERAKTYDQLWNSAQKQMVREGKMHGFLRMYWAKKILEWTTS
PEEALEIAIYLNDRYSLDGRDPNGYVGCMWSICGIHDQGWGERPVFGKIRFMNYKGCKRKFDVDTFVNRYKKY
NL
>Strongylocentrotus_pur_XP_030843606.1_cry-1
MPGGACIHWFRHGLRLHDNPALLEGMTLGKEFYPVFIFDNEVAGTKTSGYNRWRFLHDCLVDLDEQLKAAGGR
LFVFHGDPCLIFKEMFLEWGVRYLTFESDPEPIWTERDRRVKALCKEMKVECIERVSHTLWNPDIIIEKNGGT
PPITYSMFMECVTEIGHPPRPMPDPILTKVNMKIPSDFEERCALPSLEVMGVNMECTEQEKKVWKGGETRALE
LFRVRILHEEEAFKGGYCLPNQYMPDLLGTPKSLSAYLRFGCLSVRRFYWKIHDTYSELKKEVSPSHLTAQVI
WREYFYTMSVGNIHFNKMKENPICLNIEWKEDDEKLKAWTDGRTGYPWIDACMKQLKYEGWIHQVGRHATACF
LTRGDLWISWEDGLQVFDKYLLDADWSICAGNWMWISSSAFEKFLQCPNCFCPVRYGRRMDPTGEYVRRYLPV
LKDMPIRYLFEPWKAPRAVQERAKCIVGKDYPMPVVEHKSASAANHEQMEKVVNKLRDTGIVHCAPSTQREVR
EFVWLPEKMAGGGSCRADQNCEGILGL
>Strongylocentrotus_pur_XP_030853363.1_cry-2_X2
MPHNSTIHWFRKGLRVHDNPALLTAIQGTKVFRPIFILDPHFIESGKVGINRWRFLLETLQDLDNSFKALGTR
LFVVRGNPTTVFPEIFKKWNVTRLTFEVDTEPYARRRDQEVIELAKKNDVEVITKVSHTLYDTERTIKANKYK
PPMTYQRMVGLLSEIGAPAIPELPPLMANFTGVATPVKPDHDNEYGVPSLEDLGLDLEGLGPRLYPGGETEGL
QRMDLHLARKSWVCGFEKPKTSPNSLEPSTTVLSPYLKWGCLSPRKFYYAIKEVYAQQTNCTKPPVSLMGQLI
WREFFYTVAAGTPNFHQMEENPICIQVPWDENPEFLAAWKEGRTGYPYIDAIMTQLRTEGWIHHLARHSAACF
LTRGDLWQSWVKGQEVFDEWLLDADYSLNAANWMWLSSSAFFHQYYRVYSPVVFGKKTDKTGEYIRKYIPALN
KLPAEYIYEPWTAPRSVQEAAGCIIGRDYPRPIVDHSIVSKRNIGRMKDARACQPGKKAEKRPAEPSKQDNNG
KKVRKITSMLKKK
>Strongylocentrotus_pur_XP_030855350.1_cry_DASH
MAGRMKIVICLLRNDLRYRDNEVLFWAHKNATNVIPLYCFDPRHYKGTHQFGFPKTGPHRLKFLLESVRDLRT
TLQSVGSGLVVRSGNPEDVVPQLIQQFGEGEVAAVALQEEATREELDVEAGLHQSCSKLGVQVKKFWGSTLYH
REDVPFSPQGVPNVYTEFRKKVENRSHVRPPINMPKPLKGLPQGVEEGDIPVFSSFGLKDPGSDSRTAFPFQG

GETTGLARIEDYFWKSDCIAKYKETRNGLIGSEYSTKFAAWLAHGCVSPRQIHAEVKRYEKERTENQSTYWVI
FELIWRDYFKFVALKFGDKMFYLSGLLGVHKPWNKNKQWFDAWCKGQTGVPFVDANIREMAATGFMSNRGRQN
VASFLTKDLVLDWRLGAEWFEQMLVDHDVTSNYGNWLYSAGVGNDPRQDRKFNMIKQGLDYDPNGDYIRLWVP
ELSGIKGGSIHMPWTLSSAQLNQAGVSLGETYPSPIVTAPEWSRHSKRPQSGRGGAGGSQGGGSQGGGRNQSG
KGQRRNQGPPRGQQRGLDFYFSNPSQK
>Struthio_came_australis_KFV86176.1
RFLLQCLEDLDANLRKLNSRLFVIRGQPADVFPRLFKEWNIAKLSIEYDSEPFGKERDAAIKKLASEAGVEVI
VRISHTLYDLDKIIELNGGQPPLTYKRFQTLISRMEPLEMPVETITPEVMEKCTTPVSDDHDEKYGVPSLEEL
GFDTDGLPSAVWPGGETEALTRLERHLERKAWVANFERPRMNANSLLASPTGLSPYLRFGCLSCRLFYFKLTD
LYKKVKKNSSPPLSLYGQLLWREFFYTAATNNPRFDKMEGNPICVQIPWDKNPEALAKWAEGRTGFPWIDAIM
TQLRQEGWIHHLARHAVACFLTRGDLWISWEEGMKVFEELLLDADWSVNAGSWMWLSCSSFFQQFFHCYCPVG
FGRRTDPNGDYIRRYLPVLRGFPAKYIYDPWNAPESVQKAAKCIIGVNYPKPMVNHAEASRLNIERMKQIYQQ
LSRYRGLGLLATIPSNPNGNGNGGLMGYSPGENISGCGSTGGAQLGTGDGHAVGVQTCALGDAHTGGSGIQQQ
GYCQASSILHYAHGDNQQSHLLQTGRTSLGTGISAGKRPNPEEETQSIGPKVQRQSTN
>Struthio_came_australis_KFV86291.1
RFLLQSLEDLDNSLRKLSSRLFVVRGQPTDVFPRLFKEWGVTRLTFEYDSEPFGKERDAAIVKLAKEAGVEVV
IENSHTLYDLDRIIELNGHKPPLTYKRFQAIISRMELPKKPVSSIISQQMETCKVDIQENHDDVYGVPSLEEL
GFPTDGLAPAVWQGGETEALARLDKHLERKAWVANYERPRMNANSLLASPTGLSPYLRFGCLSCRLFYYRLWE
LYKKVKRNSTPPLSLYGQLLWREFFYTAATNNPKFDRMEGNPICIQIPWDRNPEALAKWAEGKTGFPWIDAIM
TQLRQEGWIHHLARHAVACFLTRGDLWISWESGVRVFDELLLDADFSVNAGSWMWLSCSAFFQQFFHCYCPVG
FGRRTDPSGDYVKRYLPKLKGFPSRYIYEPWNAPESVQKAAKCIIGVDYPKPMVNHAETSRLNIERMKQIYQQ
LSRYRGLCLLASVPSVEDLSGQVADPALGQSSSASTAMRLPQSEQASPKRKHEGAEELCTEELYKRAKVSGLP
APEVPGKS
>Struthio_came_australis_XP_009665590.1
MPHRTIHLFRKGLRLHDNPTLLAALESSEVVYPVYILDRKFMTSVMNVGALRWHFLLQSLEDLQKNLLQLDSC
LLIIQGEYESVLRDHVKKWNITQVTLDAEMEPFYKEMEANIRSLGEELGFEVLSLIGHSLYDTKRILDLNGGT
PPLTYKRFLHILSLLGDPEVPVRNLTAEDFQRCRPPDLCLAECYRVPLPVDLKIPPENLSPWKGGETEGLQRL
EQHLMNQGWVASFTKPRTIPNSLLPSTTGLSPYFSLGCLSVRTFFYRLSNIYAQSKHHSLPPVSLQGQLLWRE
FFYTVASATPNFTKMAGNPICLQINWYEDAERLHKWKTAQTGFPWIDAIMTQLRQEGWIHHLARHAAACFLTR
GDLWISWEEGMKVFEEMLLDADYSINAGNWMWLSASAFFHQYTRIFCPVRFGKRTDPEGQYIRKYLPILKNFP
SKYIYEPWIASEEEQKQAGCIIGQDYPFPMVNHKEASDHNLQLMKQVREEQYRTAQLTRDDADDPMEMKIKRD
HSEENVKKGKMARTAEQTXILSGITFWELKNSKWGEPEASSGNSCIVSS
>Struthio_came_australis_XP_009680395.1
LQDKEVPWKKDLQLFDCWKEGKTGVPFVDANMRELAATGFMSNRGRQNVASFLTKDLGLDWRMGAEWFEYLLV
DYDVCSNYGNWLYSAGIGNDPRDNRKFNMIKQGLDYDGNGDYVRLWVPELQGIKGGDIHAPWVLSSTSLSQAG
ITLGETYPLPVVTAPEWSRHISQKPQGRGHHPKGRKGPAHTPTQHKDRGIDFYFSRKKDI
>Struthio_came_australis_XP_009680452.1
MPQKKEKRKAGQGGKTEAEPDTTELPRFPPKRNKEEAALGKLEEAVQEARQKAAPSVQDFKYNKKRVRLISQG
SDLKDGAKCILYWMSRDQRVQDNWAFLYAQRLALKQELPLHVCFCLVPKFLDATIRHYGFMLRGLQEVAKVDA
HNIVPCWVTSPKQEYSARTIRGKIHSQLPEFLTEFPPVIPHPYPPSSLAEPVAWEACYSSLQVDRTVKEVEWA
TPGTAAGLAVLQSFIAERLESFGSHRNDPNKAALSNLSPWFHFGQVSTQRAILEVRKHHRKYKESVDIFVEEA
VVRRELADNFCYYNKNYDSVQGAYDWAQTTLKLHAKDKRPFLYELQELEQGNTHDPLWNAAQLQMVREGKMHG
FLRMYWAKKILEWTRSPEEALQFAIYLNDRYELDGRDPNGYVGCLWSICGIHDQGWAERAVFGKIRYMNYAGC
KRKFDVSQFERRYAPHKLKK
>Stylonychia_lem_CDW77913.1_cry_5
METISKKFTIYWFRKCQRIHDNKGLLDSSIQKNLLPIFILDPWFIKSDKTGTNRIKFLLESLSDLNANLKTYN
SNLIILYGNPSDIFEKLAKHSENLYFELDTEPYALDRDRKVQKICEKEGVQVHRNAGHTLLKLADLAQAGESC
KNMGDFLQFIKESMIDKPLEKPDDLPSLPADYEKILKSLKIEYSNQVPSLSEIDRNENEATASLKGGETEALN
IMNDYLKDKKKVNNFSKPFTSPNSLKPSTTTLSPYLKFGCLSPRLFYHNLKKVMDRNATQPPVSLVGQLFWRE
FFYSKSYTVKNFDKMVGNPICKQMNWERNEEIIRKWEMGQTGFPAIDAVMNQLRQEGWMHHLGRHLVACFLTR
GHLYQHWEAGRDVFDKYLLDADYALNNANWMWLSCSSFFSQYWKVYSPISFFQKTDKNGDFIRKYVPQLKHYP
AEFIYEPHKAPIMVQKRCKCIIGEDYPLPIIDYKKEGAINMQRMKQVFADSKDQSIKEEKKSISVTPKKGKVV
KEESKSNTKTIKDFMSDKNQVKKAAEEWALGEDQNIQNDKKAIHSNQNLSSKSRKKSLKQ
>Stylonychia_lem_CDW81657.1_cry_dash
MEKRSNKRKLDQISNQEEEKGTVPVKQENSKKVKKNPEESQQTPKTEEPSNNKMQRIILWFRNDLRLHDNAIL
NYAVTKVAKNKQIIPVFCFDPRFYGRKVQKYDTLKTGLVRQRFMIETVQNFRERLEKVGSKLLVSMDKPEIFL
PTLIDKSCDNTIVYQDEICSEELTVEKLVKEKCKKAEIKSFWGSSCYHLDDLGFHPNDLPHIYGKFREKTEGV
KVRPLFGQPKQDQLPFIEKPSKDILEAAEFMPTLKDFGFSKEEVEKPRDKRACYEFIGGEEQATKRLTEYIFE
TKSVGNYALVRNNLIGANYSSKLSPWLACGALSPRYIYFQVKEFEKKHRTNESTKVFLDELFWRDFYRFWCMK
YGSKVFSSYGIYNREYYDWTTDKETVKKWKEGNTGMPIIDALMREMNVTGFMPNRGRMIVACYLTMDLKQDWR

EGAYHFEEKLIDHDVQSNYGGWNFSAGIGPGRVLVFNSVKQSKDHDKNGDYIKMWVPELKKVPVEYIHEPWTM
PKDLQKGCKTVIGEDYPYPINCSKYTNKVAHTIEKQAKKSKRAEEKLKQTKLTK
>Stylophora_pis_XP_022786103.1_cry_DASH-l
MEGGKTVVIYLLRNDLRVHDNECLHWAHQNSDFVIPLFCFDKSILSPGAITWHYKFAKTATHRAKFILESVSD
LRESLRKLGSDLVVRSGQTAKEAVSDIINLCQAKNEGHVSLVYQREITKEETDVEEELLKLCEAEKVTVKSFW
GLTLYHLEDLPFASVKFLPDTYTDFRKNVEAKCRVRPLIPTPDCLKSLPAFLKDDQVGRMPTLQELAMDHSEE
TADQRSAFPFSGGETAALARLNQYLWDTDLVARYKETRNGLIGSDYSTKFSPWLAVGALSPGKVFDCIKKYET
ECTANQSTYWVIFELLWRDYFKFVSLKYGVSIFYLSGIMRKTGLPWKCDMVAFDKWRYGQTGVPFVDANMREL
LYTGWMSNRGRQNVASFLVKDLGLDWRMGAEWFESLLLDHDVSSNYGNWNYSAGIGNDPRENRKFNMVKQALD
YDPDGDFVRLWVPELAGLKGAKVHTPWLLSSSELKHAGITLGGNYPKPMVCPPEWSRHTSKVKDSRKTLRAQP
RGIDFYFKSPKGAGSSRKRH
>Stylophora_pis_XP_022794023.1_uncharacte
MTSNMNSLHWFRKGLRLHDNPSLRQAVEGSSTFRAVFFLDVDSLDEINLSKNKWLFLLECLLDLDKSLRRLNS
RLFIVRGQPIDVFPKLIRQWNITRVTYEYDSEPFPRRRDDSIKRLLESEGVEVLTSVSHTLYDIDEVIKSNHG
NVPVTFKQFECLVSKLNSPSNCALVVDEQLFKNCKTPVEDDDDETYGVPEYDALDFGEEEAEPRQFVGGETEA
LKRLAVLEKEVAEPTLGKPHHESNSPFPKSSKLSPYLRFGCLSVRHLFHRMNQVYREANGRSAPMSVHLPLLW
REFYFVVASKHPNFDKMKNNSLCLQFPWEEQTCKLEKFRQGKTGFPWIDAMMRHMLSEGWIPHLARQAVGCFL
TRGALWICWEEGFKIFEKYMLDAEWSINAGSWMWLSCSAFFAKHPQWTCPVGLGKHLDPQGNYVRKYVPELKD
FPSEYIYEPWDAPLELQEACNCVIGTDYPQPIVNHQQARKNCVDRLRGVYQNLVTKVSCNVGLGHNAMHWFRK
DLRLHDNPSLCEALTNCRTFHAVYILDPVSARAAKVSANRWKFLVDCLRDLDKSLRECGSRLHVIRGQPTYVL
PKLFQDWNISKLTFECEIEPFGQRRDVAVAVLGEEHGVEVISKPSHTLYDPEKIIESNEGEAPMLIKTFENVI
RQMGPPEKPVDAVNKEMFKGCICPVSNDHSTRFGIPSLDELGYRGCEVTTGDLWVGGEQEAIKRLTELEEKVL
EGSLKKSVEGLDALRPSRTQLSPYLRFGCLSPRLFHLRLTKAYMKVKCQPPPLSLYRQLVWREFFFTLASRNP
QMDRAVDNPLSFNIPWEENQDALDAWKEGHTGFPWIDAIMRQLHAEGWIHHVARQAVGCFLTRGCLWISWEEG
FKLFEQLQLDSEWSLNAGNWLWVSCSAFFHGQIPWYCPVNVGKKLDPSGNFIRRYVPELKTMPTKYIHEPWMA
PLAVQKASNCIVGQDYPDRLCDHIERRKICLERLKDVCQQIQGTNSSSQDTADVVD
>Stylophora_pis_XP_022795820.1_photolyase
MVLFGCAGLIWKTARLVSMANEPPTKRAKKEIAETNHQNNTGQNTLAKYLEKRKNVCESVAEFKFNKKRVRLI
SSTTDMPESCKGIAYWMWREQRVQDNWAMLYAQRMALKQQVPLIVIFCLPSKYLDAAFRQYNFMIKGLQEVEK
ELTELNIAFHVLLGEPDKVLPDFVKSKEIGGVVADFTPLRRPLKWLEDIAKKLPKNVPLCQVDGHNIVPCWQA
SPKLEYGARTIRPKIHNQLKQFLTEFPPVIKHPHAGDYEVKVVDWEAVDNFIEVDRAVKDVDWAKAGTKAGLD
MLESFCDSRLKYFATDRNNPTKEALSNLSPWFHAGHISVQRAILRVREFRSKAKDSVEAFIEEAVVRRELADN
FCFYNLENYDSIKGTNAWAQKTLLDHAQDKREYLYTREELEDGNTHDDLWNAAQLQLVSEGKMHGFLRMYWAK
KILEWTATPEEALEVSLYLNDRFSLDGNDPNGYVGCMWSVCGVHDQGWAERPVFGKIRYMNYNGCKRKFDVAE
FVRKNGRKKQDTTGGTDKSLKNRKRKAAGCYNMFVGENPK
>Stylophora_pis_XP_022798906.1_cry-1-like
MSNATVQRKDEHVGSVEGRSSAKNEKEKHAIHWVRKDLRLHDNPSLLEAVKGSDTLRIIYVLDTKVDQNIGIG
ANLWRFLLQCLEDVDDSLRQLNSRLFVVRGQPADVFPRLFREWNTSLLTFEEDSEPFGREKDAAIRLLAKESG
VQVAVRRSHTLYDPQLIIKNNGGTPPLTYKKFLAIISSLGPPEHPVPTLDVHLLGSCSTPISDDHEEKYGVPS
LEELGMDIRKVHAAVWHGGEKEALVRLNRHLERKAWIASFEKPKVTPNSLFPSPTGVSPYLRFGCLSPRLFYH
RLTELYRKVKSSDPPISLYGQLLWREFFFTVAANNPHFDRMATNPMCLQIPWKNNPENLARWEEGRTGFPWID
AIMIQLQQEGWIHHLARHAVGCFLTRGNLWISWEEGMKVFERWLLDAEWSLNAGNWMWLSCSAFFQQFFNCIC
PVGFGRKLDPNGDFVRKYLPVLKGFPAKYIHAPWTAPESVQKAARCIIGIDYPHPMVDHTKVSCANLEKLRNI
FKALLCYKDSSAISLEKQDNLRGQQEKLKQQMFLLEDKENE
>Stylophora_pis_XP_022808227.1_cry-1-like
MASKQRRVHHTIHWFRKGLRLHDNLSLKEACETSLTLRPVYFIDPDYVKHGNMGFNRWRFLIQSLSELDKNLK
SIGSRLFVIQGKPELELPKLFKAWKITKLTFEFDHEPEGRQRDAKIHELATKAGIQVETRICHSLYDLDKIIS
KNGGTAPLTYKKLCSVVASLGPPLKPIPTVDKITFDGCSTPVGKENKYQLPTLEELEIETPEESSTVIFLGGE
TEALRRLDEHMQKEDWVNKFEKPNTSPNSLQPSTTVLSPYVMFGCLSARLFYHRLSDVYSRAKKHSNPPVSLH
GQLLWREFFFTAAYATPNFNKMEGNPVCLQIPWEKNQEFLQAWCEAHTGYPFIDAIMTQLRREGWIHHLARHA
VACFLTRGDLWISWEEGLKVFDKLLLDHDWSLNAGNWMWLSASAFFHAYYRVYSPIAFGKKTDPSGDYIKKYL
PILKKYPPKYIYEPWTAPLSVQKTAGCVIGKDYPRPIVDHKEAVKINLARMKKARTEGDSNNGMEEDSLEKKK
SSPAKRKNSKEAQSSKKPRKITEFFKTL
>Suberites_dom_CAZ66367.1_cry
MHCNYPLATQTTFQGQSIPDTTVHWFRLDALRLHDNPAFVDAVKTDGNFKAVFIIDPWFNANYNNGGPQVNVW
RFLLEALHDLDSRLQKKPYCARLNVLYGQPTMILPELYKKWNVKKITFQASQVSSESMKHDGIIKILSEQQNV
QAVSYFSHTLYDPANVIALNNGRVPLSYKEFRRLMPLMGKPASPIPEPHPMSLCMKAPPSELVPEPEGKIPKL
QDLGLSDEFALYTNSWVGGETEALSRLSSFCSRRAAIPNEPVHWLMSKDTLSPYIKFGCLSVRQLFSQLLQFA
STSSKGQELFELLTKNLLLREFAFLVGSSSPKFDVMEGNSLCIQLPWESNNVFIQAFRNGQTGYPWIDAIIRQ
IRQDGWAHFLARQSIAVFLTRGYLWISWVLGKEFFQEFMIDFELPVSSVCWMQSSCSGFFCTQIESYDPCLVG

KQIDTDGHYIKTYVPELKDFPSEYVHQPWKCSSLHTEASWLCDREQYPKPIIDVCKQGELCCKRVQSIMKALA
DVYGVE
>Sula_dact__NWI27891.1
DNWAFLYAQRLALKQELPLHVCFCLVPKFLDATIRHYSFMLRGLQEVAKECAELNIPFHLLLGYAKDVLPTFV
VEHGVGGLVTDFCPLRLPRQWVEDVREQLPEDVPFAQVDAHNIVPCWVASPKQEYSARTIRGKIHAQLPEFLT
EFPPVVRHPYPPSCPAEPIAWEACYSSLQVDRTVKEVEWATPGTAAGLAVLQSFIAERLKSFSSHRNDPNKAA
LSNLSPWFHFGQVSTQRTILEVQKHRAKHKESVDAFVEEAVVRRELAENFCYYNENYDSVQGAYNWAQTTLKL
HAKDKRPFLYKLQELEQGTTHDPLWNAAQLQMVREGKMHGFLRMYWAKKILEWTRSPEEALQFAIYLNDRYEL
DGTDPNGYVGKLQDGGMGRGGCLWSICGIHDQGWAERAIFGKIRYMNYAGCKRKFDVGQFERRYAP
>Sula_dact__NWI29137.1
MQHSSIHWFRKGLRLHDNPALLAAAANCHRLYPLFILDPSSDRGGANAQRFLLDSLQDLDGSLREMGTRLFVV
RGCPEEVFPHLFHTWGTTRLTFEVDTEPTAWQRDATVVELAARHGVEVIREVSHTLYDTKRILALNNGKAPLT
YKHLQSLLASLGPPAKPAPALTRKHLRGCHTPCQVSCDADYGVPTLEELGQDPTTAGSNLYPGGETAALARLD
AVMERTAWVCGFKKPETEPNSLSPSTTVLSPYLKFGCLSVRTFWWRLDEIYQGREHSQPPVSLHGQLLWREFF
YTAGASIPNFDRMVGNPVCLQVDWDDNPQHLHAWREGQTGYPFIDAIMTQLRTEGWIHHLARHAVACFLTRGD
LWVSWEEGLKVFEELLLDADWSLNAGNWLWLSGSAFFHRYFHVYSPIAFGKKTDRDGAYIRKYLPVLKDFPAE
YIYEPWKAPRAVQERAGCLVGTHYPQPIVEHQVVSKRNLGRMKAAR
>Sula_dact__NWI32173.1
KVDYDVCSNYGNWLYSAGIGNDPRDNRKFNMIKQGLDYDGNGDYVRLWLPELQGIKGADIHTPWALNSAALSQ
AGVTLGETYPQPVVTAPEWSRHVTQRPQGRSPHPRRGPARTPVQRKGRGIDFYFSRKKDV
>Sula_dact__NWI32941.1
WFTSENNSFSTNPCRFLLQCLEDLDANLRKLNSRLFVIRGQPADVFPRLFKEWNIAKLSIEYDSEPFGKERDA
AIKKLASEAGVEVIVRISHTLYDLDKIIELNGGQPPLTYKRFQTLISRMEPLEMPVETITPEVMEKCTTPVSD
DHDEKYGVPSLEELGFDTDGLPSAVWPGGETEALTRLERHLERKAWVANFERPRMNANSLLASPTGLSPYLRF
GCLSCRLFYFKLTDLYKKVKKNSSPPLSLYGQLLWREFFYTAATNNPRFDKMEGNPICVQIPWDKNPEALAKW
AEGRTGFPWIDAIMTQLRQEGWIHHLARHAVACFLTRGDLWISWEEGMKVFEELLLDADWSVNAGSWMWLSCS
SFFQQFFHCYCPVGFGRRTDPNGDYIRRYLPVLRGFPAKYIYDPWNAPESIQKAAKCIIGVNYPKPMVNHAEA
SRLNIERMKQIYQQLSRYRGLGLLATVPSNPNGNGNGGLMGYSPGESISGCGSTGGAQLGTGDGHTVVQPCAL
GDSHTGASGIQQQGYCQASSILHYAHGDNQQSQLLQAGRTALDTGISAGKRPNPEEETQSVGPKVQRQSTN
>Sula_dact__NWI33849.1
MPHRTIHLFRKGLRLHDNPTLLAALESSEVVYPVYILDRKFVTSVMQIGALRWHFLLQSLEDLQKNLCQLGSC
LLVIQGEYESVLRDHIQKWNITQVTLDAEMEPFYKEMEANIRRLGEELGFEVLSLVGHSLYDTKRILDLNGGT
PPLTYKRFLHILSLLGDPEVPVRHLTAEDFQRCRPPDPGLAECYRVPLPVDLKIPVESPSPWRGGETEGLQRL
EQHLTDQGWVASFTKPRTIPNSLLPSTTGLSPYFSMGCLSVRTFFYRLSNIYAQAKHHSLPPVSLQGQLLWRE
FFYTVASATPNFTKMAGNPICLQINWYEDAERLHKWKTAQTGFPWIDAIMTQLRQEGWIHHLARHAVACFLTR
GDLWISWEEGMKVFEELLLDADYSINAGNWMWLSASAFFHQYTRIFCPVLFGKRTDPEGQYIRKYLPILKNFP
SKYIYEPWTASEEEQKQAGCIIGQDYPFPMVNHKEASDHNLQLMKQVREEQYRTAQLTRDDTDDPMEMKVKRG
HSEESVTKGKVARMTE
>Sula_dact__NWI34460.1
RFLLQSLEDLDNSLRKLNSRLFVVRGQPTDVFPRLFKEWGVTRLTFEYDSEPFGKERDAAIVKLAKEAGVDVV
IENSHTLYDLDRIIELNGHKPPLTYKRFQAIISRMELPKKPVSTVMSQQMETCKVDIQENHDDVYGVPSLEEL
GFPTDGLAPAVWQGGETEALARLDKHLERKAWVANYERPRMNANSLLASPTGLSPYLRFGCLSCRLFYYRLWE
LYKKVKRNSTPPLSLYGQLLWREFFYTAATNNPKFDRMEGNPICIQIPWDRNPEALAKWAEGKTGFPWIDAIM
TQLRQEGWIHHLARHAVACFLTRGDLWISWESGVRVFDELLLDADFSVNAGSWMWLSCSAFFQQFFHCYCPVG
FGRRTDPSGDYVKRYLPKLKGFPSRYIYEPWNAPESVQKAAKCIIGVDYPKPMVNHAETSRLNIERMKQIYQQ
LSRYRGLCLLASVPSCVEDLSGPVTDSASGQGCSTSTAMRLSQADQASPKRKHEGAEELCAEELCKRAKVTCL
PAPEIPGKS
>Suricata_suri__XP_029811092.1
MGVNAVHWFRKGLRLHDNPALKECIQGADTIRCVYILDPWFAGSSNVGINRWRFLLQCLEDLDANLRKLNSRL
FVIRGQPADVFPRLFKEWNITKLSIEYDSEPFGKERDAAIKKLATEAGVEVIVRISHTLYDLDKIIELNGGQP
PLTYKRFQTLISKMEPLEIPVETITSEVIEKCTTPLSDDHDEKYGVPSLEELGFDTDGLPSAVWPGGETEALT
RLERHLERKAWVANFERPRMNANSLLASPTGLSPYLRFGCLSCRLFYFKLTDLYKKVKKNSSPPLSLYGQLLW
REFFYTAATNNPRFDKMEGNPICVQIPWDKNPEALAKWAEGRTGFPWIDAIMTQLRQEGWIHHLARHAVACFL
TRGDLWISWEEGMKVFEELLLDADWSINAGSWMWLSCSSFFQQFFHCYCPVGFGRRTDPNGDYIRRYLPVLRG
FPAKYIYDPWNAPEGIQKVAKCLIGVNYPKPMVNHAEASRLNIERMKQIYQQLSRYRGLGLLASVPSNPNGNG
GLMGYSPGENIPGCSSSGSCSQGSGILHYAHGDSQQTHLLKQGRSSMGPGLSSGKRPSQDEDTQSIGPKVQRQ
STN
>Suricata_suri__XP_029812989.1
MAATVVTAAATAPAPAAGADGASSVHWFRKGLRLHDNPALLAAVRGARCVRCVYILDPWFAASSSVGINRWRF
LLQSLEDLDTSLRKLNSRLFVVRGQPADVFPRLFKEWGVTRLTFEYDSEPFGKERDAAIMKMAKEAGVEVVTE

NSHTLYDLDRIIELNGQKPPLTYKRFQAIISRMELPKKPVGSVTGQQMESCRADIQENHDEAYGVPSLEELGF
PTEGLGPAVWQGGETEALARLDKHLERKAWVANYERPRMNANSLLASPTGLSPYLRFGCLSCRLFYYRLWDLY
KKVKRNSTPPLSLFGQLLWREFFYTAATNNPRFDRMEGNPICIQIPWDRNPEALAKWAEGKTGFPWIDAIMTQ
LRQEGWIHHLARHAVACFLTRGDLWVSWESGVRVFDELLLDADFSVNAGSWMWLSCSAFFQQFFHCYCPVGFG
RRTDPSGDYIRRYLPILKGFPSRYIYEPWNAPESIQKAAKCIIGVDYPRPIVNHAETSRLNIERMKQIYQQLS
RYRGLCLLASVPSCVEDLSNPVAEPSSSQTGSTSSAGPRPLAIGPASPKRKLEAAEEPPGEELSKRARVAAVP
SPELPSRGV
>Symbiodinium_mic_CAE7395975.1_UVR3
MAAGVWFRKCLRLHDNEPLVKAVASGLDVVPFFILDPHFDKSRVGVNRFSFLLQSLKDLDGQLRRRGSRLLVF
RGQPEQVLVELFGEKAPVHLSAIFWEADTEPYARARDERVLAMAKEGGVHAEKLSGHTLLDIDATVAKPGFKP
PISMKSIQTIVNAVGPFRQPFDVPKIPPLRIEGHNVPDISELYDEEPSSHGFPGGETEALARLQRTCSDVQYV
CCFEKPKTASTGRPGCPWEPSTTGLSPYLKFGCLSVRTAWYAIDRCIRGKSHSQPPQSLHGQLLFREMFYLLG
AAVPNFDRNEGNSMCKTIPWGCDESLLAAWEAGKTGYPFIDALMRQLKQTGFMHHLGRHAVACFLTRGDLWQN
WTAGRDVFDRLLLDADWAVNNGNWLWLAGVAPFSAPYFRIYDPCPGPKSSLNADQTGEFVKHYVPELRNMPAK
YLYKPWTAPVAEQKKAGCIIGEDYPKPIVDHQRAREENLAKFKAALDAKGGELPAKFGGAKGDPGPMRSSYTS
GKGATGKGGKRADTPQTEDGDSRKVRRWGKKLGDTVAGG
>Symbiodinium_mic_CAE7547081.1
MSANVASSVPVVEGVDVRRCRRLNSVQLKESTDPVIYWMSRDQRSVDNWALIYAQKLAIRQSAPLHVVFSLVP
KFLDATLRQYDFLLKGLREVAVDLESKNMPATFPFHLQLGKAGDKVPALVNELSAQMVVCDMSPLRVPSQWAR
DVADACQAKEVPVVQVDAHNVVPVWAASDKQETAARTIRKKLMQSLGTYLTDFPRVEKHPHKSKGNFQPVDWV
QAEQSLQVDDSVRPVAAIVPGTAAGLAALEDFCKRLSRYDAHRNDPNENALSGLSPWLHFGQISAQRCALRVR
LIGEIKTATSSEKKSCEAFIEESVVRRELSDNFCFYNVNYDSLEGAAGWAQQTLKDHSKDKREHLYCKEDLEN
AKTHDDLWNAAQLQMVREGKMHGFLRMYWAKKILEWSPSPKEALERCIWLNDRYELDGRDPNGYVGCMWSICG
VHDMGWKEREAEQWFSVNGDVAMMLARHALLNAFEEITAGEKEHILGCGTIRVRTYEDFMQNAIGGVVTRAAK
DLATWAIEVSPHATWHGCQAVELGAGCCLVSSVLMKLGAAVIATDLPELLPHMEYNLQLNDEGGSLLGRWAVD
SLNWDSTEARVKLRGRLGNNGADAIFATNCVYGRDTVDMFLSTMFALSGPCTLALMCGVPVPPAALAPEEVSI
LDEFLAACPRFFDCYLLKVPGGIEDAGVARVQDSPLAAELGKPYGLSAAALADGVWLFKLPGSDCPSWARPVL
CIGYGTTLQANLRQWQGMCWHSHIQLLRLQRKLISGLHPQ
>Symbiodinium_mic_OLP81567.1
MEGQGVANRFEGRCDLVLLGWTAFIVLNLASDYFWTRGSSEAEDVPTVGSMGMGTADIYMGSYTLETSAAPFG
AVHSHGSWRHAAVSWHSGVFVGFRIGNSRDPNTGELNASFTISAQLPRYSIEVPPSGGDTCFADAATAFERLE
SLEAVCSQAHHDALHNARKPGTYATMEEEQRRQTPLMAVPLVMTHPVTGRKHQDDLAGANSSVFHVQRRGADG
PSASLLERAEGAEAYEDPSVDELRKWLPHATGPEFVVRWRWQPGDLVIWDNRSTMHCATGGLLETLQADRKSS
SHVQPSNGSLLEEGMLRMGFGSQPSPLMSVHANSISLSLMPKDLQRGFGPLQSQLLFAGLELGGAGRILLCGD
VRGDLPKVCKCVDGLQRKLPEEQRFRAVFCVGEFSAEEMNLDVEEKPSVPIYFIDCGPACQDLIDESPQGDEV
APNVHFLGHYGVTSVAGLKVLSRAAQLSREQKLPVLPVYLFDPRQFRETKFGTLKTGAFRALFLLQSVRVLKR
RLRSLGSDLLVKVGKPEDVLPSLLDKKSVLVTQEEVTSEERSVDKALRRELAAKGCEAWEYCWGSTLFHRDDL
PFRQDLSNAPDVFTSFKNQVEPEMAARVNEVPSSFQDKRKDKSHMGVRPCLPDLPPGALPLPEARAGLDFALV
FEPEWEDLPFEKPVEEPEVPEAAAHQFEGGEVAALGRLQYYLFETDLVAKYFETRNGMLGSDYSTKFAPWLAL
GCLSPRKIFEQIREYEKEKVANKSTYWVLFELMWRDFFRFFAAKHGNKIFKLYGVSGGDWEWQSDAKLFQLWA
RGLTGYPLVDANMRELRATGFMSNRGRQNVASFLALDMKIDWRMGADWFESYLVDYDVASNWGNWVHAAGMTT
GRINRFNVIRQSKMYDKDGRYIRHWVPELRSMPVRYIHEPWTLSPDEKLQFGAESYPAPCLDPASFREEGGGK
GKGRKRSKGGRRKIGMLHRWCTTLPVAYLSGRLKAELFEEQDDNYDEDGGQALPQADDLKSKYAAQEGDRGLF
AGAPPALETEPTSSTASRSSSGPSWEELKAKEKKKEFMQTQLFLDGRYTPLAVERLCEEIGDSGGVDILLTSE
WPKGILGGLKDAWPEEAEARRLAKSAVRQCCSPAVAELAVAAEPKYHAVGLGGVFWRRAPWRHERRGEVEAST
GTLKCGVCRMIALGAVDGSRPGVNEAIDAQRKYSYKKPEELEAQAKPQKWLHGLDLDPKSMPTDADDATPSPW
AEKAVKVEASSNPP
>Symbiodinium_mic_OLP98304.1
MCRVTKLKEERISEEDTLKAAEVQRGKEWHEHYVEEHTEDTQVVEQISQAQQALKTGSSAALLQKAPMLRAAE
RRLAASEKQRLVSFLSGERMDPSYVLGILSGMADDTKEEIATDDKNEDTAIHTYDELEDSKKVEISTLLEQFE
RKMKRIGELKVEVVNMKKSMEGAGQSLADDKKMLKELQSTCEAKAAAWEERQATRQKELSALQETVKILDSDE
SLDLFRGKASSLLQLSKGDQDKKDKALELVLKARSEAHPELNFLALALSGRKIDFSKIVKKIDGMVELLKTEG
KDDASKKDYCGKEFHEAELKTKSVASTITSLTATVEQSKGAIAQLATEIKGLQAGLVELDKTVAEAGENRKAE
HEDFQELLASNGQAVQLLDLAKNKLNQFYSPSQFVDTTTKNPFDPYAFLQSSSRKVEAPPATFEGGYQAKMSE
SHGIFSLIAQLKADIEQEMALAKSDEANSQKDYEKTLADAAEKRDADVTLTQEKAKTKADLETDLSDDKSELS
NKKKEATVVEEVVSNLHKECDWLLEHFDLRAQARAEEKENLVQAKTVLSSMCSTGFPLVDAAMRQLWAVGWMP
NYLRHVVAQMLIEYLDLTWKHGLEWFDWTLVDIDVAINSFMWQNGGHSGPDHWEFVLHPVNAAKSCDPEGHYV
RRWLPFLAGCPTEYIHRSIPSLDWRALLHEVLAGMQGSGSFNLRSSWSNPVQLSGGSRAQALLEPSPSSRPAT
TVPADFQTPQEKASPSALGYTPERCPSSILRQAFREMYSEARTSPAQEDPYGSAPPIECLLLGKHWAVAADIA
RGLGEPGVVILGLVHILVYNSYIKVGRSDDMTPQLLRRLTKYRQHSMADEARLAFPSIYVATSGEQASLLEGF

RYMHMSANHPNFRLDLPTDTISEVGREKYDRRALHILVAFMEFGSTRTVDQISAGRTVLRPWEMPLRVARPLG
LPKLEPLVRDLDAARRAHCRQVLEVRRKHPEMVSRTGHEWLRLGGGLLAKLVTRQEFRAETEDFIFYQGPRQK
GSKGGSKGAGLDAGRSVLAEEVQKHESDTFGPGL
>Synchytrium_mic_XP_031023209.1_uncharacte
MSTSSKRAAPVQDSEESQPQKKLKGPVIADGKSILMWFRTDLRTLDNTALFAASQSALKLSLPLVALFIVSPQ
EWKNHLVAPIKVDFCFRALQSLSTTLSSLGIPLLIKEVPTKTDVIQMVKQTCTDFKASELWYNIEYEVNESRR
DAKVQTELATMGINVLPCHDQCVVRPGLVKTKEGKDYTVFTPFKNSWIAYVESKKMLLKLKPAPSVMPSLSED
ISALVADHLKVPSFHPDFRLDDAKAARAKEAFPASEAHAQSRLTNFVNTKITKYGDDRDKTFMDGTSALSPYL
ASGLISARQCLEKAMAANGNKLAGQKGPSTWISEIIWREFYRQVLVAFPRVCMNKSFKPEGHKIVWKDDEEAF
RAWCQGRTGFPIVDAGMRQLNETGWMHNRARMIVASFLAKDLLINWKRGENYFHEHLIDADLASNNGGWQWAA
STGTDSQPYFRIFNPLLQSEKFDPDGKFIRKYVPELASLKGKAIHDPTSALGVKGVEKLGYVKPIVDHKAAKD
AALAAYALAFKKTAV
>Synchytrium_mic_XP_031023438.1
MAKSVTTAIFWFRNDLRLHDNVPLVASLTDQTSTHLLPIYIFDPRYIDLTRIIPSFTSPKTRHYSIPRTSYHR
LKFLIESIDDLKSSLLKHNSSLIIRYGRPEDVIPALVRGLQGDGVKVSGVYCMKEVCSEERDIERRVAERVSV
PLVSRHGGSMVAMEDLPFGVQDLSDVYTVFRKRVESLPQMVRPCIPLPHFKPLPPASPHLTSSPHPSKNLTST
LLPVIHPTPPPVPPHTSHPFVGGETSALSRLNNYLFETHAVSTYKQTRNGLMGDAYSTKFSSFLAHGNISPRK
IYEELKRYEAVYGECEGAYWVVFELLWRDYFRLISLKYTTVLFHPYSLRGTPPPNATTWSKSATSPHAVAFME
GRTGVPFVDAGVREMLQTGYTSNRTRQNLASFLTKDLFIDWRLGAEFFESWLVDYDCSSNYGNWSYAAGIGND
PREERKFNVIKQAWDYDKNGDYVRMWLPELEGVDKAVVHTPWKSVLPASVYPRPIVMAKEWDRHSGRGLEKRG
GDERKKENKPRIKK
>Synchytrium_mic_XP_031024772.1
MSKTIVWFRKSLRVHDNPALLQGSLNCQNLYPIFCFDPHYVFERAIGVRRWIFLLEALRDLDANLRKVNSRLY
VFRGDPIKAMPTIMAELGINHIAYEIDTDTEYAVMRDKKVDEIAKGMGVKVTAVLGHTMYDAKAVAAKNGGKP
PMTYQSFLKAISSMPDPPTPLTMPTRLPPPAPIDMMSESVPLPVHKSELPPTRDTTDIEAQKKLFASLAGPNG
NFDVPTLEELGLVEPPVGERSPHRGGESVALKLLDAFIAQKKRVAKFEKPKTSPAAFYPNADTTVLSPHLKFG
SLSCRLFRTKILQVYREEKSHSQPPTSLLGQLLWREFYYAVAALTPNYSTMANNKACLYIPWRDTSKDATAAA
HLKAWSEGKTGFPWIDAIMRQLAKEGWIHHLARHSVACFLTRGDLWISWERGVEVFDELLLDSDPSLNIGNWL
WLSASQFFHTYYRVYSPIAFGKQYDKEGIFIKRYVPELSKMPEKYIFTPWLAPIDVQRKAGCIIGVDYPKPIV
EHDVASKANMAKMKEAYAKKIYGWTGNEVHVASNPAMGVESKEGGNDGGDSSEESPDDGERDGAEGSDDEFDP
VVRKRRRHMSAVSEQRAAKRPRKTK
>Synechococcus_sp._HDW99197.1
MTVLLWFRTDLRLLDHQPLTRACQQGSPLIPLYCLDPRQFGQTSFGLRPRTGPFRGQFLLESLADLRQQLRSR
GSDLVICQGQPEQVIPTLAREWGVKTVYAHEEVGTEEKEVAAAVERALQPLGIRLQVDWGHTLYHPEDLPFCL
SQLPELFTRFRKQVEESIPIRDPLPIPSIPPLPSGLDPGPLPTLASLGLSPPARDPRARFTYVGGSTAAHARL
QTYFWDRDRLRLYKETRNGMLDPDDSSRLSAWLALGCISARTVYAEVKRYEQERVRNESTYWLVFELLWRDYF
RFILAKHGAKLFRPSGIQGIPIPWQQDWPRFQAWCRGETGYPLVDANMRELAATGFMSNRGRQNVASFLTKNL
GIDWRMGAEWFESLLIDYDVASNYGNWNYTAGVGNDARGFRYFHIPKQSREYDPNGDYLRHWLPELAGIPGDK
IHEPYRLTTAEQKQFGVRLGVDYPRPLVDLEKSLAANRRVYEAALACR
>Synechococcus_sp._MBC8169373.1
MAASRVLFWHRRDLRLADNLGLQAAVEISPAVTGVYVLDPALIHPPESLPPMAPARLWFLVETLRELQQRWSE
AGSRLLVVAGDPVQVLPRLASLLEVPAVVWSRDVEPYARERDRQVAKALQADGRKVLVDWDQLLLAPDLIKTG
GGDPYRVYGPFLRNWRGQVERSAPTTVDAPMALIDLEAETLVAVSGGEGSLGRLCAEGQRQLEQLQVEHGFHG
VDLCPSRPGEAAAAEQLATFADGALLAYEPDRNFPGMPGTSYLSAGLSVGTVSPRQAWCAAQGAKEIARSDEQ
RQAITVWEQELCWREFYQQALFHFPELVDGPYREQWRRFPWENNNDWFDAWQEGQTGMPIIDAAMRQLNQSGW
MHNRCRMIVASFLVKDLICDWRWGERAFMELEVDGDLAANNGGWQWSASSGMDPKPLRIFNPATQASKFDAEG
EYIREWVPELRHVNTKDLISGEIAALERRGYPELLINHKVQQAKFKALYATIRS
>Synechocystis_sp._AGF51454.1_DNA_photol
MYCYDPRQFAQTHQGFAKTGPWRSNFLQQSVQNLAESLQKVGNKLLVTTGLPEQVIPQIAKQINAKTIYYHRE
VTQEELDVERNLVKQLTILGIEAKGYWGSTLCHPEDLPFSIQDLPDLFTKFRKDIEKKKISIRPCFFAPSQLL
PSPNIKLELTAPPPEFFPQINFDHRSVLAFQGGETAGLARLQDYFWHGDRLKDYKETRNGMVGADYSSKFSPW
LALGCLSPRFIYQEVKRYEQERVSNDSTHWLIFELLWRDFFRFVAQKYGNKLFNRGGLLNKNFPWQEDQVRFE
LWRSGQTGYPLVDANMRELNLTGFMSNRGRQNVASFLCKNLGIDWRWGAEWFESCLIDYDVCSNWGNWNYTAG
IGNDARDFRYFNIPKQSQQYDPQGTYLRHWLPELKNLPGDKIHQPWLLSATEQKQWGVQLGVDYPRPCVNFHQ
SVEARRKIEQMGVIA
>Synechocystis_sp._ALJ69418.1_photolyase
MSDQSDHPLILLWHRRDLRLNDHLALAKARQKTAKIVGVFCLDNKILQAEDMAPARVAYLLGCLQSLQDHYQR
LGSELLVFQADPVQLLPKLANTLGAHGVTWTLDTEPYAQKRDLAVAQALRERGLAIATEWDQLMHHPGEVLTQ
AGSPYTVYTPFWKNWSQLPKTSPVPTPKDLQGLTPAEKEKLAPLEPLAIPQLADLGFIWDQPLPLTPGEEAAE
QRLDWFVAHGLEEYQQNRNFPALDGTSQLSAALKFGVISPRTLWQTTLEAWEQSRSEEARASIETWQQELAWR

EFYQHCLYSFPALAQGPYRSPFQEFPWEENQDHFQAWCEGRTGYPIIDAAMAQLNQTGWMHNRCRMIVASFLI
KDLILNWQWGELYFMQTLYDGDLAANNGGWQWSASSGMDPKPLRIFNPHTQAQKFDPEGEYIRTWLPQLARFD
TGDLLTGKLTPGSRRSVNYPEPIVDHNQQQREFKRRYQLVK
>Syngnathus_acus__XP_037099240.1
MVVNTIHWFRKGLRLHDNPSLKESLLGADTVRCIYILDPWFAGSSNVGINRWRFLLQSLEDLDSNLRKLNSRL
FVIRGQPTDVFPRLFKEWNISRLSYEYDSEPFGKERDAAIKKLASEAGVEVTVRISHTLYDLDKIIELNGGQS
PLTYKRFQSLISRMDPVEVPAEFITADVMGKCNTPLSDDHDDKFGVPSLEELGFDTEGLSSAVWPGGESEALT
RLERHLERKAWVANFERPRMNANSLLASPTGLSPYLRFGCLSCRLFYFKLTDLYRKVKKNSSPPLSLYGQLLW
REFFYTAATNNPCFDKMEGNPICVQIPWDRNAEALAKWAEGRTGFPWIDAIMTQLRQEGWIHHLARHAVACFL
TRGDLWISWEEGMKVFEELLLDADWSVNAGSWMWLSCSSFFQQFFHCYCPVVFGRRTDPNGDYIRRYLPVLRG
FPAKYIYDPWNAPEAVQKAARCIVGVHYPKPMVHHAEASRLNIERMKQIYQQLSCYRGLGLLASVPTTSNGNN
KGSSDVMGYEAPHAAAPSGYPAAGQPQADWQNGGVMMYLQADSQACAGAHQQGYVSSSMMCYPQGSRQSPALH
KGMEHHSSNAPMSSKRHCEDSGNSKSSKVQRH
>Syngnathus_acus__XP_037103370.1
MGHTCIHWFRKGLRLHDNPALMAALKDCKELYPVFILDPNLLDKKTVGINRWRFLLGALKDLDFSLRQLNSRL
FVVRGKPEEMFPKLFNKWQITKLSYEYDTEPYSLSRDQKVATLAKEHSVEVLYRVSHTLYDINRIIEENNGKA
SLVYKRMQAIVKTLGPPKKPIPAPTAEDMKGVKTPHSEDDANKYAIPSLDELGHDIAELGEEPFPGGEQEALR
RLEENMQNTGWVCGFEKPQTSPNSLSPSTTVLSPYVTLGCLSARTFWWRLTEVYQGKKHSDPPVSLHGQLLWR
EFFYTASVGVPNFNKMEGNPACIQIDWDTNNDYLTAWREARTGYPFIDAIMTQLRREGWIHHLARHAVACFLT
RGDLWISWEEGQKVFEELLLDGDWALNAGNWQWLSASTFFHQYFRVYSPVAFGRKTDKDGDYIKKYLPILKKF
PTEYIYEPWKAPSSVQKAAGCIVGKDYPLPIVDHKIISKKNLNRMKSAYAKRSVDTEESPSKKEGVKRKAPSV
VEMLNKKAKK
>Syngnathus_acus__XP_037110539.1
MVVNSVHWFRKCLRLHDNPALQEALNGATSVRCVYILDPWFAGASNVGINRWRFLLEALKDLDCSLQKLNSRL
FVVRGQPAEVFPRLFKEWKMTRLTFEYDPEPYGKERDSAIIKMAQEFGVETLVRNSHTLYNLDRIIEMNNNSP
PLTFKRFQTIVSRLELPRRPLPPVNQKQMDKCQTKITDNHDQLYSIPSLEELGFQTKGLPPAVWRGGESEALD
RLNKHLDKKVWVANLEHPRGNMCSLYASPTGLSPYLRFGCLSCRVLYYNLRELYMKLRKRCSPPLSLFGQLLW
REFFYTAATNNPNFDRMDGNPICVQIPWDQNPEALAKWAEGRTGYPWIDAIMTQLREEGWIHHLARHAVACFL
TRGDLWISWESGMKVFEELLLDADWSVNAGSWMWLSCSAFFQQFFHCYCPVGFGKRTNPTGDYIRRYIPILKD
YPNRYIYEPWNAPESVQKAANCVVGVDYPKPMINHAESSRLNIERMKQVYQQLSHYRGLSLLASVPTIQEEAE
PPMTDESQTSSGPDSPAIVHDEAAPNSFTVCTSSHTVLHPELEKDAMRALSHTSANSSVSQMHPSAAITSSTC
ADHSSAVDNPVPLVSTPAHSPLSESKACTPSSLCSTLPPSPSPSSAQTQTSSLGPRRKSFTRKVRRGQRQRGR
HGCHAVAREGERRGDEEETEPGDKNEKMEEGIEMEEEKMEEEAYAGNPR
>Syngnathus_acus__XP_037111271.1
MPQSGEDDGELVVVRKLLREVLLGREDPEGFFAMCLSILGHQETRARFLHVVQPLSSANRSLHATLVSIHEEY
FAKAEDDELELAVTLSLLDAQDHPRTRPQTFDGASNLNGQSKDGGSRTLEKDTCPNLSGTSQTVIAQQGMGRK
DDMDQSEKIKHSKSRRQRRKAAAQHVVGLPRSPSGAAPVLVWVRRDLRLCDNPALVGSLECGAPVIPVFIWSP
EEEEGPGMTVAMGGACKYWLHQALISFRSSLEGIGSHLVFLEATGVGASSLHMLKELVKETGARTVLANALYE
PWLKERDDKVVSTLQKDGLQCKMFHSYCTRDPYSVSTEGVGLRGIGSVSHFMSCCRQNPSGSTSGAPLDPPTS
LPTPSSWPQGITLDTLDLARMPRRKDGTTIDWAENIRKSWDFSEEGAHARLEAFLHDGVYRYDKESGRADAPN
TSTLSPYLHFGQLSPRWLLWDAKGARCRPAKFQRKLAWRDLAYWQLTLFPDLPWESLRPPYKALRWSSNGDHL
KAWQRGRTGYPLVDAAMRQLWLTGWMNNYARHVVASFLIAYLHLPWQEGYRWFQDTLVDADVAIDAMMWQNGG
MCGLDHWNFVMHPVDAAMTCDPYGNYVRKWCPEIAELPDELIHKPWKCPASMLRRAGVVLGQTYPERIVTNLE
ERRSRSLQDVAVVRRQHEEYVDKRSGCDLVPLPPRLVSEALGLAHPTTEKQFLLPVITRMEFKHQMDDPDADG
AANPYNAVLKGYVSRKRDETVAFLNERDFTASVLHEGAQRRERLESDHRRMEGLPPTRFPPRGRARRATNAKD
RFSSLPGGAVLTTHR
>Syngnathus_acus__XP_037131198.1
MRAHFFSFLTKSFTTIMSGKKRKAAGAAASECSAKQLKLASTTEGNKVKASGWLQDLVKQQRDDNKDMKVNKK
RVRFISDTQEAKQDSEGVLYWMLRDQRVQDNWALIHAQHLAMKRNLPLHVCVCLFVPKSELSTLRHYSFMLKG
LEEVEKECKSLDIQFHLLHGSVAKLLSGFVSERGFGAVVTDFSPLREPLKWLDDVKKALPDDVPLIQVDAHNI
VPCWVASPKLEYAARTIRGKITKLLPEFLTDFPVVEKHPHAATKTTKLVDWAKTVASLQVDRTVDEPEWAQPG
TMSGMAMLESFIDVRLKLFGTYRNDPNVAALSQLSPWIRFGHVSAQRVALQVQRGGKDASQSVAPFIEELVVR
RELTDNFCFYNENYDSVKGAYEWAQKSLSDHGKDKRPYLYTREQLEKAKTHDKLWNAAQYQMVSEGKMHGFLR
MYWAKKILEWTSSPEEALSIALYLNDRYELDGQDPNGFVGCMWSICGIHDQGWAERDVFGKIRYMNYKGCLRK
FDVPKFEKKYCPKGL
>Syngnathus_acus__XP_037135355.1
MTTCVCITERDRASTRACPQETTGWVLTVVCVLEPVLVVVPIGGYRRAVMSTSRTIICLLRNDLRIHDNEVFY
FAEKHADHVVPLYCFDPRHYVGTSKYNLPKTGPFRLRFLLESIQDLRKSLVNRGSNLVIRRGKPEEVVADLIQ
QLGSVSAVTFHQEATSEELTVETGVKDVCARMKVQVHTCWGSTLYHRDDLPFPHISRLPDVYTQFRKEVESKS
QVRPELPTPDRLKPLPGGLREGDVPTAADLQQTEPLNDPRSAFPCAGGESQALRRLQHYFWDTDAVASYKETR

NGLVGVDYSTKFAPWLAMGCISPRYIYHQIRQYERERTANQSTYWVIFELLWRDYFKFVALKYGNRLFQVKGL
QDKSIPWKTDMTLFNAWKEGRTGVPFVDANMRELAVTGFMSNRGRQNVASFLTKDLGLDWRMGAEYFEYLLVD
HDVCSNYGNWLYSAGIGNDPRENRKFNMVKQGLDYDSNGDYVRRWLPELHKIRGADVHTPWTLSKAALSHADV
TLGETYPTPVVVAPEWSRHVGKKQNGPYPRGRKGPSHTPKQHGDRGVDFYFSRSKNL
>Syrrhaptes_para__NXT17337.1
RIIELNGGQPPLTYKRFQTLISRMEPLEMPVETITPEVMEKCTTPVSDDHDEKYGVPSLEELGFDTDGLPSAV
WPGGETEALTRLERHLERKAWVANFERPRMNANSLLASPTGLSPYLRFGCLSCRLFYFKLTDLYKKVKKNSSP
PLSLYGQLLWREFFYTAATNNPRFDKMEGNPICVQIPWDKNPEALAKWAEGRTGFPWIDAIMTQLRQEGWIHH
LARHAVACFLTRGDLWISWEEGMKVFEELLLDADWSVNAGSWMWLSCSSFFQQFFHCYCPVGFGRRTDPNGDY
IRRYLPVLRGFPAKYIYDPWNAPESVQKAAKCIIGVNYPKPMVNHAEASRLNIERMKQIYQQLSRYRGLGMLA
TVPSNPNGNGNGGLMGYSPGESVSGCGSTGGHLFLASPGAQLGTGDGHTVVQPCALGDSHTGASGIQQQGYCQ
ASSILHYAHGDNQQSHLLQTGRTALGTGISAGKRPNPEEETQSVGPKVQRQSTN
>Syrrhaptes_para__NXT18533.1
RFLLQSLEDLDNSLRKLNSRLFVVRGQPTDVFPRLFKEWGVTRLTFEYDSEPFGKERDAAIVKLAKEAGVEVV
IENSHTLYDLDRIIELNGHKPPLTYKRFQAIISRMELPKKPVSTVISQQMEMCKVDIQENHDDVYGVPSLEEL
GFPTDGLAPAVWQGGETEALARLDKHLERKAWVANYERPRMNANSLLASPTGLSPYLRFGCLSCRLFYYRLWE
LYKKVKRNSTPPLSLYGQLLWREFFYTAATNNPKFDRMEGNPICIQIPWDRNPEALAKWAEGKTGFPWIDAIM
TQLRQEGWIHHLARHAVACFLTRGDLWISWESGVRVFDELLLDADFSVNAGSWMWLSCSAFFQQFFHCYCPVG
FGRRTDPSGDYVKRYLPKLKGFPSRYIYEPWNAPESVQKAAKCIIGVDYPKPMVNHAETSRLNIERMKQIYQQ
LSRYRGLCLLASVPSCVEDLSGPVADSASGQGCSTSTAVRLTQADQASPKRKHEGAEELCAEELYKRAKVTDL
PAPEIPGKSL
>Syrrhaptes_para__NXT20416.1
KVDYDVCSNYGNWLYSAGIGNDPRDNRKFNMIKQGLDYDGNGDYVRLWVPELQGIKGSDIHAPWALNSVALSQ
AGVTLGETYPQPVVTAPEWSRHINQKPQGRSPHPRGRREPAHPPMQPKVRGIDFYFSRKKDG
>Syrrhaptes_para__NXT21444.1
AMHSSIHWFRKGLRLHDNPALLAAATDCRCLHPLFILDPSSSRAGANAWRFLLDALQDLDGSLREMGSRLFVV
RGCPEEVFPHLFRTWGTTRLTFEVDTEPPARRRDAVVAELAAQHGVEVIQKVSHTLYDTERVLALNNGKAPLT
YKRLQTLLASLGPPEKPAPALTQEHLKGGSSPLPRWGDSGTRPARCVHGEDGQCLTPITLISGLLHLPPIPKQ
CLSPQAWVCSFKKPETAPTSLSPSTTVLSPYLKFGCLSVRTFWWRLDQVYKGRVHSQPPVSLHGQLLWREFFY
TAGASIPNFDRMAGNPVCLQVDWDDNPQHLHAWREGQTGFPFIDAIMTQLRSEGWIHHLARHAVACFLTRGDL
WVSWEEGLKVFEELLLDADWSLNAGNWLWLSGSAFFHRYFRIYSPIAFGKKTDQDGAYIRKYLPVLKDFPTEY
IYEPWKAPRAVQERASCLVGTHYPRPIVEHSTASKRNLGRMK
>Syrrhaptes_para__NXT28676.1
DNWAFLYAQRLALKQELPLHVCFCLVPKFLDATIRHYDFLLRGLQEVAKECAELNISFHLLLGYAKDVLPAFV
VEHGVGGLVTDFCPLRLPRQWVEDVKERLPEDVPFAQVDAHNIVPCWVASPKQEYTAWTIRGKIHAQLPEFLT
EFPPVICHPYPPSCPAEPITWEACYSSLQVDRTVKEVEWATPGTAAGMAVLQSFIAERLKYFGSHRNDPNKAA
LSNLSPWFHFGQVSTQRAILEVQKHRHKYKESVDTFVEEAVVRRELAENFCYYNEHYDSVQGAYSWAQTTLKL
HAKDKRPFLYTLEELEQGTTHDPLWNAAQLQMVREGKMHGFLRMYWAKKILEWTHSPEEALRFAIYLNDRYEL
DGRDPNGYGSADTLSLCPAGCLWSICGIHDHGWTERAVFGKIRYMNYAGCKRKFNVGQFEQRYAP
>Tachyglossus_aculeatus_XP_038612536.1_cryptochro
MAGNAVHWFRKGLRLHDNPALRDCIRAAHTVRCVYILDPWFAGSSNVGINRWRFLLQCLEDLDANLRKLNSRL
FVIRGQPADVFPRLFKEWNIARLSIEYDSEPFGKERDAAIKKLASEAGVEVIVRISHTLYELDKIIELNGGQP
PLTYKRFQTLISRMDPLAMPVETITAEVMGKCVTPLSDDHDEKYGVPSLEELGFDTDGLPSAVWPGGETEALT
RLERHLERKAWVANFERPRMNANSLLASPTGLSPYLRFGCLSCRLFYFKLTDLYKKVKKNSSPPLSLYGQLLW
REFFYTAATSNPRFDKMEGNPICVQIPWDRNPEALAKWAEGRTGFPWIDAIMTQLRQEGWIHHLARHAVACFL
TRGDLWISWEEGMKVFEELLLDADWSVNAGSWMWLSCSSFFQQFFHCYCPVGFGRRTDPNGDYIRRYLPVLRG
FPAKYIYDPWNAPEGIQKAAKCIIGVNYPKPMVNHAEASRLNIERMKQIYQQLSRYRGLGLLASVPSNPNANG
SGGLMAYSPGENIPGCSSGGGAQIGASESHLLQTCVLGESHLGPSGIQQQGRSSLTPGLSGGKRHCQEEESQS
IGPKVQRQSTD
>Tachyglossus_acu_XP_038620369.1
MAAGPGCGASSVHWFRKGLRLHDNPALQAALRGARSVRCVYILDPWFAASSSVGINRWRFLLQSLEDLDTSLR
KLNSRLFVVRGQPTDVFPRLFKEWGVTRLTFEYDSEPFGKERDAAIVKMAKEAGVEVVTENSHTLYDLDRIIE
LNGQKPPLTYKRFQAIISRMELPKKPVSCVTSQQMEGCKAEIQDNHDDTYSVPSLEELGFPTDGLGPAVWQGG
ETEALARLDKHLERKAWVANYERPRMNANSLLASPTGLSPYLRFGCLSCRLFYYRLWDLYKKVKRNSTPPLSL
YGQLLWREFFYTAATNNPRFDRMEGNPICIQIPWDRNPEALAKWAEGKTGFPWIDAIMTQLRQEGWIHHLARH
AVACFLTRGDLWVSWESGVRVFDELLLDADFSVNAGSWMWLSCSAFFQQFFHCYCPVGFGRRTDPSGDFIRRY
LPKLKAFPSRYIYEPWNAPESVQKAAKCVIGVDYPRPIVNHAETSRLNIERMKQIYQQLSRYRGLCLLASVPS
CMEDLSSAVAESGLGQASGSSSSTAPRPLPLPYGPTSPKRKLEAAEELPGEELCKRPKVSGRPGPELPSKDA
>Taenia_asiatica_VDK23649.1

MATQVASGLSSGGLEDVILSISGHISAQRCLWEVKLLYSEYKESVDAFMEEAFVRRELADNFCYYNPNYDSIK
GAWSWARETLRMHANDLRKPSYSEEAMESATTGDDLWNAAQRQLLREGKMHGFLRMYWAKKILEWHAEGPEKA
LQLGFHLHNRYSLDGTDPNAYVGKSASLNPYRFTFLLYFSHNSGRRRS
>Takifugu_rubr__BBA21724.1
MHSVLTSIYKEYFSTTEDDELELALALSLVETKDYELSAPVHEPQLKRPGGRRNESGSVWPTSEFQTQRRSPI
QPAEAASGTRNNSPQTGPSPKVCSPQTAVEPEHNADAWTPAKARSASCESGHQVMEVGDVDQSEKPKRPKKRR
QRRKCAAQQVVGLPRAPSAAAPVLLWFRRDLRLSDNPALVSALKVGAPVIPIFIWSPEEEEGPGVTVAMGGAC
KYWLHQALSCLCSSLENIGSHLVFLKSETRGSEGGASLRALQGLAKATGARTVVANALYEPWLKGRDDAVAAA
LQKSGVECEMFHSYCLRDPYSVSTEGVGLRGIGSVSHFMSCCRQNPGAALGAPLDPPTTLPVPAHWPPGVSLE
TLGLARMPKRKDGTTIDWAANIRKSWDFSEGGAHARLEAFLHDGVYRYEKESGRADAPNTSCVSPYLHFGQLS
PRWLLWDAKGARCRPPKFQRKLAWRDLAYWQLTLFPDLPWESLRPPYKALRWSTDRRHLEAWQRGRTGYPLVD
AAMRQLWLTGWMNNYMRHVVASFLIAYLHLPWQEGYRWFQDTLVDADVAIDAMMWQNGGMCGLDHWNFVMHPV
DAAMTCDPCGQYVRKWCPELSGLPDELIHKPWRCPTSLLRRAGVVFGQTYPERIVTDLEERRSQSLQDVALVR
KEFGQYVDKRSGCDLVPLPPRLVSEALGSSHLDGGVATGGKQFLLPVITRMEFKHQQEDPDADAASNPYNAVL
KGYVSRKRDETIAFLNERDFTASVMHEGAQRRERMESDQRRLEGLPRGPAPRGRVRRTPTAKDRFSVVPGGAV
ASLR
>Takifugu_rubr__XP_003968037.1
MSGPRTVICLLRNDLRLFDNELFHWAQRNADHIVPLYCFDPRHYMGTYHYNLPKTGPFRLRFLLESIKDLRNT
LLNKGSNLIVRRGKPEEVVASLIKQLGSVSTVAFHEEVTSEELDVEKRVKDVCAQMKVNVHTCWGSTLYHRDD
LPFHHISRLPDVYTQFRKAVESQCRVRPVFPPPEHLKPLPQGLEEGTILTAEDLEQKEPVADPRSAFPCSGGE
SQALARLKHYFWDTDAVAVYKETRNGLIGVDYSTKFSPWLALGCISPRYIYHQIKQYESERTANQSTYWVIFE
LLWRDYFRFVAVKYGTKLFQVNGLQDKSVSWRKDMKLFNAWKEGKTGVPFVDANMRELATTGFMSNRGRQNVA
SFLTKDLGLDWRMGAEWFEYLLVDHDVCSNYGNWLYSAGIGNDPRENRKFNMIKQGLDYDNNGEYVRLWVPEL
QRIMGADVHTPWTLSSAILSHAHLSLGETYPTPIIVAPEWSRHFNKKMSGAGPSPRGRKGPSHTPKQHRDRGI
DFYFSRSKNL
>Takifugu_rubr__XP_003975844.2
MFHRILSCSPRSPLQFVAGCYSPICGSTMSDRKRKAKSAAREPAAKQQKLASTKEVKEAKAGGWLQGAIQQQR
TENKEMSFNEKRLRFISDAKKIKQGSEGVLYWMLRDHRVQDNWALIHAQQLAVKEKLPLHVCVCLHVPKSELS
TLRHYSFMLKGLEEVEKECKALDVQFHLLHGSVGEVLPGFVSERHLGAVVTDFSPLREPLKWLDDLKKEFPQD
IPLIQVDAHNIVPCWVASPKLEYAARTIRGKINKHLSEFLTDFPPIEKHPHTAEQAAEPVDWEETLSSLSIDR
TVGETEWAKPGAGAGLAMLESFIDERLKLFDSQRNDPNAAALSQMSPWIRFGHVSAQRVALQVQRSGKKAGVA
VSSFIEELVVRRELTDNFCFYNENYDSVEGAHEWARKTLKDHAKDKRPYIYTRRQLEEAQTHDHLWNAAQYQM
VTEGKMHGFLRMYWAKKILEWTSSPEEALSTSLYLNDRFELDGQDPNGFVGCMWSICGVHDQGWAERPVFGKI
RYMNYKGCLRKFDVGRFEKKYSPKASS
>Takifugu_rubr__XP_011607950.1
MTHTCIHWFRKGLRLHDNPGLMAALRDCKELYPVFILDPQLHNKSVGVNRCRFLIGALKDLDLSLRQLNTRLF
VVRGKPEEVFPKLFCQWKITKLTYEYDTEPLSLSRDKTVTRLAEEHGIDVVCKVSHTLFDINRIIEENNGKTP
LTYKSMQAIVKKLGPPKRPLSAPSMEDLKDVNTPCSESHEKKYRIPTLEDFGHNLADLPEEQFPGGEQEALRR
LEEHMKRTAWVCNFEKPKTSPNSLSPSTTVLSPYVTFGCLSVRTFWWRLSDVYEGKKHSAPPVSLHGQLLWRE
FFYTASVGISNFNKMVDNPVCTQVDWDINSEYLAAWREARTGFPFIDAVMTQLRQQGWIHHLARHAVACFLTR
GDLWISWEEGQKVFEELLLDGDWALNAGNWQWLSASAFFHQFFRVYSPVAFGKKTDKNGDYIKKFLPHLKKFP
AEYIFEPWKAPQSVQQAAGCIVGKDYPHPIVQHEVVSKKNIQRMKAAYAKRSANTAKSLSKIQGLKRKPSSSV
DMLKKKKKNNTSEPLL
>Takifugu_rubr__XP_011611545.2
MVINTIHWFRKGLRLHDNPSLKDSLLGADSVRCVYILDPWFAGSSNVGINRWRFLLQSLEDLDSSLRKLNSRL
FVIRGQPTDVFPRLFKEWNISRLSYEYDSEPFGKERDAAIKKLASEAGVEVTVCISHTLYDLDKIIELNGGQS
PLTYKRFQTLISRMDPVEVPAESITAEIMGKCTTPLSDDHDDKYGVPSLEELGFDTEGLSSAVWPGGETEALT
RLERHLERKAWVANFERPRMNANSLLASPTGLSPYLRFGCLSCRLFYFKLTDLYRKVKKNSSPPLSLYGQLLW
REFFYTAATNNPCFDKMENNPICVQIPWDRNPEALAKWAEGRTGFPWIDAIMTQLRQEGWIHHLARHAVACFL
TRGDLWIGWEEGMKVFEELLLDADWSVNAGSWMWLSCSSFFQQFFHCYCPVGFGRRTDPNGDYIRRYLPILRG
FPAKYIYDPWNAPESVQKAAKCMIGVHYPKPMVNHAEASRLNIERMKQIYQQLSCYRGLGLLATVPSNSNGNR
NSNNETCFPVEASHDPSAPSGYQVPVHTQGDWQSGVMMYMQDPQSGMGAHQQGYAGTSSGVMCYTQNTQQIPG
PSTQKGLERHGGIQTTGKRHNEDSGNGKSSKVQRQSNH
>Takifugu_rubr__XP_011615916.2
MVVNSVHWFRKGLRLHDNPALQEALNGADSLRCIYILDPWFAGAANVGINRWRFLLEALEDLDCSLRKLSSRL
LVVRGQPTDVFPRLLKDWKVTRLTFEFDPEPYGKERDGAIIKLAQQFGVETIVRNSHTLYNLDRIIEVNNNSP
PLTFKRFQTIVSRLELPRRPLPTVTQHQIHKCGAKMADXQEQLYSIPSLEELGFRTEGLPPAVWRGGESEALE
RLHKHLDKKVWVANLEHSRVSTCSLYASPAGLSPYLRFGCLSCRVLYYNLRELYVKLRKGCSPPPSLFGQLLW
REFFYTAATNNPNFDRMEGNPICVQIPWDQNPEALAKWAEGHTGFPWIDAIMTQLRQEGWIHHQARRAVACFL
TRGDLWISWECGMKVFEELLLDADWSVNAGSWMWLSCSAFFQQFFKCYCPVGFGRRTDPSGDYIRPLHPHPED

YPNRYIYEPWNAPEAVQKAANCVVGVDYPRPMINHAEGSRLNIERMKQVYQQLSHYRGLSLLASVPTIQEEAE
PPMTDEFQNSSGPDSPPRPAHGAALPDSSTVCVSSSCVPHSVLGDTTPTRSSLTQSLSARSKAGVSVLRSVHR
PGLLRHPDLVSGTPEERPRPQGPPPPPSPGPARRHQGRRQEVREGGEDGGRRRAGRRSDGGRGGGQQVEDTGG
TFRYAREDRGRRVRTGLLDQDKDPCAGVSS
>Talitrus_sal_AFV96168.1_cry_2
MSPNKIASPGERSIHRTILGDNNLTFSESGSSSGLAFTAEKKNTSKVVSGKHVVHWFRRGLRLHDNPALRDAI
VNCETFRCIYILDPWFAGSSNVGVNKWRFLLQCLEDVDNSLRNLNSRLFVVRGQPANALPQLFKEWNTTVLSF
EEDPEPFGRARDASIIGIAQEMGIEVIVRTSHTLYELDKIIKKKGGKPPLTYKTFQNILAMMDPPPPPVAPIE
ASDLKHAYTPLQHDHDDKYGVPNLEHLGFETEHLPPAVWKGGETEALSRLKHHLERKAWVASFGRPKMTPQSL
FACPTGLSPYLRFGCLSARKFYTELNVLYTKIKKVPAPVSLHGHLLWREFFYTAATNNPKFDHMKGNPICVQI
PWDKNPEALAKWAHGQTGFPWIDAIMTQLRTEGWIHNVARHAVACFLTRGNLWVSWEEGMKVFDELLLDADWS
VNAGSWMWLSCSSFFQQFFHCYCPVRYGRKADPNGDFIRTYLPVLKNFPTKYIHEPWMAPESVQRNARCIIGQ
HYPLPMVDHGTQSQNNIERMKQVYQQLAHYRANISTRPCGDSKLRCKYPLHSTA
>Taphrina_def_CCG80991.1_putative_p
MSKRKCPPWQSAPSLKRAREIDDESPYEVLGEALSTNVQPDKTRNVLHWFRSKDIRLEDNRALHAASTKAEEG
DSALITLYVHSPADLDWHGTSPARTDLILRTLEQMQSGLKRLNIPLYIHETKSRDDLVPDVLKFIKDHDISHV
YANFEYEIDELRRDIRIAKGLKEEKVSFEIVHDQSAINPGTLTTGNGGPHKVFTPYHKVWLAEMSKNGKSILD
LLPLPSANADAHVGELEDAGLFKTQIPKQPESKDFKSDEERDRIRGLWPAGHEAAKERLIDFLDNKVNLYGDT
RSNPGKDSTSRMSAYFSSGTISIREALAHCRDRNKGSCDFSGSKGRGMSAWVREIVFREYYRQLIFVVPHNSL
NLPQNLKMFTVKWEDNEEAEENWKKWCEGTTGFPLVDAGMRQLNTEAWMHNRARMNTASVLRTNMLVDYRRGE
RYFAEHLVDYDLSNNFQGWQPSFTVFNPTTQGENNDVDGDYIRKWVPELKHIKGKAVFAPYERLDPQAFKKTG
YPKPCVDWKQSKARCMDAYKQATIDGGEALRD
>Taphrina_def_CCG81760.1_putative_p
MAARVAWWIRTDLRLHDNVALAAALELKPECLYPIWTWDPHYVFHTRVSPNRWRFLLDCQEDLSNSIKEKTKH
NGLLVLRGNPLTCIKQVLSDWKITHLVFEKDTDTYARIRDAAVIEMAKNLGVKVITKLGRTLFDPEAMTKLNG
GKAIYNISSIQKLGPQTGEIPRPLDTPKSLPRAGDTKINTRRDDGIEFRPDINAPQRLGSTDDVTCYERMDGP
DGSFSVPTMEEINMKAAPGPHRGGETIALQNLAEYMADKQKTATFEKPKTNPGVFLPPETTLLSPHLHFGSLS
IRKFYWDVMDVVKEYGPKSSKPPVSLEGQLYFRDMYFAAQYGTENYEQTQGNPHCRFIPWKLANTYDEHGKVN
GYDKSDAKAEEYFQRWRQGQTGFPWIDAIMRQLARDGWIHHLARHSVACFLTRGHCYISWERGADVFEELLID
HETACNVGNWQWLSCTAFFSQFYRVYSPVAFGKKNDKSGALIREYVPELKDVPEKYIYEPWKMSKTDLKAAGV
VLGGSGPGSYPKPMLDVQEEAKVALAGMKASYGAKIYGDDERVKNGAAEKIIKGENSSPKKRKGEQGLMDKFT
KKPREEPKEEHEDNEQDN
>Taphrina_def_CCG81785.1_Putative_c
MGKTLLYYHKHDLRLHDNPVLQKLRDEGPYDHFLPVYVFPPHQMELSPLLKSSVTKNPHKGGDVRGEFGAWRT
GNMRVQYLCETIWDLKQNYKKLGSDLVLKAGKAEDVLPTLLKALKAEGQDEIDLWLQDEYTTEEIDEFRKIRE
ATKDLATFKKVEDNTLVSIHDLPYDPHTKLPNFYTEFRKSVEPLHHVRECFKTPESLPPRPAFKDFENDYDVP
EDMNECITKLCKPLGGVPTFPGNTAHPMKGGETAALKRLDHYVSGGKQAPAAHYKETRNQMLGADFSTKFSSF
LSLGAISPKLIHHRITRFEEEHRLTGDVNTYWIRFELLWRDYFKFVALKHGKHLFLRTGLKQKPIHDTWLGAD
STEARRWKEGTTGVGIVDAAMRELNQTGYMSNRTRQIVASFLTKDLYVDWRIGAEHFEEKLLDHDAASNWGNW
QYQAGVGNDPRGSRRFNPAKQTHDYDAEGEYIKTWCPEVRDVREKMGFTYFWTINEKTLHQLNLSDLPGVKSP
IKGLCADERTRVNGKGGKGFRGGKARRDAGRPRRTGPSDKFAQQNDGDY
>Terrapene_carolina_triunguis_XP_024065414.2_cryptochro
MKHISIHWFRKGLRLHDNPALLAAMTDCHKLYPIFILDPWFPKNMRVSVNRWRFLVESLRDLDESLQKLNSRL
FVVRGRPTEIFPVLFKEWKVTRLTFEIDTEPYSRQRDSEVASLAAEHGVQVIQKVSNTLYDTDRVIAENNGKV
PLTYVRLQTLLAALGPPKRPVPAPTLENLKDCCTPWKDNHDAEYGIPTLEELGQDPEQAGPRLYPGGESEALC
RLDLHMNRTAWVCNFQKPQTEPNSLNPSTTVLSPYIKFGCLSVRTFWWRLAEVYQGRKHSNPPVSLHGQLLWR
EFFYTAGAGIPNFNKMEGNPVCVQVDWDNNPEHLRAWSEGRTGYPFIDAIMAQLRTEGWIHHLARHAVACFLT
RGDLWISWEQGQKVFEELLLDADWSLNAGNWQWLSASAFFHQFFRVYSPIAFGKKTDKGGEYIKKYLPFLRKF
PAEYIYEPWKAPRSVQEQAGCVIGRDYPKPIVVHEVVSKRNVERMKAAYARGSASTTAQLEGGGGKKGAKRKT
PAGPSVAELLTKKPKTKSS
>Terrapene_carolina_triunguis_XP_024070429.1_cryptochro
MAAAGSTPLGLARSVHWFRRGLRLHDNPALQAALRDASSVRCIYILDPWFAASSAVGINRWRFLLQSLEDLDN
SLRKLGSCLFVVRGQPTDVFPRLFKEWGVTRLTFEYDSEPFGKERDAAIVKLAKEAGVEVVIENSHTLYDLDR
IIELNGHKPPLTYKRFQTIISRMEHPKKPVSSITSQQMEKCKSEIQENHDDTYGVPSLEELGFPTDGLAPAVW
KGGETEALARLDKHLERKAWVANYERPRMNANSLLASPTGLSPYLRFGCLSCRLFYYRLWELYKKVKRNNTPP
LSLYGQLLWREFFYTAATNNPKFDRMEGNPICIQIPWDRNPEALAKWAEGKTGFPWIDAIMTQLKQEGWIHHL
ARHAVACFLTRGDLWISWESGVRVFDELLLDADFSVNAGSWMWLSCSAFFQQFFHCYCPVGFGRRTDPSGDYV
RRYLPKLKGFPSRYIYEPWNAPESVQKAAKCIIGVDYPKPMVNHAETSRLNIERMKQIYQQLSRYRGLCLLAS
VPSCVEDLSIPVTDPMSGQGSSTSTVQRLLHCGQTSPKRKHEGVEELCTEELYKRAKVTGLHISEIPSKNL
>Terrapene_carol_XP_026505909.1

MSQKSRNRQARGGEEISGDAARPGAAVAPRGQLKGKRKVLAEPSGAEVLTVEKRRKEGEAAEGGGAGSLGEAV
RQARLQVAPSVQDFKYNKKRVRLVSQGSDLKDAAQGVVYWMSRDQRVQDNWAFLYAQRLALKQQLPLHVCFCL
VPKFLEATIRHFGFMLKGLQEVAEECQELDIPFHLLIGFAKDVLPAFVTGRGLGGVVTDFSPLRVPMQWVEDV
KERLPGDVPFVQVDAHNIVPCWVASDKQEYGARTIRRKIHDQLSEFLTEFPPVIKHPYPSTAPAEPIDWNACR
ASLQVDCSVKEVTWATPGTAAGLAVLESFIGERLKSFGTDRNNPNRAALSNLSPWFHFGQISVQRAILEVQKY
RSKYKESVDGFIEEAVIRRELADNFCYYNRNYDKVEGAYDWAKTSLKLHAQDKRSHLYELKQLEEGKTHDPLW
NAAQLQMVHEGKMHGFLRMYWAKKILEWTRSPEEALEFTIYLNDRYELDGRDPSGYVGCMWSICGIHDQGWAE
RAVFGKIRYMNYAGCKRKFDVGQFERRYDPRKLGQ
>Terrapene_carol_XP_026507960.1
MSGAAPRTVICLLRSDLRYHDSEVLRWAQNNADYVIPLYCFDPRQYLRTYYYGFPKTGPHRLKFLLESVKDLR
QTLKKKGSNLVVRKGNPEDVVHDLITQLGSVAAVAFHEEVKERFELCCLSIKLARPFSARQYWTCLAYHFICP
PFYYICQRLPDVYTQFRKAVEAQTKVRPTVQMPDQLKPLAPGVEDGCIPVPEEFGQKDPLTDPRTAFPCSGGE
TQALMRLQHYFWDTNLVASYKETRNGLIGIDYSTKFAPWLALGCISPRYIYEQIRKYEKERTANQSTYWVIFE
LLWRDYFRFVALKYGTRIFLLKGLQDKEVPWKKDPKLFDAWKEGRTGVPFVDANMRELAATGFMSNRGLQNVA
SFLTKDLGLDWRMGAEWFEYLLVDYDVCSNYGNWLYSAGIGNDPRENRKFNMIKQGLDYDSNGDYVRLWVPEI
QCVKGGEIHTLWALSNAALSQAGIALGETYPLPVVLAREWSRHINQKPNKRDHPPKGRKDPSDTPRQHKDRGV
DFYFSRNKDLS
>Terrapene_car_XP_024068729.2_cry-1
MGVNAVHWFRKGLRLHDNPALRECIQGADSVRCVYILDPWFAGSSNVGINRWRFLLQCLEDLDANLRKLNSRL
FVIRGQPADVFPRLFKEWNIAKLSIEYDSEPFGKERDAAIKKLASEAGVEVIVRISHTLYDLDKIIELNGGQP
PLTYKRFQTLISRMEPLEMPVETITAEVMEKCTTPVSDDHDEKYGVPSLEELGFDTDGLPSAVWPGGETEALT
RLERHLERKAWVANFERPRMNANSLLASPTGLSPYLRFGCLSCRLFYFKLTDLYKKVKKNSSPPLSLYGQLLW
REFFYTAATNNPRFDKMEGNPICVQIPWDRNPEALAKWAEGRTGFPWIDAIMTQLRQEGWIHHLARHAVACFL
TRGDLWISWEEGMKVFEELLLDADWSVNAGSWMWLSCSSFFQQFFHCYCPVSFGRRTDPNGDYIRRYLPILRG
FPAKYIYDPWNAPESIQKAAKCVVGVHYPKPMVNHAEASRLNIERMKQIYQQLSRYRGLGLLATVPSNPNGNG
GLMGYSPGENISGCSSASGAQMGSNDGHTVGVQTCSLEDSHAGSSGIQQHGYSQGSSIVHYAQGDHQQSHLLQ
QGRTGSTGISTGKRPNPEKETQSIGPKVQRQSTN
>Terrapene_car_XP_024073555.1_cry-1-like
MPNMPHWTIHLFRKGLRLHDNPTLLAALESSEVVYPVYILDRKFLMSAMNMGTLRWHFLLQSLEDLQRNLTKL
GSCLLVIQGEYESVLRDHVKKWNITQVTLDAEIEPFYKEMERTIQVLGGEMGFDVLSLMGHTLYDIRRILDMN
GGTPPLTYKRFLHILSVLGDPDMPVRNLTAEDFQRCKPPPELCLAECYRVPLPEDLEIPRESQSPWRGGETEG
LQRLEQHLKNQGWIANFTKPKTIPNSLLPSTTGLSPYFSLGCLSVRSFFYRLSNIYAQSKNHSLPPVSLQGQL
LWREFFYTVASATPNFTKMAGNPICLQINWYQDAERLHKWRMAQTGFPWIDAIMTQLHQEGWIHHLARHAVAC
FLTRGDLWISWEEGMKVFEEMLLDADYSINAGNWMWLSASAFFHQYTRIFCPVRFGKRTDPEGQYIRKYLPVL
TNFPSKYIYEPWTAPEEAQKQAGCIIGQDYPLPMINHKEASDRNLQLMKQVREEQYGTAQLTRDDMDDPMEMG
LKRERSEENSKKGKRARNREQT
>Tetrabaena_soc_PNH00586.1_cry_DASH,_
MSNRGRQNVASYLALDLGVDWRRGADWFESCLLDYDVTSNWGNWAAAAGLTGGRVNHFNIAKQAGRGGEGELK
EERSREGGQGLNYSKDYDLAGEYVKTWCPELRNVPAARVHEPWLMSKEEQERSGCRIGVDYPAPIPASKHGRP
HAGAGGGGGGGGRPASGRPASDRPASGRPGPRSGGGGGGGGARGAKRSEFERYN
>Tetrabaena_soc_PNH04446.1_photo-lyas
MIRFLFQSLFRKMEKVVNEVEKEDEDEPGSALLAAVKSCRESSVRWLVTAPATRTLPLGVVIRIELAATSDRI
KELIRSHVTAPRHKEYRRNVLMLISSSAAGNEEEIERLLALPHFSLADIDVDSVSDALSAADRNGHVAIARRI
SKGEFERYEATRDELFAETTRLSVYLKFGCVGVREVFRAVVQAHGVGHALIRQLFWRAFYDQVAYHFPGVLAG
QVTPGKSNESLNRKYDEIRWVSSQPLFDAWCAGRTGFPIVDAGMRQLLATGYMHNRVRMVSANFLVKVLHIDW
RLGERFFAQNLVDYDPSSNSGGWQWASGGGADSQQYYRVFSPWLQAEHHDPGCVYIKRHIPELSTVPVADILA
WHSAHVKHSTPYPPPIVADYRVEAKRSMAMYKKNLN
>Tetrabaena_soc_PNH07791.1_cry-1
MPEFGTVIVWFRRDLRVDDNPALVAALNAAENVIPVFIWAPEEEGQFQPGRCSRWWTKHSLIDLQQALAALGS
KLVIRRSTDSTAALLQIVSEVGAEAVFFNHLYDPISLMRDHDCKRGLTAAGVAQRTFNGDMLYEPWEVLDDNK
QPLSTFSDFWARGGGGEELSGGEGAGEGGGGGGERRRKRESGDGEDGDEDSPGGGHTPQGGGGDEDMASADLD
SRSGSEDEGGRGAWGEAPGGGGAVVMEEAREAEMEGGAEAGGGEGQSHKRRRSFGAGPLA
>Tetrabaena_soc_PNH10276.1_(6-4)DNA_p
MASASASAGAKVSIVWFRKGLRVHDNPALIEACRDAAHVYPIFQSSYKVTVNRYNFLLESLRDLDNSFRARGS
RLLVLRGKPEEVLPRVFREWGAKQLCFEVDTEPYAKARDARVHQLAAEAGVEVHAAVSHTLYDTEMLVARHGG
SPPLTMQSFTKLVDRVGDPPPPAPDPPAAIPPPAPGMPAADPAATGVPTWQEVGFTEAPVTIFKGGETEALRR
LDASFADPKWVAGFQKPDTNPAAFERPATTVLSPYLKFGCLSARLFHARLLAVYREHKAHTQPPVSLRGQLLW
REFFYTVGAHTPNFDRMVGNPICKQIPWDTNEEFYNAWREARTGEQLPAPDSGAGRTCKAREVVDHYINAANW
MWLSASAFFSQYFRVYSPVTFGKKVYDPSGAFIRRFLPVLKDMPAKYIYEPWTAPLEVQRRAGCVVGVDYPRP
ILDHAVVSKRNTARMAEAYRANKCVAGPGGVVDELA

>Tetrahymena_the_XP_001015321.1_DNA_photol
MDILNSKQFSENQKKQKVNLFWFRRDLRLFDNVGLYHALKESIPVVPLFIFDKDILNELEDKKDCRVEFIHNY
LSQMQDNLRKHGSTMVVKHSNAESAFKSLMEEFDISCVFTNRDYEPYAKKRDQQIKEILNSKGIGFKDFKDQV
IFEAKEVHQDDGSAFKVYTPFSKKWISLLSEPDYQSYKSESLMNNYWKSQVEVPFPTLEQIGFQKTGIEFPPS
QLDEELVKNYDKTRDYPHIKTSLMSVHLRFGTVSIRELVRKAVLLNQKYLDELCWREFFMMMIDQFPLFATEN
FKKAYNKVEWRNNPEEFKAWCEGKTGYPLLDAGMRQLNKTGYLPNKLRMIAASFLTKNLLIDWRWGERYFAQK
LIDYDLAANVGGWQWVNGSGTDQAPYIRVLDPVMQQKKYDPDFKYVKTWVEDFNEDTYPKRIVNYEESRQRAL
DAFSIVFKKNQIKQHQQQIKEKKANYQHYYKNKK
>Tetranychus_urt_XP_015785840.1_cry-1-like
MSKAFDNSPHGNKLELSMNDNGENDSKLVNSPQTTANEFTLLNNRTDCDLIDDDSIEPCIQSFSTSPQASIKF
NAISNNTPASHKTVDCARDLASPDVNSTQCFQSPNQSSSKTPVQLKSEQSNSVLNNYVFKSNHNKDNNHSDSV
YNDNNGNETRGPAKHLVHWFRKGLRLHDQPALLKGLQGCDTFRCIYLIDPWFSTESNCGINKWRFLLQSLEDL
DSNLHKLGSRLFVIRGQPAEVFPLLFKEWMITHLSFEEDPEPFGKIRDAKIAKMCKEMNIEVICETSHTLYRL
EDIINHCSGEIPLTYLQFQNVISELGKPEEPAQAITHEVIGKAYTPVPRDHAKKYTVPTLNELGFTTQPNHRS
VWVGGETEALQRLSRHLEHKAWVATFGKPKLTSLSLLCASQTGLGPYLRFGCISPRLLYHQLSNLYSKLKKGN
PPLTLCGQLLWREFFYCVATNNPNFDKMQNNPLCVQIAWNSNAEALNKWANGQTGYPWIDAIQTQLRREGWIH
QIAKHATVCFLTRGILWISWEEGMKVFDELLIDADWSVNAGTWMWLSCSSFFQRICHIYCPVSFGRKMDPQGD
YIRRYLPVLRNIPNEYIHEPWKAPIKVQREANCKIGKHYPQPIVDHQEAMRINQERMKQVFRILIKGLDLKRE
DVQD
>Tetranychus_urt_XP_015787507.1_photo-lyas
MGPKKRQLSPTPPKKKDKTDSEDSSEEAGTTSASLDEVKSEQTADPSERLFQEINKARAAVGKSIEDFKFNKK
RVRMLSDCKVASEDCGAILYWMSRDGRVEDNWALLYAQRLALKFKVPLHVCYCLVPVFLDASIRGFHFLLGGL
EEVEKDLLSRNIHFHLLLGAPPKVLPKFVSDNNIGGVVCDFAPLKVPMRWVNEVCKKLPEDVPLCQVDAHNIV
PCWVASEKLEYAARTIRPKINNKLPEFLTHFPPVVLHPYSSDVKYEKVDWVRAEKSLECDRSVPDVNWIKPGY
RAGIKMLSDFVEKKIRWYNDKRNDPSQDVQSNLSPYFHFGQIAPQRAVLVVQKYKKQYKESVEAWVEEAVVRR
ELADNFCFYNPNYDSLKGAPEWAQKTLNDHRKDKRPYVYTKKEFEDGKTHDPLWNACQMQLVRDAKLHGYMRM
YWAKKILEWSKSPEEALEIAIYLNDKFELDGRDPNGFVGCLWSVGGIHDQGWGERAIFGKIRYMNYEGCKRKF
DINTFMAKYKTISSKINPWTASKKTKK
>Tetranychus_urt_XP_025017376.1_cry-1_X1
MSSKPTFNCIHWFRHGLRLSDMPSLYQAAKVCQKIYPIFIFDGFTAGLKQAGFNRTRYLLDCLADLDTQFKSF
GGRLICFHGNCVQILESLIKKWSITHISFEQDPEPIWKERDDLVKQLANSLGVEIIEKISHTLWDPEKVIDMN
GGEAPSNYEMFCHTVNVIGQPDRPLPKPDFTQLKTIFPLDDYCEDTAGVPSSPLVLGVKMESEKQINQVWFGG
ETISMELLADRLSLEKSAFKVGDYLPTQSTPDITITDKSLSPALRHGCLSVRKFYWDLHDLYESIFGNSVKPR
IVGQLIWREFFYTMSVNNPCYGQMANNPVCLQIKWKQDSAALEAWSTGKTGYPFIDAAMRQLIFEGWIHHAVR
NAVACFLTRGDLWLSWEHGLKVFLKYLIDADWSVCAGNWMWVSSSAFDTILQSDACISPIMYGRWLEPTGNYI
RKYVPELTKMPTKYIFNPSSAPIEVQKEANCIIGVDYPEPIINHDVAFKTNQKLMESVKNVFLDHRYQSMSNF
KHIPNHVAPSNIEETKAFLCLPEDFFNNLVI
>Tetraodon_nigr__CAF92156.1
MKDCELSAPSQERQLRQPGGGQTGSGCVGPTSVSQTPRRGQICSAGADSREGNNSPEESGDLVTEVGALDPSG
KPKRSKKRRRRRQGAGRQVVGLPPAPSAAAPVLLWFRRDLRLCDNPALTAALEVGAPVIPIFIWSPEEEEGPA
GVTVATGGACKYWLHQALSCLCASLERNGSHLVFLRSAEAGGEGGSSLQTLKELVKETGARTVMANALYEPWL
KDRDHAAAVALWKSGVECKMFHSYCLRDPYSVRTEGVGLRGIGSVSHFLSCCRQNPGPALGAPLEPPTALPVP
AHWPRGAPLEALGLARMPRRKDGTTVDWAASIRKSWDFSEEGAHARLEAFLGDGVYRYDKESGRADAPNTSCV
SPYLHFGQLSPRWVLWDAKAARCRPPKFQRKLAWRDLAYWQLSLFPELPWESLRPPYKALRWSSDRRHLKAWQ
RGGTGYPLVDAAMRQLWLTGWMNNYMRHVVASFLIAYLHLPWQEGYRWFQVRNGCRGDTGQDTLVDADVAIDA
MMWQNGGMCGLDHWNFVMHPVDAAMTCDPDGSYVRKWCPELSGLPDELIHKPWKCPASVLRRAGVVLGQTYPE
RIVTDLEERRSRSLRDVALVRKQFGQYVDERSGCDLLPLPPRLVSEALGSSPQGGVEAAGGRPFLLPLITRME
FKHQQGDPDADAASNPYNAVLKGYVSRKRDEAVAFLHERDFTASVMQEGARRRERMESDQRRLEGLPRASEPR
GRARRTPTAKDRFSVVPGGAVTPAR
>Tetraodon_nigr__CAF93038.1
MVVNSVHWFRKGLRLHDNPALQEALSGADSLRCVYVLDPWFAGAANVGINRWRFLLEALEDLDCSLKKLNSRL
FVVRGQPTDVFPRLLKVGAGPVRSLVFQSADRAGAMIRASCRCLILSVCVPQEWKVTRLTFEYDPEPYGKERD
GAIIKMAQQFGVETIVRNSHTLYNLDRIVEMNNNSPPLTFKRFQTIVSRLELPRRPLPSVTQQQMDKCGTPVA
DNHDQLYSIPSLEELAPVCPRVSRVQDGRVSSGGLAWRRVGSAGEAPQTSGKEGVGVPPGALAGQHLFPVRQP
GRPQPLPALRLPVLPGVLLQPPGALHQGRTPSALGGRLDEPLSPCPPVPPVSPSCVKAAVPRCPCLDSCCGGS
SSTRRPPTTPTLTAWRATPSVSRWVSTPVAGSQGRRPLGHPQGRVLGTPLVLGGGAAALGGRPWSGDLVVLLL
PLTLRRTPVCPGLQIPWDQNPEALAKWAEGRTGFPWIDAIMTQLRQEGWIHHQARRASACFLTRGDLWISWEC
GMKVGGPPWSRRRRRRLSARLLPQVFEELLLDADWSVNAGSWMWLSCSAFFQQFFKCYCPVGFGRRTDPSGDY
IRRYIPILKDYPNRYIYEPWNAPEAVQKAANCVVGVDYPRPMINHAEGSRLNIERMKQVYQQLSHYRGLSLLA
SVPTI

>Tetraodon_nigr__CAF97542.1
MPAKRRQATPAAKEPVGKQRKLAPTEEEKKTKAGGWLEEEVKQQRTQNKEMGFITKRLRFISDTEEMRQGSEG
VLYWMLRDHRVQDNWALIHARRLALEEKVPLHVCVCLHVPKSQLSTLRHYSFLLKGLEEVAAECKALGIQFHL
LRGPAGDLLPGFVSEQQLGAVVTDFSPLREPLTWLEDVKKKLPKDVPLIQVDAHNIVPCWVASPKLEYAARTI
RGKITKQLPEFLTDFPLVEKHPHAATRTAKPVDWRELLSSLGVDRTVGETEWARPGAAAGLAMLESFIDVRLK
RFDSQRNDPNAAALSQLSPWIRFGHVSAQRVALQVQRGGRKLGVGVSSFIEELVVRRELTDNFCFYNEKYDSV
EGAYEWARKTLKDHAKDKRPYIYTREQLEKAETHDKLWNAAQVVREGKMHGFLRMYWAKKILEWTPSPEEALA
SALYLNDRYELDGQDPNGFVGCMWSICGIHDQGWAERPVFGKIRYMNYKGCLRKFDVAAFERKYGPPASA
>Tetraodon_nigr__CAG02310.1
MGTGEATADGTTYISEKNLRFLLQCLEDLDANLRKLNSRLFVIRGQPANVFPRLFKEWKISRLTFEYDSEPFG
KERDAAIKKLAKEAGVEVIVKISHTLYDLDKIIELNGGQPPLTYKRFQTLISRMDPPEMPVEMLSGNLMGRCV
TPISEDHGEKFGVPSLEELGFDIEGLPSAVWPGGETEALTRIERHLERKAWVANFERPRMNANSLLASPTGLS
PYLRFGCLSCRLFYFKLTDLYRKVKKNTSPPLSLYGQLLWREFFYTAATNNPRFDKMEGNPICVRIPWDRNME
ALAKWAEAKTGFPWIDAIMTQLRQEGWIHHLARHAVACFLTRGDLWISWEEGMKVFEELLLDADWSVNAGSWM
WLSCSSFFQQFFHCYCPVGFGRRTDPNGDFISRSLLASSSDGGRSPSVRLLPRPALSTPSISDSQRCIVLPPT
VSAPSSTPSSDGKVGPGGVGLLASVPSTNGNGNGGMMAYSPGEQPPGTNSNANSNLLGASGGTVATGNGSGSI
LLNFDNEEPTRSNSAGQQPLRQPPQQHWWDSSCTPEDSQEKGSENQNGTVLGRRTVWRAHSTRCRGKAQRSGS
SSSLSFKYSHLGYLANMLTPHW
>Tetraodon_nigr__CAG02357.1
MGHASIHWFRKGLRLHDNPALMAALRDCKELYPVFILDPHLHNKSVGINRCRFLIGALRDLDLSLRNLNSRLF
VVRGKPEEVFPKLFSQWKVTKLTYEYDTEPYSLSRDRTVTTLAEESGVQVVYRVSHTLYDTERVLEENNGKPP
LTYNSMQAIVKKLGPPKRPISAPSMDDLKGVSTPCLEDHEKKYGIPTLEDLGHDPAGLPEEKFPGGEQEALRR
LEDQMKKTSWVCNFEKPQTSPNSLSPSTTVLSPYVTFGCLSARTFWWRLSDVYSGKKHSAPPVSLHGQLLWRE
FFYTASVGIPNFNKMADNPVCTQVDWDTDSEYLAAWREARTGFPFIDAIMTQLRQEGWIHHLARHAVACFLTR
GDLWISWEEGQKVFEELLLDGDWALNAGNWQWLSASAFFHQFFRVYSPVAFGKKTDKNGDYIKKYLPHLKKFP
AQYIYEPWKAPQSIQKAAGCIIGKDYPHPIVKHEEEIMGSRRMTRSSFSNGRSSSGEDSNGTGRPNGCSYISN
VRPENRSTLCTMMAQLAEETQPFFEITLKSKAVSENCNVKFSCVVTGHPAPQLLCCTLEDAAIYQASASNTKG
IVSCSGLLEVGEMNEFRIHERYFAKLKQKARRCKVEGGKENLEPLRTISPDRTLRKRRSTMEGFLSVPSSMED
EGAEEAHQAVEREKELEDTLAPISNGVVTAVIDGQSISENGTMSETYTFESAQKIFTAHPPKTPSTKKIKISQ
VVKSDTKGEQACETQKAEDESTKRAVTNSEEVMEVDSILTSSSSVMSSRDVTENQKVTTGLAPAEKPSKVVNG
GAGAAPQEAHLPPEAAAAAEGKGPAPKAKEESSQMQKLIPDNKIAKLQQSVAPPRPSKAKRIADAAAKKTEDK
PKTTTTGAAGLPQRGRAGHQPSEEELRPRQTKLAETGPALPREIKALESVLAYSVADVTTREPPSTGMQQDLK
DNKSKRMPEEKTPDRKIEDIPLRNIKETLLEVYQQLQLDQVQIEIHDANLAPAEASEKIPPGCAGNTGTTAQS
DNTQKLLSQETGAALVSDLPESIGQEPETPSPEKPVINAPGPNIPVIKHVKHETDQDKHGDFLMKSSTVIPLE
SEISEKLVDQSKTDKLHVEKNAFPNVQKVPDESEPITPIQAAGCNQQDSLMVEDLPQNDPSASLSPKTSSFAK
EKKLHVHSXXXXXXXXXXXXXXXXXXXXXXXXXXXXXXXXXXXXXXXXXXXXXXXXXXXXXXXXXXXX
XXXXXXXXXXXXXXXXXXXXXXXXXXXXXXXXXXXXXXXXXXXXXXXXXXXXXXXXXXXXXXXXXXXXXXXXXXX
XXXXXXXXXXXXXXXXXXXXXXXXXXXXXXXXXXXXXXXXXXXXXXXXXXXXXXXXXXXXXXXXXXXXXXXXXXX
XXXXXXXXXXXXXXXXXXXXXXXXXXXXXXXXXXXXXXXXXXXXXXXXXXXXXXXXXXXXXXXXXXXXXXXXXXX
XXXXXXXXXXXXXXXXXXXXXXXXXXXXXXXXXXXXXXXXXXXXXXXXXXXXXXXXXXXXXXASGARRKIPKQKTSPD
EALQAPSLVGDQVQLHNPSTESPKFSSSPASLPSSPSLQKRSQLLQPAATGEPTLSLARQSPTLSRRKTQSEN
LTQSQNASEEKHIQQTEEKPATKKKHDPFKAPQVIRKIRSETFADASGHLKLWCQFFNVLAESTIRWFRNEEE
IAQVKRSAGDEAQVNLAVVQASYKDSGVYRCTITNEHGTDSTDCLLSGEILTGMSIREDVGVGEEIEMTPMIF
SKGVADSGAWGNKFFGRVAMQESHIGDGCSHKVWRAKVIYGLEPVFESGNTCIIKARNYIPYGGKEESCLTDK
NLEIITQECKIRNLAREYCKIFSAEARVIDNFGPSLEVLPVYLMYRPANAVPYVTVEADLPGDYQRYCVLDHT
GRLGMRTGSDVEQKCCALQHWIFQWTNGNMLLTRLEGVDTKITNVGISVKSSGHQGLPVEGSPKVFEHFISHH
RCNYFCGLLSLRSLKVADSLPMTGKPKASKSPLLQRKMAADSSPQMGRKASGSPRLPRKTDQEGRKTPGKEPP
TDAPLI
>Tetraodon_nigr__CAG04256.1
MRLFDAWKEGKTGVPFVDANMRELATTGFMSNRGRQNVASFLTKDLGLDWRMGAEWFESLLVDHDVCSNYGNW
LYSAGIGNDPRENRKFNMIKQGLDYDSNSGAGPSPRGRKGPSHTPKQHRDRGIDFYFSRSKNL
>Thalassiosira_oce_EJK50169.1_THAOC_3088
MSPRQAMTPPCLLAELAAPATKRERIAVLLTSTPFYRAASAGRRLNRAAPVLAAAAATRTSFVGGHARGQLGT
AVRRATTTTKPKSTREMTKKRAAQKKSGDGGEAPTIAHAPDEVIPAGFDQTRTKLLTQTVMPREKKTDMENPC
IVYWMMRDCRTVDNHALLFAQGLATKYQVPLRVVFTLPPPPSSAPGEGADGSPPKPEDMSLTLRHGDFLLDGL
KVVAEELEAANVPLDVLQPACRTAVGTETIRYCINRNDALALVCDMSPLRQPRDWIETQTVPHLGQQVPVYQV
DAHNIVPVWRASPKREVGARTLRPKINNVFAKYCTQFAEFAGNAHLAAEKVDAENQHDWEQYRRYLRLDESIE
PVVGYVAGHEAAMERFREFCSTKLKNFDSMRNDPNQDVCSNLSPWINHGQVSFQRLALEVRALKKHTNGTAAY
IEEGVVRRELSDNFVYYTPSSYDSLEGAADWARDSLQLHSTDKREYVYTWKVLETGMTHDDLWNAAQLQLVRE

GKMHGFLRMYWAKKVLEWTVSPAYALATAQYFNDRYALDGNDPNGFVGVGWSIMGIHDMGWKERDVFGKIRFM
NYAGCRRKFKVDTFVARYRGAKENANKVAPAGEKVAGKKRKSV
>Thalassiosira_oce_EJK57807.1_THAOC_2211
MGKQHAVENARPVNIHWFRQTDLRLHDNPALRRSVDLSLGKSKNPVVGAAVSESPPHRGNILPVFVFDTSRIY
GSNTRSELGSLKCSARRAQFVLQAVSDLRSNLEKRGSGLIIGVGKPEDVIAELAKGVLTQSSNGAKLNIVCQE
EVCSEELATEKAMRSKLARAMNKGSKFDFETVWGGTMYDPETLPFDGGVNGIPDTFTPFRNKVEKACAIGNPL
PVPSDEELAMPKDAHTIVKESKCHASLDYMPTLADLQYSKEDIEEAELVDIRSALPAGYRGGETFALARVKDY
IWDRDLLKGYFDTRNGMIGSDYSTKFAPWLALGNVSPRYIARECRRYEETRVANKSTYWVVFELLWRDYFKMF
AKKHGASIFMIDGTIGKRDHANKRKWGNDPRHIKAWKDGMTGYPLVDANMRELKATGFQSNRGRQNVCSFLTI
DLNMDWRYGADYFEETLIDYDVHSNWGNWASGSGMTGGRLNRFNIVKQSKDYDYGGDYVRLWCPELEGVPDKY
VHEPWKMSDAMMEACGVKIGPGRDYPAPIVDPTVQPRIMSRGSNGGRGRGRGKQGGRGQGRGNGLRRENNNSN
RSKSQRKDMKSLKEGSYTFRP
>Thalassiosira_oce_EJK58052.1_THAOC_2184
MDGQATLLKDFVAEMNHLGKQNEVLIVSGSGAKARFNSVSALQRLRDDRVEVGPKISVAYNPFFPEEQDLADE
RRRLEQKLETGLVSKVYLQFGTDLDRLRSSLQELKAMQAQAEHNFDICGSIFLPTKKLIAQQKFRPWNGVFLS
DEFLAGETTARGIVLQMMKLYEEHGCEILIEAPGVRTSNDWSVVESLLKERDDKIAASGANVKPEKQRSNAAA
KDASPAKKRKATEVAADTSSEPAASGIGIPKSTLTPQQLAKPAIVLFHSHDCRLHDNVALQLASHHACVIPLF
IWSRKEQGRWGVRGCMEVILKDALRNLDEKLRSHGLELTCRQGDDSSEVLRQLCEECGSSAVYYNVEHTPESG
VREAKYRRLLGEIDVETVECQSSLLYDPTSPQLGTGFHGGHWGTLMPFLKGCKKQLGEPRKPVQRFETFAMLE
AMAGPVTWPSSSSIDGLDLAVIEGEERLLERFPMSEDAAHSNLEQFFKVGFGRYESDRSRADMEWSTSKLSAH
LRVGTLSPNVLYYKTEASELDYPQRKTFSRRLFWRDLAYFHLQSFPGMRWESIRLHYGQTEWCNGEEGDRRFL
AWKRGKTGFPLVDAGMRELYKTGWMTQSIRMVVASFLTEYLRVDWRMGAEWFHYTLADADSAINPMMWQNAGR
SGIDQWNFVMSPVAASQDASGEYTRRWVPELAKLPRGTLHKPWETPAQVLNECGIVLGRDYPHRIVTDLKAER
ALSDEAVLEMRRKNQQFNNERGYDLVQLPNGKETVVFTKKQFRIDRQGIVLKETKKTTGSGAAGRGRRGQRAR
KKAGRDKVAGKR
>Thalassiosira_oce_EJK58421.1_THAOC_2145
MSAKSGENVAMHWFRKGLRLHDNPALLHALSLTKNGGSIFPVYVVDPNSYQLLKCSVNRARFLLECCQDLDES
LKRCGSRLYVATGDPVEVLPEMWKKFGVTHMTHESDETGEPYALQRDTAVAAVAKDAGVEVIDFTSETLRPLG
NREGGYVANCGGDASSVPATMTSFQKLFSRIDKGNIPEPLDAPKKEDFPISADEYENDYLTLKHPCDIPWPRG
QNRSQIGPIWNRADANKVTSHIANGGESYALDRLQKTITARPNWTAQYEKPKTSCTEVSDPSTTVLSPYLSIG
CISPRTVWHAIETANKQAKTNRTQPPVSLHGQLLWRDFNNLMAHSANSQHAGSWGQIKNNAFCRDIPWSSDPN
MLSSWKEGRTGYPWIDACMAQLRSEGWIHHLGRHAVACFLTRGDLWQSWVEGADHFEAELLDADYALNSFNWM
WLSASGFFYQYFRCYSPIQFQKKNDPNGNYIRKWVPELAEMPAKHIYEPWKANVSILKTAGVKLGKTYPRPIV
DHAVVSKENMSKMKLAYDLHKKSQGGGKKPASTSGTEPPKKKAKKVKKQTKLK
>Thalassocalyce_inc_AQX17842.1_putative_p
MTGHATIHWFRKGLRLHDNAALLASIESTSTLIPLYILDTHCLDPQKVGANRMGFLLATLKSLDDDLREKGSK
LFVAKGRPVEVIDALVQELNVQKLTFERDTEPYNKDVDKEIMKKMKSLCVEVHECWGHTLFNPDHLLSLNGGK
SPLNMTGFLNLVSKASTPPAPMEAPTSIPAPPNELKSETIEVLQSLPKLSDLQCYGYNPAEETTWFVPGEKEA
IGRMEQFLAQKKRVAGFEKPKTDPTSLVPDTTALSPYLSCGSLSVRLFYSRLKETLSSTSSTKPPVSLEGQIY
WRELAYLIGYSTPNFGQMVGNPVCMQIPWYTGDNAKEFLKKWEEGLTGYPAVDAAMNQLRTEGWMHHLARHLV
ACFLTRGDLWVSWELGRDVFEKYLLDADWSINNFSWHWLSCSAFFHAYFRCYGPTTFFKKTDKNGAYIRKYVP
LLKKFPDEFIYEPWLASKATQQAAGCIIGEDYPKPIVDHKVVNKQNMEKMKLAYANNKKKSGDEPPAKKKK
>Thalassophryne_amaz__XP_034018857.1
MVVNTIHWFRKGLRLHDNPSLKESLLGADTVRCVYILDPWFAGSSSVGINRWRFLLQSLEDLDSSLRKLNSRL
FVIRGQPTDVFPRLFKEWKITRLSYEYDSEPFGKERDAAIKKLASEAGVEVTVRISHTLYDLDKIIELNQGQS
PLTYKRFQTLISRMEAVAVPAEPITAEVMGKCTTPLSEDHDDKFGVPSLEELGFDTEGLTSAVWPGGETEALT
RLERHLERKAWVANFERPRMNANSLLASPTGLSPYLRFGCLSCRVFYFKLSDLYRKLKKNSSPPLSLYGQLLW
REFFYTTATNNPCFDKMESNPICVQIPWDRNPEALAKWAEGKSGYPWIDAIMTQLRQEGWIHHLARHAVACFL
TRGDLWISWEEGMKVFEELLLDADWSVNAGSWMWLSCSSFFQQFFHCYCPVGFGRRTDPNGDYIRRYLPILRG
FPARYIYDPWNAPDSVQKAAKCIIGVHYPKPMVNHAEASRLNIERMKQIYQQLSCYRGLGLLETVPSNFSGNG
NGDTSDRMGLSAHDAAAPSGYQTPVQAQSGAMMFLQGEPPSHSHQQGSPGTSMMCYSKGVQQVARPALQKAAE
LATSQTSCKRHSDDSTNGTSSKVQRQSH
>Thalassophryne_amaz__XP_034021099.1
MVYYQLRRLICFKGLKDIHLSSRGLQFFSQADVDIVCDCPTWTAMPRPQTKRQKTSNYSAAKAVDKTKLVQRR
DEDFLSVIEQKRLTTAPSIVSFNFQMKRVRVLSSCEEFLDEHSGVLYWMSRDQRVQDNWALLFAQKLALRMAT
PLHVCFCLVPRFLEATIRHYGFMLKGLQEVQQECQKLDISFHLLMGYAQDVLPRFIMDCGIGGVVTDFCPLRL
PTQWVSDMRDELPEDVPFFQVDAHNVVPCWEASSKLEYGARTIRKKITDQLSTYLTEFPPVIQHPHPSKTKPQ
PIDWQSVHASLEVDRSVGEVDWATPGTTGGLRNLESFIKYRLKSFSTSRSDPNKSALSNMSPWYHFGQVSVQR
AMLQVKELSGKYSDSVAAYIEEGIIRRELADNFCYYNDNYDNVGGAWEWARKTLDEHRKDQREYVYTRVQLEE

AQTHDHLWNAAQIQMVREGKMHGFLRMYWAKKILEWTESPEQALEFAIYLNDRYQLDGRDPNGYVGCMWSICG
IHDQGWAERAIFGKIRYMNYNGCKRKFDVPAFVEKCEVAPGLNTTGKK
>Thamnophis_sirt__XP_013909206.1
MGVNSVHWFRKGLRLHDNPALREVIEGADTVRCVYILDPWFAGSSNVGINRWRFLLQCLEDLDANLRKLNSRL
FVIRGQPADVFPRLFKEWNISKLSIEYDSEPFGKERDAAIKKLAIEAGLEVIVRISHTLYDLDKIIELNGGQP
PLTYKRFQTLISRMEPLEMPVETITAEVMSKCATPVSDDHDEKYGVPSLEELGFDTDGLPSAVWPGGETEALT
RLERHLERKAWVANFERPRMNANSLLASPTGLSPYLRFGCLSCRLFYFKLTDLYKKVKKNSSPPLSLYGQLLW
REFFYTAATSNPRFDKMEGNPICVQIPWDKNPEALAKWAEGRTGFPWIDAIMTQLRQEGWIHHLARHAVACFL
TRGDLWISWEEGMKVFEELLLDADWSVNAGSWMWLSCSSFFQQFFHCYCPVGFGRRTDPNGDYIRRYLPILRG
FPAKYIYDPWNAPEGVQKAAKCIIGVNYPKPMVNHAEASRLNIERMKQIYQQLSRYRGLGLLASVPSNGNGNG
NGNGGLMGYSSNENLSGCVGTNGTQMGTSEGHTGNVQSCTHGETHKGKSGIHQQGYAQGNSGILHYAHGDSKK
THLGQQGRTSLNNGLCTGKRPNPEERIQSIGPKVQRQSSN
>Thamnophis_sirt__XP_013913565.1
MSPKKRKPGPATSTSDSSEGGRKPDDGGTEAFAAKKKLKLDDSRTQSLAESVKEARLSAGASVVEFPYNKRRV
RLISPAPDLKEGARGILYWMSRDQRVQDNWAFLYAQRLALKQRLPLHVCFCLVPKFLDATIRHFGFMLRGLEE
VAEECQLLDIPFHLLVGFAKDTLPRFVTQHVIGGVVTDFSPLRVPMQWVRDIQERLPSDVPLAQVDAHNIVPC
WVASEKQEYGARTIRRKIHDQLPKFLTEFPPVVKHPFPAGRPAEPINWEACYSSLEVDRSVKEVEWATPGSSF
GLKVLEEFVKDRLKSFGSDRNNPNRPALSNLSPWFHFGQVAVQRAILEVRKHRARYKESVEGFIEEAVVRREL
ADNFCFYNPNYDKVEGAYDWAKTTLKLHAKDKRSHLQQASDLGPLPPAVGYSQHPLI
>Thamnophis_sirt__XP_013915839.1
GAAAAARPKDRTGQSSHRRRSHLRKGKLDSPATPRHLSRSTSSPGASSAVGINRWRFLLQSLEDLDNSLRKLN
SRLFVVRGQPTDVFPRLFKEWGVTHLTFEYDSEPFGKERDAAIVKLAKEAGVEVTTENSHTLYDLDRIIELNG
HKPPLTYKRFQAIISRMDLPKKPVSTITSQQMEMCKTEIQENHDETYGVPSLEELGFPTEGLAPAVWQGGETE
ALTRLDKHLERKAWVANYERPRMNANSLLASPTGLSPYLRFGCLSCRLFYYRLWELYKKVKQNNSPPLSLYGQ
LLWREFFYTAATNNPKFDRMEGNPICIQIPWDRNPEALAKWAEGKTGFPWIDAIMTQLRQEGWIHHLARHAVA
CFLTRGDLWISWESGVRVFDELLLDADFSVNAGSWMWLSCSAFFQQFFHCYCPVGFGRRTDPSGDYIRRYLPQ
LKGFPSRYIYEPWNAPDSVQKAAKCIIGVDYPKPMVNHAETSRLNIERMKQIYQQLSRYRGLCLLASVPSCIE
DFSNITTDLSSAHGSCSNTILRQSQCTSPKRKCYTGEENSEELHKRARMMIHQGSKNSQQELLQTEFGN
>Thecamonas_tra_XP_013752806.1_cry_DASH
MAAARATAGRTAVCWLRNDLRVADSATMDHVANTCYDAAVFVYCFHPRTYTESWMGLPRTSGHRARFLLDTLS
DLRSALRGLGSDLVVRHGLPAEVIAGLVDELAADVVVYHQAVGSEEADDEAALAAALPDAVTVKSFWGRTLHH
RDDLPFALADLPPTFTPFRKALERAHSVPREIIPRPTSLPPLPDAVEPGKLPSLTDLGLERPPADSRAVHGFP
GGESGAWARIAEWMWDGDALRRYKETRNGMLGGEYSSKLSPWLALGCISPRQVASEVRRYEAERVANKSTYWL
IFELLFADYFTFYAARAGRSLFAARGPADVSNAAWDGSDAAFAAWAAGTTGIPLVDANMRELLATGYMSNRGR
QIVASVLTKNMRVDWRRGAAWFEAQLIDHDVALNYGNWAYVAGVGADPRPDRYFNVAKQAATYDPDGAYQATW
LPPADRAAPSYPATPIIDLAATYPSARNHPAARRSAPRKGRGRARGRYGRKLQKPNKRTVKRATQRAAQLSD
>Thecamonas_tra_XP_013754232.1_cry_photor
MAARMGSRQVAMVWFRKALRVDDNPALAAAADAAAAVVPVFVLDPHLLTRAYVGDTRLVFLLESLLALDSSLR
ALGSQLLVIKGTPEKGALDTLQALWREVGADSLHFEADYEPYAKIRDAKATRMAADDKLAVETSEPNLIYPME
VWESVPDAALPTTFGGMRKLIAKRGWQPQAPIEAPTELPPLPQAAVDAVAKDTERFAVPGMAAFPGRSEGDGS
SRAFPGGEAEAQERMRAFLADVDRVVTFEKPKTNPFAEPPSTTGLSAHLKFGTLSVRRFYEQLRAVEEKSKRS
VSQPPVSLVGQVLWREFYTVVGSRTPGYEAMATNPLSRIIPWDDEPDKVAAWAEGRTGFPLIDAVMRQLRATG
WVHHLARHLVACFLTRGYLWQSWEAGAGVFNSLLLDADHYLNIGNWLWLSASAFFHQYWRVYSPISFGNKYGA
SAHAYVRRWVPELARLPDKHLMAPFDAPEDVLADAGVVLGRDYPEPIVDHAAAKTANLTKMKAAYAAKRRGDP
SAAAGGNDAGSSAAAVGSKRRVSGAEGEPAGKRAKA
>Thecamonas_tra_XP_013762098.1_photolyase
MQASVRPTTDNFALVYALRRAAALRLPLVVAFALTASFPGANARSYSFLLEGVLDAFAGLADAGIGGCVLLPA
DGEAADASMPARVAELAAALASPEVVTDAGYLHIQEQWRTELAAELDDAGVPLVQVEANVVVPVEVVTDKVEY
AARTIRPKINRLRDDYLVPLDDDDASDHGLSLVARPGGAPASTQLPDGVADALGELKLSRAVPADELPREVEN
KAGQAWIAERVEAINADVSVAPVAGKHGGWRAGRDAVDAFVDGKLRQYAAERNVPSQGSTSGLSAYLHYGHIG
PVSVARRVVAAKKASSSSTTRESADAFLEEMVVRRELSMNFCHFMEGESYAKYETAHEGDARQWIYTADELDA
GLTHDPFWNAAQREMVLYGDMHGYARMYWSKKVIEWTESPKAAYAWLRAANDKYELDGRDPNGYVGVAWCFGR
HDRPWTRRDIFGSVRYMNDKGLLRKFKDGIRQWAADINAAYEAKHNLAPGVDAWQYNPNKRPPAGNAGPSAKK
QKKK
>Thelazia_cal_VDN01580.1_unnamed_pr
MVSSRIQPLNKQNIAVGKYVLLLARCIRSEQSPSFSYASQKGNEHGVPVIVTYIYESHRHNVAQRKFLLEGLK
CLESNLAHLHTPLLVIKTANDKEAMEIILRLCDAACEVVTDAAYLREDRCFQEKLSDELVTKCRKITKVEGNV
AVPVTALLNGPAYNAQSIRKVVWHFVDDLLLEKWDDIPKIHCKSLESVLNWDLECLNISKELDKAANECKTNT
KLEGGEIAARRVLNNFIVKKLSAYEKERNIPNSNKQSLLSPYLHFGMISPIAIIAEVMKSSAPKSAKDAFLEE
LVVRRELSHNFVYYYRDTYDTFDCLPDWAKKTMDEHQRDKREYIYNYKELEEGRTHDVFWNAAQFELVFAHKM

SGYLRMYWAKKVIEWSADYKTAYSFLIEQNDKYELDGRDPNGFCGVMWNFGMHDRAHAERPVFGKLRYMCADG
LRRKFKKYIDEYVRVNYKLAGRKLEIGESQPKKRNSLKDFDFKVKKVRKS
>Thelohanellus_kit_KII71670.1_photolyase
MIFSQRLALKYEIPLVVVFCLSKSYMRYSRRHFDFMIRGLDSVSTSLKALKIPMHVLLGDPTPNILKFVKNNN
VSAVIADFLPLDPYKSWLEDLKQEIPEDVSLCQVDAHNIVPAWIASPKREYAARTIRPKIHQHLDEFLTEFPP
IIPHEPNKSLSFPDFEISKYIKFVEDAADVKPITWAVPGPDQGLLELEKFVSERLGTFADTRNDAANDLTSNL
SPWFHFGHLSTQRTILRAIEFKNKYKKHVEIFIEEAVVRRELSENYCYYTEEPESPSSAPNWAVQTLKKHLKD
ERPYVYTLEELENAETHDHIWNAAQKQLTGKGKMSNYLRMYWAKKILEWSADPETALKNSLILNDKYSIDGND
CNGVVGCMWAVYGLHDRPWRERPIFGIVRYMSEVGCNRHFNIEEYVEKHIKAE
>Thinocorus_orbi__NXP02623.1
MSHRTIHLFRKGLRLHDNPTLVAALESSEVLYPLYILDRKFMTSVMHIGALRWHFLLQSLEDLQKNLSQLGSC
LLVAQGEYEAVLRDHVQKWNITQVTLDAEMEPFYKEMEAKIRRLGEELGFEVLSLVGHSLYDTKRILDLNGGT
PPLTYKRFLHILSLLGDPEVPVRNLTAEDFQRCQPPDPGLAETYGVPLPVDLKLPPENLSPWRGGETEGLQRL
ERHLTDQGWVASFTKPRTIPNSLLPSTTGLSPYFSLGCLSVRTFFYRLSNIYAQAKHHSLPPVSLQGQLLWRE
FFYTVASATPNFTQMAGNPICLQIRWYEDAERLHKWKTAQTGFPWIDAIMTQLRQEGWIHHLARHAVACFLTR
GDLWISWEEGMKVFEELLLDADYSINAGNWMWLSASAFFHQYTRIFCPVRFGRRTDPEGHYIRKYLPVLKNFP
SKYIYEPWTASEEEQQQAGCIIGRDYPFPMVNHKEASDHNLQLMKQVREEQPRTAQLTRDDTDDPMEMKVKRD
HSEETIPKGKVAR
>Thinocorus_orbi__NXP02785.1
MQHSSIHWFRKGLRLHDNPALLAAIPDCCRLHPLFILDPSSSRAGPNAQRFLLDALRDLDGSLREIGSRLLVV
RGCPEEVFPRLFHAWGTTRLTFEVDTEPLARQRDTAVAKLAAQHGVEVIQKVSHTLYDTERVLALNDGQAPLT
YKRLQNLLASLGPPEKPAPVLTREHLQGCCTPCQVSDDIDYGIPTLEDLGQDPTKLGPHLYPGGETAALARLN
AHMERTAWVRSFKKPETEPNSLNPSTTVLSPYLKLGCLSVRTFWWRLDEVCQGREHSQPPVSLHGQLLWREFF
YTAGASIPNFDRMVGNPVCLQVDWDDNPQHLQAWREGQTGYPFIDAIMTQLRSEGWIHHLARHAVACFLTRGD
LWLSWEEGLKVFEELLLDADWSLNAGNWLWLSGSAFFHRYFHVYSPVAFGKKTDRDGAYIRKYLPILKGFPTQ
YIYEPWKAPRAVQEQAGCLVGTHYPRPIVEHCGASKRNMGRMKAARAQK
>Thinocorus_orbi__NXP07809.1
RFLLQCLEDLDANLRKLNSRLFVIRGQPADVFPRLFKEWNIAKLSIEYDSEPFGKERDAAIKKLASEAGVEVI
VRISHTLYDLDKIIELNGGQPPLTYKRFQTLISRMEPLEMPVETITPEVMKKCTTPVSDDHDEKYGVPSLEEL
GFDTDGLPSAVWPGGETEALTRLERHLERKAWVANFERPRMNANSLLASPTGLSPYLRFGCLSCRLFYFKLTD
LYKKVKKNSSPPLSLYGQLLWREFFYTAATNNPRFDKMEGNPICVQIPWDKNPEALAKWAEGRTGFPWIDAIM
TQLRQEGWIHHLARHAVACFLTRGDLWISWEEGMKVFEELLLDADWSVNAGSWMWLSCSSFFQQFFHCYCPVG
FGRRTDPNGDYIRRYLPVLRGFPAKYIYDPWNAPESVQKAAKCIIGVNYPKPMVNHAEASRLNIERMKQIYQQ
LSRYRGLGLLATVPSNPNGNGNGGLMGYSPGESISGCGSTGGHLFPTSPGAQLGTGDGHTVVQPCALGDSHTG
ASGIQQQGYCQASSILHYAHGDNQQSHLLQAGRTTLGTGISAGKRPNPEEETQSVGPKVQRQSTN
>Thinocorus_orbi__NXP08128.1
RFLLQSLEDLDNSLRKLNSRLFVVRGQPTDVFPRLFKEWGVTRLTFEYDSEPFGKERDAAIIKLAKEAGVEVV
IENSHTLYDLDRIIELNGHKPPLTYKRFQAIISRMELPKKPVSTIMSQQMETCKVDIQENHDDMYGVPSLEEL
GFPTDGLAPAVWQGGETEALARLDKHLERKAWVANYERPRMNANSLLASPTGLSPYLRFGCLSCRLFYYRLWE
LYKKVKRNSTPPLSLYGQLLWREFFYTAATNNPKFDRMEGNPICIQIPWDRNPEALAKWAEGKTGFPWIDAIM
TQLRQEGWIHHLARHAVACFLTRGDLWISWESGVRVFDELLLDADFSVNAGSWMWLSCSAFFQQFFHCYCPVG
FGRRTDPSGDYVKRYLPKLKGFPSRYIYEPWNAPESVQKAAKCIIGVDYPKPMVNHAETSRLNIERMKQIYQQ
LSRYRGLCLLASVPSCVEDLSGPVADSASGQGCSTSTAMRLSQADQASPKRKHEGAEEPCTEELYKRAKVTGL
PAPEIPGKSL
>Thinocorus_orbi__NXP08178.1
DNWAFLYAQRLALKQELPLHVCFCLVPKFLDATIRHYGFMLKGLQEVAKECAELNIPFHLLLGYAKDVLPAFV
VEHDVGGLVTDFCPLRLPRQWVEDVRERLPEDVPFAQVDAHNIVPCWVASPKQEYSARTIRGKIHAQLPEFLT
EFPPVICHPYPPSCPTEPIAWEACYSSLQVDCTVKEVEWATPGTAAGLAVLQSFIAERLKSFGSHRNDPNKAA
LSNLSPWFHFGQVSTQRAILEVQKHRGKYKESVDAFVEEAVVRRELAENFCYYNENYDSVQGAYDWAQTTLKL
HAKDKRPFLYKLQELEQGTTHDPLWNAAQLQMVREGKMHGFLRMYWAKKILEWTRSPEEALQFAIYLNDRYEL
DGRDPNGYVGCLWSICGPGWA
>Thinocorus_orbi__NXP10417.1
KVDYDVCSNYGNWLYSAGIGNDPRDNRRFNMIKQGLDYDGNGDYVRLWIPELQGIKGADIHTPWALNCAALSQ
AGVTLGETYPQPVVTAPEWSRHINQRPQGQSPHPRGRRGPAHMPRQHRDRGIDFYFSRKKDA
>Thrips_pal_XP_034230704.1_cry-2_X1
MSKATAIHWFRKGLRLHDNPALIAAIKAEVELRPIFIFDPWYEENVRCGANRWRFLHQSLSDLDKNLRQLGTR
LFVFRGKPEEVLPMLFNEWKVQRLTFEIDIEPYSRQRDDQVTRIAHQLDVEVIQKVSHTLYNTELVLKANMDA
VPLTYVKAVSLMKSLGEPPQAVPAPTKLPPECRAAAALLEDHKYDIPTLEELGVDQSQLGPCLFPGGETEGLA
RLEKCLERKDWICSFEKPKTIPNSLAPSTTVLSPYIRFGCVSSRFVYHKINMVLKGRKHSQPPVSLIGQMYWR
EFYYIAASGTPNFDKMVGNKVCCKVPWDENPEWVERWAQGKTGYPFIDACMRQLRQEGWIHHLARHAVACFLT

RGDLYQSWEEGQKVFEELLLDADWALNAGNWMWMSASAFYHTFYRVYSPVAFGKKTDKTGEYIRKYVPEVSKL
PSNYLYEPWEASLSVQKAAKCVVGEDYPHRLVKHEDVYKVNIGRLSAAYKANKILQKEQDEADDDCPHPPAKK
RKGKGK
>Thrips_pal_XP_034231046.1_photolyase
MSSKMSSSKEPPKKKVKTEEASSPTTSKSDTNLKQRLVQDRKETAPSILEFKFNKKRVRILSEATDVSENCKG
IAYWMFRDERVQDNWAFLFAQRLALKNKVPLHVCFCLLTKFLDGTIRQFKFLLKGLREVRKECEELNIEFHML
YGSGAEVMPDFIKKNKIGALVIDFMPLRNVMNYADQLKKTLPKDVPLCQVDAHNIVPCWQASEKLEYGARTIR
PKIHKQLPEYLTQFPPVIKHPYSGSLKADKIDWEKCEAHLEVDRTVDEVQWAVPGYSGGISTLESFINKRLKI
FGSKRNDPTLDALSNISPWLHFGQVSAQRCILVVKALRSKHPESVDAYIEEAIVRRELSDNFCFYNPKYDSIE
GTNEWAKKTLNDHKKDKRPYLYSREELQNSKTHDDLWNSAQIQLVKEGKMHGFLRMYWAKKILEWTESPEQAL
SDAIYLNDRYSLDGRDPSGYVGCMWSVCGIHDQGWGERDVFGKIRYMNYQGCKRKFDIAAFVSRYGGKVYTKS
SSTLDKFVSKGKKK
>Thrips_pal_XP_034239242.1_cry-1-like
MSSSEPESEPSAAASATPTPTATPSATSTATPSATPSATPSATPSATPSATPSATPAAQSATSVSKSALPAPA
PGGCAPSLAGNQGNQAHSMTVPMAVPALSRQKHSVHWFRKGLRLHDNPSLREGLRGATTLRCVFILDPWFAGS
SNVGINKWRFLLQCLEDLDRSLRKFNSRLFVIRGQPADALPKLFKEWGTTNLTFEEDPEPFGRVRDQNIMTMC
KELSISVVSMVSHTLYELQKIIEKNGGKPPLTYHQFQNVVASMDLPPQAKPTITASNMQNCFTPIADDHDDKY
GVPTLEELGFDTEGLKPAVWIGGETEALARLEHHLERKAWVASFGRPKMTPQSLLPSQTGLSPYLRFGCLSTR
LFYYQLTDLYKKIKKAVPPLSLHGQLLWREFFYCAATRNPFFDKMVGNPICVQIPWDHNAEAIAKWANGQTGF
PWIDAIMTQLREEGWIHHLARHAVACFLTRGDLWISWEEGMKVFEELLLDADWSVNAGMWMWLSCSSFFQQFF
HCYCPVRFGRKADPNGDYIRRYLPVLKNFPTRFIHEPWIAPESVQRSAKCIVGKDYPKPMVNHGLASRINIER
MKQVYTQLSKYRGSGTGAGSSLLKEQLRLLATVPNSMPLSFLPKKSQSPQGSHASAYDQAENERNHEQPLLHQ
QQPAKQQHQQQPVKQQQFQQYSQHYQHPQQQEISQQSQLHLFPHQSHSHQHLFQQQPPISSAEVDSHLMAPPK
SPYQSPLAQSSQACKMQILQLVPQQQVQTLDAHQISPMQPQQLHQESHSQQLRSLNVYSQHIGSSTYNNQHVQ
QVHNLPQQMNSYQIDLPHQNYTTEHMEEGDQNQHQWSKENTWQEFNPEFKELKHERETAAETDDRGIVNYNHD
ADLENNLTL
>Thrips_pal_XP_034243121.1_cry-1
MALIEEISPKGSVLWFRRGLRLHDNPALLKALSVKQEFYPIFIFDGLSAGVTKDTSYNRVRFLLECLQDLDEQ
LQQRMGKLYFIRGDPKNVFEAIHKIKPLESVSFELDPEPIWASRDNKVKTCLENCGIKWTEEISHTLWDPHEI
IETNGGIPPLTYEMFLHTVSIIGAPPRPVDDPDWTGVQFGKLPQHLGDDIEVFQSIPSPEKLGYFPPKVFSEK
IIEWTGGEQKALLKMNERLKVEENAFLNGFYLPNQAKPDILGPPTSQSAALCFGCLSVRKFYWAIHDMYSRIY
SGHSQSNQHITGQLIWREFFYTMCVQNQYYGEMERNPICLNISWKEDVQDEIQKWENGQTGYPFIDAVMRQMH
QEGWVHHVARNAVACFLTRGDLWMNWEIGLRLFLRLLLDADWCLCAGNWMWVSSSAFEQLLDCSSCVCPVSYG
RRLDPQGEYIRRYIPELRNFPQEYLYEPWKAPLDVQERAGCFIGREYPDRMVDHADASQKNRRMMRDLRLSLV
DDTPHCCPSNVEEVRQFMWLPESCTDHLCETEKV
>Thunnus_alba__UBK24976.1
FGTQRNDPNAAALSQLSPWIRFGHLSAQRVALQVQRSGKNAGQSVPSFIEELVVRRELTDNFCFYNKKYDSVE
GAYEWAQKTLKDHAKDKRPYLYTREQLEKAKTHDKLWNGAQYQMVSEGKMHGFLRMYWAKKILEWTSSPEEAL
SIALYLNDRYELDGQDPNG
>Thunnus_alba__XP_044211749.1
MPSPVVGGAEDSKAVVRKMLREVLVGREDPEGFFAMCVSVLGHQETRSQFLSLIQPLSTANSSLHSVLTSIYR
EYFTKTEEDELELALTLSLLDMQDHQPSTPSQESRLQLPGNRPNQSSSVHLTSVSNSQGNSYTQVVDEAGRRK
NTNLPQTGPWGKVSPTQTTRESELQKSTHFDKYNKETPGMSQTVCVSRQSASFSKQDTVREGDQLMDSGELNQ
SEKSKRSKNRRQRRKGGSQQVVGLPCSPSAPAPVLLWFRRDLRLCDNPALCGSLEVGAPIIPVFIWSPEEEEG
PGITVAMGGACKYWLHQALSCFCSSLERIGSHLVFLKANGEGNEAGSSLRTLKELVKDTGARTVLANALYEPW
LKERDDMVVSALQKDGVECKMFHSYCLRDPYSVSTEGVGLRGIGSVSHFMTCCRQNTGSALGAPLDPPVSLPT
PAHWPLGVSLETLGLARMPRRKDGTMIDWAANIRKSWDFSEEGAHARLEAFLHDGVYRYEKESGRADAPNTSC
LSPYLHFGQLSPRWLLWDAKGARCRPPKFQRKLAWRDLAYWQLTLFPDLPWESLRPPYKALRWSSDHGHLKAW
QRGRTGYPLVDAAMRQLWLTGWMNNYMRHVVASFLIAYLHLPWQEGYRWFQDTLVDADVAIDAMMWQNGGMCG
LDHWNFVMHPVDAAMTCDPCGSYVRKWCPELADLPDELIHKPWKCPASMLRRAGVVFGQTYPERIVTDLEVRR
SQSLQDVALVRKEFGQYVDKRTGCDLVPLPPRLVSEALGLSHSTGGVVTAGKQFLLPVITRMEFKHQLEDPDA
DAASNPYNAVLKGYVSRKRDETIAFLNERDFTASVMYEGTQRKERLESDYRRMEGLPRPPAPRGRARRTPTAK
DRFSIVPGGAVTSLR
>Thunnus_alba__XP_044211978.1
MPVGGDMLIQKGRLAWPKSLHGVTINLYSMFNVLAGDMVVNTIHWFRKGLRLHDNPSLRDSIPGADTLRCIYI
LDPWFAGSSNVGINRWRFLLHCLEDLDASLRKLNSRLFVIRGQPTDVFPRLFKEWQITHLSYEYDSEPFGKER
DAAVKKLASEAGVEVVVRISHTLYDLDKIIQVNGGQPPLTYKRFQAVINCMDAVGLPAETITSEDIKKCATPI
SEDHDDKFGVPSLEELGFETEGLTTAVWLGGETEALLRLERHLERKAWVANFERPRMNATSLLASPTGLGPYL
RFGCLSCRLFYFKLTDLYRKVKKNSTPPLPLYGQLLWREFFYTAATNNPCFDKMEGNPICVQIPWDRNPEALA
KWVEGQTGFPWIDAVMIQLRQEGWIHHLARHAVACFLTRGDLWIRWEEGMKVFEELLLDADWSVNAGSWMWLS

CSSFFQQFFHCYCPVGFGRRTDPSGDYIRRYLPILRGFPARYIYDPWNAPEEVQKAAKCIIGVHYPKPMVNHA
EASRINIERMKQIYQQLSCYKGLGLLETVPANPNNGASGSDIGRVNSGTSQGLGRSSEDPLVLKGTSQTDRGQ
STQKRRCEEAPPESSSKSWKQSKLLQKAE
>Thunnus_alba__XP_044213520.1
MVVNSVHWFRKGLRLHDNPALQEALNGADTVRCVYILDPWFAGAANVGINRWRFLLEALEDLDSSLKKLNSRL
FVVRGQPTDVFPRLFKEWKVTRLSFEYDPEPYGKERDGAIIKMAQEFGVETVVRNSHTLYNLDRIIEMNNNSP
PLTFKRFQTIVSRLELPRRPLPPITQQQMDKCHTKIADNHDQLYSIPSLEELGFRTEGLLPAVWKGGESEALD
RLNKHLDRKVWVANFEHPRVNTCSLYASPTGLSPYLRFGCLSCRVLYYNLRELYMKLRKRCSPPLSLFGQLLW
REFFYTAATNNPNFDRMEGNPICVQIPWDQNPEALAKWAEGRTGFPWIDAIMTQLRHEGWIHHLARHAVACFL
TRGDLWISWESGMRVFEELLLDADWSVNAGSWMWLSCSAFFQQFFHCYCPVGFGRRTDPSGDYIRHYIPILKD
YPNRYIYEPWNAPESVQKAANCVVGVDYPKPMINHAEGSRLNIERMKQVYQQLSHYRGLSLLASVPTIQEEAE
PPMTDESQTSSGPDSPPRGAADGEDTGCSIAPDSSTICASSNTAPHPDLEEAATSHPSQTACTSSGSHGQYPA
AITSTSAGHPPATAAPPSPASTQAQSPLSRSKPSSPSSSCPALSPSTSPATTLTQTSCLGPRRKSLTRKVRRS
QRQRGRQSCTPAPREGERRAEDEEREEAGGDERMEEDVEQEEERMEEETCGELAGHQQ
>Thunnus_macc__XP_042255563.1
MSTSRTIICLLRNDLRIHDNELFYWAQRNAEHIVPLYCFDPRHYVGTYNYNLPKTGPFRLRFLLDSIRDLRNT
LRNKGSNLVVRRGKPEDVVADLIKQLGSVSTVAFQEEVTSEELNVEKKVKDVCAQMKVKVHSCWGTTLYHRDD
LPFHHISRLPDVYTQFRKAVETQSRVRPVFPTPEQLKPLPSGLEEGAIPTAEDLQQTEPVADPRSAFPCAGGE
SQALGRVKHYFWDTDAVATYKETRNGLIGVDYSTKFAPWLAMGCISPRYIYHQIQQYERERTANQSTYWVIFE
LLWRDYFKFVAVKYGDRLFQIEGLQDKSVPWKTDMKLFDAWKEGRTGVPFVDANMRELAMTGFMSNRGRQNVA
SFLTKDLGLDWRMGAEWFEYLLVDHDVCSNYGNWLYSAGIGNDPRENRKFNMIKQGLDYDNNGDYVRQWLPEL
QKIRGSDVHTPWTLSSAALSHAQVSLGETYPTPIIIAPEWSRHGNKKPSGTGPSPRGKKGPSHTPKQHRDRGI
DFYFSRSKNL
>Thunnus_macc__XP_042264816.1
MAPNSIHWFRKGLRLHDNPALREAVRGAGTVRCVYFLDPWFAGSSNVGVNRWRFLLQCLEDLDASLRKLNSRL
FVIRGQPANVFPRLFKEWKISRLTFEYDSEPFGKERDAAIKKLAMEAGVEVIVKISHTLYDLDKIIELNGGQP
PLTYKRFQTLISRLDPPEMPVETLSDSLMGRCVTPICEDHGDKYGVPSLEELGFDTEGLPSAVWPGGETEALT
RIERHLERKAWVANFERPRMNANSLLASPTGLSPYLRFGCLSCRLFYFKLTDLYRKVKKNSSPPLSLYGQLLW
REFFYTAATNNPRFDKMEGNPICVRIPWDKNPEALAKWAEAKTGFPWIDAIMTQLRQEGWIHHLARHAVACFL
TRGDLWISWEEGMKVFEELLLDADWSVNAGSWMWLSCSSFFQQFFHCYCPVGFGRRTDPNGDFIRRYLPVLRG
FPAKFIYDPWNAPESVQAAAKCIIGVHYPKPMVHHAEASRLNIERMKQIYQQLSRYRGLAQ
>Thunnus_macc__XP_042266478.1
MPSPVVGGADDSKAVVRKMLREVLVGREDPEGFFAMCVSVLGHQETRSQFLSLIQPLSTANSSLHSVLTSIYR
EYFTKTEEDELELALTLSLLDMQDHQPSTPSQESRLQLPGNRPNQSSSVHLTSVSNSQGNSYTQVVDEAGRRK
NTNLPQTGPWGKVSPTQTTRESELQKSTHFDKYNKETPGMSQTVCVSRQSASFSKQDTVREGDQLMDSGELNQ
SEKSKRSKNRRQRRKGGSQQVVGLPCSPSAPAPVLLWFRRDLRLCDNPALCGSLEMGAPIIPVFIWSPEEEEG
PGITVAMGGACKYWLHQALSCFCSSLERIGSHLVFLKANGEGNEAGSSLRTLKELVKDTGARTVLANALYEPW
LKERDDMVVSALQKDGVECKMFHSYCLRDPYSVSTEGVGLRGIGSVSHFMTCCRQNTGSALGAPLDPPVSLPT
PAHWPLGVSLETLGLARMPRRKDGTMIDWAANIRKSWDFSEEGAHARLEAFLHDGVYRYEKESGRADAPNTSC
LSPYLHFGQLSPRWLLWDAKGARCRPPKFQRKLAWRDLAYWQLTLFPDLPWESLRPPYKALRWSSDHGHLKAW
QRGRTGYPLVDAAMRQLWLTGWMNNYMRHVVASFLIAYLHLPWQEGYRWFQDTLVDADVAIDAMMWQNGGMCG
LDHWNFVMHPVDAAMTCDPCGSYVRKWCPELADLPDELIHKPWKCPASMLRRAGVVFGQTYPERIVTDLEVRR
SQSLQDVALVRKEFGQYVDKRTGCDLVPLPPRLVSEALGLSHSTGGVVTAGKQFLLPVITRMEFKHQLEDPDA
DAASNPYNAVLKGYVSRKRDETIAFLNERDFTASVMYEGTQRKERLESDYRRMEGLPRPPAPRGRARRTPTAK
DRFSIVPGGAVTSLR
>Thunnus_macc__XP_042266889.1
MVVNSVHWFRKGLRLHDNPALQEALNGADTVRCVYILDPWFAGAANVGINRWRFLLEALEDLDSSLKKLNSRL
FVVRGQPTDVFPRLFKEWKVTRLSFEYDPEPYGKERDGAIIKMAQEFGVETVVRNSHTLYNLDRIIEMNNNSP
PLTFKRFQTIVSRLELPRRPLPPITQQQMDKCHTKIADNHDQLYSIPSLEELGFRTEGLLPAVWKGGESEALD
RLNKHLDRKVWVANFEHPRVNTCSLYASPTGLSPYLRFGCLSCRVLYYNLRELYMKLRKRCSPPLSLFGQLLW
REFFYTAATNNPNFDRMEGNPICVQIPWDQNPEALAKWAEGRTGFPWIDAIMTQLRHEGWIHHLARHAVACFL
TRGDLWISWESGMRVFEELLLDADWSVNAGSWMWLSCSAFFQQFFHCYCPVGFGRRTDPSGDYIRHYIPILKD
YPNRYIYEPWNAPESVQKAANCVVGVDYPKPMINHAEGSRLNIERMKQVYQQLSHYRGLSLLASVPTIQEEAE
PPMTDESQTSSGPDSPPRGAADGEDTGCSIAPDSSTICASSNTAPHPDLEEAATSHPSQTACTSSGSHGQHPA
AITSTSAGHPPATAAPPSPASTQAQSPLSRSKPSSPSSSCPALSPTTSPATTLTQTSCLGPRRKSLTRKVRRS
QRQRGRQSCTPAPREGERRAEDEEREEAGGEERMEEDVEQEEERMEEETCGELAGHQQ
>Thunnus_macc__XP_042275043.1
MAHTCIHWFRKGLRLHDNPALMAALRDCKEIYPVFILDPHLHNSTCVGINRWRFLIGALEDLDCSLKKLNSRL
FVVRGKPDDVFPKLFKEWKITKLTYEYDSEPYSLSRDKRVTALAKEHGVEVIYKISHTLYDIERIIEENNGKA
PLTYNRMQAIVKTLGPPKRPISAPTMEHMKDVKTPYSENHEKKYGIPTLDELGQDMTELGEEPFPGGEQEALR

RLDENMKRTGWVCSFEKPQTSPNSLSPSTTVLSPYLTLGCLSARTFWWRLTEVYQGKKHSDPPVSLHGQLLWR
EFFYTASVGIPNFNKMEGNPVCTQVDWDTNPEYLAAWREARTGFPFIDAIMTQLRQEGWIHHLARHAVACFLT
RGDLWINWEEGQKVFEELLLDGDWALNAGNWQWLSASTFFHQFFRVYSPVAFGKKTDKNGDYIKKYLPLLKKF
PVQYIYEPWKAPRSIQQAAGCIVGKDYPHPIVQHEVISKKNIQRMKLAYAKRSKDPAESPSKKQGVKRKAPSV
IDMLKKKNKTA
>Thunnus_macc__XP_042283740.1
MLHCFCRSSPFSSSPRLYLRLVIHNRQRPGSVFSCLTNSFTSENTMADKKRKATSAVAKEPSAKQQKLAPMKE
EKTQRAAGWLQELVRQQRTEKKEMKFNKKRLRFLSDTQKIKQGSEGVLYWMSRDHRVQDNWAMIYAQRLALKE
DLPLHVCVCLVVPKSELSTLRHYSFMLKGLEEVEKKCKHLGIQFHLLHGSGGEVLPGFVSDRGLGAVVTDFSP
LREPLQWLEDLKKKLPKDIPLIQVDAHNIVPCWVASPKLEYSARTIRGKITNLLPEFLTDFPLVGKHPYTATR
TAKPVDWAETLASLQVDRTVGEPEWAKPGTKAGIAMLESFIDVRLKMFGTQRNDPNAAALSQLSPWIRFGHLS
AQRVALQVQRSGKNAGQSVPSFIEELVVRRELTDNFCFYNKKYDSVEGAYEWAQKTLKDHAKDKRPYLYTREQ
LEKAKTHDKLWNGAQYQMVSEGKMHGFLRMYWAKKILEWTSSPEEALSIALYLNDRYELDGQDPNGFVGCMWS
ICGIHDQGWAERPVFGKIRYMNYKGCLRKFDVARFERKYCPKNL
>Tigriopus_cal_TRY67619.1_TCAL_02907
MTQSTGSSAIHWFRKGLRLHDNPALLSALAQPGELRPLFILDPHFVAHAHVGVNRWRFLQQSLQDLDDQLRAR
GSRLFVARGQPETILPELFKQWRVQWLTFEVDTEPYALKRDAQIEALAQEAGVVVDSRISHTLYSVHRVIQAN
HGKAPLTYVSFQKVVAKLGDPPTAVACPPTFPTQSQLSSPADLSDVRYNVPELEELGVKAAELRTCRFPGGET
EGRRRLQEYLSDKRAGWVRAFEKPQTPPNSLEPSTTVLSPYLKFGCVSSREMYWRLQDILKKGKHSQPPVSLV
GQLLWREFYYVVAADTPHFDRMKGNPLCKQIPWDDNPEYLKAWSEARTGYPWIDAVMIQLRDEGWIHHLARHA
VACFLTRGDLYLSWEDGQRVFEELLLDADWALNAGNWMWLSASAFFHQYFRVYSPVAFGKKTDPTGEYIRKYV
PKLKKYPKEYIYEPWKAPLSVQKAAGCIIGTDYPKPIVNHDTIHKVNIGRHKAAYSQGKDSNQASGTTKPAKR
PETGSKGQTSMTKFMKKK
>Tigriopus_cal_TRY67732.1_TCAL_12461
MVPPSNIVQHFVKRSNRNKQILKMSVGLVTLSILVITYSQYLLHNHCDALECGNDFRSKRQVIETTFMSKSEK
TSAASESSESTSSVRHPTSTLGAGTSSEGELDIPVEFDSPLMPPKQSQELAKWLQKMMMDLNLNRADPTPMEE
GSKEYLFPCAHDDYKISESLTKVNPTEGQTLDGQGWVVFPEDSLCYSLKILKVTGQFNDGKLSGSAKVDFTDG
SWMRGNFVKGVLHGLVRKFWCKFGDCEYFESKKWSVPEHLKEISWYRNGVRTGFAWEFLRGGGMMAGEVNEIG
ELSGPEVSYLYPDKYTMLVGKAIKGQFQSGRESRLSGIEYHNEIIAVPLHLKAQGPLFVREVSNKTHMASNDN
SLHQSDPYEKQFVSVQDSTVRGSGRGVFLKKDVPMGMIIGFYNGVRMSDFESKIRKADRKSPYRMDNDWAKSK
QILNIPEGFREPHQYNATLGHLINHGQKPNAWYAMIDHPRFGFIRSVVTKQALKAGEEIFCDYGFLKQYAQSD
EMFKTILEVGKMIHGSDNNADFTKDGEEYHDQAELKRPADKSELSSLVERSVGAAIYRNKAGGPRIIELNMGK
PPLTYRQFIKIVSEMKPPPVPVPALSSRMLGNATSPISEDHDERYGVPSLAELGFDTKNLPPSAWHGGETEAL
SRLERHLERKAWVASFGRPKMTPQSLLASQTGLSPYLRFGCLSTRLFYHALGDLFRKIRKEEPPLSLYGQLLW
REFFYTAATKNPNFDRMYGNPICVQIPWDKNPEALAKWANGSTGFPWIDGIMTQLRQEGWIHHLARHAVACFL
TRGDLWISWEEGMKVFDELLLDADWSVNAGTWMWLSCSSFFQQFFHLYCPVRFGRKADPNGDYIRRYLPVLKN
FPTRYIHEPWTAPEQVQKAAKCIIGKD
>Tigriopus_cal_TRY68590.1_TCAL_13499
MSRKSQAAGPPTKKARPSTDSSSSSSSTTDIEAELERSRLTVDSDVSQFDFNAQRVRWLQGGPPDTPATPRPT
PGAVVYWMSRDQRVQDNWALLYGQKLAWAHGWPLHVVFCLVPGFLDATLRQYDFMIRGLQEVERELAVRQIHF
HLLRGAAAERLPAFLASRAVTCLVTDFSPLRVPRQWVERVQKQLDPQLPFAQVDAHNVVPVWVTSDKQEYAAR
TIRSKIHRKLDEFLTEFPPVSPHPHFQAADSSPIDWTEVFDWLTVDTSVPPVTSFTPGTRAALQNLAQFAGQR
IPSYADRRNDPNVAVLSNISPWAHFGQISMQRAVLYVKKHGRSSAGVAGFVEEAIVRRELSDNFCYYQPDYDN
VKGAFGWAQKTLAEHRADPRPYLYDRSELEKAHTHDDLWNAAQIQMVKEGKMHGFLRMYWAKKILEWTQSPEE
ALATAIYLNDRYQLDGRDPNGFVGCMWSICGIHDQGWAERKVFGKIRYMNYDGCKRKFDIKKFILKYGAKAHP
YLKKVKK
>Tigriopus_cal_TRY72624.1_TCAL_03947
SVVFQKEVCSEEIQVESSIVKICQAAKIQVKTFWGSTLFHRDDLPYAKKDIPDTYTQFRKTVEARCKVRGLTA
ECPTWKPLPQVENGKLGDIPSLADLKVEPVEVGRSYPDPRSAFPFAGGETSGLDRIHRYYWVSDNVAKYKETR
NGLLGSEYSTKFSAWLANGSVSPRYIYHKLKEYEAERVSNQSTYWVIFELIWRDYFRFVSWKYADLIFYPGGI
MGKAYPWQTNKTLFQMWCQGRTGVPFVDANMRELLYTGWMSNRGRQNVASFLVKDLNLDWRLGAEWFESLLLD
HDVCSNYGNWNYSAGIGNDPRENRKFNMIKQGLDYDPEGKFVQTWVPELQRVPQGKIHVPWTLSEKESGSIGL
ELGVDYPTPVITAPEWSRHFHKTSAKPKDPRNAAGRGRGSKPKSGQQKGIDFYFKSGHTKS
>Tigriopus_cal__TRY63317.1_TCAL_01827
MSSDPHSMEKPVSILWFRHGLRLHDNPSLHEAVQDAEKEGWQLLLMFIFDGETAGTKLCGLNRFNFLLECLKD
IHDQLQTLGTRLHVCHGAPIEIFEAIHQTRGIAKLCFEQDCEPVWKTRDEGVKNWCQTTGVPWVEKIGHTLWN
PHEVLALNGGTPPVTFAMFNHVISAMPKPDRPKADVDMSQVQFAAIDDELGRKIGLYPTFPGIDYFHLEPEME
TEKAYIGGERIALRALELRLTHEYEAFTRGTFLPNQRDPDVLCPPKSLSPDLRFGSLSVRKFYWGVLDAFKKS
QQNTKKPFNPRIISQLLWREFFYTMSVENDYYDEMARNEICVNIPWNPVENNPHWKAFLDARTGYPFIDAGMR
QLYKEGWTHHVVRNAIACFLTRGDLWISWENGLKVFLKYLLDADWSVCTGNWLWISSSSFEEALKSYGIIDPS

LYGRRVDPWGKYVQKYIPELANYPVEYVYEPWLAPKEVQEEAGCIIGKDYPEPIVDHQKASAENTKRMNDLKE
YLTRQLSLEPSPSVSPVEILDFESLAENFGAPEVQCCQECPS
>Tilletia_indica_KAE8226999.1
MGSSKGNVLIALLRNDLRVHDHPIFHVASNSKSGSKAASPLTSSLRFSQPITHFLPIYVLDPVFHPLSGLPNI
PSSVAEDGSPKTRTLGLWRTSPHRLSYLAHSLADLAGTLQKKGSGLHVYAGRPDRIISSLVKRIEADGYRVEG
VAMTKEVNSEELRTQSRVRKALEAASSGKPKVFTTDAKPLVPRSILPFELKELPDVYTQFRKRIEGLRPGEGS
SDYMGAVLEPLPTPESLLPLPGNTAAQSSEDAYLLDGKDSRPSVSDVENLIQCLIKPIIDNPTLGIKKAASLQ
TQAAQNGAEKVGQDGGKTAQVPSAFPFKGGETEALKRLEHYLGGTNAPGVRYKETRNGMLGEDYSTKFAAALA
HGTISPRAIAVRALAMDEATGRGSGGGYWVVFELLWRDYFYFVGARYRTSLFQLDGIAADMPSKAGEAAQKNY
PGANGFKNADTDGWVRSKSLDDKEDAFVRWAEGRTGVPLIDANMRELIQTGFMSNRGRQNVASFLTKDLGYDW
RYGAEYFESLLSDYDTNSNWGNWQYVAGVGNDPRSSRQFNPIKQAHDYDAQGEYVKAWLPELRSVPTYAVQHP
WTLNLTQQGIDIGSYPAQPVVENKGMWGRHYWREGGSGGGGGRGGGGGGGGGYRGRGRGGGGGRGGGGGGGGR
GGRGRGRGRGGD
>Tilletia_ind_KAE8230325.1_CF326_g467
MSPASTARVLYWFRTDLRLHDSPALLKALDLKPGSFFPVWCWDPKYVLSHRVGVNRFRFLLESMEDVSQSISR
INPNSQLLVVRGDPTTLIPTLCKKWGITHLCFEQDLSGYARRRDASIRQLVKEQCKGVEIVEEQGHYLYSIQD
ILEKNGKKPTMSMSALQKVVSKFPDPPRPREAPKTLPDPILPNQSNVKDRISALKALAETLSDIEHYSVDKTS
KPDLNAASAGGDRLPDTEKGRVSCYDTVYGPDGLDSKSKTEERMDELFGVPTLASLGMDPSAAGALPEAVIKG
GETEALRRLDALLKRKEYMATFSKPKTSPAQPADDPATTVMSPYLKFGAISIRRLWYDVQDVIKGYKGKDKTS
IPENYEGQLLFREMYAVGELALGDEYQQIRNNKISRYMDWYLPNVYDSDGKRVEPRPRGDEASERRLELFVQG
QTGFPWIDALVRQLRSTGWIHHLGRHSLAAFLTRGQCWISWERGSELFETWLLDWDPCANPGNLWLSCSAYFS
QYFRVYGTASFPAKYDKSGSLVRRYCPELKDFPDKYIYAPHTAPIAVQKQAKCIIGKDYPHPMLDEKEEKAKC
LARCKAAYGAGFYGTSKEVLEGKAEAILRKAHGQPDPEMPDTGKAVSKGSKIPDWAKPAVGVKDSGAKSKSAP
PKSQKTMSAKAEDHDEDEDDQHPDTADDDDEHVGEKEDGKGRGKRAVEGEGEEDEEDDRTRRSKRQRTSAGRS
AAVSKKK
>Tilletia_ind_KAE8232765.1_CF326_g219
MAPSKKRSIEEEEEESIDPTLAEHPDETEEELQPPKSKKQRKEPTKSSADKDGLDYSDLPKSPPPYGKGNNEM
NPKKKASGTPLEQLRTALAEQKKPKKTSTEKKNVLYWMRNRDLRVEDNRALAEASAFSDRQHLIALHIISPEE
FKAHDRSPRRIDFSLRNLRLIEAQLAKLNIPLVVLTHDGSRTKIAQTVVDLASEWGCGAIFGNLEYEVDELWR
DAEVVRLGSKQDIHVSFLDDAYIVPPGKISSKQDRPYSVFTPFSRTWLDHLSRNMDLMQEYPDPEANDDSIHK
DKTLSSLFSPDSKQYGIPKAVPGFECPDAEHMEYLWPAGHESAARVMTNFINGKGGVKSLDGPATADELEHVG
LKAKNSRLNTYTEGRNLLGELGTSHISPYLAAGIISARECLRRSREATGGKLTMERNSGPGMWCIEMVFRDFY
AHVLAAWPRVCMGRAFVLKYEDVVWETDEATLQAWKEGKTGYPIVDAAQRQCLKQGYCHNRSRMITAMFLTKH
LLHDWREGEKFFMQNFIDGDFASNNGGWQWSASTGSDPQPYFRIFNPISQSEKSDPNGDYIRHWVPELRNIKG
KAIHDPFNRLDKDAFKRLGYPKPIVEHKFARERALRRFKTPGEK
>Timema_gen_CAD7588414.1_unnamed_pr
MAEGTIIHWFRKGLRIHDNPALLAAIEKIYGIHYELRPVFVLDPWIVKKLRVGPNRWRFLQQALTDLDNNLHS
IGSRLYVLRGKPEEVFPRVFSEWRVRKLTFESDTEPYALERDASVEKLARSAGVSVIRKVSHTLYNPESVLCA
NLGKPPLTYQKMLSVLDSLGPPERPVAIPKDLPQACLPSRCAPGDNTLIVPTLEELGVEEDQLGPCLFPGGET
HALQRMRDCLKNKAWVCKFEKPNTSPNSLKPSTTVLSPYLKFGCLSARTFYHQLKKTIGNASHSKPPVSLIGQ
LMWREFYYVVAAGTPNFDKMAGNPVCCQVPWDTNPKYLEAWTKGQTGYPFIDSIMTQLRQEGWIHHLARHAVA
CFLTRGDLWISWEEGQKVFEELLLDADWALNAGNWMWLSASAFFHQFFRVYSPVAFGKKTDKFGDYIKKYLPV
LRKMPPEYIYEPWEAPLSVQQKAGCVVGTDYPRRIVLHEDVYKKNIARMSQAYKDNKT
>Timema_gen_CAD7600321.1_unnamed_pr
MHPYSSTATTHLTPPACVNSSTFPTKVAPSIAEFKFNSKRLKMLSKDKVVPDRATGIVYWMSRDQRVQDNWAL
LYAQGLALKAKLPLHVCFCLVPKFLDATFRHYDFLLRGLEEVAEECKQLSIEFHLLLGRAADVLPEFVSKHDL
GAVVTDFSPLREPRSWVKDVTEGLPKDVPFYQVDAHNIVPVWEASNKLEYAARTIRNKINNKLSEFLSGFPPV
VRHPHSGKLRAQVRAPLTTRRNSRSTLPTKVVRSFEENGGDDDTREEPVDWAEADALLKVDRTVGPVAWAKPG
TTAGLAVLEGFCNKRLRLFSTKRNDPTIDALSNLSPWLHFGQISVQRCVLVVREHKTKYTESVNAFCEEVIVR
RELADNFCFYNDKYDSIEGTNDWARITLNAHRKDKRQYIYSREELSEGLTHDELWNSAQLQLVKDGKMHGFLR
MYWAKKILEWTDTPERALADAIYLNDRYSLDGRDPNGFVGCMWSICGIHDQGWRERDIFGKIRYMNYEGCKRK
FNIAAFVSRWGGKKHKYVAKK
>Timema_gen_CAD7601223.1_unnamed_pr
MALNNSIMSVETKPKHTVHWFRKGLRLHDNPSLREGLIGATTFRCVFILDPWFAGSSNVGINKWRFLLQSLED
LDHSLRKLNSRLFVIRGQPADALPKLFKEWGTTNLTFEEDPEPFGRVRDHNIMAMCRAMSISVISHVSHTLYQ
LERIINKNGGKAPLTYHQFQTVIASMESPPPAEPTVNHKFLKACYTPVQDDHDELYGVPTLEELGFDTQGLRP
PLWQGGESEALARLERHLERKAWVASFGRPKMTPHSLLASQTGLSPYLRFGCLSTRLFYYQLTDLYKKIKKAV
PPLSLHGQLLWREFFYCAATKNPNFDKMLGNPICVQIPWDRNKEALAKWANGQTGYPWIDAIMTQLREEGWIH
HLARHAVACFLTRGDLWVSWEEGQKVFDELLLDADWSVNAGMWMWLSCSSFFQQFFHCYCPVRFGRKADPNGD
YIRKYLPVLKNFPTRYIHEPWNAPEAVQKAAKCIVGKEYSLPMVNHVVVSRINMERMKQVYQHLTKYRDPGAP

DTRPAVLCPDLKNTPRGTDCLGDFEQTPNIETGHIKKEVPSSLSQQAIDHWEEVTPDPAISYMDFVSMDEDVA
VCGEVTDADIVAEVLNNNIQVEDGASGDEEDNSSLVQERSISSAAEATDHIQELGRFFLKSQQCK
>Timema_gen_CAD7604462.1_unnamed_pr
MACCDADGLFTMIEPGHAGRNTDGVHPTEIRTSISPSSAVELNTTGALANYATEAVVVMFSEKGKKVNVHWFR
HGLRLHDNPAFLEALKDGDEIFPIFIFDGETAGTRVVGYNRMNFLLESLHDLDTQLRKFGGQLFLFQGSPVHI
FERLWEEVGLQKICFEQDPEPIWHTRDSSVKQFCEERGIHWVEKVSHTLWEPHQVVKTNGGTPPLTYQMFLHT
VSVIGKPSRPAEDLDFTGVKFGVLPQYLAEEFKCLVSCIPLFWITMFSWTYELTMLLAQIAFISIKHRKNYFW
GDESKCSRNSLTAQDLGLQQDVSASERMVCWVGGETRALENLSDRMQVEEAAFRRGFYLPNQSLPDLLGPPTS
QSAALRFGCLSVRRISAVHFILPLES
>Timema_she_CAD7261249.1_unnamed_pr
MDEKTMALNNSIMSVETKPKHTVHWFRKGLRLHDNPSLREGLIGATTFRCVFILDPWFAGSSNVGINKWRFLL
QSLEDLDHSLRKLNSRLFVIRGQPADALPKLFKEWGTTNLTFEEDPEPFGRVRDKNIMSMCGAMSISVISHVS
HTLYELERIINKNGGKAPLTYHQFQTVIASMECPPPAEPTVNHKFLKACYTPVQDNHDELYGVPTLEELGFDT
QGLRPPLWQGGESEALARLERHLERKAWVASFGRPKMTPHSLLASQTGLSPYLRFGCLSTRLFYYQLTDLYKK
IKKAVPPLSLHGQLLWREFFYCAATKNPNFDKMLGNPICVQIPWDRNKEALAKWANGQTGFPWIDAIMTQLRE
EGWIHHLARHAVACFLTRGDLWVSWEEGQKVFDELLLDADWSVNAGMWMWLSCSSFFQQFFHCYCPVRFGRKA
DPNGDYIRKYLPVLKNFPTRYIHEPWNAPEAVQKAAKCIVGKEYSLPMVNHVVVSRINMERMKQVYQHLAKYR
DPGSCVTRLMSSDNITPGSGNCPVTINLIQQ
>Timema_she_CAD7267052.1_unnamed_pr
MAEGTTIHWFRKGLRIHDNPALLAAIEKIDGIHYELRPVFVLDPWIVKKLRVGPNRWRFLQQALTDLDNNLHS
IGSRLYVLRGKPEEVFPRVFLEWRVRKLTFESDTEPYALERDASVEKLARSAGVSVIQKVSHTLYNPESVIRA
NLGKPPLTYQKMLSVLDSLGTPVCPVATPKDLPQACLPSRCAPGDNTLVVPTLEELGVEEDQLGPCLFPGGET
HALQRMRNCLKNKAWVCKFEKPNTSPNSLEPSTTVLSPYLKFGCLSTRTLYHQLKQTIGNASHSKPPVSLIGQ
LMWREFYYVVAAGTPNFDKMVGNPVCCQVPWDTNPKYLEAWTKLRQEGWIHHLARHAVACFLTRGDLWISWEE
GQKVFEELLLDADWALNAGNWMWLSASAFFHQFFRVYSPVAFGKKTDKFGDYIKKYLPILRKMPPEYIYEPWK
APLSVQQKAGCVVGTDYPRRIVLHEDVYKKNIARMSQAYKDNK
>Timema_she_CAD7267885.1_unnamed_pr
MFAEKGKKVSVHWFRHGLRLHDNPAFLEALKDGDEIFPIFIFDGETAAYSLGTQTVGYNRMNFLLESLHDLDT
QLRTFGGQLFLFQGSPVHIFERLWEEVGLQKVCFEQIHGDSRRARKKSWANAAPSEQGGNPKLLPIPEDQGVN
HVLRHQRIRQCDYSPVVLRVVMPLSELAEQPSVSVEAKESEEDVVMPKATPRTEEWDDSSVTSLESIVVADVQ
QIAHQEEDETETVKELTMFDLMQALRDSSKESMEKMDASMKSLRDGVISILDSHSKEWRSSHDQLKNEVTVIE
VQVQDLTEVVTEVKVQVENQVIQQGQYGQELRTTRSRRDELEERVNSRVEAVSEVVDHLQQDVSQVSGLQLAI
RGQEKITTRNCELTTEQKVVREQLERDLLSNKEEVLALTNDPEPIWHARDTSVKQFCEERGIQWVEKVSHTLW
DPHQVVKTNGGTPPLTYQMFLVRYH
>Timema_she_CAD7269262.1_unnamed_pr
ARRKDKRQYVYSREELSEGLTHDELWNSAQLQLVKDGKMHGFLRMYWAKKILEWTDTPERALADAIYLNDRYS
LDGRDPNGFVGCMWSICGIHDQGWRERDIFGKIRYMNYEGCKRKFDIAAFVSRWGGKKHKYVAKK
>Toxocara_can_KHN85742.1_photolyase
MLVDKVVMVSPRIQPLNRQQAFKGGYVLYFLRCLRVKCSPAFSFASERANAFKVPLIAVHLYIPERHNIPQRV
FLLQGLKELTESMEEIKAKLLCVKAKGYEEALSVAMKLCENACEVIVDAAYLREDREFEERLNDSLIKASRKL
TRVEGNVSVPVNLASNCVEWGARTLRPKVWQHVKAMLAEKWDDVPTTPCDSIKKSVVSGIAEMNLDEELKKAE
ADCKSDSGLTGGEKVAQRMLNFFITNRLVNYHPGRNIPGSKYQSLMSPYLHFGMISSIEIIKRAKSSNAPKHP
IDSFIEELLVRRDLSHNFVYYARDNYDSLDCLPDWAQKTLNEHKKDKREYIYTYEQLEDSRTHDPYWNAAQME
MVHTNKMHGYMRMYWGKKVIEWTPDYETAYRYLIEQNDKHELDGRDPNGFTGVIWNFGMHDRAHAERAVFGKL
RYMNAEGLHRKYKKTIGDYVRYNYKLAGRKIEGPIEPPKKMDKKAKIGKRAREESEEEWSGREEEEPKAGSSR
GKKAASIDKKAKLEVKKGKPTRGGAMSAYLLRAKAKN
>Toxoplasma_gon_CEL73623.1_TPA:_DNA_p
MPHPTQTSSPKAAGQQSMGLKRSSSAGHEQKSQKKTKKAATLPQAPGWIKNGCVDERRVRLLTEGIQKPKADG
KVVICYLQRDMRVQDNWALLLAQEAARSLNKPLVVIHLLVPGLAFQPTRRHLSFFMGGAREVETELKSLNIGF
ELPIVSKKDPKGRLDEANKKIEEVFDALQPALAVCDFNPLRLPTQVVEALTRVYAECLSPLYQVDTHGVVPCW
VASDKLETAARTLRPKLMELVKEFATPFPKVQRMTVPWTHTKLFPLDEDRILSELKPDPPEPLDSWKPGTKAA
LELLHSFATPKNLAEYGKSRNDPVAGVQSDLSPYIHFGHISVQRCLLEVSKLKSTAADKTLKEGVMSFIDEVV
VRSQLSDNFVFFNPHYDDIKGAPLWAQQTLNIHAKDKREPHYGFAALEAGKTYDELWNAAQLQLVRDGKMHGY
LRMYWAKKILEWTKSPQEALKIAIALNDKYHLDGTDPNGVTGCMWSICGVHDQGFKERPVFGKIRYMNYPGCE
RKFDVKAFVRKFPGAAENAVNVAKQGMLPFTGRAKIELKGVAEGGDSDRVASILTKKIEQQKPQNTISGPATT
KETAQTKGSAKKRQRKQSDGDLAPPRNEGVDVEEADINVEEDVHLSE
>Toxotes_jacu__XP_040886430.1
MVINTIHWFRKGLRLHDNPSLKESLQGADTVRCIYILDPWFAGSSNVGINRWRFLLQSLEDLDSSLRKLNSRL
FVIRGQPTDVFPRLFKEWKITRLSYEYDSEPFGKERDAAIKKLASEAGVEVTVRTSHTLYDLDKIIELNGGQS
PLTYKRFQTLISRMDPVEVPVESITAEIMGKCTTPLSEDHDDKFGVPSLEELGFDTEGLSSAVWPGGETEALT

RLERHLERKAWVANFERPRMNANSLLASPTGLSPYLRFGCLSCRLFYFKLTDLYRKVKKNSSPPLSLYGQLLW
REFFYTAATNNPCFDKMESNPICVQIPWDRNPEALAKWAEGRTGFPWIDAIMTQLRQEGWIHHLARHAVACFL
TRGDLWISWEEGMKVFEELLLDADWSVNAGSWMWLSCSSFFQQFFHCYCPVGFGRRTDPNGDYIRRYLPILRG
FPAKYIYDPWNAPESVQKAAKCIIGVHYPKPMVHHAEASRLNIERMKQIYQQLSCYRGLGLLATVPSNSSGNG
NGNNEASSDAMGFHVEATHDAAAASGYQMPVHSQGDWQSGVMMYLQGDPQASISTHQQGYAGTSSSMMCYTQG
SQQIPAPVIQKGPEHHSTTQTSGKRHSEDSGNGKSSKVQRQSSR
>Toxotes_jacu__XP_040893925.1
MVVNSVHWFRKGLRLHDNPALQEALNGADTVRCVYILDPWFAGAANVGINRWRFLLEALEDLDNSLKKLNSRL
FVIRGQPTDVFPRLFKEWKVTRLTFEYDPEPYGKERDGAIIKMAQEFGVETIVRNSHTLYNLDRIIEMNNNNP
PLTFKRFQTIVSRLELPRRPLPPITRQQMDKCQTKIADNHDQLYSIPSLEELGFRTEGLPPAVWRGGESEALD
RLNKHLDKKVWVANFEHPRVNTCSLYASPTGLSPYLRFGCLSCRVLYYNLRELYMKLRKRCSPPLSLFGQLLW
REFFYTAATNNPNFDRMEGNPICVQIPWDQNPEALAKWAEGRTGFPWIDAIMTQLRQEGWIHHLARHAVACFL
TRGDLWISWESGMKVFEELLLDADWSVNAGSWMWLSCSAFFQQFFHCYCPVGFGRRTDPSGDYIRRYIPILKD
YPNRYIYEPWNAPESVQKAANCVVGVDYPKPMINHAEGSRLNIERMKQVYQQLSHYRGLSLLASVPTIQEEAE
PPMTDESQTSSGPDSPPRDPVDSEAAGCSTAPDSSTVCTSATYAPHPAPGDTPNSHPPLAPPCTSSISHTQPS
AAVTSTSASHPPSAAAAASSPASTPIQSPLSRSKPSSPSSSCPTLSPSPSPAATPAQTFPKRKGLGRKVRRSQR
QRGRQSGTPSGREGERRAEEEEREETRGEERMEEDVEQDEERMEEETSGETAGHQQHEGTWWS
>Toxotes_jacu__XP_040894004.1
MIGVLTIVYNKLVFCLKKKKTFYIEYIVTFHLKMPPSAAGAEDSKAFVRKMLREVLVGREDPEGFFAVCVSVL
GHQETRSRFLSLIQPLSTANSSLHSTLTSIYREYFSKTEDDELELALALSVLEMKDHQLSTPSQQSRLQQPGD
SPNQSSCVQLTSVSQPQGSSHTQLADVAGRRKNTHSAQTGPWGMVCPPQTTQETELQKSTHVDKHDKETPGTS
KTVCVSRTYASFSKQGTVKEGEQMIEEGDLNQSEKPKRSKNRRQRRKGSGQQVVGLPCSPSAPAPVLLWFRRD
LRLCDNPALVGSLEVGAPVIPVFIWSPEEEEGPGITVAVGGACKYWLHQALSSFCSSLESIGSHLVFLKANGE
GNRMESSLQTLKELIKETGARTVLANALYEPWLKERDDMVVSALKRDGVEFRMHHSYCLRDPYSVSTEGVGLR
GIGSVSHFMSCCRQNPGSALGVPLDPPVSLPTSAHWPQGVSLDTLGLARMPHRKDGTTIDWAVNIRKSWDFSE
KGAHAKLEAFLHDGVYRYEKESGRADAPNTSCLSPYLHFGQLSPRWLLWDAKGARCRPPKFQRKLAWRDLAYW
QLTLFPDLPWESLRPPYKALRWSSDRGHLKAWQRGRTGYPLVDAAMRQLWLTGWMNNYMRHVVASFLIAYLHL
PWQDGYRWFQDTLVDADVAIDAMMWQNGGMCGLDHWNFVMHPVDAAMTCDPYGSYVRKWCPELVNLPDELIHK
PWKCPASMLRRAGVVFGQTYPERIITDLEERRSQSLQDVALVRKEFGQYVDKRTGCDLVPLPPRLVSEALGLS
HRDGGAVAEGKQFLLPVITRMEFKHQLEDPDADAASNPYNAVLKGYVSRKRDETIAFLNERDFTASVMYEGAQ
RKERLESDYRRMEGLPRPPAPRGRARRTPTAKDRFSIVPGGAVASLR
>Toxotes_jacu__XP_040898972.1
MTKTCIHWFRKGLRLHDNPALMAALRDCKKLYPVFILDPHLHNNTPVGINRLRFLLGALKDLDSSLKKLKSRL
FVVRGKPEDVFPKLFNEWKVTKLTYEYDTEPHGLIRDKKVTALAKDHGVEVIYKISHTLYDIDRVIEENNGKA
PLTYKRMQAILKSLGPPKKPVPAPTMEDMKDVKTPCLENHEKKYGIPTLEELSQDTAALGEELFPGGEQEALR
RLDEHMKRTGWVCSFEKPQTSPNSLSPSTTVLSPYVTHGCLSARTFWWRLTEVYQGKKHSDPPVSLHGQLLWR
EFFYTTSVGIPNFNKMEGNSVCTQVDWDTNSEYLAAWREARTGFPFIDAIMTQLRQEGWIHHLARHAVACFLT
RGDLWISWEEGQKVFEELLLDGDWALNSGNWQWLSASAFFHQFFRVYSPVAFGKKTDKSGDYIRKYLPLLKKF
PAEYIYEPWKAPRSIQQAAGCIVGKDYPRPIVQHEMISKKNIQRMKLAYAKRSPDSTESPSKEQGVKRKAVSI
VDMLTKKQRVK
>Toxotes_jacu__XP_040910374.1
MLLCIWRISSFSSAPKRLLKLGIHSNQRPIPTLSLLIKSFTSETIMSDKKRKAASAPAGKEPSPKQQKLAPVK
EEKKARAAGWLQDLVKQQRPEKKEMKLNKKRLRFVSDTEKIKQGSEGVLYWMSRDQRVQDNWALIQAQRLAMK
ENLPLHICVCLFVQKSELSTQRHYSFMLKGLEEVAKECKTLNIQFHLLHGTAGEVLPGFVSDRSLGAVVTDFT
PLREPLQWLEDVKKKLSKDIPLIQVDAHNIVPCWVASPKLEYSARTIRGKITNLLPEFLTDFPLVEKHPYTAT
RTAKPIDWDKTLASLQVDKAVGETEWAKPGTKAGMAMLESFIDVRLKLFGTQRNDPNAAALSQLSPWIRFGHL
SAQRVALQVQRSGKNAGQSVSSFIEELVVRRELTDNFCFYNKKYDSVEGAYEWAQKTLKDHAKDKRPYLYTRE
QLEKAKTHDKLWNAAQYQMVTEGKMHGFLRMYWAKKILEWTSSPEEALSIALYLNDRYELDGQDPNGFVGCMW
SICGIHDQGWAERPVFGKIRYMNYKGCTRKFDVARFERKYCPKNL
>Toxotes_jacu__XP_040921539.1
MKERTLLSATKRPVCVSRTNTAALSNTKSMSASRTIICLLRNDLRLHDNELFHWAQRNAEYIVPLYCFDPRHY
VGTYNYNLPKTGPFRMRFLLESIRDLRNTLLSKGSNLVVRRGKPEDVVAELIKQLGTVSTVAFHEEVTSEELN
VEKKVKDVCAQMKVKVHTCWGCSLYHRDDLPFHHISRLPDVYTQFRKAVETQSRVRPVFPTPEQLNPLPPGLE
EGAIPTAQDLQQTEPLTDPRSAFPCSGGESQALARLKHYFWDTDAVATYKETRNGLIGVDYSTKFAPWLAMGC
ISPRYIYHQIKQYERERTANQSTYWVVFELLWRDYFKFVGMKYGNRLFQVKGLQDKSVPWKKDMKLFNAWKEG
QTGVPFVDANMRELAMTGFMSNRGRQNVASFLTKDLGLDWRMGAEWFEYLLIDHDVCSNYGNWLYSAGIGNDP
RENRKFNMIKQGLDYDNNGDYVRQWVPELQGIKGADVHTPWTLSAAALSHANVSLGETYPTPIVIAPEWSRHV
NKKSSGAGTSPRGKKGPSNTPKQHRDRGIDFYFSRSKNL
>Trebouxia_sp._KAA6423187.1_DASH_famil

MAQEAIQKSRVIVWFRDDLRLHDNPTVHAAATKVKTKQAAEVVPVYCFDPRHFGTTPYGSKKTGDYRAKFLLE
SVQDLKQNLKSIGSDLLIHMGRPEDALAELANVEQKTTILLQTQITSEELNVEAKVKRAVKGKAQCESVWGHT
LYHPEDVPYKLDMSDLPDTFTPARHKIEQHAQVRAALPTPQQGELPLPEGLPKAMLECNPQAVEDLNAVVPKG
SQQLSTQELNEHAVHRFTGGESEALARLKYYLWDTDLLASYFQTRNGMLGGDYSTKFSPWLAHGNLSARTIYH
EIKKYESERKANKSTYWVIFELLWRDYFRFFTQKHGNKLFAEYGITGKKLRWSEDPQAFQRWKDGQTGLPLVD
ANMRELKQTGFMSNRGRQNVASYLVLDMGVDWRKGADWFETVLQDYDVCSNWGNWVAAAGLTGGRINHFNITK
QSKDYDLEGEYVKTWIPELKDVSSKRIHEPWLMSQSEQQQFGVSIGQDYPAPIPSSQLVRPHGSPEGGRNPGG
GRGRGRGGKRGHGRGGRQNDRHRTQPRGEYERFG
>Trebouxia_sp._KAA6427543.1_CPD_photol
MAGGRKRKEKDMVEHSDSKKSKAEKERASSSSEEEAGPSSAASAEGGIHVHPKRIRELKGGEVKKGPVIYWMS
RDQRSKDNWALLYACEVAQRSGSNVVVAFNLVTEFLGAGARQWGFLVRGLRQVEPKLKELNIPFYMMYGKAED
NVPKLVEDLGAGLLICDFVPVRAAREWREKTRDELSIPMHEVDTHNVVPVWEASDKKETAARTIRKKIHTKLP
MYLEDFPEIPKQAEWRCKRMPDKIDWDGLIADVLQRGTDVPEIPWFKPGEDAAYDALSGKEGFLTPERLKVYN
DMRNDPCSPKAVSTLSAYLHFGHLSGQRVALEASKHRKKYKEAVDGFLEEVVVRRELSDNFCHYEPNYDNIKG
AAQWAQDSLEKHKEDEREYVYSMEQFEKAQTHDDLWNAAQNEMVHLGKMHGFMRMYWAKKIFEWTRTPEDALD
ISIQLNDKWEMDGRDPNGYVGCMWSVCGVHDMGWKERPIFGKIRYMNYNGCKRKFNVQKYIDMVNEKVEELKN
MH
>Trebouxia_sp._KAA6427658.1_6-4_photol
GLRLHDNPALLEALKGCKHLYPVFVFDPWFAGNGKVGTNRFSFLLDSLHDLDASFKARGSRLIIVHGNPPDIV
PKLIKAWNIGKVCFEADTEPYSKARDKNMVEGIEATGVPWHSFVSHTLYDTAKLLQLNKGQVVTAYSAFQRLA
QKSGSPGAPVSDPPSQLPPIPVDAPKALDTVGLTENGVPSLTDIGYHDQPTSPFKGGETLALKRMEEHIADKA
WVVQFEKPKGNPAALEPATTVLSPYLKFGCLSVRVFYKRLQEIEAGAKQASQPPVSLRGQLLWREFFYFVGAN
TPNFDQMEDNPGWMHHLARHAVACFLTRGDLYCPWTAGRDVFDRLLIDDDWSLNNANWMWLSASAFFYQYHRV
YSPITFGRKYDPNGDFVRKFVPELKDFPAKWIYEPWKAPIADQKQANCRIGVDYPKPMVDHASVSKDNILKLK
DAYADQSPSKGKTLPHASSPSGTSPSHTRKKQASRPVGAQQHEDAQAQASTVQAASGEASGMHTKEPRSGFDD
MTLPDDSDHTHVARSDADGAGPSGAPDVRADSNRTGQGANQGLGAVPVMAGPGVFPDGADQDDVEPEATLPGG
TARGGSKSGSNGQTAHASDAKQKKLDWTAATGGHKRKAGSESKSPAK
>Trematomus_bern__XP_033972501.1
MAHTCIHWFRKGLRLHDNPALMAALRDCKELYPVFILDPYLYNNTLVGINRWRFLIGCLKDLDCSLRKLNSRL
FVVRGKPEEVFPKLFEKWKVTKLTYEYDTEPYSLSRDKTVTTLAKEHGVEVVYKISHTIHDMDRIIEENNGKA
PLTYNRFRTILKTIGNPKKPIPAPAIEDTKDVKTPCSENHDKNYGIPTLEDLGHDTTTLGEEKFPGGEQEALR
RLDENMARKDWVCKFEKPQTSPNSLSPSTTVLSPYVTFGCLSARTFWWRVTEVYQGKKHSEPPVSLHGQLLWR
EFYYAAGVGIPNFDKMEGNPVCTQVDWDTNPEYLAAWREARTGLPFIDAIMTQLRQEGWIHHLARHAVACFLT
RGDLWINWEEGQKVFEYLLLDGDWALNAGNWQWLSASAFFHQYFRVYSPVAFGKKTDKNGDYIKKYLPLLKKF
PAQYIYEPWKAPRSVQQAAGCIVGKDYPQPIVQHEVISKKNIQRMKLAYAKRSADSAESPNESPSKKQGVKRK
AKSVIDMLQKKDKK
>Trematomus_bern__XP_033989275.1
MVINTIHWFRKGLRLHDNPSLKDSLLGADTVRCVYILDPWFAGSSNVGINRWRFLLQSLEDLDSSLRKLNSRL
FVIRGQPTDVFPRLFKEWNISRLSFEYDSEPFGKERDAAIKKLACEAGVEVTVRISHTLYDLDKIIELNGGQS
PLTYKRFQTLISRMDAVEEPADSITADIMGKCRTPLSENHDDKFGVPSLEELGFDTEGLSSAVWPGGETEALT
RLERHLERKAWVANFERPRMNANSLLASPTGLSPYLRFGCLSCRLFYFKLTDLYKKVKKNSSPPLSLYGQLLW
REFFYTAATNNPCFDTMESNPICVQIPWDRNPEALAKWAEGRTGFPWIDAIMTQLRQEGWIHHLARHAIACFL
TRGDLWISWEEGMKVFEELLLDADWSVNAGSWMWLSCSSFFQQFFHCYCPVGFGQRTDPNGDYIRRYLPILRD
FPAKYIYDPWNAPEGVQKAAKCIIGVHYPKPMVNHAEASRLNIERMKQIYQQLSCYRGLGLLASVPSISNGNG
ETSSEGMGFSADGTHNAAAAPSGPEHHVSTLSGGKRPCEDPRNGRGSKIQRQSTH
>Trematomus_bern__XP_033993791.1
MLYSICRSSSFLRQYLKVVAQSDQGPTSLFSTKAKAFTSQAIMSEKKRKSTSAAAAVKEPSAKQQKLDPTKEK
GWLQDSLKQQRTKNKELKFNRQRLRYTSNTERIKQGSEGVLYWMSRDHRVQDNWALIHAQQLALKEKLPLHVC
FCLFVPKSLLSTLRHYSFMLKGLKEVEKECKALDIQFHLLHGSAGDVLPGFVSDRELGAVVTDFSPLREPLQW
LEDVKKTLPKDIPLIQVDAHNVVPCWVASPKLEYAARTIRGKITKLLPDFLTDLPLVEKHPYTAARTAKKVDW
EKTLASLQVDRTIEEPEWAKPGTKGGVAMLESFIDERLKLFATQRNDPNAAALSQLSPWIRFGQLSAQRVALQ
VQHCGSSAGPAVASFIEELVVRRELTDNFCFYNEKYDCVEGAYEWAQKTLKDHAEDKREYLYTREQLEKAKTH
DKLWNASQYQMITEGKMHGFLRMYWAKKILEWTSSPEEALSIALYLNDRYSLDGQDPNGFVGCMWSICGTHDQ
GWKEREVFGKIRFMNYKGCQRKFDVAKFERKYCPKNL
>Trematomus_bern__XP_033997855.1
MAPSAAASEDRKALVRRMLIEVLMGREDPEGFFAMCVSALGPQETRSQFLALIQPLSTANSSLHSGLTSIYEQ
YFSKTEDDELQLALALSLLEVKDDQLSSPSQDSPLQPPRDRPNQRSPAQKSTVSQPQGNSHTKPADGVGRREH
TITTQTEARVKASPLQTFRETELQKNNVDKYKMGTPGTSLAGCVSSPSGLFLKQNTIEKDNQMMNEGDLNQPE
TPKCSKNRRQRRKGAGQKIVGLPCSPSAQPPVLLWFRRDLRLGDNPALIGSLEVGSPVIPVFIWSPEEEEGPG
ITVAMGGACKYWLHQALSCFSSSLERIGSHLVFLKANGSSLCTLKELVRETGARTVLANALYEPWLKERDDDV

ESALQKDGVECKMYHSYCLRDPYSVSTEGVGLRGIGSVSHFMNCCRQNPGSALGAPLDPPVSLPTPAYWPQGV
SLDVLGLGRMPRRKDGTTIDWAANIRKSWDFSEDGACARLEAFLNDGVYRYEKESGRADAPNTSCLSPYLHFG
QLSPRWLLWDAKGARCRPPKFQRKLAWRDLAYWQLTLFPDLPWESLRPPYKALRWSSDRDHLKAWQRGRTGYP
LVDAAMRQLWLTGWMNNYMRHVVASFLIAYLYLPWQEGYRWFQDTLVDADVAIDAMMWQNGGMCGLDHWNFVM
HPVDAAMTCDPYGSYVRKWCSELAELPDELVHKPWKCPAPMLRRAGLVFGQTYPERIVTDLEERRSRSLQDVA
LVRKEFGQYVDKRSGCDLVPLPQRLVSEALGLSHRDGGVVTEGQQFLLPVITRMEFKHQLEDPDADAASNPYN
AVLKGYVSRKRDETIAFLNERDFTASVMYEGVQRKERLESDYRRIEGLPGLPAPRGRARRTPTAKDRFSVVSG
GAVSSLR
>Trematomus_bern__XP_034001951.1
MSTSRTIICLLRNDLRLHDNELFHWAQRNADHIVPLYCFDPRHYVGTYNYNLPKTGPFRLRFLFDSIRDLRHT
LLHKGSNLVVRRGKPEEVVADLIKQLGSVSTVVFHEEVSSEELNVEKRVKDVCAQMKVKVHTCWGSTLYHRDD
LPFNNISRLPDVYTQFRKAVESQSQVRPLIPTPEQLKPLPQGLEEGGIPTAEDLEQTEPVTDPRSAFPCTGGE
SQALARLKHYFWDTNAVATYKETRNGLIGVDYSTKFSSWLALGCISPRYIYHQIQQYEKERTANQSTYWVIFE
LLWRDYFKLVGVKYGNRLFQIKGLQEKSVPWKKDMKLFDAWKEGRTGVPFVDANMRELAMTGFMSNRGRQNVA
SFLTKDLCLDWRMGAEWFEYLLIDHDVCSNYGNWLYSAGIGNDPRENRKFNMIKQGLDYDNNGDYVRQWVPEL
QGIMGSDVHTPWTLSTASLSHADVSLGETYPTPIVMAPEWSRHANKKPSGAGSSARGKKGPSHTPKQHRDRGI
DFYFSKSKNL
>Tremella_mesent_XP_007007703.1
KVDEDPPLDQLMRLLQHGGKEVPNGESVVYWMRMEDVRIDDNTALYKASQKAQQLHLPLVVLNILSPGEWKIH
DRSSKKIDFLLRNLRDLKPKFDALNIPLYMTSFDRRLQIPRRVITDLMPLFKSHHLFVNLEYEVDELRRDIAT
VKFGQEKGVKVECFHDRLVVPPGRITSQAGKPMSVFSPWQRQWAKLLEKEPNLLEVSPLPQGNDPSVRQGPLK
DLFDATSVPEDIKDFVPEDKHLLKECWPEGTDAARQLLDRFLHTKARKKQFQLVSPLTEGAEEDDKHSRVREY
QDGRNLVDGDNSSRLSPYLALGVISGRMVLNEAAKLGKGKRLESGRDTGVGMWVQEVSWRDFYNHVMARCPRV
SMGRPFLEKFADVQWEINEENLQAWKDGKTGYPIVDAAMRACKARGWMENRVRMVSACFLVKSLLLDWRLGEK
YFMESFIDGDLAANNGGWQWTASTGTDPQPYFRIFNTLTQSQKCDPNGDYIRYWVPELKNLKGKAVHDPFHSM
PRNEFNKLGYPIPIVDHKQSRERALFRYKNVGQKEE
>Tremella_mes_RXK37313.1_M231_05455
MSKPSRVLYWFRTDLRITDSPALITALSLPDIKAFYPIWCWDPSYIYGHRVGLNRWSFLLESMKDLSDQLTSL
NPRQKLHVCRGSPEEVLPVLWREWGITHIVWEKDANAYAKIRDECIKRLAKEKGVVVVDVPGRHLFDPDLVGR
MNGGKPTMTLQQWQNITKKIGKVEQPSLTPTILPDPGDTTLRYSRKEYKWDKVDMNAHLRTGKDTCFDHFTGP
KGDFSVPTLSQMGYPPSTTSIRGGTTQAHLRLNTFLSHPQAVATFSKPHTAPTALEPSTTLISPYLKFGCVGV
REIWWGCKRVVEAYKGEKTKEPENMFGQLEFRDMYACAEALQPHFERIRGNSVCRYIDWGLQNQYDERGIEIL
PRPRGEEEDERRFEAWREGCTGFPWIDACMRQLKSEGWIHHLARHSVACFLTRGQCYISWERGMEVFDEWLID
WDPASNPGNWMWLSCSAFFSQYYRVYGLVSWPQTTDKTGALVRKYCPELEKFPDKYIYAPHTAPLSVQEAAGC
VIGQDYPLPILDERAEKDRCLARIKNAYQLGFRGADPEVLSGEADKRLKELHEKSGASTVKSEKERDEEKGRL
KREREGDGTLDGYVKRQK
>Tremella_mes_RXK40197.1_M231_02471
MSRPNTFIHLHTLDLRTHDLPSLHLSHSPRSSISKRVTHFLPVYIIDSRQLDISHLPHALPSLPPRLPNANSP
AEDGSGHGEQDKGDNNTRPSPRSRVANFHRTSPYRLRFLLESIFYLRDTYRRAGGDLLIGYGRADRLLPVLVQ
KLKETSEVEGVWAQDEVTVEEKDMLANLEVVLQKIGVKLNKVDSKTLLAIKDLPFDRRQTPDVYTAFRKNCEG
LGLSLGGMLKQPLRTSHLSQGNVKIDIDGQGLKPFPQMDFSTIPEGCGVLEKDTSVGEVYTELVKPLLDSPPV
GGWSRVAEEKDALPVFHPSSAVPFEGGEKAALERLDDYVGHEDGKGWIGGGKAKQYKATRNGMIGEGFSTKFS
CWLSLGCLSAREIGWRVGGLLGKVDRKDKDTYNNVYWIIFELLWRDFFQSTVYKYSLSSKYSLFLPKGFSEQI
NTYPRSQRPNPQEWHYPNWENEDDPARRWCEGRTGVPFVDANMRELRETGWMSNRGRQNVASFLVKDLYVDWR
VGAEFFEMHLVDYDTCSNWGNWQYQAGVGNDPRSSRQFNPLKQAKDYDPKNDYTKLWLSELKVLPPTFVQTPW
LLDDNQKPKGYPVKPVVEQQTWHKFYPRGSHTSTHRHGENTGQHDRGGGHGHVRKEGRNGRDGHEDKRAHGRS
DQGL
>Tribolium_cas_NP_001076794.1_cry_2
MSGVVGDAGRASKGQDKHMVHWFRRGLRLHDNPSLREGLKGARTFRCVFVLDPWFAGSSNVGINKWRFLLQCL
EDLDRSLRKLNSRLFVIRGQPADALPKLFKEWGTTALTFEEDPEPFGGVRDHNLTTLCQELGISVVQKVSHTL
YHLQDIIDRNGGRAPLTYHQFLAIIACMGPPPQPEPPVTFNSLNGAHTPLTDDHDEKYGVPTLEELGFDTEGR
LPPVWQGGESEALARLERHLERKAWVASFGRPKMTPQSLLPSQTGLSPYLRFGCLSTRLFYYQLTDLYKKIKK
AFPPLSLHGQLLWREFFYCAATKNPNFDKMIGNPICVQIPWDKNAEALAKWANGQTGFPWIDAIMTQLREEGW
IHHLARHAVACFLTRGDLWLSWEEGMKVFEELLLDADWSVNAGMWMWLSCSSFFQQFFHCYCPIKFGRKADPN
GDYIRKYLPVLKNMPSQYIHEPWTAPEQVQRAAKCIIGKDYSLPMVNHAAASRVNIQRMKQVYQQLSNYRNVD
NSAKFKDGFQEQPNVVTVGNPSRK
>Trichechus_manatus_latirostris_XP_004387710.1_cryptochro
MAATVVTAAAAALAPAPSIDGASSVHWFRKGLRLHDNPALLAAVRGARCVRCVYILDPWFAASSSVGINRWRF
LLQSLEDLDRSLRKLNSRLFVVRGQPADVFPRLFKEWGVTRLTFEYDSEPFGKERDAAIMKMAKEAGVEVVTE
NSHTLYDLDRIIELNGQKPPLTYKRFQAIISRMELPKKPVGSVTSQQMESCRAEIQENHDETYGVPSLEELGF

PTEGLGPAVWQGGETEAMARLDKHLERKAWVANYERPRMNANSLLASPTGLSPYLRFGCLSCRLFYYRLWDLY
KKVKRNSTPPLSLFGQLLWREFFYTAATNNPRFDRMEGNPICIQIPWDRNPEALAKWAEGKTGFPWIDAIMTQ
LRQEGWIHHLARHAVACFLTRGDLWVSWESGVRVFDELLLDADFSVNAGSWMWLSCSAFFQQFFHCYCPVGFG
RRTDPSGDYIRRYLPKLKGFPSRYIYEPWNAPESIQKAAKCIIGVDYPRPIVNHAETSRLNIERMKQIYQQLS
RYRGLCLLASVPSCAEDLSNPVAEPSLSQAGSMSSAGPRPLPSGPASPKRKLEAAEEPPGEELSKRARVAKLP
SPELPSKDV
>Trichechus_man_XP_004373851.1_cry-1
MGVNAVHWFRKGLRLHDNPALKECIQGADTIRCVYILDPWFAGSSNVGINRWRFLLQCLEDLDANLRKLNSRL
FVIRGQPADVFPRLFKEWDITKLSIEYDSEPFGKERDAAIKKLATEAGVEVIVRISHTLYDLDKIIELNGGQP
PLTYKRFQTLISKMEPLEIPEETITSEVIEKCTTPLSDDHDEKYGVPSLEELGFDTDGLPSAVWPGGETEALT
RLERHLERKAWVANFERPRMNANSLLASPTGLSPYLRFGCLSCRLFYFKLTDLYKKVKKNSSPPLSLYGQLLW
REFFYTAATNNPRFDKMEGNPICVQIPWDKNPEALAKWAEGRTGFPWIDAIMTQLRQEGWIHHLARHAVACFL
TRGDLWISWEEGMKVFEELLLDADWSINAGSWMWLSCSSFFQQFFHCYCPVGFGRRTDPNGDYIRRYLPVLRG
FPAKYIYDPWNAPEGIQKVAKCSIGVNYPKPMVNHAEASRLNIERMKQIYQQLSRYRGLGLLASVPSNPNGNG
GLMGYSPGENIPGCSSNGSCPQGNGILHYAHRDSQQAHLLKQGRSPTGTGVSSGKRPSQEEETQSIGPKVQRQ
SAN
>Trichinella_nati__KRZ50806.1
MAESNVKTAIRQQQSMELYEKSIHLLNAKACNKRGKYVLYWVQASPRVKFNVALELACQKANEHQVPVLACFV
FPTDKRLDNFPPETERRCVFMLDGLRDFEKRLKKERNIDLVVLYGKCEILIAKLAENCCEVVVDAGYMRYQNK
WYDEVNNNIECRFTKVETNVVVPVELVSEKEEFAAYTIRPKIWKYAKQYLVEPECIMVIAKNKPLFSDNLPLI
DLSQSDNVILAKLNADTTVKKVANIHGGHSEAVKLLENFVSKKLPNYDKLRNIPGLDGQSGLSPYINFGHIST
VEIILKVNASKANKSVKDAFVEQLLVRRELSINFVHYNSENYDSLKCLPKWAAETLKLQAKAKREYIYTSEQL
EKGETHDQYWNACQLELVYEGKMNGYMRMYWGKKVIEWTANVNTAYSWLMYMNDKYELDGRDPNGFAGVAWVF
GKHDRAHSSRQIFGTLRYMNSSGLERKFKPHLKDYVKHCYELAGRSFPSSPVENSTPVTKKRKLLKTTANVSR
KVK
>Trichinella_spi_XP_003381336.1_photolyase
MAESNVKTATRQQQSMELYEKSIHLLNAKACNERGKYVLYWVQTSPRVKFNVALELACQKANEHQVPVLACFV
FPTDKRLDNFPPETERRCVFMLDGLRDFEKRLKKERNIDLVVLYGKCEILIAKLAENCCEVVVDAGYMRYQNK
YDEVNNNIECRFTKVETNVVVPVELVSEKEEFAAYTIRPKIWKYAKQYLVEPECIMVIAKNKPLFSDNLPLID
LSQSDNVILAKLNADTTVKKVANIHGGHSEAVKLLDNFVSKKLPNYDKLRNIPGLDGQSGLSPYINFGHISTV
EIILKVNASKANKSVKDAFVEQLLVRRELSINFVHYNSENYDSLKCLPKWAADTLKLQAKAKREYIYTSEQLE
KGETHDQYWNACQLELVYEGKMNGYMRMYWGKKVIEWTANHTRKVMNNCAFSMNDKYELDGRDPNGFAGVAWV
FGKHDRAHSRRQIFGTLRYMNSSGLERKFKPHLKDYVKHCYELAGRSFPSSPVENSTPVAKKRKLLKTTANVS
RKVK
>Trichobilharzia_reg_VDP97938.1_unnamed_pr
MVPAYQADTLRHFKFMIGGLAEVEQECLSLNIPFHLLNAAEHGVNIEDVSSGCKFRWSDENALSNQTERIQTE
FLYDENVATAVVNLVKSLNIGCLITDFCPLRAPRSWIDAHNIVPVWCGSDKREYSARTIRTKLFAQSSVYLTE
FPPIIKHPYTSENTPNPPPIIDWETVLSKYVGDKSVKPVDWAIPGTQAGFNVLYKFIEKRLEKFDPHRSNVAN
PALSGLSPWLHFGQISPQRAVLEVVAVQKQYGRSADIFIEECFNRRELADNFCFYTPMYDSIQDKRDPAYTKL
QMENAQTADDLWNAAQRQLVRKGKMHWFLRQYWAKKILEWCAEGPEAAIQIAVYLNDRYSLDGTDPNGYVGIM
WAICGIHDQGWSERPIFGKIRYMNYKGASRKFSVPTFVARYPKNSVD
>Tricholaema_leuc__NXX38520.1
DNWAFLYAQRLALKQELPLHVCFCLVPEFLGATIRHYSFMLKGLQEVAKECAELNVPFHLLLGYAKDVLPAFV
VEHGVGGLVTDFCPLRLPRQWVEDVKERLPEDVPFAQVDAHNIVPCWVASPKQEYSARTIRGKIHAQLPEFLT
EFPPIIRHPYTSSCLAQPIAWEVCYSSLQVDHTVKEVEWATPGTAAGLAMLQSFIAERLKSFGSHRNDPNKAA
LSNLSPWFHFGQVSTQRAILEVQKHRDKYKDSVDVFVEEAVVRRELAENFCYYNENYDSVQGSAYDWAQTTLK
LHVKDKRPFLYKLEELEHGTTHDPLWNAAQLQMVREGKMHGFLRMYWAKKILEWTRSPEEALQFAIYLNDRYE
LDGRDPNGYVGCLWSICGIHDQGWPERAIFGKIRYMNYAGCKRKFDVGQFERRYAPCT
>Tricholaema_leuc__NXX40960.1
MGVNAVHWFRKGLRLHDNPALRECIQGADTVRCVYILDPWFAGSSNVGINRWRFLLQCLEDLDANLRKLNSRL
FVIRGQPADVFPRLFKEWNIAKLSIEYDSEPFGKERDAAIKKLATEAGVEVIVRISHTLYDLDKIIELNGGQP
PLTYKRFQTLISRMEPLEMPVETITPEVMAKCSTPVSDDHDEKYGVPSLEELGFDTDGLPSAVWPGGETEALT
RLERHLERKAWVANFERPRMNANSLLASPTGLSPYLRFGCLSCRLFYFKLTDLYKKVKKNSSPPLSLYGQLLW
REFFYTAATNNPRFDKMEGNPICVQIPWDKNPEALAKWAEGRTGFPWIDAIMTQLRQEGWIHHLARHAVACFL
TRGDLWISWEEGMKVFEELLLDADWSVNAGSWMWLSCSSFFQQFFHCYCPVGFGRRTDPNGDYIRRYLPVLRG
FPAKYIYDPWNAPESVQKAAKCIIGVNYPKPMVNHAEASRLNIERMKQIYQQLSRYRGLGLLATVPSNPNGNG
NGGLMSYSPGESISGCGSTGVFFFFFFLSASPGAQLGTGDGVQPCALGDSHTGASGIQQQGYCQASSILHYAH
GDNQQPHLLQAGRPALGTGISAGKRPNPEEETQSVGPKVQRQSTN
>Tricholaema_leuc__NXX45725.1

MSHHTIHLFRKGLRLHDHPTLLAALRSSRRLYPVYILDLEFLSSAMRMGPLRWHFLLQSLEDLHRSLCQLGSC
LLVIQGPYEAVLRDRVRRWSITQVTLDAEVEPFYREMEANIRCLGQELGFEVLSLVGHSLYDTKRVLDLNGGE
PPLTYKRFLHILSLLGDPELPVPSITAEDFQSCQPPGPGLAERFRVPLPADLEMPPESLSPWRGGETEGLQRL
EQHLTDQGWVTTFTKPRTVPNSLLPSTTGLSPYFSLGCLSVRTFFHRLAKICAQAKHHSLPPVSLQGQLLWRE
FFYTVASATPNFSSMAGNPLCLQISWAQDAERLHRWKTAQTGFPWIDAIMTQLRQEGWIHHLARHAAACFLTR
GDLWISWEEGMKVFEELLLDADYSINAGNWLWLSASAFCHRYRRILCPVRFGRRTDPEGHYIRKYLPILKNFG
SRHIYEPWTASEEEQKQAGCIIGRDYPFPMVNHEEVSDHNLQLMQQVREEQHRTAQLTRDDTEDSVELKVRPE
HSEEDLPRGKVARVAE
>Tricholaema_leuc__NXX45837.1
KVDYDVCSNYGNWLYSAGLGNDPRDSRKFNMIKQGLDYDGNGDYVRLWVPELQGIKGADIHTPWVLSSADLSQ
AGVTLGETYPQPVVTPPEWSRHISQRPQGRSPHPRGRRGPTRRPVQHKDRGIDFYFPHKKDV
>Tricholaema_leuc__NXX46007.1
RFLLQSLEDLDNSLRKLNSRLFVVRGQPTDVFPRLFKEWGVTRLTFEYDSEPFGKERDAAIVKLAKEAGVEVV
IENSHTLYDLDRIIELNGHKPPLTYKRFQAIISRMELPKKPVSTVISQQMETCKVDIQENHDDVYGVPSLEEL
GFPTDGLAPAVWQGGETEALARLDKHLERKAWVANYERPRMNANSLLASPTGLSPYLRFGCLSCRLFYYRLWE
LYKKVKRNSTPPLSLYGQLLWREFFYTAATNNPKFDRMEGNPICIQIPWDRNPEALAKWAEGKTGFPWIDAIM
TQLRQEGWIHHLARHAVACFLTRGDLWISWESGVRVFDELLLDADFSVNAGSWMWLSCSAFFQQFFHCYCPVG
FGRRTDPSGDYVKRYLPKLKGFPSRYIYEPWNAPESVQKAAKCIIGVDYPKPMVNHAETSRLNIERMKQIYQQ
LSRYRGLCLLASVPSCVEDLSGPVTDSASGQGCSTSTATRLSQADQASPKRKHEGAEELCAEELYKRPKVTGP
PAAEVPGKSL
>Trichoplax_adh_XP_002108560.1
MVCTVVWLRYDLRLIDNPALFHAAKRGHVVIIYILDQETPGKWKLGKASLWWLHHSLKSLQNDLLKFNIPLIL
RRGADPLTILRQVLLQSRADAVYWNRCYEPYAVKRDQSIEEALKVDDVTVGSFQAGLLYEPWAVKSAKDEPFK
LYIMYWNRCLSSDQPRKPYDKPRFNNTLDIKVQTDTLDSWHLVSAKKDWKDELKIVIGCPGEDGAIRKLKEFI
KYKLVDYRKGKETVWPSKTSQLSTHLHFGEISPFVVWHTAKESVHLRSVPSDSSQKFLTELGWRDFCFHLLHY
YPDFPEKSYKLNFDETIWKVDKEKLKLWQQGKTGYPIVDAGMRELLNTGVISYRVRTIVASFLTKHLLIPWQN
GAEWFWDTLVDADLALNSCSWQRITGCGADISSYFYIINPVTQGERFDSDGNYVRKWIPEIAKLSNDYIQQPW
EAPAAVLKKAGIALGKTYPKCIVNHKKARDVALTKFAPLKRQEPMDNLKKLPNKKKMKK
>Trichosurus_vulp__XP_036603607.1
MAPKKRTHSASGEPEKMESQESKAKRKPLQRHQFSKSNMVQKEEDKREGEKRGGAEGLQEAVRQSRLQTAPSV
LEFRFNKQRVRLISQDCHLQDRSQAFVYWMSRDQRVQDNWAFLYAQRLALKQKLPLHVCFCLAPCFLGATIRH
YDFMLRGLEEVAEECEKLCIPFHLLLGLPKDVLPAFVQTHSIGGIVTDFSPLLHHTQWVKDVQDALPKQVPFV
QVDAHNIVPCWVASDKQEYGARTIRHKIHDRLPHFLTEFPPVICHPYPSNIQAEPVDWNGCRAGLQVDRSVKE
VSWAKPGTASGLTMLQSFIAERLPYFGSDRNNPNKDALSNLSPWFHFGQVSVQRAILEVQKHRSRYPDSVTNF
VEEAVVRRELADNFCFYNKNYDKLEGAYDWAQTTLRLHAKDKRPHLYSLEQLESGKTHDPLWNAAQMQMVKEG
KMHGFLRMYWAKKILEWTRSPEEALEFAIYLNDRFQLDGRDPNGYVGCMWSICGIHDQGWAEREIFGKIRYMN
YAGCKRKFDVAEFERKYSPAD
>Trichosurus_vulp__XP_036617379.1
MGVNAVHWFRKGLRLHDNPALKECIEGADTVRCVYILDPWFAGSSNVGINRWRFLLQCLEDLDANLRKLNSRL
FVIRGQPADVFPRLFKEWNIAKLSIEYDSEPFGKERDAAIKKLASEAGVEVIVRISHTLYDLDKIIELNGGQP
PLTYKRFQTLISKMEPLATPVDTITPEVMHKCVTPVSDEHDEKYGVPSLEELGFDTDGLPSAVWPGGETEALT
RLERHLERKAWVANFERPRMNANSLLASPTGLSPYLRFGCLSCRLFYFKLTDLYKKVKKNSSPPLSLYGQLLW
REFFYTAATNNPRFDKMEGNPICVQIPWDRNPEALAKWAEGRTGFPWIDAIMTQLRQEGWIHHLARHAVACFL
TRGDLWISWEEGMKVFEELLLDADWSINAGSWMWLSCSSFFQQFFHCYCPVGFGRRTDPNGDYIRRYLPVLRG
FPAKYIYDPWNAPESVQKVAKCLIGVNYPKPMVNHAEASRLNIERMKQIYQQLSRYRGLGLLASVPSNPNGNG
GLMGYAPGENIPACSSSGGGAPAGVSDGQILQACALPEPPTGASGVQQPGRSSLSGGGKRHRQEEEAQSIGPK
MQRQSVN
>Trichosurus_vulp__XP_036620822.1
MAATTVVTVPAAAAATAPAPAPAPTPASAPEEGASSVHWFRKGLRLHDNPALQAALRGARCVRCVYILDPWFA
ASSSVGINRWRFLLQSLEDLDISLRKLNSRLFVVRGQPTDVFPRLFKEWGVTRLTFEYDSEPFGKERDAAIVK
MAKEAGVEVVTENSHTLYDLDRIIELNGQKPPLTYKRFQAIISRMELPKKPVSCVTSQQMERCQAEIQENHDD
TYGVPSLEELGFPTDGLGPAVWQGGEMEALARLDKHLERKAWVANYERPRMNATSLLASPTGLSPYLRFGCLS
CRLFYYRLWELYKKVKRNNTPPLSLYGQLLWREFFYTAATNNPKFDRMEGNPICIQIPWDRNPEALAKWAEGK
TGFPWIDAIMTQLRQEGWIHHLARHAVACFLTRGDLWVSWESGVRVFDELLLDADFSVNAGSWMWLSCSAFFQ
QFFHCYCPVGFGRRTDPSGDYVRRYLPQLKGFPSRYIYEPWNAPESVQKAAKCIIGVDYPKPIVNHAETSRLN
IERMKQIYQQLSRYRGLCLLASVPSCMEDLSNPMAETSVGQASGVSTGPKPLPCNPASPKRKLEAMEEASGEE
HSKRARVMAALPVPELSGKV
>Trichuris_tri_CDW57984.1_photolyase
MITHLVWFRQDLRLHDNLALAAACRNSSARVLALYIATPRQWATHNMSPRQAELINAQLNGLQIALAEKGIPL
LFREVDDFVASVEIVKQVCAENSVTHLFYNYQYEVNERARDVEVERALRNLVCEGFDDSVILPPGAVMTGNHE

MYKVFTPFKNAWLKRLREGMPECVAAPKVRSSGSIEPAPSITLNYPRQSFDTAHFPVEEKAAIAQLRQFCQNG
AGEYEQQRDFPAVEGTSRLSASLATGGLSPRQCLHRLLAEQPQALDGGAGSVWLSELIWREFYRHLMTYYPSL
CKHCPFIAWTDRVQWQSNPAHLQAWQEGKTGYPIVDAAMRQLNSTGWMHNRLRMITASFLVKDLLIDWREGER
YFMSQLIDGDLAANNGGWQWAASTGTDAAPYFRIFNPTTQGEKFDREGEFIRRWLPELRDVPGKVVHEPWKWA
QKAVRPR
>Trinorchestia_long__KAF2350589.1
MSKSSLSESQVLTHNVFSSRFLLQCLEDVDNSLRNLNSRLFVVRGQPANALPQLFKEWNTTVLSFEEDPEPFG
RARDASIIGIAQEMGIEVIVRTSHTLYKLDKIIKKKGGKPPLTYKTFQNILAMMDPPAPPVAVIEASDLKNAY
TPLQHDHDDKYGVPNLEHLGFETEHLPPAVWKGGETEALSRLKHHLERKAWVASFGRPKMSPQSLFACPTGLS
PYLRFGCLSARKFYTELNELYTKIKRVPAPVSLHGHLLWREFFYTAATNNPKFDHMKGNPICVQIPWDKNPEA
LAKWAHGQTGFPWIDAIMTQLRTEGWIHNVARHAVACFLTRGDLWVSWEEGMKVFDELLLDADWSVNAGSWMW
LSCSSFFQQFFHCYCPVRYGRKADPNGDFIRTYLPVLKNFPTKYIHEPWMAPESVQCNARCIIGQHYPLPMVD
HRTQSQNNIERMKQVYQQLAHYRANNLALSPKCGASIRCTTLLETNTRCTALLDTNTRCTALLDTNTRCTALL
DTNTRCTALLEASTRCTALLEASTRCTALLEASTRCTALLKASTRCTALLDTNIRCTALLNTNTRCTALLKAT
AKTSNISSRDNVVHNASRNAKYTTSLFLYN
>Trinorchestia_long__KAF2359012.1
MCRTQQTLLKPITSSAIKELETKAAPRLTANEKLHNPHSGVHKTLLERSAKLTTEDPNVQLNSVRGILNTPAH
TWPTALATPASNKRGETTNNEEDCQSPAEVLRSTVTWPVQDTKAKHHLAYTNLFKLNVHISSYDLIPITPSTI
SSYSNNQKPLNTLLVGDNILSTVKRNDIDTICRIQTISSALDRKMAVSMKKSGLQLMEEVHKNRIKTAESIAD
FKFCKQRVQVRSKSLDVPEKFSSIVYWMSREQRVQDNWALLFAQRLALKNRVPLKVVFCLVPSFMDATLRHYT
FMIQGLKEVESDCKELGIPFHLLLGQAKDVLPSFVTSNDVGGVVTDYSPLKTPKQWVADVLSNIPEDIPLCQV
DGHNVVPVWEASPKQEYAARTIRRKITDKLSKYLTEFPPLIPHPHKDDKDKNKATDWKAVEASLDVDTSVGPV
KWACGGTRSAFNMLASFCSERLKKFSEKRNDPNEDALSNLSPWIHFGHISAARCVLEVQKQGKTSSSSVAAYV
EECVIRRELSDNFCHYQPHYDSLLGTSDWARITLDEHRKDKREKLYSLAQLDRAKTYDDLWNAAQVQLVTEGK
MHGFLRMYWAKKILEWTPDPDSALAYALYLNDRYSLDGRDPNGFVGCLWSVGGLHDQGWRERAVFGKIRYMNY
AGCNRKMNIKAFVAKYKVTDDMPGATNVKTSLKVVSTANTVSKKKPDELKGKEEESEDAEEIQESPKKRAKKG
RKMVSSASNDCK
>Tritonia_tet_AWY10934.1_non-photor
MKEHNNITEDKIPQKKHVLFWFRKGLRLHDNPALFNAIEGALTYRCVYILDPWFAGASQVGINKWRFLLESLE
DLDSSLRKLNSRLFVARGQPADVLPRLFQEWGITTLAFEEDSEPYGKERDAAISTMAREFHIQVISKSSHTLY
NPRTVIAANGNNPPLTYKRFQSILSTLDPPDQPYETVSSHVVKKSATPVADDHDDRFSVPTLDELGFDTDSLG
PAKFQGGESEALARLHRHLERKAWVASFERPKMSPQSLYPSGTGLSPYLRFGCLSPKTFYWKLTELYKKVKKG
IDPPLALHGQLLWREFFYTVSTNNPKFDRMVGNTICVQIPWEHNPEALAKWAEGMTGFPWIDAIMVQLRKEGW
IHHLARHAVACFLTRGDLWISWEEGMKVFDEMLLDADWSVNAGMWMWLSCSAFFQQFFHCYCPVGFGKRADPS
GDFVRQYLPVLKNMPTQYIYEPWTAPESVQKAAKCIVGKDYPLPMVAHAEQSRVNLERMRHVYKRLVLKSTAS
KQIFLDLPKIPKVGMILSKAKMFLSSSSDEEGSAKLRRQRKQLDSSAA
>Tritonia_tet_AWY11208.1_photorecep
MDNNDTSNHVTIHWFRHGLRLHDNPALLEGLENCQEFYPVFIFDGSVAGTKNAGFSRMQFLLETLKDLDNNLK
AKGSRLYVFKGDPEQVFKNIFEEWKVTRLTFEQDPEPIWQDRDNNVKDLCESLNVEWIEKVSHMLWDPHLIIQ
ENGDTPPLTYAMFCQVAEIVGLPPRPVEEPDFSEVTLPVSENHDRLFALPTMEDLGVQAECAAQKSPVCRYLG
GETKALMLLDLRLDKERLGFSMGQAQANQIYPDLTGMPMSLSPHLRFGNLSIRKFYWALRDAYSEVNPSTCIP
PEITSQLIWREYFYCMSVNNPYYNRMKENPICLNIDWYSDDQGFEKWKDGKTGYPWIDACMRQLIQEGWIHQV
CRHAVSCFLTRGDLWIDWQKGLEVFDRHLLDADWSVCAGNWMWVSSSAFEKVLQCPRCICPVRYGRRIDPTGD
YIRRYVPELKNMPLDYLFEPWKAPKDVQEKAGCVIGTNYPPPMVDHRQASRDCKLKMEIIKGKYKDIPHVAPA
NEDEVISLSWMKMGPGKDYTCDHNHTDENHP
>Tritonia_tet_AWY11211.1_6-4_photol
MTQHRSIHWFRKGLRLHDNPAFIKACEQASEIYPIFILDPWFAKSANCRVGINRWRFLLQALRDLDNSLKTLN
LRLFVVRGNPNTELENLCQRWKISRLTFEVDTEPYAVKRDEEVERQMGEMGIEVIKSLSHTLFEPQRVIKVNG
GSAPLTYQRFQTIASKLGPPPKPLDAPTLESLLGCKTLISDNHEKEFGVPTLTELGKDPKSCGPLLYPGGEKE
ALERMERQLKKTNWICSFEKPQTSPNSLTPSTTVLSPYLKFGCLSSRLFYHQLADMYKGKKHSQPPVSLHGQL
LWREFFYTVASVTPNFDKMEGNPVCRQIPWQSNPDHLEKWANGQTGFPFIDAIMRQLRQEGWIHHLARHAVAC
FLTRGDLWISWEEGMTVFEELLLDADWSLNAANWMWLSASAFFHQYFRVYSPIAFGKKTDPNGDYIRKYVPEV
KKFPSKYIFEPWTAPLSMQQTAGCVLGKDYPKPLVDHSVASKANISKMAAAYNAAKKDKLNNASQNGMTAQNG
KKRKQNKKDGLVQKKLKFT
>Tritonia_tet_AWY11214.1_cry_dash
MLFHRNISRIFFARRQYISFSSMSSSTRVCIVLFRNDLRLHDNEALFLANKNCSHVLPLYCFDPRHFQGTYHY
DLPKTGSHRAKFLLECVTDLKTNLQSRGSDLVVRLGKPENVIPELMANFNLGQDTVVVFQREVTDEEVKVEKG
VKENSKLRVETVWGHTLHHVADLPFQPKDLPDVYTEFRKRVEGNTAVRQCFNLPDKFKPLPSSVDTGTLLTLQ
DLGLSVPETDPKSAFPFNGGETAALERLNNYLWGSDNVSTYKETRNGMIGSDYSTKFSSWLAHGCISPRKIYW
EIKKYEQERTANQSTYWVIFELLWRDYFRYVAMKFGNQLFYSSGIKGGNVHWKKNMQHFKAWQEGKTGVPYVD

ANMRELAATGFMSNRGRQNVASFLTKDLQLDWRLGAEWFESMLIDHDVCSNYGNWLYSAGIGNDPRENRKFNV
VKQGLDYDADGDYIRIWVPELRGLKSGKVHCIWTASKMIHDSAGISLGETYPYPIVMAPEWNRHVNRPTSSGA
GTSRGGGAGKGPGSGRGQHSQSSGQKRGMDFYFKNSNPQ
>Trogon_mela__NXJ73608.1
DNWAFLYAQRLALKQELPLHVCFCLVPKFLDATIRHYCFMLRGLQEVAKECAELNIPFHLLLGYAKDVLPVFV
EEHGVGGLVTDFCPLRLPRQWVEDVRERLPDDVPFAQVDAHNIVPCWVASSKQEYSAWTFRSKIHAQLPWFLT
EFPPVIRHPYPPSCSAEPIAWEACYSSLQVDRTVKEVEWATPGTAAGLAVLQSFIAERLKSFASHRNNPNKAA
LSNLSPWFHFGQVSTQRAILEVQKHQDKYKESVEVFVEEAVVRRELSENFCYYNENYDSVQGAYDWAQTTLKL
HAKDKRPYLYKLQELEQGTTHDPLWNAAQLQMVREGKMHGFLRMYWAKKILEWTRSPEEALQFAIYLNDRYEL
DGRDPNGYVGKLQDWATGRGDLITDGQGCGCLWSICGIHDHGWTERAVFGKIRYMNYAGCKRKFNVGEFERRY
APGD
>Trogon_mela__NXJ74995.1
RFLLQSLEDLDNSLRKLSSRLFVVRGQPTDVFPRLFKEWGVTRLTFEYDSEPFGKERDAAIVKLAKEAGVEVV
IENSHTLYDLDRIIELNGHKPPLTYKRFQAIISRMELPKKPVSTVLSQQMETCKVDIQENHDDVYGVPSLEEL
GFPTDGLAPAVWQGGETEALARLDKHLERKAWVANYERPRMNANSLLASPTGLSPYLRFGCLSCRLFYYRLWE
LYKKVKRNSTPPLSLYGQLLWREFFYTAATNNPKFDRMEGNPICIQIPWDRNPEALAKWAEGKTGFPWIDAIM
TQLRQEGWIHHLARHAVACFLTRGDLWISWESGVRVFDELLLDADFSVNAGSWMWLSCSAFFQQFFHCYCPVG
FGRRTDPSGDYVKRYLPKLKGFPSRYIYEPWNAPESVQKAAKCIIGVDYPKPMVNHAETSRLNIERMKQIYQQ
LSRYRGLCLLASVPSCVEDLSGPVTDSASAQGCSTSTVARLSQADQASPKRKHEGAEELCAEELYKRAKVTGL
PAPEIPGKNL
>Trogon_mela__NXJ82119.1
MGVNAVHWFRKGLRLHDNPALRECIQGADTVRCVYILDPWFAGSSNVGINRWRFLLQCLEDLDANLRKLNSRL
FVIRGQPADVFPRLFKEWNIAKLSIEYDSEPFGKERDAAIKKLASEAGVEVIVRISHTLYDLDKIIELNGGQP
PLTYKRFQTLISRMEPLEMPVETITPEVMEKCTTPVSDDHDEKYGVPSLEELGFDTDGLPSAVWPGGETEALT
RLERHLERKAWVANFERPRMNANSLLASPTGLSPYLRFGCLSCRLFYFKLTDLYKKVKKNSSPPLSLYGQLLW
REFFYTAATNNPRFDKMEGNPICVQIPWDKNPEALAKWAEGRTGFPWIDAIMTQLRQEGWIHHLARHAVACFL
TRGDLWISWEEGMKVFEELLLDADWSVNAGSWMWLSCSSFFQQFFHCYCPVGFGRRTDPNGDYIRRYLPVLRG
FPAKYIYDPWNAPESVQKAAKCIIGVNYPKPMVNHAEASRLNIERMKQIYQQLSRYRGLGLLATVPSNPNGNG
NGGLMGYSPGESISGCGSTGGAQLGTGDGHTVVQPCALGDSHTGASGIQQQGYCQASSILHYAHGDNQQSHLL
QAGRTALGTGISAGKRPNPEEETQSVGPKVQRQSTN
>Trogon_mela__NXJ82491.1
KVDYDVCSNYGNWLYSAGVGNDPRDNRKFNVIKQGLDYDGNGDYVRLWVPELRDIKGADIHSPWALSRAALSQ
AGVTLGETYPWPVVKAPEWSRHINQRRGRSPRPGGRRGPAHKDRGIDFYFSRKKDE
>Trogon_mela__NXJ84835.1
MPHRTIHLFRKGLRLHDNPTLLAALESSEVVYPVYILDRTFVTSAMHIGALRWHFLLQSLEDLRQNLCQLGSC
LLVLQGEYEAVLRDHARRWEITQVTLDAETEPFYKEMEANIQRLGEELGFQVLSLVGHSLYDTRRILELNGGT
PPLTYKRFLQVLSLLGDPKVPVRNLTAEDFQCKTPDPGLADCYRVPLPADLKIPPESLSPWRGGETEGLQRLE
QHLTDQGWVASFTKPRTIPNSLLPSTTGLSPYFSLGCLSVRTFFYRLSNIYAQVRKHLRSFPSPSLMVFWMMR
PAQVCSRVKARPLGFGVLCLPSGKLSCWRSFPACPLRRVPSVTLRLPAWFGLFFTTWLFQGRFSLSLPIPEGQ
VFEELLLDADYSINAGNWMWLSASAFFHQYTRIFCPVRFGKRTDPEGHYIRKYLPVLKNFASKYIYEPWMASE
EEQKQAGCIIGQDYPFPMVNHKEASDHNLQLMKQVREEQQRTAQLTR
>Tupaia_chin__XP_006157905.3
YILEPWFAASSSVGINRWRFLLQSLEDLDTSLRKLNSRLFVVRGQPADVFPRLFKEWGVTRLTFEYDSEPFGK
ERDAAIMKMAKEAGVEVVTENSHTLYDLDRIIELNGQKPPLTYKRFQAIISRMELPRKPVGSVTSQQMESCRA
EIQENHDETYGVPSLEELGFPTEGLGPAVWQGGETEALARLDKHLERKAWVANYERPRMNANSLLASPTGLSP
YLRFGCLSCRLFYYRLWDLYKKVKRNSTPPLSLFGQLLWREFFYTAATNNPRFDRMEGNPICIQIPWDRNPEA
LAKWAEGKTGFPWIDAIMTQLRQEGWIHHLARHAVACFLTRGDLWVSWESGVRVFDELLLDADFSVNAGSWMW
LSCSAFFQQFFHCYCPVGFGRRTDPSGDYIRRYLPKLKGFPSRYIYEPWNAPESVQKAAKCVIGVDYPRPIVN
HAETSRLNIERMKQIYQQLSRYRGLCLLASVPSCVEDLNHPVAEPSSSQAGDRSSTGPRPLPSGLASPKRKLE
AAEEPPGGELSKRARVAELPNPELPGKDV
>Tupaia_chin__XP_006170414.1
MGVNAVHWFRKGLRLHDNPALKECIQGADTIRCVYILDPWFAGSSNVGINRWRFLLQCLEDLDANLRKLNSRL
FVIRGQPADVFPRLFKEWNITKLSIEYDSEPFGKERDAAIKKLATEAGVEVIVRISHTLYDLDKIIELNGGQP
PLTYKRFQTLISKMEPLEIPVETITSEVIEKCTTPLSDDHDEKFGVPSLEELGFDTDGLSSAVWPGGETEALT
RLERHLERKAWVANFERPRMNSNSLLASPTGLSPYLRFGCLSCRLFYFKLTDLYKKVKKNSSPPLSLYGQLLW
REFFYTAATNNPRFDKMEGNPICVQIPWDKNPEALAKWAEGRTGFPWIDAIMTQLRQEGWIHHLARHAVACFL
TRGDLWISWEEGMKVFEELLLDADWSINAGSWMWLSCSSFFQQFFHCYCPVGFGRRTDPNGDYIRRYLPVLRG
FPAKYIYDPWNAPEGVQKVAKCLIGVNYPKPMVNHAEASRLNIERMKQIYQQLSRYRGLGLLASVPSNPNGNG
GLMGYSPGDNTPGCSSSGSCSQGSGILHYAHGDSQQTHLLKQGRSSMGPGLSSGKRPSQEEDTQSIGPKVQRQ
STN

```
>Tyto_alba__KFV57896.1
DNWAFLYAQRLALKQELPLHVCFCLVPKFLDATIRHYGFMLRGLQEVAEECTELNIPFHLLRGYAKDVLPAFV
AKHDVAGAVGVEDIRERLPEYVPFVQVDAHNIIPCWVTSSKQEYTARTIRGKIHAQLPEFLTEFPPVIHHPYP
PSCPAEPISWEACYSSLQVDRTVKEVEWATPGTTTGLAVLQSFITERLKSFGSHRNDPNKAALSNLSPWFHFG
QVSTQRAILEVQKHRDKYKESVDAFVEEAVVRRELAENFCYYNENYDSVQGAYDWAQTTLKLHAKDKRPFLYK
LQELEQGTTHDPLWNAAQLQMVQEGKMHGFLRMYWAKKILEWTRSPEEALQFAIYLNDRYELDGRDPNGYV
>Tyto_alba__XP_009962599.3
MPHRTIHLFRKGLRLHDNPMLLAVLESSEVIYPVYILDRKFMVSMMHIGALRWHFLLQSLEDLQKNLCRLGSC
LLVIQGEYESVLRDCIQKWNITQVSLDAEMESFYKEMEANIRRLGDELGVEVLTLVGHSLYDTKRILDLNGGT
PPLTYKRFLHILSLLGDPEVPVRNLTAEDFQRCRPPDPGLAEFYRVPLPADLKIPLKSLSPWRGGETEGLQRL
EQHLTDQGWVKSFTKPRTIPNSLLPSTTGLSPYFSMGCLSVRTFFYRLSNIYAQAKHHSLPPASLQGQLLWRE
FFYTVASATPNFTKMAGNPICLQISWCEDAERLHKWKMAQTGFPWIDAIMTQLRQEGWIHHLARHAVACFLTR
GDLWISWEEGMKVFEELLLDADYSINAGNWLWLSASAFFHQYTRIFCPVLFGKRTDPQGQYIRKYLPILKDFP
SKYIYEPWTAPEEEQKEAGCIIGHDYPFPMVNHKEASDHNLQLMKQVREEQYRTAQLTRDDTDDPMEMKVKRD
HSEENISKGKVAKMTERNEILSGITCWEPKNGRRGEPEAR
>Tyto_alba__XP_042648484.1
MGVNAVHWFRKGLRLHDNPALRECIQGADTIRCVYILDPWFAGSSNVGINRWRFLLQCLEDLDANLRKLNSRL
FVIRGQPADVFPRLFKEWNIAKLSIEYDSEPFGKERDAAIKKLASEAGVEVIVRISHTLYDLDKIIELNGGQP
PLTYKRFQTLISRMEPLEMPVETITPEVMEKCTTPVSDDHDEKYGVPSLEELGFDTDGLPSAVWPGGETEALT
RLERHLERKAWVANFERPRMNANSLLASPTGLSPYLRFGCLSCRLFYFKLTDLYKKVKKNSSPPLSLYGQLLW
REFFYTAATNNPRFDKMEGNPICVQIPWDKNPEALAKWAEGRTGFPWIDAIMTQLRQEGWIHHLARHAVACFL
TRGDLWISWEEGMKVFEELLLDADWSVNAGSWMWLSCSSFFQQFFHCYCPVGFGRRTDPNGDYIRRYLPVLRG
FPAKYIYDPWNAPESIQKAAKCIIGVNYPKPMVNHAEASRLNIERMKQIYQQLSRYRGLGLLATVPSNPNGNG
NGGLMGYSPGESISGCGGTGAQLGAGDGHTVVQPCALGEPQTGASGIQQQGYCQASSILHYAHGDNQQPHLLQ
AGRMALGTGISAGKRPNPEEETQTVSGPKVQRQSTN
>Upupa_epop__NWU92594.1
MPHRTIHLFRKGLRLHDNPTLLAALESSEVIYPIYILDRKFMTSVMHMGTLRWHFLLQSLEDLQKNLAQLGSC
LLVVQGEYESVLRDHVRRWDITQVTLDAEMEPFYKEMEANIRRLGEELGFEVLSLVGHSLYDTTRILDLNGGT
PPLTYKRFLHILSLLGDPMVPVRNPTAEDFQSCRPPEPGLAERYAVPLPEDLKMPLESISPWRGGETEGLQRL
EQHLTDQGWVMSFTKPRTIPNSLLPSTTGLSPYFSLGCLSVRTFFYRLSNIYAQAKHHSLPPVSLQGQLLWRE
FFYTVASATPNFTQMTGNPICLQINWCKDAERLHKWKMAQTGFPWIDAIMTQLRQEGWIHHLARHAVACFLTR
GDLWISWEEGMKVFEELLLDADYSINAGNWMWLSASAFFHQYTRIFCPVRFGKRTDPEGRYIRKYLPILKNFA
SKYIYEPWTASEEEQKQAGCIIGRDYPFPMVDHKEASDHNLRLMKQVREEQYRTAQLTRDDTDDPMEAKVKRD
LVEENVSKVKVARMTE
>Upupa_epop__NWU92714.1
LQEWGVTRLTFEYDSEPFGKERDAAIIKLAKEAGVEVVIENSHTLYDLDRIIELNGHKPPLTYKRFQAIISRM
ELPKKPVNTVISQQMETCKVDIQENHDDVYGVPSLEELGFPTDGLAPAVWQGGETEALARLDKHLERKAWVAN
YERPRMNANSLLASPTGLSPYLRFGCLSCRLFYYRLWELYKKVKRNSTPPLSLYGQLLWREFFYTAATNNPKF
DRMEGNPICIQIPWDRNPEALAKWAEGKTGFPWIDAIMTQLRQEGWIHHLARHAVACFLTRGDLWISWESGVR
VFDELLLDADFSVNAGSWMWLSCSAFFQQFFHCYCPVGFGRRTDPSGDYVKRYLPKLKGFPSRYIYEPWNAPE
SVQKAAKCIIGVDYPKPMVNHAETSRLNIERMKQIYQQLSRYRGLCLLASVPSCVEDLTVPVTDSASGQGCST
ST
>Upupa_epop__NWV00793.1
MGVNAVHWFRKGLRLHDNPALRECIQGADTIRCVYILDPWFAGSSNVGINRWRFLLQCLEDLDANLRKLNSRL
FVIRGQPADVFPRLFKVNAAGHLNLPWHSLLPFPPVLLLVPREEANTHLSTRASVVPCCCLTSFILFFQSRII
ELNGGQPPLTYKRFQTLISRMEPLEMPVETITPEVMEKCTTPVSDDHDEKYGVPSLEELGFDTDGLPSAVWPG
GETEALTRLERHLERKAWVANFERPRMNANSLLASPTGLSPYLRFGCLSCRLFYFKLTDLYKKVKKNSSPPLS
LYGQLLWREFFYTAATNNPRFDKMEGNPICVQIPWDKNPEALAKWAEGRTGFPWIDAIMTQLRQEGWIHHLAR
HAVACFLTRGDLWISWEEGMKVFEELLLDADWSVNAGSWMWLSCSSFFQQFFHCYCPVGFGRRTDPNGDYIRR
YLPVLRGFPAKYIYDPWNAPESVQKAAKCIIGVNYPKPMVNHAEASRLNIERMKQIYQQLSRYRGLGLLATVP
SNPNGNGNGGLMGYSPGESISGCGSTGGAQLGTGDGHTVVQPCALGDSHTGASGIQQQGYCQASSILHYAHGD
NQQSHLLQAGRTALGTGVSAGKRPNPEEETQSVGPKVQRQSTN
>Uria_aalg__NXV41793.1
LLKVDYDVCSNYGNWLYSAGIGNDPRDNRKFNMIKQGLDYDGNGDYVRLWLPELQGIKGADIHTPWALNGAAL
SQAGVTLGKTYPQPVVTAPEWSRHINQRPQGQSPHPRGRRGPAHTPAQHKDRGIDFYFSRNKDV
>Uria_aalg__NXV42407.1
MQHSSIHWFRKGLRLHDNPALLAAATDCCRLHPLFILDPSSSRVGANAQRFLLDALRDLDGRLREMGSRLFVV
RGCPEEVFPRLFHAWGTTRLTFEVDTEPPARQRDAAVAELAAQHGVEVIREASHTLYDTERILALNNGKAPLT
YKRLQSLLASLGPPEKPAPALTQEHLQGCCTPCQVSHDIDYGVPTLEELGQDPTKVGPHLYPGGETAALARLD
VHMERTAWVCSFKKPETEPTSLSPSTTVLSPYLKFGCLSVRTFWWRLDEVYQGQRKHSQPPVSLHGQLLWREF
```

FYTAGASIPSFDQMVGNPICLQVDWDDNPQHLRAWKEGQTGYPFIDAIMTQLRSEGWIHHLARHAVACFLTRG
DLWVSWEEGLKVFEELLLDADWSLNAGNWLWLSGSAFFHRYFHVYSPITFGKKTDRDGAYIRKYLPVLKDFPA
EYIYEPWKAPQAVQEQAGCLVGTHYPQPIVEHRAASKRNMGRMKAAR
>Uria_aalg__NXV42963.1
DNWAFLYAQRLALKQELPLHVCFCLVPKFLDATIRHYGFMLKGLQEVAEECAELNIPFHLLLGYAKDVLPTFV
VEHGVGGLVTDFCPLRLPRQWVENVRERLPEDVPFAQVDAHNIVPCWVASPKQEYSARTIRGKIHAQLPEFLT
EFPPIIRHPYPPSCPAEPIAWEACYSSLQVDRTVKEVEWATPGTSAGLAVLQSFITERLKSFSSHRNDPNKAA
LSNLSPWFHFGQVSTQRAILEVQKHRGKYKESVDVFVEEAVVRRELAENFCYYNESYDSVQGAYDWAQTTLKL
HAKDKRPFLYKLQELEQGTTHDPLWNAAQLQMVQEGKMHGFLRMYWAKKILEWTRSPEEALQFAIYLNDRYEL
DGRDPNGYARGRADVPSPCLAGCLWSICGIHDQGWAERAIFGKIRYMNYAGCKRKFDVDQFERRYAPRT
>Uria_aalg__NXV44637.1
MGVNAVHWFRKGLRLHDNPALRECIQGADTVRCVYILDPWFAGSSNVGINRWRFLLQCLEDLDANLRKLNSRL
FVIRGQPADVFPRLFKEWNIAKLSIEYDSEPFGKERDAAIKKLASEAGVEVIVRISHTLYDLDKIIELNGGQP
PLTYKRFQTLISRMEPLEMPVETITPEVMEKCTTPVSDDHDEKYGVPSLEELGFDTDGLPSAVWPGGETEALT
RLERHLERKAWVANFERPRMNANSLLASPTGLSPYLRFGCLSCRLFYFKLTDLYKKVKKNSSPPLSLYGQLLW
REFFYTAATNNPRFDKMEGNPICVQIPWDKNPEALAKWAEGRTGFPWIDAIMTQLRQEGWIHHLARHAVACFL
TRGDLWISWEEGMKVFEELLLDADWSVNAGSWMWLSCSSFFQQFFHCYCPVGFGRRTDPNGDYIRRYLPVLRG
FPAKYIYDPWNAPESIQKAAKCIIGVNYPKPMVNHAEASRLNIERMKQIYQQLSRYRGLGLLATVPSNPNGNG
NGGLMGYSPGESISGCGSTGGHLFPASPGAQLGTGDGHTVVQPCALGDSHTGASGIQQQGYCQASSILHYAHG
DNQQSHLLQAGRTTLGTGISAGKRPNPEEETQSVGPKVQRQSTN
>Uria_aalg__NXV48596.1
RFLLQSLEDLDNSLRKLSSRLFVVRGQPTDVFPRLFKEWGVTRLTFEYDSEPFGKERDAAIVKLAKEAGVEVV
IENSHTLYDLDRIIELNGHKPPLTYKRFQAIISRMELPKKPVSTVMSQQMETCKVDIQENHDDVYGVPTLEEL
GFPTDGLAPAVWQGGETEALARLDKHLERKAWVANYERPRMNANSLLASPTGLSPYLRFGCLSCRLFYYRLWE
LYKKVKRNSTPPLSLYGQLLWREFFYTAATNNPKFDRMEGNPICIQIPWDRNPEALAKWAEGKTGFPWIDAIM
TQLRQEGWIHHLARHAVACFLTRGDLWISWESGVRVFDELLLDADFSVNAGSWMWLSCSAFFQQFFHCYCPVG
FGRRTDPSGDYVKRYLPKLKGFPSRYIYEPWNAPESVQKAAKCIIGVDYPKPMVNHAETSRLNIERMKQIYQQ
LSRYRGLCLLASVPSCVEDLSGPVADSASGQGCSTSTAMRLSQADQASPKRKHEGAEELCAEELYKRAKVTDL
PAPEIPGKSL
>Uria_aalg__NXV53482.1
MPHRTIHLFRKGLRLHDNPALLAALESSEVLYPVYILDRKFMASVMHIGALRWHFLLQSLEDLQKNLRQLGSC
LLVIQGEYESVIRDHVQKWNITQVTLDAEMEPFYKEMEAKIRCLGEELGFEVLSLVGHSLYDTKRILDLNGGT
PPLTYKRFLHILSLLGDPEVPVRNLTAEDFQRCRSPDPVLAEGYRVPLPVDLKIPLESLSPWRGGETEGLQRL
EQHLTDQGWVASFTKPRTVPNSLLPSTTGLSPYFSMGCLSVRTFFYRLSNIYAQAKHHSLPPVSLQGQLLWRE
FFYTVASATPNFTRMAGNPICLQISWYEDAERLHKWK
>Urocolius_indi__NXX75599.1
VLQEWNIAKLSIEYDSEPFGKERDAAIKKLASEAGVEVIVRISHTLYDLDKIIELNGGQPPLTYKRFQTLISR
MEPLEIPVETITPEVMEKCTTPVSDDHDEKYGVPSLEELGFDTDGLPSAVWPGGETEALTRLERHLERKAWVA
NFERPRMNANSLLASPTGLSPYLRFGCLSCRLFYFKLTDLYKKVKKNSSPPLSLYGQLLWREFFYTAATNNPR
FDKMEGNPICVQIPWDKNPEALAKWAEGRTGFPWIDAIMTQLRQEGWIHHLARHAVACFLTRGDLWISWEEGM
KVFEELLLDADWSVNAGSWMWLSCSSFFQQFFHCYCPVGFGRRTDPNGDYIRRYLPVLRGFPAKYIYDPWNAP
ESVQKAAKCIIGVNYPKPMVNHAEASRLNIERMKQIYQQLSRYRGLGLLATVPSNPNGNGNGGLMGYSPGESI
SGCGSTGVCFVFFFFFFPASPGAQMGTGDGHTVVQPCALGESHSGGASGIQQQGYCQASSILHYAHGASQQSH
LLQAGRTTLGTGISAGKRPNPEEETQSVGPKVQRQSTS
>Urocolius_indi__NXX77873.1
MSHRTIHLFRKGLRLHDNPTLLAAVESSHVLYPVYILDREFVTSVMHIGALRWHFLLQSLQDLQKNLCQLGSC
LLVLQGEYESVLKAHIQKWNITQVTLDAEMEPFYKEMESNVRRLGEELGFQVLALVGHSLYDTKRILDLNGGS
PPLTYKKFLHILSLLGDPEVPVRNLTAEDFQRCRPPDPSLAECYRVPLPVDLNIPLESLSPWKGGETEGLQRL
EQHLTDQGWVAHFTKPKTIPNSLLPSTTGLSPYFSMGCLSVRTFFYRLSNIYAQAKHHSLPPVSLQGQLLWRE
FFYTVASATPNFTRMVGNPICLQIPWYEDAEKLHKWKTAQTGFPWIDAIMTQLQQEGWIHHLARHAVACFLTR
GDLWLSWEEGMKVFEEMLLDADYSINAGNWMWLSASAFFHQYTRIFCPVRFGRRTDPEGNYIRKYLPVLKNFP
PKYIYEPWTAPEEEQKRAGCIIGQDYPFPMVNHKEASDHNLQLMKQVREEQHRTAQLTRDDTDDPMEAKLKRG
HPEELSKGKVAKVME
>Urocolius_indi__NXX78149.1
RFLLQSLEDLDNSLRKLNSRLFVVRGQPTDVFPRLFKEWGVTRLTFEYDSEPFGKERDAAIVKLAKEAGVEVV
IENSHTLYDLDRIIELNGHKPPLTYKRFQAIISRMELPKKPVSTVISQQMETCKVDIQENHDDVYGVPSLEEL
GFPTDGLAPAVWQGGETEALARLDKHLERKAWVANYERPRMNANSLLASPTGLSPYLRFGCLSCRLFYYRLWE
LYKKVKRNSTPPLSLYGQLLWREFFYTAATNNPKFDRMEGNPICIQIPWDRNPEALAKWAEGKTGFPWIDAIM
TQLRQEGWIHHLARHAVACFLTRGDLWISWESGVRVFDELLLDADFSVNAGSWMWLSCSAFFQQFFHCYCPVG
FGRRTDPSGDYVKRYLPKLKGFPSRYIYEPWNAPESVQKAAKCIIGVDYPKPMVNHAETSRLNIERMKQIYQQ

LSRYRGLCLLASVPSCVEDLSGPVSDSASGQGCSSSTALRLSQADQASPKRKHEGAEELCTEELSKRAKVTGL
PASEIPGKSL
>Urocolius_indi__NXX82793.1
DNWAFLYAQRLALKQELPLHVCFCLAPKFLDATIRHYGFMLRGLQEVAKECEELNIPFHMLLGYAKDVLPTFV
VEHGVGGLVTDFCPLRLPRQWVEDVRQRLPEDVPFAQVDAHNIVPCWVASSKQEYSAWTFRGKIQTQLPEFLT
EFPPVIHHPYPPSRPAEPIDWEACYSSLQVDHTVKEVEWATPGTAAGLAVLQSFISQRLKSFGAHRNNPNKEA
LSNLSPWFHFGQVSTQRAILEVQKQRNKYKESVDVFVEEAVVRRELSENFCYYNDKYDSVQGAYSWAQTTLKL
HAKDKRPFLYKLQELEQGTTHDPLWNAAQLQMVREGKMHGFLRMYWAKKILEWTQSPEEALQFAIYLNDRYEL
DGRDPNGYVGCLWSICGIHDHGWTERAVFGKIRYMNYAGCKRKFNVDQFERRYAPQT
>Urocolius_indi__NXX83306.1
KVDYDVCSNYGNWLYSAGIGNDPRGNRKFNVIKQGLDYDGNGDYVRLWIPELQGIKGADVHTPWALNSAALSQ
AGVTLGKTYPQPVVTAPEWSRHINQSLRERSPHPRGRTGPTHTPVQRKQRGIDFYFSSKKAV
>Ursus_mari__XP_008703869.1
MGVNAVHWFRKGLRLHDNPALKECIQGADTIRCVYILDPWFAGSSNVGINRWRFLLQCLEDLDANLRKLNSRL
FVIRGQPADVFPRLFKEWNITKLSIEYDSEPFGKERDAAIKKLATEAGVEVIVRISHTLYDLDKIIELNGGQP
PLTYKRFQTLISKMEPLEIPVETITSEVIEKCTTPLSDDHDEKYGVPSLEELGFDTDGLPSAVWPGGETEALT
RLERHLERKAWVANFERPRMNANSLLASPTGLSPYLRFGCLSCRLFYFKLTDLYKKVKKNSSPPLSLYGQLLW
REFFYTAATNNPRFDKMEGNPICVQIPWDKNPEALAKWAEGRTGFPWIDAIMTQLRQEGWIHHLARHAVACFL
TRGDLWISWEEGMKVFEELLLDADWSINAGSWMWLSCSSFFQQFFHCYCPVGFGRRTDPNGDYIRRYLPVLRG
FPAKYIYDPWNAPEGIQKVAKCLIGVNYPKPMVNHAEASRLNIERMKQIYQQLSRYRGLGLLASVPSNPNGNG
GLMGYSPGENIPGCSSSGRRSSMGSGLSSGKRPSEEEDTQSIGPKVQRQSTN
>Ursus_mari__XP_040494929.1
MAAAVVTAAAAVPTPAAGVDGASSVHWFRKGLRLHDNPALLAAVRGARCVRCVYILDPWFAASSSVGINRWRF
LLQSLEDLDTSLRKLNSRLFVVRGQPADVFPRLFKEWGVTRLTFEYDSEPFGKERDAAIMKMAKEAGVEVVTE
NSHTLYDLDKIIELNGQKPPLTYKRFQAIISRMELPKKPVGSVTNQQMESCRADIQENHNDTYGVPSLEELGF
PTEGLGPAVWQGGETEALARLDKHLERKAWVANYERPRMNANSLLASPTGLSPYLRFGCLSCRLFYYRLWDLY
KKVKRNSTPPLSLFGQLLWREFFYTAATNNPRFDRMEGNPICIQIPWDRNPEALAKWAEGKTGFPWIDAIMTQ
LRQEGWIHHLARHAVACFLTRGDLWVSWESGVRVFDELLLDADFSVNAGSWMWLSCSAFFQQFFHCYCPVGFG
RRTDPSGDYIRRYLPILKGFPSRYIYEPWNAPEAIQKAAKCIIGVDYPRPIVNHAETSRLNIERMKQIYQQLS
RYRGLCLLASVPSCVEDLNNPVAEPSSSQTGSMSSAGPRPLPSGPASPKRKLEAAEEPPGEELSKRARVAELP
APEPPSRDV
>Verrucomicrobia_bac_DASH_family_cryptochrome_MBC7970164.1
MTQTKILLWYRSDLRSHDHEPLHTAVEKIQATSGQIVPFYCFDSRQFKQTAFGFPKTGAFRVQFLLESLADLR
RSLQALGSDLVVRQGLPEVVIPTLVKQLKLTAVYYHREVTSEETAIETALQNALKPLGVAFKSFWGHTLFHPD
DLPFELRQLPELFTHFRKQVEKQSTVNPARRSPIQLPSLPDVEAGVLPPLTDLGLEPPTYDDRAVLVFKGGET
VGLARLNHYLWQHDCIKTYKETRNGMLGADYSSKFSPWLSLGCLSPRYIYEQVQQYEQERVRNDSTYWLIFEL
LWRDYFRFICAKHGDRLFRRSGLQGIDIVWKEDWQRFDHWREGTTGFPLVDANMRELSATGFMSNRGRQNVAS
FLTKNLGIDWRMGAEWFESLLLDYDVCSNWGNWNYTAGVGNDARGFRFFNILKQSRDYDPQGAYVKHWLPMLS
NVPATKVHEPWKLLPVEQERFGIKIGVDYPNPLVDLFKSAAANEKIYNAAIETQ
>Verrucomicrobia_bac_HAL92164.1_photolyase
MIHAARIHRMDNRPPARGKHILYWMQQAQRAEFNPALEYAVEQANALRLPVVAGFALTSFPEANRRHYQFMLE
GLRETQKRLAARGIGFCIRPGPPEEVIPALAKNAALVVGDVGYLRVQRQWREAVAKHLACPFVLVETDAIVPV
AEASDHAEFAARTLRPKIHRKLAEFLKSGKPPAVARPSLDLAKRSLSLDDPAAACRALGVDESVPPSDRLAGG
LAAANKLLDRFIARRLADYATDASDPSLDGCSHMSPYLHFGQISPLDIALRVHASDAPRAATDAFLEQLIVRR
ELSLNACWFNPAYDRYDALPAWSRKTLDRHRKDKREFIYSRAQWEAGATHDPCWNAAQAEMVRSGQMHNYMRM
YWGKKILEWSRTPEEAFETALHLNNKYELDGRDPNGFTGVAWCFGLHDRPWTERAVFGNIRYMNANGLRRKFD
IEAYVERWLGPQTMSLF
>Verrucomicrobia_bac_NBU85991.1_EBS13_0151
KDIEKIFTSSFPKDAEGFFKIPTPLEEPAKIKNAPDDFSLTNISSHFPGEVHSVSSLKTKFSELGFLQATNEN
AHFKGGETEALVRLECKVSSQTDYVNGFRKPKTSSTNLPENPLEPSTTGLSPYITVGSLSVRRLWQEVEKANR
QGPHSSPPESLHGQLLFREMFYILSLSVPNWDSDQDNSMCKKINWGKNDKAKLSAWTDGQTGFPLIDALMRQL
ETTGWMHHLGRHAVSCFLTRGQLWQHWKTGRDVFDQKLLDSDWALNNANWLWLSGVAPFSMPYFRLYNPCPDG
KSSLNVDATEANFIRHWVPELKNFPVKYIYEPHLAPPAVQEDAGCVIGKDYPEPMVDRKAVAKENLSQFKASL
N
>Verrucomicrobia_bac_NCW27700.1_photolyase
MSAPPVIVWFRRDLRISDHTALHRAALEGVPLAPVFVFDPAILSGSDVGIRRPAFLHACLASLMGNLQSVGSH
LIVRKGRPEVELMALAKEIGAKKIFYNKDVEPYSRKRDKKVDQAAADCGIETVPCDDLLIHPPGRVQRATGGP
YTVFTPFSRAWLSLPPQDPLPRPKQLVPIKGIKVADLPSPERFGLSPCDISLPPAGEKVARDMLRAFCAKNLR
RYGEIRNYPLADATSRLSPHLRFGTISPRTVLAEARRVRAEDPSSRREVDIFIGELIWRDFYKQILWEFPHVA
TGSFRPEYDRVEWENDREKFATWCQGCTGFPIVDAAMRQLNQTGWMHNRLRMIVASFLTKDLLISWQWGERYF

MEKLFDGDLAANNGGWQWAAGTGTDAQPYFRIFNPSSQAEKFDPEGKFIAQYVPEADLLTYPAPMVDHAKQRV
RALEMYKKARS
>Vexillum_par_AQX17840.1_putative_p
MSKIGLHWFRYGLRLHDNPPLLKAIKTCSKVIPLYILDTDYFKPDKVGINRIGFLLDTLKALDSDLRAKGSRL
FVAQGNPNEVIRNLIQEHKVELLSFERDTEPYNKHMDNNIIETAKSLNVETFPLWGHTMFDPEYLLEMSNGKA
PLTMTSFLTLMSETGDPPKPLDPPESMPSPIDNLKCKDVFVYEGIPTLSDLKGFNFDPKDYTTWFVAGEKEAI
KKMTEFLAQKHRVSTFEKPKTLPTALQPDTTALSPYITRGSLSSRLFYHSLKDTLKGMQSSKPPVSLKGQLYW
REMAYLIGFSVPNFNQMEGNPICKQIPWLTGEEAKSLLEKWEMGQTGYPAVDAVMNQLRTEGWMHHLARHLVA
CYLTRGDLWVTWELGRDVFDKHLVDADWSINNFSWHWLSCSAFFHQYFRCYSPVAFFKKTDPNGNYIKKHVPI
LAKFPDKYIYEPWTAPKGIQTACGCIIGKDYPKPMVDHKFVSKENMDRMKKAYDAGKAKAKNSGEPPAKKLKQ
NKLGKYAKK
>Vibrio_cho_WP_076008192.1
MSKKIGLYWFTNDLRLNDNPLLEQASQQVDRLICLYCYPSITPFLARYAQQTQWGEAKKRFLNQTLADLDHSL
STLGQKLWVTPLLPYQALRHLLTQVEITDIYVDAMAGSDERQAIARIHQDFSSVHIHQQALHSLLSEPQLPFA
LEALPSTFTQFRKQVETLSLSSAPIGYPRVLPPIEQGWQLPLMDIVTEPNHSAFVGGEQAGLTHCQHYFSSML
PSRYKETRNGLDGMDYSTKFSPWLALGAVSPKTIYAMLQRYEAVHGANDSTYWIFFELLWREYFYWYARRYGA
KLFRFSGIGEKKPLTSFYAQRFLQWKHGETPFPIVNACMRQLNQTGYMSNRGRQLVASCLVHELGLDWRYGAA
YFETQLMDYDVGSNWGNWQYLAGVGADPRGSRQFNLAKQAHTYDPKGEFVAKWCGTDCGKLNALENLTLDSVD
MVDWPIAASASLFIHHPKNKESSS
>Vibrio_cho_WP_148537779.1_photolyase
MRLVWFRRDLRSFDNTALTAALNSGDPVAAMYIATPEQWHQHHLAPIQADLIWRRLAELQQELAALNVPLFYQ
QVADFQAAAVAVSQLAKTLNATQVLANRDYELDEQQRDQSAQQLLAEQGITWSAFDDKCVLPPGSVRTKQGEF
FKVFTPFKRAWLTLFQPPVIGKNRPAALWDVPSALAEWVWHPEQTFDYPRIDSMPWAADFETVRAQLRDFCRE
RVQDYHQARDFPAQEGTSSLSPYLAIGVLSARQCVARLYRESSMGELSEGAQVWLSELIWREFYQHLVAIEPN
LSKSRDFVEWGARLEWWNDNEKFQLWCEGKTGYPIVDAAMRQLNQTGWMHNRLRMIVASFLTKDLHIDWRWGE
RYFMSRLIDGDYAANNGGWQWCASTGCDGQPYFRIFNPVSQGEKFDPNGDFIRRWVPELRSVSSAYIHQPWTY
PAVNSVLYPARLVDHKQEREVTLRLYKTAKG
>Vitrella_bra_CEM03182.1_unnamed_pr
MPEGLTKAQKKNQKRKQKRDEQRPQTTAIVWLREDLRLHDNPALHQATQCGAIVIPVYIKDESDPSPWPLRGA
ALYWKYWSLKCFDNSLRGCGSRLTLRRGDALATIAQLAADTGATSVYWNRACEPWNVERDEHVKESLQMNGIQ
VHDFKGVVLYEPWEANPDDNAHRAGFGSVGFFRAACERLGEPSKPLPAVEKLKAPLLWPASLTVDDLGYQRMP
VRKGGEVVDWAASIRQRWEFGEAGALRALDEFITSDASQKFESRQRFRADRKWTAQISPYVRFGELSSRLIYW
EAKRRIGHNCQTFIRRLFWRDLAYWTLWKFPSCHTEPLRPQYAHQRWGTDAAALKKWQRGRTGFPLVDAAMRQ
LWAIGWMPNYLRHVVAGFLVEYMNMHWFEGFKWFDYTLVDSDVAINAYMWQNGGHSGLDQWNFVMHPVYAAKS
CDPEGNYVRQWVPELRHLPVEYVHCPWEAPVTLLASARVLLGRDYYRRTIDDLLAARRNSHKAVIEVREGVGK
PYVLACGNEKMQLPDGRWVRLITRIDYRMMAANPVTVQTAADAWNKSKRRLLDQMSLALADEQRIYHQSHPDA
DEEEKADL
>Vitrella_bra_CEM04076.1_unnamed_pr
MGKSAVLWFRKGLRLHDNLALLEACKDATHVYPVFIIDPHFVGPGKVGVNRYRFLLECLTDLDTSLKTKYNSR
LIVLRGNPLDVMRSLLKDKQPFAVDKLCYEFDSEPYARSRDAEVTKIAKEGGVEVTVSPGHTLFDLDEILKKF
KGTPPLSYGPFVKMVESIGPPQTDKDAPAAIPAPPAAVLGKVDESYSVPTLQEMGCEETPHNGVLRGGETVGL
QRLHDVTRNEKWIATFEKPKTKPTDFNPASTTQMSPYMKFGCVSARKFYHHLLAIYKRHKGHSQPPVSLLGQL
YWREFFYTVGYATNNYDRMAGNKICRQIAWVNNEEWIEAWQQGKTGYPWIDAAMRQLRREGWMHHLARHAVAC
FLTRGDLFCSWEVGAKTFDELLIDADWSINNGNWMWLSCSCFFYQFFRIYHPSTFAKKYDPTGAYVRRYVPEL
RHMPDKYVYEPWKAPKAVQEEAKCIIGQDYPEPIVDHAEASQECKDRMKAAYDEHKAIKRAPKSEMKPDPDDE
EEDDEEGEEEDDDEDYSIYKPLDGTNKRSRKGKKASPKKKTKA
>Vitrella_brassi_CEL93225.1
MLLSLLTVSIARGAGVVSMSSGKEGKRKNEPSAAAASSAAGGGEAFKRARPEDVKFEEKRCRELNEVKVSSAG
DGAVLYWMSRDQRVQDNWALIKAQRLAIERNTALYVSFCLVPRFLEAAYRQYAFMIKGLKEVESELRDLNIPF
HLLIGFAKDALPPFIQQHRISVVVTDFSPVRVPMQWYRDVAASLEQSGVPLIQVDAHNVVPLWVTSSKQEYGA
RTIRGKIQGVLSHYLTQFPAVKGHPIAPPEGAFAEPFDLQATLDSLEVDREVGEVDWATPGTSAGLAMLEDFC
RNRLKMFADSRNDPTKNSLSNLSPWIHFGQISVQRCVLDVQAYVRANPSPALSKGKEAYVEEAVVRRELSDNF
CFYNPNYDSIEGAADWAKQSLELHKKDKREYIYTRDEFAAGKTHDDLWNAAQLQMVQEGKMHGFLRMYWAKKI
LEWSPSPEEALATAIYLNDKYELDGRDPNGYVGCMWSIAGIHDQGWAERSVFGKIRFMNYAGCQRKFKVDDFV
RKYPKAAANAREANAKKPGALNFPKAK
>Vitrella_brassi_CEL94135.1
MAAFLPSPPLRQLRSAAARSTSPSLQLRAASDKAATPGSARHRVVVWFRNDLRLHDHPGLSTAIEQVRRLGAD
KAEVLPVFCIDPRQFGISEWGSPKTGIFRARFLVESVADLKRQLRGLGSDLLVAIGQPETILPELATLPSEKG
QPERATVVASEEVTSEETAVQDAVRRNLRSQKAELSLHWSSTLHHIDDIRSIFDRDLRTMPTVFTPFRTKVEA
RLLPGATLPTPQQGDVGAAPKIDLTKACLDASFDLVPRVEDLPWAGGRPPEAHSWSQPAVLDFRGGEEEGLKR

LSHYVWSDHIATYFDTRNGMLGADYSSKLSPWLAHGCLSPRRIYEEVAKYESERVKNKSTYWLVFELLWRDYF
RFFALKNGRRVFQQRGIAFTSPPPPPIRQDGKAEELLDAWKNGQTGVPLVDANMRELAATGFMSNRGRQNVGS
FLALDLCLDWRYGADHFESLLLDYDVCSNWGNWVAIAGRIGGRLNRFNIPRQARTYDPQGEYVRVWCPELRNV
PTEYIFEPWTMPASVQQKSGCVIGVDYPQCVVGANHWPPSSASGGGRAARPPRSPSGRGRGGKKGRVAR
>Vombatus_ursinus_XP_027693622.1_cryptochro
MAATTVVTVPAAAAAPAPAPAPAPAPAPAPAPTPTPAPAQAPAPTPAPAQAPAPAPAPEEGASSVHWFRKGLR
LHDNPALQAALRGARCVRCVYILDPWFAASSSVGINRWRFLLQSLEDLDISLRKLNSRLFVVRGQPTDVFPRL
FKEWGVTRLTFEYDSEPFGKERDAAIVKMAKEAGVEVVTENSHTLYDLDRIIELNGQKPPLTYKRFQAIISRM
ELPRKPVSCVTSQQMERCQAEIQENHDDTYGVPSLEELGFPTDGLGPAVWQGGEMEALARLDKHLERKAWVAN
YERPRMNATSLMASPTGLSPYLRFGCLSCRLFYYRLWELYKKVKRNNTPPLSLYGQLLWREFFYTAATNNPKF
DRMEGNPICIQIPWDRNPEALAKWAEGKTGFPWIDAVMAQLRQEGWIHHLARHAVACFLTRGDLWLSWESGVR
VFDELLLDADFSVNAGSWMWLSCSAFFQQFFHCYCPVGFGRRTDPSGDYVRRYLPQLKGFPSRYIYEPWNAPE
SVQKAAKCIIGVDYPKPIVNHAETSRLNIERMKQIYQQLSRYRGLCLLASVPSCMEDLSNPMAETSMGQASGV
SSTGPKPLPCSPASPKRKLEAMEEASGEEHSKRARVMAALPVPELAGTV
>Vombatus_urs_XP_027701119.1_photolyase
MAPKKRSHSTSSEPEKMESQESKAKRKPLQKHQFSKSNMVQKEEEDKREGEKRGGAEGLHEVVRQSRLRTAPS
VLEFRFNKQRVRLISQDCHLHDQSQAFVYWMSRDQRVQDNWAFLYAQRLALKQKLPLHVCFCLAPCFLGATIR
HYDFMLRGLEEVAEECQKLCIPFHLLLGLPKDVLPAFVQTHGIGGIVTDFSPLLHHTQWVKDVQDALPRQVPF
VQVDAHNIVPCWVASDKQEYGARTIRHKIHDRLPHFLTEFPPVICHPYPSNIQAEPVDWNGCRAGLQVDRSVK
EVSWAKPGTASGLTMLQSFIAERLPYFGSDRNNPNKDALSNLSPWFHFGQVSVQRAILEVQKHRSRYPDSVTN
FVEEAVVRRELADNFCFYNKNYDKLEGAYDWAQTTLRLHAKDKRPHLYSLEQLESGKTHDPLWNAAQMQMVKE
GKMHGFLRMYWAKKILEWTRSPEEALEFAIYLNDRFQLDGRDPNGYVGCMWSICGIHDQGWAEREIFGKIRYM
NYAGCKRKFDVAEFERKYSSAD
>Vombatus_urs_XP_027728959.1_cry-1_X1
MGVNAVHWFRKGLRLHDNPALKECIEGADTVRCVYILDPWFAGSSNVGINRWRFLLQCLEDLDANLRKLNSRL
FVIRGQPADVFPRLFKEWNIAKLSIEYDSEPFGKERDAAIKKLASEAGVEVIVRISHTLYDLDKIIELNGGQP
PLTYKRFQTLISKMEPLATPVETITPEVMHRCVTPLSDEHDEKYGVPSLEELGFDTDGLPSAVWPGGETEALT
RLERHLERKAWVANFERPRMNANSLLASPTGLSPYLRFGCLSCRLFYFKLTDLYKKVKKNSSPPLSLYGQLLW
REFFYTAATNNPRFDKMEGNPICVQIPWDRNPEALAKWAEGRTGFPWIDAIMTQLRQEGWIHHLARHAVACFL
TRGDLWISWEEGMKVFEELLLDADWSINAGSWMWLSCSSFFQQFFHCYCPVGFGRRTDPNGDYIRRYLPVLRG
FPAKYIYDPWNAPEGVQKVAKCLIGVNYPKPMVNHAEASRLNIERMKQIYQQLSRYRGLGLLASVPSNPNGNG
GLMGYAPGENIPACSSSGGGAPVGASDGQILQACALPEPPTGASGGQQPGYSQGSGISHYSHEDSQQTYMLKQ
GRSCLGGGKRHRPEEETQSVGPKMQRQSVN
>Vulpes_vulp__XP_025859913.1
MAAAVVTAAAAAPVPTAGVDGASSVHWFRKGLRLHDNPALLAAVRGARCVRCVYILDPWFAASSSVGINRWRF
LLQSLEDLDTSLRKLNSRLFVVRGQPADVFPRLFKEWGVTRLTFEYDSEPFGKERDAAIMKMAKEAGVEVVTE
NSHTLYDLDKIIELNGQKPPLTYKRFQAIISRMELPKKPVGSVTSQQMESCRADIQDNHDDTYGVPSLEELGF
PTEGLGPAVWQGGETEALARLDKHLERKAWVANYERPRMNANSLLASPTGLSPYLRFGCLSCRLFYYRLWDLY
KKVKRNSTPPLSLFGQLLWREFFYTAATNNPRFDRMEGNPICIQIPWDRNPEALAKWAEGKTGFPWIDAIMTQ
LRQEGWIHHLARHAVACFLTRGDLWVSWESGVRVFDELLLDADFSVNAGSWMWLSCSAFFQQFFHCYCPVGFG
RRTDPSGDYIRRYLPILKGFPSRYIYEPWNAPESIQKAAKCIIGVDYPRPIVNHAETSRLNIERMKQIYQQLS
RYRGLCLLASVPSCVEDLSNPVAEPSSSQTGSMSSAGPRPLPSGPASPKRKLEAAEEPPGEELSKRARVAELP
TPELPCRDV
>Vulpes_vulp__XP_025866721.1
MGVNAVHWFRKGLRLHDNPALKECIQGADTIRCVYILDPWFAGSSNVGINRWRFLLQCLEDLDANLRKLNSRL
FVIRGQPADVFPRLFKEWNITKLSIEYDSEPFGKERDAAIKKLATEAGVEVIVRISHTLYDLDKIIELNGGQP
PLTYKRFQTLISKMEPLEIPVETITSEVVEKCTTPLSDDHDEKYGVPSLEELGFDTDGLPSAVWPGGETEALT
RLERHLERKAWVANFERPRMNANSLLASPTGLSPYLRFGCLSCRLFYFKLTDLYKKVKKNSSPPLSLYGQLLW
REFFYTAATNNPRFDKMEGNPICVQIPWDKNPEALAKWAEGRTGFPWIDAIMTQLRQEGWIHHLARHAVACFL
TRGDLWISWEEGMKVFEELLLDADWSINAGSWMWLSCSSFFQQFFHCYCPVGFGRRTDPNGDYIRRYLPVLRG
FPAKYIYDPWNAPEGIQKVAKCLIGVNYPKPMVNHAEASRLNIERMKQIYQQLSRYRGLGLLASVPSNPNGNG
GLMGYSPGENIPGCSSGSCSQGSGILHYAHGDSQQTHLLKQGRSSVGTGLSSGKRPSEEEDTQTIIGPKVQRQ
STN
>Wallemia_ichthy_XP_009268143.1
MPARVLHWFRTDLRLHDAPALLKGLELKPEVFFPVWTWDPYFNFECPVGPNRYKFLTESMKEIDKKLSKMGNK
LHVFRAAPADLFPVLFKQWNITHLVYEKDPAPYAIERDESIKKLAKDANVEVLDITGHTLYDIDAIVEKQKGK
TITSATAFKAAAKDMKIERPLQAPKDVPKADDLKLDAIKKEVKKVKSDHDVNEEHRKGKMTAYDSVTGPKDSL
EPPSLEELGLDSEDATTQIPGGEDEALRRLDKWIKDKDATATFRKPQTSPSEYDPPATTQLSPYIKFGALGVR
EFYWRVVDLMENYDGETSSEPENLPGQLIFREMYFAAQLGIGKPFGQIRGNKICRVIDWKLCNAYDEDGEKIF
PRPKGSKEDEEALEKWKHGNTGFPWVDAAMRQLKQEGWMHHLARHCVAGFLTRGQLYISWERGAEVFDRYLVD

QDPASNAGNWMWLSCSAFFHQYYRNYGMTTFPSKYDKTGKLVRKYVPELKEYPDKYIYKPWEAPEDVQKKAGC
KIGKDYPEPMIDEKEANKECMARMKEQYAKKLYGDSLLKRKAK
>Wallemia_ich_XP_009266014.1_photolyase
MGNLKKEPSSPIQEAGVKRAATRSPSPHLHENKKQTQEKIKKEENLGGYPSYGRVKACSIAKADRIDENPPVL
RLNKAIQERTKQAHSDDEKSVVFWMRMHDLRIVDSRGLTIAYEYAKEHNIPLIIIHVFSPQDYEAHDRSCRRI
DFVLRKLKSLKEELEEMNIPLYTFTVEQRQHIPQKVIDFVKQYNSKHLYGNIEYEIDELFRDMQLINLANEQE
VEVQLEHDNMVVPPFKVLTKTTSKPPMTFTAYLKGWQKIVLEDPTHRDETERIGANNISAKEDYKALFEKQVP
DEIAGFECHDKDKMRELWPVGRTTASEILDRFLHYKNREEATESTPLGEGAEKDDRNSRIKQYADGRNITTGE
TTSKISPYLASGQISVRQCFNRAQKLKSGYNMDITRESGVGMWSQEVIWKDFYQNVLCVQPRVSMGHPFLRQY
RFVEWEYNQEHLDAWHQGKTGWPIVDAAMRQLTTTGWMHNRMRMTVASYLCKDLMLDWREGERIFAKELIDHD
LGSNNGGWQWSAGTGTDAQPYFRIFNPISQSQKADPSGEYIHHWLPELRDIKPPALFEPSKHLSKNEFQKLNY
PKPLVDHKFARDRALSRYKKALGKE
>Xenopus_lae_NP_001081129.1_cry-1
MGVNAVHWFRKGLRLHDNPALRECIQGADTVRCVYILDPWFAGSSNVGINRWRFLLQCLEDLDANLRKLNSRL
FVIRGQPADVFPRLFKEWKITKLSIEYDSEPFGKERDAAIKKLASEAGVEVIVRISHTLYDLDKIIELNGGQP
PLTYKRFQTLISKMDPLEIPVETITAEVMEKCTTPVSDDHDEKYGVPSLEELGFDTEGLPSAVWPGGETEALT
RLERHLERKAWVANFERPRMNANSLLASTTGLSPYLRFGCLSCRLFYFKLTDLYKKVKKNSSPPLSLYGQLLW
REFFYTAATNNPRFDKMDGNPICVQIPWDRNPEALAKWAEGRTGFPWIDAIMTQLRQEGWIHHLARHAVACFL
TRGDLWISWEEGMKVFEELLLDADWSVNAGSWMWLSCSSFFQQFFHCYCPVGFGKRTDPNGDYIRRYLPILKG
FPPKYIYDPWNAPETVQKAAKCIIGVNYPKPMVNHAEASRLNIERMKQIYQQLSRYRGLGLLASVPSNPNGNG
GLMSYSPGESMPGCSNNGGGQMGAIEGSSASNPNPNQGEVLPGTSGLQGYWQGSSILHYSHSDNQQSYLMQAR
NPLHSVVSSGKRPNPEEETQSVGPKVQRQSTH
>Xenopus_lae_NP_001081421.1_6-4_photol
MRHNSIHWFRKGLRLHDNPALLAAMKDCAELHPIFILDPWFPKNMQVSVNRWRFLIDALKDLDENLKKINSRL
FVVRGKPAEVFPLLFKKWKVTRLTFEVDIEPYSRQRDAEVEKLAAEHDVQVIQKVSNTLYDIDRIIAENNGKP
PLTYVRFQTVLAPLGPPKRPIKAPTLENMKDCHTPWKSSYDEKYGVPTLEELGQDPMKLGPHLYPGGESEALS
RLDLHMKRTSWVCNFKKPETEPNSLTPSTTVLSPYVKFGCLSARTFWWKIADIYQGKKHSDPPVSLHGQLLWR
EFYYTTGAGIPNFNKMEGNPVCVQVDWDNNKEHLEAWSEGRTGYPFIDAIMTQLRTEGWIHHLARHAVACFLT
RGDLWISWEEGQKVFEELLLDADWSLNAGNWLWLSASAFFHQFFRVYSPVAFGKKTDKNGDYIKKYLPILKKF
PAEYIYEPWKSPRSLQERAGCIIGKDYPKPIVEHNVVSKQNIQRMKAAYARRSGSTEGVDKDSGQNNKKGGKR
KVAAGTSVAELFKKK
>Xenopus_lae_NP_001084438.1_cry_DASH
MCVPSRVIICLLRNDLRLHDNEVLHWAHRNADQIVPLYCFDPRHYVGTHYFNFPKTGPHRLKFLLESVRDLRI
TLKKKGSNLLLRRGKPEEVIEDLVKQLGNVSAVTLHEEATKEETDVESAVKQACTRLGIKYQTFWGSTLYHRE
DLPFRHISSLPDVYTQFRKAVETQGKVRPTFQMPDKLKPLPSGLEEGSVPSHEDFDQQDPLTDPRTAFPCSGG
ESQALQRLEHYFWETNLVASYKDTRNGLIGLDYSTKFAPWLALGCVSPRYIYEQIGKYEKERTANQSTYWVIF
ELLWRDYFRFVALKYGRRIFFLRGLQDKDIPWKRDPKLFDAWKEGRTGVPFVDANMRELAMTGFMSNRGRQNV
ASFLTKDLGIDWRMGAEWFEYLLVDYDVCSNYGNWLYSAGIGNDPRENRKFNMIKQGLDYDSGGDYIRLWVPE
LQQIKGGDAHTPWALSNASLAHANLSLGETYPYPIVMAPEWSRHINQKPAGSWEKSARRGKGPSHTPKQHKNR
GIDFYFSRNKDV
>Xenopus_lae_NP_001089127.1_CPD_photol
MSHKGKQSKPKDGESGSEKSDGSQKKGMGIDAKTKTQTITKSPGKRKIPGKVLSDSSGDSSGVDAKKKKTMVE
QKGEKDVREAVKISRMAAAPSVSEFKFNKKRARLLSTEADLKEDAQGIVYWMSRDQRVQDNWAFLYAQRLALK
QKLPLHVTFCLVPKFLDATIRHYGFMLKGLQEVAEECKELNIPFHLLIGYAKDILPNFVKDHAIGGVVTDFSP
LRVPLQWVEDVGKCLPEDVPLVQVDAHNIVPCWVASIKQEYGARTIRRKVHDQLSQFLTEFPPVIKHPYEPKF
EAETVDWDKCYKSLEVDRTVGEVEWAKPGTNAGFNVLQSFISERLKHFNSDRNNPNQNALSNLSPWFHFGQLS
VQRAILEVQKYRSKFKESVDSFVEEAVVRRELADNFCFYNKNYDKIEGAYDWAKNTLKDHAKDKRTHLYTLEK
LEAGKTHDPLWNAAQLQMVHEGKMHGFLRMYWAKKILEWTSSPEEALHFSLYLNDRYELDGRDPNGYVGCMWS
ICGIHDQGWAERAVFGKIRYMNYQGCKRKFDVAQFERRYHPKKFSQ
>Xenopus_laevis_AAK94667.1_cryptochro
MEGKPSVSSVHWFRKGLRLHDNPALLAALRGANSVRCVYILDPWFAASSSGGVNRWRFLLQSLEDLDSSLRKL
NSRLFVVRGQPADVFPKLFKEWGVSRLTFEYDSEPFGKERDAVIMKLAKEAGVEVIVENSHTLYDLDRVIELN
GHSPPLTYKRFQAIISRMELPRRPAPSVTRQQMEACRAEIKRNHDETYGVPSLEELGFHSENKGHAIWPGGET
EALARLDRHLERKAWVANYERPRMSANSLLASPTGLSPYLRFGCLSCRLFYYRLQELYQKVKKNSPPPLSLYG
QLLWREFFYTAATNNPKFDQMEGNPICVQIPWDKNPKALAKWTEGKTGFPWIDAIMTQLRQEGWIHHLARHAV
ACFLTRGDLWNSWECGVKVFDELLLDADFSVNAGSWMWLSCSAFFQQFFHCYCPVGFGRRTDPSGDYVKRYLP
VLKAFPSRYIYEPWSAPESVQKEAKCIIGIDYPKPIVNHAEASRMNIERMKQTYQQLSHYRGLCILASVPSCV
EDLGGPVTDSSHNPAEAAPKQSLCNADSPKRKLEGSEEASHVKVRVRSVPVMRRPENDF
>Xenopus_laevis_XP_018100774.1

MPHRTIHIFRKGLRLHDNPTLVAALETSDIIYPVYILDKNFMTSSSVIGSKRWNFLLQSIEDLHCNLQKLNSC
LFVIQGDYQSVLREHVQKWHITQVTFDLEIEPYYKGMDERIRAMGQELGFDVVSKVAHTLYDVKSILALNYGK
PPLTYKNFLRVLSVLGDPDKPARQITLEDFIKCTTPTEFAAEEYYRIPKPEDLGICRDCAPNWKGGESEALCR
LEQHLEKQGWVANFQKPQTVPNSLLPSTTGLSPYFSFGCLSARVFYHRLSNIYAQSKNHSLPPVSLQGQLLWR
EFFYTAASSTPNFTHMVGNPICLQIEWYKNEEQLQKWREGKTGFPWIDAIMAQLHEEGWIHHLARHAVACFLT
RGDLWISWEEGMKVFEELLLDADYSINAGNWMWLSASAFFHHYTRIFCPVRFGRRTDPEGNYIRKYLPVLKNF
PAKYIYAPWTAPEEIQKQSGCLIGKDYPLPMVDHTTASEHNLQLMRLVREAQQKTAQLTTDIADDPMELNLKH
PFKTDKESVLENKVKRRCLEETAGEDGNRQSDLVQSLDPCEVKVC
>Xenopus_lae_XP_018113364.1_photolyase
MDLEAVERAHINDIVRQLETGIVQTDEFLCLVLSVLGNRRTFWHLPAIIQSLREKEPAMYRELMDLHAHYFRK
EPTDLETDLELAIALSMHEQNQLTDTASSEMSPTAKISFADAAKSSCYSHKYSEKTSSSKTEIDKLKHNVAAM
HLSQETNRCQAIRQEKTLSSCYNGQPEPEDCVLKSVDCEEVFHVEASVPRESKAKQSRRRRKKTKSAPSGGLV
AMKPVLLWFRRDLRLHDNPALVSALEHGVPVIPVFLWCINEETGQNFTLATGGATKYWLHHALLELNQSLIQK
FGSHVIFRVAQSCEKELVSLVHETGAGTVIVNAVYEPWLKERDDFISENLQKNGVEFKKHHSYCLYEPGSIST
EGVGLRGIGSVSHFMSCCKRNQSAPIGIPLDAPMILPTPCKWPDSDDLDKMKLAKMPSRKDGTLIDWAMTIRE
SWDFSEDGAYKCLSNFLEDGVKHYEKESGRADKPYTSHISPYLHFGQISPRTVLHEAYFTKKNVPKFLRKLAW
RDLAYWLLLLFPDMPSEPVRPAYKSQRWSSDLNHLRAWQKGLTGYPLVDAAMRELWLTGWMCNYSRHVVASFL
VAYLHIHWVHGYRWFQDTLLDADVAINAMMWQNGGMSGLDHWNFVMHPVDSALTCDPYGSYVRKWCPELAGLP
DEYIHKPWKCAPSQLRRAGVILGQNYPHRIVLDLEERREQSLKDVVEVRKKHLEYLDEVSGSDMIPIPDQLLA
LTLGRPNGDDEVVRNRTSSFLLPVITRKEFKYKTLQPEAKDNPYSTVLKGYVSRKRDETIAYMNEKHFTASTI
HEGAQRHERTERTSRLMEGLPAPSEPKNKSRRTPRKDPFSIIPPSYLHLAN
>Xiphias_glad__XP_039979368.1
FFFLSCALHQLRKRCSPPLSLFGQLLWREFFYTAATNNPNFDRMEGNPICVQIPWDQNPEALAKWAEGRTGFP
WIDAIMTQLRHEGWIHHLARHAVACFLTRGDLWISWESGMKVFEELLLDADWSVNAGSWMWLSCSAFFQQFFH
CYCPVGFGRRTDPSGDYIRRYIPILKDYPNRYIYEPWNAPEPVQKAANCVVGVDYPKPMINHAEGSRLNIERM
KQVYQQLSHYRGLS
>Xiphias_glad__XP_039982721.1
MSTSRTIICLLRNDLRLHDNELFHWAQRNAEYIVPLYCFDPRHYVGTYNYNLPKTGPFRLRFLLESVRDLRNT
LLNKGSNLVVRRGKPEDVVAELIKQLGSVSTVAFHEEVTSDELNVEKKVKDVCAQMKVKVHTCWGSTLYHRDD
LPFHHISRLPDVYTQFRKAVETQSRVRPVFPTPEQLKPLPPGLEEGALPTAEDLQQTEPLTDPRSAFPCSGGE
SQALARLRHYFWETDAVATYKETRNGLIGVDYSTKFAPWLAMGCISPRYIYHQIKQYERERTANQSTYWVIFE
LMWRDYFKFVGVKYGSRLFQVKGLQDKSVPWKMDMKLFNAWKEGRTGVPFVDANMRELAMTGFMSNRGRQNVA
SFLTKDLGLDWRMGAEWFESLLIDHDVCSNYGNWLYSAGIGNDPRENRKFNMIKQGLDYDNNGDYVRQWVPEL
QGIRGADVHTPWTLSTAALSHAHVSLGETYPTPIVIAPEWSRHINTKSSGTGPSPRGKKGPSHTSKQHRDRGI
DFYFSKGKNL
>Xiphias_glad__XP_039988261.1
MVVNTIHWFRKGLRLHDNPSLRDSIREADTLRCIYILDPWFAGSSNVGINRWRFLLHCLEDLDASLRKLNSRL
FVIRGQPTDVFPRLFKEWQITRLSYEYDSEPFGKERDAAIQKLANEASVEVMVRISHTLYDLDKIIELNGSQP
PLTYKRFQALINRMDAVELPAETITSEVIKKCATPIGEDHDDKFGVPSLEELGFETEGLTTAVWPGGETEALM
RLERHLERKAWVANFERPRMNANSLLASPTGLSPYLRFGCLSCRLFYFKLTDLYRKVKRNSTPPLSLYGQLLW
REFFYTTATNNPCFDKMEGNPMCVQIPWDRNPEALAKWAEGRTGFPWIDAIMTQLRQEGWIHHLARHAVACFL
TRGDLWISWEEGMKVFEELLLDADWSVNAGSWMWLSCSSFFQQFFHCYCPVGFGRRTDPNGDYIRRYLPVLRG
FPAKYIYDPWNAPEEVQKAAKCIIGVHYPKPMVNHAEASRINIERMKQIYQQLSCYRGLGLLATVPANPNSGL
SGSNVAGVNKGTSQVPGGSSEELSVQEGTSRTERGHSTQKRRREEAPSASSSKSWRQSK
>Xiphias_glad__XP_039988503.1
MACMAPSAADAEDSKALVRRLLREVLVGREDPEGFFAMCVSVLGHQETRSRFLSLIRPLSTANSSLHTDLTSI
YREYFSKTEDDELELALALSVLEIKDHQLSSPSQESLLQQLGDRPNQSSSVQLTSASQPQGGSHTRLSDLASG
RKYTHSAQTGPWGKVCPPQTTRETELQKGTHVVKCNKETPGTSQTVSVSRPSASLSKQETVIEGDQMIEEGDL
NQSERARRSKNRRQRRKGGGQQVVGLPRSPSAPPPVLLWLRRDLRLCDNPAVISSLEVGAPVIPVFIWSPEEE
EGPGITVAVGGACKYWLHQALSCFCSSLERIGSHLVFLKANGEGNGVGSSLRTLKELIKETGTRTVMANALYE
PWLKERDDAVVSALQKDGVECRMFHSNCLRDPYSVSTEGVGLRGIGSVSHFMSCCRQNPGSALGVPLDSPVSL
PTPAHWPQGVSLDTLGLARMPRRKDGTTIDWAANIRKSWDFSEEGAQAQLEAFLHDGVYRYEKESGRADSPNT
SSLSPYLHFGQLSPRWLLWDAKGARCRPPKFQRKLAWRDLAYWQLTLFPDLPWESLRPPYKALRWSSDRGHLK
AWQRGQTGYPLVDAAMRQLWLTGWMNNYMRHVVASFLIAYLHLPWQEGYRWFQDTLVDADVAIDAMMWQNGGM
CGLDHWNFVMHPVDAAMTCDPYGSYVRKWCPELADLPDELIHKPWKCPASMLRRAGLVFGQTYPDRIITDLEE
RRSQSLQDVALVRKEFGQYVDKRTGCDLVPLPPRLVSEALGLSHRNGGVVTEGKQFLLPVITRMEFKHQLEDP
DADAASNPYNAVLKGYVSRKRDETIAFLNERDFTASVMYEGAQRKERLESDYRRMEGLPRPPAPRGRARRTPT
AKDRFSIVPGGAVTSLR
>Xiphias_glad__XP_039999756.1

MLRGIRRRRLLKLVVHSDRWPSSILSRLPESFTSETIMSDKKRKAASAPAGKEPNAKQQKLGPMKAEKEERAG
GWLQHLVKQQRSEKKDMKFNKKRLRFISDAEKIKQGSEGVLYWMSRDHRVQDNWALIQAQRLAAKENLPLHIC
VCLVVPKSELSTLRHYSFMLKGLKEVAKECKALDIQFHLLDGSAGEVLPGFVSDRSLGAVVTDFSPLREPLGW
LEDVKKRLPESIPLIQVDAHNIVPCWVASPKLEYSARTIRGKITNLLPEFLTDFPLVEKHPYTATRTAKPIDW
DKILASLEVDRAVGEPEWAKPGTMAGMAMLESFIDVRLKLFGTQRNDPNAAALSQLSPWIRFGHVSAQRMALQ
VQRSGKNASQSISSFIEELVVRRELTDNFCFYNKKYDSVEGAYEWAQKTLKDHAKDKRPYLYTRQQLEKAKTH
DKLWNAAQYQMVTEGKMHGFLRMYWAKKILEWTSSPEEALSIALYLNDRYELDGQDPNGFVGCMWSICGIHDQ
GWAERPVFGKIRYMNYKGCTRKFDVPRFERKYCPKNL
>Xiphias_glad__XP_040011325.1
MAHTCIHWFRKGLRLHDNPALVSALRDCKHLYPVFILDPHLQSDTHVGVNRWRFLVGALKDLDCSLRKLNSRL
FVVRGKPEDVFPELFNKWKVTKLTYEHDTEPYSLSRDKKVTALAKEHGVEVVYKISHTLYDIDRIIEENNGKA
PLTYNRMQAIVKTLGPPKKPLPAPTMEDMKDVKTPCSESHEKKYGIPALEELCQDTAAVGEEPFPGGEQEALR
RLEEHMKRRDWVCRFEKPQTPPNSLSPSTTVLSPYVTFGCLSARTFWWKLTEVYQGKKHSDPPVSLHGQLLWR
EFFYTASVGIPNFNKMEGNPVCTQVDWDTNPDYLAAWREARTGFPFIDAIMTQLRQEGWIHHLARHAVACFLT
RGDLWISWEKGQKVFEELLLDGDWALNAGNWQWLSASAFFHQYFRVYSPVAFGKKTDKNGDYIKKYLPVLKKF
PAEYIYEPWKAPRSVQQAAGCIVGKDYPRPIVQHEVISKKNIQRMKLAYAKRSPDPTESPSKKQGVKRKASSV
VDMLKKKERRE
>Xiphophorus_macu__XP_005795951.1
MVVNSVHWFRKGLRLHDNPALQEALSGADTVRCVYVLDPWFAGAANVGINRWRFLLESLEDLDNSLKKLNSRL
FVVRGQPTEVFPRLFKEWNVTRLTFEYDPEPYGKERDGAIIKMAQEFGVETVVRNSHTLYNLDRIIEMNNNNP
PLTFKRFQTIVSRLELPRRPLSSVSQQQMNQCLGKISDNHDQLYSIPSLEELGFRTEGLPPAVWRGGESEALD
RLSRYLDKNVWVANTRVKTCSLYASPTGLSPYLRFGCLSCRMLYYNLREIYMKLCKRSSPPLSLYGQLLWREF
FYTAATNNPNFDRMEGNPICVQIPWDQNPEALAKWAEGRTGFPWIDAIMTQLRQEGWIHHRARHAVACFLTRG
DLWISWESGMKVFEELLLDADWSVNGGSWMWLSCSAFFQQFFHCYCPVGFGRRTDPSGDYIRRYIPILKDFPN
RYIYEPWNAPESLQKAANCVVGVDYPKPMINHAEGSRLNIERMKQVYQQLSHYRGLSLLASVPTIQEEAEPPM
TDESQTSSGPDSPPRVLADMEAAVCLMAPDSSTSAPDQDNVSLHRVFQDHCTSSGWPSSPASSPPQSPAPRSK
PGSPSSPCPSPSASPSSLPPRRKGVAHKGRRSQRRRERPSCPMAKEEDEEERMQEEVDEERMEEETAAAAQQ
>Xiphophorus_macu__XP_005797880.1
MSASRTVICLLRNDLRLHDNEVLHWAQRNAERIVPLYCFDPRHYVGTYNFNLPKTGPFRLRFLLESVRDLRNT
LLSKGSNLVVRKGKPEEVVADLIKQLGSVSSVAFHEEVASEELNVEKKVKEVCAQLNVKVHTCWGSTLFHRED
LPFHHISRLPDVYTEFRKAVESQSRVRPVLLTPERLNPLPLGLEEGAIPTAEDLQQTEPVTDPRSAFPCTGGE
SSALARLKHYFWDTDAVATYKETRNGLIGVDYSTKFSPWLALGCISPRYIYHQIKKYERERTANQSTYWVIFE
LLWRDYFKFVGIKYGNKMFQVKGLQGKSVPWKMDTKLFDAWKEGRTGVPFVDANMRELALTGFMSNRGRQNVA
SFLTKDLGLDWRLGAEWFEYLLIDHDVCSNYGNWLYSAGIGNDPRENRKFNMIKQALDYDNNGEYVRRWVPEL
GTIRGADIHTPWNLSSAALSHSGVSLGETYPNPIVTAPEWSRHFSKKPGGAGPSPRRKKGPSHTPKQHRDKGI
DFYFSRSKNL
>Xiphophorus_macu__XP_005802444.1
MSEKKRKAPTAAGGKEPSAKQPKLEKAEKAAGWLQSLVKRQREDTKDTKFNEKRQRFLTDTEKMKQGSEGVLY
WMQRDQRVQDNWALIRAQQLAAKEELPLHVCFCLVVPKSELSTLRHYAFLLKGLQEVATDCKRLNVQFHLLRG
APGEVLPGFVSDRGLGAVVTDFSPLREPQQWLNDVKKKLSKDIPLIQVDAHNIVPCWVASPKLEYAARTIRGK
ITKLLPEFLTDFLPVEKHQHTATRTAKPVDWEQTLASLDVDRTVGEPQWAKPGSEAGMAMLESFIDVRLKLFA
TQRNDPNTAALSQLSPWIRFGQLSAQRVALQVRRCGKNASESVAAFIEELVVRRELTDNFCFYNEKYDKVEGA
YEWAQKTLKDHAKDKRDNIYTREQLEKAQTHDKLWNGAQYQMVAEGKMHGFLRMYWAKKILEWTRSPEEALSI
ALYLNDRYELDGQDPNGFVGCMWSICGIHDQGWAERPVFGKIRYMNYKGCLRKFNVAQFERKYDPKKA
>Xiphophorus_macu__XP_005815705.1
MKNSDSMGVNTIHWFRKGLRLHDNPALRDSIRGTDTLRCIYILDPWFAGSSSVGINRWRFLLHCLEDLDASLR
KLNSRLFVIRGQPTDVFPRLFREWNIARLSYEYDSEPFGKERDAAIQKLANEAGVEVTVRISHTLYNLDKIIE
LNGGQPPLTYKRFQAIINQMDAVELPAETITSKVIKNCTTPITHDHDDKFGVPSLEELGFETEGLSTAVWPGG
ETEALMRLERHLERKAWVANFERPRMNSNSLLAAPTGLSPYLRFGCLSCRLFYFKLTDLYKKVKKNSTPPLSL
YGQLLWREFFYTTATNNPCFDKMEGNPVCLQIPWDRNPEALAKWAEGQTGYPWIDAIMTQLRQEGWIHHLARH
AVACFLTRGDLWISWEEGMKVFEELLLDADWSVNAGSWMWLSCSSFFQQFFHCYCPVGFGRRTDPSGDYIRRY
LPVLRGFPAKYIYDPWNAPEDVQKAAKCIIGTNYPRPMVNHAEASRVNIERMKQIYQQLSCYKGLGLLATVPA
NPNNGANGSNVGGANAYSAVRSFESPLLQEGTSQTVGRRQSSQKRRHEETEPECSFKSWKHSK
>Xiphophorus_macu__XP_023182506.1
MPHSAAGGQAAVRQMLREVLVGREDPEGFFAMCVSILGHQETRLQFLSIIKPLSTANIRLHSVLSFIYQGYFS
KMEDDELELALALSLLDMKDQQISTPNQMTPHPAVGGNASSVLCNSNAKTQGNSCTQQEDVPGREKSAFVAQS
MSGSKENFRENQTYKSTHVEGVDNKSLGTSQALYVPDSSTSFSITNTASVSGQVMKKEDLSKKPRCSKNRRQR
RKVAAQQIVGLKRSPSAPPPVLLWFRRDLRLCDNPALIRCLELGAPVIPVFIWSPEEEEGSGVTLAMGGACKY
WLHQALSCFCASLGRIGSHLIFLKASGDENKDGSSLHTLKTLVKETGAQTVMANALYEPWLKERDDVVFSALH
KEGVECKMFHSYCLRDPFSVSTEGVGLRGIGSVSHFMNCCRQNPGASIGVPLDPPVSLPSPTRWPQGVPLDKL

GLARMPRRKDGTIIDWAENIRKSWDFSEDGAHARLEAFLHDGVYRYEKESGRADAPNTSCLSPYLHFGQLSPR
WLLWDAKGARCRPPKFQRKLAWRDLAYWQLTLFPDLPWESLRPPYKALRWSTDCRHLKAWQKGKTGYPLVDAA
MRQLWLTGWMNNYMRHVVASFLIAYLHLPWQEGYRWFQDTLMDADVAIDAMMWQNGGMCGLDHWNFVMHPVDA
AMTCDPYGSYVRKWCPELADLPDELIHKPWKCPASILRRTGVVFGQTYPERIVTNLEEQRSKSLQDVALVRKQ
FEQYVDKRSGCDLVPLPKRLVSEALGVSHWDGAVVTEGKQFLLPVITRMEFKHQLEDPDADAASNPYNAVLKG
YVSRKRDETIAFLNETDFTASVMYEGAHRQERLERDYRRMEGLPPAPSNRGRARRTSTAKDKFSVVPGGAIAS
RN
>Xiphophorus_macu__XP_023187633.1
MAHVCIHWFRKGLRLHDNAALMAALKDCKELYPVFILDPYLHHTQVGINRQRFLIGALKDLDCSLRKLNSRLF
VLRGKPEDVFPKLFNMWNVTKLTYEYDTEPYSLRRDEKVASLAKEHGVDVIYKISHTLYNIERIIEENNGKPP
LTYKRLQAIVQNLGPPKKPIPAPTLENMRDVKTPHSEKYEDKYRIPTLEELQLDSKFLGEELFPGGEQEALRR
LDEHMKRTGWVCSFEKPQTSPNSLSPSTTVLSPYVTFGCLSARTFWWRLTEVYRGRKHSDPPVSLHGQLLWRE
FFYTCSVGIPNFNKMNQNPVCTQVDWDTNQRNLAAWREARTGFPFIDAIMTQLRQEGWIHHLARHAVACFLTR
GDLWISWEEGQKVFEDLLLDGDWALNAGNWQWLSASAFFHQFFRVYSPIAFGKKTDKNGDYIKKYLPLLKKFP
PQYIYEPWKAPRSVQQAAGCIVGKDYPHPIVQHEQISKKNIQRMKEAYAKRSSDNNESPIKKQGVKRKTPSVI
DMLQKKIRKE
>Xylona_hev_XP_018186447.1_cry_
MTSSRILIYVLRRDLRLADNPILHEVSRVLRSSQNASFTHLLPLYVFPAQQVEVSGFLSPGTTDSPYAEARSK
VGGFWRTGHHRVKFLAESVWDTKSSLEKVGSGLEIRVGVTSEVIKDMLEAYKTLDGEVSAVWMTEEEGWEEKQ
DEENVRTVVEAEGKEFQLWKDEKYFIDDIDLGPSRDIPFQDPKELPDVFTSYRKMVEPLREAPRKSVPPPSQL
LPLPDSIPPQSSPFVQPSSLENLIAALEKPLHNDDELEHPPKWPNGANSAFPFSGGEKAGHERIKHLVTSGSM
SAYKDTRNGLLGEDFSTKLAAWLALGCITARQVHEYLLEFEDGSSALGSDAIGYGNGENKGTAAVRFELLWRD
YMRLCTRKFGARLFHIDGFRHGKSVPWKYVDGAERKNKRRHSNSAVDAAEALQRFLEGRSGTGLIDASMRELY
LTGYTSNRARQNVASFLAKHLRIDWRLGAEWYETQLVDYDVSSNWGNWQYNAGVGNDPRGEGRVFNPIKQAFD
YDPQGEYVKTWVPELRDVEDPLAVFQAWRLPVDQRKALGLEGVEWVENPLKRIDFYVSKPSKGSGTFASGNRN
RQQEGQGRNDLNDEANAAEEAQGTSRGSSNHRRGRGWGRARGRGRGRSRGRGGGRGGSKGHTTDETPRRKQNM
GRMDNAATLEEAIA
>Xylona_hev_XP_018189085.1_cry-2
MTKPKPRVIYWFRTDLRLHDSPALKAALDLEPECLYPIWTWDPHYVVYRARVGPNRWQFLLDCQSDLSASITKL
NKKSKLFVIRESPTTLFPKLFKAWNISHLVFEKDTDAYARDRDNEVTEIAKKAGVQVITKVGRTLYDPDELVK
QNHGKPTMSISQVIKAGEQIGQIPRPIPAPASLPDPGDTSLNFKQDHPSSEPDINAPQRNSSDQSYAHLAGPQ
GDFSVPTYDELGLKPSTTPHHGGETAALAALDKIISNTKYTATFEKPSTAPTAFEPQSTTLLSPHLHFGSLSC
REFYWRAQDVVDNFKQGTASHPPTSLTGQLLFRDMYFGAQAALGHKFGQAYGNPSCRSVPWHLPSKVNEDTGL
VVRDGGGGGGVEYSIDSSTAETWFQRWKYGCTGFPWIDALMRQLRQEGWIHHLGRHAVACFLTRGGCYVDWER
GAAVFEEWLIDHEPACNIGNWQWLSCTAFYAQFYRCYSPISFPQKWDKDGAFVRKYVPELANFPAKYIYEPWK
APIADQKAAGCLIKSIDEEGEDGELDLSPWKKEIVAEKHEANGAQNGDEKKEKESMNIYPKPMFDFPSRRTTC
INALKNAYRVGLYGNDQKVLDGTWRDLFPDEAEGPTSTADVEAMHDADAHGEFVHDAEGHEGLHEKDHSPVGR
KRKEHGQGTLDGHVKRTKT
>Xylona_hev_XP_018189979.1_CDP_photol
MPPSRTSNRKRQAGSKSASPEPPSKRRANGNSSPKKGSTQDDDDNNGYVLREFYPPEMSNKRCLEYAHDEITR
PIEELQTAIEETAEARKNIKVKNSVVHWFKRDLRTQDNKSLALASEKAKDAGVPLIALFIVSPQDYQAHLTSS
PRVDFELRTLQILKKDLAQLDIPLYVETVEKRKKVPSRVVELCNEWGASHVFCNIEYEVDELRREAKLTRLCA
EKGIEFSAVHDTCVVPPGELASQQGKQYAVYSPWFRAWIAHLHSNPDLLEKYDSPGKNSKDSREKFKNLFDSS
IPDAPANKMLNDEEKKRFHSLWPAGEHEAHDRLDKFLNQKIGHYTETRNYPAANSTALLSVHFSSGTLSARTA
VRQARDANTTKKLDGGNSGITGWISEVAWRDFYKHVLVHWPYICMNKPFKPEYANIEWSYSEDNFQKWCQGQT
GYPIVDAAMHQLNHMGYMHNRCRMIVASFLSKHLLIDWRKGERYFMEHLIDGDFASNNGGWGFSSSTGVDPQP
YFRIFNPLMQSERFDPEGEYIRKWLPELKDVEGKAIHAPYERGAEKIAAKNNYPKPMIEHKFGRDRALSKYKE
GLGRDTA
>Zootermopsis_nev_XP_021920747.1_cry1-like
MMSEGKAKHTVHWFRKGLRLHDNPSLREGLKCATTFRCVFILDPWFAGSSNVGINKWRFLLQCLEDLDQSLRR
LNSRLFVIRGQPADALPKLFKEWDTTDLTFEEDPEPFGRVRDQNIMAMCKELGITVMSRVSHTLYKLQNIIEK
NGGKAPLTYHQFQKVVASMGSPPQAEPTINQKFIDSAYTPIAEDHDDKYGVPTLEELGFDTDGLLPPVWQGGE
SEALARLERHLERKAWVASFGRPKMTPQSLLASQTGLSPYLRFGCLSTRLFYYQLTDLYKKIKKACPPLSLHG
QLLWREFFYCAATKNSNFDRMNGNPICLAKWANGQTGFPWIDAIMTQLREEGWIHHLARHAVACFLTRGDLWI
SWEEGMKVFEELLLDADWSVNAGMWMWLSCSSFFQQFFHCYCPVRFGRKADPNGDYIRKYLPVLKNFPTRYIH
EPWNAPEAVQKAAKCIIGKEYSLPMVNHAVASRTNIERMKQVYQQLSKYRGPGLLATVPTSQSQSCNTLNHLS
VQKSLKTSPPSETKWETSPSDEIIGHSQVICDEELKNGIQIHRYKSHRRDESE
>Zootoca_vivipara_XP_034953412.1_LOW_QUALIT
MATAFPMGSGSRTSGGRDAHITGGCSVHWFRRGLRLHDNPALQAAIREGGSVRCIYILDPWFAASSAVGINRW
RFLLQSLEDLDNSLQKLNSRLFVVRGQPTDXFPRLFKEWGVTHLTFEYDSEPFGKERDAAIVKLAKEAGVAVI

TENSHTLYDLDRIIELNGHKPPLTYKRFQAIISRMDLPKKPVATVTNQQMEICRTEIQENHDETYGVPSLEEL
GFPTEGLAPAVWQGGETEALTRLDKHLERKAWVANYERPRMNANSLLASPTGLSPYLRFGCLSCRLFYYRLWE
LYRKVKRNSTPPLSLYGQLLWREFFYTAATNNPQFDRMEGNPICIQIPWDRNPEALAKWAEGKTGFPWIDAIM
TQLRQEGWIHHLARHAVACFLTRGDLWISWESGVRVFDELLLDADFSVNSGSWMWLSCSAFFQQFFHCYCPVG
FGRRTDPSGDFIRRYLPKLKGFPSRYIYEPWNAPESVQKAAKCVIGVDYPKPMVNHAEASRLNIERMKQIYQQ
LSRYRGLCLLASVPSSMEDLSAPVTDSSASQGSCTMPRQSHCGQTSPKRKLNVVEEHNEELYKRAKVMPAQIP
KNLXQEIVHDAVSKLHQ
>Zootoca_vivipar_XP_034985269.1
MAALRTALCVLRNDLRYHDNEVLHWAHSNADFVIPLYCFDARHYAETHCYQFPKTGPYRLKFLLESVEDLRET
LKKKGSNLVVRKGKPEDVICDLIAQLGSVAAVVFHEEATKEELDVEKALLQVCSEQGVVAQKFWGSTLYHRDD
LPFKHISRLPDVYTQFRKDVESQVKVRPLLQMAAQLKPLPPGVEEGTIPKVEDFGMQDPLTDHRTAFPCSGGE
TQALMRLQHYFWDTNLVASYKETRNGLIGVDYSTKFAPWLALGCISPRYIYEQIRKYEKERTANQSTYWVLFE
LVWRDYFRFVALKYGRRVFFVRGLQDKDVRWKKDPKLFDSWKEGKTGVPFVDANMRELAATGFMSNRGRQNVA
SFLTKDLGLDWRMGAEWFESLLVDYDVCSNYGNWLYSAGVGNDPRENRKFNMIKQGLDYDSNGDYVRLWVPEL
QGIKGGDVHTPWALSTAALSQAGVSLGATYPLPVVMAREWSRHINQKPNSQGPTRKGKKGPSYTPKQHKDRGV
DFYFSRNKDLS
>Zootoca_vivipar_XP_034988454.1
MSQKRRKRKPAESAEPKEEGAEEGAIFPDGLESGGRLASGAPPGAKKAAGEGGVGSGGSATLAEGVKRARLNA
APSVAEFKYNKKRVRLISQDPDLKEDAQGILYWMSRDQRVQDNWAFLYAQRLALKQKLPLHVCFCLVPKFLEA
TIRHFGFLIRGLKEVAEECKELSIPFHLLIGFAKDVLPSFVTTHGIGGVVTDFAPLRVPMQWVQDVQERLPPD
VPFVQVDAHNIVPCWVASDKQEYGARTIRRKIHDRLAEFLTEFPPVVRHPYPAEFQAEPVDWDACYASLQVDH
SVKEVPWAQPGTAAGLAVLEEFIKERLKFFGTDRNNPNRAALSNLSPWFHFGQISVQRAILEVRKLRSRYKES
AEAFIEEAVVRRELADNFCYYNPNYDKVEGAYDWAKTTLKLHAKDKRPHLYTLEQLEEGKTHDPLWNAAQLQM
VHEGKMHGFLRMYWAKKILEWTTSPEEALKFSIYLNDRYELDGRDPNGYVGQSWEEAGWLNHRVIGLQGAPRA
NLVQPPADQDSTRPMSVPAHCRERWEEECKSRDLFVLFFPLHQAACGLFVGSTTKAGQSGLFLARSAT
>Zootoca_vivipar_XP_034995129.1
MTHISIHWFRKALRLHDNPALLAAMKDSAEVYPIFIFDPWFPKNMRVSVNRWRFLVESLRDLDESLKKLNSRL
FVVRGSPVEAFPELFKKWKVTLLTFEVDTEPYARRRDAEVVRVAAEHGVQVVQKVSHTLYDTERIIIENNGKA
PLTYTRLQTVLASLGPPKRPVPAPSLENLKECSTSVKEDHDKEYGIPSLEELGQDPKTAGPHLYPGGESEALA
RLDLYMKRTSWVCNFKKPDTSPNSLTPSTTVLSPYVKFGCLSVRMFWWKLTEIYQGRKHSDPPVSLHGQLLWR
EFFYTAGAGIPNFDKMEGNPVCVQVDWDDNKEHLKAWREGRTGYPFIDAIMTQLRTEGWIHHLARHAVACFLT
RGDLWISWEEGQKVFEELLLDADWSLNAGNWQWLSASAFFHQFFRVYSPIAFGKKTDKNGEYIKKYLPFLKKF
PSDYIYEPWKAPRSVQERAGCIIGKDYPKPIVAHEEVRKKNLERMKAAYARRSQSAAAEAQDERSKKGHRVWE
DANQKRPEAPLKAEVRTKKMKIKST
>Zootoca_viv_XP_034982807.1_cry-1_X1
MGVNAVHWFRKGLRLHDNPALREVIQGADTVRCVYILDPWFAGSSNVGINRWRFLLQCLEDLDANLRKLNSRL
FVIRGQPADVFPRLFKEWNITKLSIEYDSEPFGKERDAAIKKLASEAGVEVIVRISHTLYDLDKIIELNGGQP
PLTYKRFQTLISRMEPLEMPVETITVEVMSKCTTPVSDDHDEKYGVPSLDELGFDTDGLPSAVWPGGETEALT
RLERHLERKAWVANFERPRMNANSLLASPTGLSPYLRFGCLSCRLFYFKLTDLYKKVKKNSSPPLSLYGQLLW
REFFYTAATNNPRFDKMEGNPICVQIPWDRNPEALAKWAEGRTGFPWIDAIMTQLRQEGWIHHLARHAVACFL
TRGDLWISWEEGMKVFEELLLDADWSVNAGSWMWLSCSSFFQQFFHCYCPVGFGRRTDPNGDYIRRYLPVLRG
FPAKYIYDPWNAPEGVQKVAKCVIGVNYPKPMVNHAEASRLNIERMKQIYQQLSRYRGLGLLASVPSNGNGNG
NSGLMGYSPGETLHASGSQMGTNEAHTGSVQTCTLGESHTGTSGIQQQAGYPQGSGILHYAHGEGQKTHLIQQ
GRASLVAGVCTGKRPNPEEETQSIGPKVQRQSSN
