# Supplementary material for: The Gain and Loss of Cryptochrome/Photolyase Family Members during Evolution
Source: Genes (Basel). 2022 Sep 8;13(9):1613. doi: 10.3390/genes13091613 (PMC9498864; doi:10.3390/genes13091613)
Supplement: Supplementary file 1 [file genes-13-01613-s001.zip › genes-1883542-supplementary/Supplement 3 Motif_Table_2022-09_08.pdf]

CPF-member	Name	Motif	Comment	Reference(s)
DASH-CRY	dlr	YWVxFELLWRD	• CPD lesion repair<br>• 2 missmatches allowed	[25,26,165]
	dlr1	MSNRGRQ	• Part of D-R salt bridge<br>• CPD lesion repair<br>• 1 missmatch allowed	[25,26,165]
	dlr2	DYDxxSNYGNWxY	• Part of D-R salt bridge<br>• CPD lesion repair<br>• Trp-triad<br>• 2 missmatches allowed	[25,26,53,165]
CPD class I/III	c1/3-1	DGDLxxxNxGWQW	• Generated from alignment of sequences from references<br>• 2 mismatches allowed	[9,175, 176]
	c3-2	PxFRxFNPxxQxEKFD	• From Alignment of 5 CPDIII sequences (see supplement in Nie et al., 2021)<br>• 2 mismatches allowed	[176]
	c3	WQWxAGxGxD	• From Alignment of 5 CPDIII sequences (see supplement in Nie et al., 2021)<br>• 1 mismatch allowed	[176]
CPD class II	mts	MHGFxRMYWAK	• Mitochondrial target sequence (MTS)<br>• 2 mismatches allowed	[9,173-175]
	cc1	EExxVRREL	• 1 mismatch allowed	[175]
	cc2	YMNYxGCKRKF	• 2 mismatches allowed<br>• Only conserved in eukaryots	[174,175]
	cc3	NGYxG (microorganisms & animals)<br>SGYxG (plants)	• N in microorganisms and animals<br>• S in many plants	[9]

CPF-member	Name	Motif	Comment	Reference(s)
	cab	FxLxxxFxxAxxxxYxxM	• antenna loop (antenna binding site)<br>• 1 mismatch allowed	[36]
DCRY	dcl	WVSxS	• C-terminal lid	[172]
	dct	PPHCxPSxxxxxRQFxWL	• C-terminal tail<br>• 3 mismatches allowed	[172]
	d2pu	FIFDG	• Secondary pocket upper helix	[171]
	d2pl	E**Q**D**C**EPx**W**xx**R**	• Secondary pocket lower helix<br>• 2 mismatches allowed	[171]
	dm1	HTLWxP	• DCRY motif1	New motif
MCRY	mpm	**ERKAWVA**x**F**x**RP**xMxxxSLLxS	• Phosphate motif<br>• 2 mismatches allowed	[172]
	mcl	WMWLS**C**SSF**FQQFFHCY**	• C-terminal lid<br>• 2 mismatches allowed	[172]
	ml	**YK**xx**K**	• Protrusion loop	[172]
	m2pu	D**PWFA**G**SS**N	• Secondary pocket upper helix<br>• 2 mismatches allowed	[171]

CPF-member	Name	Motif	Comment	Reference(s)
	mct	YRGLCLLASVPS	• Conserved C-terminal extension<br>• 2 mismatches allowed	[170]
	m2pl	E**Y**D**S**EPFG**KE**R (non-insects)<br>E**E**D**P**EPxG**RV**R (insects)	• Secondary pocket lower helix<br>• 1 mismatch allowed (non-insects)<br>• 2 mismatches allowes (insects)	[171]
	mnc	VYxxL	• MCRY non-chordate specific	New motif
	I	VWPGG	• MCRY1 specific	New motif
	II	RLDKHL	• MCRY2 specific<br>•	New motif
(6-4) photolyase	α15	HHLARHxVACFLTRG DL	• Conserved α-Helix 15<br>• 2 mismatches allowed	[169]
	α10	SPYLxFGCLS	• Conserved α-Helix 10<br>• 1 mismatch allowed	[169]
	sl	MxGNxxCxQIPW	• Conserved sulfur loop<br>• 1 mismatch allowed	[169]

CPF-member	Name	Motif	Comment	Reference(s)
	α12	PPxSLxGQLxWREFxYTxA	• Conserved α-helix 12<br>• 2 mismatches allowed	[169]
	α11	SxRxFxxxL	• Conserved α-helix 11<br>• no mismatches allowed	[169]
	α8	GGExEALxR	• Conserved α-helix 8<br>• 1 mismatch allowed	[169]
	α14	PxIDAIMxQLxxxG	• Conserved α-helix 14<br>• 1 mismatch allowed	[169]
	α16	WExGxxVFxxLLxD	• Conserved α-helix 16<br>• 1 mismatch allowed	[169]
	α17	DxxxNxGxWMWL	• Conserved α-helix 17<br>• 1 mismatch allowed	[169]
	α18	YxPxxFGxxxDPxG	• Conserved α-helix 18<br>• 1 mismatch allowed	[169]
	6-4	YIYEPWKAP	• 6-4 photolyase specific	New motif
PCRY	pd1	**D**QxVP	• Part of DAS-motif1	[167,168]
	pd2	STxExxS	• Part of DAS-motif2	[167,168]

CPF-member	Name	Motif	Comment	Reference(s)
	pc1	QGRTGYPLVDAGMR ELWATGWxH	• Conserved in all plant CRYs<br>• 3 mismatches allowed	[166]
	pc	DLLYEPW	• All plant CRY1s 1 mismatch allowed	[166]
	pn	VWFRRDLRxEDNPAL	• N-terminal<br>• 2 mismatches allowed	[166]
PCRY-like	pl1	VGLR	• PCRY-like specific 1	New motif
	pl2	WRDLAY	• PCRY-like specific 2	New motif
	pl3	MWQNxG	• PCRY-like specific 3	New motif
CRY4	4a	GXPPLTYKxF	• CRY4 motif a	New motif
	4b	PNSLLPSTTGL	• CRY4 motif b	New motif
	4c	TxNFTxMX	• CRY4 conserved	[83]
	4Y	WYEDAERL	• CRY4 conserved Y<br>• 2 mismatches allowed	[83]

CPF-member	Name	Motif	Comment	Reference(s)
PPL	ppl1	ESxSxxPVYCFDPR	• 2 mismatches allowed	New motif
	ppl2	BSJYGANFSCKI	• 2 mismatches allowed	New motif
	ppl3	FFRFxTxK		New motif

References

9. Kiontke, S.; Geisselbrecht, Y.; Pokorny, R.; Carell, T.; Batschauer, A.; Essen, L.O. Crystal Structures of an Archaeal Class II DNA Photolyase and Its Complex with UV-Damaged Duplex DNA. *The EMBO journal* **2011**, *30*, 4437–4449, doi:10.1038/emboj.2011.313.

25. Tagua, V.G.; Pausch, M.; Eckel, M.; Gutiérrez, G.; Miralles-Durán, A.; Sanz, C.; Eslava, A.P.; Pokorny, R.; Corrochano, L.M.; Batschauer, A. Fungal Cryptochrome with DNA Repair Activity Reveals an Early Stage in Cryptochrome Evolution. *PNAS* **2015**, *112*, 15130–15135, doi:10.1073/pnas.1514637112.

26. Navarro, E.; Niemann, N.; Kock, D.; Dadaeva, T.; Gutiérrez, G.; Engelsdorf, T.; Kiontke, S.; Corrochano, L.M.; Batschauer, A.; Garre, V. The DASH-Type Cryptochrome from the Fungus Mucor Circinelloides Is a Canonical CPD-Photolyase. *Current Biology* **2020**, *30*, 4483-4490.e4, doi:10.1016/j.cub.2020.08.051.

36. Kiontke, S.; Gnau, P.; Haselsberger, R.; Batschauer, A.; Essen, L.-O. Structural and Evolutionary Aspects of Antenna Chromophore Usage by Class II Photolyases. *J Biol Chem* **2014**, *289*, 19659–19669, doi:10.1074/jbc.M113.542431.

53. Daiyasu, H.; Ishikawa, T.; Kuma, K.; Iwai, S.; Todo, T.; Toh, H. Identification of Cryptochrome DASH from Vertebrates. *Genes to Cells* **2004**, *9*, 479–495, doi:10.1111/j.1356-9597.2004.00738.x.

83. Zoltowski, B.D.; Chelliah, Y.; Wickramaratne, A.; Jarocha, L.; Karki, N.; Xu, W.; Mouritsen, H.; Hore, P.J.; Hibbs, R.E.; Green, C.B.; et al. Chemical and Structural Analysis of a Photoactive Vertebrate Cryptochrome from Pigeon. *Proceedings of the National Academy of Sciences of the United States of America* **2019**, *116*, 19449–19457, doi:10.1073/pnas.1907875116.

165. Pokorny, R.; Klar, T.; Hennecke, U.; Carell, T.; Batschauer, A.; Essen, L.-O. Recognition and Repair of UV Lesions in Loop Structures of Duplex DNA by DASH-Type Cryptochrome. *PNAS* **2008**, *105*, 21023–21027, doi:10.1073/pnas.0805830106.

166. Cao, S.; He, S.; Lv, H.; Zhang, J.; Aslam, M.; Cheng, H.; Hu, A.; Cao, G.; Zhang, X.; Yu, Y.; et al. Genome-Wide Analysis of the Cryptochrome Gene Family in Plants. *Tropical Plant Biology* **2020**, *13*, 117–126, doi:10.1007/s12042-019-09249-9.

167. Partch, C.L.; Clarkson, M.W.; Ozgur, S.; Lee, A.L.; Sancar, A. Role of Structural Plasticity in Signal Transduction by the Cryptochrome Blue-Light Photoreceptor. *Biochemistry* **2005**, *44*, 3795–3805, doi:10.1021/bi047545g.

168. Lin, C.; Shalitin, D. Cryptochrome Structure and Signal Transduction. *Annual review of plant biology* **2003**, *54*, 469–496, doi:10.1146/annurev.arplant.54.110901.160901.

169. Hitomi, K.; DiTacchio, L.; Arvai, A.S.; Yamamoto, J.; Kim, S.T.; Todo, T.; Tainer, J.A.; Iwai, S.; Panda, S.; Getzoff, E.D. Functional Motifs in the (6-4) Photolyase Crystal Structure Make a Comparative Framework for DNA Repair Photolyases and Clock Cryptochromes. *Proceedings of the National Academy of Sciences of the United States of America* **2009**, *106*, 6962–6967, doi:10.1073/pnas.0809180106.

170. Xing, W.; Busino, L.; Hinds, T.R.; Marionni, S.T.; Saifee, N.H.; Bush, M.F.; Pagano, M.; Zheng, N. SCF(FBXL3) Ubiquitin Ligase Targets Cryptochromes at Their Cofactor Pocket. *Nature* **2013**, *496*, 64–68, doi:10.1038/nature11964.

171. Rosensweig, C.; Reynolds, K.A.; Gao, P.; Laothamatas, I.; Shan, Y.; Ranganathan, R.; Takahashi, J.S.; Green, C.B. An Evolutionary Hotspot Defines Functional Differences between CRYPTOCHROMES. *Nat Commun* **2018**, *9*, 1138, doi:10.1038/s41467-018-03503-6.

172. Zoltowski, B.D.; Vaidya, A.T.; Top, D.; Widom, J.; Young, M.W.; Crane, B.R. Structure of Full-Length Drosophila Cryptochrome. *Nature* **2011**, *480*, 396–399, doi:10.1038/nature10618.

173. Zhang, M.; Wang, L.; Shu, S.; Sancar, A.; Zhong, D. Bifurcating Electron-Transfer Pathways in DNA Photolyases Determine the Repair Quantum Yield. *Science (New York, N.Y.)* **2016**, *354*, 209–213, doi:10.1126/science.aah6071.

174. Hitomi, K.; Arvai, A.S.; Yamamoto, J.; Hitomi, C.; Teranishi, M.; Hirouchi, T.; Yamamoto, K.; Iwai, S.; Tainer, J.A.; Hidema, J.; et al. Eukaryotic Class II Cyclobutane Pyrimidine Dimer Photolyase Structure Reveals Basis for Improved Ultraviolet Tolerance in Plants. *The Journal of biological chemistry* **2012**, *287*, 12060–12069, doi:10.1074/jbc.M111.244020.

175. Takahashi, S.; Teranishi, M.; Izumi, M.; Takahashi, M.; Takahashi, F.; Hidema, J. Transport of Rice Cyclobutane Pyrimidine Dimer Photolyase into Mitochondria Relies on a Targeting Sequence Located in Its C-Terminal Internal Region. *The Plant journal : for cell and molecular biology* **2014**, *79*, 951–963, doi:10.1111/tpj.12598.

176. Nie, G.; Liu, X.; Zhou, X.; Song, Q.; Fu, M.; Xu, F.; Wang, X. Functional Analysis of a Novel Cryptochrome Gene (GbCRY1) from Ginkgo Biloba. *Plant Signal Behav* **2021**, *16*, 1850627, doi:10.1080/15592324.2020.1850627.
