# Supplementary material for: The Gain and Loss of Cryptochrome/Photolyase Family Members during Evolution
Source: Genes (Basel). 2022 Sep 8;13(9):1613. doi: 10.3390/genes13091613 (PMC9498864; doi:10.3390/genes13091613)
Supplement: Supplementary file 1 [file genes-13-01613-s001.zip › genes-1883542-supplementary/Supplement 4 Alignment Summary.pdf]

6-4 PL

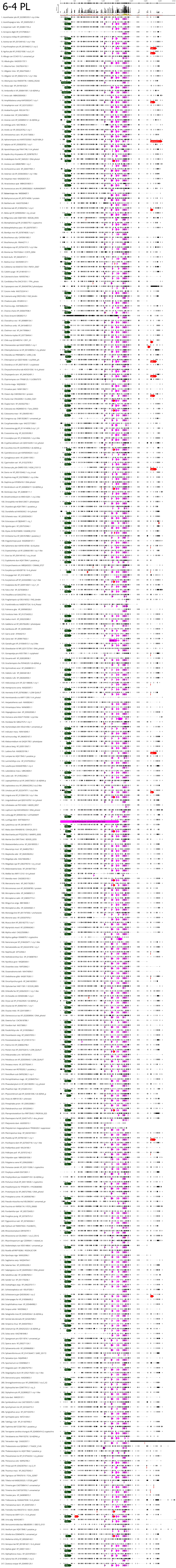

ACRY

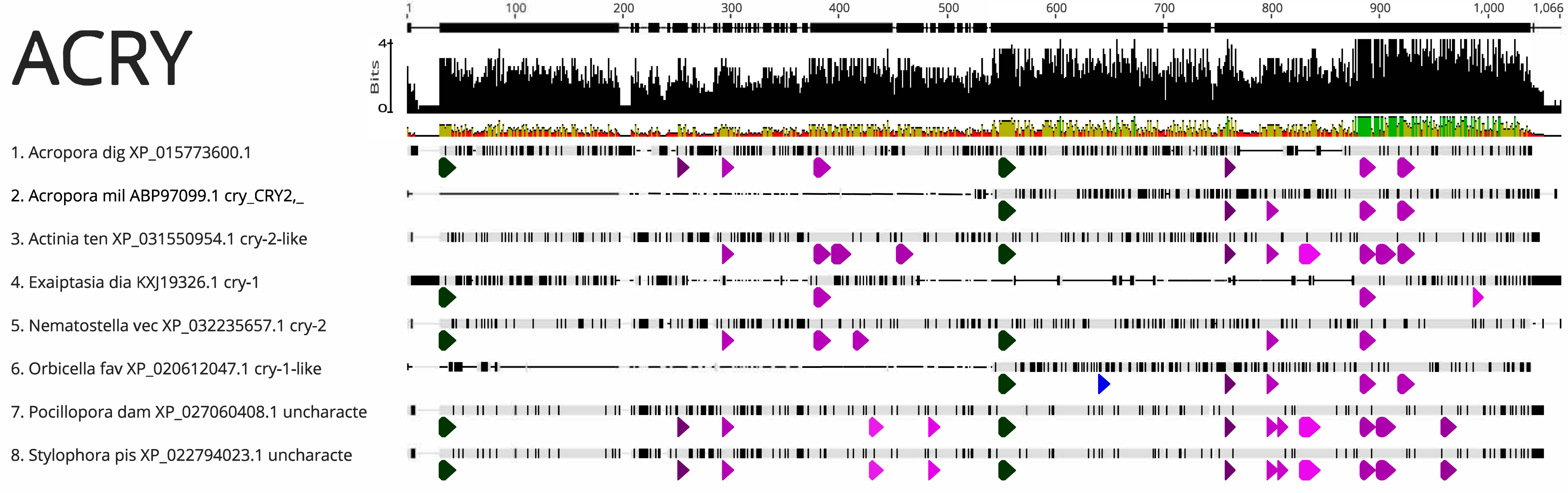
1
100
200
300
400
500
600
700
800
900
1,000
1,066
4
Bits
0
1. Acropora dig XP_015773600.1
2. Acropora mil ABP97099.1 cry_CRY2,_
3. Actinia ten XP_031550954.1 cry-2-like
4. Exaiptasia dia KXJ19326.1 cry-1
5. Nematostella vec XP_032235657.1 cry-2
6. Orbicella fav XP_020612047.1 cry-1-like
7. Pocillopora dam XP_027060408.1 uncharacte
8. Stylophora pis XP_022794023.1 uncharacte

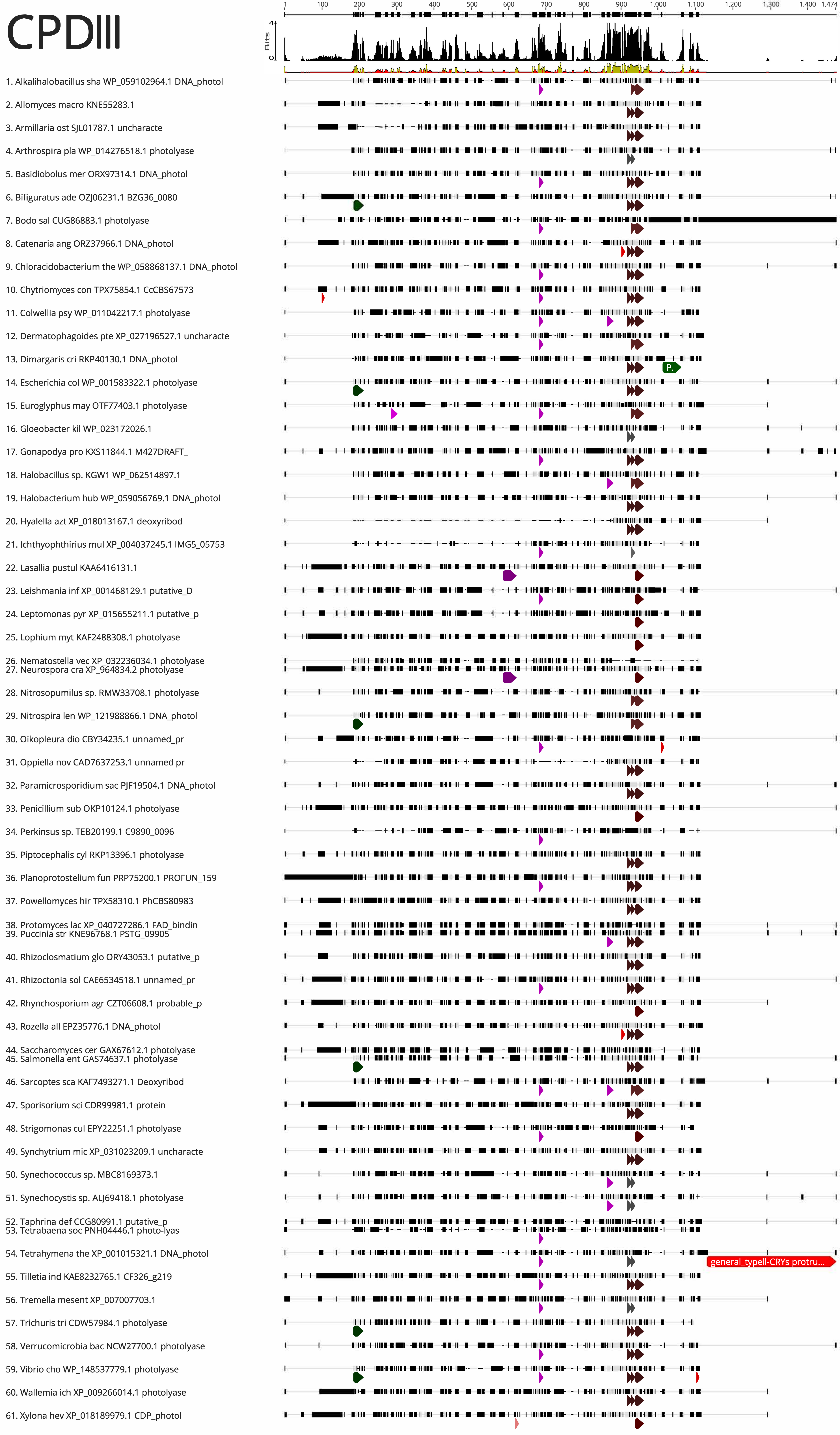
CPDIII
1
100
200
300
400
500
600
700
800
900
1,000
1,100
1,200
1,300
1,400
1,474
Bits
4
0
1. Alkalihalobacillus sha WP_059102964.1 DNA_photol
2. Allomyces macro KNE55283.1
3. Armillaria ost SJL01787.1 uncharacte
4. Arthrospira pla WP_014276518.1 photolyase
5. Basidiobolus mer ORX97314.1 DNA_photol
6. Bifiguratus ade OZJ06231.1 BZG36_0080
7. Bodo sal CUG86883.1 photolyase
8. Catenaria ang ORZ37966.1 DNA_photol
9. Chloracidobacterium the WP_058868137.1 DNA_photol
10. Chytriomyces con TPX75854.1 CcCBS67573
11. Colwellia psy WP_011042217.1 photolyase
12. Dermatophagoides pte XP_027196527.1 uncharacte
13. Dimargaris cri RKP40130.1 DNA_photol
P.
14. Escherichia col WP_001583322.1 photolyase
15. Euroglyphus may OTF77403.1 photolyase
16. Gloeobacter kil WP_023172026.1
17. Gonapodya pro KXS11844.1 M427DRAFT_
18. Halobacillus sp. KGW1 WP_062514897.1
19. Halobacterium hub WP_059056769.1 DNA_photol
20. Hyalella azt XP_018013167.1 deoxyribod
21. Ichthyophthirius mul XP_004037245.1 IMG5_05753
22. Lasallia pustul KAA6416131.1
23. Leishmania inf XP_001468129.1 putative_D
24. Leptomonas pyr XP_015655211.1 putative_p
25. Lophium myt KAF2488308.1 photolyase
26. Nematostella vec XP_032236034.1 photolyase
27. Neurospora cra XP_964834.2 photolyase
28. Nitrosopumilus sp. RMW33708.1 photolyase
29. Nitrospira len WP_121988866.1 DNA_photol
30. Oikopleura dio CBY34235.1 unnamed_pr
31. Oppiella nov CAD7637253.1 unnamed pr
32. Paramicrosporidium sac PJF19504.1 DNA_photol
33. Penicillium sub OKP10124.1 photolyase
34. Perkinsus sp. TEB20199.1 C9890_0096
35. Piptocephalis cyl RKP13396.1 photolyase
36. Planoprotostelium fun PRP75200.1 PROFUN_159
37. Powellomyces hir TPX58310.1 PhCBS80983
38. Protomyces lac XP_040727286.1 FAD_bindin
39. Puccinia str KNE96768.1 PSTG_09905
40. Rhizoclosmatium glo ORY43053.1 putative_p
41. Rhizoctonia sol CAE6534518.1 unnamed_pr
42. Rhynchosporium agr CZT06608.1 probable_p
43. Rozella all EPZ35776.1 DNA_photol
44. Saccharomyces cer GAX67612.1 photolyase
45. Salmonella ent GAS74637.1 photolyase
46. Sarcoptes sca KAF7493271.1 Deoxyribod
47. Sporisorium sci CDR99981.1 protein
48. Strigomonas cul EPY22251.1 photolyase
49. Synchytrium mic XP_031023209.1 uncharacte
50. Synechococcus sp. MBC8169373.1
51. Synechocystis sp. ALJ69418.1 photolyase
52. Taphrina def CCG80991.1 putative_p
53. Tetrabaena soc PNH04446.1 photo-lyas
54. Tetrahymena the XP_001015321.1 DNA_photol
general_typeII-CRYs protru...
55. Tilletia ind KAE8232765.1 CF326_g219
56. Tremella mesent XP_007007703.1
57. Trichuris tri CDW57984.1 photolyase
58. Verrucomicrobia bac NCW27700.1 photolyase
59. Vibrio cho WP_148537779.1 photolyase
60. Wallemia ich XP_009266014.1 photolyase
61. Xylona hev XP_018189979.1 CDP_photol

CPDII

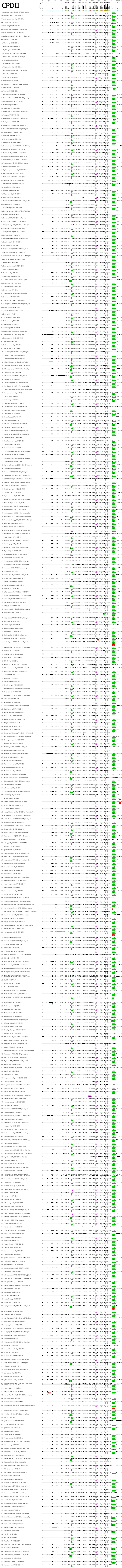

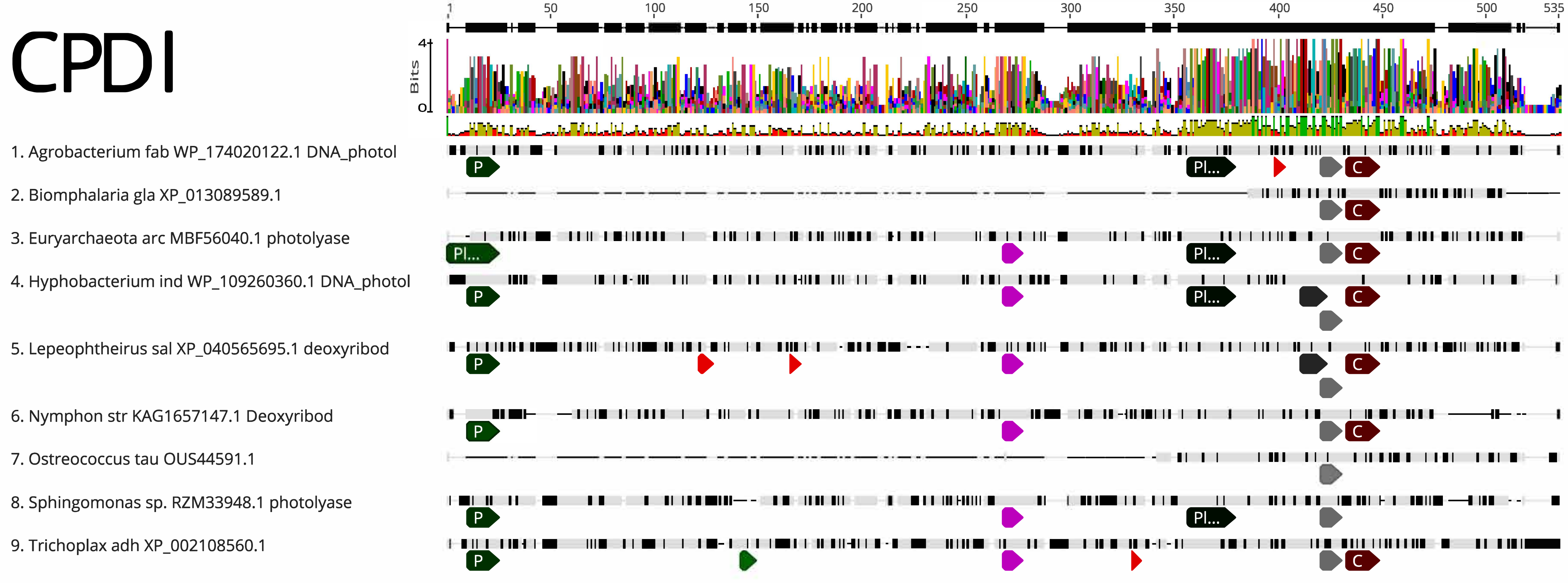
CPD I
Bits
1. Agrobacterium fab WP_174020122.1 DNA_photol
2. Biomphalaria gla XP_013089589.1
3. Euryarchaeota arc MBF56040.1 photolyase
4. Hyphobacterium ind WP_109260360.1 DNA_photol
5. Lepeophtheirus sal XP_040565695.1 deoxyribod
6. Nymphon str KAG1657147.1 Deoxyribod
7. Ostreococcus tau OUS44591.1
8. Sphingomonas sp. RZM33948.1 photolyase
9. Trichoplax adh XP_002108560.1

CRY4

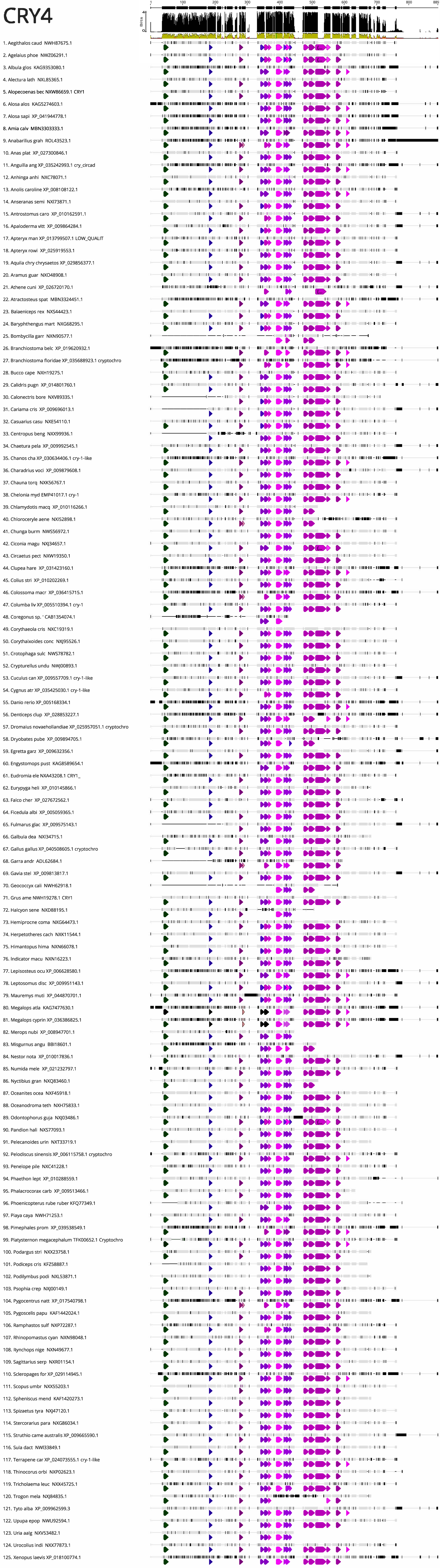
1
100
200
300
400
500
600
700
800
Bits
4
0
1. Aegithalos caud NWH87675.1
2. Agelaius phoe NWZ06291.1
3. Albula glos KAG9353080.1
4. Alectura lath NXL85365.1
5. Alopecoenas bec NXW86659.1 CRY1
6. Alosa alos KAG5274603.1
7. Alosa sapi XP_041944778.1
8. Amia calv MBN3303333.1
9. Anabarilius grah ROL43523.1
10. Anas plat XP_027300846.1
11. Anguilla ang XP_035242993.1 cry_circad
12. Anhinga anhi NXC78071.1
13. Anolis caroline XP_008108122.1
14. Anseranas semi NXI73871.1
15. Antrostomus caro XP_010162591.1
16. Apaloderma vitt XP_009864284.1
17. Apteryx man XP_013799507.1 LOW_QUALIT
18. Apteryx rowi XP_025919553.1
19. Aquila chry chrysaetos XP_029856377.1
20. Aramus guar NXO48908.1
21. Athene cuni XP_026720170.1
22. Atractosteus spat MBN3324451.1
23. Balaeniceps rex NXS44423.1
24. Baryphthengus mart NXG68295.1
25. Bombycilla garr NXN90577.1
26. Branchiostoma belc XP_019620932.1
27. Branchiostoma floridae XP_035688923.1 cryptochro
28. Bucco cape NXH19275.1
29. Calidris pugn XP_014801760.1
30. Calonectris bore NXV89335.1
31. Cariama cris XP_009696013.1
32. Casuarius casu NXE54110.1
33. Centropus beng NXX99936.1
34. Chaetura pela XP_009992545.1
35. Chanos cha XP_030634406.1 cry-1-like
36. Charadrius voci XP_009879608.1
37. Chauna torq NXK56767.1
38. Chelonia myd EMP41017.1 cry-1
39. Chlamydotis macq XP_010116266.1
40. Chloroceryle aene NXI52898.1
41. Chunga burm NWS56972.1
42. Ciconia magu NXJ34657.1
43. Circaetus pect NXW19350.1
44. Clupea hare XP_031423160.1
45. Colius stri XP_010202269.1
46. Colossoma macr XP_036415715.1
47. Columba liv XP_005510394.1 cry-1
48. Coregonus sp. ' CAB1354074.1
49. Corythaeola cris NXC19319.1
50. Corythaixoides conc NXJ95526.1
51. Crotophaga sulc NWS78782.1
52. Crypturellus undu NWJ00893.1
53. Cuculus can XP_009557709.1 cry-1-like
54. Cygnus atr XP_035425030.1 cry-1-like
55. Danio rerio XP_005168334.1
56. Denticeps clup XP_028853227.1
57. Dromaius novaehollandiae XP_025957051.1 cryptochro
58. Dryobates pube XP_009894705.1
59. Egretta garz XP_009632356.1
60. Engystomops pust KAG8589654.1
61. Eudromia ele NXA43208.1 CRY1_
62. Eurypyga heli XP_010145866.1
63. Falco cher XP_027672562.1
64. Ficedula albi XP_005059365.1
65. Fulmarus glac XP_009575143.1
66. Galbula dea NXI34715.1
67. Gallus gallus XP_040508605.1 cryptochro
68. Garra andr ADL62684.1
69. Gavia stel XP_009813817.1
70. Geococcyx cali NWH62918.1
71. Grus ame NWH19278.1 CRY1
72. Halcyon sene NXD88195.1
73. Hemiprocne coma NXG64473.1
74. Herpetotheres cach NXK11544.1
75. Himantopus hima NXN66078.1
76. Indicator macu NXN16223.1
77. Lepisosteus ocu XP_006628580.1
78. Leptosomus disc XP_009951143.1
79. Mauremys muti XP_044870701.1
80. Megalops atla KAG7477630.1
81. Megalops cyprin XP_036386825.1
82. Merops nubi XP_008947701.1
83. Misgurnus angu BBI18601.1
84. Nestor nota XP_010017836.1
85. Numida mele XP_021232797.1
86. Nyctibius gran NXQ83460.1
87. Oceanites ocea NXF45918.1
88. Oceanodroma teth NXH75833.1
89. Odontophorus guja NXJ03486.1
90. Pandion hali NXS77093.1
91. Pelecanoides urin NXT33719.1
92. Pelodiscus sinensis XP_006115758.1 cryptochro
93. Penelope pile NXC41228.1
94. Phaethon lept XP_010288559.1
95. Phalacrocorax carb XP_009513466.1
96. Phoenicopterus rube ruber KFQ77349.1
97. Piaya caya NWH71253.1
98. Pimephales prom XP_039538549.1
99. Platysternon megacephalum TFK00652.1 Cryptochro
100. Podargus stri NXX23758.1
101. Podiceps cris KFZ58887.1
102. Podilymbus podi NXL53871.1
103. Psophia crep NXJ00149.1
104. Pygocentrus natt XP_017540798.1
105. Pygoscelis papu KAF1442024.1
106. Ramphastos sulf NXP72287.1
107. Rhinopomastus cyan NXN98048.1
108. Rynchops nige NXN49677.1
109. Sagittarius serp NXR01154.1
110. Scleropages for XP_029114945.1
111. Scopus umbr NXX55203.1
112. Spheniscus mend KAF1420273.1
113. Spizaetus tyra NXJ47120.1
114. Stercorarius para NXG86034.1
115. Struthio came australis XP_009665590.1
116. Sula dact NWI33849.1
117. Terrapene car XP_024073555.1 cry-1-like
118. Thinocorus orbi NXP02623.1
119. Tricholaema leuc NXX45725.1
120. Trogon mela NXJ84835.1
121. Tyto alba XP_009962599.3
122. Upupa epop NWU92594.1
123. Uria aalg NXV53482.1
124. Urocolius indi NXX77873.1
125. Xenopus laevis XP_018100774.1

DASH

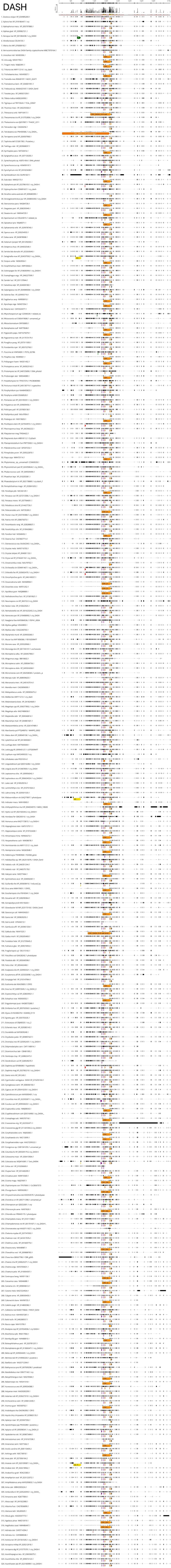

DCRY

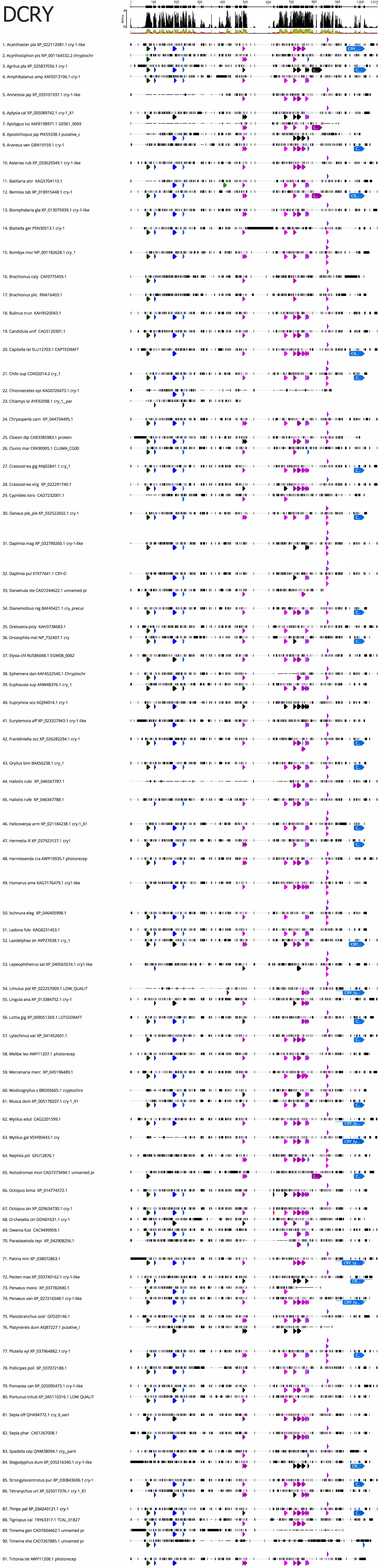
1. Acanthaster pla XP_022112081.1 cry-1-like
2. Acyrthosiphon pis NP_001164532.2 chryptochr
3. Agrilus pla XP_025837056.1 cry-1
4. Amphibalanus amp KAF0313106.1 cry-1
5. Anneissia jap XP_033101937.1 cry-1-like
6. Aplysia cal XP_005089742.1 cry-1_X1
7. Apolygus luc KAF6198971.1 GE061_0069
8. Apostichopus jap PIK55338.1 putative_c
9. Araneus ven GBN19159.1 cry-1
10. Asterias rub XP_033629349.1 cry-1-like
11. Batillaria attr KAG5704110.1
12. Bemisia tab XP_018915448.1 cry-1
13. Biomphalaria gla XP_013075939.1 cry-1-like
14. Blattella ger PSN30513.1 cry-1
15. Bombyx mor NP_001182628.1 cry_1
16. Brachionus caly CAF0775459.1
17. Brachionus plic RNA16403.1
18. Bulinus trun KAH9520043.1
19. Candidula unif CAG5120301.1
20. Capitella tel ELU12703.1 CAPTEDRAFT
21. Chilo sup CDK02014.2 cry_1
22. Chionoecetes opi KAG0726473.1 cry-1
23. Chlamys isl AYE92098.1 cry_1,_par
24. Chrysoperla carn XP_044734495.1
25. Cloeon dip CAB3385983.1 protein
26. Clunio mar CRK90905.1 CLUMA_CG00
27. Crassostrea gig ANJ02841.1 cry_1
28. Crassostrea virg XP_022291740.1
29. Cyprideis toro CAD7232001.1
30. Danaus ple_ple XP_032522602.1 cry-1
31. Daphnia mag XP_032790260.1 cry-1-like
32. Daphnia pul EFX77441.1 CRY-D
33. Darwinula ste CAD7244622.1 unnamed pr
34. Dianemobius nig BAF45421.1 cry_precur
35. Dreissena poly KAH3738683.1
36. Drosophila mel NP_732407.1 cry
37. Elysia chl RUS86048.1 EGW08_0062
38. Ephemera dan KAF4522540.1 Chryptochr
39. Euphausia sup ANW48376.1 cry_1
40. Euprymna sco AGJ94014.1 cry-1
41. Eurytemora aff XP_023327943.1 cry-1-like
42. Frankliniella occ XP_026282294.1 cry-1
43. Gryllus bim BAX56238.1 cry_1
44. Haliotis rubr XP_046567787.1
45. Haliotis rufe XP_046347788.1
46. Helicoverpa arm XP_021184238.1 cry-1_X1
47. Hermetia ill XP_037923127.1 cry1
48. Hermissenda cra AWY10935.1 photorecep
49. Homarus ame KAG7176479.1 cry1-like
50. Ischnura eleg XP_046405998.1
51. Ladona fulv KAG8231453.1
52. Laodelphax str AVP27638.1 cry_1
53. Lepeophtheirus sal XP_040563574.1 cry1-like
54. Limulus pol XP_022257009.1 LOW_QUALIT
55. Lingula ana XP_013384752.1 cry-1
56. Lottia gig XP_009051269.1 LOTGIDRAFT
57. Lytechinus var XP_041452601.1
58. Melibe leo AWY11207.1 photorecep
59. Mercenaria merc XP_045196489.1
60. Modicogryllus s BBD05665.1 cryptochro
61. Musca dom XP_005178207.1 cry-1_X1
62. Mytilus edul CAG2201599.1
63. Mytilus gal VDH90443.1 cry
64. Nephila pili GFU12876.1
65. Notodromas mon CAD7273494.1 unnamed pr
66. Octopus bima XP_014774572.1
67. Octopus sin XP_029634730.1 cry-1
68. Orchesella cin ODN01631.1 cry-1
69. Owenia fusi CAC9499959.1
70. Parasteatoda tepi XP_042908256.1
71. Patiria min XP_038072863.1
72. Pecten max XP_033740162.1 cry-1-like
73. Penaeus mono XP_037782690.1
74. Penaeus van XP_027216548.1 cry-1-like
75. Plakobranchus ocel GFO20146.1
76. Platynereis dum AEJ87227.1 putative_l
77. Plutella xyl XP_037964882.1 cry-1
78. Pollicipes poll XP_037072188.1
79. Pomacea can XP_025095473.1 cry-1-like
80. Portunus tritub XP_045110316.1 LOW QUALIT
81. Sepia off QHA94772.1 cry_6_vari
82. Sepia phar CAE1267008.1
83. Spadella cep QNM38094.1 cry_parti
84. Stegodyphus dum XP_035216340.1 cry-1-like
85. Strongylocentrotus pur XP_030843606.1 cry-1
86. Tetranychus urt XP_025017376.1 cry-1_X1
87. Thrips pal XP_034243121.1 cry-1
88. Tigriopus cal TRY63317.1 TCAL_01827
89. Timema gen CAD7604462.1 unnamed pr
90. Timema she CAD7267885.1 unnamed pr
91. Tritonia tet AWY11208.1 photorecep

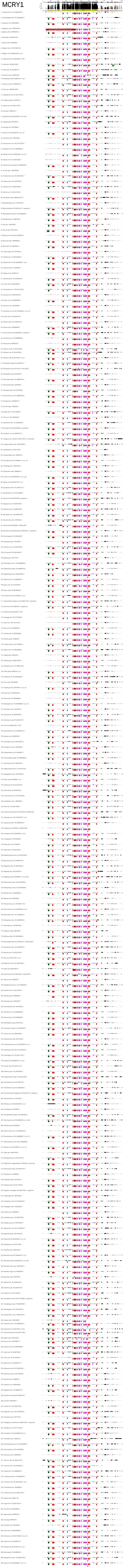
MCRY1

MCRY2

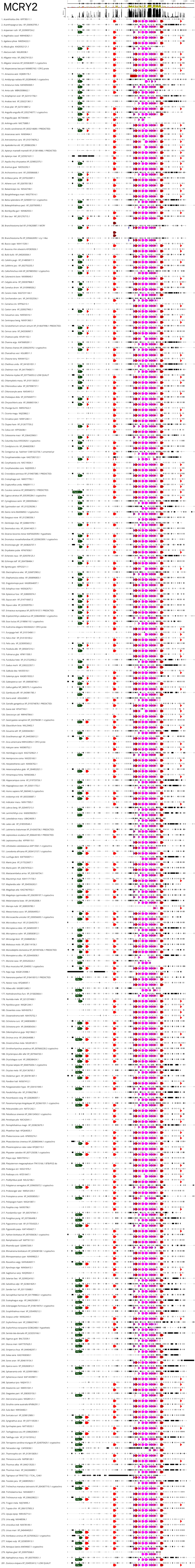

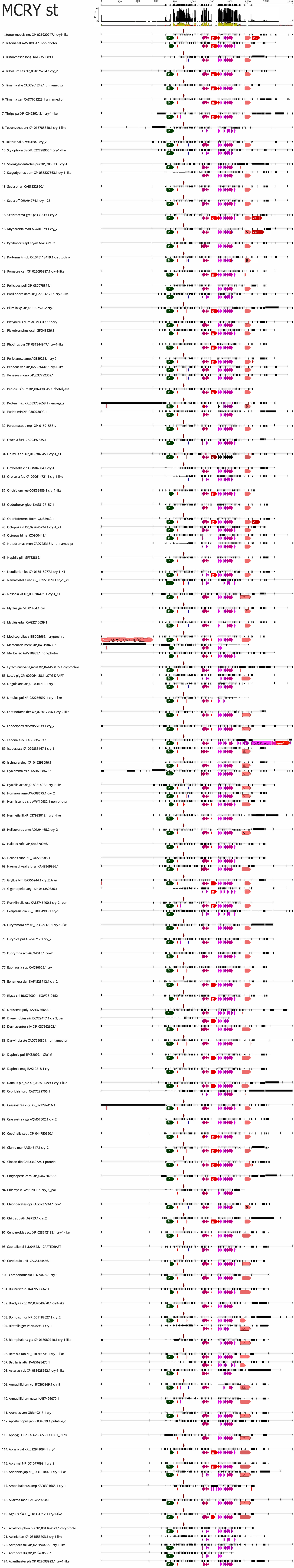
MCRY st
Bits
1. Zootermopsis nev XP_021920747.1 cry1-like
2. Tritonia tet AWY10934.1 non-photor
3. Trinorchestia long KAF2350589.1
4. Tribolium cas NP_001076794.1 cry_2
5. Timema she CAD7261249.1 unnamed pr
6. Timema gen CAD7601223.1 unnamed pr
7. Thrips pal XP_034239242.1 cry-1-like
8. Tetranychus urt XP_015785840.1 cry-1-like
9. Talitrus sal AFV96168.1 cry_2
10. Stylophora pis XP_022798906.1 cry-1-like
11. Strongylocentrotus pur XP_785873.3 cry-1
12. Stegodyphus dum XP_035227663.1 cry-1-like
13. Sepia phar CAE1232360.1
14. Sepia off QHA94774.1 cry_123
15. Schistocerca gre QVD39239.1 cry 2
16. Rhyparobia mad AGA01579.1 cry_2
17. Pyrrhocoris apt cry-m MW662132
18. Portunus tritub XP_045118419.1 cryptochro
19. Pomacea can XP_025096987.1 cry-1-like
20. Pollicipes poll XP_037075374.1
21. Pocillopora dam XP_027056122.1 cry-1-like
22. Plutella xyl XP_011557520.2 cry-1
23. Platynereis dum AGX93012.1 tr-cry
24. Plakobranchus ocel GFO43536.1
25. Photinus pyr XP_031344947.1 cry-1-like
26. Periplaneta ame AGE89265.1 cry 2
27. Penaeus van XP_027226418.1 cry-1-like
28. Penaeus mono XP_037792362.1
29. Pediculus hum XP_002430545.1 photolyase
30. Pecten max XP_033739658.1 cleavage_s
31. Patiria min XP_038073890.1
32. Parasteatoda tepi XP_015915881.1
33. Owenia fusi CAC9497535.1
34. Orussus abi XP_012284945.1 cry-1_X1
35. Orchesella cin ODN04604.1 cry-1
36. Orbicella fav XP_020614721.1 cry-1-like
37. Onchidium ree QDK59985.1 cry_1-like
38. Oedothorax gibb KAG8197157.1
39. Odontotermes form QLJ82960.1
40. Octopus sin XP_029646224.1 cry-1_X1
41. Octopus bima KOG00441.1
42. Notodromas mon CAD7283181.1 unnamed pr
43. Nephila pili GFT83862.1
44. Neodiprion lec XP_015515077.1 cry-1_X1
45. Nematostella vec XP_032226079.1 cry-1_X1
46. Nasonia vit XP_008204431.1 cry-1_X1
47. Mytilus gal VDI01404.1 cry
48. Mytilus edul CAG2210639.1
49. Modicogryllus s BBD05666.1 cryptochro
50. Mercenaria merc XP_045198496.1
51. Melibe leo AWY10933.1 non-photor
52. Lytechinus variegatus XP_041453155.1 cryptochro
53. Lottia gig XP_009064438.1 LOTGIDRAFT
54. Lingula ana XP_013416713.1 cry-1
55. Limulus pol XP_022256597.1 cry-1-like
56. Leptinotarsa dec XP_023017756.1 cry-2-like
57. Laodelphax str AVP27639.1 cry_2
58. Ladona fulv KAG8235753.1
59. Ixodes sca XP_029833167.1 cry-1
60. Ischnura eleg XP_046393096.1
61. Hyalomma asia KAH6938626.1
62. Hyalella azt XP_018021492.1 cry-1-like
63. Homarus ame AWC08575.1 cry_2
64. Hermissenda cra AWY10932.1 non-photor
65. Hermetia ill XP_037923019.1 cry1-like
66. Helicoverpa arm ADN94465.2 cry_2
67. Haliotis rufe XP_046370956.1
68. Haliotis rubr XP_046585585.1
69. Haemaphysalis long KAH9369986.1
70. Gryllus bim BAX56244.1 cry_2_tran
71. Gigantopelta aegi XP_041350836.1
72. Frankliniella occ KAE8746400.1 cry_2,_par
73. Exaiptasia dia XP_020904995.1 cry-1
74. Eurytemora aff XP_023329370.1 cry-1-like
75. Eurydice pul AGV28717.1 cry_2
76. Euprymna sco AGJ94015.1 cry-2
77. Euphausia sup CAQ86665.1 cry
78. Ephemera dan KAF4523712.1 cry_2
79. Elysia chl RUS77009.1 EGW08_0152
80. Dreissena poly KAH3736653.1
81. Dianemobius nig BCX29417.1 cry 2, par
82. Dermacentor silv XP_037562602.1
83. Darwinula ste CAD7250301.1 unnamed pr
84. Daphnia pul EFX82092.1 CRY-M
85. Daphnia mag BAS19218.1 cry
86. Danaus ple_ple XP_032511499.1 cry-1-like
87. Cyprideis toro CAD7229706.1
88. Crassostrea virg XP_022292416.1
89. Crassostrea gig AQM57602.1 cry_2
90. Coccinella sept XP_044750690.1
91. Clunio mar AFS34617.1 cry_2
92. Cloeon dip CAB3360724.1 protein
93. Chrysoperla carn XP_044730763.1
94. Chlamys isl AYE92099.1 cry_2,_par
95. Chionoecetes opi KAG0727244.1 cry-1
96. Chilo sup AHL69753.1 cry_2
97. Centruroides scu XP_023242183.1 cry-1-like
98. Capitella tel ELU04573.1 CAPTEDRAFT
99. Candidula unif CAG5124456.1
100. Camponotus flo EFN74495.1 cry-1
101. Bulinus trun KAH9508662.1
102. Bradysia cop XP_037040970.1 cry1-like
103. Bombyx mor NP_001182627.1 cry_2
104. Blattella ger PSN44595.1 cry-1
105. Biomphalaria gla XP_013080710.1 cry-1-like
106. Bemisia tab XP_018916708.1 cry-1-like
107. Batillaria attr KAG5693470.1
108. Asterias rub XP_033628662.1 cry-1-like
109. Armadillidium vul RXG60369.1 cry-2
110. Armadillidium nasa KAB7496070.1
111. Araneus ven GBM49213.1 cry-1
112. Apostichopus jap PIK34639.1 putative_c
113. Apolygus luc KAF6206655.1 GE061_0178
114. Aplysia cal XP_012941094.1 cry-1
115. Apis mel NP_001077099.1 cry_2
116. Anneissia jap XP_033101802.1 cry-1-like
117. Amphibalanus amp KAF0301665.1 cry-1
118. Allacma fusc CAG7829298.1
119. Agrilus pla XP_018331212.1 cry-1-like
120. Acyrthosiphon pis NP_001164573.1 chryptochr
121. Actinia ten XP_031553703.1 cry-1-like
122. Acropora mil XP_029194452.1 cry-1-like
123. Acropora dig XP_015760686.1
124. Acanthaster pla XP_022093922.1 cry-1-like

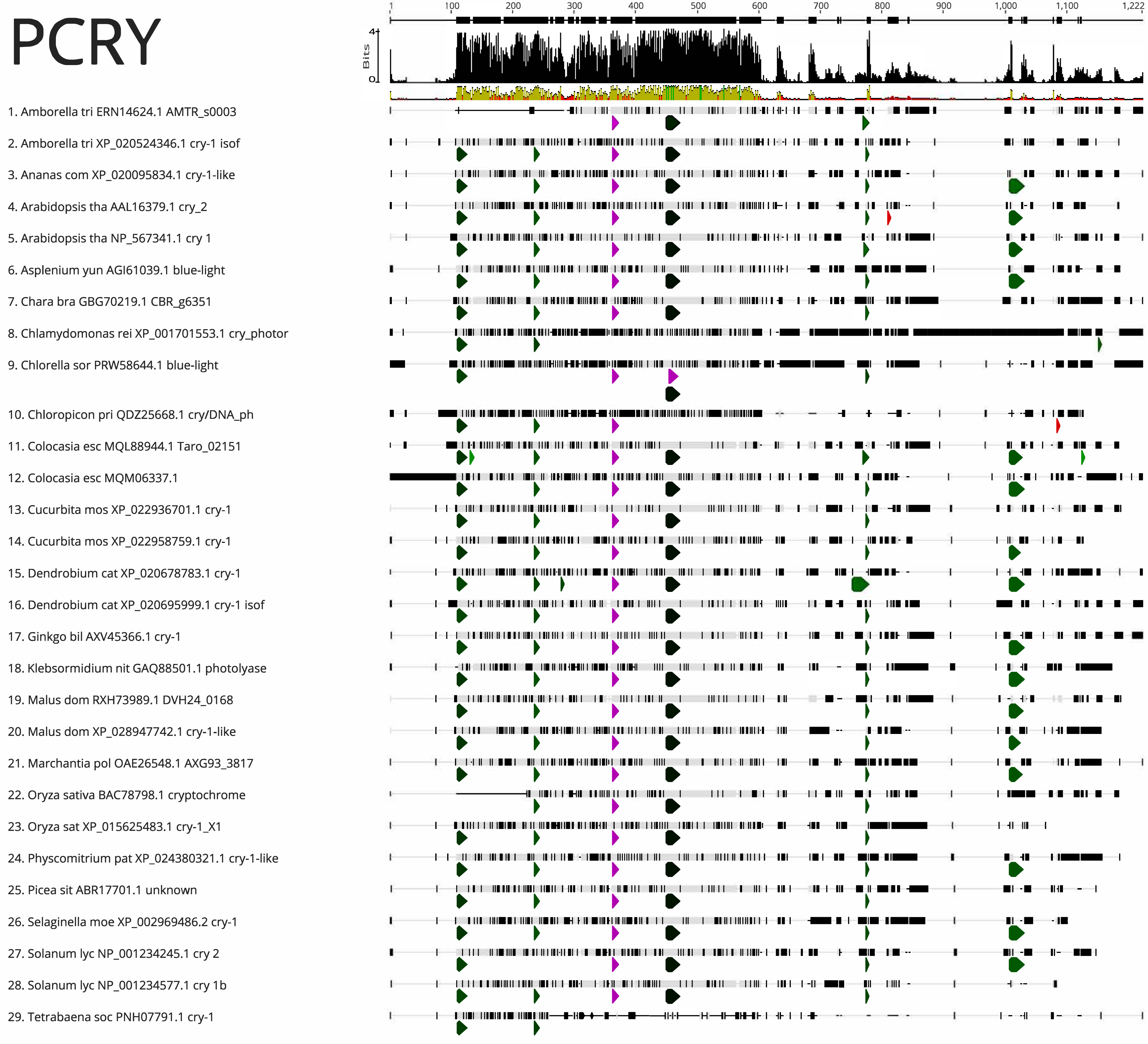
PCRY
1
100
200
300
400
500
600
700
800
900
1,000
1,100
1,222
Bits
4
0
1. Amborella tri ERN14624.1 AMTR_s0003
2. Amborella tri XP_020524346.1 cry-1 isof
3. Ananas com XP_020095834.1 cry-1-like
4. Arabidopsis tha AAL16379.1 cry_2
5. Arabidopsis tha NP_567341.1 cry 1
6. Asplenium yun AGI61039.1 blue-light
7. Chara bra GBG70219.1 CBR_g6351
8. Chlamydomonas rei XP_001701553.1 cry_photor
9. Chlorella sor PRW58644.1 blue-light
10. Chloropicon pri QDZ25668.1 cry/DNA_ph
11. Colocasia esc MQL88944.1 Taro_02151
12. Colocasia esc MQM06337.1
13. Cucurbita mos XP_022936701.1 cry-1
14. Cucurbita mos XP_022958759.1 cry-1
15. Dendrobium cat XP_020678783.1 cry-1
16. Dendrobium cat XP_020695999.1 cry-1 isof
17. Ginkgo bil AXV45366.1 cry-1
18. Klebsormidium nit GAQ88501.1 photolyase
19. Malus dom RXH73989.1 DVH24_0168
20. Malus dom XP_028947742.1 cry-1-like
21. Marchantia pol OAE26548.1 AXG93_3817
22. Oryza sativa BAC78798.1 cryptochrome
23. Oryza sat XP_015625483.1 cry-1_X1
24. Physcomitrium pat XP_024380321.1 cry-1-like
25. Picea sit ABR17701.1 unknown
26. Selaginella moe XP_002969486.2 cry-1
27. Solanum lyc NP_001234245.1 cry 2
28. Solanum lyc NP_001234577.1 cry 1b
29. Tetrabaena soc PNH07791.1 cry-1

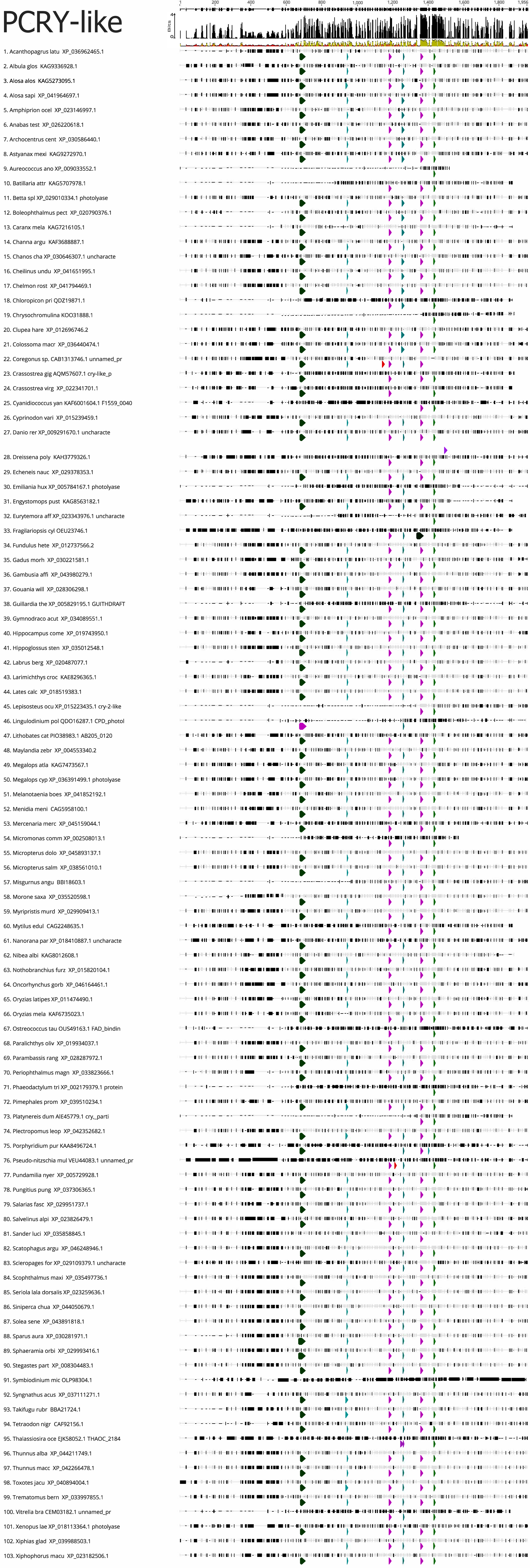
PCRY-like
1
200
400
600
800
1,000
1,200
1,400
1,600
1,800
1,956
Bits
4
0
1. Acanthopagrus latu XP_036962465.1
2. Albula glos KAG9336928.1
3. Alosa alos KAG5273095.1
4. Alosa sapi XP_041964697.1
5. Amphiprion ocel XP_023146997.1
6. Anabas test XP_026220618.1
7. Archocentrus cent XP_030586440.1
8. Astyanax mexi KAG9272970.1
9. Aureococcus ano XP_009033552.1
10. Batillaria attr KAG5707978.1
11. Betta spl XP_029010334.1 photolyase
12. Boleophthalmus pect XP_020790376.1
13. Caranx mela KAG7216105.1
14. Channa argu KAF3688887.1
15. Chanos cha XP_030646307.1 uncharacte
16. Cheilinus undu XP_041651995.1
17. Chelmon rost XP_041794469.1
18. Chloropicon pri QDZ19871.1
19. Chrysochromulina KOO31888.1
20. Clupea hare XP_012696746.2
21. Colossoma macr XP_036440474.1
22. Coregonus sp. CAB1313746.1 unnamed_pr
23. Crassostrea gig AQM57607.1 cry-like_p
24. Crassostrea virg XP_022341701.1
25. Cyanidiococcus yan KAF6001604.1 F1559_0040
26. Cyprinodon vari XP_015239459.1
27. Danio rer XP_009291670.1 uncharacte
28. Dreissena poly KAH3779326.1
29. Echeneis nauc XP_029378353.1
30. Emiliania hux XP_005784167.1 photolyase
31. Engystomops pust KAG8563182.1
32. Eurytemora aff XP_023343976.1 uncharacte
33. Fragilariopsis cyl OEU23746.1
34. Fundulus hete XP_012737566.2
35. Gadus morh XP_030221581.1
36. Gambusia affi XP_043980279.1
37. Gouania will XP_028306298.1
38. Guillardia the XP_005829195.1 GUITHDRAFT
39. Gymnodraco acut XP_034089551.1
40. Hippocampus come XP_019743950.1
41. Hippoglossus sten XP_035012548.1
42. Labrus berg XP_020487077.1
43. Larimichthys croc KAE8296365.1
44. Lates calc XP_018519383.1
45. Lepisosteus ocu XP_015223435.1 cry-2-like
46. Lingulodinium pol QDO16287.1 CPD_photol
47. Lithobates cat PIO38983.1 AB205_0120
48. Maylandia zebr XP_004553340.2
49. Megalops atla KAG7473567.1
50. Megalops cyp XP_036391499.1 photolyase
51. Melanotaenia boes XP_041852192.1
52. Menidia meni CAG5958100.1
53. Mercenaria merc XP_045159044.1
54. Micromonas comm XP_002508013.1
55. Micropterus dolo XP_045893137.1
56. Micropterus salm XP_038561010.1
57. Misgurnus angu BBI18603.1
58. Morone saxa XP_035520598.1
59. Myripristis murd XP_029909413.1
60. Mytilus edul CAG2248635.1
61. Nanorana par XP_018410887.1 uncharacte
62. Nibea albi KAG8012608.1
63. Nothobranchius furz XP_015820104.1
64. Oncorhynchus gorb XP_046164461.1
65. Oryzias latipes XP_011474490.1
66. Oryzias mela KAF6735023.1
67. Ostreococcus tau OUS49163.1 FAD_bindin
68. Paralichthys oliv XP_019934037.1
69. Parambassis rang XP_028287972.1
70. Periophthalmus magn XP_033823666.1
71. Phaeodactylum tri XP_002179379.1 protein
72. Pimephales prom XP_039510234.1
73. Platynereis dum AIE45779.1 cry,_parti
74. Plectropomus leop XP_042352682.1
75. Porphyridium pur KAA8496724.1
76. Pseudo-nitzschia mul VEU44083.1 unnamed_pr
77. Pundamilia nyer XP_005729928.1
78. Pungitius pung XP_037306365.1
79. Salarias fasc XP_029951737.1
80. Salvelinus alpi XP_023826479.1
81. Sander luci XP_035858845.1
82. Scatophagus argu XP_046248946.1
83. Scleropages for XP_029109379.1 uncharacte
84. Scophthalmus maxi XP_035497736.1
85. Seriola lala dorsalis XP_023259636.1
86. Siniperca chua XP_044050679.1
87. Solea sene XP_043891818.1
88. Sparus aura XP_030281971.1
89. Sphaeramia orbi XP_029993416.1
90. Stegastes part XP_008304483.1
91. Symbiodinium mic OLP98304.1
92. Syngnathus acus XP_037111271.1
93. Takifugu rubr BBA21724.1
94. Tetraodon nigr CAF92156.1
95. Thalassiosira oce EJK58052.1 THAOC_2184
96. Thunnus alba XP_044211749.1
97. Thunnus macc XP_042266478.1
98. Toxotes jacu XP_040894004.1
99. Trematomus bern XP_033997855.1
100. Vitrella bra CEM03182.1 unnamed_pr
101. Xenopus lae XP_018113364.1 photolyase
102. Xiphias glad XP_039988503.1
103. Xiphophorus macu XP_023182506.1

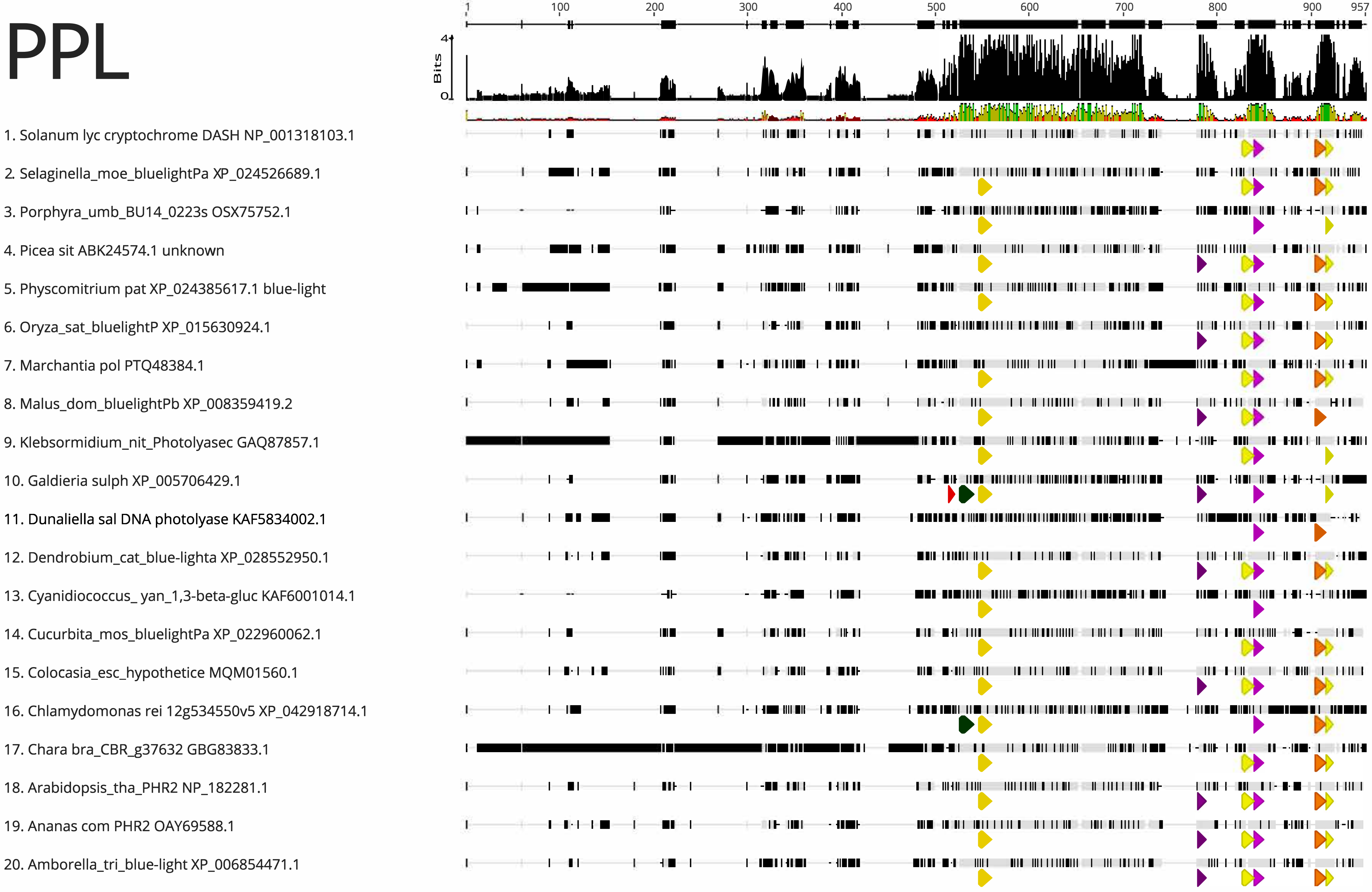

PPL
1
100
200
300
400
500
600
700
800
900
957
Bits
4
0
1. Solanum lyc cryptochrome DASH NP_001318103.1
2. Selaginella_moe_bluelightPa XP_024526689.1
3. Porphyra_umb_BU14_0223s OSX75752.1
4. Picea sit ABK24574.1 unknown
5. Physcomitrium pat XP_024385617.1 blue-light
6. Oryza_sat_bluelightP XP_015630924.1
7. Marchantia pol PTQ48384.1
8. Malus_dom_bluelightPb XP_008359419.2
9. Klebsormidium_nit_Photolyasec GAQ87857.1
10. Galdieria sulph XP_005706429.1
11. Dunaliella sal DNA photolyase KAF5834002.1
12. Dendrobium_cat_blue-lighta XP_028552950.1
13. Cyanidiococcus_ yan_1,3-beta-gluc KAF6001014.1
14. Cucurbita_mos_bluelightPa XP_022960062.1
15. Colocasia_esc_hypothetice MQM01560.1
16. Chlamydomonas rei 12g534550v5 XP_042918714.1
17. Chara bra_CBR_g37632 GBG83833.1
18. Arabidopsis_tha_PHR2 NP_182281.1
19. Ananas com PHR2 OAY69588.1
20. Amborella_tri_blue-light XP_006854471.1

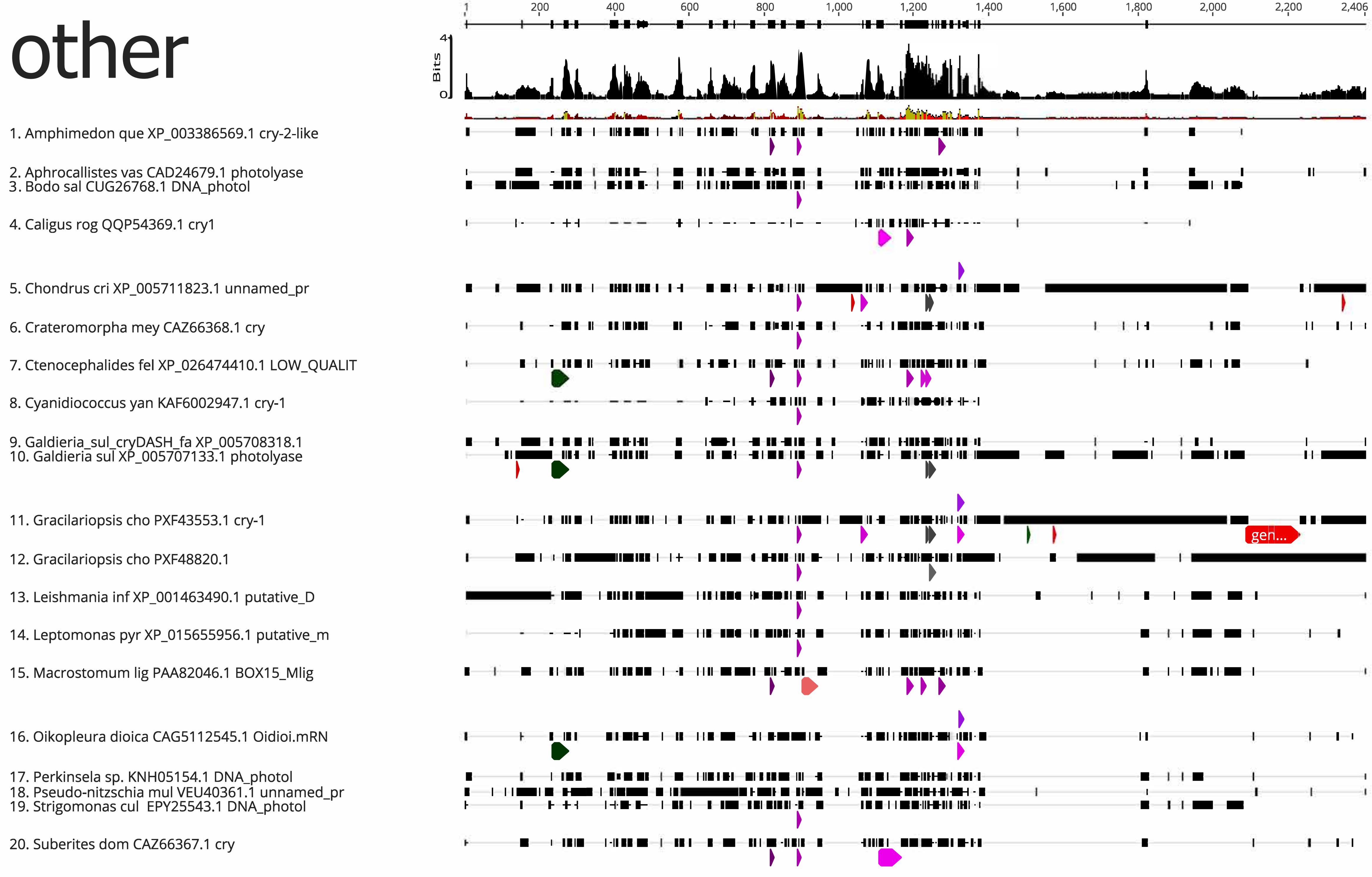

other
1
200
400
600
800
1,000
1,200
1,400
1,600
1,800
2,000
2,200
2,406
Bits
4
0
1. Amphimedon que XP_003386569.1 cry-2-like
2. Aphrocallistes vas CAD24679.1 photolyase
3. Bodo sal CUG26768.1 DNA_photol
4. Caligus rog QQP54369.1 cry1
5. Chondrus cri XP_005711823.1 unnamed_pr
6. Crateromorpha mey CAZ66368.1 cry
7. Ctenocephalides fel XP_026474410.1 LOW_QUALIT
8. Cyanidiococcus yan KAF6002947.1 cry-1
9. Galdieria_sul_cryDASH_fa XP_005708318.1
10. Galdieria sul XP_005707133.1 photolyase
11. Gracilariopsis cho PXF43553.1 cry-1
gen...
12. Gracilariopsis cho PXF48820.1
13. Leishmania inf XP_001463490.1 putative_D
14. Leptomonas pyr XP_015655956.1 putative_m
15. Macrostomum lig PAA82046.1 BOX15_Mlig
16. Oikopleura dioica CAG5112545.1 Oidioi.mRN
17. Perkinsela sp. KNH05154.1 DNA_photol
18. Pseudo-nitzschia mul VEU40361.1 unnamed_pr
19. Strigomonas cul EPY25543.1 DNA_photol
20. Suberites dom CAZ66367.1 cry
