# Supplementary material for: The Gain and Loss of Cryptochrome/Photolyase Family Members during Evolution
Source: Genes (Basel). 2022 Sep 8;13(9):1613. doi: 10.3390/genes13091613 (PMC9498864; doi:10.3390/genes13091613)
Supplement: Supplementary file 1 [file genes-13-01613-s001.zip › genes-1883542-supplementary/Supplement 5 Porifera Alignment.pdf]

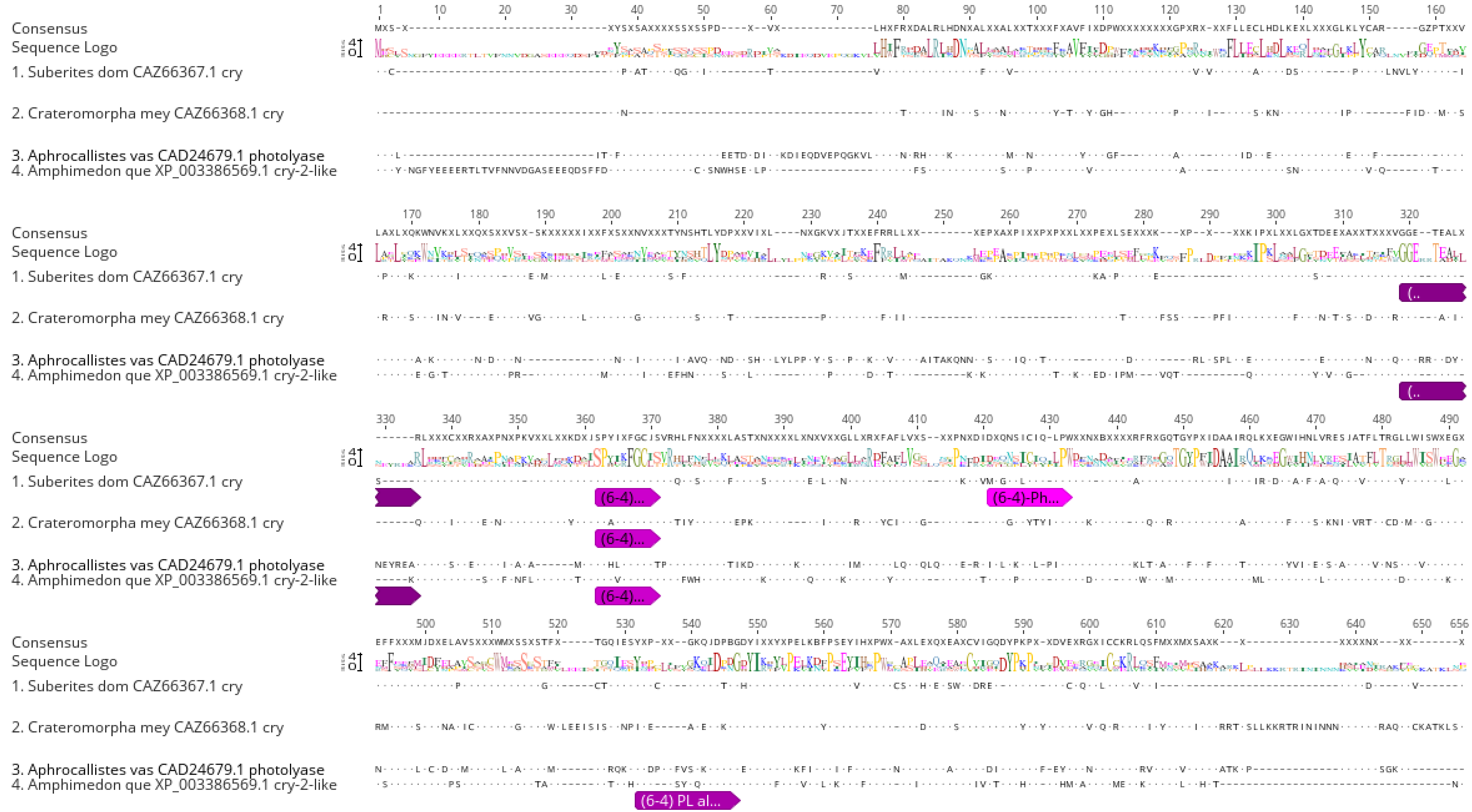
Consensus
Sequence Logo
1. Suberites dom CAZ66367.1 cry
2. Crateromorpha mey CAZ66368.1 cry
3. Aphrocallistes vas CAD24679.1 photolyase
4. Amphimedon que XP_003386569.1 cry-2-like
(6-4)...
(6-4)-Ph...
(6-4) PL al...
