# Supplementary figures and images for: The Gain and Loss of Cryptochrome/Photolyase Family Members during Evolution
Source: Genes (Basel). 2022 Sep 8;13(9):1613. doi: 10.3390/genes13091613 (PMC9498864; doi:10.3390/genes13091613)

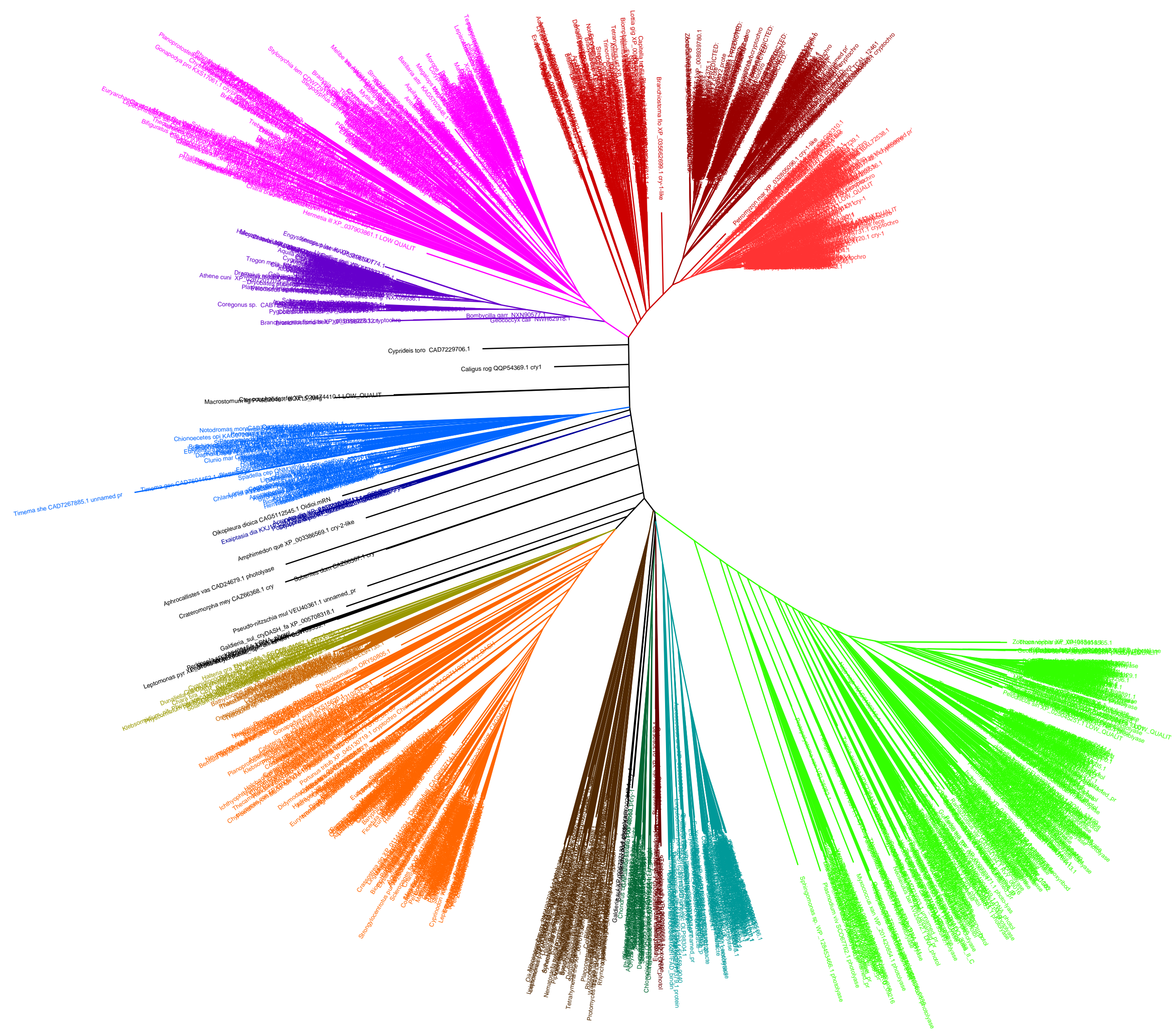

Supplement: Supplementary file 1 [file genes-13-01613-s001.zip › genes-1883542-supplementary/Supplement 6 unrooted phylogenetic tree GENERAL.pdf]
