# Supplementary material for: The Gain and Loss of Cryptochrome/Photolyase Family Members during Evolution
Source: Genes (Basel). 2022 Sep 8;13(9):1613. doi: 10.3390/genes13091613 (PMC9498864; doi:10.3390/genes13091613)

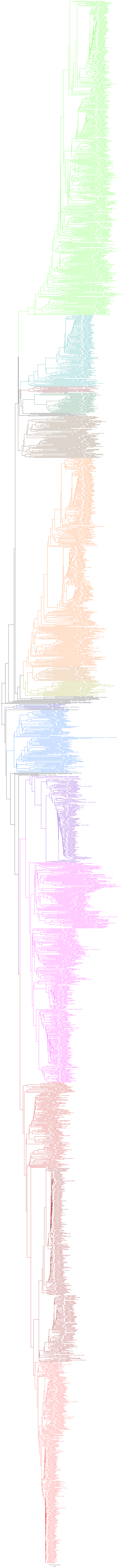

Supplement: Supplementary file 1 [file Supplement 7 rooted phylogenetic tree GENERAL.pdf › genes-1883542-supplementary/Supplement 7 rooted phylogenetic tree GENERAL.pdf]
